# A Theoretical Investigation of the Selectivity of Aza-Crown Ether Structures Chelating Alkali Metal Cations for Potential Biosensing Applications

**DOI:** 10.3390/molecules30122571

**Published:** 2025-06-12

**Authors:** Mouhmad Elayyan, Mark R. Hoffmann, Binglin Sui

**Affiliations:** Department of Chemistry, University of North Dakota, Grand Forks, ND 58202, USA; mouhmad.elayyan@und.edu

**Keywords:** aza-crown ethers, biosensors, DFT calculations

## Abstract

Aza-crown ether structures have been proven to be effective in constructing fluorescent biosensors for selectively detecting and imaging alkali metal ions in biological environments. However, choosing the right aza-crown ether for a specific alkali metal ion remains challenging for synthetic chemists because theoretical guidance on the chelating activities between aza-crown ethers and alkali metal ions has not been available up to now. Predicting the physical properties of the chelator–metal complexations poses a greater challenge due to the numerous quantum mechanical functionals and basis sets to be used in any theoretical investigation. In this study, we report a theoretical investigation of different aza-crown ether structures and their selectivities to alkali metal ions via a novel relationship between the binding energy and charge transfer calculated using twelve different quantum mechanical methods, using a myriad of bases, within the Jacob’s Ladder of Chemical Accuracies. Furthermore, this report represents a guide for the synthetic chemist in the selection of aza-crown ethers in the capturing of specific alkali metal ions, primary objectives, while benchmarking different quantum mechanical calculations, as a secondary objective.

## 1. Introduction

Alkali metal ions have crucial roles in many metabolic processes in living organisms. For example, sodium ions play important roles in water regulation and nerve and muscular functions. Potassium ions have indispensable roles in fluid balancing and muscular contractions [1,2]. These ions not only exist and serve biological purposes but are also used in behavioral medicines, such as lithium ions treating bipolar disorders [3]. Despite the advantages of alkali metal ions, there are many diseases attributed to excess alkali metal ions in a living organism. For instance, an excess intake of sodium ions can lead to kidney disease, osteoporosis, and poor sleep quality [4]. Thus, it is necessary to keep alkali metal ions within acceptable and healthy levels by measuring the concentrations of these ions in a patient’s blood sample. Hence, the development of biosensors for the selective and sensitive measurement of alkali metal ions under biological conditions is highly needed in the biomedical field.

**Figure 1:**
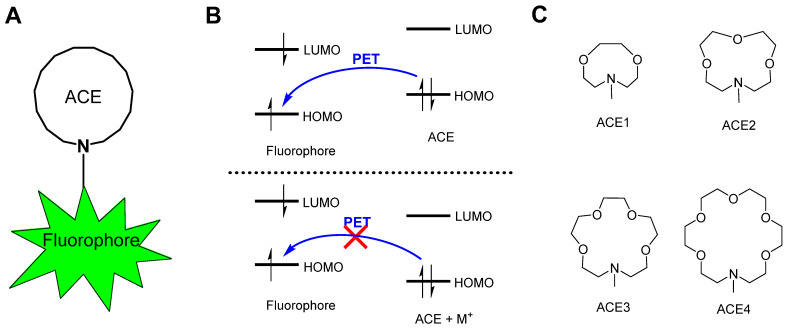
(**A**) The structure of a typical ACE-based fluorescent biosensor. (**B**) The mechanism of the photo-induced electron transfer (PET) between the fluorophore and the metal ion receptor ACE before and after chelating metal ions (M^+^). (**C**) ACE structures of interest for alkali metal ion selectivity.

Crown ethers are well-known for their ability to selectively bind to metal cations, particularly alkali metal ions. For example, the most well-known crown ether, 18-crown-6, has a high affinity for potassium ions due to its optimal cavity size [5]. In recent years, aza-crown ethers (ACEs) have attracted increasing attention in the research field of fluorescent biosensors for detecting physiological metal ions [6]. ACEs are a class of crown ethers where one of the oxygen atoms in the ring structure is replaced by a nitrogen atom [7]. This substitution imparts unique properties to aza-crown ethers, making them valuable ligands and receptors in coordination chemistry and metal ion recognition studies [8]. Compared to the divalent oxygen atom, the trivalent nitrogen atom allows for the linking of the cyclic metal ion receptors to signaling molecules, such as chromophores and fluorophores. In combination with advanced confocal fluorescence microscopy, ACE-based fluorescent biosensors have been developed to image alkali metal ions in biological environments. As shown in Figure 1A, these fluorescent biosensing molecules generally comprise two moieties: an ACE receptor and a fluorophore. Upon chelating alkali metal ions, the fluorescence emission properties of the fluorophore are changed, which can be captured by fluorescence microscopy, signaling the existence and the concentration of metal ions.

The function mechanism of ACE-based fluorescent biosensors is photo-induced electron transfer (PET), resulting from the nitrogen atom of ACEs [9]. As established, when a fluorophore absorbs a photon, an electron located in the highest occupied molecular orbital (HOMO) at the ground state is excited to the lowest unoccupied molecular orbitals (LUMOs), and a fluorescence emission is generated when the electron returns to the HOMO. As depicted in Figure 1B, in an ACE-based fluorescent biosensor molecule, when the fluorophore unit absorbs a photon and an electron is excited to the LUMO, an electron of the lone electron pair of the nitrogen atom can transit to occupy the vacancy of the HOMO of the fluorophore, which is called photo-induced electron transfer [10,11], disabling the excited electron in the LUMO to return to its original HOMO, and consequently, no fluorescence is emitted. However, upon chelating an alkali metal ion, the lone-pair electrons of the nitrogen atom bind to the metal ion and thus are no longer available for the PET process [12]. The ACE-based biosensor emits fluorescence due to the inhibition of the PET process, exhibiting a fluorescence “turn-on” behavior [13]. Such fluorescent biosensors are highly desired in the biomedical field owing to their capability for the real-time detection of targeted metal ions.

Although ACEs have been proven to be efficient in constructing fluorescent turn-on biosensors, they are still underdeveloped at present. The selectivity of ACEs in binding metal ions determines the functions and applications of the corresponding ACE-based fluorescent biosensors. So far, a relationship between the identity of ACEs and targeting metal ions has not been established. In this work, we report a theoretical study of the selectivity of four ACE structures (Figure 1C) when interacting with alkali metal ions, providing guidance for developing applicable fluorescent biosensors detecting alkali metal ions.

## 2. Results and Discussion

### 2.1. Structural Geometries of Metal-Complexed ACEs

**Figure 2:**
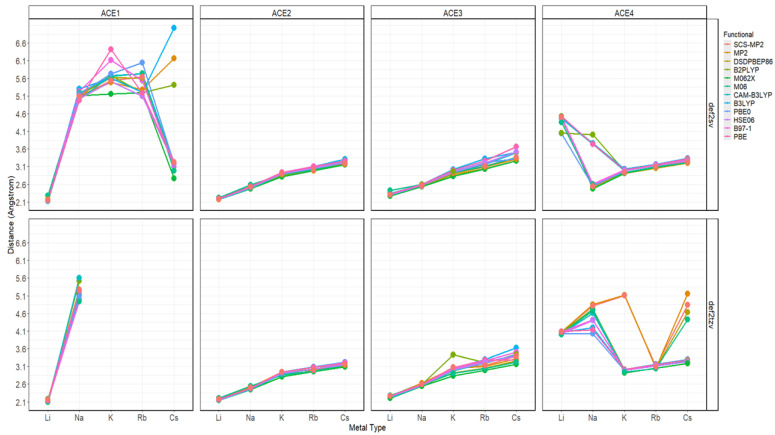
N-M^+^ distances faceted by ACE type and basis set; colored by functional.

We initially studied the structural geometries of ACEs and ACE–metal complexes with different alkali metal ions, including Li^+^, Na^+^, K^+^, Rb^+^, and Cs^+^—all final geometries are reported in the Supplementary Materials in an .xyz format. It must be noted that all geometries were evaluated using frequency calculations to ensure a “true” minima has been achieved. Since all the methods shown in Table 1 resulted in almost the same structural geometries for each ACE and ACE–metal complex, Figure 2 depicts the representative results using the wavefunction method SCS-MP2 with the def2sv basis set, through which the preference of each ACE in complexing metal ions was found. Generally, smaller alkali metal ions tend to bind to ACEs with smaller rings and vice versa. In the graphical geometries of ACE1–metal complexes, the alkali metal ions stay away from the ACE1 structure, indicating that none of them can fit in the cavity of ACE1. For ACE2, Li^+^ is located much closer to the ring than any of the other metal ions, which means complexation may occur between ACE2 and Li^+^, and it cannot bind other metal ions. ACE3 shows the potential to chelate Li^+^ and Na^+^, but it is not suitable for the others. ACE4, with the largest cavity size, makes it possible to complex all the alkali metal ions. However, it seems that the cavity size of ACE4 is too large to accommodate Li^+^, as its structural geometry was barely changed with and without the lithium-ion complexation. Therefore, ACE4 may be a receptor for alkali metal ions Na^+^, K^+^, Rb^+^, and Cs^+^.

### 2.2. Atomic Distances of ACE Heteroatoms and Metal Ions

The atomic distances between key atoms, being the metal ions, and heteroatoms—nitrogen and oxygen—of the different ACE structures show interesting results and are reported in the Supplementary Materials document. These results show whether the metal ion complexation occurs within or outside the ring of the ACE molecules; furthermore, this analysis shows the effects of the metal ion on specific atomic distances, i.e., the compression or expansion occurring to the distances due to non-covalent interactions. Due to the extensive analysis performed, this section will discuss the distances between nitrogen and other atoms and oxygen pairs—reported in Tables S1–S3 and visualized in Figures S1–S28. After investigating the effects of the metal ion on specified distances per functional and basis, the overall relationship of the metal ions’ effects on the previously mentioned distances can be investigated—in other words, the overall trend despite the functional being used per metal ion, ACE, and functional.

### 2.3. Nitrogen Distances with Metal Ions and Bonded Oxygen Atoms

**Figure 3:**
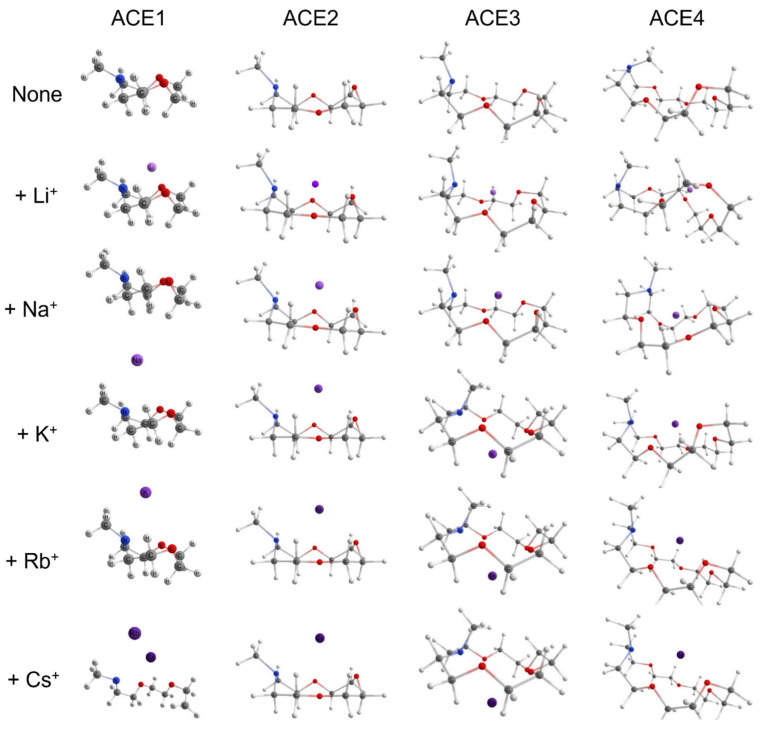
Optimized geometries of ACEs interacting with and without different alkali metal ions (Li^+^, Na^+^, K^+^, Rb^+^, and Cs^+^) using USCS-MP2/def2sv.

Starting with the N-M^+^ distance, there is an apparent trend; as the atomic radius increases—the charge density decreases, and the distance between the metal ion and nitrogen increases, with Li^+^ being closest to the nitrogen atoms, in the case of ACE1-3. Furthermore, ACE5 shows mixed results depending on the basis being used; Na^+^ and K^+^ are closest to the nitrogen atom using the split valence and triple zeta basis, respectively. It must be noted in the case of ACE1, that Cs^+^ is shown to be very near the nitrogen atom of ACE1, this is due to the optimized structure of ACE1 (def2sv) which has become disfigured completely (Figure 2 and Figure 3).

**Figure 4:**
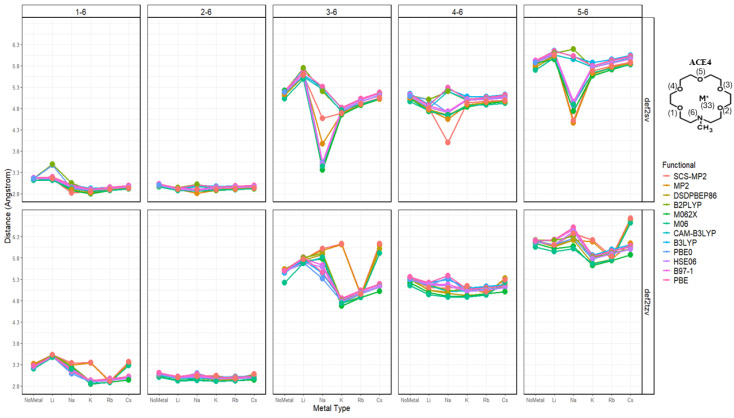
N-O_i_ distances (Å) faceted by atom pair and basis set; colored by functional.

**Figure 5:**
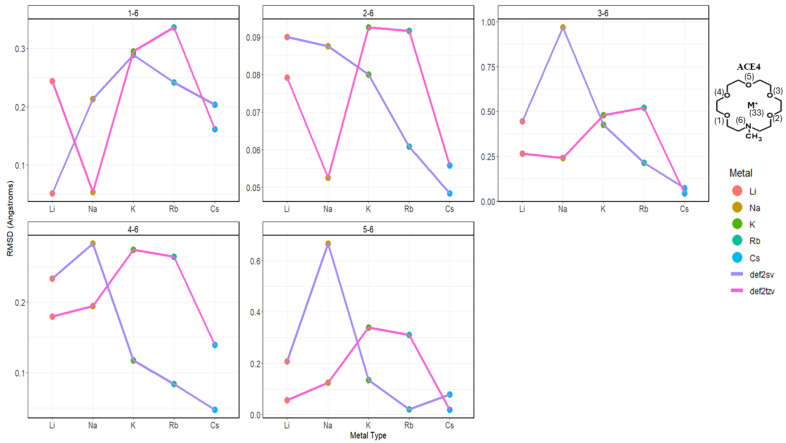
RMSD (Å) of N-O_i_ distances in ACE4 molecule faceted by atom pairs, colored by metal type and basis.

Upon the investigation of the distances of each N-O_i_ per ACE type, it can be noticed that the chelating of the metal ion onto the ACE molecule becomes unsymmetric chelating in ACE3 and ACE4, where some distances between N and O_i_ remain almost constant—less than a 0.1 Å change; furthermore, distances between the nitrogen and chelating oxygens in the ACE molecules tend to depress or compress dramatically (Figure 4). This is supported by the RMSD calculation, investigating the change in the mean distance between nitrogen and oxygen atoms, Figure 5. However, investigating the N-O_i_ and N-M^+^ is not sufficient, rather in addition to these results, other important geometrical data must be explored, including the distances between oxygen atom pairs in an ACE molecule.

### 2.4. Oxygen Pairs Distances

**Figure 6:**
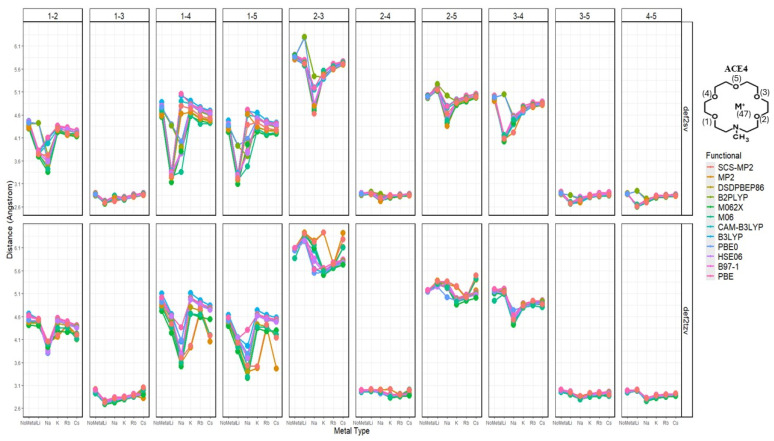
Explicit distance (Å) of O_i_–O_j_ in ACE4 molecule faceted by basis and atom pairs; colored by functional.

**Figure 7:**
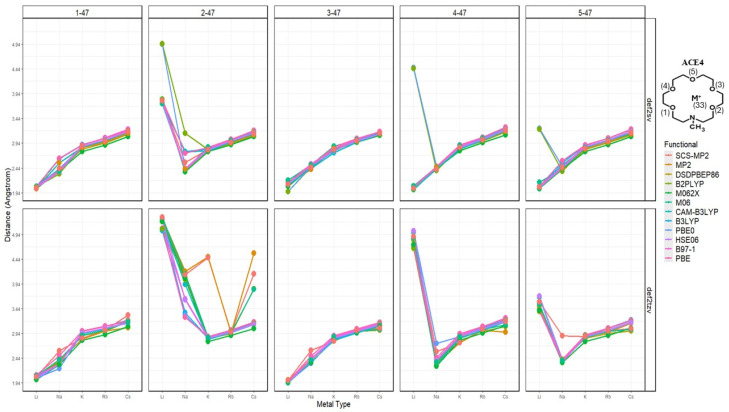
Explicit distance (Å) of O_i_–M^+^ in ACE4 molecule faceted by basis and atom pairs; colored by functional.

Thus, the following logic can hold: the decrease in the $O_{i}-O_{j}$ and/or $O_{i}-M^{+}$ pair distance(s) is (are) due to the strong non-covalent electrostatic interaction between the heteroatoms with the metal ion and indicate the “docking” of the metal ion in the ACE molecule. Beginning with the ACE1 case, the non-interacting $O_{i}-O_{j}$ pair, in the case of no metal ions, is greater than the $O_{i}-O_{j}$ pair after the complexation with a metal, with the greatest decrease in this pair’s distance being with Li^+^—and there is an agreement in all functionals and basis sets. It must be noted that Cs^+^ demonstrates a very low distance between itself and the oxygens of ACE1; this is due to the ACE ring being disfigured completely (Figure 3). In the case of ACE2, there is a clear trend in agreement with the claim that has been made before, with all functionals and basis sets in agreement; Li^+^ causes the greatest “shrinkage” of the ACE2 molecule, therefore, indicating it is firmly within the ring, Figure 6. This could apply to Na^+^; however, metal ions greater than Na^+^ show a sharper increase in the $O_{i}-M^{+}$ distances, indicating there is a significant increase in the distances between the ACE molecule and metal ions, Figure 7.

Similarly to ACE2, ACE3 displays a similar trend to the metal’s effects to the displayed $O_{i}-O_{j}$ paired distances—which is in both bases and equal across all functionals—where Li^+^ shows the greatest depression in the $O_{i}-O_{j}$ paired distances; furthermore, the ions greater than Na^+^ showed a greater increase in these lengths due to the expansion for incorporating the respective ion. It must be noted that some functionals show a decrease in $O_{i}-{Cs}^{+}$ distances; this is due to the unsymmetric interactions with the ACE3 ring accordingly, where a decrease in one side results an increase on the other. In the case of the ACE4 molecule, there are some very noticeable trends in certain atomic $O_{i}-O_{j}$ pair types; however, the system is very sensitive to the type of functional and basis being used with respect to the type of metal ion in the system. For example, the ACE4 intermolecular distances between $O_{i}\;and\;O_{j}$ under the influence of Li^+^ using the def2sv basis seems to be uniform with all functionals apart from PBE0 and B2PLYP; however, these distances become more uniform when the def2tzv basis is used— a refinement similar trend of the functionals is apparent when investigating Na^+^. Due to these effects, results can be interpreted differently; i.e., the greatest depression in atomic distances results from Li^+^ followed by Na^+^ in the def2sv basis, while Na^+^ and K^+^ arguably have the greatest depressions and smaller interaction distances than Li^+^ in most cases.

**Figure 8:**
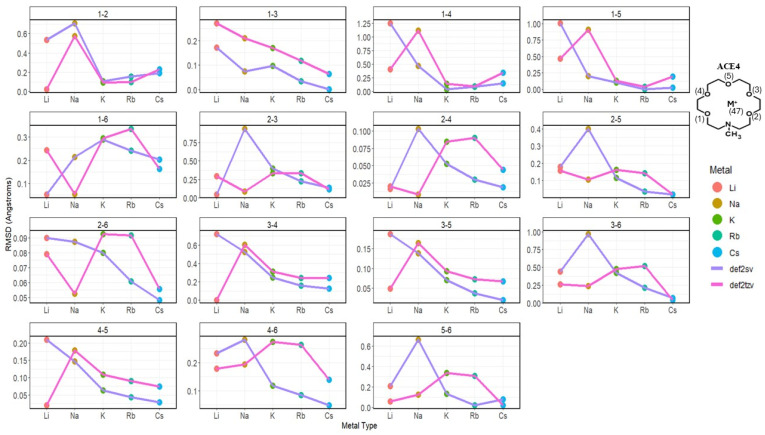
RMSD (Å) of O_i_–O_j_ distances in ACE4 molecule faceted by atom pairs and colored by metal ion (points) and basis (lines).

Similarly to the previous section, compressions in the ACE molecules tend to be unsymmetric chelating, specifically in ACE3 and ACE4. This is due to many physical factors, such as the atomic radius, charge density, ACE radius, etc.; furthermore, there is a clear trend in ACE1-3 molecules, in agreement in the basis set. Metal ions preferred by the ACE molecule tend to show, in most distances, that Li^+^ causes the greatest changes in the oxygen pair distance. This is supported by the RMSD between the oxygen pair distances before and after chelating (Figure 8).

### 2.5. Binding Energy of ACE-M^+^ Complexations Based on def2sv

The complexation between ACEs and metal ions is determined by the binding energy. The lower the binding energy, the more readily the binding occurs. With SCS-MP2 being the reference, all functionals generally agree that ACE1 and ACE2 prefer capturing Li^+^ ions (Figure S29 and Tables S4–S7), which can be ascribed to the larger charge density of lithium, according to the HSAB theory. However, as the ACE’s ring size increases, such as with ACE3 and ACE4, the lithium ion’s favorability decreases due to the disfigurations caused by the small size of Li^+^ compared to the relatively large ACE rings. Rather, the plot shows that Na^+^ becomes competitive with Li^+^, exhibiting an even lower binding energy compared to that of Li^+^. For ACE4, there is a competitiveness of the binding between Na^+^ and K^+^ ions, and Na^+^ is slightly favored.

To obtain a clearer interpretation, we further faceted the interactions between ACEs and metal ions into the different functionals (Figure S30). The forgoing rationale regarding ACE1, ACE2, and ACE3 with Li^+^ and Na^+^ ions remained the same. However, ACE4 showed a mixture of different alkali metal ion preferences. The functionals can be broadly divided into two main types: functionals/methods, whether they may be DFT- or wavefunction-based, containing some corrections, such as PBE0, B3LYP, SCS-MP2, M06, M06-2X, B97-1, DSDPBEP86, CAM-B3LYP, and HSE06, and “pure” functionals that do not contain any corrections, such as B2PLYP and PBE. Among the methods containing corrections, PBE0, SCS-MP2, M06-2X, B97-1, DSDPBEP86, and HSE06 calculations indicated that ACE4 binds the Na^+^ ion preferentially over other alkali metal ions, whereas the CAM-B3LYP calculation showed a preferred binding to the K^+^ ion for ACE4, followed by Na^+^ and Rb^+^ ions.

It needs to be noted that the calculated results by the MP2 method are largely different from those by the other DFT functionals and SCS-MP2 and are not depicted in figures, both in this manuscript and the Supporting Information, but are reported in Tables S4–S7 due to over-binding or over-estimating [15]. Also, ACE1-K^+^/Rb^+^/Cs^+^ (def2tzv) were not depicted because the binding energy of ACE1-K^+^/Rb^+^/Cs^+^ (def2tzv) were deemed unnecessary since at the lower basis, the binding energies were increasing and therefore were not compatible with this system.

### 2.6. Binding Energy of ACE-M^+^ Complexations Based on def2tzv

**Figure 9:**
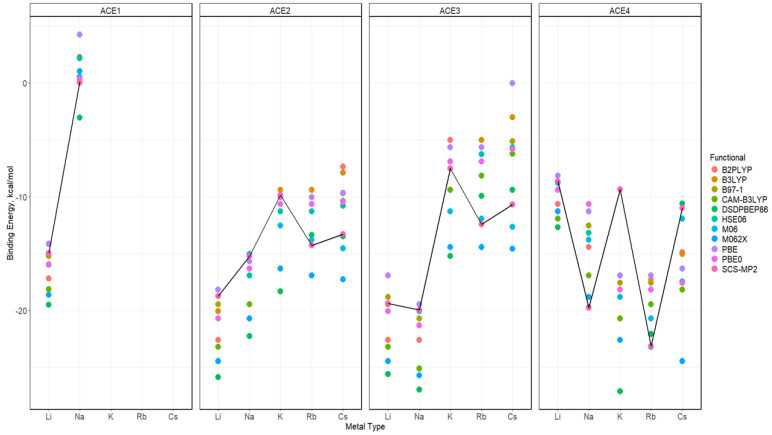
Binding energy diagrams for ACE-M^+^ faceted by the ACE type and colored by the functional based on the def2tzv and SCS-MP2 reference line.

**Figure 10:**
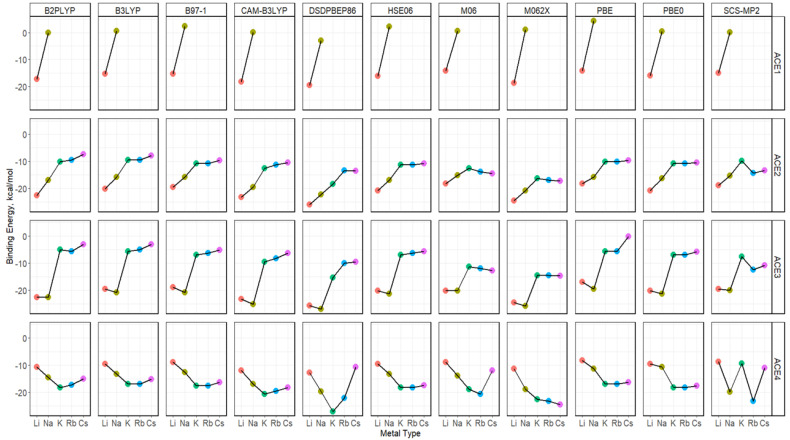
Binding energy diagrams for ACE-M^+^ faceted by the ACE type and the functional type and colored by metal ion using def2tzv basis.

As mentioned previously, alkali metal ions greater than Na^+^ were deemed unnecessary for the ACE1 complexation. Similarly to the def2sv basis set, def2tzv also found that Li^+^ is still preferred for ACE2 and ACE3 (Figure 9). Furthermore, there is a competition between Na^+^ and Li^+^ ions binding to ACE3, with Na^+^ being slightly preferred. Unlike results using the def2sv basis for the ACE4 system, the def2tzv-based calculations show mixed results. To understand the behaviors of each of the functionals, a faceted diagram with respect to the used functionals and ACE type is plotted to highlight these behaviors (Figure 10). The results indicate that all GGA, hybrid GGA, and double hybrid methods show a preference for K^+^, closely followed by Na^+^ and Rb^+^. In contrast, SCS-MP2 and Meta-GGA functionals found a preference in the Rb^+^ binding, followed by K^+^, Na^+^ and/or Cs^+^, as the dividing factor.

### 2.7. Charge Transfer of ACE-M^+^ Complexations Based on def2sv and def2tzv

**Figure 11:**
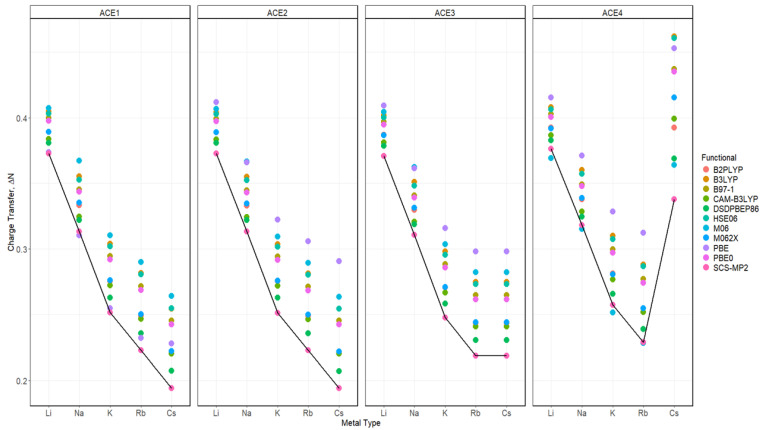
Charge transfer diagrams for ACE-M^+^ faceted by the ACE type and colored by the functional based on def2tzv and the SCS-MP2 reference line.

**Figure 12:**
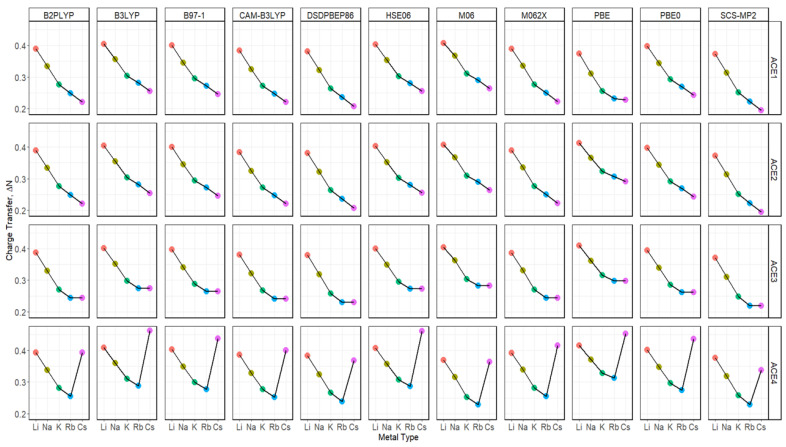
Charge transfer diagrams for ACE-M^+^ faceted by the ACE type and the functional type and colored metal ion based on def2tzv.

The Fukui function was used to determine the charge transfer in the process of the ACE-M^+^ complexation, depicted in Figure 11 and Figure S3. It was noticed that there is a trend between all functionals, where the Li^+^ ion has the greatest charge transfer for ACE1, ACE2, and ACE3, which can be attributed to the greater charge density with a small atomic radius of the Li^+^ ion. A major difference in this analysis was found in ACE4-M^+^ complexations, in which all functionals showed that Li^+^ and Cs^+^ ions are both preferred for ACE4, which is indicated by the comparable charge transfer values of chelating the two ions, despite the basis set being used. In the faceted plots (Figure 12 and Figure S21), with respect to different functionals, the observations and claims are in agreement with the data discussed above.

### 2.8. Binding Energy vs. Charge Transfer Based on def2sv

To further determine whether the ACE molecules truly select or prefer an alkali metal ion, we developed a novel approach using the binding energy and charge transfer simultaneously, assuming both factors equally contribute to the stability and selectivity of the ACE-M^+^ complexations. This allows for the comparison of experimental observations to determine whether the binding energy or charge transfer is dominant in the selectivity criteria and produces a model for designing simple ACE molecules. As shown in Figure S33, the binding energy and charge transfer values fall into different groups among all the functionals used. Logic dictates the most favorable ions for the ACE structures are ions present with the lowest binding energy values—being more spontaneous and favorable—and the highest charge transfer; in other words, the most ideal ion would be located furthest to the right, indicated by the maximum charge transfer from the plot, and would have the lowest binding energy point, indicated by the spontaneity of the binding. Based on that, the results shown in Figure S5 demonstrated that ACE1 and ACE2 prefer binding Li^+^ ions, and a discrepancy was implied for ACE3’s preference between Li^+^ and Na^+^ ions, with the binding energy preferring Na^+^ ions and the charge transfer preferring Li^+^ ions. For ACE4, it is necessary to view a more detailed outlook per functional (Figure S34). From the latter figure, the same conclusions for ACE1, ACE2, and ACE3 are still valid, which means that all functionals are in agreement. In the case of ACE4, higher levels of theory, double hybrids, and the wavefunction (SCS-MP2) show a similar trend, that Li^+^ is more preferred with Cs^+^ in competition. Furthermore, it must be noted that Li^+^ ions cause disfigurations to the ACE4 molecule in the plane of the ring, while Cs^+^ is out of the plane of the ACE4 ring, which could explain the preference for Cs^+^. Also, Na^+^ is a possible candidate for the complexation due to the lowest charge transfer. When the dispersion is included in the theoretical formulation, DSDPBEP86 versus B2PLYP is shown to be more characteristic of SCS-MP2, which is expected because DSDPBEP86 includes spin-component corrections in the theoretical formulation. Apart from the M06 functional, all other DFT functionals are in strong agreement, with the Cs^+^ ion being more favorable.

### 2.9. Binding Energy vs. Charge Transfer Based on def2tzv

**Figure 13:**
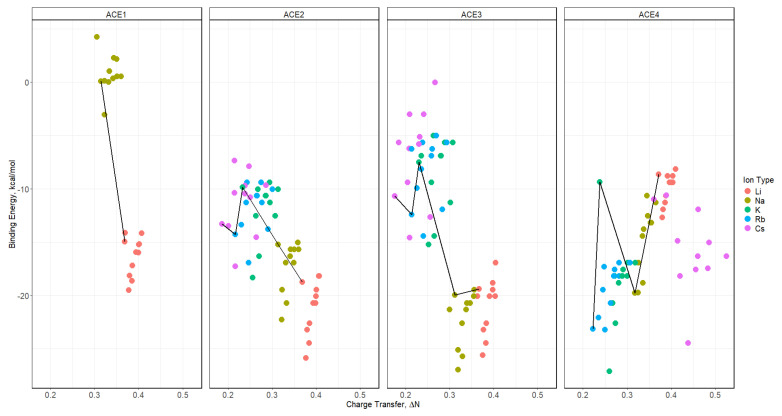
Charge transfer versus binding energy diagrams for ACE-M^+^ faceted by the ACE type, colored by the ion type, and calculated by different functionals based on def2tzv.

**Figure 14:**
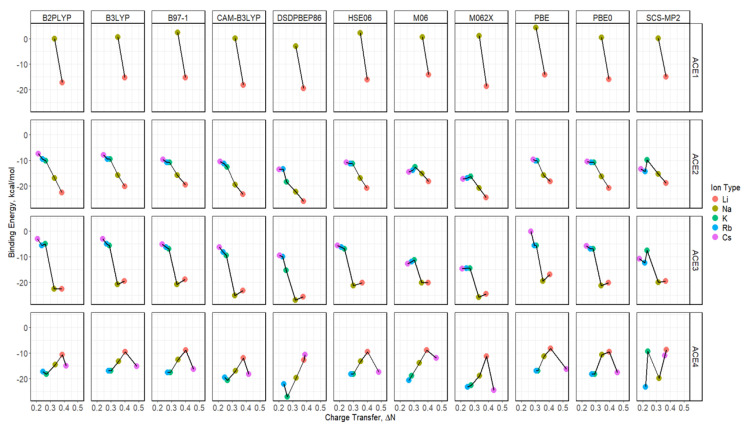
Charge transfer versus binding energy diagrams for ACE-M^+^ faceted by the ACE type and the functional type and colored by the ion type based on def2tzv.

Similarly to the def2sv basis set results, the calculations using the def2tzv basis set show similar trends, with the exception of ACE4, and ACE1 and ACE2 show a Li^+^ preference for binding, while ACE3 shows a competition between Li^+^ and Na^+^ ions—the charge transfer versus binding energy preference, Figure 13. Similarly to the above, the faceted plot detailing, Figure 14, the behavior of each functional agrees with the previously mentioned claim. However, ACE4 favors Cs^+^ and/or Li^+^ ions, showing a competition with the exception of the higher levels of functionals. The double hybrid (DSDPBEP86) and wavefunction (SCS-MP2) with the dispersion and spin-component corrections have similar results, and all other functionals are in agreement, with the Cs^+^ ion being more preferred. It must be noted that Cs^+^, Rb^+^, and K^+^ have similar values in binding energies for most functionals, while the Cs^+^ ion has a greater charge transfer.

### 2.10. Effects of Basis Set Polarization on ACE+M^+^ Systems

**Figure 15:**
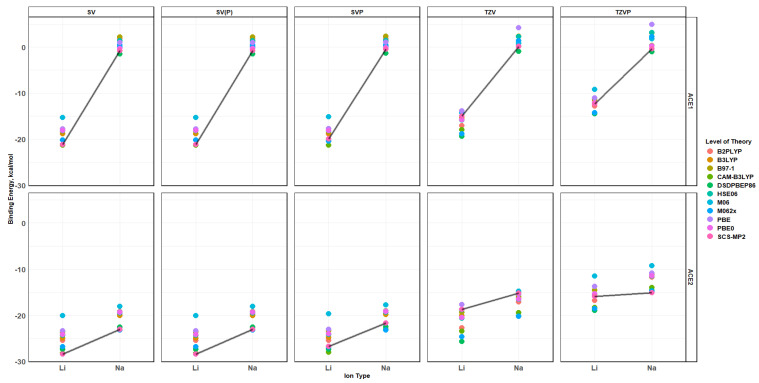
Binding energy of ACE molecules with M^+^ ions, faceted by basis and ACE type, colored by level of theory and SCS-MP2/basis black line.

**Figure 16:**
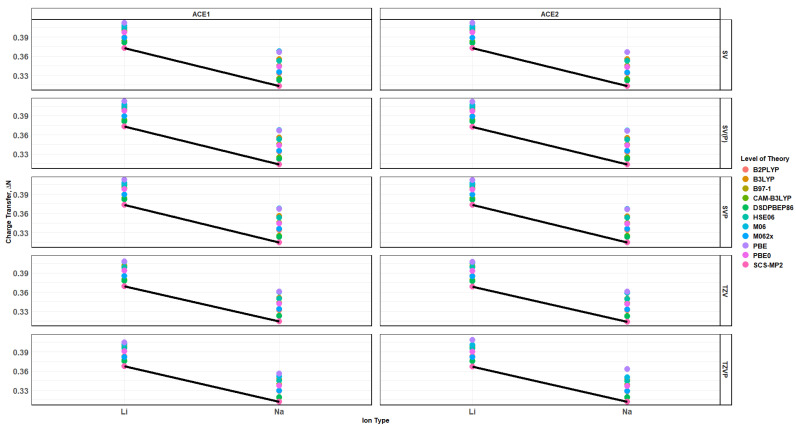
Charge Transfer faceted by basis and ACE type, colored by level of theory and SCS-MP2/basis black line.

As the name of the section might suggest, ACE1 and ACE2 in the presence of Li^+^ and Na^+^ were evaluated using six different bases, as mentioned in the following section. After optimizing and verifying that each system was at its lowest energy state, the binding energy, charge transfer, Mulliken charge, and atomic distances were used to analyze the single and double polarization in the Alderich basis and their effects on each of the mentioned properties. Similarly to before, this section will investigate the effects of adding polarization to the basis per level of theory; as well, explicit data and plots are provided in the Supplementary Material. As shown in Figure 15 and Figure 16, the binding energy and charge transfer shows the exact same trend in each type of basis and method being used; however, the energy values are different where the split valence basis, with or without polarization, differs slightly. On the other hand, the triple zeta basis without polarization shows a significant binding energy difference compared to the triple zeta with polarization; however, the trend still remains the same in both cases of ACE1,2 with Li^+^ or Na^+^. It must be noted that adding a second polarization to the hydrogen atoms in the TZVPP basis does not produce significant differences to the binding energy values in the TZVP basis—a~0.04 kcal/mol difference—and was deemed not significant enough to continue calculations with TZVPP onto systems higher than ACE1—Table 2. The trend shows the ACE+Li^+^ system has a lower binding energy than the ACE+Na^+^; thus, showing that the ACE1 molecule is selective to Li^+^, which is evidenced by the significant difference in the spread of the data points. On the other hand, ACE2 is not particularly selective to either Li^+^ and Na^+^; however, the distinguishing property would be the charge transfer evident in the Mulliken charge per ionic species, if we assumed an equal contribution or binding energy as the main contributor and charge transfer being a secondary contributor.

**Figure 17:**
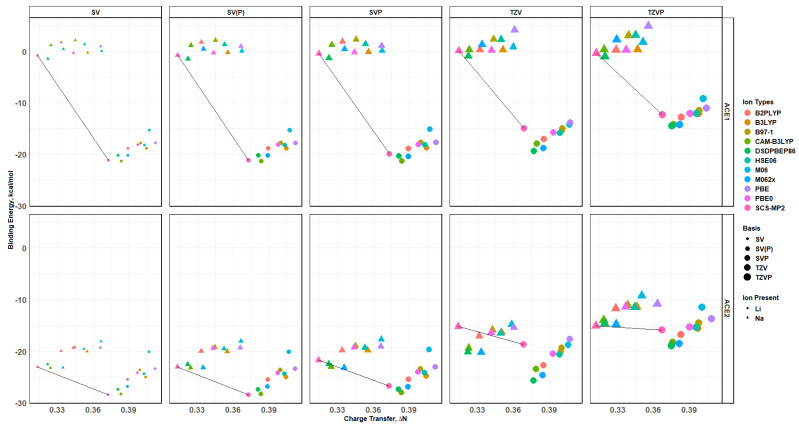
Binding energy vs. charge transfer colored by ion and faceted by basis and ACE type, SCS-MP2/basis black line.

**Figure 18:**
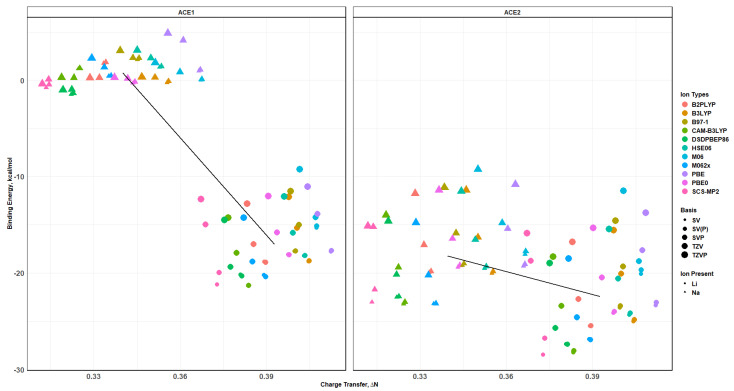
Binding energy vs. charge transfer colored by ion, sized by basis, and faceted by ACE type and cluster average black line.

**Figure 19:**
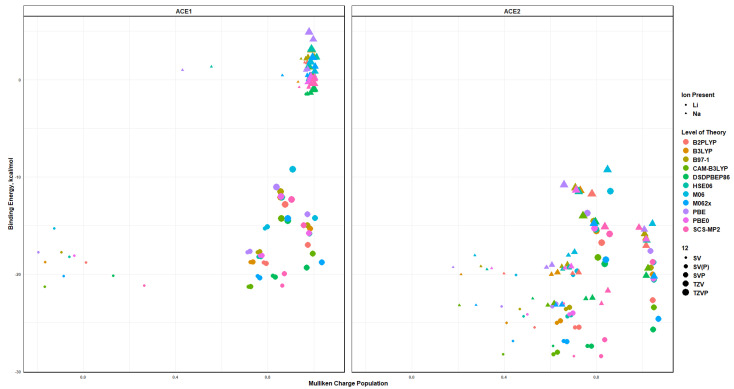
Binding energy vs. Mulliken charge of M^+^ colored by ion, sized by basis and faceted by ACE type.

The polarization effects on the ion selectivity, i.e., the binding energy and charge transfer, can be seen in Figure 17 and Figure 18. Despite the functional being used, as the size of the basis increases and with the addition of the polarization to the basis, the binding energies of the ACE2 molecule with either Li^+^ and Na^+^ become similar to each other, and the differentiating factor would be the charge transfer. It can be noticed that the charge transfer stays almost the same, but the distance between the clusters of data starts to decrease. This is verified when examining the Mulliken charge population of the metal ion in the ACE molecule with respect to the binding energy, Figure 19, which clearly depicts Na^+^ having a greater binding energy and a slightly higher Mulliken charge than Li^+^. This is supported by the distances between metal ions with heteroatoms, oxygen and nitrogen, of a particular ACE molecule as well as the Mulliken charges for the heteroatoms of a particular ACE molecule and metal ion. The distance between the oxygen atom(s) and metal ions increases as the size of the metal ion increases, which indicates the physical incompatibility of the ACE molecule to capture the metal ion; furthermore, the depression of the O-O distances in the different ACE molecules is more apparent in the Li^+^ ion than the Na^+^ ion, which indicates that there is a long-range non-covalent interaction between Na^+^ and the oxygen atom—despite the basis and functional being used.

**Figure 20:**
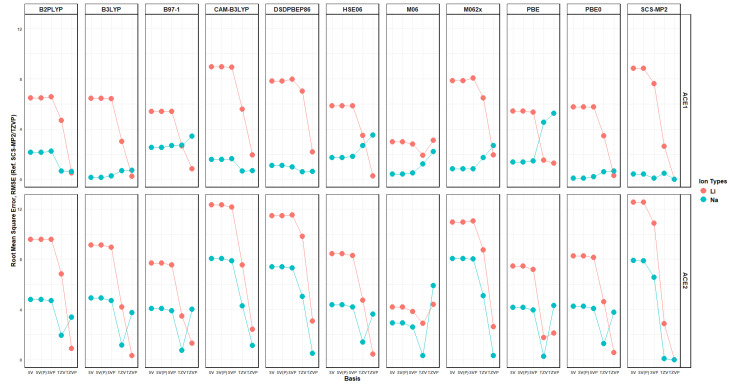
RMSE vs. basis faceted by level of theory and ACE type, colored by ion, with SCS-MP2/basis as reference.

**Figure 21:**
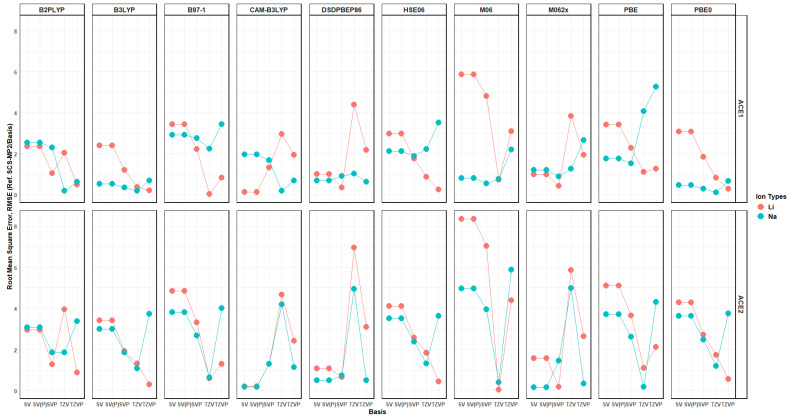
RMSE vs. basis faceted by level of theory and ACE type, colored by ion, with SCS-MP2/TZVP as reference.

Furthermore, the Mulliken charge of all heteroatoms shows a greater decrease in their partial negative charge in the presence of Li^+^ rather than Na^+^ when using a polarized basis and varies when no polarization is included. Lastly, the binding energy per functional and basis can be evaluated by using the Root Mean Squared Error (RMSE); this was achieved using two approaches, where the reference value is either SCS-MP2 in the respective basis being used or SCS-MP2/def2TZVP, as explained in later sections. When investigating the difference in both approaches, the best functionals to be used for investigating these molecules are CAM-B3LYP, M062x, DSDPBEP86, and, arguably, PBE0, with errors less than ~4 kcal/mol—Figure 20 and Figure 21. This is most likely due, as mentioned previously, to the dispersion included into their theoretical formulation and, in the case of DSDPBEP86, spin-component corrections, which allows SCS-MP2 to be a great method for investigating non-covalent interactions.

### 2.11. Excited State Calculation of ACE+M^+^ Systems

**Figure 22:**
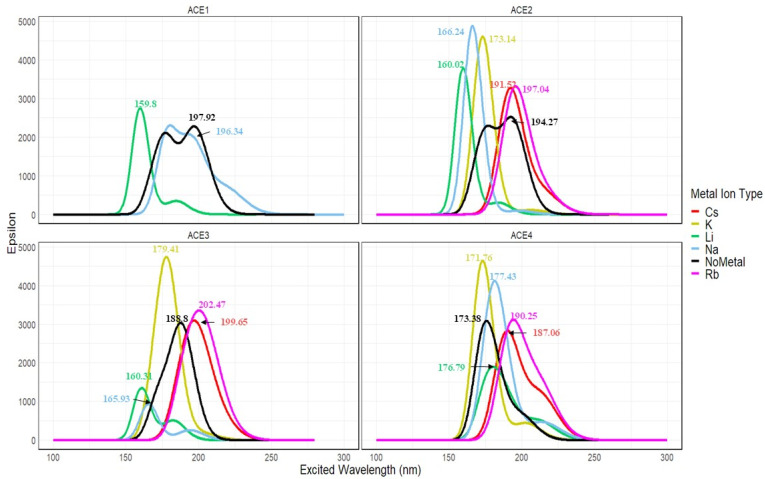
Calculated UV-Vis spectra for different ACE systems colored by metal ions and faceted by ACE type.

Excited state calculations on the various systems were performed using the unrestricted CAM-B3LYP/def2tzv, using optimized geometries from the same functional, due to its reliability in calculating conical excitations; however, these results could differ depending on the level of theory applied in the calculations. The results of the calculations are reported in Figure 22, which depicts the UV-Vis spectra of the various ACE systems complexed with the different group 1 metal ions; furthermore, numerical results of exact transitions, wavelengths, oscillatory strengths—per excited state and corresponding transition—and energies are reported in Tables S12–S15 in the Supplementary Material. From Figure 11, there is a noticeable trend for the ACE1, 2, and 3 systems upon complexations with the different metal ions, with the depression of the calculated UV-Vis spectra in each instance corresponding with the highest oscillatory strength for that specific excited state. For example, in the ACE1 calculated spectra, the “blank” spectra, no metal system, has a probable transition at 197.92 nm; furthermore, upon the complexation with lithium, there is a great shift and increase in absorption, which indicates the favorable binding. Similarly, in ACE2, the shift and increase in the absorption of the “blank” spectra has a peak at 194.27 nm; however, upon the complexation of the various ions, we can note that the greatest shift from the “blank” is the lithium ion (160.02 nm), followed closely by sodium (166.24 nm) and potassium (173.34 nm) ions, which confirms the previous results on the relationship between the binding energy and charge transfer (Figure 10, CAM-B3LYP). However, in ACE3 and ACE4 systems, it can be noticed that the more favorable binding causes shifts and depressions from the “blank”; for example, the lithium and sodium bindings have spectra of 160.31 and 165.93 nm from 188.8 nm, which indicates a competition between them.

## 3. Computational Details

Theoretically, the study of chemical and physical properties of chelators capable of predicting the binding of specific ions poses a challenge. This is due to the numerous amounts of quantum mechanical methods for studying a given system, which could also produce different results, on top of the numerous amounts of basis sets to choose from. The categorization of the different functionals’ accuracies is represented in Jacob’s Ladder of Chemical Accuracies [16], with coupled cluster methods being the gold standard for quantum mechanical-based calculations in theoretical/computational chemistry [17]. However, with the cost of computational resources increasing with the system size and level of theory, some systems are too big for convergence criteria to be met [18]; furthermore, using lower levels of theory can provide a more accurate description of experimental results than a higher level of theory due to some approximations and complications introduced in higher levels of theory. There is some common knowledge or guidelines for performing some calculations, such as that B3LYP performs well in organic systems [19], M06-2X is preferred for organometallic systems [20], CAM-B3LYP accurately describes conical excitations [21], etc. Despite these general guidelines, it is crucial to investigate a particular system using a handful of functionals to test the validity and applicability of a certain functional, as well as the associated type and size of the basis set.

In order to properly model the metal ion chelation of the ACEs, a thorough scan of different quantum mechanical methods was performed, where ten different DFT functionals and two wavefunction methods were employed (Table 1). These functionals can be classified into general gradient approximation (GGA) functionals, hybrid GGA functionals, hybrid meta-GGA functionals, double hybrid functionals, and wavefunction approaches in accordance with Jacob’s Ladder of Chemical Accuracies. Furthermore, open-shell or unrestricted calculations were used due to their capability to handle ions more thoroughly than closed-shell or restricted calculations [22]. In this study, the Spin-Component-Scaled (SCS) MP2 was used as a reference due to its capability to handle non-covalent interactions, which is the primary governing interaction in these ACE molecules; furthermore, it is capable of being within 1 kcal/mol of CCSD(T) methods [23,24,25]. In addition to using different levels of functionals and to prevent any mixing of basis sets, the Aldrich DEF2 basis sets were also used: split valence (SV) and triple ξ valence (TZV) basis sets per system with and without single and/or double polarizations (SVP, SV(P), TZVP, TZVPP) [26,27]. In choosing these bases, it must be noted, DFT and wavefunction methods work best for singly and doubly polarized larger bases; however, the wavefunction methods, i.e., MP2, suffer from the “slow convergence errors” according to the original publication. Lastly, to potentiate the applications of ACEs as biosensors in biological environments, each system was solvated implicitly during the optimization procedure using the polarizable continuum model (PCM), with water being the solvent. Each produced optimized geometry was evaluated using frequency calculations—where no imaginary frequencies were produced.

### 3.1. Binding Energy Calculations

The selectivity of ACE1-4 (Figure 1C) to different metal ions depends on the ring size of ACEs, the atomic radius of metal ions, and the non-covalent interaction strength between the ACEs and metal ions. Similarly to crown ethers, the ACE structures contain a relatively high electron density from the lone pairs of the *sp*^3^ oxygen atoms, as well as the lone pair of the *sp^3^* nitrogen atom, contributing to the non-covalent bonding between the rings and metal ions. Thus, the non-covalent bonding can be calculated using the following general reaction description and binding energy calculation expression [28].

(1) $$C_{n}H_{m}NO_{l}+M^{+}\to C_{n}H_{m}NO_{l}{-M}^{+}$$

where *C_n_H_m_NO_l_*, *M^+^*, and *C_n_H_m_NO_l_-M^+^* represent the ACE, metal ion, and ACE–metal complex, respectively.

(2a) $$E_{binding}=E_{complex}-\sum_{i=1}^{N}E_{monomer,i}$$

The monomers in the binding energy calculations are *C_n_H_m_NO_l_* (ACE) and *M^+^* (metal ion present).

(2b) $$E_{binding}=E_{complex}-\left(E_{M^{+}}+E_{C_{n}H_{m}NO_{l}}\right)$$

The energy of each monomer and complex was taken from optimization calculations.

It must be mentioned that during the binding energy calculations, errors due to basis sets can occur because of the presence of basic functions in the complex but are not present in the monomeric states, namely counterpoise corrections or the basis set superposition error (BSSE) [29,30]. This error occurs primarily due to utilizing minimal or small basis sets; however, due to the usage of these two particular basis sets, which are equivalent to the 6-31G and 6-311G Pople basis, the superposition error becomes negligible at less than 1% [15].

(3) $$E_{binding}=E_{complex}-\sum_{i=1}^{N}E_{monomer,i}+\partial_{BSSE}$$

$\partial_{BSSE}$ is not indicative of a partial derivative, but rather it is a symbol for the correction for the energy.

### 3.2. Charge Transfer and Electronegativity Calculations

In typical calculations, the charge of an atom is assigned via the Mulliken charge population, a scheme used to characterize the charge distribution, non-bonding, bonding, and anti-bonding natures for a molecule. However, it has become unreliable for determining the charge transfer because of its heavy reliance on the basis set used [31]. Another approach to calculating the charge transfer is using the Fukui function or Frontier function [32], an approach that describes the electron transfer between chemical species using frontier orbitals. In other words, the degree of the fluctuation in the electron density can be measured by adding and removing electrons from the HOMOs and LUMOs. Thus, it is typically used for density functional theory (DFT) or Kohn–Sham orbitals. Meanwhile, it could be applied to wavefunction orbitals. Electronegativity is defined as the ability to attract electrons, and it can be expressed as orbitals [32].

(4) $$\chi =-\mu ={\left(\frac{\partial E}{\partial N}\right)}_{v}=\frac{-\left(I+A\right)}{2}=\frac{-\left(E_{HOMO}+E_{LUMO}\right)}{2}$$

where χ is electronegativity, μ is the chemical potential, E is the Gibbs Free Energy, N is the continuous electronic charge, I is the ionization energy (−$E_{HOMO}$), and A is the electron affinity (−$E_{LUMO}$).

### 3.3. Chemical Hardness Calculations

Compared to electronegativity, chemical hardness, η, is a relatively newer definition defined quantitatively as the curvature of the second derivative of energy to the charge (E vs. N). The hardness of a species is the decrease in electronegativity as infinitesimally small amounts of charge are added. Hardness can be expressed in terms of the chemical potential and electronegativity as follows [32].

(5) $$\eta =\frac{1}{2}{\left(\frac{\partial^{2}E}{\partial N^{2}}\right)}_{v}=-\frac{1}{2}{\left(\frac{\partial \chi }{\partial N}\right)}_{v}=\frac{1}{2}{\left(\frac{\partial \mu }{\partial N}\right)}_{v}=\frac{\left(E_{LUMO}+E_{HOMO}\right)}{2}$$

In the scope of ACEs selectively chelating alkali metal ions, the previous definition is in agreement with the hard and soft acids and bases (HSAB) theory [33], where alkali metal ions act as hard or soft acids, depending on their atomic sizes, and ACEs act as hard bases due to their chemical structures containing oxygen and nitrogen atoms [34]. Thus, the charge transfer, Δ*N*, can be written as below.

(6) $$\Delta N=\frac{\chi_{M^{+}}-\chi_{MC}}{2\left(\eta_{M^{+}}+\eta_{MC}\right)}$$

It should be noted that greater Δ*N* values indicate a stronger complexation or preference of the ACE toward a specific metal ion; in other words, a greater charge transfer from the metal ion to the ACE, or vice versa, indicates the stabilization of the metal ion in the ACE cavity during complexation.

### 3.4. Geometric Analysis

After each of the optimization and frequency calculations, a custom program was used to calculate the atomic distances between metal ions and the C, O, and N atoms; furthermore, Root Mean Squared Deviations and Errors were achieved using a custom R script used in the RMSD and RMSE analysis—Equations (7) and (8). It must be noted that the distances used for the calculations of the RMSD are solely based on the distance between the $O_{i}-O_{j}$ and $N-O_{i}$ distance pair. Furthermore, the Root Mean Squared Error (RMSE) was achieved using two approaches, where the reference value is either SCS-MP2 or SCS-MP2/def2TZVP in the respective basis being used.

(7) $$RMSD_{i,j,k,l}=\sqrt{\sum \left({\bar{x}}_{i,j,k,l}-{\bar{x}}_{i,j,k,No\;Metal}\right)}$$

where i,j,k,and l refer to the system, basis, distance, and metal types, respectively.

(8) $$RMSE_{i,j,k-BE}=\sqrt{\sum {\left({\bar{y}}_{i,j,k}-{\bar{y}}_{i,k,SCS-MP2}\right)}^{2}}$$

where i,j and k refer to the system, basis, and level of theory, respectively.

## 4. Conclusions

In brief, our work explored the selectivity of various aza-crown ethers toward binding an array of alkali metal ions, providing a solid theoretical basis for the future development of aza-crown-ether-based biosensors. This was achieved by utilizing a total of ten DFT functionals of varied levels (PBE, PBE0, M06, M06-2X, B97-1, DSDPBEP86, B2PLYP, B3LYP, CAM-B3LYP, and HSE06), and two wavefunction methods (MP2 and SCS-MP2) and two basis sets (def2sv and def2tzv) have been used to investigate the chelating activities between a series of ACE structures and alkali metal ions as well as to investigate basis effects, specifically the polarization to the mentioned basis. Using those calculation methods, we calculated the structural geometries, binding energy, and charge transfer generated in the formation of the ACE-M^+^ complexations. Importantly, a novel approach was developed based on a combinatorial study of the binding energy and charge transfer. In spite of some minor discrepancies in the results obtained with different functionals, a general trend has been reached by the majority of the calculations. The different functionals using both the split valence and triple zeta basis show an agreement in the Li^+^ ion selectivity of ACE1 and ACE2, the competition between Li^+^ and Na^+^ for binding to ACE3, and the higher accommodating capability of ACE4 in chelating alkali metal ions. For ACE4, functionals lower than double hybrids are in all agreement with Cs^+^ being more selective, while double hybrids and wavefunction methods show that Cs^+^ and Li^+^ are in competition, with a possible preference for the Cs^+^ ion if the binding energy is a stronger contributor than the charge transfer. Our work provides solid theoretical guidance for future endeavors in developing advantageous ACE-based fluorescent biosensors for detecting and imaging alkali metal ions.

## Figures and Tables

**Table 1:** List of DFT and wavefunction methods with their classifications *.

GGA	Hybrid GGA	Hybrid Meta-GGA	Double Hybrid	Wavefunction
Pure Perdew–Burke–Ernzerhof (PBE)	Becke 3-parameter Lee–Yang–Parr (B3LYP)	Minnesota 06 (M06)	Virtual Orbital-Dependent (B2PLYP)	2nd Order Møller–Plesset (MP2)
	Coulomb-Attenuating Method of B3LYP (CAM-B3LYP)	Minnesota 06-2X (M06-2X)	Empirical Dispersion Correction (DSDPBEP86)	Spin-Component-Scaled MP2 (SCS-MP2) **
	Hybrid Perdew–Burke–Ernzerhof (PBE0)			
	Becke 97 (B97-1)			
	Heyd–Scuseria–Ernzerhof (HSE06)			

* All calculations were performed using Gaussian 09 Software Suite; ** SCS-MP2 calculations were achieved using IOP (3/125 = 0333312000) [14].

**Table 2:** Binding energy (kcal/mol) per ACE complex, basis, and level of theory.

**ACE1 + Li**							**ACE2 + Li**				
**Level of** **Theory**	**SV**	**SVP**	**SV(P)**	**TZV**	**TZVP**	**TZVPP**	**SV**	**SVP**	**SV(P)**	**TZV**	**TZVP**
B2PLYP	−18.821	−18.925	−18.821	−17.024	−12.843	−12.888	−25.462	−25.467	−25.462	−22.699	−16.785
B3LYP	−18.788	−18.757	−18.788	−15.344	−12.126	−12.151	−25.007	−24.821	−25.007	−20.075	−15.576
B971	−17.752	−17.737	−17.752	−15.008	−11.525	−11.543	−23.586	−23.421	−23.586	−19.359	−14.58
CAM-B3LYP	−21.318	−21.294	−21.318	−17.932	−14.293	−14.327	−28.218	−28.04	−28.218	−23.428	−18.318
DSDPBEP86	−20.173	−20.324	−20.174	−19.365	−14.529	−14.58	−27.352	−27.406	−27.352	−25.714	−18.985
HSE06	−18.208	−18.211	−18.208	−15.848	−12.1	−12.12	−24.323	−24.174	−24.323	−20.601	−15.445
M06	−15.317	−15.153	−15.317	−14.253	−9.247	−9.557	−20.076	−19.707	−20.076	−18.799	−11.509
M062X	−20.195	−20.398	−20.195	−18.825	−14.295	−14.258	−26.846	−26.924	−26.846	−24.611	−18.532
MP2	−18.595	−17.411	−18.595	−6.566	7.646	7.716	−28.353	−26.682	−28.353	−18.201	−15.814
PBE	−17.773	−17.695	−17.773	−13.875	−11.07	−11.083	−23.328	−23.082	−23.328	−17.646	−13.771
PBE0	−18.096	−18.118	−18.096	−15.813	−12.056	−12.084	−24.143	−24.019	−24.143	−20.495	−15.323
SCS-MP2	−21.185	−19.969	−21.185	−14.98	−12.347	−12.388	−28.427	−26.743	−28.427	−18.753	−15.89
**ACE1 + Na**							**ACE2 + Na**				
**Level of** **Theory**	**SV**	**SVP**	**SV(P)**	**TZV**	**TZVP**	**TZVPP**	**SV**	**SVP**	**SV(P)**	**TZV**	**TZVP**
B2PLYP	1.75	1.844	1.749	0.26	0.235	0.219	−25.462	−19.849	−19.945	−17.091	−11.784
B3LYP	−0.248	−0.119	−0.248	0.274	0.307	0.31	−25.007	−19.85	−20.043	−16.315	−11.41
B971	2.136	2.301	2.136	2.314	3.049	3.043	−23.586	−19.034	−19.223	−15.883	−11.132
CAM-B3LYP	1.177	1.231	1.177	0.257	0.298	0.301	−28.218	−23.019	−23.21	−19.418	−14.011
DSDPBEP86	−1.483	−1.364	−1.483	−0.964	−1.021	−1.053	−27.352	−22.449	−22.529	−20.174	−14.647
HSE06	1.337	1.413	1.338	2.291	3.119	3.118	−24.323	−19.335	−19.521	−16.555	−11.518
M06	0.03	0.094	0.026	0.836	1.81	1.791	−20.076	−17.748	−18.071	−14.826	−9.266
M062X	0.424	0.437	0.424	1.339	2.279	2.277	−26.846	−23.171	−23.197	−20.223	−14.807
MP2	−14.82	−17.355	−14.82	−94.75	−113.244	−117.004	−28.353	−21.911	−23.198	−15.069	−15.587
PBE	0.973	1.073	0.971	4.141	4.872	4.874	−23.328	−19.084	−19.313	−15.428	−10.846
PBE0	−0.314	−0.178	−0.314	0.188	0.271	0.191	−24.143	−19.22	−19.393	−16.443	−11.396
SCS-MP2	−0.789	−0.463	−0.789	0.073	−0.39	−0.358	−28.427	−21.709	−23.03	−15.231	−15.15

## Data Availability

The original contributions presented in this study are included in the article/Supplementary Material. Further inquiries can be directed to the corresponding authors.

## Supplementary Materials

The following supporting information can be downloaded at: https://www.mdpi.com/article/10.3390/molecules30122571/s1.
