# Supplementary material for: A Theoretical Investigation of the Selectivity of Aza-Crown Ether Structures Chelating Alkali Metal Cations for Potential Biosensing Applications
Source: Molecules. 2025 Jun 12;30(12):2571. doi: 10.3390/molecules30122571 (PMC12195789; doi:10.3390/molecules30122571)
Supplement: Supplementary file 1 [file molecules-30-02571-s001.zip › molecules-3522884-supplementary.pdf]

Supporting Information

A Theoretical Investigation of the Selectivity of Aza-Crown-Ether Structures Chelating Alkali Metal Cations for Potential Biosensing Applications

Mouhmad Elayyan, Mark Hoffmann,* and Binglin Sui*

Department of Chemistry, University of North Dakota, Grand Forks, North Dakota 58202, United States

* E-mail address. mark.hoffmann@und.edu; binglin.sui@und.edu

Table of Contents

Geometric Results of Calculated Distances Between Atom Pairs 4

Geometric Data (Section 2.2-2.4) 4

Geometric Data (Section 2.10) 15

Graphical Representation of Geometric Data (Section 2.2-2.4): 17

Graphical Representation of Geometric Data (Section 2.10): 32

Binding Energy Calculation Data (Sections 2.6) 36

Charge Transfer Calculation Data (Sections 2.7) 37

Binding Energy Calculation Data (Sections 2.10) 39

Binding Energy and Charge Transfer Graphical Results (Sections 2.6-2.9) 40

Binding Energy, Charge Transfer Graphical Results (Sections 2.10) 44

Excited States Calculation Results (Section 2.11) 50

Output Geometry Optimization (XYZ format) (Section 2.1-2.9) 53

Output Geometry Optimization (XYZ format) (Section 2.10) 760

Table S1. N-M+ Distances Produced From Optimized Geometry per Functional

ACE1 System										
	def2sv					def2tzv				
Functional	Li	Na	K	Rb	Cs	Li	Na	K	Rb	Cs
B2PLYP	2.14	5.11	5.68	5.19	5.41	2.13	5.52	Deemed Unnecessary		
B3LYP	2.14	5.30	5.61	5.21	7.03	2.12	5.01			
B97-1	2.15	5.23	6.12	5.54	3.09	2.13	4.95			
CAM-B3LYP	2.12	5.00	5.68	5.73	3.19	2.10	5.60			
DSDPBEP86	2.14	5.13	5.62	5.61	3.20	2.13	5.22			
HSE06	2.14	5.03	5.51	5.10	3.18	2.12	4.95			
M06	2.28	5.18	5.66	5.74	2.98	2.18	4.94			
M062X	2.13	5.11	5.16	5.20	2.76	2.10	5.13			
MP2	2.17	5.11	5.48	5.28	6.16	2.17	5.28			
PBE	2.15	4.97	6.42	5.18	3.20	2.14	5.24			
PBE0	2.14	5.14	5.73	6.04	3.18	2.12	5.10			
SCS-MP2	2.15	5.08	5.54	5.64	3.23	2.16	5.28			
ACE2 System										
B2PLYP	2.18	2.51	2.91	3.06	3.23	3.18	2.93	2.17	2.48	3.03
B3LYP	2.19	2.52	2.94	3.11	3.31	3.23	2.94	2.17	2.48	3.09
B97-1	2.19	2.53	2.94	3.10	3.26	3.21	2.94	2.17	2.49	3.08
CAM-B3LYP	2.17	2.49	2.89	3.05	3.23	3.17	2.89	2.15	2.46	3.03
DSDPBEP86	2.17	2.49	2.84	2.98	3.15	3.10	2.88	2.16	2.47	2.96
HSE06	2.18	2.51	2.90	3.06	3.22	3.15	2.91	2.16	2.48	3.03
M06	2.17	2.48	2.81	2.98	3.15	3.09	2.81	2.15	2.45	2.96
M062X	2.22	2.58	2.88	3.02	3.20	3.14	2.86	2.20	2.55	2.97
MP2	2.17	2.52	2.88	2.99	3.17	3.14	2.94	2.18	2.51	2.99
PBE	2.18	2.51	2.90	3.06	3.23	3.16	2.91	2.16	2.47	3.03
PBE0	2.19	2.53	2.94	3.09	3.26	3.19	2.95	2.17	2.49	3.08
SCS-MP2	2.18	2.52	2.90	3.01	3.19	3.16	2.94	2.18	2.51	3.00
ACE3 System										
B2PLYP	2.32	2.58	3.02	2.96	3.50	2.25	2.59	3.43	3.19	3.44
B3LYP	2.34	2.59	3.00	3.32	3.51	2.26	2.59	3.05	3.30	3.62
B97-1	2.33	2.59	2.93	3.26	3.67	2.27	2.59	3.04	3.26	3.50
CAM-B3LYP	2.29	2.57	2.87	3.17	3.50	2.22	2.56	2.98	3.20	3.43
DSDPBEP86	2.30	2.56	2.95	3.05	3.26	2.23	2.57	2.92	3.06	3.26
HSE06	2.31	2.57	2.93	3.20	3.52	2.25	2.57	3.01	3.21	3.39
M06	2.42	2.60	2.83	3.11	3.36	2.28	2.59	2.91	3.04	3.22
M062X	2.27	2.54	2.91	3.03	3.26	2.20	2.54	2.84	3.00	3.16
MP2	2.31	2.58	3.00	3.09	3.31	2.26	2.63	3.06	3.10	3.32
PBE	2.33	2.59	2.96	3.26	3.67	2.27	2.59	3.07	3.28	3.29
PBE0	2.31	2.57	2.93	3.21	3.33	2.25	2.57	3.01	3.22	3.42
SCS-MP2	2.30	2.55	3.19	3.12	3.36	2.26	2.59	3.08	3.13	3.39
ACE4 System										
B2PLYP	4.05	3.76	3.03	3.17	3.34	4.06	4.20	3.02	3.17	3.30
B3LYP	4.48	2.61	3.01	3.16	3.31	4.08	4.42	3.01	3.15	3.28
B97-1	4.51	3.74	2.98	3.13	3.29	4.01	4.61	2.99	3.11	3.25
CAM-B3LYP	4.47	2.55	2.98	3.13	3.27	4.07	4.85	5.12	3.06	5.16
DSDPBEP86	4.48	3.74	3.01	3.16	3.32	4.03	4.03	2.99	3.12	3.24

HSE06	4.48	4.00	2.98	3.13	3.29	4.09	4.13	3.02	3.15	3.28
M06	4.36	2.49	2.91	3.06	3.20	4.08	4.71	3.01	3.12	3.26
M062X	4.53	2.60	2.98	3.13	3.27	4.05	4.73	2.95	3.04	4.63
MP2	4.51	2.48	2.91	3.08	3.21	4.03	4.40	2.99	3.11	3.24
PBE	4.53	2.60	2.94	3.09	3.22	4.03	4.68	2.92	3.05	3.19
PBE0	4.04	2.54	2.92	3.08	3.22	4.00	4.71	2.95	3.04	4.43
SCS-MP2	4.50	2.55	2.94	3.09	3.24	4.07	4.82	5.11	3.09	4.84

Geometric Results of Calculated Distances Between Atom Pairs

Geometric Data (Section 2.2-2.4)

Table S2. Mean and Root Mean Squared Deviation of N-O Distances (Å) - Eq. 7

		ACE1			
**Metal**	**Atom Pair**	**Basis**	$\bar{x}_{i,j,k,l}$	$\bar{x}_{i,j,k,N\ M}$	$R\quad D_{i,j,k,l}$
Li	1-15	def2sv	2.785	2.867	0.082
Li	1-15	def2tzv	2.808	2.944	0.137
Li	1-8	def2sv	2.818	2.923	0.105
Li	1-8	def2tzv	2.844	2.993	0.149
Na	1-15	def2sv	2.895	2.867	0.028
Na	1-15	def2tzv	2.968	2.944	0.024
Na	1-8	def2sv	2.953	2.923	0.030
Na	1-8	def2tzv	3.013	2.993	0.020
K	1-15	def2sv	2.878	2.867	0.012
K	1-8	def2sv	2.927	2.923	0.003
Rb	1-15	def2sv	2.883	2.867	0.016
Rb	1-8	def2sv	2.943	2.923	0.020
Cs	1-15	def2sv	2.996	2.867	0.129
Cs	1-8	def2sv	4.934	2.923	2.011
		ACE2			
Li	1-4	def2sv	2.818	2.932	0.114
Li	1-4	def2tzv	2.843	3.028	0.184
Li	2-4	def2sv	2.744	2.933	0.189
Li	2-4	def2tzv	2.757	3.026	0.269
Li	3-4	def2sv	4.203	4.541	0.338
Li	3-4	def2tzv	4.222	4.474	0.253
Na	1-4	def2sv	2.918	2.932	0.013
Na	1-4	def2tzv	2.981	3.028	0.047
Na	2-4	def2sv	2.831	2.933	0.103
Na	2-4	def2tzv	2.863	3.026	0.163
Na	3-4	def2sv	4.438	4.541	0.103
Na	3-4	def2tzv	4.482	4.474	0.008
K	1-4	def2sv	2.967	2.932	0.035
K	1-4	def2tzv	3.042	3.028	0.014
K	2-4	def2sv	2.869	2.933	0.064
K	2-4	def2tzv	2.933	3.026	0.092
K	3-4	def2sv	4.603	4.541	0.062

K	3-4	def2tzv	4.663	4.474	0.188
Rb	1-4	def2sv	2.978	2.932	0.046
Rb	1-4	def2tzv	3.048	3.028	0.021
Rb	2-4	def2sv	2.888	2.933	0.046
Rb	2-4	def2tzv	2.943	3.026	0.083
Rb	3-4	def2sv	4.644	4.541	0.103
Rb	3-4	def2tzv	4.693	4.474	0.218
Cs	1-4	def2sv	2.991	2.932	0.059
Cs	1-4	def2tzv	3.058	3.028	0.031
Cs	2-4	def2sv	2.897	2.933	0.037
Cs	2-4	def2tzv	2.946	3.026	0.080
Cs	3-4	def2sv	4.683	4.541	0.142
Cs	3-4	def2tzv	4.732	4.474	0.258
			ACE3		
Li	1-5	def2sv	2.772	2.961	0.189
Li	1-5	def2tzv	2.797	3.051	0.254
Li	2-5	def2sv	2.782	2.944	0.163
Li	2-5	def2tzv	2.782	3.040	0.258
Li	3-5	def2sv	4.623	4.994	0.372
Li	3-5	def2tzv	4.868	5.094	0.226
Li	4-5	def2sv	4.396	4.929	0.533
Li	4-5	def2tzv	4.272	5.020	0.748
Na	1-5	def2sv	2.888	2.961	0.073
Na	1-5	def2tzv	2.918	3.051	0.133
Na	2-5	def2sv	2.880	2.944	0.064
Na	2-5	def2tzv	2.906	3.040	0.134
Na	3-5	def2sv	4.759	4.994	0.235
Na	3-5	def2tzv	4.792	5.094	0.302
Na	4-5	def2sv	4.729	4.929	0.200
Na	4-5	def2tzv	4.759	5.020	0.261
K	1-5	def2sv	2.803	2.961	0.158
K	1-5	def2tzv	2.878	3.051	0.173
K	2-5	def2sv	2.843	2.944	0.101
K	2-5	def2tzv	2.900	3.040	0.140
K	3-5	def2sv	3.825	4.994	1.169
K	3-5	def2tzv	3.897	5.094	1.198
K	4-5	def2sv	4.428	4.929	0.502
K	4-5	def2tzv	4.493	5.020	0.527
Rb	1-5	def2sv	2.829	2.961	0.132
Rb	1-5	def2tzv	2.878	3.051	0.173
Rb	2-5	def2sv	2.853	2.944	0.091
Rb	2-5	def2tzv	2.901	3.040	0.139
Rb	3-5	def2sv	3.762	4.994	1.233
Rb	3-5	def2tzv	3.921	5.094	1.173
Rb	4-5	def2sv	4.426	4.929	0.503
Rb	4-5	def2tzv	4.489	5.020	0.531
Cs	1-5	def2sv	2.803	2.961	0.158

Cs	1-5	def2tzv	2.874	3.051	0.177
Cs	2-5	def2sv	2.860	2.944	0.084
Cs	2-5	def2tzv	2.905	3.040	0.135
Cs	3-5	def2sv	3.926	4.994	1.068
Cs	3-5	def2tzv	3.938	5.094	1.156
Cs	4-5	def2sv	4.349	4.929	0.580
Cs	4-5	def2tzv	4.441	5.020	0.579
			ACE4		
Li	1-6	def2sv	3.205	3.153	0.052
Li	1-6	def2tzv	3.504	3.260	0.244
Li	2-6	def2sv	2.908	2.998	0.090
Li	2-6	def2tzv	2.988	3.067	0.079
Li	3-6	def2sv	5.605	5.160	0.445
Li	3-6	def2tzv	5.743	5.478	0.266
Li	4-6	def2sv	4.843	5.077	0.233
Li	4-6	def2tzv	5.118	5.297	0.179
Li	5-6	def2sv	6.064	5.856	0.208
Li	5-6	def2tzv	6.120	6.177	0.057
Na	1-6	def2sv	2.940	3.153	0.213
Na	1-6	def2tzv	3.206	3.260	0.054
Na	2-6	def2sv	2.911	2.998	0.088
Na	2-6	def2tzv	3.014	3.067	0.052
Na	3-6	def2sv	4.193	5.160	0.967
Na	3-6	def2tzv	5.718	5.478	0.240
Na	4-6	def2sv	4.794	5.077	0.283
Na	4-6	def2tzv	5.103	5.297	0.194
Na	5-6	def2sv	5.190	5.856	0.666
Na	5-6	def2tzv	6.301	6.177	0.124
K	1-6	def2sv	2.864	3.153	0.289
K	1-6	def2tzv	2.965	3.260	0.295
K	2-6	def2sv	2.918	2.998	0.080
K	2-6	def2tzv	2.974	3.067	0.092
K	3-6	def2sv	4.734	5.160	0.426
K	3-6	def2tzv	4.998	5.478	0.479
K	4-6	def2sv	4.959	5.077	0.118
K	4-6	def2tzv	5.023	5.297	0.274
K	5-6	def2sv	5.721	5.856	0.135
K	5-6	def2tzv	5.838	6.177	0.339
Rb	1-6	def2sv	2.912	3.153	0.242
Rb	1-6	def2tzv	2.924	3.260	0.336
Rb	2-6	def2sv	2.938	2.998	0.061
Rb	2-6	def2tzv	2.975	3.067	0.092
Rb	3-6	def2sv	4.947	5.160	0.213
Rb	3-6	def2tzv	4.957	5.478	0.521
Rb	4-6	def2sv	4.993	5.077	0.084
Rb	4-6	def2tzv	5.033	5.297	0.264
Rb	5-6	def2sv	5.836	5.856	0.020

Rb	5-6	def2tzv	5.866	6.177	0.311
Cs	1-6	def2sv	2.950	3.153	0.203
Cs	1-6	def2tzv	3.098	3.260	0.162
Cs	2-6	def2sv	2.950	2.998	0.048
Cs	2-6	def2tzv	3.011	3.067	0.056
Cs	3-6	def2sv	5.088	5.160	0.072
Cs	3-6	def2tzv	5.434	5.478	0.043
Cs	4-6	def2sv	5.029	5.077	0.047
Cs	4-6	def2tzv	5.158	5.297	0.139
Cs	5-6	def2sv	5.934	5.856	0.078
Cs	5-6	def2tzv	6.196	6.177	0.019

Table S3. Mean and RMSD Values of Oxygen Pair Distances

			ACE 1		
**Metal**	**Atom Pair**	**Basis**	$\bar{x}_{i,j,k,l}$	$\bar{x}_{i,j,k,N\;M}$	$R\;\;D_{i,j,k,l}$
Li	8-15	def2sv	2.688	2.856	0.168
Li	8-15	def2tzv	2.725	2.957	0.232
Na	8-15	def2sv	2.834	2.856	0.022
Na	8-15	def2tzv	2.935	2.957	0.022
K	8-15	def2sv	2.844	2.856	0.012
Rb	8-15	def2sv	2.880	2.856	0.024
Cs	8-15	def2sv	2.911	2.856	0.055
			ACE 2		
Li	1-2	def2sv	3.106	3.141	0.035
Li	1-2	def2tzv	3.153	3.463	0.310
Li	1-3	def2sv	2.731	2.899	0.168
Li	1-3	def2tzv	2.771	3.008	0.238
Li	1-4	def2sv	2.818	2.932	0.114
Li	1-4	def2tzv	2.843	3.028	0.184
Li	2-3	def2sv	2.627	2.879	0.253
Li	2-3	def2tzv	2.657	2.980	0.323
Li	2-4	def2sv	2.744	2.933	0.189
Li	2-4	def2tzv	2.757	3.026	0.269
Li	3-4	def2sv	4.203	4.541	0.338
Li	3-4	def2tzv	4.222	4.474	0.253
Na	1-2	def2sv	3.088	3.141	0.053
Na	1-2	def2tzv	3.163	3.463	0.300
Na	1-3	def2sv	2.831	2.899	0.068
Na	1-3	def2tzv	2.895	3.008	0.113
Na	1-4	def2sv	2.918	2.932	0.013
Na	1-4	def2tzv	2.981	3.028	0.047
Na	2-3	def2sv	2.708	2.879	0.171
Na	2-3	def2tzv	2.761	2.980	0.219
Na	2-4	def2sv	2.831	2.933	0.103
Na	2-4	def2tzv	2.863	3.026	0.163
Na	3-4	def2sv	4.438	4.541	0.103

Na	3-4	def2tzv	4.482	4.474	0.008
K	1-2	def2sv	3.038	3.141	0.103
K	1-2	def2tzv	3.137	3.463	0.327
K	1-3	def2sv	2.881	2.899	0.018
K	1-3	def2tzv	2.963	3.008	0.045
K	1-4	def2sv	2.967	2.932	0.035
K	1-4	def2tzv	3.042	3.028	0.014
K	2-3	def2sv	2.748	2.879	0.131
K	2-3	def2tzv	2.831	2.980	0.149
K	2-4	def2sv	2.869	2.933	0.064
K	2-4	def2tzv	2.933	3.026	0.092
K	3-4	def2sv	4.603	4.541	0.062
K	3-4	def2tzv	4.663	4.474	0.188
Rb	1-2	def2sv	3.028	3.141	0.113
Rb	1-2	def2tzv	3.133	3.463	0.330
Rb	1-3	def2sv	2.900	2.899	0.001
Rb	1-3	def2tzv	2.973	3.008	0.035
Rb	1-4	def2sv	2.978	2.932	0.046
Rb	1-4	def2tzv	3.048	3.028	0.021
Rb	2-3	def2sv	2.768	2.879	0.111
Rb	2-3	def2tzv	2.846	2.980	0.134
Rb	2-4	def2sv	2.888	2.933	0.046
Rb	2-4	def2tzv	2.943	3.026	0.083
Rb	3-4	def2sv	4.644	4.541	0.103
Rb	3-4	def2tzv	4.693	4.474	0.218
Cs	1-2	def2sv	3.021	3.141	0.120
Cs	1-2	def2tzv	3.109	3.463	0.354
Cs	1-3	def2sv	2.913	2.899	0.013
Cs	1-3	def2tzv	2.974	3.008	0.034
Cs	1-4	def2sv	2.991	2.932	0.059
Cs	1-4	def2tzv	3.058	3.028	0.031
Cs	2-3	def2sv	2.784	2.879	0.095
Cs	2-3	def2tzv	2.851	2.980	0.129
Cs	2-4	def2sv	2.897	2.933	0.037
Cs	2-4	def2tzv	2.946	3.026	0.080
Cs	3-4	def2sv	4.683	4.541	0.142
Cs	3-4	def2tzv	4.732	4.474	0.258
ACE 3					
Li	1-2	def2sv	3.614	3.869	0.255
Li	1-2	def2tzv	3.482	4.062	0.580
Li	1-3	def2sv	2.685	2.898	0.213
Li	1-3	def2tzv	2.785	2.992	0.207
Li	1-4	def2sv	4.001	4.453	0.452
Li	1-4	def2tzv	3.695	4.640	0.945
Li	1-5	def2sv	2.772	2.961	0.189
Li	1-5	def2tzv	2.797	3.051	0.254
Li	2-3	def2sv	4.291	4.368	0.077

Li	2-3	def2tzv	4.616	4.513	0.103
Li	2-4	def2sv	2.674	2.874	0.200
Li	2-4	def2tzv	2.677	2.978	0.302
Li	2-5	def2sv	2.782	2.944	0.163
Li	2-5	def2tzv	2.782	3.040	0.258
Li	3-4	def2sv	2.783	2.889	0.107
Li	3-4	def2tzv	2.897	2.990	0.093
Li	3-5	def2sv	4.623	4.994	0.372
Li	3-5	def2tzv	4.868	5.094	0.226
Li	4-5	def2sv	4.396	4.929	0.533
Li	4-5	def2tzv	4.272	5.020	0.748
Na	1-2	def2sv	3.870	3.869	0.001
Na	1-2	def2tzv	3.920	4.062	0.142
Na	1-3	def2sv	2.785	2.898	0.113
Na	1-3	def2tzv	2.814	2.992	0.178
Na	1-4	def2sv	4.376	4.453	0.077
Na	1-4	def2tzv	4.435	4.640	0.205
Na	1-5	def2sv	2.888	2.961	0.073
Na	1-5	def2tzv	2.918	3.051	0.133
Na	2-3	def2sv	4.241	4.368	0.127
Na	2-3	def2tzv	4.280	4.513	0.233
Na	2-4	def2sv	2.762	2.874	0.113
Na	2-4	def2tzv	2.800	2.978	0.178
Na	2-5	def2sv	2.880	2.944	0.064
Na	2-5	def2tzv	2.906	3.040	0.134
Na	3-4	def2sv	2.823	2.889	0.066
Na	3-4	def2tzv	2.855	2.990	0.135
Na	3-5	def2sv	4.759	4.994	0.235
Na	3-5	def2tzv	4.792	5.094	0.302
Na	4-5	def2sv	4.729	4.929	0.200
Na	4-5	def2tzv	4.759	5.020	0.261
K	1-2	def2sv	4.946	3.869	1.077
K	1-2	def2tzv	5.128	4.062	1.067
K	1-3	def2sv	2.783	2.898	0.116
K	1-3	def2tzv	2.916	2.992	0.076
K	1-4	def2sv	5.020	4.453	0.568
K	1-4	def2tzv	5.213	4.640	0.573
K	1-5	def2sv	2.803	2.961	0.158
K	1-5	def2tzv	2.878	3.051	0.173
K	2-3	def2sv	4.209	4.368	0.158
K	2-3	def2tzv	4.325	4.513	0.188
K	2-4	def2sv	2.809	2.874	0.065
K	2-4	def2tzv	2.868	2.978	0.111
K	2-5	def2sv	2.843	2.944	0.101
K	2-5	def2tzv	2.900	3.040	0.140
K	3-4	def2sv	2.787	2.889	0.103
K	3-4	def2tzv	2.862	2.990	0.128
K	3-5	def2sv	3.825	4.994	1.169

K	3-5	def2tzv	3.897	5.094	1.198
K	4-5	def2sv	4.428	4.929	0.502
K	4-5	def2tzv	4.493	5.020	0.527
Rb	1-2	def2sv	5.032	3.869	1.163
Rb	1-2	def2tzv	5.131	4.062	1.070
Rb	1-3	def2sv	2.803	2.898	0.096
Rb	1-3	def2tzv	2.927	2.992	0.065
Rb	1-4	def2sv	5.114	4.453	0.662
Rb	1-4	def2tzv	5.211	4.640	0.571
Rb	1-5	def2sv	2.829	2.961	0.132
Rb	1-5	def2tzv	2.878	3.051	0.173
Rb	2-3	def2sv	4.203	4.368	0.164
Rb	2-3	def2tzv	4.354	4.513	0.158
Rb	2-4	def2sv	2.814	2.874	0.060
Rb	2-4	def2tzv	2.868	2.978	0.110
Rb	2-5	def2sv	2.853	2.944	0.091
Rb	2-5	def2tzv	2.901	3.040	0.139
Rb	3-4	def2sv	2.821	2.889	0.068
Rb	3-4	def2tzv	2.876	2.990	0.114
Rb	3-5	def2sv	3.762	4.994	1.233
Rb	3-5	def2tzv	3.921	5.094	1.173
Rb	4-5	def2sv	4.426	4.929	0.503
Rb	4-5	def2tzv	4.489	5.020	0.531
Cs	1-2	def2sv	4.945	3.869	1.076
Cs	1-2	def2tzv	5.144	4.062	1.082
Cs	1-3	def2sv	2.815	2.898	0.083
Cs	1-3	def2tzv	2.945	2.992	0.047
Cs	1-4	def2sv	4.920	4.453	0.468
Cs	1-4	def2tzv	5.172	4.640	0.532
Cs	1-5	def2sv	2.803	2.961	0.158
Cs	1-5	def2tzv	2.874	3.051	0.177
Cs	2-3	def2sv	4.293	4.368	0.074
Cs	2-3	def2tzv	4.375	4.513	0.137
Cs	2-4	def2sv	2.816	2.874	0.058
Cs	2-4	def2tzv	2.872	2.978	0.106
Cs	2-5	def2sv	2.860	2.944	0.084
Cs	2-5	def2tzv	2.905	3.040	0.135
Cs	3-4	def2sv	2.842	2.889	0.047
Cs	3-4	def2tzv	2.895	2.990	0.095
Cs	3-5	def2sv	3.926	4.994	1.068
Cs	3-5	def2tzv	3.938	5.094	1.156
Cs	4-5	def2sv	4.349	4.929	0.580
Cs	4-5	def2tzv	4.441	5.020	0.579
			ACE 3		
Li	1-2	def2sv	3.867	4.401	0.534
Li	1-2	def2tzv	4.497	4.522	0.025
Li	1-3	def2sv	2.699	2.870	0.171

Li	1-3	def2tzv	2.726	2.996	0.270
Li	1-4	def2sv	3.465	4.713	1.248
Li	1-4	def2tzv	4.491	4.898	0.407
Li	1-5	def2sv	3.344	4.347	1.003
Li	1-5	def2tzv	4.045	4.514	0.469
Li	1-6	def2sv	3.205	3.153	0.052
Li	1-6	def2tzv	3.504	3.260	0.244
Li	2-3	def2sv	5.813	5.857	0.044
Li	2-3	def2tzv	6.344	6.048	0.296
Li	2-4	def2sv	2.896	2.879	0.017
Li	2-4	def2tzv	3.002	2.982	0.020
Li	2-5	def2sv	5.177	4.996	0.181
Li	2-5	def2tzv	5.320	5.163	0.158
Li	2-6	def2sv	2.908	2.998	0.090
Li	2-6	def2tzv	2.988	3.067	0.079
Li	3-4	def2sv	4.248	4.965	0.718
Li	3-4	def2tzv	5.134	5.136	0.002
Li	3-5	def2sv	2.704	2.891	0.187
Li	3-5	def2tzv	2.937	2.985	0.048
Li	3-6	def2sv	5.605	5.160	0.445
Li	3-6	def2tzv	5.743	5.478	0.266
Li	4-5	def2sv	2.669	2.879	0.210
Li	4-5	def2tzv	2.994	2.974	0.020
Li	4-6	def2sv	4.843	5.077	0.233
Li	4-6	def2tzv	5.118	5.297	0.179
Li	5-6	def2sv	6.064	5.856	0.208
Li	5-6	def2tzv	6.120	6.177	0.057
Na	1-2	def2sv	3.695	4.401	0.706
Na	1-2	def2tzv	3.947	4.522	0.575
Na	1-3	def2sv	2.796	2.870	0.074
Na	1-3	def2tzv	2.787	2.996	0.209
Na	1-4	def2sv	4.243	4.713	0.470
Na	1-4	def2tzv	3.782	4.898	1.116
Na	1-5	def2sv	4.144	4.347	0.203
Na	1-5	def2tzv	3.610	4.514	0.904
Na	1-6	def2sv	2.940	3.153	0.213
Na	1-6	def2tzv	3.206	3.260	0.054
Na	2-3	def2sv	4.921	5.857	0.936
Na	2-3	def2tzv	5.960	6.048	0.088
Na	2-4	def2sv	2.776	2.879	0.103
Na	2-4	def2tzv	2.973	2.982	0.008
Na	2-5	def2sv	4.596	4.996	0.400
Na	2-5	def2tzv	5.268	5.163	0.106
Na	2-6	def2sv	2.911	2.998	0.088
Na	2-6	def2tzv	3.014	3.067	0.052
Na	3-4	def2sv	4.439	4.965	0.526
Na	3-4	def2tzv	4.534	5.136	0.602
Na	3-5	def2sv	2.753	2.891	0.138

Na	3-5	def2tzv	2.821	2.985	0.164
Na	3-6	def2sv	4.193	5.160	0.967
Na	3-6	def2tzv	5.718	5.478	0.240
Na	4-5	def2sv	2.733	2.879	0.147
Na	4-5	def2tzv	2.796	2.974	0.178
Na	4-6	def2sv	4.794	5.077	0.283
Na	4-6	def2tzv	5.103	5.297	0.194
Na	5-6	def2sv	5.190	5.856	0.666
Na	5-6	def2tzv	6.301	6.177	0.124
K	1-2	def2sv	4.293	4.401	0.108
K	1-2	def2tzv	4.428	4.522	0.093
K	1-3	def2sv	2.773	2.870	0.097
K	1-3	def2tzv	2.826	2.996	0.170
K	1-4	def2sv	4.753	4.713	0.041
K	1-4	def2tzv	4.755	4.898	0.143
K	1-5	def2sv	4.451	4.347	0.104
K	1-5	def2tzv	4.387	4.514	0.128
K	1-6	def2sv	2.864	3.153	0.289
K	1-6	def2tzv	2.965	3.260	0.295
K	2-3	def2sv	5.462	5.857	0.395
K	2-3	def2tzv	5.717	6.048	0.332
K	2-4	def2sv	2.827	2.879	0.052
K	2-4	def2tzv	2.897	2.982	0.085
K	2-5	def2sv	4.879	4.996	0.117
K	2-5	def2tzv	5.000	5.163	0.163
K	2-6	def2sv	2.918	2.998	0.080
K	2-6	def2tzv	2.974	3.067	0.092
K	3-4	def2sv	4.719	4.965	0.246
K	3-4	def2tzv	4.827	5.136	0.309
K	3-5	def2sv	2.820	2.891	0.071
K	3-5	def2tzv	2.892	2.985	0.093
K	3-6	def2sv	4.734	5.160	0.426
K	3-6	def2tzv	4.998	5.478	0.479
K	4-5	def2sv	2.816	2.879	0.063
K	4-5	def2tzv	2.865	2.974	0.109
K	4-6	def2sv	4.959	5.077	0.118
K	4-6	def2tzv	5.023	5.297	0.274
K	5-6	def2sv	5.721	5.856	0.135
K	5-6	def2tzv	5.838	6.177	0.339
Rb	1-2	def2sv	4.246	4.401	0.155
Rb	1-2	def2tzv	4.421	4.522	0.101
Rb	1-3	def2sv	2.835	2.870	0.035
Rb	1-3	def2tzv	2.878	2.996	0.118
Rb	1-4	def2sv	4.623	4.713	0.089
Rb	1-4	def2tzv	4.800	4.898	0.098
Rb	1-5	def2sv	4.346	4.347	0.001
Rb	1-5	def2tzv	4.478	4.514	0.037
Rb	1-6	def2sv	2.912	3.153	0.242

Rb	1-6	def2tzv	2.924	3.260	0.336
Rb	2-3	def2sv	5.634	5.857	0.223
Rb	2-3	def2tzv	5.713	6.048	0.336
Rb	2-4	def2sv	2.849	2.879	0.030
Rb	2-4	def2tzv	2.892	2.982	0.090
Rb	2-5	def2sv	4.959	4.996	0.037
Rb	2-5	def2tzv	5.019	5.163	0.143
Rb	2-6	def2sv	2.938	2.998	0.061
Rb	2-6	def2tzv	2.975	3.067	0.092
Rb	3-4	def2sv	4.809	4.965	0.156
Rb	3-4	def2tzv	4.896	5.136	0.240
Rb	3-5	def2sv	2.854	2.891	0.037
Rb	3-5	def2tzv	2.913	2.985	0.072
Rb	3-6	def2sv	4.947	5.160	0.213
Rb	3-6	def2tzv	4.957	5.478	0.521
Rb	4-5	def2sv	2.835	2.879	0.044
Rb	4-5	def2tzv	2.883	2.974	0.091
Rb	4-6	def2sv	4.993	5.077	0.084
Rb	4-6	def2tzv	5.033	5.297	0.264
Rb	5-6	def2sv	5.836	5.856	0.020
Rb	5-6	def2tzv	5.866	6.177	0.311
Cs	1-2	def2sv	4.208	4.401	0.193
Cs	1-2	def2tzv	4.290	4.522	0.232
Cs	1-3	def2sv	2.872	2.870	0.002
Cs	1-3	def2tzv	2.932	2.996	0.064
Cs	1-4	def2sv	4.565	4.713	0.148
Cs	1-4	def2tzv	4.550	4.898	0.348
Cs	1-5	def2sv	4.318	4.347	0.028
Cs	1-5	def2tzv	4.321	4.514	0.193
Cs	1-6	def2sv	2.950	3.153	0.203
Cs	1-6	def2tzv	3.098	3.260	0.162
Cs	2-3	def2sv	5.719	5.857	0.138
Cs	2-3	def2tzv	5.934	6.048	0.114
Cs	2-4	def2sv	2.860	2.879	0.019
Cs	2-4	def2tzv	2.938	2.982	0.044
Cs	2-5	def2sv	5.015	4.996	0.019
Cs	2-5	def2tzv	5.183	5.163	0.020
Cs	2-6	def2sv	2.950	2.998	0.048
Cs	2-6	def2tzv	3.011	3.067	0.056
Cs	3-4	def2sv	4.841	4.965	0.124
Cs	3-4	def2tzv	4.896	5.136	0.240
Cs	3-5	def2sv	2.871	2.891	0.020
Cs	3-5	def2tzv	2.918	2.985	0.067
Cs	3-6	def2sv	5.088	5.160	0.072
Cs	3-6	def2tzv	5.434	5.478	0.043
Cs	4-5	def2sv	2.850	2.879	0.029
Cs	4-5	def2tzv	2.900	2.974	0.074
Cs	4-6	def2sv	5.029	5.077	0.047

Cs	4-6	def2tzv	5.158	5.297	0.139
Cs	5-6	def2sv	5.934	5.856	0.078
Cs	5-6	def2tzv	6.196	6.177	0.019

Geometric Data (Section 2.10)

Table S4. RMSE Data Per Basis (Reference SCS-MP2/Basis)

ACE1 + Li										
	RMSE Binding Energy (kcal/mol)					RMSE Charge Transfer				
Level of Theory	SV	SVP	SV(P)	TZV	TZVP	SV	SVP	SV(P)	TZV	TZVP
B2PLYP	2.364	1.044	2.363	2.044	0.496	0.016	0.016	0.016	0.017	0.016
B3LYP	2.397	1.213	2.397	0.364	0.221	0.032	0.031	0.032	0.032	0.030
B971	3.433	2.232	3.433	0.028	0.822	0.027	0.026	0.027	0.032	0.031
CAM-B3LYP	0.133	1.324	0.133	2.952	1.946	0.011	0.010	0.011	0.011	0.009
DSDPBEP86	1.011	0.355	1.011	4.385	2.182	0.008	0.008	0.008	0.009	0.008
HSE06	2.977	1.758	2.977	0.869	0.247	0.030	0.030	0.030	0.030	0.029
M06	5.868	4.817	5.868	0.726	3.100	0.034	0.034	0.034	0.038	0.034
M062X	0.989	0.429	0.989	3.845	1.948	0.016	0.016	0.016	0.016	0.015
PBE	3.412	2.275	3.412	1.105	1.278	0.039	0.039	0.039	0.039	0.037
PBE0	3.089	1.851	3.089	0.834	0.291	0.025	0.024	0.025	0.025	0.023
ACE1 + Na										
	RMSE Binding Energy (kcal/mol)					RMSE Charge Transfer				
Level of Theory	SV	SVP	SV(P)	TZV	TZVP	SV	SVP	SV(P)	TZV	TZVP
B2PLYP	2.539	2.306	2.538	0.187	0.625	0.020	0.020	0.020	0.018	0.017
B3LYP	0.541	0.343	0.541	0.201	0.697	0.042	0.041	0.042	0.037	0.035
B971	2.925	2.764	2.925	2.241	3.439	0.032	0.031	0.032	0.029	0.027
CAM-B3LYP	1.966	1.694	1.966	0.184	0.688	0.011	0.011	0.011	0.009	0.007
DSDPBEP86	0.694	0.902	0.695	1.036	0.631	0.009	0.008	0.009	0.008	0.007
HSE06	2.126	1.876	2.126	2.218	3.509	0.039	0.039	0.039	0.036	0.033
M06	0.818	0.557	0.815	0.763	2.200	0.054	0.053	0.054	0.046	0.039
M062X	1.212	0.899	1.212	1.267	2.669	0.022	0.022	0.022	0.019	0.017
PBE	1.761	1.535	1.760	4.068	5.262	0.053	0.052	0.053	0.047	0.044
PBE0	0.475	0.285	0.475	0.115	0.661	0.030	0.030	0.030	0.028	0.025
ACE2 + Li										
	RMSE Binding Energy (kcal/mol)					RMSE Charge Transfer				
Level of Theory	SV	SVP	SV(P)	TZV	TZVP	SV	SVP	SV(P)	TZV	TZVP
B2PLYP	2.965	1.276	2.965	3.946	0.894	0.016	0.016	0.016	0.017	0.016
B3LYP	3.420	1.922	3.420	1.322	0.315	0.031	0.031	0.032	0.031	0.030
B971	4.841	3.322	4.841	0.607	1.310	0.027	0.026	0.027	0.032	0.031
CAM-B3LYP	0.209	1.297	0.209	4.675	2.427	0.011	0.010	0.011	0.011	0.009
DSDPBEP86	1.075	0.663	1.075	6.961	3.095	0.008	0.008	0.008	0.009	0.008
HSE06	4.104	2.569	4.104	1.848	0.445	0.030	0.030	0.030	0.030	0.028
M06	8.351	7.036	8.351	0.046	4.382	0.034	0.033	0.034	0.038	0.034
M062X	1.581	0.180	1.581	5.859	2.641	0.016	0.016	0.016	0.016	0.014
PBE	5.099	3.661	5.099	1.107	2.120	0.039	0.039	0.039	0.039	0.041
PBE0	4.284	2.724	4.284	1.743	0.567	0.024	0.024	0.024	0.025	0.023
ACE2 + Na										
	RMSE Binding Energy (kcal/mol)					RMSE Charge Transfer				
Level of Theory	SV	SVP	SV(P)	TZV	TZVP	SV	SVP	SV(P)	TZV	TZVP
B2PLYP	3.086	1.860	3.086	1.861	3.366	0.020	0.020	0.020	0.018	0.016
B3LYP	2.987	1.860	2.987	1.085	3.741	0.042	0.041	0.042	0.036	0.034

B971	3.807	2.676	3.807	0.653	4.018	0.031	0.031	0.031	0.029	0.027
CAM-B3LYP	0.179	1.310	0.179	4.188	1.140	0.011	0.010	0.011	0.009	0.006
DSDPBEP86	0.501	0.740	0.501	4.944	0.503	0.009	0.008	0.009	0.008	0.007
HSE06	3.510	2.374	3.510	1.325	3.633	0.039	0.039	0.039	0.035	0.033
M06	4.960	3.961	4.960	0.404	5.884	0.053	0.052	0.053	0.045	0.038
M062X	0.166	1.462	0.167	4.992	0.343	0.021	0.021	0.021	0.019	0.017
PBE	3.717	2.625	3.717	0.197	4.305	0.053	0.052	0.053	0.047	0.051
PBE0	3.637	2.489	3.637	1.212	3.754	0.030	0.030	0.030	0.027	0.025

Table S5. RMSE Data Per Basis (Reference SCS-MP2/Basis)

ACE1 + Li										
	RMSE Binding Energy (kcal/mol)					RMSE Charge Transfer				
Level of Theory	SV	SVP	SV(P)	TZV	TZVP	SV	SVP	SV(P)	TZV	TZVP
B2PLYP	6.473	6.578	6.474	4.677	0.496	0.022	0.022	0.022	0.018	0.016
B3LYP	6.441	6.410	6.441	2.997	0.221	0.037	0.038	0.037	0.033	0.030
B971	5.405	5.390	5.405	2.661	0.822	0.032	0.033	0.032	0.034	0.031
CAM-B3LYP	8.971	8.947	8.971	5.585	1.946	0.016	0.017	0.016	0.012	0.009
DSDPBEP86	7.826	7.977	7.827	7.018	2.182	0.014	0.014	0.014	0.010	0.008
HSE06	5.861	5.864	5.861	3.501	0.247	0.036	0.036	0.036	0.032	0.029
M06	2.970	2.806	2.970	1.906	3.100	0.040	0.040	0.040	0.040	0.034
M062X	7.848	8.051	7.848	6.478	1.948	0.022	0.022	0.022	0.018	0.015
PBE	5.426	5.348	5.426	1.528	1.278	0.045	0.045	0.045	0.040	0.037
PBE0	5.749	5.771	5.749	3.466	0.291	0.030	0.031	0.030	0.026	0.023
SCS-MP2	8.838	7.622	8.838	2.633	0.000	0.006	0.006	0.006	0.002	0.000
ACE1 + Na										
	RMSE Binding Energy (kcal/mol)					RMSE Charge Transfer				
Level of Theory	SV	SVP	SV(P)	TZV	TZVP	SV	SVP	SV(P)	TZV	TZVP
B2PLYP	2.140	2.234	2.139	0.650	0.625	0.021	0.022	0.021	0.020	0.017
B3LYP	0.142	0.271	0.142	0.664	0.697	0.043	0.044	0.043	0.039	0.035
B971	2.526	2.691	2.526	2.704	3.439	0.033	0.034	0.033	0.032	0.027
CAM-B3LYP	1.567	1.621	1.567	0.647	0.688	0.013	0.013	0.013	0.011	0.007
DSDPBEP86	1.093	0.974	1.093	0.574	0.631	0.010	0.011	0.010	0.010	0.007
HSE06	1.727	1.803	1.728	2.681	3.509	0.041	0.041	0.041	0.038	0.033
M06	0.420	0.484	0.416	1.226	2.200	0.055	0.055	0.055	0.048	0.039
M062X	0.813	0.827	0.813	1.729	2.669	0.023	0.024	0.023	0.022	0.017
PBE	1.362	1.463	1.361	4.531	5.262	0.054	0.055	0.054	0.049	0.044
PBE0	0.076	0.212	0.076	0.578	0.661	0.032	0.032	0.032	0.030	0.025
SCS-MP2	0.399	0.073	0.399	0.463	0.000	0.001	0.002	0.001	0.002	0.000
ACE2 + Li										
	RMSE Binding Energy (kcal/mol)					RMSE Charge Transfer				
Level of Theory	SV	SVP	SV(P)	TZV	TZVP	SV	SVP	SV(P)	TZV	TZVP
B2PLYP	9.571	9.577	9.571	6.809	0.894	0.022	0.022	0.022	0.018	0.016
B3LYP	9.116	8.930	9.116	4.185	0.315	0.037	0.037	0.037	0.033	0.030
B971	7.696	7.531	7.696	3.469	1.310	0.032	0.032	0.032	0.033	0.031
CAM-B3LYP	12.328	12.150	12.328	7.538	2.427	0.016	0.016	0.016	0.012	0.009

DSDPBEP86	11.461	11.515	11.461	9.823	3.095	0.014	0.014	0.014	0.010	0.008
HSE06	8.432	8.283	8.432	4.711	0.445	0.035	0.036	0.035	0.032	0.028
M06	4.186	3.817	4.186	2.908	4.382	0.040	0.040	0.040	0.039	0.034
M062X	10.955	11.033	10.955	8.721	2.641	0.022	0.022	0.022	0.017	0.014
PBE	7.437	7.191	7.437	1.756	2.120	0.045	0.045	0.045	0.040	0.041
PBE0	8.252	8.128	8.252	4.605	0.567	0.030	0.030	0.030	0.026	0.023
SCS-MP2	12.537	10.853	12.537	2.862	0.000	0.005	0.006	0.005	0.001	0.000

ACE2 + Na										
	RMSE Binding Energy (kcal/mol)					RMSE Charge Transfer				
Level of Theory	SV	SVP	SV(P)	TZV	TZVP	SV	SVP	SV(P)	TZV	TZVP
B2PLYP	4.794	4.699	4.794	1.941	3.366	0.021	0.022	0.021	0.020	0.016
B3LYP	4.892	4.699	4.892	1.165	3.741	0.043	0.044	0.043	0.038	0.034
B971	4.073	3.883	4.073	0.733	4.018	0.033	0.033	0.033	0.031	0.027
CAM-B3LYP	8.059	7.868	8.059	4.268	1.140	0.012	0.013	0.012	0.011	0.006
DSDPBEP86	7.379	7.299	7.379	5.024	0.503	0.010	0.011	0.010	0.010	0.007
HSE06	4.370	4.184	4.370	1.405	3.633	0.041	0.041	0.041	0.037	0.033
M06	2.920	2.598	2.920	0.324	5.884	0.055	0.055	0.055	0.047	0.038
M062X	8.046	8.020	8.046	5.072	0.343	0.023	0.024	0.023	0.021	0.017
PBE	4.163	3.934	4.163	0.278	4.305	0.054	0.055	0.054	0.049	0.051
PBE0	4.243	4.070	4.243	1.292	3.754	0.031	0.032	0.031	0.029	0.025
SCS-MP2	7.880	6.559	7.880	0.080	0.000	0.001	0.002	0.001	0.002	0.000

Graphical Representation of Geometric Data (Section 2.2-2.4):

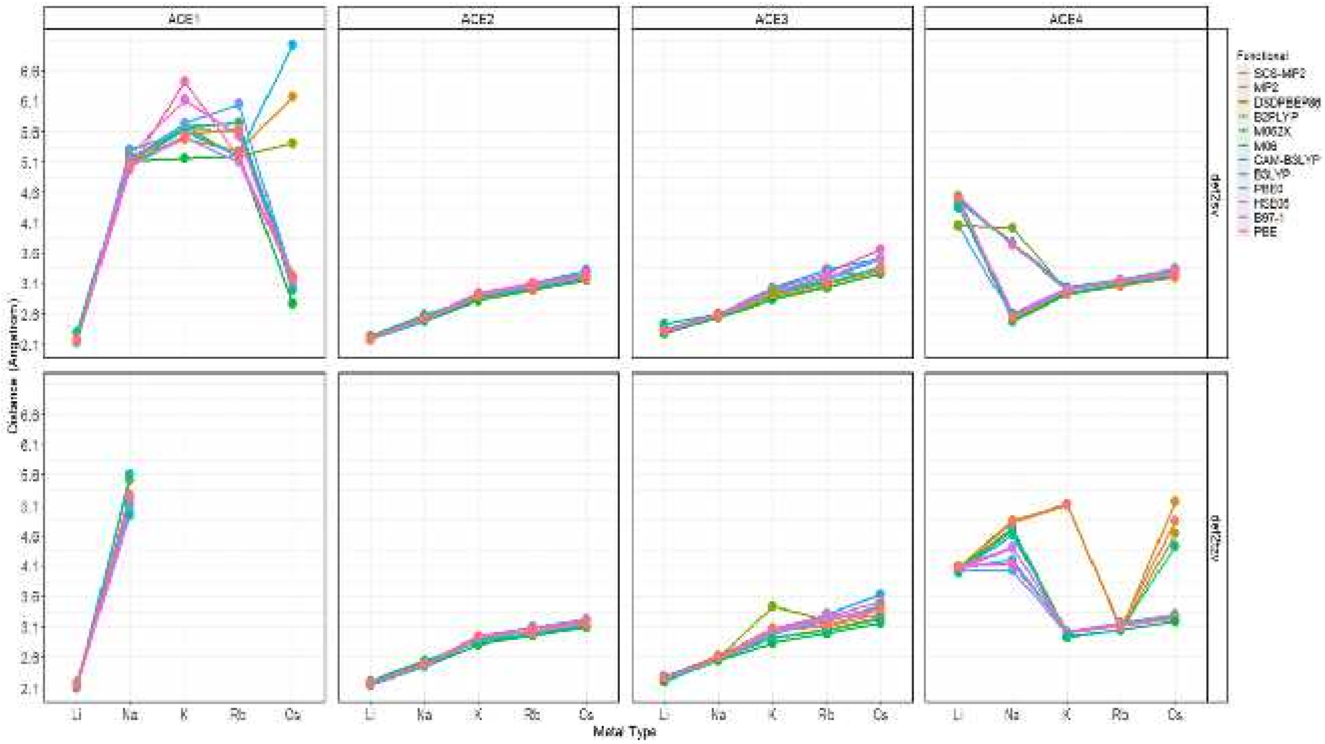

Figure S1. N-M$^+$ distances faceted by ACE type and basis set, colored by functional used

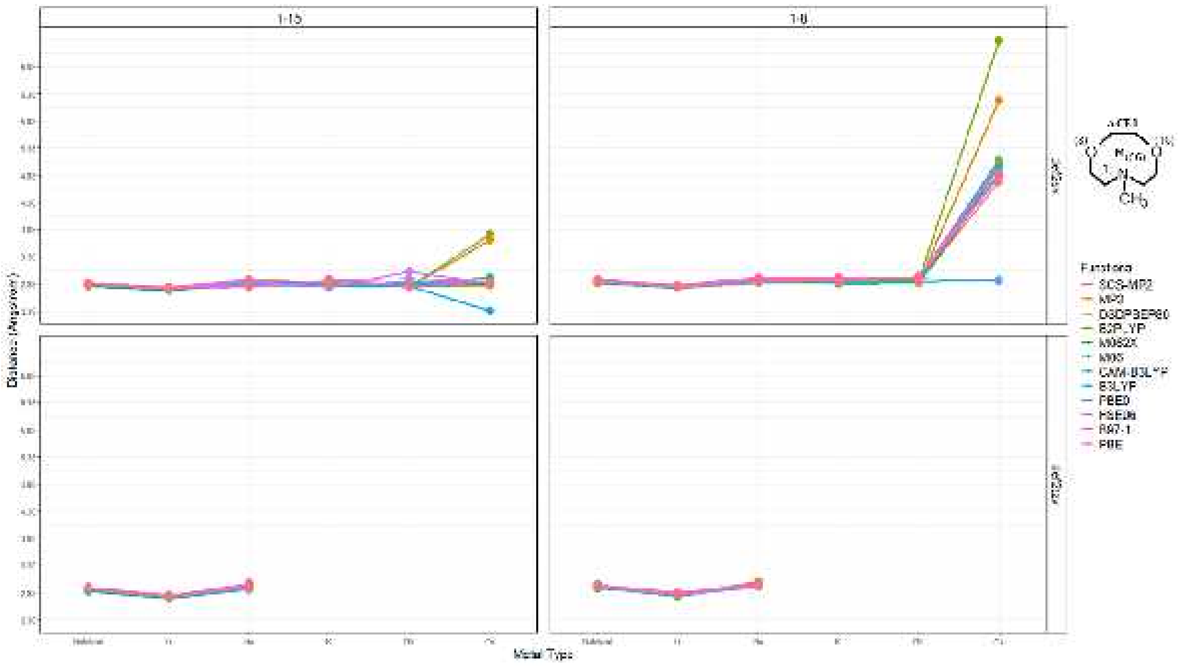

Figure S2. N-O_i distances in ACE1 molecule faceted by basis set, colored by functional

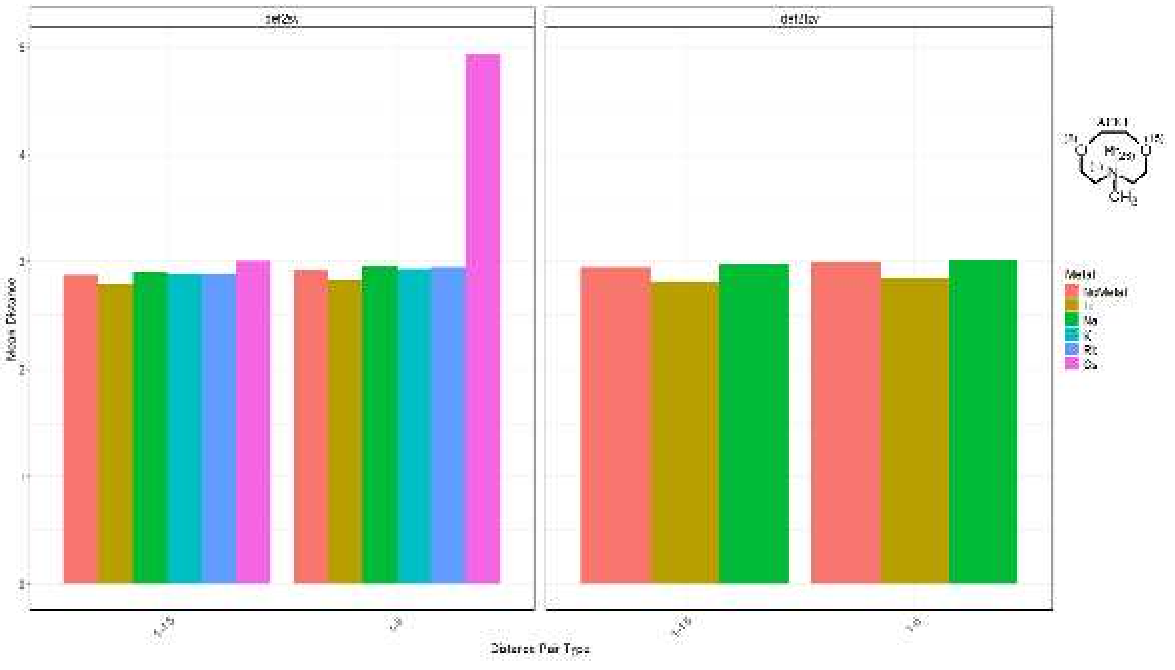

Figure S3. Mean Distance (Å) of N-O_i distances in ACE1 molecule faceted by basis and colored by metal ion type

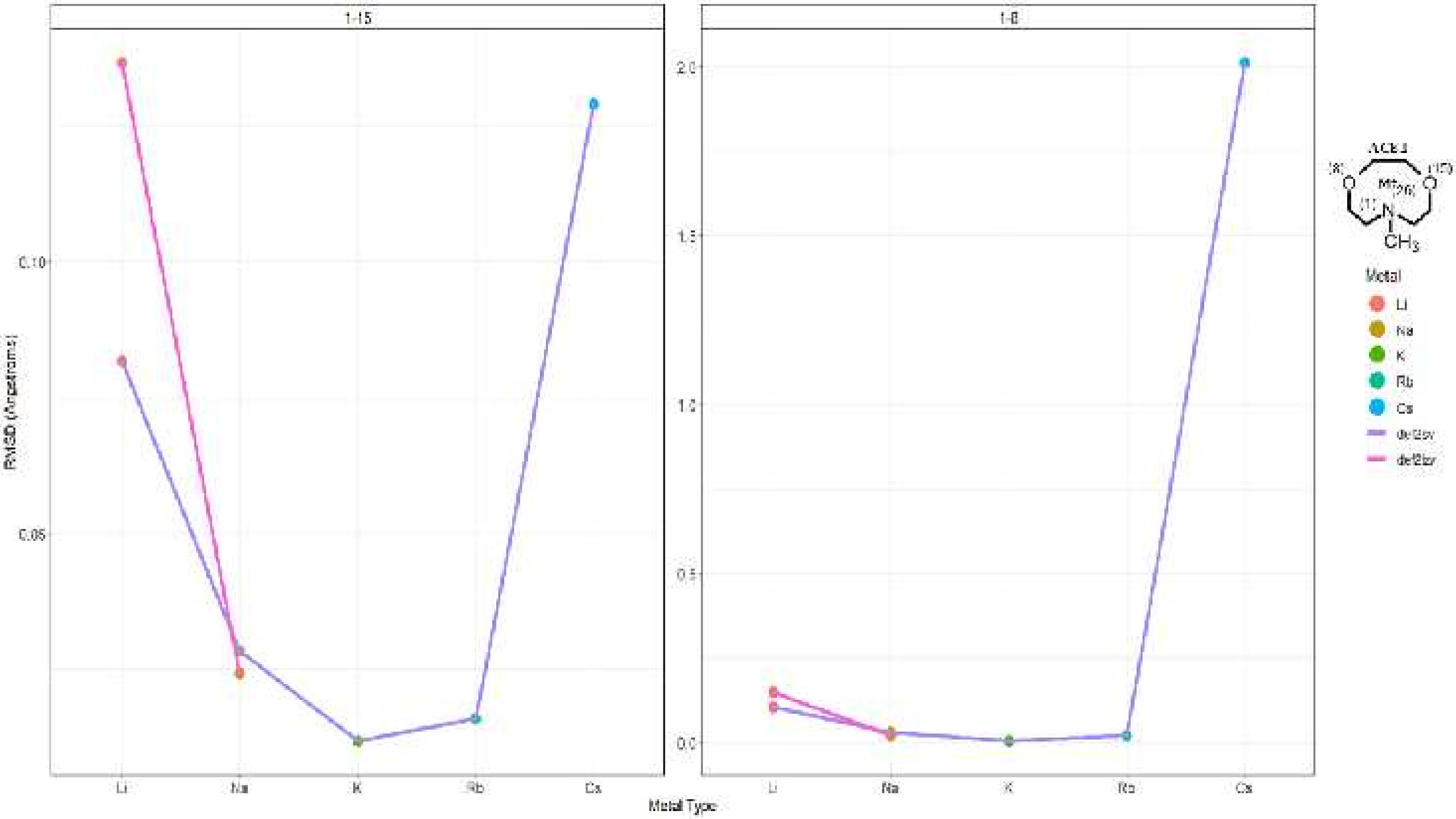

Figure S4. RMSD (Å) of N-O_i distances in ACE1 molecule faceted by atom pairs, colored by metal type and basis

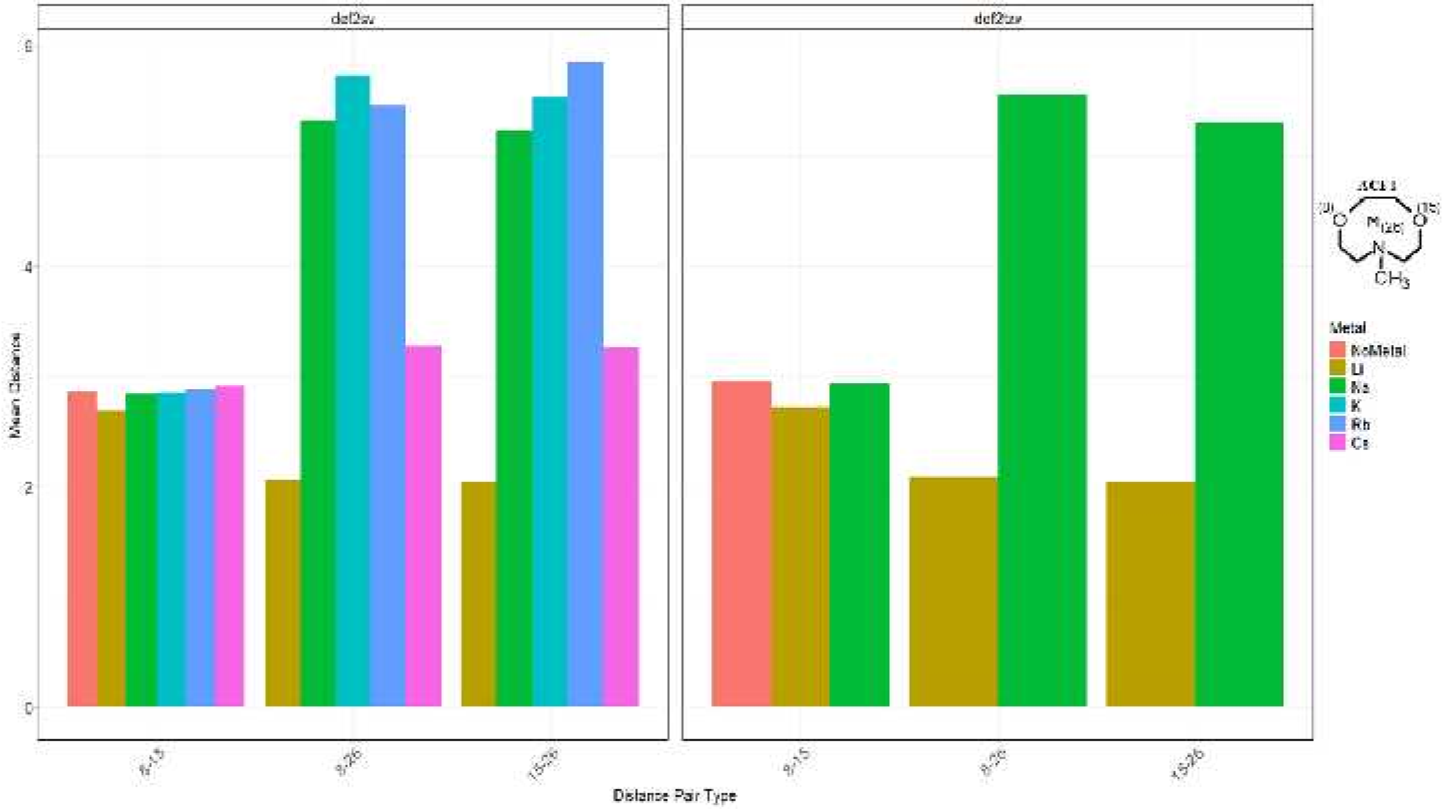

Figure S5. Mean Distance (Å) of O_i–O_j and O_i–M^+ in ACE1 molecule faceted by basis and colored by metal ion

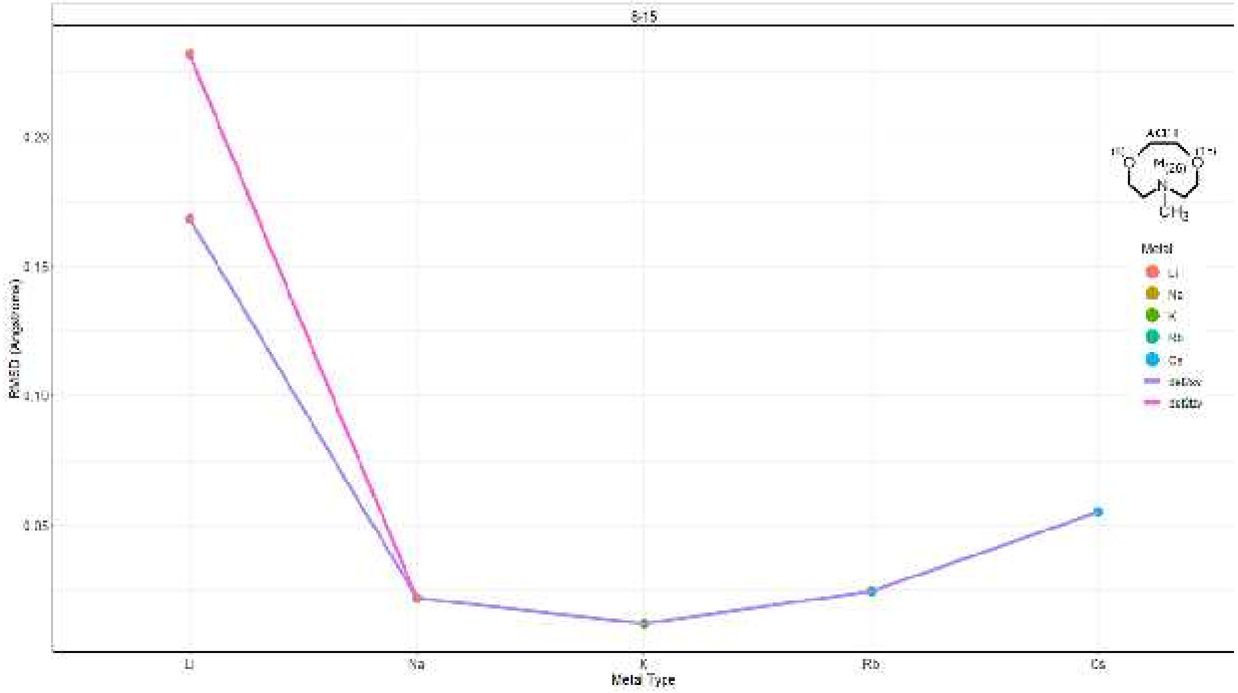

Figure S6. RMSD (Å) of O_i–O_j in ACE1 molecule faceted by Atom pairs and colored by metal ion (points) and basis (lines)

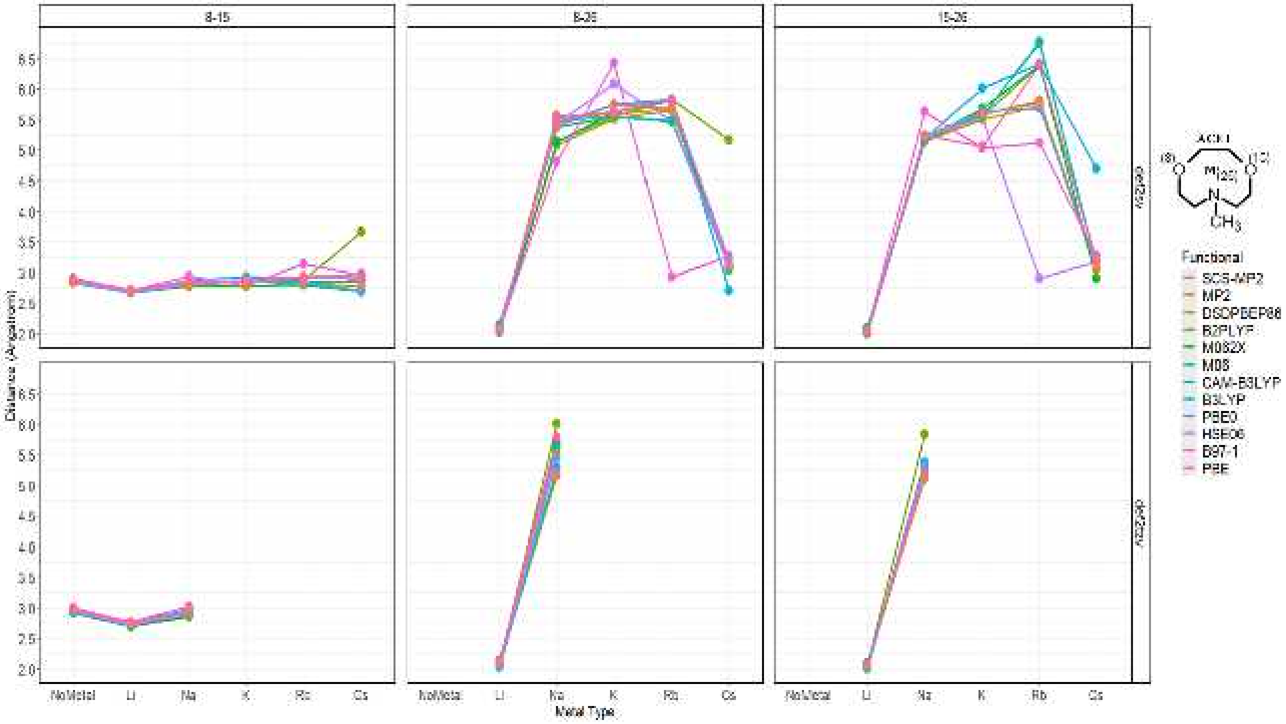

Figure S7. Explicit Distance (Å) of O_i–O_j and O_i–M^+ in ACE1 molecule faceted by basis and Atom pairs; colored by functional used

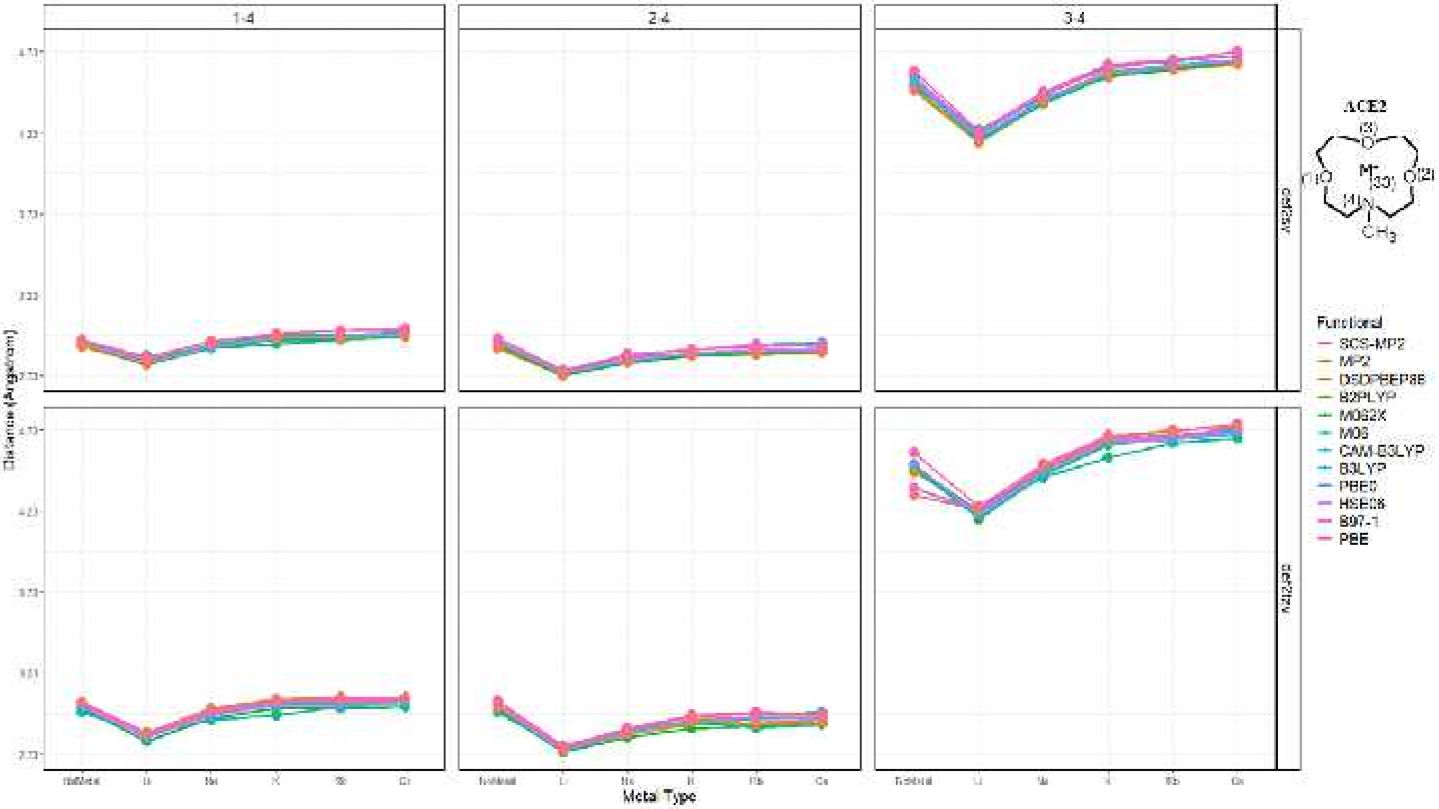

Figure S8. N-O_i distances in ACE2 molecule faceted by basis set, colored by functional

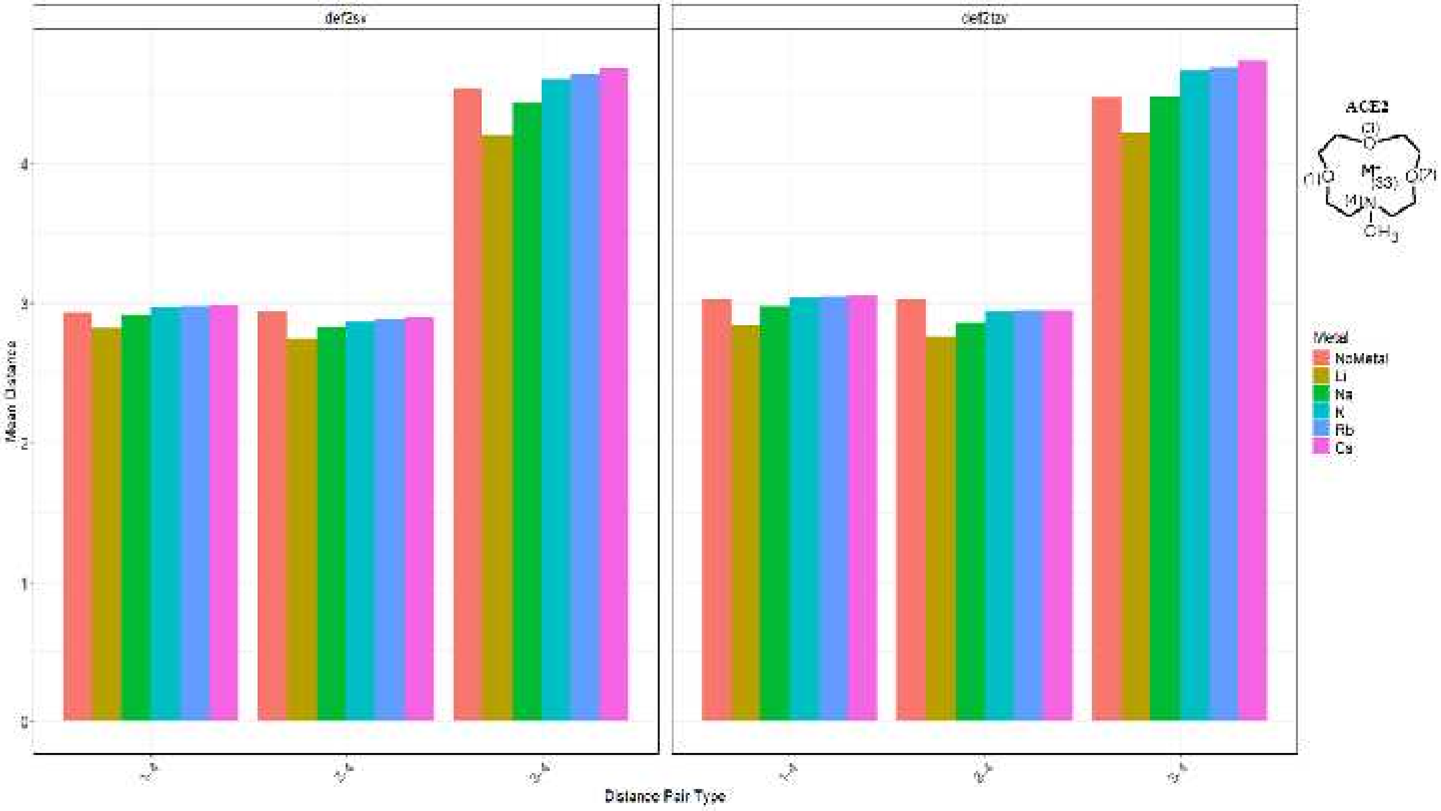

Figure S9. Mean Distance (Å) of N-O_i distances in ACE2 molecule faceted by basis and colored by metal ion type

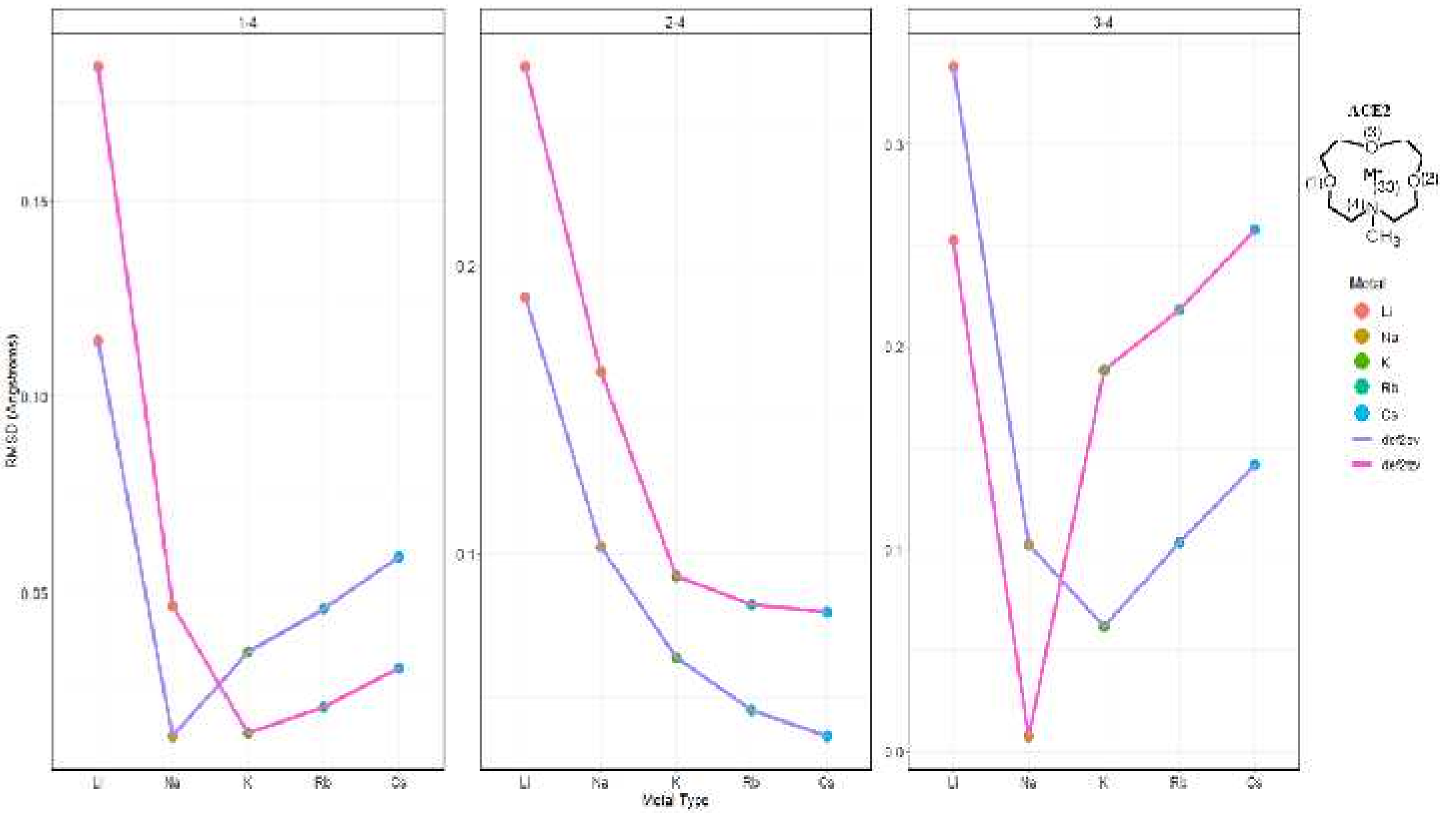

Figure S10. RMSD (Å) of N-O_i distances in ACE2 molecule faceted by atom pairs, colored by metal type and basis

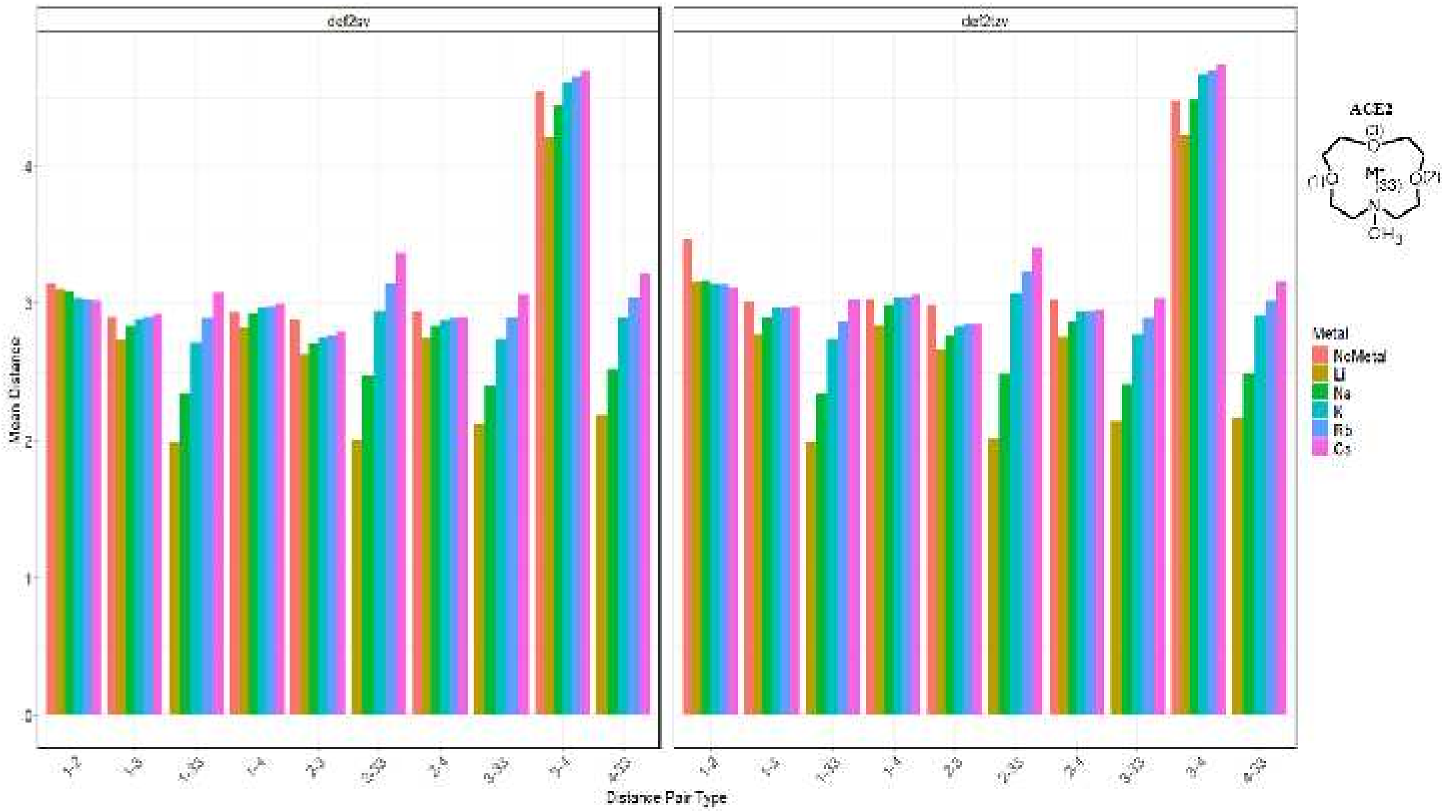

Figure S11. Mean Distance (Å) of O_i–O_j and O_i–M^+ in ACE2 molecule faceted by basis and colored by metal ion

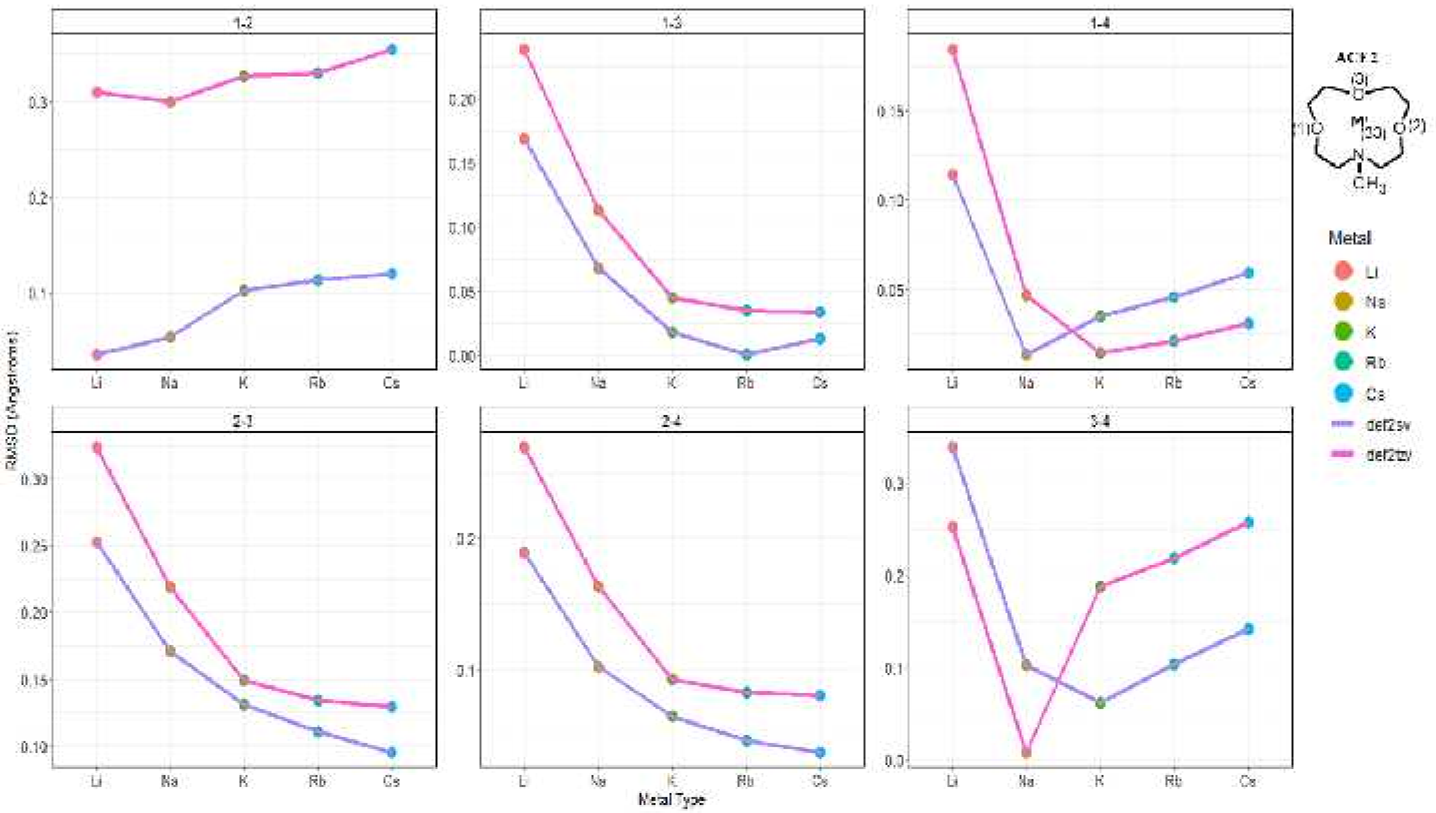

Figure S12. RMSD (Å) of O_i–O_j in ACE2 molecule faceted by Atom pairs and colored by metal ion (points) and basis (lines)

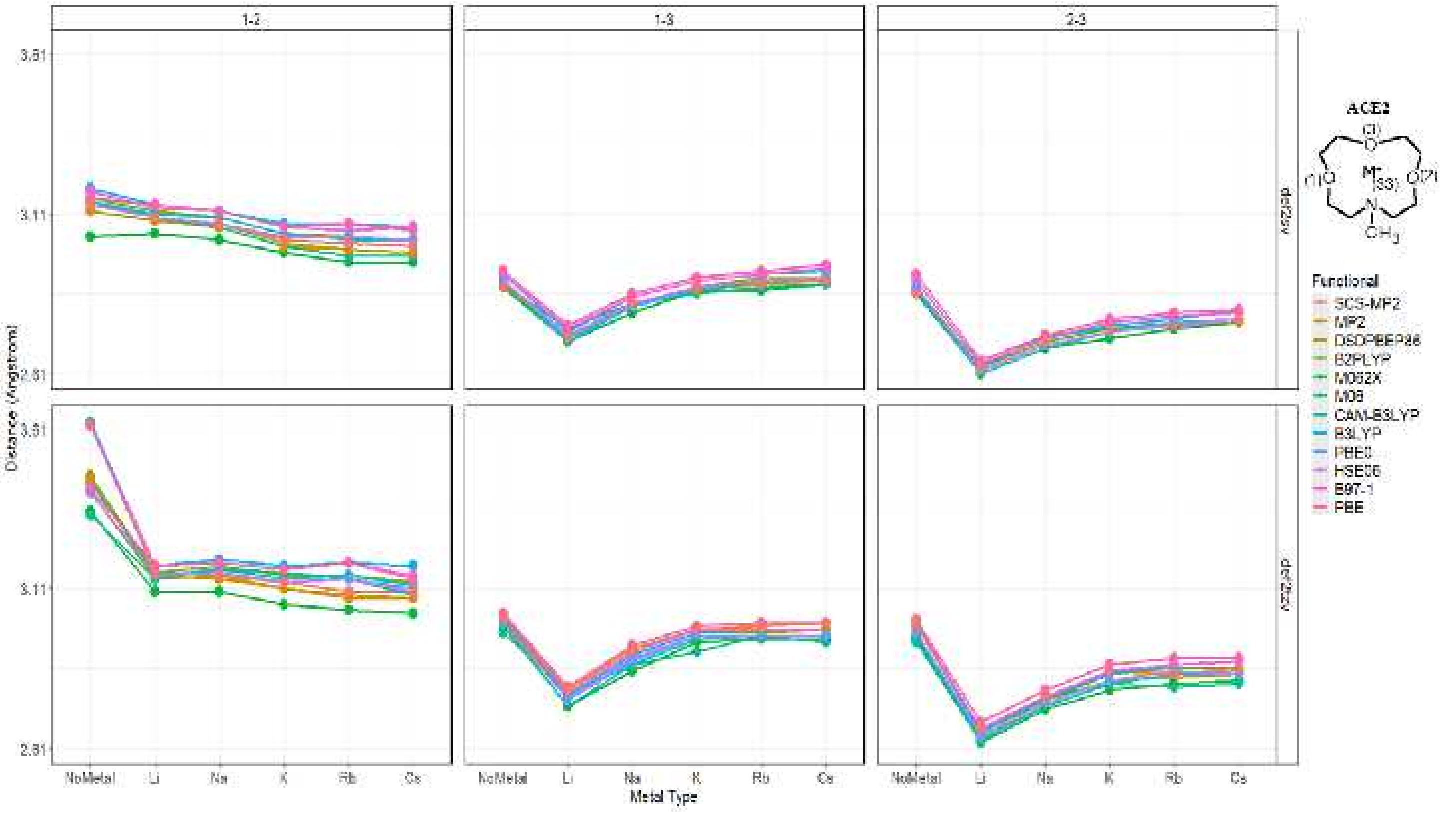

Figure S13. Explicit Distance (Å) of O_i–O_j in ACE2 molecule faceted by basis and Atom pairs; colored by functional used

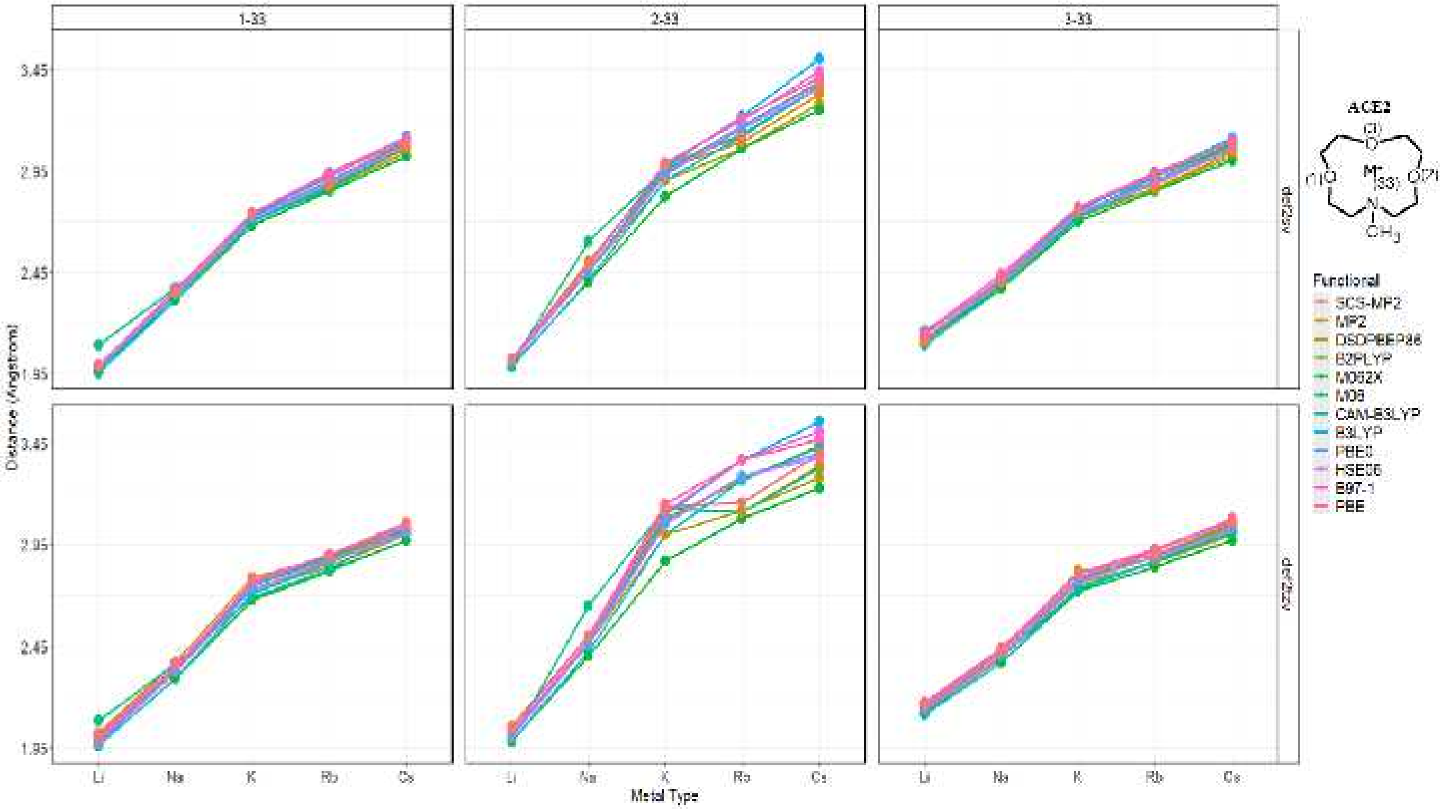

Figure S14. Explicit Distance (Å) of O_i–M^+ in ACE2 molecule faceted by basis and Atom pairs; colored by functional used

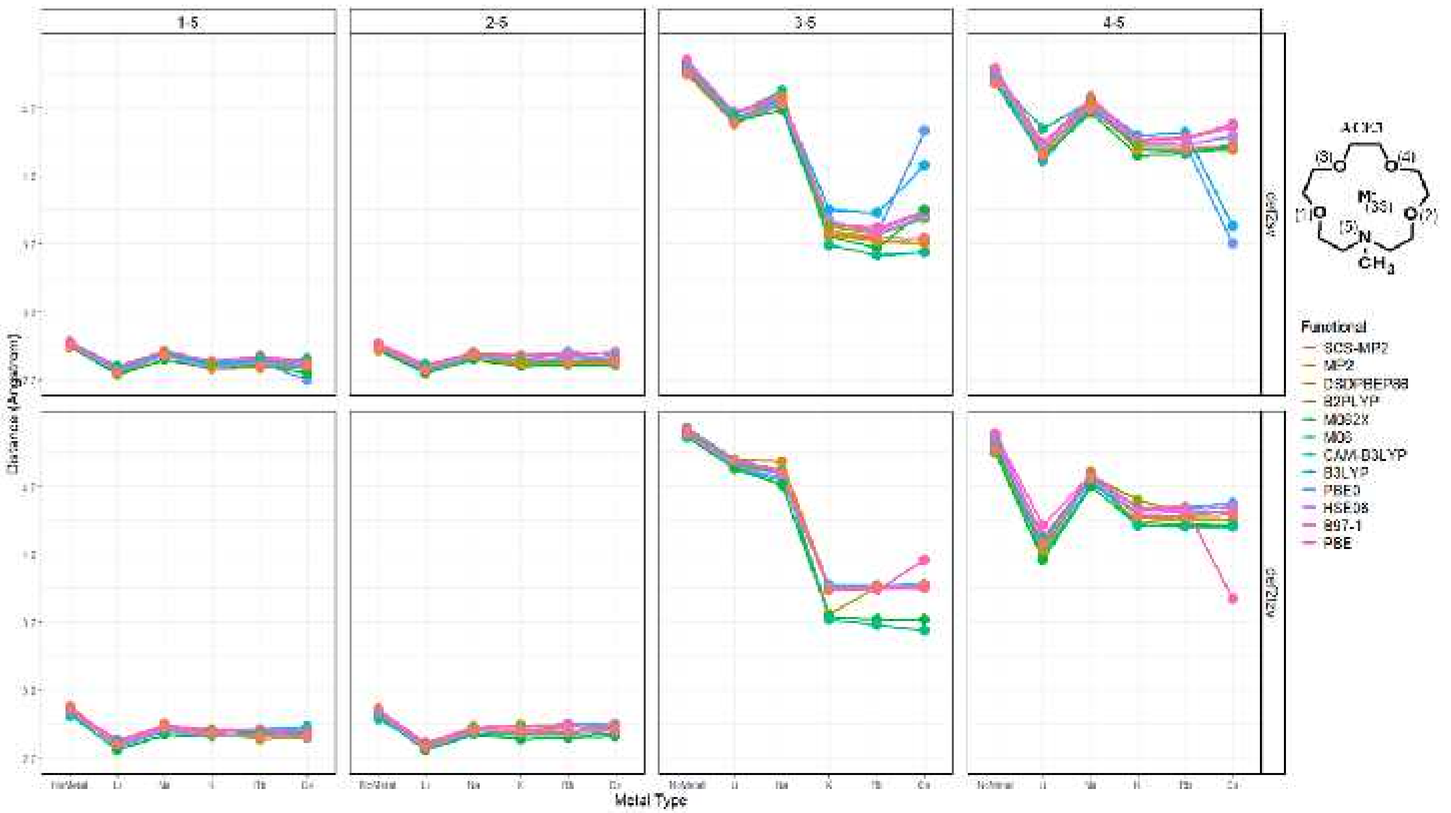

Figure S15. N-O$_i$ distances in ACE3 molecule faceted by basis set, colored by functional

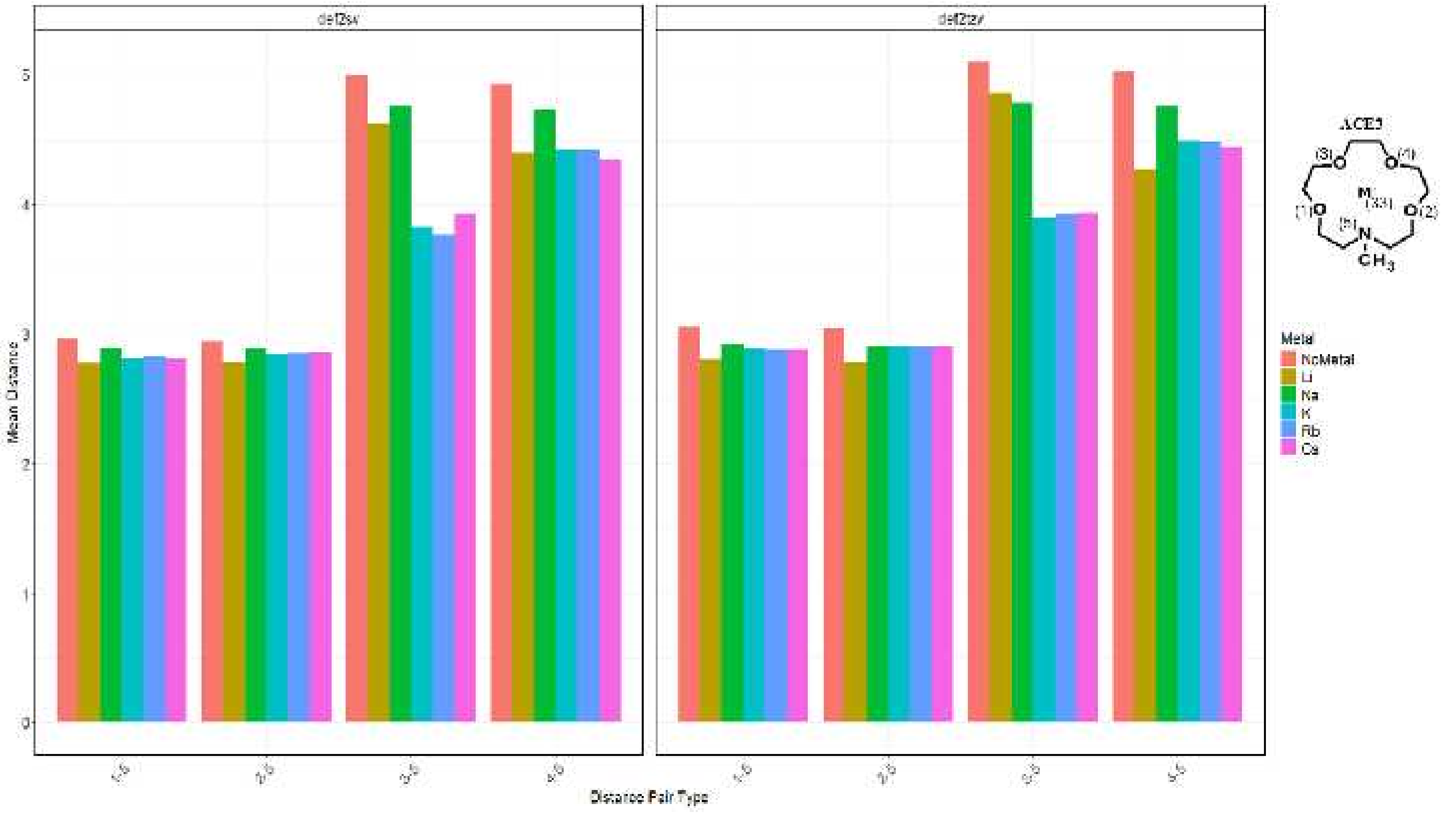

Figure S16. Mean Distance (Å) of N-O$_i$ distances in ACE3 molecule faceted by basis and colored by metal ion type

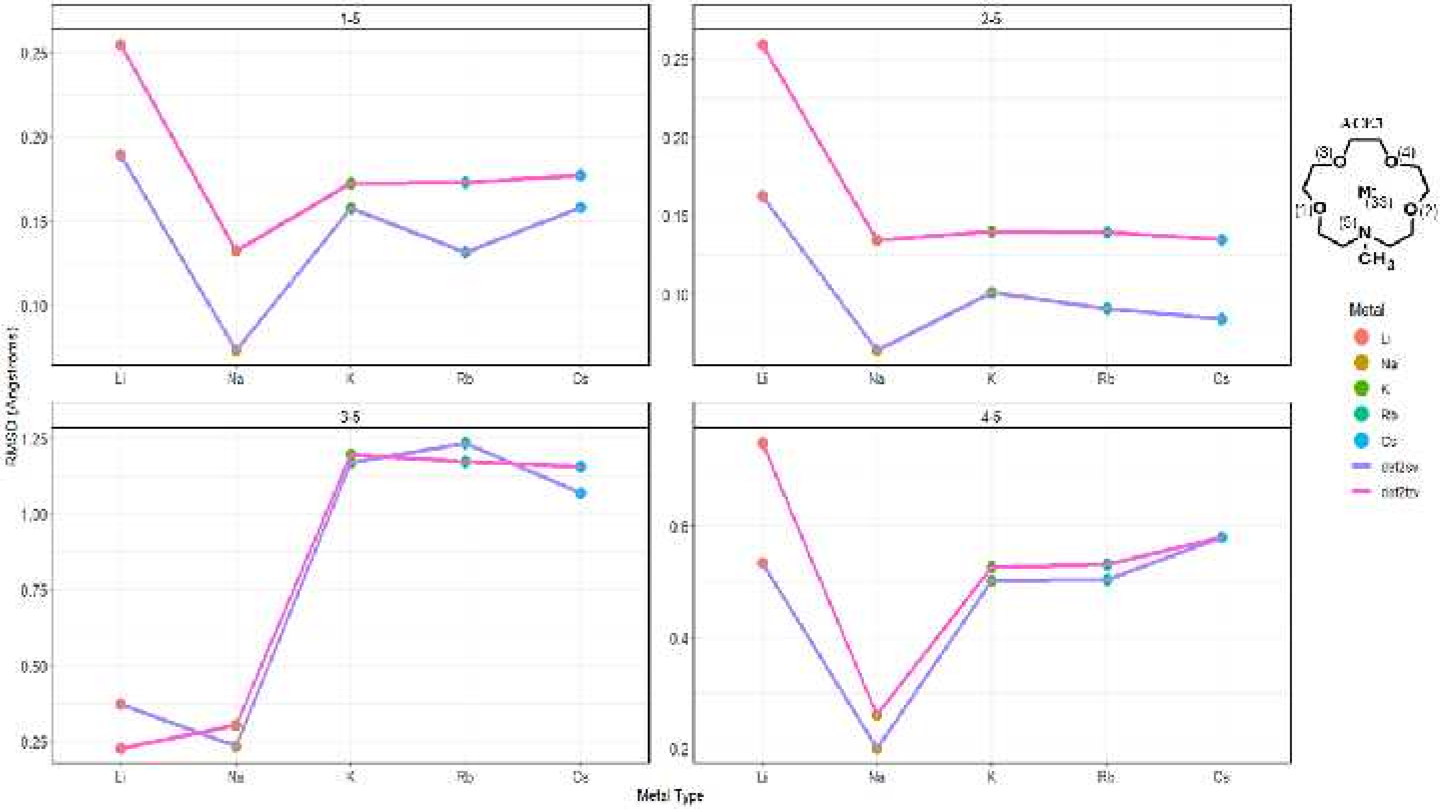

Figure S17. RMSD (Å) of N-O_i distances in ACE3 molecule faceted by atom pairs, colored by metal type and basis

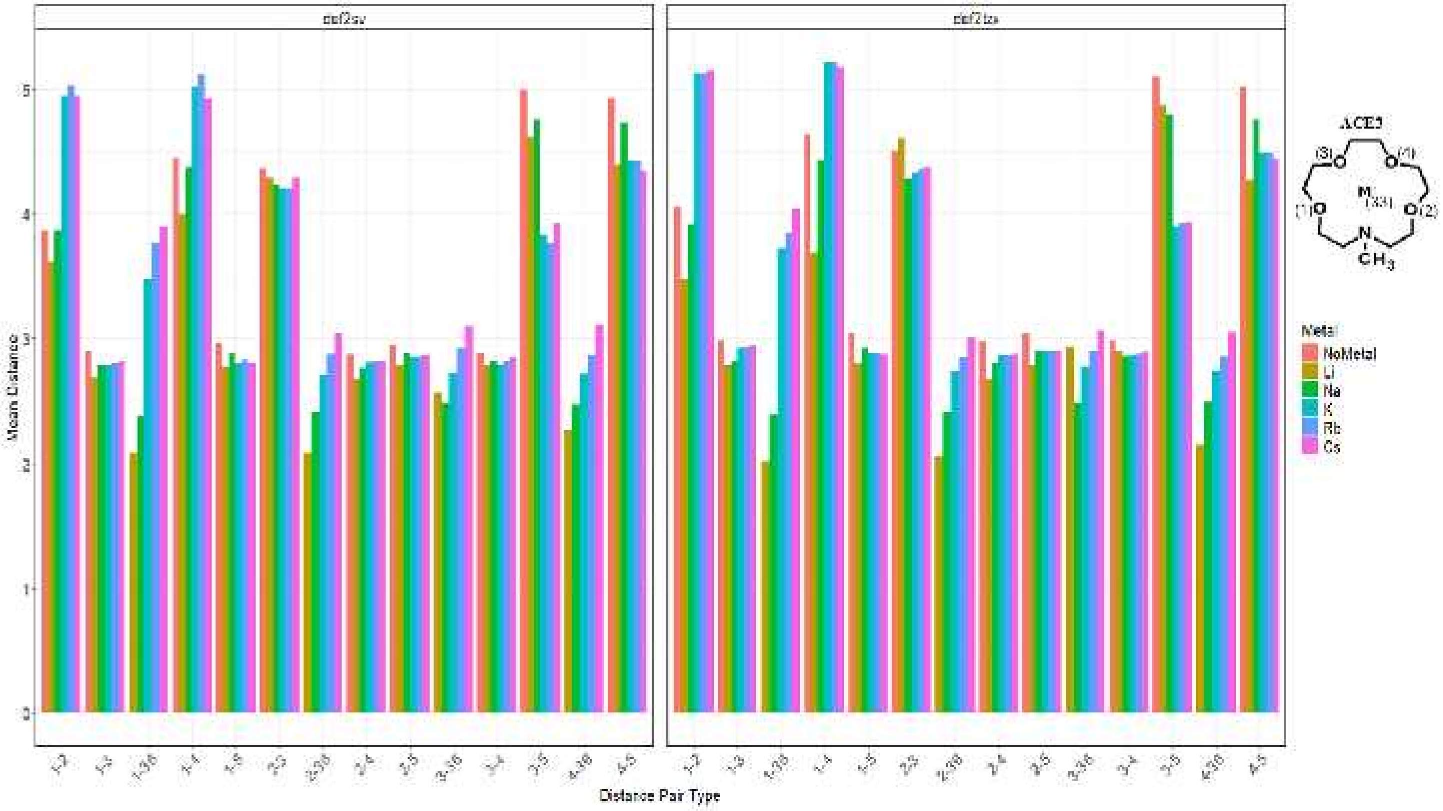

Figure S18. Mean Distance (Å) of O_i–O_j in ACE3 molecule faceted by basis and colored by metal ion

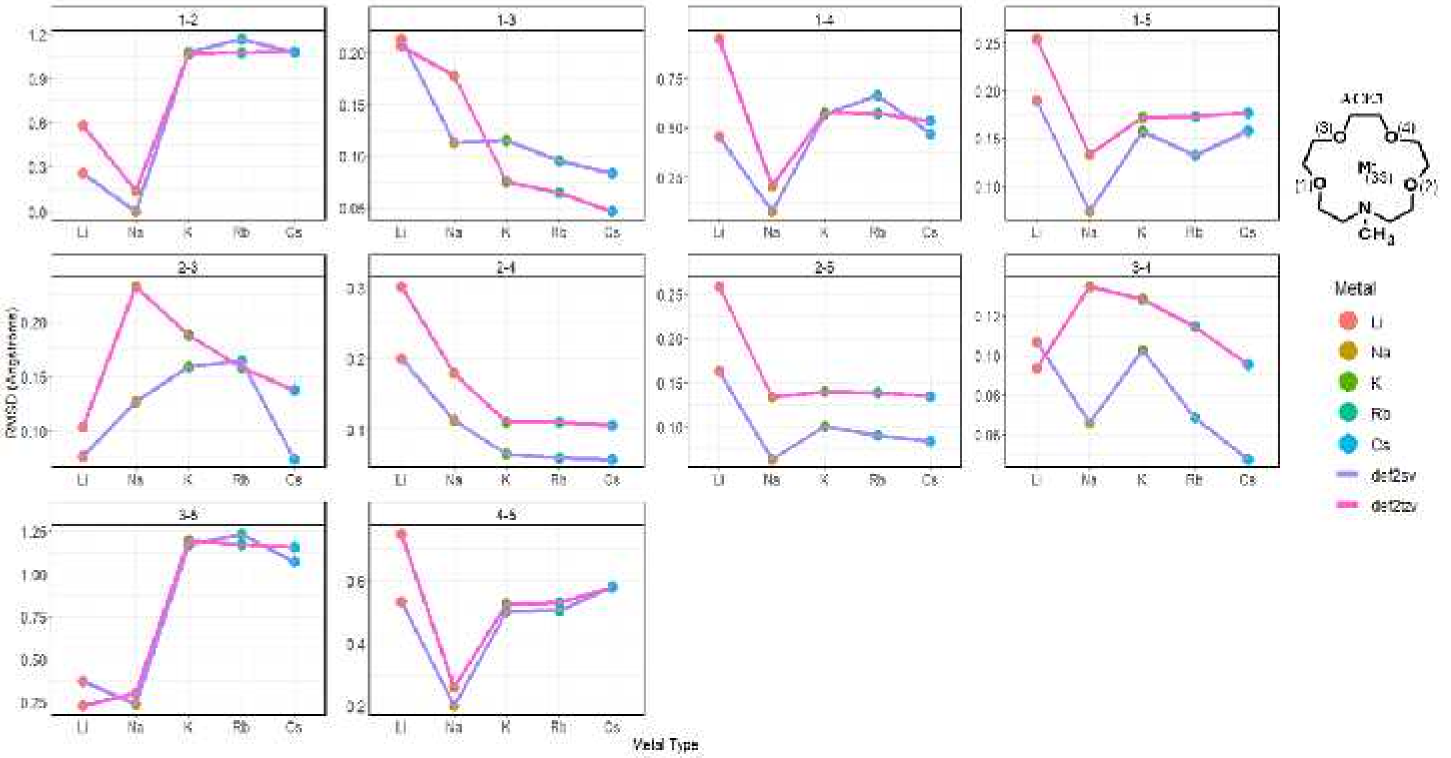

Figure S19. RMSD (Å) of O_i–O_j in ACE3 molecule faceted by Atom pairs and colored by metal ion (points) and basis (lines)

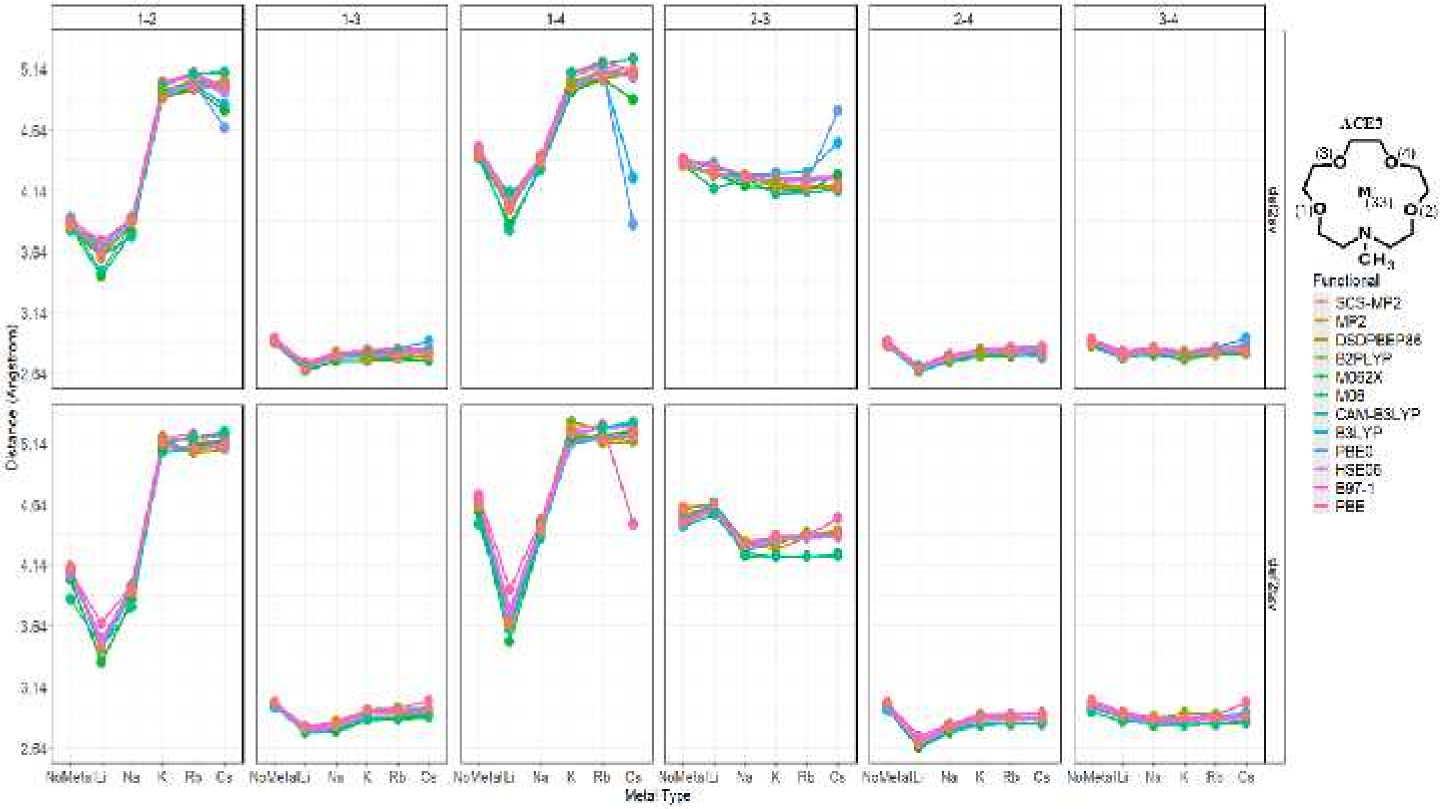

Figure S20. Explicit Distance (Å) of O_i–O_j in ACE3 molecule faceted by basis and Atom pairs; colored by functional used

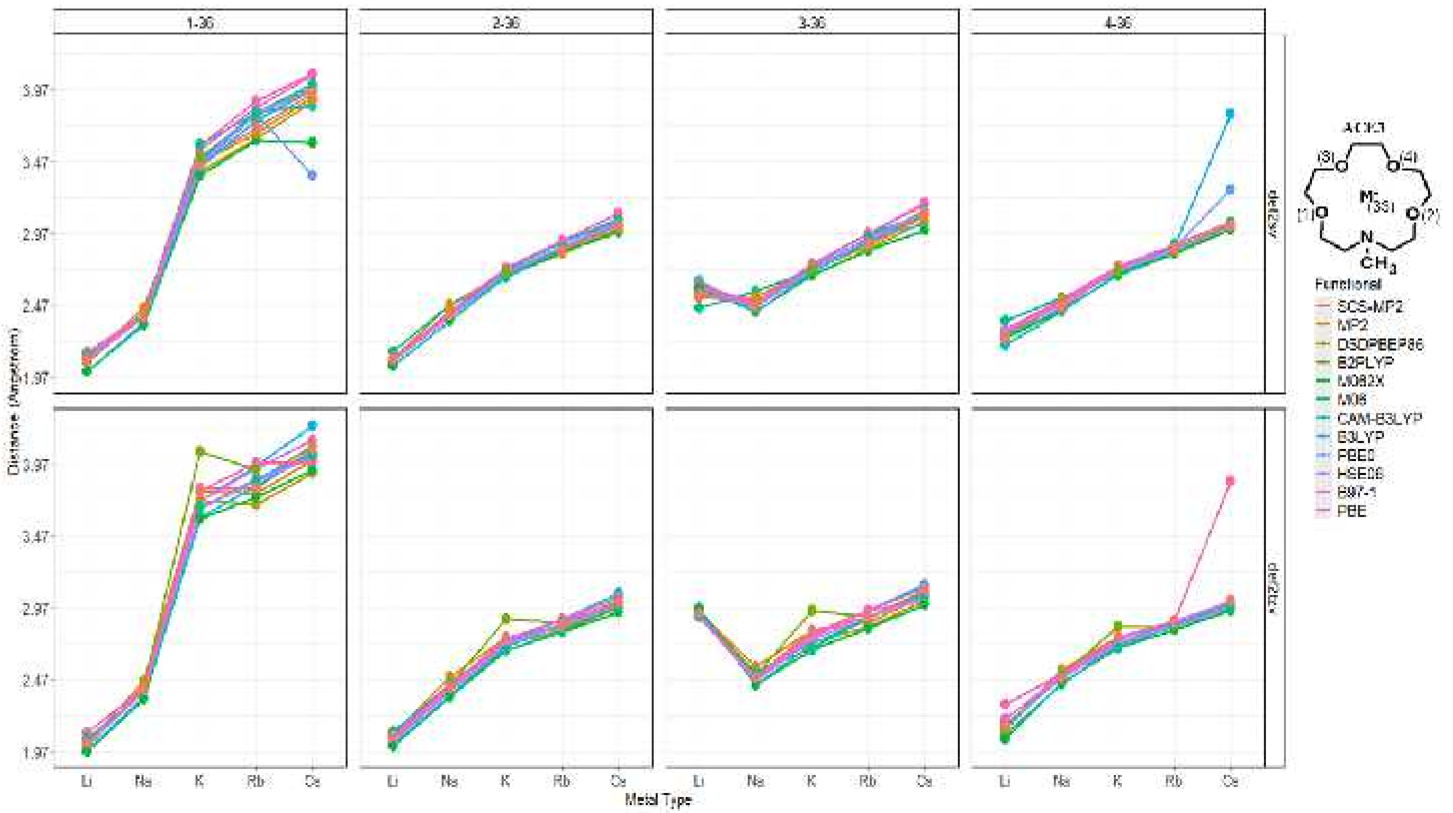

Figure S21. Explicit Distance (Å) of O_i–M⁺ in ACE3 molecule faceted by basis and Atom pairs; colored by functional used

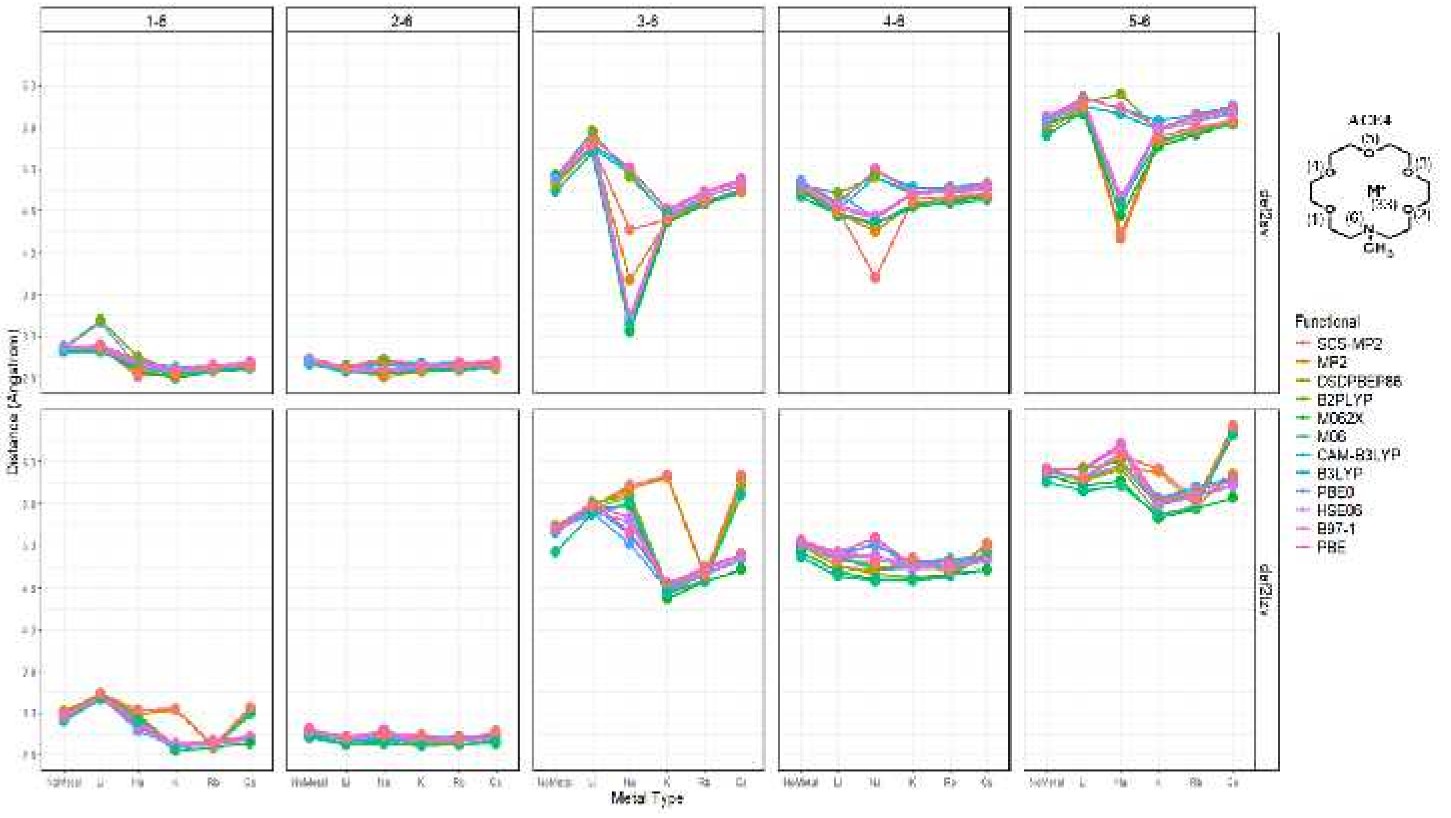

Figure S22. N-O_i distances in ACE4 molecule faceted by basis set, colored by functional

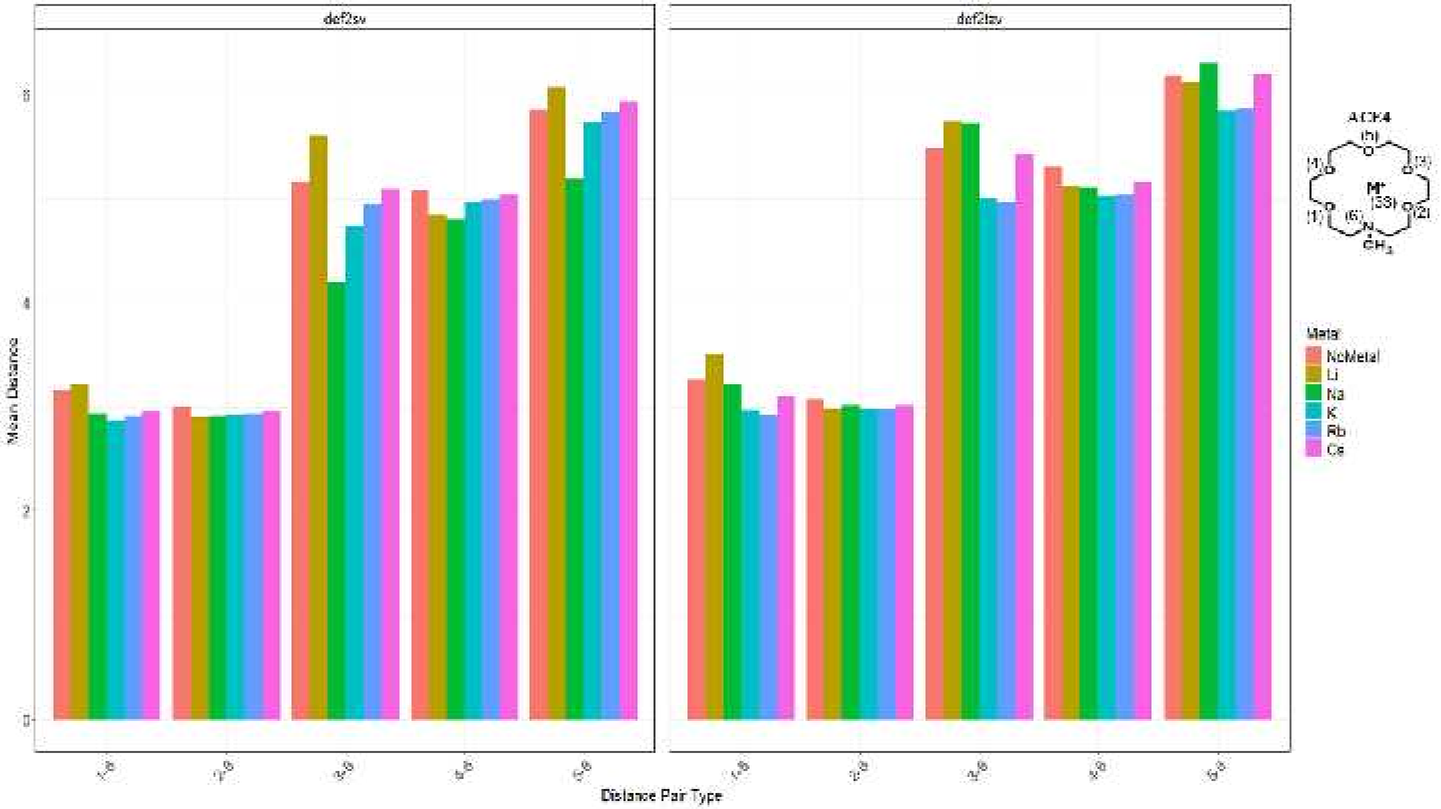

Figure S23. Mean Distance (Å) of N-Oi distances in ACE4 molecule faceted by basis and colored by metal ion type

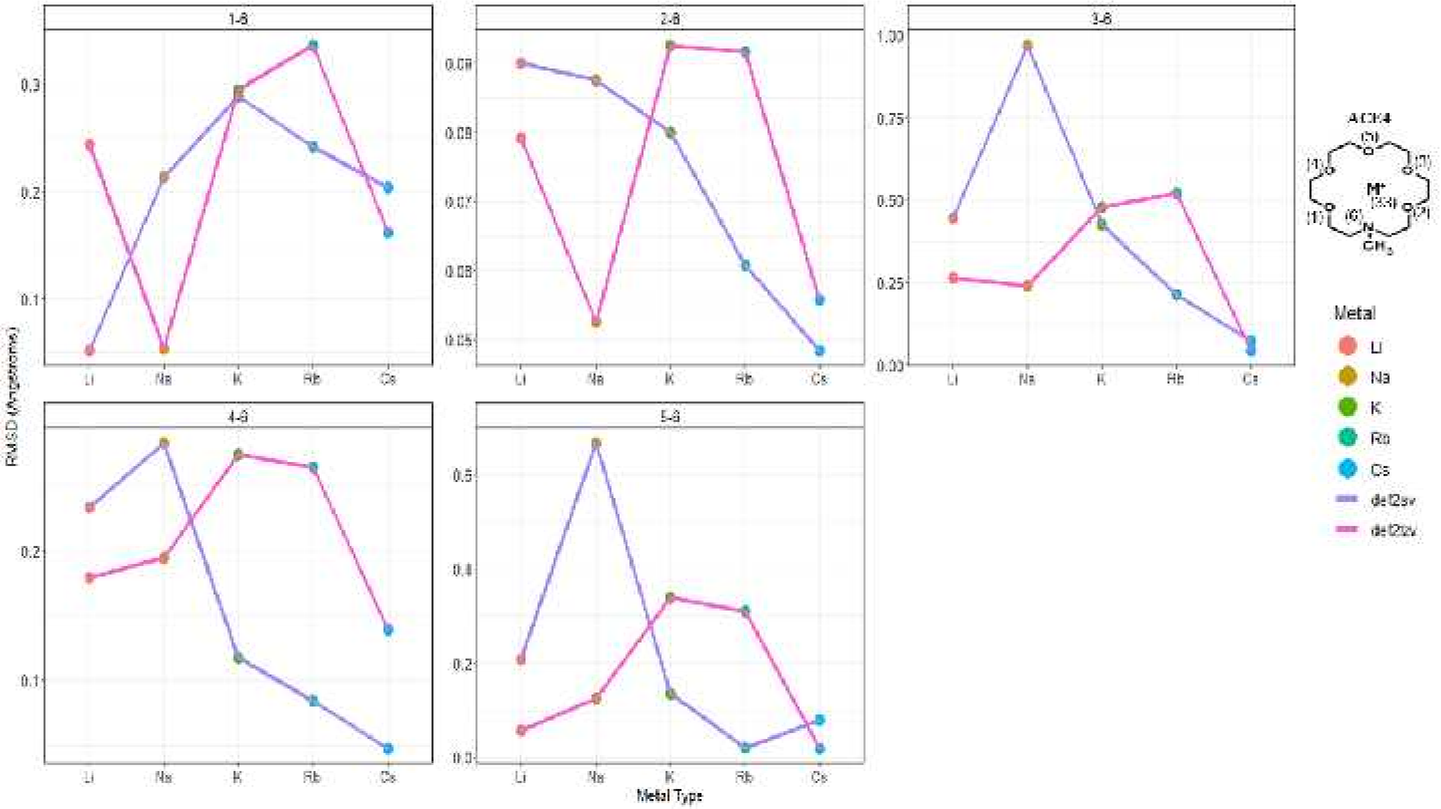

Figure S24. RMSD (Å) of N-Oi distances in ACE4 molecule faceted by atom pairs, colored by metal type and basis

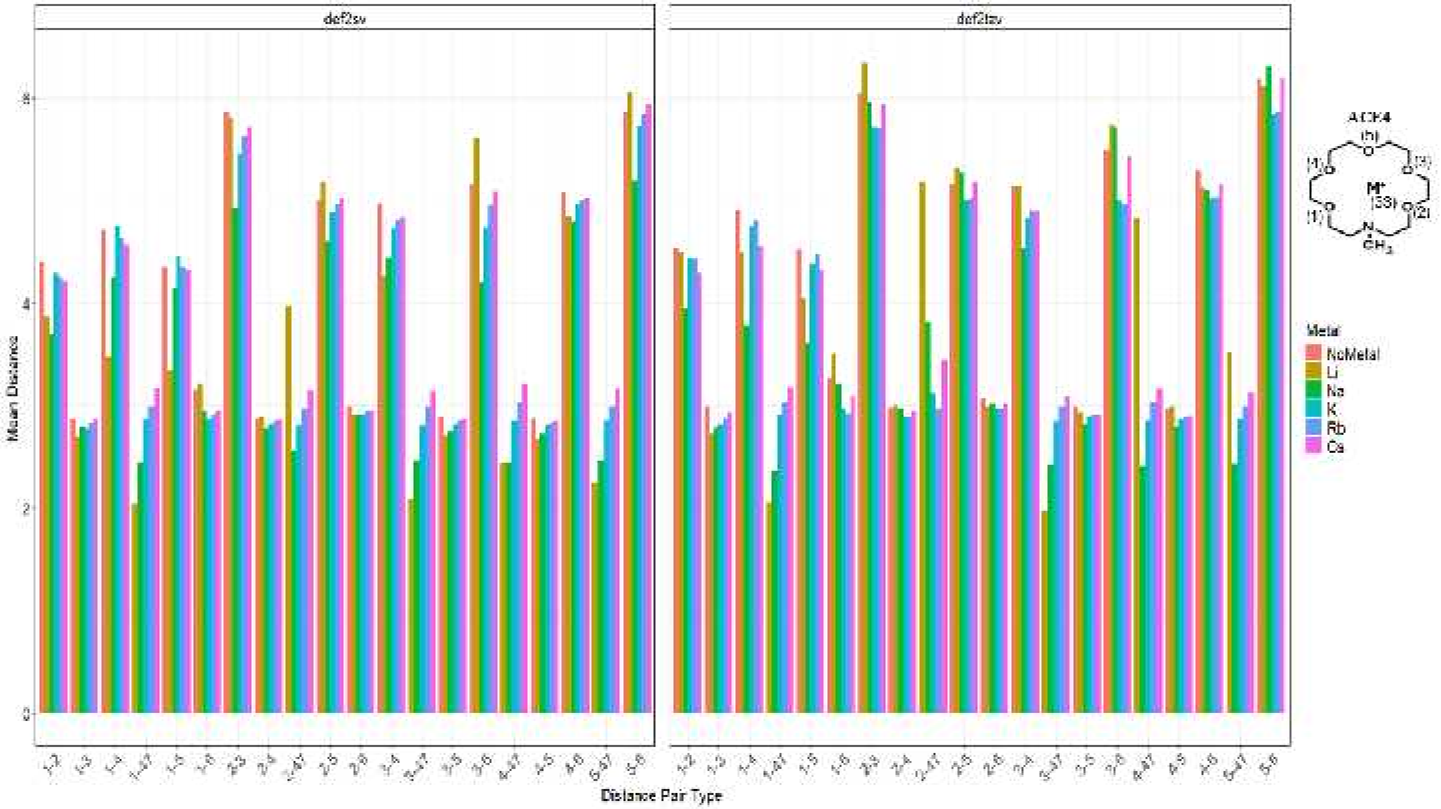

Figure S25. Mean Distance (Å) of O_i–O_j and O_i–M^+ in ACE4 molecule faceted by basis and colored by metal ion

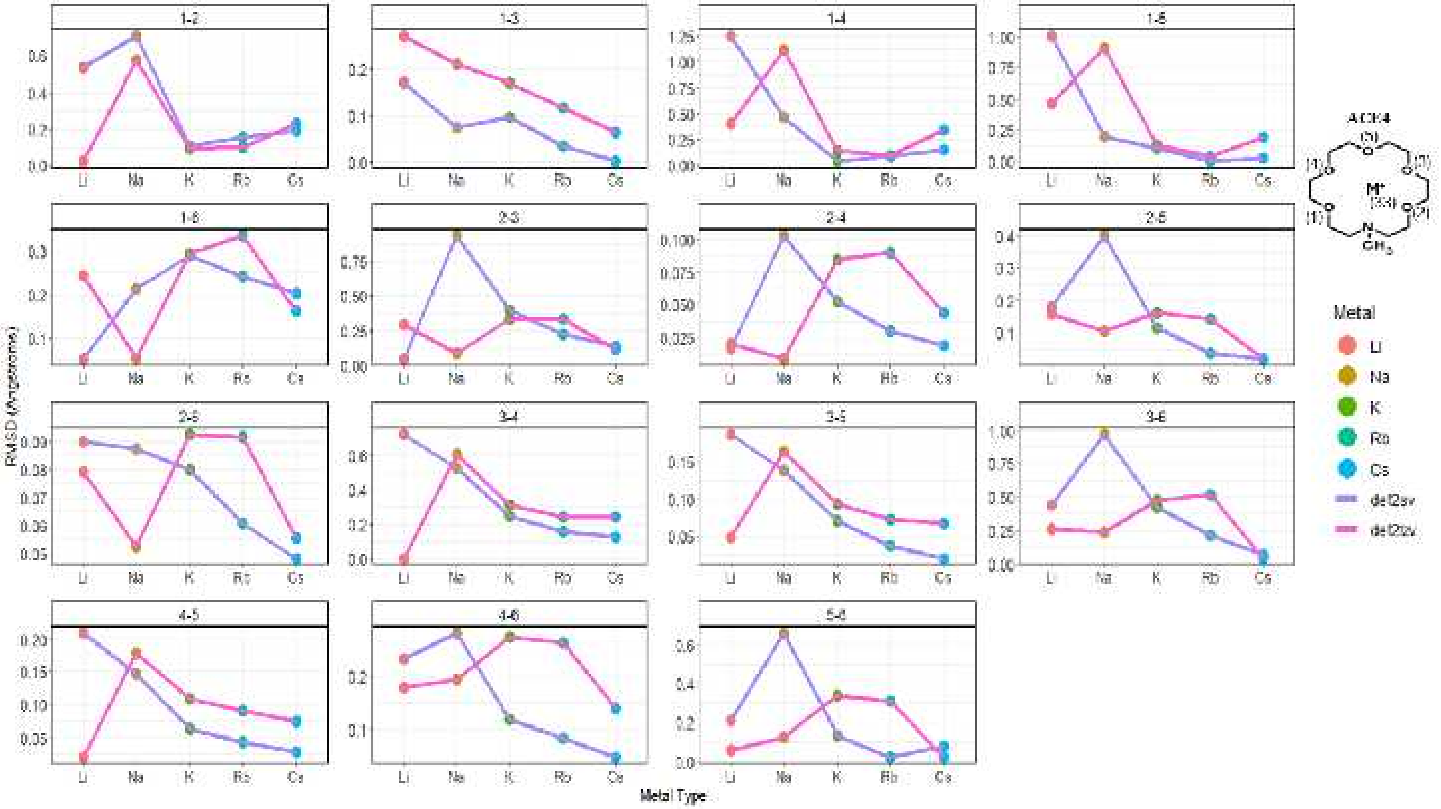

Figure S26. RMSD (Å) of O_i–O_j in ACE4 molecule faceted by Atom pairs and colored by metal ion (points) and basis (lines)

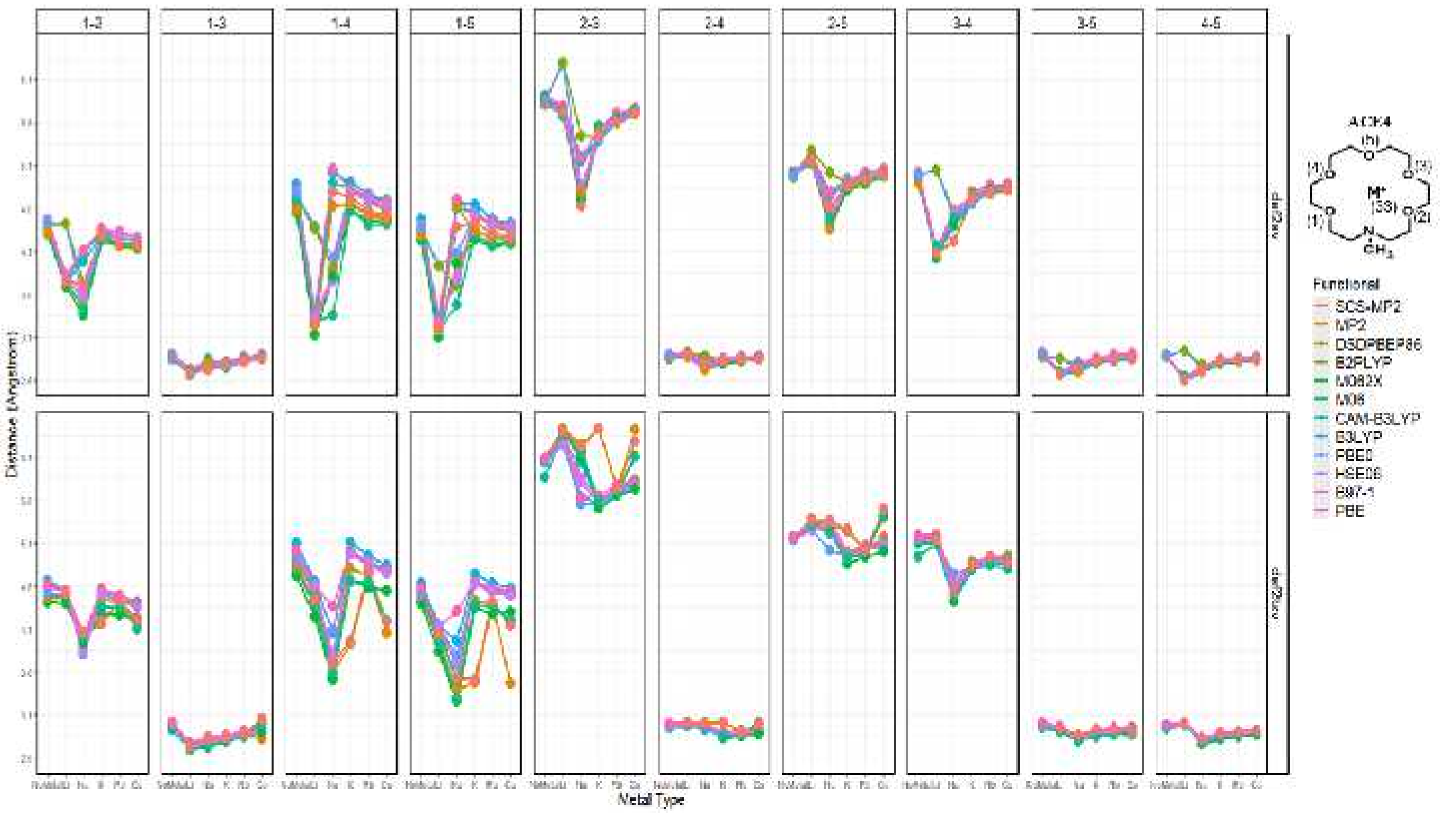

Figure S27. Explicit Distance (Å) of O_i–O_j in ACE4 molecule faceted by basis and Atom pairs; colored by functional used

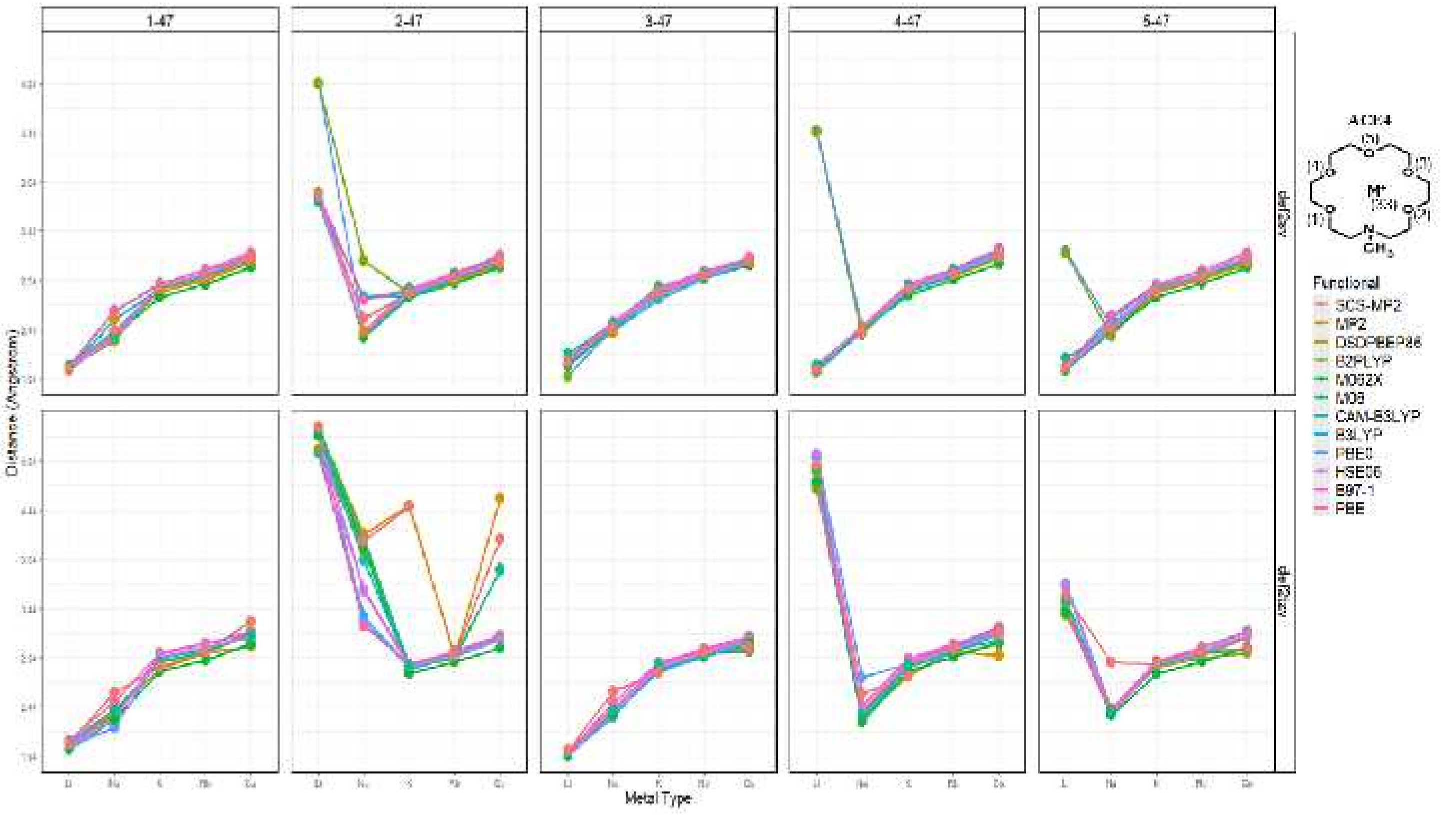

Figure S28. Explicit Distance (Å) of O_i–M^+ in ACE4 molecule faceted by basis and Atom pairs; colored by functional used

Graphical Representation of Geometric Data (Section 2.10):

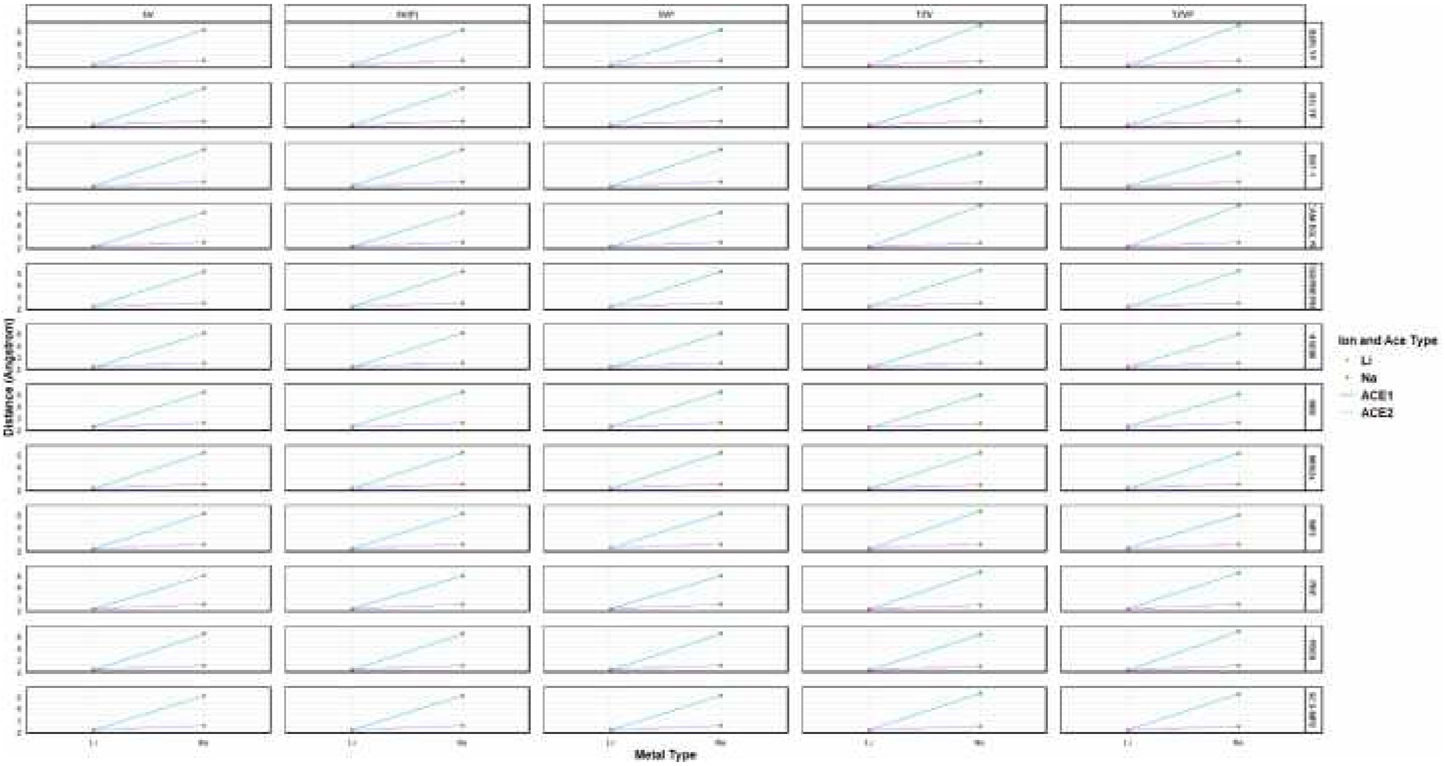

Figure S29. Explicit Distance (Å) of N–M⁺ in ACE1 molecule faceted by basis and Level of Theory; colored by Ion and ACE Type

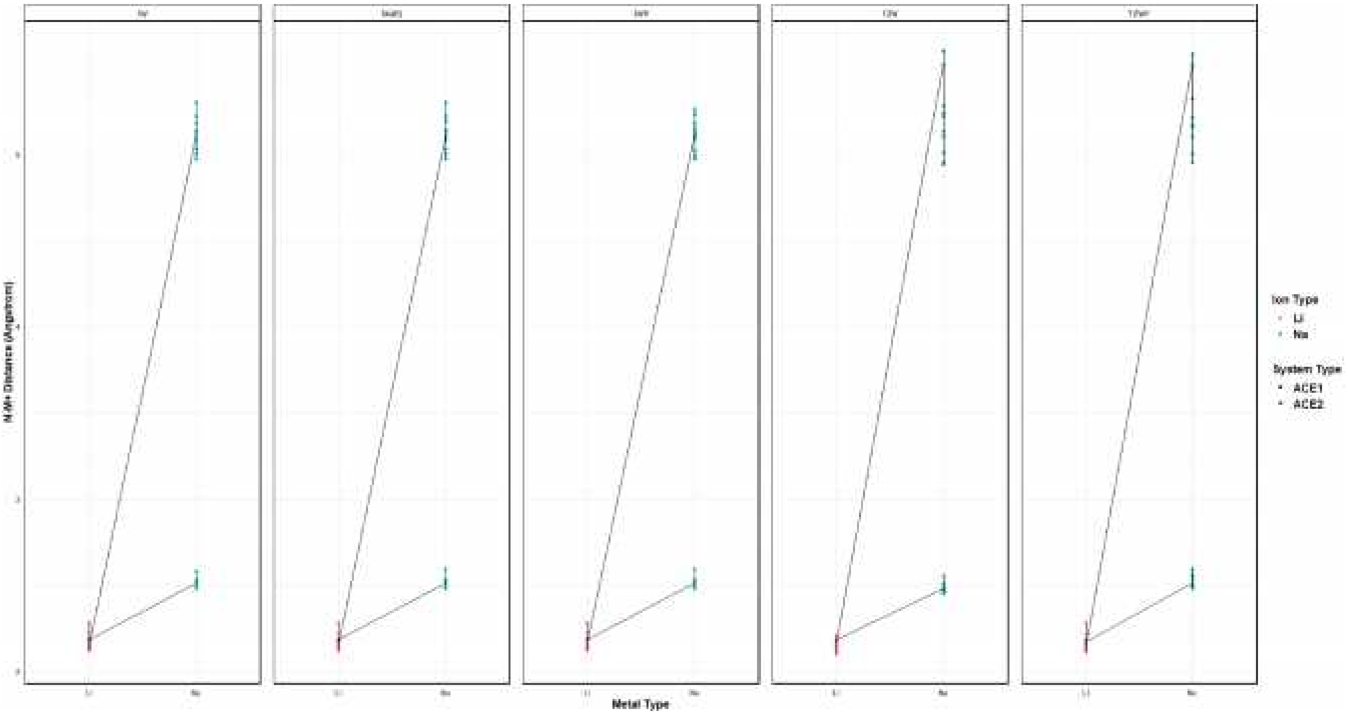

Figure S30. General Distance (Å) of N–M⁺ in ACE1 molecule faceted by basis; colored by Ion and ACE Type

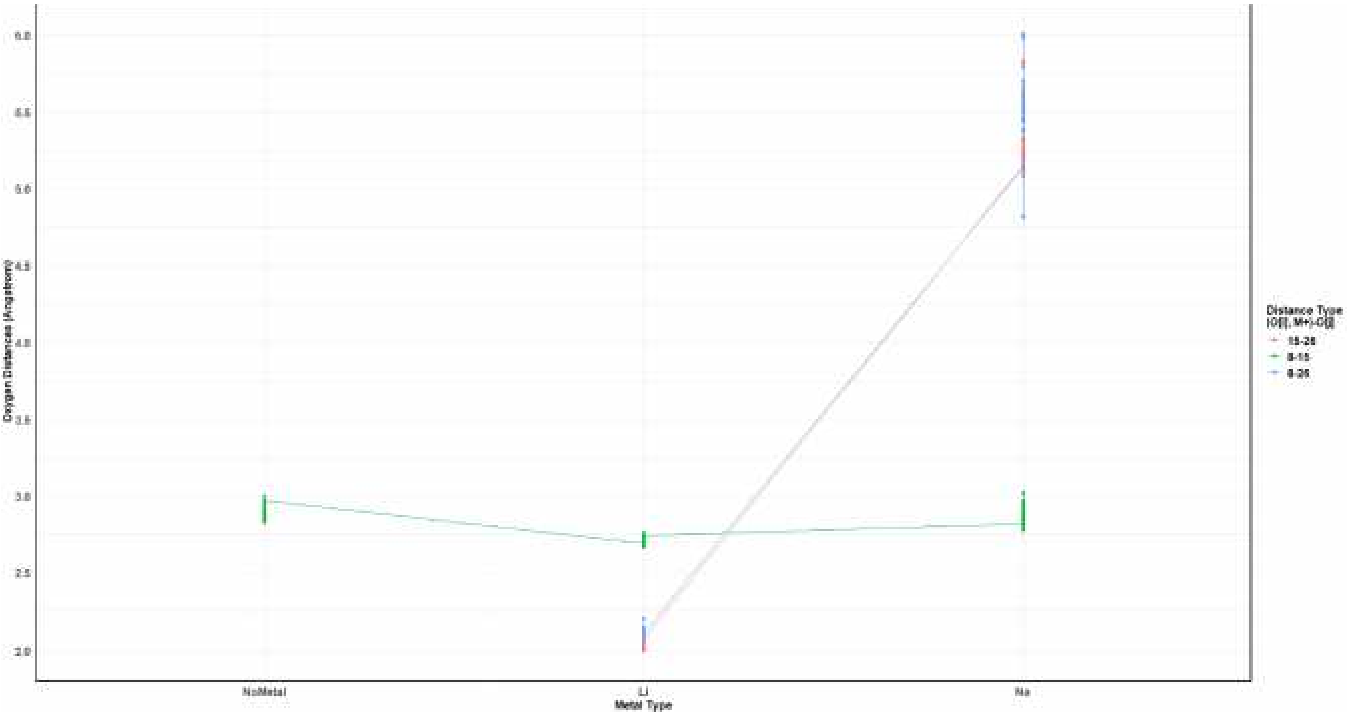

Figure S31. General Distance (Å) of O_i–M^+ and O_i–O_j in ACE1 molecule and Colored by Atom Pair

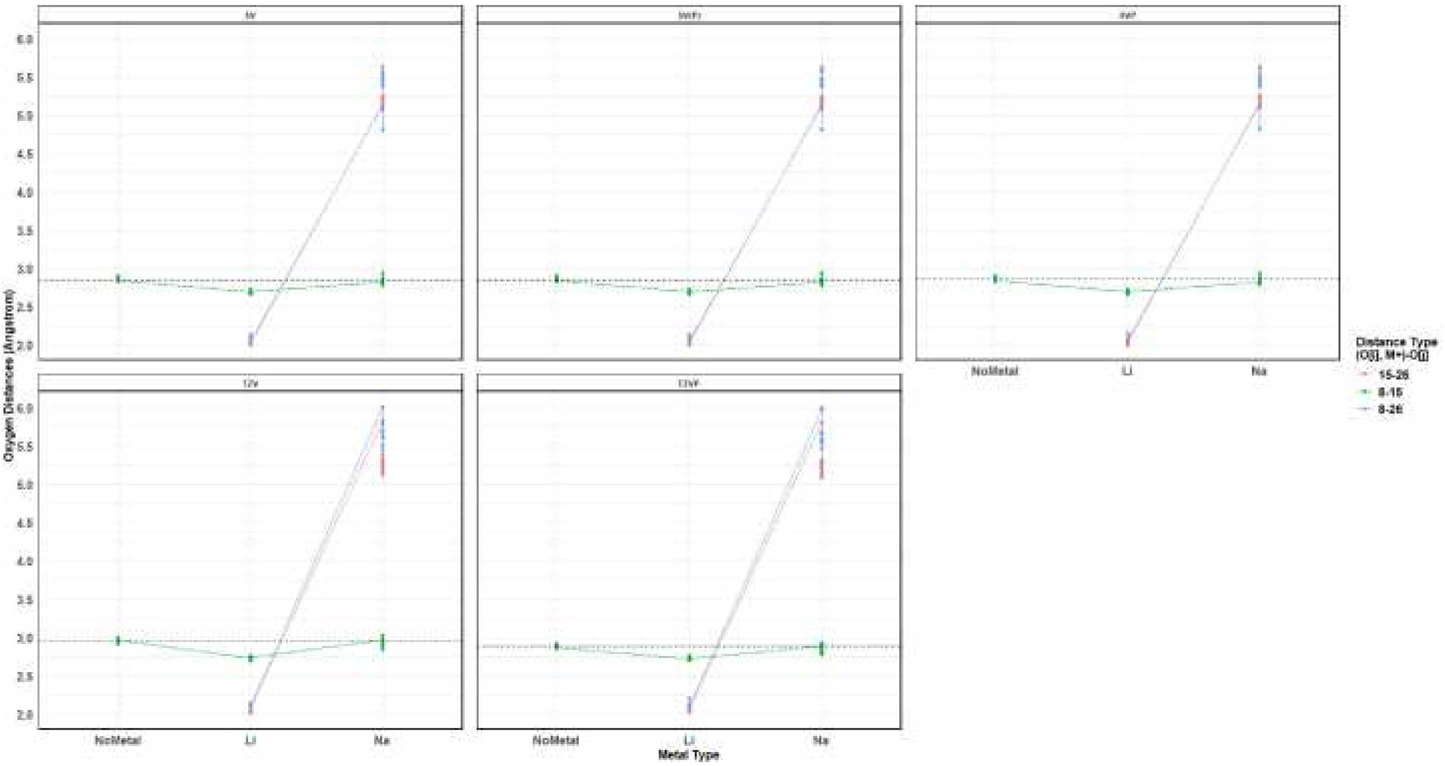

Figure S32. General Distance (Å) of O_i–M^+ and O_i–O_j in ACE1 molecule Faceted by Basis and Colored by Atom Pair

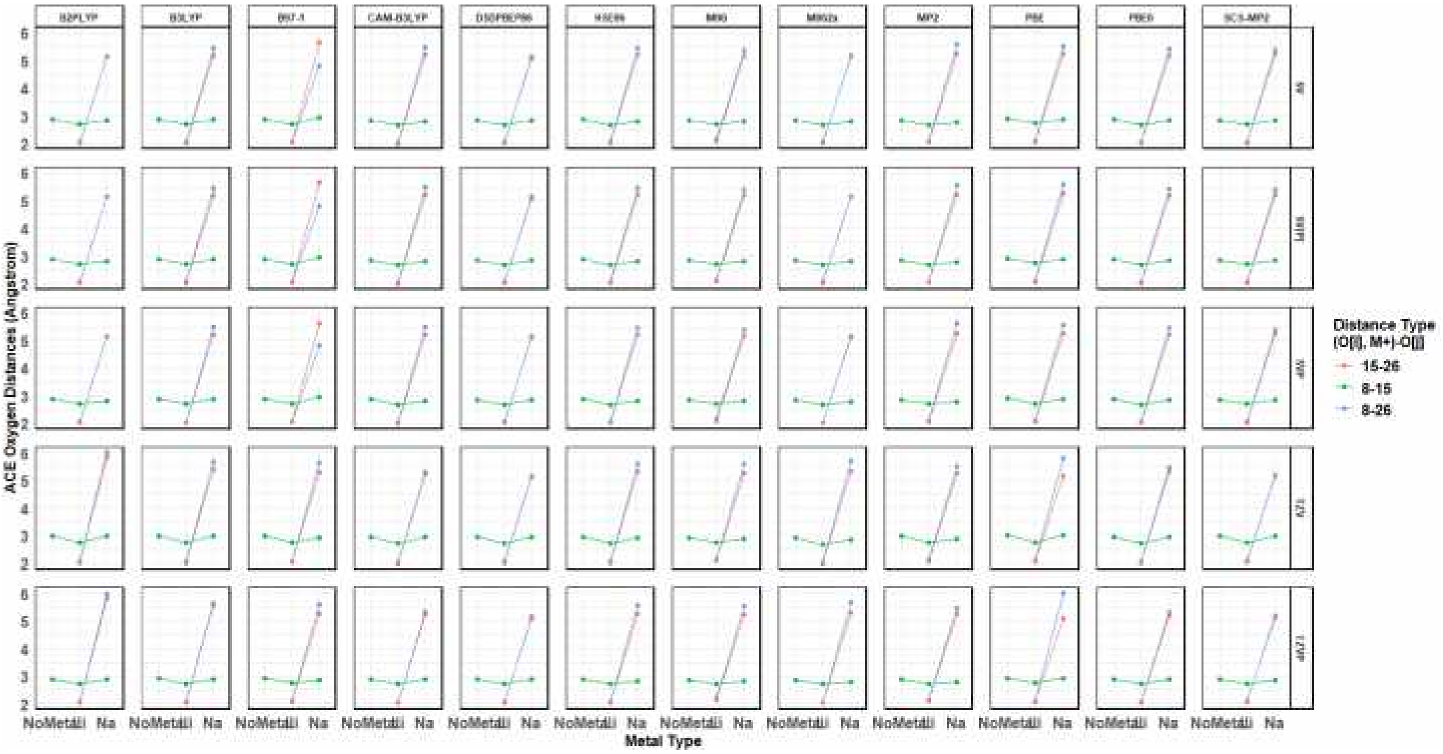

Figure S33. Explicit Distance (Å) of O_i–M^+ and O_i–O_j in ACE1 molecule Faceted by Basis and Level of Theory; Colored by Atom Pair

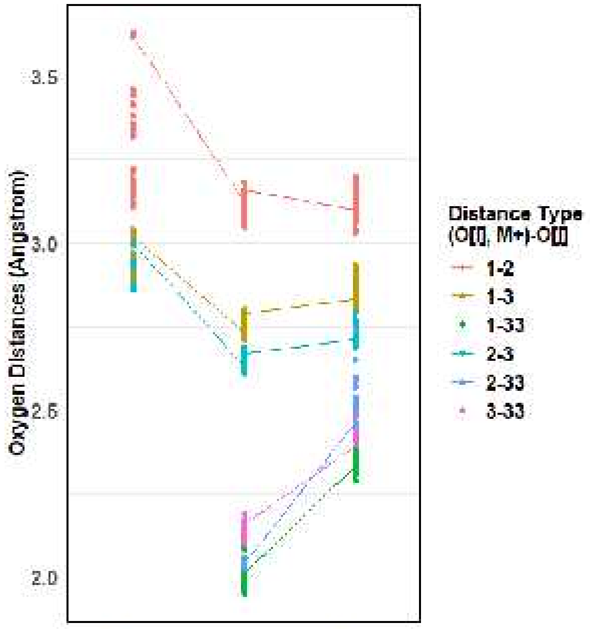

Figure S33. General Distance (Å) of O_i–M^+ and O_i–O_j in ACE2 molecule Faceted by Basis and Level of Theory; Colored by Atom Pair

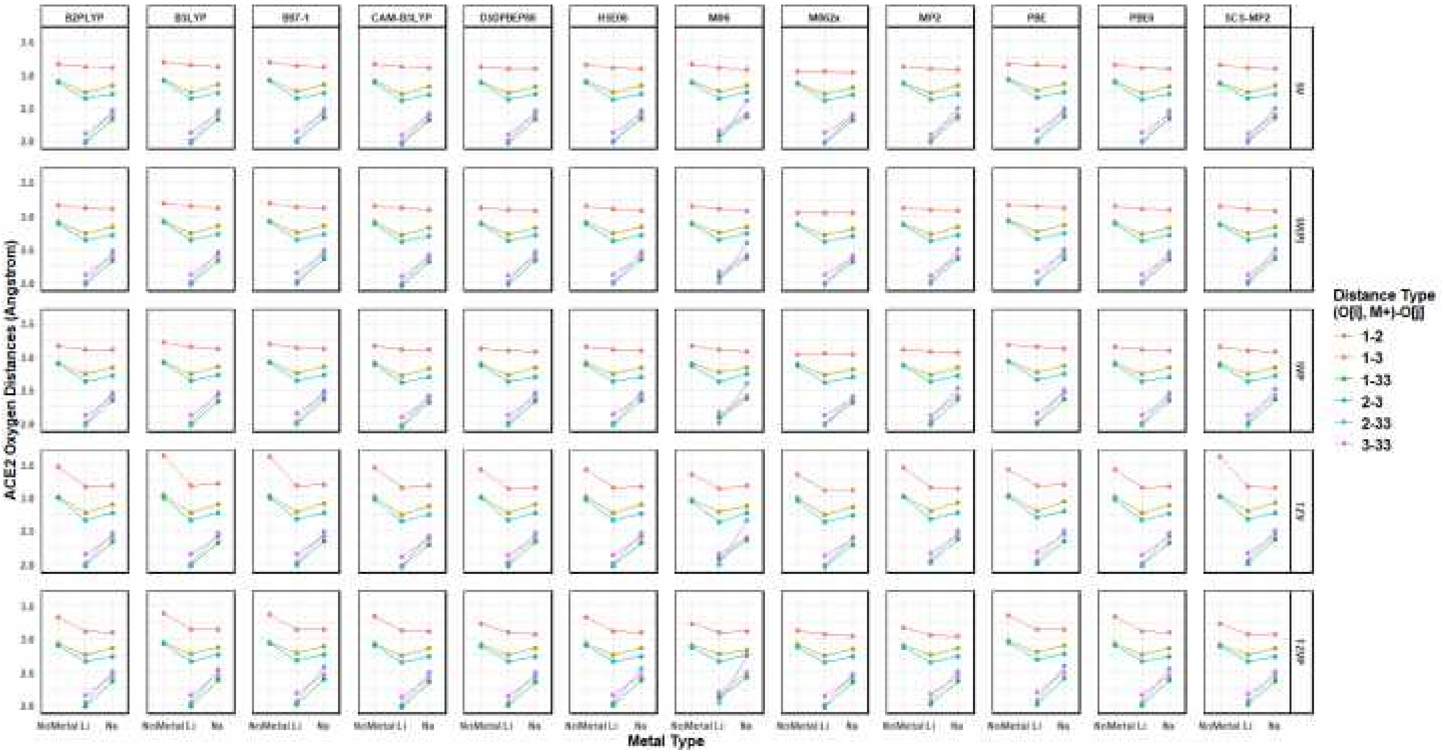

Figure S34. Explicit Distance (Å) of O_i–M^+ and O_i–O_j in ACE2 molecule Faceted by Basis and Level of Theory; Colored by Atom Pair

Binding Energy Calculation Data (Sections 2.6)

Table S5. Binding energy of ACE1-M^+ complexes per functional per basis set (kcal/mol)

	def2sv					def2tzv				
Functional	Li+	Na+	K+	Rb+	Cs+	Li+	Na+	K+	Rb+	Cs+
B2PLYP	-18.825	1.883	0.000	3.353	172.399	-17.204	0.002			
B3LYP	-18.825	-0.628	2.510	3.126	49.224	-15.248	0.561			
CAM-B3LYP	-21.335	0.628	-0.628	-1.017	-8.711	-18.126	0.129			
B97-1	-18.198	1.883	2.510	-8.734	99.836	-15.208	2.295			
PBE	-17.570	1.255	1.255	2.485	58.311	-14.149	4.254			
PBE0	-18.198	0.000	3.138	1.149	1745.951	-15.934	0.369	Deemed Unnecessary		
DSDPBEP86	-20.556	-3.549	-5.436	-3.233	43.139	-19.514	-3.049			
M06	-15.060	0.000	-0.628	-0.658	104.795	-14.186	0.552			
M062X	-20.708	0.000	1.255	2.817	120.882	-18.623	1.053			
MP2	-19.840	-14.984	-15.401	-41.612	34.194	-14.613	-140.838			
SCS-MP2	-21.185	-0.789	-1.060	-1.172	61.268	-14.980	0.073			
HSE06	-18.198	1.255	3.138	-8.346	56.795	-16.004	2.171			

Table S6. Binding energy of ACE2-M^+ complexes per functional per basis set (kcal/mol)

	def2sv					def2tzv				
Functional	Li+	Na+	K+	Rb+	Cs+	Li+	Na+	K+	Rb+	Cs+
B2PLYP	-25.100	-19.453	-15.060	-13.178	-10.668	-22.590	-16.943	-10.040	-9.413	-7.374
B3LYP	-25.100	-20.080	-16.315	-14.433	-11.923	-20.080	-15.688	-9.413	-9.413	-7.904
CAM-B3LYP	-28.238	-23.218	-19.453	-16.943	-15.060	-23.218	-19.453	-12.550	-11.295	-10.390
B97-1	-23.218	-19.453	-16.943	-14.433	-13.178	-19.453	-15.688	-10.668	-10.668	-9.695
PBE	-23.218	-19.453	-16.315	-15.060	-13.805	-18.198	-15.688	-10.040	-10.040	-9.677
PBE0	-24.473	-19.453	-16.943	-15.060	-13.805	-20.708	-16.315	-10.668	-10.668	-10.475
DSDPBEP86	-27.734	-24.596	-22.828	-18.514	-16.196	-25.863	-22.260	-18.335	-13.393	-13.476
M06	-19.453	-18.198	-18.198	-16.943	-16.943	-18.198	-15.060	-12.550	-13.805	-14.540
M062X	-27.610	-23.845	-21.963	-20.080	-19.453	-24.473	-20.708	-16.315	-16.943	-17.263
MP2	-29.599	-23.362	-22.401	-48.831	-38.537	-26.248	-61.158	-28.848	-52.598	-73.184
SCS-MP2	-28.427	-23.030	-21.373	-19.280	-18.195	-18.753	-15.230	-9.854	-14.297	-13.297
HSE06	-23.845	-19.453	-16.315	-14.433	-13.178	-20.708	-16.943	-11.295	-11.295	-10.807

Table S7. Binding energy of ACE3-M^+ complexes per functional per basis set (kcal/mol)

	def2sv					def2tzv				
Functional	Li+	Na+	K+	Rb+	Cs+	Li+	Na+	K+	Rb+	Cs+
B2PLYP	-27.610	-27.610	-13.805	-10.668	-8.158	-22.590	-22.590	-5.020	-5.648	-3.009
B3LYP	-27.610	-28.865	-15.060	-11.923	-5.648	-19.453	-20.708	-5.648	-5.020	-2.997
CAM-B3LYP	-30.748	-32.003	-18.825	-15.688	-12.550	-23.218	-25.100	-9.413	-8.158	-6.242
B97-1	-25.728	-26.983	-15.688	-12.550	-10.668	-18.825	-20.708	-6.903	-6.275	-5.141
PBE	-25.100	-26.355	-15.060	-13.178	-10.668	-16.943	-19.453	-5.648	-5.648	-0.021

PBE0	-26.355	-26.355	-15.060	-11.923	-6.275	-20.080	-21.335	-6.903	-6.903	-5.813
DSDPBEP86	-29.811	-32.177	-21.951	-16.450	-13.536	-25.594	-26.974	-15.229	-9.941	-9.405
M06	-24.473	-25.728	-18.825	-16.943	-16.315	-20.080	-20.080	-11.295	-11.923	-12.661
M062X	-29.493	-31.376	-22.590	-19.453	-18.825	-24.473	-25.728	-14.433	-14.433	-14.581
MP2	-30.189	-29.622	-48.765	-73.840	-63.183	-19.008	-16.799	-182.616	-207.010	-226.814
SCS-MP2	-31.334	-32.305	-21.383	-17.960	-16.469	-19.408	-19.956	-7.506	-12.422	-10.700
HSE06	-26.355	-26.983	-15.060	-11.923	-10.668	-20.080	-21.335	-6.903	-6.275	-5.667

Table S8. Binding energy of ACE4-M^+ complexes per functional per basis set (kcal/mol)

	def2sv					def2tzv				
Functional	Li+	Na+	K+	Rb+	Cs+	Li+	Na+	K+	Rb+	Cs+
B2PLYP	-12.550	-18.198	-25.728	-23.218	-20.080	-10.668	-14.433	-18.198	-17.307	-14.903
B3LYP	-20.080	-23.845	-25.728	-23.218	-20.708	-9.413	-13.178	-16.943	-16.943	-15.060
CAM-B3LYP	-23.845	-26.983	-29.493	-26.983	-24.473	-11.923	-16.943	-20.708	-19.453	-18.198
B97-1	-18.825	-26.355	-25.728	-23.845	-21.963	-8.785	-12.550	-17.570	-17.570	-16.315
PBE	-18.198	-22.590	-25.100	-24.473	-22.590	-8.158	-11.295	-16.943	-16.943	-16.315
PBE0	-11.295	-25.728	-25.728	-23.218	-21.963	-9.413	-10.668	-18.198	-18.198	-17.570
DSDPBEP86	-23.115	-33.476	-33.538	-28.954	-26.148	-12.682	-19.727	-27.130	-22.095	-10.621
M06	-18.198	-25.728	-26.983	-26.355	-26.983	-8.785	-13.805	-18.825	-20.708	-11.923
M062X	-24.473	-32.631	-30.120	-28.865	-28.865	-11.295	-18.825	-22.590	-23.218	-24.473
MP2	-25.251	-35.273	-34.372	-59.547	-49.691	-10.668	-21.711	-6.433	-15.959	-1.726
SCS-MP2	-23.581	-34.773	-33.271	-29.917	-29.083	-8.633	-19.768	-9.375	-23.133	-11.000
HSE06	-18.825	-26.355	-25.728	-23.218	-21.963	-9.413	-13.178	-18.198	-18.198	-17.447

Charge Transfer Calculation Data (Sections 2.7)

Table S9. Charge transfer (ΔN) of ACE1-M^+ per functional and basis

	def2sv					def2tzv				
	Li^+	Na^+	K^+	Rb^+	Cs^+	Li^+	Na^+	K^+	Rb^+	Cs^+
B2PLYP	0.3892	0.3334	0.2757	0.2492	0.2208	0.3855	0.3319	0.2683	0.2441	0.2151
B3LYP	0.4046	0.3555	0.3041	0.2818	0.2548	0.4007	0.3512	0.2957	0.2768	0.2493
CAM-B3LYP	0.3836	0.3247	0.2722	0.2470	0.2205	0.3797	0.3231	0.2642	0.2421	0.2151
B97-1	0.3997	0.3452	0.2948	0.2718	0.2456	0.4013	0.3436	0.2874	0.2676	0.2404
PBE	0.3738	0.3104	0.2549	0.2324	0.2281	0.3698	0.3052	0.2460	0.2272	0.1989
PBE0	0.3975	0.3436	0.2921	0.2689	0.2427	0.3937	0.3418	0.2857	0.2659	0.2387
DSDPBEP86	0.3809	0.3221	0.2631	0.2361	0.2074	0.3775	0.3223	0.2566	0.2310	0.2101
M06	0.4073	0.3673	0.3103	0.2902	0.2643	0.4070	0.3599	0.3093	0.2929	0.2660
M062X	0.3891	0.3352	0.2764	0.2503	0.2223	0.3851	0.3337	0.2713	0.2473	0.2179
MP2	0.3729	0.3136	0.2518	0.2232	0.1944	0.3690	0.3144	0.2445	0.2177	0.1876
SCS-MP2	0.3728	0.3135	0.2516	0.2230	0.1942	0.3689	0.3143	0.2340	0.2175	0.1790
HSE06	0.4030	0.3528	0.3021	0.2808	0.2549	0.3992	0.3498	0.2960	0.2780	0.2510

Table S10. Charge transfer (ΔN) of ACE2-M^+ per functional and basis

	def2sv					def2tzv				
	Li^+	Na^+	K^+	Rb^+	Cs^+	Li^+	Na^+	K^+	Rb^+	Cs^+
B2PLYP	0.3890	0.3332	0.2755	0.2491	0.2207	0.3851	0.3314	0.2677	0.2435	0.2145
B3LYP	0.4042	0.3550	0.3037	0.2814	0.2545	0.3999	0.3501	0.2944	0.2754	0.2479
CAM-B3LYP	0.3832	0.3243	0.2719	0.2467	0.2203	0.3792	0.3225	0.2635	0.2414	0.2145
B97-1	0.3994	0.3448	0.2944	0.2715	0.2454	0.4006	0.3425	0.2862	0.2663	0.2391
PBE	0.4119	0.3660	0.3225	0.3058	0.2907	0.4073	0.3605	0.3138	0.3010	0.2862
PBE0	0.3971	0.3432	0.2917	0.2687	0.2426	0.3933	0.3413	0.2852	0.2653	0.2382
DSDPBEP86	0.3807	0.3219	0.2629	0.2360	0.2073	0.3771	0.3219	0.2560	0.2305	0.2012
M06	0.4067	0.3665	0.3096	0.2894	0.2636	0.4061	0.3586	0.3075	0.2910	0.2640
M062X	0.3888	0.3348	0.2760	0.2501	0.2222	0.3846	0.3329	0.2705	0.2465	0.2170
MP2	0.3728	0.3135	0.2517	0.2232	0.1944	0.3687	0.3140	0.2440	0.2172	0.1871
SCS-MP2	0.3727	0.3133	0.2515	0.2229	0.1941	0.3685	0.3138	0.2335	0.2170	0.1869
HSE06	0.4027	0.3524	0.3017	0.2805	0.2547	0.3988	0.3492	0.2954	0.2773	0.2504

Table S10. Charge transfer (ΔN) of ACE3-M^+ per functional and basis

	def2sv					def2tzv				
	Li^+	Na^+	K^+	Rb^+	Cs^+	Li^+	Na^+	K^+	Rb^+	Cs^+
B2PLYP	0.3869	0.3299	0.2708	0.2437	0.2437	0.3832	0.3284	0.2635	0.2388	0.2092
B3LYP	0.4019	0.3511	0.2981	0.2750	0.2750	0.3980	0.3468	0.2896	0.2699	0.2416
CAM-B3LYP	0.3810	0.3207	0.2670	0.2411	0.2411	0.3770	0.3190	0.2587	0.2361	0.2085
B97-1	0.3970	0.3408	0.2886	0.2648	0.2648	0.3986	0.3391	0.2812	0.2606	0.2325
PBE	0.4094	0.3615	0.3160	0.2982	0.2982	0.4048	0.3561	0.3075	0.2936	0.2676
PBE0	0.3946	0.3391	0.2859	0.2619	0.2619	0.3909	0.3374	0.2796	0.2590	0.2309
DSDPBEP86	0.3787	0.3188	0.2584	0.2309	0.2309	0.3752	0.3190	0.2521	0.2261	0.2050
M06	0.4043	0.3625	0.3037	0.2825	0.2825	0.4039	0.3547	0.3017	0.2841	0.2559
M062X	0.3866	0.3313	0.2710	0.2442	0.2442	0.3822	0.3292	0.2650	0.2403	0.2099
MP2	0.3710	0.3108	0.2480	0.2190	0.2190	0.3674	0.3121	0.2416	0.2146	0.1843
SCS-MP2	0.3709	0.3107	0.2478	0.2188	0.2188	0.3673	0.3120	0.2309	0.2143	0.1754
HSE06	0.4002	0.3482	0.2956	0.2734	0.2734	0.3638	0.2999	0.2359	0.2139	0.1848

Table S11. Charge transfer (ΔN) of ACE4-M^+ per functional and basis

	def2sv					def2tzv				
	Li^+	Na^+	K^+	Rb^+	Cs^+	Li^+	Na^+	K^+	Rb^+	Cs^+
B2PLYP	0.3925	0.3380	0.2813	0.2551	0.3925	0.3881	0.3354	0.2725	0.2485	0.4147
B3LYP	0.4079	0.3603	0.3100	0.2881	0.4618	0.4037	0.3556	0.3009	0.2823	0.4861
CAM-B3LYP	0.3865	0.3287	0.2769	0.2519	0.3991	0.3820	0.3262	0.2676	0.2456	0.4203
B97-1	0.4027	0.3493	0.2998	0.2771	0.4371	0.4040	0.3471	0.2914	0.2717	0.4604
PBE	0.4154	0.3711	0.3286	0.3125	0.4527	0.4104	0.3650	0.3191	0.3067	0.5255
PBE0	0.4005	0.3478	0.2971	0.2743	0.4349	0.3961	0.3452	0.2897	0.2700	0.4561
DSDPBEP86	0.3828	0.3245	0.2659	0.2390	0.3687	0.3799	0.3256	0.2604	0.2350	0.3890

M06	0.3693	0.3152	0.2517	0.2284	0.3642	0.3917	0.3378	0.2815	0.2626	0.4613
M062X	0.3919	0.3389	0.2809	0.2550	0.4153	0.3869	0.3359	0.2736	0.2496	0.4388
MP2	0.3765	0.3183	0.2578	0.2295	0.3373	0.3716	0.3179	0.2488	0.2222	0.3611
SCS-MP2	0.3764	0.3181	0.2575	0.2291	0.3379	0.3716	0.3179	0.2386	0.2221	0.3612
HSE06	0.4062	0.3573	0.3076	0.2868	0.4603	0.4018	0.3534	0.3002	0.2824	0.4833

Binding Energy Calculation Data (Sections 2.10)

Table S12. Binding Energy (kcal/mol) Per ACE Complex, Basis and Level of Theory

	ACE1 + Li						ACE2 + Li				
Level of Theory	SV	SVP	SV(P)	TZV	TZVP	TZVPP	SV	SVP	SV(P)	TZV	TZVP
B2PLYP	-18.821	-18.925	-18.821	-17.024	-12.843	-12.888	-25.462	-25.467	-25.462	-22.699	-16.785
B3LYP	-18.788	-18.757	-18.788	-15.344	-12.126	-12.151	-25.007	-24.821	-25.007	-20.075	-15.576
B971	-17.752	-17.737	-17.752	-15.008	-11.525	-11.543	-23.586	-23.421	-23.586	-19.359	-14.58
CAM-B3LYP	-21.318	-21.294	-21.318	-17.932	-14.293	-14.327	-28.218	-28.04	-28.218	-23.428	-18.318
DSDPBEP86	-20.173	-20.324	-20.174	-19.365	-14.529	-14.58	-27.352	-27.406	-27.352	-25.714	-18.985
HSE06	-18.208	-18.211	-18.208	-15.848	-12.1	-12.12	-24.323	-24.174	-24.323	-20.601	-15.445
M06	-15.317	-15.153	-15.317	-14.253	-9.247	-9.557	-20.076	-19.707	-20.076	-18.799	-11.509
M062X	-20.195	-20.398	-20.195	-18.825	-14.295	-14.258	-26.846	-26.924	-26.846	-24.611	-18.532
MP2	-18.595	-17.411	-18.595	-6.566	7.646	7.716	-28.353	-26.682	-28.353	-18.201	-15.814
PBE	-17.773	-17.695	-17.773	-13.875	-11.07	-11.083	-23.328	-23.082	-23.328	-17.646	-13.771
PBE0	-18.096	-18.118	-18.096	-15.813	-12.056	-12.084	-24.143	-24.019	-24.143	-20.495	-15.323
SCS-MP2	-21.185	-19.969	-21.185	-14.98	-12.347	-12.388	-28.427	-26.743	-28.427	-18.753	-15.89
	ACE1 + Na						ACE2 + Na				
Level of Theory	SV	SVP	SV(P)	TZV	TZVP	TZVPP	SV	SVP	SV(P)	TZV	TZVP
B2PLYP	1.75	1.844	1.749	0.26	0.235	0.219	-25.462	-19.849	-19.945	-17.091	-11.784
B3LYP	-0.248	-0.119	-0.248	0.274	0.307	0.31	-25.007	-19.85	-20.043	-16.315	-11.41
B971	2.136	2.301	2.136	2.314	3.049	3.043	-23.586	-19.034	-19.223	-15.883	-11.132
CAM-B3LYP	1.177	1.231	1.177	0.257	0.298	0.301	-28.218	-23.019	-23.21	-19.418	-14.011
DSDPBEP86	-1.483	-1.364	-1.483	-0.964	-1.021	-1.053	-27.352	-22.449	-22.529	-20.174	-14.647
HSE06	1.337	1.413	1.338	2.291	3.119	3.118	-24.323	-19.335	-19.521	-16.555	-11.518
M06	0.03	0.094	0.026	0.836	1.81	1.791	-20.076	-17.748	-18.071	-14.826	-9.266
M062X	0.424	0.437	0.424	1.339	2.279	2.277	-26.846	-23.171	-23.197	-20.223	-14.807
MP2	-14.82	-17.355	-14.82	-94.75	-113.244	-117.004	-28.353	-21.911	-23.198	-15.069	-15.587
PBE	0.973	1.073	0.971	4.141	4.872	4.874	-23.328	-19.084	-19.313	-15.428	-10.846
PBE0	-0.314	-0.178	-0.314	0.188	0.271	0.191	-24.143	-19.22	-19.393	-16.443	-11.396
SCS-MP2	-0.789	-0.463	-0.789	0.073	-0.39	-0.358	-28.427	-21.709	-23.03	-15.231	-15.15

Table S13. Charge Transfer Per ACE Complex, Basis and Level of Theory

	ACE1 + Li						ACE2 + Li				
Level of Theory	SV	SVP	SV(P)	TZV	TZVP	TZVPP	SV	SVP	SV(P)	TZV	TZVP
B2PLYP	0.3892	0.3897	0.3892	0.3855	0.3833	0.3833	0.389	0.3894	0.389	0.3851	0.383
B3LYP	0.4046	0.4049	0.4046	0.4007	0.3978	0.3978	0.4042	0.4045	0.4042	0.3999	0.3973
B971	0.3836	0.3839	0.3836	0.3797	0.3767	0.3767	0.3832	0.3835	0.3832	0.3792	0.3763
CAM-B3LYP	0.3997	0.4001	0.3997	0.4013	0.3984	0.3984	0.3994	0.3997	0.3993	0.4006	0.398
DSDPBEP86	0.4123	0.4126	0.4123	0.4077	0.4044	0.4044	0.4119	0.4122	0.4119	0.4073	0.4085

HSE06	0.3975	0.3979	0.3975	0.3937	0.3907	0.3907	0.3971	0.3976	0.3971	0.3933	0.3902
M06	0.3809	0.3815	0.3809	0.3775	0.3753	0.3754	0.3807	0.3813	0.3807	0.3771	0.3752
M062X	0.4073	0.4074	0.4073	0.407	0.4016	0.3862	0.4067	0.4068	0.4067	0.4061	0.4008
MP2	0.3891	0.3897	0.3891	0.3851	0.3821	0.382	0.3888	0.3893	0.3888	0.3846	0.3817
PBE	0.3729	0.3736	0.3729	0.369	0.3674	0.3674	0.3728	0.3735	0.3728	0.3687	0.3673
PBE0	0.3728	0.3736	0.3728	0.3689	0.3673	0.3674	0.3727	0.3734	0.3727	0.3685	0.3672
SCS-MP2	0.403	0.4034	0.403	0.3992	0.3962	0.3962	0.4027	0.4031	0.4027	0.3988	0.3957

	ACE1 + Na						ACE2 + Na				
Level of Theory	SV	SVP	SV(P)	TZV	TZVP	TZVPP	SV	SVP	SV(P)	TZV	TZVP
B2PLYP	0.3334	0.3341	0.3334	0.3319	0.3286	0.3287	0.3332	0.3339	0.3332	0.3314	0.3282
B3LYP	0.3555	0.3559	0.3555	0.3512	0.3467	0.3468	0.355	0.3554	0.355	0.3501	0.3461
B971	0.3247	0.3251	0.3247	0.3231	0.3188	0.3188	0.3243	0.3247	0.3243	0.3225	0.3182
CAM-B3LYP	0.3452	0.3457	0.3452	0.3436	0.3391	0.3392	0.3448	0.3452	0.3447	0.3425	0.3385
DSDPBEP86	0.3665	0.3669	0.3665	0.361	0.3557	0.3558	0.366	0.3665	0.366	0.3605	0.3632
HSE06	0.3436	0.3442	0.3436	0.3418	0.3372	0.3373	0.3432	0.3438	0.3432	0.3413	0.3366
M06	0.3221	0.3229	0.3221	0.3223	0.3193	0.3194	0.3219	0.3227	0.3219	0.3219	0.319
M062X	0.3673	0.3675	0.3673	0.3599	0.3513	0.3299	0.3665	0.3667	0.3665	0.3586	0.3501
MP2	0.3352	0.336	0.3352	0.3337	0.3292	0.3292	0.3348	0.3356	0.3348	0.3329	0.3286
PBE	0.3136	0.3145	0.3136	0.3144	0.3121	0.3122	0.3135	0.3144	0.3135	0.314	0.312
PBE0	0.3135	0.3145	0.3135	0.3143	0.3121	0.3122	0.3133	0.3143	0.3133	0.3138	0.3119
SCS-MP2	0.3528	0.3535	0.3528	0.3498	0.345	0.3451	0.3524	0.353	0.3524	0.3492	0.3444

Binding Energy and Charge Transfer Graphical Results (Sections 2.6-2.9)

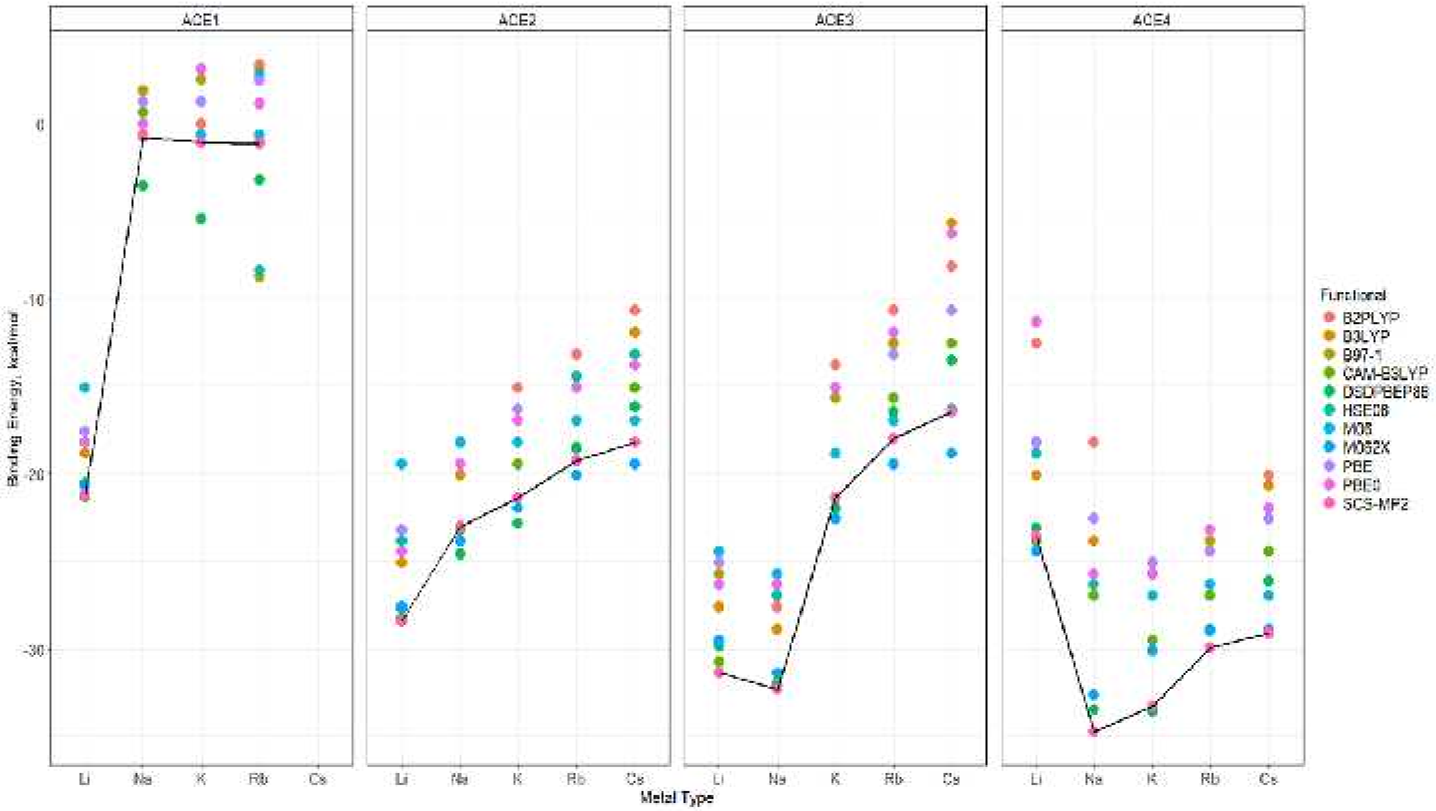

Figure S35. Binding energy diagrams for ACE-M+ faceted by the ACE type and colored by the functional based on def2sv, SCS-MP2 reference line.

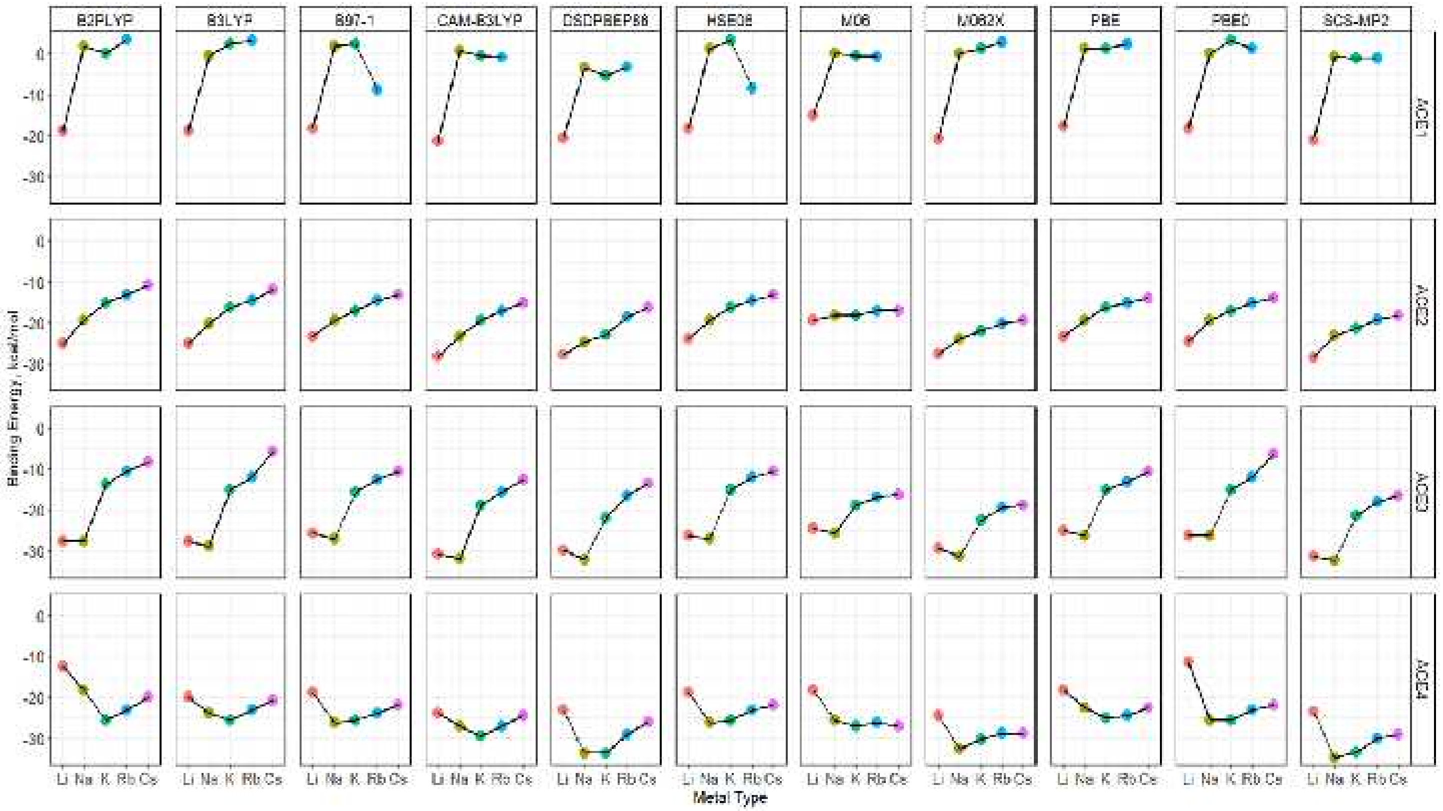

Figure S36. Binding energy diagrams for ACE-M+ faceted by the ACE type and the functional type and colored by the functional based on def2sv.

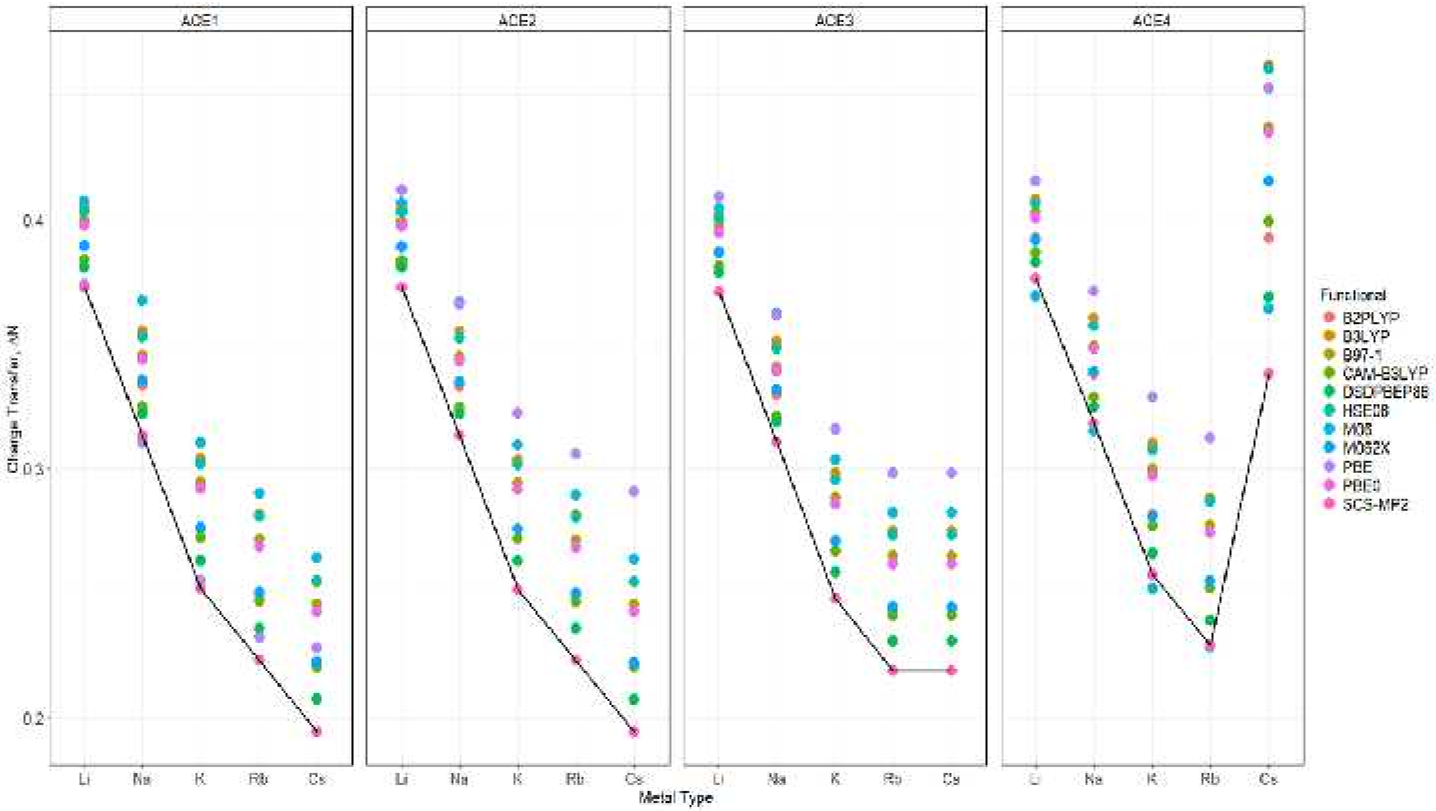

Figure S37. Charge transfer diagrams for ACE-M+ faceted by the ACE type and colored by the functional based on def2sv, SCS-MP2 reference line.

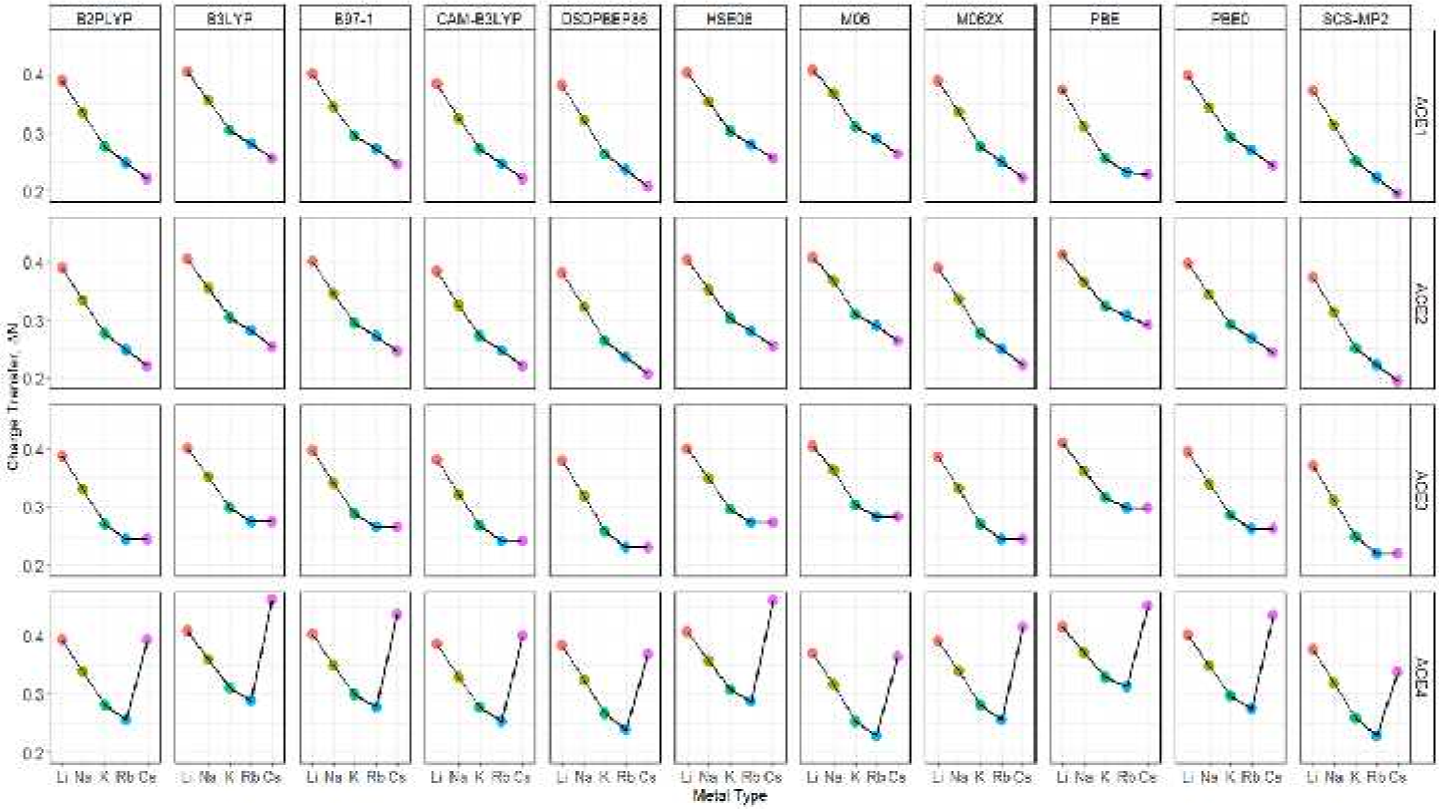

Figure S38. Charge transfer diagrams for ACE-M+ faceted by the ACE type and the functional type and colored by the functional based on def2sv.

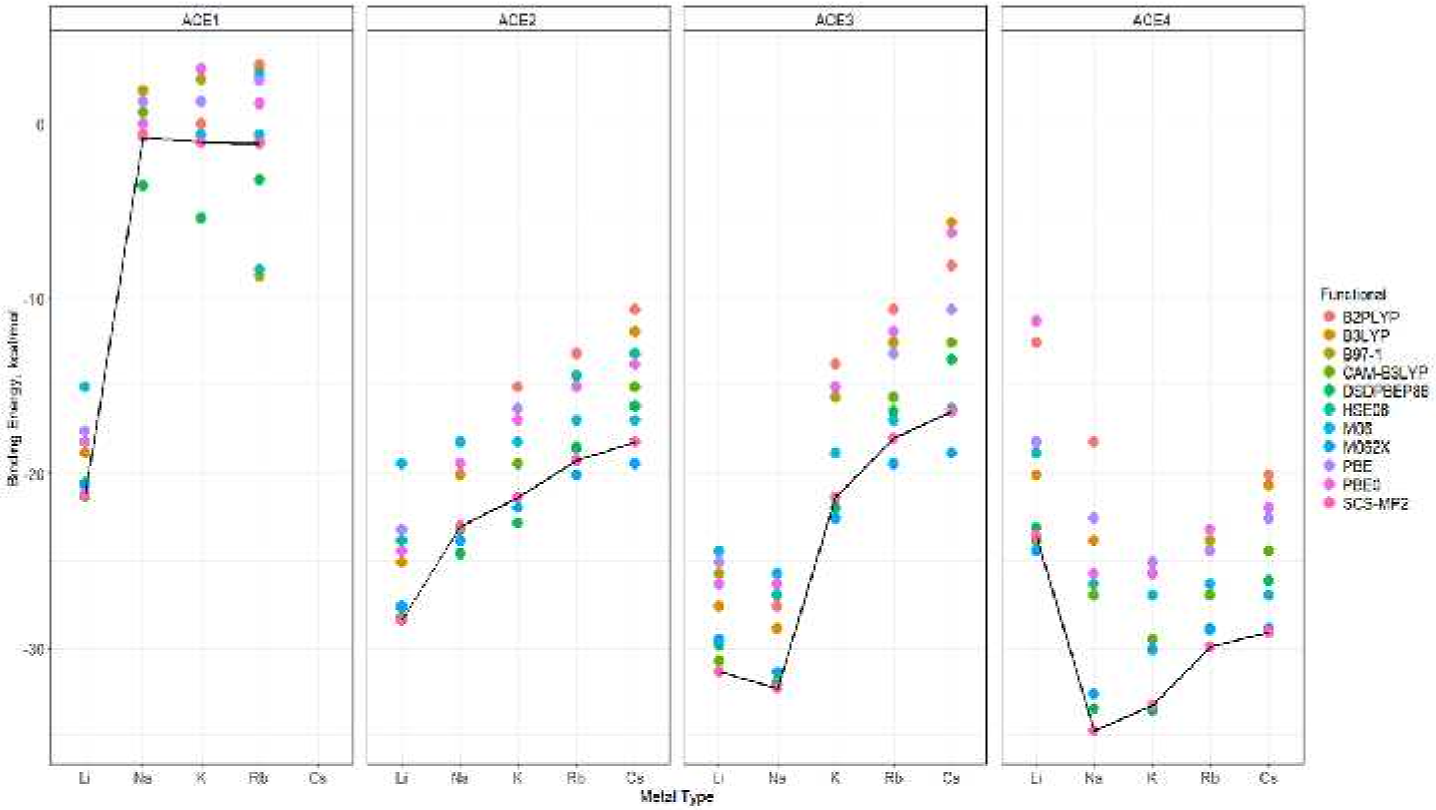

Figure S39. Charge transfer versus binding energy diagrams for ACE-M⁺ faceted by the ACE type, colored by the ion type, and calculated by different functionals based on def2sv.

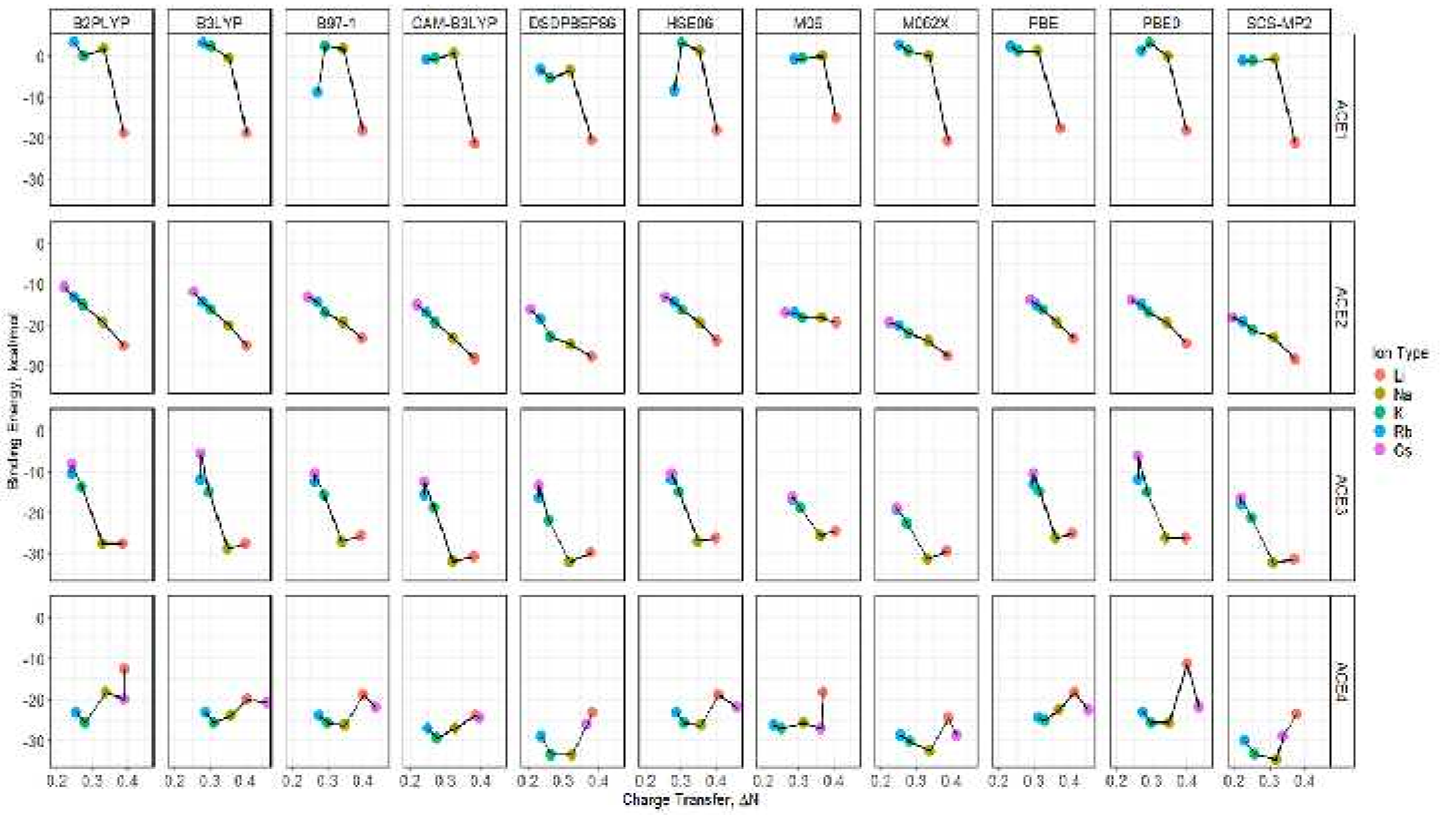

Figure S40. Charge transfer versus binding energy diagrams for ACE-M⁺ faceted by the ACE type and the functional type and colored by the ion type based on def2sv.

Binding Energy, Charge Transfer Graphical Results (Sections 2.10)

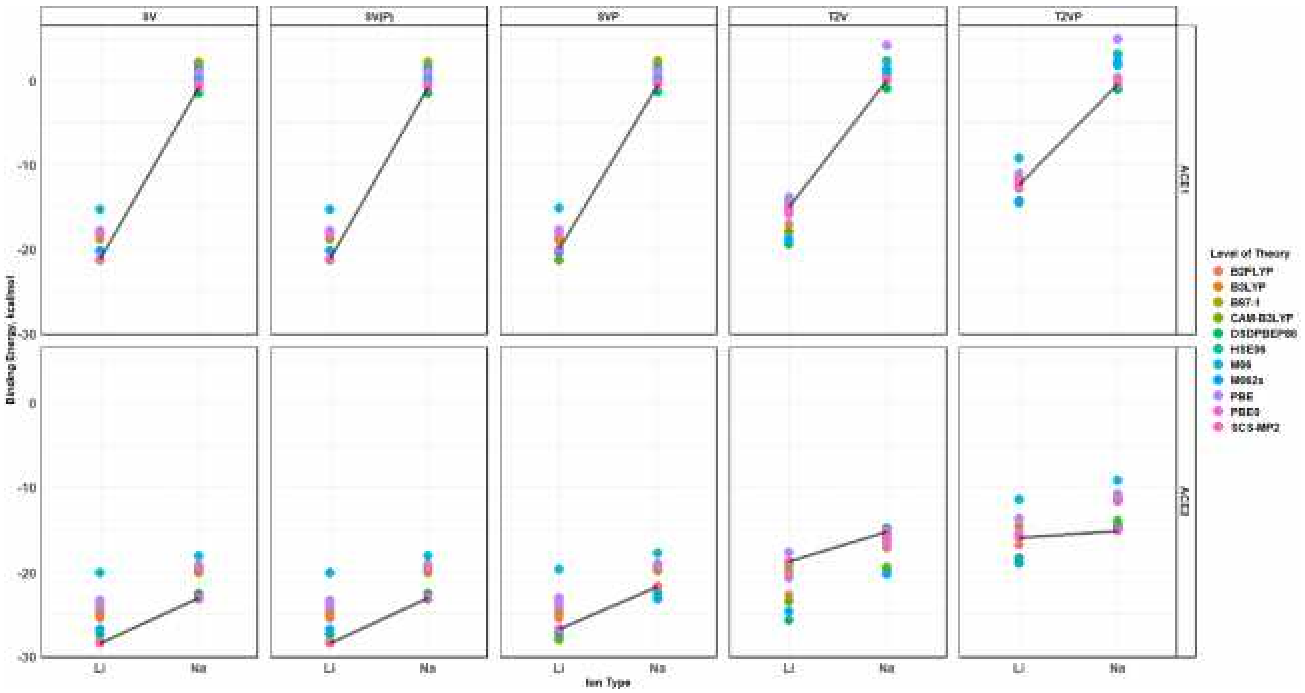

Figure S41. Binding Energy versus Ion type present, faceted by Basis and ACE Type; Colored by Level of Theory

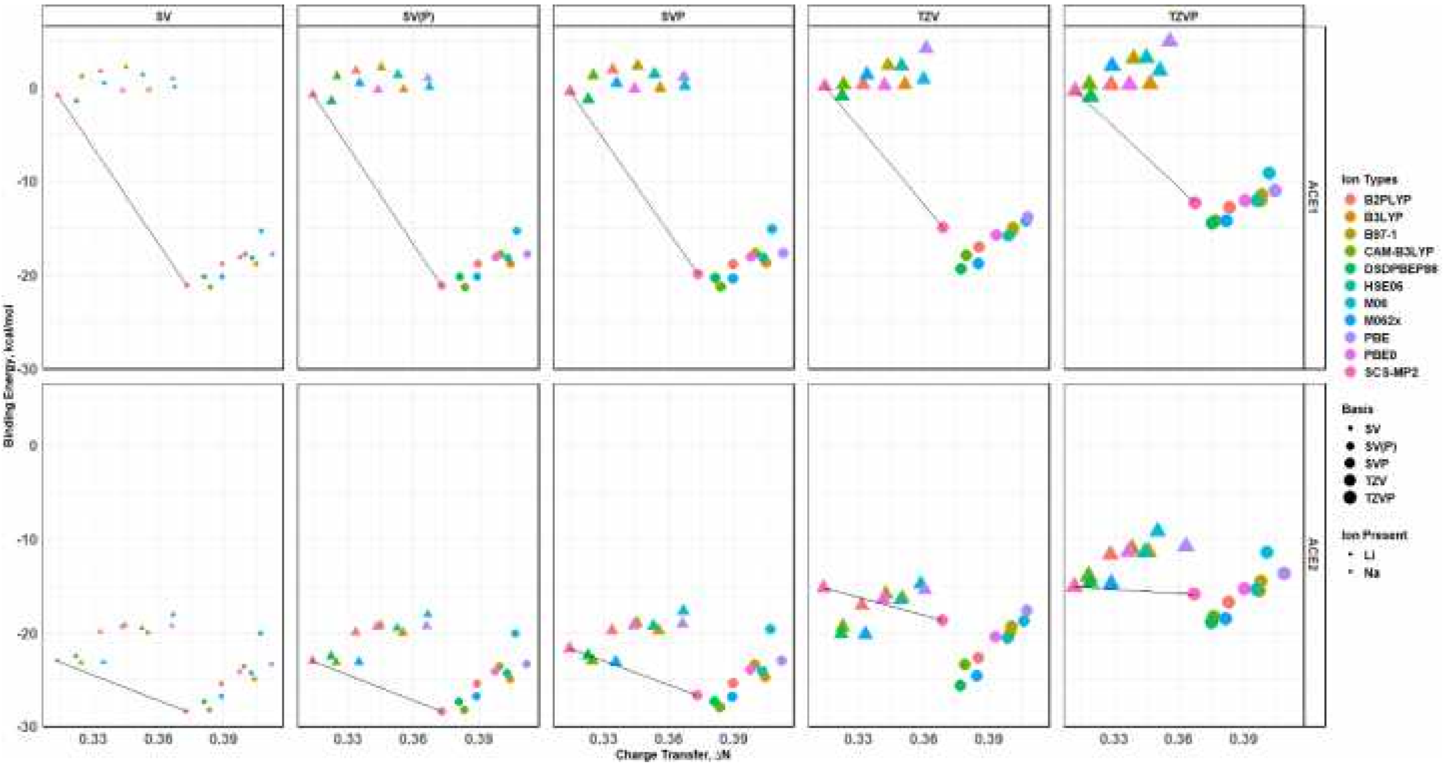

Figure S42. Binding Energy versus Charge Transfer, faceted by Basis and ACE Type; Colored by Level of Theory and Shaped by Ion Present; Line SCS-MP2/Basis

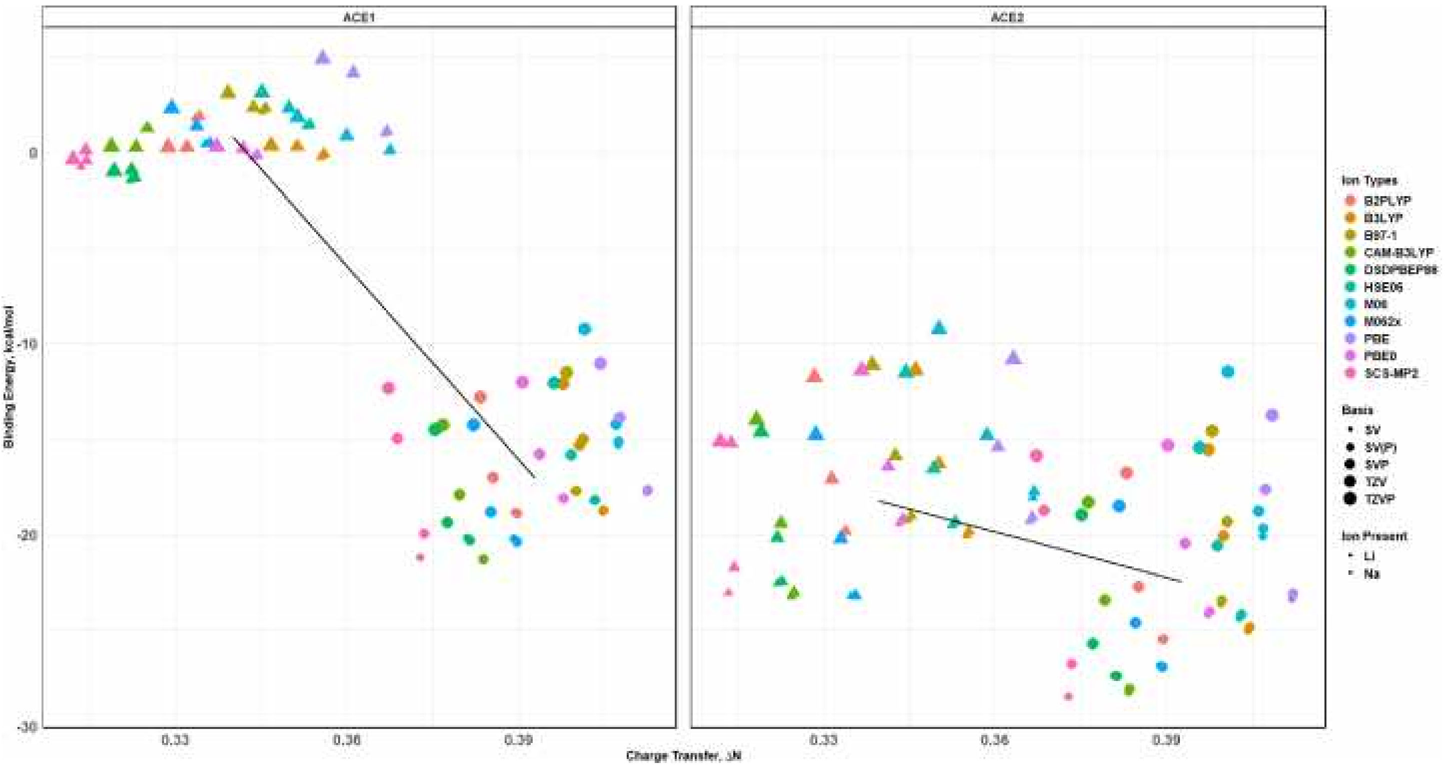

Figure S43. General Binding Energy versus Charge Transfer, faceted by ACE Type; Colored by Level of Theory; Shaped by Ion Present; Sized by Basis; Line Cluster Average

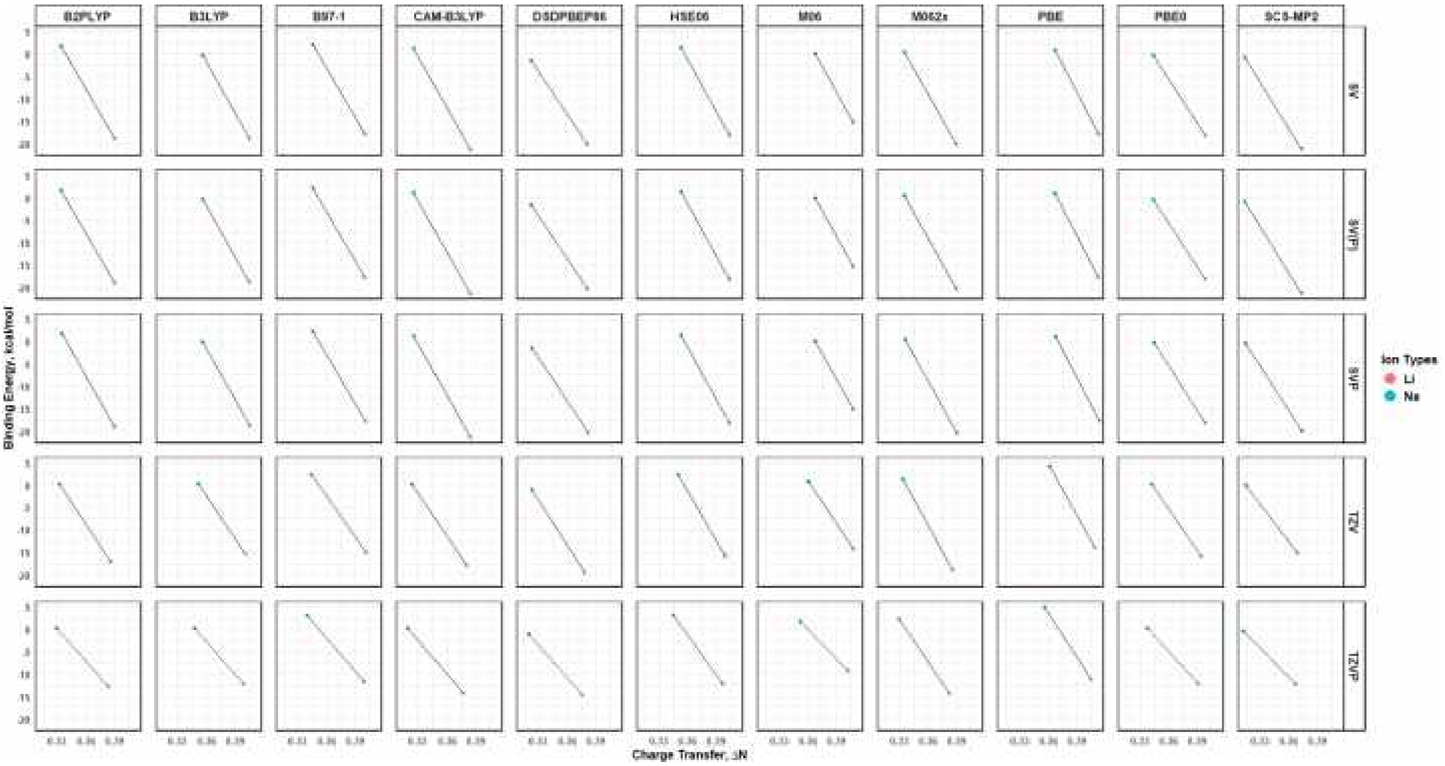

Figure S44. Binding Energy versus Charge Transfer of ACE1, faceted by Level of Theory and Basis; Colored by Ion Present

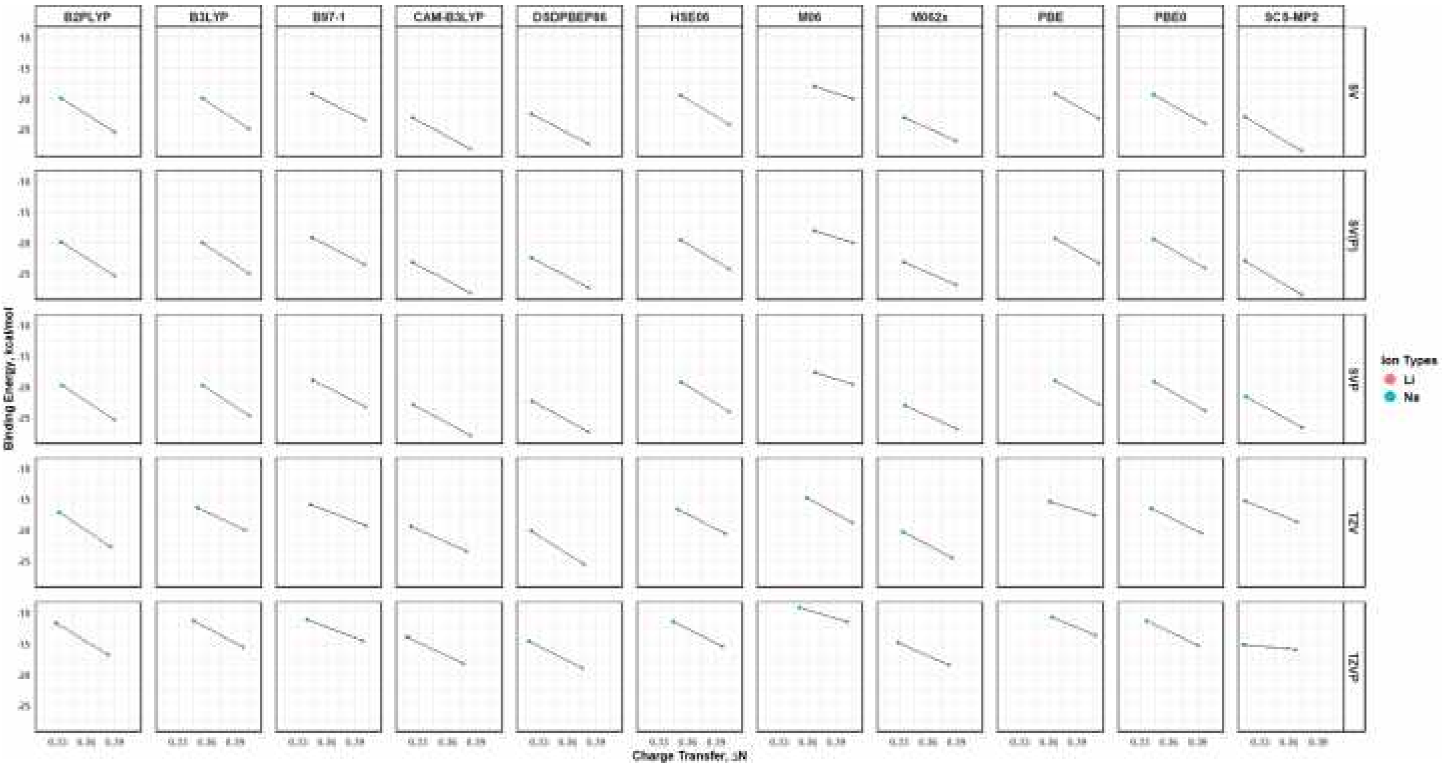

Figure S45. Binding Energy versus Charge Transfer of ACE2, faceted by Level of Theory and Basis; Colored by Ion Present

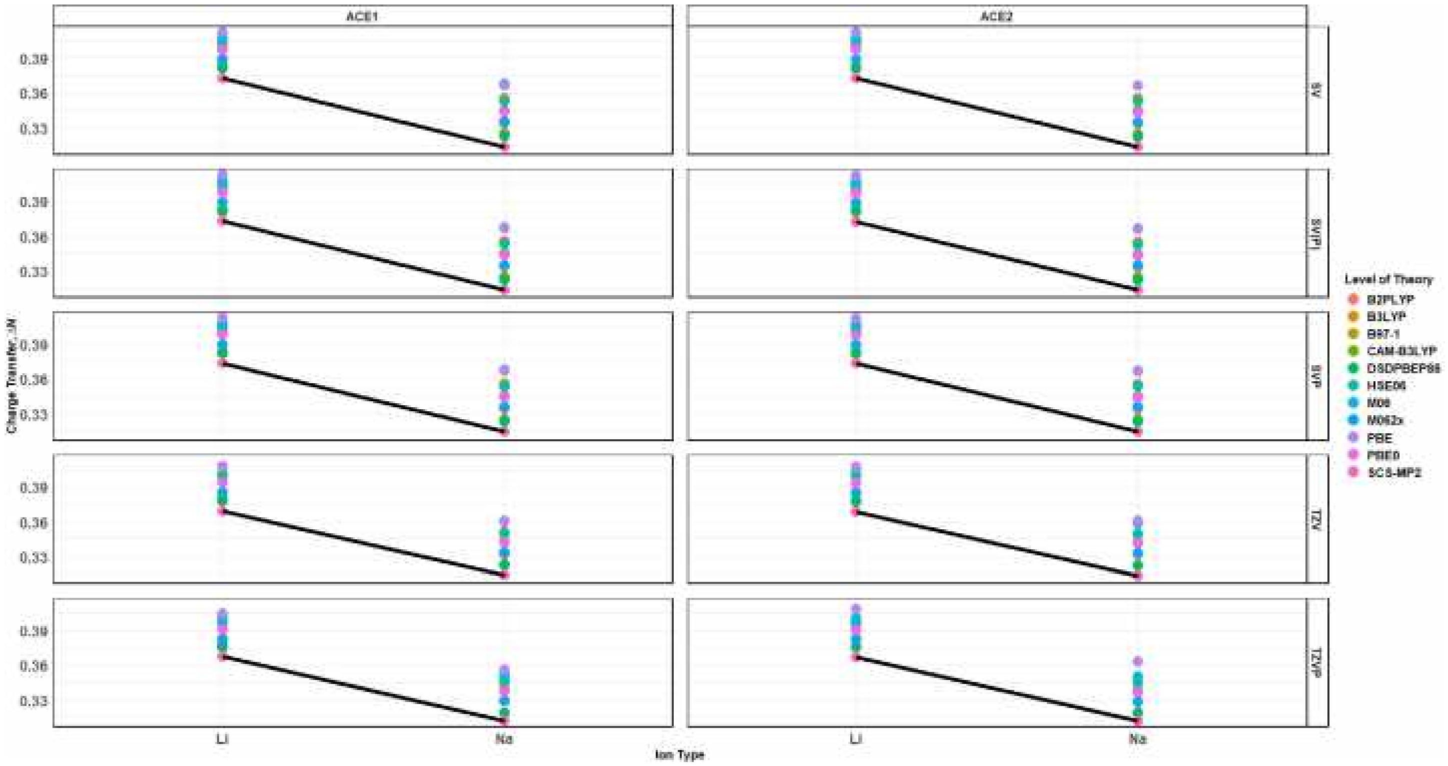

Figure S46. Charge Transfer of ACE2, faceted by Level of Theory and Basis; Colored by Ion Present

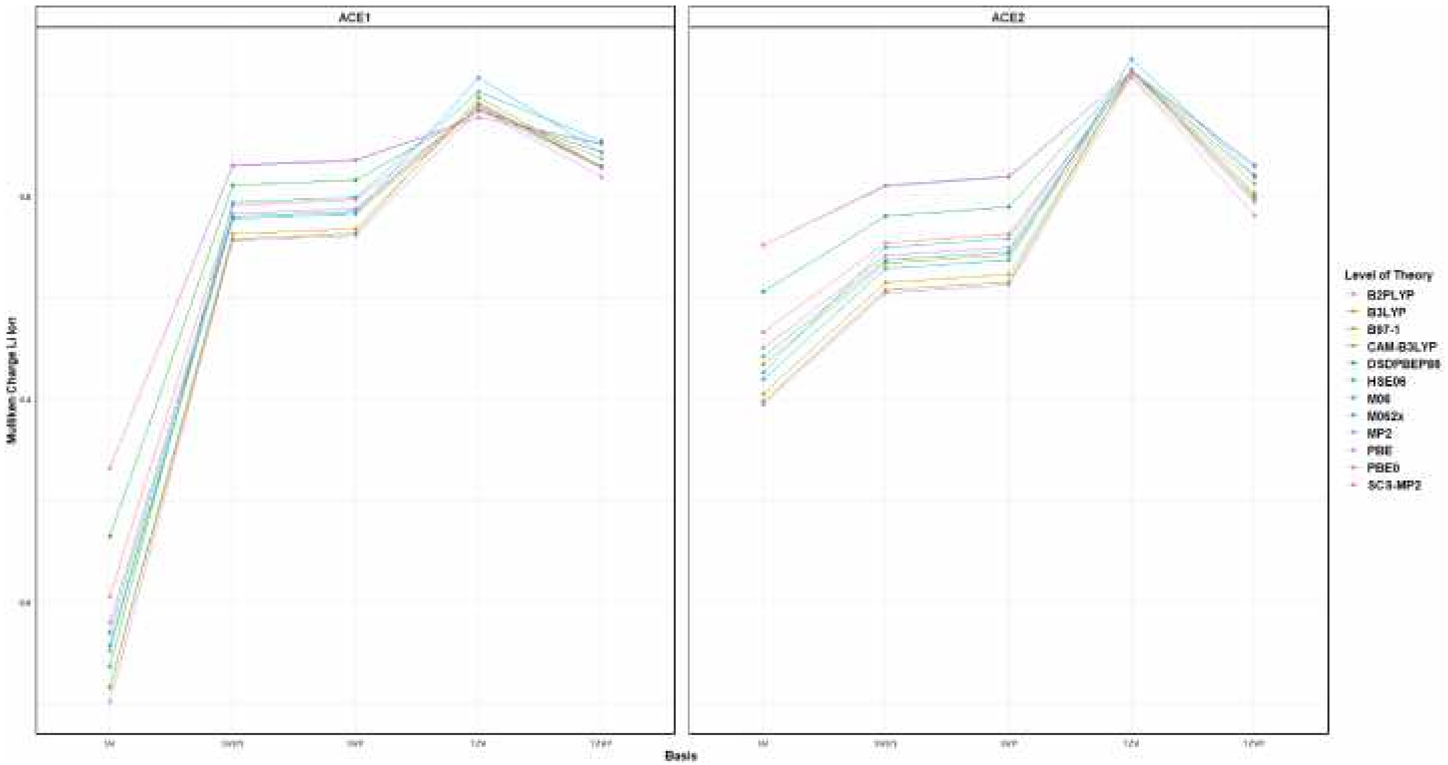

Figure S47. Mulliken Charge of Li^{+} per Basis, faceted by ACE Type; Colored by Level of Theory

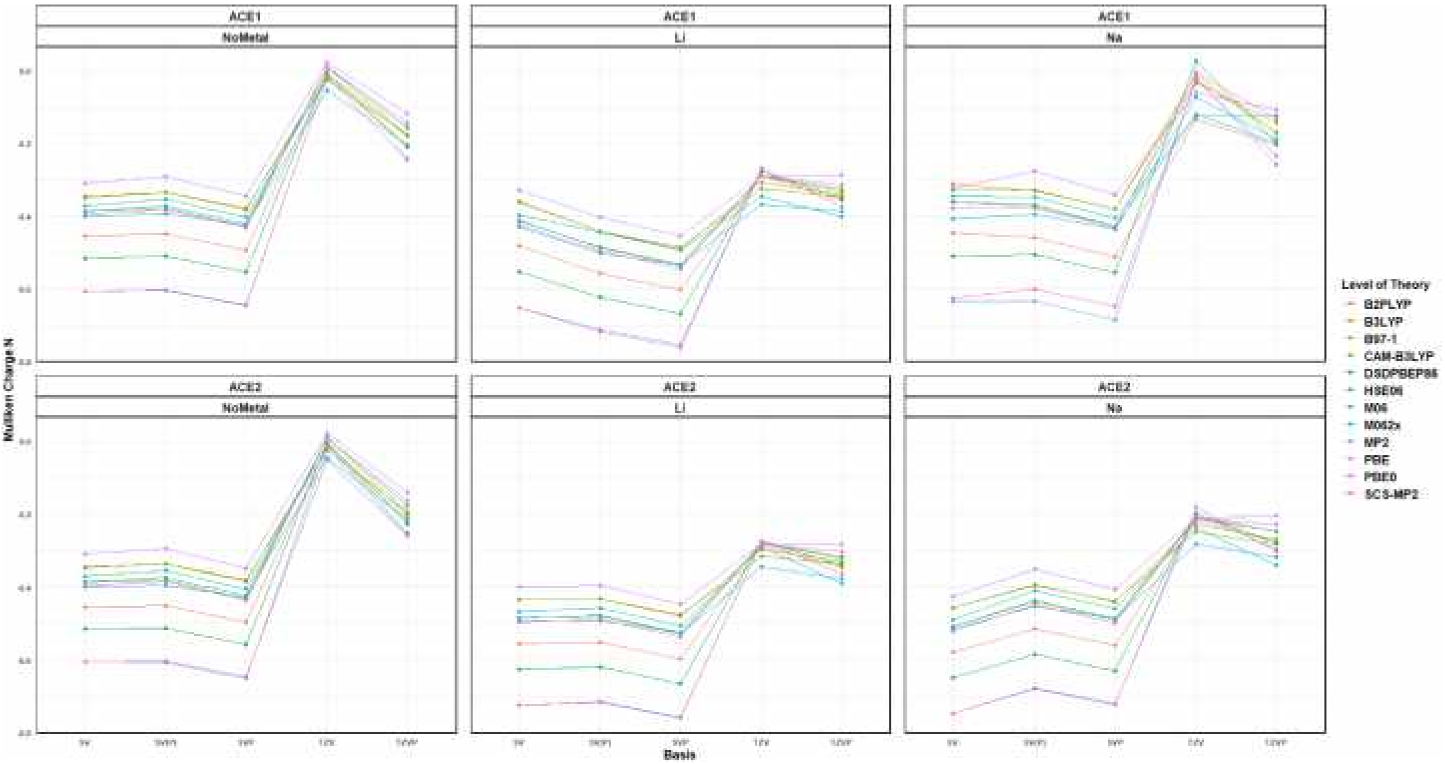

Figure S48. Mulliken Charge of Nitrogen Atom per Basis, faceted by ACE Type and Ion Present; Colored by Level of Theory

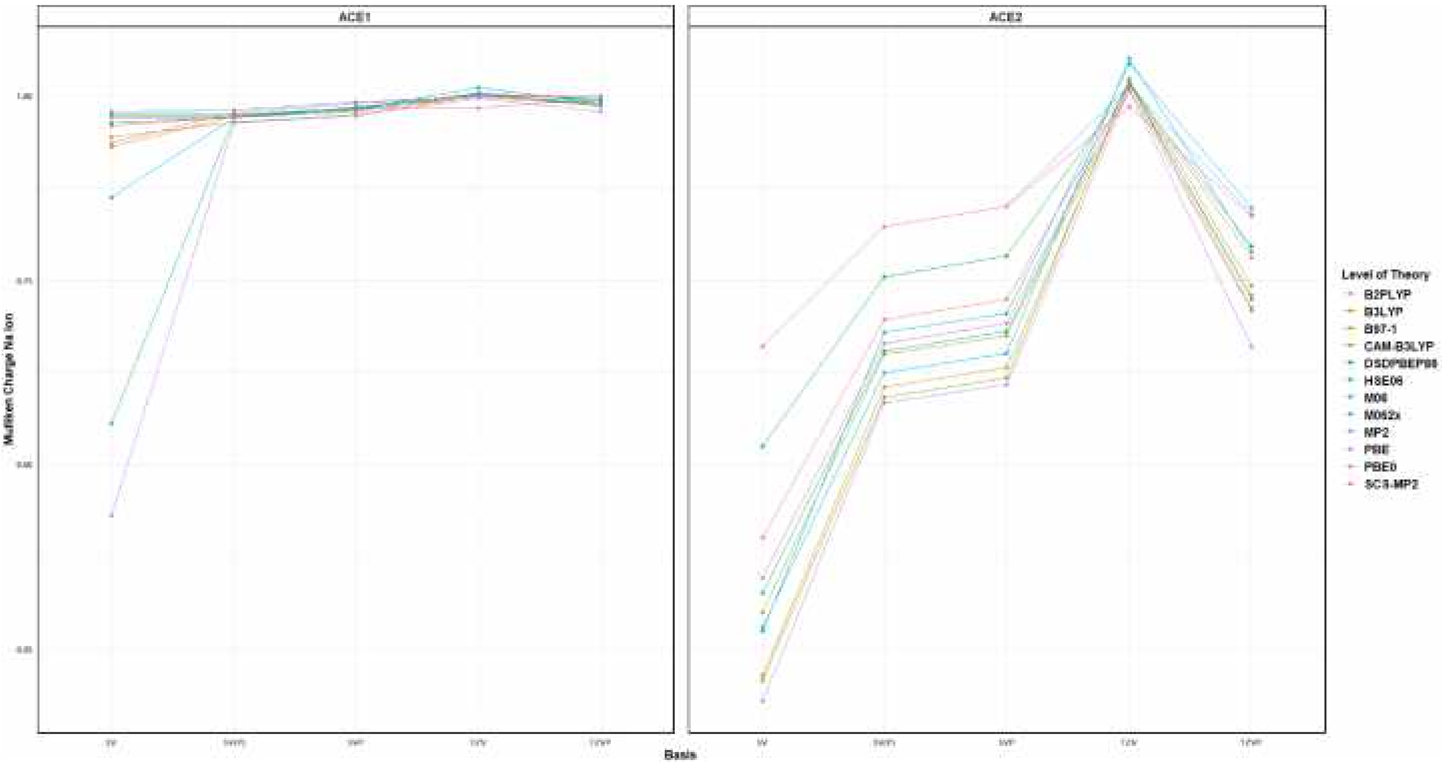

Figure S49. Mulliken Charge of Na^{+} per Basis, faceted by ACE Type; Colored by Level of Theory

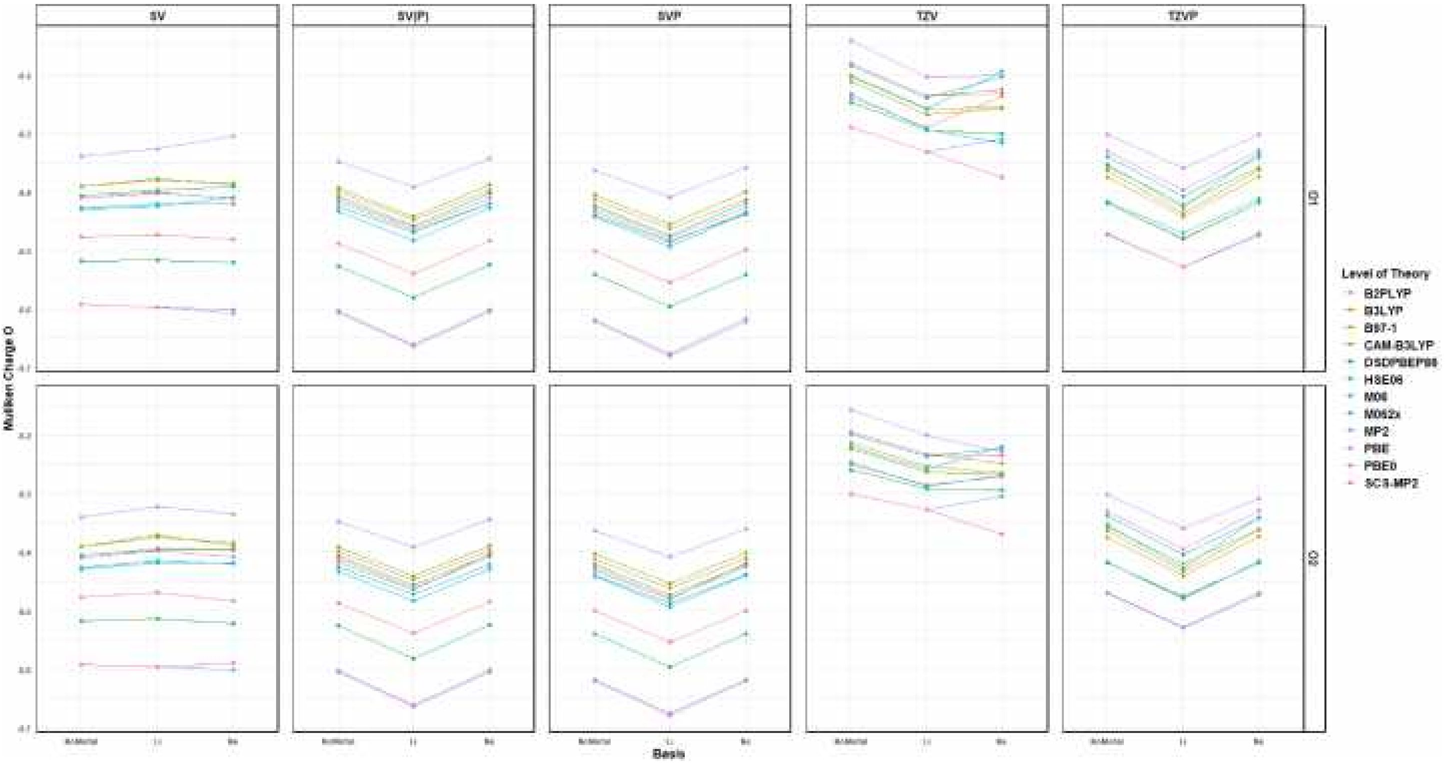

Figure S50. Mulliken Charge of Oxygen Atoms of ACE1 per Ionic Presence, faceted by Basis and Oxygen Number; Colored by Level of Theory

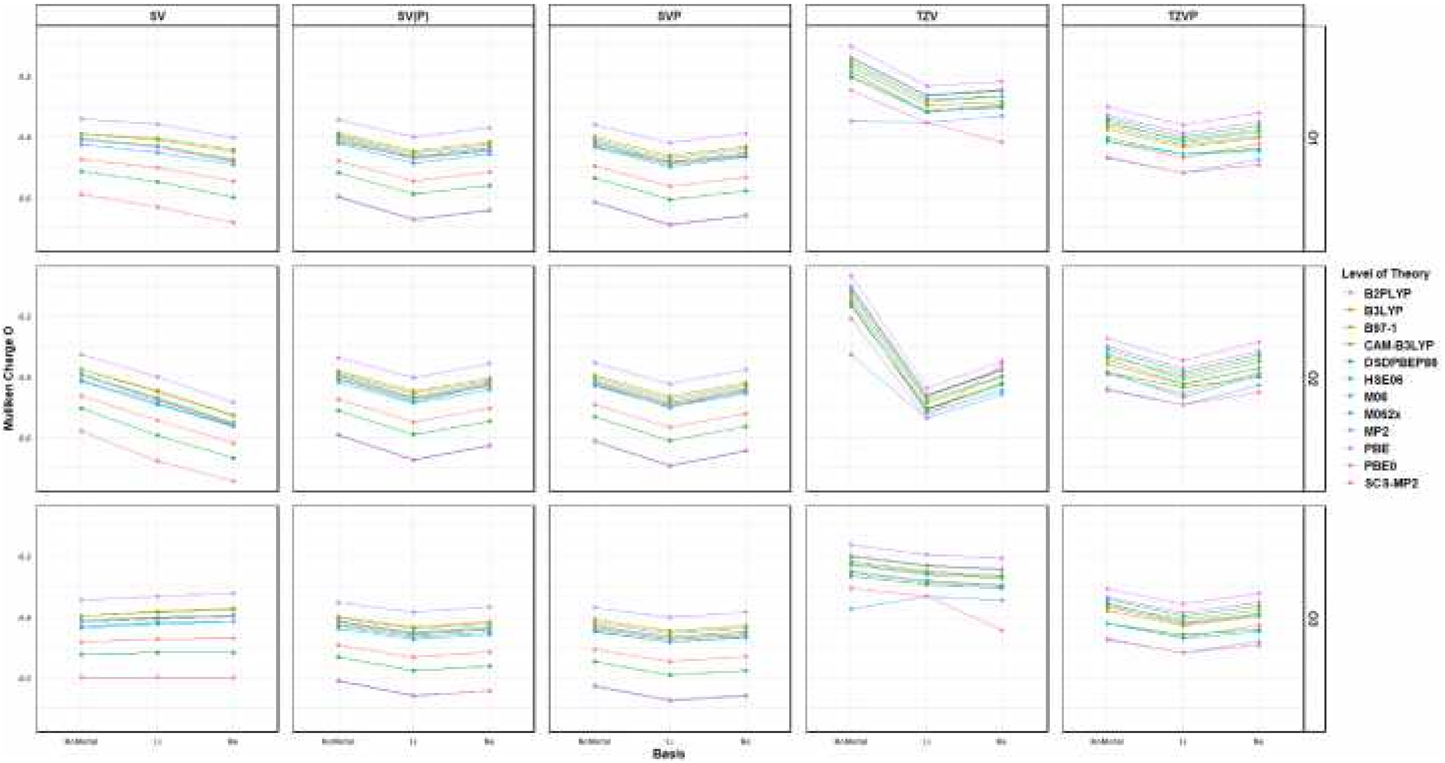

Figure S51. Mulliken Charge of Oxygen Atoms of ACE2 per Ionic Presence, faceted by Basis and Oxygen Number; Colored by Level of Theory

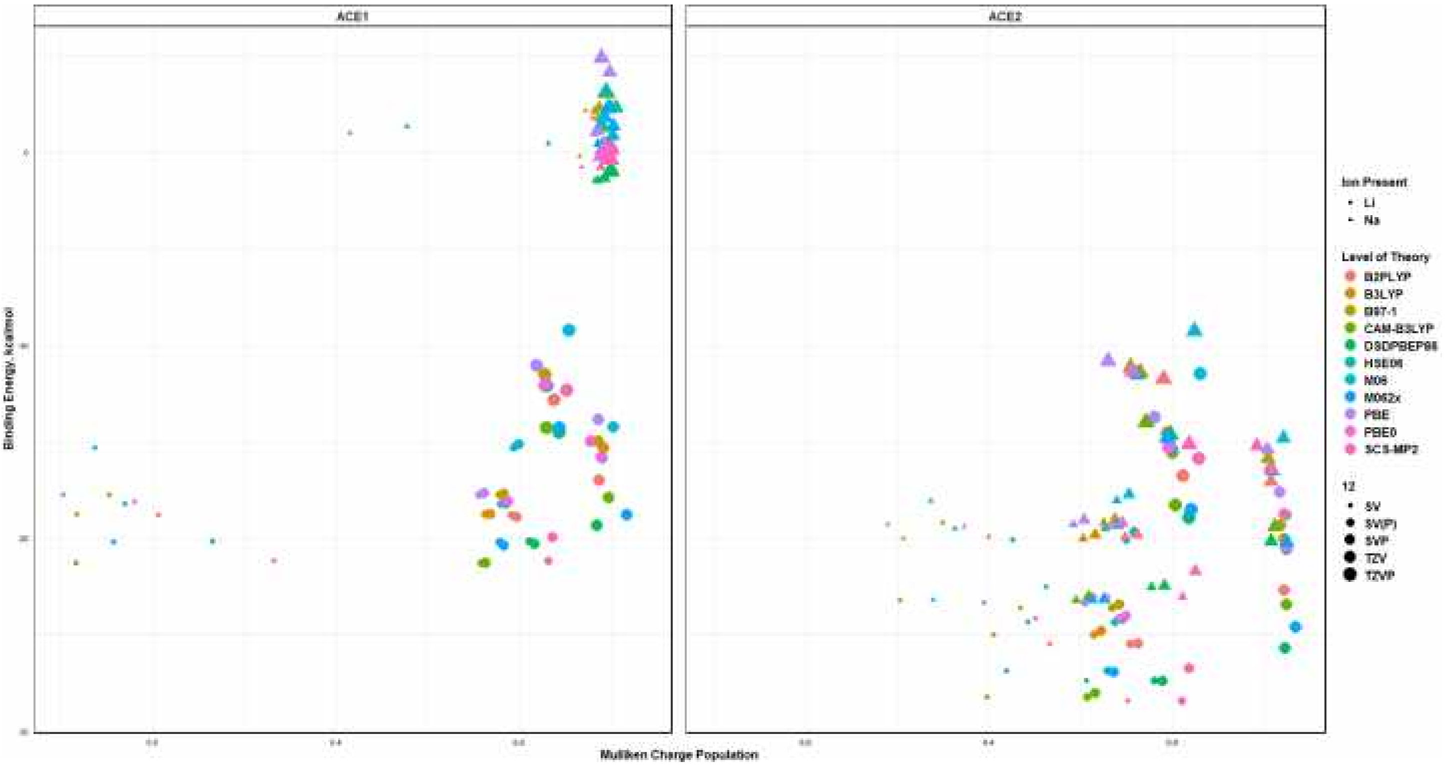

Figure S51. Binding Energy and Mulliken Charge of Metal Ion Type, faceted by ACE Type; Colored by Level of Theory; Size by Basis

Excited States Calculation Results (Section 2.11)

Table S14. Excited States Calculation Results of ACE1 and Metal Ion Types

	Wavelength (nm)	Energy (eV)	Total Oscillatory Strength (Excited State)	Electronic Transitions	Oscillatory Strength Per Transition
No Metal	197.92	6.2642	0.0537 (2)	**40 -> 41**	**0.6523**
				40 -> 42	-0.2048
Li^+	159.8	7.7589	0.0543 (7)	39 -> 42	-0.1874
				40 -> 42	0.2235
				41 -> 42	0.1200
				**41 -> 43**	**0.5239**
				41 -> 44	0.2533
				41 -> 45	-0.1311
Na^+	196.34	6.3147	0.0443 (4)	45 -> 47	0.4367
				**45 -> 48**	**0.5125**

Table S15. Excited States Calculation Results of ACE2 and Metal Ion Types

	Wavelength (nm)	Energy (eV)	Total Oscillatory Strength (Excited State)	Electronic Transitions	Oscillatory Strength Per Transition
No Metal	194.27	6.3821	0.0573 (2)	**52 -> 53**	**0.6556**
				52 -> 54	-0.1185
				52 -> 55	0.1522
				52 -> 56	0.1087

Li^+	160.02	7.748	0.0575 (7)	**52 -> 54**	**0.4511**
				52 -> 58	-0.1460
				53 -> 55	-0.1598
				53 -> 56	-0.3892
				53 -> 57	0.1308
				53 -> 60	-0.1105
Na^+	166.24	7.4583	0.1051 (7)	57 -> 58	0.1118
				57 -> 59	0.1847
				**57 -> 60**	**0.6149**
				57 -> 64	0.1565
K^+	173.14	7.1611	0.1096 (5)	61 -> 62	0.1244
				61 -> 63	0.2959
				**61 -> 64**	**0.5677**
				61 -> 66	-0.1447
				61 -> 68	0.1497
Rb^+	197.04	6.2923	0.0419 (4)	56 -> 57	0.1764
				**56 -> 58**	**0.5082**
				56 -> 59	-0.2162
				56 -> 60	-0.2019
				56 -> 61	0.2057
				56 -> 63	-0.1811
				56 -> 63	-0.1164
Cs^+	191.52	6.4737	0.0578 (6)	56 -> 57	-0.1902
				56 -> 58	-0.1857
				56 -> 59	0.3524
				**56 -> 60**	**0.4373**
				56 -> 62	-0.1205
				56 -> 63	0.1062
				56 -> 64	-0.2491

`

Table S16. Excited States Calculation Results of ACE3 and Metal Ion Types

	Wavelength (nm)	Energy (eV)	Total Oscillatory Strength (Excited State)	Electronic Transitions	Oscillatory Strength Per Transition
No Metal	188.8	6.5671	0.0697 (2)	64 -> 65	0.2998
				**64 -> 66**	**0.5393**
				64 -> 67	0.2815
Li^+	160.31	7.7342	0.0283 (9)	62 -> 66	-0.1137
				63 -> 66	-0.1578
				65 -> 67	-0.3937
				**65 -> 68**	**0.4120**
				65 -> 70	-0.1172
				65 -> 71	-0.1986
Na^+	165.93	7.4719	0.0173 (7)	**68 -> 70**	**0.4923**
				68 -> 71	0.1008
				68 -> 74	0.1431
				69 -> 71	-0.2702
				69 -> 72	0.2625
				69 -> 75	0.1155

K^+	179.41	6.9108	0.1002 (4)	**73 -> 75**	**0.6109**
				73 -> 76	-0.2168
				73 -> 77	-0.1190
				73 -> 78	-0.1417
				73 -> 83	0.1187
Rb^+	202.47	6.1235	0.0567 (4)	68 -> 69	-0.2516
				**68 -> 70**	**0.5689**
				68 -> 72	0.1289
				68 -> 76	-0.1764
				68 -> 77	0.1541
				68 -> 80	-0.1004
Cs^+	199.65	6.21	0.0542 (4)	**68 -> 70**	**0.6144**
				68 -> 72	0.1900
				68 -> 75	0.1010
				68 -> 77	-0.1538

Table S17: Excited States Calculation Results of ACE4 and Metal Ion Types

	Wavelength (nm)	Energy (eV)	Total Oscillatory Strength (Excited State)	Electronic Transitions	Oscillatory Strength Per Transition
No Metal	173.38	7.1509	0.0517 (9)	76 -> 78	0.1570
				76 -> 80	-0.2623
				76 -> 81	0.3129
				**76 -> 83**	**0.3134**
				76 -> 84	-0.2670
				76 -> 85	-0.2272
				76 -> 88	0.1626
				76 -> 92	-0.1297
Li^+	176.79	7.0131	0.0231 (8)	77 -> 81	-0.3386
				77 -> 83	-0.1719
				77 -> 85	-0.3071
				**77 -> 86**	**0.3894**
				77 -> 88	-0.1178
				77 -> 89	0.1954
				77 -> 91	0.1396
Na^+	177.43	6.9878	0.0433 (10)	81 -> 83	0.1402
				81 -> 84	0.2892
				81 -> 85	-0.3301
				**81 -> 86**	**0.3368**
				81 -> 87	-0.1771
				81 -> 88	0.1402
				81 -> 91	0.1867
				81 -> 94	0.1121
K^+	171.76	7.2184	0.0838 (7)	85 -> 86	0.1573

				85 -> 88	0.3797
				**85 -> 89**	**0.3926**
				85 -> 91	0.2708
				85 -> 92	0.1061
				85 -> 93	0.2002
				85 -> 94	0.1046
Rb^{+}	190.25	6.5168	0.0436 (8)	80 -> 81	0.2995
				**80 -> 82**	**0.3459**
				80 -> 84	-0.1042
				80 -> 85	0.2391
				80 -> 86	-0.1709
				80 -> 87	0.2607
				80 -> 88	0.1433
				80 -> 89	-0.2320
				80 -> 90	0.1443
Cs^{+}	187.06	6.6281	0.0427 (8)	80 -> 81	0.3641
				80 -> 82	0.1416
				80 -> 83	0.1825
				80 -> 84	-0.2720
				**80 -> 87**	**0.3798**
				80 -> 88	0.2061
				80 -> 90	0.1089
				80 -> 92	0.1164

Output Geometry Optimization (XYZ format) (Section 2.1-2.9)

ACE1 Cs B2PLYP def2sv

26

N	3.90222	-2.15826	0.42088
C	-5.80790	-1.11744	-0.16189
H	-5.94249	-1.71865	-1.06991
H	-6.03547	-1.70658	0.73544
C	-4.42765	-0.41987	-0.09665
H	-4.29551	0.23359	-0.98118
H	-4.38083	0.24070	0.79143
O	-3.38951	-1.38535	-0.04252
C	-2.10284	-0.80474	0.00259
H	-1.93961	-0.16936	-0.89308

H	-2.00488	-0.15561	0.89772
C	-0.99685	-1.86634	0.05168
H	-1.11042	-2.49164	0.95594
H	-1.05375	-2.51968	-0.83790
O	0.27030	-1.17983	0.07996
C	1.42394	-2.03259	0.26026
H	1.28402	-2.65275	1.16570
H	1.50005	-2.72173	-0.60588
C	2.67842	-1.18287	0.38841
H	2.73689	-0.61782	1.31034
H	2.75096	-0.48725	-0.46965
C	4.85885	-1.81569	-0.71117
H	4.31878	-1.96601	-1.63970
H	5.71337	-2.51104	-0.67710
H	5.20370	-0.79629	-0.55489
Cs	0.49341	2.02012	0.00414

ACE1 Cs B3LYP def2sv

26

N	4.88631	0.06276	-0.33588
C	-5.70201	-0.60731	-0.26774
H	-6.03185	-0.11873	0.65849
H	-5.95388	-0.10101	-1.20878
C	-5.03068	-1.76405	-0.25084
H	-4.78157	-2.27219	0.68996
H	-4.70369	-2.25412	-1.17721
O	-0.12835	-1.05623	0.15224
C	1.01219	-1.73243	0.37668
H	0.92603	-2.60060	1.10454
H	1.45632	-2.22448	-0.54589
C	2.15161	-0.89422	0.98653

H	1.79711	-0.40588	1.90919
H	3.03561	-1.51598	1.23389
O	2.55915	0.20725	0.13202
C	3.45926	-0.08006	-0.84890
H	3.38192	-1.10126	-1.25779
H	3.37072	0.66286	-1.65765
C	5.74159	-0.87546	-0.47431
H	5.44592	-1.81196	-0.96049
H	6.76793	-0.74275	-0.11353
C	5.20194	1.34732	0.30233
H	5.10820	2.14249	-0.45504
H	6.22225	1.32194	0.70686
H	4.46843	1.52293	1.10220
Cs	-2.09388	0.79070	0.02011

ACE1 Cs B97-1 def2sv

26

N	2.74367	-0.08906	-0.19616
C	-3.98845	0.85165	0.01883
H	-3.71260	1.19033	1.02510
H	-4.77956	1.37707	-0.52734
C	-3.37803	-0.33870	-0.57063
H	-4.16727	-1.14112	-0.63052
H	-3.12071	-0.15529	-1.64708
O	-2.27032	-0.78680	0.15869
C	-1.82950	-2.08780	-0.19909
H	-2.66137	-2.81450	-0.08621
H	-1.50807	-2.10658	-1.26188
C	-0.69495	-2.50652	0.72900
H	-1.03237	-2.38665	1.77437
H	-0.48186	-3.58240	0.55766

O	0.48542	-1.74226	0.60096
C	1.32566	-2.08675	-0.48881
H	1.39674	-3.19129	-0.57527
H	0.91205	-1.69852	-1.44479
C	2.72018	-1.50935	-0.26373
H	3.38124	-1.90644	-1.08053
H	3.14269	-1.96641	0.67213
C	4.05881	0.43271	-0.03737
H	4.04769	1.53834	0.01219
H	4.75197	0.13364	-0.86826
H	4.56025	0.05432	0.89334
Cs	0.09204	1.47236	0.06098

ACE1 Cs CAM-B3LYP def2sv

26

N	0.88786	-1.56736	-0.07950
C	1.44993	-1.38467	1.25213
H	0.67529	-1.71153	1.96624
H	2.32671	-2.05077	1.42355
C	1.89191	0.02086	1.64406
H	2.07527	0.01202	2.73436
H	2.85375	0.27204	1.17157
O	0.92550	1.00613	1.33143
C	1.39201	2.18567	0.71282
H	1.01298	3.06363	1.26354
H	2.49258	2.22966	0.77044
C	0.90796	2.27379	-0.74666
H	-0.10589	2.70296	-0.78623
H	1.57046	2.95954	-1.31001
O	0.80494	1.01741	-1.37269
C	1.99302	0.26017	-1.46070

H	2.43200	0.36404	-2.47072
H	2.74794	0.64134	-0.76024
C	1.71845	-1.22319	-1.22654
H	2.70182	-1.74528	-1.20172
H	1.18942	-1.60346	-2.11503
C	0.38278	-2.92493	-0.21297
H	-0.32260	-3.15089	0.60411
H	1.19144	-3.68858	-0.17859
H	-0.15191	-3.04153	-1.16989
Cs	-1.83935	0.07789	0.02132

ACE1 Cs DSDPBEP86 def2sv

26

N	-2.82765	0.00570	0.21092
C	3.73102	0.76699	-0.86880
H	3.39222	0.69294	-1.90826
H	4.50700	1.49617	-0.61542
C	3.42331	-0.33675	0.07658
H	4.20650	-1.13055	0.02981
H	3.42053	0.03340	1.12529
O	2.16578	-0.90246	-0.25155
C	1.77903	-1.93302	0.63187
H	2.57482	-2.70198	0.70789
H	1.60652	-1.52301	1.64968
C	0.51992	-2.59028	0.12086
H	0.71028	-3.07244	-0.85959
H	0.22633	-3.38008	0.84135
O	-0.50463	-1.62580	-0.00456
C	-1.78949	-2.20171	-0.12408
H	-1.76844	-3.04466	-0.84372
H	-2.11598	-2.60186	0.85786

C	-2.77671	-1.16351	-0.62230
H	-3.77463	-1.64157	-0.72716
H	-2.48111	-0.84064	-1.64647
C	-3.99754	0.80231	-0.03032
H	-4.11988	1.02337	-1.11222
H	-3.94762	1.75107	0.52908
H	-4.90796	0.25011	0.28765
Cs	0.06615	1.36021	0.13007

ACE1 Cs HSE06 def2sv

26

N	-2.63794	-0.03841	0.57202
C	3.65166	0.81423	0.73890
H	3.73806	1.23263	-0.27055
H	4.16596	1.31677	1.56340
C	3.15003	-0.56655	0.91809
H	3.97766	-1.30037	0.76038
H	2.79530	-0.72695	1.95860
O	2.11480	-0.83562	-0.00114
C	1.69194	-2.17528	0.00750
H	2.52876	-2.84899	-0.27011
H	1.36067	-2.46915	1.02461
C	0.57686	-2.35764	-0.99989
H	0.92716	-2.01763	-1.98884
H	0.33767	-3.43699	-1.07477
O	-0.58652	-1.61612	-0.71810
C	-1.39880	-2.14061	0.30572
H	-1.55940	-3.22525	0.14482
H	-0.92338	-2.01539	1.29916
C	-2.73861	-1.43603	0.29576
H	-3.40941	-1.93502	1.02874

H	-3.22211	-1.58010	-0.69621
C	-3.90446	0.61021	0.67490
H	-3.78280	1.69492	0.82854
H	-4.49068	0.19758	1.52440
H	-4.52083	0.44386	-0.23455
Cs	0.03900	1.45569	-0.28170

ACE1 Cs M062X def2sv

26

N	-2.49320	0.79477	0.04036
C	3.12512	0.76573	-0.20736
H	3.06528	0.82069	-1.30064
H	3.63285	1.57528	0.32718
C	2.90630	-0.52427	0.46281
H	3.89303	-1.05789	0.47775
H	2.64128	-0.38165	1.53247
O	1.93424	-1.26398	-0.21700
C	1.35471	-2.33139	0.50690
H	2.01265	-3.21851	0.50305
H	1.18660	-2.02208	1.55617
C	0.02419	-2.66420	-0.13319
H	0.17076	-3.04397	-1.16250
H	-0.46917	-3.45551	0.46193
O	-0.75032	-1.49137	-0.15483
C	-2.11881	-1.67331	-0.43888
H	-2.25043	-2.38279	-1.27726
H	-2.62975	-2.09587	0.44758
C	-2.71665	-0.31794	-0.83788
H	-3.81581	-0.48919	-0.94507
H	-2.35289	-0.05344	-1.84800
C	-2.72737	0.48673	1.41814

H	-2.63681	1.39504	2.03722
H	-2.04074	-0.28603	1.83964
H	-3.75378	0.07497	1.56918
Cs	0.22260	1.24822	-0.11182

ACE1 Cs M06 def2sv

26

N	2.71361	0.43806	0.07383
C	-3.47744	0.78587	-0.23672
H	-3.45780	0.95630	0.84794
H	-4.03600	1.48244	-0.87130
C	-2.96038	-0.44998	-0.79640
H	-3.83818	-1.12213	-1.02440
H	-2.51045	-0.27987	-1.80952
O	-2.06451	-1.06583	0.07182
C	-1.42052	-2.21031	-0.44392
H	-2.14951	-3.03525	-0.58834
H	-0.97402	-1.98146	-1.43819
C	-0.35631	-2.62904	0.54061
H	-0.82201	-2.74858	1.53685
H	0.05386	-3.61437	0.23872
O	0.67432	-1.68052	0.67363
C	1.77620	-1.86527	-0.18487
H	2.11198	-2.92367	-0.14108
H	1.50520	-1.64644	-1.24339
C	2.92528	-0.95943	0.26509
H	3.82892	-1.32146	-0.28636
H	3.08171	-1.17580	1.34295
C	3.00876	0.84516	-1.25236
H	2.83083	1.92947	-1.39666
H	2.42748	0.30258	-2.04761

H	4.07766	0.64360	-1.52310
Cs	-0.12685	1.31522	0.26499

ACE1 Cs MP2 def2sv

26

N	-4.48358	0.00568	0.78119
C	3.72651	-1.55546	0.47645
H	4.47741	-1.64049	1.26681
H	4.04778	-1.21382	-0.51275
C	2.43393	-2.27613	0.60840
H	2.54773	-3.34225	0.30327
H	2.09222	-2.28325	1.66418
O	1.46162	-1.65478	-0.21868
C	0.22761	-2.34440	-0.22174
H	0.34915	-3.35768	-0.65778
H	-0.14831	-2.46524	0.81471
C	-0.76321	-1.55691	-1.04985
H	-0.34081	-1.37612	-2.05265
H	-1.69209	-2.14453	-1.17287
O	-1.03573	-0.28318	-0.48222
C	-2.09083	-0.27231	0.46935
H	-2.12325	-1.21876	1.04042
H	-1.87732	0.54095	1.18338
C	-3.43581	-0.02050	-0.19729
H	-3.64248	-0.80802	-0.95481
H	-3.39286	0.93945	-0.75652
C	-5.76601	0.29309	0.20228
H	-5.76301	1.29519	-0.27474
H	-6.55316	0.27143	0.97283
H	-6.01978	-0.44076	-0.59048
Cs	1.45498	1.43825	-0.03381

ACE1 Cs PBE0 def2sv

26

N	-0.85231	-1.57546	0.08024
C	-1.40920	-1.40607	-1.25173
H	-0.61943	-1.71114	-1.96171
H	-2.26720	-2.09583	-1.43560
C	-1.88515	-0.01434	-1.64464
H	-2.06667	-0.03045	-2.73684
H	-2.85764	0.21213	-1.17615
O	-0.94772	0.99346	-1.33224
C	-1.44806	2.15330	-0.71110
H	-1.09838	3.04602	-1.26063
H	-2.55147	2.16610	-0.76776
C	-0.96482	2.25448	0.74590
H	0.04081	2.70611	0.78383
H	-1.64063	2.93187	1.30702
O	-0.83364	1.00633	1.37499
C	-2.00221	0.22390	1.45213
H	-2.45505	0.31716	2.45879
H	-2.76242	0.58682	0.74427
C	-1.69187	-1.25062	1.22214
H	-2.66344	-1.79769	1.20123
H	-1.15312	-1.61625	2.11302
C	-0.31699	-2.91731	0.21700
H	0.22397	-3.01753	1.17379
H	0.39240	-3.13044	-0.60176
H	-1.10601	-3.70350	0.18807
Cs	1.83838	0.10739	-0.02021

ACE1 Cs PBE def2sv

26

N	-2.51783	-0.39730	0.68610
C	2.88839	0.19830	2.03296
H	3.50412	0.78716	2.73410
H	2.04610	-0.39026	2.43648
C	3.33582	0.03175	0.62208
H	3.70636	1.00217	0.21424
H	4.20894	-0.67566	0.54616
O	2.30329	-0.40436	-0.26550
C	2.06662	-1.80650	-0.23290
H	2.98339	-2.36095	-0.55710
H	1.82649	-2.14977	0.80301
C	0.93253	-2.15375	-1.18995
H	1.16851	-1.74344	-2.19626
H	0.86834	-3.26613	-1.27516
O	-0.33562	-1.61061	-0.83483
C	-1.00507	-2.29642	0.21785
H	-1.02508	-3.39397	0.00703
H	-0.48472	-2.14889	1.19641
C	-2.43611	-1.78293	0.32321
H	-2.99245	-2.42593	1.05627
H	-2.95963	-1.94597	-0.65784
C	-3.85896	0.05955	0.89743
H	-3.88087	1.14676	1.12598
H	-4.34554	-0.49134	1.74576
H	-4.51046	-0.13513	0.00472
Cs	-0.17762	1.51986	-0.34924

ACE1 Cs SCS-MP2 def2sv

26

N	2.42086	-0.70906	-0.69229
C	-2.99506	-0.33415	-2.10705

H	-3.81841	-0.41557	-2.82443
H	-1.96647	-0.38014	-2.48256
C	-3.24868	0.18735	-0.72948
H	-3.28384	1.29634	-0.72655
H	-4.22983	-0.16449	-0.34911
O	-2.21605	-0.15672	0.19118
C	-2.21939	-1.52932	0.54790
H	-3.17130	-1.79530	1.05415
H	-2.13274	-2.16650	-0.35345
C	-1.07312	-1.78663	1.50994
H	-1.20005	-1.15957	2.40891
H	-1.09818	-2.85118	1.82236
O	0.20389	-1.45526	0.98472
C	0.63122	-2.27771	-0.09105
H	0.44075	-3.34479	0.14565
H	0.08518	-2.02871	-1.02289
C	2.11839	-2.06350	-0.30251
H	2.47988	-2.77336	-1.07889
H	2.66421	-2.31409	0.63310
C	3.82992	-0.50318	-0.91985
H	4.40931	-0.72158	0.00189
H	4.02323	0.53906	-1.22349
H	4.20466	-1.18472	-1.71206
Cs	0.35428	1.58485	0.24907

ACE1 K B2PLYP def2sv

26

N	1.71580	1.00315	-0.12101
C	0.70514	1.09509	0.92112
H	0.72973	2.08157	1.42882
H	-0.30187	1.02725	0.47915

C	0.87469	0.01310	1.99384
H	1.82550	0.17496	2.52651
H	0.05469	0.10521	2.73667
O	0.95733	-1.30413	1.48904
C	-0.18298	-1.79802	0.82138
H	-0.63723	-2.62286	1.40532
H	-0.95722	-1.01902	0.72852
C	0.19940	-2.35247	-0.54979
H	0.85492	-3.22631	-0.40803
H	-0.72383	-2.70337	-1.05696
O	0.93812	-1.46994	-1.36411
C	0.30913	-0.26664	-1.75249
H	0.08839	-0.29341	-2.83728
H	-0.66115	-0.14058	-1.24296
C	1.22583	0.92600	-1.48377
H	0.68471	1.84811	-1.80973
H	2.10593	0.83046	-2.14251
C	2.80408	1.94226	0.04509
H	3.21192	1.87068	1.06717
H	2.50377	3.00313	-0.12592
H	3.62091	1.70481	-0.65729
K	-3.95712	0.79863	-0.01137

ACE1 K B3LYP def2sv

26

N	1.36197	-1.28741	0.06119
C	1.27460	-1.28035	-1.38553
H	2.30697	-1.37409	-1.77290
H	0.71288	-2.15726	-1.80064
C	0.63705	-0.02933	-2.00886
H	0.82843	-0.07396	-3.09794

H	-0.46290	-0.04650	-1.88999
O	1.16121	1.18674	-1.51364
C	0.33416	1.98166	-0.68853
H	0.13476	2.95273	-1.18768
H	-0.64930	1.50069	-0.53954
C	0.98588	2.29934	0.66118
H	1.86193	2.94833	0.48813
H	0.24903	2.88429	1.25592
O	1.48571	1.21075	1.39988
C	0.59063	0.23043	1.87675
H	1.11600	-0.24741	2.72247
H	-0.33574	0.69116	2.28266
C	0.21619	-0.85418	0.83851
H	-0.56542	-0.47218	0.16780
H	-0.25995	-1.69735	1.38701
C	2.06773	-2.42693	0.59889
H	3.04984	-2.53832	0.10347
H	1.51684	-3.39255	0.47285
H	2.25553	-2.29041	1.67880
K	-4.16345	-0.33574	0.04510

ACE1 K B97-1 def2sv

26

N	1.72801	1.00535	0.34660
C	1.53792	0.03932	1.41364
H	2.08009	0.34360	2.33845
H	0.47074	0.01335	1.69713
C	2.01287	-1.39132	1.06651
H	3.11850	-1.41445	1.06462
H	1.66760	-2.08243	1.86935
O	1.64661	-1.87405	-0.20424

C	0.29892	-2.18421	-0.45907
H	0.32011	-2.86391	-1.33117
H	-0.16296	-2.73821	0.38862
C	-0.58893	-0.97513	-0.80885
H	-1.54866	-1.35888	-1.22194
H	-0.84546	-0.40514	0.09960
O	0.06995	-0.17146	-1.75893
C	0.09047	1.22429	-1.55610
H	0.79292	1.60477	-2.32018
H	-0.90623	1.67901	-1.76504
C	0.55663	1.69970	-0.15758
H	-0.27716	1.61537	0.56801
H	0.75293	2.78824	-0.24307
C	2.96605	1.75255	0.38576
H	3.82360	1.06835	0.53030
H	3.00262	2.51523	1.20418
H	3.13090	2.27749	-0.57347
K	-4.34166	0.27858	0.61410

ACE1 K CAM-B3LYP def2sv

26

N	1.70939	1.01309	-0.12486
C	0.70540	1.09862	0.91722
H	0.72742	2.08497	1.42420
H	-0.30160	1.02876	0.47588
C	0.87501	0.01978	1.98989
H	1.82558	0.18274	2.52318
H	0.05656	0.11526	2.73375
O	0.95303	-1.29272	1.48981
C	-0.17744	-1.79310	0.82406
H	-0.62929	-2.61857	1.40862

H	-0.95608	-1.01815	0.72809
C	0.20713	-2.34855	-0.54348
H	0.86486	-3.22058	-0.39891
H	-0.71426	-2.70564	-1.04940
O	0.93724	-1.46760	-1.35618
C	0.31562	-0.27031	-1.75139
H	0.09814	-0.30099	-2.83659
H	-0.65700	-0.14063	-1.24622
C	1.22990	0.92198	-1.48598
H	0.69258	1.84096	-1.82425
H	2.11380	0.82089	-2.13860
C	2.81186	1.92673	0.04603
H	3.21978	1.84567	1.06769
H	2.53190	2.99390	-0.12049
H	3.62636	1.67942	-0.65601
K	-3.96797	0.79326	-0.01390

ACE1 K DSDPBEP86 def2sv

26

N	-1.69879	0.99460	0.12077
C	-0.69666	1.08024	-0.92793
H	-0.72138	2.06690	-1.43978
H	0.31565	1.00978	-0.49392
C	-0.88628	-0.00717	-1.98767
H	-1.84268	0.16013	-2.51272
H	-0.07144	0.06559	-2.74103
O	-0.97738	-1.31360	-1.46247
C	0.17328	-1.79973	-0.81120
H	0.62372	-2.62745	-1.39757
H	0.94680	-1.01581	-0.73348
C	-0.19133	-2.34275	0.56712

H	-0.84514	-3.22247	0.44054
H	0.73926	-2.68287	1.07219
O	-0.92546	-1.45215	1.37353
C	-0.27950	-0.25443	1.74271
H	-0.03893	-0.27452	2.82541
H	0.68351	-0.14208	1.21372
C	-1.18909	0.94090	1.47536
H	-0.63509	1.86653	1.77435
H	-2.06041	0.86178	2.15125
C	-2.77405	1.94753	-0.04222
H	-3.19133	1.87221	-1.06230
H	-2.45453	3.00597	0.11813
H	-3.58868	1.72539	0.67057
K	3.91140	0.79512	-0.00612

ACE1 K HSE06 def2sv

26

N	1.13386	1.50205	-0.18431
C	1.06169	1.32067	1.24719
H	2.01508	1.69118	1.66420
H	0.25628	1.94098	1.71627
C	0.86657	-0.10380	1.74836
H	0.98412	-0.07953	2.85031
H	-0.16516	-0.45146	1.56133
O	1.80125	-0.99481	1.19489
C	1.31262	-2.16076	0.58932
H	1.89422	-3.02924	0.95360
H	0.26562	-2.34627	0.89740
C	1.45068	-2.13724	-0.93335
H	2.51765	-2.23412	-1.19665
H	0.93196	-3.03808	-1.33016

O	1.02746	-0.96542	-1.57397
C	-0.19129	-0.39036	-1.19370
H	-0.94734	-0.52756	-1.99424
H	-0.60880	-0.88115	-0.30161
C	-0.01700	1.11370	-0.96984
H	-0.97729	1.51837	-0.56339
H	0.11097	1.57454	-1.96382
C	1.59176	2.83189	-0.51192
H	2.54255	3.04327	0.00816
H	0.86995	3.63549	-0.22690
H	1.77597	2.91638	-1.59681
K	-4.13067	-0.07421	0.20993

ACE1 K M062X def2sv

26

N	0.77362	1.70698	-0.19548
C	0.26859	1.55417	1.14679
H	0.90326	2.17696	1.80070
H	-0.75664	1.97033	1.21135
C	0.21298	0.13033	1.72486
H	0.07678	0.19650	2.82196
H	-0.66954	-0.40451	1.33083
O	1.38687	-0.58727	1.42588
C	1.24931	-1.87339	0.87953
H	1.81148	-2.60148	1.49218
H	0.19184	-2.19215	0.90852
C	1.80833	-1.93269	-0.54675
H	2.90899	-1.93175	-0.50363
H	1.49088	-2.89194	-1.00526
O	1.47502	-0.83365	-1.35272
C	0.12877	-0.43282	-1.40951

H	-0.32329	-0.74170	-2.37322
H	-0.46692	-0.91305	-0.61757
C	0.05548	1.09489	-1.28638
H	-1.01234	1.38250	-1.22452
H	0.44754	1.52902	-2.22392
C	2.21382	1.80849	-0.31745
H	2.76364	0.89932	-0.01817
H	2.57092	2.65121	0.30381
H	2.46950	2.03430	-1.36655
K	-4.01791	-0.20205	-0.04737

ACE1 K M06 def2sv

26

N	1.68856	1.02130	-0.12795
C	0.69217	1.09108	0.91962
H	0.70229	2.08222	1.42754
H	-0.32226	1.01399	0.48361
C	0.88811	0.01851	1.98404
H	1.84831	0.19591	2.50523
H	0.08008	0.10665	2.74629
O	0.97419	-1.28980	1.48435
C	-0.15142	-1.79188	0.81918
H	-0.61112	-2.61776	1.40404
H	-0.93781	-1.01716	0.72177
C	0.23434	-2.34474	-0.54222
H	0.90178	-3.21430	-0.39700
H	-0.68712	-2.71761	-1.04571
O	0.95067	-1.46023	-1.35775
C	0.31041	-0.27492	-1.74673
H	0.08249	-0.30372	-2.83382
H	-0.66998	-0.16326	-1.23945

C	1.20199	0.92704	-1.48320
H	0.64790	1.84350	-1.81669
H	2.08680	0.84577	-2.14594
C	2.76988	1.95359	0.03907
H	3.18650	1.87944	1.06179
H	2.46718	3.01914	-0.12332
H	3.59040	1.72641	-0.66770
K	-3.96090	0.77367	-0.00718

ACE1 K MP2 def2sv

26

N	1.33554	1.46843	-0.25800
C	0.46603	1.35644	0.88952
H	0.40355	2.32197	1.44401
H	-0.55965	1.15694	0.53410
C	0.86805	0.31932	1.95135
H	1.76957	0.67259	2.47735
H	0.05293	0.25770	2.70450
O	1.21330	-0.95844	1.46385
C	0.16846	-1.70581	0.88447
H	-0.10310	-2.56118	1.53713
H	-0.74317	-1.09322	0.76931
C	0.64072	-2.24408	-0.45864
H	1.47071	-2.94967	-0.28762
H	-0.18763	-2.80106	-0.94634
O	1.16399	-1.24122	-1.30126
C	0.22974	-0.27409	-1.73024
H	-0.02528	-0.43455	-2.79927
H	-0.71605	-0.35983	-1.16664
C	0.81051	1.12787	-1.55946
H	0.01555	1.84602	-1.85692

H	1.63105	1.25515	-2.28719
C	2.74949	1.29894	-0.02253
H	3.04324	0.26740	0.25411
H	3.08143	1.98058	0.78486
H	3.30478	1.58421	-0.93287
K	-4.02121	0.36336	0.02894

ACE1 K PBE0 def2sv

26

N	1.55821	-1.27357	0.08274
C	1.15027	-1.16912	-1.29914
H	2.06391	-1.28274	-1.90930
H	0.46741	-2.00117	-1.60741
C	0.47501	0.12842	-1.72288
H	0.37567	0.09535	-2.82657
H	-0.55577	0.18520	-1.32871
O	1.21364	1.26391	-1.35237
C	0.54908	2.26954	-0.63803
H	0.77900	3.25108	-1.09526
H	-0.54746	2.14484	-0.72458
C	0.98973	2.34105	0.82486
H	2.01260	2.75193	0.87010
H	0.32210	3.06557	1.34217
O	1.06354	1.11918	1.50372
C	-0.00132	0.21841	1.38149
H	-0.58161	0.17745	2.32638
H	-0.71351	0.53926	0.60686
C	0.53151	-1.18748	1.09800
H	-0.34111	-1.85551	0.89371
H	0.98458	-1.55488	2.03427
C	2.42436	-2.41160	0.27972

H	3.26956	-2.37054	-0.42968
H	1.91297	-3.39391	0.13273
H	2.84247	-2.40124	1.30119
K	-4.11198	-0.45451	-0.04842

ACE1 K PBE def2sv

26

N	2.14945	-0.63673	-0.78771
C	1.56811	0.49172	-1.48271
H	2.16197	0.76243	-2.39971
H	0.56790	0.18925	-1.86305
C	1.45836	1.81719	-0.68469
H	2.47523	2.24438	-0.54751
H	0.87904	2.55034	-1.30454
O	0.93856	1.71935	0.63003
C	-0.41573	1.32609	0.75275
H	-1.03872	2.18138	1.11918
H	-0.83782	1.02905	-0.23496
C	-0.53752	0.18084	1.76482
H	-0.21373	0.54627	2.76410
H	-1.61821	-0.11089	1.84781
O	0.29135	-0.93324	1.49194
C	0.03905	-1.61745	0.27466
H	-0.40235	-2.62492	0.48621
H	-0.71151	-1.07258	-0.34442
C	1.34116	-1.81019	-0.52740
H	1.04947	-2.31727	-1.48462
H	1.98463	-2.53443	0.01895
C	3.30596	-0.38004	0.03999
H	3.08443	0.16336	0.99507
H	4.05571	0.22622	-0.52430

H	3.80118	-1.34117	0.30354
K	-4.24626	-0.09327	-0.58516

ACE1 K SCS-MP2 def2sv

26

N	-1.50561	1.17388	0.12511
C	-0.59025	1.03477	-1.00098
H	-0.50005	1.98341	-1.57464
H	0.43093	0.82036	-0.64157
C	-1.04285	-0.06089	-1.97159
H	-2.00094	0.23773	-2.43284
H	-0.29312	-0.16610	-2.78658
O	-1.30438	-1.30910	-1.35717
C	-0.19376	-1.95157	-0.76231
H	0.05836	-2.87324	-1.32782
H	0.70639	-1.31145	-0.79220
C	-0.53380	-2.35713	0.67151
H	-1.33246	-3.11867	0.64354
H	0.36393	-2.82294	1.13429
O	-1.04937	-1.31341	1.47276
C	-0.17881	-0.22487	1.71626
H	0.14103	-0.23212	2.77925
H	0.74299	-0.31068	1.11490
C	-0.89177	1.09821	1.43992
H	-0.16202	1.92573	1.63074
H	-1.70024	1.20729	2.18653
C	-2.39320	2.31488	-0.00936
H	-2.90011	2.27933	-0.99038
H	-1.86574	3.29597	0.06950
H	-3.17264	2.28045	0.77329
K	3.98945	0.54987	-0.10985

ACE1 Li B2PLYP def2sv

26

N	-1.40041	-0.10234	-0.10925
C	-1.02156	0.94307	0.86094
H	-1.91333	1.44414	1.27972
H	-0.50742	0.48674	1.71983
C	-0.11702	1.99298	0.21298
H	-0.67380	2.56657	-0.54336
H	0.26535	2.70180	0.96835
O	0.96339	1.38596	-0.50020
C	1.99684	0.80375	0.29432
H	2.90866	1.42335	0.23969
H	1.68780	0.76384	1.35051
C	2.28843	-0.59554	-0.24751
H	2.81506	-0.52763	-1.21116
H	2.92642	-1.15968	0.45487
O	1.08003	-1.29797	-0.53076
C	0.32745	-1.75200	0.59666
H	0.48047	-2.83666	0.73018
H	0.68583	-1.25850	1.51352
C	-1.15469	-1.47276	0.35991
H	-1.71377	-1.71083	1.28939
H	-1.52397	-2.15429	-0.42374
C	-2.77179	0.06733	-0.59269
H	-2.90130	1.08687	-0.98806
H	-3.52229	-0.09596	0.20791
H	-2.97004	-0.64435	-1.40970
Li	0.04196	0.00204	-1.69108

ACE1 Li B2PLYP def2tzv

26

N	-1.40634	-0.10274	-0.11817
C	-1.02879	0.94975	0.87375
H	-1.91809	1.43173	1.29233
H	-0.50452	0.49456	1.71313
C	-0.14501	2.02057	0.22468
H	-0.69271	2.57957	-0.53017
H	0.24212	2.71427	0.97227
O	0.97445	1.40759	-0.51885
C	2.03165	0.80325	0.31053
H	2.92270	1.42819	0.26350
H	1.70269	0.75282	1.34765
C	2.33538	-0.58646	-0.25126
H	2.84463	-0.51591	-1.20871
H	2.95609	-1.15326	0.44375
O	1.09306	-1.31669	-0.55043
C	0.31579	-1.77444	0.61724
H	0.47430	-2.84466	0.74348
H	0.67468	-1.27159	1.51450
C	-1.16676	-1.49264	0.36477
H	-1.72347	-1.70837	1.28758
H	-1.53576	-2.16527	-0.40983
C	-2.80133	0.06732	-0.60240
H	-2.93005	1.07846	-0.98473
H	-3.53408	-0.10315	0.19625
H	-2.99243	-0.63478	-1.41235
Li	0.02423	-0.00492	-1.69039

ACE1 Li B3LYP def2sv

26

N	-1.41088	-0.10123	-0.10274
C	-1.02697	0.95038	0.86301

H	-1.91735	1.45948	1.27761
H	-0.51879	0.49455	1.72725
C	-0.11207	1.99795	0.21622
H	-0.66367	2.57879	-0.54068
H	0.27072	2.70439	0.97552
O	0.96864	1.38972	-0.49787
C	2.01087	0.80503	0.28562
H	2.92580	1.42117	0.21496
H	1.71736	0.77337	1.34814
C	2.29373	-0.60227	-0.25131
H	2.81835	-0.54309	-1.21854
H	2.93500	-1.16398	0.45230
O	1.08242	-1.30423	-0.52700
C	0.32363	-1.75889	0.59812
H	0.47229	-2.84596	0.72879
H	0.68228	-1.26993	1.51913
C	-1.16113	-1.47544	0.36120
H	-1.71994	-1.72091	1.29052
H	-1.53249	-2.15465	-0.42531
C	-2.77753	0.07075	-0.60203
H	-2.90218	1.08924	-1.00599
H	-3.54053	-0.08572	0.19073
H	-2.97158	-0.64507	-1.41871
Li	0.03645	0.00266	-1.67419

ACE1 Li B3LYP def2tzv

26

N	-1.41481	-0.10219	-0.11082
C	-1.03196	0.95503	0.87282
H	-1.91853	1.44567	1.28613
H	-0.51685	0.50277	1.71915

C	-0.13997	2.01961	0.22446
H	-0.68549	2.58237	-0.52996
H	0.24239	2.71612	0.97227
O	0.97793	1.40782	-0.51556
C	2.03884	0.80232	0.30371
H	2.93237	1.42360	0.24528
H	1.72426	0.75893	1.34563
C	2.33496	-0.58990	-0.25463
H	2.84001	-0.52136	-1.21515
H	2.96528	-1.15318	0.43507
O	1.09469	-1.32051	-0.54446
C	0.31279	-1.77363	0.61904
H	0.46908	-2.84414	0.74878
H	0.66780	-1.27222	1.51859
C	-1.16830	-1.49300	0.36222
H	-1.72896	-1.72495	1.27876
H	-1.53004	-2.16294	-0.41839
C	-2.80219	0.06927	-0.60716
H	-2.92706	1.07555	-1.00409
H	-3.54572	-0.08767	0.18500
H	-2.99396	-0.64033	-1.41099
Li	0.02107	-0.00650	-1.67432

ACE1 Li B97-1 def2sv

26

N	-1.40931	-0.10268	-0.10266
C	-1.02733	0.94867	0.86431
H	-1.91978	1.45786	1.27903
H	-0.51740	0.49243	1.72994
C	-0.11128	1.99841	0.21510
H	-0.66562	2.57837	-0.54313

H	0.26971	2.70739	0.97567
O	0.96800	1.39249	-0.49653
C	2.00468	0.80728	0.28852
H	2.92175	1.42377	0.22266
H	1.70738	0.77202	1.35203
C	2.29084	-0.60171	-0.25195
H	2.81577	-0.53787	-1.22062
H	2.93648	-1.16195	0.45171
O	1.08479	-1.30660	-0.52779
C	0.33022	-1.75734	0.59786
H	0.47913	-2.84575	0.73214
H	0.68918	-1.26513	1.51962
C	-1.15864	-1.47630	0.36335
H	-1.71620	-1.72086	1.29564
H	-1.53047	-2.15800	-0.42334
C	-2.77783	0.06742	-0.59923
H	-2.90340	1.08752	-1.00330
H	-3.53998	-0.09055	0.19626
H	-2.97159	-0.64991	-1.41679
Li	0.02796	0.00794	-1.70069

ACE1 Li B97-1 def2tzv

26

N	-1.41153	-0.10240	-0.11423
C	-1.02979	0.95305	0.87256
H	-1.91851	1.44419	1.28743
H	-0.51155	0.49910	1.71976
C	-0.13730	2.02074	0.22095
H	-0.68805	2.58125	-0.53530
H	0.24208	2.72121	0.97059
O	0.97982	1.41256	-0.51669

C	2.03075	0.80394	0.30806
H	2.92912	1.42363	0.25861
H	1.70843	0.75638	1.35051
C	2.33070	-0.59096	-0.25322
H	2.83951	-0.51743	-1.21435
H	2.96338	-1.15328	0.43928
O	1.09553	-1.32329	-0.54642
C	0.31797	-1.77303	0.61750
H	0.47344	-2.84599	0.75026
H	0.67375	-1.26874	1.51878
C	-1.16772	-1.49288	0.36274
H	-1.72792	-1.72202	1.28322
H	-1.53127	-2.16587	-0.41818
C	-2.80322	0.06847	-0.60104
H	-2.93064	1.07765	-0.99655
H	-3.54225	-0.09029	0.19821
H	-2.99952	-0.64233	-1.40598
Li	0.01652	-0.01025	-1.68905

ACE1 Li CAM-B3LYP def2sv

26

N	-1.39974	-0.10268	-0.10289
C	-1.01948	0.94149	0.86067
H	-1.90986	1.44308	1.28002
H	-0.50523	0.48575	1.71963
C	-0.11502	1.98806	0.21338
H	-0.67122	2.56533	-0.54075
H	0.27023	2.69571	0.96793
O	0.95744	1.37788	-0.49628
C	1.99570	0.80358	0.28551
H	2.90653	1.42320	0.22010

H 1.69733 0.76831 1.34529

C 2.28256 -0.59623 -0.25097

H 2.81002 -0.53384 -1.21467

H 2.91941 -1.16026 0.45215

O 1.07483 -1.28941 -0.52640

C 0.32626 -1.75018 0.59254

H 0.47993 -2.83514 0.72094

H 0.68442 -1.26130 1.51233

C -1.15359 -1.47083 0.35900

H -1.71208 -1.71203 1.28715

H -1.52449 -2.15002 -0.42562

C -2.76067 0.07026 -0.59913

H -2.88615 1.08892 -0.99848

H -3.51946 -0.08898 0.19441

H -2.95595 -0.64172 -1.41679

Li 0.04067 0.00238 -1.65602

ACE1 Li CAM-B3LYP def2tzv

26

N -1.40291 -0.10473 -0.10785

C -1.02468 0.94454 0.87049

H -1.91143 1.42798 1.28793

H -0.50406 0.49399 1.71281

C -0.14379 2.00696 0.22110

H -0.69443 2.56549 -0.53089

H 0.23991 2.70608 0.96366

O 0.96296 1.39271 -0.51079

C 2.02125 0.80194 0.30113

H 2.91015 1.42718 0.24488

H 1.70662 0.75503 1.34192

C 2.32082 -0.58246 -0.25637

H	2.82727	-0.51059	-1.21436
H	2.94935	-1.14699	0.43160
O	1.08619	-1.30206	-0.54140
C	0.31709	-1.76313	0.61202
H	0.47863	-2.83172	0.73888
H	0.67086	-1.26351	1.51191
C	-1.15861	-1.48760	0.35985
H	-1.71889	-1.71745	1.27487
H	-1.51967	-2.15733	-0.41946
C	-2.77977	0.06797	-0.60669
H	-2.90492	1.07517	-0.99780
H	-3.52303	-0.09358	0.18247
H	-2.96999	-0.63609	-1.41393
Li	0.02563	-0.00500	-1.65037

ACE1 Li DSDPBEP86 def2sv

26

N	-1.38861	-0.10694	-0.11015
C	-1.01710	0.93283	0.86366
H	-1.91338	1.42372	1.29090
H	-0.49256	0.47620	1.71877
C	-0.12709	1.98854	0.21049
H	-0.69503	2.55334	-0.54722
H	0.25372	2.70601	0.96165
O	0.95201	1.38607	-0.50227
C	1.97812	0.80822	0.29948
H	2.88865	1.43434	0.25876
H	1.65755	0.75965	1.35404
C	2.28173	-0.58430	-0.24787
H	2.80899	-0.50577	-1.21270
H	2.92495	-1.14867	0.45286

O	1.08049	-1.29230	-0.53381
C	0.33682	-1.74649	0.59545
H	0.49642	-2.83186	0.73227
H	0.69577	-1.24738	1.51160
C	-1.14519	-1.47388	0.36021
H	-1.70652	-1.71039	1.29116
H	-1.51225	-2.15842	-0.42502
C	-2.75881	0.06259	-0.59041
H	-2.88876	1.08627	-0.98066
H	-3.50830	-0.10600	0.21255
H	-2.95644	-0.64632	-1.41258
Li	0.03887	-0.00382	-1.69760

ACE1 Li DSDPBEP86 def2tzv

26

N	-1.39535	-0.10722	-0.12036
C	-1.02538	0.93998	0.87528
H	-1.91990	1.41411	1.30101
H	-0.49096	0.48391	1.71223
C	-0.15284	2.01756	0.22306
H	-0.70991	2.57220	-0.53307
H	0.23410	2.71671	0.97071
O	0.96480	1.40878	-0.52002
C	2.01334	0.80696	0.31650
H	2.90508	1.43752	0.28523
H	1.67089	0.74570	1.35236
C	2.32883	-0.57769	-0.25211
H	2.84036	-0.49748	-1.21132
H	2.95311	-1.14565	0.44403
O	1.09289	-1.31194	-0.55505
C	0.32568	-1.77015	0.61462

H	0.48894	-2.84268	0.74433
H	0.68692	-1.26189	1.51232
C	-1.15919	-1.49296	0.36764
H	-1.71387	-1.70119	1.29737
H	-1.53231	-2.17126	-0.40485
C	-2.78976	0.06221	-0.60107
H	-2.92007	1.07940	-0.97646
H	-3.52197	-0.11616	0.20088
H	-2.97984	-0.63677	-1.41825
Li	0.02375	-0.00534	-1.69897

ACE1 Li HSE06 def2sv

26

N	-1.39780	-0.10446	-0.09986
C	-1.01539	0.93634	0.86111
H	-1.90418	1.43554	1.29114
H	-0.49411	0.47859	1.71734
C	-0.11668	1.98424	0.21228
H	-0.67675	2.55834	-0.54358
H	0.26125	2.69842	0.96677
O	0.95736	1.38314	-0.49546
C	1.98533	0.80254	0.28965
H	2.89934	1.42136	0.23767
H	1.67987	0.76116	1.34918
C	2.27598	-0.59257	-0.25135
H	2.80078	-0.52356	-1.21769
H	2.92374	-1.15313	0.44703
O	1.07705	-1.29416	-0.52648
C	0.32923	-1.74365	0.59362
H	0.48353	-2.82846	0.73423
H	0.68583	-1.24698	1.51229

C	-1.14898	-1.46921	0.35849
H	-1.70917	-1.71910	1.28559
H	-1.51681	-2.14896	-0.42951
C	-2.75491	0.06903	-0.59498
H	-2.87927	1.08806	-0.99679
H	-3.51783	-0.08816	0.19715
H	-2.95090	-0.64401	-1.41313
Li	0.03220	0.00330	-1.69203

ACE1 Li HSE06 def2tzv

26

N	-1.40083	-0.10514	-0.11007
C	-1.01799	0.93995	0.86786
H	-1.90313	1.42284	1.29695
H	-0.48962	0.48602	1.70762
C	-0.14267	2.00463	0.21794
H	-0.69853	2.56091	-0.53604
H	0.23245	2.70981	0.96349
O	0.96832	1.40193	-0.51355
C	2.00928	0.79814	0.30913
H	2.90494	1.42056	0.27547
H	1.67890	0.74198	1.34770
C	2.31458	-0.58086	-0.25402
H	2.82289	-0.50041	-1.21357
H	2.95190	-1.14245	0.43280
O	1.08892	-1.31018	-0.54474
C	0.31867	-1.75705	0.61145
H	0.47873	-2.82702	0.75273
H	0.67100	-1.24859	1.51082
C	-1.15593	-1.48514	0.35750
H	-1.71698	-1.72044	1.27445

H	-1.51628	-2.15737	-0.42402
C	-2.77920	0.06771	-0.59647
H	-2.90745	1.07650	-0.98900
H	-3.52034	-0.09239	0.19881
H	-2.97702	-0.63849	-1.40330
Li	0.01866	-0.00463	-1.67950

ACE1 Li M062X def2sv

26

N	-1.38724	-0.10595	-0.10666
C	-1.00861	0.93136	0.86470
H	-1.90049	1.41805	1.29930
H	-0.47619	0.47115	1.71160
C	-0.12184	1.98691	0.20807
H	-0.69144	2.54982	-0.54744
H	0.26125	2.70307	0.95567
O	0.95143	1.37994	-0.49937
C	1.97514	0.80428	0.29748
H	2.88299	1.42959	0.26029
H	1.65251	0.75137	1.35070
C	2.27630	-0.58765	-0.25163
H	2.80221	-0.50884	-1.21505
H	2.91496	-1.15566	0.44636
O	1.07238	-1.28395	-0.53117
C	0.33424	-1.74539	0.59195
H	0.49654	-2.82818	0.72387
H	0.69219	-1.24707	1.50843
C	-1.14791	-1.47361	0.35905
H	-1.70911	-1.70896	1.28700
H	-1.51543	-2.15433	-0.42680
C	-2.75255	0.07034	-0.59125

H -2.87703 1.09278 -0.98102

H -3.50154 -0.09485 0.20964

H -2.95056 -0.63755 -1.41134

Li 0.03691 -0.00800 -1.68351

ACE1 Li M062X def2tzv

26

N -1.39041 -0.10993 -0.11200

C -1.01676 0.93364 0.87573

H -1.90617 1.40199 1.30604

H -0.47801 0.48117 1.70720

C -0.15401 2.00580 0.21483

H -0.71961 2.54744 -0.53990

H 0.22639 2.71596 0.94927

O 0.95347 1.39348 -0.51241

C 2.00008 0.80598 0.31258

H 2.88494 1.43900 0.28193

H 1.66428 0.74008 1.34723

C 2.31610 -0.57140 -0.25863

H 2.81972 -0.48044 -1.21748

H 2.94891 -1.13874 0.42369

O 1.08610 -1.29428 -0.54567

C 0.32905 -1.75891 0.61053

H 0.50140 -2.82579 0.74044

H 0.67967 -1.25072 1.50875

C -1.15105 -1.49372 0.35852

H -1.71381 -1.71904 1.27320

H -1.50670 -2.16496 -0.42337

C -2.77265 0.06547 -0.60023

H -2.89829 1.07855 -0.97765

H -3.50662 -0.10679 0.19530

H	-2.96441	-0.63134	-1.41390
Li	0.02672	-0.01051	-1.66405

ACE1 Li M06 def2sv

26

N	-1.40292	-0.10622	-0.09422
C	-1.01531	0.93512	0.85897
H	-1.90531	1.43539	1.29384
H	-0.49487	0.48080	1.72174
C	-0.12716	1.98043	0.20240
H	-0.69816	2.53906	-0.56180
H	0.23899	2.71278	0.95000
O	0.95773	1.39025	-0.49421
C	1.97399	0.81032	0.30090
H	2.89122	1.43044	0.26778
H	1.65897	0.77028	1.36142
C	2.27715	-0.58054	-0.23263
H	2.81433	-0.50919	-1.19529
H	2.92721	-1.13475	0.47318
O	1.09162	-1.29794	-0.51633
C	0.33083	-1.74217	0.59387
H	0.48260	-2.82905	0.74219
H	0.68228	-1.24788	1.52006
C	-1.14234	-1.47017	0.35033
H	-1.71128	-1.73554	1.27257
H	-1.50295	-2.14807	-0.44789
C	-2.76107	0.06511	-0.57642
H	-2.89393	1.09002	-0.96808
H	-3.52020	-0.10169	0.22222
H	-2.96530	-0.64127	-1.40133
Li	0.06851	-0.03165	-1.83045

ACE1 Li M06 def2tzv

26

N	-1.39956	-0.10796	-0.10351
C	-1.01717	0.93886	0.86930
H	-1.90417	1.42157	1.29514
H	-0.49017	0.48921	1.71281
C	-0.14809	2.00137	0.21047
H	-0.71495	2.54037	-0.54939
H	0.21386	2.72131	0.94942
O	0.96715	1.40954	-0.50783
C	1.99881	0.80608	0.31222
H	2.89826	1.42391	0.28523
H	1.66983	0.74795	1.35228
C	2.30804	-0.57269	-0.24945
H	2.81613	-0.48615	-1.20903
H	2.95389	-1.12549	0.43747
O	1.09733	-1.31380	-0.53624
C	0.32697	-1.75565	0.61206
H	0.48488	-2.82529	0.76057
H	0.67432	-1.24646	1.51404
C	-1.14669	-1.48675	0.35592
H	-1.71156	-1.72817	1.26944
H	-1.50131	-2.15659	-0.43138
C	-2.77757	0.06373	-0.58571
H	-2.90583	1.07562	-0.97186
H	-3.51494	-0.10081	0.21292
H	-2.97687	-0.63975	-1.39458
Li	0.00795	-0.03036	-1.76831

ACE1 Li MP2 def2sv

26

N	-1.38838	-0.10439	-0.11754
C	-1.01645	0.93261	0.85888
H	-1.91261	1.42587	1.28467
H	-0.49552	0.47589	1.71590
C	-0.12446	1.98720	0.20866
H	-0.69183	2.55115	-0.54982
H	0.25397	2.70504	0.96103
O	0.95796	1.38562	-0.50312
C	1.97715	0.80449	0.30781
H	2.88940	1.42908	0.27659
H	1.64837	0.75363	1.35921
C	2.28243	-0.58591	-0.24131
H	2.81324	-0.50383	-1.20365
H	2.92321	-1.15224	0.46030
O	1.08095	-1.29460	-0.53503
C	0.33500	-1.74256	0.59692
H	0.49278	-2.82778	0.73950
H	0.69264	-1.24079	1.51156
C	-1.14551	-1.46969	0.35800
H	-1.70846	-1.70224	1.28941
H	-1.51017	-2.15761	-0.42522
C	-2.76727	0.06084	-0.57846
H	-2.90556	1.08479	-0.96314
H	-3.50363	-0.11329	0.23461
H	-2.97260	-0.64640	-1.39932
Li	0.04963	-0.00017	-1.74220

ACE1 Li MP2 def2tzv

26

N	-1.39959	-0.10140	-0.12707
C	-1.02392	0.94696	0.87546

H	-1.91853	1.42326	1.30309
H	-0.48757	0.48512	1.70946
C	-0.14586	2.02788	0.22657
H	-0.69655	2.58590	-0.53202
H	0.24558	2.71881	0.98080
O	0.98146	1.41909	-0.52427
C	2.02758	0.80271	0.32327
H	2.92423	1.42767	0.29645
H	1.67571	0.74210	1.35674
C	2.33779	-0.58769	-0.24591
H	2.85366	-0.51470	-1.20356
H	2.94869	-1.16123	0.45870
O	1.09139	-1.32232	-0.56003
C	0.31731	-1.78059	0.61786
H	0.47369	-2.85547	0.74264
H	0.68588	-1.27494	1.51504
C	-1.17094	-1.49111	0.37572
H	-1.71813	-1.67866	1.31593
H	-1.55892	-2.17208	-0.38786
C	-2.80853	0.06894	-0.59533
H	-2.93995	1.09010	-0.96027
H	-3.53019	-0.11827	0.21468
H	-3.00029	-0.62539	-1.41628
Li	0.01883	-0.01974	-1.76513

ACE1 Li PBE0 def2sv

26

N	-1.39721	-0.10431	-0.09907
C	-1.01547	0.93715	0.86105
H	-1.90473	1.43729	1.28909
H	-0.49608	0.47991	1.71857

C	-0.11560	1.98413	0.21227
H	-0.67539	2.55926	-0.54309
H	0.26254	2.69778	0.96732
O	0.95725	1.38307	-0.49555
C	1.98493	0.80282	0.28891
H	2.89930	1.42122	0.23577
H	1.68051	0.76184	1.34867
C	2.27484	-0.59262	-0.25162
H	2.79940	-0.52407	-1.21822
H	2.92283	-1.15315	0.44669
O	1.07618	-1.29362	-0.52611
C	0.32955	-1.74430	0.59312
H	0.48278	-2.82972	0.73123
H	0.68739	-1.25044	1.51276
C	-1.14905	-1.46858	0.36022
H	-1.70724	-1.71753	1.28874
H	-1.51852	-2.14861	-0.42677
C	-2.75344	0.06943	-0.59606
H	-2.87647	1.08804	-0.99946
H	-3.51759	-0.08615	0.19520
H	-2.94880	-0.64450	-1.41366
Li	0.02951	-0.00163	-1.69112

ACE1 Li PBE0 def2tzv

26

N	-1.39968	-0.10508	-0.11033
C	-1.01880	0.94017	0.86788
H	-1.90481	1.42324	1.29561
H	-0.49144	0.48615	1.70850
C	-0.14261	2.00442	0.21785
H	-0.69910	2.56191	-0.53528

H	0.23373	2.70898	0.96391
O	0.96602	1.40121	-0.51420
C	2.00739	0.79998	0.30750
H	2.90337	1.42253	0.27133
H	1.67909	0.74580	1.34715
C	2.31340	-0.58025	-0.25329
H	2.82320	-0.50078	-1.21259
H	2.95085	-1.14027	0.43530
O	1.08950	-1.31009	-0.54409
C	0.32018	-1.75656	0.61157
H	0.47968	-2.82706	0.75256
H	0.67293	-1.24848	1.51126
C	-1.15466	-1.48468	0.35803
H	-1.71617	-1.71989	1.27500
H	-1.51495	-2.15726	-0.42373
C	-2.77804	0.06615	-0.59695
H	-2.90727	1.07504	-0.98979
H	-3.51935	-0.09475	0.19837
H	-2.97488	-0.64057	-1.40403
Li	0.01923	-0.00782	-1.67679

ACE1 Li PBE def2sv

26

N	-1.40941	-0.10319	-0.10289
C	-1.02308	0.94700	0.86589
H	-1.91846	1.45312	1.29768
H	-0.50115	0.48442	1.73010
C	-0.11593	2.00162	0.21497
H	-0.67715	2.57978	-0.54995
H	0.26090	2.72094	0.97881
O	0.97297	1.40302	-0.50414

C	2.00324	0.80531	0.29477
H	2.92943	1.42380	0.24810
H	1.69001	0.76313	1.36102
C	2.29381	-0.59874	-0.25164
H	2.81869	-0.52952	-1.22816
H	2.95015	-1.16182	0.45166
O	1.08623	-1.31446	-0.53420
C	0.32687	-1.75886	0.60114
H	0.47703	-2.85324	0.74679
H	0.68851	-1.25652	1.52527
C	-1.15965	-1.47897	0.36195
H	-1.72456	-1.73238	1.29682
H	-1.53180	-2.16210	-0.43333
C	-2.77848	0.07118	-0.59817
H	-2.90196	1.09630	-1.00808
H	-3.54759	-0.08188	0.20210
H	-2.97798	-0.65164	-1.41858
Li	0.02582	-0.00325	-1.70227

ACE1 Li PBE def2tzv

26

N	-1.41280	-0.10256	-0.11715
C	-1.02402	0.95213	0.87135
H	-1.91538	1.44032	1.30411
H	-0.49392	0.49137	1.71685
C	-0.14221	2.02492	0.22047
H	-0.69788	2.58421	-0.54340
H	0.23240	2.73443	0.97481
O	0.98922	1.42656	-0.52629
C	2.02760	0.79804	0.32017
H	2.93493	1.42000	0.29841

H	1.68020	0.74112	1.36139
C	2.33558	-0.58701	-0.24989
H	2.84594	-0.50541	-1.21781
H	2.97827	-1.15299	0.44235
O	1.10024	-1.33575	-0.55660
C	0.31470	-1.77239	0.62188
H	0.47129	-2.85069	0.77044
H	0.67396	-1.25384	1.52230
C	-1.16689	-1.49504	0.36083
H	-1.73481	-1.73114	1.28459
H	-1.53105	-2.17126	-0.42700
C	-2.80987	0.06891	-0.59278
H	-2.94056	1.08249	-0.99372
H	-3.54912	-0.08645	0.21723
H	-3.01570	-0.64868	-1.39829
Li	0.00872	-0.01315	-1.71376

ACE1 Li SCS-MP2 def2sv

26

N	-1.39103	-0.10434	-0.11642
C	-1.02049	0.93731	0.86129
H	-1.91822	1.43335	1.28124
H	-0.50364	0.48203	1.72208
C	-0.12371	1.99155	0.21011
H	-0.68912	2.55787	-0.54891
H	0.25789	2.70797	0.96275
O	0.95850	1.38681	-0.50391
C	1.98427	0.80636	0.30366
H	2.89605	1.43182	0.26249
H	1.66265	0.75951	1.35782
C	2.28657	-0.58807	-0.24472

H	2.81593	-0.50857	-1.20877
H	2.92685	-1.15547	0.45712
O	1.08164	-1.29614	-0.53603
C	0.33545	-1.74823	0.59735
H	0.49295	-2.83475	0.73356
H	0.69532	-1.25071	1.51379
C	-1.14805	-1.47292	0.36042
H	-1.71005	-1.70445	1.29280
H	-1.51406	-2.16088	-0.42287
C	-2.77056	0.06042	-0.58529
H	-2.90743	1.08482	-0.97188
H	-3.51175	-0.11365	0.22400
H	-2.97115	-0.64765	-1.40774
Li	0.04433	0.00175	-1.70997

ACE1 Li SCS-MP2 def2tzv

26

N	-1.40270	-0.10077	-0.12873
C	-1.02888	0.95078	0.87534
H	-1.92462	1.43231	1.29482
H	-0.50028	0.49012	1.71539
C	-0.14177	2.02944	0.22631
H	-0.69092	2.59105	-0.53184
H	0.25177	2.71964	0.98092
O	0.98174	1.41619	-0.52400
C	2.03281	0.80517	0.32015
H	2.92964	1.43036	0.28247
H	1.68924	0.75091	1.35715
C	2.33993	-0.59194	-0.24522
H	2.85900	-0.52188	-1.20223
H	2.94885	-1.16582	0.46164

O	1.09218	-1.32165	-0.55941
C	0.31873	-1.78245	0.61735
H	0.47467	-2.85847	0.73839
H	0.68769	-1.28015	1.51661
C	-1.17314	-1.49183	0.37591
H	-1.72053	-1.68003	1.31604
H	-1.56008	-2.17442	-0.38759
C	-2.81353	0.06750	-0.59652
H	-2.94702	1.08865	-0.96309
H	-3.53558	-0.11973	0.21378
H	-3.00543	-0.62805	-1.41747
Li	0.02210	-0.01520	-1.74883

ACE1 Na B2PLYP def2sv

26

N	-1.23594	1.28126	0.26520
C	-0.37154	1.36628	-0.89130
H	-0.52390	2.32377	-1.43899
H	0.67434	1.39089	-0.54404
C	-0.54385	0.26894	-1.96075
H	-1.50300	0.41494	-2.48028
H	0.25853	0.39353	-2.71730
O	-0.59355	-1.05766	-1.48136
C	0.58369	-1.56776	-0.89710
H	1.03080	-2.34398	-1.54922
H	1.34214	-0.77509	-0.78193
C	0.24902	-2.20133	0.44969
H	-0.41443	-3.06475	0.28389
H	1.18378	-2.57613	0.91549
O	-0.45913	-1.34443	1.31819
C	0.22725	-0.18607	1.74578

H	0.50015	-0.28081	2.81617
H	1.17375	-0.06181	1.19239
C	-0.64948	1.05820	1.56888
H	-0.03015	1.92868	1.87163
H	-1.47851	1.00416	2.29322
C	-2.59707	0.85504	0.03737
H	-2.69855	-0.21506	-0.23150
H	-3.05003	1.45345	-0.77570
H	-3.19699	1.03863	0.94425
Na	3.85609	1.09614	-0.06041

ACE1 Na B2PLYP def2tzv

26

N	1.04860	-1.28593	0.15836
C	0.20469	-1.03081	-1.02255
H	0.04052	-1.95350	-1.59699
H	-0.78662	-0.70052	-0.71070
C	0.81404	-0.00381	-1.98665
H	1.76664	-0.36498	-2.36846
H	0.12940	0.15002	-2.82796
O	1.14634	1.28975	-1.37778
C	0.03712	2.01731	-0.76433
H	-0.12641	2.94067	-1.32587
H	-0.88205	1.43460	-0.82037
C	0.36772	2.40203	0.67627
H	1.24149	3.04946	0.69326
H	-0.48661	2.95078	1.08852
O	0.73067	1.29296	1.55872
C	-0.25580	0.22291	1.71266
H	-0.63068	0.24630	2.73824
H	-1.10685	0.39084	1.05255

C	0.38186	-1.14579	1.46355
H	-0.41214	-1.90231	1.61082
H	1.13815	-1.32076	2.23061
C	1.83389	-2.52660	0.05677
H	2.39351	-2.53236	-0.87815
H	1.20427	-3.43163	0.08680
H	2.54775	-2.57720	0.87876
Na	-4.42617	-0.69555	-0.29286

ACE1 Na B3LYP def2sv

26

N	1.53624	-0.73706	0.10062
C	0.74001	-0.77634	-1.11768
H	1.09136	-1.57126	-1.80976
H	-0.30152	-1.04999	-0.87829
C	0.75911	0.55168	-1.89411
H	1.78413	0.75083	-2.25152
H	0.10830	0.45185	-2.79027
O	0.40919	1.69361	-1.13709
C	-0.89890	1.74575	-0.60813
H	-1.46560	2.57315	-1.08331
H	-1.45939	0.81986	-0.83040
C	-0.86235	2.01628	0.89955
H	-0.43884	3.02112	1.06894
H	-1.90787	2.02692	1.27873
O	-0.04352	1.14569	1.64855
C	-0.37082	-0.22882	1.65532
H	-0.73847	-0.52069	2.66014
H	-1.19263	-0.44702	0.95032
C	0.86021	-1.08400	1.33794
H	0.53933	-2.15605	1.37967

H	1.58804	-0.94744	2.15820
C	2.85229	-1.32457	-0.03215
H	3.36878	-0.90944	-0.91589
H	2.83549	-2.43708	-0.14427
H	3.46855	-1.08700	0.85379
Na	-3.58509	-1.95206	-0.53514

ACE1 Na B3LYP def2tzv

26

N	-0.74020	-1.41845	-0.22039
C	-0.09648	-1.02591	1.04262
H	0.15473	-1.90686	1.65005
H	0.85544	-0.53521	0.83940
C	-0.96900	-0.11427	1.91602
H	-1.88771	-0.62775	2.19404
H	-0.42034	0.13664	2.83126
O	-1.43107	1.11074	1.26182
C	-0.40182	2.01552	0.76215
H	-0.46480	2.95436	1.31947
H	0.59273	1.60498	0.93655
C	-0.62151	2.34173	-0.71355
H	-1.58232	2.83727	-0.83944
H	0.16839	3.03279	-1.03100
O	-0.69048	1.19813	-1.61652
C	0.45758	0.29558	-1.64678
H	0.93412	0.37936	-2.62648
H	1.19862	0.59091	-0.90321
C	0.02013	-1.15772	-1.45104
H	0.93342	-1.77958	-1.52133
H	-0.61724	-1.44085	-2.29100
C	-1.34345	-2.75620	-0.18821

H	-2.00139	-2.84865	0.67598
H	-0.59291	-3.56381	-0.13212
H	-1.94486	-2.91439	-1.08399
Na	4.05048	-0.18667	0.54863

ACE1 Na B97-1 def2sv

26

N	1.43786	0.84395	-0.06825
C	0.88646	1.45361	1.12606
H	1.66670	1.38992	1.91042
H	0.65978	2.54646	1.00663
C	-0.39881	0.80379	1.67078
H	-0.57133	1.22222	2.68235
H	-1.27862	1.09753	1.06313
O	-0.32820	-0.60226	1.77683
C	-1.09667	-1.37314	0.87869
H	-1.85374	-1.95841	1.44416
H	-1.66073	-0.72168	0.18400
C	-0.24745	-2.38296	0.09379
H	0.14813	-3.13734	0.79834
H	-0.92978	-2.90974	-0.61282
O	0.88467	-1.88179	-0.57052
C	0.71060	-0.96030	-1.62175
H	1.63108	-1.04361	-2.22929
H	-0.14349	-1.24595	-2.27544
C	0.54397	0.51222	-1.16334
H	-0.49946	0.69858	-0.86518
H	0.71072	1.16393	-2.05157
C	2.71245	1.39389	-0.47139
H	3.41694	1.39187	0.38265
H	2.64506	2.44538	-0.85106

H	3.16040	0.77716	-1.27307
Na	-3.66198	1.41507	-1.05119

ACE1 Na B97-1 def2tzv

26

N	0.69582	-1.56162	0.26093
C	0.17118	-1.25755	-1.06801
H	0.15943	-2.16395	-1.69872
H	-0.87578	-0.95465	-0.96815
C	0.95697	-0.22024	-1.91117
H	1.92328	-0.63635	-2.19966
H	0.38580	-0.02355	-2.82912
O	1.30160	1.03143	-1.24979
C	0.19178	1.87465	-0.83479
H	0.18217	2.78359	-1.44758
H	-0.76148	1.36344	-0.99455
C	0.36454	2.28983	0.62729
H	1.28293	2.86702	0.74110
H	-0.48517	2.92591	0.91158
O	0.50943	1.17949	1.55906
C	-0.59607	0.22925	1.59217
H	-1.11110	0.32196	2.55592
H	-1.32295	0.46294	0.80932
C	-0.09371	-1.21940	1.44450
H	-0.99399	-1.85331	1.49846
H	0.51231	-1.46738	2.32016
C	2.14642	-1.68606	0.39235
H	2.68509	-0.73032	0.31376
H	2.53472	-2.35679	-0.38368
H	2.38801	-2.13521	1.35903
Na	-4.06445	-0.47476	-0.52206

ACE1 Na CAM-B3LYP def2sv

26

N	0.82630	1.50051	-0.18054
C	0.22143	1.21761	1.09883
H	0.25041	2.10729	1.76693
H	-0.84932	1.00920	0.93722
C	0.86165	0.08294	1.91858
H	1.84016	0.41701	2.29677
H	0.22184	-0.11113	2.80391
O	1.13319	-1.10344	1.21611
C	0.03915	-1.85062	0.75343
H	-0.04498	-2.79947	1.31904
H	-0.90765	-1.30618	0.91258
C	0.23470	-2.18789	-0.71994
H	1.11236	-2.84573	-0.82245
H	-0.65126	-2.74946	-1.08156
O	0.50494	-1.06946	-1.52548
C	-0.51017	-0.10116	-1.61929
H	-0.97960	-0.13545	-2.62288
H	-1.31209	-0.30756	-0.89078
C	0.05629	1.29966	-1.38463
H	-0.80116	2.00367	-1.42451
H	0.70567	1.56018	-2.23600
C	2.26369	1.44526	-0.25995
H	2.68038	0.41921	-0.21397
H	2.71371	2.02991	0.56467
H	2.60218	1.90864	-1.20191
Na	-4.04275	0.56769	0.44632

ACE1 Na CAM-B3LYP def2tzv

26

N	1.70480	0.55935	-0.06757
C	0.81880	0.83867	1.06171
H	1.21811	1.65088	1.68233
H	-0.14351	1.19952	0.70179
C	0.60747	-0.36199	1.98178
H	1.55632	-0.67369	2.41132
H	-0.06425	-0.07510	2.79692
O	0.10251	-1.55498	1.32419
C	-1.16635	-1.44159	0.63836
H	-1.89397	-2.08249	1.14011
H	-1.54712	-0.42252	0.69251
C	-1.04942	-1.91643	-0.80068
H	-0.75721	-2.96319	-0.81469
H	-2.03218	-1.82596	-1.27420
O	-0.04028	-1.24905	-1.59647
C	-0.14139	0.19003	-1.73354
H	-0.39344	0.42282	-2.76924
H	-0.94762	0.57804	-1.11277
C	1.18253	0.86259	-1.39888
H	1.04361	1.94707	-1.55548
H	1.92772	0.53317	-2.12354
C	3.07760	1.01517	0.12897
H	3.46240	0.64737	1.07874
H	3.16390	2.11350	0.12927
H	3.71639	0.62518	-0.66219
Na	-3.70146	1.97485	0.27855

ACE1 Na DSDPBEP86 def2sv

26

N	1.58735	0.53642	-0.11772
C	0.64148	1.02164	0.87301

H 0.98489 1.97138 1.33794

H -0.31704 1.27132 0.38865

C 0.40632 0.00561 1.99239

H 1.34361 -0.13587 2.55801

H -0.35518 0.40602 2.69720

O 0.05026 -1.28259 1.53974

C -1.17249 -1.38223 0.84684

H -1.90246 -1.96727 1.44400

H -1.62497 -0.38732 0.68828

C -0.96475 -2.10301 -0.48152

H -0.65602 -3.14226 -0.27634

H -1.93535 -2.13921 -1.02294

O 0.05961 -1.56676 -1.28487

C -0.12500 -0.24779 -1.74788

H -0.32059 -0.25548 -2.83979

H -1.00939 0.22023 -1.27848

C 1.13161 0.57908 -1.49197

H 0.93966 1.61881 -1.85965

H 1.94237 0.16591 -2.11985

C 2.91648 1.07970 0.05408

H 3.25113 0.93094 1.09632

H 2.97925 2.17206 -0.17151

H 3.62949 0.55348 -0.60595

Na -3.26743 2.18538 -0.13823

ACE1 Na DSDPBEP86 def2tzv

26

N 1.66095 0.46279 -0.10764

C 0.76128 0.87643 0.98399

H 1.16958 1.74486 1.52681

H -0.19751 1.20939 0.57614

C	0.53240	-0.23673	2.01408
H	1.47762	-0.51770	2.48153
H	-0.15815	0.12324	2.78953
O	0.02825	-1.49112	1.44856
C	-1.25090	-1.39468	0.75294
H	-1.99927	-1.97049	1.31002
H	-1.59315	-0.35695	0.72374
C	-1.15349	-1.99351	-0.64980
H	-0.89823	-3.05189	-0.58191
H	-2.13522	-1.89436	-1.13421
O	-0.11007	-1.42933	-1.50187
C	-0.19451	0.00605	-1.76051
H	-0.43226	0.15443	-2.81963
H	-1.00800	0.44926	-1.18060
C	1.14101	0.69271	-1.46566
H	1.00547	1.77259	-1.68205
H	1.89153	0.31248	-2.16611
C	3.02927	0.98127	0.05445
H	3.40837	0.71226	1.04359
H	3.07973	2.08112	-0.04967
H	3.68370	0.53095	-0.69621
Na	-3.22333	2.29415	0.13291

ACE1 Na HSE06 def2sv

26

N	0.90744	1.46381	-0.19192
C	0.27134	1.23417	1.07820
H	0.35024	2.12978	1.73785
H	-0.81168	1.09714	0.90987
C	0.82889	0.07513	1.92274
H	1.81759	0.36075	2.31829

H 0.16172 -0.07027 2.79936

O 1.05088 -1.13004 1.24115

C -0.07285 -1.82108 0.76980

H -0.22615 -2.75327 1.35145

H -0.99369 -1.22272 0.89871

C 0.13114 -2.19705 -0.69174

H 0.97452 -2.90380 -0.76594

H -0.77773 -2.72047 -1.05838

O 0.47664 -1.11424 -1.51340

C -0.47970 -0.09361 -1.63199

H -0.93851 -0.11388 -2.64261

H -1.30552 -0.24579 -0.91343

C 0.15454 1.27805 -1.40507

H -0.66698 2.02406 -1.47745

H 0.83406 1.49309 -2.24746

C 2.33902 1.35575 -0.23522

H 2.72353 0.31766 -0.14816

H 2.79127 1.95096 0.58263

H 2.71583 1.77833 -1.18304

Na -4.02319 0.69071 0.41086

ACE1 Na HSE06 def2tzv

26

N -0.70483 -1.52175 -0.28300

C -0.12155 -1.25609 1.01517

H -0.05520 -2.17880 1.61687

H 0.91050 -0.92626 0.87321

C -0.87851 -0.26662 1.91310

H -1.82962 -0.69990 2.22167

H -0.27974 -0.09487 2.81681

O -1.24682 0.99217 1.30182

C	-0.15795	1.83544	0.86666
H	-0.10822	2.72320	1.50577
H	0.79638	1.31289	0.96645
C	-0.38899	2.29302	-0.56097
H	-1.30833	2.87500	-0.61727
H	0.44701	2.93843	-0.85948
O	-0.57252	1.21853	-1.51189
C	0.51901	0.27331	-1.62036
H	0.99172	0.38932	-2.60164
H	1.28435	0.48220	-0.86821
C	0.01899	-1.16389	-1.48904
H	0.90728	-1.80326	-1.61500
H	-0.63519	-1.38037	-2.33646
C	-2.14178	-1.70182	-0.33844
H	-2.71470	-0.77573	-0.18945
H	-2.46135	-2.41800	0.42713
H	-2.42323	-2.12059	-1.30631
Na	4.07922	-0.49425	0.44643

ACE1 Na M062X def2sv

26

N	1.20118	1.30437	-0.27582
C	0.30611	1.38447	0.85641
H	0.40074	2.36241	1.37754
H	-0.73269	1.34661	0.48579
C	0.51056	0.32467	1.95142
H	1.46266	0.51640	2.47030
H	-0.29931	0.43707	2.70092
O	0.60163	-1.00162	1.49740
C	-0.54886	-1.54495	0.90514
H	-0.99731	-2.31211	1.56516

H	-1.31611	-0.76570	0.75260
C	-0.16728	-2.19589	-0.41796
H	0.51281	-3.03896	-0.21778
H	-1.07742	-2.59911	-0.90617
O	0.53649	-1.32883	-1.26927
C	-0.18379	-0.21977	-1.74825
H	-0.42903	-0.35825	-2.81962
H	-1.14788	-0.11669	-1.21719
C	0.64238	1.05676	-1.58439
H	0.00156	1.90530	-1.89774
H	1.48372	1.02369	-2.29638
C	2.54542	0.85079	-0.00996
H	2.60776	-0.21428	0.28681
H	2.99703	1.45961	0.79590
H	3.16388	0.99869	-0.91053
Na	-3.88827	0.99382	0.02016

ACE1 Na M062X def2tzv

26

N	0.79471	1.55523	-0.14156
C	0.25503	1.17122	1.15868
H	0.25958	2.02530	1.84962
H	-0.79325	0.89388	1.03393
C	1.01296	0.06024	1.90294
H	1.99930	0.41505	2.19278
H	0.45824	-0.18306	2.81445
O	1.27368	-1.14181	1.14030
C	0.11967	-1.88300	0.68948
H	0.06334	-2.82921	1.23094
H	-0.79635	-1.32927	0.89853
C	0.26601	-2.17580	-0.79471

H	1.14950	-2.78762	-0.96111
H	-0.61401	-2.72558	-1.14204
O	0.47331	-0.98697	-1.59067
C	-0.60420	-0.02037	-1.57134
H	-1.11749	-0.03833	-2.53546
H	-1.33775	-0.28007	-0.80586
C	-0.04219	1.37942	-1.32274
H	-0.89915	2.06176	-1.28165
H	0.55265	1.67345	-2.18812
C	2.24170	1.45493	-0.30345
H	2.60311	0.42212	-0.29140
H	2.74311	2.00793	0.49471
H	2.52881	1.91548	-1.24791
Na	-4.16659	0.45290	0.54357

ACE1 Na M06 def2sv

26

N	-1.13002	-1.35506	-0.09166
C	-0.40906	-1.15825	1.13787
H	-0.56632	-2.00757	1.84720
H	0.67600	-1.17036	0.91462
C	-0.77240	0.09766	1.93599
H	-1.77998	-0.02695	2.37411
H	-0.06337	0.18716	2.79035
O	-0.83875	1.29014	1.20179
C	0.35573	1.76904	0.65178
H	0.68533	2.69224	1.17622
H	1.17697	1.03536	0.78144
C	0.14878	2.09708	-0.81526
H	-0.58042	2.92399	-0.89924
H	1.11088	2.45819	-1.24506

O	-0.38950	1.03968	-1.55942
C	0.39985	-0.11409	-1.66818
H	0.80787	-0.20818	-2.69877
H	1.28137	-0.05770	-0.99692
C	-0.41731	-1.35665	-1.34323
H	0.27639	-2.22667	-1.40720
H	-1.16482	-1.50539	-2.14549
C	-2.53187	-1.04670	-0.08484
H	-2.76231	0.04236	-0.05254
H	-3.02561	-1.51913	0.79040
H	-3.01550	-1.46703	-0.98645
Na	4.00338	-0.91200	0.39776

ACE1 Na M06 def2tzv

26

N	0.70638	1.48936	-0.26505
C	0.18360	1.24023	1.05783
H	0.17755	2.15947	1.66844
H	-0.86461	0.93781	0.96991
C	0.96896	0.22175	1.89687
H	1.93523	0.64425	2.17434
H	0.41041	0.02890	2.82188
O	1.29679	-1.01401	1.23372
C	0.19167	-1.83919	0.82858
H	0.16302	-2.74473	1.44349
H	-0.75759	-1.31825	0.98215
C	0.36287	-2.25513	-0.61950
H	1.28179	-2.83086	-0.72703
H	-0.48201	-2.89692	-0.90070
O	0.49957	-1.15540	-1.53918
C	-0.60205	-0.22781	-1.58538

H	-1.11865	-0.32594	-2.54594
H	-1.32979	-0.45882	-0.80260
C	-0.09841	1.20424	-1.43350
H	-0.98809	1.85233	-1.46946
H	0.50219	1.45386	-2.31149
C	2.13615	1.66235	-0.39532
H	2.70710	0.72921	-0.28770
H	2.50267	2.36531	0.36245
H	2.36855	2.08995	-1.37229
Na	-4.06182	0.47323	0.52691

ACE1 Na MP2 def2sv

26

N	-0.89705	-1.50576	-0.10642
C	-0.31239	-1.15476	1.16743
H	-0.38801	-1.99917	1.89166
H	0.76941	-0.98703	1.02530
C	-0.93924	0.04545	1.89584
H	-1.94305	-0.23373	2.25435
H	-0.32242	0.27000	2.79261
O	-1.14468	1.20056	1.11254
C	0.01690	1.85914	0.66178
H	0.14049	2.82837	1.18767
H	0.92140	1.26403	0.87906
C	-0.12074	2.12534	-0.83063
H	-0.95647	2.82641	-0.99179
H	0.80724	2.60596	-1.20765
O	-0.44866	0.96953	-1.56991
C	0.54422	-0.03362	-1.58229
H	1.04552	-0.06538	-2.57291
H	1.33178	0.17998	-0.83882

C	-0.08238	-1.39565	-1.29388
H	0.74780	-2.13473	-1.26412
H	-0.71573	-1.67619	-2.15365
C	-2.32401	-1.32916	-0.23698
H	-2.65166	-0.27131	-0.22605
H	-2.84705	-1.86155	0.58132
H	-2.65974	-1.78600	-1.18403
Na	4.09567	-0.58914	0.50393

ACE1 Na MP2 def2tzv

26

N	1.16630	1.43141	-0.02564
C	0.42601	1.11669	1.20738
H	0.54465	1.92498	1.95021
H	-0.64189	1.07871	0.96512
C	0.84351	-0.17125	1.95806
H	1.86104	-0.07963	2.34023
H	0.15632	-0.31178	2.80490
O	0.89488	-1.40083	1.14849
C	-0.38310	-1.83318	0.57146
H	-0.70493	-2.75866	1.06311
H	-1.15297	-1.07522	0.74343
C	-0.19006	-2.10847	-0.92067
H	0.53635	-2.90877	-1.06650
H	-1.15400	-2.40412	-1.35753
O	0.37450	-0.96685	-1.65790
C	-0.45418	0.24899	-1.64853
H	-0.88570	0.39195	-2.64725
H	-1.28052	0.13946	-0.93838
C	0.41147	1.47047	-1.29041
H	-0.26449	2.34071	-1.28610

H	1.14217	1.62969	-2.09045
C	2.57876	1.01006	-0.07501
H	2.71298	-0.07941	-0.05873
H	3.12221	1.44592	0.77241
H	3.02473	1.40656	-0.99161
Na	-4.06632	0.88922	0.47620

ACE1 Na PBE0 def2sv

26

N	1.43299	-0.81541	0.13757
C	0.64081	-0.85017	-1.07234
H	0.92263	-1.70572	-1.72255
H	-0.42060	-1.02381	-0.82340
C	0.77307	0.42903	-1.90103
H	1.81014	0.52064	-2.26678
H	0.11491	0.35342	-2.79372
O	0.52987	1.61391	-1.18903
C	-0.76345	1.79335	-0.67827
H	-1.26573	2.63582	-1.19654
H	-1.39363	0.90328	-0.85897
C	-0.70836	2.12892	0.80771
H	-0.21424	3.10724	0.93195
H	-1.74904	2.23362	1.18412
O	0.04114	1.23911	1.58781
C	-0.40363	-0.08974	1.65485
H	-0.80055	-0.30507	2.66747
H	-1.23913	-0.26417	0.95376
C	0.73894	-1.05916	1.37807
H	0.32603	-2.09596	1.45978
H	1.47830	-0.95629	2.19265
C	2.69790	-1.49180	0.03046

H	3.24357	-1.14328	-0.86408
H	2.60625	-2.60286	-0.04392
H	3.32736	-1.26557	0.90946
Na	-3.56335	-1.87910	-0.47265

ACE1 Na PBE0 def2tzv

26

N	1.44026	0.80623	-0.14487
C	0.55678	0.90326	1.01468
H	0.76442	1.81338	1.59769
H	-0.48003	1.00520	0.68657
C	0.67733	-0.27795	1.97380
H	1.69016	-0.33235	2.37404
H	-0.01670	-0.12777	2.81128
O	0.45786	-1.57882	1.37279
C	-0.82560	-1.78328	0.74140
H	-1.37025	-2.55950	1.28964
H	-1.43232	-0.87606	0.78999
C	-0.66484	-2.26605	-0.69044
H	-0.13786	-3.22026	-0.69513
H	-1.66751	-2.42579	-1.10913
O	0.12062	-1.41683	-1.55710
C	-0.32746	-0.05018	-1.71550
H	-0.66433	0.08951	-2.74751
H	-1.18799	0.15211	-1.07256
C	0.80335	0.93456	-1.45182
H	0.39154	1.94899	-1.62452
H	1.57825	0.77927	-2.20760
C	2.65503	1.60216	-0.02328
H	3.16216	1.37366	0.91584
H	2.45972	2.68950	-0.04751

H	3.34115	1.36353	-0.83834
Na	-3.48979	2.02481	0.29753

ACE1 Na PBE def2sv

26

N	0.78343	1.51346	-0.21518
C	0.20704	1.24294	1.08458
H	0.24271	2.15167	1.74778
H	-0.87648	1.02510	0.95093
C	0.88137	0.12506	1.92353
H	1.86744	0.48925	2.28490
H	0.25021	-0.05773	2.83207
O	1.18231	-1.08153	1.24474
C	0.08602	-1.85427	0.79101
H	0.01145	-2.80537	1.37674
H	-0.87734	-1.31498	0.94493
C	0.28008	-2.21981	-0.68591
H	1.18505	-2.85868	-0.78322
H	-0.60002	-2.82839	-1.02155
O	0.51497	-1.11256	-1.53783
C	-0.53372	-0.16156	-1.62003
H	-1.02907	-0.21686	-2.62308
H	-1.32482	-0.37899	-0.86625
C	0.00265	1.26612	-1.40931
H	-0.88728	1.94860	-1.45544
H	0.64051	1.53546	-2.27956
C	2.22524	1.53404	-0.30920
H	2.70990	0.52579	-0.24372
H	2.65501	2.16665	0.50471
H	2.53286	1.99604	-1.27344
Na	-4.04132	0.54417	0.46430

ACE1 Na PBE def2tzv

26

N	-0.69891	1.66849	0.23866
C	-0.64306	1.56736	-1.21787
H	-1.56914	2.02054	-1.60524
H	0.19567	2.18479	-1.59813
C	-0.46220	0.17618	-1.87112
H	-0.67997	0.26606	-2.94995
H	0.58212	-0.16159	-1.77463
O	-1.36466	-0.84128	-1.30481
C	-0.70874	-2.02812	-0.74543
H	-1.20423	-2.91907	-1.16638
H	0.34309	-2.06128	-1.07252
C	-0.82736	-2.13576	0.77770
H	-1.87771	-2.28791	1.06070
H	-0.25403	-3.03280	1.08361
O	-0.41864	-0.96833	1.56561
C	0.78510	-0.24113	1.14547
H	1.57373	-0.38550	1.90612
H	1.17062	-0.63101	0.19424
C	0.45942	1.26713	1.03385
H	1.36402	1.76231	0.63050
H	0.30829	1.65916	2.05351
C	-2.02637	1.53930	0.85645
H	-2.43866	0.51628	0.84110
H	-2.73228	2.20754	0.33606
H	-1.96607	1.86657	1.90595
Na	4.26205	0.08397	-0.31603

ACE1 Na SCS-MP2 def2sv

26

N 1.44763 0.75918 -0.14086

C 0.58754 0.88872 1.02938

H 0.85039 1.78496 1.63310

H -0.45929 1.04927 0.71993

C 0.67733 -0.33815 1.94234

H 1.69939 -0.40916 2.35423

H -0.02427 -0.21543 2.79685

O 0.46093 -1.57063 1.28011

C -0.82913 -1.76032 0.73279

H -1.36114 -2.56313 1.28543

H -1.44709 -0.84993 0.83263

C -0.71264 -2.18956 -0.72916

H -0.22767 -3.18030 -0.76863

H -1.73419 -2.29390 -1.15631

O 0.10078 -1.35051 -1.52374

C -0.34929 -0.01996 -1.69322

H -0.69468 0.12831 -2.73764

H -1.21839 0.19351 -1.04614

C 0.79049 0.96068 -1.42142

H 0.38866 1.99797 -1.54920

H 1.55415 0.81654 -2.20839

C 2.67534 1.52531 -0.02263

H 3.18079 1.27670 0.92790

H 2.50943 2.62937 -0.04487

H 3.36318 1.26505 -0.84767

Na -3.45857 2.00226 0.33776

ACE1 Na SCS-MP2 def2tzv

26

N -1.67762 0.45510 0.10293

C -0.77065 0.87626 -0.99420

H	-1.18537	1.74103	-1.54095
H	0.18739	1.21371	-0.58415
C	-0.53380	-0.24456	-2.02584
H	-1.47587	-0.53269	-2.49739
H	0.16669	0.11184	-2.79539
O	-0.02960	-1.50760	-1.45033
C	1.26016	-1.40526	-0.75194
H	2.00624	-1.98520	-1.30988
H	1.59984	-0.36544	-0.72673
C	1.16135	-2.00217	0.66031
H	0.90646	-3.06197	0.59969
H	2.13955	-1.89148	1.15115
O	0.10367	-1.43503	1.51252
C	0.19081	0.01126	1.77060
H	0.42114	0.15735	2.83298
H	1.01029	0.45095	1.19432
C	-1.14972	0.70269	1.46652
H	-1.00733	1.78640	1.66364
H	-1.90313	0.33285	2.17112
C	-3.04833	1.00264	-0.05510
H	-3.42804	0.74329	-1.04769
H	-3.07821	2.10274	0.05692
H	-3.70631	0.55386	0.69467
Na	3.25805	2.30481	-0.13659

ACE1 NoMetal B3LYP def2sv

25

N	-1.43437	-0.11386	-0.12280
C	-1.02666	0.94261	0.79333
H	-1.90368	1.47315	1.22200
H	-0.50789	0.50655	1.66323

C	-0.12615	1.99975	0.13147
H	-0.69915	2.53183	-0.64756
H	0.16779	2.75016	0.89756
O	1.00838	1.48667	-0.53879
C	1.96887	0.80336	0.23918
H	2.92275	1.37045	0.24398
H	1.64977	0.72616	1.29375
C	2.25730	-0.58196	-0.34897
H	2.71937	-0.45513	-1.34314
H	3.00208	-1.08947	0.30253
O	1.12382	-1.39059	-0.57534
C	0.34120	-1.75975	0.54232
H	0.45424	-2.84595	0.73567
H	0.68471	-1.24720	1.45830
C	-1.14185	-1.47808	0.28072
H	-1.70904	-1.80105	1.19017
H	-1.47180	-2.13971	-0.54064
C	-2.76265	0.05994	-0.67125
H	-2.87436	1.07666	-1.08835
H	-3.57874	-0.08661	0.07928
H	-2.93340	-0.65673	-1.49494

ACE1 NoMetal B3LYP def2tzv

25

N	-1.45284	-0.11377	-0.14442
C	-1.03156	0.94866	0.78287
H	-1.90109	1.46051	1.21864
H	-0.49917	0.51582	1.63029
C	-0.16256	2.02891	0.12389
H	-0.71991	2.53002	-0.66565
H	0.11557	2.77332	0.87870

O	1.03853	1.53325	-0.55034
C	1.99375	0.79674	0.26979
H	2.92675	1.36575	0.31216
H	1.63038	0.69934	1.29276
C	2.31216	-0.56498	-0.34315
H	2.75822	-0.42859	-1.32657
H	3.04317	-1.06695	0.30175
O	1.16721	-1.43564	-0.58874
C	0.32692	-1.77401	0.55780
H	0.43682	-2.84220	0.76006
H	0.66278	-1.24362	1.44906
C	-1.14818	-1.49372	0.26261
H	-1.71787	-1.80312	1.15979
H	-1.46557	-2.14804	-0.55164
C	-2.82262	0.04672	-0.64770
H	-2.95482	1.04690	-1.06048
H	-3.58734	-0.10218	0.13440
H	-3.01130	-0.67133	-1.44642

ACE1 NoMetal B2PLYP def2sv

25

N	-1.42060	-0.11213	-0.13682
C	-1.01967	0.93710	0.78929
H	-1.89879	1.45998	1.21951
H	-0.49852	0.49840	1.65468
C	-0.12616	1.99412	0.12882
H	-0.70204	2.52325	-0.64762
H	0.17344	2.74274	0.89187
O	1.00375	1.47564	-0.54438
C	1.95936	0.80027	0.24554
H	2.90880	1.37120	0.26091

H	1.62722	0.72081	1.29404
C	2.25378	-0.57979	-0.34150
H	2.72503	-0.45040	-1.32889
H	2.98601	-1.09362	0.31595
O	1.11700	-1.37956	-0.58147
C	0.34185	-1.75202	0.53952
H	0.46047	-2.83579	0.73385
H	0.68547	-1.23541	1.45111
C	-1.13786	-1.47348	0.27894
H	-1.70593	-1.78191	1.19054
H	-1.46889	-2.13940	-0.53651
C	-2.76050	0.05865	-0.65724
H	-2.88082	1.07651	-1.06441
H	-3.55653	-0.09560	0.10937
H	-2.94153	-0.65369	-1.48006

ACE1 NoMetal B2PLYP def2tzv

25

N	-1.44206	-0.11032	-0.16234
C	-1.02702	0.94627	0.77935
H	-1.90035	1.45116	1.21582
H	-0.49206	0.50760	1.62215
C	-0.16132	2.03026	0.12360
H	-0.71773	2.53080	-0.66617
H	0.12363	2.76954	0.88011
O	1.04050	1.52980	-0.55459
C	1.99104	0.79323	0.27648
H	2.92209	1.36351	0.32889
H	1.61351	0.69187	1.29371
C	2.31322	-0.56697	-0.33920
H	2.76362	-0.43013	-1.31976

H	3.03275	-1.07577	0.31214
O	1.16158	-1.43272	-0.59501
C	0.32545	-1.77236	0.55798
H	0.43758	-2.83975	0.76082
H	0.66449	-1.23729	1.44513
C	-1.14973	-1.49062	0.26332
H	-1.71922	-1.77725	1.16746
H	-1.47428	-2.15031	-0.54328
C	-2.82728	0.04691	-0.63696
H	-2.96744	1.05032	-1.03793
H	-3.57027	-0.11145	0.16259
H	-3.02468	-0.66763	-1.43590

ACE1 NoMetal B97-1 def2sv

25

N	-1.43079	-0.11435	-0.12551
C	-1.02536	0.94079	0.79515
H	-1.90450	1.47005	1.22522
H	-0.50447	0.50231	1.66509
C	-0.12442	2.00078	0.13133
H	-0.70092	2.53337	-0.64713
H	0.17122	2.75168	0.89868
O	1.00671	1.48761	-0.54042
C	1.96456	0.80617	0.23951
H	2.92015	1.37333	0.24460
H	1.64354	0.73024	1.29570
C	2.25460	-0.58345	-0.34728
H	2.72176	-0.45706	-1.34082
H	2.99720	-1.09126	0.30910
O	1.12263	-1.38985	-0.57748
C	0.34433	-1.76141	0.54010

H	0.45815	-2.84951	0.73140
H	0.68923	-1.24931	1.45836
C	-1.14255	-1.47932	0.28208
H	-1.70860	-1.79845	1.19522
H	-1.47588	-2.14259	-0.53886
C	-2.76184	0.06099	-0.67028
H	-2.87151	1.07961	-1.08751
H	-3.57647	-0.08477	0.08382
H	-2.93408	-0.65667	-1.49476

ACE1 NoMetal B97-1 def2tzv

25

N	-1.44885	-0.11193	-0.15343
C	-1.03017	0.94814	0.78068
H	-1.90263	1.46175	1.21453
H	-0.49946	0.51176	1.63103
C	-0.15621	2.03031	0.12267
H	-0.71668	2.53508	-0.66607
H	0.12305	2.77362	0.88207
O	1.04060	1.53428	-0.55268
C	1.98965	0.79812	0.27090
H	2.92746	1.36409	0.31488
H	1.62326	0.70280	1.29612
C	2.30873	-0.56990	-0.33942
H	2.76285	-0.43370	-1.32201
H	3.03729	-1.07121	0.31323
O	1.16601	-1.43743	-0.59081
C	0.32933	-1.77448	0.55552
H	0.43664	-2.84542	0.75898
H	0.66668	-1.24321	1.44937
C	-1.14999	-1.49201	0.26265

H	-1.71904	-1.79347	1.16577
H	-1.47215	-2.15124	-0.54925
C	-2.82633	0.04712	-0.64087
H	-2.96305	1.04994	-1.05213
H	-3.58263	-0.10347	0.15209
H	-3.02252	-0.67255	-1.43951

ACE1 NoMetal CAM-B3LYP def2sv

25

N	-1.42375	-0.11576	-0.12217
C	-1.01868	0.93465	0.79197
H	-1.89567	1.45819	1.22432
H	-0.49496	0.49844	1.65682
C	-0.12885	1.98954	0.12926
H	-0.70669	2.51755	-0.64673
H	0.16842	2.74088	0.89021
O	0.99747	1.47394	-0.53799
C	1.95453	0.80134	0.24001
H	2.90376	1.37229	0.25126
H	1.62924	0.72072	1.29092
C	2.24757	-0.57591	-0.34694
H	2.71426	-0.44572	-1.33660
H	2.98579	-1.08699	0.30561
O	1.11619	-1.37460	-0.57592
C	0.34397	-1.75002	0.53738
H	0.46408	-2.83368	0.73025
H	0.68646	-1.23527	1.45082
C	-1.13398	-1.47469	0.27968
H	-1.70051	-1.79359	1.18769
H	-1.46271	-2.13597	-0.54022
C	-2.74747	0.05934	-0.66673

H	-2.85895	1.07592	-1.08016
H	-3.55839	-0.08859	0.08536
H	-2.91972	-0.65398	-1.49079

ACE1 NoMetal CAM-B3LYP def2tzv

25

N	-1.44319	-0.11499	-0.14028
C	-1.02223	0.94071	0.78074
H	-1.89068	1.44859	1.21889
H	-0.48703	0.50876	1.62495
C	-0.16236	2.01602	0.12014
H	-0.72398	2.51081	-0.66845
H	0.11550	2.76405	0.86897
O	1.02957	1.51889	-0.54533
C	1.97942	0.79235	0.26947
H	2.90946	1.36228	0.31635
H	1.61379	0.69152	1.29004
C	2.29818	-0.56113	-0.34363
H	2.74538	-0.42027	-1.32423
H	3.02613	-1.06675	0.29875
O	1.15629	-1.41823	-0.58663
C	0.32826	-1.76244	0.55229
H	0.44405	-2.82828	0.75489
H	0.66238	-1.23085	1.44204
C	-1.14083	-1.48818	0.26105
H	-1.71090	-1.79600	1.15547
H	-1.45613	-2.14116	-0.55322
C	-2.80366	0.04854	-0.64431
H	-2.93446	1.04926	-1.05269
H	-3.56616	-0.10258	0.13642
H	-2.99262	-0.66501	-1.44504

ACE1 NoMetal DSDPBEP86 def2sv

25

N	-1.40858	-0.11415	-0.14412
C	-1.01468	0.92915	0.78933
H	-1.89847	1.44421	1.22492
H	-0.48798	0.48776	1.65259
C	-0.13029	1.98919	0.12768
H	-0.71374	2.51626	-0.64735
H	0.17406	2.74044	0.88912
O	0.99416	1.46861	-0.54889
C	1.94663	0.80217	0.24915
H	2.89413	1.37969	0.27294
H	1.60492	0.72055	1.29652
C	2.24937	-0.57464	-0.33672
H	2.73041	-0.44218	-1.32106
H	2.97528	-1.09288	0.32734
O	1.11336	-1.36919	-0.58703
C	0.34717	-1.74594	0.53634
H	0.47332	-2.83060	0.73226
H	0.69208	-1.22456	1.44726
C	-1.13209	-1.47255	0.27899
H	-1.70145	-1.77284	1.19463
H	-1.46481	-2.14364	-0.53443
C	-2.75291	0.05608	-0.65062
H	-2.87677	1.07791	-1.05215
H	-3.54084	-0.10333	0.12540
H	-2.93932	-0.65406	-1.47666

ACE1 NoMetal DSDPBEP86 def2tzv

25

N	-1.43152	-0.11022	-0.17292

C	-1.02130	0.93989	0.77751
H	-1.89924	1.43984	1.21893
H	-0.48154	0.49611	1.61882
C	-0.16077	2.02719	0.12222
H	-0.72343	2.52839	-0.66732
H	0.12823	2.76715	0.88152
O	1.03606	1.52533	-0.55866
C	1.98004	0.79350	0.28023
H	2.91270	1.36644	0.34220
H	1.59188	0.68957	1.29686
C	2.30809	-0.56636	-0.33470
H	2.76861	-0.42745	-1.31386
H	3.02188	-1.07880	0.32567
O	1.15717	-1.42645	-0.60014
C	0.32896	-1.76737	0.55516
H	0.44487	-2.83705	0.76191
H	0.66975	-1.22668	1.44265
C	-1.14704	-1.48811	0.26286
H	-1.71765	-1.76283	1.17386
H	-1.47668	-2.15425	-0.54098
C	-2.82325	0.04627	-0.62840
H	-2.96928	1.05573	-1.02106
H	-3.55513	-0.11972	0.18395
H	-3.02859	-0.66605	-1.43159

ACE1 NoMetal HSE06 def2sv

25

N	-1.41887	-0.11834	-0.12060
C	-1.01544	0.92862	0.79347
H	-1.89192	1.44816	1.23645
H	-0.48502	0.48937	1.65537

C -0.13145 1.98688 0.13154

H -0.71309 2.51613 -0.64265

H 0.16272 2.73967 0.89460

O 0.99294 1.47803 -0.53907

C 1.94465 0.80220 0.23937

H 2.89573 1.37263 0.25839

H 1.61569 0.71899 1.29101

C 2.24037 -0.57310 -0.34730

H 2.70835 -0.44189 -1.33766

H 2.98345 -1.08041 0.30535

O 1.11491 -1.37562 -0.57809

C 0.34591 -1.74728 0.53602

H 0.46730 -2.83115 0.73480

H 0.68956 -1.22903 1.44963

C -1.13134 -1.47396 0.28159

H -1.69652 -1.79693 1.19142

H -1.46209 -2.13643 -0.53870

C -2.73623 0.06007 -0.66997

H -2.84145 1.07660 -1.08828

H -3.55492 -0.08172 0.07732

H -2.90729 -0.65543 -1.49393

ACE1 NoMetal HSE06 def2tzv

25

N -1.43770 -0.11529 -0.14591

C -1.01834 0.93643 0.77797

H -1.88714 1.44327 1.22437

H -0.47922 0.50004 1.62174

C -0.16048 2.01447 0.12178

H -0.72586 2.51558 -0.66409

H 0.11462 2.76089 0.87778

O	1.02909	1.52403	-0.54893
C	1.96962	0.79162	0.26975
H	2.90500	1.35791	0.32716
H	1.59753	0.68893	1.29127
C	2.29117	-0.56162	-0.34050
H	2.74621	-0.42065	-1.32061
H	3.02166	-1.06221	0.30837
O	1.15577	-1.42260	-0.59028
C	0.32924	-1.75896	0.55040
H	0.44241	-2.82638	0.76234
H	0.66476	-1.22251	1.44087
C	-1.13945	-1.48482	0.26203
H	-1.70722	-1.79151	1.16258
H	-1.46013	-2.14328	-0.54949
C	-2.79862	0.04838	-0.64106
H	-2.93053	1.04974	-1.05393
H	-3.56177	-0.09798	0.14426
H	-2.99412	-0.66923	-1.43993

ACE1 NoMetal M062X def2sv

25

N	-1.40827	-0.11418	-0.13736
C	-1.00733	0.92721	0.79199
H	-1.88660	1.43979	1.23310
H	-0.47582	0.48180	1.64874
C	-0.12554	1.98680	0.12792
H	-0.71026	2.51342	-0.64404
H	0.18169	2.73572	0.88645
O	0.99383	1.46362	-0.54442
C	1.94441	0.79731	0.24743
H	2.88943	1.37321	0.27334

H	1.60121	0.71139	1.29324
C	2.24333	-0.57770	-0.34215
H	2.71973	-0.44313	-1.32629
H	2.96756	-1.09975	0.31604
O	1.10451	-1.36165	-0.58364
C	0.34417	-1.74470	0.53462
H	0.47354	-2.82670	0.72721
H	0.68744	-1.22338	1.44548
C	-1.13494	-1.47339	0.27865
H	-1.70496	-1.77522	1.18936
H	-1.46544	-2.13889	-0.53771
C	-2.74447	0.06495	-0.65475
H	-2.85771	1.08346	-1.06244
H	-3.53556	-0.08316	0.11655
H	-2.93090	-0.64804	-1.47533

ACE1 NoMetal M062X def2tzv

25

N	-1.42900	-0.11483	-0.15173
C	-1.01318	0.93367	0.78390
H	-1.88461	1.43281	1.22708
H	-0.47193	0.49526	1.62236
C	-0.16007	2.01338	0.11942
H	-0.73085	2.50392	-0.66662
H	0.12415	2.76199	0.86561
O	1.02536	1.51077	-0.54909
C	1.97034	0.79097	0.27485
H	2.89834	1.36357	0.33012
H	1.59417	0.68580	1.29239
C	2.29239	-0.56290	-0.34127
H	2.74807	-0.41662	-1.31783

H	3.00959	-1.07735	0.30622
O	1.14440	-1.40589	-0.59413
C	0.33086	-1.75907	0.55003
H	0.45582	-2.82417	0.75229
H	0.66495	-1.22242	1.43836
C	-1.14150	-1.48965	0.26219
H	-1.71348	-1.78148	1.15980
H	-1.45856	-2.14639	-0.54932
C	-2.79876	0.05450	-0.63768
H	-2.92788	1.05977	-1.03671
H	-3.54661	-0.10008	0.15545
H	-2.99667	-0.65528	-1.43995

ACE1 NoMetal M06 def2sv

25

N	-1.41589	-0.12141	-0.12690
C	-1.01633	0.92763	0.78850
H	-1.89921	1.44125	1.23208
H	-0.48524	0.49237	1.65619
C	-0.14343	1.98312	0.12120
H	-0.73225	2.50224	-0.65917
H	0.14802	2.74900	0.87604
O	0.98299	1.47175	-0.54201
C	1.93613	0.80958	0.24303
H	2.88332	1.39053	0.27214
H	1.60143	0.72636	1.29664
C	2.24667	-0.55945	-0.33800
H	2.72650	-0.42594	-1.32570
H	2.98880	-1.06347	0.32247
O	1.12811	-1.36745	-0.57631
C	0.35434	-1.74416	0.53130

H	0.47958	-2.82963	0.73236
H	0.69523	-1.22820	1.45141
C	-1.11772	-1.47750	0.26911
H	-1.69289	-1.80746	1.17352
H	-1.44028	-2.14055	-0.55881
C	-2.74416	0.04677	-0.65058
H	-2.86949	1.06676	-1.06173
H	-3.54567	-0.10675	0.11605
H	-2.92836	-0.66703	-1.47587

ACE1 NoMetal M06 def2tzv

25

N	-1.42994	-0.12665	-0.12720
C	-1.02012	0.92805	0.79352
H	-1.89334	1.42635	1.24069
H	-0.47110	0.49973	1.63533
C	-0.17865	2.00613	0.11979
H	-0.76014	2.48818	-0.66735
H	0.09492	2.76703	0.86183
O	1.00293	1.51358	-0.54903
C	1.95425	0.80688	0.26739
H	2.88293	1.38411	0.32215
H	1.58807	0.70006	1.29152
C	2.28599	-0.54380	-0.34152
H	2.74406	-0.39701	-1.31965
H	3.01687	-1.04203	0.30869
O	1.15567	-1.39947	-0.59205
C	0.34594	-1.76028	0.54472
H	0.47125	-2.82833	0.74676
H	0.67830	-1.23023	1.44134
C	-1.12468	-1.49692	0.26347

H	-1.69255	-1.81814	1.15797
H	-1.43805	-2.14662	-0.55883
C	-2.77553	0.04285	-0.65307
H	-2.89396	1.04827	-1.06209
H	-3.55519	-0.10542	0.11512
H	-2.95450	-0.66976	-1.46026

ACE1 NoMetal MP2 def2sv

25

N	-1.40428	-0.11071	-0.15449
C	-1.01333	0.92917	0.78526
H	-1.89816	1.44440	1.21853
H	-0.49021	0.48718	1.64995
C	-0.12690	1.98763	0.12626
H	-0.70980	2.51506	-0.64837
H	0.18035	2.73749	0.88762
O	0.99663	1.46242	-0.55310
C	1.94749	0.79915	0.25324
H	2.89430	1.37753	0.28073
H	1.60108	0.71821	1.29839
C	2.25085	-0.57672	-0.33196
H	2.73548	-0.44310	-1.31388
H	2.97130	-1.09924	0.33421
O	1.11016	-1.36658	-0.59009
C	0.34517	-1.74217	0.53696
H	0.47146	-2.82647	0.73493
H	0.68954	-1.21952	1.44650
C	-1.13251	-1.46829	0.27711
H	-1.70440	-1.76108	1.19345
H	-1.46369	-2.14288	-0.53360
C	-2.75856	0.05547	-0.64100

H	-2.89069	1.07829	-1.03500
H	-3.53273	-0.11174	0.14565
H	-2.95153	-0.65125	-1.46746

ACE1 NoMetal MP2 def2tzv

25

N	-1.43053	-0.10712	-0.18453
C	-1.02448	0.94370	0.77879
H	-1.90629	1.44046	1.21981
H	-0.48305	0.49522	1.61779
C	-0.16174	2.03734	0.12713
H	-0.71935	2.54196	-0.66397
H	0.13602	2.76917	0.89125
O	1.04234	1.53131	-0.56438
C	1.98961	0.79294	0.28428
H	2.92155	1.36748	0.35026
H	1.59240	0.68645	1.29770
C	2.31931	-0.56929	-0.33466
H	2.77758	-0.43382	-1.31540
H	3.02553	-1.08708	0.33006
O	1.15759	-1.43387	-0.60651
C	0.32628	-1.77541	0.55991
H	0.44079	-2.84627	0.76409
H	0.67251	-1.23365	1.44549
C	-1.15327	-1.49009	0.26787
H	-1.72174	-1.74617	1.18662
H	-1.49106	-2.15926	-0.53067
C	-2.83689	0.04585	-0.62928
H	-2.98513	1.05847	-1.01333
H	-3.55824	-0.12908	0.19042
H	-3.04016	-0.66368	-1.43548

ACE1 NoMetal PBE0 def2sv

25

N	-1.41898	-0.11830	-0.11998
C	-1.01618	0.92925	0.79348
H	-1.89304	1.44987	1.23426
H	-0.48773	0.49076	1.65682
C	-0.13090	1.98667	0.13179
H	-0.71218	2.51687	-0.64208
H	0.16389	2.73897	0.89513
O	0.99245	1.47758	-0.53906
C	1.94456	0.80271	0.23870
H	2.89583	1.37301	0.25540
H	1.61745	0.72106	1.29095
C	2.23982	-0.57325	-0.34686
H	2.70841	-0.44273	-1.33706
H	2.98258	-1.08033	0.30640
O	1.11491	-1.37539	-0.57792
C	0.34632	-1.74766	0.53558
H	0.46694	-2.83203	0.73246
H	0.69079	-1.23144	1.44991
C	-1.13110	-1.47365	0.28243
H	-1.69546	-1.79645	1.19270
H	-1.46250	-2.13612	-0.53755
C	-2.73579	0.05953	-0.67064
H	-2.84057	1.07587	-1.08957
H	-3.55529	-0.08218	0.07567
H	-2.90556	-0.65622	-1.49469

ACE1 NoMetal PBE0 def2tzv

25

N	-1.43787	-0.11427	-0.14701

C	-1.01841	0.93750	0.77672
H	-1.88742	1.44643	1.22080
H	-0.48175	0.50092	1.62225
C	-0.15774	2.01418	0.12132
H	-0.72263	2.51712	-0.66423
H	0.11783	2.75984	0.87849
O	1.03025	1.52320	-0.54909
C	1.96965	0.79128	0.26935
H	2.90631	1.35632	0.32534
H	1.59848	0.69031	1.29165
C	2.29009	-0.56339	-0.33934
H	2.74702	-0.42326	-1.31911
H	3.01982	-1.06381	0.31116
O	1.15527	-1.42288	-0.59001
C	0.32905	-1.75876	0.54961
H	0.44060	-2.82689	0.76086
H	0.66511	-1.22365	1.44097
C	-1.13989	-1.48344	0.26216
H	-1.70709	-1.78904	1.16369
H	-1.46171	-2.14260	-0.54878
C	-2.79985	0.04835	-0.63947
H	-2.93301	1.04949	-1.05328
H	-3.56160	-0.09770	0.14757
H	-2.99648	-0.67033	-1.43755

ACE1 NoMetal PBE def2sv

25

N	-1.43030	-0.11740	-0.12210
C	-1.02543	0.93759	0.79855
H	-1.90845	1.46489	1.24382
H	-0.49388	0.49435	1.66887

C	-0.13107	2.00319	0.13514
H	-0.71369	2.53702	-0.64822
H	0.15909	2.76175	0.90880
O	1.00629	1.49682	-0.54484
C	1.96207	0.80649	0.24241
H	2.92597	1.37512	0.26136
H	1.63158	0.72349	1.30303
C	2.25517	-0.57934	-0.35015
H	2.71966	-0.44703	-1.35224
H	3.01271	-1.08546	0.30466
O	1.12357	-1.39643	-0.58385
C	0.34466	-1.76257	0.54371
H	0.45960	-2.85626	0.74766
H	0.69246	-1.23978	1.46408
C	-1.14163	-1.48254	0.28425
H	-1.71229	-1.81236	1.20154
H	-1.47578	-2.14857	-0.54374
C	-2.75558	0.06168	-0.67736
H	-2.85920	1.08402	-1.10486
H	-3.58581	-0.07521	0.07413
H	-2.92793	-0.66421	-1.50379

ACE1 NoMetal PBE def2tzv

25

N	-1.44882	-0.11455	-0.15381
C	-1.02901	0.94548	0.78137
H	-1.90546	1.45563	1.23171
H	-0.48673	0.50339	1.63095
C	-0.16456	2.03393	0.12625
H	-0.72961	2.54199	-0.66699
H	0.11200	2.78178	0.89343

O	1.04149	1.54562	-0.56166
C	1.98640	0.79782	0.27562
H	2.93059	1.36699	0.33690
H	1.60434	0.69716	1.30227
C	2.31301	-0.56421	-0.33671
H	2.77135	-0.42554	-1.32503
H	3.04989	-1.06416	0.32130
O	1.17098	-1.44681	-0.60032
C	0.32861	-1.77540	0.55826
H	0.43644	-2.85169	0.77380
H	0.67028	-1.23299	1.45276
C	-1.14830	-1.49485	0.26338
H	-1.72076	-1.80355	1.17291
H	-1.47377	-2.15865	-0.55312
C	-2.82584	0.04557	-0.64274
H	-2.96178	1.05200	-1.06283
H	-3.59135	-0.09874	0.15414
H	-3.02548	-0.68228	-1.44229

ACE1 NoMetal SCS-MP2 def2sv

25

N	-1.41017	-0.10845	-0.15589
C	-1.01590	0.93496	0.78418
H	-1.90070	1.45557	1.21162
H	-0.49840	0.49355	1.65312
C	-0.12278	1.99134	0.12497
H	-0.70241	2.52105	-0.65149
H	0.18595	2.74075	0.88682
O	1.00297	1.46414	-0.55320
C	1.95542	0.79871	0.25318
H	2.90335	1.37649	0.27469

H	1.61232	0.72207	1.30010
C	2.25593	-0.58122	-0.33061
H	2.74268	-0.45073	-1.31271
H	2.97380	-1.10581	0.33744
O	1.11277	-1.37052	-0.58947
C	0.34322	-1.74601	0.53730
H	0.46697	-2.83192	0.73104
H	0.68894	-1.22735	1.44894
C	-1.13648	-1.46895	0.27673
H	-1.70838	-1.76064	1.19360
H	-1.46854	-2.14423	-0.53390
C	-2.76945	0.05569	-0.63883
H	-2.90457	1.07859	-1.03423
H	-3.54118	-0.11186	0.15070
H	-2.96435	-0.65257	-1.46469

ACE1 NoMetal SCS-MP2 def2tzv

25

N	-1.43571	-0.10526	-0.18610
C	-1.02773	0.94824	0.77761
H	-1.90947	1.45125	1.21156
H	-0.49351	0.50053	1.62206
C	-0.15568	2.03884	0.12503
H	-0.71180	2.54727	-0.66572
H	0.14407	2.77047	0.88959
O	1.04593	1.52929	-0.56542
C	1.99542	0.79476	0.28339
H	2.92872	1.36918	0.34062
H	1.60397	0.69550	1.30010
C	2.32242	-0.57455	-0.33100
H	2.78690	-0.44247	-1.31015

H	3.02485	-1.09368	0.33766
O	1.15955	-1.43460	-0.60573
C	0.32644	-1.77745	0.55872
H	0.43912	-2.84998	0.75879
H	0.67314	-1.24012	1.44711
C	-1.15634	-1.48999	0.26675
H	-1.72511	-1.74710	1.18517
H	-1.49349	-2.16018	-0.53205
C	-2.84576	0.04584	-0.62528
H	-2.99751	1.05888	-1.00918
H	-3.56464	-0.13041	0.19684
H	-3.05173	-0.66389	-1.43175

ACE1 Rb B2PLYP def2sv

26

N	-1.28060	1.61337	-0.17545
C	-0.59568	1.02738	-1.31278
H	-1.02092	1.49395	-2.21724
H	0.49656	1.27230	-1.31906
C	-0.71015	-0.48524	-1.47955
H	-0.25999	-0.73982	-2.45882
H	-0.11230	-1.01379	-0.71948
O	-2.05457	-0.92252	-1.45368
C	-2.38218	-1.96840	-0.56512
H	-2.98658	-2.72027	-1.10495
H	-1.46833	-2.49051	-0.22990
C	-3.21979	-1.48365	0.62429
H	-4.23306	-1.23684	0.26929
H	-3.31411	-2.33011	1.33809
O	-2.76849	-0.30959	1.25999
C	-1.40064	-0.20112	1.58157

H	-1.25859	-0.29004	2.67684
H	-0.81430	-1.01430	1.13421
C	-0.87231	1.17385	1.14869
H	0.23692	1.17920	1.28290
H	-1.27338	1.91151	1.86159
C	-1.29133	3.06170	-0.28136
H	-1.71444	3.36371	-1.25371
H	-0.27572	3.51736	-0.20043
H	-1.92056	3.49632	0.51228
Rb	3.52162	-0.34007	0.13275

ACE1 Rb B3LYP def2sv

26

N	-1.28158	1.61289	-0.18088
C	-0.60134	1.01968	-1.31946
H	-1.02951	1.48363	-2.22562
H	0.49271	1.26507	-1.33332
C	-0.70951	-0.49837	-1.48520
H	-0.25219	-0.74832	-2.46417
H	-0.10870	-1.02439	-0.72303
O	-2.04903	-0.95383	-1.46843
C	-2.38823	-1.97736	-0.55754
H	-2.99960	-2.73397	-1.08639
H	-1.47846	-2.50463	-0.21291
C	-3.22807	-1.47586	0.62814
H	-4.23613	-1.21169	0.26423
H	-3.34569	-2.32797	1.33509
O	-2.76878	-0.31415	1.28192
C	-1.40008	-0.19141	1.59878
H	-1.25647	-0.26572	2.69658
H	-0.80695	-1.00784	1.16162

C	-0.87579	1.18570	1.14997
H	0.23448	1.19395	1.28869
H	-1.27864	1.93067	1.85614
C	-1.33548	3.05972	-0.30343
H	-1.77574	3.34088	-1.27660
H	-0.33254	3.55040	-0.23713
H	-1.97103	3.48821	0.49100
Rb	3.53754	-0.33263	0.13396

ACE1 Rb B97-1 def2sv

26

N	2.48630	0.67308	-0.15768
C	1.38536	1.62206	-0.31139
H	1.76543	2.66499	-0.35213
H	0.86045	1.45149	-1.27245
C	0.35807	1.52840	0.82070
H	0.84696	1.69975	1.80042
H	-0.40813	2.31687	0.68766
O	-0.32693	0.28361	0.82446
C	0.20881	-0.76840	1.62234
H	1.18650	-0.47454	2.04234
H	-0.49167	-0.96399	2.46186
C	0.37623	-2.06845	0.83319
H	0.50539	-2.88639	1.56639
H	-0.55798	-2.28113	0.26672
O	1.50183	-2.13187	-0.00955
C	1.48079	-1.36742	-1.20155
H	1.64680	-2.05170	-2.05945
H	0.49347	-0.89048	-1.34765
C	2.60270	-0.32298	-1.20990
H	2.64775	0.13026	-2.23235

H	3.55612	-0.86236	-1.06108
C	3.74373	1.29231	0.22232
H	3.59810	1.92541	1.11730
H	4.18499	1.93487	-0.57961
H	4.48604	0.51517	0.48229
Rb	-3.02858	0.22576	-0.31325

ACE1 Rb CAM-B3LYP def2sv

26

N	1.83323	1.44037	0.09938
C	0.50308	0.98985	0.45642
H	-0.17592	1.84453	0.65687
H	0.04796	0.45768	-0.39342
C	0.49337	0.08843	1.69372
H	0.82534	0.66865	2.56986
H	-0.54778	-0.24451	1.89148
O	1.36897	-1.01015	1.62449
C	1.11899	-1.96896	0.62875
H	0.81933	-2.92938	1.09260
H	0.27990	-1.66197	-0.01862
C	2.37789	-2.22227	-0.19524
H	3.14009	-2.68019	0.45539
H	2.13586	-2.95495	-0.99304
O	2.98394	-1.06852	-0.71596
C	2.25094	-0.30346	-1.64013
H	2.70060	-0.39929	-2.64738
H	1.21538	-0.67309	-1.72958
C	2.27069	1.17217	-1.25281
H	1.68256	1.72980	-2.02164
H	3.31091	1.53017	-1.33781
C	2.14611	2.77041	0.56016

H	1.91612	2.86613	1.63468
H	1.58080	3.56829	0.02250
H	3.22239	2.97686	0.43206
Rb	-3.69665	-0.01916	-0.24549

ACE1 Rb DSDPBEP86 def2sv

26

N	-2.08360	-1.21918	-0.11225
C	-1.05324	-1.08409	0.90306
H	-0.83498	-2.05581	1.39780
H	-0.10100	-0.78167	0.43600
C	-1.44007	-0.07783	1.98918
H	-2.31726	-0.45784	2.54092
H	-0.60204	0.01917	2.71430
O	-1.83227	1.18492	1.49670
C	-0.84104	1.92148	0.81798
H	-0.56987	2.82472	1.40328
H	0.08728	1.33356	0.70540
C	-1.36569	2.37957	-0.53998
H	-2.19153	3.09322	-0.37865
H	-0.55239	2.91846	-1.07391
O	-1.91589	1.35292	-1.33079
C	-1.03288	0.33655	-1.74865
H	-0.84149	0.42650	-2.83779
H	-0.05103	0.43484	-1.25245
C	-1.64262	-1.03595	-1.47957
H	-0.90402	-1.81010	-1.81013
H	-2.52999	-1.14793	-2.12934
C	-2.90376	-2.39690	0.06469
H	-3.29532	-2.43063	1.09710
H	-2.35212	-3.35028	-0.12241

H	-3.76891	-2.36292	-0.62177
Rb	3.43435	-0.23456	-0.01757

ACE1 Rb HSE06 def2sv

26

N	-2.12152	1.18133	0.07780
C	-1.85688	0.37607	-1.10355
H	-2.19216	0.90357	-2.02001
H	-0.76965	0.22323	-1.21880
C	-2.54868	-0.97990	-1.06210
H	-3.63166	-0.83360	-0.90965
H	-2.41371	-1.49496	-2.03675
O	-2.11923	-1.80457	-0.00609
C	-0.85013	-2.38989	-0.15096
H	-0.94464	-3.48892	-0.04521
H	-0.43334	-2.19345	-1.15773
C	0.12059	-1.89369	0.90316
H	-0.26901	-2.12483	1.91194
H	1.07786	-2.43424	0.78869
O	0.38256	-0.51197	0.78661
C	-0.35129	0.36268	1.62860
H	-1.15691	-0.19061	2.13964
H	0.33600	0.76574	2.39851
C	-0.96703	1.52700	0.87146
H	-0.17143	2.03966	0.27600
H	-1.28869	2.25370	1.63796
C	-3.01280	2.28777	-0.17043
H	-3.93498	1.92799	-0.65894
H	-2.57176	3.07763	-0.82512
H	-3.30781	2.76331	0.78118
Rb	2.89765	0.36023	-0.36075

ACE1 Rb M062X def2sv

26

N	-1.27964	1.62029	-0.16359
C	-0.59490	1.04832	-1.30444
H	-1.02363	1.51825	-2.20615
H	0.49495	1.29717	-1.30642
C	-0.71503	-0.46073	-1.46817
H	-0.27397	-0.72494	-2.44854
H	-0.11569	-0.98816	-0.70672
O	-2.05908	-0.87296	-1.42300
C	-2.38401	-1.95485	-0.58957
H	-2.98330	-2.68773	-1.15900
H	-1.46876	-2.48509	-0.26961
C	-3.22197	-1.50171	0.61161
H	-4.24508	-1.27853	0.27002
H	-3.28531	-2.34480	1.33065
O	-2.77880	-0.32124	1.22640
C	-1.41831	-0.22472	1.55597
H	-1.28071	-0.33165	2.64950
H	-0.83705	-1.03736	1.09792
C	-0.87651	1.15093	1.14827
H	0.23235	1.14361	1.27898
H	-1.26995	1.87907	1.87599
C	-1.25468	3.06707	-0.23972
H	-1.65560	3.39766	-1.21200
H	-0.22834	3.49076	-0.13508
H	-1.88430	3.50028	0.55420
Rb	3.52101	-0.34818	0.13028

ACE1 Rb M06 def2sv

26

N	-1.86305	1.42446	-0.08376
C	-0.52816	1.00895	-0.45668
H	0.13167	1.88522	-0.65203
H	-0.04479	0.48193	0.38766
C	-0.51151	0.12735	-1.69944
H	-0.86795	0.71558	-2.56661
H	0.53898	-0.17572	-1.91885
O	-1.35638	-0.99102	-1.63512
C	-1.07592	-1.94460	-0.64744
H	-0.74404	-2.89744	-1.11410
H	-0.23595	-1.61961	-0.00032
C	-2.31751	-2.23865	0.17756
H	-3.07254	-2.71536	-0.47476
H	-2.05149	-2.97771	0.96755
O	-2.95000	-1.11062	0.71306
C	-2.22745	-0.34028	1.63598
H	-2.66061	-0.45764	2.65255
H	-1.17815	-0.69010	1.71587
C	-2.28378	1.13183	1.26576
H	-1.70482	1.70114	2.03968
H	-3.33595	1.46742	1.36223
C	-2.20071	2.75073	-0.52269
H	-1.97903	2.86657	-1.60078
H	-1.64371	3.55420	0.02311
H	-3.28236	2.93998	-0.38628
Rb	3.68901	-0.00564	0.24350

ACE1 Rb MP2 def2sv

26

N	1.59863	-1.57031	0.03940
C	1.07115	-1.31011	-1.28582

H	1.77367	-1.76947	-2.00374
H	0.07921	-1.80465	-1.45178
C	0.91394	0.15417	-1.67385
H	0.69314	0.18788	-2.76041
H	0.04329	0.60716	-1.17122
O	2.08939	0.88955	-1.40255
C	1.93205	2.09734	-0.69037
H	2.46698	2.90788	-1.22207
H	0.86782	2.39626	-0.66220
C	2.52487	1.99722	0.71724
H	3.62504	2.00583	0.64304
H	2.21896	2.90207	1.28762
O	2.22299	0.80876	1.41168
C	0.87648	0.39745	1.43783
H	0.43765	0.58188	2.44087
H	0.26143	0.96980	0.72984
C	0.80180	-1.10414	1.15910
H	-0.27426	-1.39990	1.07398
H	1.19316	-1.61223	2.05718
C	1.90135	-2.98395	0.17865
H	2.57632	-3.30343	-0.63362
H	0.99263	-3.63113	0.14153
H	2.41247	-3.16778	1.13881
Rb	-3.38343	0.16345	0.02667

ACE1 Rb PBE0 def2sv

26

N	-2.28502	1.36158	0.14914
C	-1.43707	1.29348	-1.00983
H	-1.53009	2.20994	-1.63849
H	-0.38684	1.28051	-0.67075

C	-1.69211	0.12277	-1.97430
H	-2.64367	0.28994	-2.50549
H	-0.89031	0.12496	-2.74410
O	-1.82913	-1.14024	-1.38146
C	-0.69589	-1.68023	-0.76038
H	-0.31572	-2.54791	-1.33820
H	0.12665	-0.94264	-0.72743
C	-1.05068	-2.15866	0.64080
H	-1.77703	-2.98512	0.56339
H	-0.13525	-2.56324	1.12441
O	-1.67239	-1.18600	1.43463
C	-0.91028	-0.04835	1.74132
H	-0.62146	-0.05981	2.81325
H	0.03425	-0.03903	1.16677
C	-1.70252	1.22799	1.45831
H	-1.02337	2.08074	1.67829
H	-2.52514	1.29799	2.19044
C	-3.67739	1.06051	-0.03026
H	-3.89743	-0.01629	-0.19068
H	-4.07942	1.61842	-0.89881
H	-4.24773	1.39422	0.85432
Rb	3.64636	0.24409	-0.04163

ACE1 Rb PBE def2sv

26

N	1.26240	1.61705	0.19061
C	0.58909	1.00440	1.32428
H	1.02130	1.46423	2.24152
H	-0.51619	1.24730	1.34959
C	0.70006	-0.51736	1.47421
H	0.22173	-0.77749	2.45139

H 0.10538 -1.04179 0.69183

O 2.04389 -0.97466 1.47496

C 2.38935 -1.97946 0.53891

H 3.00350 -2.75495 1.05650

H 1.47413 -2.50614 0.17615

C 3.23751 -1.45208 -0.63396

H 4.24903 -1.18616 -0.25471

H 3.36882 -2.30245 -1.35648

O 2.77917 -0.27642 -1.27192

C 1.40662 -0.17482 -1.59964

H 1.26451 -0.24801 -2.70742

H 0.81705 -1.00754 -1.16382

C 0.86226 1.19554 -1.14482

H -0.25722 1.19221 -1.29173

H 1.26180 1.95761 -1.84832

C 1.28771 3.06434 0.32715

H 1.72814 3.34616 1.30954

H 0.26715 3.54189 0.26915

H 1.91668 3.51623 -0.47104

Rb -3.51845 -0.34041 -0.13858

ACE1 Rb SCS-MP2 def2sv

26

N 2.10041 1.22147 -0.10407

C 1.04625 1.09026 0.89458

H 0.82217 2.06420 1.38314

H 0.10045 0.78841 0.41330

C 1.41593 0.08744 1.99292

H 2.28211 0.47320 2.55868

H 0.56472 -0.01181 2.70279

O 1.82472 -1.18000 1.51207

C	0.84033	-1.92510	0.82270
H	0.56573	-2.82595	1.41118
H	-0.08801	-1.34090	0.69410
C	1.38867	-2.38949	-0.52614
H	2.21302	-3.10126	-0.34694
H	0.58578	-2.92995	-1.07454
O	1.95525	-1.36173	-1.31275
C	1.07189	-0.34815	-1.75235
H	0.90308	-0.44572	-2.84527
H	0.08052	-0.44725	-1.27549
C	1.67543	1.02919	-1.48044
H	0.93935	1.80088	-1.82244
H	2.57133	1.13843	-2.11968
C	2.89109	2.42676	0.06922
H	3.26019	2.48486	1.10902
H	2.32015	3.36215	-0.14558
H	3.77072	2.40020	-0.59937
Rb	-3.45436	0.23104	-0.02789

ACE2 Cs B2PLYP def2sv

33

O	0.32634	0.72486	1.70963
O	0.31082	1.35140	-1.25930
O	2.47561	0.01599	-0.12716
N	-2.16650	0.60110	0.04735
C	-2.07070	1.03350	1.43996
C	-2.02316	1.67548	-0.94296
C	-0.73869	1.64930	1.83649
C	-0.91569	1.41287	-1.95344
C	1.59468	1.34766	1.75418
C	1.44376	1.05806	-2.04173

C 2.67256 0.42007 1.21949

C 2.65183 1.01393 -1.12466

H -2.25522 0.15347 2.07977

H -2.85924 1.77799 1.69621

H -1.80830 2.63303 -0.44216

H -2.97001 1.82548 -1.49487

H -0.52555 2.53739 1.21578

H -0.82052 1.99734 2.88556

H -1.10425 0.46355 -2.49642

H -0.89940 2.22669 -2.70663

H 1.86030 1.62890 2.79358

H 1.57013 2.27770 1.15823

H 1.60766 1.82840 -2.82212

H 1.32205 0.08449 -2.56090

H 3.65357 0.91736 1.34028

H 2.69718 -0.50945 1.81027

H 3.55305 0.78219 -1.72058

H 2.79633 2.00583 -0.66581

C -3.36249 -0.19747 -0.18305

H -3.37888 -1.06490 0.49835

H -3.36606 -0.57900 -1.21719

H -4.30276 0.37650 -0.02544

Cs -0.00035 -1.79617 -0.04124

ACE2 Cs B2PLYP def2tzv

33

O -0.36657 -0.54573 1.80539

O -0.33035 -1.51306 -1.16858

O -2.50719 0.06003 -0.17718

N 2.18045 -0.58125 0.08593

C 2.04799 -0.94320 1.51859

C	2.05277	-1.73486	-0.84636
C	0.70512	-1.54288	1.90579
C	0.94789	-1.54665	-1.87850
C	-1.68969	-1.17027	1.84331
C	-1.48108	-1.19394	-2.00692
C	-2.72804	-0.23927	1.24283
C	-2.69294	-1.06574	-1.10465
H	2.21728	-0.03917	2.10666
H	2.82291	-1.67069	1.81480
H	1.85688	-2.65449	-0.29201
H	2.99545	-1.88855	-1.38288
H	0.45314	-2.38480	1.26001
H	0.76566	-1.90270	2.93720
H	1.09147	-0.61052	-2.42855
H	0.95417	-2.37515	-2.59397
H	-1.96899	-1.38934	2.87867
H	-1.65197	-2.11032	1.28996
H	-1.65425	-1.98739	-2.74058
H	-1.30571	-0.25765	-2.54652
H	-3.71807	-0.68288	1.37898
H	-2.71199	0.72923	1.73847
H	-3.57963	-0.86841	-1.70846
H	-2.84561	-1.99320	-0.55252
C	3.42528	0.18553	-0.15316
H	3.43655	1.07605	0.47540
H	3.46636	0.49989	-1.19525
H	4.32752	-0.40402	0.06785
Cs	0.03445	1.75419	-0.14384

ACE2 Cs B3LYP def2sv

33

O	0.30680	0.65858	1.74225
O	0.29449	1.47285	-1.21794
O	2.48067	0.08296	-0.13572
N	-2.19377	0.59512	0.05482
C	-2.10402	0.92730	1.47752
C	-2.05044	1.73185	-0.86654
C	-0.78460	1.54152	1.93216
C	-0.93947	1.54719	-1.89749
C	1.56527	1.29904	1.81886
C	1.43148	1.21471	-2.00567
C	2.66392	0.42187	1.23162
C	2.64325	1.12718	-1.08918
H	-2.27286	-0.00249	2.05020
H	-2.90891	1.63439	1.79018
H	-1.84032	2.65610	-0.30274
H	-2.99817	1.91582	-1.40915
H	-0.58989	2.48563	1.38924
H	-0.88403	1.80282	3.00638
H	-1.11357	0.62630	-2.49485
H	-0.94711	2.40391	-2.60464
H	1.83019	1.52648	2.87348
H	1.52376	2.26326	1.27698
H	1.60200	2.02034	-2.75125
H	1.31183	0.26573	-2.57242
H	3.63521	0.93329	1.38320
H	2.70993	-0.53822	1.77363
H	3.54288	0.92604	-1.70151
H	2.79003	2.09847	-0.58504
C	-3.37076	-0.21191	-0.24087
H	-3.37582	-1.12647	0.37898

H	-3.36063	-0.52359	-1.29980
H	-4.32799	0.32857	-0.05286
Cs	0.03721	-1.83945	-0.10551

ACE2 Cs B3LYP def2tzv

33

O	-0.35156	-0.48370	1.82334
O	-0.32561	-1.62742	-1.14138
O	-2.49591	0.01078	-0.18019
N	2.18871	-0.58729	0.09397
C	2.06999	-0.86118	1.54552
C	2.05520	-1.78531	-0.77714
C	0.73835	-1.44578	1.99279
C	0.95990	-1.65476	-1.82901
C	-1.66534	-1.11630	1.89517
C	-1.47418	-1.30734	-1.97382
C	-2.71517	-0.23156	1.24702
C	-2.68014	-1.14216	-1.06978
H	2.23944	0.07812	2.07522
H	2.85413	-1.55998	1.88411
H	1.84721	-2.67035	-0.17409
H	3.00056	-1.98050	-1.29499
H	0.49743	-2.34585	1.42481
H	0.81687	-1.72020	3.04932
H	1.09378	-0.73796	-2.41310
H	0.99777	-2.50709	-2.51596
H	-1.94407	-1.28232	2.94110
H	-1.62184	-2.08673	1.39645
H	-1.66849	-2.11107	-2.69205
H	-1.29132	-0.38631	-2.53758
H	-3.69897	-0.68286	1.40406

H	-2.71902	0.75715	1.70238
H	-3.56859	-0.96537	-1.67843
H	-2.83481	-2.05116	-0.48801
C	3.41039	0.18753	-0.20943
H	3.41924	1.11256	0.36787
H	3.43025	0.44813	-1.26720
H	4.33130	-0.36865	0.02464
Cs	0.01253	1.78391	-0.19652

ACE2 Cs B97-1 def2sv

33

O	0.31843	0.71197	1.72866
O	0.32398	1.39216	-1.26071
O	2.49039	0.02414	-0.12420
N	-2.17521	0.62078	0.04542
C	-2.08634	1.01951	1.45178
C	-2.01571	1.71204	-0.92786
C	-0.75092	1.62551	1.87982
C	-0.90410	1.45887	-1.94811
C	1.58752	1.32948	1.78179
C	1.45871	1.09841	-2.03676
C	2.67251	0.40764	1.22905
C	2.66936	1.03061	-1.11122
H	-2.27846	0.12077	2.06846
H	-2.87653	1.76055	1.72604
H	-1.78956	2.65951	-0.40653
H	-2.96161	1.88519	-1.48052
H	-0.53785	2.54136	1.29264
H	-0.84421	1.93762	2.94272
H	-1.09492	0.51291	-2.50410
H	-0.89410	2.28353	-2.69467

H	1.85776	1.59192	2.82857
H	1.56597	2.27455	1.20204
H	1.63870	1.87857	-2.80892
H	1.33239	0.13030	-2.57308
H	3.65454	0.90530	1.36961
H	2.69489	-0.53483	1.80585
H	3.56975	0.79736	-1.71372
H	2.82509	2.02000	-0.64194
C	-3.36220	-0.18470	-0.21734
H	-3.38259	-1.06695	0.45035
H	-3.34750	-0.55089	-1.26052
H	-4.31435	0.37808	-0.06466
Cs	-0.00484	-1.81601	-0.05841

ACE2 Cs B97-1 def2tzv

33

O	-0.35434	-0.54204	1.80531
O	-0.35270	-1.55634	-1.17464
O	-2.51567	0.05588	-0.16369
N	2.18094	-0.60271	0.07835
C	2.06396	-0.93550	1.51875
C	2.03154	-1.76542	-0.83847
C	0.72109	-1.52075	1.94430
C	0.92362	-1.58180	-1.87502
C	-1.67038	-1.16603	1.85580
C	-1.49784	-1.21164	-1.99887
C	-2.71999	-0.24245	1.25258
C	-2.70786	-1.06329	-1.08948
H	2.24738	-0.01845	2.08716
H	2.83984	-1.66117	1.82620
H	1.82352	-2.67572	-0.26884

H	2.97143	-1.94432	-1.37685
H	0.47285	-2.40116	1.34358
H	0.79634	-1.83365	2.99319
H	1.06870	-0.64506	-2.43021
H	0.94260	-2.41239	-2.59276
H	-1.94799	-1.37690	2.89672
H	-1.63852	-2.11562	1.31152
H	-1.69675	-1.99775	-2.73888
H	-1.31126	-0.27494	-2.54100
H	-3.70807	-0.69264	1.40452
H	-2.70735	0.72858	1.75028
H	-3.59350	-0.85773	-1.69760
H	-2.87375	-1.99301	-0.53910
C	3.41802	0.16101	-0.19445
H	3.44381	1.06213	0.42353
H	3.43997	0.46633	-1.24275
H	4.33019	-0.42327	0.01435
Cs	0.04078	1.77420	-0.15197

ACE2 Cs CAM-B3LYP def2sv

33

O	-0.31924	-0.72398	1.70570
O	-0.30469	-1.34267	-1.26076
O	-2.46920	-0.02842	-0.12331
N	2.17374	-0.59423	0.04655
C	2.07611	-1.01252	1.43903
C	2.02500	-1.66615	-0.93811
C	0.74978	-1.63243	1.84050
C	0.91803	-1.40957	-1.94791
C	-1.58028	-1.34628	1.75391
C	-1.43729	-1.06255	-2.03664

C	-2.66204	-0.42686	1.21892
C	-2.64312	-1.01836	-1.11959
H	2.25420	-0.12587	2.07132
H	2.86926	-1.74794	1.70403
H	1.80984	-2.62118	-0.43321
H	2.97041	-1.82064	-1.49023
H	0.54393	-2.52887	1.22866
H	0.83421	-1.97094	2.89223
H	1.10677	-0.46436	-2.49846
H	0.90228	-2.22773	-2.69604
H	-1.84322	-1.62640	2.79405
H	-1.55360	-2.27841	1.16057
H	-1.59916	-1.83813	-2.81203
H	-1.32201	-0.09185	-2.56327
H	-3.64056	-0.92794	1.34287
H	-2.69148	0.50299	1.80951
H	-3.54397	-0.78743	-1.71623
H	-2.78817	-2.01132	-0.66250
C	3.35461	0.21513	-0.19253
H	3.36638	1.08729	0.48345
H	3.35304	0.59241	-1.22848
H	4.30213	-0.34692	-0.03524
Cs	-0.01103	1.78747	-0.04283

ACE2 Cs CAM-B3LYP def2tzv

33

O	-0.35212	-0.54241	1.79469
O	-0.32910	-1.50864	-1.17606
O	-2.48510	0.04495	-0.16565
N	2.17645	-0.58055	0.08459
C	2.05323	-0.91566	1.51338

C	2.03678	-1.72703	-0.83585
C	0.72218	-1.51166	1.92033
C	0.94197	-1.53947	-1.86872
C	-1.66064	-1.16285	1.84148
C	-1.47446	-1.19062	-1.99634
C	-2.70036	-0.24440	1.24219
C	-2.67527	-1.06271	-1.09082
H	2.22147	-0.00327	2.08630
H	2.83373	-1.62966	1.82013
H	1.82823	-2.63882	-0.27681
H	2.97847	-1.89965	-1.36515
H	0.48007	-2.37626	1.30254
H	0.79009	-1.84334	2.95937
H	1.09005	-0.60580	-2.41997
H	0.95643	-2.36504	-2.58566
H	-1.93588	-1.37836	2.87734
H	-1.62506	-2.10649	1.29564
H	-1.65480	-1.97942	-2.73159
H	-1.30488	-0.25531	-2.53783
H	-3.68786	-0.68786	1.38667
H	-2.68754	0.72292	1.73845
H	-3.56459	-0.86533	-1.68881
H	-2.82612	-1.99303	-0.54503
C	3.38880	0.20478	-0.18318
H	3.39961	1.10056	0.43636
H	3.40751	0.51489	-1.22631
H	4.30747	-0.36206	0.02278
Cs	0.02356	1.73930	-0.14580

ACE2 Cs DSDPBEP86 def2tzv

33

O	-0.36503	-0.66981	1.76550
O	-0.32688	-1.33712	-1.23825
O	-2.51155	0.11426	-0.15135
N	2.17161	-0.55681	0.06257
C	2.04095	-1.05390	1.45175
C	2.05280	-1.62127	-0.96779
C	0.69293	-1.68287	1.76643
C	0.93943	-1.33720	-1.96762
C	-1.68768	-1.29093	1.74345
C	-1.47820	-0.98763	-2.06223
C	-2.72272	-0.30062	1.23857
C	-2.69566	-0.93687	-1.15855
H	2.20860	-0.20804	2.12699
H	2.81638	-1.81080	1.67573
H	1.86582	-2.59322	-0.49917
H	2.99630	-1.71586	-1.52315
H	0.43744	-2.44899	1.02840
H	0.74232	-2.15361	2.75646
H	1.09303	-0.35807	-2.44310
H	0.92019	-2.10712	-2.74977
H	-1.96733	-1.61293	2.75488
H	-1.65206	-2.17293	1.09568
H	-1.63184	-1.73821	-2.84779
H	-1.31601	-0.01210	-2.54046
H	-3.71914	-0.74554	1.34629
H	-2.68864	0.62442	1.81690
H	-3.58385	-0.69652	-1.75000
H	-2.84632	-1.90801	-0.67876
C	3.41376	0.23102	-0.10065
H	3.41694	1.06354	0.60839

H	3.45655	0.63871	-1.11361
H	4.31876	-0.37597	0.07107
Cs	0.04047	1.69549	-0.04073

ACE2 Cs DSDPBEP86 def2sv

33

O	-0.33505	-0.80564	1.66159
O	-0.33668	-1.22879	-1.30115
O	-2.47653	0.06097	-0.10086
N	2.14127	-0.60061	0.02567
C	2.04897	-1.12588	1.38296
C	1.99014	-1.60566	-1.02993
C	0.71095	-1.75369	1.72749
C	0.88186	-1.25787	-2.00916
C	-1.60711	-1.41679	1.66203
C	-1.46039	-0.87213	-2.06870
C	-2.67164	-0.43394	1.21281
C	-2.66985	-0.86853	-1.15583
H	2.23691	-0.29081	2.08320
H	2.83490	-1.89198	1.58534
H	1.76638	-2.59440	-0.59333
H	2.93624	-1.72621	-1.59437
H	0.48901	-2.59054	1.03878
H	0.77945	-2.18016	2.75024
H	1.08078	-0.27110	-2.48188
H	0.84546	-2.01360	-2.82215
H	-1.87020	-1.78195	2.67760
H	-1.59525	-2.29394	0.98708
H	-1.62831	-1.59007	-2.89889
H	-1.32245	0.13309	-2.52411
H	-3.66373	-0.91925	1.30621

H	-2.66969	0.45125	1.87184
H	-3.56798	-0.58510	-1.73710
H	-2.83072	-1.88665	-0.75942
C	3.34313	0.19872	-0.15415
H	3.36566	1.02308	0.58176
H	3.35040	0.64395	-1.16488
H	4.27852	-0.39429	-0.03198
Cs	0.03610	1.73755	0.03754

ACE2 Cs HSE06 def2sv

33

O	-0.32349	-0.73077	1.70064
O	-0.30975	-1.32664	-1.26716
O	-2.47068	-0.01022	-0.12292
N	2.16552	-0.60225	0.04687
C	2.06640	-1.03550	1.43116
C	2.01435	-1.65990	-0.94839
C	0.73638	-1.64740	1.82560
C	0.91003	-1.38865	-1.95508
C	-1.58560	-1.34556	1.74327
C	-1.43981	-1.03771	-2.03922
C	-2.66146	-0.41842	1.21380
C	-2.64365	-0.99459	-1.12166
H	2.25427	-0.15697	2.07464
H	2.85356	-1.78200	1.69100
H	1.79417	-2.62118	-0.45344
H	2.96080	-1.81502	-1.50161
H	0.52454	-2.53775	1.20288
H	0.81983	-2.00097	2.87397
H	1.10322	-0.43735	-2.49702
H	0.89438	-2.19868	-2.71411

H	-1.85373	-1.63287	2.78168
H	-1.56454	-2.27670	1.14413
H	-1.60831	-1.80789	-2.82066
H	-1.32250	-0.06404	-2.56353
H	-3.64346	-0.91570	1.33926
H	-2.68675	0.50869	1.81139
H	-3.54543	-0.76176	-1.71846
H	-2.79058	-1.99121	-0.66909
C	3.34195	0.20927	-0.18447
H	3.35546	1.07256	0.50469
H	3.33581	0.60307	-1.21548
H	4.29385	-0.35190	-0.03990
Cs	0.00121	1.77882	-0.03333

ACE2 Cs HSE06 def2tzv

33

O	-0.35467	-0.56043	1.78437
O	-0.33487	-1.46246	-1.18576
O	-2.49578	0.06977	-0.16062
N	2.16980	-0.57966	0.07861
C	2.04695	-0.94439	1.49818
C	2.02659	-1.70904	-0.86039
C	0.71241	-1.53706	1.89501
C	0.92950	-1.50329	-1.88664
C	-1.66219	-1.17998	1.82014
C	-1.47933	-1.14500	-2.00496
C	-2.70287	-0.25133	1.24004
C	-2.68069	-1.02640	-1.09906
H	2.22709	-0.04419	2.09180
H	2.82191	-1.67464	1.79177
H	1.81818	-2.63263	-0.31473

H	2.96809	-1.87628	-1.39718
H	0.46720	-2.39430	1.26202
H	0.78109	-1.88917	2.93037
H	1.08736	-0.56708	-2.43733
H	0.93473	-2.32719	-2.61008
H	-1.94094	-1.41699	2.85357
H	-1.62978	-2.11662	1.25576
H	-1.66094	-1.93199	-2.74629
H	-1.31438	-0.20506	-2.54641
H	-3.69080	-0.70149	1.38303
H	-2.69125	0.70784	1.75785
H	-3.57055	-0.82435	-1.69977
H	-2.83358	-1.96681	-0.56532
C	3.38822	0.19755	-0.17214
H	3.40712	1.08236	0.46702
H	3.41024	0.53016	-1.21072
H	4.30544	-0.38112	0.02198
Cs	0.03502	1.72556	-0.12587

ACE2 Cs M062X def2sv

33

O	-0.33533	-0.83881	1.63093
O	-0.31534	-1.21220	-1.30688
O	-2.46806	0.05155	-0.09906
N	2.15989	-0.57979	0.03301
C	2.04914	-1.14534	1.37090
C	2.02515	-1.54868	-1.05496
C	0.70641	-1.78681	1.66432
C	0.90109	-1.19294	-2.01224
C	-1.60378	-1.44726	1.63010
C	-1.43539	-0.84421	-2.06841

C	-2.66889	-0.45646	1.20365
C	-2.65303	-0.85670	-1.16697
H	2.22001	-0.33384	2.10007
H	2.83353	-1.91285	1.55820
H	1.83411	-2.55747	-0.65265
H	2.96625	-1.61972	-1.63056
H	0.49506	-2.58307	0.92665
H	0.75586	-2.26517	2.66221
H	1.07615	-0.18499	-2.44665
H	0.87498	-1.91481	-2.85236
H	-1.85612	-1.83297	2.63784
H	-1.59612	-2.30982	0.93680
H	-1.59399	-1.54379	-2.91243
H	-1.29549	0.16950	-2.50156
H	-3.66058	-0.93872	1.28801
H	-2.66301	0.41634	1.87693
H	-3.54336	-0.56485	-1.75073
H	-2.81553	-1.87992	-0.78759
C	3.34617	0.24623	-0.10773
H	3.34504	1.04537	0.65342
H	3.35709	0.72249	-1.10252
H	4.28792	-0.33397	0.00662
Cs	0.01468	1.72127	0.05832

ACE2 Cs M062X def2tzv

33

O	-0.34438	-0.71958	1.71337
O	-0.31964	-1.28880	-1.25867
O	-2.49501	0.10277	-0.12776
N	2.17491	-0.54607	0.05013
C	2.05758	-1.05462	1.42738

C	2.05028	-1.57789	-0.99901
C	0.72197	-1.70275	1.73054
C	0.93664	-1.26508	-1.98111
C	-1.64857	-1.34658	1.69005
C	-1.46365	-0.91843	-2.06273
C	-2.69181	-0.34955	1.23737
C	-2.67938	-0.89794	-1.16510
H	2.20921	-0.21649	2.11033
H	2.84318	-1.79531	1.64355
H	1.86011	-2.55574	-0.55488
H	2.98795	-1.66548	-1.55635
H	0.49146	-2.47301	0.99267
H	0.76652	-2.17138	2.71708
H	1.08914	-0.27381	-2.42326
H	0.91522	-2.00251	-2.78795
H	-1.90735	-1.70884	2.68852
H	-1.61677	-2.20105	1.01051
H	-1.61222	-1.63741	-2.87221
H	-1.30523	0.07011	-2.50707
H	-3.68321	-0.79505	1.34423
H	-2.64978	0.54910	1.85005
H	-3.56275	-0.63706	-1.74703
H	-2.83113	-1.88335	-0.72461
C	3.37536	0.28542	-0.12388
H	3.36543	1.10654	0.59293
H	3.38598	0.70803	-1.12795
H	4.30128	-0.28779	0.02092
Cs	0.01910	1.67095	-0.00285

ACE2 Cs M06 def2sv

33

O	-0.36215	-0.77580	1.65056
O	-0.34965	-1.29523	-1.28760
O	-2.48145	0.05509	-0.12413
N	2.13361	-0.66924	0.04941
C	2.00989	-1.14365	1.41760
C	1.96127	-1.68250	-0.98730
C	0.66249	-1.72897	1.77129
C	0.87006	-1.32958	-1.97461
C	-1.64385	-1.34174	1.70779
C	-1.46693	-0.94167	-2.04949
C	-2.68173	-0.36610	1.20528
C	-2.67089	-0.90485	-1.14055
H	2.21055	-0.28697	2.09308
H	2.78193	-1.91557	1.66106
H	1.71550	-2.66232	-0.53496
H	2.90929	-1.83995	-1.54271
H	0.43349	-2.59915	1.11908
H	0.71045	-2.11762	2.81266
H	1.08076	-0.33777	-2.44347
H	0.83936	-2.07692	-2.79879
H	-1.90572	-1.63486	2.74908
H	-1.66787	-2.27182	1.09987
H	-1.64433	-1.66772	-2.87388
H	-1.31849	0.05678	-2.52592
H	-3.68724	-0.82069	1.33159
H	-2.66132	0.55362	1.82039
H	-3.57137	-0.64970	-1.73465
H	-2.83481	-1.91203	-0.70864
C	3.33131	0.11627	-0.14973
H	3.37416	0.95585	0.57134

H	3.33964	0.54706	-1.16962
H	4.26794	-0.47937	-0.02569
Cs	0.07289	1.78015	0.00076

ACE2 Cs M06 def2tzv

33

O	-0.36242	-0.68327	1.75256
O	-0.32140	-1.35677	-1.26118
O	-2.47159	0.07794	-0.15642
N	2.14949	-0.60269	0.07158
C	2.02440	-1.04257	1.46682
C	2.01803	-1.66445	-0.94010
C	0.69183	-1.66631	1.81083
C	0.93869	-1.36345	-1.95816
C	-1.67273	-1.27964	1.75763
C	-1.46273	-1.00879	-2.06179
C	-2.68873	-0.29989	1.22163
C	-2.66112	-0.95881	-1.14841
H	2.18020	-0.16784	2.10620
H	2.80946	-1.77284	1.72952
H	1.78540	-2.61972	-0.46308
H	2.96846	-1.80679	-1.46863
H	0.45343	-2.47780	1.11535
H	0.74684	-2.09240	2.81928
H	1.11759	-0.38392	-2.42604
H	0.93235	-2.12119	-2.75072
H	-1.95925	-1.56567	2.77647
H	-1.65848	-2.18979	1.14783
H	-1.62968	-1.74979	-2.85225
H	-1.30840	-0.03306	-2.54229
H	-3.68957	-0.72631	1.34671

H	-2.64642	0.63351	1.78504
H	-3.55864	-0.73594	-1.72998
H	-2.79751	-1.93199	-0.66978
C	3.34130	0.22097	-0.14027
H	3.34597	1.06383	0.55468
H	3.33972	0.61827	-1.15753
H	4.27487	-0.34497	0.00581
Cs	0.04212	1.72178	-0.04648

ACE2 Cs MP2 def2tzv

33

O	0.37885	0.62657	1.79413
O	0.32085	1.40017	-1.18745
O	2.52296	-0.10421	-0.17580
N	-2.17500	0.56586	0.07455
C	-2.03183	1.03045	1.48134
C	-2.07181	1.67343	-0.92112
C	-0.67877	1.65693	1.79449
C	-0.95082	1.43515	-1.92965
C	1.71175	1.25342	1.77353
C	1.47317	1.07647	-2.04129
C	2.74236	0.27032	1.23642
C	2.70194	0.99287	-1.14987
H	-2.18803	0.16566	2.13629
H	-2.80786	1.78168	1.72788
H	-1.90164	2.63356	-0.41945
H	-3.01681	1.76778	-1.47679
H	-0.41643	2.41905	1.05474
H	-0.72250	2.12555	2.78652
H	-1.10005	0.47583	-2.44544
H	-0.92350	2.24145	-2.67484

H	1.99942	1.55270	2.79028
H	1.66666	2.14723	1.14256
H	1.61785	1.85725	-2.79932
H	1.30208	0.11695	-2.54827
H	3.74177	0.70895	1.34885
H	2.70472	-0.67454	1.78208
H	3.58446	0.77027	-1.75737
H	2.85894	1.94497	-0.63376
C	-3.43656	-0.20783	-0.09280
H	-3.43255	-1.06093	0.59149
H	-3.49636	-0.58051	-1.11850
H	-4.32992	0.40610	0.11399
Cs	-0.04460	-1.73141	-0.08138

ACE2 Cs MP2 def2sv

33

O	0.34412	0.76986	1.67418
O	0.33345	1.28651	-1.26699
O	2.46714	-0.04904	-0.11774
N	-2.13379	0.61169	0.03952
C	-2.03541	1.11385	1.40473
C	-1.99416	1.64416	-0.99067
C	-0.69416	1.72798	1.75264
C	-0.88532	1.32860	-1.97709
C	1.61946	1.37843	1.69242
C	1.45464	0.94927	-2.05055
C	2.67639	0.40599	1.20996
C	2.66396	0.91657	-1.14116
H	-2.22428	0.26987	2.09331
H	-2.81664	1.88117	1.62056
H	-1.78052	2.62547	-0.53295

H	-2.94192	1.76868	-1.55159
H	-0.46738	2.56926	1.07218
H	-0.75726	2.14392	2.78009
H	-1.08146	0.35420	-2.47398
H	-0.85218	2.10556	-2.77007
H	1.88569	1.71140	2.71810
H	1.61027	2.27510	1.04499
H	1.62197	1.69092	-2.85964
H	1.31439	-0.04162	-2.53359
H	3.67126	0.88389	1.31001
H	2.67486	-0.49737	1.84297
H	3.56279	0.65299	-1.73063
H	2.82365	1.92104	-0.71242
C	-3.34890	-0.16808	-0.14560
H	-3.37549	-1.00595	0.57319
H	-3.36920	-0.59120	-1.16463
H	-4.27377	0.43529	-0.00330
Cs	-0.03973	-1.76861	0.00285

ACE2 Cs PBE0 def2sv

33

O	-0.32371	-0.73118	1.70262
O	-0.31363	-1.33991	-1.26523
O	-2.47088	-0.01174	-0.12250
N	2.16350	-0.60891	0.04653
C	2.06509	-1.03904	1.43165
C	2.01116	-1.66846	-0.94630
C	0.73469	-1.64810	1.82940
C	0.90581	-1.39956	-1.95249
C	-1.58577	-1.34454	1.74570
C	-1.44186	-1.04518	-2.03653

C	-2.66098	-0.41763	1.21417
C	-2.64598	-0.99635	-1.11956
H	2.25470	-0.15924	2.07287
H	2.85140	-1.78599	1.69262
H	1.79228	-2.62902	-0.44950
H	2.95693	-1.82420	-1.50048
H	0.52141	-2.54052	1.21028
H	0.81941	-1.99796	2.87902
H	1.09751	-0.44794	-2.49442
H	0.89215	-2.20973	-2.71155
H	-1.85502	-1.62930	2.78461
H	-1.56549	-2.27680	1.14843
H	-1.61452	-1.81399	-2.81854
H	-1.32011	-0.07171	-2.56032
H	-3.64329	-0.91408	1.34119
H	-2.68555	0.51049	1.81033
H	-3.54625	-0.76081	-1.71775
H	-2.79744	-1.99202	-0.66639
C	3.34106	0.20013	-0.18716
H	3.35688	1.06445	0.50072
H	3.33439	0.59253	-1.21877
H	4.29223	-0.36235	-0.04284
Cs	0.00437	1.78774	-0.03596

ACE2 Cs PBE0 def2tzv

33

O	-0.35565	-0.55490	1.78527
O	-0.34039	-1.48256	-1.17979
O	-2.49651	0.06902	-0.16152
N	2.16776	-0.58634	0.07894
C	2.04470	-0.94472	1.50002

C	2.02238	-1.72023	-0.85415
C	0.70870	-1.53210	1.90088
C	0.92330	-1.51988	-1.87962
C	-1.66294	-1.17213	1.82470
C	-1.48193	-1.15862	-1.99856
C	-2.70323	-0.24564	1.23937
C	-2.68377	-1.03010	-1.09408
H	2.22776	-0.04217	2.08974
H	2.81786	-1.67618	1.79634
H	1.81564	-2.64169	-0.30366
H	2.96285	-1.88956	-1.39257
H	0.46185	-2.39316	1.27322
H	0.77817	-1.87846	2.93857
H	1.07844	-0.58362	-2.43159
H	0.93199	-2.34484	-2.60245
H	-1.94271	-1.40270	2.85973
H	-1.63237	-2.11246	1.26577
H	-1.66940	-1.94531	-2.73930
H	-1.31093	-0.22021	-2.54149
H	-3.69144	-0.69524	1.38520
H	-2.69142	0.71595	1.75344
H	-3.57188	-0.82737	-1.69790
H	-2.84178	-1.96781	-0.55642
C	3.39012	0.18379	-0.17440
H	3.41343	1.07103	0.46175
H	3.41403	0.51262	-1.21447
H	4.30463	-0.39889	0.02210
Cs	0.03941	1.73472	-0.13191

ACE2 Cs PBE def2sv

33

O	-0.32033	-0.71930	1.72701
O	-0.31451	-1.36425	-1.26613
O	-2.50146	-0.01493	-0.12575
N	2.18415	-0.61040	0.04594
C	2.08819	-1.03348	1.44594
C	2.02889	-1.68700	-0.94574
C	0.75186	-1.64393	1.85908
C	0.91672	-1.42645	-1.96264
C	-1.59242	-1.34734	1.76943
C	-1.45465	-1.07328	-2.04792
C	-2.68072	-0.42445	1.22863
C	-2.66686	-1.02348	-1.12481
H	2.28436	-0.14076	2.08233
H	2.88063	-1.78604	1.71573
H	1.80628	-2.64957	-0.43490
H	2.98135	-1.85165	-1.50418
H	0.53364	-2.55082	1.24514
H	0.84434	-1.98730	2.92041
H	1.10709	-0.47080	-2.51804
H	0.90638	-2.25078	-2.72098
H	-1.86685	-1.63252	2.81761
H	-1.56392	-2.29087	1.17216
H	-1.62897	-1.85329	-2.83274
H	-1.33450	-0.09437	-2.58304
H	-3.66644	-0.93261	1.36589
H	-2.70998	0.51700	1.82027
H	-3.57430	-0.79704	-1.73319
H	-2.81401	-2.02326	-0.65772
C	3.37044	0.20218	-0.19866
H	3.38716	1.07826	0.48816

H	3.35937	0.58970	-1.24105
H	4.33192	-0.36222	-0.05208
Cs	-0.00056	1.80533	-0.04531

ACE2 Cs PBE def2tzv

33

O	-0.36007	-0.53120	1.80607
O	-0.34966	-1.53458	-1.16596
O	-2.52889	0.07741	-0.16888
N	2.18664	-0.59377	0.08077
C	2.05909	-0.94592	1.51823
C	2.03851	-1.74981	-0.84911
C	0.71431	-1.52968	1.92809
C	0.92986	-1.56467	-1.88148
C	-1.68354	-1.16406	1.85114
C	-1.50127	-1.19866	-2.00322
C	-2.73268	-0.24045	1.25499
C	-2.71145	-1.05667	-1.09777
H	2.24926	-0.03248	2.10344
H	2.83487	-1.68488	1.82403
H	1.83578	-2.67205	-0.28218
H	2.98431	-1.92315	-1.39352
H	0.45713	-2.39885	1.30111
H	0.78405	-1.87076	2.97634
H	1.07416	-0.62336	-2.44411
H	0.94398	-2.40217	-2.60295
H	-1.96417	-1.38782	2.89681
H	-1.64661	-2.11633	1.29658
H	-1.69468	-1.99381	-2.74660
H	-1.31687	-0.25759	-2.55398
H	-3.72622	-0.69761	1.40465

H	-2.72554	0.73474	1.76029
H	-3.60407	-0.85937	-1.71185
H	-2.87335	-1.99013	-0.53860
C	3.43281	0.16437	-0.17338
H	3.46144	1.06263	0.46001
H	3.46576	0.48555	-1.22329
H	4.34667	-0.43179	0.03549
Cs	0.04441	1.76065	-0.14876

ACE2 Cs SCS-MP2 def2sv

33

O	-0.34515	-0.74843	1.69199
O	-0.32915	-1.32780	-1.26124
O	-2.46818	0.03210	-0.12624
N	2.14039	-0.61333	0.04580
C	2.04213	-1.09133	1.42480
C	2.00488	-1.66874	-0.96893
C	0.69904	-1.70354	1.78876
C	0.89522	-1.37423	-1.96732
C	-1.62301	-1.35906	1.72271
C	-1.45280	-0.99733	-2.04930
C	-2.68276	-0.39716	1.21218
C	-2.66421	-0.95242	-1.13643
H	2.22929	-0.23274	2.09687
H	2.82559	-1.85284	1.65486
H	1.79296	-2.64243	-0.49291
H	2.95499	-1.80038	-1.52556
H	0.47348	-2.56104	1.12807
H	0.76541	-2.09471	2.82647
H	1.08764	-0.40669	-2.47976
H	0.86911	-2.16594	-2.74691

H	-1.89057	-1.66371	2.75758
H	-1.61224	-2.27298	1.09936
H	-1.62190	-1.74982	-2.84897
H	-1.31147	-0.01174	-2.54386
H	-3.67792	-0.87518	1.31668
H	-2.68623	0.52043	1.82603
H	-3.56470	-0.69745	-1.72878
H	-2.82247	-1.95078	-0.69149
C	3.35886	0.16680	-0.15236
H	3.38282	1.01855	0.55174
H	3.37885	0.57225	-1.17981
H	4.28508	-0.43288	0.00185
Cs	0.03337	1.78561	-0.01860

ACE2 Cs SCS-MP2 def2tzv

33

O	0.38036	0.59976	1.80384
O	0.32458	1.45324	-1.18079
O	2.51931	-0.08765	-0.18184
N	-2.17675	0.57372	0.07996
C	-2.03602	1.00829	1.49982
C	-2.07156	1.70450	-0.89486
C	-0.67960	1.62708	1.83543
C	-0.95149	1.48427	-1.91456
C	1.71368	1.22617	1.80470
C	1.47546	1.12593	-2.03425
C	2.74738	0.25698	1.23586
C	2.70387	1.02538	-1.13708
H	-2.19613	0.12880	2.13491
H	-2.81163	1.75516	1.76132
H	-1.89568	2.65330	-0.37326

H	-3.01799	1.81474	-1.44580
H	-0.41855	2.41514	1.12259
H	-0.72671	2.06218	2.84331
H	-1.09671	0.52935	-2.44040
H	-0.93623	2.29939	-2.65161
H	2.00059	1.49042	2.83207
H	1.67237	2.14110	1.20403
H	1.62910	1.91146	-2.78661
H	1.29928	0.17092	-2.54903
H	3.74648	0.69709	1.35209
H	2.71741	-0.70000	1.76214
H	3.58916	0.81084	-1.74496
H	2.85993	1.96926	-0.60491
C	-3.44477	-0.19034	-0.10524
H	-3.44609	-1.05945	0.55996
H	-3.50593	-0.54055	-1.13966
H	-4.33591	0.42328	0.11445
Cs	-0.04410	-1.75129	-0.10507

ACE2 K B2PLYP def2sv

33

O	-0.32299	-1.68932	-0.21643
O	-0.28936	1.32179	-0.68182
O	-2.42382	0.12190	0.57268
N	2.14540	-0.04201	0.00559
C	2.07089	-1.41418	-0.49666
C	2.03122	0.99358	-1.03075
C	0.74537	-1.78915	-1.14100
C	0.94149	2.01251	-0.72941
C	-1.59605	-1.72377	-0.83171
C	-1.42949	2.09430	-0.38325

C	-2.65833	-1.20575	0.12436
C	-2.62551	1.15983	-0.37879
H	2.24965	-2.09264	0.35457
H	2.86926	-1.62640	-1.24358
H	1.80386	0.53414	-2.00539
H	2.99106	1.52838	-1.15920
H	0.53755	-1.13827	-2.00858
H	0.82690	-2.82619	-1.52223
H	1.13984	2.52313	0.23516
H	0.92485	2.78857	-1.52068
H	-1.86317	-2.75722	-1.13105
H	-1.57914	-1.10809	-1.74912
H	-1.58885	2.89006	-1.13787
H	-1.32192	2.58938	0.60375
H	-3.64725	-1.28793	-0.36397
H	-2.68118	-1.82813	1.03287
H	-3.53743	1.72778	-0.12010
H	-2.76119	0.74139	-1.38938
C	3.32238	0.15684	0.84303
H	3.31781	-0.56039	1.68033
H	3.31121	1.17326	1.26848
H	4.27522	0.02735	0.28354
K	-0.04249	-0.03917	1.92647

ACE2 K B2PLYP def2tzv

33

O	-0.34370	-1.75471	-0.26556
O	-0.29585	1.35980	-0.73519
O	-2.46328	0.12622	0.62156
N	2.17333	-0.04031	0.02126
C	2.07392	-1.40183	-0.56310

C	2.07988	1.05464	-0.98417
C	0.74764	-1.70805	-1.24434
C	0.98464	2.06315	-0.65963
C	-1.65952	-1.68750	-0.90231
C	-1.45499	2.14200	-0.31811
C	-2.70050	-1.22790	0.10444
C	-2.65919	1.22011	-0.34158
H	2.23486	-2.12044	0.24240
H	2.86710	-1.57021	-1.31117
H	1.88650	0.64438	-1.97658
H	3.03297	1.59160	-1.04738
H	0.50893	-0.95137	-1.99284
H	0.82474	-2.67755	-1.74514
H	1.12332	2.47564	0.34527
H	1.00098	2.88628	-1.38077
H	-1.94343	-2.67467	-1.27971
H	-1.60912	-0.99655	-1.74594
H	-1.62196	2.97960	-1.00211
H	-1.29631	2.54282	0.68795
H	-3.68827	-1.28077	-0.36064
H	-2.69586	-1.86801	0.98425
H	-3.55355	1.77508	-0.05541
H	-2.80159	0.82084	-1.34591
C	3.38991	0.08781	0.85829
H	3.37603	-0.66648	1.64440
H	3.40860	1.07109	1.32619
H	4.31304	-0.03612	0.27250
K	-0.02991	-0.14569	1.94360

ACE2 K B3LYP def2sv

33

O	-0.32151	-1.69925	-0.22472
O	-0.28928	1.34542	-0.66989
O	-2.43777	0.11962	0.56311
N	2.16022	-0.03865	-0.00069
C	2.08270	-1.41702	-0.49334
C	2.03246	0.99955	-1.03613
C	0.75698	-1.80777	-1.13856
C	0.94578	2.02771	-0.72981
C	-1.59607	-1.73514	-0.83948
C	-1.43561	2.10934	-0.37420
C	-2.66525	-1.20929	0.11168
C	-2.63268	1.16889	-0.37971
H	2.26577	-2.08861	0.36418
H	2.88176	-1.63788	-1.23942
H	1.79396	0.53786	-2.00844
H	2.99193	1.53417	-1.17827
H	0.55203	-1.17335	-2.02122
H	0.84734	-2.85162	-1.50342
H	1.15359	2.54654	0.23028
H	0.92983	2.79973	-1.52737
H	-1.86597	-2.77171	-1.13186
H	-1.57794	-1.12769	-1.76456
H	-1.59788	2.90860	-1.12718
H	-1.33517	2.60416	0.61589
H	-3.65191	-1.29502	-0.38416
H	-2.69636	-1.83136	1.02243
H	-3.54569	1.73700	-0.11794
H	-2.76829	0.75977	-1.39598
C	3.33097	0.16951	0.84519
H	3.32877	-0.54831	1.68423

H	3.31139	1.18655	1.27312
H	4.29157	0.04784	0.29286
K	-0.05039	-0.05406	1.93577

ACE2 K B3LYP def2tzv

33

O	-0.32644	-1.76235	-0.25577
O	-0.31173	1.38249	-0.74126
O	-2.46616	0.11220	0.61086
N	2.17673	-0.02793	0.01185
C	2.09613	-1.40426	-0.53595
C	2.05627	1.05169	-1.00467
C	0.77919	-1.75851	-1.21440
C	0.97195	2.07217	-0.67564
C	-1.63536	-1.70492	-0.90021
C	-1.47281	2.14691	-0.31364
C	-2.68839	-1.24027	0.09183
C	-2.66826	1.21317	-0.33938
H	2.26736	-2.09802	0.28911
H	2.89440	-1.58821	-1.27521
H	1.83367	0.62477	-1.98321
H	3.00840	1.58426	-1.10842
H	0.54862	-1.04926	-2.01107
H	0.87443	-2.75244	-1.66212
H	1.12221	2.49363	0.32421
H	0.99506	2.89183	-1.40141
H	-1.91555	-2.69682	-1.26976
H	-1.58369	-1.02516	-1.75330
H	-1.65551	2.98938	-0.98875
H	-1.31620	2.54604	0.69415
H	-3.67071	-1.30316	-0.38422

H	-2.69426	-1.88070	0.97224
H	-3.56647	1.76010	-0.04763
H	-2.81160	0.82320	-1.34741
C	3.37461	0.14409	0.86169
H	3.37540	-0.60013	1.65806
H	3.36279	1.13193	1.32113
H	4.31187	0.04076	0.29314
K	-0.03488	-0.14082	1.94048

ACE2 K B97-1 def2sv

33

O	-0.31558	-1.69840	-0.22649
O	-0.29763	1.33874	-0.68869
O	-2.43698	0.11379	0.56864
N	2.15535	-0.03502	-0.00133
C	2.08737	-1.41500	-0.49246
C	2.02864	0.99997	-1.04102
C	0.76010	-1.80919	-1.13935
C	0.93392	2.02448	-0.73776
C	-1.58685	-1.73124	-0.84284
C	-1.43861	2.10183	-0.37897
C	-2.65910	-1.21237	0.11408
C	-2.63918	1.16047	-0.37193
H	2.27070	-2.08515	0.36844
H	2.88939	-1.63383	-1.23827
H	1.79444	0.53431	-2.01468
H	2.98778	1.53931	-1.18039
H	0.55663	-1.17461	-2.02508
H	0.85199	-2.85525	-1.50286
H	1.13550	2.54023	0.22763
H	0.92129	2.80139	-1.53310

H	-1.85659	-2.76719	-1.14373
H	-1.56957	-1.11426	-1.76427
H	-1.61001	2.90405	-1.12928
H	-1.32734	2.59513	0.61282
H	-3.64755	-1.30190	-0.38141
H	-2.68478	-1.83921	1.02372
H	-3.54976	1.73127	-0.10141
H	-2.78517	0.75127	-1.38887
C	3.32362	0.18112	0.84754
H	3.32226	-0.53691	1.68856
H	3.29535	1.20035	1.27413
H	4.28754	0.06421	0.29708
K	-0.04355	-0.04956	1.94468

ACE2 K B97-1 def2tzv

33

O	-0.32608	-1.75822	-0.25922
O	-0.31456	1.37577	-0.75253
O	-2.47037	0.10944	0.61663
N	2.17349	-0.02718	0.01434
C	2.09625	-1.40195	-0.54026
C	2.05809	1.05296	-1.00384
C	0.77431	-1.75193	-1.21966
C	0.96396	2.06945	-0.67780
C	-1.63149	-1.69764	-0.90477
C	-1.47037	2.14012	-0.31688
C	-2.68887	-1.23848	0.09189
C	-2.67192	1.20692	-0.33386
H	2.26812	-2.10081	0.28393
H	2.89502	-1.58144	-1.28365
H	1.84409	0.62579	-1.98737

H	3.01031	1.59197	-1.09829
H	0.54456	-1.03523	-2.01423
H	0.86894	-2.74566	-1.67454
H	1.10956	2.48787	0.32732
H	0.98924	2.89429	-1.40161
H	-1.91337	-2.68907	-1.28195
H	-1.58037	-1.00950	-1.75515
H	-1.65859	2.98542	-0.99113
H	-1.30715	2.53920	0.69300
H	-3.67233	-1.30290	-0.38771
H	-2.69284	-1.88617	0.97022
H	-3.56772	1.76098	-0.03875
H	-2.82193	0.81587	-1.34359
C	3.37782	0.14119	0.85788
H	3.37996	-0.60553	1.65529
H	3.36959	1.13123	1.31850
H	4.31331	0.03559	0.28251
K	-0.02902	-0.14008	1.95114

ACE2 K CAM-B3LYP def2sv

33

O	-0.32099	-1.68879	-0.20472
O	-0.28903	1.32130	-0.66470
O	-2.41556	0.12149	0.56998
N	2.14528	-0.04324	0.00251
C	2.06935	-1.41764	-0.48105
C	2.02242	0.98298	-1.03504
C	0.74612	-1.80264	-1.11951
C	0.93720	2.00509	-0.73683
C	-1.59081	-1.73238	-0.81160
C	-1.42992	2.08950	-0.38904

C	-2.65123	-1.20459	0.13799
C	-2.62118	1.15187	-0.37899
H	2.24978	-2.08593	0.37770
H	2.86767	-1.63892	-1.22433
H	1.78730	0.51570	-2.00380
H	2.98137	1.51532	-1.17605
H	0.53973	-1.16610	-1.99850
H	0.82860	-2.84503	-1.48487
H	1.14561	2.53181	0.21717
H	0.91273	2.76889	-1.53929
H	-1.85731	-2.76933	-1.09812
H	-1.57688	-1.12839	-1.73727
H	-1.58640	2.87250	-1.15709
H	-1.33161	2.60162	0.59047
H	-3.64012	-1.29079	-0.34933
H	-2.67659	-1.82007	1.05132
H	-3.53496	1.71810	-0.12337
H	-2.75601	0.72893	-1.38815
C	3.30793	0.16858	0.84812
H	3.30239	-0.54200	1.69142
H	3.28957	1.18771	1.26744
H	4.26687	0.04089	0.29877
K	-0.04017	-0.01941	1.89260

ACE2 K CAM-B3LYP def2tzv

33

O	-0.32806	-1.75059	-0.22935
O	-0.30434	1.34904	-0.72526
O	-2.44695	0.12009	0.61174
N	2.16600	-0.03603	0.01436
C	2.07916	-1.40848	-0.51661

C	2.04958	1.02798	-1.00428
C	0.76409	-1.75413	-1.18639
C	0.96707	2.04192	-0.68478
C	-1.63092	-1.70512	-0.86212
C	-1.46135	2.12306	-0.33876
C	-2.67342	-1.23221	0.12636
C	-2.65204	1.19416	-0.34993
H	2.24459	-2.09660	0.31262
H	2.87621	-1.60036	-1.25190
H	1.83111	0.59435	-1.97928
H	3.00075	1.55903	-1.10800
H	0.53346	-1.04211	-1.97892
H	0.84949	-2.74638	-1.63590
H	1.12399	2.47765	0.30673
H	0.97708	2.85019	-1.42081
H	-1.90942	-2.69963	-1.22016
H	-1.58488	-1.03410	-1.72098
H	-1.63159	2.94422	-1.03981
H	-1.31615	2.55078	0.65751
H	-3.65899	-1.30400	-0.33798
H	-2.66893	-1.85913	1.01459
H	-3.55106	1.74409	-0.07286
H	-2.79069	0.78592	-1.35010
C	3.35189	0.13942	0.86506
H	3.34591	-0.59400	1.66964
H	3.34399	1.13166	1.31203
H	4.28867	0.02418	0.30192
K	-0.03132	-0.09166	1.88910

ACE2 K DSDPBEP86 def2sv

33

O	-0.32270	-1.68811	-0.18834
O	-0.29388	1.28978	-0.70743
O	-2.40876	0.12585	0.59567
N	2.12651	-0.04977	0.00851
C	2.05884	-1.42137	-0.48658
C	2.02680	0.97444	-1.03640
C	0.73134	-1.78873	-1.12531
C	0.93170	1.98740	-0.74736
C	-1.59700	-1.72540	-0.79529
C	-1.42715	2.07006	-0.40829
C	-2.64780	-1.20547	0.16891
C	-2.62293	1.13975	-0.37482
H	2.23281	-2.09826	0.36978
H	2.86067	-1.63533	-1.23186
H	1.81030	0.50705	-2.01217
H	2.98956	1.51022	-1.15705
H	0.51775	-1.12820	-1.98681
H	0.80320	-2.82570	-1.51420
H	1.12043	2.49820	0.22172
H	0.91510	2.76574	-1.53886
H	-1.86482	-2.76132	-1.09213
H	-1.58663	-1.10816	-1.71408
H	-1.59093	2.85691	-1.17376
H	-1.30808	2.57903	0.57256
H	-3.64609	-1.29537	-0.30329
H	-2.65297	-1.81905	1.08577
H	-3.53467	1.71302	-0.11959
H	-2.76708	0.69918	-1.37695
C	3.29260	0.15493	0.85635
H	3.27436	-0.55424	1.70311

H	3.27787	1.17835	1.27026
H	4.25262	0.01606	0.30852
K	-0.01393	0.01665	1.88016

ACE2 K DSDPBEP86 def2tzv

33

O	-0.34617	-1.75295	-0.23680
O	-0.29144	1.31702	-0.73644
O	-2.45872	0.13599	0.63680
N	2.16440	-0.04882	0.02931
C	2.06198	-1.40854	-0.55256
C	2.08536	1.03597	-0.98531
C	0.72989	-1.69872	-1.22797
C	0.97944	2.03611	-0.67494
C	-1.66267	-1.69040	-0.86664
C	-1.44782	2.11826	-0.35249
C	-2.69636	-1.22490	0.14545
C	-2.65668	1.20157	-0.35261
H	2.21803	-2.13022	0.25580
H	2.85607	-1.57899	-1.30376
H	1.90897	0.61811	-1.98177
H	3.03953	1.57902	-1.03504
H	0.48616	-0.92464	-1.96195
H	0.79520	-2.66355	-1.74645
H	1.11630	2.46120	0.32910
H	0.98301	2.85345	-1.40742
H	-1.94894	-2.68197	-1.23983
H	-1.61644	-0.99954	-1.71503
H	-1.60468	2.93699	-1.06589
H	-1.29207	2.54980	0.64512
H	-3.69196	-1.28872	-0.30917

H	-2.67617	-1.85423	1.03667
H	-3.55160	1.76818	-0.07934
H	-2.80149	0.77635	-1.34969
C	3.38159	0.07411	0.86399
H	3.35731	-0.67382	1.66048
H	3.41022	1.06576	1.32146
H	4.30468	-0.06775	0.27651
K	-0.01688	-0.08763	1.90394

ACE2 K HSE06 def2sv

33

O	-0.31741	-1.68592	-0.20850
O	-0.29313	1.31717	-0.67778
O	-2.41785	0.11877	0.57067
N	2.14094	-0.04173	-0.00329
C	2.06878	-1.41288	-0.48642
C	2.01595	0.98228	-1.03858
C	0.74675	-1.79962	-1.12210
C	0.93029	2.00144	-0.74057
C	-1.58373	-1.72628	-0.81627
C	-1.42991	2.08279	-0.39181
C	-2.64491	-1.20391	0.13306
C	-2.62039	1.14660	-0.37816
H	2.25307	-2.08172	0.37311
H	2.86713	-1.63656	-1.23184
H	1.77987	0.51364	-2.00862
H	2.97500	1.51649	-1.18395
H	0.54051	-1.16333	-2.00417
H	0.83209	-2.84265	-1.48958
H	1.13612	2.52572	0.21726
H	0.91159	2.77101	-1.53985

H	-1.85256	-2.76225	-1.11010
H	-1.57030	-1.11764	-1.74148
H	-1.59398	2.87140	-1.15479
H	-1.32667	2.59276	0.59016
H	-3.63444	-1.29679	-0.35531
H	-2.66907	-1.82306	1.04561
H	-3.53399	1.71602	-0.12368
H	-2.75825	0.72346	-1.38876
C	3.29785	0.17502	0.84262
H	3.29650	-0.53886	1.68466
H	3.27096	1.19328	1.26666
H	4.26183	0.05705	0.29667
K	-0.03311	-0.02099	1.91554

ACE2 K HSE06 def2tzv

33

O	-0.31796	-1.74378	-0.24660
O	-0.31460	1.34823	-0.73509
O	-2.45656	0.10716	0.61515
N	2.16198	-0.02566	0.01445
C	2.08710	-1.39247	-0.52703
C	2.04033	1.03965	-1.00033
C	0.77543	-1.74243	-1.19919
C	0.95175	2.04582	-0.68094
C	-1.61347	-1.68369	-0.88912
C	-1.46649	2.11595	-0.32787
C	-2.66548	-1.23510	0.09933
C	-2.65800	1.18887	-0.33728
H	2.26078	-2.08754	0.29841
H	2.88581	-1.57588	-1.26768
H	1.82551	0.60669	-1.97999

H	2.98987	1.57935	-1.10381
H	0.54727	-1.02855	-1.99566
H	0.86911	-2.73499	-1.65368
H	1.10352	2.47809	0.31621
H	0.96664	2.86205	-1.41267
H	-1.89264	-2.67094	-1.27529
H	-1.56234	-0.99064	-1.73442
H	-1.64857	2.94812	-1.01770
H	-1.31299	2.53562	0.67390
H	-3.64809	-1.30898	-0.37776
H	-2.66531	-1.88198	0.97673
H	-3.55534	1.74360	-0.05322
H	-2.80491	0.78918	-1.34298
C	3.35190	0.15263	0.85475
H	3.35917	-0.58844	1.65559
H	3.33946	1.14318	1.31101
H	4.28898	0.05000	0.28397
K	-0.03080	-0.12193	1.92507

ACE2 K M062X def2sv

33

O	-0.31673	-1.68294	-0.16749
O	-0.30109	1.25605	-0.69454
O	-2.40801	0.11991	0.60467
N	2.12824	-0.04828	0.00984
C	2.06373	-1.42803	-0.45850
C	2.02082	0.95711	-1.04956
C	0.73741	-1.79917	-1.09599
C	0.91414	1.96192	-0.77757
C	-1.58588	-1.72059	-0.77457
C	-1.43393	2.04434	-0.43126

C	-2.63815	-1.21310	0.19352
C	-2.63384	1.12006	-0.37051
H	2.23252	-2.08824	0.40986
H	2.86759	-1.65248	-1.19430
H	1.81341	0.47185	-2.01694
H	2.97584	1.50056	-1.17361
H	0.52856	-1.14535	-1.96315
H	0.80307	-2.83818	-1.47303
H	1.10893	2.50468	0.17144
H	0.87304	2.71286	-1.59028
H	-1.84515	-2.75252	-1.08270
H	-1.57506	-1.09080	-1.68454
H	-1.58761	2.80426	-1.22147
H	-1.31736	2.58312	0.53223
H	-3.63680	-1.31417	-0.26996
H	-2.62839	-1.82319	1.11081
H	-3.53663	1.69925	-0.10945
H	-2.79402	0.66652	-1.36326
C	3.28193	0.17982	0.86530
H	3.25908	-0.51112	1.72458
H	3.25855	1.21017	1.25647
H	4.24492	0.03456	0.32920
K	-0.00489	0.06358	1.84173

ACE2 K M062X def2tzv

33

O	-0.32869	-1.73502	-0.18076
O	-0.30366	1.27234	-0.73220
O	-2.44612	0.12736	0.63800
N	2.15341	-0.04734	0.02261
C	2.07143	-1.42970	-0.48295

C	2.05693	0.99047	-1.02531
C	0.74998	-1.75946	-1.14983
C	0.95483	1.99311	-0.73454
C	-1.63059	-1.70141	-0.81176
C	-1.45463	2.07837	-0.38701
C	-2.66753	-1.23557	0.18736
C	-2.65806	1.16398	-0.35921
H	2.22018	-2.10481	0.36153
H	2.87276	-1.63495	-1.20937
H	1.86245	0.53426	-1.99625
H	3.00517	1.52959	-1.11653
H	0.52423	-1.03805	-1.93741
H	0.81225	-2.75335	-1.59998
H	1.11316	2.46267	0.24262
H	0.93412	2.77662	-1.49646
H	-1.89881	-2.69751	-1.17311
H	-1.59094	-1.02199	-1.66631
H	-1.60509	2.87063	-1.12414
H	-1.30694	2.54240	0.59341
H	-3.66105	-1.32676	-0.25669
H	-2.63178	-1.84827	1.08566
H	-3.54952	1.73094	-0.09275
H	-2.80741	0.72115	-1.34396
C	3.32716	0.14623	0.88911
H	3.29580	-0.56090	1.71731
H	3.31607	1.15498	1.29966
H	4.27072	0.00331	0.34478
K	-0.00624	0.01042	1.82391

ACE2 K M06 def2sv

33

O	-0.30468	-1.66033	-0.20998
O	-0.29883	1.30497	-0.72909
O	-2.40396	0.11320	0.58069
N	2.12251	-0.04270	-0.01049
C	2.07063	-1.41557	-0.48601
C	2.01244	0.98100	-1.04599
C	0.75539	-1.80644	-1.12125
C	0.92462	1.98883	-0.74426
C	-1.57085	-1.71639	-0.81217
C	-1.42606	2.06452	-0.39891
C	-2.62425	-1.21097	0.14632
C	-2.61744	1.13827	-0.36706
H	2.25334	-2.08047	0.38184
H	2.88208	-1.63586	-1.22251
H	1.78623	0.51458	-2.02329
H	2.97728	1.51389	-1.18221
H	0.55430	-1.18701	-2.02140
H	0.82627	-2.85947	-1.47171
H	1.11055	2.47647	0.24300
H	0.91827	2.79617	-1.50958
H	-1.82626	-2.75658	-1.11301
H	-1.56873	-1.10407	-1.73990
H	-1.60219	2.87650	-1.13845
H	-1.29373	2.55678	0.59365
H	-3.62327	-1.31437	-0.32625
H	-2.62870	-1.83441	1.06008
H	-3.52778	1.71307	-0.10322
H	-2.77585	0.71200	-1.37740
C	3.24276	0.19303	0.87447
H	3.21194	-0.50331	1.73436

H	3.19775	1.22287	1.27654
H	4.23047	0.06738	0.36879
K	-0.02648	-0.01240	1.90739

ACE2 K M06 def2tzv

33

O	-0.31347	-1.71965	-0.26912
O	-0.31989	1.40107	-0.77936
O	-2.39167	0.10695	0.61642
N	2.11769	-0.02949	-0.01226
C	2.07549	-1.39668	-0.54609
C	2.01492	1.04152	-1.01733
C	0.77039	-1.75900	-1.22032
C	0.95436	2.06009	-0.65573
C	-1.61146	-1.68814	-0.89144
C	-1.45598	2.13319	-0.29476
C	-2.63703	-1.22689	0.11665
C	-2.63287	1.19162	-0.31216
H	2.24342	-2.08394	0.28855
H	2.88695	-1.57526	-1.27283
H	1.76328	0.62125	-1.99374
H	2.97887	1.55312	-1.13647
H	0.55193	-1.06805	-2.04138
H	0.85602	-2.76429	-1.64849
H	1.10141	2.41019	0.37660
H	1.00247	2.93003	-1.32108
H	-1.88945	-2.68440	-1.25477
H	-1.58568	-1.01252	-1.75377
H	-1.66519	3.00457	-0.92591
H	-1.27457	2.49411	0.72647
H	-3.63358	-1.29219	-0.33175

H	-2.61724	-1.87367	0.99479
H	-3.53996	1.72335	-0.01541
H	-2.77680	0.80590	-1.32510
C	3.24855	0.17563	0.89518
H	3.22087	-0.55862	1.70280
H	3.18953	1.17103	1.33972
H	4.21989	0.08642	0.38273
K	-0.00074	-0.16714	1.91091

ACE2 K MP2 def2sv

33

O	-0.32971	-1.67871	-0.20977
O	-0.28792	1.29129	-0.70812
O	-2.40938	0.12894	0.58758
N	2.12769	-0.05028	0.00839
C	2.05016	-1.41089	-0.51379
C	2.03538	0.98766	-1.02322
C	0.72050	-1.75601	-1.15562
C	0.93779	1.99273	-0.72666
C	-1.60384	-1.70863	-0.82164
C	-1.41857	2.07475	-0.39809
C	-2.65400	-1.19801	0.14527
C	-2.61701	1.15033	-0.37817
H	2.22094	-2.10725	0.32696
H	2.84866	-1.61335	-1.26597
H	1.82976	0.53715	-2.00915
H	2.99803	1.52719	-1.12668
H	0.50542	-1.07151	-1.99679
H	0.78790	-2.78217	-1.57313
H	1.12078	2.48499	0.25236
H	0.92624	2.78486	-1.50459

H	-1.87228	-2.74063	-1.13116
H	-1.59151	-1.08103	-1.73239
H	-1.57817	2.87293	-1.15264
H	-1.29858	2.56827	0.58980
H	-3.65182	-1.27899	-0.32922
H	-2.66183	-1.82188	1.05470
H	-3.52836	1.72454	-0.12356
H	-2.75708	0.71916	-1.38428
C	3.31217	0.12715	0.83838
H	3.29999	-0.59479	1.67314
H	3.31554	1.14335	1.26758
H	4.25915	-0.01410	0.27054
K	-0.02242	-0.02060	1.93050

ACE2 K MP2 def2tzv

33

O	-0.35822	-1.74867	-0.30389
O	-0.28588	1.33446	-0.73514
O	-2.47168	0.12915	0.63903
N	2.17075	-0.04472	0.02874
C	2.05714	-1.38734	-0.60695
C	2.10879	1.07060	-0.96363
C	0.72263	-1.63314	-1.30107
C	0.99187	2.06150	-0.64696
C	-1.68019	-1.64487	-0.94286
C	-1.43799	2.13704	-0.30019
C	-2.71694	-1.21941	0.08777
C	-2.66163	1.23540	-0.32219
H	2.20000	-2.14072	0.17547
H	2.85391	-1.53211	-1.36259
H	1.95858	0.68034	-1.97712

H	3.06240	1.61951	-0.97223
H	0.47648	-0.81427	-1.98377
H	0.78381	-2.56667	-1.87577
H	1.11460	2.46431	0.36793
H	0.99712	2.89153	-1.36593
H	-1.97038	-2.61609	-1.36495
H	-1.61998	-0.91135	-1.75391
H	-1.59188	2.98540	-0.97957
H	-1.26148	2.52037	0.71332
H	-3.71132	-1.25300	-0.37421
H	-2.70323	-1.88149	0.95520
H	-3.54634	1.80444	-0.02046
H	-2.82088	0.84312	-1.33126
C	3.40844	0.03263	0.85547
H	3.37735	-0.73980	1.62795
H	3.45603	1.01086	1.33958
H	4.31916	-0.10746	0.24810
K	-0.02083	-0.18832	1.98824

ACE2 K PBE0 def2sv

33

O	-0.31825	-1.68643	-0.21086
O	-0.29299	1.31844	-0.68094
O	-2.41701	0.11879	0.56988
N	2.14039	-0.04189	-0.00444
C	2.06734	-1.41205	-0.48965
C	2.01643	0.98390	-1.03781
C	0.74487	-1.79798	-1.12491
C	0.93000	2.00226	-0.73982
C	-1.58405	-1.72553	-0.81832
C	-1.42869	2.08303	-0.39122

C	-2.64490	-1.20258	0.13120
C	-2.61971	1.14752	-0.37689
H	2.25202	-2.08222	0.36873
H	2.86496	-1.63495	-1.23602
H	1.78254	0.51728	-2.00929
H	2.97541	1.51896	-1.18038
H	0.53798	-1.16046	-2.00583
H	0.83080	-2.84046	-1.49409
H	1.13423	2.52481	0.21930
H	0.91339	2.77339	-1.53776
H	-1.85406	-2.76123	-1.11221
H	-1.57038	-1.11680	-1.74338
H	-1.59474	2.87325	-1.15223
H	-1.32352	2.59116	0.59154
H	-3.63448	-1.29461	-0.35738
H	-2.66974	-1.82253	1.04328
H	-3.53239	1.71763	-0.12019
H	-2.75973	0.72630	-1.38794
C	3.29762	0.17242	0.84145
H	3.29605	-0.54328	1.68200
H	3.27126	1.18977	1.26782
H	4.26148	0.05515	0.29514
K	-0.03217	-0.02370	1.92164

ACE2 K PBE0 def2tzv

33

O	-0.31888	-1.74273	-0.24929
O	-0.31423	1.34909	-0.73623
O	-2.45656	0.10718	0.61453
N	2.16183	-0.02588	0.01415
C	2.08549	-1.39100	-0.53095

C	2.04133	1.04171	-0.99813
C	0.77261	-1.73906	-1.20227
C	0.95083	2.04630	-0.67896
C	-1.61362	-1.68261	-0.89086
C	-1.46495	2.11558	-0.32724
C	-2.66581	-1.23329	0.09771
C	-2.65733	1.18898	-0.33607
H	2.25992	-2.08848	0.29272
H	2.88315	-1.57293	-1.27344
H	1.82994	0.61106	-1.97988
H	2.99057	1.58320	-1.09741
H	0.54393	-1.02313	-1.99712
H	0.86649	-2.73076	-1.65953
H	1.10134	2.47732	0.31930
H	0.96730	2.86402	-1.40958
H	-1.89394	-2.67013	-1.27656
H	-1.56305	-0.98986	-1.73682
H	-1.64850	2.94882	-1.01600
H	-1.31072	2.53446	0.67514
H	-3.64840	-1.30682	-0.38034
H	-2.66649	-1.88149	0.97461
H	-3.55381	1.74520	-0.05069
H	-2.80600	0.79083	-1.34245
C	3.35348	0.14827	0.85265
H	3.36095	-0.59536	1.65157
H	3.34321	1.13776	1.31201
H	4.28968	0.04587	0.27990
K	-0.03017	-0.12505	1.92990

ACE2 K PBE def2sv

33

O	-0.31769	-1.70304	-0.21699
O	-0.29477	1.33153	-0.68810
O	-2.44784	0.11820	0.57636
N	2.15942	-0.03750	-0.00123
C	2.08742	-1.41764	-0.49495
C	2.03307	0.99873	-1.04124
C	0.75972	-1.80943	-1.13897
C	0.94038	2.02475	-0.73974
C	-1.59313	-1.73242	-0.84004
C	-1.43988	2.10290	-0.38299
C	-2.66618	-1.21417	0.11449
C	-2.64079	1.16392	-0.37905
H	2.27490	-2.09285	0.37016
H	2.89259	-1.64262	-1.24770
H	1.79507	0.52890	-2.02024
H	2.99899	1.53939	-1.18682
H	0.54998	-1.16531	-2.02662
H	0.85239	-2.85923	-1.51495
H	1.14355	2.54811	0.23030
H	0.92737	2.80584	-1.54174
H	-1.86814	-2.77294	-1.14993
H	-1.57141	-1.10947	-1.76714
H	-1.60942	2.91059	-1.13949
H	-1.33077	2.60367	0.61429
H	-3.66035	-1.30914	-0.38560
H	-2.69328	-1.84347	1.03110
H	-3.55894	1.74311	-0.12075
H	-2.77979	0.74394	-1.40048
C	3.32888	0.17663	0.84693
H	3.33008	-0.54855	1.69065

H	3.30061	1.19986	1.28094
H	4.30077	0.06272	0.29370
K	-0.04130	-0.04280	1.94538

ACE2 K PBE def2tzv

33

O	-0.31823	-1.76326	-0.25972
O	-0.32586	1.36648	-0.76845
O	-2.48554	0.09887	0.63234
N	2.17621	-0.01914	0.01535
C	2.10578	-1.39354	-0.54673
C	2.05440	1.06356	-1.00270
C	0.78889	-1.74656	-1.22799
C	0.95668	2.07350	-0.67997
C	-1.62512	-1.68324	-0.92263
C	-1.48291	2.13593	-0.31112
C	-2.69006	-1.24944	0.07222
C	-2.68455	1.20763	-0.32393
H	2.28546	-2.09918	0.27939
H	2.91120	-1.56966	-1.29629
H	1.84308	0.63221	-1.99330
H	3.01030	1.61074	-1.09984
H	0.55306	-1.02084	-2.02341
H	0.88904	-2.74270	-1.69452
H	1.09264	2.49133	0.33482
H	0.98364	2.90820	-1.40408
H	-1.90725	-2.66973	-1.33433
H	-1.56160	-0.96732	-1.75915
H	-1.67875	2.99411	-0.97980
H	-1.30612	2.53117	0.70625
H	-3.67577	-1.31221	-0.42014

H	-2.70009	-1.91547	0.94572
H	-3.58317	1.76869	-0.02275
H	-2.84330	0.81394	-1.33903
C	3.38478	0.15265	0.85418
H	3.39717	-0.60343	1.65170
H	3.37232	1.14519	1.32449
H	4.32616	0.05745	0.27142
K	-0.02378	-0.15253	1.97075

ACE2 K SCS-MP2 def2sv

33

O	-0.33335	-1.68545	-0.21627
O	-0.28618	1.30481	-0.70640
O	-2.41424	0.13033	0.58446
N	2.13409	-0.05028	0.00872
C	2.05400	-1.41404	-0.51847
C	2.04227	0.99494	-1.02295
C	0.72089	-1.76094	-1.16305
C	0.94412	2.00453	-0.72216
C	-1.61097	-1.71485	-0.82893
C	-1.41990	2.08734	-0.39177
C	-2.66305	-1.19810	0.13954
C	-2.62071	1.15897	-0.37888
H	2.22481	-2.11220	0.32186
H	2.85262	-1.61500	-1.27160
H	1.83596	0.54752	-2.01088
H	3.00628	1.53379	-1.12397
H	0.50564	-1.07769	-2.00551
H	0.79001	-2.78866	-1.57834
H	1.12766	2.49392	0.25870
H	0.93436	2.79837	-1.49939

H	-1.88057	-2.74907	-1.13247
H	-1.59834	-1.09141	-1.74290
H	-1.57928	2.88932	-1.14335
H	-1.30051	2.57497	0.59972
H	-3.66144	-1.27432	-0.33622
H	-2.67412	-1.82229	1.04988
H	-3.53356	1.73089	-0.12089
H	-2.75933	0.73253	-1.38792
C	3.32533	0.12181	0.83843
H	3.31168	-0.60198	1.67304
H	3.33293	1.13837	1.26975
H	4.27102	-0.02219	0.26774
K	-0.02664	-0.03202	1.93930

ACE2 K SCS-MP2 def2tzv

33

O	-0.36052	-1.75275	-0.29778
O	-0.28524	1.34881	-0.74051
O	-2.46835	0.13293	0.63496
N	2.17185	-0.04717	0.02804
C	2.05989	-1.39427	-0.60548
C	2.10999	1.07024	-0.96742
C	0.72039	-1.65005	-1.29663
C	0.99568	2.06870	-0.64511
C	-1.68482	-1.66206	-0.93470
C	-1.43925	2.14687	-0.30347
C	-2.72221	-1.21790	0.09451
C	-2.66205	1.23691	-0.32883
H	2.20775	-2.14489	0.17958
H	2.85470	-1.53987	-1.36324
H	1.95139	0.67939	-1.97971

H	3.06626	1.61508	-0.98231
H	0.47462	-0.84156	-1.99208
H	0.78248	-2.59262	-1.85775
H	1.11968	2.46648	0.37222
H	1.00733	2.90286	-1.36034
H	-1.97516	-2.64266	-1.33622
H	-1.63023	-0.94480	-1.76087
H	-1.59630	2.99693	-0.98105
H	-1.26573	2.52841	0.71191
H	-3.71741	-1.25023	-0.36734
H	-2.71286	-1.87375	0.96786
H	-3.55122	1.80199	-0.02979
H	-2.81578	0.84296	-1.33860
C	3.41272	0.03280	0.85427
H	3.38171	-0.73685	1.63085
H	3.46102	1.01355	1.33540
H	4.32400	-0.10996	0.24749
K	-0.02097	-0.18209	1.98587

ACE2 Li B2PLYP def2sv

33

O	-0.32196	-1.69309	0.06766
O	-0.24015	1.39078	-0.38854
O	-2.17748	0.14142	0.86794
N	1.97438	-0.07750	0.30353
C	2.04036	-1.46325	-0.17988
C	2.02278	0.93243	-0.76920
C	0.74683	-1.91003	-0.84714
C	1.01270	2.04549	-0.52137
C	-1.62947	-1.80638	-0.47837
C	-1.38896	2.16982	-0.10214

C	-2.59133	-1.17873	0.51943
C	-2.53720	1.17839	-0.04621
H	2.21808	-2.10949	0.69424
H	2.88268	-1.62599	-0.88397
H	1.77006	0.45795	-1.72913
H	3.03828	1.35853	-0.87828
H	0.55257	-1.34466	-1.77674
H	0.81710	-2.98046	-1.11260
H	1.24587	2.61259	0.40005
H	0.99695	2.75334	-1.36991
H	-1.89726	-2.86530	-0.64928
H	-1.66986	-1.28070	-1.44984
H	-1.57212	2.92321	-0.88937
H	-1.26656	2.69420	0.86478
H	-3.61859	-1.18124	0.11668
H	-2.58557	-1.75312	1.45794
H	-3.46521	1.67485	0.28690
H	-2.70526	0.76103	-1.05278
C	2.97364	0.18804	1.33907
H	2.81700	-0.48907	2.19339
H	2.86796	1.22277	1.70073
H	4.01055	0.05447	0.96614
Li	-0.07894	0.00400	1.04156

ACE2 Li B2PLYP def2tzv

33

O	-0.32581	-1.73001	0.07625
O	-0.23925	1.39418	-0.40215
O	-2.19518	0.14850	0.89553
N	1.98116	-0.07882	0.31501
C	2.06084	-1.47805	-0.19338

C	2.05760	0.94774	-0.76885
C	0.77200	-1.91469	-0.88244
C	1.05340	2.07167	-0.52741
C	-1.67311	-1.82052	-0.49501
C	-1.41806	2.20489	-0.10191
C	-2.62440	-1.21124	0.52564
C	-2.56798	1.21352	-0.05602
H	2.23952	-2.12703	0.66372
H	2.89724	-1.61211	-0.89298
H	1.82292	0.48170	-1.72497
H	3.06852	1.36346	-0.84502
H	0.56286	-1.31636	-1.77198
H	0.83802	-2.96469	-1.17183
H	1.26702	2.62252	0.39230
H	1.03480	2.76713	-1.36829
H	-1.93688	-2.86300	-0.68182
H	-1.69492	-1.27226	-1.43938
H	-1.58668	2.94842	-0.88269
H	-1.28410	2.70906	0.85806
H	-3.64260	-1.19699	0.13504
H	-2.60564	-1.77579	1.45396
H	-3.48477	1.69420	0.28364
H	-2.72925	0.78622	-1.04589
C	2.99239	0.17244	1.37450
H	2.82253	-0.50640	2.20841
H	2.89220	1.19422	1.73685
H	4.01593	0.03162	1.00348
Li	-0.06634	-0.00936	1.02555

ACE2 Li B3LYP def2sv

33

O	-0.32212	-1.69886	0.06297
O	-0.23922	1.40661	-0.38278
O	-2.18604	0.14101	0.86280
N	1.98617	-0.07844	0.29919
C	2.04750	-1.46815	-0.18080
C	2.02862	0.93771	-0.77128
C	0.75154	-1.92410	-0.84558
C	1.01659	2.05496	-0.52416
C	-1.63357	-1.82257	-0.47333
C	-1.39400	2.17968	-0.10230
C	-2.59731	-1.18233	0.52065
C	-2.54392	1.18398	-0.04705
H	2.22988	-2.11108	0.69627
H	2.88835	-1.63702	-0.88724
H	1.77758	0.46333	-1.73316
H	3.04334	1.36813	-0.88210
H	0.55850	-1.37116	-1.78496
H	0.82550	-2.99868	-1.09913
H	1.25417	2.63032	0.39294
H	1.00134	2.75863	-1.37811
H	-1.90092	-2.88593	-0.62686
H	-1.68074	-1.31366	-1.45529
H	-1.57941	2.93147	-0.89270
H	-1.27670	2.71012	0.86398
H	-3.62522	-1.19067	0.11588
H	-2.59438	-1.75019	1.46517
H	-3.47115	1.68269	0.29007
H	-2.71737	0.77087	-1.05619
C	2.97277	0.18956	1.34785
H	2.81249	-0.49214	2.19997

H	2.85809	1.22303	1.71515
H	4.01749	0.06485	0.98805
Li	-0.07810	0.00458	1.02866

ACE2 Li B3LYP def2tzv

33

O	-0.32194	-1.73039	0.07211
O	-0.24333	1.40972	-0.40324
O	-2.19779	0.14360	0.88865
N	1.98892	-0.07598	0.30905
C	2.06661	-1.47942	-0.18414
C	2.05108	0.94931	-0.77508
C	0.78209	-1.93332	-0.87082
C	1.05068	2.07680	-0.53196
C	-1.66808	-1.83369	-0.49162
C	-1.42423	2.20888	-0.09755
C	-2.62056	-1.21559	0.52257
C	-2.57073	1.21345	-0.05334
H	2.24345	-2.11781	0.68119
H	2.90583	-1.62759	-0.87744
H	1.80699	0.48017	-1.72705
H	3.06034	1.36538	-0.86789
H	0.57957	-1.36053	-1.77897
H	0.85514	-2.98968	-1.13567
H	1.27050	2.63256	0.38383
H	1.03708	2.77108	-1.37450
H	-1.92997	-2.88000	-0.66213
H	-1.69803	-1.30342	-1.44631
H	-1.60135	2.95718	-0.87263
H	-1.29265	2.71232	0.86390
H	-3.63929	-1.21170	0.13264

H	-2.60195	-1.77647	1.45381
H	-3.48762	1.69261	0.28992
H	-2.73626	0.79384	-1.04585
C	2.98414	0.18862	1.37633
H	2.82115	-0.49477	2.20842
H	2.86648	1.20648	1.74516
H	4.01528	0.06769	1.01790
Li	-0.06623	-0.00639	1.00727

ACE2 Li B97-1 def2sv

33

O	-0.31949	-1.70101	0.06591
O	-0.24259	1.39955	-0.38288
O	-2.19351	0.13991	0.86638
N	1.98795	-0.07583	0.29893
C	2.05065	-1.46614	-0.18023
C	2.02663	0.93884	-0.77389
C	0.75049	-1.92111	-0.84411
C	1.00781	2.05295	-0.52387
C	-1.62632	-1.81586	-0.47706
C	-1.39448	2.17353	-0.10467
C	-2.59644	-1.18174	0.51952
C	-2.54772	1.17707	-0.04721
H	2.23348	-2.10939	0.69886
H	2.89167	-1.63539	-0.88891
H	1.77506	0.46214	-1.73661
H	3.04104	1.37383	-0.88579
H	0.55612	-1.36298	-1.78260
H	0.82571	-2.99613	-1.10322
H	1.24576	2.62829	0.39563
H	0.98962	2.75904	-1.37804

H	-1.89674	-2.87836	-0.64297
H	-1.66856	-1.29520	-1.45541
H	-1.58008	2.92474	-0.89797
H	-1.27769	2.70672	0.86225
H	-3.62497	-1.19504	0.11152
H	-2.59225	-1.75467	1.46290
H	-3.47645	1.67924	0.28586
H	-2.71895	0.76070	-1.05753
C	2.97985	0.19574	1.34264
H	2.82395	-0.48662	2.19708
H	2.86266	1.23104	1.70909
H	4.02441	0.07308	0.97717
Li	-0.06919	0.00616	1.05803

ACE2 Li B97-1 def2tzv

33

O	-0.31931	-1.73501	0.07488
O	-0.24776	1.40221	-0.41296
O	-2.20231	0.14169	0.89593
N	1.98725	-0.07303	0.31202
C	2.06947	-1.47602	-0.18351
C	2.05064	0.95096	-0.77427
C	0.78051	-1.92917	-0.87098
C	1.04148	2.07558	-0.53142
C	-1.66036	-1.82553	-0.49713
C	-1.42339	2.20247	-0.09981
C	-2.61906	-1.21425	0.52160
C	-2.57428	1.20617	-0.04841
H	2.24754	-2.11723	0.68291
H	2.90950	-1.62137	-0.88021
H	1.80949	0.47990	-1.72923

H	3.06057	1.37243	-0.86349
H	0.57603	-1.34822	-1.77713
H	0.85653	-2.98581	-1.14462
H	1.25648	2.62835	0.39086
H	1.03041	2.77562	-1.37257
H	-1.92787	-2.87049	-0.68286
H	-1.68412	-1.28053	-1.44713
H	-1.60610	2.95293	-0.87511
H	-1.28610	2.70628	0.86377
H	-3.63892	-1.21283	0.12725
H	-2.59995	-1.78376	1.45058
H	-3.49136	1.69085	0.29451
H	-2.74203	0.78352	-1.04243
C	2.99030	0.19255	1.37287
H	2.83107	-0.49121	2.20854
H	2.87403	1.21365	1.74068
H	4.02101	0.07025	1.00678
Li	-0.06126	-0.00622	1.02275

ACE2 Li CAM-B3LYP def2sv

33

O	-0.32232	-1.68308	0.05958
O	-0.23733	1.39439	-0.38579
O	-2.16351	0.13970	0.85924
N	1.97057	-0.07943	0.30039
C	2.03470	-1.46246	-0.17702
C	2.01922	0.92810	-0.76747
C	0.74451	-1.91082	-0.84376
C	1.01167	2.04081	-0.52382
C	-1.62652	-1.80776	-0.47336
C	-1.38304	2.16723	-0.10334

C	-2.58274	-1.17420	0.52239
C	-2.52894	1.17694	-0.04113
H	2.21116	-2.10642	0.69871
H	2.87813	-1.62802	-0.87808
H	1.76844	0.45323	-1.72755
H	3.03461	1.35331	-0.87572
H	0.55535	-1.35360	-1.77972
H	0.81286	-2.98336	-1.10023
H	1.24687	2.61297	0.39412
H	0.99667	2.74506	-1.37512
H	-1.89120	-2.86916	-0.63206
H	-1.67542	-1.29287	-1.45063
H	-1.56864	2.91730	-0.89295
H	-1.26006	2.69682	0.86080
H	-3.61130	-1.17548	0.12347
H	-2.57703	-1.74590	1.46275
H	-3.45368	1.67318	0.30087
H	-2.70646	0.76380	-1.04815
C	2.95266	0.18853	1.34447
H	2.79178	-0.48923	2.19764
H	2.84098	1.22223	1.70780
H	3.99430	0.05999	0.98311
Li	-0.08174	0.00529	1.01408

ACE2 Li CAM-B3LYP def2tzv

33

O	-0.32383	-1.71086	0.06554
O	-0.23773	1.39969	-0.39954
O	-2.17247	0.14464	0.88024
N	1.97243	-0.07967	0.30776
C	2.04965	-1.47446	-0.18106

C	2.04055	0.93626	-0.77068
C	0.77085	-1.91813	-0.86887
C	1.04733	2.06000	-0.52925
C	-1.66136	-1.81778	-0.48800
C	-1.40927	2.19548	-0.09995
C	-2.60301	-1.20324	0.52795
C	-2.55036	1.20469	-0.05137
H	2.21994	-2.11446	0.68286
H	2.89129	-1.62128	-0.86886
H	1.79524	0.46812	-1.72142
H	3.05103	1.34575	-0.86194
H	0.57387	-1.34090	-1.77401
H	0.83707	-2.97248	-1.13770
H	1.26894	2.61347	0.38608
H	1.03361	2.75477	-1.36949
H	-1.92155	-2.86264	-0.65960
H	-1.69696	-1.28543	-1.44017
H	-1.58454	2.94058	-0.87641
H	-1.27763	2.70081	0.85894
H	-3.62380	-1.19550	0.14731
H	-2.57834	-1.76516	1.45682
H	-3.46665	1.68137	0.29219
H	-2.71596	0.78465	-1.04264
C	2.95695	0.18483	1.37275
H	2.78900	-0.49186	2.20755
H	2.84363	1.20426	1.73493
H	3.98660	0.05692	1.01769
Li	-0.06921	-0.00565	0.99152

ACE2 Li DSDPBEP86 def2tzv

33

O	-0.32761	-1.72610	0.07517
O	-0.23755	1.37352	-0.42076
O	-2.18721	0.15116	0.90602
N	1.97111	-0.08174	0.32028
C	2.05183	-1.47415	-0.19831
C	2.06245	0.94341	-0.76006
C	0.76022	-1.88959	-0.89359
C	1.05004	2.05943	-0.52353
C	-1.67043	-1.79819	-0.50114
C	-1.40515	2.19167	-0.10697
C	-2.61868	-1.20310	0.53048
C	-2.56039	1.20756	-0.04967
H	2.22446	-2.13537	0.65516
H	2.89228	-1.60188	-0.89936
H	1.84082	0.47864	-1.72392
H	3.07647	1.36281	-0.81998
H	0.54848	-1.26117	-1.76593
H	0.81986	-2.93423	-1.21444
H	1.24978	2.60103	0.40907
H	1.03705	2.76657	-1.35892
H	-1.94023	-2.83777	-0.71244
H	-1.68659	-1.22571	-1.43538
H	-1.57639	2.94029	-0.88665
H	-1.25652	2.69254	0.85650
H	-3.64186	-1.18470	0.14371
H	-2.59339	-1.77891	1.45548
H	-3.47690	1.69584	0.28996
H	-2.72573	0.77328	-1.03980
C	2.98275	0.16047	1.37875
H	2.80395	-0.51732	2.21584

H	2.89051	1.18752	1.73846
H	4.00743	0.00810	1.00602
Li	-0.06275	-0.01036	1.03514

ACE2 Li DSDPBEP86 def2sv

33

O	-0.32130	-1.68447	0.06554
O	-0.24062	1.37034	-0.41305
O	-2.16576	0.14164	0.87818
N	1.96162	-0.07885	0.30817
C	2.03161	-1.45897	-0.17957
C	2.02351	0.92669	-0.76294
C	0.73997	-1.88884	-0.85525
C	1.00834	2.03168	-0.52065
C	-1.62391	-1.78392	-0.48573
C	-1.37726	2.15471	-0.10508
C	-2.58069	-1.17311	0.52329
C	-2.52813	1.17158	-0.03779
H	2.19813	-2.11317	0.69361
H	2.88117	-1.61860	-0.87864
H	1.78015	0.45209	-1.72752
H	3.04198	1.35363	-0.86061
H	0.54676	-1.30120	-1.77348
H	0.80403	-2.95590	-1.14295
H	1.22667	2.58774	0.41358
H	1.00061	2.75169	-1.36152
H	-1.89426	-2.84021	-0.67948
H	-1.66138	-1.23607	-1.44736
H	-1.56591	2.91685	-0.88516
H	-1.23658	2.67041	0.86631
H	-3.61334	-1.17298	0.12880

H	-2.56469	-1.75873	1.45685
H	-3.45495	1.67270	0.29759
H	-2.70138	0.74911	-1.04350
C	2.95536	0.18360	1.34578
H	2.79076	-0.49304	2.20138
H	2.85024	1.22117	1.70541
H	3.99461	0.04523	0.97623
Li	-0.07509	0.00685	1.04188

ACE2 Li HSE06 def2sv

33

O	-0.31647	-1.68684	0.06668
O	-0.24218	1.38498	-0.37909
O	-2.17843	0.13754	0.86352
N	1.97586	-0.07464	0.29685
C	2.03813	-1.45561	-0.17547
C	2.01095	0.92903	-0.77078
C	0.74817	-1.90509	-0.83742
C	1.00054	2.03584	-0.52272
C	-1.61458	-1.79771	-0.47713
C	-1.38721	2.15652	-0.10443
C	-2.57899	-1.17595	0.51532
C	-2.53189	1.16715	-0.04595
H	2.21685	-2.09740	0.70351
H	2.88103	-1.62864	-0.87818
H	1.75484	0.45015	-1.72950
H	3.02428	1.35998	-0.89107
H	0.55570	-1.34468	-1.77309
H	0.82210	-2.97691	-1.10146
H	1.23968	2.61203	0.39376
H	0.98093	2.74088	-1.37509

H	-1.88421	-2.85632	-0.65507
H	-1.65444	-1.26883	-1.44928
H	-1.57175	2.90635	-0.89645
H	-1.27071	2.69075	0.85979
H	-3.60680	-1.18937	0.11057
H	-2.57415	-1.75307	1.45399
H	-3.46111	1.66744	0.28192
H	-2.70060	0.74915	-1.05424
C	2.95984	0.20012	1.33224
H	2.80736	-0.47767	2.18845
H	2.84275	1.23425	1.69610
H	4.00292	0.07871	0.96814
Li	-0.06960	0.00482	1.05945

ACE2 Li HSE06 def2tzv

33

O	-0.31676	-1.72049	0.07497
O	-0.24600	1.38806	-0.40513
O	-2.18687	0.14007	0.89121
N	1.97558	-0.07277	0.30861
C	2.05470	-1.46539	-0.17913
C	2.03375	0.93998	-0.77095
C	0.77614	-1.91119	-0.86354
C	1.03404	2.05649	-0.52870
C	-1.64781	-1.80587	-0.49530
C	-1.41449	2.18415	-0.10023
C	-2.59986	-1.20643	0.51833
C	-2.55588	1.19482	-0.04860
H	2.22940	-2.10586	0.68716
H	2.89621	-1.61550	-0.87114
H	1.78801	0.46747	-1.72288

H	3.04293	1.35838	-0.86879
H	0.57392	-1.32638	-1.76655
H	0.85117	-2.96458	-1.14485
H	1.25293	2.61066	0.39052
H	1.02140	2.75614	-1.36849
H	-1.91641	-2.84697	-0.69312
H	-1.67034	-1.25303	-1.43963
H	-1.59563	2.93240	-0.87622
H	-1.28056	2.69170	0.86052
H	-3.61952	-1.20713	0.12677
H	-2.58006	-1.77991	1.44348
H	-3.47483	1.67854	0.28656
H	-2.71943	0.77044	-1.04152
C	2.96821	0.19584	1.36143
H	2.81085	-0.48211	2.20047
H	2.85370	1.21707	1.72543
H	3.99853	0.07264	0.99837
Li	-0.06236	-0.00674	1.02182

ACE2 Li M062X def2sv

33

O	-0.31214	-1.66622	0.06240
O	-0.25229	1.35003	-0.39974
O	-2.17457	0.12862	0.88260
N	1.97046	-0.07014	0.30791
C	2.04147	-1.45006	-0.17576
C	2.01644	0.93306	-0.76465
C	0.74904	-1.87815	-0.84970
C	0.98460	2.02267	-0.52458
C	-1.60763	-1.76716	-0.49316
C	-1.38998	2.13439	-0.11329

C	-2.57434	-1.18372	0.52204
C	-2.54194	1.15341	-0.02904
H	2.20916	-2.10101	0.69754
H	2.88810	-1.60766	-0.87478
H	1.78218	0.45271	-1.72737
H	3.02585	1.37477	-0.86007
H	0.55866	-1.29271	-1.76915
H	0.80712	-2.94428	-1.13302
H	1.20170	2.58881	0.40223
H	0.95828	2.73316	-1.36981
H	-1.86408	-2.81942	-0.71157
H	-1.64362	-1.19636	-1.44054
H	-1.57290	2.87973	-0.90728
H	-1.25143	2.66697	0.84718
H	-3.60451	-1.19062	0.12721
H	-2.55089	-1.77967	1.44680
H	-3.46211	1.65712	0.31205
H	-2.72502	0.72647	-1.03005
C	2.96914	0.19967	1.33686
H	2.81860	-0.47881	2.19094
H	2.85985	1.23470	1.69729
H	4.00266	0.06909	0.95509
Li	-0.06652	0.01124	1.05922

ACE2 Li M062X def2tzv

33

O	-0.31740	-1.70001	0.06768
O	-0.24768	1.36464	-0.41948
O	-2.17159	0.13713	0.89732
N	1.96586	-0.07502	0.31512
C	2.05190	-1.46835	-0.17860

C	2.04068	0.93614	-0.76894
C	0.77005	-1.89058	-0.87625
C	1.02947	2.04486	-0.53007
C	-1.64769	-1.78303	-0.50392
C	-1.40883	2.17159	-0.10534
C	-2.59477	-1.20707	0.53100
C	-2.55638	1.18706	-0.03942
H	2.21032	-2.11536	0.68382
H	2.89856	-1.60732	-0.86198
H	1.80729	0.46461	-1.72241
H	3.04878	1.35439	-0.84710
H	0.57279	-1.28240	-1.76227
H	0.82496	-2.93791	-1.17459
H	1.23397	2.58658	0.39729
H	1.01096	2.74978	-1.36177
H	-1.90386	-2.81902	-0.72750
H	-1.67165	-1.20059	-1.42827
H	-1.58367	2.91854	-0.87999
H	-1.25853	2.67121	0.85435
H	-3.61824	-1.19972	0.15629
H	-2.55443	-1.78873	1.44784
H	-3.46990	1.66735	0.30643
H	-2.72555	0.75831	-1.02762
C	2.96064	0.19240	1.37278
H	2.79148	-0.47785	2.21310
H	2.85348	1.21720	1.72369
H	3.98488	0.05344	1.00675
Li	-0.06323	0.00013	1.00886

ACE2 Li M06 def2sv

33

O	-0.32866	-1.70612	0.07598
O	-0.24271	1.35766	-0.39908
O	-2.20495	0.14630	0.87337
N	1.99622	-0.08109	0.30339
C	2.01979	-1.45168	-0.20256
C	2.02868	0.94266	-0.74767
C	0.71871	-1.85415	-0.85946
C	0.99814	2.02295	-0.49786
C	-1.62592	-1.77916	-0.47114
C	-1.38042	2.13720	-0.11013
C	-2.59005	-1.16965	0.52097
C	-2.53746	1.17000	-0.04737
H	2.19915	-2.12390	0.65753
H	2.85520	-1.61857	-0.92090
H	1.80455	0.47709	-1.72500
H	3.03881	1.39352	-0.84114
H	0.50642	-1.22081	-1.74843
H	0.78343	-2.90398	-1.21242
H	1.20814	2.57686	0.44446
H	0.98402	2.75966	-1.32658
H	-1.91128	-2.82956	-0.68784
H	-1.65406	-1.22514	-1.43432
H	-1.55294	2.90498	-0.89109
H	-1.24627	2.66140	0.86186
H	-3.62070	-1.18958	0.11586
H	-2.58431	-1.75301	1.45887
H	-3.46368	1.68962	0.26660
H	-2.71117	0.74482	-1.05578
C	3.01968	0.14599	1.30956
H	2.88347	-0.55093	2.15561

H 2.94345 1.17517 1.70457

H 4.04788 0.00762 0.90275

Li -0.06000 0.07486 1.12779

ACE2 Li M06 def2tzv

33

O -0.30753 -1.73453 0.08888

O -0.26204 1.36674 -0.40371

O -2.20024 0.13444 0.88578

N 1.98876 -0.06292 0.30490

C 2.05759 -1.45476 -0.18173

C 2.02015 0.95035 -0.77477

C 0.77595 -1.89821 -0.85599

C 1.00676 2.04798 -0.51754

C -1.62903 -1.80053 -0.49114

C -1.42608 2.16404 -0.10345

C -2.59095 -1.21212 0.51709

C -2.56875 1.17876 -0.05388

H 2.23676 -2.09551 0.68436

H 2.89584 -1.60255 -0.87884

H 1.77387 0.47566 -1.72645

H 3.02239 1.38382 -0.87852

H 0.56563 -1.30208 -1.75178

H 0.85604 -2.94536 -1.16104

H 1.22113 2.58682 0.41397

H 0.98361 2.76654 -1.34123

H -1.90277 -2.83548 -0.71535

H -1.64274 -1.23068 -1.42766

H -1.59735 2.91823 -0.87583

H -1.29184 2.66699 0.86126

H -3.60954 -1.22738 0.12171

H	-2.56675	-1.78771	1.44149
H	-3.49041	1.66366	0.27249
H	-2.72808	0.75360	-1.04841
C	3.00588	0.20505	1.33180
H	2.87345	-0.47917	2.17017
H	2.89830	1.22456	1.70392
H	4.02577	0.08585	0.93772
Li	-0.06510	0.06802	1.09125

ACE2 Li MP2 def2tzv

33

O	-0.34425	-1.74294	0.07887
O	-0.22470	1.36286	-0.41289
O	-2.20384	0.16759	0.91476
N	1.98413	-0.09295	0.32578
C	2.04214	-1.47924	-0.23088
C	2.09210	0.95245	-0.74356
C	0.73469	-1.85658	-0.92404
C	1.06800	2.06314	-0.50994
C	-1.69979	-1.79566	-0.49888
C	-1.39664	2.20009	-0.11315
C	-2.64515	-1.19493	0.53691
C	-2.56386	1.22506	-0.05979
H	2.21867	-2.16561	0.60270
H	2.87158	-1.59301	-0.94942
H	1.89616	0.50122	-1.72074
H	3.10586	1.37899	-0.77082
H	0.50691	-1.17890	-1.75468
H	0.78357	-2.88408	-1.29960
H	1.25782	2.60130	0.42696
H	1.05088	2.77107	-1.34521

H	-1.97969	-2.83168	-0.71677
H	-1.70761	-1.21404	-1.42771
H	-1.55190	2.94210	-0.90297
H	-1.25049	2.70501	0.84861
H	-3.66740	-1.16393	0.14641
H	-2.62578	-1.76651	1.46485
H	-3.47826	1.72355	0.27196
H	-2.72548	0.78358	-1.04794
C	3.02350	0.10587	1.37915
H	2.83417	-0.58084	2.20677
H	2.96091	1.13073	1.75191
H	4.03728	-0.06977	0.98654
Li	-0.05790	-0.01989	1.09595

ACE2 Li MP2 def2sv

33

O	-0.32370	-1.68474	0.06996
O	-0.24120	1.36217	-0.41562
O	-2.16808	0.14408	0.88156
N	1.96083	-0.07887	0.31132
C	2.02716	-1.45561	-0.18436
C	2.02372	0.92689	-0.75856
C	0.73454	-1.87785	-0.85902
C	1.00755	2.02847	-0.51539
C	-1.62627	-1.77777	-0.48693
C	-1.37583	2.15133	-0.10409
C	-2.58315	-1.17131	0.52201
C	-2.52754	1.17165	-0.04004
H	2.19308	-2.11651	0.68356
H	2.87525	-1.61219	-0.88613
H	1.78245	0.45610	-1.72515

H	3.04216	1.35470	-0.85421
H	0.53774	-1.28025	-1.76924
H	0.79845	-2.94149	-1.15926
H	1.22303	2.57917	0.42219
H	1.00164	2.75273	-1.35263
H	-1.89910	-2.83190	-0.68846
H	-1.66032	-1.22367	-1.44428
H	-1.56238	2.91698	-0.88109
H	-1.23475	2.66224	0.86932
H	-3.61541	-1.16999	0.12682
H	-2.56764	-1.75974	1.45329
H	-3.45570	1.67392	0.28979
H	-2.69589	0.74832	-1.04551
C	2.97150	0.17431	1.33566
H	2.81236	-0.50123	2.19211
H	2.87979	1.21188	1.69623
H	4.00321	0.02678	0.95124
Li	-0.07667	0.00450	1.06643

ACE2 Li PBE0 def2sv

33

O	-0.31695	-1.68612	0.06632
O	-0.24140	1.38514	-0.37956
O	-2.17747	0.13820	0.86290
N	1.97502	-0.07516	0.29658
C	2.03702	-1.45578	-0.17631
C	2.01131	0.92903	-0.77022
C	0.74665	-1.90512	-0.83762
C	1.00078	2.03568	-0.52223
C	-1.61467	-1.79807	-0.47630
C	-1.38569	2.15642	-0.10466

C	-2.57866	-1.17448	0.51557
C	-2.53064	1.16736	-0.04593
H	2.21599	-2.09787	0.70240
H	2.87957	-1.62870	-0.87941
H	1.75612	0.45094	-1.72952
H	3.02477	1.36001	-0.88920
H	0.55428	-1.34547	-1.77372
H	0.82058	-2.97725	-1.10084
H	1.23972	2.61164	0.39449
H	0.98194	2.74096	-1.37454
H	-1.88466	-2.85713	-0.65160
H	-1.65543	-1.27152	-1.44968
H	-1.57068	2.90630	-0.89664
H	-1.26913	2.69070	0.85960
H	-3.60653	-1.18786	0.11071
H	-2.57439	-1.75109	1.45466
H	-3.45953	1.66824	0.28236
H	-2.70001	0.74996	-1.05436
C	2.95825	0.19858	1.33274
H	2.80484	-0.47982	2.18837
H	2.84091	1.23247	1.69735
H	4.00161	0.07725	0.96942
Li	-0.06823	0.00497	1.05884

ACE2 Li PBE0 def2tzv

33

O	-0.31704	-1.72000	0.07461
O	-0.24538	1.38834	-0.40582
O	-2.18583	0.14066	0.89080
N	1.97422	-0.07314	0.30878
C	2.05357	-1.46510	-0.18029

C	2.03391	0.94014	-0.76988
C	0.77421	-1.91086	-0.86384
C	1.03358	2.05647	-0.52796
C	-1.64729	-1.80612	-0.49439
C	-1.41285	2.18373	-0.10057
C	-2.59915	-1.20464	0.51884
C	-2.55455	1.19428	-0.04835
H	2.22923	-2.10643	0.68557
H	2.89461	-1.61440	-0.87342
H	1.78931	0.46821	-1.72266
H	3.04337	1.35892	-0.86603
H	0.57191	-1.32623	-1.76732
H	0.84967	-2.96463	-1.14516
H	1.25228	2.61058	0.39176
H	1.02214	2.75662	-1.36786
H	-1.91690	-2.84779	-0.68996
H	-1.67074	-1.25519	-1.44021
H	-1.59509	2.93220	-0.87660
H	-1.27902	2.69154	0.86047
H	-3.61903	-1.20572	0.12686
H	-2.57980	-1.77815	1.44445
H	-3.47344	1.67901	0.28683
H	-2.71862	0.77027	-1.04169
C	2.96701	0.19393	1.36160
H	2.80950	-0.48522	2.20007
H	2.85268	1.21503	1.72696
H	3.99750	0.07090	0.99818
Li	-0.06125	-0.00683	1.02020

ACE2 Li PBE def2sv

33

O	-0.31323	-1.70975	0.07353
O	-0.24953	1.39197	-0.39493
O	-2.20265	0.13653	0.87915
N	1.99050	-0.07093	0.30111
C	2.05813	-1.46287	-0.17744
C	2.02565	0.94283	-0.77478
C	0.76230	-1.92119	-0.84373
C	1.00540	2.05454	-0.52755
C	-1.62270	-1.81155	-0.48485
C	-1.40357	2.17128	-0.10297
C	-2.59690	-1.19144	0.51402
C	-2.55588	1.17625	-0.04494
H	2.24240	-2.10731	0.70928
H	2.90758	-1.63613	-0.88722
H	1.77210	0.45881	-1.74152
H	3.04556	1.38214	-0.89323
H	0.56254	-1.35527	-1.78587
H	0.84378	-3.00088	-1.11337
H	1.23825	2.63237	0.40101
H	0.99095	2.76912	-1.38444
H	-1.89871	-2.87667	-0.67468
H	-1.65497	-1.27078	-1.46149
H	-1.59558	2.93407	-0.89429
H	-1.27868	2.70299	0.87275
H	-3.63177	-1.21039	0.10239
H	-2.59059	-1.77417	1.45983
H	-3.49160	1.68442	0.28482
H	-2.72675	0.75346	-1.06065
C	2.98211	0.20694	1.34444
H	2.83163	-0.48109	2.20393

H	2.85710	1.24684	1.71509
H	4.03522	0.09283	0.97774
Li	-0.06528	0.00448	1.06235

ACE2 Li PBE def2tzv

33

O	-0.31261	-1.74787	0.08666
O	-0.25757	1.38891	-0.43435
O	-2.21574	0.13809	0.91484
N	1.99210	-0.06708	0.31512
C	2.07721	-1.47127	-0.18413
C	2.05055	0.95910	-0.77317
C	0.79270	-1.92421	-0.87333
C	1.03811	2.07719	-0.53182
C	-1.65719	-1.81704	-0.50737
C	-1.43444	2.19783	-0.09725
C	-2.62145	-1.22579	0.51270
C	-2.58482	1.20635	-0.04558
H	2.25837	-2.11623	0.68777
H	2.92463	-1.61835	-0.88400
H	1.81064	0.48199	-1.73367
H	3.06544	1.38738	-0.86572
H	0.57863	-1.32866	-1.77694
H	0.87499	-2.98337	-1.16431
H	1.24056	2.62557	0.40478
H	1.03327	2.79077	-1.37124
H	-1.93094	-2.86255	-0.72385
H	-1.66559	-1.24627	-1.45110
H	-1.62382	2.96357	-0.86657
H	-1.28277	2.69460	0.87649
H	-3.64661	-1.22792	0.11199

H	-2.60320	-1.80775	1.44249
H	-3.50864	1.69758	0.29374
H	-2.75236	0.77430	-1.04340
C	3.00085	0.20079	1.37223
H	2.85007	-0.49126	2.21126
H	2.87923	1.22535	1.74789
H	4.03814	0.08715	0.99948
Li	-0.05552	-0.00901	1.04275

ACE2 Li SCS-MP2 def2sv

33

O	-0.32572	-1.68990	0.06852
O	-0.24063	1.37074	-0.41745
O	-2.17068	0.14492	0.88143
N	1.96506	-0.08010	0.31194
C	2.03150	-1.46045	-0.18803
C	2.03120	0.93224	-0.75865
C	0.73467	-1.88575	-0.86285
C	1.01209	2.03707	-0.51543
C	-1.63288	-1.78775	-0.48554
C	-1.37869	2.16020	-0.10406
C	-2.59018	-1.17315	0.52466
C	-2.53292	1.17696	-0.03959
H	2.19985	-2.12270	0.67940
H	2.87869	-1.61461	-0.89218
H	1.79356	0.46312	-1.72782
H	3.05025	1.36110	-0.84912
H	0.53694	-1.29130	-1.77540
H	0.79829	-2.95158	-1.15798
H	1.22600	2.58787	0.42306
H	1.00722	2.76109	-1.35383

H	-1.90452	-2.84489	-0.67666
H	-1.67002	-1.24131	-1.44764
H	-1.56581	2.92568	-0.88211
H	-1.23699	2.67096	0.87001
H	-3.62330	-1.16930	0.12973
H	-2.57569	-1.75861	1.45889
H	-3.46060	1.67823	0.29567
H	-2.70463	0.75509	-1.04584
C	2.97724	0.16915	1.34238
H	2.81329	-0.50829	2.19791
H	2.88680	1.20737	1.70499
H	4.01033	0.01936	0.96032
Li	-0.07701	0.00540	1.05587

ACE2 Li SCS-MP2 def2tzv

33

O	-0.34184	-1.74193	0.07955
O	-0.22758	1.37430	-0.41017
O	-2.20325	0.16457	0.91048
N	1.98445	-0.09055	0.32518
C	2.04976	-1.48194	-0.22581
C	2.08952	0.95444	-0.74935
C	0.74042	-1.87536	-0.91702
C	1.06597	2.07116	-0.51257
C	-1.69726	-1.80815	-0.49692
C	-1.40209	2.20735	-0.11151
C	-2.64700	-1.19773	0.53631
C	-2.56880	1.22440	-0.06008
H	2.23076	-2.16245	0.61241
H	2.87950	-1.59597	-0.94437
H	1.88607	0.50091	-1.72445

H	3.10415	1.37927	-0.78413
H	0.51546	-1.21729	-1.76456
H	0.79251	-2.91165	-1.26954
H	1.26109	2.61208	0.42235
H	1.04835	2.77742	-1.35028
H	-1.97257	-2.84915	-0.70016
H	-1.71016	-1.23922	-1.43399
H	-1.55947	2.94973	-0.90156
H	-1.25996	2.71282	0.85137
H	-3.66899	-1.16732	0.14337
H	-2.63071	-1.76700	1.46674
H	-3.48531	1.71828	0.27534
H	-2.73007	0.78609	-1.05030
C	3.02530	0.11629	1.37886
H	2.83684	-0.56651	2.21110
H	2.96099	1.14371	1.74681
H	4.04027	-0.05988	0.98799
Li	-0.06116	-0.01610	1.07980

ACE2 Na B2PLYP def2sv

33

O	-0.32402	-1.71116	-0.03483
O	-0.27322	1.34693	-0.52558
O	-2.31935	0.13316	0.76589
N	2.07726	-0.05902	0.18968
C	2.05542	-1.43777	-0.30663
C	2.02706	0.96295	-0.86816
C	0.74201	-1.84188	-0.96091
C	0.96807	2.02225	-0.59609
C	-1.61413	-1.76584	-0.61704
C	-1.41518	2.12138	-0.22479

C	-2.62792	-1.19663	0.36374
C	-2.59017	1.16152	-0.18260
H	2.23238	-2.10342	0.55404
H	2.87208	-1.63151	-1.03629
H	1.78870	0.49146	-1.83362
H	3.01070	1.45371	-0.99393
H	0.53319	-1.21777	-1.84810
H	0.82604	-2.89017	-1.30606
H	1.17187	2.55423	0.35477
H	0.96184	2.77204	-1.41076
H	-1.89410	-2.80866	-0.86318
H	-1.61992	-1.18616	-1.55779
H	-1.59442	2.89688	-0.99409
H	-1.29272	2.63146	0.75151
H	-3.63882	-1.25166	-0.07875
H	-2.62795	-1.79375	1.28860
H	-3.50999	1.70232	0.10317
H	-2.74166	0.73181	-1.18611
C	3.19134	0.16639	1.10621
H	3.12456	-0.52850	1.95874
H	3.14572	1.19349	1.50180
H	4.17990	0.02745	0.61714
Na	-0.05041	0.00583	1.52044

ACE2 Na B2PLYP def2tzv

33

O	-0.34323	-1.77743	-0.02841
O	-0.26908	1.35328	-0.55508
O	-2.34467	0.14845	0.80950
N	2.09645	-0.06567	0.21030
C	2.06099	-1.45162	-0.33030

C	2.07472	0.98620	-0.84842
C	0.74685	-1.81425	-1.01081
C	1.01816	2.05158	-0.57788
C	-1.67604	-1.76477	-0.63609
C	-1.43735	2.16072	-0.21086
C	-2.66973	-1.21996	0.37816
C	-2.61808	1.20772	-0.17753
H	2.22747	-2.13403	0.50399
H	2.87047	-1.61816	-1.05788
H	1.86185	0.53451	-1.81734
H	3.05451	1.46978	-0.92965
H	0.51170	-1.11689	-1.81662
H	0.82401	-2.81958	-1.43166
H	1.18553	2.54315	0.38564
H	1.02236	2.80677	-1.36787
H	-1.96796	-2.77823	-0.92268
H	-1.65094	-1.14171	-1.53233
H	-1.60705	2.93735	-0.96059
H	-1.28773	2.63545	0.76314
H	-3.67602	-1.25171	-0.04427
H	-2.65076	-1.81140	1.29043
H	-3.52363	1.73790	0.11797
H	-2.76910	0.77067	-1.16456
C	3.23983	0.11738	1.13865
H	3.15970	-0.59109	1.96174
H	3.21579	1.12553	1.54947
H	4.20637	-0.03180	0.63696
Na	-0.04199	-0.02175	1.47331

ACE2 Na B3LYP def2sv

33

O	-0.32243	-1.72070	-0.03625
O	-0.27598	1.36495	-0.51652
O	-2.33092	0.13143	0.76151
N	2.09063	-0.05711	0.18408
C	2.06563	-1.44191	-0.30312
C	2.02762	0.96784	-0.87367
C	0.75055	-1.86159	-0.95451
C	0.96885	2.03327	-0.59781
C	-1.61605	-1.78292	-0.61230
C	-1.42491	2.13242	-0.22302
C	-2.63363	-1.20227	0.36471
C	-2.60016	1.16551	-0.18332
H	2.24829	-2.10045	0.56353
H	2.88136	-1.64375	-1.03389
H	1.78268	0.49424	-1.83794
H	3.00994	1.46134	-1.00952
H	0.54263	-1.25509	-1.85637
H	0.84195	-2.91652	-1.28224
H	1.18025	2.57175	0.34973
H	0.96252	2.78007	-1.41735
H	-1.89735	-2.83048	-0.84417
H	-1.62600	-1.21690	-1.56335
H	-1.60702	2.90610	-0.99589
H	-1.30979	2.64874	0.75310
H	-3.64444	-1.26474	-0.08084
H	-2.63681	-1.79361	1.29546
H	-3.52090	1.70677	0.10515
H	-2.75429	0.73968	-1.18998
C	3.19709	0.17716	1.10973
H	3.13177	-0.51905	1.96354

H	3.14229	1.20452	1.50819
H	4.19331	0.04709	0.62902
Na	-0.04819	0.00451	1.51139

ACE2 Na B3LYP def2tzv

33

O	-0.34004	-1.77951	-0.02509
O	-0.27295	1.37456	-0.55296
O	-2.34722	0.14453	0.80167
N	2.10337	-0.06407	0.20255
C	2.06783	-1.45627	-0.31880
C	2.06484	0.98490	-0.85677
C	0.75668	-1.84170	-0.99430
C	1.01745	2.05895	-0.57981
C	-1.67136	-1.78147	-0.62849
C	-1.44409	2.16870	-0.20458
C	-2.66805	-1.22310	0.37576
C	-2.61944	1.20836	-0.17734
H	2.23795	-2.12587	0.52517
H	2.87805	-1.63725	-1.04218
H	1.83385	0.52912	-1.81923
H	3.04440	1.46424	-0.96147
H	0.52710	-1.17230	-1.82556
H	0.84200	-2.85861	-1.38557
H	1.19445	2.55375	0.38076
H	1.02882	2.81442	-1.37014
H	-1.96245	-2.80104	-0.89583
H	-1.65312	-1.17878	-1.53915
H	-1.62239	2.95048	-0.94784
H	-1.30014	2.64205	0.77171
H	-3.67315	-1.26307	-0.04954

H	-2.65488	-1.80987	1.29191
H	-3.52800	1.73467	0.11802
H	-2.76869	0.77824	-1.16785
C	3.23309	0.13739	1.13903
H	3.16150	-0.57262	1.96217
H	3.19140	1.14347	1.55445
H	4.20895	0.00677	0.64896
Na	-0.04083	-0.01824	1.45602

ACE2 Na B97-1 def2sv

33

O	-0.31985	-1.71950	-0.03782
O	-0.28008	1.35822	-0.52729
O	-2.33525	0.12946	0.76330
N	2.09000	-0.05516	0.18325
C	2.06797	-1.43997	-0.30563
C	2.02735	0.96969	-0.87549
C	0.74915	-1.85793	-0.95728
C	0.96059	2.03088	-0.59944
C	-1.60995	-1.77773	-0.61656
C	-1.42447	2.12606	-0.22680
C	-2.63192	-1.20197	0.36389
C	-2.60400	1.15974	-0.18194
H	2.25090	-2.10020	0.56200
H	2.88429	-1.64049	-1.03856
H	1.78669	0.49517	-1.84259
H	3.00936	1.46845	-1.00732
H	0.54124	-1.24637	-1.85837
H	0.84042	-2.91328	-1.28967
H	1.16743	2.56632	0.35324
H	0.95594	2.78298	-1.41659

H	-1.89210	-2.82500	-0.85655
H	-1.61910	-1.20335	-1.56511
H	-1.61038	2.90263	-0.99838
H	-1.30412	2.64140	0.75133
H	-3.64373	-1.26847	-0.08333
H	-2.63264	-1.79737	1.29401
H	-3.52375	1.70512	0.10788
H	-2.76144	0.73272	-1.18972
C	3.19902	0.18027	1.10669
H	3.13559	-0.51811	1.96093
H	3.14183	1.20889	1.50619
H	4.19564	0.05213	0.62286
Na	-0.04300	0.00575	1.53407

ACE2 Na B97-1 def2tzv

33

O	-0.33494	-1.77659	-0.02648
O	-0.27957	1.36514	-0.56365
O	-2.35582	0.14046	0.80592
N	2.10298	-0.05982	0.20464
C	2.07297	-1.45145	-0.32023
C	2.06341	0.98786	-0.85710
C	0.75758	-1.83703	-0.99613
C	1.00480	2.05640	-0.58149
C	-1.66164	-1.77569	-0.63369
C	-1.44632	2.15955	-0.20990
C	-2.66575	-1.22473	0.37393
C	-2.62664	1.19878	-0.17524
H	2.24524	-2.12410	0.52436
H	2.88392	-1.62859	-1.04755
H	1.83772	0.52958	-1.82283

H	3.04205	1.47552	-0.95753
H	0.52845	-1.16364	-1.82837
H	0.84463	-2.85529	-1.39116
H	1.17675	2.54891	0.38463
H	1.01700	2.81717	-1.37042
H	-1.95336	-2.79555	-0.90997
H	-1.64289	-1.16406	-1.54204
H	-1.62957	2.94308	-0.95396
H	-1.29796	2.63432	0.76819
H	-3.67152	-1.27108	-0.05603
H	-2.64998	-1.81839	1.28870
H	-3.53443	1.73153	0.12030
H	-2.77946	0.76597	-1.16716
C	3.24086	0.14259	1.13229
H	3.17566	-0.56954	1.95736
H	3.20022	1.15112	1.54846
H	4.21477	0.01279	0.63291
Na	-0.03646	-0.01552	1.47898

ACE2 Na CAM-B3LYP def2sv

33

O	-0.32208	-1.70689	-0.03380
O	-0.27330	1.35030	-0.51413
O	-2.30748	0.12897	0.76123
N	2.07397	-0.05725	0.18778
C	2.05430	-1.43755	-0.29169
C	2.01828	0.95536	-0.87214
C	0.74631	-1.85282	-0.94510
C	0.96397	2.01743	-0.60403
C	-1.60672	-1.76664	-0.61214
C	-1.41273	2.11908	-0.21956

C	-2.61998	-1.19473	0.36409
C	-2.58506	1.15909	-0.17354
H	2.23023	-2.09341	0.57650
H	2.87394	-1.63820	-1.01510
H	1.77427	0.47678	-1.83244
H	3.00155	1.44335	-1.00735
H	0.54279	-1.24499	-1.84524
H	0.83308	-2.90642	-1.27223
H	1.17672	2.56099	0.33845
H	0.95223	2.75790	-1.42672
H	-1.88506	-2.81065	-0.85405
H	-1.61320	-1.19114	-1.55596
H	-1.59295	2.89151	-0.99136
H	-1.29126	2.63321	0.75499
H	-3.63022	-1.24724	-0.07970
H	-2.62461	-1.79256	1.28876
H	-3.50296	1.69834	0.12086
H	-2.74414	0.73442	-1.17843
C	3.17281	0.18163	1.11183
H	3.10443	-0.50688	1.96968
H	3.11895	1.21089	1.50138
H	4.16702	0.04598	0.63342
Na	-0.05208	0.01042	1.48249

ACE2 Na CAM-B3LYP def2tzv

33

O	-0.33778	-1.76513	-0.02595
O	-0.27140	1.36143	-0.54869
O	-2.32081	0.14110	0.79705
N	2.08581	-0.06333	0.20324
C	2.05564	-1.44974	-0.30675

C	2.05216	0.97195	-0.85537
C	0.75318	-1.83237	-0.98392
C	1.00910	2.04118	-0.58601
C	-1.65792	-1.76395	-0.62714
C	-1.43155	2.15297	-0.20091
C	-2.64871	-1.21455	0.37692
C	-2.60232	1.19866	-0.16774
H	2.21888	-2.11569	0.53986
H	2.87063	-1.63106	-1.02166
H	1.82292	0.51204	-1.81475
H	3.03195	1.44754	-0.95990
H	0.52852	-1.16290	-1.81499
H	0.83596	-2.84802	-1.37472
H	1.18918	2.54185	0.36954
H	1.01588	2.79089	-1.37965
H	-1.94754	-2.77915	-0.90526
H	-1.63751	-1.15167	-1.53008
H	-1.60875	2.93413	-0.94257
H	-1.28306	2.62452	0.77400
H	-3.65426	-1.24928	-0.04393
H	-2.63332	-1.80667	1.28792
H	-3.50879	1.72236	0.13274
H	-2.75573	0.77124	-1.15763
C	3.20094	0.14555	1.14183
H	3.12406	-0.55490	1.97093
H	3.15855	1.15463	1.54647
H	4.17737	0.00866	0.65880
Na	-0.04444	-0.01478	1.42887

ACE2 Na DSDPBEP86 def2sv

33

O	-0.32050	-1.70521	-0.03298
O	-0.27509	1.32700	-0.54486
O	-2.30812	0.12995	0.77565
N	2.06160	-0.05787	0.19391
C	2.05102	-1.43454	-0.29875
C	2.02403	0.95417	-0.86953
C	0.74164	-1.83197	-0.95986
C	0.96143	2.00831	-0.60654
C	-1.60418	-1.74593	-0.62401
C	-1.40594	2.10652	-0.22423
C	-2.61722	-1.19412	0.36395
C	-2.58406	1.15469	-0.17127
H	2.21915	-2.10108	0.56588
H	2.87649	-1.62664	-1.02186
H	1.79267	0.47674	-1.83614
H	3.01108	1.44450	-0.98937
H	0.53492	-1.19666	-1.84204
H	0.82202	-2.87930	-1.31499
H	1.15753	2.53974	0.34874
H	0.95590	2.76127	-1.42094
H	-1.88459	-2.78457	-0.89457
H	-1.60334	-1.14339	-1.55275
H	-1.58949	2.89017	-0.98694
H	-1.26639	2.60864	0.75615
H	-3.63178	-1.24551	-0.07571
H	-2.61143	-1.80293	1.28339
H	-3.50174	1.69937	0.12092
H	-2.74405	0.72302	-1.17485
C	3.16650	0.17479	1.11622
H	3.09068	-0.51405	1.97544

H	3.11565	1.20739	1.50240
H	4.16018	0.03123	0.63519
Na	-0.04585	0.01379	1.51148

ACE2 Na DSDPBEP86 def2tzv

33

O	-0.33920	-1.77359	-0.03105
O	-0.27246	1.32765	-0.57413
O	-2.33838	0.14441	0.82161
N	2.08418	-0.06260	0.21709
C	2.05807	-1.44486	-0.32582
C	2.07601	0.98152	-0.84597
C	0.74585	-1.79542	-1.01483
C	1.00727	2.03646	-0.58859
C	-1.66498	-1.74021	-0.64658
C	-1.42843	2.14242	-0.21219
C	-2.66013	-1.21802	0.37786
C	-2.61659	1.19961	-0.16373
H	2.21679	-2.13297	0.50993
H	2.87567	-1.60649	-1.05041
H	1.88146	0.52473	-1.82040
H	3.05682	1.47307	-0.91161
H	0.51068	-1.07832	-1.80803
H	0.82047	-2.79557	-1.45662
H	1.16367	2.52924	0.38039
H	1.00932	2.79467	-1.38015
H	-1.95947	-2.74670	-0.96551
H	-1.63094	-1.08735	-1.52542
H	-1.60109	2.92575	-0.95880
H	-1.25993	2.61192	0.76513
H	-3.67046	-1.24770	-0.04372

H	-2.63406	-1.82395	1.28428
H	-3.51775	1.73866	0.14053
H	-2.78127	0.76023	-1.15148
C	3.22326	0.12148	1.14806
H	3.13406	-0.58229	1.97860
H	3.20084	1.13662	1.55065
H	4.19323	-0.03753	0.64913
Na	-0.03990	-0.01849	1.47210

ACE2 Na HSE06 def2sv

33

O	-0.31731	-1.70249	-0.03538
O	-0.27771	1.34087	-0.52289
O	-2.31840	0.12698	0.76035
N	2.07607	-0.05502	0.18178
C	2.05532	-1.43166	-0.29702
C	2.01304	0.95642	-0.87417
C	0.74715	-1.84527	-0.94674
C	0.95499	2.01152	-0.60384
C	-1.59769	-1.75906	-0.61610
C	-1.41398	2.10833	-0.22558
C	-2.61497	-1.19537	0.35834
C	-2.58667	1.15133	-0.17914
H	2.23373	-2.08858	0.57196
H	2.87404	-1.63733	-1.02307
H	1.76889	0.47724	-1.83632
H	2.99453	1.45015	-1.01443
H	0.54298	-1.23502	-1.84774
H	0.83571	-2.89857	-1.27956
H	1.16445	2.55341	0.34246
H	0.94650	2.75813	-1.42315

H	-1.87718	-2.80220	-0.86743
H	-1.60387	-1.17766	-1.55862
H	-1.59737	2.88415	-0.99550
H	-1.29209	2.62410	0.75002
H	-3.62536	-1.26028	-0.08753
H	-2.61719	-1.79569	1.28307
H	-3.50549	1.69601	0.10720
H	-2.74380	0.72384	-1.18500
C	3.17305	0.18660	1.10106
H	3.11124	-0.50582	1.95779
H	3.11350	1.21480	1.49588
H	4.17002	0.05964	0.62221
Na	-0.04544	0.01135	1.52707

ACE2 Na HSE06 def2tzv

33

O	-0.33055	-1.76364	-0.02484
O	-0.27978	1.34802	-0.55882
O	-2.33862	0.13567	0.80254
N	2.08880	-0.05736	0.20186
C	2.06070	-1.44069	-0.30946
C	2.04573	0.97553	-0.85635
C	0.75799	-1.82468	-0.98318
C	0.99491	2.03592	-0.58751
C	-1.64491	-1.75380	-0.63435
C	-1.43676	2.13958	-0.20657
C	-2.64517	-1.21815	0.36653
C	-2.60917	1.18815	-0.16912
H	2.22987	-2.10912	0.53768
H	2.87459	-1.62356	-1.03021
H	1.81874	0.51237	-1.81827

H	3.02312	1.46140	-0.96501
H	0.53233	-1.15178	-1.81560
H	0.84612	-2.84046	-1.38077
H	1.17069	2.53659	0.37245
H	1.00354	2.79135	-1.37978
H	-1.93693	-2.76688	-0.92939
H	-1.62040	-1.12931	-1.53274
H	-1.61994	2.92361	-0.94813
H	-1.28603	2.61470	0.76967
H	-3.64912	-1.26445	-0.06455
H	-2.63212	-1.81938	1.27496
H	-3.51619	1.72089	0.12401
H	-2.76321	0.75699	-1.16050
C	3.21118	0.15414	1.12673
H	3.14715	-0.55031	1.95676
H	3.16790	1.16384	1.53622
H	4.18724	0.02437	0.63445
Na	-0.03767	-0.01325	1.47038

ACE2 Na M062X def2sv

33

O	-0.32566	-1.68631	-0.03398
O	-0.27294	1.30654	-0.53522
O	-2.30213	0.12812	0.77825
N	2.06736	-0.06236	0.19776
C	2.04476	-1.43597	-0.29984
C	2.03122	0.95195	-0.86221
C	0.73039	-1.81153	-0.96140
C	0.95579	1.99398	-0.60763
C	-1.60650	-1.73421	-0.61972
C	-1.40093	2.09191	-0.23230

C	-2.61779	-1.19143	0.37400
C	-2.58498	1.14833	-0.16398
H	2.20648	-2.10795	0.55956
H	2.86611	-1.62775	-1.02357
H	1.81863	0.47528	-1.83219
H	3.01197	1.45230	-0.96551
H	0.52679	-1.15879	-1.83091
H	0.79588	-2.85202	-1.33164
H	1.14780	2.53357	0.34237
H	0.93864	2.73855	-1.42576
H	-1.87723	-2.77114	-0.89584
H	-1.61127	-1.12473	-1.54331
H	-1.57462	2.86335	-1.00558
H	-1.25936	2.60525	0.74028
H	-3.63271	-1.23982	-0.05922
H	-2.60557	-1.80277	1.28965
H	-3.49334	1.69867	0.13630
H	-2.75782	0.71320	-1.16290
C	3.17364	0.16006	1.11863
H	3.09831	-0.53350	1.97140
H	3.12934	1.18892	1.51050
H	4.16151	0.01465	0.63155
Na	-0.04626	0.03145	1.48771

ACE2 Na M062X def2tzv

33

O	-0.33847	-1.73507	-0.02698
O	-0.27130	1.31597	-0.56074
O	-2.31935	0.14057	0.80980
N	2.08024	-0.06725	0.20954
C	2.04994	-1.44969	-0.31209

C	2.06222	0.96697	-0.85201
C	0.74030	-1.79745	-0.99557
C	0.99892	2.01733	-0.58808
C	-1.65466	-1.73348	-0.63201
C	-1.42150	2.12499	-0.21366
C	-2.64565	-1.21241	0.38839
C	-2.60425	1.18452	-0.16476
H	2.19838	-2.12696	0.52954
H	2.86737	-1.62521	-1.02564
H	1.85534	0.50731	-1.81792
H	3.03910	1.45351	-0.93293
H	0.52115	-1.09712	-1.80421
H	0.80053	-2.80396	-1.41410
H	1.16519	2.51042	0.37518
H	0.99320	2.77393	-1.37534
H	-1.93223	-2.74428	-0.93731
H	-1.63726	-1.09312	-1.51707
H	-1.58666	2.90429	-0.95967
H	-1.26070	2.59570	0.76036
H	-3.65584	-1.24921	-0.02213
H	-2.61068	-1.81549	1.29245
H	-3.50494	1.71826	0.13543
H	-2.76367	0.74648	-1.15013
C	3.20032	0.13350	1.14710
H	3.11459	-0.56538	1.97721
H	3.16453	1.14593	1.54593
H	4.17239	-0.01521	0.65936
Na	-0.03839	0.00668	1.43397

ACE2 Na M06 def2sv

33

O	-0.30478	-1.69134	-0.04614
O	-0.29356	1.33349	-0.59642
O	-2.32741	0.11219	0.75749
N	2.07787	-0.04619	0.18334
C	2.06247	-1.41937	-0.30309
C	2.01118	0.97108	-0.86707
C	0.76026	-1.82538	-0.95624
C	0.94056	2.00315	-0.59264
C	-1.57702	-1.73283	-0.64285
C	-1.41259	2.08733	-0.22006
C	-2.60451	-1.20609	0.33270
C	-2.59243	1.14816	-0.16786
H	2.24238	-2.08470	0.56374
H	2.88894	-1.61522	-1.02828
H	1.78660	0.49865	-1.84151
H	2.99143	1.47717	-0.99408
H	0.55552	-1.20165	-1.85253
H	0.84227	-2.87599	-1.30845
H	1.11095	2.49203	0.39580
H	0.95993	2.80306	-1.36443
H	-1.84714	-2.76920	-0.94018
H	-1.57156	-1.11630	-1.56797
H	-1.61296	2.90988	-0.94027
H	-1.24728	2.55612	0.77780
H	-3.61525	-1.27628	-0.11817
H	-2.60416	-1.82627	1.24787
H	-3.50333	1.70328	0.13230
H	-2.77217	0.73235	-1.17896
C	3.18411	0.19320	1.08899
H	3.13371	-0.49675	1.95118

H	3.13512	1.22558	1.48151
H	4.17650	0.06028	0.59551
Na	-0.07462	-0.02027	1.61409

ACE2 Na M06 def2tzv

33

O	-0.31106	-1.75100	-0.04357
O	-0.30099	1.36287	-0.64591
O	-2.31994	0.11628	0.78799
N	2.07355	-0.04568	0.19115
C	2.06995	-1.43200	-0.30459
C	2.02469	0.98072	-0.86975
C	0.77907	-1.82836	-0.98709
C	0.97498	2.03247	-0.58564
C	-1.61194	-1.73471	-0.66598
C	-1.43304	2.12553	-0.19261
C	-2.61859	-1.22679	0.33935
C	-2.60603	1.17968	-0.15508
H	2.23065	-2.08939	0.55381
H	2.89803	-1.61229	-1.00972
H	1.78838	0.51711	-1.82981
H	3.00289	1.46383	-0.98654
H	0.56357	-1.16710	-1.83364
H	0.86895	-2.84895	-1.37326
H	1.12221	2.46777	0.41302
H	1.01914	2.84103	-1.32319
H	-1.89576	-2.73990	-0.99489
H	-1.58222	-1.08246	-1.54667
H	-1.64474	2.96196	-0.86711
H	-1.23649	2.53408	0.80733
H	-3.62294	-1.27560	-0.09083

H	-2.59790	-1.84398	1.23765
H	-3.50807	1.70900	0.15894
H	-2.77644	0.76882	-1.15378
C	3.18177	0.19372	1.12265
H	3.12289	-0.50339	1.95937
H	3.11837	1.20811	1.51925
H	4.16298	0.07414	0.63709
Na	-0.05977	-0.05725	1.58085

ACE2 Na MP2 def2sv

33

O	-0.32604	-1.70233	-0.03578
O	-0.27115	1.32140	-0.54559
O	-2.31464	0.13600	0.77182
N	2.06526	-0.06097	0.19331
C	2.04353	-1.42982	-0.31910
C	2.03011	0.96037	-0.86064
C	0.72897	-1.80971	-0.97589
C	0.96481	2.00808	-0.59224
C	-1.61158	-1.74025	-0.62766
C	-1.40251	2.10527	-0.22905
C	-2.62363	-1.18945	0.35930
C	-2.58148	1.15660	-0.18373
H	2.21335	-2.11121	0.53312
H	2.86275	-1.61461	-1.05132
H	1.80541	0.49461	-1.83431
H	3.01665	1.45414	-0.97079
H	0.51616	-1.15480	-1.84121
H	0.80456	-2.84904	-1.35494
H	1.15585	2.52823	0.36973
H	0.96243	2.77013	-1.39828

H	-1.89358	-2.77764	-0.90116
H	-1.61076	-1.13587	-1.55424
H	-1.58032	2.88988	-0.99211
H	-1.26852	2.60591	0.75222
H	-3.63788	-1.24093	-0.08097
H	-2.61826	-1.79814	1.27826
H	-3.50164	1.70330	0.09674
H	-2.73147	0.72253	-1.18709
C	3.18986	0.14868	1.09817
H	3.12084	-0.54824	1.95034
H	3.15769	1.17702	1.49501
H	4.17183	-0.00173	0.59747
Na	-0.04870	0.00128	1.56379

ACE2 Na MP2 def2tzv

33

O	-0.36323	-1.77860	-0.04569
O	-0.25109	1.31833	-0.56121
O	-2.35494	0.16719	0.82539
N	2.10302	-0.08027	0.22042
C	2.03728	-1.44561	-0.37937
C	2.11561	0.99766	-0.81894
C	0.70501	-1.73407	-1.06400
C	1.03302	2.04236	-0.55705
C	-1.70807	-1.74002	-0.64677
C	-1.41635	2.15244	-0.22656
C	-2.69042	-1.20609	0.38917
C	-2.61562	1.21799	-0.18387
H	2.19102	-2.17134	0.42576
H	2.84095	-1.58997	-1.12482
H	1.95362	0.56802	-1.81338

H	3.09525	1.49779	-0.83835
H	0.45482	-0.95661	-1.79302
H	0.75771	-2.70177	-1.57717
H	1.17555	2.51970	0.42194
H	1.03272	2.80989	-1.34036
H	-2.01077	-2.74737	-0.95661
H	-1.67930	-1.08997	-1.52801
H	-1.56878	2.92387	-0.99032
H	-1.25760	2.63236	0.74730
H	-3.70481	-1.22627	-0.02496
H	-2.65934	-1.80224	1.30203
H	-3.51551	1.76787	0.10622
H	-2.77285	0.76746	-1.16837
C	3.27593	0.03418	1.13527
H	3.17463	-0.69133	1.94550
H	3.29714	1.03849	1.56445
H	4.22620	-0.14687	0.60628
Na	-0.03782	-0.03711	1.53253

ACE2 Na PBE0 def2sv

33

O	-0.31733	-1.70336	-0.03496
O	-0.27777	1.34149	-0.52350
O	-2.31665	0.12689	0.76014
N	2.07454	-0.05498	0.18157
C	2.05466	-1.43163	-0.29686
C	2.01239	0.95632	-0.87445
C	0.74663	-1.84697	-0.94581
C	0.95440	2.01167	-0.60478
C	-1.59721	-1.76008	-0.61528
C	-1.41347	2.10841	-0.22565

C	-2.61422	-1.19460	0.35856
C	-2.58595	1.15105	-0.17819
H	2.23373	-2.08799	0.57241
H	2.87326	-1.63712	-1.02299
H	1.76907	0.47709	-1.83670
H	2.99397	1.45002	-1.01390
H	0.54229	-1.23874	-1.84808
H	0.83629	-2.90090	-1.27659
H	1.16412	2.55420	0.34113
H	0.94632	2.75787	-1.42455
H	-1.87749	-2.80351	-0.86478
H	-1.60358	-1.18021	-1.55871
H	-1.59811	2.88399	-0.99558
H	-1.29122	2.62440	0.74983
H	-3.62453	-1.25897	-0.08772
H	-2.61755	-1.79468	1.28352
H	-3.50442	1.69574	0.10960
H	-2.74445	0.72431	-1.18411
C	3.17079	0.18684	1.10151
H	3.10832	-0.50545	1.95836
H	3.11066	1.21508	1.49626
H	4.16815	0.05999	0.62353
Na	-0.04370	0.01305	1.52548

ACE2 Na PBE0 def2tzv

33

O	-0.33047	-1.76349	-0.02502
O	-0.28004	1.34786	-0.55993
O	-2.33788	0.13544	0.80257
N	2.08759	-0.05721	0.20212
C	2.05996	-1.43990	-0.31049

C	2.04554	0.97610	-0.85546
C	0.75659	-1.82450	-0.98318
C	0.99329	2.03578	-0.58782
C	-1.64386	-1.75402	-0.63386
C	-1.43599	2.13863	-0.20714
C	-2.64438	-1.21711	0.36669
C	-2.60873	1.18706	-0.16807
H	2.23045	-2.10909	0.53617
H	2.87328	-1.62171	-1.03250
H	1.82077	0.51324	-1.81835
H	3.02284	1.46319	-0.96193
H	0.53063	-1.15205	-1.81630
H	0.84562	-2.84069	-1.38061
H	1.16857	2.53725	0.37222
H	1.00285	2.79117	-1.38064
H	-1.93689	-2.76756	-0.92774
H	-1.61983	-1.13044	-1.53326
H	-1.62081	2.92257	-0.94892
H	-1.28488	2.61452	0.76907
H	-3.64835	-1.26373	-0.06526
H	-2.63203	-1.81890	1.27520
H	-3.51530	1.72090	0.12592
H	-2.76423	0.75639	-1.15976
C	3.21065	0.15291	1.12628
H	3.14690	-0.55273	1.95576
H	3.16784	1.16239	1.53719
H	4.18659	0.02343	0.63317
Na	-0.03623	-0.01235	1.47119

ACE2 Na PBE def2sv

33

O	-0.31849	-1.72309	-0.03370
O	-0.28319	1.35025	-0.54022
O	-2.34615	0.12967	0.77477
N	2.09453	-0.05470	0.18463
C	2.07062	-1.43917	-0.30917
C	2.03058	0.97281	-0.87323
C	0.75287	-1.85321	-0.96187
C	0.96193	2.03098	-0.59940
C	-1.61252	-1.77446	-0.62022
C	-1.42937	2.12405	-0.22878
C	-2.63533	-1.20761	0.36254
C	-2.60978	1.16110	-0.18266
H	2.25503	-2.10543	0.56250
H	2.89259	-1.64400	-1.04667
H	1.79033	0.49494	-1.84708
H	3.01797	1.47650	-1.00776
H	0.53980	-1.22911	-1.86315
H	0.84608	-2.91145	-1.30988
H	1.16213	2.56595	0.36416
H	0.96303	2.79426	-1.41664
H	-1.89950	-2.82510	-0.87612
H	-1.61759	-1.18737	-1.57041
H	-1.61995	2.91178	-0.99916
H	-1.30218	2.63959	0.75744
H	-3.65405	-1.28085	-0.08657
H	-2.63237	-1.80942	1.29708
H	-3.53543	1.71511	0.10130
H	-2.76651	0.72591	-1.19505
C	3.20516	0.17907	1.10731
H	3.14629	-0.52864	1.96286

H	3.14518	1.21071	1.51669
H	4.20949	0.05758	0.61979
Na	-0.03859	0.00872	1.54276

ACE2 Na PBE def2tzv

33

O	-0.32672	-1.78819	-0.01875
O	-0.29102	1.35082	-0.58397
O	-2.37107	0.13358	0.82516
N	2.10626	-0.05235	0.20823
C	2.08214	-1.44542	-0.32034
C	2.06104	0.99614	-0.85642
C	0.77213	-1.83402	-0.99820
C	0.99780	2.05740	-0.58575
C	-1.65591	-1.76543	-0.64483
C	-1.45982	2.15311	-0.21001
C	-2.66558	-1.23805	0.36455
C	-2.63954	1.19701	-0.16941
H	2.25967	-2.12212	0.52904
H	2.90065	-1.62220	-1.05150
H	1.83879	0.53111	-1.82800
H	3.04361	1.49187	-0.95965
H	0.53470	-1.14969	-1.82931
H	0.86560	-2.85426	-1.40701
H	1.15773	2.54950	0.39109
H	1.01313	2.82801	-1.37547
H	-1.95153	-2.78265	-0.95559
H	-1.62271	-1.12517	-1.54220
H	-1.65076	2.94778	-0.95163
H	-1.29730	2.62673	0.77489
H	-3.67621	-1.28941	-0.07232

H	-2.64921	-1.84516	1.27908
H	-3.55177	1.73733	0.12646
H	-2.79770	0.75558	-1.16448
C	3.24895	0.15465	1.13137
H	3.19364	-0.56556	1.95881
H	3.20306	1.16670	1.55529
H	4.22899	0.03435	0.62480
Na	-0.02935	-0.01532	1.49045

ACE2 Na SCS-MP2 def2sv

33

O	-0.32921	-1.70854	-0.03535
O	-0.26967	1.33135	-0.54294
O	-2.31708	0.13845	0.77208
N	2.07003	-0.06301	0.19428
C	2.04669	-1.43549	-0.32217
C	2.03679	0.96484	-0.86066
C	0.72750	-1.81794	-0.97871
C	0.97048	2.01722	-0.58983
C	-1.61991	-1.75176	-0.62403
C	-1.40559	2.11549	-0.22901
C	-2.63156	-1.19046	0.36426
C	-2.58583	1.16154	-0.18553
H	2.21853	-2.11807	0.52974
H	2.86480	-1.61869	-1.05671
H	1.81263	0.50116	-1.83625
H	3.02455	1.45816	-0.96776
H	0.51330	-1.16602	-1.84637
H	0.80289	-2.85979	-1.35294
H	1.16213	2.53690	0.37285
H	0.96850	2.77908	-1.39697

H	-1.90188	-2.79325	-0.88446
H	-1.62246	-1.15718	-1.55736
H	-1.58261	2.89827	-0.99510
H	-1.27437	2.61762	0.75249
H	-3.64728	-1.23938	-0.07473
H	-2.62723	-1.79490	1.28712
H	-3.50778	1.70594	0.09674
H	-2.73498	0.72726	-1.18965
C	3.19875	0.14237	1.10257
H	3.12613	-0.55590	1.95482
H	3.16926	1.17147	1.50101
H	4.18120	-0.01094	0.60209
Na	-0.04835	0.00313	1.55363

ACE2 Na SCS-MP2 def2tzv

33

O	-0.36328	-1.77972	-0.04178
O	-0.25206	1.32978	-0.55946
O	-2.35429	0.16751	0.82177
N	2.10424	-0.08052	0.22032
C	2.04252	-1.45042	-0.37697
C	2.11536	0.99898	-0.82258
C	0.70633	-1.75049	-1.05942
C	1.03395	2.04980	-0.55548
C	-1.70918	-1.75312	-0.64170
C	-1.41974	2.16066	-0.22676
C	-2.69440	-1.20619	0.39183
C	-2.61865	1.21837	-0.18715
H	2.20112	-2.17274	0.43116
H	2.84525	-1.59495	-1.12366
H	1.94609	0.56851	-1.81581

H	3.09652	1.49659	-0.84791
H	0.45728	-0.98572	-1.80259
H	0.76082	-2.72730	-1.55648
H	1.18043	2.52607	0.42413
H	1.03554	2.81893	-1.33824
H	-2.00956	-2.76687	-0.93501
H	-1.68543	-1.11678	-1.53340
H	-1.57341	2.93231	-0.99103
H	-1.26579	2.64119	0.74834
H	-3.70898	-1.22606	-0.02355
H	-2.66679	-1.79862	1.30827
H	-3.52188	1.76434	0.10305
H	-2.77258	0.76851	-1.17300
C	3.28028	0.03853	1.13453
H	3.18011	-0.68417	1.94867
H	3.30107	1.04521	1.56058
H	4.23115	-0.14387	0.60602
Na	-0.03840	-0.03257	1.52421

ACE2 NoMetal B2PLYP def2sv

32

O	-0.30905	-1.72344	0.12224
O	-0.31631	1.35429	-0.58176
O	-2.39264	0.10304	0.96961
N	2.09070	-0.04909	0.34166
C	2.06362	-1.42832	-0.11412
C	2.03217	0.96190	-0.70913
C	0.76004	-1.82919	-0.78665
C	0.93684	1.98693	-0.45639
C	-1.57219	-1.69625	-0.49286
C	-1.38948	2.05534	-0.00506

C	-2.61512	-1.20867	0.49765
C	-2.60740	1.15568	0.04878
H	2.20394	-2.07609	0.76765
H	2.89631	-1.66559	-0.81896
H	1.84032	0.49110	-1.68677
H	3.00102	1.49271	-0.80985
H	0.56809	-1.18983	-1.66806
H	0.86001	-2.87043	-1.16003
H	1.05404	2.40708	0.56253
H	1.02549	2.82874	-1.17561
H	-1.86985	-2.70555	-0.85071
H	-1.54888	-1.03010	-1.37563
H	-1.63258	2.97108	-0.58465
H	-1.13470	2.37165	1.02645
H	-3.61726	-1.30383	0.03338
H	-2.59509	-1.85490	1.38995
H	-3.47888	1.75577	0.37401
H	-2.82712	0.77266	-0.96309
C	3.14086	0.20766	1.30579
H	3.04627	-0.47995	2.16288
H	3.05476	1.23685	1.69251
H	4.16717	0.09078	0.88367

ACE2 NoMetal B2PLYP def2tzv

32

O	-0.32273	-1.90540	0.10748
O	-0.32596	1.46274	-0.68879
O	-2.37255	0.11096	1.01381
N	2.07660	-0.05626	0.36654
C	2.07107	-1.44666	-0.12728
C	2.04801	0.97586	-0.69496

C	0.78734	-1.84497	-0.84270
C	0.99399	2.04650	-0.44031
C	-1.62904	-1.72337	-0.51658
C	-1.42011	2.12548	0.01462
C	-2.62757	-1.24413	0.52171
C	-2.61437	1.19453	0.05394
H	2.21164	-2.10521	0.73133
H	2.90891	-1.63508	-0.82300
H	1.83807	0.51786	-1.66245
H	3.02831	1.46506	-0.78397
H	0.54417	-1.12609	-1.62709
H	0.92592	-2.82642	-1.30757
H	1.05315	2.38828	0.59694
H	1.14581	2.90348	-1.10435
H	-1.98124	-2.67218	-0.93598
H	-1.54274	-0.99730	-1.32769
H	-1.69275	3.05328	-0.49891
H	-1.11175	2.36625	1.03594
H	-3.63431	-1.30262	0.09593
H	-2.58299	-1.87646	1.40575
H	-3.49581	1.74963	0.38189
H	-2.80742	0.79480	-0.94302
C	3.14961	0.18356	1.34891
H	3.05262	-0.51615	2.17830
H	3.06469	1.19466	1.74635
H	4.15589	0.07073	0.91170

ACE2 NoMetal B3LYP def2sv

32

O	-0.30984	-1.73503	0.11856
O	-0.31611	1.37962	-0.58579

O	-2.40767	0.10492	0.96344
N	2.10924	-0.05169	0.33235
C	2.07132	-1.43532	-0.11510
C	2.03749	0.96672	-0.71236
C	0.76409	-1.84621	-0.78506
C	0.94311	1.99881	-0.45223
C	-1.57821	-1.71549	-0.48702
C	-1.39469	2.06761	-0.00296
C	-2.62236	-1.21236	0.50176
C	-2.61392	1.16147	0.04390
H	2.21457	-2.07789	0.77178
H	2.90177	-1.68478	-0.82136
H	1.83853	0.49878	-1.69159
H	3.00581	1.49995	-0.82121
H	0.57246	-1.21830	-1.67731
H	0.87013	-2.89245	-1.14804
H	1.06091	2.41443	0.57051
H	1.04263	2.84634	-1.16617
H	-1.88070	-2.73128	-0.82817
H	-1.56130	-1.06467	-1.38389
H	-1.64489	2.98882	-0.57451
H	-1.14169	2.37927	1.03263
H	-3.62541	-1.31876	0.03740
H	-2.60258	-1.84973	1.40285
H	-3.48743	1.76326	0.36671
H	-2.83104	0.78229	-0.97198
C	3.14342	0.20995	1.31317
H	3.04515	-0.48274	2.16811
H	3.04568	1.23755	1.70645
H	4.18020	0.10456	0.90712

ACE2 NoMetal B3LYP def2tzv

32

O	-0.29295	-1.98783	0.09480
O	-0.34269	1.54676	-0.73124
O	-2.29341	0.09320	0.99935
N	2.02392	-0.04264	0.35298
C	2.08562	-1.44166	-0.10476
C	2.01165	0.97454	-0.71760
C	0.83459	-1.92306	-0.82997
C	0.99125	2.08181	-0.47256
C	-1.59501	-1.76784	-0.52083
C	-1.42992	2.15624	0.01950
C	-2.57615	-1.25733	0.52036
C	-2.59218	1.18570	0.07037
H	2.23681	-2.07174	0.77323
H	2.94559	-1.61971	-0.77586
H	1.77338	0.50671	-1.67298
H	3.00531	1.43368	-0.83291
H	0.58588	-1.25532	-1.65748
H	1.02526	-2.91776	-1.24593
H	1.05571	2.43320	0.56110
H	1.18187	2.92933	-1.13910
H	-1.97827	-2.70641	-0.93700
H	-1.49615	-1.04700	-1.33582
H	-1.75082	3.08668	-0.46158
H	-1.10142	2.38865	1.03686
H	-3.58839	-1.30617	0.10572
H	-2.53700	-1.88690	1.40759
H	-3.48397	1.70719	0.42705
H	-2.79584	0.80103	-0.93077

C	3.00508	0.25655	1.40661
H	2.88240	-0.43844	2.23714
H	2.84732	1.26501	1.78936
H	4.04779	0.18975	1.05049

ACE2 NoMetal B97-1 def2sv

32

O	-0.30546	-1.72927	0.12201
O	-0.32054	1.36942	-0.58392
O	-2.41590	0.09946	0.96656
N	2.10846	-0.04680	0.33490
C	2.07785	-1.43352	-0.10721
C	2.03444	0.96539	-0.71743
C	0.76883	-1.84976	-0.77726
C	0.93417	1.99578	-0.45910
C	-1.56793	-1.70780	-0.49232
C	-1.39547	2.06094	-0.00268
C	-2.62279	-1.21364	0.49475
C	-2.61976	1.15633	0.04935
H	2.22243	-2.07197	0.78462
H	2.91024	-1.68313	-0.81335
H	1.83486	0.49033	-1.69516
H	3.00238	1.50180	-0.82979
H	0.58113	-1.22769	-1.67712
H	0.87501	-2.90063	-1.13170
H	1.05528	2.41772	0.56287
H	1.02717	2.84085	-1.17929
H	-1.86658	-2.72291	-0.84374
H	-1.54641	-1.04770	-1.38496
H	-1.64597	2.98174	-0.57793
H	-1.14039	2.37631	1.03326

H	-3.62315	-1.32001	0.02072
H	-2.60771	-1.85865	1.39232
H	-3.49019	1.76274	0.37666
H	-2.84295	0.77831	-0.96770
C	3.14816	0.22378	1.30937
H	3.05514	-0.46620	2.16903
H	3.04648	1.25469	1.69756
H	4.18412	0.12004	0.89741

ACE2 NoMetal B97-1 def2tzv

32

O	-0.29802	-1.98121	0.09682
O	-0.34030	1.54036	-0.73138
O	-2.29958	0.09475	1.00369
N	2.02126	-0.04439	0.35847
C	2.08325	-1.44425	-0.10138
C	2.01409	0.96934	-0.71790
C	0.82639	-1.92044	-0.82801
C	0.99013	2.07864	-0.47427
C	-1.59477	-1.75972	-0.52400
C	-1.42182	2.15333	0.02053
C	-2.58289	-1.25038	0.51761
C	-2.59172	1.18497	0.07368
H	2.23319	-2.07693	0.77826
H	2.94384	-1.62243	-0.77546
H	1.77540	0.49785	-1.67453
H	3.01021	1.42883	-0.83218
H	0.58143	-1.24645	-1.65569
H	1.01442	-2.91674	-1.24826
H	1.05688	2.43053	0.56226
H	1.18099	2.92777	-1.14291

H	-1.97938	-2.69822	-0.94585
H	-1.49330	-1.03311	-1.33776
H	-1.74218	3.08749	-0.45949
H	-1.09123	2.38328	1.04079
H	-3.59481	-1.29654	0.09495
H	-2.54899	-1.88716	1.40303
H	-3.48241	1.71450	0.42893
H	-2.79665	0.79975	-0.93010
C	3.01966	0.25251	1.39873
H	2.90281	-0.44061	2.23478
H	2.87137	1.26582	1.77954
H	4.05885	0.17787	1.02745

ACE2 NoMetal CAM-B3LYP def2sv

32

O	-0.30556	-1.71819	0.11819
O	-0.31748	1.35329	-0.57707
O	-2.39556	0.10098	0.96302
N	2.09933	-0.04845	0.33247
C	2.06409	-1.42646	-0.11229
C	2.02830	0.96182	-0.71079
C	0.76188	-1.82827	-0.78202
C	0.93099	1.98142	-0.45604
C	-1.56521	-1.69377	-0.48929
C	-1.38949	2.04847	-0.00692
C	-2.60796	-1.20844	0.49909
C	-2.60635	1.15093	0.04871
H	2.20240	-2.06925	0.77327
H	2.89570	-1.67234	-0.81447
H	1.83629	0.49114	-1.68833
H	2.99346	1.49773	-0.81472

H	0.57236	-1.19271	-1.66723
H	0.86133	-2.87073	-1.15162
H	1.04956	2.40283	0.56255
H	1.01771	2.82397	-1.17436
H	-1.86151	-2.70349	-0.84652
H	-1.54492	-1.02838	-1.37327
H	-1.63312	2.96307	-0.58785
H	-1.13650	2.36809	1.02431
H	-3.60985	-1.31250	0.03617
H	-2.58398	-1.85310	1.39273
H	-3.47577	1.75381	0.37403
H	-2.82880	0.76934	-0.96348
C	3.12933	0.21526	1.30871
H	3.03184	-0.47417	2.16431
H	3.03131	1.24259	1.69839
H	4.16244	0.10883	0.90058

ACE2 NoMetal CAM-B3LYP def2tzv

32

O	-0.30981	-1.89070	0.10677
O	-0.33160	1.46332	-0.67839
O	-2.36057	0.10367	0.99719
N	2.07956	-0.05298	0.34850
C	2.07139	-1.43816	-0.12566
C	2.02974	0.97574	-0.69828
C	0.79304	-1.84638	-0.83030
C	0.97737	2.03659	-0.43468
C	-1.60689	-1.72346	-0.50695
C	-1.42370	2.11225	0.01002
C	-2.60356	-1.24412	0.52249
C	-2.60624	1.17763	0.05015

H	2.21265	-2.08823	0.73776
H	2.90849	-1.63799	-0.81613
H	1.81435	0.52073	-1.66423
H	3.00462	1.47017	-0.79888
H	0.55402	-1.14173	-1.62771
H	0.93295	-2.83277	-1.28136
H	1.04297	2.37520	0.60236
H	1.12917	2.89814	-1.09107
H	-1.95501	-2.67518	-0.91993
H	-1.52956	-1.00440	-1.32421
H	-1.70292	3.03635	-0.50440
H	-1.12465	2.36155	1.03123
H	-3.60993	-1.31822	0.10115
H	-2.55370	-1.87323	1.40763
H	-3.49043	1.72787	0.37542
H	-2.79764	0.78119	-0.94735
C	3.11402	0.20504	1.34867
H	3.01945	-0.49850	2.17395
H	3.00104	1.20986	1.75279
H	4.13204	0.11890	0.93624

ACE2 NoMetal DSDPBEP86 def2tzv

32

O	-0.32201	-1.88668	0.11205
O	-0.32516	1.43125	-0.68972
O	-2.38443	0.10662	1.02192
N	2.06675	-0.05340	0.37603
C	2.06950	-1.44210	-0.11921
C	2.04979	0.96793	-0.69545
C	0.78572	-1.83142	-0.83783
C	0.98770	2.03197	-0.45393

C	-1.61740	-1.69333	-0.52696
C	-1.40794	2.10521	0.01823
C	-2.62999	-1.24057	0.50951
C	-2.61484	1.19010	0.06247
H	2.20832	-2.10474	0.74092
H	2.91202	-1.62414	-0.81614
H	1.85147	0.50068	-1.66497
H	3.03235	1.46140	-0.77553
H	0.54688	-1.10326	-1.61996
H	0.91736	-2.81413	-1.30991
H	1.04782	2.38453	0.58350
H	1.12818	2.88611	-1.12941
H	-1.96167	-2.63445	-0.97770
H	-1.51956	-0.94199	-1.31800
H	-1.67020	3.04109	-0.49290
H	-1.09143	2.33722	1.04276
H	-3.63502	-1.29535	0.07080
H	-2.58995	-1.88951	1.38564
H	-3.48997	1.75904	0.39398
H	-2.81771	0.79071	-0.93663
C	3.15006	0.18716	1.34648
H	3.05332	-0.50624	2.18536
H	3.07460	1.20638	1.73402
H	4.15316	0.06119	0.89804

ACE2 NoMetal DSDPBEP86 def2sv

32

O	-0.30468	-1.71202	0.12652
O	-0.31878	1.32835	-0.58026
O	-2.38734	0.09751	0.97628
N	2.07739	-0.04449	0.34795

C	2.06216	-1.42197	-0.10673
C	2.02679	0.95536	-0.71152
C	0.76168	-1.81811	-0.78252
C	0.92731	1.97364	-0.46699
C	-1.55937	-1.67385	-0.50133
C	-1.38411	2.03995	-0.00590
C	-2.60848	-1.20691	0.48903
C	-2.60525	1.14866	0.05720
H	2.19899	-2.07141	0.77712
H	2.90122	-1.65383	-0.80866
H	1.84133	0.47595	-1.68857
H	2.99743	1.48755	-0.80958
H	0.57156	-1.17132	-1.66134
H	0.85709	-2.85975	-1.16166
H	1.04647	2.40270	0.55023
H	1.00432	2.81160	-1.19487
H	-1.85143	-2.67759	-0.88423
H	-1.52690	-0.98591	-1.36919
H	-1.62204	2.95547	-0.59148
H	-1.12393	2.36004	1.02516
H	-3.61062	-1.29543	0.01891
H	-2.59059	-1.86707	1.37339
H	-3.47359	1.75305	0.38874
H	-2.83436	0.76427	-0.95427
C	3.13211	0.21796	1.30365
H	3.03857	-0.46357	2.16815
H	3.04757	1.25269	1.68146
H	4.15667	0.09490	0.87530

ACE2 NoMetal HSE06 def2sv

32

O	-0.30237	-1.71317	0.12331
O	-0.31807	1.34866	-0.57600
O	-2.40298	0.09858	0.96416
N	2.09973	-0.04727	0.33130
C	2.06590	-1.42308	-0.11020
C	2.02372	0.95962	-0.71104
C	0.76357	-1.82834	-0.77462
C	0.92705	1.97781	-0.45505
C	-1.55735	-1.68895	-0.48898
C	-1.38820	2.04271	-0.00716
C	-2.60691	-1.20697	0.49219
C	-2.60495	1.14669	0.04851
H	2.20910	-2.06413	0.77784
H	2.89627	-1.67415	-0.81508
H	1.82739	0.48612	-1.68852
H	2.98909	1.49675	-0.82372
H	0.57478	-1.19726	-1.66606
H	0.86487	-2.87323	-1.14126
H	1.04723	2.40044	0.56488
H	1.01496	2.82260	-1.17291
H	-1.85321	-2.69851	-0.85171
H	-1.53464	-1.02133	-1.37403
H	-1.63443	2.95887	-0.58743
H	-1.13604	2.36514	1.02534
H	-3.60566	-1.31438	0.01969
H	-2.59140	-1.85541	1.38490
H	-3.47491	1.75370	0.36885
H	-2.82689	0.76443	-0.96545
C	3.12741	0.22157	1.30343
H	3.03482	-0.46909	2.16005

H	3.02297	1.24887	1.69491
H	4.16350	0.12259	0.89642

ACE2 NoMetal HSE06 def2tzv

32

O	-0.30100	-1.87572	0.11872
O	-0.33977	1.44497	-0.67384
O	-2.38313	0.09483	1.00245
N	2.08580	-0.04597	0.35116
C	2.08046	-1.42873	-0.12321
C	2.02326	0.97918	-0.69640
C	0.79956	-1.84106	-0.81908
C	0.96228	2.03041	-0.43184
C	-1.59099	-1.71459	-0.50819
C	-1.43074	2.09794	0.00888
C	-2.60560	-1.24815	0.50888
C	-2.61626	1.16720	0.05225
H	2.23269	-2.07986	0.74108
H	2.91396	-1.62995	-0.82302
H	1.80892	0.51989	-1.66414
H	2.99474	1.48500	-0.80380
H	0.56237	-1.14055	-1.62551
H	0.94083	-2.83222	-1.26679
H	1.02900	2.37101	0.60782
H	1.10927	2.89626	-1.08882
H	-1.93209	-2.66735	-0.93311
H	-1.51130	-0.98859	-1.32378
H	-1.71197	3.02303	-0.50933
H	-1.13440	2.35473	1.03237
H	-3.60557	-1.32937	0.06624
H	-2.56930	-1.88767	1.39069

H	-3.49924	1.72765	0.37318
H	-2.81101	0.77001	-0.94763
C	3.13489	0.21595	1.33123
H	3.05923	-0.49075	2.15901
H	3.02292	1.22145	1.74060
H	4.15032	0.13752	0.90296

ACE2 NoMetal M062X def2sv

32

O	-0.32059	-1.67053	0.11843
O	-0.30720	1.29333	-0.54732
O	-2.41622	0.11011	0.97558
N	2.11808	-0.05863	0.34755
C	2.04698	-1.41977	-0.14536
C	2.05284	0.97187	-0.67993
C	0.72391	-1.74569	-0.81213
C	0.92480	1.95532	-0.42562
C	-1.58286	-1.66112	-0.48474
C	-1.38323	2.02109	-0.02310
C	-2.62654	-1.20015	0.51339
C	-2.61321	1.14245	0.03836
H	2.17458	-2.10080	0.71359
H	2.86426	-1.65347	-0.86762
H	1.90681	0.52010	-1.67601
H	3.00534	1.53445	-0.73618
H	0.53087	-1.03913	-1.64226
H	0.78446	-2.76241	-1.25358
H	1.03295	2.36943	0.59786
H	0.98352	2.80432	-1.13816
H	-1.85978	-2.67113	-0.85436
H	-1.57495	-0.98173	-1.35976

H	-1.59751	2.91998	-0.63753
H	-1.14585	2.36554	1.00389
H	-3.63074	-1.30689	0.05707
H	-2.58839	-1.84854	1.40350
H	-3.47757	1.75998	0.34564
H	-2.83180	0.74036	-0.96734
C	3.20248	0.14807	1.28073
H	3.12147	-0.56489	2.11800
H	3.14840	1.16667	1.70018
H	4.20845	0.02281	0.81709

ACE2 NoMetal M062X def2tzv

32

O	-0.30241	-1.85744	0.11429
O	-0.33960	1.40065	-0.66008
O	-2.38803	0.09600	1.00734
N	2.08424	-0.04408	0.35882
C	2.07921	-1.42923	-0.12145
C	2.03286	0.97836	-0.69815
C	0.79387	-1.81251	-0.82876
C	0.95493	2.01408	-0.43734
C	-1.59020	-1.68323	-0.51608
C	-1.42822	2.07974	0.00537
C	-2.60633	-1.24859	0.51591
C	-2.62572	1.16208	0.05196
H	2.21253	-2.08555	0.73970
H	2.91690	-1.62233	-0.81239
H	1.83702	0.51712	-1.66656
H	2.99931	1.49087	-0.78480
H	0.55976	-1.08399	-1.60764
H	0.91470	-2.79176	-1.30033

H	1.02375	2.35649	0.59939
H	1.07152	2.87575	-1.10067
H	-1.91746	-2.62278	-0.97165
H	-1.50342	-0.92709	-1.30024
H	-1.68312	3.00029	-0.52712
H	-1.13483	2.33507	1.02736
H	-3.60870	-1.33168	0.08658
H	-2.54681	-1.89322	1.39004
H	-3.50316	1.72468	0.37364
H	-2.82048	0.75814	-0.94302
C	3.14963	0.21315	1.33123
H	3.06716	-0.48361	2.16395
H	3.05340	1.22425	1.72528
H	4.15245	0.11118	0.88820

ACE2 NoMetal M06 def2sv

32

O	-0.29806	-1.71811	0.12747
O	-0.32650	1.34871	-0.58471
O	-2.38682	0.08880	0.96293
N	2.08113	-0.04024	0.33616
C	2.06235	-1.41991	-0.09692
C	2.00830	0.95825	-0.71664
C	0.77148	-1.83711	-0.76405
C	0.91815	1.97595	-0.46361
C	-1.54528	-1.67485	-0.49695
C	-1.38933	2.03524	0.00409
C	-2.59552	-1.21195	0.48315
C	-2.60177	1.14178	0.05816
H	2.20596	-2.05651	0.79858
H	2.90620	-1.66447	-0.79244

H	1.81059	0.47727	-1.69397
H	2.98014	1.48842	-0.83581
H	0.58142	-1.21438	-1.66615
H	0.87493	-2.88600	-1.12701
H	1.03801	2.39829	0.56075
H	1.00409	2.82765	-1.17757
H	-1.83736	-2.67463	-0.89588
H	-1.50956	-0.97882	-1.36429
H	-1.63349	2.96742	-0.55622
H	-1.12687	2.33777	1.04377
H	-3.59737	-1.31319	0.00972
H	-2.58202	-1.87320	1.36997
H	-3.47641	1.74368	0.38394
H	-2.83007	0.76519	-0.96052
C	3.12060	0.23695	1.29279
H	3.04014	-0.44289	2.16180
H	3.02981	1.27189	1.67418
H	4.15109	0.12675	0.86848

ACE2 NoMetal M06 def2tzv

32

O	-0.29426	-1.83996	0.12311
O	-0.34466	1.40277	-0.65884
O	-2.38208	0.08865	0.99888
N	2.07934	-0.03694	0.34655
C	2.08084	-1.42642	-0.10310
C	2.01334	0.97352	-0.71324
C	0.80703	-1.84377	-0.80328
C	0.94520	2.01199	-0.44871
C	-1.57136	-1.68637	-0.51587
C	-1.42451	2.07361	0.01141

C	-2.59455	-1.24731	0.50077
C	-2.61762	1.15775	0.05540
H	2.21839	-2.06181	0.77601
H	2.92484	-1.63737	-0.78630
H	1.79900	0.50272	-1.67575
H	2.98243	1.48217	-0.82623
H	0.58505	-1.16234	-1.63226
H	0.94026	-2.84662	-1.22760
H	1.02390	2.36298	0.58838
H	1.06579	2.87633	-1.11329
H	-1.89563	-2.63315	-0.96663
H	-1.49085	-0.93966	-1.31483
H	-1.68643	3.00417	-0.50737
H	-1.12911	2.32541	1.03770
H	-3.59350	-1.33484	0.05684
H	-2.54992	-1.89697	1.37505
H	-3.49696	1.72312	0.37676
H	-2.81709	0.76305	-0.94542
C	3.12007	0.24517	1.32772
H	3.03978	-0.44616	2.16795
H	3.00592	1.25914	1.71594
H	4.13612	0.15771	0.90341

ACE2 NoMetal MP2 def2tzv

32

O	-0.34512	-1.91378	0.09838
O	-0.30662	1.44061	-0.70570
O	-2.36570	0.12573	1.03489
N	2.05452	-0.06649	0.38853
C	2.05111	-1.45501	-0.13012
C	2.07398	0.96181	-0.68643

C	0.76491	-1.82139	-0.86318
C	1.01809	2.03945	-0.45956
C	-1.65343	-1.69443	-0.53183
C	-1.38835	2.12541	0.01887
C	-2.64545	-1.22773	0.52325
C	-2.60147	1.21403	0.06581
H	2.17963	-2.13062	0.72218
H	2.89575	-1.62635	-0.82840
H	1.88696	0.49866	-1.66141
H	3.06551	1.44299	-0.74281
H	0.51925	-1.06422	-1.61518
H	0.89441	-2.78812	-1.36841
H	1.06829	2.39116	0.57888
H	1.16773	2.88895	-1.13896
H	-2.01644	-2.62914	-0.98143
H	-1.54868	-0.94024	-1.31932
H	-1.64438	3.06413	-0.49006
H	-1.05999	2.34602	1.04215
H	-3.65772	-1.25928	0.09848
H	-2.60128	-1.87258	1.40214
H	-3.47567	1.78364	0.39937
H	-2.80409	0.80978	-0.93199
C	3.14981	0.14466	1.36704
H	3.02704	-0.54649	2.20429
H	3.09853	1.16712	1.75008
H	4.14785	-0.01119	0.91706

ACE2 NoMetal MP2 def2sv

32

O	-0.31012	-1.71129	0.12702
O	-0.31412	1.32476	-0.58047

O	-2.37177	0.10305	0.97828
N	2.06721	-0.04819	0.35354
C	2.05236	-1.42047	-0.11624
C	2.02940	0.95498	-0.70324
C	0.75100	-1.80599	-0.79201
C	0.93197	1.97293	-0.46030
C	-1.56762	-1.67382	-0.49941
C	-1.37961	2.04237	-0.00845
C	-2.60841	-1.20173	0.49441
C	-2.59782	1.15030	0.05450
H	2.18833	-2.08043	0.75930
H	2.89048	-1.64317	-0.82173
H	1.84914	0.48239	-1.68413
H	3.00257	1.48392	-0.79179
H	0.55712	-1.14664	-1.65944
H	0.84337	-2.84224	-1.18576
H	1.04875	2.39628	0.55879
H	1.01099	2.81338	-1.18473
H	-1.86246	-2.67777	-0.87861
H	-1.53752	-0.98811	-1.36789
H	-1.61510	2.95642	-0.59659
H	-1.12177	2.36273	1.02230
H	-3.61389	-1.28320	0.03133
H	-2.58833	-1.86060	1.37893
H	-3.46972	1.75100	0.38287
H	-2.82241	0.76289	-0.95598
C	3.13533	0.19887	1.30008
H	3.04088	-0.48384	2.16236
H	3.06510	1.23263	1.68049
H	4.15253	0.06485	0.86067

ACE2 NoMetal PBE0 def2sv

32

O	-0.30246	-1.71341	0.12339
O	-0.31757	1.34891	-0.57583
O	-2.40188	0.09895	0.96360
N	2.09925	-0.04747	0.33090
C	2.06543	-1.42298	-0.11115
C	2.02400	0.96009	-0.71066
C	0.76273	-1.82883	-0.77461
C	0.92699	1.97805	-0.45512
C	-1.55717	-1.69009	-0.48839
C	-1.38736	2.04305	-0.00773
C	-2.60659	-1.20599	0.49205
C	-2.60414	1.14699	0.04880
H	2.20935	-2.06416	0.77664
H	2.89525	-1.67369	-0.81666
H	1.82904	0.48733	-1.68868
H	2.98931	1.49755	-0.82184
H	0.57349	-1.19869	-1.66649
H	0.86440	-2.87408	-1.14024
H	1.04733	2.40130	0.56449
H	1.01503	2.82235	-1.17358
H	-1.85363	-2.70041	-0.84857
H	-1.53470	-1.02464	-1.37494
H	-1.63412	2.95853	-0.58891
H	-1.13544	2.36662	1.02444
H	-3.60523	-1.31288	0.01918
H	-2.59220	-1.85404	1.38512
H	-3.47355	1.75424	0.37036
H	-2.82741	0.76547	-0.96510

C	3.12586	0.22076	1.30418
H	3.03192	-0.47025	2.16042
H	3.02069	1.24786	1.69607
H	4.16242	0.12193	0.89852

ACE2 NoMetal PBE0 def2tzv

32

O	-0.30210	-1.87274	0.11969
O	-0.33870	1.44523	-0.67332
O	-2.38457	0.09545	1.00170
N	2.08651	-0.04690	0.35137
C	2.07926	-1.42879	-0.12545
C	2.02409	0.97980	-0.69466
C	0.79634	-1.83968	-0.81900
C	0.96202	2.03020	-0.42965
C	-1.59141	-1.71531	-0.50707
C	-1.42917	2.09744	0.00873
C	-2.60693	-1.24648	0.50864
C	-2.61558	1.16712	0.05196
H	2.23331	-2.08151	0.73769
H	2.91091	-1.62936	-0.82795
H	1.81138	0.52196	-1.66377
H	2.99534	1.48692	-0.79988
H	0.55907	-1.13898	-1.62562
H	0.93641	-2.83134	-1.26692
H	1.02831	2.36975	0.61070
H	1.10990	2.89711	-1.08570
H	-1.93229	-2.67004	-0.92869
H	-1.51302	-0.99206	-1.32556
H	-1.71093	3.02289	-0.50936
H	-1.13348	2.35419	1.03278

H	-3.60657	-1.32803	0.06445
H	-2.57206	-1.88601	1.39094
H	-3.49793	1.72945	0.37262
H	-2.81090	0.77067	-0.94842
C	3.13867	0.21173	1.32890
H	3.06445	-0.49695	2.15555
H	3.02860	1.21668	1.74096
H	4.15308	0.13326	0.89765

ACE2 NoMetal PBE def2sv

32

O	-0.30149	-1.71899	0.13169
O	-0.32377	1.35586	-0.58642
O	-2.44001	0.09666	0.97471
N	2.11973	-0.04441	0.33402
C	2.08534	-1.43264	-0.10355
C	2.03636	0.96629	-0.71915
C	0.77625	-1.84828	-0.77161
C	0.93285	1.99206	-0.45973
C	-1.56418	-1.70065	-0.49562
C	-1.40024	2.05357	-0.00080
C	-2.62955	-1.21997	0.48560
C	-2.62969	1.15704	0.04757
H	2.23041	-2.07115	0.79695
H	2.92246	-1.69520	-0.81320
H	1.83295	0.48493	-1.70129
H	3.00759	1.51039	-0.84201
H	0.58835	-1.22504	-1.68100
H	0.88169	-2.90687	-1.12884
H	1.05254	2.41594	0.57037
H	1.02476	2.84703	-1.18105

H	-1.85949	-2.72033	-0.86302
H	-1.53859	-1.02926	-1.39023
H	-1.64884	2.98701	-0.57266
H	-1.14259	2.36638	1.04412
H	-3.63148	-1.33782	-0.00114
H	-2.61629	-1.87362	1.38593
H	-3.50257	1.77707	0.36912
H	-2.85337	0.77562	-0.97631
C	3.16008	0.23204	1.30516
H	3.07695	-0.46547	2.16847
H	3.05122	1.26662	1.70082
H	4.20500	0.13962	0.88985

ACE2 NoMetal PBE def2tzv

32

O	-0.30569	-1.87996	0.13573
O	-0.34471	1.44428	-0.68116
O	-2.42924	0.09414	1.01976
N	2.11351	-0.04416	0.35792
C	2.09911	-1.43905	-0.12276
C	2.04123	0.99031	-0.69954
C	0.80763	-1.85283	-0.81929
C	0.97128	2.04541	-0.42888
C	-1.60615	-1.72327	-0.51643
C	-1.44593	2.11023	0.01542
C	-2.64035	-1.26203	0.49694
C	-2.64566	1.18226	0.04994
H	2.25609	-2.09352	0.74865
H	2.93505	-1.64810	-0.83320
H	1.82282	0.52582	-1.67346
H	3.01757	1.50454	-0.81334

H	0.56649	-1.15095	-1.63500
H	0.94718	-2.85525	-1.26462
H	1.03291	2.38349	0.62083
H	1.11646	2.92105	-1.08859
H	-1.94117	-2.68599	-0.94841
H	-1.51834	-0.99150	-1.33768
H	-1.72315	3.04827	-0.50166
H	-1.14458	2.36085	1.04838
H	-3.64162	-1.34368	0.03556
H	-2.61476	-1.90905	1.38417
H	-3.53190	1.75532	0.37005
H	-2.84106	0.78056	-0.95692
C	3.19619	0.21300	1.32187
H	3.13684	-0.50390	2.15268
H	3.09304	1.22381	1.74142
H	4.21075	0.13548	0.86743

ACE2 NoMetal SCS-MP2 def2sv

32

O	-0.31612	-1.72336	0.12395
O	-0.31130	1.34082	-0.58164
O	-2.36948	0.10867	0.97742
N	2.07186	-0.05217	0.35443
C	2.05199	-1.42631	-0.12510
C	2.03707	0.96120	-0.70001
C	0.74519	-1.81018	-0.80110
C	0.93987	1.98450	-0.45223
C	-1.58106	-1.68887	-0.49543
C	-1.38240	2.05654	-0.00966
C	-2.61610	-1.20121	0.50352
C	-2.59965	1.15603	0.04959

H	2.19028	-2.09163	0.74713
H	2.88805	-1.64543	-0.83475
H	1.85760	0.49441	-1.68469
H	3.01213	1.48863	-0.78305
H	0.54708	-1.14679	-1.66480
H	0.83816	-2.84561	-1.19882
H	1.05446	2.40203	0.56999
H	1.02411	2.82838	-1.17315
H	-1.88070	-2.69785	-0.85907
H	-1.55580	-1.01436	-1.37321
H	-1.61990	2.96933	-0.60016
H	-1.12760	2.37808	1.02200
H	-3.62577	-1.28012	0.04749
H	-2.59416	-1.85293	1.39450
H	-3.47673	1.75170	0.37634
H	-2.81880	0.76603	-0.96173
C	3.14831	0.18383	1.30186
H	3.05117	-0.50304	2.16205
H	3.08562	1.21728	1.68789
H	4.16378	0.04483	0.85850

ACE2 NoMetal SCS-MP2 def2tzv

32

O	-0.36684	-1.98998	0.07588
O	-0.27757	1.53686	-0.73891
O	-2.26937	0.15461	1.02130
N	2.00440	-0.09265	0.38158
C	2.02108	-1.47997	-0.14962
C	2.07573	0.94768	-0.68317
C	0.73678	-1.85735	-0.89055
C	1.06419	2.07122	-0.44501

C	-1.68966	-1.74144	-0.51632
C	-1.36008	2.18564	0.01542
C	-2.62848	-1.19749	0.55881
C	-2.54955	1.23620	0.05576
H	2.15105	-2.16119	0.69882
H	2.87218	-1.63741	-0.84295
H	1.86357	0.50196	-1.66125
H	3.08992	1.38190	-0.73627
H	0.47592	-1.08997	-1.62731
H	0.88102	-2.81312	-1.41322
H	1.10745	2.39536	0.60279
H	1.26736	2.93004	-1.09913
H	-2.10214	-2.67828	-0.91666
H	-1.58987	-1.02133	-1.33627
H	-1.64503	3.12753	-0.47264
H	-1.02473	2.39452	1.03913
H	-3.65564	-1.19548	0.16823
H	-2.58354	-1.82092	1.45399
H	-3.44565	1.77424	0.38718
H	-2.72969	0.82876	-0.94550
C	3.05955	0.10724	1.40925
H	2.89052	-0.58014	2.24273
H	3.00401	1.13194	1.78832
H	4.07540	-0.06219	1.00543

ACE2 Rb B2PLYP def2sv

33

O	-0.31006	-1.27385	-1.22307
O	-0.30084	1.62749	-0.30493
O	-2.45160	-0.01959	0.28810
N	2.15700	0.11054	-0.27126

C	2.08705	-0.90060	-1.32489
C	2.02888	1.49614	-0.74117
C	0.76915	-0.95080	-2.08135
C	0.92747	2.26347	-0.02426
C	-1.57211	-0.99608	-1.79801
C	-1.43341	2.16421	0.33686
C	-2.65474	-0.98668	-0.73214
C	-2.63890	1.34197	-0.07902
H	2.26119	-1.88519	-0.85786
H	2.89221	-0.76482	-2.08292
H	1.81005	1.51586	-1.82053
H	2.98157	2.04345	-0.61134
H	0.56752	0.01833	-2.57081
H	0.86091	-1.70884	-2.88430
H	1.11519	2.28112	1.06923
H	0.91410	3.31492	-0.37566
H	-1.83579	-1.75799	-2.55913
H	-1.53710	-0.01677	-2.30855
H	-1.59802	3.22279	0.05238
H	-1.30942	2.13406	1.43954
H	-3.63371	-0.83759	-1.22502
H	-2.68559	-1.96086	-0.21878
H	-3.54136	1.73722	0.42094
H	-2.78526	1.43666	-1.16761
C	3.33665	-0.07158	0.56469
H	3.33955	-1.08322	1.00414
H	3.32673	0.65690	1.39188
H	4.28757	0.06008	0.00193
Rb	-0.03053	-0.86521	1.63234

ACE2 Rb B2PLYP def2tzv

33

O	-0.34341	-1.27693	-1.29134
O	-0.30346	1.71422	-0.29685
O	-2.47395	-0.02417	0.36313
N	2.17993	0.11052	-0.25142
C	2.07305	-0.82131	-1.40121
C	2.08165	1.54784	-0.62704
C	0.74310	-0.77307	-2.13866
C	0.97767	2.28253	0.12308
C	-1.66061	-0.92535	-1.82443
C	-1.45777	2.19407	0.45597
C	-2.70346	-0.98678	-0.72169
C	-2.66545	1.39125	0.01359
H	2.23567	-1.83300	-1.02531
H	2.86216	-0.62245	-2.14626
H	1.89956	1.65041	-1.69826
H	3.03024	2.05612	-0.42110
H	0.50057	0.24682	-2.43955
H	0.81598	-1.39210	-3.03759
H	1.09999	2.16012	1.20459
H	1.00549	3.35113	-0.11208
H	-1.94209	-1.62567	-2.61677
H	-1.61235	0.07820	-2.25111
H	-1.63149	3.25638	0.25917
H	-1.28529	2.06476	1.52922
H	-3.69101	-0.82328	-1.16103
H	-2.69706	-1.96090	-0.23721
H	-3.55597	1.75429	0.52820
H	-2.81313	1.50141	-1.06095
C	3.39905	-0.16773	0.54381

H	3.38471	-1.20072	0.89089
H	3.42452	0.48645	1.41421
H	4.32024	-0.00939	-0.03678
Rb	-0.00992	-0.96394	1.55237

ACE2 Rb B3LYP def2sv

33

O	-0.31189	-0.89781	-1.53216
O	-0.30126	1.67841	0.15826
O	-2.46086	-0.07684	0.26483
N	2.17762	0.19261	-0.23798
C	2.09572	-0.50349	-1.52442
C	2.03394	1.65530	-0.30854
C	0.77263	-0.35916	-2.26887
C	0.93335	2.20261	0.59734
C	-1.57848	-0.48609	-2.01114
C	-1.44221	2.02270	0.90776
C	-2.66394	-0.74449	-0.97337
C	-2.64546	1.33484	0.27873
H	2.27797	-1.57682	-1.33611
H	2.89557	-0.17048	-2.22761
H	1.80721	1.96661	-1.34196
H	2.98537	2.15523	-0.04120
H	0.56916	0.70490	-2.49206
H	0.87112	-0.88092	-3.24333
H	1.12281	1.92084	1.65558
H	0.93003	3.31194	0.54645
H	-1.84732	-1.03288	-2.93961
H	-1.54655	0.59130	-2.26251
H	-1.61332	3.11983	0.90967
H	-1.32793	1.70693	1.96769

H	-3.64192	-0.46840	-1.41441
H	-2.70478	-1.81989	-0.72952
H	-3.55131	1.58191	0.86434
H	-2.79011	1.72011	-0.74582
C	3.35802	-0.20802	0.51951
H	3.37108	-1.30359	0.66056
H	3.34352	0.25991	1.51924
H	4.31208	0.08123	0.02004
Rb	-0.03501	-1.30625	1.34776

ACE2 Rb B3LYP def2tzv

33

O	-0.32621	-1.03483	-1.48988
O	-0.31394	1.79312	-0.00372
O	-2.47013	-0.06506	0.33503
N	2.18469	0.18103	-0.22492
C	2.09566	-0.57603	-1.49678
C	2.06487	1.65639	-0.37282
C	0.77681	-0.43922	-2.24464
C	0.97132	2.26892	0.49471
C	-1.63484	-0.59491	-1.96494
C	-1.46945	2.11642	0.81773
C	-2.68992	-0.82647	-0.89707
C	-2.66737	1.38685	0.24140
H	2.26470	-1.62959	-1.26710
H	2.89179	-0.27363	-2.19856
H	1.85995	1.91645	-1.41212
H	3.01403	2.13957	-0.11518
H	0.54352	0.60986	-2.43339
H	0.87018	-0.94571	-3.21019
H	1.09938	1.97821	1.54279

H	1.01456	3.36163	0.43406
H	-1.91570	-1.15542	-2.86265
H	-1.58148	0.46386	-2.22607
H	-1.66366	3.19425	0.80814
H	-1.29730	1.81188	1.85558
H	-3.67082	-0.58640	-1.31660
H	-2.69978	-1.86939	-0.58506
H	-3.56201	1.64895	0.80877
H	-2.81660	1.68360	-0.79715
C	3.38618	-0.20138	0.54739
H	3.39307	-1.27785	0.71941
H	3.37657	0.29647	1.51647
H	4.32116	0.06837	0.03209
Rb	-0.02302	-1.27004	1.37297

ACE2 Rb B97-1 def2sv

33

O	-0.31588	-0.68198	-1.63771
O	-0.30641	1.63166	0.36746
O	-2.46868	-0.12660	0.25290
N	2.17773	0.21807	-0.22039
C	2.09064	-0.29073	-1.59225
C	2.03381	1.67707	-0.08674
C	0.75927	-0.04294	-2.29922
C	0.92407	2.08884	0.88199
C	-1.58296	-0.21935	-2.05769
C	-1.44401	1.87037	1.15929
C	-2.66807	-0.62266	-1.06145
C	-2.65225	1.26924	0.44857
H	2.27345	-1.38170	-1.55752
H	2.88678	0.13996	-2.24725

H	1.81508	2.13210	-1.06944
H	2.98354	2.13451	0.25756
H	0.55413	1.04428	-2.36621
H	0.85069	-0.42094	-3.34045
H	1.11067	1.65694	1.89123
H	0.91928	3.19560	0.99114
H	-1.85027	-0.63958	-3.05220
H	-1.55946	0.88435	-2.16274
H	-1.61654	2.95844	1.31028
H	-1.32626	1.41322	2.16806
H	-3.64917	-0.29532	-1.46324
H	-2.70151	-1.72316	-0.96464
H	-3.55655	1.43949	1.06596
H	-2.80062	1.78585	-0.51806
C	3.36466	-0.28163	0.46677
H	3.37946	-1.38774	0.45184
H	3.35333	0.04202	1.52386
H	4.31615	0.07842	0.00696
Rb	-0.02296	-1.45560	1.17840

ACE2 Rb B97-1 def2tzv

33

O	-0.33335	-1.01156	-1.50654
O	-0.31559	1.79222	0.01599
O	-2.47260	-0.07643	0.34192
N	2.17969	0.18081	-0.22655
C	2.08767	-0.54759	-1.51607
C	2.06597	1.66015	-0.34837
C	0.76004	-0.39401	-2.25283
C	0.96565	2.25723	0.52817
C	-1.64216	-0.56851	-1.97053

C	-1.46574	2.09809	0.84803
C	-2.69543	-0.81800	-0.89849
C	-2.66903	1.37374	0.26475
H	2.25935	-1.60855	-1.31057
H	2.88053	-0.22569	-2.21664
H	1.86679	1.94127	-1.38643
H	3.01626	2.13706	-0.07377
H	0.52424	0.66280	-2.41092
H	0.84958	-0.87457	-3.23491
H	1.09243	1.94349	1.57303
H	1.01091	3.35349	0.49053
H	-1.92789	-1.11814	-2.87653
H	-1.59421	0.49700	-2.21769
H	-1.66419	3.17770	0.85924
H	-1.28824	1.77380	1.88205
H	-3.68004	-0.57361	-1.31368
H	-2.70070	-1.86848	-0.60288
H	-3.56300	1.63256	0.83950
H	-2.82052	1.68522	-0.77214
C	3.38915	-0.21682	0.52758
H	3.39609	-1.29906	0.67810
H	3.38661	0.26328	1.50844
H	4.32140	0.06364	0.00833
Rb	-0.01156	-1.28455	1.35833

ACE2 Rb CAM-B3LYP def2sv

33

O	-0.30821	-1.54888	-0.83793
O	-0.30456	1.48168	-0.69523
O	-2.45014	0.05461	0.28737
N	2.16247	0.03524	-0.29892

C	2.08507	-1.21541	-1.04374
C	2.01948	1.24816	-1.10524
C	0.76406	-1.46623	-1.74966
C	0.91736	2.16985	-0.60878
C	-1.57060	-1.44383	-1.45225
C	-1.44222	2.17164	-0.25388
C	-2.64890	-1.15039	-0.42579
C	-2.64138	1.26106	-0.42855
H	2.26662	-2.04139	-0.33499
H	2.88410	-1.28547	-1.81606
H	1.79706	0.98581	-2.15142
H	2.96807	1.81624	-1.12819
H	0.55893	-0.66279	-2.47956
H	0.85351	-2.41007	-2.32276
H	1.11309	2.48115	0.43844
H	0.89391	3.08906	-1.22765
H	-1.83373	-2.38371	-1.97784
H	-1.54465	-0.63913	-2.20941
H	-1.60414	3.10101	-0.83555
H	-1.33354	2.46593	0.81092
H	-3.62930	-1.14581	-0.93777
H	-2.67621	-1.95044	0.33158
H	-3.54558	1.77434	-0.05504
H	-2.78875	1.05696	-1.50218
C	3.33104	0.08050	0.56210
H	3.33356	-0.78099	1.25119
H	3.31762	0.99872	1.17230
H	4.28631	0.06277	-0.00840
Rb	-0.02255	-0.39487	1.78583

ACE2 Rb CAM-B3LYP def2tzv

33

O	-0.32037	-1.31296	-1.24076
O	-0.31454	1.71147	-0.35018
O	-2.45599	-0.01899	0.34054
N	2.17042	0.11847	-0.25293
C	2.08773	-0.87963	-1.33296
C	2.05026	1.52197	-0.69665
C	0.77707	-0.89087	-2.09283
C	0.95956	2.28656	0.02854
C	-1.61718	-0.97430	-1.79229
C	-1.45885	2.19401	0.38685
C	-2.67002	-1.00316	-0.70789
C	-2.65633	1.37485	-0.02916
H	2.24847	-1.86341	-0.89130
H	2.88919	-0.73062	-2.07356
H	1.84269	1.56444	-1.76526
H	2.99787	2.04812	-0.54571
H	0.54893	0.09998	-2.48524
H	0.86607	-1.57861	-2.93716
H	1.09385	2.21433	1.11206
H	0.98977	3.34336	-0.25027
H	-1.89236	-1.69061	-2.57075
H	-1.56181	0.01707	-2.24378
H	-1.64404	3.24907	0.16869
H	-1.28401	2.09505	1.46227
H	-3.65134	-0.85720	-1.16390
H	-2.67044	-1.96533	-0.20176
H	-3.54711	1.74354	0.47860
H	-2.80902	1.46447	-1.10394
C	3.35246	-0.09668	0.59316

H	3.34688	-1.10923	0.99390
H	3.34119	0.59818	1.43077
H	4.29186	0.04729	0.04103
Rb	-0.02191	-0.91221	1.57239

ACE2 Rb DSDPBEP86 def2tzv

33

O	-0.33959	-1.64880	-0.75580
O	-0.30765	1.44448	-0.81542
O	-2.48429	0.07937	0.37962
N	2.16850	0.00793	-0.26199
C	2.07093	-1.26367	-1.01548
C	2.07203	1.21894	-1.11792
C	0.74127	-1.46583	-1.72646
C	0.96014	2.15600	-0.66468
C	-1.65055	-1.48442	-1.37956
C	-1.45980	2.18067	-0.30829
C	-2.69632	-1.19321	-0.31714
C	-2.67469	1.28168	-0.44017
H	2.22746	-2.08436	-0.30758
H	2.86680	-1.33455	-1.78090
H	1.89205	0.94017	-2.16126
H	3.02061	1.77387	-1.09900
H	0.49943	-0.60313	-2.35458
H	0.80963	-2.35472	-2.36602
H	1.10248	2.43922	0.38763
H	0.95188	3.06795	-1.27535
H	-1.93014	-2.40167	-1.91348
H	-1.59672	-0.66301	-2.10151
H	-1.61536	3.10009	-0.88665
H	-1.29760	2.45442	0.74299

H	-3.68799	-1.20046	-0.78478
H	-2.67272	-1.95176	0.46706
H	-3.56382	1.80785	-0.08103
H	-2.82706	1.00995	-1.48848
C	3.38207	0.02955	0.58489
H	3.36372	-0.81763	1.27559
H	3.40165	0.95213	1.17001
H	4.30816	-0.02626	-0.01239
Rb	0.00405	-0.32222	1.73636

ACE2 Rb DSDPBEP86 def2sv

33

O	-0.32585	-1.75379	-0.08207
O	-0.30252	1.01070	-1.25521
O	-2.44442	0.18139	0.27786
N	2.14028	-0.11791	-0.26846
C	2.05842	-1.56418	-0.44056
C	2.02520	0.64767	-1.51310
C	0.72645	-2.05715	-0.97615
C	0.91921	1.68739	-1.45103
C	-1.59710	-1.91896	-0.67346
C	-1.43226	1.84089	-1.13010
C	-2.65835	-1.21461	0.15089
C	-2.63758	0.95274	-0.89843
H	2.23628	-2.03310	0.54480
H	2.85381	-1.94645	-1.12340
H	1.81763	-0.02620	-2.36233
H	2.98022	1.15742	-1.75094
H	0.51178	-1.59947	-1.96027
H	0.79560	-3.15412	-1.13083
H	1.10573	2.40061	-0.61848

H	0.89705	2.27431	-2.39322
H	-1.86216	-2.99473	-0.74923
H	-1.58255	-1.50755	-1.70092
H	-1.59180	2.44919	-2.04478
H	-1.30845	2.54688	-0.28011
H	-3.65178	-1.42175	-0.29443
H	-2.66311	-1.61332	1.17989
H	-3.54248	1.57987	-0.78633
H	-2.78322	0.30024	-1.77736
C	3.31900	0.26260	0.49555
H	3.31134	-0.23826	1.48054
H	3.31655	1.35325	0.66929
H	4.27070	-0.00380	-0.01894
Rb	0.00409	0.43183	1.73216

ACE2 Rb HSE06 def2sv

33

O	-0.29632	-1.25425	-1.23817
O	-0.31106	1.62903	-0.28203
O	-2.44783	-0.03333	0.27889
N	2.15415	0.12523	-0.27209
C	2.09352	-0.88302	-1.31863
C	2.00865	1.50435	-0.73084
C	0.78431	-0.93976	-2.08221
C	0.90988	2.26153	-0.00621
C	-1.54741	-0.96540	-1.80908
C	-1.43834	2.14781	0.36352
C	-2.63635	-0.97534	-0.75476
C	-2.63898	1.32637	-0.05645
H	2.26846	-1.86740	-0.84782
H	2.90411	-0.75167	-2.07347

H	1.77791	1.52405	-1.80932
H	2.95879	2.06113	-0.61330
H	0.58832	0.02788	-2.58278
H	0.88514	-1.70099	-2.88277
H	1.10364	2.27674	1.08816
H	0.89475	3.31657	-0.35080
H	-1.80997	-1.70949	-2.58987
H	-1.50961	0.02633	-2.30067
H	-1.61271	3.21000	0.09334
H	-1.31253	2.10903	1.46775
H	-3.61189	-0.82193	-1.25613
H	-2.67361	-1.96142	-0.26137
H	-3.54025	1.71127	0.45613
H	-2.79583	1.44237	-1.14357
C	3.31255	-0.04865	0.58014
H	3.32348	-1.06501	1.01215
H	3.28045	0.67087	1.41640
H	4.27532	0.09982	0.03891
Rb	-0.03022	-0.88577	1.61787

ACE2 Rb HSE06 def2tzv

33

O	-0.31427	-1.21340	-1.32953
O	-0.33036	1.73518	-0.24577
O	-2.46431	-0.06152	0.34313
N	2.16577	0.14096	-0.24587
C	2.08920	-0.76524	-1.40170
C	2.03865	1.57244	-0.58130
C	0.77544	-0.72644	-2.15306
C	0.93946	2.27344	0.19207
C	-1.60902	-0.83751	-1.85675

C	-1.47044	2.14882	0.53601
C	-2.66694	-0.95427	-0.78433
C	-2.66698	1.35404	0.07420
H	2.26403	-1.78235	-1.04152
H	2.88557	-0.54943	-2.13613
H	1.83920	1.69646	-1.64837
H	2.98312	2.09423	-0.38350
H	0.54416	0.29364	-2.47218
H	0.86547	-1.34851	-3.05044
H	1.06581	2.10958	1.26964
H	0.97674	3.35309	0.00531
H	-1.88301	-1.49122	-2.69305
H	-1.55821	0.18943	-2.23083
H	-1.67055	3.21829	0.40244
H	-1.28526	1.97189	1.60280
H	-3.64638	-0.77311	-1.23896
H	-2.67326	-1.95779	-0.35895
H	-3.55537	1.68741	0.61557
H	-2.83263	1.52087	-0.99246
C	3.35571	-0.12822	0.56945
H	3.36570	-1.17260	0.88653
H	3.34349	0.49629	1.46360
H	4.29295	0.07187	0.02537
Rb	-0.00867	-1.02485	1.51313

ACE2 Rb M062X def2sv

33

O	-0.31727	-1.73589	0.08297
O	-0.30896	0.85515	-1.35415
O	-2.44024	0.19409	0.26070
N	2.14410	-0.13734	-0.25353

C	2.06711	-1.59250	-0.25761
C	2.02527	0.48381	-1.57366
C	0.74158	-2.13957	-0.75358
C	0.90642	1.50894	-1.62813
C	-1.57815	-1.96348	-0.49689
C	-1.42898	1.69986	-1.30279
C	-2.64729	-1.20375	0.26381
C	-2.64238	0.85504	-0.97349
H	2.22685	-1.94355	0.77702
H	2.87218	-2.04681	-0.87771
H	1.83288	-0.28121	-2.34358
H	2.97211	0.97810	-1.85999
H	0.54254	-1.79056	-1.78388
H	0.80478	-3.24475	-0.78538
H	1.08709	2.31098	-0.88088
H	0.87530	1.98624	-2.62730
H	-1.82797	-3.04286	-0.48775
H	-1.56097	-1.63246	-1.55276
H	-1.58811	2.21681	-2.26923
H	-1.28836	2.48216	-0.52684
H	-3.63796	-1.45361	-0.15909
H	-2.64580	-1.51105	1.32212
H	-3.53442	1.50186	-0.90697
H	-2.80977	0.12562	-1.78447
C	3.31016	0.33818	0.47194
H	3.29698	-0.04587	1.50611
H	3.30015	1.43982	0.51743
H	4.26503	0.02009	-0.00084
Rb	-0.00492	0.60720	1.67797

ACE2 Rb M062X def2tzv

33

O	-0.32426	-1.69189	-0.57155
O	-0.31346	1.32753	-0.95348
O	-2.47202	0.10131	0.35841
N	2.16275	-0.01891	-0.26844
C	2.08009	-1.37335	-0.84175
C	2.05095	1.07222	-1.25712
C	0.76198	-1.66795	-1.53142
C	0.94345	2.04777	-0.90516
C	-1.61924	-1.61003	-1.21195
C	-1.45616	2.10709	-0.53145
C	-2.66978	-1.22771	-0.19262
C	-2.66842	1.20569	-0.56562
H	2.22376	-2.09040	-0.03157
H	2.88407	-1.54503	-1.57421
H	1.85480	0.66840	-2.25067
H	2.99386	1.62430	-1.32420
H	0.54249	-0.90870	-2.28411
H	0.82708	-2.63807	-2.03065
H	1.09861	2.45280	0.10089
H	0.92234	2.88055	-1.61305
H	-1.88064	-2.57520	-1.65386
H	-1.57090	-0.86481	-2.00905
H	-1.60773	2.95985	-1.19796
H	-1.29571	2.48722	0.48280
H	-3.65798	-1.29864	-0.65218
H	-2.63479	-1.90305	0.65973
H	-3.55319	1.76349	-0.26045
H	-2.82358	0.83337	-1.57830
C	3.33874	0.13689	0.60123

H	3.31568	-0.61101	1.39339
H	3.32406	1.12361	1.06245
H	4.28114	0.02667	0.04729
Rb	-0.00201	-0.11601	1.74435

ACE2 Rb M06 def2sv

33

O	-0.32735	-1.42125	-0.96764
O	-0.31741	1.53176	-0.56880
O	-2.45809	0.02666	0.31932
N	2.13929	0.06444	-0.33073
C	2.05279	-1.11250	-1.17827
C	1.99008	1.34409	-1.01700
C	0.72820	-1.28939	-1.88583
C	0.90239	2.20275	-0.40934
C	-1.59470	-1.29835	-1.55553
C	-1.43999	2.16493	-0.02875
C	-2.65378	-1.10789	-0.49430
C	-2.64099	1.28872	-0.28621
H	2.22906	-2.00245	-0.53979
H	2.85507	-1.12614	-1.95733
H	1.74538	1.18124	-2.08410
H	2.94508	1.91056	-1.00955
H	0.52514	-0.42918	-2.55922
H	0.79532	-2.18970	-2.53544
H	1.11141	2.38304	0.67320
H	0.87423	3.19929	-0.90367
H	-1.84787	-2.20215	-2.15330
H	-1.59772	-0.43963	-2.26145
H	-1.60272	3.16639	-0.48477
H	-1.31113	2.32792	1.06790

H	-3.64865	-1.07802	-0.98698
H	-2.65617	-1.97550	0.19257
H	-3.54768	1.77622	0.12513
H	-2.78846	1.18442	-1.37956
C	3.29715	0.03595	0.53519
H	3.29962	-0.88117	1.15557
H	3.27870	0.90418	1.22106
H	4.26171	0.06487	-0.02690
Rb	0.02920	-0.55926	1.74105

ACE2 Rb M06 def2tzv

33

O	-0.33048	-1.67475	-0.70647
O	-0.30613	1.46681	-0.79762
O	-2.46123	0.09671	0.34267
N	2.14369	0.01642	-0.31190
C	2.06969	-1.29943	-0.95730
C	2.01005	1.17867	-1.20326
C	0.75767	-1.58799	-1.65426
C	0.95644	2.15134	-0.71374
C	-1.62075	-1.52772	-1.33344
C	-1.45494	2.18816	-0.32881
C	-2.66820	-1.18073	-0.30171
C	-2.64348	1.27172	-0.48350
H	2.23373	-2.05732	-0.18483
H	2.87262	-1.43127	-1.70379
H	1.73060	0.85440	-2.20822
H	2.96824	1.70552	-1.29974
H	0.53043	-0.80268	-2.38307
H	0.84709	-2.53297	-2.20155
H	1.15887	2.45218	0.32440

H	0.94428	3.05565	-1.33362
H	-1.91086	-2.45947	-1.83264
H	-1.56287	-0.74220	-2.09533
H	-1.61391	3.10179	-0.91340
H	-1.32428	2.47933	0.72217
H	-3.65272	-1.21060	-0.78013
H	-2.66130	-1.91488	0.50470
H	-3.55473	1.78874	-0.17442
H	-2.74847	0.98986	-1.53452
C	3.29428	0.13223	0.58448
H	3.27675	-0.67136	1.32383
H	3.25358	1.08466	1.11699
H	4.25426	0.08132	0.04623
Rb	0.01435	-0.29604	1.74137

ACE2 Rb MP2 def2tzv

33

O	-0.35993	-1.59622	-0.88005
O	-0.28364	1.48076	-0.69906
O	-2.49742	0.07600	0.38321
N	2.18514	0.01050	-0.25675
C	2.05506	-1.18601	-1.13325
C	2.10953	1.29652	-1.01222
C	0.71247	-1.28926	-1.84700
C	0.98825	2.19626	-0.49800
C	-1.68617	-1.38800	-1.48563
C	-1.44540	2.21052	-0.16949
C	-2.72386	-1.15963	-0.39488
C	-2.67043	1.33317	-0.37445
H	2.20004	-2.07476	-0.50899
H	2.84345	-1.19086	-1.91146

H	1.95393	1.11059	-2.08161
H	3.05909	1.84443	-0.91853
H	0.46108	-0.35202	-2.35240
H	0.76426	-2.09277	-2.59330
H	1.12556	2.40502	0.57271
H	0.97075	3.14648	-1.04814
H	-1.97226	-2.27063	-2.07275
H	-1.63196	-0.52206	-2.15388
H	-1.57524	3.16058	-0.70363
H	-1.29708	2.42124	0.89838
H	-3.71968	-1.12812	-0.85418
H	-2.69766	-1.96132	0.34563
H	-3.56003	1.84411	0.00610
H	-2.80992	1.12400	-1.43941
C	3.42923	-0.07425	0.55829
H	3.40574	-0.98484	1.16285
H	3.48121	0.78947	1.22559
H	4.33533	-0.09092	-0.07169
Rb	-0.00316	-0.44787	1.72981

ACE2 Rb MP2 def2sv

33

O	0.32204	1.75058	0.08060
O	0.30171	-0.87917	-1.35355
O	2.44042	-0.20173	0.24839
N	-2.13575	0.13637	-0.25490
C	-2.06128	1.59244	-0.28772
C	-2.02416	-0.49847	-1.57095
C	-0.73463	2.13856	-0.77766
C	-0.92014	-1.53845	-1.61083
C	1.58831	1.97219	-0.50783

C	1.42833	-1.72454	-1.30107
C	2.65596	1.20144	0.24188
C	2.63415	-0.86286	-0.99481
H	-2.23609	1.96487	0.73819
H	-2.86089	2.03456	-0.92832
H	-1.81550	0.25479	-2.34979
H	-2.98022	-0.98026	-1.85837
H	-0.52340	1.78054	-1.80217
H	-0.80692	3.24517	-0.82394
H	-1.10662	-2.32541	-0.84847
H	-0.89906	-2.03394	-2.60418
H	1.85061	3.05112	-0.49134
H	1.56492	1.65171	-1.56624
H	1.58544	-2.25262	-2.26457
H	1.30234	-2.49926	-0.51418
H	3.64531	1.44621	-0.19276
H	2.66975	1.50964	1.30086
H	3.53995	-1.49629	-0.94169
H	2.77685	-0.13444	-1.81181
C	-3.32547	-0.31297	0.45438
H	-3.32659	0.08542	1.48409
H	-3.32860	-1.41480	0.51488
H	-4.26858	0.00946	-0.04176
Rb	0.00561	-0.59493	1.70567

ACE2 Rb PBE0 def2sv

33

O	-0.30773	-1.12479	-1.35480
O	-0.30877	1.65030	-0.12431
O	-2.44645	-0.05743	0.28332
N	2.15193	0.14794	-0.27319

C	2.08015	-0.74083	-1.42182
C	2.01172	1.56906	-0.57889
C	0.76196	-0.71850	-2.17202
C	0.91226	2.24634	0.21987
C	-1.56535	-0.79780	-1.88725
C	-1.43689	2.10510	0.56570
C	-2.64389	-0.90401	-0.82732
C	-2.63680	1.32604	0.07016
H	2.26162	-1.76995	-1.06274
H	2.88135	-0.52651	-2.16748
H	1.78732	1.70785	-1.64986
H	2.96268	2.10644	-0.39633
H	0.55876	0.29656	-2.56424
H	0.85510	-1.38972	-3.05041
H	1.10421	2.14330	1.30997
H	0.90086	3.33239	-0.00937
H	-1.83153	-1.47484	-2.72582
H	-1.53900	0.23193	-2.29396
H	-1.61184	3.18796	0.39661
H	-1.31323	1.96212	1.66156
H	-3.62450	-0.70934	-1.30412
H	-2.67521	-1.93014	-0.42292
H	-3.53918	1.66447	0.61302
H	-2.79085	1.53985	-1.00241
C	3.31260	-0.12285	0.55010
H	3.31996	-1.18022	0.86944
H	3.28693	0.50152	1.45992
H	4.27443	0.07924	0.02487
Rb	-0.01628	-1.03590	1.53194

ACE2 Rb PBE0 def2tzv

33

O	-0.31825	-1.18852	-1.35133
O	-0.33024	1.73385	-0.21351
O	-2.46755	-0.06720	0.34188
N	2.16740	0.14451	-0.24790
C	2.08445	-0.73700	-1.42233
C	2.03927	1.58266	-0.55276
C	0.76736	-0.68365	-2.16732
C	0.93747	2.26694	0.23253
C	-1.61351	-0.80821	-1.87065
C	-1.47083	2.13805	0.57057
C	-2.67023	-0.94144	-0.79838
C	-2.66785	1.35138	0.09484
H	2.26118	-1.76166	-1.08407
H	2.87730	-0.50545	-2.15613
H	1.84364	1.72966	-1.61791
H	2.98259	2.10103	-0.34001
H	0.53438	0.34282	-2.46503
H	0.85579	-1.28698	-3.07812
H	1.06507	2.08532	1.30744
H	0.97302	3.35006	0.06408
H	-1.88853	-1.45106	-2.71550
H	-1.56580	0.22395	-2.23136
H	-1.66991	3.20975	0.45102
H	-1.28921	1.94665	1.63590
H	-3.65027	-0.75415	-1.25020
H	-2.67551	-1.95192	-0.38884
H	-3.55655	1.67825	0.64049
H	-2.83208	1.53587	-0.96944
C	3.36339	-0.14212	0.55236

H	3.37610	-1.19381	0.84535
H	3.35707	0.46179	1.46094
H	4.29682	0.07101	0.00619
Rb	-0.00421	-1.04939	1.50192

ACE2 Rb PBE def2sv

33

O	-0.31457	-0.94041	-1.51303
O	-0.31101	1.67105	0.11291
O	-2.47267	-0.09402	0.28125
N	2.17210	0.18937	-0.25116
C	2.09303	-0.52934	-1.52732
C	2.02715	1.65190	-0.35066
C	0.76433	-0.40350	-2.26819
C	0.92328	2.21542	0.54499
C	-1.58503	-0.53041	-1.99696
C	-1.45119	2.02958	0.86642
C	-2.67121	-0.77906	-0.95451
C	-2.65755	1.32337	0.25859
H	2.28415	-1.60658	-1.31886
H	2.89490	-0.20404	-2.24701
H	1.79620	1.94246	-1.39886
H	2.98527	2.16476	-0.09461
H	0.55160	0.66662	-2.50559
H	0.86271	-0.93822	-3.24639
H	1.11658	1.95706	1.61890
H	0.91621	3.33285	0.47090
H	-1.86020	-1.08535	-2.93027
H	-1.55248	0.55513	-2.25822
H	-1.63051	3.13493	0.84867
H	-1.32913	1.73565	1.94226

H	-3.65731	-0.51274	-1.40699
H	-2.71022	-1.85948	-0.69265
H	-3.56830	1.58991	0.84530
H	-2.80692	1.68551	-0.78360
C	3.35202	-0.19534	0.51733
H	3.36879	-1.29711	0.67492
H	3.33016	0.28950	1.51806
H	4.31685	0.09224	0.01660
Rb	-0.01341	-1.26371	1.38158

ACE2 Rb PBE def2tzv

33

O	-0.32520	-0.99632	-1.52292
O	-0.32855	1.78518	0.03318
O	-2.49013	-0.10245	0.34566
N	2.18525	0.18906	-0.21811
C	2.09811	-0.51399	-1.52454
C	2.06021	1.67196	-0.31323
C	0.77407	-0.35304	-2.25996
C	0.95616	2.24769	0.56885
C	-1.63578	-0.52123	-1.98284
C	-1.48256	2.07528	0.88352
C	-2.69783	-0.80497	-0.93313
C	-2.68452	1.36118	0.29215
H	2.28113	-1.58397	-1.33909
H	2.89527	-0.17238	-2.22430
H	1.86140	1.97145	-1.35407
H	3.01312	2.15483	-0.02877
H	0.52977	0.71291	-2.39616
H	0.86844	-0.81297	-3.25967
H	1.07694	1.90991	1.61486

H	0.99914	3.35219	0.55552
H	-1.92050	-1.03180	-2.92100
H	-1.57507	0.56081	-2.18534
H	-1.68576	3.16150	0.91808
H	-1.29684	1.73147	1.91790
H	-3.68423	-0.54540	-1.35474
H	-2.70978	-1.87061	-0.66725
H	-3.58319	1.61288	0.87686
H	-2.84131	1.68960	-0.74631
C	3.40249	-0.21219	0.52401
H	3.42187	-1.30369	0.65221
H	3.39909	0.24879	1.52105
H	4.33791	0.09025	0.00594
Rb	-0.00744	-1.30796	1.33959

ACE2 Rb SCS-MP2 def2sv

33

O	-0.32955	-1.73978	-0.25755
O	-0.29551	1.13602	-1.16847
O	-2.44212	0.16254	0.27194
N	2.14185	-0.08617	-0.27958
C	2.06042	-1.51263	-0.59590
C	2.03541	0.79483	-1.45244
C	0.72742	-1.95330	-1.18000
C	0.93092	1.82994	-1.29668
C	-1.60229	-1.84940	-0.87137
C	-1.42745	1.95570	-0.96386
C	-2.66639	-1.21776	0.01101
C	-2.63377	1.04485	-0.83012
H	2.23753	-2.07845	0.33800
H	2.85691	-1.82331	-1.31370

H	1.82979	0.20560	-2.36334
H	2.99403	1.32138	-1.63669
H	0.51324	-1.40383	-2.11545
H	0.79891	-3.03121	-1.43817
H	1.11764	2.46222	-0.40140
H	0.91253	2.50051	-2.18233
H	-1.86857	-2.91458	-1.04214
H	-1.58244	-1.34859	-1.85775
H	-1.58214	2.65287	-1.81443
H	-1.30865	2.57018	-0.04487
H	-3.65940	-1.37382	-0.45693
H	-2.67878	-1.71216	0.99824
H	-3.54366	1.65368	-0.66292
H	-2.76941	0.48062	-1.76970
C	3.33901	0.20913	0.50407
H	3.33416	-0.37981	1.43926
H	3.35087	1.27981	0.77569
H	4.28055	-0.02062	-0.04532
Rb	-0.00725	0.25093	1.80636

ACE2 Rb SCS-MP2 def2tzv

33

O	-0.35986	-1.54591	-0.96999
O	-0.29374	1.53806	-0.62907
O	-2.49552	0.05374	0.38423
N	2.18578	0.03075	-0.26297
C	2.05963	-1.11996	-1.20415
C	2.10308	1.35985	-0.94666
C	0.71247	-1.19625	-1.92246
C	0.98113	2.23036	-0.37498
C	-1.68796	-1.31443	-1.56341

C	-1.45574	2.22662	-0.04865
C	-2.72738	-1.13718	-0.45849
C	-2.67926	1.35177	-0.29930
H	2.21167	-2.04005	-0.62751
H	2.84599	-1.07929	-1.98358
H	1.94015	1.23353	-2.02392
H	3.05261	1.90389	-0.82903
H	0.46034	-0.23972	-2.39048
H	0.76694	-1.96912	-2.70133
H	1.11519	2.36580	0.70833
H	0.97191	3.21620	-0.86034
H	-1.97274	-2.17289	-2.18719
H	-1.63970	-0.42039	-2.19440
H	-1.59613	3.20670	-0.52402
H	-1.30267	2.37415	1.02962
H	-3.72377	-1.07792	-0.91555
H	-2.70506	-1.97836	0.23808
H	-3.57098	1.83427	0.11441
H	-2.82229	1.20380	-1.37444
C	3.43655	-0.09191	0.54152
H	3.41904	-1.03303	1.09929
H	3.48883	0.73663	1.25344
H	4.34054	-0.07248	-0.09223
Rb	0.00426	-0.55209	1.71567

ACE3 Cs B3LYP def2sv

40

O	1.94576	-1.58105	0.93927
O	-2.39591	0.40355	0.09947
O	2.06248	1.18995	0.10290
O	-0.43092	1.89493	1.46199

N	-0.81598	-1.87900	0.90369
C	0.03864	-3.04209	0.66084
C	-2.18320	-2.00669	0.41074
C	1.44985	-2.66054	0.18232
C	-3.03770	-0.75104	0.61782
C	3.11055	-0.94012	0.46760
C	-2.68351	1.63860	0.73362
C	3.06660	0.52765	0.85830
C	-1.45317	2.53516	0.73060
C	1.75801	2.51986	0.49818
C	0.77495	2.60372	1.66998
H	-0.40561	-3.68060	-0.12242
H	0.10945	-3.67473	1.57113
H	-2.74097	-2.83999	0.90472
H	-2.14997	-2.25483	-0.66614
H	2.11432	-3.54794	0.25318
H	1.41624	-2.37789	-0.89148
H	-4.01658	-0.91203	0.12264
H	-3.24079	-0.60680	1.69466
H	3.18874	-1.02466	-0.63555
H	4.02161	-1.40235	0.90037
H	-2.98740	1.46258	1.78280
H	-3.51518	2.16287	0.22118
H	2.86422	0.60456	1.94354
H	4.05567	0.98921	0.66166
H	-1.13929	2.75410	-0.31153
H	-1.72543	3.50614	1.19466
H	1.33504	3.01482	-0.39347
H	2.68126	3.07146	0.76602
H	1.22697	2.15973	2.57282

H	0.57766	3.67355	1.88848
Cs	-0.06965	0.12583	-1.87948
C	-0.74714	-1.44528	2.29639
H	0.29986	-1.46778	2.62639
H	-1.34401	-2.10418	2.97197
H	-1.09779	-0.40748	2.40270

ACE3 Cs B3LYP def2tzv

40

O	3.05140	-0.00711	0.61213
O	-1.95070	-1.40684	0.21319
O	0.82480	1.94468	0.71920
O	-2.05256	1.45913	0.60906
N	0.92350	-2.01286	0.53314
C	2.28307	-2.25880	-0.00295
C	-0.07417	-3.00305	0.07422
C	3.00033	-0.99035	-0.46513
C	-1.48943	-2.74696	0.58660
C	3.13753	1.39095	0.20163
C	-2.92147	-0.83672	1.14246
C	2.21855	2.24557	1.05046
C	-3.22742	0.59891	0.74132
C	-0.16065	2.66227	1.53357
C	-1.33945	1.76395	1.84789
H	2.21656	-2.91188	-0.87627
H	2.90198	-2.78571	0.73593
H	0.18568	-4.02703	0.39921
H	-0.07709	-3.00844	-1.01696
H	4.01523	-1.24282	-0.79005
H	2.47379	-0.56163	-1.32140
H	-2.16206	-3.49372	0.15725

H	-1.52913	-2.84926	1.67277
H	2.87162	1.49459	-0.85335
H	4.16422	1.74436	0.32727
H	-2.52414	-0.89084	2.15940
H	-3.85710	-1.40446	1.11774
H	2.39226	2.04733	2.11146
H	2.43055	3.30044	0.85084
H	-3.69712	0.62373	-0.23969
H	-3.92398	1.02502	1.46970
H	-0.48741	3.55491	0.99629
H	0.29163	2.96942	2.47976
H	-0.98565	0.84648	2.32037
H	-2.01235	2.28211	2.53827
Cs	-0.45902	0.45453	-1.72015
C	0.94653	-1.84949	2.00097
H	1.73527	-1.15181	2.26193
H	1.13019	-2.80533	2.51973
H	0.00479	-1.44100	2.36105

ACE3 Cs B97-1 def2sv

40

O	2.86605	0.11134	0.63458
O	-1.83251	-1.45726	0.20118
O	0.69311	1.90269	0.87769
O	-2.08897	1.34717	0.63756
N	0.99154	-2.01039	0.53366
C	2.35998	-2.17978	0.03784
C	0.04355	-2.99833	0.02564
C	3.01452	-0.85390	-0.37909
C	-1.39699	-2.77305	0.49642
C	2.96632	1.46318	0.25427

C	-2.86172	-0.97894	1.04127
C	2.02829	2.30619	1.10860
C	-3.19998	0.46992	0.68252
C	-0.28433	2.56832	1.65392
C	-1.48283	1.65717	1.87733
H	2.35853	-2.82939	-0.85691
H	2.99539	-2.69318	0.79319
H	0.30908	-4.04221	0.32940
H	0.07291	-2.97510	-1.08066
H	4.08714	-1.03259	-0.61632
H	2.53751	-0.49404	-1.31648
H	-2.05147	-3.51951	-0.00104
H	-1.47363	-2.95424	1.58713
H	2.70162	1.59023	-0.81737
H	4.00317	1.84248	0.38066
H	-2.54909	-1.06121	2.10374
H	-3.79004	-1.58004	0.92868
H	2.28467	2.17712	2.18157
H	2.17405	3.37688	0.84937
H	-3.64291	0.50571	-0.32915
H	-3.96391	0.83760	1.39950
H	-0.60048	3.50899	1.15450
H	0.12420	2.83306	2.65047
H	-1.13781	0.73940	2.39436
H	-2.20777	2.17478	2.54141
Cs	-0.40078	0.49230	-1.75749
C	0.96248	-1.87430	1.98630
H	1.74863	-1.16983	2.29596
H	1.12912	-2.84791	2.51015
H	-0.00384	-1.46111	2.32580

ACE3 Cs B97-1 def2tzv

40

O	2.99262	-0.00941	0.67909
O	-1.99721	-1.34933	0.20741
O	0.81299	1.98450	0.64952
O	-2.05116	1.53157	0.47951
N	0.85497	-1.96980	0.60745
C	2.22852	-2.28402	0.14129
C	-0.14319	-2.97729	0.17977
C	3.00902	-1.05258	-0.33772
C	-1.56926	-2.67657	0.64993
C	3.11624	1.35767	0.19194
C	-2.97910	-0.72539	1.08496
C	2.20380	2.27777	0.98625
C	-3.24462	0.70217	0.61384
C	-0.17144	2.74465	1.41880
C	-1.37759	1.87359	1.72806
H	2.17590	-2.96889	-0.71154
H	2.79697	-2.80253	0.92893
H	0.09899	-3.98573	0.56745
H	-0.12241	-3.04143	-0.91231
H	4.04271	-1.34312	-0.56517
H	2.56250	-0.66899	-1.26274
H	-2.24482	-3.43433	0.23825
H	-1.63244	-2.73099	1.74143
H	2.85564	1.40903	-0.87160
H	4.15104	1.69992	0.30436
H	-2.60997	-0.74366	2.11704
H	-3.92815	-1.27533	1.05771
H	2.36174	2.12991	2.06074

H	2.44222	3.31822	0.73395
H	-3.68765	0.69198	-0.38259
H	-3.95466	1.17151	1.30569
H	-0.46980	3.63053	0.84923
H	0.26406	3.06872	2.37044
H	-1.05103	0.96843	2.24893
H	-2.06550	2.42918	2.37747
Cs	-0.36330	0.34512	-1.71373
C	0.83585	-1.73537	2.06788
H	1.63660	-1.04497	2.32133
H	0.97631	-2.67204	2.63699
H	-0.10860	-1.28480	2.37488

ACE3 Cs CAM-B3LYP def2sv

40

O	2.83428	0.02190	0.68503
O	-1.92507	-1.33553	0.22130
O	0.75739	1.92245	0.75238
O	-2.02806	1.47458	0.47731
N	0.84501	-1.96875	0.61576
C	2.21202	-2.25393	0.19650
C	-0.12751	-2.95602	0.17497
C	2.96983	-1.00799	-0.25755
C	-1.55433	-2.64238	0.60635
C	3.01477	1.33864	0.23731
C	-2.93589	-0.75470	1.00900
C	2.09965	2.26639	1.00796
C	-3.18758	0.67397	0.55330
C	-0.20442	2.67494	1.45644
C	-1.43926	1.83763	1.70517
H	2.20223	-2.95129	-0.65733

H	2.77404	-2.76355	1.00636
H	0.09940	-3.97554	0.56454
H	-0.08518	-3.02454	-0.92612
H	4.03560	-1.26621	-0.42384
H	2.56893	-0.67472	-1.23510
H	-2.23479	-3.38772	0.15199
H	-1.65070	-2.74468	1.70196
H	2.79015	1.41645	-0.84443
H	4.06220	1.67128	0.37675
H	-2.64041	-0.78269	2.07542
H	-3.88800	-1.31427	0.92065
H	2.31150	2.18025	2.09121
H	2.30765	3.31046	0.70187
H	-3.61198	0.66722	-0.46327
H	-3.93568	1.13232	1.22823
H	-0.46690	3.59037	0.89114
H	0.19421	2.98571	2.43971
H	-1.14859	0.94322	2.28494
H	-2.15508	2.42459	2.31323
Cs	-0.30125	0.35688	-1.73179
C	0.77520	-1.69540	2.04116
H	1.58256	-1.00513	2.31174
H	0.87653	-2.62165	2.65248
H	-0.17722	-1.21086	2.30446

ACE3 Cs CAM-B3LYP def2tzv

40

O	2.93498	-0.02679	0.69137
O	-1.99636	-1.31788	0.21489
O	0.81978	2.01438	0.58745
O	-2.02272	1.54084	0.42336

N	0.81832	-1.96204	0.61909
C	2.18637	-2.29151	0.18296
C	-0.17992	-2.95964	0.20363
C	2.97738	-1.08233	-0.29869
C	-1.59246	-2.64006	0.66516
C	3.10230	1.32486	0.20088
C	-2.97083	-0.67061	1.06907
C	2.20056	2.26275	0.96415
C	-3.22124	0.73575	0.56478
C	-0.16854	2.78473	1.32552
C	-1.36077	1.92150	1.65657
H	2.14250	-2.99170	-0.65257
H	2.73405	-2.79710	0.98795
H	0.05593	-3.95965	0.60344
H	-0.15871	-3.03944	-0.88345
H	4.01234	-1.37514	-0.49380
H	2.55649	-0.71496	-1.23712
H	-2.27790	-3.38702	0.26147
H	-1.65798	-2.68242	1.75287
H	2.86532	1.37278	-0.86411
H	4.13927	1.63978	0.33105
H	-2.60403	-0.66710	2.09772
H	-3.91806	-1.21538	1.05322
H	2.32074	2.10601	2.03794
H	2.47260	3.29368	0.72629
H	-3.65598	0.70375	-0.43053
H	-3.92787	1.23071	1.23530
H	-0.47216	3.64361	0.72660
H	0.26255	3.14558	2.26100
H	-1.03173	1.03653	2.20126

H	-2.05423	2.48656	2.28454
Cs	-0.33119	0.30477	-1.68236
C	0.77567	-1.67732	2.05995
H	1.58122	-0.99534	2.30348
H	0.88829	-2.59356	2.65960
H	-0.16171	-1.20139	2.33381

ACE3 Cs MP2 def2tzv

40

O	2.88883	-0.16327	0.77582
O	-2.09510	-1.17816	0.19174
O	0.92670	2.02767	0.49795
O	-1.94025	1.68199	0.32117
N	0.64587	-1.93577	0.65918
C	2.02473	-2.40664	0.33949
C	-0.38070	-2.93777	0.26156
C	2.90942	-1.27378	-0.18857
C	-1.79798	-2.53859	0.68172
C	3.17971	1.16499	0.21498
C	-3.04880	-0.45199	1.04532
C	2.32569	2.19002	0.94144
C	-3.21322	0.95942	0.48824
C	-0.03759	2.85859	1.24851
C	-1.26446	2.02724	1.58293
H	1.97159	-3.16934	-0.44776
H	2.49350	-2.87551	1.22086
H	-0.18020	-3.92493	0.72433
H	-0.33215	-3.06167	-0.82615
H	3.93774	-1.62765	-0.33817
H	2.52039	-0.92210	-1.15179
H	-2.51808	-3.24709	0.25674

H	-1.89772	-2.55631	1.77266
H	2.95113	1.18263	-0.85796
H	4.24075	1.40189	0.35363
H	-2.66925	-0.43738	2.07445
H	-4.02557	-0.95233	1.04020
H	2.38448	2.02703	2.02391
H	2.67256	3.20230	0.70144
H	-3.64562	0.92914	-0.51270
H	-3.87533	1.53070	1.15232
H	-0.31056	3.72941	0.64488
H	0.41869	3.19491	2.18753
H	-0.95753	1.12044	2.11250
H	-1.94732	2.61096	2.21513
Cs	-0.23525	0.23477	-1.69006
C	0.53677	-1.58882	2.10279
H	1.37664	-0.94565	2.35746
H	0.55416	-2.49137	2.74220
H	-0.38765	-1.04162	2.28952

ACE3 Cs MP2 def2tzv

40

O	-2.88883	0.16327	0.77582
O	2.09510	1.17816	0.19174
O	-0.92670	-2.02767	0.49795
O	1.94025	-1.68199	0.32117
N	-0.64587	1.93577	0.65918
C	-2.02473	2.40664	0.33949
C	0.38070	2.93777	0.26156
C	-2.90942	1.27378	-0.18857
C	1.79798	2.53859	0.68172
C	-3.17971	-1.16499	0.21498

C	3.04880	0.45199	1.04532
C	-2.32569	-2.19002	0.94144
C	3.21322	-0.95942	0.48824
C	0.03759	-2.85859	1.24851
C	1.26446	-2.02724	1.58293
H	-1.97159	3.16934	-0.44776
H	-2.49350	2.87551	1.22086
H	0.18020	3.92493	0.72433
H	0.33215	3.06167	-0.82615
H	-3.93774	1.62765	-0.33817
H	-2.52039	0.92210	-1.15179
H	2.51808	3.24709	0.25674
H	1.89772	2.55631	1.77266
H	-2.95113	-1.18263	-0.85796
H	-4.24075	-1.40189	0.35363
H	2.66925	0.43738	2.07445
H	4.02557	0.95233	1.04020
H	-2.38448	-2.02703	2.02391
H	-2.67256	-3.20230	0.70144
H	3.64562	-0.92914	-0.51270
H	3.87533	-1.53070	1.15232
H	0.31056	-3.72941	0.64488
H	-0.41869	-3.19491	2.18753
H	0.95753	-1.12044	2.11250
H	1.94732	-2.61096	2.21513
Cs	0.23525	-0.23477	-1.69006
C	-0.53677	1.58882	2.10279
H	-1.37664	0.94565	2.35746
H	-0.55416	2.49137	2.74220
H	0.38765	1.04162	2.28952

ACE3 Cs PBE0 def2sv

40

O	1.29287	-1.82305	0.82652
O	-2.28841	1.02096	-0.07270
O	2.49620	0.65667	0.09878
O	0.06941	1.82610	1.10697
N	-1.39767	-1.55150	0.78666
C	-0.85629	-2.88929	0.61564
C	-2.71809	-1.34541	0.22723
C	0.62689	-2.89184	0.21286
C	-3.22996	0.08214	0.39232
C	2.65352	-1.68156	0.52457
C	-2.21099	2.24077	0.62310
C	3.09793	-0.29760	0.94324
C	-0.79789	2.78898	0.56928
C	2.45180	1.98451	0.57021
C	1.33300	2.23055	1.57453
H	-1.41142	-3.42227	-0.17514
H	-0.99492	-3.48593	1.54172
H	-3.49199	-2.00563	0.68919
H	-2.69207	-1.60638	-0.84723
H	1.08392	-3.86462	0.48767
H	0.72243	-2.80665	-0.89176
H	-4.18629	0.17728	-0.15900
H	-3.45401	0.27879	1.45732
H	2.83221	-1.81424	-0.56391
H	3.26464	-2.44016	1.05490
H	-2.48545	2.08670	1.68449
H	-2.90738	2.99200	0.19895
H	2.81135	-0.13802	1.99958

H	4.20194	-0.22874	0.87576
H	-0.52149	3.05394	-0.47334
H	-0.77022	3.72709	1.15836
H	2.30934	2.61895	-0.32166
H	3.41817	2.27636	1.02774
H	1.52223	1.65152	2.49530
H	1.33450	3.30169	1.85574
Cs	0.10663	0.09668	-1.67890
C	-1.27544	-1.10479	2.15973
H	-0.27409	-1.36343	2.52993
H	-2.03295	-1.57878	2.82801
H	-1.36659	-0.01036	2.22756

ACE3 Cs PBE0 def2tzv

40

O	2.93382	-0.00702	0.69795
O	-1.98580	-1.32906	0.20591
O	0.79933	2.00977	0.59261
O	-2.03919	1.53492	0.41929
N	0.83005	-1.95018	0.61502
C	2.19812	-2.27349	0.17966
C	-0.15838	-2.95631	0.20471
C	2.98769	-1.05884	-0.29150
C	-1.57249	-2.64297	0.66369
C	3.08600	1.34119	0.20063
C	-2.96100	-0.69158	1.06211
C	2.17622	2.27762	0.95739
C	-3.22408	0.71431	0.56323
C	-0.18596	2.77045	1.33841
C	-1.38196	1.90650	1.65495
H	2.15801	-2.96720	-0.66534

H	2.74804	-2.78847	0.98172
H	0.08043	-3.95678	0.61135
H	-0.13480	-3.04339	-0.88488
H	4.02712	-1.35153	-0.48271
H	2.57302	-0.69315	-1.23765
H	-2.25243	-3.40301	0.26621
H	-1.63594	-2.68158	1.75529
H	2.84860	1.38329	-0.86804
H	4.12159	1.67338	0.32714
H	-2.59447	-0.68799	2.09466
H	-3.90966	-1.24076	1.04865
H	2.30737	2.13711	2.03563
H	2.44160	3.31038	0.70482
H	-3.66389	0.68029	-0.43324
H	-3.93946	1.19657	1.23900
H	-0.48665	3.64513	0.75480
H	0.24104	3.11468	2.28573
H	-1.05496	1.01703	2.20017
H	-2.07591	2.47181	2.28779
Cs	-0.33020	0.30398	-1.68148
C	0.79106	-1.67867	2.05607
H	1.59031	-0.98592	2.30263
H	0.91878	-2.59951	2.65131
H	-0.15374	-1.21632	2.33930

ACE3 Cs PBE def2sv

40

O	2.87537	0.14862	0.63682
O	-1.80905	-1.48829	0.19287
O	0.66434	1.90119	0.87794
O	-2.11630	1.32268	0.63577

N	1.03031	-2.00096	0.52239
C	2.39884	-2.14846	0.01875
C	0.09332	-3.00496	0.02313
C	3.03635	-0.81148	-0.38865
C	-1.34632	-2.80004	0.50096
C	2.95046	1.50797	0.25340
C	-2.84325	-1.02546	1.04690
C	1.99812	2.33422	1.10659
C	-3.21241	0.41494	0.68982
C	-0.32241	2.56065	1.66053
C	-1.51095	1.63859	1.88327
H	2.40078	-2.79189	-0.88879
H	3.04841	-2.66927	0.76868
H	0.37388	-4.05258	0.33174
H	0.11899	-2.98779	-1.09027
H	4.11755	-0.97796	-0.63411
H	2.55102	-0.44669	-1.32942
H	-1.99555	-3.57094	0.01701
H	-1.41255	-2.96945	1.60212
H	2.68213	1.62836	-0.82633
H	3.98625	1.91391	0.37685
H	-2.51659	-1.10227	2.11395
H	-3.76938	-1.64664	0.94767
H	2.25998	2.21430	2.18742
H	2.12681	3.41412	0.84303
H	-3.66382	0.44403	-0.32611
H	-3.98647	0.76744	1.41536
H	-0.64906	3.50792	1.16257
H	0.08672	2.82822	2.66445
H	-1.15531	0.71796	2.40422

H	-2.24542	2.15178	2.55322
Cs	-0.41836	0.49430	-1.75319
C	1.00895	-1.86342	1.97529
H	1.79082	-1.14148	2.27896
H	1.19534	-2.83917	2.50532
H	0.03383	-1.46061	2.32414

ACE3 Cs PBE def2tzv

40

O	2.46454	-1.00667	1.06044
O	-2.47215	-0.20548	0.06795
O	1.64411	1.65619	-0.10658
O	-0.93748	1.87584	1.41969
N	-0.24456	-1.89946	0.94198
C	0.87444	-2.86386	0.78696
C	-1.56207	-2.45855	0.55555
C	2.17195	-2.21274	0.28118
C	-2.72641	-1.48368	0.75592
C	3.33452	-0.02133	0.40685
C	-3.06350	0.97709	0.71550
C	2.83503	1.37802	0.71701
C	-2.12141	2.16364	0.60250
C	0.98474	2.93871	0.18653
C	0.09567	2.91718	1.43086
H	0.59880	-3.63209	0.04674
H	1.06596	-3.39600	1.74064
H	-1.82389	-3.36333	1.15349
H	-1.51321	-2.78071	-0.49689
H	3.00092	-2.93947	0.36134
H	2.07357	-1.94465	-0.78587
H	-3.64717	-1.94550	0.36155

H	-2.87954	-1.29042	1.82937
H	3.34900	-0.17977	-0.68524
H	4.36644	-0.12906	0.78157
H	-3.24829	0.76297	1.78089
H	-4.02492	1.22502	0.23586
H	2.59516	1.45751	1.78947
H	3.62284	2.11268	0.47412
H	-1.85243	2.35202	-0.45127
H	-2.63789	3.06404	0.98239
H	0.39967	3.16945	-0.71467
H	1.73971	3.73646	0.30490
H	0.68869	2.70454	2.32998
H	-0.35953	3.91764	1.55097
Cs	-0.14553	-0.08460	-1.80358
C	-0.26174	-1.33036	2.31151
H	0.75895	-1.04427	2.57992
H	-0.63746	-2.06648	3.05626
H	-0.87887	-0.42538	2.34344

ACE3 Cs B2PLYP def2sv

40

O	2.83611	0.01752	0.69608
O	-1.92925	-1.33216	0.21706
O	0.75854	1.91885	0.76024
O	-2.02959	1.48362	0.47948
N	0.83906	-1.96549	0.61609
C	2.21115	-2.25957	0.20183
C	-0.13226	-2.95882	0.17222
C	2.96976	-1.01302	-0.25545
C	-1.56125	-2.64622	0.60488
C	3.01888	1.33857	0.24072

C	-2.94241	-0.75168	1.01353
C	2.10615	2.26746	1.01702
C	-3.19445	0.67860	0.55638
C	-0.20328	2.67615	1.47229
C	-1.43991	1.83659	1.71713
H	2.20171	-2.95933	-0.65076
H	2.77047	-2.76668	1.01531
H	0.09606	-3.97901	0.56137
H	-0.08896	-3.02450	-0.92905
H	4.03522	-1.27086	-0.42620
H	2.56302	-0.67881	-1.22973
H	-2.24273	-3.38942	0.14823
H	-1.65849	-2.74887	1.70004
H	2.78828	1.41478	-0.83951
H	4.06781	1.66892	0.37527
H	-2.64315	-0.77972	2.07857
H	-3.89473	-1.31156	0.92675
H	2.31997	2.17931	2.09951
H	2.31192	3.31215	0.71107
H	-3.61519	0.67200	-0.46137
H	-3.94299	1.13744	1.23066
H	-0.46371	3.59457	0.91127
H	0.19716	2.97985	2.45717
H	-1.14846	0.93785	2.28893
H	-2.15593	2.41923	2.32941
Cs	-0.29678	0.35984	-1.74302
C	0.76845	-1.70948	2.04967
H	1.58093	-1.02940	2.32943
H	0.86083	-2.64430	2.64934
H	-0.18101	-1.22049	2.31368

ACE3 Cs B2PLYP def2tzv

40

O	-2.95827	0.06100	0.70701
O	2.02370	1.30081	0.21101
O	-0.85033	-2.00772	0.58366
O	2.01350	-1.57087	0.43249
N	-0.79341	1.95955	0.61876
C	-2.17041	2.32393	0.19855
C	0.21141	2.96745	0.20505
C	-2.97938	1.12457	-0.29674
C	1.63036	2.64084	0.66585
C	-3.14114	-1.29854	0.20059
C	2.99087	0.64363	1.09031
C	-2.24549	-2.24909	0.96892
C	3.23248	-0.77368	0.59082
C	0.13250	-2.79532	1.33998
C	1.33466	-1.93656	1.67755
H	-2.11951	3.03422	-0.63026
H	-2.70573	2.82464	1.01669
H	-0.02331	3.96583	0.61492
H	0.18586	3.05021	-0.88290
H	-4.01049	1.42968	-0.49968
H	-2.54979	0.74952	-1.22920
H	2.32024	3.38075	0.25394
H	1.70058	2.68258	1.75423
H	-2.89908	-1.34119	-0.86442
H	-4.18284	-1.60191	0.32912
H	2.60685	0.64807	2.11356
H	3.94346	1.18148	1.08160
H	-2.35922	-2.08817	2.04366

H	-2.52304	-3.27935	0.72973
H	3.67917	-0.75463	-0.40033
H	3.91912	-1.27756	1.27740
H	0.43068	-3.66058	0.74607
H	-0.31267	-3.14276	2.27537
H	1.00946	-1.04262	2.21125
H	2.02045	-2.50547	2.31275
Cs	0.33264	-0.30953	-1.70306
C	-0.74442	1.68224	2.07283
H	-1.55991	1.01300	2.32260
H	-0.83888	2.60535	2.66782
H	0.19011	1.19371	2.33773

ACE3 Cs DSDPBEP86 def2sv

40

O	-2.85035	-0.01181	0.74703
O	2.00073	1.24896	0.20798
O	-0.76070	-1.85358	0.71065
O	2.00643	-1.56046	0.26251
N	-0.73163	1.84665	0.69364
C	-2.10809	2.24946	0.40986
C	0.23561	2.88472	0.35504
C	-2.95864	1.09608	-0.11378
C	1.66494	2.51975	0.73303
C	-3.01516	-1.28859	0.18068
C	3.03098	0.58533	0.90685
C	-2.10060	-2.26257	0.89381
C	3.20474	-0.80926	0.32583
C	0.19002	-2.72489	1.28797
C	1.48835	-1.98162	1.50797
H	-2.11088	3.03227	-0.37051

H	-2.58423	2.69896	1.30918
H	0.01015	3.85328	0.86311
H	0.17967	3.07579	-0.73369
H	-4.01392	1.42763	-0.21637
H	-2.61018	0.82167	-1.12971
H	2.35160	3.29745	0.34183
H	1.77862	2.50902	1.83351
H	-2.76792	-1.27401	-0.90038
H	-4.06378	-1.63973	0.27127
H	2.78337	0.53959	1.98670
H	3.99656	1.12456	0.81364
H	-2.34552	-2.28386	1.97534
H	-2.26499	-3.28054	0.48351
H	3.56148	-0.73221	-0.71532
H	3.97595	-1.34845	0.91255
H	0.35368	-3.60792	0.63629
H	-0.16606	-3.08727	2.27320
H	1.29224	-1.11885	2.17083
H	2.20987	-2.65330	2.01716
Cs	0.17916	-0.19320	-1.68644
C	-0.59559	1.39342	2.07163
H	-1.40270	0.68408	2.29147
H	-0.64416	2.23990	2.79728
H	0.36039	0.86549	2.20924

ACE3 Cs DSDPBEP86 def2tzv

40

O	-2.85018	0.15363	0.77038
O	2.10744	1.16294	0.17498
O	-0.92072	-2.02514	0.48574
O	1.93287	-1.69771	0.26271

N	-0.62557	1.93189	0.67013
C	-1.99612	2.39972	0.34533
C	0.40685	2.92216	0.28538
C	-2.89172	1.27092	-0.16731
C	1.81774	2.50253	0.69335
C	-3.16010	-1.15595	0.20600
C	3.06385	0.41563	0.98612
C	-2.31197	-2.19899	0.90658
C	3.20221	-0.98484	0.40449
C	0.03979	-2.86154	1.21108
C	1.28147	-2.04967	1.52349
H	-1.94296	3.15263	-0.44956
H	-2.45957	2.88410	1.21984
H	0.22129	3.90621	0.75775
H	0.35928	3.06491	-0.79954
H	-3.92137	1.63107	-0.28710
H	-2.53581	0.93405	-1.14829
H	2.54169	3.21775	0.28791
H	1.91880	2.49637	1.78377
H	-2.94943	-1.16632	-0.87039
H	-4.22132	-1.38599	0.35300
H	2.71387	0.38800	2.02519
H	4.04718	0.90180	0.96785
H	-2.38455	-2.07248	1.99291
H	-2.66187	-3.20207	0.63511
H	3.60738	-0.93596	-0.60694
H	3.88638	-1.56608	1.03545
H	0.29037	-3.73569	0.60261
H	-0.39958	-3.19730	2.15751
H	0.99818	-1.14627	2.07170

H	1.96804	-2.64722	2.13701
Cs	0.20239	-0.20252	-1.64495
C	-0.52850	1.56521	2.10121
H	-1.37034	0.92232	2.34702
H	-0.54624	2.45816	2.75255
H	0.39206	1.01237	2.29115

ACE3 Cs HSE06 def2sv

40

O	2.82694	0.06400	0.67374
O	-1.88642	-1.36697	0.22135
O	0.72375	1.93005	0.76482
O	-2.04989	1.44025	0.49803
N	0.89111	-1.96899	0.60423
C	2.25526	-2.21728	0.16331
C	-0.06878	-2.95970	0.15541
C	2.97903	-0.94907	-0.28034
C	-1.49560	-2.66825	0.59584
C	2.98380	1.38484	0.23584
C	-2.90514	-0.81344	1.01394
C	2.05904	2.29305	1.01582
C	-3.18731	0.61223	0.57127
C	-0.24352	2.65597	1.48375
C	-1.46330	1.79703	1.72591
H	2.25004	-2.90141	-0.70291
H	2.84100	-2.73016	0.95650
H	0.16820	-3.98497	0.52970
H	-0.02975	-3.01448	-0.94808
H	4.04994	-1.18171	-0.46121
H	2.56277	-0.61380	-1.25307
H	-2.16819	-3.42317	0.14211

H	-1.58395	-2.77993	1.69343
H	2.75287	1.46766	-0.84608
H	4.02741	1.73538	0.37046
H	-2.60889	-0.84470	2.08211
H	-3.84878	-1.38987	0.92422
H	2.27911	2.20490	2.09893
H	2.25272	3.34289	0.71519
H	-3.61471	0.60538	-0.44548
H	-3.94745	1.04632	1.25110
H	-0.52291	3.57795	0.93461
H	0.15340	2.95875	2.47182
H	-1.15550	0.90007	2.29603
H	-2.18504	2.36605	2.34644
Cs	-0.32309	0.37074	-1.73044
C	0.83327	-1.72537	2.03169
H	1.63100	-1.02347	2.30756
H	0.95944	-2.66071	2.62720
H	-0.12612	-1.26325	2.31592

ACE3 Cs HSE06 def2tzv

40

O	2.91932	-0.00599	0.71060
O	-1.99315	-1.32246	0.20164
O	0.79429	2.02131	0.57229
O	-2.04285	1.54281	0.38389
N	0.81817	-1.94505	0.62359
C	2.18810	-2.27549	0.19883
C	-0.17036	-2.95462	0.22134
C	2.98479	-1.06523	-0.27174
C	-1.58471	-2.63426	0.67390
C	3.08276	1.34022	0.20808

C	-2.97316	-0.67412	1.04604
C	2.16972	2.28334	0.95188
C	-3.23151	0.72557	0.52880
C	-0.19753	2.79087	1.30278
C	-1.39525	1.93050	1.62118
H	2.15062	-2.97234	-0.64328
H	2.72996	-2.78951	1.00668
H	0.06653	-3.95070	0.63893
H	-0.14425	-3.05313	-0.86691
H	4.02593	-1.35928	-0.44839
H	2.58204	-0.70508	-1.22490
H	-2.26586	-3.39546	0.28194
H	-1.65121	-2.66126	1.76534
H	2.85637	1.37786	-0.86283
H	4.11838	1.66761	0.34320
H	-2.61159	-0.65906	2.07993
H	-3.92135	-1.22333	1.03375
H	2.28751	2.14551	2.03161
H	2.44215	3.31378	0.69915
H	-3.66387	0.68070	-0.47012
H	-3.95008	1.21751	1.19348
H	-0.49404	3.65740	0.70588
H	0.22322	3.14727	2.24794
H	-1.07307	1.04800	2.17978
H	-2.09466	2.50348	2.24034
Cs	-0.30774	0.28336	-1.66910
C	0.77239	-1.65486	2.06109
H	1.57055	-0.95938	2.30185
H	0.89661	-2.56794	2.66837
H	-0.17350	-1.18875	2.33315

ACE3 Cs M062X def2sv

40

O	2.59798	-0.11586	0.77739
O	-2.05082	-1.12658	0.12791
O	0.83687	1.97858	0.61472
O	-1.91714	1.67333	0.21090
N	0.56870	-1.96368	0.70619
C	1.93815	-2.39395	0.44378
C	-0.44008	-2.90885	0.25121
C	2.82952	-1.24157	-0.02494
C	-1.85672	-2.45094	0.57469
C	3.02122	1.12338	0.27869
C	-3.05779	-0.42026	0.80907
C	2.18821	2.20185	0.94093
C	-3.14918	0.98680	0.23985
C	-0.10123	2.79181	1.27999
C	-1.38456	2.01280	1.47040
H	1.94328	-3.15936	-0.35002
H	2.37800	-2.86985	1.34419
H	-0.31315	-3.91623	0.70951
H	-0.33404	-3.03892	-0.84119
H	3.89279	-1.55151	0.00521
H	2.59962	-1.00122	-1.08358
H	-2.58004	-3.13432	0.09193
H	-2.03381	-2.50361	1.66467
H	2.88533	1.16929	-0.82201
H	4.09395	1.30301	0.48446
H	-2.83116	-0.39804	1.89379
H	-4.04434	-0.90822	0.68776
H	2.32514	2.14889	2.03802

H	2.52538	3.19748	0.59506
H	-3.48983	0.93658	-0.80662
H	-3.89955	1.55835	0.81885
H	-0.29241	3.71562	0.70147
H	0.27661	3.08057	2.27793
H	-1.15669	1.10454	2.05862
H	-2.10824	2.62771	2.03962
Cs	-0.05195	0.21593	-1.63432
C	0.39037	-1.58859	2.09992
H	1.22434	-0.94647	2.40796
H	0.35454	-2.47871	2.76872
H	-0.54185	-1.01665	2.23095

ACE3 Cs M062X def2tzv

40

O	2.94328	0.02731	0.74586
O	-2.02198	-1.26915	0.20647
O	0.75565	1.90311	0.59303
O	-2.03345	1.57209	0.17670
N	0.74166	-1.81385	0.68132
C	2.13406	-2.23963	0.44204
C	-0.22355	-2.89601	0.40344
C	3.00310	-1.12530	-0.12545
C	-1.64875	-2.53995	0.79963
C	3.04654	1.32860	0.12889
C	-3.05808	-0.55987	0.92441
C	2.12946	2.28762	0.85107
C	-3.25244	0.79417	0.26854
C	-0.22561	2.80404	1.16836
C	-1.50059	2.04226	1.43871
H	2.14081	-3.05362	-0.28551

H	2.58063	-2.62949	1.36658
H	0.03606	-3.81583	0.95121
H	-0.18245	-3.12853	-0.66256
H	4.03553	-1.46892	-0.22329
H	2.64490	-0.85174	-1.12101
H	-2.32960	-3.32026	0.45572
H	-1.73692	-2.46901	1.88576
H	2.76364	1.27433	-0.92583
H	4.07432	1.69130	0.18710
H	-2.76786	-0.46170	1.97452
H	-4.00062	-1.11114	0.88335
H	2.32048	2.25257	1.92636
H	2.30886	3.30353	0.48987
H	-3.58405	0.66221	-0.75889
H	-4.01538	1.35378	0.81523
H	-0.40702	3.63141	0.47974
H	0.14607	3.20488	2.11441
H	-1.28465	1.20035	2.09693
H	-2.22783	2.69875	1.92340
Cs	-0.18851	0.13227	-1.62201
C	0.57892	-1.24855	2.03065
H	1.34909	-0.50287	2.18738
H	0.65451	-2.02846	2.80425
H	-0.38827	-0.76028	2.11854

ACE3 Cs M06 def2sv

40

O	2.94575	0.00724	0.69702
O	-1.98265	-1.26415	0.23770
O	0.75920	1.79022	0.78988
O	-2.03618	1.53038	0.36545

N	0.77840	-1.84325	0.66400
C	2.13447	-2.21718	0.28762
C	-0.19723	-2.86934	0.33730
C	2.96306	-1.04974	-0.21947
C	-1.61304	-2.52151	0.74905
C	3.02238	1.31644	0.21140
C	-3.04187	-0.64265	0.91244
C	2.08683	2.20238	0.99409
C	-3.21780	0.76533	0.38254
C	-0.19278	2.57798	1.45912
C	-1.47593	1.80963	1.62317
H	2.09804	-2.96309	-0.52959
H	2.65699	-2.71838	1.13708
H	0.04563	-3.84824	0.82399
H	-0.16539	-3.04945	-0.75725
H	4.00117	-1.39547	-0.42155
H	2.55515	-0.71090	-1.19874
H	-2.30093	-3.31345	0.38289
H	-1.70220	-2.51323	1.85643
H	2.75231	1.35356	-0.86721
H	4.05415	1.71989	0.29322
H	-2.83945	-0.63585	2.00664
H	-3.99797	-1.19297	0.77140
H	2.33747	2.14435	2.07689
H	2.22775	3.25809	0.67397
H	-3.56397	0.72841	-0.66755
H	-4.00806	1.27308	0.97666
H	-0.37148	3.52702	0.90638
H	0.17347	2.84711	2.47304
H	-1.25077	0.87415	2.17673

H	-2.18413	2.40557	2.23954
Cs	-0.23004	0.27133	-1.74086
C	0.71492	-1.43375	2.05318
H	1.50025	-0.68811	2.24749
H	0.85237	-2.29845	2.74999
H	-0.25423	-0.95918	2.28601

ACE3 Cs M06 def2tzv

40

O	3.02227	0.00922	0.69291
O	-2.03281	-1.24933	0.25344
O	0.78919	1.85188	0.63960
O	-2.02292	1.58401	0.23553
N	0.75660	-1.79066	0.69639
C	2.12233	-2.22060	0.35473
C	-0.22830	-2.85780	0.46199
C	2.98488	-1.10732	-0.21356
C	-1.63767	-2.48878	0.87999
C	3.07592	1.32626	0.11993
C	-3.08372	-0.54519	0.93929
C	2.15721	2.24275	0.88366
C	-3.24480	0.81949	0.30536
C	-0.18037	2.71971	1.26757
C	-1.45336	1.95597	1.50649
H	2.06897	-2.99909	-0.41186
H	2.61524	-2.67134	1.23106
H	0.03303	-3.77637	1.01907
H	-0.21265	-3.11338	-0.60165
H	3.99701	-1.48544	-0.39603
H	2.57928	-0.78751	-1.18110
H	-2.32221	-3.29237	0.59067

H	-1.69930	-2.38077	1.96813
H	2.77488	1.29963	-0.93366
H	4.09656	1.71825	0.16383
H	-2.83717	-0.46436	2.00486
H	-4.03499	-1.08321	0.86081
H	2.37064	2.17944	1.95722
H	2.32180	3.27577	0.55619
H	-3.57199	0.71264	-0.72912
H	-4.01181	1.37658	0.85495
H	-0.36136	3.59402	0.63367
H	0.19786	3.06546	2.23605
H	-1.22523	1.06564	2.09961
H	-2.16009	2.57957	2.06582
Cs	-0.24109	0.14701	-1.67501
C	0.69641	-1.26889	2.06674
H	1.46834	-0.51521	2.18885
H	0.84573	-2.07249	2.80904
H	-0.26562	-0.79593	2.25943

ACE3 Cs MP2 def2sv

40

O	2.84529	0.03206	0.74675
O	-1.96520	-1.26778	0.22407
O	0.74214	1.84969	0.73472
O	-2.02003	1.53157	0.30635
N	0.76538	-1.84775	0.67792
C	2.14342	-2.23213	0.37766
C	-0.19052	-2.89151	0.32772
C	2.96957	-1.05893	-0.13569
C	-1.61806	-2.54722	0.72466
C	3.00020	1.31920	0.19643

C	-3.00473	-0.63423	0.94080
C	2.07920	2.27088	0.92733
C	-3.20804	0.76116	0.37574
C	-0.21389	2.70555	1.33151
C	-1.49714	1.94102	1.55532
H	2.14977	-3.00326	-0.41447
H	2.63325	-2.68813	1.26592
H	0.05003	-3.86604	0.81744
H	-0.14310	-3.06266	-0.76417
H	4.02988	-1.36899	-0.25340
H	2.60466	-0.77621	-1.14255
H	-2.30265	-3.32240	0.32479
H	-1.72255	-2.55782	1.82540
H	2.74757	1.31937	-0.88258
H	4.04624	1.67738	0.28844
H	-2.75003	-0.59619	2.01864
H	-3.96023	-1.19092	0.84772
H	2.32463	2.27312	2.00844
H	2.23319	3.29788	0.53640
H	-3.57431	0.68766	-0.66181
H	-3.98128	1.28289	0.97525
H	-0.39630	3.59300	0.69161
H	0.14721	3.06153	2.31697
H	-1.27837	1.07254	2.20216
H	-2.22392	2.59309	2.08218
Cs	-0.20389	0.22117	-1.71617
C	0.63741	-1.44183	2.07162
H	1.44638	-0.74167	2.30969
H	0.69045	-2.31243	2.76682
H	-0.31629	-0.91809	2.23118

ACE3 Cs SCS-MP2 def2sv

40

O	2.85030	0.02873	0.74195
O	-1.96323	-1.28172	0.22651
O	0.74724	1.86542	0.74472
O	-2.02501	1.52606	0.34415
N	0.78024	-1.87331	0.67213
C	2.16181	-2.24320	0.34767
C	-0.18013	-2.91008	0.29630
C	2.97511	-1.05226	-0.15790
C	-1.61243	-2.57393	0.70078
C	3.01009	1.32589	0.20795
C	-2.99751	-0.65749	0.96512
C	2.08940	2.27461	0.95149
C	-3.21181	0.74865	0.41989
C	-0.21119	2.71216	1.35850
C	-1.49031	1.93500	1.59222
H	2.16467	-3.00194	-0.45754
H	2.66664	-2.70927	1.22259
H	0.05985	-3.89742	0.76099
H	-0.13350	-3.05314	-0.80046
H	4.03815	-1.35108	-0.28687
H	2.59937	-0.75948	-1.15844
H	-2.29591	-3.34050	0.28077
H	-1.71912	-2.60991	1.80122
H	2.75992	1.34230	-0.87188
H	4.05846	1.67721	0.30888
H	-2.73069	-0.63245	2.04080
H	-3.95309	-1.21588	0.87421
H	2.32931	2.25739	2.03429

H	2.25156	3.30722	0.57702
H	-3.59152	0.68756	-0.61463
H	-3.97859	1.26125	1.03642
H	-0.40622	3.60215	0.72484
H	0.15797	3.06430	2.34293
H	-1.25959	1.06420	2.23297
H	-2.21693	2.58070	2.12845
Cs	-0.22217	0.24771	-1.73244
C	0.66308	-1.50773	2.08273
H	1.47534	-0.81529	2.33706
H	0.72012	-2.39867	2.75236
H	-0.28968	-0.98748	2.26597

ACE3 Cs SCS-MP2 def2tzv

40

O	-2.93839	0.09518	0.74136
O	2.04345	1.24543	0.21396
O	-0.87566	-2.02057	0.54744
O	1.98927	-1.61905	0.38893
N	-0.73751	1.93947	0.64255
C	-2.12532	2.35845	0.27833
C	0.27128	2.95600	0.22700
C	-2.96106	1.18342	-0.24846
C	1.69846	2.60546	0.67197
C	-3.16000	-1.25473	0.19949
C	3.01604	0.56964	1.08684
C	-2.27712	-2.23580	0.96045
C	3.23478	-0.85000	0.55769
C	0.09848	-2.82972	1.30980
C	1.31172	-1.97240	1.64771
H	-2.07897	3.10680	-0.52380

H	-2.63211	2.82887	1.13773
H	0.04173	3.95220	0.65630
H	0.23376	3.04584	-0.86513
H	-3.99565	1.50256	-0.43497
H	-2.53401	0.82381	-1.19278
H	2.40142	3.32659	0.23729
H	1.78767	2.65133	1.76336
H	-2.91140	-1.28239	-0.86895
H	-4.21342	-1.53326	0.32405
H	2.63230	0.56192	2.11478
H	3.97573	1.10352	1.07622
H	-2.36845	-2.06463	2.03999
H	-2.57666	-3.26483	0.72414
H	3.67800	-0.82256	-0.43961
H	3.90979	-1.38587	1.23888
H	0.38999	-3.70066	0.71339
H	-0.35749	-3.16803	2.24881
H	0.98557	-1.06892	2.17268
H	2.00141	-2.54178	2.28632
Cs	0.29435	-0.27313	-1.71280
C	-0.64911	1.64806	2.10245
H	-1.47940	0.99607	2.36888
H	-0.69831	2.57291	2.70808
H	0.28379	1.12829	2.32713

ACE3 K B3LYP def2sv

40

O	2.69773	0.08949	0.33072
O	-2.02247	-1.22919	-0.28164
O	0.69922	2.05274	0.12070
O	-2.03398	1.60516	-0.34220

N	0.69992	-1.89329	0.38053
C	2.08710	-2.23038	0.03932
C	-0.27925	-2.89993	-0.03115
C	2.87926	-1.03291	-0.50373
C	-1.72663	-2.50486	0.26742
C	2.96960	1.36226	-0.21272
C	-3.07898	-0.53071	0.35071
C	2.04315	2.38749	0.42181
C	-3.23352	0.84726	-0.28799
C	-0.28891	2.93166	0.63397
C	-1.58044	2.16873	0.87737
H	2.10218	-3.00732	-0.74406
H	2.61344	-2.66284	0.91604
H	-0.11181	-3.88468	0.46675
H	-0.16674	-3.07150	-1.11714
H	3.95190	-1.30868	-0.58606
H	2.53514	-0.79664	-1.53316
H	-2.40075	-3.27441	-0.15861
H	-1.90421	-2.48836	1.35872
H	2.82125	1.35903	-1.31209
H	4.02059	1.66477	-0.02600
H	-2.87485	-0.44616	1.43693
H	-4.04164	-1.07086	0.23972
H	2.19152	2.39961	1.52020
H	2.29957	3.39265	0.03053
H	-3.55302	0.73136	-1.33704
H	-4.02517	1.40316	0.25217
H	-0.46043	3.76645	-0.07487
H	0.03760	3.36349	1.59935
H	-1.39745	1.38248	1.63463

H	-2.33944	2.86733	1.28407
K	-0.14609	0.16296	-1.66661
C	0.57693	-1.51495	1.78637
H	1.38322	-0.81440	2.03937
H	0.64079	-2.39530	2.46839
H	-0.37909	-1.00003	1.97501

ACE3 K B3LYP def2tzv

40

O	2.84706	0.03971	0.37856
O	-2.09985	-1.23628	-0.29587
O	0.74883	2.05846	0.06208
O	-2.06499	1.65474	-0.34532
N	0.68762	-1.88955	0.36615
C	2.07514	-2.25815	-0.01047
C	-0.31510	-2.90735	-0.02783
C	2.89222	-1.08290	-0.55300
C	-1.75329	-2.52192	0.31444
C	3.06148	1.36472	-0.19477
C	-3.12863	-0.48642	0.41816
C	2.12461	2.36560	0.45185
C	-3.29845	0.87629	-0.23907
C	-0.26428	2.96664	0.60473
C	-1.53759	2.20022	0.90456
H	2.04920	-3.01099	-0.80137
H	2.59821	-2.71352	0.84071
H	-0.12907	-3.87760	0.46515
H	-0.22973	-3.07346	-1.10319
H	3.92791	-1.40044	-0.71135
H	2.49272	-0.76749	-1.52140
H	-2.42779	-3.29574	-0.06041

H	-1.88987	-2.45518	1.39553
H	2.89314	1.34339	-1.27479
H	4.09370	1.67884	-0.01939
H	-2.84524	-0.39348	1.47023
H	-4.08789	-1.01146	0.37276
H	2.21329	2.32363	1.54061
H	2.39487	3.37109	0.11532
H	-3.63443	0.75526	-1.26672
H	-4.05552	1.44262	0.31122
H	-0.45277	3.76433	-0.11687
H	0.09262	3.41321	1.53627
H	-1.32774	1.39813	1.61458
H	-2.27156	2.88030	1.34754
K	-0.22123	0.16305	-1.70524
C	0.60564	-1.53072	1.79896
H	1.40568	-0.83595	2.02965
H	0.70086	-2.41536	2.44939
H	-0.33938	-1.04048	2.02204

ACE3 K B97-1 def2sv

40

O	2.76599	0.05915	0.33446
O	-2.04777	-1.20739	-0.27452
O	0.72288	1.98922	0.15527
O	-2.01849	1.62079	-0.35069
N	0.67358	-1.85849	0.37713
C	2.05742	-2.23775	0.06311
C	-0.31211	-2.87840	0.00949
C	2.88603	-1.07129	-0.49723
C	-1.75812	-2.46736	0.30632
C	2.99365	1.32483	-0.23977

C	-3.10581	-0.49674	0.33574
C	2.06832	2.34874	0.40630
C	-3.22936	0.88468	-0.31072
C	-0.24790	2.88728	0.66051
C	-1.56346	2.15324	0.87961
H	2.06091	-3.03076	-0.70667
H	2.56325	-2.66607	0.95648
H	-0.14245	-3.84586	0.54255
H	-0.20861	-3.08815	-1.07255
H	3.94670	-1.39085	-0.59756
H	2.53069	-0.82797	-1.52271
H	-2.43914	-3.24650	-0.09510
H	-1.92817	-2.42068	1.40023
H	2.80941	1.29974	-1.33517
H	4.04548	1.65084	-0.09296
H	-2.92107	-0.41107	1.42746
H	-4.07502	-1.02493	0.20817
H	2.25212	2.38444	1.50081
H	2.29726	3.35145	-0.01257
H	-3.53675	0.77020	-1.36520
H	-4.02105	1.45513	0.21784
H	-0.38996	3.73445	-0.04325
H	0.07510	3.30277	1.63670
H	-1.40627	1.35082	1.62852
H	-2.30845	2.86665	1.29104
K	-0.15116	0.14728	-1.69470
C	0.54774	-1.41816	1.76590
H	1.36177	-0.71493	1.99169
H	0.59776	-2.27087	2.48564
H	-0.40591	-0.88522	1.92418

ACE3 K B97-1 def2tzv

40

O	2.84665	0.01562	0.38709
O	-2.11244	-1.21508	-0.30025
O	0.76904	2.05381	0.05315
O	-2.04801	1.67583	-0.34559
N	0.66208	-1.88319	0.36211
C	2.05047	-2.27687	0.00965
C	-0.34354	-2.90159	-0.02813
C	2.89130	-1.11331	-0.53304
C	-1.78342	-2.50286	0.30896
C	3.07286	1.32974	-0.20009
C	-3.13171	-0.45918	0.41565
C	2.14500	2.34820	0.44236
C	-3.28721	0.91139	-0.23918
C	-0.23153	2.95881	0.61652
C	-1.51224	2.19353	0.90953
H	2.02495	-3.03683	-0.77807
H	2.55612	-2.73259	0.87434
H	-0.16377	-3.87071	0.47461
H	-0.25602	-3.07524	-1.10487
H	3.92696	-1.44675	-0.67588
H	2.50596	-0.80344	-1.51227
H	-2.46399	-3.27278	-0.07059
H	-1.92286	-2.43789	1.39284
H	2.89772	1.30105	-1.28180
H	4.11060	1.63861	-0.03249
H	-2.84773	-0.37151	1.47123
H	-4.09991	-0.97299	0.36824
H	2.23459	2.30895	1.53399

H	2.42904	3.35093	0.10055
H	-3.62745	0.79474	-1.26860
H	-4.03974	1.48385	0.31629
H	-0.41991	3.77343	-0.08998
H	0.13250	3.38576	1.55766
H	-1.30121	1.37555	1.60569
H	-2.23983	2.87226	1.37119
K	-0.20975	0.16206	-1.71559
C	0.56805	-1.51538	1.79324
H	1.37906	-0.83134	2.03033
H	0.63959	-2.39982	2.45080
H	-0.37402	-1.00625	2.00025

ACE3 K CAM-B3LYP def2sv

40

O	2.69737	0.06706	0.31990
O	-2.02013	-1.20109	-0.27730
O	0.71063	2.00163	0.14081
O	-1.99769	1.60452	-0.36348
N	0.67124	-1.86211	0.36903
C	2.05071	-2.23133	0.06139
C	-0.30769	-2.87699	0.00104
C	2.86619	-1.06430	-0.49293
C	-1.74388	-2.46262	0.29400
C	2.96325	1.32511	-0.24040
C	-3.08333	-0.49512	0.31786
C	2.05177	2.35178	0.39880
C	-3.20538	0.87281	-0.33454
C	-0.26033	2.89096	0.64644
C	-1.56171	2.14964	0.86209
H	2.06223	-3.02594	-0.70194

H	2.55334	-2.65047	0.95649
H	-0.13786	-3.83946	0.53491
H	-0.20326	-3.08860	-1.07720
H	3.93270	-1.35834	-0.56265
H	2.53278	-0.83777	-1.52620
H	-2.42907	-3.23041	-0.11247
H	-1.91671	-2.41879	1.38398
H	2.79272	1.30937	-1.33512
H	4.01678	1.62590	-0.07834
H	-2.90671	-0.40361	1.40684
H	-4.04440	-1.02929	0.18564
H	2.22853	2.38243	1.49099
H	2.28683	3.35115	-0.01508
H	-3.50213	0.75033	-1.38778
H	-3.99878	1.44648	0.18063
H	-0.40795	3.73411	-0.05537
H	0.06308	3.30607	1.61871
H	-1.40146	1.35497	1.61336
H	-2.31760	2.85321	1.26092
K	-0.12515	0.13931	-1.62307
C	0.53698	-1.41823	1.74831
H	1.34807	-0.71745	1.97655
H	0.58139	-2.26766	2.46682
H	-0.41423	-0.88548	1.89956

ACE3 K CAM-B3LYP def2tzv

40

O	2.78792	0.01117	0.37559
O	-2.08825	-1.19799	-0.29436
O	0.76980	2.06426	0.03713
O	-2.02088	1.66305	-0.35580

N	0.65140	-1.88923	0.35560
C	2.03236	-2.27504	0.01181
C	-0.35155	-2.89365	-0.03836
C	2.86375	-1.11816	-0.52767
C	-1.77810	-2.48837	0.29742
C	3.04740	1.31505	-0.19772
C	-3.10997	-0.44359	0.40199
C	2.13782	2.33623	0.43891
C	-3.25845	0.91115	-0.26076
C	-0.23061	2.96045	0.59201
C	-1.49507	2.19171	0.88884
H	2.01369	-3.04042	-0.76515
H	2.53343	-2.71947	0.87982
H	-0.17471	-3.86067	0.45932
H	-0.26489	-3.06486	-1.11155
H	3.90204	-1.43662	-0.65059
H	2.49306	-0.81991	-1.51176
H	-2.46588	-3.24233	-0.08889
H	-1.91896	-2.43093	1.37735
H	2.87899	1.29406	-1.27677
H	4.08683	1.59831	-0.02279
H	-2.83486	-0.34859	1.45496
H	-4.07145	-0.96029	0.35092
H	2.21548	2.28641	1.52677
H	2.43449	3.33477	0.10979
H	-3.58838	0.78687	-1.28845
H	-4.01040	1.49129	0.27918
H	-0.42605	3.76376	-0.11891
H	0.13350	3.39732	1.52376
H	-1.27717	1.38162	1.58551

H	-2.22900	2.86105	1.34411
K	-0.18714	0.15123	-1.64820
C	0.54455	-1.50435	1.77077
H	1.35333	-0.82371	2.00767
H	0.60459	-2.37900	2.43556
H	-0.39328	-0.99001	1.96286

ACE3 K MP2 def2tzv

40

O	2.82778	-0.09368	0.42215
O	-2.13023	-1.11389	-0.28920
O	0.86889	2.05350	-0.01457
O	-1.97636	1.73937	-0.33020
N	0.57282	-1.86556	0.34344
C	1.95849	-2.34917	0.07436
C	-0.44642	-2.88193	-0.03917
C	2.84970	-1.24330	-0.49600
C	-1.87522	-2.44423	0.29356
C	3.14062	1.20752	-0.18927
C	-3.14017	-0.34224	0.45043
C	2.25446	2.26469	0.44627
C	-3.26229	1.03371	-0.19959
C	-0.11327	2.94458	0.63577
C	-1.37380	2.15558	0.94657
H	1.92130	-3.15211	-0.67246
H	2.41205	-2.76959	0.98696
H	-0.27549	-3.84097	0.48919
H	-0.35332	-3.06726	-1.11445
H	3.87779	-1.60563	-0.62456
H	2.46697	-0.93117	-1.47552
H	-2.58520	-3.16951	-0.12022

H	-2.02402	-2.39516	1.37789
H	2.96255	1.17193	-1.27093
H	4.19331	1.45514	-0.01222
H	-2.83364	-0.26650	1.50106
H	-4.11485	-0.84449	0.40668
H	2.28905	2.17813	1.53856
H	2.59457	3.26323	0.14587
H	-3.62973	0.94373	-1.22219
H	-3.96056	1.64564	0.38617
H	-0.33357	3.78749	-0.02616
H	0.30024	3.32093	1.57928
H	-1.11722	1.28057	1.55150
H	-2.08228	2.78893	1.49695
K	-0.21866	0.17710	-1.79855
C	0.42853	-1.46452	1.77054
H	1.26562	-0.81695	2.02408
H	0.42427	-2.34318	2.44201
H	-0.49729	-0.90580	1.91452

ACE3 K PBE0 def2sv

40

O	2.70389	0.07717	0.33294
O	-2.01515	-1.20857	-0.27312
O	0.70156	1.99829	0.14494
O	-2.00949	1.60050	-0.35780
N	0.67940	-1.85702	0.37123
C	2.05702	-2.21845	0.06090
C	-0.29383	-2.87133	-0.00040
C	2.86870	-1.04691	-0.48523
C	-1.72964	-2.46511	0.29637
C	2.95628	1.33384	-0.23038

C	-3.07082	-0.50822	0.33379
C	2.03714	2.35640	0.40164
C	-3.20679	0.85849	-0.31532
C	-0.26932	2.88534	0.64511
C	-1.56976	2.14554	0.86278
H	2.07137	-3.00723	-0.71043
H	2.56271	-2.64730	0.95191
H	-0.12269	-3.83967	0.52653
H	-0.18958	-3.07725	-1.08144
H	3.93589	-1.34302	-0.56301
H	2.53255	-0.81530	-1.51828
H	-2.41127	-3.24016	-0.10639
H	-1.89902	-2.42445	1.38910
H	2.78826	1.31338	-1.32701
H	4.00804	1.64678	-0.07003
H	-2.88400	-0.41652	1.42301
H	-4.03464	-1.04361	0.21367
H	2.21441	2.39572	1.49517
H	2.27098	3.35566	-0.01726
H	-3.51375	0.73458	-1.36694
H	-4.00366	1.42385	0.20686
H	-0.41770	3.72889	-0.05863
H	0.04956	3.30597	1.61824
H	-1.40846	1.35303	1.61848
H	-2.32291	2.85255	1.26494
K	-0.13302	0.13794	-1.65963
C	0.54671	-1.42394	1.75000
H	1.35345	-0.71625	1.97989
H	0.60049	-2.27609	2.46701
H	-0.40894	-0.89838	1.90786

ACE3 K PBE0 def2tzv

40

O	2.80152	0.01328	0.39163
O	-2.09176	-1.20348	-0.29457
O	0.76671	2.05538	0.03998
O	-2.02960	1.66594	-0.34768
N	0.65317	-1.87925	0.35455
C	2.03197	-2.26691	0.01154
C	-0.34501	-2.88671	-0.03508
C	2.86872	-1.10913	-0.51691
C	-1.77117	-2.48560	0.30336
C	3.04803	1.31432	-0.18804
C	-3.09868	-0.45089	0.41933
C	2.13069	2.33568	0.43825
C	-3.25826	0.90664	-0.23471
C	-0.22813	2.94869	0.60194
C	-1.49689	2.18516	0.89493
H	2.01306	-3.02522	-0.77659
H	2.53155	-2.72613	0.87699
H	-0.16751	-3.85653	0.46491
H	-0.25943	-3.06131	-1.11101
H	3.90695	-1.43723	-0.64636
H	2.49789	-0.80469	-1.50273
H	-2.45669	-3.25142	-0.07291
H	-1.90595	-2.42471	1.38751
H	2.88330	1.28795	-1.27079
H	4.08655	1.61385	-0.01402
H	-2.80923	-0.35918	1.47245
H	-4.06698	-0.96342	0.38334
H	2.21181	2.29846	1.52983

H	2.42928	3.33423	0.09965
H	-3.60605	0.78476	-1.26012
H	-4.00988	1.47883	0.32057
H	-0.42165	3.76455	-0.10041
H	0.13472	3.37710	1.54188
H	-1.28149	1.37148	1.59334
H	-2.22453	2.86108	1.35854
K	-0.20095	0.15561	-1.69907
C	0.55149	-1.50824	1.77118
H	1.36002	-0.82397	2.01139
H	0.61864	-2.38911	2.43230
H	-0.39001	-0.99821	1.97298

ACE3 K PBE def2sv

40

O	2.78006	0.07773	0.34337
O	-2.04159	-1.22602	-0.27440
O	0.70505	1.98589	0.15780
O	-2.03791	1.61200	-0.36157
N	0.69186	-1.85424	0.37195
C	2.07653	-2.22345	0.04677
C	-0.29084	-2.88433	0.02039
C	2.89838	-1.04873	-0.50512
C	-1.73546	-2.48083	0.32638
C	2.99020	1.35302	-0.23325
C	-3.10569	-0.51810	0.34417
C	2.05209	2.36426	0.41180
C	-3.24584	0.85733	-0.30890
C	-0.27446	2.88860	0.65804
C	-1.58685	2.15152	0.87572
H	2.07931	-3.01171	-0.73759

H	2.59234	-2.66665	0.93713
H	-0.11244	-3.85424	0.56333
H	-0.19222	-3.10569	-1.06689
H	3.96586	-1.36904	-0.61990
H	2.53474	-0.79214	-1.53314
H	-2.41993	-3.27848	-0.05346
H	-1.89484	-2.41541	1.42889
H	2.80531	1.32420	-1.33623
H	4.04434	1.69926	-0.08892
H	-2.91196	-0.42585	1.44191
H	-4.07985	-1.05673	0.22955
H	2.23504	2.40471	1.51427
H	2.27374	3.37646	-0.00863
H	-3.55665	0.73435	-1.36890
H	-4.04848	1.42459	0.22252
H	-0.41688	3.73956	-0.05352
H	0.04485	3.31272	1.64025
H	-1.42854	1.34733	1.63376
H	-2.34036	2.86805	1.28719
K	-0.16054	0.14108	-1.70184
C	0.57783	-1.41102	1.76131
H	1.39081	-0.69170	1.97263
H	0.64869	-2.26499	2.48977
H	-0.38579	-0.88584	1.93167

ACE3 K PBE def2tzv

40

O	2.86706	0.00283	0.40800
O	-2.12697	-1.21751	-0.30081
O	0.77933	2.04431	0.04274
O	-2.05412	1.68951	-0.35186

N	0.65849	-1.88445	0.35520
C	2.04334	-2.27994	-0.01504
C	-0.35375	-2.90592	-0.01640
C	2.88876	-1.11537	-0.54253
C	-1.78673	-2.50327	0.33235
C	3.09750	1.32930	-0.17806
C	-3.13203	-0.44890	0.44208
C	2.16001	2.34379	0.45037
C	-3.29783	0.91632	-0.21456
C	-0.22283	2.96343	0.60941
C	-1.50454	2.20835	0.90971
H	2.00534	-3.02778	-0.82340
H	2.55552	-2.76386	0.84079
H	-0.17187	-3.87848	0.49770
H	-0.27428	-3.09391	-1.09822
H	3.92569	-1.46070	-0.70755
H	2.49373	-0.77705	-1.51655
H	-2.47730	-3.28630	-0.02320
H	-1.91199	-2.41590	1.42393
H	2.94325	1.29973	-1.27009
H	4.13838	1.64448	0.00818
H	-2.82016	-0.35938	1.49760
H	-4.11105	-0.96084	0.42221
H	2.23631	2.30875	1.55081
H	2.45030	3.35328	0.10869
H	-3.65781	0.79770	-1.24469
H	-4.04673	1.49393	0.35535
H	-0.40792	3.78338	-0.10361
H	0.14789	3.39471	1.55439
H	-1.29647	1.38527	1.61223

H	-2.23036	2.89761	1.37697
K	-0.23164	0.15968	-1.74823
C	0.58560	-1.52056	1.78987
H	1.40194	-0.82859	2.01555
H	0.67303	-2.41172	2.44950
H	-0.36105	-1.01370	2.01556

ACE3 K B2PLYP def2tzv

40

O	2.90300	0.08336	0.37582
O	-2.03726	-1.25347	-0.24001
O	0.71897	2.04601	0.11131
O	-2.14550	1.63314	-0.23737
N	0.77122	-1.87583	0.37809
C	2.15997	-2.20840	-0.05068
C	-0.22868	-2.90475	-0.03075
C	2.89890	-0.99488	-0.61252
C	-1.66527	-2.54475	0.33992
C	3.05199	1.43713	-0.17148
C	-3.11549	-0.57443	0.48028
C	2.08680	2.37632	0.52109
C	-3.34331	0.79690	-0.14229
C	-0.31436	2.85936	0.76596
C	-1.53820	2.00985	1.03902
H	2.12653	-2.95622	-0.84636
H	2.72916	-2.64638	0.77863
H	-0.02079	-3.88120	0.43949
H	-0.15945	-3.03604	-1.11188
H	3.92814	-1.26959	-0.87273
H	2.39538	-0.64995	-1.51975
H	-2.33308	-3.32515	-0.04389

H	-1.78830	-2.50087	1.42870
H	2.85942	1.43321	-1.24631
H	4.07393	1.78294	-0.00351
H	-2.84803	-0.49750	1.53746
H	-4.04559	-1.14574	0.40459
H	2.17596	2.27685	1.60664
H	2.32372	3.40677	0.23662
H	-3.68406	0.69206	-1.16943
H	-4.11527	1.31638	0.43527
H	-0.56588	3.70767	0.12698
H	0.06111	3.23338	1.72192
H	-1.24345	1.12076	1.59813
H	-2.25663	2.58610	1.63132
K	-0.36752	0.30978	-2.01206
C	0.72493	-1.58261	1.82556
H	1.52799	-0.88219	2.05771
H	0.85172	-2.49832	2.43551
H	-0.22560	-1.11272	2.09476

ACE3 K DSDPBEP86 def2tzv

40

O	-2.89989	-0.01264	0.37935
O	2.08757	1.20480	-0.28705
O	-0.75325	-1.95386	0.09543
O	2.03702	-1.64946	-0.38863
N	-0.64915	1.79939	0.33345
C	-2.04229	2.25055	0.09226
C	0.33231	2.87398	0.04903
C	-2.91334	1.14842	-0.50668
C	1.76894	2.47161	0.37378
C	-3.05821	-1.31550	-0.25786

C	3.15080	0.45515	0.37418
C	-2.13381	-2.30877	0.41844
C	3.28869	-0.89619	-0.31517
C	0.23654	-2.87716	0.65183
C	1.52944	-2.12534	0.89697
H	-2.03191	3.07796	-0.62637
H	-2.49810	2.63091	1.02090
H	0.11741	3.78588	0.63867
H	0.25073	3.13736	-1.01044
H	-3.94104	1.50777	-0.64157
H	-2.51915	0.86481	-1.49000
H	2.45365	3.25313	0.02606
H	1.90359	2.35648	1.45469
H	-2.81600	-1.25133	-1.32544
H	-4.09510	-1.65481	-0.15813
H	2.90543	0.34278	1.43736
H	4.10544	0.99006	0.29753
H	-2.27325	-2.27993	1.50538
H	-2.35650	-3.31907	0.05315
H	3.59133	-0.76146	-1.35399
H	4.05134	-1.48760	0.20658
H	0.39338	-3.70999	-0.04182
H	-0.12109	-3.27121	1.61074
H	1.33953	-1.28316	1.56836
H	2.26437	-2.79675	1.35854
K	0.18323	-0.12652	-1.69923
C	-0.49920	1.26001	1.70399
H	-1.29263	0.53674	1.87288
H	-0.55776	2.05971	2.46492
H	0.45815	0.74865	1.80584

ACE3 K DSDPBEP86 def2sv

40

O	2.69397	0.04170	0.32233
O	-2.02520	-1.17045	-0.28571
O	0.72956	1.95680	0.15952
O	-1.96788	1.62794	-0.38598
N	0.63682	-1.83099	0.36126
C	2.01711	-2.24299	0.10376
C	-0.34056	-2.86084	0.02389
C	2.85760	-1.10666	-0.47413
C	-1.77311	-2.42971	0.31013
C	2.96739	1.28710	-0.27199
C	-3.09293	-0.45296	0.29550
C	2.07606	2.32545	0.37827
C	-3.18876	0.90978	-0.37295
C	-0.22739	2.85900	0.67742
C	-1.53780	2.12615	0.86562
H	2.02840	-3.06673	-0.63229
H	2.49228	-2.63681	1.02785
H	-0.16893	-3.80903	0.58712
H	-0.23836	-3.10034	-1.05110
H	3.92278	-1.41613	-0.52989
H	2.53011	-0.89841	-1.51484
H	-2.46999	-3.19481	-0.08712
H	-1.94592	-2.36531	1.40103
H	2.76798	1.25635	-1.36357
H	4.03009	1.57717	-0.13980
H	-2.92782	-0.35356	1.38745
H	-4.05833	-0.98120	0.15407
H	2.28332	2.37107	1.46638

H	2.29220	3.32167	-0.05852
H	-3.46379	0.77940	-1.43275
H	-3.98644	1.49971	0.12181
H	-0.35833	3.71890	-0.01018
H	0.09791	3.24823	1.66243
H	-1.38427	1.30334	1.58914
H	-2.29192	2.82307	1.28478
K	-0.09409	0.11902	-1.61504
C	0.47855	-1.34175	1.72533
H	1.29628	-0.64456	1.94768
H	0.49127	-2.17049	2.47153
H	-0.46917	-0.78923	1.82925

ACE3 K HSE06 def2sv

40

O	2.68971	0.08565	0.33111
O	-2.00852	-1.21536	-0.27419
O	0.69324	2.00898	0.14016
O	-2.01358	1.59495	-0.36165
N	0.68559	-1.86055	0.36925
C	2.06474	-2.21550	0.05778
C	-0.28493	-2.87676	-0.00442
C	2.87086	-1.03811	-0.48506
C	-1.72144	-2.47335	0.29310
C	2.94841	1.34371	-0.22765
C	-3.06610	-0.51724	0.33361
C	2.02849	2.36558	0.40422
C	-3.20824	0.84727	-0.31855
C	-0.28191	2.89260	0.64004
C	-1.57844	2.14613	0.85871
H	2.08257	-3.00253	-0.71521

H	2.57217	-2.64398	0.94798
H	-0.11169	-3.84550	0.52116
H	-0.17989	-3.08082	-1.08572
H	3.94104	-1.32483	-0.55486
H	2.54004	-0.80995	-1.52079
H	-2.40244	-3.24817	-0.11084
H	-1.89064	-2.43438	1.38598
H	2.78421	1.32664	-1.32507
H	4.00034	1.65318	-0.06240
H	-2.87712	-0.42256	1.42224
H	-4.02786	-1.05680	0.21657
H	2.20076	2.40067	1.49862
H	2.26482	3.36604	-0.01008
H	-3.51377	0.71946	-1.37000
H	-4.00758	1.41046	0.20203
H	-0.43442	3.73430	-0.06486
H	0.03555	3.31593	1.61235
H	-1.41360	1.35628	1.61647
H	-2.33642	2.84952	1.25793
K	-0.12674	0.13765	-1.64664
C	0.55153	-1.43029	1.74895
H	1.35510	-0.71914	1.97930
H	0.60982	-2.28357	2.46431
H	-0.40670	-0.90982	1.90833

ACE3 K HSE06 def2tzv

40

O	2.79956	0.01600	0.39207
O	-2.09058	-1.20824	-0.29422
O	0.76364	2.05784	0.03796
O	-2.03089	1.66211	-0.35072

N	0.65599	-1.88024	0.35230
C	2.03538	-2.26596	0.00909
C	-0.34144	-2.88975	-0.03451
C	2.87041	-1.10655	-0.51790
C	-1.76716	-2.48998	0.30608
C	3.04677	1.31894	-0.18601
C	-3.09717	-0.45414	0.42103
C	2.12778	2.33859	0.43973
C	-3.25962	0.90098	-0.23623
C	-0.23358	2.95353	0.59508
C	-1.50132	2.19013	0.89099
H	2.01738	-3.02339	-0.77946
H	2.53522	-2.72532	0.87402
H	-0.16160	-3.85837	0.46643
H	-0.25734	-3.06573	-1.11001
H	3.90926	-1.43175	-0.64619
H	2.50000	-0.80173	-1.50341
H	-2.45293	-3.25646	-0.06714
H	-1.89980	-2.42644	1.39004
H	2.88429	1.29314	-1.26882
H	4.08462	1.61793	-0.00950
H	-2.80441	-0.35948	1.47265
H	-4.06427	-0.96837	0.38922
H	2.20607	2.30021	1.53110
H	2.42567	3.33756	0.10298
H	-3.60690	0.77640	-1.26111
H	-4.01141	1.47378	0.31746
H	-0.42742	3.76461	-0.11208
H	0.12866	3.38764	1.53222
H	-1.28692	1.38113	1.59458

H	-2.23123	2.86745	1.34806
K	-0.20078	0.15296	-1.69178
C	0.55419	-1.50452	1.76784
H	1.36149	-0.81815	2.00497
H	0.62311	-2.38291	2.43166
H	-0.38795	-0.99564	1.96808

ACE3 K M062X def2sv

40

O	2.69776	0.03611	0.31607
O	-2.01945	-1.15876	-0.27556
O	0.72998	1.93249	0.17752
O	-1.94385	1.61380	-0.39503
N	0.62486	-1.81909	0.35794
C	2.00535	-2.24153	0.12869
C	-0.35037	-2.85480	0.03522
C	2.85985	-1.12157	-0.45884
C	-1.78132	-2.41691	0.31695
C	2.96198	1.27354	-0.28679
C	-3.09232	-0.43971	0.28243
C	2.07379	2.31249	0.36639
C	-3.17151	0.91724	-0.39856
C	-0.21840	2.84526	0.67934
C	-1.53980	2.12950	0.85289
H	2.02209	-3.07966	-0.58706
H	2.46305	-2.61479	1.06773
H	-0.17416	-3.79066	0.61165
H	-0.24842	-3.10840	-1.03484
H	3.92051	-1.43797	-0.49924
H	2.54273	-0.92493	-1.50402
H	-2.48212	-3.17323	-0.08255

H -1.95511 -2.34972 1.40667

H 2.74823 1.23387 -1.37439

H 4.02269 1.56655 -0.16983

H -2.94055 -0.33212 1.37489

H -4.05296 -0.96859 0.13156

H 2.29777 2.37070 1.44894

H 2.27028 3.30397 -0.08384

H -3.43333 0.77838 -1.45896

H -3.96567 1.52044 0.07999

H -0.33082 3.69991 -0.01492

H 0.10258 3.23520 1.66317

H -1.41224 1.31804 1.59349

H -2.29547 2.83906 1.24028

K -0.08059 0.10344 -1.59867

C 0.44435 -1.28639 1.70178

H 1.25343 -0.57804 1.91625

H 0.44903 -2.09221 2.47006

H -0.50951 -0.73942 1.77306

ACE3 K M062X def2tzv

40

O 2.85238 0.05131 0.35247

O -2.04415 -1.20899 -0.27347

O 0.71684 1.94247 0.13013

O -2.02804 1.61498 -0.41209

N 0.66628 -1.79535 0.33143

C 2.06056 -2.22352 0.10412

C -0.30514 -2.87071 0.04780

C 2.91687 -1.11765 -0.49768

C -1.73668 -2.47052 0.37312

C 3.00344 1.33859 -0.28563

C	-3.13169	-0.47602	0.33585
C	2.08773	2.32765	0.39702
C	-3.26346	0.85734	-0.37658
C	-0.27547	2.86118	0.65346
C	-1.56761	2.10996	0.86973
H	2.07159	-3.05919	-0.59792
H	2.51167	-2.58062	1.03883
H	-0.08408	-3.77776	0.63207
H	-0.22747	-3.13209	-1.00883
H	3.95138	-1.45368	-0.60000
H	2.54344	-0.86768	-1.49469
H	-2.42308	-3.24263	0.02148
H	-1.87132	-2.36343	1.45165
H	2.74519	1.27120	-1.34618
H	4.03721	1.67941	-0.20704
H	-2.92716	-0.34512	1.40241
H	-4.07106	-1.02517	0.23557
H	2.26283	2.32334	1.47538
H	2.27899	3.33077	0.00761
H	-3.52570	0.69472	-1.41909
H	-4.05198	1.44470	0.10013
H	-0.41771	3.68306	-0.05063
H	0.05909	3.26647	1.61135
H	-1.39279	1.28068	1.55677
H	-2.31779	2.77842	1.29939
K	-0.14874	0.10493	-1.61288
C	0.49259	-1.22310	1.67737
H	1.27004	-0.48583	1.84170
H	0.54844	-2.00038	2.45467
H	-0.47054	-0.72278	1.75258

ACE3 K M06 def2sv

40

O	2.79767	0.16431	0.31950
O	-1.96665	-1.24591	-0.24346
O	0.62350	1.92188	0.18089
O	-2.08215	1.52364	-0.37402
N	0.75776	-1.76270	0.35654
C	2.14055	-2.12334	0.06680
C	-0.18379	-2.82305	0.03181
C	2.94542	-0.96354	-0.49519
C	-1.62056	-2.47153	0.35650
C	2.91226	1.42828	-0.26688
C	-3.07390	-0.60009	0.32761
C	1.93956	2.37151	0.39389
C	-3.25582	0.74540	-0.34266
C	-0.36666	2.78856	0.67878
C	-1.65573	2.03389	0.86447
H	2.16444	-2.93308	-0.68663
H	2.63893	-2.53435	0.97521
H	0.04964	-3.77178	0.57718
H	-0.09597	-3.05029	-1.05053
H	4.01270	-1.26223	-0.59135
H	2.59514	-0.73492	-1.52747
H	-2.28694	-3.28513	-0.00044
H	-1.76528	-2.40218	1.45557
H	2.70265	1.37680	-1.35884
H	3.94035	1.83649	-0.16125
H	-2.91854	-0.48850	1.42379
H	-4.00433	-1.19372	0.19583
H	2.14675	2.42092	1.48561

H	2.07881	3.39295	-0.02172
H	-3.54425	0.59711	-1.39936
H	-4.08033	1.29401	0.16033
H	-0.51235	3.64395	-0.01638
H	-0.06146	3.20222	1.66308
H	-1.49086	1.21634	1.59852
H	-2.42361	2.71624	1.28905
K	-0.18571	0.11846	-1.67650
C	0.61266	-1.28787	1.72112
H	1.43251	-0.59152	1.95239
H	0.63835	-2.12729	2.45889
H	-0.33523	-0.73458	1.85548

ACE3 K M06 def2tzv

40

O	2.87855	0.06911	0.33715
O	-2.04778	-1.20516	-0.27562
O	0.70736	1.94016	0.11278
O	-2.04615	1.61108	-0.37945
N	0.69212	-1.77598	0.34759
C	2.07526	-2.19809	0.06930
C	-0.28626	-2.83390	0.05009
C	2.92698	-1.08676	-0.51873
C	-1.71276	-2.44514	0.38344
C	2.99485	1.35614	-0.29236
C	-3.14973	-0.49497	0.31857
C	2.06920	2.32272	0.39673
C	-3.27377	0.85231	-0.36014
C	-0.28313	2.80512	0.71052
C	-1.55530	2.02727	0.91130
H	2.06398	-3.01187	-0.66088

H	2.54820	-2.59614	0.97991
H	-0.06875	-3.75704	0.61736
H	-0.21097	-3.08137	-1.01274
H	3.95930	-1.43389	-0.64016
H	2.55224	-0.82568	-1.51611
H	-2.39143	-3.24026	0.05899
H	-1.83701	-2.32627	1.46543
H	2.73069	1.28916	-1.35497
H	4.02324	1.72455	-0.22762
H	-2.97752	-0.38609	1.39648
H	-4.08907	-1.04359	0.18758
H	2.23881	2.29920	1.47948
H	2.26352	3.33852	0.03457
H	-3.53929	0.71852	-1.40841
H	-4.06930	1.42451	0.12993
H	-0.45325	3.67411	0.06701
H	0.06476	3.15774	1.68761
H	-1.34589	1.15684	1.54217
H	-2.29991	2.65388	1.41486
K	-0.19592	0.12936	-1.65971
C	0.56025	-1.25895	1.71474
H	1.35490	-0.54043	1.89671
H	0.62492	-2.06950	2.46087
H	-0.39392	-0.74691	1.84370

ACE3 K MP2 def2sv

40

O	2.70267	0.06013	0.33887
O	-2.01180	-1.18154	-0.27513
O	0.71278	1.94691	0.16633
O	-1.98622	1.61132	-0.37589

N	0.65407	-1.81512	0.35650
C	2.03675	-2.22409	0.10860
C	-0.31210	-2.85625	0.02254
C	2.87318	-1.08472	-0.46410
C	-1.74568	-2.44085	0.31830
C	2.96118	1.30887	-0.25967
C	-3.08352	-0.47790	0.32016
C	2.05578	2.33414	0.38804
C	-3.20186	0.88099	-0.34875
C	-0.24916	2.85120	0.67752
C	-1.55293	2.11249	0.87478
H	2.05567	-3.04727	-0.62825
H	2.50786	-2.61681	1.03514
H	-0.12662	-3.80270	0.58449
H	-0.21465	-3.09353	-1.05311
H	3.93968	-1.38960	-0.52113
H	2.54315	-0.87570	-1.50273
H	-2.43787	-3.21173	-0.07609
H	-1.91224	-2.38071	1.40969
H	2.76338	1.27442	-1.35062
H	4.01972	1.61329	-0.12697
H	-2.90699	-0.37517	1.40932
H	-4.04384	-1.01798	0.19010
H	2.26122	2.38227	1.47591
H	2.25970	3.33291	-0.04857
H	-3.48607	0.74488	-1.40500
H	-4.00039	1.46396	0.15258
H	-0.38680	3.70381	-0.01719
H	0.07522	3.25086	1.65847
H	-1.38850	1.29302	1.59822

H	-2.30883	2.80536	1.29752
K	-0.11112	0.12517	-1.67524
C	0.49000	-1.33926	1.72472
H	1.30744	-0.64614	1.95620
H	0.49948	-2.17539	2.46179
H	-0.45623	-0.78672	1.82816

ACE3 K SCS-MP2 def2sv

40

O	2.70569	0.06272	0.34114
O	-2.01790	-1.18844	-0.27831
O	0.71434	1.96571	0.15893
O	-1.99559	1.61532	-0.36962
N	0.65917	-1.82882	0.36075
C	2.04729	-2.23115	0.10325
C	-0.30932	-2.86800	0.00793
C	2.87932	-1.08198	-0.46760
C	-1.74929	-2.45690	0.30290
C	2.96902	1.31609	-0.25382
C	-3.08629	-0.48467	0.33061
C	2.06093	2.34517	0.39370
C	-3.21214	0.88063	-0.33344
C	-0.25155	2.87320	0.66667
C	-1.55675	2.13156	0.87692
H	2.06701	-3.05050	-0.63912
H	2.52457	-2.62694	1.02574
H	-0.12544	-3.82293	0.55671
H	-0.20761	-3.08942	-1.07159
H	3.94814	-1.38121	-0.52493
H	2.54755	-0.87107	-1.50575
H	-2.43784	-3.22425	-0.10653

H	-1.92215	-2.41122	1.39445
H	2.77653	1.28572	-1.34620
H	4.02833	1.61691	-0.11294
H	-2.90015	-0.38567	1.41896
H	-4.04774	-1.02537	0.20547
H	2.25789	2.38674	1.48404
H	2.27244	3.34553	-0.03736
H	-3.50543	0.74815	-1.38861
H	-4.00689	1.46160	0.17737
H	-0.39392	3.71850	-0.03698
H	0.07574	3.28335	1.64294
H	-1.38943	1.31899	1.60850
H	-2.31268	2.82875	1.29411
K	-0.11622	0.13161	-1.67903
C	0.49602	-1.37575	1.74178
H	1.31629	-0.68929	1.98783
H	0.50253	-2.22518	2.46445
H	-0.44936	-0.82156	1.85524

ACE3 K SCS-MP2 def2tzv

40

O	2.84586	-0.06420	0.41702
O	-2.12193	-1.13985	-0.28859
O	0.84238	2.05435	-0.00095
O	-2.00009	1.72107	-0.32639
N	0.60006	-1.86531	0.34760
C	1.99311	-2.33195	0.06811
C	-0.41183	-2.89044	-0.04241
C	2.87049	-1.21097	-0.50529
C	-1.84999	-2.46859	0.29106
C	3.13103	1.24401	-0.19408

C	-3.14106	-0.38048	0.45163
C	2.22789	2.28923	0.44851
C	-3.27794	0.99972	-0.19703
C	-0.14500	2.95264	0.63211
C	-1.40993	2.16402	0.94787
H	1.96264	-3.13360	-0.68122
H	2.45757	-2.74914	0.97699
H	-0.23224	-3.85271	0.47743
H	-0.31738	-3.06689	-1.11988
H	3.90209	-1.56286	-0.64141
H	2.47863	-0.90102	-1.48225
H	-2.55040	-3.20147	-0.12776
H	-2.00245	-2.42652	1.37555
H	2.95050	1.20819	-1.27568
H	4.18023	1.51145	-0.02018
H	-2.83875	-0.30391	1.50375
H	-4.11086	-0.89329	0.40342
H	2.27363	2.20699	1.54130
H	2.54911	3.29318	0.14234
H	-3.64322	0.90574	-1.22111
H	-3.98568	1.60268	0.38761
H	-0.36315	3.78559	-0.04442
H	0.26231	3.34465	1.57246
H	-1.15762	1.30140	1.57311
H	-2.12458	2.80764	1.47890
K	-0.23036	0.17979	-1.79894
C	0.45859	-1.48059	1.78226
H	1.29072	-0.82764	2.04115
H	0.46548	-2.36639	2.44485
H	-0.47216	-0.93151	1.93651

ACE3 Li B3LYP def2sv

40

O	-0.16794	-1.89378	-0.26308
O	-0.69437	1.72553	-0.59305
O	2.26240	-1.17520	0.67664
O	1.71347	1.56587	0.58261
N	-2.30821	-0.27890	0.51611
C	-2.48710	-1.69462	0.16996
C	-2.79858	0.62272	-0.53366
C	-1.43976	-2.18652	-0.82004
C	-2.09171	1.96900	-0.51160
C	0.95916	-2.42984	-0.94095
C	0.11457	2.88947	-0.54679
C	2.10923	-2.44046	0.05591
C	1.55888	2.44227	-0.52482
C	3.19900	-0.29217	0.08706
C	3.02722	1.05015	0.77416
H	-3.50207	-1.90608	-0.23035
H	-2.37407	-2.28223	1.09557
H	-3.89563	0.78480	-0.45909
H	-2.60858	0.15847	-1.51423
H	-1.55520	-3.27846	-0.96047
H	-1.54141	-1.71082	-1.81545
H	-2.42645	2.58070	-1.37148
H	-2.31594	2.53865	0.41143
H	1.19183	-1.81688	-1.83445
H	0.75587	-3.46392	-1.27857
H	-0.11870	3.47301	0.36579
H	-0.06970	3.53232	-1.42900
H	1.87885	-3.15807	0.86160

H	3.04355	-2.76409	-0.43950
H	1.81417	1.92914	-1.47261
H	2.21879	3.32457	-0.41964
H	3.02870	-0.19543	-1.00367
H	4.23457	-0.65956	0.23661
H	3.16019	0.91968	1.86124
H	3.78504	1.76888	0.41394
Li	-0.00071	0.07680	0.46386
C	-2.88036	0.03646	1.82572
H	-2.41163	-0.59327	2.60017
H	-3.98062	-0.12697	1.85713
H	-2.68376	1.08929	2.08606

ACE3 Li B3LYP def2tzv

40

O	0.08196	-1.71791	-0.35300
O	-0.94037	1.63139	-0.66309
O	2.56165	-1.07406	0.76475
O	1.44172	1.60558	0.57293
N	-2.27870	-0.49987	0.55839
C	-2.23926	-1.94581	0.21582
C	-2.93314	0.31970	-0.49695
C	-1.18729	-2.25943	-0.84025
C	-2.39126	1.74099	-0.51934
C	1.28596	-2.13035	-1.07551
C	-0.20851	2.89152	-0.58683
C	2.41805	-2.26290	-0.06768
C	1.25955	2.53497	-0.54868
C	3.31047	0.03994	0.18656
C	2.83854	1.29251	0.89622
H	-3.21584	-2.31437	-0.12876

H	-1.98585	-2.49085	1.12437
H	-4.02305	0.34806	-0.36397
H	-2.73731	-0.13523	-1.46623
H	-1.10307	-3.34132	-0.96224
H	-1.42216	-1.82021	-1.81241
H	-2.80910	2.29386	-1.36344
H	-2.61993	2.28418	0.40110
H	1.50306	-1.39405	-1.85205
H	1.12346	-3.09846	-1.55434
H	-0.50010	3.42757	0.31967
H	-0.42649	3.51353	-1.45771
H	2.19622	-3.06968	0.62730
H	3.35175	-2.49357	-0.58830
H	1.56909	2.05904	-1.48143
H	1.85787	3.43380	-0.38895
H	3.13829	0.11257	-0.88962
H	4.38078	-0.10612	0.35685
H	2.86918	1.13948	1.97285
H	3.47548	2.14147	0.64215
Li	-0.10797	0.12745	0.44823
C	-2.88623	-0.27176	1.89069
H	-2.32140	-0.81351	2.64756
H	-3.93350	-0.60319	1.92798
H	-2.85353	0.78742	2.13930

ACE3 Li B97-1 def2sv

40

O	-0.17245	-1.92344	-0.24202
O	-0.69373	1.73352	-0.59042
O	2.26302	-1.17530	0.67560
O	1.71836	1.57446	0.58791

N	-2.30293	-0.27699	0.51052
C	-2.49309	-1.69182	0.16088
C	-2.79480	0.62689	-0.53821
C	-1.43723	-2.18963	-0.82157
C	-2.08841	1.97678	-0.51036
C	0.95313	-2.44294	-0.92962
C	0.11306	2.89565	-0.53890
C	2.11230	-2.44222	0.06168
C	1.56016	2.44651	-0.51969
C	3.18579	-0.29323	0.06730
C	3.02954	1.05172	0.76054
H	-3.50755	-1.89297	-0.24970
H	-2.39240	-2.28338	1.08753
H	-3.89392	0.78629	-0.46460
H	-2.60009	0.16507	-1.52124
H	-1.56852	-3.27958	-0.97791
H	-1.51778	-1.69822	-1.81362
H	-2.42403	2.59074	-1.37061
H	-2.31646	2.54424	0.41551
H	1.17372	-1.82350	-1.82433
H	0.76066	-3.48049	-1.26944
H	-0.11878	3.47668	0.37787
H	-0.07010	3.54360	-1.41975
H	1.88980	-3.15874	0.87264
H	3.04618	-2.76390	-0.43951
H	1.81071	1.93023	-1.46930
H	2.22150	3.33054	-0.41966
H	2.99158	-0.19425	-1.02135
H	4.22579	-0.66139	0.19449
H	3.17707	0.91724	1.84686

H	3.78845	1.76730	0.39103
Li	-0.00418	0.07159	0.48078
C	-2.87500	0.03878	1.82139
H	-2.40977	-0.59809	2.59428
H	-3.97780	-0.11706	1.85076
H	-2.66947	1.09090	2.08490

ACE3 Li B97-1 def2tzv

40

O	0.03761	-1.74694	-0.32173
O	-0.89162	1.64087	-0.64363
O	2.53538	-1.10629	0.76455
O	1.50313	1.60545	0.59265
N	-2.30393	-0.45703	0.55039
C	-2.30306	-1.90046	0.19164
C	-2.92335	0.38653	-0.50801
C	-1.23601	-2.23038	-0.84950
C	-2.33738	1.79394	-0.51252
C	1.23042	-2.16978	-1.05220
C	-0.13822	2.88644	-0.58529
C	2.37126	-2.30324	-0.04847
C	1.32746	2.50495	-0.54969
C	3.30476	-0.02437	0.15863
C	2.89517	1.25041	0.87634
H	-3.28488	-2.23517	-0.17896
H	-2.08288	-2.46582	1.09970
H	-4.01587	0.44549	-0.38725
H	-2.72643	-0.06808	-1.48040
H	-1.18900	-3.31455	-0.99389
H	-1.43406	-1.76075	-1.81908
H	-2.73217	2.36729	-1.35759

H	-2.56048	2.33650	0.41317
H	1.44836	-1.43886	-1.83802
H	1.05902	-3.14351	-1.52291
H	-0.41498	3.44069	0.31833
H	-0.34873	3.50341	-1.46483
H	2.14204	-3.10055	0.65886
H	3.29939	-2.55371	-0.57512
H	1.61896	1.99980	-1.47636
H	1.94253	3.40080	-0.42030
H	3.10139	0.05074	-0.91508
H	4.37673	-0.20618	0.29727
H	2.95327	1.09983	1.95471
H	3.55540	2.07717	0.59762
Li	-0.10642	0.10748	0.49658
C	-2.93515	-0.22809	1.87251
H	-2.40073	-0.79632	2.63554
H	-3.99400	-0.53116	1.88172
H	-2.87764	0.82935	2.13506

ACE3 Li CAM-B3LYP def2sv

40

O	-0.05804	-1.73067	-0.38895
O	-0.78624	1.65917	-0.61257
O	2.31018	-1.12050	0.72803
O	1.58545	1.56704	0.56767
N	-2.26646	-0.37064	0.54661
C	-2.34400	-1.78587	0.19396
C	-2.83582	0.49739	-0.48234
C	-1.31726	-2.17152	-0.85572
C	-2.17930	1.86256	-0.49186
C	1.09317	-2.25857	-1.02060

C	-0.01129	2.83738	-0.59926
C	2.17910	-2.34106	0.03631
C	1.43633	2.42189	-0.54960
C	3.19994	-0.18304	0.16876
C	2.91129	1.14244	0.83481
H	-3.35668	-2.07808	-0.15330
H	-2.12985	-2.36830	1.10334
H	-3.93209	0.61303	-0.36064
H	-2.66875	0.03732	-1.46715
H	-1.31731	-3.26946	-0.97552
H	-1.53525	-1.72744	-1.84448
H	-2.55766	2.45729	-1.34333
H	-2.39155	2.42876	0.43453
H	1.38671	-1.61112	-1.86734
H	0.88993	-3.26847	-1.41702
H	-0.26345	3.44083	0.29297
H	-0.20388	3.44721	-1.50082
H	1.89100	-3.09471	0.78586
H	3.13715	-2.65250	-0.41810
H	1.71749	1.89938	-1.48295
H	2.07729	3.31540	-0.44429
H	3.05861	-0.10037	-0.92538
H	4.25075	-0.47925	0.35287
H	2.99549	1.02459	1.92646
H	3.63543	1.90842	0.50938
Li	-0.02579	0.08757	0.46132
C	-2.83447	-0.10494	1.86160
H	-2.30641	-0.69730	2.62531
H	-3.91632	-0.35217	1.90902
H	-2.71502	0.95848	2.12044

ACE3 Li CAM-B3LYP def2tzv

40

O	0.11079	-1.65142	-0.39127
O	-0.96486	1.60046	-0.67622
O	2.56097	-1.05443	0.76455
O	1.38375	1.57843	0.55820
N	-2.24259	-0.52381	0.56744
C	-2.16916	-1.96282	0.24274
C	-2.91895	0.26389	-0.48512
C	-1.13595	-2.25442	-0.82847
C	-2.40538	1.68797	-0.52511
C	1.31344	-2.03864	-1.10719
C	-0.25011	2.85775	-0.60056
C	2.42923	-2.20973	-0.09717
C	1.21385	2.51152	-0.54778
C	3.29162	0.07815	0.22887
C	2.76363	1.30327	0.93259
H	-3.14162	-2.36017	-0.07495
H	-1.88050	-2.48949	1.15019
H	-4.00600	0.26948	-0.34151
H	-2.72212	-0.19554	-1.45066
H	-1.00813	-3.33276	-0.92964
H	-1.41453	-1.84898	-1.80200
H	-2.83805	2.22709	-1.36864
H	-2.63571	2.23322	0.39228
H	1.54198	-1.27359	-1.84931
H	1.15430	-2.98420	-1.62673
H	-0.55156	3.39212	0.30209
H	-0.46684	3.47528	-1.47292
H	2.19796	-3.03839	0.56610

H	3.36797	-2.42376	-0.61290
H	1.53720	2.04630	-1.47965
H	1.80518	3.41008	-0.37174
H	3.15130	0.16037	-0.85017
H	4.35792	-0.04042	0.43040
H	2.75949	1.13516	2.00595
H	3.38424	2.17220	0.71382
Li	-0.12471	0.13715	0.43277
C	-2.84321	-0.29671	1.89330
H	-2.25954	-0.80992	2.65385
H	-3.87778	-0.65944	1.93930
H	-2.84223	0.76492	2.12772

ACE3 Li MP2 def2tzv

40

O	0.06056	-1.69848	-0.33458
O	-0.91670	1.59943	-0.66059
O	2.55031	-1.11564	0.80243
O	1.45969	1.56719	0.58728
N	-2.29782	-0.47891	0.56573
C	-2.29159	-1.90090	0.10754
C	-2.97880	0.40735	-0.42866
C	-1.21048	-2.13426	-0.94493
C	-2.35801	1.80001	-0.42496
C	1.27052	-2.04555	-1.10350
C	-0.13451	2.84585	-0.65732
C	2.39478	-2.26953	-0.09963
C	1.32112	2.42355	-0.61108
C	3.34287	-0.00222	0.24879
C	2.85789	1.25583	0.95175
H	-3.27185	-2.20014	-0.30176

H	-2.07704	-2.53190	0.97507
H	-4.05816	0.48244	-0.21574
H	-2.86243	-0.02048	-1.42814
H	-1.14768	-3.20026	-1.19111
H	-1.38479	-1.56431	-1.86411
H	-2.78245	2.41870	-1.22337
H	-2.49264	2.30794	0.53750
H	1.49506	-1.23382	-1.80378
H	1.09817	-2.96856	-1.66922
H	-0.39241	3.43535	0.22981
H	-0.34098	3.42528	-1.56335
H	2.15430	-3.11030	0.55177
H	3.33385	-2.47206	-0.62988
H	1.59286	1.86303	-1.51164
H	1.96738	3.30186	-0.51061
H	3.19030	0.07560	-0.83382
H	4.40724	-0.16911	0.45038
H	2.85495	1.10782	2.03197
H	3.49409	2.11073	0.69742
Li	-0.11252	0.10895	0.56837
C	-2.93448	-0.35942	1.91141
H	-2.34229	-0.91837	2.63902
H	-3.96527	-0.74912	1.90618
H	-2.95472	0.69100	2.20938

ACE3 Li PBE0 def2sv

40

O	-0.19347	-1.89341	-0.24706
O	-0.66537	1.71276	-0.59314
O	2.22498	-1.18212	0.66490
O	1.72239	1.55020	0.59362

N	-2.28950	-0.25183	0.51272
C	-2.49210	-1.65764	0.18177
C	-2.76489	0.63843	-0.53952
C	-1.45832	-2.16675	-0.80183
C	-2.04925	1.97123	-0.52241
C	0.91719	-2.42326	-0.93430
C	0.14552	2.86239	-0.55072
C	2.06864	-2.43844	0.04927
C	1.57907	2.40584	-0.51755
C	3.16405	-0.31721	0.07681
C	3.01916	1.01700	0.77048
H	-3.51193	-1.85705	-0.21129
H	-2.38343	-2.24023	1.11125
H	-3.86126	0.80748	-0.47444
H	-2.57067	0.16557	-1.51556
H	-1.59187	-3.25670	-0.94116
H	-1.55354	-1.69191	-1.79855
H	-2.37444	2.58080	-1.38723
H	-2.27485	2.54922	0.39544
H	1.14408	-1.80648	-1.82675
H	0.71139	-3.45530	-1.27622
H	-0.08651	3.45454	0.35660
H	-0.02763	3.50211	-1.43710
H	1.84165	-3.15823	0.85362
H	2.99935	-2.76377	-0.45102
H	1.83042	1.87896	-1.45894
H	2.24613	3.28409	-0.42535
H	2.98699	-0.21089	-1.01222
H	4.19509	-0.70030	0.21447
H	3.15999	0.87557	1.85492

H	3.78677	1.72590	0.41141
Li	-0.00367	0.06382	0.48639
C	-2.85337	0.08450	1.80969
H	-2.39701	-0.54525	2.59099
H	-3.95582	-0.05834	1.84140
H	-2.63779	1.13569	2.06069

ACE3 Li PBE0 def2tzv

40

O	0.04886	-1.72329	-0.32517
O	-0.89523	1.62394	-0.64424
O	2.52532	-1.09186	0.75729
O	1.47368	1.59245	0.58682
N	-2.27934	-0.46341	0.54850
C	-2.26654	-1.89887	0.21032
C	-2.89699	0.35699	-0.51149
C	-1.21298	-2.22856	-0.82785
C	-2.33196	1.76068	-0.52428
C	1.23292	-2.13838	-1.05176
C	-0.15789	2.86557	-0.57914
C	2.36642	-2.27496	-0.05722
C	1.30003	2.49711	-0.53724
C	3.27839	-0.00862	0.16089
C	2.85683	1.25124	0.87477
H	-3.24744	-2.24893	-0.14552
H	-2.03377	-2.45076	1.12254
H	-3.99019	0.40217	-0.39985
H	-2.68923	-0.10230	-1.47849
H	-1.15468	-3.31332	-0.95678
H	-1.42850	-1.77999	-1.80273
H	-2.73026	2.32311	-1.37383

H	-2.56845	2.30780	0.39449
H	1.45093	-1.40566	-1.83454
H	1.06418	-3.10836	-1.52891
H	-0.44248	3.41505	0.32400
H	-0.36767	3.48596	-1.45526
H	2.14090	-3.07933	0.64198
H	3.29387	-2.52069	-0.58549
H	1.60156	2.00694	-1.46771
H	1.90683	3.39565	-0.39576
H	3.07781	0.06845	-0.91244
H	4.35170	-0.17694	0.29864
H	2.91502	1.09774	1.95194
H	3.51121	2.08376	0.60324
Li	-0.10687	0.11631	0.48984
C	-2.90500	-0.22288	1.85724
H	-2.36765	-0.77428	2.62908
H	-3.96032	-0.53409	1.87257
H	-2.85777	0.83713	2.10671

ACE3 Li PBE def2sv

40

O	-0.17295	-1.93498	-0.22611
O	-0.69078	1.73425	-0.59383
O	2.27364	-1.18098	0.68689
O	1.72370	1.58419	0.60025
N	-2.30871	-0.27736	0.50741
C	-2.49896	-1.69261	0.15220
C	-2.79494	0.62936	-0.54362
C	-1.43928	-2.18942	-0.82457
C	-2.09200	1.97936	-0.50859
C	0.95681	-2.45240	-0.92325

C	0.11460	2.90538	-0.52917
C	2.11653	-2.45230	0.06607
C	1.56129	2.45975	-0.51374
C	3.18136	-0.29253	0.04818
C	3.04222	1.05022	0.74695
H	-3.51843	-1.89590	-0.26566
H	-2.40386	-2.28875	1.08479
H	-3.90270	0.78758	-0.48003
H	-2.59076	0.16538	-1.53171
H	-1.57797	-3.28404	-0.99486
H	-1.50794	-1.68627	-1.82062
H	-2.43330	2.60439	-1.36834
H	-2.31979	2.54507	0.42791
H	1.17471	-1.82744	-1.82431
H	0.76488	-3.49642	-1.26844
H	-0.12265	3.48097	0.39913
H	-0.07040	3.56784	-1.40847
H	1.89153	-3.17076	0.88442
H	3.05503	-2.78074	-0.43856
H	1.81262	1.94151	-1.47104
H	2.22594	3.35122	-0.41636
H	2.95656	-0.19146	-1.04221
H	4.23294	-0.66114	0.14436
H	3.20671	0.91027	1.83762
H	3.80413	1.76816	0.36679
Li	-0.00363	0.07174	0.48917
C	-2.89065	0.03731	1.81484
H	-2.43016	-0.60462	2.59581
H	-4.00158	-0.11623	1.83777
H	-2.68435	1.09507	2.08322

ACE3 Li PBE def2tzv

40

O	-0.00192	-1.86125	-0.23156
O	-0.84967	1.67876	-0.62088
O	2.51797	-1.12001	0.75750
O	1.57634	1.65055	0.62587
N	-2.31832	-0.42124	0.52091
C	-2.37151	-1.86754	0.16228
C	-2.89785	0.44013	-0.54924
C	-1.28596	-2.25002	-0.83613
C	-2.30694	1.84259	-0.52140
C	1.18611	-2.30020	-0.97969
C	-0.09559	2.93337	-0.52415
C	2.34544	-2.36736	0.00379
C	1.36839	2.55787	-0.51890
C	3.26427	-0.06990	0.04841
C	2.98112	1.22564	0.78356
H	-3.36104	-2.16039	-0.24386
H	-2.21125	-2.44319	1.08469
H	-4.00174	0.50415	-0.46623
H	-2.66844	-0.01152	-1.52398
H	-1.30412	-3.33904	-1.00603
H	-1.40689	-1.74517	-1.80991
H	-2.68072	2.43234	-1.37433
H	-2.55081	2.38082	0.41041
H	1.37552	-1.60091	-1.81152
H	1.01805	-3.30508	-1.40180
H	-0.37019	3.46008	0.40553
H	-0.31737	3.58423	-1.38591
H	2.13888	-3.13022	0.76508

H	3.27167	-2.64028	-0.53065
H	1.64365	2.05420	-1.46032
H	1.98707	3.46250	-0.40193
H	2.94736	-0.00063	-1.00575
H	4.34538	-0.29285	0.07451
H	3.13065	1.08085	1.86139
H	3.65681	2.02246	0.43485
Li	-0.11238	0.09558	0.51149
C	-2.96269	-0.16756	1.83443
H	-2.46206	-0.76182	2.60973
H	-4.04068	-0.42726	1.82725
H	-2.86490	0.89212	2.10278

ACE3 Li B2PLYP def2sv

40

O	-0.17823	-1.87231	-0.26541
O	-0.67752	1.70379	-0.60862
O	2.23461	-1.17820	0.67577
O	1.70517	1.54984	0.59498
N	-2.29042	-0.26350	0.52409
C	-2.48504	-1.67634	0.18500
C	-2.78540	0.62890	-0.52838
C	-1.45191	-2.16761	-0.81495
C	-2.06987	1.96635	-0.51661
C	0.94206	-2.39999	-0.96041
C	0.13660	2.86353	-0.57234
C	2.08660	-2.43499	0.03616
C	1.57258	2.40340	-0.53253
C	3.18782	-0.30197	0.10405
C	3.01525	1.03149	0.80026
H	-3.50570	-1.87942	-0.20143

H	-2.36112	-2.26313	1.10832
H	-3.88012	0.79410	-0.44638
H	-2.60160	0.15928	-1.50601
H	-1.56528	-3.25836	-0.95413
H	-1.56174	-1.69019	-1.80652
H	-2.40298	2.57805	-1.37522
H	-2.27894	2.53892	0.40612
H	1.17712	-1.76734	-1.83720
H	0.73127	-3.42352	-1.31942
H	-0.09696	3.45812	0.33068
H	-0.03756	3.49431	-1.46333
H	1.85085	-3.16438	0.82701
H	3.02301	-2.74753	-0.45885
H	1.82620	1.86671	-1.46545
H	2.24251	3.27745	-0.43970
H	3.02999	-0.19501	-0.98589
H	4.21580	-0.68068	0.26395
H	3.13231	0.89063	1.88587
H	3.77632	1.75242	0.45560
Li	0.00636	0.06659	0.47960
C	-2.86958	0.05986	1.82727
H	-2.40492	-0.56551	2.60543
H	-3.96804	-0.10393	1.85035
H	-2.67355	1.11301	2.07984

ACE3 Li B2PLYP def2tzv

40

O	0.07975	-1.69925	-0.35527
O	-0.93515	1.61814	-0.66632
O	2.55371	-1.08337	0.77477
O	1.43375	1.59061	0.57576

N	-2.26895	-0.49589	0.56306
C	-2.24124	-1.93885	0.20022
C	-2.93953	0.32988	-0.47997
C	-1.18855	-2.23498	-0.86056
C	-2.38557	1.74664	-0.50244
C	1.28486	-2.09485	-1.09142
C	-0.19581	2.87849	-0.60719
C	2.41073	-2.25991	-0.08193
C	1.26934	2.50830	-0.56239
C	3.31215	0.03385	0.20680
C	2.83048	1.28276	0.91762
H	-3.22063	-2.29030	-0.15385
H	-1.99178	-2.49978	1.10023
H	-4.02584	0.36443	-0.32168
H	-2.76354	-0.12241	-1.45445
H	-1.09906	-3.31393	-1.00117
H	-1.41891	-1.77520	-1.82355
H	-2.80556	2.30941	-1.33831
H	-2.59237	2.28484	0.42563
H	1.50710	-1.33339	-1.84080
H	1.11664	-3.04684	-1.59917
H	-0.48361	3.42576	0.29314
H	-0.41037	3.48688	-1.48775
H	2.18066	-3.07769	0.59630
H	3.34730	-2.47847	-0.60199
H	1.57552	2.01493	-1.48653
H	1.87730	3.40110	-0.40812
H	3.14449	0.11045	-0.86964
H	4.37966	-0.11565	0.38644
H	2.84695	1.12701	1.99340

H	3.46281	2.13639	0.66828
Li	-0.11005	0.12721	0.46677
C	-2.88454	-0.28918	1.89849
H	-2.31039	-0.82896	2.64927
H	-3.92521	-0.63992	1.92563
H	-2.86889	0.76902	2.15187

ACE3 Li DSDPBEP86 def2sv

40

O	-0.17909	-1.85941	-0.26700
O	-0.67585	1.68042	-0.62535
O	2.21698	-1.17600	0.67869
O	1.68370	1.54413	0.60641
N	-2.26778	-0.25993	0.53225
C	-2.47299	-1.67012	0.19971
C	-2.78110	0.62320	-0.51578
C	-1.45133	-2.15476	-0.81291
C	-2.06171	1.95629	-0.51814
C	0.93741	-2.35779	-0.98438
C	0.14044	2.83580	-0.60566
C	2.07895	-2.41796	0.01188
C	1.57068	2.36536	-0.54443
C	3.17338	-0.29615	0.12297
C	2.98942	1.02904	0.82891
H	-3.50201	-1.87011	-0.17155
H	-2.33385	-2.25903	1.12224
H	-3.87671	0.78670	-0.41694
H	-2.60971	0.14899	-1.49584
H	-1.56255	-3.24678	-0.95972
H	-1.56855	-1.66744	-1.80095
H	-2.40107	2.56837	-1.37665

H	-2.25579	2.53372	0.40734
H	1.16903	-1.69148	-1.83954
H	0.72953	-3.37107	-1.37852
H	-0.09493	3.44839	0.28715
H	-0.02471	3.45174	-1.51111
H	1.84026	-3.16832	0.78458
H	3.02121	-2.71484	-0.48613
H	1.82366	1.79833	-1.46182
H	2.25110	3.23560	-0.47079
H	3.02367	-0.18080	-0.96940
H	4.20204	-0.67579	0.28909
H	3.08840	0.87667	1.91654
H	3.75519	1.75748	0.50366
Li	0.00477	0.05869	0.49005
C	-2.83268	0.06924	1.83798
H	-2.35620	-0.55227	2.61492
H	-3.93222	-0.09687	1.87290
H	-2.63421	1.12612	2.08173

ACE3 Li DSDPBEP86 def2tzv

40

O	0.06946	-1.67820	-0.35301
O	-0.91955	1.59523	-0.68288
O	2.53662	-1.10032	0.77824
O	1.42498	1.57337	0.59326
N	-2.25946	-0.48177	0.57050
C	-2.24412	-1.92091	0.20005
C	-2.93618	0.34378	-0.46621
C	-1.19456	-2.20649	-0.86694
C	-2.36189	1.75185	-0.49691
C	1.26614	-2.04121	-1.11331

C	-0.16783	2.84612	-0.64312
C	2.39359	-2.24851	-0.11330
C	1.29140	2.45448	-0.57393
C	3.30223	0.02053	0.23280
C	2.81238	1.26095	0.95454
H	-3.23020	-2.26285	-0.15485
H	-1.99213	-2.49223	1.09712
H	-4.02271	0.38978	-0.29135
H	-2.77604	-0.11377	-1.44486
H	-1.10472	-3.28679	-1.02269
H	-1.42593	-1.73105	-1.82596
H	-2.78601	2.32587	-1.32732
H	-2.54446	2.29173	0.43944
H	1.48697	-1.24356	-1.82998
H	1.09376	-2.97216	-1.66396
H	-0.45598	3.41588	0.24721
H	-0.36715	3.44051	-1.54049
H	2.15931	-3.09083	0.53815
H	3.33274	-2.45051	-0.64222
H	1.59610	1.92489	-1.48247
H	1.91588	3.34333	-0.44100
H	3.14218	0.11093	-0.84736
H	4.37090	-0.13345	0.41994
H	2.80981	1.08829	2.03100
H	3.45059	2.12048	0.72608
Li	-0.11753	0.12410	0.49561
C	-2.87679	-0.28035	1.90439
H	-2.29557	-0.81656	2.65704
H	-3.91717	-0.64176	1.92928
H	-2.86968	0.78191	2.15583

ACE3 Li HSE06 def2sv

40

O	-0.19523	-1.88813	-0.25011
O	-0.66242	1.71094	-0.59542
O	2.22255	-1.18432	0.66466
O	1.72321	1.54789	0.59508
N	-2.29032	-0.24937	0.51446
C	-2.49320	-1.65596	0.18700
C	-2.76345	0.63864	-0.54079
C	-1.46235	-2.16612	-0.79914
C	-2.04680	1.97083	-0.52525
C	0.91479	-2.42065	-0.93792
C	0.14968	2.86058	-0.55385
C	2.06537	-2.44009	0.04651
C	1.58265	2.40267	-0.51806
C	3.16517	-0.31983	0.08011
C	3.01986	1.01355	0.77509
H	-3.51419	-1.85670	-0.20257
H	-2.38132	-2.23651	1.11740
H	-3.85986	0.80847	-0.47789
H	-2.56782	0.16343	-1.51545
H	-1.59370	-3.25673	-0.93455
H	-1.56249	-1.69467	-1.79697
H	-2.37093	2.58008	-1.39060
H	-2.27172	2.54983	0.39215
H	1.14378	-1.80315	-1.82922
H	0.70575	-3.45141	-1.28136
H	-0.08327	3.45405	0.35232
H	-0.02221	3.49901	-1.44128
H	1.83673	-3.16098	0.84930

H	2.99628	-2.76521	-0.45338
H	1.83497	1.87390	-1.45808
H	2.25094	3.27986	-0.42583
H	2.99111	-0.21204	-1.00926
H	4.19506	-0.70494	0.22006
H	3.15742	0.87082	1.85969
H	3.78853	1.72262	0.41878
Li	-0.00023	0.06389	0.48668
C	-2.85586	0.09104	1.80970
H	-2.40094	-0.53631	2.59370
H	-3.95842	-0.05157	1.84011
H	-2.64073	1.14306	2.05758

ACE3 Li HSE06 def2tzv

40

O	0.05125	-1.72141	-0.32695
O	-0.89766	1.62301	-0.64558
O	2.52621	-1.09076	0.75738
O	1.46994	1.59196	0.58611
N	-2.27871	-0.46572	0.54941
C	-2.26318	-1.90184	0.21361
C	-2.89767	0.35254	-0.51167
C	-1.21165	-2.23048	-0.82659
C	-2.33576	1.75725	-0.52543
C	1.23649	-2.13512	-1.05473
C	-0.16091	2.86635	-0.58043
C	2.36901	-2.27361	-0.05992
C	1.29682	2.49859	-0.53822
C	3.27968	-0.00483	0.16362
C	2.85418	1.25301	0.87791
H	-3.24395	-2.25469	-0.13913

H	-2.02703	-2.45154	1.12593
H	-3.99079	0.39534	-0.40057
H	-2.68864	-0.10715	-1.47792
H	-1.15060	-3.31479	-0.95476
H	-1.42994	-1.78306	-1.80101
H	-2.73456	2.31854	-1.37499
H	-2.57255	2.30431	0.39290
H	1.45432	-1.40025	-1.83503
H	1.06745	-3.10336	-1.53448
H	-0.44641	3.41508	0.32244
H	-0.37154	3.48607	-1.45635
H	2.14364	-3.07892	0.63765
H	3.29718	-2.51705	-0.58725
H	1.59930	2.00894	-1.46822
H	1.90358	3.39637	-0.39500
H	3.08140	0.07265	-0.90978
H	4.35245	-0.17215	0.30374
H	2.90924	1.09867	1.95472
H	3.50680	2.08734	0.60918
Li	-0.10452	0.11835	0.48712
C	-2.90281	-0.22313	1.85860
H	-2.36417	-0.77230	2.63067
H	-3.95762	-0.53512	1.87603
H	-2.85621	0.83715	2.10577

ACE3 Li M062X def2sv

40

O	-0.13351	-1.73818	-0.36040
O	-0.70386	1.64501	-0.60443
O	2.23305	-1.16652	0.72199
O	1.65658	1.54230	0.60115

N	-2.27630	-0.29573	0.54800
C	-2.41965	-1.70406	0.18791
C	-2.80558	0.59204	-0.48598
C	-1.39944	-2.11684	-0.85833
C	-2.08405	1.92321	-0.48395
C	0.99865	-2.26137	-1.02575
C	0.11314	2.79387	-0.64725
C	2.08658	-2.38336	0.02504
C	1.54347	2.32481	-0.57016
C	3.17787	-0.27633	0.17674
C	2.96013	1.05532	0.85959
H	-3.44357	-1.94365	-0.16624
H	-2.23176	-2.30198	1.09403
H	-3.89671	0.75163	-0.36834
H	-2.64738	0.12690	-1.47089
H	-1.44193	-3.21165	-0.99866
H	-1.58100	-1.64291	-1.84091
H	-2.42563	2.54347	-1.33225
H	-2.26656	2.48958	0.44918
H	1.29368	-1.58934	-1.85334
H	0.77571	-3.25633	-1.44914
H	-0.11705	3.44707	0.21531
H	-0.05816	3.36213	-1.57901
H	1.78145	-3.13885	0.76572
H	3.04080	-2.70253	-0.43061
H	1.79606	1.72830	-1.46731
H	2.22329	3.19426	-0.52441
H	3.04183	-0.17502	-0.91756
H	4.20800	-0.63620	0.35993
H	3.04010	0.92012	1.94914

H 3.71823 1.78986 0.53732

Li -0.03296 0.07168 0.51728

C -2.86388 -0.00925 1.85103

H -2.36638 -0.61343 2.62555

H -3.95209 -0.22822 1.87122

H -2.71949 1.05277 2.10351

ACE3 Li M062X def2tzv

40

O 0.09636 -1.59510 -0.39855

O -0.93881 1.56426 -0.68401

O 2.54184 -1.09465 0.77714

O 1.37540 1.52888 0.57674

N -2.24758 -0.50556 0.58023

C -2.17932 -1.93727 0.21701

C -2.93082 0.30239 -0.45557

C -1.14424 -2.18355 -0.86712

C -2.36924 1.70999 -0.49346

C 1.29308 -1.91725 -1.15272

C -0.18787 2.80142 -0.66868

C 2.40196 -2.19049 -0.15583

C 1.26501 2.40465 -0.58030

C 3.29713 0.04696 0.30178

C 2.73471 1.25617 1.01173

H -3.15390 -2.32051 -0.11095

H -1.88343 -2.49082 1.10729

H -4.01271 0.33492 -0.28163

H -2.76777 -0.15467 -1.42986

H -1.01046 -3.25638 -1.01466

H -1.42124 -1.73317 -1.82187

H -2.80098 2.27847 -1.31832

H	-2.55106	2.24737	0.44051
H	1.52932	-1.07837	-1.80926
H	1.12643	-2.80350	-1.76689
H	-0.47444	3.38812	0.20648
H	-0.38164	3.37807	-1.57363
H	2.14494	-3.05898	0.44513
H	3.34197	-2.38259	-0.67786
H	1.57863	1.87415	-1.48088
H	1.89136	3.28646	-0.44190
H	3.20002	0.15632	-0.78125
H	4.35333	-0.08125	0.54440
H	2.67850	1.05937	2.07926
H	3.35834	2.13461	0.84133
Li	-0.14810	0.14177	0.49618
C	-2.86542	-0.31217	1.90610
H	-2.28164	-0.83362	2.66156
H	-3.89538	-0.68937	1.92857
H	-2.87906	0.74687	2.15619

ACE3 Li M06 def2sv

40

O	-0.31901	-1.89820	-0.20709
O	-0.53489	1.68303	-0.57879
O	2.14282	-1.24999	0.66305
O	1.87205	1.49997	0.63225
N	-2.36615	-0.13783	0.51935
C	-2.61723	-1.51934	0.12892
C	-2.72314	0.80296	-0.53645
C	-1.57376	-2.04295	-0.82862
C	-1.89091	2.05787	-0.48578
C	0.77790	-2.39883	-0.93510

C	0.34786	2.77870	-0.60712
C	1.93070	-2.49246	0.03175
C	1.74804	2.24496	-0.55583
C	3.17369	-0.46298	0.11846
C	3.12294	0.87416	0.81173
H	-3.63302	-1.65034	-0.31254
H	-2.58102	-2.14248	1.04212
H	-3.80576	1.06507	-0.49978
H	-2.54799	0.32664	-1.51830
H	-1.77208	-3.11249	-1.04953
H	-1.58068	-1.50359	-1.80123
H	-2.16215	2.72630	-1.32949
H	-2.05504	2.63150	0.45254
H	1.01190	-1.72043	-1.78633
H	0.54801	-3.40105	-1.35241
H	0.15846	3.43732	0.26799
H	0.19814	3.38056	-1.52726
H	1.68151	-3.22382	0.82276
H	2.84686	-2.84383	-0.48201
H	1.93957	1.60952	-1.44776
H	2.47541	3.08295	-0.57253
H	3.04536	-0.34682	-0.98046
H	4.16393	-0.93790	0.28515
H	3.24812	0.72366	1.89928
H	3.94495	1.52828	0.45795
Li	0.04633	0.00853	0.63923
C	-3.02674	0.18960	1.76915
H	-2.65933	-0.46641	2.57816
H	-4.13411	0.07494	1.69907
H	-2.81082	1.23294	2.05950

ACE3 Li M06 def2tzv

40

O	0.02198	-1.73406	-0.28375
O	-0.86058	1.59240	-0.65071
O	2.51462	-1.12943	0.76635
O	1.50689	1.52988	0.59485
N	-2.33305	-0.43293	0.55278
C	-2.31674	-1.84374	0.12618
C	-2.92299	0.45253	-0.47087
C	-1.23424	-2.10455	-0.89555
C	-2.27386	1.81452	-0.45135
C	1.19695	-2.02153	-1.07511
C	-0.06150	2.79179	-0.64473
C	2.33910	-2.25408	-0.11665
C	1.37144	2.34684	-0.59308
C	3.32132	-0.05337	0.24296
C	2.86907	1.19703	0.94886
H	-3.29010	-2.15838	-0.27903
H	-2.11329	-2.45673	1.00648
H	-4.00858	0.55110	-0.33114
H	-2.76321	0.01565	-1.45773
H	-1.21835	-3.16582	-1.16099
H	-1.38022	-1.52615	-1.81491
H	-2.67403	2.44696	-1.24954
H	-2.42690	2.32714	0.50585
H	1.39268	-1.18205	-1.75286
H	1.03854	-2.92058	-1.67809
H	-0.30546	3.39174	0.23896
H	-0.25170	3.38789	-1.54168
H	2.10913	-3.10673	0.52105

H	3.25856	-2.46894	-0.67065
H	1.62558	1.76557	-1.48580
H	2.03540	3.21353	-0.53383
H	3.18877	0.04514	-0.84057
H	4.38102	-0.24367	0.43944
H	2.87235	1.02986	2.02534
H	3.53227	2.03571	0.72062
Li	-0.11487	0.10121	0.65029
C	-3.00138	-0.26836	1.85115
H	-2.48280	-0.85309	2.61068
H	-4.05192	-0.59318	1.81061
H	-2.97803	0.77902	2.15272

ACE3 Li MP2 def2sv

40

O	-0.19907	-1.88255	-0.24395
O	-0.65562	1.68871	-0.61794
O	2.20431	-1.18370	0.67719
O	1.70974	1.53902	0.60882
N	-2.27493	-0.24301	0.52536
C	-2.49753	-1.64752	0.17882
C	-2.77106	0.65122	-0.52175
C	-1.46844	-2.14080	-0.82086
C	-2.04104	1.97784	-0.50807
C	0.91550	-2.38395	-0.96676
C	0.16722	2.84252	-0.59087
C	2.06019	-2.43630	0.02469
C	1.59443	2.36328	-0.54233
C	3.17054	-0.32012	0.10611
C	3.01519	1.00795	0.81218
H	-3.52440	-1.82786	-0.20885

H	-2.38070	-2.24693	1.09752
H	-3.86671	0.82236	-0.43257
H	-2.59331	0.18511	-1.50425
H	-1.60192	-3.22788	-0.98581
H	-1.55506	-1.63681	-1.80332
H	-2.37399	2.60140	-1.36095
H	-2.23136	2.54678	0.42295
H	1.14387	-1.72197	-1.82590
H	0.70757	-3.39995	-1.35434
H	-0.05839	3.44755	0.30914
H	0.00077	3.46802	-1.48954
H	1.82247	-3.17648	0.80695
H	2.99984	-2.74100	-0.47320
H	1.83502	1.79696	-1.46289
H	2.28076	3.22923	-0.47249
H	3.00919	-0.20355	-0.98387
H	4.19455	-0.71603	0.26155
H	3.12916	0.85599	1.89804
H	3.78453	1.72638	0.47353
Li	0.01953	0.05355	0.50883
C	-2.86214	0.08009	1.82417
H	-2.40342	-0.55061	2.60350
H	-3.96239	-0.08046	1.83636
H	-2.66212	1.13311	2.07946

ACE3 Li SCS-MP2 def2sv

40

O	-0.17532	-1.87327	-0.25851
O	-0.68171	1.68253	-0.62924
O	2.22365	-1.17235	0.68265
O	1.68156	1.54654	0.60371

N	-2.27109	-0.26485	0.53203
C	-2.47748	-1.67403	0.18048
C	-2.79323	0.63035	-0.50659
C	-1.44585	-2.15042	-0.82896
C	-2.06959	1.96357	-0.50317
C	0.94631	-2.35951	-0.98475
C	0.13722	2.84169	-0.60848
C	2.09100	-2.41745	0.01053
C	1.56880	2.36801	-0.55249
C	3.18582	-0.29037	0.12906
C	2.99331	1.03703	0.83257
H	-3.50434	-1.86665	-0.20176
H	-2.34672	-2.27586	1.09636
H	-3.88811	0.79487	-0.39703
H	-2.63114	0.16652	-1.49340
H	-1.56324	-3.23971	-0.99430
H	-1.54564	-1.64586	-1.81016
H	-2.41590	2.58451	-1.35320
H	-2.25188	2.53193	0.43011
H	1.17201	-1.68503	-1.83528
H	0.74537	-3.37236	-1.38503
H	-0.09304	3.45163	0.28739
H	-0.03090	3.45983	-1.51244
H	1.85712	-3.17122	0.78184
H	3.03467	-2.70786	-0.48946
H	1.81703	1.79905	-1.46984
H	2.25120	3.23735	-0.48088
H	3.04330	-0.17769	-0.96424
H	4.21399	-0.66825	0.30478
H	3.08794	0.88747	1.92125

H	3.75684	1.76924	0.50916
Li	0.00745	0.05864	0.49462
C	-2.84531	0.04378	1.84374
H	-2.36295	-0.58161	2.61428
H	-3.94273	-0.13579	1.87334
H	-2.65913	1.10003	2.09997

ACE3 Li SCS-MP2 def2tzv

40

O	0.05896	-1.70664	-0.33701
O	-0.91550	1.60831	-0.65930
O	2.54551	-1.11324	0.79803
O	1.46222	1.57309	0.58488
N	-2.29443	-0.47734	0.56592
C	-2.29378	-1.90550	0.12111
C	-2.97543	0.40296	-0.43673
C	-1.21271	-2.15653	-0.93292
C	-2.35901	1.80155	-0.43780
C	1.26709	-2.06550	-1.10166
C	-0.13868	2.85688	-0.65175
C	2.39630	-2.27450	-0.09380
C	1.32246	2.44039	-0.60450
C	3.34067	-0.00388	0.24212
C	2.85986	1.25942	0.94647
H	-3.27582	-2.20648	-0.28310
H	-2.07932	-2.52832	0.99514
H	-4.05649	0.47542	-0.23054
H	-2.85209	-0.02923	-1.43390
H	-1.15012	-3.22772	-1.15804
H	-1.39174	-1.60583	-1.86350
H	-2.78040	2.41144	-1.24547

H	-2.50599	2.31724	0.51916
H	1.49001	-1.26421	-1.81520
H	1.09543	-2.99775	-1.65305
H	-0.40038	3.44386	0.23657
H	-0.34651	3.43807	-1.55714
H	2.15934	-3.11186	0.56453
H	3.33679	-2.47805	-0.62212
H	1.60051	1.88900	-1.50961
H	1.96355	3.32195	-0.49491
H	3.18838	0.07366	-0.84091
H	4.40520	-0.17415	0.44386
H	2.85987	1.11140	2.02745
H	3.49938	2.11210	0.69144
Li	-0.10996	0.11044	0.54422
C	-2.92914	-0.34404	1.91282
H	-2.33691	-0.89855	2.64500
H	-3.96151	-0.73084	1.91412
H	-2.94566	0.70931	2.20308

ACE3 Na B3LYP def2sv

40

O	-0.40497	-2.07638	-0.17259
O	-0.49253	1.81831	-0.56405
O	2.16364	-1.30390	0.59442
O	2.02031	1.52180	0.56258
N	-2.44600	-0.09332	0.40778
C	-2.71392	-1.47548	-0.01001
C	-2.68589	0.87819	-0.66605
C	-1.62585	-2.07705	-0.89224
C	-1.86892	2.15678	-0.52761
C	0.70722	-2.62067	-0.86042

C	0.39931	2.91495	-0.48962
C	1.89265	-2.60765	0.09371
C	1.81452	2.37972	-0.55044
C	3.13114	-0.54489	-0.11589
C	3.25091	0.80834	0.56783
H	-3.68933	-1.56913	-0.53732
H	-2.78305	-2.09400	0.90028
H	-3.76268	1.14989	-0.73459
H	-2.41274	0.41738	-1.62849
H	-1.91439	-3.11430	-1.15582
H	-1.50533	-1.51822	-1.84186
H	-2.11852	2.83569	-1.36731
H	-2.10003	2.69539	0.41324
H	0.91980	-2.02872	-1.77418
H	0.50388	-3.66473	-1.17370
H	0.24136	3.47427	0.45481
H	0.23642	3.61289	-1.33492
H	1.65667	-3.23474	0.97017
H	2.78297	-3.03180	-0.40558
H	1.96131	1.83299	-1.50205
H	2.52966	3.22523	-0.52382
H	2.83763	-0.42449	-1.17739
H	4.11522	-1.05413	-0.09144
H	3.51903	0.65440	1.62659
H	4.05216	1.40159	0.08996
Na	0.10018	0.00012	0.87601
C	-3.17579	0.25018	1.62704
H	-2.89290	-0.43804	2.44131
H	-4.28014	0.19842	1.49113
H	-2.92082	1.27297	1.95001

ACE3 Na B3LYP def2tzv

40

O	-0.41653	-2.11509	-0.14959
O	-0.49252	1.82555	-0.60569
O	2.17275	-1.31464	0.60551
O	2.02756	1.53453	0.58683
N	-2.45308	-0.08825	0.41957
C	-2.74080	-1.48604	0.00424
C	-2.71295	0.88822	-0.66877
C	-1.67132	-2.08772	-0.89710
C	-1.91003	2.17297	-0.52814
C	0.72603	-2.65508	-0.87984
C	0.41756	2.95967	-0.48539
C	1.90248	-2.65521	0.07931
C	1.82734	2.42284	-0.56122
C	3.17685	-0.53407	-0.11972
C	3.29533	0.80079	0.59142
H	-3.71258	-1.56441	-0.50660
H	-2.80260	-2.09361	0.90685
H	-3.78175	1.14594	-0.72022
H	-2.44672	0.43167	-1.62069
H	-1.95953	-3.10647	-1.17050
H	-1.53293	-1.51352	-1.81726
H	-2.16664	2.85528	-1.34310
H	-2.10750	2.68056	0.41993
H	0.92321	-2.04195	-1.76408
H	0.51980	-3.67908	-1.20438
H	0.24932	3.46925	0.46742
H	0.24869	3.66839	-1.30062
H	1.67046	-3.27001	0.94654

H	2.78955	-3.05957	-0.41234
H	1.97120	1.87443	-1.49482
H	2.54248	3.24776	-0.51903
H	2.87903	-0.40764	-1.16355
H	4.14008	-1.05033	-0.09077
H	3.53807	0.63905	1.63942
H	4.08520	1.40093	0.13472
Na	0.10343	-0.00451	0.82409
C	-3.18398	0.26281	1.65848
H	-2.90828	-0.42750	2.45417
H	-4.27498	0.22205	1.52120
H	-2.91876	1.26841	1.97963

ACE3 Na B97-1 def2sv

40

O	-0.39795	-2.07405	-0.17518
O	-0.49568	1.81106	-0.56300
O	2.17565	-1.30411	0.60237
O	2.02256	1.53001	0.56571
N	-2.44741	-0.09818	0.40484
C	-2.70985	-1.48074	-0.01789
C	-2.68902	0.87393	-0.66935
C	-1.61296	-2.07324	-0.90045
C	-1.86878	2.15336	-0.52747
C	0.71573	-2.60349	-0.86775
C	0.39363	2.90748	-0.49729
C	1.90165	-2.60039	0.09114
C	1.81183	2.37134	-0.55605
C	3.12628	-0.53998	-0.12059
C	3.24999	0.81604	0.56437
H	-3.68457	-1.57707	-0.54925

H	-2.77757	-2.10361	0.89181
H	-3.76731	1.14590	-0.73767
H	-2.41373	0.41370	-1.63362
H	-1.89814	-3.11076	-1.17399
H	-1.49031	-1.50500	-1.84654
H	-2.11731	2.83455	-1.36800
H	-2.10144	2.69154	0.41565
H	0.92942	-1.99653	-1.77380
H	0.51729	-3.64536	-1.19703
H	0.23727	3.47520	0.44452
H	0.23034	3.60004	-1.34916
H	1.66071	-3.23471	0.96297
H	2.79274	-3.02478	-0.41073
H	1.95381	1.81183	-1.50322
H	2.52704	3.21940	-0.54418
H	2.81618	-0.41810	-1.17938
H	4.11524	-1.04413	-0.11212
H	3.52401	0.66032	1.62302
H	4.05140	1.40885	0.08198
Na	0.08597	-0.00027	0.91951
C	-3.18814	0.24014	1.62019
H	-2.90633	-0.44953	2.43578
H	-4.29263	0.18338	1.47595
H	-2.93863	1.26518	1.94614

ACE3 Na B97-1 def2tzv

40

O	-0.42035	-2.11639	-0.14714
O	-0.48527	1.81726	-0.61022
O	2.17924	-1.32292	0.61396
O	2.03701	1.53905	0.59683

N	-2.45367	-0.08383	0.41898
C	-2.74482	-1.48037	-0.00166
C	-2.71122	0.89287	-0.67139
C	-1.66805	-2.08081	-0.90169
C	-1.89761	2.17519	-0.52962
C	0.72122	-2.63918	-0.88702
C	0.42446	2.94948	-0.49629
C	1.90217	-2.65360	0.07333
C	1.83741	2.40818	-0.56292
C	3.16596	-0.53848	-0.12635
C	3.29729	0.79715	0.59054
H	-3.71597	-1.55485	-0.51936
H	-2.81128	-2.09202	0.90116
H	-3.78085	1.15724	-0.72155
H	-2.44608	0.43419	-1.62574
H	-1.95939	-3.09893	-1.18471
H	-1.52312	-1.49882	-1.81944
H	-2.15301	2.86197	-1.34481
H	-2.09195	2.68285	0.42253
H	0.91774	-2.00790	-1.76232
H	0.51833	-3.66027	-1.23092
H	0.25503	3.46935	0.45388
H	0.26094	3.65331	-1.32027
H	1.66678	-3.27941	0.93490
H	2.78794	-3.05803	-0.42652
H	1.97929	1.84500	-1.49141
H	2.55434	3.23562	-0.53462
H	2.84709	-0.40515	-1.16630
H	4.13311	-1.05364	-0.11933
H	3.54702	0.62779	1.63829

H	4.09095	1.39342	0.12896
Na	0.09408	-0.00470	0.86282
C	-3.20215	0.26601	1.64918
H	-2.93303	-0.42451	2.45024
H	-4.29331	0.22148	1.49705
H	-2.94275	1.27530	1.97219

ACE3 Na CAM-B3LYP def2sv

40

O	-0.40668	-2.05449	-0.17524
O	-0.48518	1.80241	-0.56076
O	2.13491	-1.29475	0.58658
O	2.00075	1.50222	0.56076
N	-2.42291	-0.08786	0.41229
C	-2.70179	-1.46368	0.00665
C	-2.66829	0.87190	-0.66102
C	-1.62738	-2.06678	-0.88025
C	-1.85325	2.14478	-0.53001
C	0.69480	-2.60046	-0.86387
C	0.40334	2.89327	-0.49987
C	1.87435	-2.59298	0.08736
C	1.81126	2.35458	-0.54988
C	3.11595	-0.54686	-0.10176
C	3.22798	0.79878	0.58400
H	-3.68242	-1.55257	-0.50752
H	-2.76134	-2.07803	0.91866
H	-3.74449	1.13917	-0.72532
H	-2.39684	0.40785	-1.62074
H	-1.91271	-3.10579	-1.13273
H	-1.51821	-1.51450	-1.83318
H	-2.09738	2.81880	-1.37287

H	-2.08372	2.68795	0.40644
H	0.90925	-2.00553	-1.77336
H	0.48496	-3.64049	-1.17953
H	0.24554	3.46216	0.43679
H	0.24183	3.57951	-1.35263
H	1.63741	-3.22501	0.95800
H	2.76702	-3.00986	-0.41027
H	1.96134	1.80410	-1.49708
H	2.53035	3.19423	-0.51964
H	2.84121	-0.42354	-1.16645
H	4.09425	-1.06194	-0.06024
H	3.48270	0.64111	1.64379
H	4.03166	1.39607	0.11886
Na	0.10395	0.00219	0.84455
C	-3.14068	0.26539	1.62837
H	-2.85803	-0.41847	2.44436
H	-4.24353	0.21733	1.49576
H	-2.88002	1.28750	1.94396

ACE3 Na CAM-B3LYP def2tzv

40

O	-0.41105	-2.09062	-0.15715
O	-0.49180	1.80787	-0.59789
O	2.14768	-1.30007	0.59515
O	2.00225	1.51832	0.58058
N	-2.43043	-0.08853	0.42270
C	-2.72103	-1.47871	0.01881
C	-2.69783	0.87658	-0.66079
C	-1.66336	-2.07628	-0.88686
C	-1.89807	2.15529	-0.52749
C	0.72088	-2.62848	-0.88351

C	0.41360	2.93371	-0.49915
C	1.88968	-2.63238	0.07386
C	1.81540	2.39356	-0.56508
C	3.16181	-0.52800	-0.10200
C	3.26475	0.79923	0.61099
H	-3.69728	-1.55603	-0.47957
H	-2.77180	-2.08398	0.92213
H	-3.76627	1.12963	-0.70452
H	-2.43642	0.41895	-1.61210
H	-1.94637	-3.09680	-1.15311
H	-1.53839	-1.50580	-1.80969
H	-2.15086	2.83324	-1.34529
H	-2.09707	2.66763	0.41617
H	0.92113	-2.01175	-1.76303
H	0.51144	-3.64856	-1.21262
H	0.24832	3.45775	0.44468
H	0.24431	3.62846	-1.32417
H	1.65548	-3.25182	0.93543
H	2.77937	-3.03096	-0.41486
H	1.95979	1.83814	-1.49308
H	2.53417	3.21345	-0.52586
H	2.88547	-0.39878	-1.15016
H	4.12158	-1.04572	-0.05480
H	3.48987	0.63400	1.66086
H	4.05844	1.40453	0.17110
Na	0.10484	-0.00331	0.79784
C	-3.14384	0.26778	1.65984
H	-2.86125	-0.41580	2.45709
H	-4.23390	0.22399	1.53088
H	-2.87845	1.27463	1.97239

ACE3 Na MP2 def2tzv

40

O	-0.36217	-2.10339	-0.16688
O	-0.53735	1.76519	-0.61901
O	2.24814	-1.29684	0.65815
O	2.01092	1.57810	0.59354
N	-2.48232	-0.13920	0.41976
C	-2.71402	-1.52470	-0.08501
C	-2.78207	0.87438	-0.63536
C	-1.58778	-2.02204	-0.98546
C	-1.95616	2.14479	-0.46905
C	0.81630	-2.54900	-0.93282
C	0.37451	2.92227	-0.55867
C	1.97999	-2.61620	0.04538
C	1.78876	2.38366	-0.62633
C	3.22282	-0.47868	-0.09325
C	3.30781	0.86922	0.60821
H	-3.66534	-1.58960	-0.64461
H	-2.78592	-2.19158	0.77966
H	-3.85452	1.14056	-0.62488
H	-2.55208	0.45026	-1.61659
H	-1.83411	-3.01924	-1.37057
H	-1.40814	-1.34861	-1.83197
H	-2.22282	2.87106	-1.24652
H	-2.10474	2.60296	0.51625
H	1.01428	-1.83552	-1.74253
H	0.63137	-3.54162	-1.36143
H	0.21660	3.47067	0.37755
H	0.18790	3.58640	-1.41081
H	1.74336	-3.28648	0.87241

H	2.87959	-2.97366	-0.46925
H	1.91334	1.76348	-1.52082
H	2.50439	3.21343	-0.65053
H	2.89199	-0.36438	-1.13189
H	4.20392	-0.96827	-0.07824
H	3.55849	0.73498	1.66088
H	4.07144	1.49102	0.12594
Na	0.07006	0.00167	1.03770
C	-3.30616	0.10989	1.63886
H	-2.98705	-0.56739	2.43396
H	-4.38027	-0.04606	1.44017
H	-3.15635	1.13774	1.97542

ACE3 Na PBE0 def2sv

40

O	-0.40157	-2.05772	-0.17063
O	-0.48650	1.79652	-0.55938
O	2.15499	-1.29851	0.59645
O	2.01077	1.51392	0.56398
N	-2.42955	-0.09192	0.40196
C	-2.69619	-1.46497	-0.01020
C	-2.66559	0.86815	-0.66899
C	-1.61164	-2.05981	-0.88756
C	-1.85096	2.13872	-0.53124
C	0.70236	-2.58641	-0.86239
C	0.39730	2.88654	-0.49356
C	1.88173	-2.58653	0.08736
C	1.80575	2.35367	-0.54907
C	3.10227	-0.54188	-0.12220
C	3.23017	0.80288	0.56013
H	-3.67283	-1.56170	-0.53407

H	-2.76196	-2.08327	0.90072
H	-3.74219	1.13778	-0.74430
H	-2.38728	0.40457	-1.62900
H	-1.89866	-3.09607	-1.15583
H	-1.49244	-1.49815	-1.83599
H	-2.09590	2.81621	-1.37322
H	-2.08724	2.68102	0.40657
H	0.91519	-1.98068	-1.76715
H	0.50159	-3.62532	-1.19284
H	0.24045	3.45403	0.44617
H	0.23554	3.57905	-1.34320
H	1.64236	-3.22275	0.95595
H	2.77069	-3.01086	-0.41427
H	1.94964	1.79801	-1.49615
H	2.52070	3.19938	-0.53473
H	2.79256	-0.41688	-1.17890
H	4.08775	-1.04850	-0.11734
H	3.50698	0.64401	1.61570
H	4.03110	1.39480	0.08009
Na	0.08756	0.00019	0.91340
C	-3.15939	0.25476	1.60829
H	-2.88157	-0.43007	2.42676
H	-4.26265	0.20435	1.46782
H	-2.90451	1.27800	1.92940

ACE3 Na PBE0 def2tzv

40

O	-0.42380	-2.09866	-0.14334
O	-0.47505	1.80130	-0.60509
O	2.15848	-1.31782	0.60672
O	2.02706	1.52349	0.59542

N	-2.43641	-0.07654	0.41582
C	-2.72931	-1.46287	0.00732
C	-2.68506	0.88624	-0.67183
C	-1.66514	-2.06585	-0.88625
C	-1.87627	2.15839	-0.53557
C	0.70586	-2.61965	-0.88075
C	0.42833	2.92431	-0.49328
C	1.88091	-2.63716	0.06889
C	1.83142	2.38709	-0.55452
C	3.13871	-0.54090	-0.12735
C	3.27525	0.78315	0.58608
H	-3.70234	-1.53858	-0.50461
H	-2.79507	-2.07002	0.91224
H	-3.75340	1.15038	-0.72967
H	-2.41749	0.42246	-1.62213
H	-1.96018	-3.08326	-1.16449
H	-1.52532	-1.49048	-1.80817
H	-2.12798	2.84089	-1.35429
H	-2.07703	2.67297	0.41068
H	0.90202	-1.99012	-1.75634
H	0.50195	-3.63894	-1.22640
H	0.25818	3.44760	0.45400
H	0.26792	3.62772	-1.31702
H	1.64761	-3.26688	0.92713
H	2.76371	-3.04288	-0.43328
H	1.97571	1.82667	-1.48340
H	2.54723	3.21435	-0.52670
H	2.82082	-0.40401	-1.16634
H	4.10436	-1.05705	-0.12529
H	3.52949	0.60994	1.63127

H 4.07063 1.37622 0.12541

Na 0.09484 -0.00403 0.86000

C -3.17515 0.28278 1.63407

H -2.91039 -0.40061 2.44133

H -4.26568 0.24160 1.48394

H -2.91388 1.29249 1.95078

ACE3 Na PBE def2sv

40

O -0.40663 -2.08248 -0.16457

O -0.48803 1.80750 -0.57100

O 2.18151 -1.31356 0.61190

O 2.03453 1.53584 0.57853

N -2.45322 -0.09214 0.40417

C -2.71871 -1.47490 -0.02121

C -2.68867 0.88128 -0.67190

C -1.62283 -2.06983 -0.90112

C -1.86611 2.15776 -0.52979

C 0.71119 -2.60633 -0.86913

C 0.40437 2.90986 -0.49620

C 1.89783 -2.61087 0.08735

C 1.82078 2.37322 -0.55506

C 3.12260 -0.54375 -0.13212

C 3.26214 0.80701 0.55773

H -3.69995 -1.57223 -0.55678

H -2.79128 -2.10083 0.89448

H -3.77340 1.15793 -0.74750

H -2.40984 0.41503 -1.64047

H -1.91591 -3.11003 -1.18684

H -1.49042 -1.49342 -1.85044

H -2.11792 2.84796 -1.37208

H	-2.09576	2.69884	0.42185
H	0.92283	-1.98800	-1.77751
H	0.51329	-3.65243	-1.21146
H	0.24438	3.47683	0.45478
H	0.24273	3.61413	-1.34890
H	1.65372	-3.25098	0.96327
H	2.79204	-3.04115	-0.42023
H	1.96060	1.80406	-1.50577
H	2.54182	3.22659	-0.55327
H	2.79079	-0.41445	-1.19158
H	4.11758	-1.05311	-0.14850
H	3.54498	0.64248	1.62033
H	4.06999	1.40004	0.06956
Na	0.08416	-0.00165	0.93227
C	-3.19765	0.25009	1.61655
H	-2.92177	-0.44443	2.43904
H	-4.30987	0.20058	1.46887
H	-2.94248	1.28004	1.94546

ACE3 Na PBE def2tzv

40

O	-0.43283	-2.13337	-0.12872
O	-0.47270	1.81032	-0.63034
O	2.18480	-1.33694	0.62737
O	2.04840	1.54462	0.61790
N	-2.45753	-0.07418	0.42005
C	-2.75487	-1.47121	-0.00425
C	-2.70915	0.90392	-0.67399
C	-1.68127	-2.07489	-0.90117
C	-1.89178	2.18091	-0.53479
C	0.71447	-2.63894	-0.89257

C	0.43998	2.95177	-0.49770
C	1.89760	-2.66707	0.06026
C	1.85043	2.41190	-0.55803
C	3.16023	-0.54214	-0.13893
C	3.31032	0.78397	0.58570
H	-3.73282	-1.54327	-0.52637
H	-2.82842	-2.08616	0.90431
H	-3.78529	1.17398	-0.72911
H	-2.44376	0.43738	-1.63269
H	-1.98407	-3.09267	-1.20145
H	-1.51909	-1.48078	-1.81755
H	-2.15268	2.87769	-1.34976
H	-2.07536	2.68992	0.42761
H	0.90224	-1.98585	-1.76314
H	0.51373	-3.66089	-1.25900
H	0.25960	3.46736	0.46162
H	0.28177	3.66862	-1.32159
H	1.66463	-3.30626	0.92187
H	2.78627	-3.07027	-0.45116
H	1.99688	1.83879	-1.48846
H	2.57265	3.24484	-0.53402
H	2.81437	-0.39997	-1.17695
H	4.13305	-1.06260	-0.16100
H	3.57081	0.60439	1.63673
H	4.10961	1.38149	0.11833
Na	0.08972	-0.00759	0.87821
C	-3.21459	0.27995	1.64543
H	-2.95411	-0.41524	2.45414
H	-4.31262	0.24204	1.48535
H	-2.95110	1.29413	1.97213

ACE3 Na B2PLYP def2sv

40

O	-0.39597	-2.07242	-0.16817
O	-0.49432	1.79957	-0.56958
O	2.17253	-1.30018	0.60392
O	2.01243	1.52474	0.56654
N	-2.43642	-0.09922	0.40360
C	-2.70143	-1.47780	-0.01933
C	-2.68204	0.86754	-0.67014
C	-1.61004	-2.06440	-0.90103
C	-1.86985	2.14393	-0.52641
C	0.72048	-2.58903	-0.87220
C	0.39303	2.90099	-0.49810
C	1.89988	-2.59611	0.08326
C	1.80505	2.36737	-0.55808
C	3.12101	-0.53303	-0.12373
C	3.24489	0.81378	0.56366
H	-3.67540	-1.56947	-0.54700
H	-2.76645	-2.10153	0.88628
H	-3.75941	1.13156	-0.73848
H	-2.40300	0.41071	-1.63121
H	-1.89248	-3.09713	-1.18185
H	-1.48076	-1.49024	-1.83796
H	-2.11843	2.82752	-1.36051
H	-2.09609	2.67396	0.41836
H	0.93029	-1.96704	-1.76411
H	0.52277	-3.62257	-1.21639
H	0.23554	3.45996	0.44435
H	0.22646	3.59406	-1.34455
H	1.65865	-3.23409	0.94788

H	2.79111	-3.01218	-0.41848
H	1.94632	1.80772	-1.50085
H	2.52077	3.21060	-0.54393
H	2.80391	-0.40771	-1.17599
H	4.10607	-1.03746	-0.12182
H	3.51812	0.65568	1.61851
H	4.03963	1.41142	0.08340
Na	0.08430	0.00239	0.94067
C	-3.18701	0.23736	1.61081
H	-2.90820	-0.44774	2.42699
H	-4.28627	0.17309	1.45616
H	-2.94668	1.26173	1.93455

ACE3 Na B2PLYP def2tzv

40

O	-0.40653	-2.12203	-0.14678
O	-0.49991	1.80958	-0.61054
O	2.19340	-1.31398	0.62087
O	2.02782	1.54835	0.59256
N	-2.45426	-0.09834	0.41662
C	-2.73515	-1.49269	-0.01904
C	-2.72478	0.88368	-0.66653
C	-1.65278	-2.07253	-0.91891
C	-1.91991	2.16623	-0.51897
C	0.74815	-2.62624	-0.89511
C	0.41017	2.95197	-0.50105
C	1.92096	-2.64911	0.06808
C	1.81989	2.41458	-0.57737
C	3.17965	-0.51955	-0.12452
C	3.30128	0.81426	0.58872
H	-3.70021	-1.56176	-0.54407

H	-2.80392	-2.11332	0.87423
H	-3.79447	1.14051	-0.70327
H	-2.46357	0.43436	-1.62336
H	-1.93368	-3.08386	-1.22380
H	-1.49496	-1.47056	-1.81716
H	-2.17564	2.85723	-1.32621
H	-2.10621	2.66167	0.43711
H	0.94165	-1.97466	-1.75155
H	0.54953	-3.63830	-1.25747
H	0.24417	3.46645	0.44870
H	0.23531	3.64989	-1.32353
H	1.68841	-3.27957	0.92281
H	2.81033	-3.03771	-0.43207
H	1.95556	1.84466	-1.49868
H	2.53576	3.23898	-0.55410
H	2.85472	-0.39113	-1.15947
H	4.14553	-1.03050	-0.11648
H	3.55259	0.65510	1.63430
H	4.08100	1.41891	0.12071
Na	0.08723	-0.00391	0.90322
C	-3.21528	0.23217	1.64680
H	-2.93248	-0.45332	2.44372
H	-4.30127	0.16186	1.48731
H	-2.97936	1.24436	1.96905

ACE3 Na DSDPBEP86 def2sv

40

O	-0.39507	-2.07588	-0.16472
O	-0.49606	1.78083	-0.59061
O	2.17364	-1.30078	0.61341
O	2.00186	1.52719	0.57527

N	-2.42085	-0.10224	0.40446
C	-2.69212	-1.47604	-0.01928
C	-2.68246	0.86118	-0.66458
C	-1.60217	-2.05118	-0.90678
C	-1.86767	2.13362	-0.52462
C	0.72465	-2.55425	-0.88827
C	0.38871	2.88307	-0.51544
C	1.90042	-2.58511	0.06838
C	1.79905	2.34834	-0.56475
C	3.10658	-0.52503	-0.12265
C	3.23264	0.81574	0.57254
H	-3.67149	-1.56406	-0.54185
H	-2.74963	-2.10608	0.88525
H	-3.76335	1.12254	-0.72032
H	-2.41093	0.40617	-1.63113
H	-1.88493	-3.07954	-1.21026
H	-1.46408	-1.45645	-1.83182
H	-2.12481	2.82637	-1.35112
H	-2.07742	2.65656	0.43012
H	0.92931	-1.89386	-1.75598
H	0.53585	-3.57718	-1.27293
H	0.22648	3.44287	0.42790
H	0.22553	3.57585	-1.36528
H	1.65312	-3.24024	0.92058
H	2.79492	-2.99344	-0.43869
H	1.93936	1.76983	-1.49854
H	2.51835	3.19113	-0.56438
H	2.77190	-0.39268	-1.17069
H	4.09534	-1.02622	-0.13934
H	3.50300	0.64991	1.62878

H	4.03018	1.41758	0.09715
Na	0.07078	0.00636	0.96988
C	-3.16208	0.23225	1.61546
H	-2.87301	-0.45325	2.43048
H	-4.26395	0.16429	1.46844
H	-2.92055	1.25924	1.93694

ACE3 Na DSDPBEP86 def2tzv

40

O	-0.39890	-2.12112	-0.15057
O	-0.50662	1.78374	-0.63443
O	2.20254	-1.31350	0.63631
O	2.01624	1.55693	0.60381
N	-2.44499	-0.10601	0.42065
C	-2.72364	-1.49469	-0.02601
C	-2.73521	0.87756	-0.65334
C	-1.63624	-2.05176	-0.93344
C	-1.91992	2.15321	-0.51068
C	0.76125	-2.57706	-0.91908
C	0.40255	2.92670	-0.53246
C	1.93082	-2.63180	0.04714
C	1.81227	2.38625	-0.59174
C	3.17307	-0.50600	-0.11297
C	3.28936	0.82501	0.60706
H	-3.69206	-1.56022	-0.55226
H	-2.78628	-2.12836	0.86288
H	-3.80790	1.13801	-0.66652
H	-2.49214	0.43123	-1.62031
H	-1.91313	-3.05738	-1.27070
H	-1.46962	-1.42058	-1.81410
H	-2.18598	2.85790	-1.30706

H	-2.08289	2.63877	0.45859
H	0.95121	-1.87928	-1.74389
H	0.57267	-3.57511	-1.33166
H	0.23185	3.45235	0.41420
H	0.23200	3.61663	-1.36662
H	1.69301	-3.28881	0.88414
H	2.82466	-3.00603	-0.46339
H	1.94703	1.78640	-1.49819
H	2.53176	3.21211	-0.59215
H	2.83397	-0.37257	-1.14639
H	4.14613	-1.01017	-0.11902
H	3.53514	0.66155	1.65650
H	4.07205	1.43504	0.14295
Na	0.07059	-0.00189	0.94655
C	-3.20744	0.21020	1.65209
H	-2.90659	-0.47106	2.45056
H	-4.29517	0.11759	1.49408
H	-2.98859	1.23075	1.97142

ACE3 Na HSE06 def2sv

40

O	-0.40140	-2.05718	-0.17086
O	-0.48669	1.79617	-0.55964
O	2.15524	-1.29859	0.59650
O	2.01098	1.51446	0.56450
N	-2.43039	-0.09196	0.40239
C	-2.69659	-1.46554	-0.00877
C	-2.66587	0.86722	-0.66963
C	-1.61254	-2.05961	-0.88721
C	-1.85189	2.13811	-0.53154
C	0.70273	-2.58657	-0.86307

C	0.39748	2.88676	-0.49435
C	1.88196	-2.58720	0.08680
C	1.80574	2.35358	-0.54982
C	3.10297	-0.54126	-0.12207
C	3.23100	0.80297	0.56110
H	-3.67380	-1.56294	-0.53158
H	-2.76100	-2.08356	0.90247
H	-3.74260	1.13616	-0.74606
H	-2.38636	0.40305	-1.62907
H	-1.89881	-3.09588	-1.15599
H	-1.49415	-1.49722	-1.83535
H	-2.09661	2.81614	-1.37305
H	-2.08822	2.67979	0.40665
H	0.91562	-1.98097	-1.76789
H	0.50115	-3.62522	-1.19364
H	0.24052	3.45438	0.44522
H	0.23525	3.57885	-1.34414
H	1.64247	-3.22348	0.95522
H	2.77116	-3.01098	-0.41471
H	1.94907	1.79655	-1.49617
H	2.52124	3.19873	-0.53660
H	2.79289	-0.41547	-1.17856
H	4.08810	-1.04843	-0.11791
H	3.50708	0.64369	1.61671
H	4.03188	1.39528	0.08159
Na	0.08924	0.00015	0.91387
C	-3.16083	0.25606	1.60810
H	-2.88308	-0.42731	2.42775
H	-4.26401	0.20475	1.46707
H	-2.90687	1.28002	1.92761

ACE3 Na HSE06 def2tzv

40

O	-0.42381	-2.09792	-0.14367
O	-0.47548	1.80109	-0.60568
O	2.15852	-1.31783	0.60660
O	2.02663	1.52354	0.59566
N	-2.43738	-0.07669	0.41632
C	-2.73005	-1.46362	0.00917
C	-2.68586	0.88543	-0.67205
C	-1.66700	-2.06591	-0.88573
C	-1.87799	2.15782	-0.53576
C	0.70650	-2.61942	-0.88192
C	0.42864	2.92494	-0.49416
C	1.88096	-2.63817	0.06779
C	1.83121	2.38739	-0.55561
C	3.14063	-0.54011	-0.12637
C	3.27614	0.78316	0.58791
H	-3.70366	-1.54000	-0.50106
H	-2.79418	-2.07021	0.91420
H	-3.75413	1.14872	-0.73067
H	-2.41761	0.42135	-1.62172
H	-1.96091	-3.08332	-1.16369
H	-1.52820	-1.49038	-1.80732
H	-2.12945	2.84049	-1.35390
H	-2.07815	2.67183	0.41056
H	0.90279	-1.98921	-1.75655
H	0.50143	-3.63781	-1.22839
H	0.25838	3.44786	0.45289
H	0.26752	3.62786	-1.31770
H	1.64777	-3.26778	0.92566

H	2.76399	-3.04292	-0.43398
H	1.97529	1.82612	-1.48361
H	2.54760	3.21366	-0.52794
H	2.82371	-0.40299	-1.16527
H	4.10553	-1.05686	-0.12337
H	3.52850	0.60996	1.63315
H	4.07129	1.37710	0.12880
Na	0.09633	-0.00460	0.85821
C	-3.17426	0.28418	1.63537
H	-2.90868	-0.39818	2.44278
H	-4.26476	0.24315	1.48680
H	-2.91239	1.29391	1.95051

ACE3 Na M062X def2sv

40

O	-0.39809	-2.01754	-0.18753
O	-0.48267	1.76363	-0.56920
O	2.12803	-1.28961	0.59690
O	1.98479	1.49916	0.57390
N	-2.40609	-0.08779	0.42064
C	-2.69344	-1.46354	0.02124
C	-2.67029	0.86230	-0.65729
C	-1.62382	-2.05023	-0.88228
C	-1.84438	2.12810	-0.53522
C	0.69999	-2.53771	-0.90056
C	0.40720	2.85483	-0.54587
C	1.87295	-2.57235	0.05831
C	1.81142	2.30519	-0.57193
C	3.11271	-0.53742	-0.07978
C	3.21388	0.80222	0.61951
H	-3.68264	-1.55120	-0.47648

H	-2.73093	-2.07979	0.93416
H	-3.74755	1.12850	-0.70485
H	-2.40935	0.39310	-1.61842
H	-1.89229	-3.09392	-1.13254
H	-1.53178	-1.49272	-1.83436
H	-2.08029	2.80231	-1.37988
H	-2.06362	2.67488	0.40250
H	0.91963	-1.90091	-1.78055
H	0.48427	-3.56139	-1.26073
H	0.25068	3.45343	0.37234
H	0.24640	3.50981	-1.42242
H	1.62286	-3.23052	0.90526
H	2.77069	-2.97296	-0.44356
H	1.95662	1.71219	-1.49468
H	2.53967	3.13695	-0.57178
H	2.84190	-0.40578	-1.14529
H	4.08988	-1.05351	-0.03527
H	3.44491	0.63398	1.68278
H	4.02320	1.40749	0.17447
Na	0.08714	-0.00225	0.88592
C	-3.13129	0.27136	1.63197
H	-2.83753	-0.39766	2.45602
H	-4.23173	0.20269	1.49324
H	-2.88494	1.30264	1.92935

ACE3 Na M062X def2tzv

40

O	-0.40107	-2.05031	-0.17698
O	-0.49076	1.77145	-0.59678
O	2.13888	-1.29261	0.61247
O	1.99255	1.51899	0.59012

N	-2.41670	-0.09276	0.42854
C	-2.71273	-1.47991	0.01467
C	-2.70552	0.87459	-0.64931
C	-1.65132	-2.04982	-0.90724
C	-1.88940	2.14375	-0.51456
C	0.73126	-2.57179	-0.91175
C	0.41722	2.89761	-0.54190
C	1.88471	-2.61385	0.06577
C	1.81607	2.34301	-0.59208
C	3.15007	-0.52150	-0.08642
C	3.25373	0.80234	0.63646
H	-3.69395	-1.55126	-0.47485
H	-2.74621	-2.09746	0.91178
H	-3.77405	1.13020	-0.66839
H	-2.45980	0.42123	-1.60803
H	-1.91471	-3.07230	-1.18675
H	-1.53618	-1.46142	-1.82086
H	-2.13468	2.83240	-1.32601
H	-2.07212	2.64935	0.43682
H	0.94831	-1.92124	-1.76371
H	0.51553	-3.57715	-1.28002
H	0.25971	3.45132	0.38674
H	0.24377	3.56257	-1.39029
H	1.62417	-3.24729	0.90997
H	2.78195	-3.00817	-0.41346
H	1.94679	1.74237	-1.49493
H	2.54628	3.15407	-0.59036
H	2.86357	-0.38469	-1.13212
H	4.10806	-1.04355	-0.04815
H	3.46429	0.62715	1.68823

H	4.05199	1.41060	0.20777
Na	0.09018	-0.00610	0.83423
C	-3.13741	0.25563	1.66658
H	-2.84006	-0.42026	2.46561
H	-4.22555	0.18911	1.53222
H	-2.88919	1.27031	1.97061

ACE3 Na M06 def2sv

40

O	-0.36187	-2.00873	-0.18439
O	-0.50618	1.72458	-0.59542
O	2.22042	-1.29248	0.62137
O	1.99759	1.52147	0.57534
N	-2.46014	-0.11816	0.40720
C	-2.67224	-1.49236	-0.02880
C	-2.70155	0.84499	-0.66102
C	-1.56525	-2.01763	-0.91150
C	-1.86332	2.09213	-0.52915
C	0.75533	-2.44072	-0.91994
C	0.37488	2.81768	-0.55042
C	1.92675	-2.53580	0.02447
C	1.78189	2.29743	-0.57979
C	3.14677	-0.50223	-0.08430
C	3.23123	0.83786	0.60033
H	-3.64987	-1.61254	-0.55416
H	-2.71595	-2.13299	0.87254
H	-3.77954	1.12470	-0.71576
H	-2.44962	0.38235	-1.63245
H	-1.81152	-3.05278	-1.23226
H	-1.44934	-1.41335	-1.83833
H	-2.11061	2.79604	-1.35254

H	-2.06152	2.62713	0.42650
H	0.96411	-1.72700	-1.74951
H	0.56383	-3.43529	-1.37754
H	0.20514	3.40708	0.37778
H	0.20772	3.49462	-1.41511
H	1.67361	-3.23410	0.84322
H	2.81374	-2.93882	-0.50439
H	1.92887	1.69127	-1.49977
H	2.49626	3.14675	-0.61626
H	2.83692	-0.38282	-1.14576
H	4.14762	-0.98494	-0.08664
H	3.48983	0.68403	1.66389
H	4.03003	1.45316	0.13922
Na	0.04919	0.00053	1.07744
C	-3.25545	0.18915	1.58098
H	-2.97460	-0.47357	2.41933
H	-4.34888	0.07005	1.39063
H	-3.08020	1.23060	1.90483

ACE3 Na M06 def2tzv

40

O	-0.37944	-2.03768	-0.18066
O	-0.50327	1.72634	-0.62515
O	2.20215	-1.29546	0.62729
O	1.99636	1.53255	0.59849
N	-2.46038	-0.10688	0.42730
C	-2.69848	-1.49459	-0.00611
C	-2.72434	0.86241	-0.65017
C	-1.61616	-2.01708	-0.92201
C	-1.89081	2.11312	-0.51849
C	0.76119	-2.46677	-0.95044

C	0.40609	2.84451	-0.58310
C	1.92051	-2.57117	0.00717
C	1.80049	2.29420	-0.61470
C	3.18821	-0.49488	-0.06085
C	3.25710	0.82811	0.65760
H	-3.67516	-1.59720	-0.50643
H	-2.72364	-2.12445	0.88590
H	-3.79097	1.13487	-0.68009
H	-2.48353	0.40438	-1.61063
H	-1.86829	-3.02978	-1.25446
H	-1.49553	-1.39104	-1.81408
H	-2.14393	2.81735	-1.31834
H	-2.05542	2.61658	0.44192
H	0.96171	-1.74111	-1.74975
H	0.56890	-3.44209	-1.41067
H	0.24414	3.42143	0.33472
H	0.24184	3.50224	-1.44284
H	1.66428	-3.25617	0.81501
H	2.80637	-2.95334	-0.50770
H	1.92389	1.65514	-1.49575
H	2.52903	3.10912	-0.66772
H	2.90720	-0.36195	-1.11193
H	4.16472	-0.98860	-0.02737
H	3.45986	0.65942	1.71460
H	4.05921	1.44394	0.23995
Na	0.06471	-0.00729	1.00197
C	-3.23715	0.21144	1.63166
H	-2.96072	-0.46523	2.44025
H	-4.32062	0.12224	1.45275
H	-3.02875	1.23081	1.95678

ACE3 Na MP2 def2sv

40

O	-0.39145	-2.07026	-0.16303
O	-0.49368	1.77255	-0.58702
O	2.18825	-1.30079	0.61812
O	2.01134	1.52799	0.57241
N	-2.43251	-0.10434	0.39791
C	-2.69312	-1.47621	-0.03976
C	-2.68265	0.86246	-0.67151
C	-1.59211	-2.04027	-0.91942
C	-1.86591	2.13160	-0.51974
C	0.73154	-2.54314	-0.88985
C	0.39085	2.87807	-0.51478
C	1.90564	-2.58302	0.06740
C	1.80057	2.34409	-0.57199
C	3.11240	-0.52519	-0.13270
C	3.24469	0.81644	0.55818
H	-3.66701	-1.56364	-0.57315
H	-2.75678	-2.11429	0.85858
H	-3.76290	1.12489	-0.73683
H	-2.40188	0.41293	-1.63748
H	-1.87026	-3.06652	-1.23454
H	-1.44533	-1.43798	-1.83762
H	-2.11926	2.83062	-1.34226
H	-2.07625	2.64771	0.43822
H	0.93835	-1.87644	-1.75158
H	0.54347	-3.56308	-1.28286
H	0.23441	3.43754	0.42933
H	0.22310	3.57088	-1.36374
H	1.65340	-3.23895	0.91709

H	2.79810	-2.99574	-0.44002
H	1.93254	1.76243	-1.50440
H	2.51946	3.18730	-0.58089
H	2.76542	-0.39337	-1.17608
H	4.10151	-1.02537	-0.16030
H	3.52637	0.65306	1.61152
H	4.03646	1.41832	0.07323
Na	0.06609	0.01035	1.04581
C	-3.21105	0.21774	1.59054
H	-2.93514	-0.46662	2.41024
H	-4.30660	0.13535	1.41217
H	-2.99151	1.24635	1.91990

ACE3 Na SCS-MP2 def2sv

40

O	-0.39377	-2.07432	-0.16702
O	-0.49947	1.78595	-0.58966
O	2.15797	-1.29081	0.61226
O	1.98823	1.52154	0.57157
N	-2.41208	-0.10447	0.41226
C	-2.69175	-1.47818	-0.02097
C	-2.68907	0.86717	-0.65135
C	-1.60147	-2.05319	-0.91225
C	-1.87224	2.14114	-0.51192
C	0.72520	-2.56883	-0.88516
C	0.38512	2.89140	-0.51458
C	1.89854	-2.58597	0.07782
C	1.79656	2.35353	-0.56548
C	3.11407	-0.52588	-0.11035
C	3.22730	0.81955	0.58227
H	-3.67272	-1.55762	-0.54238

H	-2.74921	-2.11439	0.87966
H	-3.77142	1.12626	-0.69353
H	-2.42665	0.41951	-1.62403
H	-1.88377	-3.08322	-1.21251
H	-1.46486	-1.46009	-1.83850
H	-2.13521	2.83611	-1.33545
H	-2.07489	2.66096	0.44597
H	0.93473	-1.92191	-1.76186
H	0.53341	-3.59770	-1.25353
H	0.22557	3.44935	0.43040
H	0.22009	3.58507	-1.36392
H	1.65008	-3.23470	0.93514
H	2.79945	-2.99295	-0.41953
H	1.93838	1.78219	-1.50349
H	2.51880	3.19433	-0.55390
H	2.80399	-0.39923	-1.16654
H	4.10109	-1.03154	-0.09913
H	3.48841	0.65793	1.64190
H	4.02528	1.42645	0.11315
Na	0.09051	0.00407	0.91008
C	-3.15364	0.21955	1.63205
H	-2.85586	-0.46975	2.44096
H	-4.25504	0.14489	1.48811
H	-2.91609	1.24587	1.95865

ACE3 Na SCS-MP2 def2tzv

40

O	-0.37378	-2.09892	-0.16840
O	-0.53034	1.78427	-0.60408
O	2.20217	-1.28870	0.64165
O	1.99438	1.55886	0.58685

N	-2.45136	-0.12869	0.42822
C	-2.72020	-1.51839	-0.05363
C	-2.76998	0.87791	-0.63097
C	-1.61198	-2.05116	-0.96349
C	-1.94941	2.15870	-0.48070
C	0.79130	-2.58966	-0.92038
C	0.37840	2.93956	-0.54655
C	1.95722	-2.62539	0.06306
C	1.79535	2.39655	-0.61204
C	3.21262	-0.49450	-0.08318
C	3.29276	0.85844	0.61749
H	-3.68146	-1.57413	-0.59666
H	-2.78979	-2.17135	0.82233
H	-3.84423	1.13704	-0.61358
H	-2.54475	0.44741	-1.61096
H	-1.86740	-3.06284	-1.30410
H	-1.45193	-1.41162	-1.84038
H	-2.21176	2.86551	-1.27819
H	-2.11303	2.64042	0.49136
H	0.99598	-1.91289	-1.76020
H	0.59484	-3.59814	-1.30594
H	0.22011	3.49026	0.38898
H	0.19299	3.60308	-1.40020
H	1.72230	-3.27900	0.90495
H	2.86298	-2.98703	-0.43807
H	1.93016	1.79896	-1.52112
H	2.51464	3.22412	-0.60529
H	2.91706	-0.38249	-1.13336
H	4.18573	-0.99840	-0.03252
H	3.53296	0.72398	1.67345

H	4.06192	1.48075	0.14418
Na	0.09047	-0.00188	0.91192
C	-3.23202	0.14652	1.67182
H	-2.90267	-0.53360	2.46139
H	-4.31556	0.01337	1.50958
H	-3.04860	1.17261	1.99897

ACE3 NoMetal B2PLYP def2sv

39

O	-0.36235	-2.08525	-0.06277
O	-0.52874	1.76536	-0.52957
O	2.30331	-1.30155	0.75733
O	2.07810	1.57996	0.67085
N	-2.53812	-0.14629	0.46187
C	-2.68414	-1.49953	-0.05677
C	-2.74671	0.88740	-0.54478
C	-1.49944	-1.96765	-0.88683
C	-1.87885	2.11740	-0.33241
C	0.78921	-2.48949	-0.76338
C	0.36192	2.84549	-0.38309
C	1.96116	-2.55386	0.20089
C	1.78068	2.33481	-0.48386
C	3.13507	-0.50156	-0.05399
C	3.29005	0.85670	0.60725
H	-3.61117	-1.62202	-0.66359
H	-2.77435	-2.18660	0.80044
H	-3.81328	1.20450	-0.59057
H	-2.49698	0.48436	-1.53825
H	-1.74954	-2.94816	-1.34380
H	-1.29480	-1.26677	-1.72065
H	-2.18401	2.90590	-1.05208

H	-2.02256	2.53327	0.68563
H	1.00320	-1.78622	-1.59397
H	0.64607	-3.49328	-1.21693
H	0.22166	3.33981	0.59952
H	0.19090	3.60863	-1.17054
H	1.68388	-3.20486	1.04599
H	2.83045	-3.00983	-0.31250
H	1.87430	1.72609	-1.40340
H	2.47586	3.19415	-0.57583
H	2.71379	-0.38171	-1.07043
H	4.13610	-0.96825	-0.16634
H	3.62143	0.70448	1.64746
H	4.07213	1.43624	0.07934
C	-3.34368	0.07914	1.64789
H	-3.06265	-0.63582	2.43865
H	-4.43790	-0.02586	1.45767
H	-3.16993	1.09483	2.03882

ACE3 NoMetal B2PLYP def2tzv

39

O	-0.40660	-2.22135	-0.01863
O	-0.50780	1.84079	-0.63031
O	2.35328	-1.36632	0.78166
O	2.15192	1.62651	0.72133
N	-2.57544	-0.12288	0.47013
C	-2.73362	-1.49702	-0.05509
C	-2.74662	0.91881	-0.56747
C	-1.55766	-1.98345	-0.89015
C	-1.90109	2.16056	-0.31938
C	0.79798	-2.57018	-0.76574
C	0.41617	2.93339	-0.33712

C	1.97028	-2.65181	0.19637
C	1.83050	2.41761	-0.47006
C	3.13463	-0.50719	-0.10851
C	3.36612	0.81867	0.59450
H	-3.64715	-1.58972	-0.66778
H	-2.84664	-2.17057	0.79522
H	-3.80384	1.22278	-0.64253
H	-2.45945	0.51487	-1.53783
H	-1.83280	-2.91792	-1.39067
H	-1.28308	-1.25349	-1.65648
H	-2.23997	2.97657	-0.96637
H	-1.97653	2.48715	0.72173
H	0.97510	-1.82358	-1.54549
H	0.66843	-3.54483	-1.24918
H	0.25102	3.30292	0.67903
H	0.25443	3.75417	-1.04302
H	1.70812	-3.28543	1.04054
H	2.82836	-3.08974	-0.32375
H	1.91193	1.80877	-1.37347
H	2.52804	3.25693	-0.54443
H	2.61112	-0.35635	-1.05499
H	4.09990	-0.98083	-0.31702
H	3.70689	0.63745	1.61140
H	4.13429	1.38371	0.05793
C	-3.45316	0.11764	1.63318
H	-3.21994	-0.59488	2.42303
H	-4.52122	0.02198	1.37582
H	-3.28647	1.12106	2.02182

ACE3 NoMetal B3LYP def2sv

39

O	-0.37639	-2.09909	-0.06255
O	-0.52156	1.78710	-0.52559
O	2.31107	-1.31539	0.75232
O	2.10708	1.58469	0.66673
N	-2.56125	-0.13772	0.45472
C	-2.70279	-1.49597	-0.05674
C	-2.74616	0.90074	-0.55359
C	-1.51662	-1.98147	-0.88237
C	-1.87446	2.13270	-0.33572
C	0.77655	-2.51861	-0.75192
C	0.37557	2.86031	-0.36842
C	1.95440	-2.57086	0.21258
C	1.79745	2.34895	-0.47918
C	3.13980	-0.51692	-0.06423
C	3.30903	0.84570	0.59418
H	-3.62764	-1.62471	-0.66865
H	-2.80173	-2.17672	0.80615
H	-3.81058	1.22810	-0.61440
H	-2.48876	0.49431	-1.54522
H	-1.77700	-2.96583	-1.32947
H	-1.30999	-1.29228	-1.72802
H	-2.18003	2.92187	-1.05722
H	-2.02548	2.55138	0.68217
H	0.99292	-1.83280	-1.59888
H	0.63287	-3.53208	-1.18811
H	0.23798	3.34705	0.62067
H	0.20703	3.63531	-1.14742
H	1.67733	-3.20924	1.06982
H	2.81786	-3.04356	-0.29929
H	1.88827	1.74876	-1.40683

H	2.49047	3.21249	-0.56976
H	2.71344	-0.39685	-1.08062
H	4.14030	-0.98726	-0.18436
H	3.64734	0.69252	1.63407
H	4.09695	1.41433	0.05975
C	-3.35503	0.09858	1.64697
H	-3.08118	-0.62156	2.43792
H	-4.45503	0.01144	1.46717
H	-3.16472	1.11202	2.04082

ACE3 NoMetal B3LYP def2tzv

39

O	-0.43114	-2.21814	-0.01455
O	-0.48323	1.84207	-0.60575
O	2.34284	-1.38305	0.77056
O	2.18208	1.61611	0.71680
N	-2.59139	-0.09974	0.46091
C	-2.75639	-1.47978	-0.03931
C	-2.73135	0.93480	-0.58412
C	-1.59137	-1.99731	-0.87221
C	-1.87899	2.17369	-0.34044
C	0.76572	-2.58719	-0.75724
C	0.44352	2.93156	-0.33115
C	1.94342	-2.66526	0.19860
C	1.85733	2.41475	-0.46415
C	3.13089	-0.53224	-0.11619
C	3.37994	0.79136	0.58513
H	-3.67486	-1.58488	-0.64314
H	-2.87033	-2.13707	0.82355
H	-3.78314	1.25142	-0.68376
H	-2.43611	0.51854	-1.54663

H	-1.88241	-2.94312	-1.34257
H	-1.32472	-1.29475	-1.66715
H	-2.19902	2.97652	-1.01406
H	-1.98202	2.53001	0.68906
H	0.94625	-1.85869	-1.55388
H	0.62924	-3.56930	-1.22498
H	0.28219	3.31928	0.67960
H	0.28369	3.74632	-1.04570
H	1.67878	-3.28915	1.05022
H	2.79234	-3.12380	-0.31944
H	1.94183	1.81589	-1.37438
H	2.55348	3.25636	-0.53493
H	2.61237	-0.37577	-1.06504
H	4.09204	-1.01451	-0.32695
H	3.72063	0.60345	1.60162
H	4.16077	1.34253	0.05191
C	-3.44482	0.17169	1.62997
H	-3.23072	-0.54653	2.42066
H	-4.52053	0.11476	1.38964
H	-3.24183	1.16824	2.02019

ACE3 NoMetal B97-1 def2sv

39

O	-0.37210	-2.09050	-0.06256
O	-0.52450	1.77461	-0.53036
O	2.31886	-1.31634	0.75461
O	2.09979	1.58419	0.67293
N	-2.56003	-0.13900	0.46096
C	-2.70266	-1.50091	-0.04326
C	-2.75069	0.89250	-0.55514
C	-1.51649	-1.98447	-0.87538

C	-1.87491	2.12643	-0.34580
C	0.77758	-2.49327	-0.76437
C	0.37019	2.84908	-0.38126
C	1.96188	-2.56188	0.19641
C	1.79559	2.33600	-0.48057
C	3.14787	-0.51280	-0.05394
C	3.30493	0.85279	0.60902
H	-3.63200	-1.63585	-0.64972
H	-2.79273	-2.17847	0.82543
H	-3.81663	1.22004	-0.61418
H	-2.49539	0.47894	-1.54644
H	-1.77444	-2.97400	-1.31662
H	-1.32004	-1.29621	-1.72679
H	-2.17809	2.91139	-1.07541
H	-2.02734	2.55352	0.67051
H	0.98984	-1.78882	-1.59972
H	0.63483	-3.50006	-1.21989
H	0.22869	3.34715	0.60356
H	0.20529	3.61654	-1.17069
H	1.68746	-3.21600	1.04450
H	2.82445	-3.02622	-0.32786
H	1.88968	1.72570	-1.40358
H	2.48926	3.20039	-0.57719
H	2.72718	-0.39258	-1.07483
H	4.15354	-0.97731	-0.16798
H	3.63628	0.69973	1.65272
H	4.09534	1.42664	0.08038
C	-3.35835	0.10340	1.65054
H	-3.08362	-0.61299	2.44668
H	-4.45893	0.01347	1.46777

H	-3.16841	1.12106	2.03828

ACE3 NoMetal B97-1 def2tzv

39

O	-0.43175	-2.20792	-0.01333
O	-0.47891	1.82874	-0.60948
O	2.34510	-1.38671	0.77381
O	2.18167	1.61339	0.72386
N	-2.58825	-0.09585	0.46757
C	-2.76011	-1.47771	-0.02886
C	-2.73151	0.93226	-0.58525
C	-1.59426	-1.99745	-0.86630
C	-1.87064	2.17106	-0.34876
C	0.75938	-2.56650	-0.76585
C	0.44491	2.91949	-0.34107
C	1.94367	-2.65909	0.18749
C	1.86318	2.39999	-0.46387
C	3.13224	-0.53564	-0.10973
C	3.37889	0.79149	0.59532
H	-3.68230	-1.58283	-0.63136
H	-2.87094	-2.13409	0.83851
H	-3.78472	1.25288	-0.68239
H	-2.43923	0.50880	-1.54861
H	-1.88609	-2.94869	-1.33140
H	-1.33501	-1.29570	-1.66861
H	-2.18730	2.97294	-1.02939
H	-1.97477	2.53434	0.68140
H	0.93891	-1.82465	-1.55462
H	0.62207	-3.54442	-1.24805
H	0.28070	3.31662	0.66854
H	0.28917	3.73049	-1.06466

H	1.67855	-3.29629	1.03230
H	2.79115	-3.11357	-0.34185
H	1.94971	1.79207	-1.37135
H	2.56026	3.24366	-0.54077
H	2.61398	-0.37693	-1.06184
H	4.09725	-1.01608	-0.32050
H	3.71803	0.60083	1.61449
H	4.16327	1.34342	0.06264
C	-3.45357	0.18156	1.62818
H	-3.24346	-0.53285	2.42668
H	-4.52903	0.12062	1.37773
H	-3.25429	1.18360	2.01328

ACE3 NoMetal CAM-B3LYP def2sv

39

O	-0.35723	-2.08436	-0.06843
O	-0.53525	1.76764	-0.52920
O	2.30438	-1.29661	0.75225
O	2.07416	1.58071	0.66387
N	-2.54366	-0.15182	0.45686
C	-2.67549	-1.50251	-0.05811
C	-2.74680	0.88293	-0.54208
C	-1.49020	-1.96485	-0.88583
C	-1.87993	2.11054	-0.32899
C	0.79305	-2.48373	-0.75965
C	0.35437	2.83920	-0.38135
C	1.96241	-2.54423	0.20390
C	1.77101	2.33054	-0.48420
C	3.13145	-0.49575	-0.05105
C	3.28044	0.86167	0.60733
H	-3.60073	-1.63322	-0.66474

H	-2.76221	-2.18922	0.79941
H	-3.81220	1.20200	-0.58679
H	-2.49933	0.48212	-1.53689
H	-1.73827	-2.94309	-1.34834
H	-1.28714	-1.26001	-1.71703
H	-2.18950	2.90162	-1.04366
H	-2.02271	2.52291	0.69087
H	1.00750	-1.78125	-1.59110
H	0.65437	-3.48775	-1.21371
H	0.21610	3.33071	0.60315
H	0.18301	3.60599	-1.16504
H	1.68389	-3.19412	1.04974
H	2.83119	-3.00344	-0.30742
H	1.86216	1.72080	-1.40359
H	2.46473	3.19035	-0.58097
H	2.71335	-0.37802	-1.06934
H	4.13443	-0.95824	-0.16152
H	3.60958	0.70994	1.64855
H	4.06474	1.44091	0.08243
C	-3.32965	0.07177	1.64953
H	-3.04383	-0.64572	2.43643
H	-4.42657	-0.02820	1.47283
H	-3.14941	1.08529	2.04378

ACE3 NoMetal CAM-B3LYP def2tzv

39

O	-0.41910	-2.20572	-0.01916
O	-0.48922	1.83172	-0.60386
O	2.33499	-1.36929	0.76808
O	2.16064	1.60909	0.70959
N	-2.57629	-0.10835	0.45785

C	-2.73244	-1.47986	-0.04254
C	-2.72072	0.92182	-0.57825
C	-1.56835	-1.98250	-0.87213
C	-1.87418	2.15468	-0.33148
C	0.77364	-2.56046	-0.75408
C	0.43000	2.91319	-0.32935
C	1.94164	-2.64325	0.20187
C	1.83756	2.39976	-0.46315
C	3.10960	-0.52042	-0.11403
C	3.35427	0.79700	0.58494
H	-3.64973	-1.58485	-0.64430
H	-2.84108	-2.14092	0.81646
H	-3.77265	1.23258	-0.67251
H	-2.42620	0.51102	-1.54185
H	-1.85138	-2.92273	-1.35460
H	-1.30178	-1.26984	-1.65616
H	-2.19794	2.96271	-0.99434
H	-1.97291	2.49980	0.70082
H	0.95541	-1.82259	-1.53991
H	0.64193	-3.53542	-1.23370
H	0.27103	3.29490	0.68245
H	0.26681	3.72945	-1.03874
H	1.67087	-3.26832	1.04872
H	2.79225	-3.10058	-0.31135
H	1.91811	1.79872	-1.37075
H	2.53391	3.23868	-0.53779
H	2.58518	-0.36366	-1.05809
H	4.06936	-0.99841	-0.33148
H	3.69441	0.60711	1.59963
H	4.13243	1.35173	0.05448

C	-3.42536	0.15524	1.62143
H	-3.20544	-0.55847	2.41269
H	-4.49772	0.08683	1.37890
H	-3.23324	1.15315	2.00988

ACE3 NoMetal DSDPBEP86 def2sv

39

O	-0.34635	-2.07275	-0.06539
O	-0.54106	1.73984	-0.53689
O	2.30542	-1.29027	0.76384
O	2.04748	1.57893	0.67756
N	-2.52230	-0.15737	0.47090
C	-2.66655	-1.50742	-0.05087
C	-2.75424	0.87009	-0.53464
C	-1.48208	-1.95478	-0.88890
C	-1.88658	2.09883	-0.33200
C	0.80427	-2.44557	-0.78179
C	0.34413	2.82478	-0.40792
C	1.97501	-2.53142	0.17947
C	1.76168	2.31240	-0.49166
C	3.13817	-0.48193	-0.03545
C	3.26936	0.87436	0.63089
H	-3.59986	-1.63182	-0.65106
H	-2.74287	-2.20156	0.80492
H	-3.82534	1.18005	-0.56482
H	-2.51539	0.46671	-1.53306
H	-1.72215	-2.93310	-1.36043
H	-1.28269	-1.23755	-1.71255
H	-2.19363	2.88823	-1.05280
H	-2.02254	2.51621	0.68866
H	1.01252	-1.71140	-1.58937

H	0.66583	-3.43645	-1.26841
H	0.19944	3.33709	0.56686
H	0.17356	3.57315	-1.21205
H	1.69911	-3.20644	1.00844
H	2.85048	-2.96873	-0.34365
H	1.85858	1.68488	-1.40056
H	2.46123	3.16938	-0.59452
H	2.72540	-0.36311	-1.05751
H	4.14631	-0.93984	-0.13765
H	3.58874	0.72322	1.67690
H	4.05135	1.46802	0.11424
C	-3.33060	0.05928	1.65530
H	-3.03657	-0.65013	2.44904
H	-4.42418	-0.06384	1.46349
H	-3.17069	1.08099	2.04211

ACE3 NoMetal DSDPBEP86 def2tzv

39

O	-0.36086	-2.21587	-0.03039
O	-0.55288	1.82187	-0.67427
O	2.37900	-1.34382	0.78750
O	2.07802	1.63550	0.73071
N	-2.54501	-0.15840	0.48944
C	-2.69329	-1.53326	-0.03389
C	-2.76983	0.86996	-0.55075
C	-1.52060	-1.98916	-0.88946
C	-1.93937	2.12547	-0.32820
C	0.84258	-2.50216	-0.80141
C	0.35962	2.91949	-0.37222
C	2.02025	-2.61034	0.15201
C	1.77829	2.40690	-0.47649

C	3.15718	-0.45622	-0.07378
C	3.32187	0.87268	0.64272
H	-3.61919	-1.63699	-0.63184
H	-2.77742	-2.21340	0.81901
H	-3.83822	1.15129	-0.59571
H	-2.49999	0.46480	-1.52948
H	-1.78270	-2.92449	-1.40240
H	-1.26605	-1.23981	-1.64896
H	-2.30651	2.93999	-0.96697
H	-1.99210	2.44864	0.71906
H	1.00250	-1.70768	-1.54145
H	0.72748	-3.45449	-1.33738
H	0.17747	3.29154	0.64371
H	0.20464	3.73892	-1.08581
H	1.77041	-3.28802	0.96941
H	2.88710	-3.00870	-0.39173
H	1.87469	1.78241	-1.37199
H	2.47734	3.24924	-0.55136
H	2.65471	-0.31608	-1.03718
H	4.14578	-0.89837	-0.25694
H	3.63689	0.69705	1.67225
H	4.08786	1.46910	0.13044
C	-3.41902	0.06443	1.65856
H	-3.15151	-0.63265	2.45570
H	-4.48761	-0.07304	1.40942
H	-3.28292	1.08130	2.03312

ACE3 NoMetal HSE06 def2sv

39

O	-0.37478	-2.08055	-0.05477
O	-0.51342	1.76347	-0.53019

O	2.29980	-1.31458	0.74849
O	2.08813	1.57131	0.67436
N	-2.54065	-0.13203	0.45722
C	-2.68917	-1.48753	-0.03231
C	-2.72429	0.88443	-0.56001
C	-1.51624	-1.97849	-0.85899
C	-1.85785	2.11245	-0.35595
C	0.76512	-2.48153	-0.75828
C	0.37320	2.83380	-0.37547
C	1.94511	-2.55219	0.19013
C	1.79052	2.32809	-0.46821
C	3.11677	-0.51281	-0.06070
C	3.28391	0.83960	0.60162
H	-3.62108	-1.62457	-0.63083
H	-2.77812	-2.15762	0.84010
H	-3.78913	1.20714	-0.63164
H	-2.46058	0.46456	-1.54473
H	-1.77776	-2.96815	-1.29248
H	-1.32200	-1.29897	-1.71561
H	-2.15799	2.89236	-1.08900
H	-2.01617	2.54511	0.65513
H	0.97399	-1.77743	-1.59231
H	0.61979	-3.48472	-1.21568
H	0.22619	3.32824	0.60825
H	0.21010	3.60280	-1.16079
H	1.67611	-3.21099	1.03381
H	2.80547	-3.01215	-0.33751
H	1.89111	1.72624	-1.39389
H	2.48247	3.19215	-0.55566
H	2.68485	-0.38562	-1.07419

H	4.11741	-0.97881	-0.19116
H	3.62302	0.68057	1.63993
H	4.07208	1.41197	0.07228
C	-3.32903	0.12391	1.63775
H	-3.05846	-0.58435	2.43981
H	-4.42849	0.03885	1.45867
H	-3.13444	1.14202	2.01637

ACE3 NoMetal HSE06 def2tzv

39

O	-0.43394	-2.19533	-0.00912
O	-0.46983	1.81488	-0.60709
O	2.32924	-1.38527	0.76816
O	2.16886	1.60126	0.72376
N	-2.56979	-0.09031	0.46352
C	-2.74438	-1.46383	-0.01901
C	-2.70651	0.92395	-0.58632
C	-1.59245	-1.98908	-0.85085
C	-1.85315	2.15471	-0.35675
C	0.74742	-2.54647	-0.76083
C	0.44595	2.89889	-0.33978
C	1.92655	-2.64481	0.17964
C	1.85517	2.38556	-0.45405
C	3.10156	-0.53410	-0.11148
C	3.35407	0.78011	0.59058
H	-3.66899	-1.57147	-0.61510
H	-2.85464	-2.11452	0.85127
H	-3.75844	1.24257	-0.69110
H	-2.41150	0.49591	-1.54564
H	-1.88883	-2.94018	-1.31028
H	-1.33678	-1.29469	-1.65934

H	-2.16834	2.95255	-1.04073
H	-1.96339	2.52472	0.66929
H	0.92473	-1.80163	-1.54579
H	0.61046	-3.51920	-1.25060
H	0.27820	3.29899	0.66687
H	0.29301	3.70897	-1.06304
H	1.66553	-3.28971	1.01828
H	2.77019	-3.09658	-0.35563
H	1.94665	1.78183	-1.36259
H	2.55051	3.22922	-0.52857
H	2.57531	-0.36982	-1.05705
H	4.06292	-1.01208	-0.33723
H	3.69934	0.58470	1.60550
H	4.13847	1.32921	0.05758
C	-3.41911	0.19791	1.61762
H	-3.21056	-0.50908	2.42129
H	-4.49517	0.14059	1.37457
H	-3.21560	1.20054	1.99511

ACE3 NoMetal M062X def2sv

39

O	-0.32706	-2.06801	-0.06921
O	-0.55483	1.73415	-0.54549
O	2.31927	-1.28154	0.75980
O	2.02641	1.57736	0.67488
N	-2.52452	-0.17170	0.47096
C	-2.65018	-1.52441	-0.04199
C	-2.76135	0.84952	-0.53642
C	-1.46273	-1.95369	-0.88442
C	-1.89818	2.08106	-0.33835
C	0.82260	-2.42618	-0.78481

C	0.32193	2.81843	-0.40714
C	1.99443	-2.51656	0.17344
C	1.74358	2.31771	-0.48448
C	3.13931	-0.46218	-0.03208
C	3.25365	0.89292	0.63835
H	-3.58268	-1.66374	-0.63495
H	-2.70960	-2.21474	0.81598
H	-3.83102	1.15482	-0.56219
H	-2.52511	0.44334	-1.53291
H	-1.69084	-2.92766	-1.36463
H	-1.27231	-1.22564	-1.69899
H	-2.20998	2.86903	-1.05459
H	-2.03271	2.49400	0.68282
H	1.02645	-1.68124	-1.58196
H	0.68743	-3.40907	-1.28230
H	0.16915	3.32026	0.56982
H	0.14999	3.56945	-1.20469
H	1.72127	-3.19790	0.99560
H	2.86910	-2.94518	-0.35335
H	1.85274	1.69897	-1.39669
H	2.43631	3.17829	-0.57258
H	2.72200	-0.34263	-1.05135
H	4.14892	-0.90891	-0.13883
H	3.56896	0.74068	1.68353
H	4.02877	1.49642	0.12799
C	-3.32734	0.04531	1.65553
H	-3.02384	-0.65256	2.45290
H	-4.41729	-0.09193	1.46682
H	-3.17870	1.07064	2.03214

ACE3 NoMetal M062X def2tzv

39

O	-0.36673	-2.18062	-0.04165
O	-0.53966	1.79244	-0.64387
O	2.36140	-1.34204	0.77583
O	2.07173	1.61242	0.71795
N	-2.54409	-0.14924	0.48371
C	-2.69165	-1.52496	-0.01314
C	-2.75686	0.86802	-0.55693
C	-1.53176	-1.98240	-0.87623
C	-1.91786	2.11225	-0.33926
C	0.82524	-2.46071	-0.80749
C	0.37051	2.88624	-0.39128
C	1.99563	-2.59105	0.14319
C	1.78240	2.36829	-0.48399
C	3.15185	-0.46300	-0.05941
C	3.30594	0.85916	0.66032
H	-3.62485	-1.64485	-0.58638
H	-2.75246	-2.19177	0.84746
H	-3.81803	1.15576	-0.60700
H	-2.48938	0.45792	-1.52994
H	-1.78961	-2.92391	-1.37024
H	-1.30007	-1.24471	-1.64941
H	-2.26102	2.91707	-0.99618
H	-1.99187	2.45559	0.69664
H	0.99163	-1.65918	-1.53368
H	0.70478	-3.39912	-1.35762
H	0.19754	3.29551	0.60788
H	0.21394	3.67816	-1.12863
H	1.72792	-3.26858	0.95056
H	2.85533	-3.00291	-0.39299

H	1.87816	1.73884	-1.37188
H	2.48390	3.20245	-0.56613
H	2.67109	-0.31833	-1.02945
H	4.13746	-0.90690	-0.22653
H	3.59648	0.67347	1.69177
H	4.08560	1.45229	0.17471
C	-3.38602	0.09846	1.65965
H	-3.12142	-0.59278	2.45762
H	-4.45644	-0.02073	1.43057
H	-3.22844	1.11201	2.02425

ACE3 NoMetal M06 def2sv

39

O	-0.34654	-2.05090	-0.05770
O	-0.53442	1.72298	-0.54470
O	2.32237	-1.29239	0.75730
O	2.05890	1.55774	0.67463
N	-2.55440	-0.15403	0.46940
C	-2.66696	-1.50401	-0.04364
C	-2.75294	0.87250	-0.53724
C	-1.48016	-1.93981	-0.86986
C	-1.87080	2.08220	-0.33619
C	0.79745	-2.40065	-0.77893
C	0.35153	2.79391	-0.39763
C	1.97199	-2.51268	0.16243
C	1.76509	2.29201	-0.48120
C	3.16338	-0.48721	-0.02039
C	3.27831	0.86661	0.63653
H	-3.59903	-1.64910	-0.64614
H	-2.74257	-2.19566	0.81764
H	-3.82005	1.20118	-0.57798

H	-2.52176	0.46133	-1.53777
H	-1.70905	-2.91858	-1.35178
H	-1.28459	-1.22141	-1.69916
H	-2.17382	2.88583	-1.04668
H	-1.99961	2.50057	0.68936
H	0.99687	-1.64416	-1.57391
H	0.65510	-3.37613	-1.29992
H	0.20092	3.29383	0.58671
H	0.18161	3.56252	-1.18585
H	1.69984	-3.20426	0.98217
H	2.83743	-2.95562	-0.37694
H	1.86834	1.67193	-1.39927
H	2.46270	3.15318	-0.58586
H	2.77413	-0.37625	-1.05686
H	4.17587	-0.94394	-0.10972
H	3.59998	0.72305	1.68552
H	4.06181	1.46485	0.12272
C	-3.38310	0.06377	1.62837
H	-3.10757	-0.63471	2.44020
H	-4.47370	-0.07046	1.41306
H	-3.24731	1.09091	2.01499

ACE3 NoMetal M06 def2tzv

39

O	-0.34973	-2.09112	-0.05115
O	-0.55088	1.72257	-0.60344
O	2.39095	-1.32049	0.78449
O	2.07555	1.58688	0.70835
N	-2.59774	-0.16041	0.48462
C	-2.69200	-1.52842	-0.02620
C	-2.78929	0.86925	-0.53994

C	-1.51582	-1.93533	-0.88248
C	-1.91755	2.08393	-0.32268
C	0.83586	-2.35847	-0.81966
C	0.36603	2.81544	-0.41356
C	1.99986	-2.53951	0.12266
C	1.76793	2.28893	-0.51296
C	3.22915	-0.46163	-0.01249
C	3.32692	0.87354	0.68200
H	-3.61875	-1.68169	-0.60819
H	-2.73753	-2.20680	0.82950
H	-3.84430	1.18834	-0.58326
H	-2.54464	0.46024	-1.52223
H	-1.73915	-2.88463	-1.38609
H	-1.31303	-1.18707	-1.65920
H	-2.23237	2.89596	-0.99085
H	-1.99846	2.44533	0.71063
H	1.01704	-1.53072	-1.51897
H	0.70624	-3.27404	-1.41124
H	0.21466	3.26940	0.57363
H	0.19928	3.58560	-1.17649
H	1.71707	-3.23664	0.91131
H	2.84968	-2.96219	-0.42584
H	1.83914	1.62306	-1.38066
H	2.47358	3.11596	-0.65379
H	2.81930	-0.34316	-1.02208
H	4.23015	-0.89981	-0.10947
H	3.60770	0.71740	1.72347
H	4.10239	1.48034	0.19977
C	-3.45395	0.05933	1.64630
H	-3.20004	-0.64594	2.43833

H	-4.52470	-0.05965	1.40262
H	-3.30917	1.06841	2.03485

ACE3 NoMetal MP2 def2sv

39

O	-0.34376	-2.07148	-0.06459
O	-0.54034	1.73768	-0.53620
O	2.29611	-1.28390	0.76682
O	2.04024	1.57650	0.67664
N	-2.51217	-0.15916	0.47229
C	-2.66122	-1.50418	-0.06291
C	-2.75308	0.87231	-0.52793
C	-1.47422	-1.94556	-0.89859
C	-1.88630	2.09979	-0.32180
C	0.80812	-2.45239	-0.78006
C	0.34317	2.82766	-0.40737
C	1.97397	-2.53203	0.18591
C	1.75983	2.31560	-0.49300
C	3.13412	-0.48136	-0.03678
C	3.26676	0.87457	0.62764
H	-3.59339	-1.61839	-0.66665
H	-2.74089	-2.20690	0.78519
H	-3.82543	1.17846	-0.55176
H	-2.51647	0.47682	-1.52960
H	-1.71235	-2.91985	-1.37897
H	-1.26826	-1.22152	-1.71382
H	-2.19556	2.89319	-1.03696
H	-2.01701	2.50992	0.70187
H	1.01987	-1.72346	-1.59056
H	0.66858	-3.44612	-1.25994
H	0.20035	3.33770	0.56819

H	0.16999	3.57603	-1.21060
H	1.69533	-3.20295	1.01668
H	2.85394	-2.96856	-0.32971
H	1.85623	1.69213	-1.40395
H	2.46023	3.17228	-0.59065
H	2.72242	-0.36223	-1.05842
H	4.14080	-0.94265	-0.13616
H	3.58817	0.72437	1.67271
H	4.04573	1.46998	0.10903
C	-3.33397	0.04463	1.65179
H	-3.03842	-0.66528	2.44355
H	-4.42324	-0.08914	1.44871
H	-3.18652	1.06563	2.04298

ACE3 NoMetal MP2 def2tzv

39

O	-0.31703	-2.23512	-0.06646
O	-0.61865	1.80729	-0.70703
O	2.41239	-1.31072	0.81431
O	2.02807	1.66210	0.72871
N	-2.55823	-0.20781	0.50961
C	-2.66063	-1.56847	-0.07970
C	-2.83776	0.85526	-0.49414
C	-1.46015	-1.93410	-0.94593
C	-1.99418	2.10550	-0.27166
C	0.91494	-2.45587	-0.83656
C	0.30977	2.91284	-0.42096
C	2.08198	-2.57580	0.13378
C	1.72753	2.38652	-0.52013
C	3.22832	-0.40610	-0.01294
C	3.31073	0.93933	0.69460

H	-3.57806	-1.66985	-0.69212
H	-2.72429	-2.29065	0.74076
H	-3.90779	1.13545	-0.47407
H	-2.61514	0.47956	-1.49766
H	-1.69227	-2.82056	-1.55221
H	-1.18977	-1.10836	-1.61546
H	-2.38796	2.94453	-0.86133
H	-1.98570	2.38344	0.79002
H	1.06507	-1.61851	-1.53024
H	0.82959	-3.38442	-1.41822
H	0.13520	3.29451	0.59276
H	0.15478	3.72096	-1.14711
H	1.83636	-3.28033	0.92959
H	2.96523	-2.93446	-0.41193
H	1.80981	1.72089	-1.38694
H	2.43215	3.22066	-0.63154
H	2.78154	-0.29322	-1.00701
H	4.23834	-0.82378	-0.12428
H	3.59114	0.79480	1.73889
H	4.06610	1.56177	0.19576
C	-3.44631	-0.07082	1.69363
H	-3.13169	-0.77530	2.46692
H	-4.50501	-0.26366	1.43935
H	-3.36377	0.94302	2.09244

ACE3 NoMetal PBE0 def2sv

39

O	-0.37570	-2.08156	-0.05479
O	-0.51301	1.76456	-0.52784
O	2.29771	-1.31370	0.74812
O	2.08940	1.57133	0.67312

N	-2.54021	-0.13159	0.45621
C	-2.68921	-1.48609	-0.03571
C	-2.72404	0.88678	-0.55897
C	-1.51531	-1.97787	-0.86060
C	-1.85682	2.11417	-0.35413
C	0.76401	-2.48515	-0.75617
C	0.37365	2.83436	-0.37423
C	1.94376	-2.55230	0.19293
C	1.79083	2.32837	-0.46838
C	3.11533	-0.51416	-0.06174
C	3.28403	0.83895	0.59889
H	-3.62003	-1.62132	-0.63616
H	-2.78059	-2.15722	0.83563
H	-3.78863	1.21034	-0.62913
H	-2.46168	0.46839	-1.54460
H	-1.77762	-2.96710	-1.29460
H	-1.31887	-1.29841	-1.71668
H	-2.15646	2.89397	-1.08753
H	-2.01546	2.54704	0.65673
H	0.97329	-1.78445	-1.59285
H	0.61906	-3.49025	-1.20949
H	0.22788	3.32929	0.60942
H	0.21025	3.60310	-1.15976
H	1.67463	-3.20893	1.03834
H	2.80437	-3.01371	-0.33305
H	1.89034	1.72717	-1.39455
H	2.48260	3.19260	-0.55603
H	2.68402	-0.38792	-1.07550
H	4.11571	-0.98106	-0.19101
H	3.62473	0.68067	1.63686

H	4.07196	1.41012	0.06788
C	-3.32775	0.12208	1.63773
H	-3.05665	-0.58816	2.43792
H	-4.42734	0.03798	1.45946
H	-3.13209	1.13912	2.01868

ACE3 NoMetal PBE0 def2tzv

39

O	-0.43333	-2.19536	-0.00927
O	-0.47045	1.81495	-0.60731
O	2.32940	-1.38416	0.76806
O	2.16783	1.60087	0.72308
N	-2.56941	-0.09090	0.46380
C	-2.74337	-1.46370	-0.02076
C	-2.70679	0.92435	-0.58494
C	-1.59006	-1.98848	-0.85145
C	-1.85236	2.15472	-0.35552
C	0.74743	-2.54554	-0.76039
C	0.44483	2.89793	-0.33983
C	1.92728	-2.64277	0.17990
C	1.85441	2.38446	-0.45388
C	3.10246	-0.53427	-0.11037
C	3.35286	0.78137	0.59058
H	-3.66715	-1.57072	-0.61866
H	-2.85499	-2.11550	0.84889
H	-3.75882	1.24389	-0.68811
H	-2.41319	0.49695	-1.54527
H	-1.88676	-2.93936	-1.31204
H	-1.33372	-1.29340	-1.65954
H	-2.16873	2.95308	-1.03895
H	-1.96195	2.52428	0.67108

H	0.92444	-1.80084	-1.54597
H	0.61175	-3.51901	-1.24983
H	0.27736	3.29822	0.66717
H	0.29267	3.70861	-1.06312
H	1.66645	-3.28827	1.01862
H	2.77078	-3.09486	-0.35603
H	1.94566	1.78099	-1.36298
H	2.54946	3.22882	-0.52869
H	2.57857	-0.37136	-1.05780
H	4.06532	-1.01136	-0.33333
H	3.69846	0.58675	1.60594
H	4.13745	1.33044	0.05712
C	-3.42101	0.19486	1.61674
H	-3.21311	-0.51345	2.41986
H	-4.49684	0.13706	1.37180
H	-3.21875	1.19729	1.99628

ACE3 NoMetal PBE def2sv

39

O	-0.37670	-2.08884	-0.05094
O	-0.51767	1.76539	-0.53786
O	2.32982	-1.32593	0.76173
O	2.10806	1.58730	0.68657
N	-2.56885	-0.13367	0.46252
C	-2.71065	-1.49912	-0.03367
C	-2.74983	0.89210	-0.56153
C	-1.52754	-1.98726	-0.86582
C	-1.87289	2.12490	-0.35630
C	0.77306	-2.48706	-0.76747
C	0.37715	2.84675	-0.38021
C	1.96190	-2.56794	0.18587

C	1.80294	2.33734	-0.47604
C	3.14942	-0.51445	-0.05989
C	3.31490	0.84675	0.60858
H	-3.64875	-1.64579	-0.63963
H	-2.79803	-2.17441	0.84599
H	-3.82208	1.22434	-0.63554
H	-2.48554	0.46731	-1.55373
H	-1.78974	-2.98446	-1.30777
H	-1.33253	-1.29960	-1.72890
H	-2.17697	2.91475	-1.09261
H	-2.02781	2.56105	0.66490
H	0.98262	-1.77045	-1.60424
H	0.62799	-3.49529	-1.23907
H	0.22971	3.34507	0.61287
H	0.21366	3.62565	-1.17028
H	1.68708	-3.23295	1.03494
H	2.82447	-3.03639	-0.35130
H	1.89926	1.72094	-1.40429
H	2.49967	3.20894	-0.57954
H	2.71166	-0.38679	-1.08139
H	4.16077	-0.98043	-0.19665
H	3.65181	0.68516	1.65705
H	4.11284	1.42260	0.07711
C	-3.37207	0.12065	1.64563
H	-3.10820	-0.59843	2.45227
H	-4.48130	0.04098	1.45829
H	-3.17505	1.14451	2.03300

ACE3 NoMetal PBE def2tzv

39

O	-0.45320	-2.21241	0.00387

O	-0.45607	1.82004	-0.61855
O	2.33955	-1.40648	0.78265
O	2.20373	1.61227	0.74321
N	-2.59230	-0.07653	0.46915
C	-2.77865	-1.46019	-0.02169
C	-2.72108	0.94775	-0.59115
C	-1.62221	-1.99762	-0.85664
C	-1.85428	2.18049	-0.35951
C	0.73751	-2.57271	-0.76768
C	0.47172	2.91910	-0.34222
C	1.92556	-2.67748	0.17545
C	1.88734	2.39903	-0.45866
C	3.12222	-0.55124	-0.11860
C	3.39466	0.76600	0.58972
H	-3.71027	-1.56371	-0.62564
H	-2.89772	-2.11384	0.85559
H	-3.77908	1.27716	-0.70185
H	-2.42395	0.51180	-1.55572
H	-1.92663	-2.95566	-1.31786
H	-1.35495	-1.30175	-1.67212
H	-2.16609	2.98770	-1.04843
H	-1.95721	2.55344	0.67560
H	0.91346	-1.82158	-1.55931
H	0.59287	-3.55307	-1.26092
H	0.29990	3.31916	0.67343
H	0.31936	3.73796	-1.06954
H	1.66370	-3.32614	1.02202
H	2.77369	-3.13322	-0.36738
H	1.97741	1.78262	-1.36918
H	2.59091	3.24694	-0.54005

H	2.58362	-0.38050	-1.06612
H	4.08455	-1.04315	-0.35499
H	3.74436	0.56515	1.61126
H	4.18703	1.31335	0.04886
C	-3.46032	0.21814	1.62411
H	-3.26652	-0.50153	2.43096
H	-4.54384	0.17611	1.36810
H	-3.24553	1.22355	2.01096

ACE3 NoMetal SCS-MP2 def2sv

39

O	-0.35011	-2.08000	-0.06396
O	-0.53575	1.75121	-0.53227
O	2.29310	-1.28698	0.76547
O	2.05424	1.57837	0.67332
N	-2.51778	-0.15531	0.46867
C	-2.66937	-1.50171	-0.07148
C	-2.75269	0.88304	-0.53101
C	-1.47865	-1.94964	-0.90354
C	-1.88430	2.11144	-0.31648
C	0.80401	-2.47671	-0.77140
C	0.35062	2.84065	-0.39611
C	1.96959	-2.54384	0.19999
C	1.76900	2.32712	-0.49167
C	3.13295	-0.49037	-0.04582
C	3.27796	0.86850	0.61603
H	-3.59889	-1.61041	-0.68066
H	-2.75719	-2.20590	0.77532
H	-3.82452	1.19132	-0.55821
H	-2.51272	0.49052	-1.53360
H	-1.71956	-2.92446	-1.38229

H	-1.26539	-1.22821	-1.71942
H	-2.19269	2.90844	-1.02861
H	-2.01545	2.51625	0.70944
H	1.01916	-1.76216	-1.59388
H	0.66182	-3.47871	-1.23385
H	0.21104	3.34224	0.58466
H	0.17494	3.59560	-1.19315
H	1.68840	-3.20238	1.04063
H	2.85061	-2.98912	-0.30721
H	1.86217	1.70942	-1.40728
H	2.47009	3.18425	-0.58477
H	2.71673	-0.37264	-1.06609
H	4.13676	-0.95834	-0.14734
H	3.60612	0.71862	1.65977
H	4.05664	1.45922	0.09089
C	-3.34463	0.04631	1.64918
H	-3.05124	-0.66636	2.44053
H	-4.43392	-0.08625	1.44356
H	-3.19717	1.06667	2.04439

ACE3 NoMetal SCS-MP2 def2tzv

39

O	-0.32211	-2.23869	-0.05962
O	-0.60994	1.82109	-0.69723
O	2.40683	-1.31307	0.80875
O	2.03972	1.66121	0.72508
N	-2.55900	-0.20234	0.50421
C	-2.66855	-1.56683	-0.08056
C	-2.83160	0.86095	-0.50477
C	-1.46517	-1.94954	-0.94208
C	-1.98987	2.11616	-0.27648

C	0.90702	-2.48543	-0.82538
C	0.31454	2.92743	-0.40541
C	2.07769	-2.58833	0.14881
C	1.73774	2.40559	-0.51109
C	3.21936	-0.41606	-0.02917
C	3.31823	0.93299	0.67770
H	-3.58407	-1.66481	-0.69666
H	-2.74212	-2.28487	0.74332
H	-3.90200	1.14081	-0.49449
H	-2.60064	0.48379	-1.50615
H	-1.70113	-2.84639	-1.53255
H	-1.19306	-1.13727	-1.62762
H	-2.38074	2.95216	-0.87361
H	-1.99329	2.39931	0.78418
H	1.06096	-1.66761	-1.54143
H	0.81607	-3.42899	-1.38260
H	0.13933	3.30414	0.61048
H	0.15634	3.73948	-1.12754
H	1.83126	-3.27965	0.95685
H	2.96126	-2.95794	-0.39000
H	1.82530	1.75501	-1.38935
H	2.43936	3.24484	-0.60740
H	2.76416	-0.30229	-1.01962
H	4.22633	-0.84029	-0.14884
H	3.60950	0.78601	1.71948
H	4.07324	1.55082	0.17189
C	-3.45125	-0.05756	1.68644
H	-3.14032	-0.75961	2.46461
H	-4.51036	-0.24895	1.43140
H	-3.36725	0.95811	2.08225

ACE3 Rb B3LYP def2sv

40

O	2.79768	0.05083	0.54548
O	-2.00689	-1.26131	-0.01208
O	0.74432	2.00181	0.40781
O	-2.04577	1.58192	0.03479
N	0.75409	-1.92464	0.55340
C	2.13122	-2.24787	0.16803
C	-0.23144	-2.91759	0.12988
C	2.91523	-1.03441	-0.34700
C	-1.67294	-2.54909	0.48435
C	3.02337	1.34509	0.03186
C	-3.02992	-0.59366	0.70133
C	2.08883	2.33704	0.70527
C	-3.23129	0.80669	0.12785
C	-0.23597	2.84545	0.98894
C	-1.51037	2.06339	1.25570
H	2.12644	-2.99181	-0.64749
H	2.67767	-2.71850	1.01320
H	-0.04573	-3.92070	0.58457
H	-0.14795	-3.04878	-0.96441
H	3.97789	-1.32318	-0.49560
H	2.52450	-0.74372	-1.34431
H	-2.34896	-3.31588	0.05583
H	-1.81456	-2.57106	1.58098
H	2.85510	1.36685	-1.06404
H	4.07034	1.66820	0.20652
H	-2.76709	-0.54682	1.77723
H	-3.99542	-1.13535	0.62499
H	2.24843	2.31638	1.80222

H	2.33353	3.35677	0.34352
H	-3.61548	0.73231	-0.90340
H	-3.99484	1.33018	0.73721
H	-0.44332	3.70777	0.32299
H	0.12135	3.24302	1.95835
H	-1.27903	1.22923	1.94519
H	-2.24102	2.73079	1.75659
Rb	-0.23851	0.23991	-1.76628
C	0.66331	-1.59760	1.97355
H	1.47810	-0.91071	2.23661
H	0.73747	-2.50300	2.62221
H	-0.28597	-1.08688	2.20292

ACE3 Rb B3LYP def2tzv

40

O	2.91960	0.01829	0.56078
O	-2.06329	-1.27359	-0.02751
O	0.78218	2.03501	0.32376
O	-2.06455	1.61838	0.02263
N	0.75544	-1.91959	0.53766
C	2.13552	-2.26849	0.12147
C	-0.24837	-2.93189	0.13853
C	2.93597	-1.07545	-0.40521
C	-1.68052	-2.57351	0.53017
C	3.09786	1.36101	0.01697
C	-3.07095	-0.56234	0.75267
C	2.16239	2.33257	0.70792
C	-3.28338	0.82198	0.15674
C	-0.22541	2.89736	0.94706
C	-1.46991	2.09519	1.26972
H	2.09623	-2.99853	-0.69012

H	2.67977	-2.74583	0.94753
H	-0.04288	-3.91523	0.59790
H	-0.18876	-3.06711	-0.94269
H	3.96681	-1.38708	-0.60308
H	2.50796	-0.73019	-1.35037
H	-2.35587	-3.34410	0.15032
H	-1.78694	-2.54306	1.61624
H	2.90560	1.36368	-1.05909
H	4.12889	1.68686	0.17726
H	-2.74712	-0.50491	1.79537
H	-4.02572	-1.09686	0.72629
H	2.26436	2.25129	1.79332
H	2.42431	3.35105	0.40543
H	-3.66913	0.73825	-0.85699
H	-4.01786	1.35807	0.76513
H	-0.46000	3.72067	0.26920
H	0.16285	3.31194	1.88071
H	-1.20827	1.25467	1.91471
H	-2.18512	2.73546	1.79527
Rb	-0.29006	0.22511	-1.74262
C	0.70148	-1.59626	1.97932
H	1.50100	-0.90117	2.21059
H	0.81702	-2.49563	2.60652
H	-0.24234	-1.11979	2.23443

ACE3 Rb B97-1 def2sv

40

O	2.85068	0.02195	0.55188
O	-2.03659	-1.23698	-0.00980
O	0.76219	1.93550	0.44132
O	-2.03062	1.60089	0.00433

N	0.71655	-1.88298	0.55276
C	2.09303	-2.25624	0.20826
C	-0.27248	-2.89799	0.18638
C	2.92291	-1.07852	-0.32408
C	-1.71406	-2.50747	0.52934
C	3.03990	1.30676	0.00693
C	-3.06936	-0.55144	0.66684
C	2.10663	2.30063	0.68698
C	-3.23089	0.85058	0.07538
C	-0.20258	2.80685	0.99999
C	-1.50827	2.06177	1.23611
H	2.08148	-3.02074	-0.59034
H	2.60892	-2.72099	1.07850
H	-0.08515	-3.87666	0.69407
H	-0.19356	-3.08636	-0.90176
H	3.97468	-1.41136	-0.46936
H	2.54090	-0.78946	-1.32688
H	-2.39606	-3.28582	0.12747
H	-1.85425	-2.49100	1.62854
H	2.84105	1.30436	-1.08582
H	4.08695	1.65122	0.14570
H	-2.83826	-0.49929	1.75190
H	-4.04218	-1.07937	0.56575
H	2.30172	2.31106	1.78040
H	2.32257	3.31632	0.29152
H	-3.59138	0.77325	-0.96583
H	-4.00110	1.39342	0.66223
H	-0.36947	3.67850	0.33152
H	0.14425	3.19173	1.98092
H	-1.31577	1.21853	1.92913

H	-2.22862	2.75146	1.72527
Rb	-0.21151	0.21452	-1.76842
C	0.61557	-1.46957	1.95109
H	1.42991	-0.76676	2.17598
H	0.68291	-2.33617	2.65385
H	-0.33716	-0.94531	2.13840

ACE3 Rb B97-1 def2tzv

40

O	2.91383	-0.02715	0.57155
O	-2.09611	-1.23029	-0.03501
O	0.82011	2.02887	0.30923
O	-2.03232	1.66122	0.00617
N	0.70436	-1.91358	0.54129
C	2.08252	-2.30167	0.14833
C	-0.31204	-2.91816	0.14776
C	2.91879	-1.12924	-0.38118
C	-1.74336	-2.53177	0.53147
C	3.12118	1.30212	0.01186
C	-3.09189	-0.49811	0.73592
C	2.20234	2.30267	0.69368
C	-3.26762	0.89411	0.13459
C	-0.16968	2.89308	0.95141
C	-1.42921	2.10061	1.26005
H	2.03636	-3.03946	-0.65945
H	2.60510	-2.78391	0.98868
H	-0.12217	-3.90127	0.61954
H	-0.25055	-3.06392	-0.93480
H	3.94655	-1.46862	-0.56306
H	2.50949	-0.78606	-1.33891
H	-2.43105	-3.29511	0.15152

H	-1.85363	-2.49508	1.61998
H	2.92766	1.29781	-1.06701
H	4.16047	1.61144	0.16862
H	-2.77569	-0.44574	1.78440
H	-4.06185	-1.00968	0.70205
H	2.30280	2.22720	1.78247
H	2.48778	3.31491	0.38234
H	-3.65013	0.81521	-0.88350
H	-3.99555	1.44771	0.73995
H	-0.39476	3.73685	0.29150
H	0.22581	3.28123	1.89651
H	-1.17549	1.24025	1.88707
H	-2.13372	2.74162	1.80428
Rb	-0.26324	0.20973	-1.73854
C	0.64196	-1.57947	1.98162
H	1.45826	-0.90157	2.21785
H	0.72677	-2.47916	2.61708
H	-0.29520	-1.07576	2.22120

ACE3 Rb CAM-B3LYP def2sv

40

O	2.81963	0.02838	0.54785
O	-2.02596	-1.22530	-0.02158
O	0.75065	1.91789	0.43058
O	-2.01673	1.58742	-0.03352
N	0.70398	-1.85807	0.54534
C	2.07759	-2.24225	0.23557
C	-0.27581	-2.87731	0.19438
C	2.91497	-1.08501	-0.30122
C	-1.70930	-2.48658	0.52829
C	3.01438	1.29984	-0.01116

C	-3.06460	-0.53780	0.63346
C	2.08971	2.29487	0.65728
C	-3.21830	0.84841	0.02829
C	-0.21191	2.79713	0.96652
C	-1.51376	2.06250	1.19488
H	2.07706	-3.02338	-0.54226
H	2.57218	-2.68707	1.12402
H	-0.08282	-3.84227	0.71701
H	-0.19587	-3.08293	-0.88742
H	3.96718	-1.41553	-0.41600
H	2.55498	-0.81535	-1.31355
H	-2.39114	-3.26438	0.13488
H	-1.85139	-2.45614	1.62332
H	2.80991	1.28242	-1.09950
H	4.06048	1.64013	0.11728
H	-2.84434	-0.47465	1.71652
H	-4.03061	-1.06951	0.52766
H	2.29271	2.32340	1.74536
H	2.29210	3.30290	0.24539
H	-3.56729	0.75997	-1.01256
H	-3.98906	1.40142	0.59818
H	-0.36833	3.65779	0.28694
H	0.12846	3.19184	1.94203
H	-1.33226	1.22957	1.89749
H	-2.23946	2.75543	1.66307
Rb	-0.18291	0.18762	-1.71422
C	0.58073	-1.40537	1.92220
H	1.39014	-0.69951	2.13866
H	0.63555	-2.25054	2.64607
H	-0.37118	-0.87690	2.07856

ACE3 Rb CAM-B3LYP def2tzv

40

O	2.85004	-0.03276	0.56690
O	-2.07804	-1.21268	-0.04137
O	0.81717	2.04379	0.28148
O	-2.00928	1.65151	-0.02095
N	0.68583	-1.91607	0.53457
C	2.05797	-2.30139	0.15979
C	-0.32668	-2.90857	0.13903
C	2.88998	-1.13925	-0.36591
C	-1.74575	-2.51385	0.51499
C	3.09271	1.28441	0.01736
C	-3.07624	-0.47804	0.70777
C	2.18964	2.28966	0.68743
C	-3.24356	0.89765	0.09534
C	-0.17471	2.89909	0.91277
C	-1.41819	2.10341	1.22401
H	2.02156	-3.04751	-0.63523
H	2.57005	-2.77081	1.00847
H	-0.14110	-3.88717	0.61109
H	-0.26270	-3.05738	-0.93899
H	3.92135	-1.46651	-0.52088
H	2.49979	-0.81149	-1.33230
H	-2.43974	-3.26294	0.13038
H	-1.86043	-2.47956	1.59893
H	2.91054	1.28534	-1.05947
H	4.13299	1.56954	0.18409
H	-2.76957	-0.41634	1.75423
H	-4.04030	-0.99117	0.67060
H	2.27060	2.20218	1.77268

H	2.49264	3.29676	0.39190
H	-3.61376	0.80981	-0.92231
H	-3.97260	1.46043	0.68318
H	-0.40673	3.73046	0.24671
H	0.21892	3.29954	1.84871
H	-1.15889	1.25184	1.85345
H	-2.12965	2.73642	1.76011
Rb	-0.23105	0.19657	-1.68676
C	0.60489	-1.55663	1.95745
H	1.41943	-0.88237	2.19261
H	0.67417	-2.44329	2.60577
H	-0.32808	-1.04411	2.17558

ACE3 Rb MP2 def2tzv

40

O	2.82029	-0.13926	0.60445
O	-2.12648	-1.10444	-0.06878
O	0.91074	2.04310	0.21781
O	-1.93913	1.74662	-0.07201
N	0.56788	-1.89468	0.52951
C	1.94750	-2.38873	0.24619
C	-0.46276	-2.89576	0.13881
C	2.84410	-1.28380	-0.31905
C	-1.88574	-2.44958	0.48704
C	3.16142	1.16147	0.00854
C	-3.12263	-0.33253	0.68998
C	2.30336	2.22683	0.66914
C	-3.23267	1.05390	0.05979
C	-0.05994	2.92067	0.90392
C	-1.31664	2.12302	1.20842
H	1.89821	-3.18484	-0.50729

H	2.40375	-2.82098	1.15191
H	-0.29789	-3.86435	0.65182
H	-0.37800	-3.06893	-0.93976
H	3.87152	-1.64888	-0.44558
H	2.46798	-0.96617	-1.29965
H	-2.60566	-3.15775	0.06116
H	-2.02959	-2.41988	1.57280
H	2.97406	1.14610	-1.07258
H	4.22129	1.38185	0.17921
H	-2.80671	-0.27556	1.73894
H	-4.10353	-0.82248	0.64583
H	2.34757	2.12228	1.75950
H	2.65763	3.22352	0.37957
H	-3.61015	0.98265	-0.96093
H	-3.91876	1.66613	0.65970
H	-0.28664	3.78072	0.26671
H	0.36683	3.27067	1.85168
H	-1.04928	1.22841	1.77907
H	-2.01742	2.73680	1.79004
Rb	-0.16993	0.16322	-1.66772
C	0.43475	-1.50915	1.96168
H	1.27728	-0.86940	2.21723
H	0.42902	-2.39520	2.62359
H	-0.48711	-0.94715	2.11744

ACE3 Rb MP2 def2tzv

40

O	-2.82029	0.13926	0.60445
O	2.12648	1.10444	-0.06878
O	-0.91074	-2.04310	0.21781
O	1.93913	-1.74662	-0.07201

N	-0.56788	1.89468	0.52951
C	-1.94750	2.38873	0.24619
C	0.46276	2.89576	0.13881
C	-2.84410	1.28380	-0.31905
C	1.88574	2.44958	0.48704
C	-3.16142	-1.16147	0.00854
C	3.12263	0.33253	0.68998
C	-2.30336	-2.22683	0.66914
C	3.23267	-1.05390	0.05979
C	0.05994	-2.92067	0.90392
C	1.31664	-2.12302	1.20842
H	-1.89821	3.18484	-0.50729
H	-2.40375	2.82098	1.15191
H	0.29789	3.86435	0.65182
H	0.37800	3.06893	-0.93976
H	-3.87152	1.64888	-0.44558
H	-2.46798	0.96617	-1.29965
H	2.60566	3.15775	0.06116
H	2.02959	2.41988	1.57280
H	-2.97406	-1.14610	-1.07258
H	-4.22129	-1.38185	0.17921
H	2.80671	0.27556	1.73894
H	4.10353	0.82248	0.64583
H	-2.34757	-2.12228	1.75950
H	-2.65763	-3.22352	0.37957
H	3.61015	-0.98265	-0.96093
H	3.91876	-1.66613	0.65970
H	0.28664	-3.78072	0.26671
H	-0.36683	-3.27067	1.85168
H	1.04928	-1.22841	1.77907

H	2.01742	-2.73680	1.79004
Rb	0.16993	-0.16322	-1.66772
C	-0.43475	1.50915	1.96168
H	-1.27728	0.86940	2.21723
H	-0.42902	2.39520	2.62359
H	0.48711	0.94715	2.11744

ACE3 Rb PBE0 def2sv

40

O	2.82622	0.04579	0.55202
O	-2.01060	-1.23656	-0.00624
O	0.73941	1.91902	0.43557
O	-2.02803	1.57639	-0.01662
N	0.72418	-1.85503	0.54690
C	2.09508	-2.22482	0.22301
C	-0.24978	-2.87392	0.19308
C	2.92109	-1.05724	-0.30530
C	-1.68175	-2.49376	0.53729
C	3.00662	1.31808	-0.00332
C	-3.04437	-0.56016	0.66059
C	2.07249	2.30481	0.66180
C	-3.21676	0.82411	0.05917
C	-0.22363	2.79260	0.97142
C	-1.52154	2.05518	1.20519
H	2.09480	-2.99602	-0.56664
H	2.60044	-2.68197	1.10135
H	-0.05269	-3.84594	0.70526
H	-0.17411	-3.07065	-0.89232
H	3.97512	-1.38340	-0.43190
H	2.55458	-0.78181	-1.31556
H	-2.36059	-3.27831	0.14783

H -1.81642 -2.46940 1.63554

H 2.80665 1.30144 -1.09400

H 4.04962 1.67158 0.12690

H -2.81413 -0.49647 1.74356

H -4.01020 -1.09766 0.56648

H 2.27613 2.33866 1.75132

H 2.27171 3.31451 0.24835

H -3.57622 0.73250 -0.97933

H -3.99149 1.36688 0.63633

H -0.38558 3.65437 0.29199

H 0.11485 3.19169 1.94752

H -1.33471 1.22537 1.91271

H -2.24626 2.74906 1.67704

Rb -0.20604 0.18935 -1.74115

C 0.61033 -1.42131 1.92666

H 1.41581 -0.70881 2.14396

H 0.67958 -2.27324 2.64374

H -0.34543 -0.90163 2.09858

ACE3 Rb PBE0 def2tzv

40

O 2.86279 -0.02301 0.57530

O -2.07137 -1.22031 -0.03685

O 0.80980 2.03577 0.28928

O -2.01996 1.64883 -0.00407

N 0.69741 -1.90658 0.53153

C 2.06771 -2.28897 0.15362

C -0.30902 -2.90310 0.13936

C 2.90025 -1.12264 -0.36161

C -1.72781 -2.51529 0.52029

C 3.08958 1.29183 0.01948

C	-3.06250	-0.49555	0.72583
C	2.17962	2.29563	0.68356
C	-3.24299	0.88366	0.12532
C	-0.17674	2.88103	0.93534
C	-1.42135	2.08435	1.24018
H	2.03141	-3.02698	-0.65310
H	2.58177	-2.77234	0.99760
H	-0.12097	-3.88520	0.61164
H	-0.24683	-3.05287	-0.94175
H	3.93304	-1.45447	-0.52246
H	2.50969	-0.79062	-1.33027
H	-2.41894	-3.27549	0.14289
H	-1.83577	-2.48173	1.60862
H	2.90724	1.28772	-1.06074
H	4.12939	1.59350	0.18215
H	-2.74810	-0.43910	1.77400
H	-4.03029	-1.00948	0.69646
H	2.27084	2.22207	1.77251
H	2.47941	3.30369	0.37562
H	-3.62857	0.79936	-0.89024
H	-3.97380	1.43474	0.72800
H	-0.41101	3.72850	0.28487
H	0.21728	3.26695	1.88096
H	-1.15993	1.22480	1.86356
H	-2.12736	2.71668	1.79082
Rb	-0.24963	0.20424	-1.71436
C	0.62337	-1.56515	1.95665
H	1.43579	-0.88514	2.19571
H	0.70379	-2.45950	2.59844
H	-0.31446	-1.06101	2.18772

ACE3 Rb PBE def2sv

40

O	2.87782	0.03037	0.55601
O	-2.03835	-1.24859	-0.00952
O	0.75771	1.92186	0.44370
O	-2.04307	1.60064	-0.00284
N	0.72805	-1.87285	0.54560
C	2.10409	-2.24678	0.19702
C	-0.26027	-2.89929	0.20460
C	2.93805	-1.07067	-0.33101
C	-1.69911	-2.51176	0.55562
C	3.04735	1.32195	0.00311
C	-3.07192	-0.55997	0.67727
C	2.10524	2.30693	0.68189
C	-3.24393	0.83774	0.08155
C	-0.21045	2.80042	1.00308
C	-1.51657	2.05846	1.23711
H	2.08904	-3.01084	-0.61140
H	2.62361	-2.72483	1.06831
H	-0.06500	-3.87605	0.73098
H	-0.18750	-3.10984	-0.88694
H	3.99329	-1.41468	-0.48754
H	2.55191	-0.76945	-1.33732
H	-2.38521	-3.31067	0.18087
H	-1.82677	-2.46952	1.66354
H	2.84378	1.31151	-1.09657
H	4.09695	1.68677	0.13457
H	-2.82874	-0.50287	1.76766
H	-4.05236	-1.09309	0.59114
H	2.30618	2.32857	1.78213

H	2.31151	3.32975	0.27772
H	-3.61189	0.75666	-0.96439
H	-4.02028	1.38057	0.67480
H	-0.37467	3.68087	0.33208
H	0.13648	3.18697	1.99194
H	-1.32445	1.20741	1.93289
H	-2.23972	2.75231	1.73375
Rb	-0.22720	0.20907	-1.77412
C	0.63858	-1.44425	1.94074
H	1.45404	-0.72666	2.14667
H	0.72254	-2.30692	2.65882
H	-0.32207	-0.92273	2.13473

ACE3 Rb PBE def2tzv

40

O	2.93013	-0.00470	0.58407
O	-2.08442	-1.25411	-0.03851
O	0.79898	2.02933	0.30509
O	-2.05809	1.65246	0.00512
N	0.72956	-1.90822	0.53344
C	2.11007	-2.28444	0.13148
C	-0.28054	-2.92805	0.15712
C	2.93910	-1.10449	-0.38743
C	-1.70964	-2.55276	0.54870
C	3.11676	1.33586	0.01559
C	-3.08568	-0.52910	0.75126
C	2.18652	2.32410	0.69402
C	-3.28689	0.85766	0.15256
C	-0.19900	2.88945	0.96468
C	-1.45051	2.08881	1.27072
H	2.06385	-3.01690	-0.69051

H	2.64093	-2.78138	0.96871
H	-0.07932	-3.91193	0.64267
H	-0.22208	-3.08925	-0.93030
H	3.97478	-1.44099	-0.57747
H	2.52501	-0.74930	-1.34722
H	-2.39826	-3.33533	0.18850
H	-1.80930	-2.49724	1.64499
H	2.92202	1.32327	-1.07036
H	4.15859	1.66526	0.16913
H	-2.75404	-0.47438	1.80287
H	-4.05733	-1.05495	0.73238
H	2.28642	2.25388	1.79087
H	2.46361	3.34598	0.37932
H	-3.68114	0.77605	-0.86851
H	-4.02229	1.40312	0.77001
H	-0.43032	3.74617	0.31091
H	0.20110	3.27173	1.91856
H	-1.19041	1.21969	1.89578
H	-2.16243	2.72607	1.82517
Rb	-0.27956	0.21292	-1.75196
C	0.67788	-1.57241	1.97509
H	1.48972	-0.87515	2.20052
H	0.78678	-2.47481	2.61659
H	-0.27055	-1.08169	2.22685

ACE3 Rb B2PLYP def2sv

40

O	2.82722	0.02837	0.55623
O	-2.02652	-1.22534	-0.02070
O	0.75166	1.91464	0.43472
O	-2.01992	1.59222	-0.02890

N	0.70499	-1.85283	0.54411
C	2.08246	-2.24371	0.23588
C	-0.27338	-2.87978	0.19343
C	2.91941	-1.08490	-0.30353
C	-1.70869	-2.49218	0.53273
C	3.02081	1.30444	-0.00967
C	-3.06766	-0.53925	0.64568
C	2.09528	2.29811	0.66409
C	-3.22592	0.84802	0.03893
C	-0.21186	2.79827	0.97912
C	-1.51338	2.05935	1.20841
H	2.08032	-3.02520	-0.54237
H	2.57664	-2.68795	1.12508
H	-0.07616	-3.84534	0.71497
H	-0.19527	-3.08237	-0.88913
H	3.97127	-1.41532	-0.42493
H	2.55249	-0.81219	-1.31209
H	-2.39124	-3.26990	0.14003
H	-1.84809	-2.46137	1.62777
H	2.81140	1.28579	-1.09659
H	4.06738	1.64494	0.11538
H	-2.84054	-0.47560	1.72696
H	-4.03314	-1.07316	0.54445
H	2.29881	2.32354	1.75193
H	2.29430	3.30773	0.25411
H	-3.57551	0.75861	-1.00135
H	-3.99545	1.40074	0.61098
H	-0.36977	3.66117	0.30305
H	0.13144	3.18808	1.95569
H	-1.32719	1.22158	1.90330

H -2.23935 2.74741 1.68370

Rb -0.18808 0.19115 -1.73310

C 0.58398 -1.41749 1.93183

H 1.39638 -0.71812 2.15620

H 0.63543 -2.27263 2.64429

H -0.36599 -0.88762 2.09223

ACE3 Rb B2PLYP def2tzv

40

O 2.90440 -0.10878 0.58432

O -2.11755 -1.18207 -0.02552

O 0.88765 2.02909 0.25254

O -1.99167 1.68616 -0.00502

N 0.64547 -1.91686 0.51097

C 2.01525 -2.34480 0.12718

C -0.38513 -2.91401 0.13376

C 2.87562 -1.19438 -0.39506

C -1.79969 -2.50229 0.53330

C 3.17482 1.22844 0.05417

C -3.09569 -0.43054 0.75955

C 2.26240 2.23401 0.72608

C -3.25149 0.95715 0.15346

C -0.10538 2.90922 0.88704

C -1.35154 2.12191 1.23793

H 1.94982 -3.07973 -0.67863

H 2.51961 -2.83689 0.96987

H -0.19717 -3.89156 0.61150

H -0.33669 -3.06464 -0.94595

H 3.89156 -1.55105 -0.59087

H 2.46409 -0.82057 -1.33631

H -2.51220 -3.23861 0.15516

H -1.89842 -2.46268 1.61955

H 3.01850 1.24781 -1.02735

H 4.21480 1.49353 0.25690

H -2.76075 -0.38008 1.79871

H -4.06894 -0.92964 0.73864

H 2.29427 2.10473 1.81036

H 2.59234 3.24570 0.47501

H -3.65481 0.88573 -0.85343

H -3.94228 1.53759 0.77180

H -0.34446 3.72283 0.20144

H 0.30635 3.32833 1.80788

H -1.08246 1.26055 1.85110

H -2.04314 2.75898 1.79727

Rb -0.26116 0.21935 -1.71364

C 0.58544 -1.56681 1.94946

H 1.42245 -0.91723 2.17901

H 0.63563 -2.46272 2.58944

H -0.33375 -1.03110 2.17526

ACE3 Rb B2PLYP def2tzv

40

O -2.86827 0.06831 0.58171

O 2.10525 1.18598 -0.04512

O -0.85175 -2.04330 0.26370

O 1.99620 -1.68779 -0.02815

N -0.65533 1.90864 0.53460

C -2.03344 2.33343 0.17744

C 0.36718 2.91115 0.14883

C -2.88762 1.18537 -0.36186

C 1.79060 2.50246 0.52304

C -3.13279 -1.25355 0.01463

C	3.10453	0.43620	0.71516
C	-2.23724	-2.27493	0.68601
C	3.25457	-0.94816	0.09947
C	0.13578	-2.91074	0.91921
C	1.38634	-2.11441	1.23316
H	-1.98627	3.08944	-0.60994
H	-2.53150	2.79845	1.03862
H	0.18457	3.88499	0.63611
H	0.30129	3.06984	-0.92890
H	-3.91466	1.52827	-0.51977
H	-2.49346	0.85054	-1.32520
H	2.49179	3.24393	0.13367
H	1.90909	2.46139	1.60730
H	-2.94839	-1.24788	-1.06273
H	-4.17784	-1.52387	0.18294
H	2.79186	0.37935	1.76096
H	4.07434	0.94067	0.67581
H	-2.30636	-2.18077	1.77236
H	-2.55178	-3.28030	0.39292
H	3.63055	-0.87025	-0.91768
H	3.96606	-1.52551	0.69694
H	0.36586	-3.75159	0.26327
H	-0.27200	-3.29286	1.85809
H	1.12510	-1.24724	1.84145
H	2.09067	-2.74330	1.78585
Rb	0.22686	-0.18797	-1.70122
C	-0.57165	1.54706	1.96904
H	-1.39754	0.88513	2.20422
H	-0.62367	2.43665	2.61747
H	0.35676	1.02028	2.17744

ACE3 Rb DSDPBEP86 def2sv

40

O	2.76975	0.04594	0.55359
O	-2.01878	-1.21183	-0.05893
O	0.72347	1.90229	0.43560
O	-2.00229	1.59544	-0.10526
N	0.67725	-1.82081	0.54745
C	2.05966	-2.23132	0.30241
C	-0.28673	-2.86489	0.21682
C	2.90999	-1.09515	-0.25799
C	-1.72439	-2.46781	0.52448
C	2.97032	1.29903	-0.05289
C	-3.07639	-0.51678	0.56561
C	2.06378	2.30968	0.61847
C	-3.21050	0.85699	-0.07255
C	-0.23593	2.79800	0.95764
C	-1.54553	2.06568	1.14733
H	2.07756	-3.04807	-0.44182
H	2.52367	-2.63654	1.22812
H	-0.09023	-3.81233	0.77400
H	-0.19215	-3.10153	-0.85990
H	3.97056	-1.41810	-0.32630
H	2.57857	-0.86843	-1.29219
H	-2.40709	-3.24926	0.13421
H	-1.88158	-2.41396	1.61838
H	2.73214	1.25278	-1.13573
H	4.02476	1.63210	0.03702
H	-2.87956	-0.43414	1.65372
H	-4.04032	-1.05323	0.44523
H	2.29822	2.36707	1.70087

H	2.24001	3.31028	0.17265
H	-3.51783	0.74534	-1.12612
H	-4.00269	1.42330	0.45760
H	-0.36920	3.66410	0.27719
H	0.08945	3.18260	1.94500
H	-1.38323	1.22875	1.85169
H	-2.28939	2.75651	1.59444
Rb	-0.10857	0.13973	-1.65098
C	0.51078	-1.32770	1.90885
H	1.31674	-0.61701	2.12956
H	0.53535	-2.15375	2.65848
H	-0.44495	-0.79005	2.00889

ACE3 Rb DSDPBEP86 def2tzv

40

O	-2.79554	0.10351	0.59000
O	2.11647	1.12992	-0.07764
O	-0.87494	-2.04696	0.23014
O	1.95450	-1.72661	-0.09544
N	-0.58733	1.89594	0.52680
C	-1.96396	2.36457	0.22828
C	0.43838	2.89527	0.14651
C	-2.85193	1.24527	-0.31721
C	1.85761	2.45212	0.49655
C	-3.12148	-1.19094	0.00011
C	3.11818	0.35980	0.65265
C	-2.25926	-2.25036	0.65780
C	3.22954	-1.01785	0.01254
C	0.09985	-2.91287	0.89844
C	1.35983	-2.11830	1.18162
H	-1.92295	3.14620	-0.53889

H	-2.42616	2.81195	1.12244
H	0.27234	3.86278	0.65842
H	0.36042	3.07525	-0.93090
H	-3.88523	1.59926	-0.42257
H	-2.49768	0.93956	-1.30975
H	2.57537	3.17540	0.09421
H	1.99236	2.40579	1.58237
H	-2.93390	-1.17549	-1.08065
H	-4.17956	-1.42306	0.16475
H	2.82406	0.29248	1.70718
H	4.09734	0.85169	0.59963
H	-2.32189	-2.16178	1.74849
H	-2.60276	-3.24666	0.35512
H	3.58468	-0.92990	-1.01470
H	3.94309	-1.62086	0.58797
H	0.31730	-3.77508	0.26084
H	-0.30801	-3.26548	1.85281
H	1.10585	-1.23203	1.77123
H	2.06964	-2.73799	1.74427
Rb	0.14945	-0.15285	-1.62622
C	-0.46734	1.49778	1.94781
H	-1.30568	0.84988	2.19328
H	-0.47360	2.37586	2.61897
H	0.45597	0.94007	2.11111

ACE3 Rb HSE06 def2sv

40

O	2.82532	0.04725	0.55239
O	-2.01046	-1.23788	-0.00759
O	0.73743	1.91943	0.43226
O	-2.02936	1.57465	-0.02261

N	0.72439	-1.85320	0.54387
C	2.09591	-2.22417	0.22320
C	-0.24880	-2.87439	0.19382
C	2.92350	-1.05716	-0.30388
C	-1.68052	-2.49437	0.53882
C	3.00599	1.31972	-0.00391
C	-3.04525	-0.56064	0.65814
C	2.07078	2.30635	0.65960
C	-3.21866	0.82193	0.05348
C	-0.22703	2.79406	0.96574
C	-1.52457	2.05619	1.19961
H	2.09683	-2.99604	-0.56574
H	2.59891	-2.68085	1.10324
H	-0.04988	-3.84455	0.70890
H	-0.17391	-3.07437	-0.89102
H	3.97796	-1.38296	-0.42691
H	2.55992	-0.78233	-1.31543
H	-2.35986	-3.27986	0.15246
H	-1.81375	-2.46665	1.63722
H	2.80671	1.30171	-1.09474
H	4.04874	1.67355	0.12667
H	-2.81433	-0.49414	1.74085
H	-4.01031	-1.09964	0.56592
H	2.27287	2.34143	1.74931
H	2.26944	3.31583	0.24551
H	-3.57721	0.72806	-0.98504
H	-3.99364	1.36585	0.62908
H	-0.38860	3.65429	0.28440
H	0.11077	3.19521	1.94118
H	-1.33816	1.22750	1.90859

H	-2.25069	2.75007	1.66914
Rb	-0.20361	0.18670	-1.73339
C	0.60909	-1.41383	1.92193
H	1.41312	-0.69877	2.13609
H	0.67996	-2.26295	2.64224
H	-0.34802	-0.89583	2.09136

ACE3 Rb HSE06 def2tzv

40

O	2.85741	-0.02321	0.57622
O	-2.07331	-1.21896	-0.03752
O	0.80977	2.04086	0.28014
O	-2.01879	1.65039	-0.01596
N	0.69422	-1.90542	0.53072
C	2.06470	-2.29023	0.15535
C	-0.31308	-2.90385	0.14476
C	2.89976	-1.12545	-0.35908
C	-1.73085	-2.51309	0.52550
C	3.08960	1.29185	0.02021
C	-3.06543	-0.48932	0.72133
C	2.17964	2.29698	0.68125
C	-3.24414	0.88665	0.11395
C	-0.17891	2.88847	0.92264
C	-1.42295	2.09205	1.22880
H	2.02847	-3.02887	-0.65034
H	2.57637	-2.77340	1.00062
H	-0.12466	-3.88314	0.62205
H	-0.25203	-3.05955	-0.93525
H	3.93305	-1.45720	-0.51395
H	2.51428	-0.79484	-1.32996
H	-2.42365	-3.27325	0.15231

H	-1.83776	-2.47379	1.61344
H	2.91090	1.28715	-1.06023
H	4.12930	1.59057	0.18626
H	-2.75139	-0.42814	1.76901
H	-4.03272	-1.00346	0.69380
H	2.26517	2.22166	1.77017
H	2.48243	3.30458	0.37618
H	-3.62725	0.79863	-0.90184
H	-3.97493	1.44159	0.71239
H	-0.41258	3.73269	0.26848
H	0.21437	3.27826	1.86656
H	-1.16255	1.23527	1.85592
H	-2.13107	2.72545	1.77475
Rb	-0.24280	0.19425	-1.70342
C	0.61948	-1.55375	1.95349
H	1.43158	-0.87216	2.18762
H	0.69960	-2.44344	2.60131
H	-0.31845	-1.04837	2.18011

ACE3 Rb M062X def2sv

40

O	2.78186	0.06033	0.55430
O	-2.00220	-1.22001	-0.04843
O	0.70267	1.87036	0.46898
O	-2.00313	1.56181	-0.11615
N	0.68321	-1.80273	0.54068
C	2.06943	-2.21370	0.33330
C	-0.26902	-2.86265	0.22920
C	2.92813	-1.08932	-0.23598
C	-1.70831	-2.47098	0.53385
C	2.95182	1.30794	-0.05954

C	-3.07352	-0.53625	0.55382
C	2.03468	2.30550	0.61672
C	-3.20972	0.83054	-0.09731
C	-0.25835	2.77982	0.95113
C	-1.57776	2.06310	1.12986
H	2.10270	-3.04733	-0.38775
H	2.51400	-2.59181	1.27776
H	-0.05917	-3.79345	0.80357
H	-0.17345	-3.11720	-0.84177
H	3.98631	-1.41264	-0.28692
H	2.60648	-0.87697	-1.27571
H	-2.38914	-3.25122	0.14546
H	-1.86359	-2.41295	1.62702
H	2.70231	1.24954	-1.13840
H	3.99812	1.66073	0.01534
H	-2.89013	-0.44336	1.64291
H	-4.02623	-1.08543	0.42509
H	2.28816	2.38073	1.69211
H	2.17681	3.30231	0.15666
H	-3.50388	0.70680	-1.15175
H	-4.00590	1.40041	0.41768
H	-0.37292	3.62654	0.24633
H	0.05324	3.18622	1.93184
H	-1.44259	1.24611	1.86209
H	-2.32605	2.77097	1.53479
Rb	-0.09421	0.13670	-1.64823
C	0.48106	-1.25170	1.87336
H	1.27405	-0.52537	2.08436
H	0.49179	-2.04539	2.65486
H	-0.48174	-0.71980	1.92439

ACE3 Rb M062X def2tzv

40

O	2.89130	0.06344	0.55539
O	-2.02104	-1.24737	-0.04079
O	0.71065	1.92091	0.37770
O	-2.04802	1.58403	-0.13539
N	0.71358	-1.79891	0.52052
C	2.10995	-2.21538	0.28846
C	-0.24725	-2.88438	0.24257
C	2.96252	-1.09997	-0.30125
C	-1.68001	-2.50843	0.59030
C	3.00168	1.35710	-0.07682
C	-3.10267	-0.53567	0.60342
C	2.07957	2.32426	0.62832
C	-3.26920	0.80517	-0.08668
C	-0.28418	2.82630	0.91918
C	-1.56762	2.06359	1.14449
H	2.12580	-3.04331	-0.42276
H	2.56287	-2.58081	1.21953
H	-0.00726	-3.79158	0.81964
H	-0.17904	-3.14149	-0.81589
H	3.99887	-1.43108	-0.40112
H	2.59355	-0.84524	-1.29831
H	-2.35864	-3.28971	0.24387
H	-1.79930	-2.41196	1.67160
H	2.72462	1.29061	-1.13262
H	4.02951	1.71919	-0.01676
H	-2.87399	-0.41592	1.66635
H	-4.03720	-1.09565	0.51907
H	2.27105	2.31273	1.70389

H	2.24995	3.33385	0.24575
H	-3.55040	0.65317	-1.12607
H	-4.05796	1.37213	0.41376
H	-0.44100	3.65327	0.22400
H	0.05702	3.22653	1.87689
H	-1.37490	1.22600	1.81563
H	-2.31486	2.72062	1.59666
Rb	-0.15212	0.11790	-1.62240
C	0.54048	-1.22815	1.86646
H	1.30962	-0.48176	2.02609
H	0.61015	-2.00486	2.64370
H	-0.42763	-0.73935	1.94649

ACE3 Rb M06 def2sv

40

O	2.89366	0.06997	0.53360
O	-2.01426	-1.21121	0.00228
O	0.71545	1.85235	0.47061
O	-2.02867	1.57460	-0.06955
N	0.71265	-1.76659	0.56975
C	2.08398	-2.17758	0.29575
C	-0.25299	-2.81651	0.28382
C	2.93660	-1.06431	-0.28507
C	-1.68211	-2.43526	0.61153
C	2.97126	1.33021	-0.06763
C	-3.09413	-0.53355	0.58592
C	2.04190	2.28453	0.63784
C	-3.22531	0.83317	-0.05189
C	-0.24569	2.72205	1.01232
C	-1.54950	1.99095	1.18400
H	2.08237	-3.00089	-0.44394

H	2.56350	-2.59062	1.21448
H	-0.03057	-3.75413	0.85342
H	-0.17904	-3.07976	-0.79182
H	3.97885	-1.42894	-0.42014
H	2.56266	-0.81222	-1.30287
H	-2.36134	-3.24446	0.26833
H	-1.81885	-2.35196	1.71074
H	2.68285	1.26969	-1.14112
H	4.00519	1.73500	-0.03555
H	-2.93759	-0.45011	1.68436
H	-4.04971	-1.08374	0.44234
H	2.29279	2.32635	1.72125
H	2.18053	3.30594	0.22039
H	-3.52329	0.72243	-1.11095
H	-4.03149	1.39551	0.46635
H	-0.37716	3.61148	0.35713
H	0.08038	3.08623	2.01023
H	-1.37614	1.12329	1.85486
H	-2.28688	2.65974	1.67867
Rb	-0.16260	0.12404	-1.73796
C	0.58179	-1.24432	1.91734
H	1.37052	-0.49940	2.09854
H	0.66257	-2.05224	2.68684
H	-0.38696	-0.73245	2.05571

ACE3 Rb M06 def2tzv

40

O	2.96073	0.01200	0.52256
O	-2.06468	-1.18569	0.00329
O	0.77980	1.89541	0.35405
O	-2.00885	1.63961	-0.10694

N	0.68618	-1.77334	0.55737
C	2.05476	-2.21999	0.25093
C	-0.30977	-2.82882	0.31750
C	2.92064	-1.12898	-0.35277
C	-1.72548	-2.41900	0.67331
C	3.06517	1.30941	-0.08766
C	-3.13608	-0.44823	0.61861
C	2.14775	2.26695	0.62478
C	-3.24693	0.89970	-0.06081
C	-0.19065	2.77904	0.95754
C	-1.47471	2.02749	1.17453
H	2.01062	-3.03018	-0.48238
H	2.53834	-2.63179	1.15062
H	-0.08700	-3.73734	0.90634
H	-0.26247	-3.11123	-0.73848
H	3.93138	-1.51458	-0.52775
H	2.51033	-0.83512	-1.32654
H	-2.41777	-3.21281	0.37568
H	-1.82644	-2.28344	1.75541
H	2.79356	1.25934	-1.14883
H	4.09375	1.67729	-0.02493
H	-2.93802	-0.34009	1.69187
H	-4.09084	-0.97491	0.51060
H	2.33053	2.22766	1.70518
H	2.34216	3.28737	0.27525
H	-3.53666	0.76872	-1.10313
H	-4.02299	1.48605	0.44380
H	-0.34961	3.65072	0.31465
H	0.17238	3.12647	1.93116
H	-1.26679	1.14447	1.78613

H	-2.19309	2.66242	1.70534
Rb	-0.21214	0.08043	-1.68199
C	0.60124	-1.21665	1.91210
H	1.37028	-0.45791	2.02637
H	0.73790	-1.99884	2.67896
H	-0.36539	-0.74097	2.07486

ACE3 Rb MP2 def2sv

40

O	2.76615	0.06546	0.56258
O	-1.99413	-1.22179	-0.04364
O	0.70744	1.90309	0.44024
O	-2.01351	1.57596	-0.08304
N	0.70015	-1.81317	0.54289
C	2.08464	-2.21490	0.29565
C	-0.25316	-2.86441	0.20624
C	2.92072	-1.06818	-0.25942
C	-1.69016	-2.48296	0.52768
C	2.95946	1.32294	-0.04177
C	-3.05859	-0.54626	0.59501
C	2.04539	2.32100	0.63389
C	-3.21599	0.82503	-0.03898
C	-0.25591	2.79188	0.97277
C	-1.55462	2.04584	1.17046
H	2.10861	-3.02750	-0.45322
H	2.55256	-2.62128	1.21857
H	-0.04362	-3.81519	0.75310
H	-0.16421	-3.08905	-0.87316
H	3.98507	-1.37743	-0.33343
H	2.58120	-0.83993	-1.28958
H	-2.37073	-3.26489	0.13466

H	-1.84013	-2.44055	1.62237
H	2.71921	1.27994	-1.12343
H	4.01167	1.66274	0.04831
H	-2.85363	-0.46412	1.68099
H	-4.01530	-1.09648	0.48057
H	2.27539	2.36968	1.71714
H	2.21575	3.32665	0.19764
H	-3.53213	0.71044	-1.08904
H	-4.00882	1.38267	0.49905
H	-0.40264	3.65858	0.29656
H	0.07342	3.17614	1.95872
H	-1.37590	1.20924	1.87000
H	-2.30312	2.72626	1.62571
Rb	-0.13033	0.15261	-1.68768
C	0.53462	-1.35302	1.91630
H	1.34372	-0.65256	2.15380
H	0.55644	-2.19741	2.64434
H	-0.41764	-0.81378	2.02802

ACE3 Rb SCS-MP2 def2sv

40

O	2.77465	0.06881	0.56485
O	-1.99707	-1.23058	-0.04213
O	0.70808	1.91793	0.44009
O	-2.02378	1.57656	-0.06762
N	0.70991	-1.82707	0.54846
C	2.09954	-2.22039	0.28814
C	-0.24573	-2.87630	0.19313
C	2.92822	-1.06256	-0.26629
C	-1.68887	-2.50060	0.51571
C	2.96787	1.33253	-0.03451

C	-3.05972	-0.55840	0.61027
C	2.04962	2.33104	0.64396
C	-3.22623	0.82022	-0.01550
C	-0.25904	2.80713	0.97450
C	-1.55798	2.05597	1.18367
H	2.12338	-3.02859	-0.46676
H	2.57673	-2.62971	1.20543
H	-0.03661	-3.83603	0.72514
H	-0.15428	-3.08333	-0.89043
H	3.99464	-1.36578	-0.34659
H	2.58166	-0.83017	-1.29364
H	-2.36597	-3.27905	0.10789
H	-1.84367	-2.47376	1.61069
H	2.73178	1.29499	-1.11771
H	4.02060	1.67119	0.06289
H	-2.84637	-0.48158	1.69543
H	-4.01625	-1.11096	0.49870
H	2.27368	2.37270	1.72935
H	2.22415	3.33924	0.21350
H	-3.55239	0.71120	-1.06404
H	-4.01499	1.37387	0.53372
H	-0.41226	3.66960	0.29328
H	0.07446	3.19769	1.95717
H	-1.37432	1.22281	1.88716
H	-2.30660	2.73805	1.63778
Rb	-0.14054	0.16286	-1.70381
C	0.54787	-1.39096	1.93504
H	1.35979	-0.69659	2.18564
H	0.56910	-2.24889	2.64807
H	-0.40383	-0.85117	2.05918

ACE3 Rb SCS-MP2 def2tzv

40

O	-2.83766	0.10445	0.60126
O	2.11600	1.13547	-0.05503
O	-0.88382	-2.04847	0.23112
O	1.96582	-1.72378	-0.05899
N	-0.60460	1.89372	0.53559
C	-1.99058	2.36776	0.23359
C	0.41975	2.90416	0.14121
C	-2.87039	1.24264	-0.32984
C	1.85084	2.47693	0.49900
C	-3.15338	-1.20568	0.01131
C	3.11862	0.37662	0.70799
C	-2.27461	-2.25577	0.67891
C	3.24907	-1.01314	0.07836
C	0.09423	-2.93130	0.90022
C	1.35169	-2.13116	1.21606
H	-1.94572	3.15732	-0.52782
H	-2.46140	2.80281	1.13083
H	0.24180	3.87666	0.64263
H	0.33983	3.06605	-0.94027
H	-3.90278	1.59353	-0.46318
H	-2.48589	0.92445	-1.30739
H	2.56217	3.19573	0.07428
H	1.99114	2.45233	1.58569
H	-2.96678	-1.19360	-1.07019
H	-4.20977	-1.44428	0.18423
H	2.80224	0.31675	1.75700
H	4.09430	0.87865	0.66458
H	-2.32443	-2.15034	1.76948

H	-2.61017	-3.26000	0.39005
H	3.63014	-0.93629	-0.94155
H	3.94260	-1.61579	0.68040
H	0.32400	-3.77919	0.24649
H	-0.32733	-3.30197	1.84297
H	1.08398	-1.25039	1.80888
H	2.05834	-2.75520	1.78043
Rb	0.18909	-0.16240	-1.68903
C	-0.48148	1.52774	1.97657
H	-1.32112	0.88450	2.23533
H	-0.48823	2.42265	2.62706
H	0.44362	0.97422	2.14805

ACE4 Cs B3LYP def2sv

47

O	0.40646	-2.05478	1.11210
O	1.98135	1.81288	0.27412
O	-2.14188	-2.14666	-0.25069
O	-0.80822	2.44689	0.62232
O	-2.93020	0.63103	-0.03581
N	2.89833	-1.00224	-0.16678
C	2.78513	-1.69169	1.12610
C	3.73452	0.19655	-0.13256
C	1.46398	-1.47847	1.85958
C	3.22593	1.33082	0.74539
C	-0.82160	-2.12921	1.80890
C	1.51013	2.93469	0.99511
C	-1.84985	-2.84816	0.95099
C	0.19592	3.42253	0.42273
C	-3.46878	-1.67664	-0.42281
C	-2.10846	2.87170	0.26139

C	-3.78754	-0.41752	0.36876
C	-3.12341	1.83131	0.68667
H	3.60813	-1.39637	1.80919
H	2.89725	-2.77775	0.96679
H	3.82229	0.57938	-1.16439
H	4.77022	-0.03994	0.21007
H	1.53264	-1.96832	2.85314
H	1.26446	-0.40176	2.03237
H	3.13479	1.01198	1.80367
H	3.98038	2.14394	0.72087
H	-1.18018	-1.11324	2.07564
H	-0.69319	-2.69859	2.75352
H	2.24494	3.76424	0.93967
H	1.37389	2.67509	2.06521
H	-1.45318	-3.84370	0.67756
H	-2.76523	-3.00287	1.55086
H	-0.07691	4.36636	0.93720
H	0.31094	3.65005	-0.65802
H	-4.20163	-2.46199	-0.15310
H	-3.57877	-1.46878	-1.50079
H	-2.17261	3.04625	-0.83325
H	-2.35684	3.82624	0.76839
H	-4.84927	-0.14683	0.18903
H	-3.67625	-0.60225	1.45743
H	-3.02309	1.64298	1.77521
H	-4.14133	2.23343	0.50488
C	3.30581	-1.89457	-1.24739
H	2.60892	-2.74675	-1.31880
H	4.33558	-2.30106	-1.11445
H	3.28146	-1.35555	-2.21113

Cs	-0.06757	-0.03312	-1.35060

ACE4 Cs B3LYP def2tzv

47

O	0.43859	-2.17265	1.06454
O	1.99925	1.86459	0.22705
O	-2.14130	-2.16142	-0.31088
O	-0.82715	2.48182	0.63622
O	-2.97337	0.64998	-0.08924
N	2.90865	-0.98894	-0.20387
C	2.82776	-1.72313	1.08491
C	3.76120	0.21895	-0.15602
C	1.51429	-1.54991	1.83787
C	3.25828	1.33477	0.74583
C	-0.83051	-2.24075	1.78286
C	1.50666	3.00130	0.99971
C	-1.85065	-2.93353	0.90146
C	0.20532	3.48943	0.40972
C	-3.51212	-1.67910	-0.46725
C	-2.16300	2.91172	0.23104
C	-3.82630	-0.44577	0.35794
C	-3.16707	1.87654	0.67857
H	3.64293	-1.42147	1.75533
H	2.96315	-2.78956	0.89585
H	3.84259	0.61022	-1.17076
H	4.78349	-0.03221	0.17675
H	1.59160	-2.04337	2.81254
H	1.27846	-0.49512	2.00245
H	3.11499	0.99354	1.77516
H	4.00784	2.13258	0.76097
H	-1.16313	-1.23375	2.05064

H	-0.70250	-2.82034	2.70347
H	2.23977	3.81362	0.96761
H	1.35876	2.70526	2.04231
H	-1.46227	-3.90039	0.58091
H	-2.76496	-3.09994	1.47534
H	-0.07788	4.42482	0.90196
H	0.31570	3.67956	-0.66197
H	-4.22134	-2.47116	-0.21236
H	-3.61388	-1.45126	-1.52652
H	-2.20147	3.04265	-0.85441
H	-2.40393	3.86764	0.70608
H	-4.87964	-0.18317	0.21269
H	-3.66501	-0.62726	1.42508
H	-3.04056	1.66912	1.74492
H	-4.17861	2.25994	0.51351
C	3.29484	-1.86461	-1.33078
H	2.60243	-2.70176	-1.40193
H	4.31427	-2.26780	-1.22599
H	3.24944	-1.30436	-2.26494
Cs	-0.06129	-0.02013	-1.26244

ACE4 Cs B97-1 def2sv

47

O	0.35769	-2.03518	1.14009
O	2.01869	1.78746	0.25257
O	-2.17409	-2.13047	-0.25304
O	-0.75914	2.45253	0.62599
O	-2.90376	0.66412	-0.04036
N	2.85842	-1.04699	-0.15173
C	2.73823	-1.70539	1.15763
C	3.72972	0.12789	-0.14953

C	1.41450	-1.45329	1.88006
C	3.25709	1.28996	0.71716
C	-0.87825	-2.06192	1.82245
C	1.56903	2.91379	0.97601
C	-1.90729	-2.79617	0.97215
C	0.25233	3.41508	0.41324
C	-3.48585	-1.63157	-0.44020
C	-2.05075	2.89255	0.25995
C	-3.78924	-0.36356	0.34990
C	-3.08294	1.86391	0.68274
H	3.56508	-1.40345	1.83529
H	2.83547	-2.79836	1.02483
H	3.81305	0.49159	-1.19064
H	4.76443	-0.13020	0.18524
H	1.46963	-1.91627	2.88922
H	1.23070	-0.36651	2.01978
H	3.17223	0.98826	1.78322
H	4.03001	2.08706	0.67010
H	-1.22429	-1.02904	2.04712
H	-0.77323	-2.59960	2.79024
H	2.31267	3.73712	0.91252
H	1.43848	2.65688	2.04939
H	-1.51708	-3.80492	0.73436
H	-2.83330	-2.92337	1.56557
H	-0.00473	4.36582	0.92684
H	0.36096	3.63690	-0.67124
H	-4.24109	-2.40138	-0.17970
H	-3.57754	-1.42017	-1.52081
H	-2.10924	3.06576	-0.83709
H	-2.29052	3.85229	0.76512

H	-4.84361	-0.06978	0.15387
H	-3.69879	-0.55268	1.44175
H	-2.98782	1.67322	1.77325
H	-4.09567	2.28189	0.49785
C	3.22865	-1.97378	-1.21762
H	2.50411	-2.80673	-1.26303
H	4.24878	-2.40818	-1.08709
H	3.20815	-1.45223	-2.19304
Cs	-0.05550	-0.04432	-1.35285

ACE4 Cs B97-1 def2tzv

47

O	0.38274	-2.13985	1.09987
O	2.03836	1.83315	0.20238
O	-2.17850	-2.15039	-0.31239
O	-0.77399	2.48656	0.64661
O	-2.94319	0.68418	-0.09447
N	2.86913	-1.03996	-0.18760
C	2.77624	-1.73084	1.12546
C	3.75848	0.14335	-0.17722
C	1.45721	-1.50880	1.86344
C	3.29353	1.29148	0.71059
C	-0.89409	-2.15461	1.80276
C	1.57254	2.97512	0.97927
C	-1.91359	-2.87283	0.93294
C	0.26609	3.47927	0.40391
C	-3.53254	-1.63345	-0.48055
C	-2.09884	2.93362	0.23206
C	-3.82639	-0.38822	0.34232
C	-3.12338	1.91010	0.67348
H	3.59454	-1.41812	1.79065

H	2.89445	-2.80787	0.97266
H	3.83635	0.51197	-1.20363
H	4.77952	-0.12935	0.15019
H	1.51767	-1.96967	2.85798
H	1.23991	-0.44107	1.98800
H	3.16208	0.97179	1.75167
H	4.06223	2.07464	0.69640
H	-1.21678	-1.12904	2.02142
H	-0.78976	-2.69678	2.75178
H	2.31552	3.78146	0.93652
H	1.43298	2.68210	2.02686
H	-1.52772	-3.85721	0.65751
H	-2.83875	-3.00886	1.50272
H	0.00199	4.42231	0.89801
H	0.36678	3.66520	-0.67236
H	-4.26680	-2.40748	-0.22930
H	-3.61945	-1.40337	-1.54323
H	-2.12897	3.06344	-0.85665
H	-2.33264	3.89518	0.70535
H	-4.87424	-0.10157	0.18400
H	-3.68269	-0.57493	1.41415
H	-3.00535	1.69975	1.74313
H	-4.12980	2.31143	0.50271
C	3.23709	-1.96328	-1.28357
H	2.52065	-2.78518	-1.32925
H	4.24722	-2.38989	-1.16001
H	3.20916	-1.43084	-2.23746
Cs	-0.05254	-0.03351	-1.27440

ACE4 Cs CAM-B3LYP def2sv

47

O	0.36716	-2.01520	1.12077
O	1.99735	1.77105	0.24527
O	-2.14377	-2.11438	-0.26208
O	-0.76031	2.43090	0.60352
O	-2.88499	0.64821	-0.04318
N	2.84921	-1.03811	-0.16396
C	2.73361	-1.68565	1.14156
C	3.71027	0.13443	-0.16988
C	1.41799	-1.43096	1.85752
C	3.23919	1.28469	0.69716
C	-0.86264	-2.05863	1.80213
C	1.55214	2.89697	0.96171
C	-1.87812	-2.79060	0.95114
C	0.24438	3.39464	0.39965
C	-3.45626	-1.63345	-0.45355
C	-2.05159	2.86958	0.25582
C	-3.76481	-0.38022	0.33901
C	-3.06900	1.84061	0.68038
H	3.56056	-1.38036	1.81198
H	2.83036	-2.77612	1.01522
H	3.78383	0.49952	-1.20776
H	4.74486	-0.12067	0.15497
H	1.46754	-1.88578	2.86662
H	1.23384	-0.34655	1.98662
H	3.16361	0.98168	1.75975
H	4.00053	2.08756	0.64658
H	-1.21695	-1.03347	2.03134
H	-0.74980	-2.59854	2.76355
H	2.29723	3.71395	0.89558
H	1.42131	2.64375	2.03228

H	-1.48138	-3.79177	0.70752
H	-2.80382	-2.92816	1.53661
H	-0.01963	4.33887	0.91402
H	0.35556	3.61927	-0.68035
H	-4.19961	-2.41151	-0.19850
H	-3.54973	-1.41761	-1.52984
H	-2.12130	3.04808	-0.83616
H	-2.28444	3.82319	0.76794
H	-4.81614	-0.08724	0.14629
H	-3.67208	-0.57422	1.42616
H	-2.96552	1.64753	1.76611
H	-4.08352	2.24793	0.50278
C	3.21181	-1.96263	-1.22415
H	2.49318	-2.79692	-1.26199
H	4.23118	-2.39068	-1.09730
H	3.18391	-1.44750	-2.19925
Cs	-0.06388	-0.03700	-1.31488

ACE4 Cs CAM-B3LYP def2tzv

47

O	0.38634	-2.13966	1.07740
O	2.03147	1.81282	0.19666
O	-2.15335	-2.12103	-0.32720
O	-0.75755	2.47243	0.62217
O	-2.91497	0.68276	-0.08363
N	2.84618	-1.04105	-0.19631
C	2.76374	-1.73326	1.10315
C	3.73254	0.13048	-0.19806
C	1.45612	-1.52081	1.84043
C	3.28034	1.26736	0.69239
C	-0.88513	-2.16865	1.77107

C	1.57258	2.95306	0.96351
C	-1.89322	-2.86862	0.89327
C	0.27931	3.45735	0.38692
C	-3.50035	-1.61172	-0.50241
C	-2.08219	2.91866	0.23863
C	-3.79469	-0.38895	0.33282
C	-3.08894	1.89692	0.68758
H	3.58143	-1.41909	1.76108
H	2.88842	-2.80534	0.94713
H	3.79808	0.50182	-1.22014
H	4.75295	-0.14638	0.11214
H	1.51336	-1.98697	2.82801
H	1.24080	-0.45776	1.97135
H	3.15077	0.94272	1.72768
H	4.04596	2.04759	0.68192
H	-1.20907	-1.15040	2.00045
H	-0.78099	-2.71940	2.71007
H	2.31944	3.75012	0.92139
H	1.42889	2.66391	2.00721
H	-1.50674	-3.84321	0.60064
H	-2.81809	-3.01683	1.45287
H	0.01275	4.39692	0.87701
H	0.38432	3.64182	-0.68491
H	-4.22945	-2.39070	-0.27038
H	-3.58116	-1.36589	-1.55794
H	-2.13204	3.05549	-0.84425
H	-2.30402	3.87392	0.72068
H	-4.83742	-0.09746	0.18021
H	-3.65110	-0.59070	1.39759
H	-2.95351	1.68148	1.74985

H -4.09698 2.29013 0.53508

C 3.15006 -1.94868 -1.31008

H 2.42161 -2.75567 -1.33846

H 4.15276 -2.39242 -1.23144

H 3.09547 -1.40741 -2.25364

Cs -0.05975 -0.01764 -1.23065

ACE4 Cs MP2 def2tzv

47

O 0.30156 -1.65831 -0.28574

O -3.05816 0.80154 0.06385

O 3.06315 -1.13865 -0.05165

O -0.45981 2.31718 -0.07790

O 1.88774 1.22270 -1.37421

N -2.92166 -2.22217 0.28214

C -1.99720 -2.52008 -0.83359

C -4.10325 -1.41076 -0.08799

C -0.91364 -1.45353 -1.10922

C -3.86828 -0.07202 -0.80137

C 1.21575 -2.68094 -0.81034

C -2.90059 2.15108 -0.50168

C 2.56102 -2.52199 -0.10008

C -1.79240 2.85044 0.26927

C 3.55458 -0.57209 -1.31686

C 0.14455 2.92608 -1.28065

C 2.48149 0.11942 -2.14878

C 0.78524 1.84634 -2.12855

H -2.60687 -2.66265 -1.73497

H -1.49950 -3.47601 -0.62235

H -4.67539 -1.22802 0.82837

H -4.73092 -2.01569 -0.75795

H	-0.62349	-1.46754	-2.17046
H	-1.25726	-0.45644	-0.83074
H	-3.35062	-0.21438	-1.76088
H	-4.83947	0.40306	-1.00238
H	1.31511	-2.55393	-1.89497
H	0.83581	-3.69159	-0.61404
H	-3.84285	2.70588	-0.40155
H	-2.64363	2.07615	-1.56750
H	2.47645	-2.80941	0.94924
H	3.30368	-3.16406	-0.59240
H	-1.82032	3.92870	0.07293
H	-1.91241	2.67343	1.33971
H	4.01328	-1.36495	-1.92409
H	4.32323	0.14746	-1.02855
H	0.88032	3.67418	-0.96902
H	-0.63284	3.41381	-1.88193
H	2.95077	0.51974	-3.05883
H	1.68811	-0.57326	-2.43966
H	0.03946	1.08357	-2.38115
H	1.17861	2.29145	-3.05238
C	-2.24200	-1.82918	1.53720
H	-1.54949	-2.62589	1.82817
H	-2.99728	-1.71474	2.32051
H	-1.68209	-0.88887	1.45485
Cs	1.21243	0.54896	1.62905

ACE4 Cs PBE0 def2sv

47

O	0.35664	-2.01894	1.12969
O	2.00103	1.77038	0.24339
O	-2.15144	-2.11314	-0.25487

O	-0.75369	2.43310	0.61798
O	-2.87897	0.65775	-0.04844
N	2.83630	-1.04085	-0.15540
C	2.72007	-1.68643	1.14824
C	3.70158	0.12477	-0.16189
C	1.40537	-1.43585	1.86409
C	3.23532	1.27892	0.69968
C	-0.87124	-2.04446	1.80952
C	1.55993	2.89309	0.96149
C	-1.89443	-2.77209	0.96613
C	0.25040	3.39161	0.40774
C	-3.45610	-1.62193	-0.44837
C	-2.03754	2.87303	0.25693
C	-3.76142	-0.36361	0.33523
C	-3.06284	1.84933	0.67075
H	3.54665	-1.38021	1.82123
H	2.82151	-2.77825	1.02313
H	3.77964	0.48696	-1.20215
H	4.73712	-0.13194	0.16491
H	1.45966	-1.89201	2.87423
H	1.22081	-0.35038	1.99908
H	3.15703	0.97790	1.76473
H	4.00531	2.07590	0.65035
H	-1.21592	-1.01339	2.03480
H	-0.76645	-2.57979	2.77633
H	2.30356	3.71333	0.89375
H	1.43444	2.64065	2.03456
H	-1.51068	-3.78323	0.73650
H	-2.82155	-2.89234	1.55589
H	-0.00565	4.33961	0.92265

H	0.35661	3.61749	-0.67419
H	-4.20977	-2.39253	-0.19374
H	-3.54546	-1.41096	-1.52742
H	-2.09687	3.05278	-0.83712
H	-2.27683	3.82899	0.76514
H	-4.81342	-0.07043	0.13736
H	-3.67588	-0.55318	1.42559
H	-2.97252	1.65822	1.75967
H	-4.07393	2.26505	0.48406
C	3.19140	-1.96535	-1.21333
H	2.46519	-2.79460	-1.25240
H	4.20865	-2.40334	-1.09039
H	3.16637	-1.44902	-2.18928
Cs	-0.05461	-0.04463	-1.32816

ACE4 Cs PBE0 def2tzv

47

O	0.37824	-2.11806	1.09495
O	2.02159	1.81200	0.19204
O	-2.15985	-2.13507	-0.31297
O	-0.76496	2.46467	0.63076
O	-2.91519	0.67752	-0.10254
N	2.84633	-1.03692	-0.19054
C	2.75342	-1.71022	1.11744
C	3.72864	0.13623	-0.19045
C	1.44397	-1.48405	1.84650
C	3.27064	1.27724	0.69005
C	-0.89116	-2.11509	1.78841
C	1.56546	2.95044	0.95865
C	-1.90608	-2.83460	0.93439
C	0.26621	3.45103	0.39248

C	-3.50184	-1.62167	-0.49231
C	-2.08099	2.91186	0.22830
C	-3.79783	-0.38511	0.32158
C	-3.09800	1.89287	0.66012
H	3.57142	-1.39286	1.77915
H	2.87398	-2.78772	0.97577
H	3.80287	0.50184	-1.21728
H	4.75085	-0.13524	0.13080
H	1.50361	-1.93086	2.84646
H	1.22897	-0.41533	1.95948
H	3.14737	0.95852	1.73177
H	4.03846	2.05990	0.67320
H	-1.20995	-1.08560	1.98976
H	-0.79441	-2.63964	2.74695
H	2.30842	3.75520	0.90973
H	1.43107	2.66580	2.00833
H	-1.52883	-3.82712	0.68012
H	-2.83234	-2.95473	1.50435
H	0.00405	4.39255	0.88854
H	0.36572	3.64286	-0.68202
H	-4.23961	-2.39335	-0.24786
H	-3.58326	-1.39243	-1.55473
H	-2.11679	3.05287	-0.85802
H	-2.31413	3.86898	0.70880
H	-4.84345	-0.09784	0.15669
H	-3.66599	-0.57359	1.39393
H	-2.98434	1.68250	1.72941
H	-4.10344	2.29411	0.48922
C	3.19957	-1.96012	-1.27438
H	2.48080	-2.77890	-1.31372

H	4.20706	-2.39127	-1.15488
H	3.17047	-1.43539	-2.23138
Cs	-0.05061	-0.03720	-1.24748

ACE4 Cs PBE def2sv

47

O	0.36988	-2.03926	1.12489
O	2.01188	1.79423	0.24427
O	-2.17154	-2.14663	-0.26006
O	-0.77003	2.44906	0.62627
O	-2.91333	0.65969	-0.05739
N	2.87580	-1.03661	-0.16195
C	2.75299	-1.70068	1.14583
C	3.73989	0.14497	-0.14699
C	1.43078	-1.45566	1.87176
C	3.25543	1.30001	0.71979
C	-0.86711	-2.07259	1.82021
C	1.55864	2.92350	0.97505
C	-1.89704	-2.80885	0.97456
C	0.24143	3.42234	0.41513
C	-3.49305	-1.64606	-0.43177
C	-2.06655	2.89364	0.25523
C	-3.79213	-0.37533	0.35325
C	-3.09800	1.86365	0.67160
H	3.58544	-1.40252	1.83111
H	2.85238	-2.79964	1.00628
H	3.83238	0.51398	-1.19287
H	4.78157	-0.10800	0.19780
H	1.48953	-1.92797	2.88487
H	1.24268	-0.36319	2.02257
H	3.16402	0.99165	1.79179

H	4.03036	2.10646	0.68418
H	-1.21777	-1.03536	2.05547
H	-0.75318	-2.61694	2.79241
H	2.30486	3.75523	0.91462
H	1.42998	2.66242	2.05582
H	-1.50213	-3.82393	0.73589
H	-2.82622	-2.93922	1.57564
H	-0.01973	4.37739	0.93546
H	0.34866	3.65242	-0.67590
H	-4.25065	-2.41975	-0.15752
H	-3.59672	-1.44128	-1.51997
H	-2.12225	3.07451	-0.84877
H	-2.31115	3.85833	0.76571
H	-4.85905	-0.08815	0.17036
H	-3.68691	-0.55791	1.45295
H	-3.00648	1.66912	1.76974
H	-4.11791	2.28461	0.48596
C	3.26472	-1.95675	-1.22794
H	2.54334	-2.80030	-1.28596
H	4.29400	-2.38768	-1.09021
H	3.25080	-1.42880	-2.20761
Cs	-0.06191	-0.04508	-1.34208

ACE4 Cs PBE def2tzv

47

O	0.38991	-2.14853	1.08702
O	2.03285	1.83887	0.18927
O	-2.18155	-2.16577	-0.31859
O	-0.78230	2.47756	0.64967
O	-2.95141	0.68165	-0.12063
N	2.88253	-1.03195	-0.19569

C	2.78511	-1.72576	1.11871
C	3.76834	0.15671	-0.17293
C	1.46853	-1.50668	1.85685
C	3.29467	1.29924	0.71242
C	-0.89166	-2.14961	1.80432
C	1.56501	2.98545	0.97818
C	-1.91573	-2.87278	0.94917
C	0.25806	3.48610	0.40876
C	-3.54745	-1.64471	-0.47447
C	-2.11220	2.93559	0.22519
C	-3.83597	-0.39482	0.33758
C	-3.13956	1.91434	0.65501
H	3.60937	-1.41530	1.79062
H	2.90534	-2.80914	0.96178
H	3.85730	0.52836	-1.20476
H	4.79535	-0.11444	0.16542
H	1.53092	-1.96922	2.85876
H	1.24455	-0.43280	1.98233
H	3.15495	0.97566	1.75941
H	4.06586	2.09059	0.70699
H	-1.20979	-1.11359	2.01718
H	-0.78254	-2.68549	2.76536
H	2.31077	3.79962	0.93580
H	1.43117	2.68683	2.03288
H	-1.53532	-3.87064	0.68490
H	-2.84641	-2.99576	1.52638
H	-0.01177	4.43133	0.91238
H	0.35475	3.68199	-0.67384
H	-4.28468	-2.42157	-0.20835
H	-3.64280	-1.42596	-1.54622

H	-2.13427	3.07477	-0.87024
H	-2.34841	3.90189	0.70522
H	-4.89338	-0.11004	0.18470
H	-3.68353	-0.57076	1.41779
H	-3.03181	1.69886	1.73248
H	-4.15120	2.32009	0.47676
C	3.27466	-1.95319	-1.28730
H	2.56176	-2.78587	-1.34474
H	4.29330	-2.37541	-1.15010
H	3.25654	-1.41808	-2.24739
Cs	-0.05245	-0.03821	-1.26481

ACE4 Cs B2PLYP def2sv

47

O	-0.34794	-2.00350	-1.13769
O	-2.01293	1.75877	-0.23571
O	2.16011	-2.11831	0.25772
O	0.74353	2.42843	-0.61429
O	2.87819	0.65548	0.04543
N	-2.83863	-1.05688	0.15524
C	-2.71825	-1.68573	-1.16440
C	-3.71842	0.10680	0.17134
C	-1.40123	-1.40263	-1.87095
C	-3.26000	1.26908	-0.68999
C	0.89076	-2.01255	-1.81854
C	-1.57628	2.89277	-0.95786
C	1.90642	-2.76489	-0.98202
C	-0.26653	3.39383	-0.39852
C	3.47381	-1.62461	0.45576
C	2.03604	2.87728	-0.25683
C	3.77796	-0.36573	-0.33381

C	3.06167	1.85379	-0.68148
H	-3.54867	-1.37937	-1.83097
H	-2.80101	-2.77937	-1.05488
H	-3.79425	0.46325	1.21225
H	-4.75080	-0.15796	-0.15540
H	-1.44239	-1.83324	-2.89119
H	-1.22280	-0.31459	-1.96955
H	-3.18742	0.97660	-1.75541
H	-4.02577	2.06710	-0.62703
H	1.23728	-0.97766	-2.01101
H	0.78709	-2.52261	-2.79733
H	-2.32453	3.70647	-0.88589
H	-1.44923	2.64106	-2.02898
H	1.51337	-3.77370	-0.76703
H	2.83713	-2.88141	-1.56420
H	-0.00765	4.34111	-0.91009
H	-0.37298	3.61220	0.68299
H	4.22470	-2.39641	0.20268
H	3.55698	-1.41058	1.53292
H	2.09957	3.05218	0.83584
H	2.26518	3.83374	-0.76548
H	4.82393	-0.06164	-0.12867
H	3.69826	-0.55721	-1.42201
H	2.96278	1.66105	-1.76743
H	4.07339	2.26590	-0.49867
C	-3.21312	-2.00692	1.19526
H	-2.48670	-2.83440	1.22697
H	-4.22676	-2.44057	1.04361
H	-3.20381	-1.50760	2.17858
Cs	0.06020	-0.04262	1.33268

ACE4 Cs B2PLYP def2tzv

47

O	-0.36453	-2.13210	-1.09167
O	-2.05234	1.80669	-0.18255
O	2.17848	-2.13382	0.33476
O	0.73890	2.47905	-0.62922
O	2.91597	0.69653	0.08530
N	-2.84208	-1.06718	0.18656
C	-2.75527	-1.73645	-1.13729
C	-3.75190	0.10003	0.19907
C	-1.43793	-1.49316	-1.86144
C	-3.31025	1.25282	-0.68820
C	0.92101	-2.14131	-1.79055
C	-1.60515	2.96415	-0.95799
C	1.92979	-2.86130	-0.91753
C	-0.30579	3.47413	-0.38260
C	3.53400	-1.60424	0.50754
C	2.07402	2.94114	-0.24608
C	3.81736	-0.37502	-0.33505
C	3.08746	1.91868	-0.70102
H	-3.57139	-1.40750	-1.79310
H	-2.87354	-2.81401	-1.00558
H	-3.81980	0.46032	1.22632
H	-4.76924	-0.18827	-0.11810
H	-1.47924	-1.93709	-2.86128
H	-1.22706	-0.42475	-1.95842
H	-3.17707	0.93876	-1.72712
H	-4.08129	2.02911	-0.66618
H	1.24139	-1.11560	-1.99411
H	0.81962	-2.67490	-2.74112

H	-2.35834	3.75592	-0.90299
H	-1.46622	2.67811	-2.00412
H	1.54785	-3.84506	-0.64766
H	2.86221	-2.98668	-1.47282
H	-0.04105	4.41337	-0.87673
H	-0.40468	3.65527	0.69120
H	4.26873	-2.37838	0.27110
H	3.60872	-1.35869	1.56437
H	2.12570	3.07893	0.83738
H	2.28614	3.89634	-0.73504
H	4.85699	-0.06961	-0.17980
H	3.67352	-0.57796	-1.40024
H	2.94750	1.69744	-1.76238
H	4.09552	2.31493	-0.54911
C	-3.17797	-2.01167	1.27579
H	-2.45119	-2.82170	1.29184
H	-4.18267	-2.44662	1.16188
H	-3.13816	-1.49217	2.23323
Cs	0.05733	-0.02194	1.25258

ACE4 Cs DSDPBEP86 def2sv

47

O	-0.26889	-1.94989	-1.17975
O	-2.07065	1.68724	-0.16356
O	2.18650	-2.08298	0.27819
O	0.64869	2.41146	-0.60798
O	2.80907	0.69783	0.07382
N	-2.74347	-1.14000	0.14415
C	-2.62947	-1.68521	-1.20923
C	-3.68689	-0.03709	0.24959
C	-1.32036	-1.32103	-1.88685

C	-3.31756	1.17411	-0.58373
C	0.98665	-1.84722	-1.81616
C	-1.68253	2.82887	-0.89624
C	1.99284	-2.64249	-1.01134
C	-0.37364	3.35457	-0.36337
C	3.47216	-1.54825	0.52877
C	1.92844	2.89157	-0.25282
C	3.76626	-0.28498	-0.25468
C	2.97413	1.88768	-0.66729
H	-3.47310	-1.35493	-1.84908
H	-2.68703	-2.78779	-1.16977
H	-3.73379	0.27545	1.30876
H	-4.71871	-0.34594	-0.04479
H	-1.33564	-1.67519	-2.93890
H	-1.17057	-0.22162	-1.89698
H	-3.27942	0.92305	-1.66362
H	-4.10852	1.94216	-0.45751
H	1.30176	-0.78497	-1.88933
H	0.93320	-2.26097	-2.84507
H	-2.44942	3.62571	-0.80659
H	-1.57250	2.57778	-1.97147
H	1.61207	-3.67243	-0.88492
H	2.94749	-2.70000	-1.56676
H	-0.14293	4.31254	-0.87244
H	-0.46216	3.56034	0.72422
H	4.25886	-2.29816	0.31110
H	3.50209	-1.32640	1.60898
H	1.98720	3.07275	0.84107
H	2.13621	3.85182	-0.76755
H	4.78943	0.06327	-0.00146

H 3.74637 -0.48458 -1.34596

H 2.88064 1.67954 -1.75293

H 3.97887 2.32117 -0.48546

C -3.00950 -2.16246 1.14335

H -2.22615 -2.93873 1.09995

H -3.99898 -2.65709 1.00857

H -2.98999 -1.71260 2.15255

Cs 0.04433 -0.05022 1.27383

ACE4 Cs DSDPBEP86 def2tzv

47

O 0.27210 -1.92482 0.81813

O 2.63925 1.32802 0.03011

O -2.57038 -1.86386 -0.22203

O -0.14720 2.23690 0.61260

O -2.72503 0.93002 0.45472

N 3.43257 -1.63365 -0.08930

C 2.72167 -2.06172 1.12010

C 4.33554 -0.48258 0.05821

C 1.42865 -1.32605 1.52004

C 3.79875 0.77716 0.74078

C -0.93486 -1.90057 1.64936

C 2.25451 2.60856 0.62732

C -2.05579 -2.62194 0.92260

C 0.92944 3.07425 0.07279

C -3.83535 -1.16355 0.01065

C -1.42621 2.94659 0.70122

C -3.72022 0.00134 0.98043

C -2.43872 2.03958 1.36110

H 3.43042 -1.98357 1.95361

H 2.46151 -3.12224 1.01453

H	4.70254	-0.22333	-0.94014
H	5.20727	-0.79154	0.65319
H	1.27696	-1.45133	2.59959
H	1.46135	-0.26118	1.27879
H	3.51061	0.56536	1.77938
H	4.60009	1.52881	0.76160
H	-1.20632	-0.86278	1.87098
H	-0.73052	-2.42273	2.59318
H	3.02588	3.35665	0.40420
H	2.17162	2.49630	1.71564
H	-1.68253	-3.56830	0.52642
H	-2.87156	-2.82988	1.62511
H	0.77111	4.11320	0.38483
H	0.92529	3.03182	-1.02280
H	-4.58605	-1.87019	0.38422
H	-4.14200	-0.80652	-0.97338
H	-1.76824	3.23702	-0.29939
H	-1.29988	3.84964	1.30998
H	-4.69475	0.49896	1.05948
H	-3.42145	-0.33220	1.98134
H	-2.04336	1.65386	2.30846
H	-3.35854	2.60338	1.55723
C	2.66144	-1.65492	-1.33814
H	1.99571	-2.52170	-1.33433
H	3.34264	-1.74439	-2.19046
H	2.06644	-0.74582	-1.48297
Cs	-0.64064	0.05402	-1.50909

ACE4 Cs HSE06 def2sv

47

O	0.35893	-2.01810	1.12595

O	2.00077	1.76919	0.23994
O	-2.14636	-2.11204	-0.25840
O	-0.75295	2.43257	0.61358
O	-2.87769	0.65717	-0.05282
N	2.83469	-1.03999	-0.16254
C	2.72238	-1.68670	1.14115
C	3.70103	0.12489	-0.17028
C	1.40943	-1.43653	1.86031
C	3.23644	1.27745	0.69414
C	-0.86864	-2.04724	1.80746
C	1.56043	2.89257	0.95873
C	-1.89114	-2.77370	0.96236
C	0.25149	3.39196	0.40463
C	-3.45219	-1.62291	-0.45460
C	-2.03795	2.87312	0.25460
C	-3.75988	-0.36578	0.32967
C	-3.06215	1.84835	0.66823
H	3.55073	-1.38053	1.81203
H	2.82370	-2.77846	1.01510
H	3.77672	0.48844	-1.21022
H	4.73718	-0.13307	0.15374
H	1.46468	-1.89451	2.86951
H	1.22562	-0.35113	1.99731
H	3.15924	0.97431	1.75873
H	4.00566	2.07513	0.64558
H	-1.21442	-1.01724	2.03603
H	-0.76188	-2.58501	2.77263
H	2.30501	3.71191	0.89131
H	1.43471	2.63963	2.03160
H	-1.50756	-3.78457	0.73154

H	-2.81914	-2.89425	1.55062
H	-0.00537	4.33929	0.92020
H	0.35810	3.61865	-0.67702
H	-4.20474	-2.39506	-0.20172
H	-3.53971	-1.41133	-1.53360
H	-2.09844	3.05460	-0.83904
H	-2.27634	3.82824	0.76465
H	-4.81190	-0.07307	0.13163
H	-3.67450	-0.55603	1.41993
H	-2.97079	1.65569	1.75675
H	-4.07383	2.26303	0.48285
C	3.18440	-1.96375	-1.22312
H	2.45697	-2.79196	-1.26046
H	4.20149	-2.40335	-1.10427
H	3.15688	-1.44609	-2.19826
Cs	-0.05754	-0.04317	-1.31500

ACE4 Cs HSE06 def2tzv

47

O	0.38332	-2.11852	1.09106
O	2.02004	1.81144	0.18973
O	-2.15324	-2.13453	-0.31568
O	-0.76640	2.46325	0.62492
O	-2.91429	0.67497	-0.10708
N	2.84603	-1.03417	-0.19890
C	2.75858	-1.71059	1.10802
C	3.72820	0.13903	-0.20013
C	1.45199	-1.48606	1.84210
C	3.27187	1.27710	0.68452
C	-0.88635	-2.11639	1.78637
C	1.56343	2.95040	0.95735

C	-1.90101	-2.83569	0.93258
C	0.26543	3.45137	0.38962
C	-3.49729	-1.62465	-0.49918
C	-2.08394	2.91092	0.22362
C	-3.79724	-0.38977	0.31497
C	-3.09969	1.89127	0.65557
H	3.57892	-1.39429	1.76687
H	2.87908	-2.78746	0.96370
H	3.79852	0.50689	-1.22608
H	4.75144	-0.13274	0.11669
H	1.51360	-1.93552	2.84032
H	1.23719	-0.41793	1.95801
H	3.15062	0.95561	1.72533
H	4.03776	2.06109	0.66821
H	-1.20491	-1.08720	1.98783
H	-0.78794	-2.64076	2.74439
H	2.30693	3.75423	0.90953
H	1.42814	2.66477	2.00626
H	-1.52477	-3.82812	0.67807
H	-2.82777	-2.95478	1.50127
H	0.00124	4.39153	0.88632
H	0.36623	3.64423	-0.68418
H	-4.23293	-2.39860	-0.25734
H	-3.57580	-1.39525	-1.56139
H	-2.12011	3.05281	-0.86218
H	-2.31614	3.86733	0.70511
H	-4.84237	-0.10306	0.14853
H	-3.66676	-0.57858	1.38711
H	-2.98657	1.68107	1.72457
H	-4.10547	2.29021	0.48364

C	3.18984	-1.95434	-1.28844
H	2.46965	-2.77154	-1.32533
H	4.19715	-2.38749	-1.17770
H	3.15487	-1.42653	-2.24319
Cs	-0.05346	-0.03599	-1.23232

ACE4 Cs M062X def2sv

47

O	0.25616	-1.90209	1.18490
O	2.07893	1.65858	0.14042
O	-2.18787	-2.08509	-0.28752
O	-0.62962	2.39703	0.59047
O	-2.79023	0.68981	-0.07107
N	2.74552	-1.16353	-0.13990
C	2.61525	-1.66938	1.22478
C	3.69045	-0.06678	-0.26872
C	1.30544	-1.26502	1.87837
C	3.32622	1.15311	0.55335
C	-1.00352	-1.77007	1.79712
C	1.70247	2.81625	0.84469
C	-1.99707	-2.60285	1.01395
C	0.38824	3.32965	0.31360
C	-3.46303	-1.54496	-0.55424
C	-1.91164	2.87847	0.26302
C	-3.75574	-0.28566	0.23563
C	-2.94994	1.86897	0.68024
H	3.45811	-1.33556	1.85982
H	2.65294	-2.77164	1.21408
H	3.72838	0.23149	-1.33084
H	4.72016	-0.37559	0.02299
H	1.30227	-1.57982	2.94033

H	1.17146	-0.16330	1.84514
H	3.29462	0.91104	1.63482
H	4.11177	1.92101	0.41533
H	-1.31846	-0.70449	1.81147
H	-0.96208	-2.13058	2.84360
H	2.46950	3.60551	0.72541
H	1.60349	2.59170	1.92538
H	-1.59873	-3.62747	0.92097
H	-2.95354	-2.65869	1.56281
H	0.16248	4.29762	0.80015
H	0.46421	3.50583	-0.77880
H	-4.25371	-2.29113	-0.35025
H	-3.47926	-1.31567	-1.63169
H	-1.98643	3.06935	-0.82681
H	-2.10856	3.83168	0.78983
H	-4.77041	0.07463	-0.02330
H	-3.74698	-0.49490	1.32442
H	-2.84337	1.64906	1.76093
H	-3.95615	2.29867	0.51357
C	3.00009	-2.20747	-1.11667
H	2.21334	-2.97648	-1.05247
H	3.98585	-2.70212	-0.97293
H	2.97982	-1.77993	-2.13369
Cs	-0.05566	-0.05104	-1.24508

ACE4 Cs M062X def2tzv

47

O	0.29250	-1.98077	1.18481
O	2.07991	1.69445	0.11606
O	-2.16841	-2.12035	-0.31789
O	-0.65943	2.42478	0.60865

O	-2.81836	0.68459	-0.11218
N	2.75810	-1.15399	-0.18628
C	2.66353	-1.68871	1.18431
C	3.70606	-0.03986	-0.32250
C	1.37016	-1.29235	1.87147
C	3.35756	1.15720	0.53707
C	-1.00551	-1.81913	1.80509
C	1.68592	2.85465	0.88679
C	-1.98408	-2.66080	1.01811
C	0.39092	3.38469	0.33457
C	-3.48068	-1.57041	-0.59365
C	-1.96725	2.90240	0.20675
C	-3.78817	-0.33486	0.22163
C	-3.00073	1.90285	0.64849
H	3.51183	-1.36031	1.79464
H	2.70336	-2.77883	1.15165
H	3.71339	0.27246	-1.36766
H	4.72931	-0.35466	-0.06164
H	1.38461	-1.59309	2.92262
H	1.21080	-0.21020	1.81174
H	3.30778	0.88935	1.59667
H	4.13023	1.92128	0.41938
H	-1.30040	-0.76356	1.78923
H	-0.96734	-2.16066	2.84273
H	2.45603	3.62676	0.81244
H	1.56196	2.57610	1.93673
H	-1.58443	-3.66745	0.90926
H	-2.93826	-2.71914	1.54493
H	0.15300	4.33742	0.81371
H	0.47638	3.54289	-0.74405

H	-4.24862	-2.32556	-0.41144
H	-3.47426	-1.32271	-1.65262
H	-1.99947	3.02657	-0.87919
H	-2.16832	3.86734	0.67834
H	-4.79590	0.01385	-0.01887
H	-3.74591	-0.54338	1.29498
H	-2.88948	1.69149	1.71511
H	-4.00044	2.30530	0.46810
C	2.99039	-2.19197	-1.19958
H	2.21671	-2.95372	-1.12263
H	3.97076	-2.67544	-1.08618
H	2.94040	-1.74991	-2.19455
Cs	-0.05634	-0.03742	-1.18339

ACE4 Cs M06 def2sv

47

O	0.21216	-1.91408	1.23066
O	2.13944	1.64882	0.10837
O	-2.24541	-2.03354	-0.26141
O	-0.57042	2.39278	0.64544
O	-2.78668	0.74524	-0.06227
N	2.71073	-1.20522	-0.07976
C	2.57081	-1.66054	1.29796
C	3.67811	-0.14283	-0.27392
C	1.25174	-1.26812	1.92393
C	3.37955	1.11792	0.49956
C	-1.04994	-1.74220	1.82085
C	1.77309	2.78234	0.84768
C	-2.06357	-2.54256	1.04151
C	0.45592	3.30877	0.35865
C	-3.50367	-1.46957	-0.54345

C	-1.83954	2.89408	0.31179
C	-3.78307	-0.20040	0.22150
C	-2.90224	1.91236	0.70801
H	3.39878	-1.28689	1.93584
H	2.64686	-2.76519	1.33277
H	3.69660	0.10988	-1.35354
H	4.71458	-0.46822	-0.00566
H	1.23861	-1.56708	2.99561
H	1.10620	-0.16296	1.88604
H	3.37909	0.92877	1.59665
H	4.18998	1.85599	0.31128
H	-1.32748	-0.66047	1.83493
H	-1.03357	-2.09416	2.87621
H	2.54154	3.57939	0.74438
H	1.69795	2.52817	1.92941
H	-1.69855	-3.58529	0.95881
H	-3.02169	-2.57288	1.59734
H	0.25225	4.28309	0.85308
H	0.50768	3.49800	-0.73851
H	-4.31783	-2.19838	-0.34528
H	-3.50491	-1.25634	-1.62876
H	-1.89982	3.09877	-0.78209
H	-2.02497	3.85598	0.83720
H	-4.78667	0.18411	-0.06490
H	-3.81804	-0.39762	1.31693
H	-2.80562	1.66826	1.79016
H	-3.89959	2.38018	0.55902
C	2.89111	-2.28559	-1.02342
H	2.07253	-3.02146	-0.91517
H	3.86042	-2.82475	-0.89268

H	2.86392	-1.89906	-2.06093
Cs	-0.03902	-0.06743	-1.31709

ACE4 Cs M06 def2tzv

47

O	0.27226	-1.89667	0.92339
O	2.61675	1.36947	0.07179
O	-2.53604	-1.88721	-0.13064
O	-0.21291	2.24985	0.59409
O	-2.77739	0.92977	0.34020
N	3.36077	-1.51020	-0.10202
C	2.71226	-2.01405	1.08793
C	4.29768	-0.41227	0.04031
C	1.43821	-1.32276	1.58080
C	3.78848	0.84071	0.73333
C	-0.92499	-1.76151	1.72248
C	2.17330	2.57732	0.72522
C	-2.04535	-2.53053	1.06773
C	0.88760	3.06886	0.13030
C	-3.79965	-1.20012	-0.00551
C	-1.48608	2.93226	0.51697
C	-3.74467	0.01533	0.88728
C	-2.54064	2.08608	1.16662
H	3.44582	-1.97302	1.90066
H	2.46088	-3.07194	0.94668
H	4.65874	-0.14979	-0.95740
H	5.17338	-0.73864	0.61862
H	1.34352	-1.49041	2.66008
H	1.46009	-0.24330	1.39126
H	3.54257	0.62617	1.78240
H	4.58349	1.59719	0.73399

H	-1.18312	-0.70020	1.83633
H	-0.74420	-2.17725	2.72076
H	2.94175	3.35252	0.62088
H	2.02258	2.38575	1.79549
H	-1.67507	-3.51391	0.77424
H	-2.86107	-2.67218	1.78347
H	0.73080	4.10377	0.45233
H	0.93641	3.05761	-0.96513
H	-4.56708	-1.88625	0.36782
H	-4.06538	-0.90139	-1.01967
H	-1.74981	3.12727	-0.52972
H	-1.42189	3.89290	1.03879
H	-4.73537	0.48228	0.92173
H	-3.46645	-0.25283	1.91419
H	-2.20950	1.77154	2.16363
H	-3.46317	2.66702	1.27284
C	2.70137	-1.64019	-1.38357
H	2.00077	-2.47651	-1.35252
H	3.42989	-1.84202	-2.17568
H	2.15650	-0.73388	-1.67996
Cs	-0.58031	-0.02956	-1.49658

ACE4 Cs MP2 def2sv

47

O	0.27596	-1.93067	1.19279
O	2.05694	1.68826	0.17289
O	-2.17936	-2.08890	-0.25318
O	-0.66261	2.39845	0.61103
O	-2.81338	0.68139	-0.06931
N	2.75091	-1.12740	-0.13259
C	2.63342	-1.66315	1.22373

C	3.68961	-0.02016	-0.23116
C	1.32570	-1.28667	1.89337
C	3.30866	1.18716	0.59929
C	-0.97918	-1.81661	1.83307
C	1.66328	2.83007	0.90641
C	-1.98366	-2.62867	1.04642
C	0.35566	3.34931	0.36879
C	-3.47208	-1.56724	-0.50539
C	-1.94291	2.87800	0.24955
C	-3.77143	-0.30063	0.26737
C	-2.98621	1.87531	0.66700
H	3.48038	-1.33838	1.86285
H	2.67966	-2.76622	1.19121
H	3.74326	0.29408	-1.28918
H	4.72051	-0.32453	0.07146
H	1.33921	-1.62395	2.95095
H	1.17513	-0.18856	1.88318
H	3.27078	0.93834	1.67899
H	4.09336	1.96151	0.47309
H	-1.29714	-0.75491	1.88433
H	-0.92175	-2.20991	2.86964
H	2.42725	3.62969	0.81891
H	1.54967	2.57923	1.98074
H	-1.60040	-3.65928	0.93728
H	-2.93723	-2.67979	1.60393
H	0.11844	4.30576	0.87736
H	0.44802	3.55590	-0.71771
H	-4.25228	-2.32112	-0.27896
H	-3.50695	-1.35492	-1.58684
H	-2.00054	3.05406	-0.84469

H	-2.15290	3.83997	0.75973
H	-4.79487	0.04303	0.00965
H	-3.75266	-0.49111	1.35949
H	-2.89402	1.67419	1.75350
H	-3.99158	2.30479	0.48020
C	3.05852	-2.15831	-1.11252
H	2.28753	-2.94610	-1.07695
H	4.05182	-2.63334	-0.94596
H	3.05602	-1.72075	-2.12623
Cs	-0.04385	-0.06063	-1.31129

ACE4 Cs SCS-MP2 def2sv

47

O	-0.28985	-1.96534	-1.18270
O	-2.05511	1.70744	-0.18898
O	2.18205	-2.09311	0.25826
O	0.67718	2.41267	-0.62094
O	2.82940	0.68102	0.05593
N	-2.77007	-1.11637	0.13489
C	-2.65417	-1.67785	-1.21670
C	-3.70324	0.00356	0.21508
C	-1.33915	-1.32579	-1.89450
C	-3.30722	1.20538	-0.62442
C	0.96558	-1.87462	-1.83298
C	-1.65296	2.85048	-0.92237
C	1.97644	-2.66816	-1.02814
C	-0.34195	3.36729	-0.37840
C	3.48179	-1.57437	0.49709
C	1.96272	2.88684	-0.25818
C	3.78110	-0.30974	-0.28711
C	3.00412	1.87794	-0.68131

H	-3.49639	-1.35309	-1.86298
H	-2.71330	-2.78033	-1.16565
H	-3.76150	0.32814	1.27052
H	-4.73446	-0.29621	-0.09190
H	-1.35795	-1.68499	-2.94540
H	-1.17801	-0.22871	-1.90728
H	-3.25962	0.94813	-1.70224
H	-4.09031	1.98419	-0.51044
H	1.28562	-0.81521	-1.91785
H	0.90128	-2.29929	-2.85739
H	-2.41535	3.65275	-0.83693
H	-1.53578	2.59876	-1.99667
H	1.59380	-3.69615	-0.88781
H	2.92705	-2.73381	-1.59044
H	-0.10099	4.32426	-0.88563
H	-0.43608	3.57151	0.70900
H	4.25653	-2.33435	0.26956
H	3.52542	-1.35511	1.57778
H	2.02149	3.05879	0.83724
H	2.17587	3.84972	-0.76670
H	4.80872	0.03061	-0.03889
H	3.75068	-0.50476	-1.37875
H	2.90723	1.67801	-1.76817
H	4.01233	2.30326	-0.49621
C	-3.09302	-2.13314	1.13166
H	-2.32951	-2.93030	1.10955
H	-4.09155	-2.60003	0.96854
H	-3.08792	-1.68089	2.14009
Cs	0.04698	-0.05325	1.32527

ACE4 Cs SCS-MP2 def2tzv

47

O	0.24395	-1.95767	0.78105
O	2.79671	1.29079	0.04199
O	-2.66685	-1.80417	-0.11792
O	-0.00876	2.21491	0.58719
O	-2.65689	0.99502	0.58998
N	3.45972	-1.70566	-0.15053
C	2.71154	-2.16033	1.04252
C	4.41136	-0.59197	0.07999
C	1.44132	-1.37138	1.45207
C	3.90298	0.67006	0.79749
C	-0.92946	-1.94520	1.67857
C	2.41029	2.57338	0.65691
C	-2.10693	-2.61872	0.97935
C	1.09532	3.05054	0.06514
C	-3.89514	-1.05843	0.21778
C	-1.26264	2.97443	0.76776
C	-3.66532	0.10408	1.18343
C	-2.26551	2.09165	1.49075
H	3.41718	-2.15331	1.88385
H	2.40233	-3.20083	0.87702
H	4.82361	-0.30846	-0.89548
H	5.24271	-0.97464	0.69091
H	1.30414	-1.46307	2.53806
H	1.49695	-0.31567	1.17711
H	3.55536	0.43502	1.81347
H	4.73350	1.38708	0.87859
H	-1.16501	-0.91099	1.95046
H	-0.68771	-2.51111	2.58934
H	3.19474	3.31767	0.46310

H	2.29368	2.44198	1.74082
H	-1.77850	-3.55783	0.52737
H	-2.89337	-2.83039	1.71588
H	0.93512	4.09162	0.37251
H	1.11754	2.99917	-1.02999
H	-4.63500	-1.74506	0.64965
H	-4.26422	-0.69003	-0.74151
H	-1.65768	3.29051	-0.20606
H	-1.06248	3.86203	1.38155
H	-4.60994	0.64697	1.32221
H	-3.31494	-0.24385	2.16291
H	-1.81926	1.68186	2.40581
H	-3.15138	2.68596	1.74973
C	2.63816	-1.57112	-1.37372
H	2.03331	-2.47575	-1.49473
H	3.29967	-1.47322	-2.24088
H	1.98039	-0.69527	-1.34207
Cs	-0.79254	0.07011	-1.62132

ACE4 K B3LYP def2sv

47

O	0.67988	-2.18046	0.78336
O	1.73078	1.94136	0.01565
O	-1.78632	-2.13924	-0.48002
O	-1.06227	2.39212	0.36662
O	-2.93952	0.43987	-0.45387
N	2.80665	-0.75018	-0.61533
C	2.98567	-1.56333	0.59715
C	3.55764	0.50386	-0.61117
C	1.76050	-1.61831	1.50416
C	3.04536	1.54927	0.36985

C	-0.49476	-2.38330	1.54128
C	1.21386	2.99280	0.80858
C	-1.56528	-2.97942	0.64411
C	-0.12195	3.44360	0.25695
C	-3.12880	-1.90102	-0.87143
C	-2.36388	2.73274	-0.07343
C	-3.72448	-0.69871	-0.15679
C	-3.31796	1.60363	0.25340
H	3.84156	-1.19793	1.19816
H	3.23481	-2.59942	0.30848
H	3.49886	0.94018	-1.62289
H	4.63884	0.33865	-0.39392
H	1.99555	-2.24139	2.39223
H	1.48864	-0.60689	1.87087
H	3.06445	1.17285	1.41293
H	3.72379	2.42588	0.33043
H	-0.84892	-1.42214	1.96958
H	-0.30347	-3.07778	2.38580
H	1.90669	3.85863	0.80086
H	1.09528	2.66086	1.86038
H	-1.24375	-3.97852	0.29083
H	-2.48783	-3.10735	1.23914
H	-0.46293	4.32782	0.83296
H	-0.00662	3.75127	-0.80278
H	-3.76184	-2.79096	-0.69677
H	-3.10763	-1.70709	-1.95756
H	-2.36365	2.92982	-1.16542
H	-2.71705	3.65127	0.43728
H	-4.77248	-0.55297	-0.49242
H	-3.74546	-0.87172	0.93918

H	-3.30272	1.40951	1.34535
H	-4.34663	1.91332	-0.02337
C	3.01944	-1.51205	-1.84293
H	2.36327	-2.39834	-1.85166
H	4.07151	-1.85901	-1.96728
H	2.76153	-0.89280	-2.71982
K	-0.10700	0.02777	-0.94660

ACE4 K B3LYP def2tzv

47

O	0.62726	-2.34709	0.73266
O	1.84615	1.98416	-0.09129
O	-1.87129	-2.12517	-0.59272
O	-0.95850	2.50002	0.40918
O	-2.92794	0.56525	-0.41737
N	2.76396	-0.83473	-0.56153
C	2.94471	-1.63860	0.67481
C	3.59978	0.38324	-0.61979
C	1.69067	-1.74570	1.53416
C	3.15497	1.48593	0.32914
C	-0.61688	-2.56494	1.46200
C	1.36235	3.09065	0.72797
C	-1.65201	-3.09064	0.48890
C	0.02875	3.55448	0.19276
C	-3.25753	-1.77346	-0.89523
C	-2.30566	2.86647	-0.01975
C	-3.74464	-0.59521	-0.07454
C	-3.25509	1.75588	0.36088
H	3.73579	-1.20840	1.29727
H	3.27269	-2.64779	0.41076
H	3.55682	0.77300	-1.63732

H	4.65771	0.15988	-0.40347
H	1.89595	-2.37182	2.40892
H	1.36852	-0.75997	1.88507
H	3.09498	1.13054	1.36237
H	3.88256	2.30288	0.29411
H	-0.95437	-1.62683	1.91332
H	-0.46228	-3.29828	2.26023
H	2.07313	3.92187	0.68679
H	1.26237	2.76656	1.76795
H	-1.30921	-4.02468	0.04155
H	-2.58163	-3.28187	1.02910
H	-0.27574	4.46140	0.72367
H	0.10297	3.78107	-0.87472
H	-3.91577	-2.63033	-0.73501
H	-3.27224	-1.51856	-1.95349
H	-2.31938	3.03123	-1.10102
H	-2.61617	3.78924	0.47940
H	-4.79500	-0.39668	-0.31129
H	-3.66419	-0.79964	0.99759
H	-3.16927	1.53475	1.42856
H	-4.28176	2.06930	0.14859
C	2.88085	-1.65163	-1.78794
H	2.18008	-2.48274	-1.73952
H	3.89304	-2.06138	-1.93074
H	2.63370	-1.04611	-2.66013
K	-0.10779	0.06317	-0.86430

ACE4 K B97-1 def2sv

47

O	0.51338	-2.16771	0.81822
O	1.86976	1.86148	-0.05522

O	-1.91150	-2.09506	-0.55717
O	-0.88628	2.44105	0.36823
O	-2.87045	0.57798	-0.41527
N	2.73507	-0.91556	-0.55788
C	2.84621	-1.65078	0.71190
C	3.58328	0.27295	-0.63741
C	1.57685	-1.60777	1.56227
C	3.16252	1.40556	0.29332
C	-0.69866	-2.30474	1.52730
C	1.42702	2.95279	0.72449
C	-1.74169	-2.90605	0.59577
C	0.09115	3.44819	0.20423
C	-3.23473	-1.73117	-0.90608
C	-2.18455	2.83912	-0.02374
C	-3.72661	-0.51067	-0.13780
C	-3.17471	1.74333	0.32153
H	3.68412	-1.26139	1.32426
H	3.08243	-2.71199	0.50646
H	3.53978	0.65453	-1.67389
H	4.65376	0.03807	-0.42328
H	1.74285	-2.18675	2.49650
H	1.33131	-0.56292	1.85521
H	3.18077	1.08206	1.35637
H	3.89763	2.23262	0.19347
H	-1.04542	-1.31666	1.90348
H	-0.56825	-2.97322	2.40591
H	2.15582	3.78942	0.67237
H	1.32798	2.65219	1.78963
H	-1.41095	-3.91417	0.27505
H	-2.69068	-3.01890	1.15405

H	-0.19283	4.35985	0.77151
H	0.18422	3.72843	-0.86711
H	-3.93721	-2.57408	-0.75031
H	-3.21629	-1.49881	-1.98606
H	-2.21352	3.04912	-1.11473
H	-2.48431	3.76597	0.50952
H	-4.76933	-0.28024	-0.44684
H	-3.74054	-0.71840	0.95430
H	-3.13096	1.53135	1.41133
H	-4.19918	2.10076	0.08372
C	2.88635	-1.77904	-1.72642
H	2.15894	-2.60824	-1.67361
H	3.90834	-2.21832	-1.81878
H	2.67915	-1.20374	-2.64778
K	-0.08773	0.02632	-0.98326

ACE4 K B97-1 def2tzv

47

O	0.49372	-2.30747	0.78473
O	1.94175	1.89915	-0.12126
O	-1.96594	-2.09716	-0.63094
O	-0.83504	2.50914	0.40876
O	-2.87905	0.65479	-0.41209
N	2.71998	-0.95662	-0.53675
C	2.83900	-1.71101	0.73883
C	3.62139	0.21304	-0.62739
C	1.56091	-1.70349	1.57536
C	3.23349	1.36307	0.29536
C	-0.77461	-2.41835	1.49120
C	1.50903	3.02527	0.69549
C	-1.80279	-2.98193	0.52470

C	0.17977	3.53003	0.17546
C	-3.32748	-1.65998	-0.92202
C	-2.17042	2.92894	0.00044
C	-3.75342	-0.46395	-0.08478
C	-3.15702	1.84563	0.38060
H	3.64521	-1.29690	1.35729
H	3.11035	-2.75173	0.52795
H	3.59712	0.57770	-1.65771
H	4.66835	-0.06050	-0.40225
H	1.71653	-2.27986	2.49643
H	1.27894	-0.67856	1.85151
H	3.17642	1.03901	1.34218
H	3.99617	2.14890	0.22604
H	-1.08865	-1.43320	1.85946
H	-0.67320	-3.09445	2.34970
H	2.24642	3.83556	0.63760
H	1.41254	2.71211	1.74211
H	-1.47213	-3.95592	0.15452
H	-2.75345	-3.11170	1.05180
H	-0.08763	4.45031	0.70871
H	0.24784	3.75155	-0.89634
H	-4.03620	-2.48158	-0.77236
H	-3.32645	-1.38921	-1.97880
H	-2.19228	3.10626	-1.08150
H	-2.44403	3.85829	0.51457
H	-4.79464	-0.21008	-0.32116
H	-3.68760	-0.68619	0.98796
H	-3.06803	1.61071	1.44795
H	-4.17587	2.19916	0.18112
C	2.83565	-1.83407	-1.72170

H	2.09263	-2.63017	-1.65845
H	3.83331	-2.29661	-1.81345
H	2.64343	-1.25534	-2.62836
K	-0.08997	0.05010	-0.93853

ACE4 K CAM-B3LYP def2sv

47

O	0.62257	-2.15538	0.80244
O	1.76476	1.89442	-0.01686
O	-1.79952	-2.10087	-0.49681
O	-0.98952	2.39188	0.36408
O	-2.88268	0.48206	-0.44593
N	2.74259	-0.80851	-0.60376
C	2.92218	-1.57416	0.63033
C	3.52964	0.41319	-0.66541
C	1.69372	-1.59327	1.52313
C	3.07692	1.48980	0.30109
C	-0.55989	-2.32870	1.54155
C	1.28908	2.95445	0.77738
C	-1.61483	-2.92503	0.63647
C	-0.04166	3.42622	0.24826
C	-3.12563	-1.83397	-0.89891
C	-2.28262	2.75415	-0.05990
C	-3.69746	-0.63318	-0.17572
C	-3.24577	1.64036	0.26417
H	3.77272	-1.18310	1.21829
H	3.17549	-2.61846	0.38181
H	3.44702	0.82326	-1.68492
H	4.60989	0.21967	-0.48102
H	1.90599	-2.19536	2.42878
H	1.43152	-0.56910	1.85736

H	3.12267	1.13605	1.34972
H	3.76751	2.35148	0.21904
H	-0.90995	-1.35558	1.94181
H	-0.39069	-3.00607	2.40192
H	1.99921	3.80346	0.75316
H	1.18211	2.62840	1.83049
H	-1.29609	-3.93038	0.30520
H	-2.55146	-3.03420	1.21020
H	-0.36420	4.31100	0.83050
H	0.06480	3.73436	-0.81040
H	-3.77593	-2.71280	-0.74151
H	-3.09079	-1.62584	-1.98021
H	-2.28796	2.95724	-1.14897
H	-2.61565	3.67318	0.45953
H	-4.73555	-0.45373	-0.51907
H	-3.73362	-0.82100	0.91590
H	-3.22880	1.44052	1.35334
H	-4.27012	1.96071	-0.00873
C	2.89194	-1.62167	-1.79949
H	2.20502	-2.48131	-1.75445
H	3.92547	-2.01093	-1.93429
H	2.63360	-1.02942	-2.69308
K	-0.10152	0.02850	-0.89828

ACE4 K CAM-B3LYP def2tzv

47

O	0.55356	-2.30101	0.75952
O	1.88212	1.91621	-0.11127
O	-1.89309	-2.08106	-0.60916
O	-0.87985	2.49287	0.39975
O	-2.87090	0.61031	-0.40579

N	2.70952	-0.90009	-0.54950
C	2.86970	-1.65988	0.70491
C	3.57625	0.28114	-0.65401
C	1.61235	-1.70503	1.55097
C	3.18326	1.40656	0.27862
C	-0.69551	-2.47782	1.46838
C	1.44017	3.03394	0.69658
C	-1.71406	-3.01318	0.49357
C	0.11730	3.52045	0.17499
C	-3.25489	-1.69392	-0.92616
C	-2.21506	2.88408	-0.00602
C	-3.71821	-0.52275	-0.09473
C	-3.17377	1.79163	0.37573
H	3.66844	-1.22805	1.31320
H	3.17313	-2.68428	0.47729
H	3.52100	0.65177	-1.67676
H	4.62923	0.02803	-0.45710
H	1.78715	-2.30341	2.44923
H	1.31333	-0.69917	1.86017
H	3.14628	1.07258	1.31876
H	3.92316	2.20796	0.20811
H	-1.02672	-1.52281	1.88502
H	-0.56607	-3.18724	2.28991
H	2.16996	3.84545	0.63935
H	1.34448	2.72229	1.73931
H	-1.37461	-3.96254	0.08142
H	-2.65769	-3.17648	1.01590
H	-0.16647	4.43318	0.70421
H	0.18751	3.74157	-0.89250
H	-3.93466	-2.53649	-0.79047

H	-3.24875	-1.41986	-1.97804
H	-2.23959	3.05472	-1.08477
H	-2.50094	3.80846	0.50145
H	-4.75826	-0.29013	-0.33764
H	-3.65536	-0.74784	0.97332
H	-3.08076	1.56339	1.43990
H	-4.19666	2.11883	0.17475
C	2.78037	-1.75519	-1.74170
H	2.04933	-2.55529	-1.65937
H	3.77351	-2.20676	-1.87663
H	2.55005	-1.17114	-2.63137
K	-0.10414	0.05816	-0.84375

ACE4 K MP2 def2tzv

47

O	-0.58424	-1.49460	-0.00691
O	2.90400	0.76994	-0.20634
O	-3.27902	-0.92618	-0.63473
O	0.31387	2.28565	-0.53266
O	-2.22140	1.51299	0.58255
N	2.65980	-2.23562	-0.01910
C	1.57146	-2.34892	0.97570
C	3.81482	-1.41583	0.41033
C	0.51471	-1.22161	0.94905
C	3.54723	0.01967	0.88401
C	-1.59885	-2.42789	0.49827
C	2.70511	2.18917	0.12753
C	-2.83778	-2.30935	-0.39081
C	1.67982	2.75308	-0.84421
C	-3.92591	-0.23432	0.49061
C	-0.38110	3.10090	0.48471

C	-2.95409	0.52296	1.38797
C	-1.19081	2.19171	1.38652
H	2.03830	-2.38588	1.96834
H	1.06209	-3.30957	0.82074
H	4.52409	-1.38694	-0.42427
H	4.30464	-1.93575	1.24614
H	0.07930	-1.08521	1.95034
H	0.94205	-0.28271	0.59704
H	2.89538	0.03522	1.76934
H	4.50188	0.49517	1.15197
H	-1.82584	-2.18217	1.54268
H	-1.24077	-3.46439	0.45804
H	3.65718	2.72771	0.03463
H	2.34591	2.27764	1.16208
H	-2.63176	-2.70131	-1.38778
H	-3.65884	-2.88095	0.06166
H	1.70600	3.84892	-0.82558
H	1.89064	2.40226	-1.85565
H	-4.48093	-0.95911	1.10192
H	-4.63107	0.46172	0.03296
H	-1.01973	3.83372	-0.01857
H	0.35333	3.62892	1.10514
H	-3.52636	1.03454	2.17446
H	-2.23442	-0.15055	1.86025
H	-0.53271	1.44426	1.84446
H	-1.66990	2.78655	2.17524
C	2.18684	-2.00873	-1.40385
H	1.52083	-2.83072	-1.68684
H	3.05350	-2.01339	-2.07183
H	1.64977	-1.06139	-1.53632

K	-1.23924	0.48712	-1.91932

ACE4 K PBE0 def2sv

47

O	0.55068	-2.14613	0.81858
O	1.81963	1.86124	-0.04780
O	-1.85850	-2.09077	-0.53211
O	-0.92309	2.40450	0.37020
O	-2.86411	0.53545	-0.43155
N	2.73042	-0.87643	-0.56781
C	2.86027	-1.60380	0.69203
C	3.54789	0.32038	-0.64873
C	1.60769	-1.57579	1.54793
C	3.11630	1.43150	0.28526
C	-0.64899	-2.27664	1.53338
C	1.36880	2.93701	0.73408
C	-1.69233	-2.88296	0.62348
C	0.03713	3.41859	0.22025
C	-3.17699	-1.76724	-0.90465
C	-2.21415	2.78895	-0.02903
C	-3.70157	-0.55865	-0.16105
C	-3.18960	1.68888	0.29875
H	3.70044	-1.20840	1.29448
H	3.10607	-2.66062	0.48488
H	3.48800	0.70664	-1.68088
H	4.62344	0.10635	-0.44827
H	1.78854	-2.14876	2.48085
H	1.35282	-0.53565	1.84217
H	3.15088	1.10302	1.34449
H	3.83318	2.27176	0.18577
H	-0.99340	-1.28641	1.90015

H	-0.51293	-2.93228	2.41825
H	2.08783	3.77972	0.68810
H	1.27348	2.63041	1.79585
H	-1.37320	-3.89825	0.31986
H	-2.63776	-2.97926	1.18748
H	-0.25807	4.32051	0.79353
H	0.12983	3.71078	-0.84598
H	-3.85998	-2.62415	-0.75219
H	-3.14955	-1.54653	-1.98501
H	-2.23580	3.00456	-1.11719
H	-2.52817	3.70895	0.50379
H	-4.74174	-0.35019	-0.48635
H	-3.72976	-0.75790	0.93048
H	-3.15713	1.47596	1.38685
H	-4.21357	2.03482	0.05122
C	2.89055	-1.72927	-1.72958
H	2.18002	-2.57052	-1.67718
H	3.91767	-2.14986	-1.82712
H	2.67058	-1.15942	-2.64902
K	-0.08693	0.02262	-0.96608

ACE4 K PBE0 def2tzv

47

O	0.49777	-2.28578	0.78311
O	1.92050	1.88412	-0.12406
O	-1.94332	-2.08250	-0.62242
O	-0.83501	2.48575	0.40942
O	-2.85949	0.64343	-0.41453
N	2.70343	-0.94726	-0.53328
C	2.82372	-1.68338	0.73822
C	3.59166	0.21620	-0.63435

C	1.55395	-1.67933	1.56589
C	3.20802	1.35923	0.27969
C	-0.76107	-2.39115	1.48449
C	1.49337	3.00246	0.68636
C	-1.78521	-2.95281	0.53027
C	0.16952	3.50116	0.17838
C	-3.29469	-1.65636	-0.92125
C	-2.16074	2.90152	0.00593
C	-3.72636	-0.46888	-0.09537
C	-3.14003	1.82255	0.37403
H	3.62580	-1.25989	1.35395
H	3.10536	-2.72264	0.53747
H	3.56046	0.57670	-1.66505
H	4.64079	-0.05145	-0.41714
H	1.71082	-2.24798	2.49048
H	1.26930	-0.65502	1.83802
H	3.16334	1.03813	1.32732
H	3.96715	2.14668	0.20412
H	-1.07403	-1.40498	1.84854
H	-0.66234	-3.06060	2.34729
H	2.22751	3.81419	0.62474
H	1.40423	2.69335	1.73397
H	-1.46232	-3.93268	0.17114
H	-2.73467	-3.07368	1.06006
H	-0.09608	4.41966	0.71378
H	0.23369	3.72837	-0.89168
H	-4.00130	-2.47919	-0.77493
H	-3.29240	-1.38862	-1.97795
H	-2.18340	3.08685	-1.07386
H	-2.43812	3.82691	0.52328

H	-4.76665	-0.22012	-0.33667
H	-3.66896	-0.69061	0.97722
H	-3.05651	1.58593	1.44064
H	-4.15774	2.17625	0.17398
C	2.81231	-1.82637	-1.70202
H	2.07121	-2.62213	-1.63133
H	3.80831	-2.28977	-1.79460
H	2.61782	-1.25800	-2.61355
K	-0.08871	0.04557	-0.92895

ACE4 K PBE def2sv

47

O	0.46132	-2.17332	0.81138
O	1.91213	1.83580	-0.08202
O	-1.96287	-2.08837	-0.59875
O	-0.83088	2.46078	0.37513
O	-2.85107	0.62734	-0.40182
N	2.72364	-0.96623	-0.53253
C	2.80366	-1.67962	0.75325
C	3.60082	0.20141	-0.62470
C	1.51879	-1.61264	1.57701
C	3.20067	1.35899	0.28169
C	-0.76723	-2.29853	1.50884
C	1.49546	2.95348	0.68721
C	-1.79665	-2.90025	0.56409
C	0.16075	3.45752	0.17684
C	-3.28893	-1.67171	-0.90558
C	-2.13298	2.88077	-0.00564
C	-3.72896	-0.44919	-0.11146
C	-3.13268	1.79860	0.34809
H	3.63648	-1.28253	1.38024

H	3.04132	-2.75241	0.57142
H	3.57524	0.56589	-1.67536
H	4.67267	-0.05301	-0.39712
H	1.65923	-2.18445	2.52881
H	1.27788	-0.55413	1.85445
H	3.20985	1.05391	1.35856
H	3.95929	2.17382	0.17072
H	-1.11589	-1.30010	1.87891
H	-0.65300	-2.96710	2.39966
H	2.24032	3.78455	0.61085
H	1.40762	2.67303	1.76699
H	-1.45096	-3.91089	0.24125
H	-2.75683	-3.02588	1.11440
H	-0.10361	4.39090	0.73345
H	0.24183	3.71994	-0.90823
H	-4.02057	-2.49893	-0.74547
H	-3.28715	-1.42300	-1.98948
H	-2.17094	3.09492	-1.10366
H	-2.41754	3.81835	0.53348
H	-4.78194	-0.19079	-0.39119
H	-3.72245	-0.66922	0.98642
H	-3.07955	1.57977	1.44420
H	-4.16245	2.17406	0.12500
C	2.86529	-1.85543	-1.68337
H	2.11183	-2.66938	-1.62355
H	3.88272	-2.32720	-1.75989
H	2.68179	-1.28950	-2.62354
K	-0.08198	0.02287	-0.99909

ACE4 K PBE def2tzv

47

O	0.46114	-2.32210	0.77887
O	1.97109	1.88639	-0.14694
O	-2.00362	-2.09521	-0.65536
O	-0.79973	2.51554	0.42698
O	-2.87118	0.68815	-0.41933
N	2.71828	-0.98911	-0.52154
C	2.81318	-1.73161	0.76572
C	3.63770	0.16918	-0.61187
C	1.52656	-1.70850	1.58450
C	3.25915	1.33248	0.29350
C	-0.82112	-2.40339	1.48711
C	1.55432	3.02922	0.67357
C	-1.84898	-2.96815	0.52527
C	0.22393	3.53653	0.16753
C	-3.37166	-1.62426	-0.91789
C	-2.13946	2.95590	0.01668
C	-3.76397	-0.42428	-0.07500
C	-3.13507	1.88312	0.39138
H	3.61836	-1.31345	1.39580
H	3.08628	-2.78233	0.56866
H	3.63152	0.52393	-1.65334
H	4.68694	-0.11664	-0.37184
H	1.66759	-2.27762	2.52133
H	1.24270	-0.67242	1.84613
H	3.18541	1.01908	1.35055
H	4.04039	2.11071	0.22527
H	-1.12317	-1.40170	1.84270
H	-0.73291	-3.07185	2.36297
H	2.30091	3.84029	0.60049
H	1.47108	2.72291	1.73134

H	-1.52311	-3.95576	0.16492
H	-2.80879	-3.08607	1.05307
H	-0.03188	4.47036	0.69868
H	0.27696	3.75040	-0.91453
H	-4.09919	-2.43748	-0.75832
H	-3.38032	-1.35211	-1.98178
H	-2.15856	3.14163	-1.07168
H	-2.40564	3.89254	0.53778
H	-4.81250	-0.15328	-0.29643
H	-3.68703	-0.64747	1.00449
H	-3.04607	1.63529	1.46368
H	-4.15768	2.25289	0.19873
C	2.84445	-1.88275	-1.69498
H	2.08704	-2.67437	-1.63534
H	3.84424	-2.36261	-1.76716
H	2.67347	-1.30983	-2.61740
K	-0.08472	0.04292	-0.96605

ACE4 K B2PLYP def2sv

47

O	-0.53419	-2.15223	-0.82309
O	-1.84137	1.85231	0.05626
O	1.87173	-2.08528	0.54125
O	0.89945	2.41685	-0.37145
O	2.85675	0.55404	0.43034
N	-2.71314	-0.89675	0.56921
C	-2.85169	-1.62426	-0.69847
C	-3.55626	0.28977	0.66323
C	-1.59839	-1.57916	-1.55855
C	-3.14505	1.41033	-0.27466
C	0.67629	-2.27354	-1.53987

C	-1.40156	2.94047	-0.73196
C	1.71489	-2.88497	-0.62258
C	-0.07187	3.43267	-0.21357
C	3.19698	-1.74956	0.91580
C	2.19689	2.81184	0.03099
C	3.71184	-0.53931	0.16155
C	3.17750	1.71542	-0.30794
H	-3.69704	-1.22986	-1.29205
H	-3.08573	-2.68312	-0.49489
H	-3.49179	0.67432	1.69420
H	-4.62772	0.05637	0.46941
H	-1.77017	-2.14764	-2.49420
H	-1.34964	-0.53603	-1.83967
H	-3.17953	1.08490	-1.33260
H	-3.86576	2.24438	-0.16701
H	1.01875	-1.28003	-1.89350
H	0.54378	-2.92249	-2.42839
H	-2.13017	3.77280	-0.68281
H	-1.30238	2.63361	-1.79133
H	1.39064	-3.89675	-0.31925
H	2.66551	-2.98031	-1.17508
H	0.22060	4.33664	-0.78213
H	-0.16601	3.71459	0.85330
H	3.88254	-2.60311	0.76612
H	3.16469	-1.52149	1.99276
H	2.21795	3.01774	1.11910
H	2.50010	3.73594	-0.49792
H	4.74767	-0.31572	0.48588
H	3.73859	-0.74251	-0.92718
H	3.13606	1.50187	-1.39363

H	4.20104	2.06120	-0.06482
C	-2.86027	-1.76482	1.73169
H	-2.14305	-2.59734	1.66399
H	-3.88261	-2.19293	1.82914
H	-2.63899	-1.19871	2.65155
K	0.09157	0.02611	0.95085

ACE4 K B2PLYP def2tzv

47

O	0.49977	-2.28723	0.77850
O	1.92957	1.89070	-0.12043
O	-1.95060	-2.08954	-0.62898
O	-0.83715	2.49950	0.39475
O	-2.87328	0.64460	-0.40625
N	2.71347	-0.95099	-0.53654
C	2.84262	-1.69992	0.74120
C	3.61633	0.21732	-0.63155
C	1.56919	-1.68220	1.57593
C	3.22914	1.35831	0.29602
C	-0.76929	-2.41425	1.49210
C	1.49917	3.02740	0.69265
C	-1.78895	-2.98327	0.52661
C	0.17703	3.52890	0.16355
C	-3.32075	-1.66419	-0.92774
C	-2.18149	2.92091	-0.00084
C	-3.75203	-0.47943	-0.08515
C	-3.15605	1.83698	0.39204
H	3.64983	-1.28046	1.35081
H	3.11144	-2.73965	0.53488
H	3.58649	0.58305	-1.65844
H	4.66038	-0.05983	-0.40948

H	1.71385	-2.25574	2.49686
H	1.28940	-0.65755	1.84081
H	3.16977	1.03111	1.33805
H	3.98259	2.14893	0.22888
H	-1.08965	-1.43441	1.85857
H	-0.65461	-3.08943	2.34562
H	2.24026	3.82957	0.62858
H	1.39819	2.71924	1.73678
H	-1.45729	-3.95276	0.15536
H	-2.74092	-3.11009	1.04665
H	-0.10031	4.44401	0.69425
H	0.24934	3.74538	-0.90549
H	-4.01608	-2.49356	-0.77951
H	-3.31646	-1.39191	-1.98073
H	-2.21400	3.09499	-1.07954
H	-2.44476	3.84737	0.51724
H	-4.78884	-0.22137	-0.32162
H	-3.68382	-0.70477	0.98324
H	-3.05401	1.60238	1.45480
H	-4.17681	2.17836	0.19840
C	2.83146	-1.83304	-1.71956
H	2.10220	-2.63647	-1.64236
H	3.83358	-2.27798	-1.81478
H	2.62305	-1.26079	-2.62342
K	-0.09431	0.04915	-0.93004

ACE4 K DSDPBEP86 def2sv

47

O	0.29533	-2.09286	0.87854
O	2.02539	1.69226	-0.17008
O	-2.02603	-1.98663	-0.63851

O	-0.62733	2.44813	0.37813
O	-2.71953	0.73656	-0.38337
N	2.56403	-1.12459	-0.48797
C	2.63266	-1.73544	0.84173
C	3.54537	-0.07302	-0.70492
C	1.35152	-1.53267	1.63099
C	3.29254	1.15410	0.14820
C	-0.96006	-2.07655	1.51998
C	1.71063	2.83372	0.59737
C	-1.95863	-2.71433	0.57860
C	0.39121	3.39544	0.13307
C	-3.30151	-1.48413	-0.98926
C	-1.91250	2.93116	0.04748
C	-3.68766	-0.26855	-0.17268
C	-2.93862	1.88680	0.40501
H	3.47881	-1.32570	1.42494
H	2.81732	-2.82255	0.75010
H	3.49133	0.23290	-1.76498
H	4.58825	-0.42121	-0.51689
H	1.42901	-2.02865	2.62096
H	1.16794	-0.45066	1.80611
H	3.33821	0.90884	1.22917
H	4.08688	1.90277	-0.05115
H	-1.26496	-1.03440	1.75710
H	-0.92458	-2.64741	2.47121
H	2.49319	3.61094	0.47857
H	1.64791	2.56932	1.67301
H	-1.63257	-3.74626	0.35126
H	-2.94754	-2.76749	1.06981
H	0.18584	4.33519	0.68515

H	0.44306	3.63561	-0.94885
H	-4.07943	-2.26577	-0.88469
H	-3.23670	-1.20311	-2.05376
H	-1.97187	3.16164	-1.03615
H	-2.13310	3.86105	0.61030
H	-4.69233	0.08263	-0.48748
H	-3.74565	-0.52172	0.90620
H	-2.85993	1.63477	1.48237
H	-3.95130	2.29854	0.21883
C	2.56677	-2.11177	-1.55685
H	1.75084	-2.83452	-1.38949
H	3.52588	-2.67476	-1.62713
H	2.39011	-1.61270	-2.52647
K	-0.06925	0.02035	-0.95906

ACE4 K DSDPBEP86 def2tzv

47

O	0.27480	-2.19539	0.84992
O	2.09119	1.72704	-0.22173
O	-2.08214	-1.99317	-0.70422
O	-0.59142	2.50628	0.39975
O	-2.74823	0.80077	-0.37873
N	2.57591	-1.16092	-0.47197
C	2.64152	-1.80048	0.86568
C	3.60007	-0.11693	-0.67438
C	1.35065	-1.61424	1.65091
C	3.35605	1.11540	0.18179
C	-1.03014	-2.17738	1.50093
C	1.77660	2.90918	0.57478
C	-2.01868	-2.78935	0.52757
C	0.46073	3.47350	0.09548

C	-3.39635	-1.42819	-1.01130
C	-1.92367	3.01484	0.08304
C	-3.73840	-0.24178	-0.12989
C	-2.93643	1.96754	0.47907
H	3.46584	-1.37901	1.45508
H	2.84228	-2.87443	0.76002
H	3.57766	0.17851	-1.72760
H	4.61675	-0.49222	-0.45332
H	1.41018	-2.12191	2.62223
H	1.15012	-0.54729	1.81917
H	3.31093	0.86300	1.24886
H	4.17331	1.83256	0.03324
H	-1.31397	-1.14547	1.74728
H	-1.00104	-2.76837	2.42508
H	2.56392	3.66309	0.44991
H	1.70773	2.63497	1.63464
H	-1.69689	-3.79690	0.25390
H	-3.00659	-2.84784	0.99750
H	0.25809	4.41646	0.61777
H	0.49271	3.66120	-0.98444
H	-4.17124	-2.19663	-0.90877
H	-3.33825	-1.11704	-2.05497
H	-1.99555	3.22949	-0.98986
H	-2.11188	3.93654	0.64710
H	-4.74119	0.12512	-0.38298
H	-3.72570	-0.51516	0.93286
H	-2.80109	1.68187	1.52912
H	-3.94793	2.36900	0.34259
C	2.57654	-2.15319	-1.56735
H	1.76269	-2.86186	-1.40620

H	3.52631	-2.71224	-1.62875
H	2.41378	-1.64328	-2.52073
K	-0.06931	0.04402	-0.95647

ACE4 K HSE06 def2sv

47

O	0.55334	-2.14459	0.81950
O	1.81810	1.86100	-0.04883
O	-1.85449	-2.09011	-0.53147
O	-0.92440	2.40348	0.37000
O	-2.86434	0.53405	-0.43324
N	2.72884	-0.87539	-0.56936
C	2.86237	-1.60032	0.69169
C	3.54596	0.32143	-0.65486
C	1.61057	-1.57299	1.54872
C	3.11695	1.43260	0.28016
C	-0.64660	-2.27684	1.53470
C	1.36754	2.93640	0.73489
C	-1.68883	-2.88374	0.62408
C	0.03599	3.41855	0.22148
C	-3.17363	-1.76916	-0.90665
C	-2.21612	2.78794	-0.02944
C	-3.70085	-0.56171	-0.16335
C	-3.19100	1.68736	0.29792
H	3.70237	-1.20164	1.29220
H	3.11037	-2.65703	0.48628
H	3.48217	0.70673	-1.68715
H	4.62234	0.10781	-0.45809
H	1.79174	-2.14533	2.48189
H	1.35494	-0.53281	1.84224
H	3.15514	1.10420	1.33933

H	3.83229	2.27382	0.17846
H	-0.99197	-1.28697	1.90156
H	-0.50951	-2.93242	2.41936
H	2.08698	3.77871	0.68975
H	1.27243	2.62824	1.79618
H	-1.36866	-3.89847	0.31992
H	-2.63482	-2.98093	1.18690
H	-0.26002	4.31958	0.79564
H	0.12872	3.71178	-0.84440
H	-3.85491	-2.62749	-0.75520
H	-3.14476	-1.54820	-1.98686
H	-2.23744	3.00375	-1.11752
H	-2.52999	3.70781	0.50356
H	-4.74108	-0.35433	-0.48896
H	-3.72908	-0.76106	0.92817
H	-3.15842	1.47364	1.38583
H	-4.21524	2.03237	0.05046
C	2.88414	-1.73049	-1.73028
H	2.17358	-2.57133	-1.67350
H	3.91075	-2.15170	-1.83071
H	2.66116	-1.16227	-2.65000
K	-0.08818	0.02350	-0.95905

ACE4 K HSE06 def2tzv

47

O	0.49836	-2.28490	0.78401
O	1.92069	1.88363	-0.12492
O	-1.94216	-2.08276	-0.62296
O	-0.83459	2.48613	0.40880
O	-2.85854	0.64336	-0.41474
N	2.70229	-0.94826	-0.53428

C	2.82448	-1.68282	0.73816
C	3.59057	0.21505	-0.63879
C	1.55569	-1.67772	1.56676
C	3.20999	1.35792	0.27622
C	-0.76168	-2.39014	1.48556
C	1.49415	3.00253	0.68693
C	-1.78446	-2.95314	0.53129
C	0.17116	3.50219	0.17878
C	-3.29442	-1.65626	-0.92314
C	-2.16124	2.90231	0.00472
C	-3.72633	-0.46992	-0.09655
C	-3.14016	1.82379	0.37350
H	3.62703	-1.25830	1.35205
H	3.10620	-2.72198	0.53851
H	3.55629	0.57511	-1.66922
H	4.64008	-0.05292	-0.42496
H	1.71199	-2.24563	2.49146
H	1.27104	-0.65346	1.83765
H	3.16706	1.03683	1.32360
H	3.96844	2.14536	0.19928
H	-1.07465	-1.40385	1.84823
H	-0.66249	-3.05830	2.34885
H	2.22915	3.81304	0.62587
H	1.40490	2.69238	1.73385
H	-1.46076	-3.93247	0.17260
H	-2.73439	-3.07416	1.05948
H	-0.09501	4.41996	0.71441
H	0.23552	3.72967	-0.89084
H	-4.00028	-2.47948	-0.77807
H	-3.29073	-1.38803	-1.97932

H	-2.18329	3.08683	-1.07485
H	-2.43767	3.82794	0.52135
H	-4.76607	-0.22024	-0.33772
H	-3.66903	-0.69209	0.97561
H	-3.05694	1.58758	1.43986
H	-4.15777	2.17618	0.17271
C	2.80622	-1.82857	-1.70274
H	2.06519	-2.62360	-1.62854
H	3.80142	-2.29251	-1.79843
H	2.60889	-1.26106	-2.61381
K	-0.08916	0.04579	-0.92344

ACE4 K M062X def2sv

47

O	0.26131	-2.02558	0.89099
O	2.03168	1.65445	-0.18367
O	-2.02628	-1.97211	-0.65202
O	-0.59723	2.43430	0.35549
O	-2.68716	0.73704	-0.36354
N	2.54697	-1.15654	-0.47219
C	2.60034	-1.72659	0.87447
C	3.53721	-0.12146	-0.71510
C	1.31601	-1.47646	1.64523
C	3.29850	1.11916	0.12109
C	-0.99572	-2.01754	1.51895
C	1.73997	2.81397	0.55719
C	-1.97186	-2.68081	0.57139
C	0.41853	3.37554	0.09949
C	-3.29031	-1.46149	-1.01388
C	-1.88284	2.92778	0.06262
C	-3.67232	-0.25187	-0.18707

C	-2.90608	1.88268	0.42386
H	3.44991	-1.31503	1.44718
H	2.76232	-2.81752	0.81477
H	3.47862	0.16603	-1.77843
H	4.57429	-0.47810	-0.52690
H	1.36642	-1.95262	2.64361
H	1.15688	-0.38665	1.79395
H	3.35621	0.88846	1.20379
H	4.08858	1.86325	-0.09749
H	-1.31514	-0.97637	1.73880
H	-0.96048	-2.57477	2.47515
H	2.52681	3.57792	0.40795
H	1.69163	2.57422	1.63770
H	-1.62517	-3.70805	0.36404
H	-2.96783	-2.74354	1.04298
H	0.21748	4.31614	0.64678
H	0.46065	3.60771	-0.98288
H	-4.07174	-2.23783	-0.92127
H	-3.21524	-1.17114	-2.07344
H	-1.96201	3.17389	-1.01456
H	-2.08421	3.84829	0.64305
H	-4.66250	0.12118	-0.51323
H	-3.75190	-0.51959	0.88606
H	-2.82438	1.62814	1.49882
H	-3.91812	2.29074	0.24071
C	2.53507	-2.17033	-1.51320
H	1.70981	-2.87593	-1.32941
H	3.48469	-2.74709	-1.56268
H	2.37065	-1.69544	-2.49488
K	-0.07277	0.01785	-0.94384

ACE4 K M062X def2tzv

47

O	0.31948	-2.07511	0.91725
O	2.01924	1.70541	-0.19449
O	-1.99552	-2.03439	-0.63675
O	-0.65395	2.44823	0.37545
O	-2.73077	0.70805	-0.41585
N	2.57973	-1.12996	-0.51218
C	2.66689	-1.72497	0.83578
C	3.55838	-0.06266	-0.75421
C	1.41139	-1.46328	1.64693
C	3.31727	1.14662	0.12517
C	-0.97196	-2.01688	1.56302
C	1.69462	2.85661	0.61998
C	-1.93889	-2.72479	0.64228
C	0.38847	3.42595	0.13813
C	-3.30084	-1.53388	-1.01877
C	-1.96642	2.93577	0.00449
C	-3.70816	-0.33517	-0.19363
C	-2.98281	1.89215	0.37775
H	3.53143	-1.33448	1.37954
H	2.80597	-2.80557	0.75416
H	3.47571	0.24447	-1.79720
H	4.58970	-0.41100	-0.58974
H	1.48872	-1.90373	2.64425
H	1.23424	-0.38589	1.74692
H	3.34404	0.88023	1.18608
H	4.09207	1.89581	-0.05602
H	-1.27343	-0.97390	1.71458
H	-0.93540	-2.51795	2.53346

H	2.47831	3.61259	0.52615
H	1.61542	2.55580	1.66804
H	-1.58869	-3.73886	0.45614
H	-2.92759	-2.77294	1.10147
H	0.16452	4.34507	0.68479
H	0.44415	3.65213	-0.92966
H	-4.05249	-2.32029	-0.92530
H	-3.21211	-1.25165	-2.06504
H	-1.99679	3.12920	-1.07070
H	-2.18236	3.86425	0.53853
H	-4.69881	0.00695	-0.50312
H	-3.74455	-0.58097	0.87211
H	-2.90413	1.64568	1.43967
H	-3.98759	2.26976	0.17375
C	2.56381	-2.13557	-1.58323
H	1.75089	-2.83654	-1.40481
H	3.50593	-2.69826	-1.64332
H	2.39482	-1.64652	-2.54213
K	-0.08044	0.02155	-0.89971

ACE4 K M06 def2sv

47

O	0.26469	-2.05159	0.89705
O	2.05742	1.67916	-0.15643
O	-2.09006	-2.00089	-0.60181
O	-0.60825	2.42445	0.37157
O	-2.72640	0.74127	-0.39236
N	2.58696	-1.14607	-0.47993
C	2.60530	-1.75217	0.84846
C	3.56614	-0.09320	-0.67049
C	1.33261	-1.50501	1.62701

C	3.30555	1.12711	0.17676
C	-0.98346	-1.97602	1.53013
C	1.72421	2.78941	0.63281
C	-1.99595	-2.65521	0.64452
C	0.41746	3.36266	0.16909
C	-3.35202	-1.47820	-0.94099
C	-1.87321	2.91814	0.01309
C	-3.69792	-0.24199	-0.15191
C	-2.92541	1.90755	0.36219
H	3.46764	-1.39038	1.44390
H	2.74397	-2.84831	0.75782
H	3.53620	0.21500	-1.73389
H	4.60774	-0.44518	-0.46857
H	1.39797	-1.97960	2.63106
H	1.17080	-0.41242	1.79127
H	3.33200	0.87968	1.26232
H	4.11283	1.87264	0.00521
H	-1.27172	-0.91064	1.70141
H	-0.95178	-2.47631	2.52297
H	2.51076	3.57153	0.55787
H	1.64844	2.49127	1.70292
H	-1.67559	-3.70200	0.47068
H	-2.97681	-2.68848	1.15835
H	0.20622	4.29513	0.73599
H	0.48392	3.63378	-0.90840
H	-4.14823	-2.23963	-0.80605
H	-3.30464	-1.22880	-2.01683
H	-1.90574	3.14110	-1.07706
H	-2.08816	3.86500	0.55416
H	-4.70673	0.11959	-0.44915

H	-3.74365	-0.47174	0.93722
H	-2.87941	1.67273	1.44932
H	-3.92841	2.33970	0.15584
C	2.61101	-2.12070	-1.55067
H	1.79368	-2.85297	-1.41767
H	3.57232	-2.68600	-1.60608
H	2.45868	-1.61958	-2.52576
K	-0.06085	-0.00821	-1.08176

ACE4 K M06 def2tzv

47

O	0.26831	-2.09598	0.91622
O	2.09091	1.68751	-0.19958
O	-2.08501	-2.00814	-0.62497
O	-0.59257	2.44204	0.40046
O	-2.74247	0.77092	-0.41721
N	2.57829	-1.16670	-0.48235
C	2.62143	-1.77628	0.85557
C	3.58131	-0.12636	-0.70885
C	1.36257	-1.51269	1.65255
C	3.35898	1.09597	0.14878
C	-1.01729	-1.98070	1.55247
C	1.75958	2.81216	0.63680
C	-2.01210	-2.67446	0.65865
C	0.46318	3.39695	0.16353
C	-3.37407	-1.46222	-0.97544
C	-1.88766	2.95662	0.02753
C	-3.72898	-0.24585	-0.16067
C	-2.93230	1.94697	0.39430
H	3.48187	-1.40795	1.42455
H	2.75278	-2.85991	0.76365

H	3.52528	0.17319	-1.75852
H	4.60606	-0.49427	-0.52748
H	1.43060	-1.96539	2.64886
H	1.19882	-0.43172	1.77674
H	3.36387	0.84175	1.21668
H	4.16559	1.81860	-0.02160
H	-1.28407	-0.92123	1.68267
H	-1.00168	-2.45594	2.53992
H	2.54631	3.57300	0.57727
H	1.67079	2.48657	1.68078
H	-1.68758	-3.70219	0.48441
H	-2.99228	-2.70075	1.14250
H	0.25814	4.32467	0.70954
H	0.52122	3.63037	-0.90626
H	-4.15702	-2.21901	-0.86164
H	-3.30107	-1.19444	-2.02932
H	-1.91332	3.15657	-1.05032
H	-2.08625	3.89479	0.55788
H	-4.72589	0.10957	-0.44575
H	-3.74958	-0.47976	0.91171
H	-2.85057	1.68250	1.45560
H	-3.92759	2.37007	0.21821
C	2.52266	-2.14994	-1.56441
H	1.66988	-2.81228	-1.41164
H	3.43371	-2.76601	-1.62802
H	2.39215	-1.63871	-2.52057
K	-0.06344	0.00542	-1.04935

ACE4 K MP2 def2sv

47

O	-0.33732	-2.07958	-0.88543

O	-1.98934	1.71149	0.14910
O	1.99655	-2.01629	0.60769
O	0.67961	2.42769	-0.38351
O	2.74374	0.69159	0.40173
N	-2.58957	-1.08662	0.50061
C	-2.66746	-1.70423	-0.82481
C	-3.54905	-0.01176	0.69781
C	-1.39472	-1.49680	-1.62293
C	-3.26701	1.19547	-0.17193
C	0.91339	-2.05309	-1.54032
C	-1.64964	2.83946	-0.63150
C	1.91568	-2.71874	-0.62506
C	-0.33170	3.38919	-0.15312
C	3.28548	-1.54885	0.96506
C	1.96363	2.89629	-0.02034
C	3.69436	-0.32820	0.17009
C	2.98464	1.84783	-0.37457
H	-3.52485	-1.30833	-1.40235
H	-2.83788	-2.79251	-0.72633
H	-3.49241	0.31126	1.75237
H	-4.59823	-0.34115	0.51018
H	-1.48416	-1.97726	-2.61956
H	-1.20463	-0.41425	-1.78118
H	-3.31234	0.93607	-1.24889
H	-4.04669	1.96297	0.01256
H	1.22280	-1.00768	-1.75244
H	0.86600	-2.59969	-2.50509
H	-2.42368	3.62782	-0.53479
H	-1.57227	2.56028	-1.70186
H	1.58586	-3.75367	-0.41904

H	2.89952	-2.76582	-1.12653
H	-0.10892	4.32466	-0.70530
H	-0.39670	3.63276	0.92675
H	4.04591	-2.34502	0.84465
H	3.22878	-1.28542	2.03403
H	2.00582	3.11012	1.06698
H	2.20332	3.83154	-0.56570
H	4.70611	-0.00226	0.48890
H	3.74504	-0.56343	-0.91244
H	2.91544	1.60799	-1.45471
H	3.99861	2.24823	-0.17236
C	-2.64062	-2.06844	1.57424
H	-1.84432	-2.81508	1.42235
H	-3.61680	-2.60093	1.63109
H	-2.46367	-1.57001	2.54316
K	0.07071	0.01512	1.00626

ACE4 K SCS-MP2 def2sv

47

O	-0.44434	-2.12654	-0.86563
O	-1.91506	1.78773	0.10306
O	1.92463	-2.05511	0.56166
O	0.79627	2.41906	-0.38959
O	2.80304	0.61588	0.43029
N	-2.64569	-0.98766	0.54605
C	-2.76930	-1.67192	-0.74869
C	-3.55364	0.14561	0.69281
C	-1.51181	-1.54257	-1.59374
C	-3.20915	1.30721	-0.22307
C	0.78928	-2.13829	-1.55893
C	-1.52243	2.88989	-0.69560

C	1.82014	-2.78563	-0.65608
C	-0.19625	3.41263	-0.19586
C	3.23494	-1.66504	0.94499
C	2.08561	2.85027	0.01287
C	3.71122	-0.44294	0.18221
C	3.09066	1.77618	-0.32944
H	-3.63179	-1.28348	-1.32605
H	-2.96386	-2.74919	-0.58843
H	-3.48558	0.50869	1.73429
H	-4.61688	-0.14079	0.51208
H	-1.65058	-2.06583	-2.56365
H	-1.29170	-0.47569	-1.80983
H	-3.24675	1.00761	-1.29039
H	-3.96068	2.11170	-0.08046
H	1.10241	-1.10348	-1.81547
H	0.70586	-2.71619	-2.50359
H	-2.27475	3.70307	-0.63192
H	-1.42995	2.58480	-1.75820
H	1.50736	-3.82163	-0.42392
H	2.79069	-2.82878	-1.18462
H	0.06472	4.33292	-0.75803
H	-0.27823	3.67435	0.87915
H	3.95465	-2.49683	0.81203
H	3.17984	-1.42527	2.02062
H	2.10438	3.05183	1.10382
H	2.36332	3.78464	-0.51699
H	4.73327	-0.17062	0.52047
H	3.76213	-0.65443	-0.90572
H	3.03829	1.54865	-1.41375
H	4.11060	2.14828	-0.10045

C	-2.74494	-1.91214	1.67280
H	-1.98564	-2.70509	1.56214
H	-3.74690	-2.39236	1.75476
H	-2.54319	-1.37280	2.61588
K	0.08139	0.02063	0.97842

ACE4 K SCS-MP2 def2tzv

47

O	-0.57267	-1.52318	0.01152
O	2.90339	0.79887	-0.20261
O	-3.26683	-0.95091	-0.64578
O	0.29891	2.29847	-0.52586
O	-2.24869	1.51228	0.56188
N	2.69162	-2.22737	-0.02050
C	1.60300	-2.36361	0.97514
C	3.83599	-1.38749	0.40837
C	0.52720	-1.24817	0.96583
C	3.55455	0.04944	0.88377
C	-1.59756	-2.44242	0.52293
C	2.69397	2.21580	0.13608
C	-2.82932	-2.33228	-0.38334
C	1.66090	2.77822	-0.83533
C	-3.94257	-0.25022	0.45783
C	-0.41645	3.11733	0.47457
C	-2.99275	0.52963	1.36681
C	-1.23531	2.20715	1.37470
H	2.07134	-2.40551	1.96755
H	1.10587	-3.32929	0.80916
H	4.54460	-1.35046	-0.42734
H	4.33457	-1.90056	1.24404
H	0.09588	-1.12873	1.97124

H	0.94141	-0.29900	0.62577
H	2.90589	0.05903	1.77184
H	4.50747	0.53109	1.14947
H	-1.83525	-2.17926	1.56095
H	-1.24276	-3.48136	0.50292
H	3.64262	2.76159	0.04206
H	2.33584	2.29965	1.17171
H	-2.60930	-2.73409	-1.37439
H	-3.65577	-2.90169	0.06302
H	1.67965	3.87470	-0.81237
H	1.87516	2.43413	-1.84926
H	-4.50563	-0.97084	1.06716
H	-4.64339	0.43523	-0.02385
H	-1.05442	3.84031	-0.04530
H	0.30432	3.65794	1.10087
H	-3.58625	1.05046	2.13195
H	-2.27973	-0.13212	1.86650
H	-0.57863	1.46907	1.85104
H	-1.73162	2.80659	2.15002
C	2.21656	-2.00856	-1.40818
H	1.55329	-2.83547	-1.68612
H	3.08273	-2.01417	-2.07810
H	1.67579	-1.06340	-1.54555
K	-1.21880	0.46446	-1.90998

ACE4 Li B3LYP def2sv

47

O	0.32497	-1.25580	-0.26915
O	-2.48679	1.15231	0.46670
O	2.64288	-1.21589	1.14532
O	0.28767	2.05748	0.40506

O	2.65891	1.11475	-0.16783
N	-2.80011	-1.70929	-0.03601
C	-1.90404	-1.96002	-1.14319
C	-3.85065	-0.72459	-0.20388
C	-0.65910	-1.06551	-1.29549
C	-3.44641	0.73073	-0.47763
C	1.17611	-2.38226	-0.43317
C	-2.10874	2.50582	0.36374
C	2.06525	-2.47129	0.79974
C	-0.79127	2.69791	1.09187
C	3.84215	-0.81293	0.48401
C	1.15630	2.92076	-0.32327
C	3.55124	0.13589	-0.67456
C	2.13255	2.05115	-1.09400
H	-2.48007	-1.86288	-2.08305
H	-1.57130	-3.01701	-1.10085
H	-4.47013	-0.74847	0.71021
H	-4.51124	-1.02610	-1.04299
H	-0.18871	-1.25346	-2.28102
H	-0.93087	-0.00352	-1.23445
H	-3.03490	0.83557	-1.50426
H	-4.35653	1.36690	-0.43149
H	1.76968	-2.26903	-1.36163
H	0.59648	-3.31971	-0.52032
H	-2.87949	3.16689	0.81469
H	-1.99409	2.80496	-0.69880
H	1.45766	-2.76421	1.67137
H	2.84820	-3.23622	0.64786
H	-0.57531	3.77327	1.21443
H	-0.85728	2.24339	2.09491

H	4.41262	-1.68926	0.12895
H	4.44679	-0.28671	1.24131
H	1.69970	3.57830	0.38233
H	0.58968	3.55496	-1.02978
H	4.49312	0.60084	-1.02517
H	3.08852	-0.38838	-1.53247
H	1.61987	1.51904	-1.91969
H	2.93414	2.67865	-1.52744
C	-2.29601	-1.93126	1.30415
H	-1.70981	-2.86898	1.32547
H	-3.14516	-2.05565	2.00267
H	-1.65069	-1.12196	1.70444
Li	1.12485	0.25488	0.85874

ACE4 Li B3LYP def2tzv

47

O	0.36294	-2.12976	-0.43702
O	-2.69076	1.20484	0.03212
O	2.88493	-1.71106	0.56146
O	0.00767	2.50839	-0.30079
O	2.61357	1.23090	0.49805
N	-2.87390	-1.70108	0.79290
C	-2.08320	-2.49030	-0.14974
C	-3.96111	-0.87628	0.25858
C	-0.97301	-1.77696	-0.96155
C	-3.61737	0.31023	-0.65279
C	1.44177	-1.85893	-1.39554
C	-2.37804	2.41013	-0.72649
C	2.73901	-2.32637	-0.76435
C	-1.26854	3.16541	-0.03219
C	3.97905	-0.75906	0.75642

C	1.13342	3.15952	0.36010
C	3.80653	0.55115	0.01258
C	2.42808	2.54410	-0.11572
H	-2.77914	-2.94608	-0.85779
H	-1.60538	-3.31017	0.39507
H	-4.53104	-0.49928	1.11024
H	-4.63577	-1.51995	-0.31708
H	-1.00865	-2.09938	-2.00438
H	-1.08036	-0.69356	-0.92089
H	-3.17293	-0.02543	-1.59574
H	-4.54440	0.84446	-0.89382
H	1.46960	-0.79044	-1.61773
H	1.25713	-2.41212	-2.31968
H	-3.26789	3.04521	-0.78748
H	-2.06920	2.14708	-1.74296
H	2.72464	-3.40402	-0.60941
H	3.58548	-2.07466	-1.40342
H	-1.23495	4.19166	-0.41257
H	-1.45628	3.19969	1.04509
H	4.91918	-1.22544	0.45437
H	4.00399	-0.58295	1.82919
H	1.04104	3.06124	1.44587
H	1.13932	4.22530	0.10760
H	4.69459	1.16523	0.19948
H	3.73120	0.39352	-1.06803
H	2.41568	2.44811	-1.20535
H	3.26059	3.19610	0.16748
C	-2.17425	-1.19662	1.97717
H	-1.64906	-2.02286	2.46345
H	-2.90176	-0.79935	2.68611

H	-1.45146	-0.40024	1.76285
Li	1.13404	-1.72221	1.43660

ACE4 Li B97-1 def2sv

47

O	0.38970	-1.23365	-0.22660
O	-2.54562	1.08700	0.47988
O	2.72898	-1.13502	1.15005
O	0.20845	2.04604	0.45815
O	2.60561	1.18889	-0.18566
N	-2.73117	-1.78857	-0.05908
C	-1.80454	-1.98224	-1.15490
C	-3.81481	-0.83934	-0.23347
C	-0.58423	-1.04171	-1.25808
C	-3.45934	0.63416	-0.49206
C	1.26124	-2.33884	-0.40403
C	-2.19652	2.44652	0.37509
C	2.17901	-2.40236	0.81437
C	-0.89052	2.66636	1.12408
C	3.88393	-0.68986	0.44563
C	0.99774	2.90827	-0.35115
C	3.51499	0.23830	-0.71147
C	1.98091	2.04513	-1.12544
H	-2.36652	-1.88041	-2.10427
H	-1.43756	-3.03019	-1.13087
H	-4.44550	-0.89272	0.67372
H	-4.45275	-1.15932	-1.08490
H	-0.09441	-1.18128	-2.24464
H	-0.89289	0.01023	-1.16508
H	-3.02204	0.75684	-1.50827
H	-4.39654	1.23445	-0.47113

H	1.83596	-2.21624	-1.34543
H	0.70283	-3.29271	-0.47525
H	-2.98814	3.09531	0.81097
H	-2.07155	2.74322	-0.68908
H	1.59533	-2.71167	1.69884
H	2.97936	-3.14831	0.64613
H	-0.69577	3.74745	1.25126
H	-0.96284	2.20800	2.12633
H	4.47923	-1.54606	0.07674
H	4.49254	-0.13194	1.17891
H	1.53947	3.62941	0.29393
H	0.36978	3.47711	-1.06431
H	4.42722	0.73448	-1.10084
H	3.04303	-0.31386	-1.54881
H	1.45274	1.44172	-1.89328
H	2.72396	2.68857	-1.63713
C	-2.22433	-1.98347	1.28600
H	-1.59920	-2.89767	1.31237
H	-3.07342	-2.13997	1.98016
H	-1.61333	-1.14512	1.68480
Li	1.14366	0.28305	0.94956

ACE4 Li B97-1 def2tzv

47

O	0.38387	-2.09357	-0.46101
O	-2.72234	1.18140	0.04196
O	2.91803	-1.72085	0.54553
O	-0.01540	2.46751	-0.29133
O	2.59778	1.20976	0.50745
N	-2.86857	-1.73421	0.79714
C	-2.05208	-2.47298	-0.16803

C	-3.96632	-0.91541	0.26872
C	-0.95218	-1.69832	-0.94485
C	-3.63435	0.27722	-0.64450
C	1.45318	-1.75309	-1.40322
C	-2.40024	2.37062	-0.73296
C	2.75531	-2.26465	-0.80679
C	-1.28885	3.13237	-0.03979
C	3.99797	-0.75987	0.75599
C	1.09761	3.12604	0.37926
C	3.81099	0.56449	0.03196
C	2.40610	2.52636	-0.09153
H	-2.73352	-2.92624	-0.89486
H	-1.55221	-3.29620	0.35668
H	-4.53664	-0.54443	1.12579
H	-4.63793	-1.56567	-0.30742
H	-1.00248	-1.94160	-2.01123
H	-1.05783	-0.61765	-0.82023
H	-3.18251	-0.05718	-1.58815
H	-4.56999	0.79971	-0.89037
H	1.48104	-0.66815	-1.54780
H	1.26598	-2.24112	-2.36582
H	-3.28745	3.01187	-0.81130
H	-2.08278	2.09010	-1.74517
H	2.72868	-3.35105	-0.70714
H	3.60090	-1.98999	-1.44209
H	-1.24984	4.15600	-0.43390
H	-1.48567	3.18064	1.03816
H	4.94707	-1.20820	0.44471
H	4.02082	-0.60022	1.83391
H	0.99981	3.02148	1.46669

H	1.09668	4.19558	0.13116
H	4.68645	1.19193	0.24707
H	3.75922	0.42514	-1.05551
H	2.40518	2.44435	-1.18525
H	3.22980	3.18623	0.20887
C	-2.15878	-1.21049	1.96972
H	-1.61479	-2.02883	2.45379
H	-2.88450	-0.81860	2.68678
H	-1.44856	-0.40349	1.73899
Li	1.15979	-1.73089	1.43630

ACE4 Li CAM-B3LYP def2sv

47

O	0.37092	-1.19566	-0.24440
O	-2.50404	1.07956	0.48235
O	2.66665	-1.14195	1.15024
O	0.23494	2.01269	0.44194
O	2.60120	1.15238	-0.18849
N	-2.71797	-1.75227	-0.06437
C	-1.81048	-1.93090	-1.16983
C	-3.80388	-0.81181	-0.21415
C	-0.60224	-0.99232	-1.26753
C	-3.44603	0.65129	-0.46595
C	1.21781	-2.31494	-0.41493
C	-2.15205	2.43357	0.39445
C	2.10978	-2.39935	0.80951
C	-0.84528	2.63304	1.12744
C	3.84188	-0.72034	0.47469
C	1.02300	2.87486	-0.36079
C	3.50970	0.19666	-0.69027
C	2.00012	2.01501	-1.12935

H	-2.38146	-1.81623	-2.10841
H	-1.44423	-2.97570	-1.16758
H	-4.41610	-0.86953	0.70135
H	-4.45600	-1.12645	-1.05231
H	-0.11472	-1.11941	-2.25296
H	-0.91238	0.05403	-1.15947
H	-3.03468	0.78224	-1.48806
H	-4.37075	1.26163	-0.41156
H	1.80520	-2.19926	-1.34495
H	0.64104	-3.25209	-0.49960
H	-2.93429	3.07672	0.84625
H	-2.03755	2.74218	-0.66393
H	1.51047	-2.70442	1.68075
H	2.90304	-3.15025	0.65254
H	-0.63674	3.70720	1.26328
H	-0.91201	2.16569	2.12225
H	4.43055	-1.58683	0.12954
H	4.44007	-0.16527	1.21382
H	1.56358	3.58873	0.28724
H	0.39575	3.44639	-1.06699
H	4.43025	0.68190	-1.06363
H	3.04930	-0.35426	-1.53022
H	1.47680	1.42121	-1.90287
H	2.75375	2.65091	-1.62741
C	-2.19598	-1.96066	1.26606
H	-1.56547	-2.86760	1.27435
H	-3.03199	-2.13273	1.96744
H	-1.58945	-1.12390	1.66355
Li	1.14283	0.27668	0.89965

ACE4 Li CAM-B3LYP def2tzv

47

O	0.33049	-2.11118	-0.52259
O	-2.65401	1.18784	-0.00214
O	2.82747	-1.75133	0.44423
O	0.04073	2.45559	-0.31392
O	2.58366	1.16980	0.56934
N	-2.82709	-1.65201	0.87901
C	-2.07125	-2.44082	-0.08387
C	-3.92800	-0.84393	0.36250
C	-1.01599	-1.69939	-0.92372
C	-3.60128	0.28159	-0.61496
C	1.35717	-1.66772	-1.45756
C	-2.31219	2.32629	-0.82634
C	2.68073	-2.17666	-0.94116
C	-1.23486	3.12175	-0.14163
C	3.90917	-0.84080	0.77248
C	1.11339	3.08847	0.42463
C	3.79114	0.51565	0.12038
C	2.43274	2.49904	0.01027
H	-2.78530	-2.91841	-0.75542
H	-1.55513	-3.24315	0.44913
H	-4.45102	-0.41625	1.21812
H	-4.63551	-1.50501	-0.14637
H	-1.12946	-1.93751	-1.98219
H	-1.09543	-0.62150	-0.79071
H	-3.18556	-0.11018	-1.54800
H	-4.52437	0.81582	-0.86215
H	1.34892	-0.57835	-1.50854
H	1.15298	-2.07793	-2.44754
H	-3.19589	2.95377	-0.96978

H	-1.96169	1.99114	-1.80613
H	2.70556	-3.26407	-0.94745
H	3.50073	-1.80532	-1.55305
H	-1.18196	4.12239	-0.57890
H	-1.46860	3.21823	0.92126
H	4.86044	-1.29400	0.48962
H	3.87530	-0.74348	1.85358
H	0.95771	2.95585	1.49791
H	1.12603	4.16002	0.20610
H	4.66844	1.10463	0.40416
H	3.78416	0.43572	-0.96990
H	2.48926	2.45148	-1.07989
H	3.24207	3.13804	0.37315
C	-2.05467	-1.09982	1.98762
H	-1.53831	-1.91037	2.50612
H	-2.72873	-0.62401	2.69796
H	-1.31350	-0.35388	1.68246
Li	1.13508	-2.12244	1.32075

ACE4 Li MP2 def2tzv

47

O	0.40057	-1.97637	-0.51631
O	-2.78122	1.11730	-0.00073
O	2.95630	-1.75492	0.40751
O	-0.03943	2.33191	-0.29090
O	2.56196	1.14693	0.61376
N	-2.80158	-1.72207	0.92827
C	-1.98083	-2.41704	-0.08151
C	-3.95804	-0.97323	0.39129
C	-0.97483	-1.54378	-0.87567
C	-3.67933	0.13905	-0.62883

C	1.41936	-1.47286	-1.46272
C	-2.38035	2.19429	-0.91563
C	2.74323	-2.07724	-1.01958
C	-1.33273	3.03498	-0.21697
C	4.03431	-0.79391	0.70218
C	0.98376	3.00406	0.52724
C	3.81055	0.57978	0.08922
C	2.35494	2.51650	0.11260
H	-2.66723	-2.91540	-0.77515
H	-1.39946	-3.19507	0.43025
H	-4.49705	-0.54489	1.24329
H	-4.62481	-1.69798	-0.09689
H	-1.09309	-1.68693	-1.95657
H	-1.07782	-0.48274	-0.63111
H	-3.21040	-0.26118	-1.53880
H	-4.62377	0.62433	-0.91487
H	1.44059	-0.38014	-1.41599
H	1.16943	-1.80250	-2.47781
H	-3.25400	2.81079	-1.16288
H	-1.96195	1.77059	-1.83836
H	2.71945	-3.16555	-1.09850
H	3.56937	-1.69201	-1.62535
H	-1.23901	4.00938	-0.71327
H	-1.61976	3.18590	0.83066
H	4.98233	-1.20164	0.33300
H	4.06118	-0.73597	1.79023
H	0.80625	2.80454	1.59067
H	0.93230	4.08647	0.34860
H	4.66020	1.22078	0.36201
H	3.75665	0.52817	-1.00547

H	2.43484	2.52711	-0.98170
H	3.12239	3.17546	0.53893
C	-2.00771	-1.05027	1.98075
H	-1.47471	-1.81217	2.56227
H	-2.68937	-0.51899	2.65022
H	-1.27955	-0.32427	1.59300
Li	1.22954	-2.01078	1.38024

ACE4 Li PBE0 def2sv

47

O	0.40991	-2.00140	-0.47235
O	-2.69086	1.11102	0.00022
O	2.91227	-1.66762	0.51511
O	-0.05570	2.35175	-0.21457
O	2.54093	1.16174	0.46912
N	-2.81498	-1.71336	0.78049
C	-1.97795	-2.39704	-0.16891
C	-3.91157	-0.93671	0.25438
C	-0.88425	-1.58613	-0.89753
C	-3.58407	0.24848	-0.64762
C	1.43947	-1.65174	-1.37447
C	-2.38828	2.26785	-0.72685
C	2.73921	-2.16778	-0.80136
C	-1.27535	3.02333	-0.04717
C	3.95648	-0.73836	0.73298
C	1.01787	3.01760	0.39143
C	3.74815	0.59351	0.04862
C	2.33399	2.44307	-0.06476
H	-2.63956	-2.85824	-0.92333
H	-1.46177	-3.23342	0.34249
H	-4.49960	-0.56803	1.11378

H	-4.58033	-1.60258	-0.32610
H	-0.95304	-1.75261	-1.98857
H	-1.00083	-0.50323	-0.70581
H	-3.14964	-0.09949	-1.60993
H	-4.52987	0.77540	-0.90024
H	1.47564	-0.55266	-1.50195
H	1.25625	-2.11282	-2.36447
H	-3.27867	2.92706	-0.79834
H	-2.07732	2.01362	-1.76170
H	2.70748	-3.26924	-0.73571
H	3.58550	-1.88809	-1.45128
H	-1.21591	4.04126	-0.48625
H	-1.51840	3.13938	1.02966
H	4.92124	-1.16925	0.40680
H	3.99647	-0.59340	1.82408
H	0.94639	2.95947	1.49799
H	1.00840	4.09267	0.11588
H	4.60928	1.24519	0.30746
H	3.75799	0.47695	-1.05584
H	2.35155	2.40984	-1.17392
H	3.14473	3.12690	0.26149
C	-2.15495	-1.20755	1.95512
H	-1.59455	-2.02766	2.44405
H	-2.90648	-0.84222	2.67652
H	-1.44558	-0.37233	1.76810
Li	1.17016	-1.62134	1.40943

ACE4 Li PBE0 def2tzv

47

O	0.33350	-2.12988	-0.52613
O	-2.65198	1.19556	0.01581

O	2.83932	-1.75743	0.45136
O	0.03565	2.45925	-0.31673
O	2.56934	1.17337	0.57240
N	-2.83027	-1.67078	0.86694
C	-2.07002	-2.44517	-0.10087
C	-3.91760	-0.84877	0.35009
C	-1.00477	-1.69931	-0.92422
C	-3.58594	0.28901	-0.61085
C	1.35927	-1.66669	-1.44768
C	-2.32127	2.33575	-0.80651
C	2.68586	-2.17088	-0.93424
C	-1.23352	3.12955	-0.13612
C	3.90841	-0.83151	0.76458
C	1.10630	3.09767	0.41456
C	3.77418	0.52493	0.11597
C	2.42658	2.50086	0.01320
H	-2.78251	-2.91315	-0.78552
H	-1.55941	-3.25948	0.42603
H	-4.44953	-0.43084	1.20900
H	-4.62582	-1.50058	-0.17636
H	-1.11953	-1.91642	-1.99058
H	-1.07863	-0.61957	-0.77308
H	-3.16179	-0.09442	-1.54782
H	-4.51548	0.81720	-0.86329
H	1.34488	-0.57352	-1.48811
H	1.16601	-2.06588	-2.44811
H	-3.20665	2.96960	-0.93640
H	-1.98490	2.00649	-1.79697
H	2.71619	-3.26131	-0.94987
H	3.50233	-1.79245	-1.55203

H	-1.18621	4.13112	-0.58028
H	-1.45971	3.23528	0.93108
H	4.86741	-1.27010	0.47215
H	3.88656	-0.73458	1.84914
H	0.94850	2.98352	1.49316
H	1.12803	4.16961	0.18204
H	4.65637	1.11584	0.39415
H	3.76434	0.44501	-0.97791
H	2.49387	2.45639	-1.07991
H	3.23468	3.14329	0.38279
C	-2.05985	-1.12793	1.97788
H	-1.54971	-1.94506	2.49811
H	-2.73563	-0.64953	2.68899
H	-1.30933	-0.38255	1.68175
Li	1.13837	-2.14020	1.34697

ACE4 Li PBE def2sv

47

O	0.39265	-1.23977	-0.20845
O	-2.55559	1.08273	0.47785
O	2.75311	-1.13995	1.15217
O	0.20505	2.04423	0.46616
O	2.61619	1.20292	-0.17425
N	-2.74250	-1.79199	-0.05764
C	-1.80520	-1.99040	-1.14305
C	-3.82408	-0.84317	-0.24758
C	-0.58842	-1.04566	-1.24389
C	-3.46833	0.63073	-0.50503
C	1.27663	-2.33976	-0.41126
C	-2.20580	2.44859	0.36758
C	2.20345	-2.41152	0.79799

C	-0.90566	2.67022	1.12405
C	3.90337	-0.68307	0.43219
C	0.99441	2.91454	-0.34752
C	3.52224	0.24753	-0.71709
C	1.97891	2.05496	-1.12075
H	-2.36229	-1.90000	-2.10486
H	-1.42974	-3.04339	-1.10765
H	-4.47015	-0.89717	0.65739
H	-4.45667	-1.16487	-1.11225
H	-0.09479	-1.17495	-2.23841
H	-0.90234	0.01101	-1.13756
H	-3.02204	0.75460	-1.52609
H	-4.41208	1.23578	-0.49288
H	1.84478	-2.19837	-1.36333
H	0.72338	-3.30488	-0.49301
H	-3.00521	3.10666	0.79615
H	-2.07386	2.74338	-0.70457
H	1.62541	-2.73524	1.68997
H	3.01106	-3.15691	0.61383
H	-0.71027	3.75813	1.25404
H	-0.98483	2.20908	2.13252
H	4.50537	-1.53946	0.05298
H	4.51796	-0.12154	1.16885
H	1.53551	3.64460	0.30145
H	0.36186	3.48451	-1.06725
H	4.43708	0.74578	-1.11987
H	3.03694	-0.30364	-1.55762
H	1.44971	1.44001	-1.88990
H	2.71949	2.70584	-1.64313
C	-2.25122	-1.98719	1.29269

H	-1.63211	-2.91348	1.32964
H	-3.11356	-2.13252	1.98407
H	-1.63015	-1.15069	1.70237
Li	1.15198	0.28187	0.96695

ACE4 Li PBE def2tzv

47

O	0.36089	-2.11998	-0.46619
O	-2.70176	1.20036	0.06157
O	2.91385	-1.75602	0.54832
O	0.01116	2.47291	-0.30934
O	2.60512	1.19522	0.52778
N	-2.88833	-1.72435	0.79285
C	-2.08591	-2.47050	-0.17920
C	-3.97624	-0.88602	0.27255
C	-0.97990	-1.71358	-0.96084
C	-3.63697	0.30887	-0.63246
C	1.43617	-1.73774	-1.40294
C	-2.38348	2.39725	-0.72367
C	2.73666	-2.26739	-0.82720
C	-1.26336	3.15300	-0.04473
C	4.00243	-0.78748	0.75497
C	1.13003	3.13535	0.36992
C	3.82043	0.53972	0.04131
C	2.43492	2.52148	-0.08255
H	-2.78212	-2.91620	-0.90763
H	-1.59246	-3.30963	0.34278
H	-4.54419	-0.51466	1.14017
H	-4.66326	-1.52623	-0.31121
H	-1.02703	-1.96691	-2.03281
H	-1.07933	-0.62334	-0.84517

H	-3.19313	-0.02415	-1.58935
H	-4.57418	0.84890	-0.86991
H	1.45969	-0.64083	-1.50829
H	1.24786	-2.19347	-2.38979
H	-3.27538	3.04625	-0.79264
H	-2.07969	2.11341	-1.74741
H	2.70689	-3.36302	-0.75065
H	3.58589	-1.98081	-1.46452
H	-1.22214	4.18301	-0.44370
H	-1.44983	3.20760	1.04262
H	4.95591	-1.23886	0.43495
H	4.03024	-0.63750	1.84143
H	1.01972	3.04445	1.46526
H	1.14061	4.21003	0.11001
H	4.70843	1.16313	0.25792
H	3.76586	0.40855	-1.05483
H	2.44866	2.43545	-1.18359
H	3.26795	3.17847	0.22661
C	-2.17084	-1.22122	1.97044
H	-1.63356	-2.05620	2.44908
H	-2.89533	-0.82534	2.69675
H	-1.44494	-0.41472	1.75001
Li	1.14648	-1.76908	1.45110

ACE4 Li B2PLYP def2sv

47

O	0.41660	-1.99453	-0.47392
O	-2.70816	1.10250	-0.00889
O	2.92514	-1.66904	0.50499
O	-0.06557	2.33499	-0.20948
O	2.54213	1.15468	0.47319

N	-2.81810	-1.72081	0.80719
C	-1.97247	-2.40355	-0.14775
C	-3.92448	-0.95088	0.26951
C	-0.89203	-1.57558	-0.88767
C	-3.59966	0.22208	-0.65572
C	1.44452	-1.62604	-1.38710
C	-2.39631	2.25347	-0.75936
C	2.74930	-2.15495	-0.82781
C	-1.29290	3.01976	-0.06888
C	3.97956	-0.73767	0.73011
C	1.00520	3.00825	0.41643
C	3.76613	0.59997	0.05005
C	2.32941	2.45071	-0.04877
H	-2.63137	-2.87797	-0.89470
H	-1.44152	-3.22501	0.36907
H	-4.50824	-0.56747	1.12365
H	-4.59041	-1.62951	-0.29704
H	-0.96981	-1.73674	-1.97740
H	-1.00561	-0.49696	-0.68565
H	-3.15646	-0.13863	-1.60671
H	-4.54429	0.74266	-0.91928
H	1.47857	-0.52669	-1.49207
H	1.25072	-2.07194	-2.38029
H	-3.28705	2.90697	-0.85430
H	-2.06736	1.97953	-1.78152
H	2.71577	-3.25534	-0.77573
H	3.59427	-1.86392	-1.47200
H	-1.21913	4.02939	-0.52108
H	-1.55211	3.14791	1.00082
H	4.94058	-1.16962	0.39901

H	4.01676	-0.59902	1.82050
H	0.92993	2.92960	1.51960
H	0.98374	4.08509	0.15529
H	4.61605	1.25913	0.32016
H	3.77935	0.49188	-1.05294
H	2.34712	2.42993	-1.15631
H	3.13581	3.13129	0.28999
C	-2.13666	-1.17226	1.96098
H	-1.56930	-1.97701	2.46499
H	-2.87817	-0.78408	2.67881
H	-1.43260	-0.34573	1.73252
Li	1.18421	-1.61685	1.39703

ACE4 Li B2PLYP def2tzv

47

O	0.38745	-2.07174	-0.47190
O	-2.72625	1.15760	0.02031
O	2.91365	-1.72883	0.51863
O	-0.01206	2.43269	-0.28517
O	2.59556	1.18645	0.52305
N	-2.87464	-1.72728	0.82730
C	-2.03240	-2.44616	-0.13584
C	-3.97507	-0.92481	0.27221
C	-0.95972	-1.62606	-0.89664
C	-3.63103	0.23757	-0.66686
C	1.44679	-1.70325	-1.42297
C	-2.38419	2.33337	-0.77711
C	2.74684	-2.23793	-0.85302
C	-1.29505	3.10468	-0.06862
C	4.00544	-0.77589	0.74590
C	1.08705	3.09275	0.41916

C	3.81826	0.55300	0.03877
C	2.40329	2.52047	-0.05056
H	-2.69621	-2.92382	-0.85913
H	-1.50655	-3.24301	0.39759
H	-4.54945	-0.53371	1.11389
H	-4.63545	-1.59448	-0.28933
H	-1.03671	-1.79286	-1.97295
H	-1.04856	-0.56006	-0.68807
H	-3.15851	-0.12054	-1.58755
H	-4.55516	0.75850	-0.94288
H	1.47594	-0.61748	-1.52785
H	1.24197	-2.15980	-2.39376
H	-3.26934	2.96650	-0.89109
H	-2.03828	2.02791	-1.76906
H	2.71966	-3.32340	-0.78407
H	3.59078	-1.94270	-1.47666
H	-1.24149	4.11975	-0.47413
H	-1.51412	3.16211	1.00105
H	4.94579	-1.22778	0.42367
H	4.02855	-0.63485	1.82315
H	0.97577	2.95915	1.49854
H	1.07130	4.16373	0.19290
H	4.68483	1.18385	0.26333
H	3.76390	0.42783	-1.04662
H	2.41487	2.46114	-1.14233
H	3.21786	3.17325	0.27742
C	-2.15762	-1.15668	1.97775
H	-1.61948	-1.95676	2.49222
H	-2.88002	-0.73092	2.67399
H	-1.44518	-0.36815	1.70968

Li	1.16115	-1.73940	1.41818

ACE4 Li DSDPBEP86 def2sv

47

O	0.41789	-1.15018	-0.21620
O	-2.53268	1.00815	0.50023
O	2.71491	-1.08047	1.16650
O	0.16551	1.99469	0.46874
O	2.55988	1.21299	-0.19250
N	-2.64490	-1.83280	-0.07737
C	-1.72542	-1.93216	-1.18844
C	-3.76027	-0.91972	-0.21776
C	-0.55666	-0.94114	-1.24143
C	-3.44505	0.55254	-0.47107
C	1.26839	-2.26809	-0.40135
C	-2.22297	2.37723	0.39827
C	2.16769	-2.34536	0.82050
C	-0.92784	2.61139	1.14373
C	3.85592	-0.63776	0.43950
C	0.90206	2.86108	-0.38254
C	3.45937	0.25746	-0.72294
C	1.88207	2.00428	-1.15083
H	-2.30164	-1.80743	-2.12545
H	-1.31668	-2.96263	-1.22138
H	-4.36469	-0.99507	0.70456
H	-4.40780	-1.25657	-1.05363
H	-0.05756	-1.00714	-2.23031
H	-0.90708	0.08769	-1.08437
H	-3.01613	0.69255	-1.48688
H	-4.39039	1.13633	-0.43533
H	1.84763	-2.15007	-1.33813

H	0.69550	-3.21075	-0.47884
H	-3.03125	3.00185	0.83468
H	-2.10305	2.67576	-0.66430
H	1.57101	-2.65429	1.69443
H	2.96920	-3.09051	0.66161
H	-0.73382	3.69224	1.26862
H	-0.99323	2.15056	2.14352
H	4.45404	-1.49651	0.08363
H	4.46544	-0.05771	1.15220
H	1.43895	3.61298	0.22779
H	0.23477	3.38911	-1.08949
H	4.35743	0.74928	-1.14578
H	2.96768	-0.31270	-1.53319
H	1.34804	1.35048	-1.86886
H	2.58872	2.64363	-1.71410
C	-2.07449	-1.96579	1.24822
H	-1.39074	-2.83553	1.26066
H	-2.88601	-2.16648	1.97298
H	-1.50961	-1.08082	1.60198
Li	1.14824	0.30869	0.98252

ACE4 Li DSDPBEP86 def2tzv

47

O	0.40960	-1.98172	-0.53096
O	-2.76576	1.11037	0.00019
O	2.94407	-1.74518	0.40214
O	-0.04950	2.34381	-0.29251
O	2.54418	1.14636	0.60619
N	-2.78744	-1.72646	0.91634
C	-1.96304	-2.42080	-0.08046
C	-3.93706	-0.97947	0.38128

C	-0.95873	-1.55451	-0.87787
C	-3.65642	0.13971	-0.62567
C	1.42033	-1.46536	-1.46275
C	-2.38958	2.20155	-0.89110
C	2.74654	-2.05583	-1.01948
C	-1.33556	3.03468	-0.19941
C	4.00481	-0.79088	0.72577
C	0.97912	3.00780	0.50512
C	3.79779	0.58422	0.11880
C	2.34127	2.50137	0.09411
H	-2.64046	-2.92908	-0.77428
H	-1.38143	-3.19527	0.43503
H	-4.48189	-0.55853	1.23238
H	-4.60510	-1.69592	-0.11553
H	-1.08603	-1.69609	-1.95707
H	-1.06140	-0.49278	-0.63594
H	-3.19074	-0.25396	-1.53980
H	-4.60214	0.62305	-0.90942
H	1.43120	-0.37245	-1.41103
H	1.17960	-1.78837	-2.48147
H	-3.26954	2.81902	-1.11153
H	-1.99024	1.80083	-1.83204
H	2.73240	-3.14358	-1.10609
H	3.57069	-1.66192	-1.62026
H	-1.25422	4.01523	-0.68547
H	-1.61157	3.17875	0.85216
H	4.96495	-1.19316	0.38450
H	4.00526	-0.73509	1.81429
H	0.80411	2.82474	1.57203
H	0.94205	4.08940	0.31959

H	4.64123	1.22070	0.41862
H	3.77947	0.54108	-0.97752
H	2.41975	2.50038	-1.00015
H	3.11419	3.16246	0.50650
C	-2.01326	-1.07058	1.98270
H	-1.44746	-1.83352	2.53009
H	-2.70298	-0.58883	2.68015
H	-1.31328	-0.30588	1.61796
Li	1.22923	-2.00883	1.33108

ACE4 Li HSE06 def2sv

47

O	0.38082	-1.21413	-0.22010
O	-2.51653	1.07523	0.47453
O	2.70253	-1.13547	1.13965
O	0.21588	2.02833	0.45885
O	2.59558	1.17581	-0.18024
N	-2.71438	-1.77085	-0.06292
C	-1.79422	-1.95089	-1.15377
C	-3.79261	-0.82885	-0.22955
C	-0.58785	-1.01104	-1.24302
C	-3.43555	0.63247	-0.48600
C	1.23796	-2.31893	-0.40435
C	-2.17231	2.42848	0.37426
C	2.14967	-2.39310	0.80429
C	-0.87662	2.64750	1.11942
C	3.85402	-0.70088	0.43899
C	0.99995	2.88569	-0.34658
C	3.49113	0.22443	-0.70742
C	1.97497	2.02729	-1.11542
H	-2.35525	-1.84361	-2.10096

H	-1.42502	-2.99671	-1.14256
H	-4.41738	-0.88236	0.67956
H	-4.43716	-1.14670	-1.07446
H	-0.09766	-1.13063	-2.22995
H	-0.90273	0.03563	-1.13241
H	-3.00667	0.75562	-1.50399
H	-4.36686	1.23773	-0.45828
H	1.81090	-2.19682	-1.34488
H	0.67395	-3.26683	-0.48123
H	-2.96450	3.07446	0.80783
H	-2.04727	2.72720	-0.68755
H	1.56607	-2.70305	1.68621
H	2.94471	-3.14169	0.63541
H	-0.68062	3.72652	1.24602
H	-0.95190	2.19351	2.12137
H	4.44209	-1.55963	0.07001
H	4.46870	-0.14971	1.16957
H	1.54167	3.60573	0.29657
H	0.37327	3.45512	-1.05733
H	4.40367	0.71191	-1.10141
H	3.01237	-0.32212	-1.54226
H	1.44659	1.42446	-1.88074
H	2.71640	2.66735	-1.62936
C	-2.20883	-1.97395	1.27085
H	-1.58306	-2.88546	1.29023
H	-3.05421	-2.13854	1.96453
H	-1.59991	-1.13993	1.67576
Li	1.14201	0.27902	0.95099

ACE4 Li HSE06 def2tzv

47

O	0.32879	-2.12986	-0.52649
O	-2.64730	1.19620	0.01236
O	2.83554	-1.76174	0.44983
O	0.04171	2.45918	-0.31648
O	2.57268	1.16935	0.57305
N	-2.83530	-1.66610	0.86989
C	-2.07487	-2.44201	-0.09691
C	-3.92072	-0.84292	0.35019
C	-1.01073	-1.69552	-0.92110
C	-3.58434	0.29063	-0.61372
C	1.35540	-1.66712	-1.44926
C	-2.31469	2.33693	-0.81041
C	2.68094	-2.17430	-0.93722
C	-1.22845	3.13044	-0.13805
C	3.90815	-0.83821	0.76389
C	1.11252	3.09620	0.41788
C	3.77670	0.51807	0.11533
C	2.43244	2.49942	0.01662
H	-2.78688	-2.91177	-0.78032
H	-1.56339	-3.25449	0.43126
H	-4.45183	-0.42102	1.20725
H	-4.63008	-1.49425	-0.17464
H	-1.12742	-1.90908	-1.98757
H	-1.08234	-0.61641	-0.76611
H	-3.15976	-0.09683	-1.54850
H	-4.51094	0.82178	-0.86916
H	1.34271	-0.57424	-1.48808
H	1.15991	-2.06473	-2.44948
H	-3.19990	2.97000	-0.94195
H	-1.97678	2.00691	-1.79969

H	2.70953	-3.26438	-0.95332
H	3.49800	-1.79668	-1.55415
H	-1.17943	4.13170	-0.58182
H	-1.45611	3.23600	0.92850
H	4.86533	-1.27969	0.47130
H	3.88621	-0.74183	1.84812
H	0.95306	2.97974	1.49563
H	1.13437	4.16826	0.18758
H	4.65975	1.10760	0.39237
H	3.76526	0.43810	-0.97823
H	2.50126	2.45729	-1.07614
H	3.24096	3.13914	0.38891
C	-2.06386	-1.12097	1.97944
H	-1.55181	-1.93669	2.49927
H	-2.73905	-0.64318	2.69110
H	-1.31557	-0.37458	1.68131
Li	1.13328	-2.14093	1.34638

ACE4 Li M062X def2sv

47

O	0.46026	-1.07279	-0.17990
O	-2.54278	0.94604	0.51963
O	2.75381	-1.01576	1.17330
O	0.13712	1.96529	0.51296
O	2.51579	1.23154	-0.23576
N	-2.56808	-1.85619	-0.13881
C	-1.63724	-1.86715	-1.24150
C	-3.71802	-0.98624	-0.25094
C	-0.48961	-0.85095	-1.21756
C	-3.43943	0.49800	-0.46270
C	1.26276	-2.22622	-0.32019

C	-2.25139	2.31558	0.44856
C	2.17981	-2.27799	0.89052
C	-0.95886	2.55413	1.19654
C	3.84676	-0.60190	0.37027
C	0.80825	2.83072	-0.38455
C	3.37808	0.26220	-0.78886
C	1.78310	1.98803	-1.17353
H	-2.20279	-1.68955	-2.17351
H	-1.20859	-2.88317	-1.34184
H	-4.31403	-1.10659	0.66941
H	-4.35387	-1.31914	-1.09373
H	0.03677	-0.87544	-2.19289
H	-0.86948	0.16433	-1.03660
H	-3.00590	0.67084	-1.47068
H	-4.39306	1.06206	-0.41784
H	1.82971	-2.17887	-1.26957
H	0.65557	-3.14777	-0.34010
H	-3.06573	2.91921	0.89578
H	-2.13796	2.63529	-0.60762
H	1.59192	-2.54164	1.78292
H	2.96320	-3.04199	0.74765
H	-0.77808	3.63322	1.33824
H	-1.01922	2.07597	2.18621
H	4.41534	-1.47323	0.00339
H	4.50165	-0.00562	1.02382
H	1.34176	3.61240	0.18672
H	0.09696	3.31868	-1.07386
H	4.24512	0.73911	-1.28107
H	2.83315	-0.32692	-1.54956
H	1.23993	1.30728	-1.85700

H	2.44943	2.63184	-1.77462
C	-2.01175	-2.03357	1.18548
H	-1.27476	-2.85624	1.15914
H	-2.81681	-2.32973	1.88095
H	-1.51168	-1.13739	1.59998
Li	1.20654	0.35801	1.04895

ACE4 Li M062X def2tzv

47

O	0.42858	-1.89248	-0.52424
O	-2.77855	1.07571	0.00032
O	2.94797	-1.72980	0.39651
O	-0.07154	2.31268	-0.27352
O	2.52921	1.12413	0.58761
N	-2.76154	-1.72656	0.89490
C	-1.93020	-2.38673	-0.10719
C	-3.92625	-1.00706	0.37845
C	-0.93308	-1.49450	-0.87493
C	-3.65183	0.10776	-0.62385
C	1.43758	-1.39370	-1.44731
C	-2.39634	2.16083	-0.87401
C	2.74423	-2.02127	-1.01436
C	-1.34985	2.98839	-0.17531
C	3.99639	-0.78550	0.73064
C	0.95640	2.96486	0.50906
C	3.78490	0.58162	0.12240
C	2.30949	2.46407	0.08115
H	-2.59359	-2.88320	-0.81655
H	-1.34369	-3.16714	0.38517
H	-4.46554	-0.58921	1.22931
H	-4.59061	-1.72613	-0.10905

H	-1.04883	-1.62346	-1.95295
H	-1.06264	-0.43960	-0.62343
H	-3.17747	-0.28781	-1.52922
H	-4.59233	0.58380	-0.91870
H	1.47689	-0.30420	-1.38410
H	1.18637	-1.69700	-2.46497
H	-3.27072	2.77455	-1.10476
H	-1.98793	1.76339	-1.80827
H	2.69150	-3.10380	-1.10771
H	3.57814	-1.65538	-1.61256
H	-1.27318	3.97311	-0.64355
H	-1.62806	3.11677	0.87406
H	4.95796	-1.18331	0.40177
H	3.98913	-0.72249	1.81547
H	0.79533	2.77209	1.57253
H	0.91600	4.04450	0.33835
H	4.61353	1.22709	0.42766
H	3.78161	0.53510	-0.97089
H	2.36834	2.46030	-1.01078
H	3.08406	3.12824	0.47347
C	-2.00679	-1.08285	1.97049
H	-1.43263	-1.84229	2.50615
H	-2.69961	-0.62171	2.67241
H	-1.31926	-0.30571	1.61763
Li	1.24161	-1.94601	1.31146

ACE4 Li M06 def2sv

47

O	0.43866	-1.12303	-0.28354
O	-2.56419	0.98850	0.47617
O	2.73406	-1.11914	1.15932

O	0.11437	2.04305	0.48621
O	2.55668	1.24654	-0.12096
N	-2.57756	-1.84340	-0.01149
C	-1.71172	-1.95116	-1.15565
C	-3.74090	-0.99802	-0.11942
C	-0.56840	-0.94728	-1.27157
C	-3.49618	0.46799	-0.42978
C	1.32606	-2.19338	-0.51718
C	-2.26969	2.34031	0.27789
C	2.20427	-2.34610	0.70457
C	-1.02258	2.66978	1.05452
C	3.86822	-0.60082	0.49432
C	0.87449	2.86464	-0.37527
C	3.48157	0.32152	-0.63913
C	1.86988	1.99594	-1.09252
H	-2.33003	-1.85889	-2.07120
H	-1.28339	-2.97750	-1.19231
H	-4.29641	-1.07274	0.83553
H	-4.42034	-1.38734	-0.90885
H	-0.10084	-1.02372	-2.27782
H	-0.93244	0.08582	-1.13424
H	-3.11751	0.58978	-1.47258
H	-4.45845	1.02707	-0.37898
H	1.91636	-1.99653	-1.43866
H	0.78305	-3.14663	-0.68035
H	-3.11209	2.98793	0.60943
H	-2.10435	2.54956	-0.80528
H	1.59675	-2.73479	1.54236
H	3.01362	-3.07571	0.50281
H	-0.86762	3.76460	1.10566

H	-1.12599	2.29252	2.08828
H	4.51897	-1.41766	0.12493
H	4.43580	-0.03002	1.25157
H	1.39444	3.64645	0.21782
H	0.22820	3.37290	-1.11950
H	4.38036	0.83687	-1.03787
H	3.01994	-0.23604	-1.48113
H	1.35125	1.30828	-1.79781
H	2.56733	2.62394	-1.68409
C	-1.97857	-1.99252	1.28856
H	-1.28029	-2.85338	1.27886
H	-2.76335	-2.21434	2.03844
H	-1.41338	-1.10539	1.65196
Li	1.06259	0.29196	1.08658

ACE4 Li M06 def2tzv

47

O	0.45247	-1.98585	-0.54612
O	-2.78921	1.07244	0.01472
O	2.96055	-1.67331	0.42183
O	-0.11625	2.33229	-0.28599
O	2.49455	1.18170	0.57893
N	-2.69823	-1.71148	0.88392
C	-1.90378	-2.43043	-0.09377
C	-3.88385	-1.02650	0.39346
C	-0.90068	-1.60513	-0.91889
C	-3.66013	0.10507	-0.59712
C	1.45824	-1.46534	-1.44826
C	-2.43845	2.16262	-0.85377
C	2.78301	-2.00942	-0.97640
C	-1.39287	2.99437	-0.17053

C	3.97970	-0.70600	0.74925
C	0.91632	3.00645	0.45905
C	3.75385	0.64888	0.13209
C	2.26022	2.49682	0.03262
H	-2.59085	-2.94388	-0.77138
H	-1.32553	-3.20714	0.42142
H	-4.42520	-0.63241	1.25767
H	-4.53936	-1.75892	-0.09191
H	-1.02386	-1.79778	-1.98982
H	-1.02531	-0.53073	-0.74042
H	-3.20872	-0.26885	-1.52719
H	-4.62089	0.56663	-0.86090
H	1.44447	-0.36970	-1.42142
H	1.25172	-1.80059	-2.46941
H	-3.32344	2.77192	-1.07193
H	-2.04156	1.77777	-1.80261
H	2.78938	-3.09787	-1.04665
H	3.60327	-1.61933	-1.58208
H	-1.33530	3.98610	-0.63435
H	-1.66088	3.12146	0.88522
H	4.95861	-1.08221	0.43660
H	3.96496	-0.63331	1.83567
H	0.77055	2.85241	1.53476
H	0.87416	4.08415	0.25886
H	4.57793	1.30631	0.43636
H	3.76709	0.59643	-0.96439
H	2.30550	2.45774	-1.06305
H	3.03923	3.18312	0.38511
C	-1.94905	-1.09164	1.96339
H	-1.39822	-1.86136	2.51488

H	-2.63953	-0.61003	2.65694
H	-1.23346	-0.32601	1.62691
Li	1.26028	-2.02679	1.36867

ACE4 Li MP2 def2sv

47

O	0.42935	-1.16729	-0.17741
O	-2.54890	0.99047	0.49063
O	2.75241	-1.05965	1.15432
O	0.13956	1.98384	0.47728
O	2.54066	1.22966	-0.19698
N	-2.64317	-1.84934	-0.07780
C	-1.70237	-1.94985	-1.16980
C	-3.75412	-0.93755	-0.24867
C	-0.54317	-0.95095	-1.20480
C	-3.43534	0.53112	-0.50418
C	1.29789	-2.26777	-0.38820
C	-2.24616	2.36316	0.39389
C	2.21866	-2.33332	0.81546
C	-0.95966	2.59605	1.15037
C	3.87395	-0.60184	0.40567
C	0.87178	2.86430	-0.36550
C	3.44493	0.28814	-0.74652
C	1.84787	2.01965	-1.14695
H	-2.26221	-1.83821	-2.11822
H	-1.28476	-2.97660	-1.18551
H	-4.37829	-1.00506	0.66049
H	-4.38310	-1.28123	-1.09540
H	-0.04035	-0.99709	-2.19285
H	-0.90292	0.07065	-1.03039
H	-2.98118	0.66627	-1.50871

H	-4.38209	1.11306	-0.49569
H	1.85784	-2.13200	-1.33348
H	0.74388	-3.22171	-0.46215
H	-3.06129	2.98249	0.82419
H	-2.11655	2.66506	-0.66570
H	1.64182	-2.65264	1.69817
H	3.02820	-3.06620	0.64175
H	-0.76759	3.67604	1.28429
H	-1.03238	2.12851	2.14587
H	4.47673	-1.45229	0.03825
H	4.48930	-0.01443	1.10646
H	1.41053	3.60900	0.25111
H	0.20147	3.39980	-1.06333
H	4.32859	0.79333	-1.18318
H	2.94897	-0.28749	-1.54893
H	1.31033	1.36687	-1.86188
H	2.54431	2.66637	-1.71416
C	-2.08757	-1.95487	1.25611
H	-1.41366	-2.83042	1.29492
H	-2.90932	-2.13043	1.97426
H	-1.51834	-1.06847	1.59392
Li	1.16311	0.31605	1.00883

ACE4 Li SCS-MP2 def2sv

47

O	0.41223	-1.18362	-0.20215
O	-2.53695	1.02068	0.49055
O	2.72603	-1.08838	1.15689
O	0.16547	2.00560	0.46582
O	2.56604	1.21480	-0.18631
N	-2.67111	-1.83592	-0.06832

C	-1.74080	-1.95541	-1.17329
C	-3.77447	-0.90590	-0.22704
C	-0.56397	-0.97172	-1.23113
C	-3.44306	0.56251	-0.49182
C	1.27795	-2.29315	-0.40206
C	-2.22528	2.39393	0.38296
C	2.18803	-2.36290	0.81446
C	-0.93304	2.62786	1.13737
C	3.86916	-0.64076	0.42784
C	0.92277	2.88241	-0.36400
C	3.46868	0.26273	-0.72918
C	1.89795	2.02559	-1.14162
H	-2.31068	-1.84061	-2.11635
H	-1.33921	-2.98914	-1.18727
H	-4.38790	-0.96396	0.69105
H	-4.41958	-1.24450	-1.06473
H	-0.06605	-1.04320	-2.22056
H	-0.90770	0.05926	-1.07649
H	-3.00226	0.69075	-1.50360
H	-4.38517	1.15285	-0.47024
H	1.84928	-2.16240	-1.34177
H	0.71610	-3.24283	-0.47964
H	-3.03684	3.01969	0.81262
H	-2.09691	2.68755	-0.67959
H	1.60001	-2.67710	1.69268
H	2.99597	-3.10065	0.65064
H	-0.73724	3.70894	1.26144
H	-1.00496	2.16878	2.13788
H	4.46697	-1.49777	0.06592
H	4.48024	-0.06423	1.14265

H	1.46607	3.61442	0.26481
H	0.26732	3.43260	-1.06568
H	4.36549	0.76089	-1.14849
H	2.97724	-0.30222	-1.54288
H	1.35921	1.38664	-1.86922
H	2.61421	2.66518	-1.69334
C	-2.10686	-1.95537	1.26543
H	-1.43902	-2.83708	1.29430
H	-2.92609	-2.13086	1.98827
H	-1.52883	-1.07480	1.60603
Li	1.14771	0.30120	0.97459

ACE4 Li SCS-MP2 def2tzv

47

O	0.39689	-2.03744	-0.52050
O	-2.76460	1.13267	-0.00555
O	2.93784	-1.74374	0.42251
O	-0.03383	2.37377	-0.30113
O	2.55914	1.17118	0.60314
N	-2.80630	-1.72103	0.93426
C	-1.99367	-2.44328	-0.06679
C	-3.95363	-0.95810	0.39018
C	-0.97528	-1.59679	-0.88173
C	-3.66592	0.15597	-0.63211
C	1.41624	-1.54673	-1.47282
C	-2.38109	2.22223	-0.91339
C	2.75180	-2.10998	-0.99781
C	-1.33095	3.06810	-0.21470
C	4.01679	-0.78416	0.71505
C	0.99381	3.04356	0.51346
C	3.80398	0.58861	0.08501

C	2.36517	2.54198	0.09895
H	-2.68752	-2.94404	-0.75221
H	-1.42388	-3.22178	0.45788
H	-4.49170	-0.52428	1.24095
H	-4.62693	-1.67673	-0.09936
H	-1.10152	-1.75841	-1.95959
H	-1.06367	-0.52982	-0.65691
H	-3.20045	-0.24534	-1.54363
H	-4.60992	0.64490	-0.91611
H	1.41828	-0.45193	-1.46304
H	1.18299	-1.91321	-2.47970
H	-3.26274	2.83478	-1.14489
H	-1.96954	1.81133	-1.84542
H	2.75410	-3.20121	-1.04838
H	3.57529	-1.72094	-1.60534
H	-1.24756	4.04610	-0.70727
H	-1.61140	3.21268	0.83623
H	4.96753	-1.19987	0.36014
H	4.03429	-0.71262	1.80337
H	0.81525	2.84876	1.57831
H	0.94979	4.12622	0.33120
H	4.65864	1.22563	0.35378
H	3.75132	0.52645	-1.00968
H	2.44706	2.55048	-0.99564
H	3.13796	3.19531	0.52589
C	-2.00505	-1.05083	1.98560
H	-1.46765	-1.81622	2.55987
H	-2.68346	-0.52403	2.66328
H	-1.27971	-0.32176	1.59699
Li	1.22081	-2.05607	1.37945

ACE4 Na B3LYP def2sv

47

O	-1.12085	-2.05309	-0.35730
O	-1.41260	2.01041	-0.24413
O	1.68379	-2.16511	-0.28824
O	1.41256	2.27813	-0.30657
O	2.93010	0.13806	0.49218
N	-3.06374	-0.17809	0.96962
C	-3.37027	-1.40791	0.28503
C	-3.54226	1.07333	0.44003
C	-2.44146	-1.85441	-0.85039
C	-2.73602	1.73863	-0.68103
C	-0.39252	-3.07401	-1.01554
C	-0.75645	3.01671	-0.99141
C	0.88573	-3.33277	-0.25166
C	0.53347	3.38867	-0.29752
C	2.86260	-2.23889	0.49255
C	2.60803	2.49325	0.42368
C	3.69995	-1.01330	0.20315
C	3.56113	1.35413	0.14167
H	-4.38767	-1.33431	-0.15006
H	-3.39628	-2.22836	1.02817
H	-3.61261	1.79900	1.27261
H	-4.56977	0.93507	0.04496
H	-2.83382	-2.80607	-1.26003
H	-2.42574	-1.11792	-1.67702
H	-2.71271	1.10901	-1.59295
H	-3.24268	2.68814	-0.94985
H	-0.16029	-2.78104	-2.06013
H	-0.98247	-4.01108	-1.04689

H	-1.39098	3.92292	-1.06014
H	-0.54942	2.66854	-2.02480
H	0.64532	-3.60094	0.79670
H	1.42464	-4.18490	-0.71230
H	0.99745	4.24807	-0.82192
H	0.31731	3.69652	0.74501
H	3.44683	-3.14471	0.23632
H	2.60375	-2.28962	1.56964
H	2.38446	2.55469	1.50795
H	3.08583	3.44452	0.11727
H	4.61992	-1.03832	0.82099
H	4.00201	-1.00821	-0.86369
H	3.83110	1.34433	-0.93389
H	4.49072	1.50020	0.72764
C	-2.19299	-0.18423	2.11900
H	-2.29341	-1.14379	2.65968
H	-2.48149	0.62599	2.81614
H	-1.10924	-0.04784	1.90508
Na	0.49890	0.00223	-0.21406

ACE4 Na B3LYP def2tzv

47

O	-0.13333	-1.57448	-0.52683
O	-2.24549	1.55528	0.29574
O	2.26368	-1.76102	0.90897
O	0.59608	2.37918	-0.05062
O	2.95009	0.82291	0.12033
N	-3.13592	-1.36927	0.32726
C	-2.56908	-1.98101	-0.87009
C	-3.99557	-0.19626	0.18857
C	-1.27000	-1.38308	-1.44000

C	-3.42392	1.08467	-0.43275
C	0.76288	-2.68343	-0.84403
C	-1.78753	2.84879	-0.20441
C	1.61301	-2.96329	0.38484
C	-0.48190	3.23885	0.44734
C	3.62698	-1.45244	0.46975
C	1.92670	2.99060	-0.00446
C	3.65298	-0.29212	-0.50999
C	2.89754	2.04685	-0.67596
H	-3.32106	-1.93588	-1.66549
H	-2.38976	-3.04239	-0.66721
H	-4.38324	0.04233	1.18067
H	-4.86165	-0.45665	-0.43455
H	-1.04353	-1.84528	-2.40494
H	-1.35455	-0.30948	-1.58287
H	-3.15692	0.92676	-1.48240
H	-4.20367	1.85488	-0.40163
H	1.37320	-2.41948	-1.71178
H	0.19078	-3.58386	-1.08344
H	-2.53850	3.61350	0.02091
H	-1.65575	2.80198	-1.28959
H	0.98072	-3.32081	1.19508
H	2.35638	-3.72936	0.15423
H	-0.26412	4.27760	0.18658
H	-0.54386	3.15546	1.53482
H	4.09196	-2.32953	0.01574
H	4.17981	-1.18762	1.36961
H	2.21833	3.17080	1.03324
H	1.91363	3.94288	-0.54019
H	4.69050	-0.01689	-0.71946

H	3.15964	-0.54753	-1.45152
H	2.56137	1.81413	-1.68975
H	3.88854	2.50451	-0.72506
C	-2.44753	-1.55843	1.60359
H	-2.04475	-2.57141	1.64889
H	-3.16114	-1.44294	2.42344
H	-1.62072	-0.86240	1.78421
Na	0.76492	0.13714	0.75969

ACE4 Na B97-1 def2sv

47

O	-0.37291	-0.94682	-1.56035
O	-1.47486	1.98361	0.25518
O	0.96302	-2.18565	0.61758
O	1.19370	2.28892	-0.31726
O	2.60994	0.08804	0.55538
N	-2.27537	-0.76067	0.71303
C	-2.55442	-1.52432	-0.51354
C	-3.14645	0.40450	0.91524
C	-1.77260	-1.07549	-1.76159
C	-2.82489	1.61857	0.04656
C	0.45814	-2.07435	-1.78193
C	-1.03981	3.10173	-0.49221
C	0.64400	-2.95092	-0.53612
C	0.38583	3.42555	-0.08258
C	2.29911	-2.21028	1.09152
C	2.53456	2.42756	0.10903
C	3.19600	-1.18895	0.40045
C	3.26396	1.12285	-0.15310
H	-3.63865	-1.50257	-0.76957
H	-2.31121	-2.58152	-0.31598

H	-3.03481	0.72020	1.96764
H	-4.22086	0.14666	0.76539
H	-1.97638	-1.79513	-2.58004
H	-2.11305	-0.08538	-2.10671
H	-3.01023	1.42560	-1.02947
H	-3.49847	2.44919	0.34599
H	1.43251	-1.66713	-2.10547
H	0.06069	-2.69806	-2.60846
H	-1.68177	3.98473	-0.29214
H	-1.08855	2.88017	-1.57962
H	-0.28774	-3.49994	-0.30982
H	1.42923	-3.70495	-0.74198
H	0.74814	4.29571	-0.66871
H	0.41563	3.69797	0.99381
H	2.73916	-3.22135	0.98499
H	2.24184	-1.97134	2.16846
H	2.56755	2.67006	1.19250
H	3.04237	3.24588	-0.44279
H	4.20563	-1.21809	0.86294
H	3.31546	-1.42879	-0.67731
H	3.26538	0.89816	-1.24077
H	4.31784	1.22367	0.17962
C	-2.29192	-1.62546	1.89769
H	-3.29113	-2.08227	2.08728
H	-2.00790	-1.03800	2.78918
H	-1.55079	-2.43482	1.78027
Na	0.14523	0.18957	0.46534

ACE4 Na B97-1 def2tzv

47

O	0.10314	-1.44860	-0.41347

O	-2.48713	1.33894	0.35328
O	2.54361	-1.59785	0.97038
O	0.31994	2.30020	0.07462
O	2.85348	1.03379	0.01946
N	-3.02977	-1.65125	0.17415
C	-2.25695	-2.04464	-1.00427
C	-4.01884	-0.57934	0.02975
C	-1.00018	-1.21875	-1.35475
C	-3.56440	0.80220	-0.47229
C	1.02054	-2.51786	-0.78386
C	-2.09058	2.67342	-0.07798
C	1.92362	-2.79831	0.41340
C	-0.80016	3.08403	0.60378
C	3.80464	-1.14375	0.38523
C	1.55809	3.05794	-0.10846
C	3.61207	-0.02650	-0.63407
C	2.54743	2.17233	-0.83979
H	-2.92582	-2.00798	-1.87329
H	-1.95305	-3.09321	-0.88682
H	-4.50696	-0.45666	1.00112
H	-4.79591	-0.90290	-0.67828
H	-0.66500	-1.46547	-2.36964
H	-1.20176	-0.15100	-1.29854
H	-3.22587	0.74651	-1.51551
H	-4.42956	1.47950	-0.44155
H	1.59208	-2.21773	-1.66979
H	0.47281	-3.43665	-1.02375
H	-2.87765	3.39383	0.18071
H	-1.94875	2.68877	-1.16576
H	1.32929	-3.20868	1.23070

H	2.69065	-3.52757	0.13231
H	-0.63007	4.14722	0.40496
H	-0.86543	2.93600	1.68603
H	4.33509	-1.98024	-0.08087
H	4.39804	-0.77378	1.22258
H	1.95900	3.36422	0.86395
H	1.36298	3.95178	-0.71067
H	4.59420	0.34664	-0.94762
H	3.07016	-0.37186	-1.52145
H	2.11396	1.81925	-1.78251
H	3.46055	2.73746	-1.05578
C	-2.39044	-1.78535	1.48668
H	-1.81962	-2.71755	1.51416
H	-3.16249	-1.83754	2.26157
H	-1.71057	-0.96344	1.74591
Na	0.89225	0.18141	1.06041

ACE4 Na CAM-B3LYP def2sv

47

O	-1.05072	-2.00533	-0.39972
O	-1.45463	1.94933	-0.24677
O	1.72049	-2.11147	-0.29575
O	1.34027	2.26689	-0.30999
O	2.89455	0.19269	0.49622
N	-3.01826	-0.24656	0.99367
C	-3.30174	-1.45881	0.27976
C	-3.53793	0.99982	0.50650
C	-2.37599	-1.83573	-0.87351
C	-2.78670	1.68173	-0.63173
C	-0.32865	-3.03934	-1.03042
C	-0.82763	2.94294	-1.02147

C	0.94038	-3.28229	-0.25835
C	0.44966	3.35840	-0.34287
C	2.88670	-2.17070	0.49215
C	2.51377	2.52518	0.42822
C	3.69682	-0.93041	0.22051
C	3.50003	1.41844	0.16470
H	-4.32599	-1.40028	-0.13642
H	-3.29293	-2.30148	0.99543
H	-3.58071	1.71102	1.35065
H	-4.57845	0.85333	0.15555
H	-2.73854	-2.78405	-1.31100
H	-2.38998	-1.07353	-1.67320
H	-2.80043	1.06627	-1.55132
H	-3.30458	2.63230	-0.86595
H	-0.09176	-2.76953	-2.07809
H	-0.92145	-3.97255	-1.04231
H	-1.48102	3.83139	-1.11197
H	-0.61662	2.57069	-2.04372
H	0.69409	-3.54339	0.78866
H	1.49178	-4.13025	-0.70743
H	0.90285	4.20470	-0.89350
H	0.22460	3.69591	0.68660
H	3.48767	-3.06313	0.23535
H	2.61749	-2.23251	1.56432
H	2.27540	2.58579	1.50755
H	2.96199	3.48806	0.12019
H	4.60512	-0.93116	0.85265
H	4.01438	-0.91486	-0.83992
H	3.78971	1.41856	-0.90395
H	4.41221	1.58443	0.76896

C	-2.12253	-0.25722	2.11726
H	-2.16487	-1.24033	2.61782
H	-2.43046	0.50757	2.85386
H	-1.05483	-0.06026	1.88322
Na	0.50784	-0.00506	-0.22128

ACE4 Na CAM-B3LYP def2tzv

47

O	0.38999	-1.40667	-0.18152
O	-2.68284	1.04542	0.34529
O	2.89697	-1.17702	0.98713
O	0.05257	2.19972	0.50107
O	2.56248	1.32578	-0.23885
N	-2.78764	-1.91066	0.00982
C	-1.82546	-2.16508	-1.05329
C	-3.88023	-0.98482	-0.26798
C	-0.64995	-1.19080	-1.18385
C	-3.53533	0.44886	-0.66115
C	1.36307	-2.42844	-0.50668
C	-2.35239	2.43127	0.09034
C	2.37571	-2.48218	0.61948
C	-1.18414	2.80793	0.96756
C	3.93867	-0.59425	0.15828
C	0.89608	3.04707	-0.32624
C	3.38597	0.33401	-0.90213
C	1.80498	2.16800	-1.14430
H	-2.36552	-2.16409	-2.00282
H	-1.42341	-3.17459	-0.92791
H	-4.51761	-0.95987	0.61616
H	-4.48781	-1.38655	-1.08538
H	-0.20129	-1.27929	-2.17686

H	-0.97395	-0.16770	-1.02927
H	-3.02759	0.47963	-1.62976
H	-4.46376	1.02082	-0.75499
H	1.83115	-2.19137	-1.46402
H	0.88880	-3.40754	-0.59549
H	-3.21190	3.06627	0.32090
H	-2.09506	2.56968	-0.96364
H	1.90388	-2.85535	1.52426
H	3.19333	-3.15211	0.34885
H	-1.07278	3.89216	1.00333
H	-1.35093	2.44322	1.97807
H	4.53390	-1.37871	-0.31168
H	4.57438	-0.03480	0.83956
H	1.47173	3.71419	0.31644
H	0.28634	3.64728	-1.00320
H	4.21316	0.81924	-1.42457
H	2.78277	-0.20369	-1.63432
H	1.21891	1.53877	-1.81750
H	2.48008	2.78751	-1.73761
C	-2.27981	-1.94655	1.37787
H	-1.65423	-2.83054	1.50497
H	-3.11746	-2.02344	2.07179
H	-1.68889	-1.06968	1.65357
Na	1.06280	0.28248	1.27273

ACE4 Na MP2 def2tzv

47

O	0.53111	-1.34987	-0.01352
O	-2.82212	0.87010	0.36661
O	3.11748	-0.98272	0.96182
O	-0.10805	2.14553	0.59221

O	2.42132	1.45639	-0.34822
N	-2.66648	-2.10686	-0.04495
C	-1.60110	-2.19152	-1.07142
C	-3.80998	-1.23014	-0.39378
C	-0.51343	-1.09942	-1.03297
C	-3.52019	0.23057	-0.75906
C	1.51365	-2.37653	-0.37999
C	-2.53696	2.29438	0.13454
C	2.62318	-2.34137	0.67242
C	-1.39922	2.68715	1.06433
C	3.98798	-0.37274	-0.05975
C	0.59118	3.03425	-0.35846
C	3.21233	0.45279	-1.08055
C	1.48496	2.17538	-1.22919
H	-2.09277	-2.15973	-2.05264
H	-1.12379	-3.17573	-0.98359
H	-4.50270	-1.25253	0.45460
H	-4.32709	-1.67444	-1.25702
H	-0.02584	-1.02346	-2.01694
H	-0.92084	-0.13322	-0.74071
H	-2.89739	0.30353	-1.66193
H	-4.47036	0.74793	-0.95605
H	1.89468	-2.16671	-1.38697
H	1.06473	-3.37659	-0.37828
H	-3.43125	2.89386	0.34705
H	-2.24591	2.44633	-0.91372
H	2.25133	-2.70150	1.63255
H	3.45969	-2.97019	0.34526
H	-1.32748	3.77781	1.14208
H	-1.56208	2.26037	2.05506

H	4.55087	-1.15720	-0.58149
H	4.68237	0.26235	0.49221
H	1.16624	3.78124	0.19818
H	-0.13843	3.54020	-1.00173
H	3.91893	0.95324	-1.75488
H	2.53408	-0.16889	-1.67182
H	0.88022	1.45122	-1.78680
H	2.04124	2.80622	-1.93266
C	-2.16993	-1.98076	1.34871
H	-1.48525	-2.81118	1.54963
H	-3.03255	-2.06054	2.01963
H	-1.65016	-1.03848	1.55313
Na	1.19656	0.41694	1.45477

ACE4 Na PBE0 def2sv

47

O	-0.81427	-0.93189	-1.55878
O	-1.16795	2.18915	0.12275
O	0.63668	-2.25594	0.43161
O	1.54036	2.08900	-0.33723
O	2.57255	-0.26454	0.59914
N	-2.28822	-0.34184	0.89249
C	-2.61145	-1.46235	0.02058
C	-3.07580	0.86380	0.64850
C	-2.17429	-1.29645	-1.42946
C	-2.45633	1.84907	-0.32790
C	0.09752	-1.95093	-1.90271
C	-0.53238	3.17836	-0.64679
C	0.35990	-2.93663	-0.77511
C	0.88715	3.32126	-0.16204
C	1.88973	-2.48291	1.03729

C	2.84733	2.04713	0.17710
C	2.97286	-1.60351	0.45155
C	3.40637	0.66544	-0.04548
H	-3.70501	-1.67871	0.02107
H	-2.11442	-2.35142	0.43912
H	-3.18859	1.39422	1.60807
H	-4.10274	0.62068	0.30079
H	-2.37704	-2.24013	-1.96934
H	-2.76777	-0.51408	-1.93060
H	-2.39532	1.44436	-1.35573
H	-3.10123	2.74997	-0.36913
H	1.03129	-1.42853	-2.16871
H	-0.24925	-2.50327	-2.79847
H	-1.05499	4.15080	-0.55048
H	-0.53638	2.89314	-1.71841
H	-0.52050	-3.58481	-0.61221
H	1.19758	-3.59547	-1.06856
H	1.39439	4.12281	-0.73463
H	0.89343	3.60885	0.90886
H	2.18499	-3.54567	0.95941
H	1.76188	-2.24294	2.10587
H	2.83889	2.28300	1.26070
H	3.49634	2.78884	-0.32917
H	3.92965	-1.79183	0.97924
H	3.13210	-1.84029	-0.62077
H	3.45480	0.44548	-1.13125
H	4.43677	0.62044	0.35900
C	-2.34163	-0.74989	2.28773
H	-1.65576	-1.59796	2.45164
H	-3.36038	-1.06086	2.61055

H	-2.01330	0.07971	2.93551
Na	0.15048	0.17327	0.37370

ACE4 Na PBE0 def2tzv

47

O	-0.07439	-1.50019	-0.58805
O	-2.14006	1.56031	0.33082
O	2.21505	-1.81489	0.95469
O	0.57866	2.50100	-0.13986
O	2.82092	0.81466	0.13004
N	-3.03143	-1.40129	0.27665
C	-2.50253	-1.91517	-0.96533
C	-3.87320	-0.21682	0.24370
C	-1.20424	-1.28497	-1.48089
C	-3.33387	1.08584	-0.34233
C	0.75694	-2.64706	-0.86771
C	-1.78876	2.88110	-0.15764
C	1.54126	-2.96770	0.38683
C	-0.47254	3.33009	0.41068
C	3.51585	-1.47099	0.41167
C	1.91730	3.02757	0.02567
C	3.46830	-0.29450	-0.54103
C	2.86672	2.05608	-0.62029
H	-3.25886	-1.78475	-1.74948
H	-2.35168	-2.99729	-0.86360
H	-4.20562	-0.02641	1.26736
H	-4.77779	-0.43212	-0.34509
H	-0.96995	-1.68682	-2.47296
H	-1.29957	-0.20351	-1.56026
H	-3.11839	0.96757	-1.41130
H	-4.12331	1.84364	-0.25022

H	1.41507	-2.42533	-1.71438
H	0.14702	-3.51815	-1.12985
H	-2.56408	3.60031	0.13164
H	-1.72482	2.86536	-1.25178
H	0.86082	-3.30802	1.16698
H	2.25949	-3.76665	0.17678
H	-0.30981	4.37536	0.12766
H	-0.47169	3.26192	1.50376
H	3.95501	-2.33245	-0.10090
H	4.14314	-1.21946	1.26744
H	2.15094	3.14400	1.08954
H	2.00321	4.00458	-0.46041
H	4.49420	-0.02166	-0.81278
H	2.91995	-0.53646	-1.45736
H	2.56943	1.87457	-1.65847
H	3.88157	2.46538	-0.60999
C	-2.35423	-1.73667	1.51625
H	-1.86420	-2.70808	1.40875
H	-3.08163	-1.82027	2.32904
H	-1.59351	-1.00952	1.83660
Na	0.70106	-0.03314	0.90800

ACE4 Na PBE def2sv

47

O	-1.16183	-2.06181	-0.32814
O	-1.37156	2.03062	-0.24156
O	1.65555	-2.18216	-0.30810
O	1.46217	2.26518	-0.32741
O	2.93436	0.09966	0.52420
N	-3.07445	-0.13129	0.97680
C	-3.40125	-1.36413	0.30492

C	-3.52478	1.12287	0.42711
C	-2.48166	-1.84497	-0.82801
C	-2.69725	1.76811	-0.69489
C	-0.43982	-3.06900	-1.02427
C	-0.70473	3.03105	-0.99815
C	0.84641	-3.34872	-0.27727
C	0.59300	3.38944	-0.30699
C	2.82913	-2.28094	0.48720
C	2.65266	2.46450	0.42443
C	3.68813	-1.06366	0.21723
C	3.58991	1.30512	0.16189
H	-4.42529	-1.27733	-0.13520
H	-3.44156	-2.18084	1.06493
H	-3.59285	1.86413	1.25840
H	-4.55807	0.99586	0.01821
H	-2.89639	-2.80329	-1.22662
H	-2.45558	-1.11520	-1.67372
H	-2.67172	1.12329	-1.60807
H	-3.19503	2.72806	-0.98209
H	-0.21447	-2.74792	-2.07250
H	-1.03540	-4.01291	-1.07696
H	-1.33357	3.95243	-1.06985
H	-0.50246	2.67612	-2.04056
H	0.61132	-3.63140	0.77862
H	1.37626	-4.20831	-0.75691
H	1.06478	4.25683	-0.83137
H	0.38081	3.69631	0.74688
H	3.41030	-3.19910	0.22731
H	2.55746	-2.34036	1.57065
H	2.41201	2.53729	1.51427

H	3.15924	3.41272	0.12034
H	4.61613	-1.11707	0.83837
H	3.99448	-1.04857	-0.85842
H	3.86875	1.27927	-0.92120
H	4.52739	1.44731	0.75427
C	-2.20035	-0.13927	2.12426
H	-2.32818	-1.09236	2.68787
H	-2.46739	0.69944	2.80957
H	-1.10253	-0.03729	1.91066
Na	0.46517	0.00250	-0.23102

ACE4 Na PBE def2tzv

47

O	-0.35123	-1.87552	-0.50042
O	-2.18089	1.61930	0.24332
O	2.17145	-1.90019	0.79324
O	0.68270	2.35405	-0.21976
O	2.96087	0.76183	0.29029
N	-3.24142	-1.24251	0.55207
C	-2.81882	-2.01180	-0.61212
C	-4.03210	-0.02993	0.35297
C	-1.52895	-1.62113	-1.36126
C	-3.41353	1.14925	-0.40921
C	0.82727	-2.37301	-1.22961
C	-1.69863	2.84047	-0.41516
C	1.80576	-2.92309	-0.20748
C	-0.38667	3.28278	0.18619
C	3.58463	-1.48745	0.82246
C	2.02184	2.94461	-0.05378
C	3.86138	-0.32418	-0.11433
C	3.04309	1.95332	-0.56193

H	-3.63588	-1.97683	-1.35537
H	-2.70408	-3.06916	-0.31259
H	-4.35194	0.32169	1.34666
H	-4.95378	-0.28509	-0.20685
H	-1.45636	-2.24937	-2.26604
H	-1.52436	-0.56392	-1.66764
H	-3.18866	0.87293	-1.45558
H	-4.15414	1.97189	-0.43362
H	1.27846	-1.56105	-1.82613
H	0.53117	-3.18774	-1.91285
H	-2.44082	3.64914	-0.28347
H	-1.57109	2.65966	-1.49752
H	1.33949	-3.75140	0.34483
H	2.70223	-3.29967	-0.72462
H	-0.15465	4.29245	-0.19348
H	-0.44697	3.32500	1.28677
H	4.24376	-2.33509	0.57755
H	3.77301	-1.18309	1.86046
H	2.20168	3.17664	1.00975
H	2.09371	3.87714	-0.63872
H	4.91417	-0.00760	-0.01243
H	3.67654	-0.59379	-1.16908
H	2.83462	1.67925	-1.61045
H	4.05070	2.39842	-0.50250
C	-2.46685	-1.35359	1.78651
H	-2.08686	-2.38062	1.88447
H	-3.11424	-1.14561	2.65435
H	-1.60276	-0.66576	1.85214
Na	0.67121	0.07052	0.67587

ACE4 Na B2PLYP def2sv

47

O	-0.05431	-1.44473	-0.61971
O	-2.15827	1.48053	0.31160
O	2.18095	-1.73588	0.96722
O	0.54199	2.38400	-0.09154
O	2.80527	0.80003	0.08227
N	-2.96631	-1.42955	0.28073
C	-2.43241	-1.93392	-0.96265
C	-3.85174	-0.28824	0.25166
C	-1.15891	-1.27461	-1.50815
C	-3.33998	1.02920	-0.32904
C	0.76609	-2.57686	-0.85568
C	-1.81558	2.78427	-0.11613
C	1.53681	-2.87688	0.41958
C	-0.48036	3.21146	0.44351
C	3.49318	-1.42360	0.51900
C	1.83918	2.95829	-0.06461
C	3.50182	-0.29109	-0.49188
C	2.79919	1.98000	-0.69999
H	-3.21492	-1.84368	-1.73934
H	-2.23195	-3.01668	-0.84737
H	-4.18944	-0.10591	1.28552
H	-4.75777	-0.53517	-0.33859
H	-0.91040	-1.69413	-2.50057
H	-1.29188	-0.19022	-1.62120
H	-3.15645	0.92820	-1.41711
H	-4.14352	1.78430	-0.21142
H	1.44481	-2.37729	-1.70618
H	0.15903	-3.46482	-1.11004
H	-2.58568	3.51020	0.21288

H	-1.76653	2.82253	-1.22187
H	0.83149	-3.21844	1.19278
H	2.26522	-3.68616	0.23299
H	-0.30795	4.26304	0.15057
H	-0.47968	3.15614	1.54885
H	3.98417	-2.31292	0.08727
H	4.06299	-1.11099	1.40883
H	2.14686	3.17127	0.97682
H	1.85144	3.90735	-0.63209
H	4.54986	-0.01319	-0.71540
H	3.01754	-0.59096	-1.44032
H	2.47222	1.75309	-1.73227
H	3.81121	2.42458	-0.74409
C	-2.24171	-1.67442	1.50439
H	-1.74666	-2.65764	1.44882
H	-2.94019	-1.69861	2.36104
H	-1.46501	-0.92151	1.74563
Na	0.64882	0.19191	0.86382

ACE4 Na B2PLYP def2tzv

47

O	0.42491	-1.40995	-0.13170
O	-2.73815	1.00722	0.35315
O	2.97280	-1.14464	0.98161
O	0.00535	2.18621	0.51740
O	2.54962	1.37113	-0.25293
N	-2.79087	-1.97334	-0.00119
C	-1.78666	-2.18301	-1.05070
C	-3.89277	-1.04901	-0.30664
C	-0.62939	-1.16860	-1.13507
C	-3.55736	0.39708	-0.69303

C	1.41883	-2.41146	-0.51185
C	-2.41736	2.41076	0.09845
C	2.46843	-2.46550	0.59042
C	-1.24872	2.79153	0.98602
C	3.99178	-0.52624	0.12628
C	0.81606	3.04698	-0.35286
C	3.39568	0.38852	-0.93250
C	1.73633	2.17014	-1.17225
H	-2.30786	-2.18211	-2.01238
H	-1.35902	-3.18289	-0.92406
H	-4.55246	-1.03544	0.56279
H	-4.46904	-1.46056	-1.14324
H	-0.17450	-1.20144	-2.13035
H	-0.97140	-0.16075	-0.92397
H	-3.01668	0.43689	-1.64451
H	-4.49158	0.95716	-0.81285
H	1.85378	-2.14006	-1.47658
H	0.96205	-3.39977	-0.60732
H	-3.28453	3.03660	0.33010
H	-2.15704	2.54999	-0.95557
H	2.03450	-2.86973	1.50164
H	3.29806	-3.10338	0.27692
H	-1.14041	3.87734	1.02258
H	-1.41142	2.41892	1.99499
H	4.59680	-1.29802	-0.35514
H	4.62168	0.04849	0.80135
H	1.38256	3.74684	0.26391
H	0.17271	3.60882	-1.03363
H	4.20230	0.89541	-1.46904
H	2.79208	-0.16675	-1.65188

H	1.15478	1.50306	-1.81334
H	2.37992	2.79522	-1.79615
C	-2.28295	-1.95636	1.37959
H	-1.65090	-2.83283	1.53362
H	-3.12734	-2.01775	2.06817
H	-1.70037	-1.06489	1.62802
Na	1.10108	0.30058	1.35262

ACE4 Na DSDPBEP86 def2sv

47

O	-0.75686	-0.79897	-1.59284
O	-1.19345	2.16508	0.19127
O	0.56445	-2.23022	0.40162
O	1.50200	2.07429	-0.33429
O	2.50559	-0.26912	0.60924
N	-2.21838	-0.41397	0.87132
C	-2.52912	-1.50237	-0.05105
C	-3.05771	0.77359	0.69969
C	-2.10404	-1.24194	-1.49349
C	-2.48679	1.80950	-0.25633
C	0.20344	-1.78031	-1.94433
C	-0.57878	3.16235	-0.59852
C	0.39674	-2.84113	-0.86989
C	0.84810	3.31132	-0.13259
C	1.79480	-2.47354	1.06234
C	2.81575	2.03402	0.18366
C	2.90953	-1.61800	0.49625
C	3.36330	0.64635	-0.04155
H	-3.61884	-1.74074	-0.05153
H	-2.00427	-2.39673	0.31807
H	-3.15590	1.26606	1.68194

H	-4.08589	0.50665	0.36979
H	-2.25233	-2.16563	-2.08502
H	-2.73296	-0.46432	-1.95618
H	-2.42791	1.44008	-1.29660
H	-3.15180	2.69743	-0.25564
H	1.14331	-1.22331	-2.09585
H	-0.06665	-2.26931	-2.90209
H	-1.11040	4.12973	-0.49543
H	-0.59607	2.86828	-1.66762
H	-0.47979	-3.51206	-0.81805
H	1.27005	-3.46584	-1.13525
H	1.34868	4.11826	-0.70418
H	0.86734	3.58118	0.94263
H	2.07019	-3.54418	1.01063
H	1.62751	-2.20847	2.11946
H	2.80381	2.26468	1.26822
H	3.46318	2.77691	-0.32368
H	3.84779	-1.79831	1.05914
H	3.09928	-1.87129	-0.56706
H	3.40488	0.42310	-1.12689
H	4.39110	0.58247	0.36758
C	-2.24247	-0.89851	2.24889
H	-3.24751	-1.26209	2.56086
H	-1.93708	-0.08880	2.93328
H	-1.52118	-1.72675	2.35457
Na	0.14634	0.19675	0.39793

ACE4 Na DSDPBEP86 def2tzv

47

O	0.48577	-1.32039	-0.10932
O	-2.77015	0.91558	0.37121

O	3.01797	-1.06257	0.98755
O	-0.06570	2.14391	0.54340
O	2.47482	1.40558	-0.29075
N	-2.69046	-2.04290	-0.01882
C	-1.67648	-2.15409	-1.07586
C	-3.83596	-1.16745	-0.31181
C	-0.57364	-1.07927	-1.10108
C	-3.55445	0.29053	-0.68947
C	1.45625	-2.34070	-0.48526
C	-2.48609	2.32528	0.12232
C	2.51764	-2.38507	0.60821
C	-1.32668	2.72298	1.01619
C	3.98687	-0.43588	0.08672
C	0.67254	3.00000	-0.38905
C	3.32925	0.43623	-0.97290
C	1.59166	2.12260	-1.20892
H	-2.19847	-2.13440	-2.04046
H	-1.20041	-3.13989	-0.99382
H	-4.49134	-1.18537	0.56512
H	-4.39870	-1.60237	-1.14954
H	-0.11427	-1.03725	-2.09859
H	-0.96480	-0.09990	-0.83186
H	-3.00212	0.35570	-1.63741
H	-4.50868	0.82068	-0.81688
H	1.88283	-2.09470	-1.46441
H	0.98593	-3.32823	-0.55418
H	-3.37088	2.93172	0.35304
H	-2.22377	2.47339	-0.93396
H	2.09122	-2.78712	1.52784
H	3.35023	-3.02224	0.28908

H	-1.23605	3.81364	1.06172
H	-1.48365	2.33543	2.02370
H	4.59991	-1.20368	-0.39922
H	4.62302	0.17508	0.72850
H	1.23696	3.74758	0.17741
H	-0.02132	3.51013	-1.06650
H	4.10477	0.95662	-1.54786
H	2.72013	-0.15519	-1.66269
H	1.00615	1.39936	-1.78809
H	2.18173	2.74078	-1.89507
C	-2.15538	-1.96608	1.35102
H	-1.45383	-2.79286	1.50161
H	-2.98207	-2.07916	2.05927
H	-1.63592	-1.02589	1.56964
Na	1.13834	0.35064	1.40345

ACE4 Na HSE06 def2sv

47

O	-0.37906	-0.92401	-1.55062
O	-1.45190	1.97222	0.25416
O	0.93600	-2.17157	0.60452
O	1.19873	2.26321	-0.31508
O	2.58754	0.06989	0.55300
N	-2.26126	-0.74708	0.71128
C	-2.53953	-1.50468	-0.50574
C	-3.12110	0.41315	0.91044
C	-1.77100	-1.05502	-1.74747
C	-2.79617	1.61577	0.04598
C	0.44772	-2.04328	-1.77910
C	-1.01583	3.08416	-0.48740
C	0.62466	-2.92467	-0.54954

C	0.40204	3.39839	-0.08185
C	2.26065	-2.20962	1.08642
C	2.53245	2.39471	0.11028
C	3.16336	-1.20389	0.40415
C	3.24986	1.09436	-0.14717
H	-3.62375	-1.49089	-0.75513
H	-2.29111	-2.55939	-0.31036
H	-3.00867	0.73038	1.96055
H	-4.19524	0.15989	0.76394
H	-1.97519	-1.77189	-2.56574
H	-2.11685	-0.06815	-2.09087
H	-2.98147	1.42230	-1.02818
H	-3.46544	2.44877	0.34087
H	1.42248	-1.63577	-2.09452
H	0.05541	-2.65645	-2.61347
H	-1.65317	3.96778	-0.28660
H	-1.06688	2.86604	-1.57341
H	-0.30639	-3.47590	-0.33469
H	1.40869	-3.67714	-0.75567
H	0.76844	4.26587	-0.66553
H	0.43389	3.67040	0.99267
H	2.69037	-3.22371	0.98608
H	2.19983	-1.96985	2.16106
H	2.56513	2.63753	1.19180
H	3.04496	3.20923	-0.43868
H	4.16908	-1.23964	0.86964
H	3.28708	-1.44599	-0.67078
H	3.25610	0.87077	-1.23309
H	4.30112	1.18708	0.18930
C	-2.27665	-1.60452	1.88775

H	-1.53596	-2.41208	1.77277
H	-3.27345	-2.06157	2.07780
H	-1.99430	-1.01902	2.77864
Na	0.14734	0.19035	0.46403

ACE4 Na HSE06 def2tzv

47

O	0.09774	-1.42356	-0.40601
O	-2.46275	1.32753	0.35201
O	2.51814	-1.59788	0.95914
O	0.32696	2.28295	0.07871
O	2.83655	1.01670	0.02190
N	-3.00683	-1.63416	0.16555
C	-2.24122	-2.00455	-1.01150
C	-3.99075	-0.57028	0.03778
C	-0.99520	-1.17806	-1.33940
C	-3.53851	0.79797	-0.46190
C	0.99555	-2.49480	-0.77841
C	-2.06732	2.65116	-0.07941
C	1.89088	-2.78464	0.40730
C	-0.78739	3.06320	0.59620
C	3.76920	-1.15360	0.37033
C	1.55816	3.03302	-0.09922
C	3.57726	-0.04007	-0.63543
C	2.54022	2.15411	-0.82508
H	-2.90878	-1.95256	-1.87918
H	-1.93949	-3.05505	-0.91708
H	-4.46738	-0.44939	1.01369
H	-4.77838	-0.88824	-0.65943
H	-0.65661	-1.40127	-2.35741
H	-1.20043	-0.11348	-1.26187

H	-3.20670	0.73965	-1.50622
H	-4.39973	1.47817	-0.43106
H	1.56848	-2.20058	-1.66409
H	0.43942	-3.40615	-1.02137
H	-2.85359	3.37286	0.17256
H	-1.92452	2.66365	-1.16604
H	1.29525	-3.19245	1.22326
H	2.64913	-3.52085	0.12527
H	-0.61865	4.12429	0.39137
H	-0.85629	2.92619	1.67850
H	4.29070	-1.99066	-0.10203
H	4.37404	-0.79180	1.20156
H	1.95833	3.33757	0.87276
H	1.36784	3.92875	-0.69795
H	4.55794	0.32523	-0.95817
H	3.02697	-0.37851	-1.51883
H	2.10952	1.80954	-1.77085
H	3.45455	2.71636	-1.03678
C	-2.37363	-1.79079	1.46590
H	-1.79624	-2.71767	1.47585
H	-3.14319	-1.86550	2.23973
H	-1.70005	-0.97207	1.74457
Na	0.89536	0.17920	1.06557

ACE4 Na M062X def2sv

47

O	-0.77570	-0.69572	-1.56704
O	-1.15163	2.14225	0.17568
O	0.52680	-2.22864	0.35233
O	1.53628	2.02635	-0.28195
O	2.49274	-0.32737	0.61385

N	-2.23835	-0.40502	0.87991
C	-2.51442	-1.49326	-0.05189
C	-3.05364	0.78950	0.65759
C	-2.09971	-1.19578	-1.48932
C	-2.43671	1.80343	-0.29465
C	0.21659	-1.62324	-1.95772
C	-0.51924	3.13771	-0.59385
C	0.38583	-2.75490	-0.95488
C	0.90232	3.27216	-0.10746
C	1.73699	-2.52343	1.02047
C	2.84539	1.97344	0.23332
C	2.87547	-1.67640	0.48984
C	3.37754	0.58088	0.00027
H	-3.59447	-1.76203	-0.05163
H	-1.95676	-2.37202	0.30647
H	-3.17886	1.29798	1.62678
H	-4.07076	0.52948	0.29638
H	-2.20241	-2.11505	-2.09343
H	-2.76431	-0.44418	-1.94229
H	-2.35613	1.42378	-1.32856
H	-3.08274	2.70199	-0.31817
H	1.15124	-1.04310	-2.02998
H	-0.01012	-2.04160	-2.95645
H	-1.04495	4.10558	-0.49154
H	-0.52333	2.84875	-1.66296
H	-0.49365	-3.42217	-0.96805
H	1.26172	-3.36353	-1.24073
H	1.42415	4.06150	-0.67993
H	0.90983	3.55364	0.96339
H	1.98537	-3.59618	0.93529

H	1.56446	-2.28838	2.08200
H	2.83394	2.19843	1.31742
H	3.49686	2.71175	-0.27022
H	3.79968	-1.88002	1.06294
H	3.07826	-1.91454	-0.57384
H	3.43838	0.37310	-1.08576
H	4.39210	0.49141	0.43067
C	-2.34329	-0.88415	2.25330
H	-1.64828	-1.72684	2.39817
H	-3.36971	-1.22636	2.50628
H	-2.06078	-0.08064	2.95141
Na	0.12936	0.18577	0.46217

ACE4 Na M062X def2tzv

47

O	0.52764	-1.25234	-0.12217
O	-2.75167	0.85879	0.37089
O	3.01000	-0.98191	0.99108
O	-0.09777	2.12974	0.56332
O	2.39956	1.40844	-0.33591
N	-2.57899	-2.05400	-0.04182
C	-1.59039	-2.12991	-1.11383
C	-3.76089	-1.23310	-0.30349
C	-0.50487	-1.04583	-1.12998
C	-3.52776	0.22827	-0.67369
C	1.45767	-2.32749	-0.38924
C	-2.50094	2.26515	0.15183
C	2.49339	-2.30957	0.72178
C	-1.35571	2.66660	1.05292
C	3.91681	-0.41394	0.01176
C	0.60694	2.99477	-0.36716

C	3.19300	0.41599	-1.03016
C	1.50640	2.13408	-1.21625
H	-2.12107	-2.09107	-2.06794
H	-1.10296	-3.10832	-1.07159
H	-4.39263	-1.27330	0.58499
H	-4.32744	-1.68146	-1.12525
H	-0.02793	-1.01440	-2.11551
H	-0.92018	-0.06888	-0.89247
H	-2.99074	0.31098	-1.62522
H	-4.49197	0.73348	-0.78645
H	1.90956	-2.18696	-1.37420
H	0.95557	-3.29639	-0.38167
H	-3.39563	2.84620	0.38801
H	-2.23821	2.44071	-0.89695
H	2.03610	-2.62420	1.65689
H	3.31147	-2.99033	0.47949
H	-1.28813	3.75273	1.13297
H	-1.50821	2.24748	2.04474
H	4.49023	-1.20377	-0.47817
H	4.59825	0.21982	0.57434
H	1.18180	3.73096	0.19656
H	-0.10044	3.51550	-1.01554
H	3.92358	0.90473	-1.67874
H	2.53278	-0.19558	-1.64630
H	0.90794	1.42010	-1.78804
H	2.08013	2.75534	-1.90661
C	-2.03323	-1.99051	1.31590
H	-1.28020	-2.77180	1.43272
H	-2.83523	-2.17437	2.03145
H	-1.57033	-1.02912	1.55231

Na 1.14657 0.39829 1.37718

ACE4 Na M06 def2sv

47

O	-0.10619	-0.64970	-1.48029
O	-1.70909	1.72290	0.39114
O	1.14922	-2.09170	0.61741
O	0.86830	2.30796	-0.25336
O	2.53416	0.30111	0.57669
N	-2.14730	-1.09619	0.56297
C	-2.27866	-1.58796	-0.80633
C	-3.13735	-0.09320	0.93146
C	-1.45553	-0.83265	-1.84229
C	-3.00889	1.22322	0.20302
C	0.81486	-1.67485	-1.76648
C	-1.41792	2.88312	-0.34489
C	0.95845	-2.69082	-0.64520
C	-0.03539	3.34710	0.02281
C	2.46834	-1.99958	1.10418
C	2.20108	2.58961	0.08722
C	3.26060	-0.89399	0.44784
C	3.03022	1.36749	-0.19348
H	-3.34384	-1.58872	-1.13945
H	-1.96836	-2.64888	-0.82019
H	-3.01965	0.10814	2.01361
H	-4.17734	-0.47561	0.78656
H	-1.52440	-1.36744	-2.81277
H	-1.85863	0.18140	-2.00997
H	-3.22975	1.12101	-0.88160
H	-3.76033	1.93503	0.60780
H	1.78226	-1.17063	-1.95109

H	0.53713	-2.20561	-2.70147
H	-2.15191	3.68714	-0.12499
H	-1.47194	2.66597	-1.43522
H	0.04393	-3.30902	-0.56862
H	1.79396	-3.38092	-0.88582
H	0.22011	4.26010	-0.55627
H	0.00480	3.60875	1.10348
H	3.00285	-2.96447	0.98562
H	2.37627	-1.79452	2.18621
H	2.27090	2.85839	1.16459
H	2.58850	3.44793	-0.50192
H	4.25784	-0.80761	0.93094
H	3.44066	-1.11834	-0.62643
H	2.97557	1.10715	-1.27439
H	4.09335	1.57249	0.05313
C	-2.15864	-2.18572	1.52397
H	-1.33871	-2.89382	1.30724
H	-3.12245	-2.74841	1.52352
H	-1.99609	-1.79107	2.54333
Na	0.11528	0.17724	0.72724

ACE4 Na M06 def2tzv

47

O	0.55602	-1.30369	-0.09704
O	-2.79104	0.83542	0.34547
O	3.08055	-0.94317	0.95887
O	-0.16486	2.15856	0.58747
O	2.34685	1.44330	-0.33967
N	-2.53913	-2.06325	-0.01964
C	-1.55200	-2.18700	-1.07635
C	-3.73330	-1.28539	-0.30805

C	-0.46124	-1.11392	-1.11470
C	-3.54094	0.17518	-0.68996
C	1.53130	-2.31892	-0.39693
C	-2.54744	2.22878	0.08807
C	2.59305	-2.27308	0.68146
C	-1.44674	2.67536	1.01529
C	3.92957	-0.32691	-0.03396
C	0.54497	3.01334	-0.34078
C	3.16074	0.48848	-1.04977
C	1.43695	2.16127	-1.19775
H	-2.07543	-2.17584	-2.03756
H	-1.06678	-3.16873	-1.00169
H	-4.38117	-1.33577	0.57139
H	-4.27935	-1.75665	-1.13480
H	0.02075	-1.10500	-2.10134
H	-0.87970	-0.12601	-0.91217
H	-3.00553	0.26568	-1.64579
H	-4.52202	0.65142	-0.81736
H	1.95562	-2.14140	-1.39278
H	1.08321	-3.31830	-0.40053
H	-3.45831	2.81434	0.25784
H	-2.24016	2.37152	-0.95794
H	2.17365	-2.61758	1.62662
H	3.42166	-2.93316	0.40599
H	-1.41037	3.76650	1.07674
H	-1.62832	2.27631	2.01315
H	4.52725	-1.08435	-0.55139
H	4.60350	0.32628	0.52035
H	1.12527	3.75225	0.21976
H	-0.15743	3.54309	-0.99151

H	3.86843	1.00551	-1.70697
H	2.51807	-0.14225	-1.67211
H	0.83222	1.44782	-1.77158
H	1.99145	2.79170	-1.90062
C	-2.02402	-2.02632	1.33961
H	-1.30281	-2.83883	1.47172
H	-2.84563	-2.17612	2.04385
H	-1.51665	-1.08742	1.59776
Na	1.16882	0.42265	1.47187

ACE4 Na MP2 def2sv

47

O	-1.88674	-0.55168	-1.35358
O	-0.01287	2.42102	-0.14362
O	0.04723	-2.35328	-0.46782
O	2.35259	1.16726	-0.61453
O	2.18738	-1.23009	0.70747
N	-1.78036	0.72793	1.23456
C	-2.83963	-0.20438	0.87363
C	-2.10018	2.11937	0.89700
C	-3.07965	-0.28435	-0.62524
C	-1.40090	2.59187	-0.36619
C	-1.76077	-1.86301	-1.88288
C	0.82571	2.88556	-1.18250
C	-1.22046	-2.84087	-0.86117
C	2.23888	2.57383	-0.75569
C	0.63581	-3.02359	0.63157
C	3.38718	0.77128	0.26713
C	2.09572	-2.64237	0.63919
C	3.44343	-0.73418	0.28165
H	-3.80786	0.05871	1.36307

H	-2.55102	-1.20112	1.24704
H	-1.75732	2.77178	1.71803
H	-3.19513	2.27798	0.80368
H	-3.83822	-1.06385	-0.82610
H	-3.49052	0.66736	-1.00390
H	-1.71274	2.01519	-1.25740
H	-1.63833	3.66045	-0.54495
H	-1.04493	-1.78945	-2.71816
H	-2.72750	-2.22228	-2.28376
H	0.70849	3.97744	-1.33229
H	0.58597	2.37523	-2.13774
H	-1.89706	-2.92198	0.01131
H	-1.12823	-3.84834	-1.31537
H	2.96752	2.95433	-1.49813
H	2.43419	3.06908	0.21448
H	0.54694	-4.12177	0.52121
H	0.13807	-2.72453	1.57596
H	3.18621	1.15855	1.28591
H	4.36670	1.16602	-0.06821
H	2.61698	-3.11609	1.49454
H	2.56123	-3.00279	-0.29820
H	3.66937	-1.10715	-0.73675
H	4.25246	-1.06701	0.96194
C	-1.41776	0.57495	2.64013
H	-1.11419	-0.46904	2.82857
H	-2.25344	0.82664	3.32995
H	-0.56083	1.22802	2.87324
Na	0.25461	0.01660	-0.11027

ACE4 Na SCS-MP2 def2sv

47

O	-0.13964	-2.41857	-0.15387
O	-1.84535	0.65454	-1.35840
O	2.29447	-1.28110	-0.60142
O	0.18039	2.35301	-0.45749
O	2.27024	1.12792	0.71942
N	-1.84779	-0.65168	1.23102
C	-2.22628	-2.03011	0.87876
C	-2.85763	0.33531	0.85301
C	-1.53556	-2.53085	-0.38327
C	-3.06621	0.43909	-0.65314
C	0.68469	-2.92315	-1.18998
C	-1.64754	1.96036	-1.88719
C	2.11221	-2.68264	-0.75135
C	-1.05960	2.90951	-0.85898
C	3.35985	-0.94033	0.27160
C	0.80764	3.00313	0.63660
C	3.49738	0.56396	0.28365
C	2.24996	2.54592	0.64478
H	-3.32674	-2.13902	0.77251
H	-1.92117	-2.70148	1.70033
H	-2.52833	1.31545	1.23903
H	-3.84669	0.11538	1.32231
H	-1.81599	-3.58971	-0.56197
H	-1.81766	-1.94321	-1.27752
H	-3.51659	-0.48808	-1.04944
H	-3.78174	1.25768	-0.85899
H	0.47653	-2.39896	-2.14574
H	0.51412	-4.00844	-1.34157
H	-2.59250	2.37073	-2.29255
H	-0.93199	1.84912	-2.71974

H	2.27843	-3.19260	0.21743
H	2.82615	-3.09298	-1.49344
H	-0.90852	3.91052	-1.31341
H	-1.73576	3.02748	0.01051
H	4.31316	-1.38799	-0.07513
H	3.14839	-1.31653	1.29310
H	0.29613	2.73981	1.58489
H	0.77573	4.10403	0.51467
H	4.32804	0.85444	0.95850
H	3.73479	0.92322	-0.73767
H	2.73324	2.87832	-0.29459
H	2.79550	2.99509	1.49924
C	-1.51678	-0.52540	2.65202
H	-2.38274	-0.74439	3.31639
H	-1.17074	0.50280	2.85854
H	-0.69631	-1.21931	2.90318
Na	0.25401	-0.02679	-0.07373

ACE4 Na SCS-MP2 def2tzv

47

O	0.47667	-1.41948	-0.08788
O	-2.76574	0.97111	0.34831
O	3.07037	-1.09247	0.93371
O	-0.02867	2.19177	0.53834
O	2.53519	1.43349	-0.32149
N	-2.79527	-2.03595	-0.04674
C	-1.73431	-2.19289	-1.06786
C	-3.87854	-1.09687	-0.41398
C	-0.58108	-1.15763	-1.09275
C	-3.51433	0.36102	-0.75962
C	1.49590	-2.38069	-0.53606

C	-2.45381	2.38654	0.08705
C	2.58855	-2.42984	0.53436
C	-1.30095	2.78523	1.00141
C	4.04812	-0.43799	0.04104
C	0.71829	3.04573	-0.40975
C	3.39645	0.45961	-1.01565
C	1.63764	2.15836	-1.23870
H	-2.23088	-2.17482	-2.04792
H	-1.29297	-3.19179	-0.94269
H	-4.59298	-1.10101	0.41800
H	-4.39655	-1.49800	-1.29785
H	-0.10980	-1.15614	-2.08673
H	-0.93251	-0.15681	-0.84588
H	-2.89873	0.40944	-1.66949
H	-4.44135	0.92502	-0.94146
H	1.89476	-2.06130	-1.50581
H	1.06234	-3.38248	-0.64994
H	-3.33989	3.00302	0.28861
H	-2.16127	2.51279	-0.96469
H	2.20246	-2.86964	1.45554
H	3.43123	-3.03132	0.17060
H	-1.20179	3.87681	1.03875
H	-1.47012	2.40295	2.00988
H	4.66219	-1.19921	-0.45667
H	4.67889	0.16063	0.70115
H	1.28574	3.79538	0.15237
H	0.01962	3.54921	-1.08899
H	4.17904	0.98553	-1.57789
H	2.78621	-0.12022	-1.71532
H	1.04408	1.43592	-1.81238

H	2.22669	2.77625	-1.92777
C	-2.35592	-1.98308	1.37212
H	-1.72187	-2.85487	1.57190
H	-3.25113	-2.05359	1.99887
H	-1.80596	-1.07193	1.65089
Na	1.02206	0.23287	1.82769

ACE4 NoMetal HSE06 def2sv

46

O	-0.00632	-2.30470	-0.63058
O	-2.37751	1.35163	0.16855
O	2.49515	-1.85067	0.69100
O	0.23388	2.45090	-0.35417
O	2.59494	1.02971	0.46279
N	-2.66307	-1.55250	0.87634
C	-2.36243	-2.35015	-0.28733
C	-3.70420	-0.56338	0.73672
C	-1.22105	-1.87934	-1.18906
C	-3.48286	0.59113	-0.23190
C	1.13263	-1.84857	-1.30201
C	-2.13077	2.45808	-0.65139
C	2.35625	-2.39480	-0.59922
C	-0.92601	3.20731	-0.14483
C	3.63860	-1.07250	0.92132
C	1.38454	3.06172	0.15837
C	3.69755	0.22859	0.14674
C	2.60590	2.25944	-0.20665
H	-3.28259	-2.43158	-0.89152
H	-2.09730	-3.38187	0.02473
H	-3.90233	-0.13722	1.73755
H	-4.63723	-1.06899	0.41858

H	-1.34166	-2.31153	-2.20667
H	-1.23288	-0.77487	-1.29193
H	-3.33497	0.20876	-1.26489
H	-4.40063	1.21967	-0.25310
H	1.16847	-0.73902	-1.30535
H	1.13054	-2.19397	-2.35903
H	-3.00288	3.14574	-0.65479
H	-1.95376	2.13993	-1.70065
H	2.24567	-3.49207	-0.50557
H	3.25109	-2.20523	-1.22081
H	-0.85811	4.18081	-0.67526
H	-1.06244	3.42587	0.93515
H	4.56411	-1.64508	0.70164
H	3.63437	-0.84483	2.00056
H	1.31914	3.15589	1.26303
H	1.50206	4.08547	-0.25561
H	4.64868	0.74143	0.40760
H	3.73137	0.03538	-0.94679
H	2.62894	2.10589	-1.30626
H	3.50894	2.84552	0.06595
C	-1.54897	-1.22535	1.73189
H	-0.99915	-2.14831	1.99214
H	-1.92824	-0.77695	2.66801
H	-0.81386	-0.51948	1.29231

ACE4 NoMetal HSE06 def2tzv

46

O	-0.02614	-2.36426	-0.55748
O	-2.40727	1.39957	0.19904
O	2.61080	-1.91457	0.77521
O	0.28808	2.52545	-0.36516

O	2.71731	1.04688	0.47781
N	-2.86295	-1.57298	0.79813
C	-2.43961	-2.33694	-0.36778
C	-3.85317	-0.52807	0.57451
C	-1.23262	-1.82143	-1.15367
C	-3.50796	0.63580	-0.34666
C	1.17949	-1.93650	-1.22892
C	-2.10798	2.57673	-0.58498
C	2.36843	-2.53339	-0.51482
C	-0.90119	3.26944	-0.01576
C	3.79350	-1.08485	0.85869
C	1.49265	3.10376	0.18573
C	3.76735	0.17026	0.01818
C	2.68039	2.29439	-0.25369
H	-3.29409	-2.40798	-1.04614
H	-2.19487	-3.36017	-0.05789
H	-4.13513	-0.12334	1.55048
H	-4.75432	-0.98983	0.15228
H	-1.29605	-2.15338	-2.19797
H	-1.18962	-0.72740	-1.13905
H	-3.24997	0.27985	-1.35199
H	-4.39453	1.27774	-0.44077
H	1.24287	-0.84306	-1.23150
H	1.16873	-2.28759	-2.26934
H	-2.96270	3.26344	-0.56210
H	-1.91776	2.29902	-1.62829
H	2.17855	-3.59295	-0.33254
H	3.25441	-2.45178	-1.15224
H	-0.83840	4.28332	-0.42913
H	-0.99501	3.34595	1.07336

H	4.67915	-1.66646	0.57473
H	3.87987	-0.81853	1.91156
H	1.43677	3.12531	1.27991
H	1.60979	4.13290	-0.17469
H	4.74287	0.66363	0.11771
H	3.62293	-0.06261	-1.04378
H	2.61598	2.09895	-1.33020
H	3.59737	2.86361	-0.06066
C	-1.82951	-1.30203	1.79012
H	-1.34194	-2.23955	2.06816
H	-2.29168	-0.87850	2.68461
H	-1.05335	-0.60746	1.44752

ACE4 NoMetal SCS-MP2 def2sv

46

O	-0.03660	-2.23604	-0.70996
O	-2.35155	1.32449	0.18056
O	2.44911	-1.89124	0.64136
O	0.26590	2.38121	-0.35447
O	2.60412	0.97221	0.52235
N	-2.67227	-1.56876	0.91393
C	-2.38592	-2.27948	-0.32221
C	-3.70707	-0.55204	0.82190
C	-1.26009	-1.71745	-1.19791
C	-3.50672	0.59102	-0.16915
C	1.10271	-1.70079	-1.34882
C	-2.09846	2.39922	-0.69881
C	2.32189	-2.32971	-0.70276
C	-0.89782	3.16768	-0.20022
C	3.62121	-1.15693	0.93402
C	1.40469	3.01091	0.19483

C	3.72619	0.17523	0.21092
C	2.63902	2.21337	-0.15002
H	-3.32114	-2.32935	-0.91045
H	-2.10021	-3.32620	-0.08935
H	-3.84399	-0.11447	1.82861
H	-4.66429	-1.04471	0.55352
H	-1.40612	-2.02609	-2.25712
H	-1.24724	-0.61051	-1.15882
H	-3.41883	0.20108	-1.20554
H	-4.40476	1.24818	-0.14508
H	1.14009	-0.59889	-1.22374
H	1.08788	-1.93409	-2.43678
H	-2.97256	3.08408	-0.73895
H	-1.90590	2.02988	-1.72820
H	2.19406	-3.42945	-0.70185
H	3.22663	-2.09843	-1.29585
H	-0.80431	4.11502	-0.77383
H	-1.05525	3.42908	0.86696
H	4.53263	-1.75138	0.70940
H	3.59307	-0.97235	2.02172
H	1.31121	3.09607	1.29802
H	1.51924	4.03619	-0.21760
H	4.66736	0.67313	0.53239
H	3.79666	0.02788	-0.88684
H	2.68177	2.06103	-1.24888
H	3.53807	2.79393	0.14935
C	-1.50750	-1.23710	1.72043
H	-0.93898	-2.16121	1.93330
H	-1.85017	-0.81217	2.68218
H	-0.80951	-0.51364	1.25399

ACE4 NoMetal SCS-MP2 def2tzv

46

O	-0.00434	-2.35598	-0.61890
O	-2.43844	1.33956	0.17841
O	2.65711	-1.94646	0.75413
O	0.26364	2.50138	-0.36508
O	2.72100	1.04566	0.51707
N	-2.87698	-1.63049	0.86173
C	-2.42521	-2.33924	-0.35891
C	-3.89541	-0.57917	0.62636
C	-1.23600	-1.71404	-1.12411
C	-3.56501	0.55816	-0.35440
C	1.20981	-1.82441	-1.25822
C	-2.14479	2.51780	-0.65029
C	2.41008	-2.49286	-0.59506
C	-0.96050	3.25094	-0.04650
C	3.85372	-1.09316	0.85566
C	1.45578	3.10430	0.24903
C	3.80178	0.18463	0.02180
C	2.67769	2.32712	-0.20521
H	-3.29095	-2.42112	-1.02762
H	-2.12266	-3.35744	-0.08005
H	-4.14560	-0.13894	1.59900
H	-4.79927	-1.07403	0.24196
H	-1.32133	-1.90856	-2.20332
H	-1.17071	-0.63334	-0.95320
H	-3.30918	0.17226	-1.35126
H	-4.44731	1.20898	-0.45337
H	1.25073	-0.73680	-1.12317
H	1.19128	-2.06231	-2.33202

H	-3.02084	3.18074	-0.66263
H	-1.90946	2.20882	-1.67771
H	2.21228	-3.56156	-0.47493
H	3.30299	-2.36705	-1.22069
H	-0.89130	4.25896	-0.47801
H	-1.08350	3.32864	1.04126
H	4.73893	-1.66920	0.55084
H	3.92914	-0.84784	1.91642
H	1.37234	3.07640	1.34283
H	1.54615	4.14787	-0.08262
H	4.76660	0.70232	0.12637
H	3.64104	-0.03028	-1.04286
H	2.62138	2.14697	-1.28682
H	3.58636	2.90181	0.01977
C	-1.77877	-1.26979	1.79249
H	-1.26323	-2.18689	2.09819
H	-2.21651	-0.79897	2.67894
H	-1.03938	-0.58127	1.36173

ACE4 Rb B3LYP def2sv

47

O	0.43102	-2.13069	0.92343
O	1.95884	1.82479	0.09125
O	-2.06956	-2.11819	-0.45481
O	-0.81703	2.45266	0.47377
O	-2.89850	0.63017	-0.23131
N	2.82542	-0.98707	-0.37349
C	2.79571	-1.70568	0.90869
C	3.68106	0.19813	-0.37450
C	1.48764	-1.56824	1.68173
C	3.21419	1.33409	0.52493

C	-0.79452	-2.24142	1.61985
C	1.50357	2.93891	0.83385
C	-1.82902	-2.89023	0.71554
C	0.18371	3.43412	0.28163
C	-3.39722	-1.67059	-0.67793
C	-2.11614	2.87465	0.10378
C	-3.76212	-0.43143	0.12429
C	-3.12681	1.81913	0.49955
H	3.61964	-1.37160	1.57037
H	2.96676	-2.78160	0.72947
H	3.72893	0.58319	-1.40792
H	4.72703	-0.05122	-0.07654
H	1.58727	-2.10138	2.64992
H	1.26726	-0.50438	1.90715
H	3.15083	1.01054	1.58406
H	3.97286	2.14230	0.48056
H	-1.14437	-1.24121	1.95128
H	-0.66989	-2.87086	2.52577
H	2.23936	3.76729	0.77736
H	1.38191	2.66582	1.90242
H	-1.46299	-3.88658	0.40400
H	-2.75972	-3.03690	1.29315
H	-0.08750	4.36959	0.81170
H	0.28962	3.67517	-0.79689
H	-4.12684	-2.47299	-0.45561
H	-3.46520	-1.44151	-1.75516
H	-2.16692	3.06298	-0.98923
H	-2.37717	3.81988	0.62144
H	-4.81848	-0.16556	-0.09019
H	-3.68779	-0.63553	1.21270

H	-3.04833	1.62048	1.58817
H	-4.14552	2.20925	0.29802
C	3.11828	-1.85967	-1.50636
H	2.40180	-2.69798	-1.53371
H	4.14775	-2.28712	-1.47493
H	3.01711	-1.29737	-2.45161
Rb	-0.07848	0.00449	-1.17541

ACE4 Rb B3LYP def2tzv

47

O	0.48307	-2.25468	0.88430
O	1.96645	1.89251	0.04355
O	-2.05166	-2.13630	-0.50014
O	-0.84525	2.49369	0.50421
O	-2.93961	0.64107	-0.25747
N	2.84058	-0.96209	-0.38677
C	2.85244	-1.72717	0.88597
C	3.70607	0.23612	-0.38038
C	1.55321	-1.65104	1.67819
C	3.23558	1.35716	0.53341
C	-0.78356	-2.37348	1.59920
C	1.49017	3.02560	0.83090
C	-1.80571	-2.99104	0.66619
C	0.17577	3.51090	0.26857
C	-3.42633	-1.68300	-0.70416
C	-2.18716	2.91234	0.10795
C	-3.79184	-0.47547	0.13743
C	-3.17296	1.84892	0.52828
H	3.66266	-1.37952	1.53787
H	3.05764	-2.77871	0.67307
H	3.75070	0.62293	-1.39896

H	4.73867	-0.01804	-0.08557
H	1.66719	-2.20155	2.61798
H	1.29067	-0.61538	1.91393
H	3.11265	1.01537	1.56547
H	3.98888	2.15142	0.53109
H	-1.11346	-1.38834	1.94236
H	-0.65803	-3.02092	2.47354
H	2.22053	3.83966	0.78636
H	1.36471	2.72501	1.87512
H	-1.43797	-3.94850	0.29607
H	-2.73299	-3.16372	1.21658
H	-0.10527	4.44032	0.77296
H	0.26620	3.70840	-0.80352
H	-4.12834	-2.49469	-0.49698
H	-3.49100	-1.43032	-1.76077
H	-2.22584	3.06774	-0.97415
H	-2.44395	3.85238	0.60562
H	-4.84313	-0.22365	-0.03748
H	-3.66223	-0.67990	1.20474
H	-3.05428	1.62612	1.59244
H	-4.19090	2.21180	0.35699
C	3.10059	-1.81504	-1.56546
H	2.38602	-2.63589	-1.59003
H	4.11599	-2.24180	-1.56692
H	2.97915	-1.23040	-2.47750
Rb	-0.08054	0.02124	-1.10744

ACE4 Rb B97-1 def2sv

47

O	0.38094	-2.10895	0.95466
O	1.99611	1.79286	0.07046

O	-2.10073	-2.10661	-0.46319
O	-0.76484	2.45565	0.48530
O	-2.86939	0.66101	-0.22637
N	2.78927	-1.03866	-0.35548
C	2.74878	-1.72106	0.94715
C	3.67695	0.12309	-0.39208
C	1.43525	-1.54086	1.70768
C	3.24625	1.28693	0.49449
C	-0.85498	-2.17032	1.63413
C	1.56658	2.91332	0.81499
C	-1.88455	-2.84035	0.73374
C	0.24308	3.42367	0.27714
C	-3.41315	-1.62672	-0.69394
C	-2.05754	2.89376	0.11928
C	-3.75977	-0.38103	0.11282
C	-3.08205	1.84615	0.51181
H	3.57576	-1.37811	1.60275
H	2.90539	-2.80585	0.79938
H	3.71871	0.48778	-1.43518
H	4.72185	-0.14654	-0.10241
H	1.51827	-2.04775	2.69347
H	1.23092	-0.46484	1.89986
H	3.19195	0.98110	1.56153
H	4.02210	2.07938	0.42496
H	-1.19552	-1.15014	1.91868
H	-0.75709	-2.76548	2.56819
H	2.31221	3.73422	0.74649
H	1.45415	2.64455	1.88758
H	-1.51956	-3.84937	0.45905
H	-2.82631	-2.96388	1.30242

H	-0.00965	4.36586	0.80805
H	0.33890	3.65991	-0.80527
H	-4.16532	-2.41366	-0.48193
H	-3.46556	-1.39011	-1.77197
H	-2.10844	3.08699	-0.97470
H	-2.30863	3.84100	0.64194
H	-4.80899	-0.09088	-0.11323
H	-3.70346	-0.59337	1.20274
H	-3.00370	1.63989	1.60093
H	-4.09729	2.25149	0.31395
C	3.05394	-1.95091	-1.46477
H	2.31195	-2.76945	-1.46503
H	4.07247	-2.40639	-1.42469
H	2.96381	-1.41065	-2.42578
Rb	-0.06874	0.00160	-1.19797

ACE4 Rb B97-1 def2tzv

47

O	0.41128	-2.22281	0.92796
O	2.01842	1.84561	0.01846
O	-2.10092	-2.12281	-0.51032
O	-0.77412	2.49564	0.51663
O	-2.90152	0.68571	-0.25711
N	2.79604	-1.03204	-0.36799
C	2.78850	-1.74887	0.93384
C	3.70453	0.13471	-0.40434
C	1.48080	-1.61000	1.71064
C	3.28185	1.29171	0.49388
C	-0.86811	-2.26779	1.62284
C	1.57630	2.98447	0.81288
C	-1.88769	-2.91733	0.70125

C	0.25882	3.49446	0.26874
C	-3.45532	-1.62254	-0.72157
C	-2.10504	2.94111	0.12131
C	-3.79223	-0.40261	0.12202
C	-3.11413	1.89106	0.53436
H	3.60348	-1.39254	1.57879
H	2.97063	-2.81507	0.76301
H	3.74449	0.49722	-1.43500
H	4.73461	-0.14751	-0.11734
H	1.57204	-2.12306	2.67686
H	1.24118	-0.55558	1.89871
H	3.17311	0.97383	1.53835
H	4.05804	2.06657	0.45790
H	-1.18236	-1.25372	1.90091
H	-0.77720	-2.86721	2.53800
H	2.32048	3.78887	0.75823
H	1.45945	2.68489	1.86138
H	-1.52495	-3.89897	0.38664
H	-2.82813	-3.05127	1.24555
H	0.00177	4.43034	0.77967
H	0.33930	3.69266	-0.80686
H	-4.18852	-2.41177	-0.52067
H	-3.50318	-1.36407	-1.78021
H	-2.14051	3.10309	-0.96276
H	-2.34778	3.88477	0.62500
H	-4.83568	-0.11848	-0.06604
H	-3.68402	-0.61605	1.19303
H	-3.00158	1.66011	1.60035
H	-4.12633	2.27676	0.36240
C	3.03508	-1.93864	-1.51159

H	2.29404	-2.73955	-1.50571
H	4.03955	-2.39540	-1.49218
H	2.93262	-1.38440	-2.44763
Rb	-0.06924	0.01272	-1.13851

ACE4 Rb CAM-B3LYP def2sv

47

O	0.39932	-2.08936	0.93604
O	1.96563	1.78526	0.06713
O	-2.05907	-2.08902	-0.46447
O	-0.77739	2.43615	0.46737
O	-2.85544	0.64133	-0.23231
N	2.77788	-1.01691	-0.36808
C	2.75219	-1.69650	0.92685
C	3.65227	0.14483	-0.41147
C	1.44957	-1.52757	1.68957
C	3.22080	1.29459	0.47704
C	-0.82673	-2.18211	1.61856
C	1.53742	2.90413	0.80460
C	-1.84437	-2.84221	0.71382
C	0.22165	3.40712	0.26684
C	-3.37361	-1.63564	-0.70621
C	-2.06946	2.86848	0.11319
C	-3.73471	-0.40547	0.09931
C	-3.07843	1.81875	0.50466
H	3.57986	-1.34898	1.57335
H	2.91515	-2.77685	0.77748
H	3.68180	0.51258	-1.45041
H	4.69774	-0.11951	-0.13364
H	1.53332	-2.04103	2.66771
H	1.24272	-0.45710	1.88948

H	3.17782	0.98652	1.54020
H	3.98233	2.09548	0.40403
H	-1.17847	-1.17566	1.92358
H	-0.71409	-2.79173	2.53714
H	2.28223	3.72073	0.73335
H	1.42530	2.63883	1.87444
H	-1.47446	-3.84218	0.42595
H	-2.78551	-2.97808	1.27434
H	-0.04017	4.34302	0.79707
H	0.31961	3.64467	-0.81148
H	-4.10954	-2.43456	-0.50089
H	-3.42554	-1.39651	-1.78042
H	-2.12704	3.06460	-0.97635
H	-2.31838	3.80991	0.63984
H	-4.78218	-0.12248	-0.12600
H	-3.67672	-0.62049	1.18501
H	-2.99517	1.61130	1.58960
H	-4.09562	2.21093	0.30989
C	3.03115	-1.92112	-1.47690
H	2.29508	-2.74081	-1.47194
H	4.04864	-2.37090	-1.44618
H	2.93051	-1.38202	-2.43392
Rb	-0.07690	0.00649	-1.14443

ACE4 Rb CAM-B3LYP def2tzv

47

O	0.43302	-2.22368	0.90193
O	1.99445	1.83998	0.01675
O	-2.05875	-2.09482	-0.51143
O	-0.77946	2.48562	0.49965
O	-2.88203	0.67393	-0.25256

N	2.77574	-1.01156	-0.38126
C	2.79110	-1.73569	0.90303
C	3.67198	0.15058	-0.43022
C	1.49781	-1.62588	1.68609
C	3.25753	1.29191	0.47343
C	-0.83566	-2.30280	1.59511
C	1.55372	2.97382	0.80306
C	-1.84635	-2.92550	0.66437
C	0.24760	3.47913	0.25756
C	-3.41002	-1.61720	-0.73704
C	-2.10765	2.92015	0.11552
C	-3.76071	-0.41820	0.11045
C	-3.10004	1.87153	0.53280
H	3.60397	-1.37266	1.53933
H	2.99045	-2.79331	0.72134
H	3.69334	0.51757	-1.45561
H	4.70360	-0.12893	-0.16444
H	1.59523	-2.15630	2.63738
H	1.25257	-0.58146	1.89668
H	3.15824	0.96729	1.51230
H	4.02461	2.06985	0.43805
H	-1.15559	-1.30388	1.90293
H	-0.73390	-2.92453	2.48874
H	2.29824	3.77228	0.75034
H	1.43389	2.67567	1.84722
H	-1.48364	-3.89327	0.32202
H	-2.78444	-3.07593	1.20044
H	-0.01490	4.41164	0.76287
H	0.33198	3.67412	-0.81408
H	-4.13002	-2.41670	-0.55267

H	-3.45209	-1.34942	-1.78951
H	-2.15012	3.07835	-0.96464
H	-2.34834	3.86106	0.61619
H	-4.80114	-0.13901	-0.07610
H	-3.65293	-0.63974	1.17556
H	-2.97991	1.64305	1.59418
H	-4.11347	2.24441	0.36644
C	2.95314	-1.89662	-1.53916
H	2.20470	-2.68499	-1.51632
H	3.94717	-2.36561	-1.56382
H	2.82443	-1.33142	-2.46103
Rb	-0.07634	0.02387	-1.06999

ACE4 Rb MP2 def2tzv

47

O	-0.23182	-2.13967	-0.95595
O	-2.14771	1.69845	0.08172
O	2.19250	-2.02971	0.58943
O	0.56156	2.50317	-0.51176
O	2.77373	0.80735	0.22028
N	-2.64890	-1.20449	0.31690
C	-2.62140	-1.82196	-1.03874
C	-3.68740	-0.15376	0.45213
C	-1.30904	-1.53725	-1.76198
C	-3.39337	1.07693	-0.39851
C	1.08299	-2.08640	-1.61318
C	-1.81607	2.91331	-0.67898
C	2.07693	-2.76769	-0.68643
C	-0.50341	3.45563	-0.15554
C	3.51271	-1.41616	0.82787
C	1.90143	3.02211	-0.19146

C	3.78356	-0.21995	-0.07251
C	2.92095	1.99549	-0.63567
H	-3.44615	-1.43704	-1.65664
H	-2.75629	-2.90917	-0.96184
H	-3.72889	0.14586	1.50450
H	-4.68782	-0.53301	0.16490
H	-1.31074	-1.98321	-2.76581
H	-1.14144	-0.45467	-1.84796
H	-3.28179	0.82084	-1.45973
H	-4.21949	1.79347	-0.29918
H	1.36661	-1.04080	-1.79204
H	1.04223	-2.61884	-2.57274
H	-2.60519	3.66314	-0.53934
H	-1.72517	2.67166	-1.74531
H	1.73576	-3.77824	-0.44973
H	3.05559	-2.82563	-1.17834
H	-0.29778	4.42589	-0.62482
H	-0.54622	3.57907	0.93363
H	4.30089	-2.16504	0.68113
H	3.49470	-1.10988	1.87472
H	1.98610	3.20069	0.88743
H	2.06903	3.96227	-0.73184
H	4.78736	0.17172	0.13868
H	3.72815	-0.49264	-1.13386
H	2.75511	1.72359	-1.68523
H	3.93047	2.40902	-0.51879
C	-2.75233	-2.21733	1.39947
H	-1.94218	-2.94042	1.28506
H	-3.71624	-2.75575	1.38127
H	-2.65049	-1.72070	2.36893

Rb	0.05370	0.01229	1.09341

ACE4 Rb PBE0 def2sv

47

O	0.37819	-2.09408	0.94425
O	1.98116	1.77672	0.06405
O	-2.08307	-2.08632	-0.46348
O	-0.75676	2.43763	0.48024
O	-2.84647	0.65782	-0.22785
N	2.76785	-1.03336	-0.35312
C	2.72964	-1.70485	0.94280
C	3.65032	0.11843	-0.39680
C	1.42406	-1.52923	1.69575
C	3.22575	1.27596	0.48227
C	-0.84988	-2.15717	1.62091
C	1.56022	2.89499	0.80089
C	-1.87379	-2.81788	0.72561
C	0.24362	3.40113	0.27151
C	-3.38835	-1.61286	-0.69624
C	-2.04247	2.87592	0.12241
C	-3.73362	-0.37739	0.10618
C	-3.05966	1.83392	0.50860
H	3.55446	-1.35746	1.59564
H	2.89285	-2.78778	0.80074
H	3.68872	0.48077	-1.43904
H	4.69502	-0.15066	-0.11336
H	1.50501	-2.03443	2.68033
H	1.21915	-0.45548	1.88928
H	3.17634	0.97273	1.54856
H	3.99971	2.06725	0.41036
H	-1.18918	-1.14000	1.90988

H	-0.75192	-2.75345	2.55184
H	2.30563	3.71282	0.72622
H	1.45325	2.63286	1.87368
H	-1.51441	-3.82767	0.45351
H	-2.81544	-2.93839	1.29163
H	-0.00771	4.34182	0.80162
H	0.33627	3.63882	-0.80889
H	-4.13851	-2.40027	-0.48880
H	-3.44134	-1.37526	-1.77216
H	-2.09689	3.07535	-0.96835
H	-2.29144	3.81950	0.64849
H	-4.78077	-0.08666	-0.11853
H	-3.67947	-0.59165	1.19399
H	-2.98229	1.62577	1.59550
H	-4.07350	2.23843	0.31323
C	3.01952	-1.94329	-1.45303
H	2.27428	-2.75623	-1.44994
H	4.03315	-2.40494	-1.41683
H	2.92971	-1.40764	-2.41447
Rb	-0.06735	0.00041	-1.17797

ACE4 Rb PBE0 def2tzv

47

O	0.40326	-2.20617	0.92169
O	2.00689	1.82599	0.00954
O	-2.08666	-2.10441	-0.51216
O	-0.76017	2.47784	0.51276
O	-2.87317	0.68510	-0.25620
N	2.77037	-1.03313	-0.36357
C	2.76269	-1.73332	0.93310
C	3.67402	0.12143	-0.41140

C	1.46262	-1.59713	1.69985
C	3.26113	1.27425	0.47690
C	-0.86828	-2.24400	1.60811
C	1.57609	2.96069	0.79545
C	-1.88272	-2.88690	0.69488
C	0.26482	3.46809	0.26412
C	-3.42826	-1.60334	-0.72645
C	-2.08172	2.92314	0.12784
C	-3.76098	-0.39384	0.11325
C	-3.08443	1.87914	0.53198
H	3.57400	-1.36873	1.57623
H	2.95358	-2.79891	0.77259
H	3.71095	0.47887	-1.44305
H	4.70450	-0.16129	-0.13034
H	1.55193	-2.10574	2.66761
H	1.22426	-0.54315	1.88707
H	3.15796	0.95917	1.52213
H	4.03970	2.04530	0.43815
H	-1.17996	-1.22914	1.88269
H	-0.78357	-2.83819	2.52616
H	2.32023	3.76339	0.73401
H	1.46669	2.66823	1.84591
H	-1.52803	-3.87396	0.39090
H	-2.82377	-3.01156	1.23876
H	0.01298	4.40376	0.77614
H	0.34080	3.67025	-0.81027
H	-4.16554	-2.38915	-0.53178
H	-3.47499	-1.34193	-1.78355
H	-2.12193	3.09403	-0.95391
H	-2.32363	3.86326	0.63657

H	-4.80309	-0.10801	-0.07389
H	-3.65772	-0.61233	1.18300
H	-2.97457	1.64654	1.59708
H	-4.09532	2.26675	0.36239
C	2.99166	-1.94087	-1.49352
H	2.24566	-2.73561	-1.47994
H	3.99105	-2.40637	-1.47787
H	2.89000	-1.39472	-2.43327
Rb	-0.06718	0.01352	-1.12065

ACE4 Rb PBE def2sv

47

O	0.37738	-2.12147	0.93955
O	2.00448	1.79726	0.05918
O	-2.11637	-2.11369	-0.47686
O	-0.75917	2.45842	0.48554
O	-2.87467	0.67311	-0.23700
N	2.79752	-1.04126	-0.35524
C	2.74884	-1.72859	0.94608
C	3.68844	0.11979	-0.38027
C	1.43368	-1.55343	1.70338
C	3.25291	1.28350	0.50166
C	-0.86283	-2.18388	1.62665
C	1.57491	2.92235	0.81005
C	-1.89390	-2.85009	0.72679
C	0.25195	3.43291	0.27502
C	-3.43587	-1.61986	-0.68380
C	-2.05594	2.90625	0.11822
C	-3.76388	-0.37166	0.12466
C	-3.08308	1.86260	0.50901
H	3.57798	-1.38737	1.61256

H	2.90958	-2.81939	0.79402
H	3.74446	0.48620	-1.42949
H	4.73798	-0.15226	-0.07809
H	1.51513	-2.06797	2.69402
H	1.22642	-0.47149	1.90392
H	3.18351	0.97438	1.57529
H	4.03950	2.07727	0.44540
H	-1.20342	-1.15808	1.92076
H	-0.76286	-2.78695	2.56490
H	2.32532	3.74971	0.74376
H	1.46377	2.65070	1.89024
H	-1.52554	-3.86471	0.44659
H	-2.83960	-2.97851	1.30170
H	-0.00085	4.38183	0.81019
H	0.34579	3.67396	-0.81465
H	-4.19656	-2.40542	-0.45775
H	-3.50347	-1.38455	-1.76872
H	-2.10686	3.10556	-0.98275
H	-2.30602	3.86025	0.64558
H	-4.82530	-0.08044	-0.08159
H	-3.68805	-0.58193	1.22190
H	-3.00391	1.65143	1.60518
H	-4.10434	2.27634	0.31560
C	3.06918	-1.94956	-1.46702
H	2.32280	-2.77290	-1.47714
H	4.09401	-2.41017	-1.42517
H	2.98495	-1.40252	-2.43246
Rb	-0.06991	-0.00122	-1.19143

ACE4 Rb PBE def2tzv

47

O	0.40650	-2.24132	0.91613
O	2.02702	1.84892	0.00275
O	-2.11849	-2.13168	-0.52233
O	-0.76624	2.49393	0.52806
O	-2.90368	0.69793	-0.27161
N	2.80194	-1.03762	-0.36358
C	2.78609	-1.75528	0.94056
C	3.71545	0.12796	-0.39187
C	1.47699	-1.61992	1.71090
C	3.29075	1.28632	0.49899
C	-0.88063	-2.27072	1.62075
C	1.58797	2.99372	0.80971
C	-1.90421	-2.91933	0.70758
C	0.26989	3.50365	0.27504
C	-3.48224	-1.61520	-0.70980
C	-2.10194	2.95375	0.12620
C	-3.79923	-0.39177	0.13120
C	-3.11556	1.90906	0.53094
H	3.60250	-1.39630	1.59516
H	2.97523	-2.82762	0.76936
H	3.76912	0.48890	-1.42987
H	4.75021	-0.15772	-0.09295
H	1.56541	-2.13549	2.68462
H	1.23128	-0.55927	1.89933
H	3.16649	0.96846	1.54994
H	4.07709	2.06202	0.47268
H	-1.18704	-1.24553	1.89577
H	-0.79127	-2.86808	2.54682
H	2.33702	3.80394	0.75390
H	1.47829	2.68905	1.86549

H	-1.54629	-3.91225	0.39662
H	-2.85035	-3.04554	1.25799
H	0.01318	4.44419	0.79391
H	0.34276	3.70863	-0.80762
H	-4.22387	-2.40288	-0.49358
H	-3.54308	-1.36183	-1.77647
H	-2.13187	3.12456	-0.96442
H	-2.34353	3.90296	0.63663
H	-4.85225	-0.10312	-0.04234
H	-3.67544	-0.59983	1.20935
H	-3.00836	1.67033	1.60359
H	-4.13249	2.30334	0.35767
C	3.05197	-1.94618	-1.50513
H	2.30635	-2.75162	-1.50658
H	4.06258	-2.40802	-1.47819
H	2.95833	-1.38982	-2.44845
Rb	-0.06747	0.01036	-1.14588

ACE4 Rb B2PLYP def2sv

47

O	-0.37614	-2.07330	-0.95455
O	-1.98191	1.76860	-0.06127
O	2.07742	-2.09390	0.46227
O	0.75807	2.43230	-0.47668
O	2.84857	0.64962	0.23615
N	-2.76725	-1.03898	0.36072
C	-2.73442	-1.70188	-0.94826
C	-3.66060	0.11317	0.41181
C	-1.43087	-1.49813	-1.70450
C	-3.24194	1.27331	-0.47286
C	0.85952	-2.12843	-1.63783

C	-1.56355	2.89612	-0.80394
C	1.87581	-2.81384	-0.74712
C	-0.24726	3.40463	-0.26734
C	3.39313	-1.62640	0.70821
C	2.05119	2.87640	-0.11433
C	3.74723	-0.38976	-0.09462
C	3.06883	1.83240	-0.50584
H	-3.56845	-1.35876	-1.59002
H	-2.87753	-2.78722	-0.81467
H	-3.69315	0.47472	1.45305
H	-4.70346	-0.16237	0.13203
H	-1.50479	-1.98800	-2.69566
H	-1.23374	-0.42110	-1.87403
H	-3.20170	0.97378	-1.53821
H	-4.00788	2.06913	-0.38909
H	1.20411	-1.10839	-1.90198
H	0.75734	-2.70539	-2.57865
H	-2.31286	3.70823	-0.72767
H	-1.45358	2.63270	-1.87419
H	1.50881	-3.82332	-0.49190
H	2.82225	-2.92758	-1.30370
H	0.00953	4.34317	-0.79554
H	-0.34255	3.63726	0.81204
H	4.13707	-2.41838	0.50341
H	3.43621	-1.38942	1.78294
H	2.10336	3.07007	0.97568
H	2.29529	3.82049	-0.63864
H	4.78960	-0.09512	0.13990
H	3.69947	-0.60178	-1.18098
H	2.98881	1.62373	-1.59051

H	4.08314	2.23059	-0.30807
C	-3.03654	-1.96765	1.45179
H	-2.29519	-2.78214	1.44144
H	-4.05120	-2.42127	1.39569
H	-2.95307	-1.44343	2.41835
Rb	0.07145	0.00098	1.17340

ACE4 Rb B2PLYP def2tzv

47

O	0.38829	-2.20063	0.91813
O	2.02909	1.81855	0.00073
O	-2.09939	-2.10210	-0.52904
O	-0.74132	2.49467	0.50216
O	-2.87329	0.69990	-0.25027
N	2.76459	-1.05523	-0.36812
C	2.76595	-1.75748	0.94150
C	3.69464	0.09358	-0.42564
C	1.46266	-1.59791	1.71260
C	3.29617	1.25444	0.47193
C	-0.89642	-2.25283	1.61432
C	1.60787	2.97710	0.78814
C	-1.90351	-2.90386	0.68734
C	0.29806	3.49157	0.24132
C	-3.45543	-1.59091	-0.74726
C	-2.07950	2.95122	0.12433
C	-3.78346	-0.38527	0.11251
C	-3.08113	1.90579	0.55164
H	3.58197	-1.39127	1.57525
H	2.94441	-2.82416	0.78461
H	3.72599	0.44879	-1.45631
H	4.71954	-0.20638	-0.14797

H	1.53446	-2.10903	2.67796
H	1.23596	-0.54235	1.89031
H	3.18773	0.94244	1.51452
H	4.07362	2.02315	0.42651
H	-1.21369	-1.24206	1.88733
H	-0.80108	-2.85070	2.52625
H	2.36310	3.76543	0.71676
H	1.49157	2.68965	1.83662
H	-1.54244	-3.88354	0.37640
H	-2.85113	-3.02908	1.21587
H	0.04253	4.42847	0.74438
H	0.37659	3.67536	-0.83356
H	-4.18811	-2.37977	-0.55922
H	-3.49118	-1.32016	-1.79994
H	-2.12799	3.11056	-0.95619
H	-2.30407	3.89314	0.63288
H	-4.81829	-0.08198	-0.07460
H	-3.67687	-0.61256	1.17721
H	-2.95281	1.67079	1.61151
H	-4.09383	2.28576	0.39017
C	2.97204	-1.97862	-1.50583
H	2.22354	-2.76783	-1.47201
H	3.96994	-2.44327	-1.49620
H	2.85841	-1.43400	-2.44309
Rb	-0.07121	0.01749	-1.10010

ACE4 Rb DSDPBEP86 def2sv

47

O	0.30282	-1.98746	1.01599
O	2.02580	1.69300	0.00479
O	-2.09643	-2.07329	-0.45704

O	-0.67766	2.40575	0.47536
O	-2.78401	0.67282	-0.25974
N	2.69181	-1.11530	-0.34955
C	2.65437	-1.69115	0.99661
C	3.63412	-0.01414	-0.48242
C	1.36187	-1.38167	1.73047
C	3.29195	1.18794	0.37488
C	-0.94843	-1.91923	1.66427
C	1.65215	2.82416	0.76117
C	-1.95133	-2.67596	0.82024
C	0.33924	3.35943	0.24918
C	-3.38686	-1.57612	-0.75761
C	-1.95554	2.87734	0.10180
C	-3.73810	-0.32707	0.02409
C	-2.99556	1.85476	0.48221
H	3.51032	-1.34135	1.60802
H	2.75017	-2.79028	0.93353
H	3.63918	0.30853	-1.53935
H	4.67611	-0.32612	-0.23104
H	1.40950	-1.78662	2.76283
H	1.19939	-0.28557	1.80012
H	3.29533	0.92781	1.45331
H	4.07122	1.96348	0.22496
H	-1.26787	-0.86238	1.78789
H	-0.89164	-2.37989	2.67256
H	2.41984	3.62021	0.67385
H	1.55561	2.55600	1.83370
H	-1.59169	-3.71144	0.67683
H	-2.91967	-2.72319	1.35203
H	0.10689	4.30448	0.78102

H	0.42174	3.58807	-0.83403
H	-4.16000	-2.34932	-0.57763
H	-3.37947	-1.34488	-1.83614
H	-1.99344	3.06944	-0.99102
H	-2.18415	3.82887	0.62364
H	-4.75609	0.00717	-0.26576
H	-3.75569	-0.53621	1.11368
H	-2.93145	1.64206	1.56918
H	-4.00195	2.27071	0.27156
C	2.89727	-2.11875	-1.38284
H	2.11225	-2.89111	-1.31253
H	3.88966	-2.62062	-1.31014
H	2.82567	-1.64765	-2.37975
Rb	-0.05912	-0.02073	-1.13897

ACE4 Rb DSDPBEP86 def2tzv

47

O	-0.25017	-2.14416	-0.96742
O	-2.13177	1.70312	0.07969
O	2.16866	-2.02972	0.57304
O	0.57625	2.49345	-0.51464
O	2.77286	0.79606	0.24334
N	-2.64167	-1.18812	0.33583
C	-2.63004	-1.80580	-1.01250
C	-3.66384	-0.13483	0.49166
C	-1.32702	-1.54200	-1.75359
C	-3.38415	1.09230	-0.36187
C	1.05539	-2.07753	-1.61664
C	-1.79525	2.88895	-0.70265
C	2.05127	-2.75687	-0.69647
C	-0.48638	3.44465	-0.19440

C	3.48082	-1.43345	0.82612
C	1.90037	3.00406	-0.16592
C	3.76673	-0.23013	-0.05328
C	2.93119	1.98539	-0.58970
H	-3.45848	-1.42141	-1.62277
H	-2.77375	-2.89020	-0.92883
H	-3.68081	0.16618	1.54356
H	-4.67387	-0.50532	0.23282
H	-1.35260	-1.99417	-2.75364
H	-1.14908	-0.46276	-1.85576
H	-3.30545	0.83501	-1.42572
H	-4.20425	1.81191	-0.24349
H	1.33431	-1.02988	-1.79156
H	1.02243	-2.60201	-2.58021
H	-2.58181	3.64528	-0.58794
H	-1.70579	2.62197	-1.76283
H	1.70845	-3.76747	-0.46322
H	3.02640	-2.81947	-1.19222
H	-0.28341	4.40351	-0.68679
H	-0.53205	3.60127	0.89023
H	4.26755	-2.18322	0.68106
H	3.46076	-1.13623	1.87534
H	1.96021	3.17983	0.91502
H	2.08285	3.94821	-0.69360
H	4.77326	0.14743	0.16787
H	3.72029	-0.49020	-1.11832
H	2.79397	1.72145	-1.64522
H	3.93552	2.40368	-0.45047
C	-2.71317	-2.19083	1.41823
H	-1.89504	-2.90398	1.29994

H	-3.66732	-2.74587	1.41874
H	-2.60536	-1.69209	2.38568
Rb	0.05602	0.01249	1.05934

ACE4 Rb HSE06 def2sv

47

O	0.38034	-2.09517	0.94149
O	1.98065	1.77730	0.06035
O	-2.07816	-2.08508	-0.46760
O	-0.75641	2.43774	0.47741
O	-2.84511	0.65756	-0.22876
N	2.76566	-1.03275	-0.35612
C	2.73158	-1.70487	0.93983
C	3.64895	0.11847	-0.40214
C	1.42681	-1.53229	1.69481
C	3.22594	1.27576	0.47784
C	-0.84806	-2.16263	1.61830
C	1.56044	2.89645	0.79744
C	-1.87059	-2.82112	0.71995
C	0.24369	3.40246	0.26851
C	-3.38441	-1.61273	-0.70187
C	-2.04393	2.87630	0.12379
C	-3.73148	-0.37978	0.10335
C	-3.05889	1.83248	0.51060
H	3.55664	-1.35531	1.59121
H	2.89740	-2.78739	0.79726
H	3.68556	0.48070	-1.44448
H	4.69405	-0.15134	-0.12065
H	1.50819	-2.04083	2.67757
H	1.22159	-0.45925	1.89201
H	3.17663	0.97170	1.54394

H	3.99973	2.06716	0.40634
H	-1.18868	-1.14699	1.91103
H	-0.74914	-2.76207	2.54703
H	2.30635	3.71370	0.72211
H	1.45406	2.63438	1.87027
H	-1.51078	-3.82992	0.44510
H	-2.81324	-2.94316	1.28389
H	-0.00839	4.34266	0.79896
H	0.33564	3.64039	-0.81184
H	-4.13352	-2.40180	-0.49746
H	-3.43585	-1.37249	-1.77719
H	-2.10121	3.07785	-0.96636
H	-2.29161	3.81866	0.65249
H	-4.77891	-0.08932	-0.12011
H	-3.67643	-0.59645	1.19065
H	-2.97890	1.62220	1.59688
H	-4.07385	2.23534	0.31807
C	3.01195	-1.94248	-1.45758
H	2.26573	-2.75441	-1.45215
H	4.02512	-2.40545	-1.42509
H	2.91964	-1.40620	-2.41834
Rb	-0.06995	0.00303	-1.16590

ACE4 Rb HSE06 def2tzv

47

O	0.40046	-2.21102	0.91846
O	2.01302	1.82386	0.00279
O	-2.08730	-2.09819	-0.51806
O	-0.75181	2.48073	0.51112
O	-2.86833	0.69159	-0.25510
N	2.76350	-1.03835	-0.36453

C	2.76009	-1.73800	0.93260
C	3.67240	0.11180	-0.41943
C	1.46052	-1.60494	1.70008
C	3.26727	1.26745	0.46826
C	-0.87293	-2.25028	1.60365
C	1.58594	2.96052	0.78995
C	-1.88640	-2.88787	0.68633
C	0.27512	3.47026	0.26079
C	-3.42897	-1.59402	-0.73282
C	-2.07498	2.92883	0.13077
C	-3.75936	-0.38735	0.11108
C	-3.07721	1.88540	0.53616
H	3.57034	-1.36947	1.57411
H	2.95531	-2.80278	0.77305
H	3.70624	0.46682	-1.45170
H	4.70269	-0.17459	-0.14252
H	1.54869	-2.11713	2.66563
H	1.22231	-0.55187	1.89077
H	3.16462	0.95409	1.51376
H	4.04802	2.03561	0.42702
H	-1.18330	-1.23626	1.88147
H	-0.78953	-2.84771	2.51926
H	2.33195	3.76086	0.72698
H	1.47804	2.66807	1.84019
H	-1.53273	-3.87340	0.37762
H	-2.82859	-3.01367	1.22725
H	0.02496	4.40574	0.77319
H	0.34914	3.67211	-0.81341
H	-4.16700	-2.37950	-0.54181
H	-3.47361	-1.32909	-1.78875

H	-2.11775	3.10150	-0.95021
H	-2.31331	3.86822	0.64174
H	-4.80031	-0.09779	-0.07459
H	-3.65596	-0.60961	1.17970
H	-2.96495	1.65058	1.60014
H	-4.08814	2.27312	0.36948
C	2.96747	-1.94781	-1.49643
H	2.21537	-2.73611	-1.47578
H	3.96285	-2.42140	-1.48932
H	2.86228	-1.40132	-2.43523
Rb	-0.06903	0.01604	-1.10719

ACE4 Rb M062X def2sv

47

O	0.22699	-1.94476	1.01119
O	2.07458	1.63511	-0.03080
O	-2.13873	-2.03345	-0.51064
O	-0.59097	2.41379	0.46146
O	-2.72911	0.71511	-0.20954
N	2.65732	-1.19063	-0.30711
C	2.57939	-1.70599	1.05938
C	3.63309	-0.12675	-0.47539
C	1.27183	-1.34283	1.74033
C	3.32429	1.10799	0.34676
C	-1.03517	-1.88345	1.62850
C	1.74633	2.80850	0.67184
C	-2.00860	-2.64781	0.75663
C	0.42790	3.34146	0.17191
C	-3.40669	-1.48584	-0.79694
C	-1.87529	2.90309	0.15602
C	-3.71573	-0.25747	0.03340

C	-2.90700	1.88103	0.55818
H	3.42290	-1.34151	1.67467
H	2.65826	-2.80663	1.04609
H	3.64608	0.16208	-1.54039
H	4.66175	-0.46346	-0.21307
H	1.27190	-1.71187	2.78438
H	1.13579	-0.24055	1.76438
H	3.32040	0.87531	1.43069
H	4.12042	1.85845	0.17736
H	-1.36329	-0.82806	1.74029
H	-0.99666	-2.34048	2.63648
H	2.52586	3.57988	0.52151
H	1.67549	2.59623	1.75720
H	-1.61994	-3.66939	0.60655
H	-2.98633	-2.72614	1.26356
H	0.22086	4.30734	0.67058
H	0.48274	3.52388	-0.92036
H	-4.20183	-2.23942	-0.64624
H	-3.39080	-1.21277	-1.86394
H	-1.95845	3.11408	-0.92914
H	-2.06841	3.84486	0.70423
H	-4.71746	0.12533	-0.24262
H	-3.74149	-0.50830	1.11328
H	-2.80570	1.64699	1.63649
H	-3.91572	2.30375	0.38992
C	2.82391	-2.23753	-1.30052
H	2.01614	-2.97948	-1.19698
H	3.79868	-2.76537	-1.20890
H	2.76412	-1.80473	-2.31344
Rb	-0.06617	-0.01023	-1.12883

ACE4 Rb M062X def2tzv

47

O	0.27321	-2.02556	1.02666
O	2.07953	1.67995	-0.06115
O	-2.11855	-2.07258	-0.52015
O	-0.62303	2.44467	0.48415
O	-2.75707	0.71340	-0.25566
N	2.66587	-1.17512	-0.34969
C	2.63794	-1.72954	1.01657
C	3.64555	-0.09735	-0.53545
C	1.35451	-1.38302	1.74812
C	3.35763	1.11658	0.32314
C	-1.02706	-1.90933	1.65004
C	1.73000	2.84473	0.72364
C	-1.99318	-2.69083	0.78948
C	0.43071	3.39957	0.20720
C	-3.42202	-1.51643	-0.82305
C	-1.93082	2.93309	0.09764
C	-3.74818	-0.30340	0.01790
C	-2.96178	1.92101	0.51606
H	3.49120	-1.37346	1.60213
H	2.71907	-2.81763	0.97187
H	3.62241	0.20427	-1.58325
H	4.66818	-0.43896	-0.30937
H	1.38281	-1.75197	2.77685
H	1.19525	-0.29877	1.76404
H	3.33432	0.85730	1.38575
H	4.14364	1.86145	0.17510
H	-1.32472	-0.85604	1.70840
H	-0.99830	-2.32594	2.65998

H	2.51203	3.60269	0.63165
H	1.62931	2.56407	1.77553
H	-1.60768	-3.69776	0.63968
H	-2.96610	-2.75751	1.27948
H	0.21240	4.34570	0.70820
H	0.49600	3.57522	-0.86978
H	-4.19574	-2.27476	-0.68513
H	-3.38288	-1.23853	-1.87356
H	-1.96416	3.08287	-0.98470
H	-2.13173	3.88583	0.59350
H	-4.74374	0.06309	-0.24543
H	-3.74243	-0.54358	1.08547
H	-2.86420	1.69792	1.58156
H	-3.96203	2.31744	0.32580
C	2.76973	-2.20543	-1.39156
H	1.96644	-2.92890	-1.26614
H	3.72993	-2.73929	-1.35886
H	2.66760	-1.74469	-2.37391
Rb	-0.06591	-0.00652	-1.05666

ACE4 Rb M06 def2sv

47

O	0.20685	-1.91601	1.08690
O	2.12562	1.62257	-0.06336
O	-2.18875	-2.01045	-0.44100
O	-0.55889	2.37777	0.50221
O	-2.75141	0.74370	-0.23597
N	2.64784	-1.21565	-0.25805
C	2.55886	-1.66971	1.12474
C	3.63355	-0.17978	-0.49444
C	1.25295	-1.28542	1.78273

C	3.37928	1.08988	0.27990
C	-1.05727	-1.75493	1.67415
C	1.78741	2.75032	0.69815
C	-2.05610	-2.53932	0.86098
C	0.46894	3.29985	0.23971
C	-3.44491	-1.46330	-0.76443
C	-1.82554	2.89076	0.17629
C	-3.75913	-0.20279	0.00049
C	-2.89044	1.90128	0.54446
H	3.40028	-1.28263	1.73511
H	2.64938	-2.77356	1.16209
H	3.62185	0.06405	-1.57573
H	4.67103	-0.52463	-0.25676
H	1.25201	-1.60333	2.84883
H	1.11066	-0.17884	1.76506
H	3.42116	0.90731	1.37738
H	4.18472	1.82227	0.05304
H	-1.33723	-0.67360	1.70717
H	-1.05048	-2.12853	2.72189
H	2.56310	3.53976	0.59181
H	1.72982	2.48143	1.77755
H	-1.69842	-3.58455	0.77791
H	-3.03116	-2.56560	1.38613
H	0.27364	4.25664	0.77049
H	0.51091	3.52737	-0.85001
H	-4.25543	-2.20376	-0.59762
H	-3.41303	-1.24350	-1.84758
H	-1.87978	3.11910	-0.91270
H	-2.01148	3.84034	0.72326
H	-4.75077	0.18467	-0.32113

H	-3.83507	-0.41048	1.09185
H	-2.81395	1.64545	1.62563
H	-3.88704	2.36555	0.38073
C	2.74670	-2.29688	-1.21306
H	1.91945	-3.01473	-1.06078
H	3.70903	-2.85930	-1.14059
H	2.66703	-1.90411	-2.24527
Rb	-0.04563	-0.05604	-1.24675

ACE4 Rb M06 def2tzv

47

O	0.26176	-2.08696	1.02041
O	2.12151	1.69068	-0.05692
O	-2.15022	-2.02151	-0.49699
O	-0.59057	2.44647	0.53978
O	-2.77882	0.77612	-0.25990
N	2.63999	-1.17555	-0.32251
C	2.62580	-1.77310	1.01983
C	3.63480	-0.12168	-0.51647
C	1.34270	-1.49452	1.77099
C	3.37820	1.10070	0.33220
C	-1.02436	-1.97106	1.65795
C	1.76527	2.82278	0.75926
C	-2.02762	-2.67984	0.78576
C	0.46816	3.38732	0.26302
C	-3.44389	-1.45538	-0.79251
C	-1.88491	2.95341	0.15332
C	-3.75841	-0.23617	0.03545
C	-2.93479	1.95920	0.54806
H	3.46805	-1.40832	1.61840
H	2.75080	-2.85844	0.94147

H	3.60707	0.17675	-1.56793
H	4.65814	-0.47749	-0.30418
H	1.38309	-1.93274	2.77550
H	1.17598	-0.41250	1.87587
H	3.35081	0.84886	1.40008
H	4.18895	1.82427	0.18630
H	-1.29179	-0.91141	1.78099
H	-0.99955	-2.43635	2.65029
H	2.54460	3.59117	0.69921
H	1.66559	2.51116	1.80643
H	-1.68897	-3.70170	0.60396
H	-2.99454	-2.72447	1.29506
H	0.26483	4.33746	0.76960
H	0.52708	3.57610	-0.81586
H	-4.23101	-2.20328	-0.65040
H	-3.40929	-1.18564	-1.84799
H	-1.91174	3.12271	-0.93015
H	-2.07895	3.90728	0.65666
H	-4.76404	0.12267	-0.21299
H	-3.74163	-0.46850	1.10819
H	-2.83286	1.70113	1.60914
H	-3.92815	2.39425	0.39076
C	2.64076	-2.16483	-1.39929
H	1.80051	-2.84839	-1.26819
H	3.56828	-2.75843	-1.43392
H	2.52543	-1.66276	-2.36283
Rb	-0.04557	-0.00794	-1.14033

ACE4 Rb MP2 def2sv

47

O	-0.30640	-1.97045	-1.02130

O	-2.01359	1.69095	-0.01300
O	2.09213	-2.07870	0.44072
O	0.68918	2.39141	-0.47568
O	2.78828	0.65789	0.25870
N	-2.69984	-1.10619	0.34165
C	-2.65510	-1.67319	-1.00743
C	-3.63866	-0.00114	0.46384
C	-1.36254	-1.34899	-1.73092
C	-3.28342	1.19625	-0.39168
C	0.94419	-1.89409	-1.67452
C	-1.63525	2.82382	-0.76852
C	1.94540	-2.66586	-0.84543
C	-0.32364	3.35254	-0.25107
C	3.38897	-1.59293	0.74117
C	1.96840	2.86391	-0.10042
C	3.74342	-0.34175	-0.03252
C	3.00525	1.84130	-0.48299
H	-3.51385	-1.33035	-1.61974
H	-2.73842	-2.77346	-0.95185
H	-3.65321	0.32465	1.51941
H	-4.67927	-0.30963	0.20215
H	-1.40627	-1.73544	-2.77048
H	-1.19957	-0.25287	-1.77801
H	-3.28405	0.93719	-1.46967
H	-4.05741	1.97752	-0.24495
H	1.26705	-0.83729	-1.78056
H	0.88311	-2.33855	-2.68971
H	-2.40048	3.62202	-0.68186
H	-1.53545	2.55775	-1.84067
H	1.58387	-3.70223	-0.71670

H	2.91281	-2.70802	-1.37892
H	-0.08490	4.29666	-0.78134
H	-0.40988	3.58075	0.83141
H	4.15642	-2.36962	0.55305
H	3.38694	-1.36911	1.82079
H	2.00780	3.05436	0.99209
H	2.19776	3.81556	-0.62125
H	4.76172	-0.01114	0.25970
H	3.76090	-0.54402	-1.12262
H	2.93983	1.63161	-1.56989
H	4.01251	2.25422	-0.27155
C	-2.94853	-2.11777	1.35873
H	-2.17441	-2.90075	1.29947
H	-3.94580	-2.60185	1.25325
H	-2.89556	-1.65785	2.36094
Rb	0.05727	-0.02833	1.17894

ACE4 Rb SCS-MP2 def2sv

47

O	-0.33088	-2.00886	-1.00694
O	-2.00372	1.71741	-0.03142
O	2.08679	-2.08606	0.44241
O	0.71565	2.40514	-0.48116
O	2.81016	0.65181	0.25400
N	-2.72220	-1.08316	0.35113
C	-2.68595	-1.68427	-0.98890
C	-3.65099	0.03906	0.45096
C	-1.38859	-1.39475	-1.72712
C	-3.27584	1.22565	-0.41896
C	0.91783	-1.95799	-1.67309
C	-1.61135	2.84920	-0.78759

C	1.92867	-2.70869	-0.82921
C	-0.29651	3.37256	-0.26007
C	3.39261	-1.61019	0.73397
C	1.99970	2.86925	-0.09951
C	3.75406	-0.36034	-0.04683
C	3.03405	1.83901	-0.48577
H	-3.54081	-1.34235	-1.60821
H	-2.78544	-2.78229	-0.90702
H	-3.66763	0.37961	1.50263
H	-4.69371	-0.26124	0.18717
H	-1.44362	-1.81056	-2.75544
H	-1.21232	-0.30207	-1.80676
H	-3.26678	0.95359	-1.49422
H	-4.04545	2.01501	-0.28843
H	1.24203	-0.90538	-1.81569
H	0.84631	-2.43489	-2.67349
H	-2.37291	3.65239	-0.70655
H	-1.50527	2.58054	-1.85906
H	1.56980	-3.74252	-0.66871
H	2.89204	-2.76305	-1.37025
H	-0.04991	4.31629	-0.78876
H	-0.38750	3.59918	0.82296
H	4.15149	-2.39559	0.54402
H	3.39841	-1.38268	1.81378
H	2.03692	3.05432	0.99461
H	2.23567	3.82203	-0.61668
H	4.77842	-0.03926	0.23739
H	3.75897	-0.56345	-1.13747
H	2.96726	1.63269	-1.57378
H	4.04425	2.24529	-0.27175

C	-2.98518	-2.07215	1.39339
H	-2.22243	-2.86885	1.34932
H	-3.99070	-2.54325	1.30018
H	-2.92184	-1.59137	2.38639
Rb	0.06003	-0.02199	1.18279

ACE4 Rb SCS-MP2 def2tzv

47

O	-0.27674	-2.16262	-0.94633
O	-2.11231	1.73938	0.05167
O	2.16708	-2.05763	0.57081
O	0.61943	2.50657	-0.51083
O	2.81106	0.77354	0.22952
N	-2.69099	-1.16033	0.33371
C	-2.66976	-1.80957	-1.01155
C	-3.70153	-0.07397	0.43448
C	-1.35429	-1.55603	-1.74931
C	-3.36481	1.13676	-0.43589
C	1.03050	-2.13711	-1.62050
C	-1.75429	2.94615	-0.71035
C	2.03323	-2.81355	-0.69295
C	-0.43686	3.47547	-0.17313
C	3.50201	-1.47700	0.80728
C	1.96299	3.00500	-0.17266
C	3.79402	-0.27403	-0.08450
C	2.97639	1.96605	-0.61726
H	-3.49395	-1.43409	-1.63724
H	-2.81193	-2.89369	-0.90745
H	-3.74657	0.24776	1.48078
H	-4.70912	-0.43138	0.14363
H	-1.37474	-2.01911	-2.74591

H	-1.16867	-0.47814	-1.85695
H	-3.24552	0.86068	-1.49172
H	-4.17617	1.87370	-0.35930
H	1.32404	-1.09826	-1.82399
H	0.97174	-2.68821	-2.56927
H	-2.53502	3.70781	-0.58287
H	-1.65525	2.69872	-1.77516
H	1.68855	-3.81939	-0.43786
H	3.00561	-2.88468	-1.19624
H	-0.21315	4.44057	-0.64617
H	-0.49032	3.60673	0.91527
H	4.27361	-2.24161	0.65026
H	3.49724	-1.17914	1.85760
H	2.03876	3.17444	0.90900
H	2.14878	3.94766	-0.70393
H	4.80894	0.09213	0.12245
H	3.72288	-0.53375	-1.14874
H	2.81328	1.70201	-1.66989
H	3.99060	2.36726	-0.49258
C	-2.85413	-2.15053	1.43394
H	-2.06937	-2.90643	1.35076
H	-3.83750	-2.65327	1.40541
H	-2.75220	-1.63968	2.39675
Rb	0.05901	0.01308	1.10468

ACE4 NoMetal B2PLYP def2tzv

46

O	-0.03073	-2.36962	-0.57172
O	-2.41133	1.38508	0.18286
O	2.62115	-1.93357	0.77342
O	0.29615	2.52896	-0.36587

O	2.73659	1.04116	0.48419
N	-2.89247	-1.58456	0.82244
C	-2.45554	-2.33972	-0.36170
C	-3.88873	-0.52839	0.58576
C	-1.25143	-1.79080	-1.14696
C	-3.53773	0.62531	-0.35917
C	1.18688	-1.92108	-1.24379
C	-2.11124	2.57890	-0.60555
C	2.37593	-2.54514	-0.53775
C	-0.90958	3.27598	-0.01303
C	3.82246	-1.10211	0.86374
C	1.50842	3.11159	0.20576
C	3.79985	0.15831	0.01702
C	2.70193	2.30629	-0.24891
H	-3.31201	-2.41677	-1.03497
H	-2.19172	-3.35672	-0.05513
H	-4.16005	-0.10904	1.55674
H	-4.78829	-0.99816	0.17314
H	-1.31946	-2.08543	-2.19998
H	-1.19692	-0.70159	-1.08499
H	-3.28586	0.25985	-1.35995
H	-4.41183	1.28141	-0.44893
H	1.24909	-0.83044	-1.21192
H	1.16785	-2.24774	-2.28991
H	-2.97166	3.25531	-0.58257
H	-1.90804	2.30215	-1.64434
H	2.17786	-3.60225	-0.36336
H	3.26503	-2.45762	-1.16721
H	-0.84048	4.29050	-0.41854
H	-1.01023	3.33577	1.07420

H	4.69954	-1.69236	0.57847
H	3.90041	-0.84031	1.91626
H	1.44644	3.11155	1.29750
H	1.61682	4.14363	-0.14292
H	4.77027	0.65646	0.12129
H	3.65054	-0.07036	-1.04249
H	2.63295	2.11429	-1.32350
H	3.61942	2.86834	-0.04848
C	-1.83277	-1.27918	1.79859
H	-1.33601	-2.20762	2.08487
H	-2.28826	-0.84122	2.68782
H	-1.07343	-0.58551	1.42455

ACE4 NoMetal B3LYP def2tzv

46

O	-0.05252	-2.47949	-0.51073
O	-2.41122	1.48299	0.18739
O	2.57667	-1.89289	0.79363
O	0.31109	2.60142	-0.37622
O	2.74333	1.07884	0.45113
N	-2.86131	-1.52106	0.80659
C	-2.48097	-2.40539	-0.29638
C	-3.84979	-0.47547	0.53771
C	-1.27404	-2.00806	-1.16327
C	-3.48674	0.68675	-0.39503
C	1.16371	-2.12592	-1.22972
C	-2.09759	2.67324	-0.59425
C	2.35850	-2.63774	-0.44794
C	-0.88270	3.35487	-0.01029
C	3.78639	-1.07848	0.85156
C	1.53920	3.16475	0.17107

C	3.77348	0.16470	-0.01999
C	2.71454	2.33561	-0.28930
H	-3.34984	-2.51023	-0.95008
H	-2.26228	-3.40200	0.10342
H	-4.15625	-0.05436	1.49836
H	-4.73771	-0.94654	0.10113
H	-1.35432	-2.48035	-2.14946
H	-1.22000	-0.92537	-1.30051
H	-3.18238	0.32566	-1.38304
H	-4.37742	1.31384	-0.52695
H	1.22148	-1.04128	-1.35756
H	1.15540	-2.59444	-2.22131
H	-2.94846	3.36243	-0.56890
H	-1.90424	2.39952	-1.63619
H	2.18641	-3.67788	-0.16915
H	3.24939	-2.59482	-1.07959
H	-0.81088	4.37230	-0.40956
H	-0.97238	3.41400	1.07860
H	4.65649	-1.68435	0.57740
H	3.88041	-0.79062	1.89683
H	1.49238	3.17917	1.26422
H	1.66409	4.19266	-0.18598
H	4.75812	0.64138	0.05238
H	3.59834	-0.08341	-1.07180
H	2.63158	2.13761	-1.36228
H	3.64218	2.88867	-0.10815
C	-1.83767	-1.24445	1.81896
H	-1.38895	-2.18544	2.14366
H	-2.30769	-0.77334	2.68443
H	-1.03099	-0.58878	1.47369

ACE4 NoMetal B97-1 def2tzv

46

O	-0.03228	-2.40493	-0.55706
O	-2.42190	1.43486	0.20262
O	2.60697	-1.91683	0.78545
O	0.29501	2.55661	-0.36955
O	2.73650	1.06145	0.47522
N	-2.87581	-1.57370	0.80662
C	-2.46152	-2.36639	-0.35647
C	-3.86764	-0.51750	0.57479
C	-1.24733	-1.87432	-1.17085
C	-3.51965	0.65603	-0.35478
C	1.18238	-1.99413	-1.24367
C	-2.11580	2.61575	-0.59187
C	2.37738	-2.57266	-0.50042
C	-0.89940	3.31055	-0.01591
C	3.80359	-1.08955	0.86870
C	1.51198	3.13580	0.18016
C	3.78874	0.17107	0.01490
C	2.70213	2.31390	-0.26808
H	-3.32190	-2.44173	-1.02898
H	-2.22230	-3.38585	-0.02651
H	-4.15085	-0.10971	1.55053
H	-4.76808	-0.98022	0.14889
H	-1.31722	-2.24441	-2.20321
H	-1.19836	-0.77958	-1.19446
H	-3.24917	0.30048	-1.35799
H	-4.41115	1.29251	-0.45430
H	1.24292	-0.89989	-1.28093
H	1.17549	-2.38101	-2.27269

H	-2.96982	3.30541	-0.57224
H	-1.92232	2.33122	-1.63392
H	2.19002	-3.62885	-0.29085
H	3.26888	-2.50040	-1.13313
H	-0.83200	4.32488	-0.43074
H	-0.99199	3.38468	1.07459
H	4.68656	-1.68038	0.59127
H	3.88395	-0.81732	1.92181
H	1.45961	3.15757	1.27564
H	1.63101	4.16449	-0.18470
H	4.76770	0.66012	0.11615
H	3.64130	-0.06507	-1.04697
H	2.62939	2.11202	-1.34401
H	3.62388	2.87968	-0.08062
C	-1.83521	-1.30435	1.80753
H	-1.36226	-2.24789	2.09605
H	-2.29842	-0.86587	2.69592
H	-1.04649	-0.62346	1.46149

ACE4 NoMetal CAM-B3LYP def2tzv

46

O	-0.02270	-2.36527	-0.55265
O	-2.41646	1.39298	0.18609
O	2.61472	-1.90008	0.77894
O	0.28512	2.52985	-0.35634
O	2.72862	1.05339	0.46386
N	-2.86275	-1.56528	0.79722
C	-2.43724	-2.34365	-0.36101
C	-3.86443	-0.52948	0.56788
C	-1.23250	-1.82713	-1.15027
C	-3.51860	0.62882	-0.36028

C	1.18845	-1.95078	-1.22533
C	-2.11000	2.57619	-0.58850
C	2.37004	-2.54687	-0.49815
C	-0.90972	3.26498	-0.00095
C	3.80293	-1.07507	0.85904
C	1.49462	3.10555	0.19054
C	3.77679	0.17169	0.00528
C	2.67839	2.30119	-0.26874
H	-3.28986	-2.42635	-1.03609
H	-2.18678	-3.35852	-0.03951
H	-4.14661	-0.11961	1.53850
H	-4.75957	-1.00010	0.15046
H	-1.29276	-2.16339	-2.18999
H	-1.18939	-0.73640	-1.13771
H	-3.25638	0.27040	-1.35992
H	-4.39911	1.27290	-0.45741
H	1.25543	-0.86076	-1.23740
H	1.17620	-2.31310	-2.25852
H	-2.96564	3.25704	-0.56910
H	-1.90809	2.30306	-1.62757
H	2.16724	-3.59647	-0.29188
H	3.25842	-2.48765	-1.12982
H	-0.84400	4.28219	-0.39738
H	-1.00766	3.32094	1.08592
H	4.68181	-1.66469	0.58279
H	3.88662	-0.79877	1.90665
H	1.44704	3.11269	1.28222
H	1.60171	4.13591	-0.16026
H	4.74843	0.66732	0.09595
H	3.62362	-0.06876	-1.05033

H	2.59637	2.10279	-1.34035
H	3.59620	2.86769	-0.08910
C	-1.83265	-1.28548	1.79415
H	-1.34233	-2.21734	2.07651
H	-2.29912	-0.86116	2.68305
H	-1.06333	-0.58959	1.44872

ACE4 NoMetal DSDPBEP86 def2tzv

46

O	-0.55757	-1.52928	-1.68598
O	-1.49344	2.43172	0.13451
O	1.85598	-2.62359	-0.25449
O	1.47710	2.50671	-0.02545
O	3.06953	0.03013	0.37186
N	-3.10219	-0.15657	-0.12660
C	-2.79854	-0.62602	-1.48829
C	-3.60723	1.22355	-0.04003
C	-1.37688	-0.35720	-2.01128
C	-2.74586	2.35161	-0.61188
C	0.82556	-1.39279	-2.13270
C	-0.67666	3.56611	-0.28423
C	1.57881	-2.63564	-1.69336
C	0.61288	3.55738	0.50249
C	3.25415	-2.37787	0.09476
C	2.73228	2.40218	0.71114
C	3.79222	-1.02224	-0.32970
C	3.59651	1.35396	0.05146
H	-3.52904	-0.16938	-2.16484
H	-2.94759	-1.71242	-1.52970
H	-3.80672	1.43774	1.01533
H	-4.56834	1.26538	-0.57047

H	-1.38498	-0.22005	-3.10152
H	-0.93915	0.53047	-1.54078
H	-2.53178	2.19207	-1.67756
H	-3.29366	3.30036	-0.51432
H	1.27275	-0.49478	-1.69065
H	0.85045	-1.30849	-3.22845
H	-1.21987	4.49993	-0.08846
H	-0.45939	3.49922	-1.35810
H	0.96510	-3.51733	-1.89303
H	2.51420	-2.72668	-2.25784
H	1.11337	4.52905	0.39801
H	0.40249	3.37644	1.56370
H	3.88707	-3.15479	-0.35501
H	3.29300	-2.47115	1.18060
H	2.53630	2.12968	1.75533
H	3.25331	3.36839	0.68591
H	4.85918	-0.98083	-0.06945
H	3.70267	-0.87226	-1.41338
H	3.59735	1.50471	-1.03545
H	4.62576	1.43778	0.42358
C	-2.10291	-0.54261	0.88543
H	-2.00234	-1.63232	0.88536
H	-2.45793	-0.22365	1.86970
H	-1.11061	-0.10399	0.71702

ACE4 NoMetal M062X def2tzv

46

O	0.01944	-2.25905	-0.61998
O	-2.45041	1.30318	0.20208
O	2.65693	-1.90705	0.75921
O	0.22875	2.44420	-0.32163

O	2.69606	1.03864	0.48589
N	-2.82390	-1.63551	0.81360
C	-2.37953	-2.30690	-0.40910
C	-3.86245	-0.62076	0.62908
C	-1.19747	-1.66244	-1.14063
C	-3.55882	0.53305	-0.31809
C	1.22615	-1.74240	-1.22558
C	-2.15492	2.46597	-0.60431
C	2.40184	-2.43259	-0.56844
C	-0.97288	3.18667	-0.01296
C	3.82510	-1.05982	0.87207
C	1.42044	3.06141	0.21515
C	3.77574	0.19635	0.02986
C	2.62247	2.28303	-0.24797
H	-3.23427	-2.37486	-1.08367
H	-2.07451	-3.32852	-0.16602
H	-4.10843	-0.21060	1.60974
H	-4.76193	-1.11330	0.24852
H	-1.25661	-1.85544	-2.21601
H	-1.17540	-0.58119	-0.97326
H	-3.31402	0.16614	-1.32093
H	-4.44419	1.17246	-0.40075
H	1.28840	-0.66100	-1.07319
H	1.21811	-1.95524	-2.29925
H	-3.02309	3.13067	-0.61940
H	-1.92635	2.16153	-1.62999
H	2.17062	-3.49080	-0.45602
H	3.29318	-2.33862	-1.19281
H	-0.90154	4.19050	-0.44115
H	-1.09466	3.27310	1.07004

H	4.71878	-1.62713	0.59610
H	3.88846	-0.79266	1.92403
H	1.37819	3.07278	1.30735
H	1.49492	4.09120	-0.14604
H	4.73028	0.72118	0.13549
H	3.63797	-0.03815	-1.03046
H	2.53715	2.07915	-1.31918
H	3.53080	2.86544	-0.07092
C	-1.74572	-1.30069	1.74774
H	-1.19802	-2.20957	1.99878
H	-2.17911	-0.89194	2.66015
H	-1.03313	-0.57122	1.34820

ACE4 NoMetal M06 def2tzv

46

O	-0.08619	-2.33624	-0.69194
O	-2.37791	1.40668	0.22476
O	2.44366	-1.91254	0.70368
O	0.32369	2.42100	-0.37050
O	2.73542	1.02049	0.56973
N	-2.75574	-1.50967	0.87218
C	-2.45548	-2.27295	-0.32931
C	-3.78112	-0.48342	0.75234
C	-1.30813	-1.75706	-1.19588
C	-3.52271	0.65543	-0.21866
C	1.10092	-1.83177	-1.32480
C	-2.05354	2.48962	-0.66506
C	2.28611	-2.44999	-0.62878
C	-0.85306	3.21834	-0.13820
C	3.65677	-1.17200	0.93929
C	1.51644	3.04274	0.14425

C	3.76608	0.10982	0.15240
C	2.70466	2.21210	-0.23794
H	-3.36667	-2.32582	-0.93111
H	-2.19470	-3.30202	-0.05207
H	-3.95187	-0.05949	1.74619
H	-4.71915	-0.95866	0.44157
H	-1.44868	-2.05458	-2.24301
H	-1.24129	-0.66065	-1.15587
H	-3.34942	0.27416	-1.23438
H	-4.40578	1.30793	-0.25900
H	1.13834	-0.73675	-1.24155
H	1.10633	-2.09990	-2.38948
H	-2.90140	3.18189	-0.73723
H	-1.84053	2.10067	-1.66950
H	2.12251	-3.52641	-0.53801
H	3.19087	-2.29424	-1.22401
H	-0.75359	4.18367	-0.64970
H	-0.97650	3.40891	0.93472
H	4.53152	-1.79323	0.71257
H	3.65930	-0.94936	2.00597
H	1.45492	3.13589	1.23501
H	1.62530	4.04795	-0.28134
H	4.75701	0.54869	0.32669
H	3.67736	-0.08077	-0.92558
H	2.63395	1.94248	-1.29952
H	3.62502	2.78949	-0.08981
C	-1.62519	-1.22962	1.74423
H	-1.10807	-2.16413	1.97709
H	-1.98885	-0.79060	2.67571
H	-0.88548	-0.53989	1.31124

ACE4 NoMetal MP2 def2tzv

46

O	-0.00152	-2.33479	-0.63248
O	-2.43733	1.32077	0.17778
O	2.66155	-1.95328	0.74425
O	0.25757	2.48699	-0.36651
O	2.71439	1.04150	0.53044
N	-2.86251	-1.63350	0.86704
C	-2.41375	-2.32756	-0.35997
C	-3.88804	-0.59070	0.64084
C	-1.23591	-1.68360	-1.11918
C	-3.56860	0.53983	-0.34461
C	1.21105	-1.78437	-1.25859
C	-2.14467	2.49218	-0.66015
C	2.40778	-2.46908	-0.61529
C	-0.97038	3.23340	-0.05806
C	3.85344	-1.09374	0.85120
C	1.44238	3.09142	0.25898
C	3.79411	0.18473	0.02713
C	2.66669	2.32366	-0.18978
H	-3.28227	-2.41168	-1.02364
H	-2.09823	-3.34438	-0.09256
H	-4.12990	-0.14810	1.61375
H	-4.79244	-1.09067	0.26615
H	-1.32492	-1.85317	-2.20167
H	-1.17256	-0.60740	-0.92237
H	-3.32004	0.14937	-1.34120
H	-4.45021	1.19114	-0.44034
H	1.25044	-0.70169	-1.09193
H	1.19133	-1.99162	-2.33810

H	-3.02344	3.15017	-0.68371
H	-1.90107	2.17524	-1.68269
H	2.20606	-3.53857	-0.51780
H	3.29968	-2.33337	-1.23958
H	-0.90352	4.23849	-0.49510
H	-1.09935	3.31663	1.02803
H	4.74025	-1.66266	0.53978
H	3.92877	-0.85632	1.91286
H	1.35203	3.05762	1.35144
H	1.53049	4.13635	-0.06699
H	4.75798	0.70363	0.12879
H	3.62866	-0.02517	-1.03732
H	2.61371	2.14480	-1.27129
H	3.57148	2.90200	0.03814
C	-1.75988	-1.26691	1.78695
H	-1.24113	-2.18047	2.09487
H	-2.19248	-0.79022	2.67174
H	-1.02446	-0.58066	1.34685

ACE4 NoMetal PBE0 def2tzv

46

O	-0.02463	-2.36759	-0.55686
O	-2.40802	1.40167	0.20033
O	2.61062	-1.91193	0.77608
O	0.28695	2.52609	-0.36526
O	2.71659	1.04911	0.47769
N	-2.86266	-1.57367	0.79615
C	-2.43813	-2.33763	-0.36906
C	-3.85189	-0.52810	0.57215
C	-1.22919	-1.82420	-1.15368
C	-3.50627	0.63760	-0.34691

C	1.18018	-1.94134	-1.22835
C	-2.10884	2.57761	-0.58350
C	2.36985	-2.53417	-0.51133
C	-0.90092	3.27006	-0.01543
C	3.79227	-1.08254	0.85813
C	1.49075	3.10462	0.18427
C	3.76572	0.17323	0.01789
C	2.67894	2.29488	-0.25450
H	-3.29181	-2.40741	-1.04899
H	-2.19533	-3.36156	-0.05883
H	-4.13527	-0.12469	1.54859
H	-4.75280	-0.98926	0.14795
H	-1.29282	-2.15676	-2.19816
H	-1.18497	-0.72994	-1.14006
H	-3.24730	0.28305	-1.35280
H	-4.39422	1.27823	-0.44098
H	1.24273	-0.84752	-1.23531
H	1.17136	-2.29642	-2.26779
H	-2.96301	3.26554	-0.56034
H	-1.91921	2.29994	-1.62729
H	2.18131	-3.59394	-0.32658
H	3.25592	-2.45308	-1.14924
H	-0.83874	4.28409	-0.42943
H	-0.99427	3.34721	1.07403
H	4.67846	-1.66358	0.57340
H	3.87979	-0.81580	1.91119
H	1.43561	3.12757	1.27881
H	1.60839	4.13364	-0.17741
H	4.74185	0.66612	0.11766
H	3.62144	-0.05956	-1.04442

H	2.61419	2.09869	-1.33122
H	3.59567	2.86549	-0.06238
C	-1.83170	-1.30569	1.79107
H	-1.34748	-2.24488	2.07070
H	-2.29562	-0.88104	2.68447
H	-1.05208	-0.61322	1.45111

ACE4 NoMetal PBE def2sv

46

O	-0.00399	-2.32230	-0.63654
O	-2.39686	1.36268	0.17547
O	2.52347	-1.87416	0.70219
O	0.23423	2.46394	-0.35767
O	2.61232	1.03632	0.46820
N	-2.68719	-1.56746	0.87799
C	-2.37625	-2.36930	-0.29196
C	-3.73476	-0.57121	0.73313
C	-1.22964	-1.89421	-1.20461
C	-3.51311	0.59812	-0.23575
C	1.14483	-1.84752	-1.30798
C	-2.14781	2.48063	-0.65240
C	2.37560	-2.40896	-0.60713
C	-0.93439	3.23070	-0.14041
C	3.67411	-1.07867	0.92473
C	1.39508	3.08336	0.15984
C	3.72954	0.23290	0.14738
C	2.62425	2.27769	-0.20947
H	-3.30267	-2.46009	-0.90112
H	-2.09922	-3.40639	0.02608
H	-3.94094	-0.14749	1.74323
H	-4.67266	-1.07977	0.40172

H	-1.35119	-2.33293	-2.23060
H	-1.24290	-0.78056	-1.31216
H	-3.36350	0.21877	-1.28016
H	-4.44040	1.23114	-0.25455
H	1.17855	-0.72814	-1.29396
H	1.14521	-2.17940	-2.38016
H	-3.02578	3.17714	-0.65591
H	-1.97044	2.16140	-1.71190
H	2.25421	-3.51519	-0.51889
H	3.27665	-2.22089	-1.23640
H	-0.86654	4.21588	-0.67076
H	-1.07024	3.44786	0.95024
H	4.61259	-1.64787	0.69976
H	3.66913	-0.85177	2.01350
H	1.32999	3.17933	1.27450
H	1.51397	4.11694	-0.25669
H	4.68836	0.75146	0.41287
H	3.76757	0.04145	-0.95639
H	2.64799	2.12290	-1.31903
H	3.53454	2.87131	0.06397
C	-1.57429	-1.24176	1.75190
H	-1.01395	-2.17163	2.00278
H	-1.96715	-0.80653	2.69930
H	-0.83297	-0.51713	1.32803

ACE4 NoMetal PBE def2tzv

46

O	-0.02931	-2.44041	-0.55241
O	-2.42254	1.43996	0.21160
O	2.61330	-1.92872	0.78648
O	0.29310	2.56327	-0.39437

O	2.72753	1.06790	0.49637
N	-2.87085	-1.57126	0.81669
C	-2.46021	-2.38425	-0.33440
C	-3.86652	-0.52063	0.57290
C	-1.25515	-1.90870	-1.16898
C	-3.52299	0.65326	-0.35597
C	1.18238	-2.00125	-1.24923
C	-2.12323	2.62338	-0.60100
C	2.38763	-2.56962	-0.52055
C	-0.90822	3.32441	-0.03695
C	3.81298	-1.08615	0.86246
C	1.50863	3.14920	0.17806
C	3.78818	0.17900	0.02064
C	2.70342	2.32981	-0.25260
H	-3.33156	-2.47816	-1.00185
H	-2.21368	-3.40425	0.01462
H	-4.16243	-0.11058	1.55223
H	-4.76809	-0.99169	0.13920
H	-1.33485	-2.29338	-2.20369
H	-1.20181	-0.80707	-1.20788
H	-3.24539	0.29697	-1.36549
H	-4.42342	1.29024	-0.46078
H	1.22636	-0.89847	-1.27672
H	1.17408	-2.37945	-2.29003
H	-2.98525	3.31552	-0.58429
H	-1.93450	2.32865	-1.64921
H	2.21900	-3.63949	-0.32485
H	3.28130	-2.47255	-1.15911
H	-0.84477	4.34378	-0.46054
H	-0.99565	3.40779	1.06113

H	4.70491	-1.67212	0.57291
H	3.89865	-0.82323	1.92505
H	1.43859	3.17273	1.28032
H	1.63369	4.18573	-0.18598
H	4.77363	0.67324	0.12099
H	3.63723	-0.04942	-1.05040
H	2.64765	2.12703	-1.33721
H	3.62872	2.90034	-0.05194
C	-1.82998	-1.28480	1.81351
H	-1.33841	-2.22606	2.10367
H	-2.29916	-0.84931	2.70887
H	-1.04710	-0.58576	1.46549

ACE4 NoMetal B2PLYP def2sv

46

O	0.00862	-2.28140	-0.65215
O	-2.38662	1.32070	0.16591
O	2.51387	-1.85439	0.68961
O	0.21870	2.43712	-0.34845
O	2.59462	1.02746	0.46782
N	-2.67056	-1.58504	0.88179
C	-2.35329	-2.34093	-0.31529
C	-3.72060	-0.59182	0.75434
C	-1.21056	-1.81651	-1.19337
C	-3.51132	0.56454	-0.22099
C	1.15613	-1.80207	-1.31223
C	-2.15103	2.43866	-0.65746
C	2.37532	-2.37904	-0.61872
C	-0.95103	3.19577	-0.14133
C	3.66208	-1.06953	0.92842
C	1.37036	3.05955	0.17308

C	3.71752	0.23728	0.15549
C	2.59959	2.26744	-0.20183
H	-3.27039	-2.41353	-0.92362
H	-2.07223	-3.37616	-0.03770
H	-3.90490	-0.16541	1.75586
H	-4.65448	-1.09996	0.44624
H	-1.32792	-2.19060	-2.23252
H	-1.21632	-0.70992	-1.22702
H	-3.38241	0.18547	-1.25544
H	-4.42284	1.19973	-0.22180
H	1.19434	-0.69574	-1.27440
H	1.15221	-2.11370	-2.37836
H	-3.02944	3.11590	-0.65515
H	-1.97022	2.12435	-1.70524
H	2.24970	-3.47420	-0.54166
H	3.27498	-2.18826	-1.23030
H	-0.88116	4.16825	-0.67059
H	-1.09285	3.40886	0.93712
H	4.58738	-1.63977	0.70841
H	3.64980	-0.84557	2.00685
H	1.30390	3.14174	1.27678
H	1.47581	4.08566	-0.23455
H	4.65712	0.76083	0.43031
H	3.76199	0.05040	-0.93639
H	2.62010	2.11746	-1.30006
H	3.49897	2.85473	0.07460
C	-1.54460	-1.24632	1.73234
H	-0.97760	-2.16283	1.97113
H	-1.92282	-0.81798	2.67677
H	-0.83175	-0.52251	1.29137

ACE4 NoMetal B3LYP def2sv

46

O	-0.10198	-2.37073	-0.61819
O	-2.32549	1.43680	0.13718
O	2.41372	-1.89376	0.67176
O	0.33294	2.48199	-0.36241
O	2.65653	0.98702	0.46596
N	-2.70984	-1.45869	0.90877
C	-2.46506	-2.33938	-0.21720
C	-3.72210	-0.43274	0.74625
C	-1.33057	-1.96663	-1.18502
C	-3.46515	0.70300	-0.24987
C	1.04112	-1.95327	-1.32482
C	-2.04060	2.53903	-0.69153
C	2.27544	-2.47922	-0.61062
C	-0.82074	3.26851	-0.16942
C	3.60105	-1.17340	0.92266
C	1.50390	3.06549	0.16073
C	3.73273	0.13447	0.15291
C	2.71008	2.22607	-0.20279
H	-3.40296	-2.42331	-0.79364
H	-2.22720	-3.35866	0.15323
H	-3.90463	0.02187	1.73788
H	-4.67132	-0.91379	0.43733
H	-1.48467	-2.48179	-2.15915
H	-1.32320	-0.87567	-1.38231
H	-3.33479	0.30089	-1.27720
H	-4.36109	1.36278	-0.27452
H	1.08382	-0.84504	-1.37747
H	1.02569	-2.34556	-2.36567

H	-2.89724	3.24635	-0.70899
H	-1.85767	2.21036	-1.73674
H	2.17449	-3.57548	-0.48830
H	3.16422	-2.29513	-1.24227
H	-0.72495	4.23762	-0.70471
H	-0.96536	3.49490	0.90807
H	4.49869	-1.79169	0.71125
H	3.59199	-0.95147	2.00356
H	1.43434	3.15909	1.26542
H	1.65030	4.08652	-0.25197
H	4.70308	0.60240	0.42891
H	3.76801	-0.05304	-0.94140
H	2.73577	2.07543	-1.30291
H	3.62861	2.78401	0.07924
C	-1.57521	-1.15115	1.75866
H	-1.05947	-2.08762	2.04062
H	-1.93477	-0.66526	2.68481
H	-0.81587	-0.48299	1.30153

ACE4 NoMetal B97-1 def2sv

46

O	-0.00980	-2.31617	-0.64234
O	-2.39412	1.36414	0.16495
O	2.50945	-1.86567	0.68920
O	0.23824	2.46067	-0.34992
O	2.61585	1.03198	0.46554
N	-2.67971	-1.55886	0.88759
C	-2.37814	-2.36553	-0.28198
C	-3.72694	-0.56209	0.74583
C	-1.23456	-1.89343	-1.20008
C	-3.50800	0.60029	-0.23219

C	1.13405	-1.84887	-1.31344
C	-2.13859	2.47069	-0.66492
C	2.36665	-2.40552	-0.61083
C	-0.92749	3.22503	-0.15013
C	3.65924	-1.08605	0.92809
C	1.39569	3.07505	0.16420
C	3.72724	0.22788	0.15401
C	2.62512	2.26855	-0.20719
H	-3.30204	-2.45185	-0.88311
H	-2.10735	-3.39539	0.03689
H	-3.92297	-0.13386	1.74805
H	-4.66084	-1.06980	0.42725
H	-1.36090	-2.33518	-2.21515
H	-1.24797	-0.78810	-1.31074
H	-3.36514	0.21719	-1.26741
H	-4.42724	1.23055	-0.24667
H	1.16798	-0.73718	-1.30534
H	1.13265	-2.18621	-2.37524
H	-3.01022	3.16219	-0.67669
H	-1.95481	2.14481	-1.71274
H	2.25243	-3.50458	-0.52041
H	3.26167	-2.21389	-1.23462
H	-0.85389	4.19648	-0.68786
H	-1.07014	3.44962	0.92976
H	4.58619	-1.66053	0.70922
H	3.64781	-0.86285	2.01016
H	1.33171	3.16804	1.27097
H	1.51255	4.10041	-0.25148
H	4.67740	0.73966	0.42897
H	3.77097	0.03952	-0.94174

H	2.64610	2.11501	-1.30880
H	3.52919	2.85653	0.06637
C	-1.55801	-1.22638	1.74822
H	-1.00268	-2.14978	2.00299
H	-1.93996	-0.78331	2.68823
H	-0.82707	-0.51276	1.30898

ACE4 NoMetal CAM-B3LYP def2sv

46

O	0.01509	-2.27803	-0.65005
O	-2.39063	1.31450	0.16178
O	2.52071	-1.84350	0.69229
O	0.20981	2.44203	-0.34810
O	2.58948	1.03360	0.45537
N	-2.65559	-1.57939	0.87023
C	-2.34197	-2.34459	-0.31585
C	-3.71213	-0.59918	0.74997
C	-1.20025	-1.82853	-1.19413
C	-3.50905	0.55906	-0.21984
C	1.16071	-1.80428	-1.30219
C	-2.15653	2.43328	-0.64853
C	2.37750	-2.37523	-0.60466
C	-0.95808	3.18794	-0.13236
C	3.65781	-1.05406	0.92918
C	1.35919	3.05722	0.16791
C	3.70807	0.24670	0.15098
C	2.58647	2.26801	-0.20970
H	-3.25786	-2.42007	-0.92472
H	-2.06309	-3.37876	-0.03224
H	-3.89851	-0.17748	1.75289
H	-4.64340	-1.11147	0.44233

H	-1.31397	-2.21357	-2.22943
H	-1.21275	-0.72201	-1.24079
H	-3.38202	0.18299	-1.25593
H	-4.42200	1.19159	-0.21765
H	1.19802	-0.69738	-1.26985
H	1.16174	-2.11944	-2.36709
H	-3.03533	3.10981	-0.64113
H	-1.97770	2.12688	-1.69923
H	2.24868	-3.46961	-0.52026
H	3.27671	-2.19257	-1.21940
H	-0.89472	4.16582	-0.65220
H	-1.09689	3.39135	0.94856
H	4.58690	-1.61989	0.71428
H	3.64472	-0.82371	2.00644
H	1.29624	3.13688	1.27221
H	1.46406	4.08478	-0.23601
H	4.64648	0.77407	0.42182
H	3.75391	0.05341	-0.94007
H	2.60399	2.11862	-1.30830
H	3.48540	2.85772	0.06259
C	-1.54087	-1.25012	1.73053
H	-0.97076	-2.16618	1.96411
H	-1.92298	-0.83194	2.67808
H	-0.82655	-0.52023	1.30123

ACE4 NoMetal DSDPBEP86 def2sv

46

O	0.05678	-2.20898	-0.77983
O	-2.42686	1.24270	0.20831
O	2.54428	-1.85535	0.65353
O	0.14473	2.39104	-0.39709

O	2.53691	1.03747	0.51625
N	-2.54804	-1.65253	0.89769
C	-2.26654	-2.32163	-0.35496
C	-3.64243	-0.71038	0.86767
C	-1.17355	-1.69495	-1.22853
C	-3.54480	0.45574	-0.11178
C	1.18746	-1.58626	-1.32621
C	-2.22254	2.31039	-0.67427
C	2.40362	-2.24036	-0.69562
C	-1.04015	3.11631	-0.19530
C	3.64641	-1.04165	0.94733
C	1.25188	3.01754	0.19390
C	3.68447	0.29112	0.21814
C	2.51476	2.26885	-0.15293
H	-3.21068	-2.39109	-0.92702
H	-1.93479	-3.36140	-0.15762
H	-3.76351	-0.29429	1.88497
H	-4.58092	-1.25375	0.62923
H	-1.32896	-1.96014	-2.30000
H	-1.18423	-0.58913	-1.14028
H	-3.46612	0.07997	-1.15604
H	-4.47810	1.06272	-0.05654
H	1.18391	-0.49906	-1.10302
H	1.21577	-1.72500	-2.43234
H	-3.11856	2.96981	-0.70784
H	-2.03043	1.94498	-1.70635
H	2.25590	-3.33603	-0.72659
H	3.31380	-2.00613	-1.28375
H	-1.00288	4.08270	-0.74687
H	-1.18381	3.34371	0.88185

H	4.60420	-1.56934	0.73519
H	3.59604	-0.85006	2.03245
H	1.14111	3.05458	1.29828
H	1.34970	4.06347	-0.17469
H	4.60455	0.83305	0.53626
H	3.76859	0.13934	-0.88022
H	2.55990	2.12222	-1.25393
H	3.39071	2.88915	0.14223
C	-1.38160	-1.27638	1.67962
H	-0.72397	-2.15520	1.79624
H	-1.71670	-0.95130	2.68213
H	-0.77291	-0.46115	1.23865

ACE4 NoMetal M062X def2sv

46

O	0.05188	-2.16198	-0.68257
O	-2.42104	1.24955	0.19178
O	2.56619	-1.86060	0.67048
O	0.17160	2.36966	-0.32260
O	2.57063	1.01373	0.49134
N	-2.64390	-1.64129	0.86535
C	-2.29786	-2.30369	-0.37583
C	-3.71900	-0.67456	0.77279
C	-1.16433	-1.67220	-1.18873
C	-3.53223	0.48621	-0.19479
C	1.19187	-1.62375	-1.29555
C	-2.18833	2.34709	-0.64798
C	2.40357	-2.28022	-0.66504
C	-0.99909	3.12064	-0.13822
C	3.69008	-1.05669	0.92162
C	1.30447	3.01298	0.19689

C	3.70566	0.25853	0.16695
C	2.54785	2.24712	-0.17633
H	-3.20799	-2.36463	-0.99561
H	-1.97942	-3.34406	-0.16806
H	-3.89251	-0.26112	1.78145
H	-4.64565	-1.19985	0.47406
H	-1.26101	-1.94265	-2.26083
H	-1.19485	-0.56620	-1.10770
H	-3.39662	0.10975	-1.23095
H	-4.44921	1.11193	-0.19568
H	1.23645	-0.52591	-1.14088
H	1.17729	-1.82662	-2.38662
H	-3.07310	3.01456	-0.66711
H	-1.99370	2.00922	-1.68652
H	2.24346	-3.37284	-0.66750
H	3.30645	-2.07224	-1.26665
H	-0.92857	4.08392	-0.68222
H	-1.15027	3.34740	0.93627
H	4.62607	-1.60122	0.68402
H	3.68063	-0.84890	2.00311
H	1.23464	3.09223	1.30057
H	1.38671	4.03998	-0.21204
H	4.63175	0.80548	0.43915
H	3.74482	0.08509	-0.92845
H	2.56654	2.09270	-1.27439
H	3.43666	2.85038	0.09688
C	-1.51827	-1.28120	1.70338
H	-0.91590	-2.18225	1.91401
H	-1.89607	-0.88449	2.66103
H	-0.83964	-0.52501	1.26083

ACE4 NoMetal M06 def2sv

46

O	0.05613	-2.27578	-0.70896
O	-2.42370	1.27566	0.19807
O	2.51223	-1.80814	0.64856
O	0.13684	2.39782	-0.37690
O	2.51985	1.06617	0.51475
N	-2.50742	-1.59243	0.91140
C	-2.26558	-2.37434	-0.27708
C	-3.61630	-0.67088	0.86317
C	-1.18209	-1.86107	-1.21505
C	-3.52178	0.47326	-0.12710
C	1.16635	-1.70655	-1.33558
C	-2.21524	2.33034	-0.69374
C	2.40535	-2.26138	-0.67765
C	-1.03904	3.14178	-0.23390
C	3.62184	-1.00851	0.94979
C	1.25578	3.05112	0.14827
C	3.66152	0.31392	0.22285
C	2.50102	2.28102	-0.17897
H	-3.21927	-2.46615	-0.83064
H	-1.96373	-3.40920	0.00143
H	-3.75090	-0.24027	1.87602
H	-4.54650	-1.23212	0.63287
H	-1.32887	-2.26493	-2.24396
H	-1.21933	-0.74925	-1.28904
H	-3.42170	0.08627	-1.16910
H	-4.46391	1.07034	-0.10365
H	1.15098	-0.59594	-1.22817
H	1.17146	-1.94383	-2.42475

H	-3.11419	2.98529	-0.74752
H	-2.02610	1.94283	-1.72188
H	2.32969	-3.36815	-0.66882
H	3.30184	-2.00265	-1.27638
H	-0.98094	4.08312	-0.82586
H	-1.19078	3.43017	0.83107
H	4.57432	-1.54604	0.74254
H	3.57622	-0.82170	2.03852
H	1.16162	3.16398	1.25247
H	1.34769	4.07484	-0.28000
H	4.58407	0.85903	0.52935
H	3.74416	0.16008	-0.87814
H	2.54305	2.10010	-1.27807
H	3.39013	2.89607	0.08739
C	-1.34458	-1.20571	1.67074
H	-0.72865	-2.09935	1.89380
H	-1.66725	-0.76137	2.63235
H	-0.67535	-0.46817	1.16967

ACE4 NoMetal MP2 def2sv

46

O	-0.00136	-2.19403	-0.73050
O	-2.37015	1.28729	0.19936
O	2.47442	-1.88308	0.63983
O	0.22541	2.36392	-0.35633
O	2.57491	0.98186	0.52692
N	-2.63686	-1.60582	0.89773
C	-2.34102	-2.27537	-0.35380
C	-3.68838	-0.61080	0.83024
C	-1.22413	-1.67145	-1.20619
C	-3.51456	0.54240	-0.14717

C	1.13569	-1.63144	-1.34223
C	-2.13583	2.36193	-0.67983
C	2.34795	-2.28195	-0.71323
C	-0.94106	3.13846	-0.19327
C	3.62641	-1.12597	0.93988
C	1.35601	3.00196	0.19172
C	3.70996	0.20659	0.22331
C	2.59497	2.21797	-0.14875
H	-3.27426	-2.32481	-0.94347
H	-2.03630	-3.32251	-0.15228
H	-3.82303	-0.18807	1.84258
H	-4.63923	-1.11518	0.56387
H	-1.36269	-1.94371	-2.27574
H	-1.22421	-0.56672	-1.12777
H	-3.42853	0.16242	-1.18696
H	-4.42194	1.18519	-0.11266
H	1.17026	-0.53622	-1.17228
H	1.12689	-1.82186	-2.43799
H	-3.01580	3.03862	-0.71283
H	-1.94946	1.99519	-1.71068
H	2.21145	-3.37918	-0.74251
H	3.25592	-2.04323	-1.29716
H	-0.86086	4.08706	-0.76554
H	-1.09269	3.39791	0.87460
H	4.55145	-1.69864	0.71682
H	3.59033	-0.94600	2.02709
H	1.26149	3.08837	1.29410
H	1.46251	4.02745	-0.22075
H	4.64019	0.71950	0.55076
H	3.79152	0.06354	-0.87369

H	2.63976	2.06231	-1.24652
H	3.48691	2.80982	0.14712
C	-1.47853	-1.26907	1.70354
H	-0.88938	-2.18397	1.89298
H	-1.82519	-0.87218	2.67447
H	-0.79777	-0.52176	1.25094

ACE4 NoMetal PBE0 def2sv

46

O	0.00190	-2.30443	-0.63041
O	-2.38300	1.34692	0.16903
O	2.50465	-1.84666	0.69260
O	0.22590	2.45055	-0.35324
O	2.59231	1.03487	0.46245
N	-2.66166	-1.55914	0.87326
C	-2.35480	-2.35332	-0.29105
C	-3.70506	-0.57274	0.73268
C	-1.21200	-1.87794	-1.18861
C	-3.48567	0.58443	-0.23324
C	1.14049	-1.84583	-1.29980
C	-2.13874	2.45455	-0.64910
C	2.36460	-2.39098	-0.59680
C	-0.93455	3.20482	-0.14265
C	3.64448	-1.06333	0.91978
C	1.37527	3.06317	0.15873
C	3.69703	0.23804	0.14511
C	2.59852	2.26405	-0.20704
H	-3.27291	-2.43608	-0.89815
H	-2.08816	-3.38496	0.01985
H	-3.90705	-0.14936	1.73385
H	-4.63585	-1.08051	0.41145

H	-1.33084	-2.30622	-2.20808
H	-1.22480	-0.77312	-1.28706
H	-3.33653	0.20474	-1.26699
H	-4.40535	1.21019	-0.25327
H	1.17465	-0.73625	-1.30206
H	1.14025	-2.19016	-2.35721
H	-3.01157	3.14136	-0.65056
H	-1.96231	2.13849	-1.69911
H	2.25403	-3.48818	-0.50221
H	3.25908	-2.20222	-1.21916
H	-0.86865	4.17881	-0.67249
H	-1.07076	3.42242	0.93756
H	4.57220	-1.63182	0.69854
H	3.64152	-0.83539	1.99902
H	1.31038	3.15706	1.26344
H	1.49078	4.08724	-0.25510
H	4.64666	0.75405	0.40528
H	3.73058	0.04514	-0.94843
H	2.62110	2.11054	-1.30666
H	3.49980	2.85327	0.06471
C	-1.55198	-1.23173	1.73415
H	-1.00142	-2.15428	1.99432
H	-1.93610	-0.78689	2.66996
H	-0.81669	-0.52316	1.29937

ACE3 K B2PLYP def2sv

40

O	2.72866	0.06471	0.33473
O	-2.02749	-1.20226	-0.27691
O	0.71339	1.98169	0.15219
O	-2.00620	1.60994	-0.36132

N	0.67050	-1.84643	0.36643
C	2.05446	-2.23017	0.07352
C	-0.30564	-2.87377	0.00757
C	2.87875	-1.07068	-0.48728
C	-1.74481	-2.46489	0.30466
C	2.97497	1.32581	-0.24428
C	-3.09116	-0.49541	0.33104
C	2.05879	2.34836	0.39957
C	-3.21849	0.87329	-0.32358
C	-0.25739	2.88234	0.65645
C	-1.56234	2.14411	0.87346
H	2.06593	-3.03082	-0.68433
H	2.54879	-2.64437	0.97594
H	-0.12859	-3.83253	0.54728
H	-0.20382	-3.08981	-1.07021
H	3.94185	-1.37566	-0.56727
H	2.53567	-0.84021	-1.51582
H	-2.42823	-3.23741	-0.09625
H	-1.91405	-2.41554	1.39463
H	2.79076	1.29897	-1.33610
H	4.02726	1.63891	-0.09725
H	-2.90470	-0.40212	1.41790
H	-4.05296	-1.03068	0.20702
H	2.24412	2.38540	1.48997
H	2.28048	3.34804	-0.02142
H	-3.51823	0.74981	-1.37564
H	-4.00968	1.44692	0.19532
H	-0.40097	3.72398	-0.04789
H	0.06726	3.29788	1.62832
H	-1.40065	1.34329	1.61708

H	-2.31420	2.84749	1.28062
K	-0.13635	0.14132	-1.66716
C	0.53124	-1.39931	1.74904
H	1.34374	-0.70116	1.97825
H	0.56925	-2.24775	2.46924
H	-0.41899	-0.86351	1.89118

Output Geometry Optimization (XYZ format) (Section 2.10)

ACE1_Li_ub2plyp_def2svpp

26

N	-1.40041	-0.10234	-0.10925
C	-1.02156	0.94307	0.86094
H	-1.91333	1.44414	1.27972
H	-0.50742	0.48674	1.71983
C	-0.11702	1.99298	0.21298
H	-0.67380	2.56657	-0.54336
H	0.26535	2.70180	0.96835
O	0.96339	1.38596	-0.50020
C	1.99684	0.80375	0.29432
H	2.90866	1.42335	0.23969
H	1.68780	0.76384	1.35051
C	2.28843	-0.59554	-0.24751
H	2.81506	-0.52763	-1.21116
H	2.92642	-1.15968	0.45487
O	1.08003	-1.29797	-0.53076
C	0.32745	-1.75200	0.59666
H	0.48047	-2.83666	0.73018
H	0.68583	-1.25850	1.51352
C	-1.15469	-1.47276	0.35991
H	-1.71377	-1.71083	1.28939

H	-1.52397	-2.15429	-0.42374
C	-2.77179	0.06733	-0.59269
H	-2.90130	1.08687	-0.98806
H	-3.52229	-0.09596	0.20791
H	-2.97004	-0.64435	-1.40970
Li	0.04196	0.00204	-1.69108

ACE1_Li_ub2plyp_def2svp

26

N	-1.40006	-0.10199	-0.11066
C	-1.01769	0.94155	0.85924
H	-1.90462	1.44098	1.28405
H	-0.49957	0.48489	1.71302
C	-0.11695	1.99243	0.21195
H	-0.67458	2.56602	-0.54088
H	0.26147	2.70013	0.96699
O	0.96467	1.38731	-0.50176
C	1.99350	0.80097	0.29589
H	2.90581	1.41671	0.25167
H	1.67972	0.75674	1.34851
C	2.28730	-0.59513	-0.24675
H	2.81633	-0.52525	-1.20669
H	2.92568	-1.15510	0.45505
O	1.08046	-1.29956	-0.53205
C	0.32624	-1.74872	0.59645
H	0.47964	-2.82985	0.73974
H	0.68038	-1.24967	1.50974
C	-1.15384	-1.47224	0.35796
H	-1.71158	-1.71131	1.28508
H	-1.52303	-2.15296	-0.42345
C	-2.77262	0.06756	-0.58896

H	-2.90503	1.08302	-0.98518
H	-3.51852	-0.09326	0.21201
H	-2.97530	-0.64396	-1.40087
Li	0.04231	0.00212	-1.69274

ACE1_Li_ub2plyp_def2sv

26

N	-1.40041	-0.10234	-0.10925
C	-1.02156	0.94307	0.86094
H	-1.91333	1.44414	1.27972
H	-0.50742	0.48674	1.71983
C	-0.11702	1.99298	0.21298
H	-0.67380	2.56657	-0.54336
H	0.26535	2.70180	0.96835
O	0.96339	1.38596	-0.50020
C	1.99684	0.80375	0.29432
H	2.90866	1.42335	0.23969
H	1.68780	0.76384	1.35051
C	2.28843	-0.59554	-0.24751
H	2.81506	-0.52763	-1.21116
H	2.92642	-1.15968	0.45487
O	1.08003	-1.29797	-0.53076
C	0.32745	-1.75200	0.59666
H	0.48047	-2.83666	0.73018
H	0.68583	-1.25850	1.51352
C	-1.15469	-1.47276	0.35991
H	-1.71377	-1.71083	1.28939
H	-1.52397	-2.15429	-0.42374
C	-2.77179	0.06733	-0.59269
H	-2.90130	1.08687	-0.98806
H	-3.52229	-0.09596	0.20791

H -2.97004 -0.64435 -1.40970

Li 0.04196 0.00204 -1.69108

ACE1_Li_ub2plyp_def2tzvpp

26

N -1.39286 -0.10147 -0.12415

C -1.01438 0.93996 0.85102

H -1.89820 1.43209 1.26372

H -0.50284 0.48586 1.69672

C -0.12240 1.99512 0.20668

H -0.67549 2.55635 -0.54340

H 0.24730 2.69558 0.95720

O 0.97304 1.40359 -0.50763

C 1.97882 0.79533 0.31206

H 2.88347 1.40393 0.29261

H 1.63854 0.74245 1.34420

C 2.28646 -0.58804 -0.24225

H 2.80730 -0.50362 -1.19326

H 2.92209 -1.13961 0.45211

O 1.09049 -1.31635 -0.53800

C 0.32824 -1.74253 0.60167

H 0.47870 -2.81154 0.75238

H 0.68294 -1.23452 1.49629

C -1.14787 -1.46893 0.35525

H -1.70173 -1.69427 1.27579

H -1.51130 -2.14754 -0.41621

C -2.77874 0.05745 -0.57224

H -2.92230 1.06228 -0.96306

H -3.49466 -0.10454 0.24155

H -2.98981 -0.65317 -1.36848

Li 0.03232 -0.00247 -1.70909

ACE1_Li_ub2plyp_def2tzvp

26

N	-1.39275	-0.10170	-0.12332
C	-1.01537	0.94044	0.85170
H	-1.90076	1.43338	1.26385
H	-0.50423	0.48612	1.69905
C	-0.12282	1.99532	0.20737
H	-0.67629	2.55763	-0.54371
H	0.24769	2.69670	0.95878
O	0.97233	1.40337	-0.50754
C	1.97950	0.79588	0.31136
H	2.88501	1.40589	0.29030
H	1.64016	0.74341	1.34517
C	2.28678	-0.58770	-0.24301
H	2.80758	-0.50370	-1.19575
H	2.92373	-1.13993	0.45194
O	1.09031	-1.31599	-0.53799
C	0.32871	-1.74322	0.60201
H	0.47969	-2.81384	0.75161
H	0.68447	-1.23556	1.49790
C	-1.14754	-1.46935	0.35619
H	-1.70193	-1.69514	1.27800
H	-1.51161	-2.14879	-0.41614
C	-2.77814	0.05695	-0.57390
H	-2.92143	1.06306	-0.96508
H	-3.49647	-0.10562	0.23940
H	-2.98806	-0.65398	-1.37187
Li	0.03131	-0.00222	-1.70878

ACE1_Li_ub2plyp_def2tzv

26

N	-1.40634	-0.10274	-0.11817
C	-1.02879	0.94975	0.87375
H	-1.91809	1.43173	1.29233
H	-0.50452	0.49456	1.71313
C	-0.14501	2.02057	0.22468
H	-0.69271	2.57957	-0.53017
H	0.24212	2.71427	0.97227
O	0.97445	1.40759	-0.51885
C	2.03165	0.80325	0.31053
H	2.92270	1.42819	0.26350
H	1.70269	0.75282	1.34765
C	2.33538	-0.58646	-0.25126
H	2.84463	-0.51591	-1.20871
H	2.95609	-1.15326	0.44375
O	1.09306	-1.31669	-0.55043
C	0.31579	-1.77443	0.61724
H	0.47430	-2.84466	0.74348
H	0.67468	-1.27159	1.51450
C	-1.16676	-1.49264	0.36477
H	-1.72347	-1.70837	1.28758
H	-1.53576	-2.16527	-0.40983
C	-2.80133	0.06732	-0.60240
H	-2.93005	1.07846	-0.98473
H	-3.53408	-0.10315	0.19625
H	-2.99243	-0.63478	-1.41235
Li	0.02423	-0.00492	-1.69039

ACE1_Li_ub3lyp_def2svpp

26

N	-1.41088	-0.10123	-0.10274
C	-1.02697	0.95038	0.86301

H	-1.91735	1.45948	1.27761
H	-0.51879	0.49455	1.72725
C	-0.11207	1.99795	0.21622
H	-0.66367	2.57879	-0.54068
H	0.27072	2.70439	0.97552
O	0.96864	1.38972	-0.49787
C	2.01087	0.80503	0.28562
H	2.92580	1.42117	0.21496
H	1.71735	0.77337	1.34814
C	2.29373	-0.60227	-0.25131
H	2.81835	-0.54309	-1.21854
H	2.93500	-1.16398	0.45230
O	1.08242	-1.30423	-0.52700
C	0.32363	-1.75889	0.59812
H	0.47229	-2.84596	0.72879
H	0.68228	-1.26993	1.51913
C	-1.16113	-1.47544	0.36120
H	-1.71993	-1.72091	1.29052
H	-1.53249	-2.15465	-0.42531
C	-2.77753	0.07075	-0.60203
H	-2.90218	1.08924	-1.00599
H	-3.54053	-0.08572	0.19073
H	-2.97158	-0.64507	-1.41871
Li	0.03645	0.00266	-1.67419

ACE1_Li_ub3lyp_def2svp

26

N	-1.41031	-0.10049	-0.10398
C	-1.02319	0.94930	0.86197
H	-1.90924	1.45690	1.28268
H	-0.51154	0.49353	1.72219

C	-0.11189	1.99758	0.21561
H	-0.66443	2.57884	-0.53795
H	0.26738	2.70398	0.97402
O	0.97007	1.39050	-0.49945
C	2.00884	0.80180	0.28710
H	2.92436	1.41486	0.22563
H	1.71197	0.76644	1.34694
C	2.29295	-0.60213	-0.25163
H	2.81904	-0.54068	-1.21612
H	2.93605	-1.16097	0.44974
O	1.08193	-1.30554	-0.52824
C	0.32187	-1.75585	0.59881
H	0.47146	-2.83991	0.73857
H	0.67686	-1.26228	1.51697
C	-1.16073	-1.47498	0.35964
H	-1.71941	-1.72209	1.28618
H	-1.53153	-2.15395	-0.42486
C	-2.77843	0.07139	-0.59942
H	-2.90592	1.08590	-1.00498
H	-3.53804	-0.08252	0.19307
H	-2.97651	-0.64466	-1.41127
Li	0.03641	0.00259	-1.67467

ACE1_Li_ub3lyp_def2sv

26

N	-1.41088	-0.10123	-0.10274
C	-1.02697	0.95038	0.86301
H	-1.91735	1.45948	1.27761
H	-0.51879	0.49455	1.72725
C	-0.11207	1.99795	0.21622
H	-0.66367	2.57879	-0.54068

H	0.27072	2.70439	0.97552
O	0.96864	1.38972	-0.49787
C	2.01087	0.80503	0.28562
H	2.92580	1.42117	0.21496
H	1.71735	0.77337	1.34814
C	2.29373	-0.60227	-0.25131
H	2.81835	-0.54309	-1.21854
H	2.93500	-1.16398	0.45230
O	1.08242	-1.30423	-0.52700
C	0.32363	-1.75889	0.59812
H	0.47229	-2.84596	0.72879
H	0.68228	-1.26993	1.51913
C	-1.16113	-1.47544	0.36120
H	-1.71993	-1.72091	1.29052
H	-1.53249	-2.15465	-0.42531
C	-2.77753	0.07075	-0.60203
H	-2.90218	1.08924	-1.00599
H	-3.54053	-0.08572	0.19073
H	-2.97158	-0.64507	-1.41871
Li	0.03645	0.00266	-1.67419

ACE1_Li_ub3lyp_def2tzvpp

26

N	-1.40409	-0.10050	-0.11457
C	-1.02100	0.94820	0.85428
H	-1.90337	1.44874	1.26298
H	-0.51554	0.49588	1.70603
C	-0.11789	1.99979	0.21111
H	-0.66555	2.56916	-0.53906
H	0.25252	2.69828	0.96524
O	0.97756	1.40684	-0.50454

C	1.99534	0.79695	0.30171
H	2.90301	1.40327	0.26395
H	1.67328	0.75221	1.34154
C	2.29182	-0.59422	-0.24799
H	2.80907	-0.51914	-1.20369
H	2.93223	-1.14445	0.44548
O	1.09175	-1.32164	-0.53372
C	0.32383	-1.75153	0.60287
H	0.46982	-2.82371	0.74726
H	0.68004	-1.25163	1.50305
C	-1.15439	-1.47154	0.35868
H	-1.70703	-1.70536	1.27950
H	-1.52064	-2.14776	-0.41529
C	-2.78298	0.06146	-0.58584
H	-2.91904	1.06547	-0.98521
H	-3.51677	-0.09448	0.21540
H	-2.98529	-0.65177	-1.38413
Li	0.02633	-0.00250	-1.68799

ACE1_Li_ub3lyp_def2tzvp

26

N	-1.40397	-0.10062	-0.11408
C	-1.02123	0.94836	0.85475
H	-1.90438	1.44942	1.26329
H	-0.51545	0.49581	1.70714
C	-0.11804	1.99991	0.21159
H	-0.66619	2.56982	-0.53903
H	0.25283	2.69862	0.96650
O	0.97718	1.40680	-0.50456
C	1.99552	0.79708	0.30132
H	2.90387	1.40389	0.26253

H	1.67390	0.75228	1.34212
C	2.29182	-0.59409	-0.24845
H	2.80909	-0.51893	-1.20509
H	2.93287	-1.14450	0.44565
O	1.09153	-1.32160	-0.53384
C	0.32396	-1.75179	0.60306
H	0.46988	-2.82491	0.74694
H	0.68050	-1.25170	1.50395
C	-1.15430	-1.47175	0.35925
H	-1.70697	-1.70569	1.28101
H	-1.52089	-2.14853	-0.41520
C	-2.78246	0.06140	-0.58686
H	-2.91786	1.06618	-0.98665
H	-3.51763	-0.09465	0.21420
H	-2.98387	-0.65228	-1.38603
Li	0.02563	-0.00227	-1.68785

ACE1_Li_ub3lyp_def2tzv

26

N	-1.41481	-0.10219	-0.11082
C	-1.03196	0.95503	0.87282
H	-1.91853	1.44567	1.28613
H	-0.51685	0.50277	1.71915
C	-0.13997	2.01961	0.22446
H	-0.68549	2.58237	-0.52996
H	0.24239	2.71612	0.97227
O	0.97793	1.40782	-0.51556
C	2.03884	0.80232	0.30371
H	2.93237	1.42360	0.24528
H	1.72426	0.75893	1.34563
C	2.33496	-0.58990	-0.25463

H	2.84001	-0.52136	-1.21515
H	2.96528	-1.15318	0.43507
O	1.09469	-1.32051	-0.54446
C	0.31279	-1.77363	0.61904
H	0.46908	-2.84414	0.74878
H	0.66780	-1.27222	1.51859
C	-1.16830	-1.49300	0.36222
H	-1.72896	-1.72495	1.27876
H	-1.53004	-2.16294	-0.41839
C	-2.80219	0.06927	-0.60716
H	-2.92706	1.07555	-1.00409
H	-3.54572	-0.08767	0.18500
H	-2.99396	-0.64033	-1.41099
Li	0.02107	-0.00650	-1.67432

ACE1_Li_ub971_def2svpp

26

N	-1.40931	-0.10268	-0.10266
C	-1.02733	0.94867	0.86431
H	-1.91978	1.45786	1.27903
H	-0.51740	0.49243	1.72994
C	-0.11128	1.99841	0.21510
H	-0.66562	2.57837	-0.54313
H	0.26971	2.70739	0.97567
O	0.96800	1.39249	-0.49653
C	2.00468	0.80728	0.28852
H	2.92175	1.42377	0.22266
H	1.70738	0.77202	1.35203
C	2.29084	-0.60171	-0.25195
H	2.81577	-0.53787	-1.22062
H	2.93648	-1.16195	0.45171

O	1.08479	-1.30660	-0.52779
C	0.33022	-1.75734	0.59786
H	0.47913	-2.84575	0.73214
H	0.68918	-1.26513	1.51962
C	-1.15864	-1.47630	0.36335
H	-1.71620	-1.72086	1.29564
H	-1.53046	-2.15800	-0.42334
C	-2.77783	0.06742	-0.59923
H	-2.90340	1.08752	-1.00330
H	-3.53998	-0.09055	0.19626
H	-2.97159	-0.64991	-1.41679
Li	0.02796	0.00794	-1.70069

ACE1_Li_ub971_def2svp

26

N	-1.40858	-0.10230	-0.10366
C	-1.02393	0.94757	0.86380
H	-1.91217	1.45482	1.28420
H	-0.51095	0.49155	1.72545
C	-0.11188	1.99795	0.21422
H	-0.66787	2.57693	-0.54094
H	0.26495	2.70801	0.97285
O	0.96873	1.39330	-0.49818
C	2.00244	0.80480	0.28982
H	2.91962	1.41845	0.23292
H	1.70226	0.76627	1.35060
C	2.29037	-0.60100	-0.25210
H	2.81706	-0.53517	-1.21764
H	2.93764	-1.15802	0.44941
O	1.08465	-1.30741	-0.52885
C	0.32895	-1.75440	0.59856

H	0.47947	-2.83943	0.74190
H	0.68411	-1.25740	1.51721
C	-1.15787	-1.47633	0.36170
H	-1.71508	-1.72302	1.29098
H	-1.52908	-2.15741	-0.42302
C	-2.77854	0.06772	-0.59693
H	-2.90651	1.08341	-1.00330
H	-3.53748	-0.08676	0.19801
H	-2.97621	-0.65016	-1.40912
Li	0.02868	0.00637	-1.70067

ACE1_Li_ub971_def2sv

26

N	-1.40931	-0.10268	-0.10266
C	-1.02733	0.94867	0.86431
H	-1.91978	1.45786	1.27903
H	-0.51740	0.49243	1.72994
C	-0.11128	1.99841	0.21510
H	-0.66562	2.57837	-0.54313
H	0.26971	2.70739	0.97567
O	0.96800	1.39249	-0.49653
C	2.00468	0.80728	0.28852
H	2.92175	1.42377	0.22266
H	1.70738	0.77202	1.35203
C	2.29084	-0.60171	-0.25195
H	2.81577	-0.53787	-1.22062
H	2.93648	-1.16195	0.45171
O	1.08479	-1.30660	-0.52779
C	0.33022	-1.75734	0.59786
H	0.47913	-2.84575	0.73214
H	0.68918	-1.26513	1.51962

C	-1.15864	-1.47630	0.36335
H	-1.71620	-1.72086	1.29564
H	-1.53046	-2.15800	-0.42334
C	-2.77783	0.06742	-0.59923
H	-2.90340	1.08752	-1.00330
H	-3.53998	-0.09055	0.19626
H	-2.97159	-0.64991	-1.41679
Li	0.02796	0.00794	-1.70069

ACE1_Li_ub971_def2tzvpp

26

N	-1.40098	-0.09982	-0.11806
C	-1.01797	0.94710	0.85387
H	-1.90210	1.44846	1.26385
H	-0.51037	0.49242	1.70642
C	-0.11312	2.00140	0.20785
H	-0.66536	2.56977	-0.54341
H	0.25454	2.70262	0.96453
O	0.98106	1.41195	-0.50539
C	1.98816	0.79816	0.30550
H	2.90115	1.40134	0.27589
H	1.65927	0.75076	1.34596
C	2.28695	-0.59674	-0.24637
H	2.80783	-0.51755	-1.20251
H	2.92941	-1.14578	0.44999
O	1.09198	-1.32566	-0.53479
C	0.32817	-1.75120	0.60200
H	0.47233	-2.82567	0.74967
H	0.68539	-1.24876	1.50372
C	-1.15472	-1.47063	0.35993
H	-1.70669	-1.70038	1.28474

H	-1.52349	-2.14994	-0.41361
C	-2.78460	0.06192	-0.57883
H	-2.92284	1.06850	-0.97712
H	-3.51278	-0.09502	0.23019
H	-2.99224	-0.65293	-1.37730
Li	0.01707	-0.00983	-1.71228

ACE1_Li_ub971_def2tzvp

26

N	-1.40088	-0.09994	-0.11757
C	-1.01827	0.94730	0.85434
H	-1.90316	1.44916	1.26417
H	-0.51047	0.49237	1.70753
C	-0.11324	2.00154	0.20841
H	-0.66591	2.57052	-0.54325
H	0.25485	2.70298	0.96587
O	0.98071	1.41197	-0.50540
C	1.98844	0.79827	0.30506
H	2.90214	1.40188	0.27441
H	1.66013	0.75086	1.34647
C	2.28696	-0.59665	-0.24691
H	2.80776	-0.51738	-1.20403
H	2.93022	-1.14584	0.44991
O	1.09173	-1.32567	-0.53491
C	0.32829	-1.75147	0.60225
H	0.47245	-2.82683	0.74956
H	0.68589	-1.24882	1.50460
C	-1.15464	-1.47083	0.36060
H	-1.70665	-1.70067	1.28632
H	-1.52381	-2.15071	-0.41332
C	-2.78409	0.06185	-0.57996

H	-2.92169	1.06913	-0.97876
H	-3.51379	-0.09510	0.22874
H	-2.99085	-0.65344	-1.37928
Li	0.01631	-0.00967	-1.71210

ACE1_Li_ub971_def2tzv

26

N	-1.41153	-0.10240	-0.11423
C	-1.02979	0.95305	0.87256
H	-1.91851	1.44419	1.28743
H	-0.51155	0.49910	1.71976
C	-0.13730	2.02074	0.22095
H	-0.68805	2.58125	-0.53530
H	0.24208	2.72121	0.97059
O	0.97982	1.41256	-0.51669
C	2.03075	0.80394	0.30806
H	2.92912	1.42363	0.25861
H	1.70843	0.75638	1.35051
C	2.33070	-0.59096	-0.25322
H	2.83951	-0.51743	-1.21435
H	2.96338	-1.15328	0.43928
O	1.09553	-1.32329	-0.54642
C	0.31797	-1.77303	0.61750
H	0.47344	-2.84599	0.75026
H	0.67375	-1.26874	1.51878
C	-1.16772	-1.49288	0.36274
H	-1.72792	-1.72202	1.28322
H	-1.53127	-2.16587	-0.41818
C	-2.80322	0.06847	-0.60104
H	-2.93064	1.07765	-0.99655
H	-3.54225	-0.09029	0.19821

H	-2.99952	-0.64233	-1.40598
Li	0.01652	-0.01025	-1.68905

ACE1_Li_ucam-b3lyp_def2svpp

26

N	-1.39973	-0.10268	-0.10289
C	-1.01948	0.94149	0.86067
H	-1.90986	1.44308	1.28002
H	-0.50523	0.48575	1.71963
C	-0.11502	1.98806	0.21338
H	-0.67122	2.56533	-0.54075
H	0.27023	2.69571	0.96793
O	0.95744	1.37788	-0.49628
C	1.99570	0.80358	0.28551
H	2.90653	1.42320	0.22010
H	1.69733	0.76831	1.34529
C	2.28256	-0.59623	-0.25097
H	2.81002	-0.53384	-1.21467
H	2.91941	-1.16026	0.45215
O	1.07483	-1.28941	-0.52640
C	0.32626	-1.75018	0.59254
H	0.47993	-2.83514	0.72094
H	0.68442	-1.26130	1.51233
C	-1.15359	-1.47083	0.35900
H	-1.71208	-1.71203	1.28715
H	-1.52449	-2.15002	-0.42562
C	-2.76067	0.07026	-0.59913
H	-2.88615	1.08892	-0.99848
H	-3.51946	-0.08898	0.19441
H	-2.95595	-0.64172	-1.41679
Li	0.04067	0.00238	-1.65602

ACE1_Li_ucam-b3lyp_def2svp

26

N	-1.39914	-0.10217	-0.10388
C	-1.01633	0.94057	0.85956
H	-1.90295	1.44100	1.28409
H	-0.49945	0.48495	1.71525
C	-0.11509	1.98766	0.21303
H	-0.67218	2.56589	-0.53775
H	0.26701	2.69494	0.96723
O	0.95830	1.37880	-0.49781
C	1.99349	0.80083	0.28645
H	2.90501	1.41765	0.22957
H	1.69222	0.76219	1.34393
C	2.28179	-0.59568	-0.25141
H	2.81067	-0.53128	-1.21270
H	2.92086	-1.15653	0.44985
O	1.07480	-1.29084	-0.52746
C	0.32500	-1.74716	0.59329
H	0.47934	-2.82947	0.73050
H	0.68007	-1.25397	1.51045
C	-1.15275	-1.47027	0.35793
H	-1.71090	-1.71296	1.28387
H	-1.52344	-2.14960	-0.42457
C	-2.76112	0.07021	-0.59711
H	-2.88953	1.08517	-0.99815
H	-3.51725	-0.08670	0.19590
H	-2.95984	-0.64212	-1.41038
Li	0.03985	0.00179	-1.65606

ACE1_Li_ucam-b3lyp_def2sv

26

N	-1.39973	-0.10268	-0.10289
C	-1.01948	0.94149	0.86067
H	-1.90986	1.44308	1.28002
H	-0.50523	0.48575	1.71963
C	-0.11502	1.98806	0.21338
H	-0.67122	2.56533	-0.54075
H	0.27023	2.69571	0.96793
O	0.95744	1.37788	-0.49628
C	1.99570	0.80358	0.28551
H	2.90653	1.42320	0.22010
H	1.69733	0.76831	1.34529
C	2.28256	-0.59623	-0.25097
H	2.81002	-0.53384	-1.21467
H	2.91941	-1.16026	0.45215
O	1.07483	-1.28941	-0.52640
C	0.32626	-1.75018	0.59254
H	0.47993	-2.83514	0.72094
H	0.68442	-1.26130	1.51233
C	-1.15359	-1.47083	0.35900
H	-1.71208	-1.71203	1.28715
H	-1.52449	-2.15002	-0.42562
C	-2.76067	0.07026	-0.59913
H	-2.88615	1.08892	-0.99848
H	-3.51946	-0.08898	0.19441
H	-2.95595	-0.64172	-1.41679
Li	0.04067	0.00238	-1.65602

ACE1_Li_ucam-b3lyp_def2tzvpp

26

N	-1.39214	-0.10322	-0.11393
C	-1.01484	0.93809	0.85220

H	-1.89835	1.43093	1.26472
H	-0.50407	0.48684	1.70006
C	-0.12335	1.98904	0.20828
H	-0.67689	2.55470	-0.53905
H	0.24941	2.68932	0.95798
O	0.96299	1.39476	-0.50317
C	1.97768	0.79746	0.30070
H	2.88088	1.40848	0.26779
H	1.65193	0.74897	1.33843
C	2.28029	-0.58550	-0.24833
H	2.79942	-0.50580	-1.20149
H	2.91897	-1.13641	0.44440
O	1.08578	-1.30641	-0.53258
C	0.32849	-1.74189	0.59739
H	0.47964	-2.81238	0.74046
H	0.68383	-1.24194	1.49699
C	-1.14475	-1.46739	0.35593
H	-1.69777	-1.69902	1.27532
H	-1.50909	-2.14396	-0.41728
C	-2.76346	0.05885	-0.58476
H	-2.90104	1.06398	-0.97835
H	-3.49503	-0.10188	0.21576
H	-2.96535	-0.64969	-1.38592
Li	0.03266	-0.00277	-1.66159

ACE1_Li_ucam-b3lyp_def2tzvp

26

N	-1.39200	-0.10342	-0.11344
C	-1.01527	0.93828	0.85260
H	-1.89969	1.43168	1.26472
H	-0.50443	0.48699	1.70137

C	-0.12364	1.98918	0.20875
H	-0.67763	2.55540	-0.53904
H	0.24950	2.68968	0.95930
O	0.96250	1.39486	-0.50319
C	1.97786	0.79775	0.30029
H	2.88170	1.40933	0.26617
H	1.65265	0.74941	1.33903
C	2.28032	-0.58518	-0.24876
H	2.79927	-0.50545	-1.20298
H	2.91969	-1.13632	0.44450
O	1.08573	-1.30647	-0.53266
C	0.32877	-1.74223	0.59760
H	0.47981	-2.81371	0.73995
H	0.68466	-1.24233	1.49796
C	-1.14452	-1.46759	0.35658
H	-1.69749	-1.69934	1.27699
H	-1.50918	-2.14486	-0.41702
C	-2.76294	0.05858	-0.58582
H	-2.89988	1.06447	-0.97991
H	-3.49588	-0.10219	0.21455
H	-2.96393	-0.65045	-1.38784
Li	0.03186	-0.00274	-1.66141

ACE1_Li_ucam-b3lyp_def2tzv

26

N	-1.40291	-0.10473	-0.10785
C	-1.02468	0.94454	0.87049
H	-1.91143	1.42798	1.28793
H	-0.50406	0.49399	1.71281
C	-0.14379	2.00696	0.22110
H	-0.69443	2.56549	-0.53089

H	0.23991	2.70608	0.96366
O	0.96296	1.39271	-0.51079
C	2.02125	0.80194	0.30113
H	2.91015	1.42718	0.24488
H	1.70662	0.75503	1.34192
C	2.32082	-0.58246	-0.25637
H	2.82727	-0.51059	-1.21436
H	2.94935	-1.14699	0.43160
O	1.08619	-1.30206	-0.54140
C	0.31709	-1.76313	0.61202
H	0.47863	-2.83171	0.73888
H	0.67086	-1.26351	1.51191
C	-1.15861	-1.48760	0.35985
H	-1.71889	-1.71745	1.27487
H	-1.51967	-2.15733	-0.41946
C	-2.77977	0.06797	-0.60669
H	-2.90492	1.07517	-0.99780
H	-3.52303	-0.09358	0.18247
H	-2.96999	-0.63609	-1.41393
Li	0.02563	-0.00500	-1.65037

ACE1_Li_udsdpbepb86_def2svpp

26

N	-1.38861	-0.10694	-0.11015
C	-1.01710	0.93283	0.86366
H	-1.91338	1.42372	1.29090
H	-0.49256	0.47620	1.71877
C	-0.12709	1.98854	0.21049
H	-0.69503	2.55334	-0.54722
H	0.25372	2.70601	0.96165
O	0.95201	1.38607	-0.50227

C	1.97812	0.80822	0.29948
H	2.88865	1.43434	0.25876
H	1.65755	0.75965	1.35404
C	2.28173	-0.58430	-0.24787
H	2.80899	-0.50577	-1.21270
H	2.92495	-1.14867	0.45286
O	1.08049	-1.29229	-0.53381
C	0.33682	-1.74649	0.59545
H	0.49642	-2.83186	0.73227
H	0.69577	-1.24738	1.51160
C	-1.14519	-1.47388	0.36021
H	-1.70652	-1.71039	1.29116
H	-1.51225	-2.15842	-0.42502
C	-2.75881	0.06259	-0.59041
H	-2.88876	1.08627	-0.98066
H	-3.50830	-0.10600	0.21255
H	-2.95644	-0.64632	-1.41258
Li	0.03887	-0.00382	-1.69760

ACE1_Li_udsdpbepb86_def2svp

26

N	-1.38866	-0.10679	-0.11126
C	-1.01332	0.93125	0.86160
H	-1.90416	1.42066	1.29472
H	-0.48450	0.47390	1.71065
C	-0.12664	1.98806	0.20964
H	-0.69477	2.55357	-0.54397
H	0.25021	2.70282	0.96144
O	0.95353	1.38780	-0.50343
C	1.97447	0.80527	0.30106
H	2.88526	1.42694	0.27185

H	1.64796	0.75125	1.35118
C	2.28036	-0.58410	-0.24713
H	2.80986	-0.50370	-1.20787
H	2.92323	-1.14374	0.45349
O	1.08096	-1.29398	-0.53507
C	0.33581	-1.74342	0.59475
H	0.49543	-2.82481	0.74152
H	0.69031	-1.23829	1.50676
C	-1.14449	-1.47342	0.35846
H	-1.70320	-1.71048	1.28746
H	-1.51185	-2.15663	-0.42426
C	-2.75950	0.06290	-0.58672
H	-2.89175	1.08241	-0.97738
H	-3.50430	-0.10315	0.21632
H	-2.96124	-0.64559	-1.40366
Li	0.03935	-0.00248	-1.70046

ACE1_Li_udsdpbepb86_def2sv

26

N	-1.38861	-0.10694	-0.11015
C	-1.01710	0.93283	0.86366
H	-1.91338	1.42372	1.29090
H	-0.49256	0.47620	1.71877
C	-0.12709	1.98854	0.21049
H	-0.69503	2.55334	-0.54722
H	0.25372	2.70601	0.96165
O	0.95201	1.38607	-0.50227
C	1.97812	0.80822	0.29948
H	2.88865	1.43434	0.25876
H	1.65755	0.75965	1.35404
C	2.28173	-0.58430	-0.24787

H	2.80899	-0.50577	-1.21270
H	2.92495	-1.14867	0.45286
O	1.08049	-1.29229	-0.53381
C	0.33682	-1.74649	0.59545
H	0.49642	-2.83186	0.73227
H	0.69577	-1.24738	1.51160
C	-1.14519	-1.47388	0.36021
H	-1.70652	-1.71039	1.29116
H	-1.51225	-2.15842	-0.42502
C	-2.75881	0.06259	-0.59041
H	-2.88876	1.08627	-0.98066
H	-3.50830	-0.10600	0.21255
H	-2.95644	-0.64632	-1.41258
Li	0.03887	-0.00382	-1.69760

ACE1_Li_udsdpbepb86_def2tzvpp

26

N	-1.38014	-0.10609	-0.12796
C	-1.01017	0.92892	0.85218
H	-1.89890	1.41108	1.27191
H	-0.48857	0.47330	1.69362
C	-0.13165	1.99067	0.20406
H	-0.69610	2.54378	-0.54692
H	0.23641	2.69781	0.95236
O	0.96184	1.40426	-0.50971
C	1.95788	0.79963	0.31780
H	2.86187	1.41322	0.31411
H	1.60393	0.73716	1.34721
C	2.27877	-0.57764	-0.24175
H	2.80112	-0.48161	-1.19337
H	2.91877	-1.12812	0.45288

O	1.09135	-1.31172	-0.54122
C	0.33858	-1.73490	0.60073
H	0.49558	-2.80398	0.75910
H	0.69250	-1.21670	1.49264
C	-1.13832	-1.46947	0.35506
H	-1.69370	-1.69065	1.27804
H	-1.49930	-2.15235	-0.41709
C	-2.76635	0.05154	-0.56821
H	-2.91177	1.05994	-0.95575
H	-3.47745	-0.11371	0.25193
H	-2.97991	-0.65919	-1.36669
Li	0.03286	-0.00675	-1.71670

ACE1_Li_udsdpbepb86_def2tzvp

26

N	-1.38031	-0.10609	-0.12693
C	-1.01158	0.92982	0.85269
H	-1.90246	1.41366	1.27102
H	-0.49229	0.47424	1.69778
C	-0.13158	1.99063	0.20507
H	-0.69579	2.54640	-0.54692
H	0.23808	2.69873	0.95457
O	0.96103	1.40323	-0.50955
C	1.95952	0.80013	0.31644
H	2.86458	1.41579	0.30968
H	1.60822	0.73956	1.34871
C	2.27930	-0.57761	-0.24248
H	2.80220	-0.48327	-1.19621
H	2.92082	-1.12924	0.45297
O	1.09100	-1.31098	-0.54095
C	0.33874	-1.73571	0.60106

H	0.49613	-2.80710	0.75753
H	0.69462	-1.21955	1.49544
C	-1.13809	-1.46948	0.35611
H	-1.69470	-1.69136	1.28053
H	-1.50053	-2.15350	-0.41694
C	-2.76575	0.05097	-0.57008
H	-2.91197	1.06166	-0.95602
H	-3.48031	-0.11733	0.24876
H	-2.97759	-0.65846	-1.37241
Li	0.03117	-0.00603	-1.71628

ACE1_Li_udsdpbepb86_def2tzv

26

N	-1.39535	-0.10722	-0.12036
C	-1.02538	0.93998	0.87528
H	-1.91990	1.41411	1.30101
H	-0.49096	0.48391	1.71223
C	-0.15284	2.01756	0.22306
H	-0.70991	2.57220	-0.53307
H	0.23410	2.71671	0.97071
O	0.96480	1.40878	-0.52002
C	2.01334	0.80696	0.31650
H	2.90508	1.43752	0.28523
H	1.67089	0.74570	1.35236
C	2.32883	-0.57769	-0.25211
H	2.84036	-0.49748	-1.21132
H	2.95311	-1.14565	0.44403
O	1.09289	-1.31194	-0.55505
C	0.32568	-1.77015	0.61462
H	0.48894	-2.84268	0.74433
H	0.68692	-1.26189	1.51232

C	-1.15919	-1.49296	0.36764
H	-1.71387	-1.70119	1.29737
H	-1.53231	-2.17126	-0.40485
C	-2.78976	0.06221	-0.60107
H	-2.92007	1.07940	-0.97646
H	-3.52197	-0.11616	0.20088
H	-2.97984	-0.63677	-1.41825
Li	0.02375	-0.00534	-1.69897

ACE1_Li_uhse06_def2svpp

26

N	-1.39780	-0.10446	-0.09986
C	-1.01539	0.93634	0.86111
H	-1.90418	1.43554	1.29114
H	-0.49411	0.47859	1.71734
C	-0.11668	1.98424	0.21228
H	-0.67675	2.55834	-0.54358
H	0.26125	2.69842	0.96677
O	0.95736	1.38314	-0.49546
C	1.98533	0.80254	0.28965
H	2.89934	1.42136	0.23767
H	1.67987	0.76116	1.34918
C	2.27598	-0.59257	-0.25135
H	2.80078	-0.52356	-1.21769
H	2.92374	-1.15313	0.44703
O	1.07705	-1.29416	-0.52648
C	0.32923	-1.74365	0.59362
H	0.48353	-2.82846	0.73423
H	0.68583	-1.24698	1.51229
C	-1.14898	-1.46921	0.35848
H	-1.70917	-1.71910	1.28559

H	-1.51681	-2.14896	-0.42951
C	-2.75491	0.06903	-0.59498
H	-2.87927	1.08806	-0.99679
H	-3.51783	-0.08816	0.19715
H	-2.95090	-0.64401	-1.41313
Li	0.03220	0.00330	-1.69203

ACE1_Li_uhse06_def2svp

26

N	-1.39736	-0.10414	-0.10064
C	-1.01151	0.93511	0.86024
H	-1.89560	1.43206	1.29739
H	-0.48599	0.47701	1.71164
C	-0.11695	1.98408	0.21167
H	-0.67814	2.55862	-0.54063
H	0.25709	2.69829	0.96530
O	0.95850	1.38445	-0.49713
C	1.98268	0.79945	0.29107
H	2.89699	1.41539	0.25014
H	1.67270	0.75312	1.34750
C	2.27539	-0.59189	-0.25218
H	2.80136	-0.52038	-1.21585
H	2.92550	-1.14918	0.44368
O	1.07686	-1.29548	-0.52782
C	0.32800	-1.74041	0.59429
H	0.48381	-2.82180	0.74519
H	0.68089	-1.23809	1.50957
C	-1.14836	-1.46910	0.35738
H	-1.70733	-1.72101	1.28216
H	-1.51590	-2.14863	-0.42835
C	-2.75558	0.06945	-0.59290

H	-2.88230	1.08412	-0.99735
H	-3.51596	-0.08407	0.19835
H	-2.95549	-0.64430	-1.40580
Li	0.03166	0.00196	-1.69212

ACE1_Li_uhse06_def2sv

26

N	-1.39780	-0.10446	-0.09986
C	-1.01539	0.93634	0.86111
H	-1.90418	1.43554	1.29114
H	-0.49411	0.47859	1.71734
C	-0.11668	1.98424	0.21228
H	-0.67675	2.55834	-0.54358
H	0.26125	2.69842	0.96677
O	0.95736	1.38314	-0.49546
C	1.98533	0.80254	0.28965
H	2.89934	1.42136	0.23767
H	1.67987	0.76116	1.34918
C	2.27598	-0.59257	-0.25135
H	2.80078	-0.52356	-1.21769
H	2.92374	-1.15313	0.44703
O	1.07705	-1.29416	-0.52648
C	0.32923	-1.74365	0.59362
H	0.48353	-2.82846	0.73423
H	0.68583	-1.24698	1.51229
C	-1.14898	-1.46921	0.35848
H	-1.70917	-1.71910	1.28559
H	-1.51681	-2.14896	-0.42951
C	-2.75491	0.06903	-0.59498
H	-2.87927	1.08806	-0.99679
H	-3.51783	-0.08816	0.19715

H	-2.95090	-0.64401	-1.41313
Li	0.03220	0.00330	-1.69203

ACE1_Li_uhse06_def2tzvpp

26

N	-1.39017	-0.10371	-0.11502
C	-1.00898	0.93364	0.84970
H	-1.89120	1.42517	1.27319
H	-0.49101	0.47876	1.69512
C	-0.12217	1.98653	0.20512
H	-0.68009	2.55018	-0.54412
H	0.24274	2.69260	0.95706
O	0.96656	1.40189	-0.50379
C	1.96570	0.79484	0.30693
H	2.87443	1.40307	0.29313
H	1.62749	0.73903	1.34332
C	2.27343	-0.58416	-0.24597
H	2.79449	-0.49778	-1.20032
H	2.92023	-1.13154	0.44602
O	1.08828	-1.31315	-0.53366
C	0.33065	-1.73477	0.59747
H	0.48111	-2.80584	0.75551
H	0.68449	-1.22511	1.49586
C	-1.14169	-1.46449	0.35414
H	-1.69562	-1.70173	1.27477
H	-1.50498	-2.14332	-0.42124
C	-2.76169	0.05799	-0.57541
H	-2.90167	1.06391	-0.97174
H	-3.49145	-0.10025	0.23053
H	-2.97003	-0.65376	-1.37499
Li	0.02737	-0.00494	-1.69973

ACE1_Li_uhse06_def2tzvp

26

N	-1.39010	-0.10389	-0.11440
C	-1.00959	0.93400	0.85021
H	-1.89261	1.42613	1.27322
H	-0.49171	0.47916	1.69651
C	-0.12248	1.98677	0.20575
H	-0.68080	2.55102	-0.54384
H	0.24281	2.69299	0.95849
O	0.96599	1.40201	-0.50379
C	1.96608	0.79521	0.30633
H	2.87544	1.40389	0.29098
H	1.62886	0.73978	1.34376
C	2.27349	-0.58386	-0.24650
H	2.79418	-0.49750	-1.20193
H	2.92119	-1.13126	0.44587
O	1.08824	-1.31333	-0.53368
C	0.33095	-1.73525	0.59778
H	0.48124	-2.80724	0.75508
H	0.68538	-1.22581	1.49686
C	-1.14146	-1.46470	0.35495
H	-1.69524	-1.70225	1.27652
H	-1.50504	-2.14415	-0.42076
C	-2.76118	0.05763	-0.57676
H	-2.90049	1.06414	-0.97372
H	-3.49260	-0.10065	0.22864
H	-2.96834	-0.65458	-1.37717
Li	0.02657	-0.00485	-1.69950

ACE1_Li_uhse06_def2tzv

26

N	-1.40083	-0.10514	-0.11007
C	-1.01799	0.93995	0.86786
H	-1.90312	1.42284	1.29695
H	-0.48962	0.48602	1.70762
C	-0.14267	2.00463	0.21794
H	-0.69853	2.56091	-0.53604
H	0.23245	2.70981	0.96349
O	0.96832	1.40193	-0.51355
C	2.00928	0.79814	0.30913
H	2.90494	1.42056	0.27547
H	1.67890	0.74198	1.34770
C	2.31458	-0.58086	-0.25402
H	2.82289	-0.50041	-1.21357
H	2.95190	-1.14245	0.43280
O	1.08892	-1.31018	-0.54474
C	0.31867	-1.75704	0.61145
H	0.47873	-2.82702	0.75273
H	0.67100	-1.24859	1.51082
C	-1.15593	-1.48514	0.35750
H	-1.71698	-1.72044	1.27445
H	-1.51628	-2.15737	-0.42402
C	-2.77920	0.06771	-0.59647
H	-2.90745	1.07650	-0.98900
H	-3.52034	-0.09239	0.19881
H	-2.97702	-0.63849	-1.40330
Li	0.01866	-0.00463	-1.67950

ACE1_Li_um062x_def2svpp

26

N	-1.38724	-0.10595	-0.10666
C	-1.00861	0.93136	0.86470

H	-1.90049	1.41805	1.29930
H	-0.47619	0.47115	1.71160
C	-0.12184	1.98691	0.20807
H	-0.69144	2.54982	-0.54744
H	0.26125	2.70307	0.95567
O	0.95143	1.37994	-0.49937
C	1.97514	0.80428	0.29748
H	2.88299	1.42959	0.26029
H	1.65251	0.75137	1.35070
C	2.27630	-0.58765	-0.25163
H	2.80221	-0.50884	-1.21505
H	2.91496	-1.15566	0.44636
O	1.07238	-1.28395	-0.53116
C	0.33424	-1.74539	0.59195
H	0.49654	-2.82818	0.72387
H	0.69219	-1.24707	1.50843
C	-1.14791	-1.47361	0.35905
H	-1.70911	-1.70896	1.28700
H	-1.51543	-2.15433	-0.42680
C	-2.75255	0.07034	-0.59125
H	-2.87703	1.09278	-0.98102
H	-3.50154	-0.09485	0.20964
H	-2.95056	-0.63755	-1.41134
Li	0.03691	-0.00800	-1.68351

ACE1_Li_um062x_def2svp

26

N	-1.38675	-0.10533	-0.10800
C	-1.00660	0.93133	0.86321
H	-1.89498	1.41732	1.30011
H	-0.47425	0.47146	1.70779

C	-0.12169	1.98692	0.20760
H	-0.69116	2.55064	-0.54458
H	0.25845	2.70214	0.95452
O	0.95258	1.38101	-0.50067
C	1.97321	0.80176	0.29873
H	2.88077	1.42425	0.26973
H	1.64711	0.74590	1.34872
C	2.27591	-0.58716	-0.25188
H	2.80266	-0.50646	-1.21250
H	2.91597	-1.15215	0.44398
O	1.07272	-1.28554	-0.53213
C	0.33340	-1.74317	0.59261
H	0.49556	-2.82306	0.73131
H	0.68948	-1.24227	1.50615
C	-1.14709	-1.47280	0.35847
H	-1.70706	-1.70850	1.28442
H	-1.51473	-2.15332	-0.42453
C	-2.75357	0.06933	-0.58937
H	-2.88162	1.08807	-0.97948
H	-3.49896	-0.09513	0.21098
H	-2.95397	-0.63813	-1.40540
Li	0.03674	-0.00818	-1.68302

ACE1_Li_um062x_def2sv

26

N	-1.38724	-0.10595	-0.10666
C	-1.00861	0.93136	0.86470
H	-1.90049	1.41805	1.29930
H	-0.47619	0.47115	1.71160
C	-0.12184	1.98691	0.20807
H	-0.69144	2.54982	-0.54744

H 0.26125 2.70307 0.95567

O 0.95143 1.37994 -0.49937

C 1.97514 0.80428 0.29748

H 2.88299 1.42959 0.26029

H 1.65251 0.75137 1.35070

C 2.27630 -0.58765 -0.25163

H 2.80221 -0.50884 -1.21505

H 2.91496 -1.15566 0.44636

O 1.07238 -1.28395 -0.53116

C 0.33424 -1.74539 0.59195

H 0.49654 -2.82818 0.72387

H 0.69219 -1.24707 1.50843

C -1.14791 -1.47361 0.35905

H -1.70911 -1.70896 1.28700

H -1.51543 -2.15433 -0.42680

C -2.75255 0.07034 -0.59125

H -2.87703 1.09278 -0.98102

H -3.50154 -0.09485 0.20964

H -2.95056 -0.63755 -1.41134

Li 0.03691 -0.00800 -1.68351

ACE1_Li_um062x_def2tzvpp

26

N -1.38096 -0.10625 -0.11956

C -1.00891 0.93013 0.85670

H -1.89640 1.41171 1.27634

H -0.48720 0.47596 1.69814

C -0.12953 1.98843 0.20397

H -0.69505 2.54094 -0.54600

H 0.24231 2.69655 0.94774

O 0.95674 1.39456 -0.50433

C	1.96219	0.80011	0.31040
H	2.86243	1.41673	0.29553
H	1.62083	0.73871	1.34416
C	2.27616	-0.57883	-0.24921
H	2.79310	-0.48438	-1.20337
H	2.91614	-1.13398	0.43992
O	1.08366	-1.29998	-0.53597
C	0.33698	-1.73853	0.59744
H	0.49668	-2.80821	0.74163
H	0.69123	-1.23240	1.49622
C	-1.14055	-1.47132	0.35611
H	-1.69577	-1.69467	1.27672
H	-1.50204	-2.15050	-0.41774
C	-2.75827	0.05793	-0.57937
H	-2.89588	1.06812	-0.96230
H	-3.48013	-0.11102	0.22862
H	-2.96231	-0.64633	-1.38455
Li	0.03571	-0.00924	-1.68267

ACE1_Li_um062x_def2tzvp

26

N	-1.38084	-0.10639	-0.11901
C	-1.00971	0.93063	0.85710
H	-1.89812	1.41284	1.27580
H	-0.48880	0.47651	1.69976
C	-0.12964	1.98870	0.20464
H	-0.69514	2.54216	-0.54566
H	0.24279	2.69671	0.94922
O	0.95631	1.39466	-0.50432
C	1.96269	0.80052	0.30978
H	2.86348	1.41763	0.29325

H	1.62227	0.74001	1.34453
C	2.27619	-0.57866	-0.24959
H	2.79296	-0.48468	-1.20470
H	2.91664	-1.13409	0.44001
O	1.08354	-1.30008	-0.53600
C	0.33726	-1.73924	0.59764
H	0.49677	-2.80984	0.74062
H	0.69257	-1.23381	1.49708
C	-1.14037	-1.47150	0.35704
H	-1.69543	-1.69476	1.27857
H	-1.50242	-2.15132	-0.41693
C	-2.75782	0.05758	-0.58071
H	-2.89484	1.06844	-0.96387
H	-3.48109	-0.11152	0.22686
H	-2.96081	-0.64683	-1.38689
Li	0.03486	-0.00918	-1.68248

ACE1_Li_um062x_def2tzv

26

N	-1.39041	-0.10993	-0.11199
C	-1.01676	0.93364	0.87573
H	-1.90617	1.40199	1.30604
H	-0.47801	0.48117	1.70720
C	-0.15401	2.00579	0.21483
H	-0.71961	2.54744	-0.53990
H	0.22639	2.71596	0.94927
O	0.95347	1.39348	-0.51241
C	2.00008	0.80598	0.31258
H	2.88494	1.43900	0.28193
H	1.66428	0.74008	1.34723
C	2.31610	-0.57140	-0.25863

H	2.81971	-0.48044	-1.21748
H	2.94891	-1.13874	0.42369
O	1.08610	-1.29428	-0.54567
C	0.32905	-1.75891	0.61053
H	0.50140	-2.82579	0.74044
H	0.67967	-1.25072	1.50875
C	-1.15105	-1.49372	0.35852
H	-1.71381	-1.71904	1.27320
H	-1.50670	-2.16496	-0.42337
C	-2.77265	0.06547	-0.60023
H	-2.89829	1.07855	-0.97765
H	-3.50662	-0.10679	0.19530
H	-2.96441	-0.63134	-1.41390
Li	0.02672	-0.01051	-1.66405

ACE1_Li_um06_def2svpp

26

N	-1.40292	-0.10622	-0.09422
C	-1.01531	0.93512	0.85897
H	-1.90531	1.43539	1.29384
H	-0.49487	0.48080	1.72174
C	-0.12716	1.98043	0.20240
H	-0.69816	2.53906	-0.56180
H	0.23899	2.71278	0.95000
O	0.95773	1.39025	-0.49421
C	1.97399	0.81032	0.30090
H	2.89122	1.43044	0.26778
H	1.65897	0.77028	1.36142
C	2.27715	-0.58054	-0.23263
H	2.81433	-0.50919	-1.19529
H	2.92721	-1.13475	0.47318

O	1.09162	-1.29794	-0.51633
C	0.33083	-1.74217	0.59387
H	0.48260	-2.82905	0.74219
H	0.68228	-1.24788	1.52006
C	-1.14234	-1.47017	0.35033
H	-1.71128	-1.73554	1.27257
H	-1.50295	-2.14807	-0.44789
C	-2.76107	0.06511	-0.57642
H	-2.89393	1.09001	-0.96808
H	-3.52020	-0.10168	0.22221
H	-2.96530	-0.64127	-1.40133
Li	0.06851	-0.03165	-1.83045

ACE1_Li_um06_def2svp

26

N	-1.40161	-0.10709	-0.09533
C	-1.01208	0.93351	0.85773
H	-1.89689	1.42965	1.30045
H	-0.48525	0.48159	1.71483
C	-0.13023	1.98070	0.20028
H	-0.70346	2.53729	-0.56004
H	0.22912	2.71491	0.94514
O	0.95723	1.39261	-0.49587
C	1.96871	0.80853	0.30386
H	2.88480	1.42598	0.28561
H	1.64744	0.76078	1.35975
C	2.27692	-0.57729	-0.23286
H	2.81625	-0.50115	-1.19102
H	2.93033	-1.12608	0.46968
O	1.09367	-1.29928	-0.51835
C	0.33177	-1.73921	0.59411

H	0.48512	-2.82150	0.75361
H	0.67883	-1.23901	1.51601
C	-1.13949	-1.47107	0.34891
H	-1.70734	-1.73876	1.26751
H	-1.49936	-2.14883	-0.44600
C	-2.76188	0.06277	-0.57305
H	-2.89947	1.08345	-0.96375
H	-3.51653	-0.10375	0.22456
H	-2.96987	-0.64150	-1.39397
Li	0.06931	-0.03256	-1.83170

ACE1_Li_um06_def2sv

26

N	-1.40292	-0.10622	-0.09422
C	-1.01531	0.93512	0.85897
H	-1.90531	1.43539	1.29384
H	-0.49487	0.48080	1.72174
C	-0.12716	1.98043	0.20240
H	-0.69816	2.53906	-0.56180
H	0.23899	2.71278	0.95000
O	0.95773	1.39025	-0.49421
C	1.97399	0.81032	0.30090
H	2.89122	1.43044	0.26778
H	1.65897	0.77028	1.36142
C	2.27715	-0.58054	-0.23263
H	2.81433	-0.50919	-1.19529
H	2.92721	-1.13475	0.47318
O	1.09162	-1.29794	-0.51633
C	0.33083	-1.74217	0.59387
H	0.48260	-2.82905	0.74219
H	0.68228	-1.24788	1.52006

C	-1.14234	-1.47017	0.35033
H	-1.71128	-1.73554	1.27257
H	-1.50295	-2.14807	-0.44789
C	-2.76107	0.06511	-0.57642
H	-2.89393	1.09001	-0.96808
H	-3.52020	-0.10168	0.22221
H	-2.96530	-0.64127	-1.40133
Li	0.06851	-0.03165	-1.83045

ACE1_Li_um06_def2tzvpp

26

N	-1.39043	-0.10655	-0.10372
C	-1.00831	0.93348	0.85263
H	-1.89129	1.42451	1.27639
H	-0.49021	0.48656	1.70295
C	-0.13246	1.98256	0.19655
H	-0.70327	2.52571	-0.56001
H	0.21557	2.70976	0.93788
O	0.96388	1.40960	-0.49814
C	1.95031	0.80327	0.31700
H	2.86110	1.40985	0.32148
H	1.60668	0.74409	1.35275
C	2.26874	-0.57105	-0.23250
H	2.79515	-0.47762	-1.18397
H	2.92432	-1.10769	0.46103
O	1.09974	-1.31560	-0.52148
C	0.33698	-1.73395	0.59957
H	0.48545	-2.80493	0.76488
H	0.68442	-1.22792	1.50387
C	-1.13144	-1.46654	0.35240
H	-1.69203	-1.71684	1.26692

H	-1.48820	-2.14113	-0.43102
C	-2.76004	0.05195	-0.56093
H	-2.90598	1.06096	-0.94835
H	-3.48994	-0.11634	0.24395
H	-2.96918	-0.65275	-1.36651
Li	0.04623	-0.04024	-1.86921

ACE1_Li_um06_def2tzvp

26

N	-1.39034	-0.10665	-0.10363
C	-1.00856	0.93342	0.85250
H	-1.89293	1.42499	1.27594
H	-0.49052	0.48589	1.70392
C	-0.13259	1.98211	0.19665
H	-0.70427	2.52590	-0.56049
H	0.21568	2.70993	0.93911
O	0.96360	1.40937	-0.49828
C	1.95050	0.80366	0.31673
H	2.86238	1.41087	0.32051
H	1.60695	0.74476	1.35374
C	2.26861	-0.57072	-0.23200
H	2.79628	-0.47774	-1.18417
H	2.92433	-1.10777	0.46308
O	1.09974	-1.31529	-0.52151
C	0.33694	-1.73431	0.59918
H	0.48508	-2.80667	0.76422
H	0.68447	-1.22831	1.50482
C	-1.13111	-1.46650	0.35214
H	-1.69243	-1.71712	1.26767
H	-1.48851	-2.14158	-0.43219
C	-2.75956	0.05212	-0.56079

H	-2.90499	1.06250	-0.94776
H	-3.48987	-0.11655	0.24527
H	-2.96909	-0.65282	-1.36748
Li	0.04590	-0.04032	-1.86961

ACE1_Li_um06_def2tzv

26

N	-1.39956	-0.10796	-0.10350
C	-1.01717	0.93886	0.86930
H	-1.90417	1.42157	1.29514
H	-0.49017	0.48921	1.71281
C	-0.14809	2.00137	0.21047
H	-0.71495	2.54037	-0.54939
H	0.21386	2.72131	0.94942
O	0.96715	1.40954	-0.50783
C	1.99881	0.80608	0.31222
H	2.89826	1.42391	0.28523
H	1.66983	0.74795	1.35228
C	2.30804	-0.57269	-0.24945
H	2.81613	-0.48615	-1.20903
H	2.95389	-1.12549	0.43747
O	1.09733	-1.31380	-0.53624
C	0.32697	-1.75565	0.61206
H	0.48488	-2.82529	0.76057
H	0.67432	-1.24646	1.51404
C	-1.14669	-1.48675	0.35592
H	-1.71156	-1.72817	1.26944
H	-1.50131	-2.15659	-0.43138
C	-2.77757	0.06373	-0.58571
H	-2.90583	1.07562	-0.97186
H	-3.51494	-0.10081	0.21292

H	-2.97687	-0.63975	-1.39458
Li	0.00795	-0.03036	-1.76831

ACE1_Li_ump2_def2svpp

26

N	-1.38838	-0.10439	-0.11754
C	-1.01645	0.93261	0.85888
H	-1.91261	1.42587	1.28467
H	-0.49552	0.47589	1.71590
C	-0.12446	1.98720	0.20866
H	-0.69183	2.55115	-0.54982
H	0.25397	2.70504	0.96103
O	0.95796	1.38562	-0.50312
C	1.97715	0.80449	0.30781
H	2.88940	1.42908	0.27659
H	1.64837	0.75363	1.35920
C	2.28243	-0.58591	-0.24131
H	2.81324	-0.50383	-1.20365
H	2.92321	-1.15224	0.46030
O	1.08095	-1.29460	-0.53503
C	0.33500	-1.74256	0.59692
H	0.49278	-2.82778	0.73950
H	0.69264	-1.24079	1.51156
C	-1.14551	-1.46969	0.35800
H	-1.70846	-1.70224	1.28941
H	-1.51017	-2.15761	-0.42522
C	-2.76727	0.06084	-0.57846
H	-2.90556	1.08479	-0.96314
H	-3.50363	-0.11329	0.23461
H	-2.97260	-0.64640	-1.39932
Li	0.04963	-0.00017	-1.74220

ACE1_Li_ump2_def2svp

26

N	-1.38774	-0.10494	-0.11901
C	-1.01174	0.92962	0.85590
H	-1.90126	1.41992	1.28876
H	-0.48488	0.47204	1.70455
C	-0.12442	1.98602	0.20713
H	-0.69256	2.54897	-0.54657
H	0.24857	2.69993	0.96034
O	0.95872	1.38806	-0.50406
C	1.96980	0.80200	0.31086
H	2.88158	1.42099	0.29596
H	1.63161	0.74267	1.35546
C	2.27973	-0.58440	-0.23896
H	2.81353	-0.49891	-1.19538
H	2.91908	-1.14384	0.46343
O	1.08263	-1.29633	-0.53612
C	0.33543	-1.73806	0.59538
H	0.49248	-2.81785	0.74998
H	0.68730	-1.22888	1.50447
C	-1.14341	-1.46852	0.35665
H	-1.70167	-1.69929	1.28655
H	-1.50931	-2.15503	-0.42224
C	-2.76684	0.06024	-0.57221
H	-2.90783	1.07869	-0.95791
H	-3.49587	-0.10953	0.24227
H	-2.97821	-0.64709	-1.38545
Li	0.04986	-0.00116	-1.75937

ACE1_Li_ump2_def2sv

26

N	-1.38838	-0.10439	-0.11754
C	-1.01645	0.93261	0.85888
H	-1.91261	1.42587	1.28467
H	-0.49552	0.47589	1.71590
C	-0.12446	1.98720	0.20866
H	-0.69183	2.55115	-0.54982
H	0.25397	2.70504	0.96103
O	0.95796	1.38562	-0.50312
C	1.97715	0.80449	0.30781
H	2.88940	1.42908	0.27659
H	1.64837	0.75363	1.35920
C	2.28243	-0.58591	-0.24131
H	2.81324	-0.50383	-1.20365
H	2.92321	-1.15224	0.46030
O	1.08095	-1.29460	-0.53503
C	0.33500	-1.74256	0.59692
H	0.49278	-2.82778	0.73950
H	0.69264	-1.24079	1.51156
C	-1.14551	-1.46969	0.35800
H	-1.70846	-1.70224	1.28941
H	-1.51017	-2.15761	-0.42522
C	-2.76727	0.06084	-0.57846
H	-2.90556	1.08479	-0.96314
H	-3.50363	-0.11329	0.23461
H	-2.97260	-0.64640	-1.39932
Li	0.04963	-0.00017	-1.74220

ACE1_Li_ump2_def2tzvpp

26

N	-1.37615	-0.10269	-0.13683
C	-1.00328	0.92673	0.84646

H	-1.88978	1.40713	1.26891
H	-0.48105	0.46828	1.68375
C	-0.12779	1.98914	0.20019
H	-0.69309	2.53875	-0.54959
H	0.23704	2.69487	0.94891
O	0.96865	1.40562	-0.51281
C	1.95003	0.79515	0.32860
H	2.85468	1.40410	0.34218
H	1.58033	0.72899	1.35017
C	2.27645	-0.57833	-0.23115
H	2.80344	-0.47786	-1.17715
H	2.91089	-1.12809	0.46619
O	1.09141	-1.31474	-0.54098
C	0.33617	-1.72793	0.60319
H	0.49115	-2.79388	0.77079
H	0.68737	-1.20297	1.48985
C	-1.13824	-1.46392	0.35238
H	-1.69498	-1.67813	1.27427
H	-1.49697	-2.14863	-0.41660
C	-2.77216	0.05260	-0.54878
H	-2.92598	1.05964	-0.93044
H	-3.46263	-0.11521	0.28543
H	-2.99915	-0.65688	-1.34166
Li	0.03474	-0.02300	-1.79405

ACE1_Li_ump2_def2tzvp

26

N	-1.37645	-0.10273	-0.13490
C	-1.00564	0.92838	0.84741
H	-1.89556	1.41145	1.26756
H	-0.48762	0.47036	1.69092

C	-0.12779	1.98920	0.20167
H	-0.69249	2.54270	-0.55010
H	0.23923	2.69722	0.95169
O	0.96759	1.40429	-0.51233
C	1.95297	0.79597	0.32660
H	2.85935	1.40828	0.33512
H	1.58787	0.73343	1.35284
C	2.27721	-0.57828	-0.23257
H	2.80461	-0.48053	-1.18214
H	2.91448	-1.13043	0.46516
O	1.09067	-1.31369	-0.54051
C	0.33623	-1.72969	0.60374
H	0.49199	-2.79939	0.76774
H	0.69114	-1.20881	1.49447
C	-1.13819	-1.46397	0.35439
H	-1.69675	-1.67918	1.27863
H	-1.49978	-2.15057	-0.41559
C	-2.77111	0.05198	-0.55235
H	-2.92577	1.06216	-0.93261
H	-3.46769	-0.11928	0.27957
H	-2.99582	-0.65600	-1.35067
Li	0.03326	-0.02288	-1.79297

ACE1_Li_ump2_def2tzv

26

N	-1.39959	-0.10139	-0.12707
C	-1.02392	0.94696	0.87546
H	-1.91853	1.42326	1.30309
H	-0.48757	0.48512	1.70946
C	-0.14586	2.02788	0.22657
H	-0.69655	2.58590	-0.53202

H	0.24558	2.71881	0.98080
O	0.98146	1.41909	-0.52427
C	2.02758	0.80271	0.32327
H	2.92423	1.42767	0.29645
H	1.67571	0.74210	1.35674
C	2.33779	-0.58769	-0.24591
H	2.85366	-0.51470	-1.20356
H	2.94869	-1.16123	0.45870
O	1.09139	-1.32232	-0.56003
C	0.31731	-1.78059	0.61786
H	0.47369	-2.85547	0.74264
H	0.68588	-1.27494	1.51504
C	-1.17094	-1.49111	0.37572
H	-1.71813	-1.67866	1.31593
H	-1.55892	-2.17208	-0.38786
C	-2.80853	0.06894	-0.59533
H	-2.93995	1.09010	-0.96027
H	-3.53019	-0.11827	0.21468
H	-3.00029	-0.62539	-1.41628
Li	0.01883	-0.01974	-1.76513

ACE1_Li_upbe0_def2svpp

26

N	-1.39721	-0.10431	-0.09907
C	-1.01547	0.93715	0.86105
H	-1.90473	1.43729	1.28909
H	-0.49608	0.47991	1.71857
C	-0.11560	1.98413	0.21227
H	-0.67539	2.55926	-0.54309
H	0.26254	2.69778	0.96732
O	0.95725	1.38307	-0.49555

C	1.98493	0.80282	0.28891
H	2.89930	1.42122	0.23577
H	1.68051	0.76184	1.34867
C	2.27484	-0.59262	-0.25162
H	2.79940	-0.52407	-1.21822
H	2.92283	-1.15315	0.44668
O	1.07618	-1.29361	-0.52611
C	0.32955	-1.74430	0.59312
H	0.48278	-2.82972	0.73123
H	0.68739	-1.25043	1.51276
C	-1.14905	-1.46858	0.36022
H	-1.70724	-1.71753	1.28874
H	-1.51852	-2.14861	-0.42677
C	-2.75344	0.06943	-0.59606
H	-2.87647	1.08804	-0.99946
H	-3.51759	-0.08615	0.19520
H	-2.94880	-0.64450	-1.41366
Li	0.02951	-0.00163	-1.69112

ACE1_Li_upbe0_def2svp

26

N	-1.39681	-0.10389	-0.09992
C	-1.01200	0.93599	0.86029
H	-1.89663	1.43416	1.29487
H	-0.48888	0.47871	1.71342
C	-0.11603	1.98383	0.21171
H	-0.67687	2.55927	-0.54020
H	0.25847	2.69762	0.96560
O	0.95800	1.38387	-0.49711
C	1.98262	0.80006	0.28999
H	2.89718	1.41560	0.24645

H	1.67493	0.75527	1.34705
C	2.27439	-0.59195	-0.25225
H	2.80031	-0.52119	-1.21607
H	2.92463	-1.14908	0.44369
O	1.07624	-1.29483	-0.52729
C	0.32841	-1.74103	0.59401
H	0.48297	-2.82310	0.74197
H	0.68263	-1.24187	1.51035
C	-1.14816	-1.46841	0.35886
H	-1.70619	-1.71953	1.28436
H	-1.51699	-2.14814	-0.42607
C	-2.75430	0.06943	-0.59395
H	-2.88030	1.08385	-0.99929
H	-3.51579	-0.08352	0.19628
H	-2.95312	-0.64470	-1.40682
Li	0.02928	-0.00196	-1.69095

ACE1_Li_upbe0_def2sv

26

N	-1.39721	-0.10431	-0.09907
C	-1.01547	0.93715	0.86105
H	-1.90473	1.43729	1.28909
H	-0.49608	0.47991	1.71857
C	-0.11560	1.98413	0.21227
H	-0.67539	2.55926	-0.54309
H	0.26254	2.69778	0.96732
O	0.95725	1.38307	-0.49555
C	1.98493	0.80282	0.28891
H	2.89930	1.42122	0.23577
H	1.68051	0.76184	1.34867
C	2.27484	-0.59262	-0.25162

H	2.79940	-0.52407	-1.21822
H	2.92283	-1.15315	0.44668
O	1.07618	-1.29361	-0.52611
C	0.32955	-1.74430	0.59312
H	0.48278	-2.82972	0.73123
H	0.68739	-1.25043	1.51276
C	-1.14905	-1.46858	0.36022
H	-1.70724	-1.71753	1.28874
H	-1.51852	-2.14861	-0.42677
C	-2.75344	0.06943	-0.59606
H	-2.87647	1.08804	-0.99946
H	-3.51759	-0.08615	0.19520
H	-2.94880	-0.64450	-1.41366
Li	0.02951	-0.00163	-1.69112

ACE1_Li_upbe0_def2tzvpp

26

N	-1.38919	-0.10342	-0.11550
C	-1.00916	0.93410	0.84925
H	-1.89212	1.42661	1.27059
H	-0.49317	0.47926	1.69609
C	-0.12081	1.98630	0.20511
H	-0.67874	2.55126	-0.54357
H	0.24469	2.69168	0.95784
O	0.96631	1.40152	-0.50421
C	1.96463	0.79495	0.30636
H	2.87406	1.40270	0.29200
H	1.62680	0.73978	1.34310
C	2.27205	-0.58457	-0.24609
H	2.79367	-0.49816	-1.20051
H	2.91900	-1.13182	0.44634

O 1.08764 -1.31308 -0.53392

C 0.33147 -1.73446 0.59711

H 0.48119 -2.80609 0.75450

H 0.68633 -1.22565 1.49580

C -1.14127 -1.46363 0.35532

H -1.69438 -1.69927 1.27705

H -1.50584 -2.14332 -0.41905

C -2.76087 0.05740 -0.57568

H -2.90100 1.06297 -0.97356

H -3.49047 -0.09987 0.23083

H -2.96910 -0.65575 -1.37441

Li 0.02519 -0.00612 -1.69595

ACE1_Li_upbe0_def2tzvp

26

N -1.38913 -0.10359 -0.11491

C -1.00974 0.93447 0.84970

H -1.89348 1.42762 1.27057

H -0.49388 0.47968 1.69745

C -0.12106 1.98653 0.20570

H -0.67937 2.55208 -0.54338

H 0.24476 2.69210 0.95925

O 0.96585 1.40167 -0.50418

C 1.96500 0.79523 0.30583

H 2.87509 1.40343 0.29010

H 1.62804 0.74035 1.34357

C 2.27209 -0.58431 -0.24664

H 2.79329 -0.49786 -1.20217

H 2.91998 -1.13163 0.44610

O 1.08759 -1.31327 -0.53397

C 0.33174 -1.73494 0.59740

H	0.48128	-2.80749	0.75406
H	0.68721	-1.22637	1.49677
C	-1.14107	-1.46382	0.35614
H	-1.69401	-1.69966	1.27884
H	-1.50604	-2.14414	-0.41851
C	-2.76038	0.05706	-0.57697
H	-2.89986	1.06320	-0.97555
H	-3.49158	-0.10015	0.22907
H	-2.96753	-0.65661	-1.37646
Li	0.02435	-0.00599	-1.69570

ACE1_Li_upbe0_def2tzv

26

N	-1.39968	-0.10508	-0.11033
C	-1.01880	0.94017	0.86788
H	-1.90481	1.42324	1.29561
H	-0.49144	0.48615	1.70850
C	-0.14261	2.00442	0.21785
H	-0.69910	2.56191	-0.53528
H	0.23373	2.70898	0.96391
O	0.96602	1.40121	-0.51420
C	2.00739	0.79998	0.30750
H	2.90337	1.42253	0.27133
H	1.67909	0.74580	1.34715
C	2.31340	-0.58025	-0.25329
H	2.82320	-0.50078	-1.21259
H	2.95085	-1.14027	0.43530
O	1.08950	-1.31009	-0.54409
C	0.32018	-1.75656	0.61157
H	0.47968	-2.82706	0.75256
H	0.67293	-1.24848	1.51126

C	-1.15466	-1.48468	0.35803
H	-1.71617	-1.71989	1.27500
H	-1.51495	-2.15726	-0.42373
C	-2.77804	0.06615	-0.59695
H	-2.90727	1.07504	-0.98979
H	-3.51935	-0.09475	0.19837
H	-2.97488	-0.64057	-1.40403
Li	0.01923	-0.00782	-1.67679

ACE1_Li_upbe_def2svpp

26

N	-1.40941	-0.10319	-0.10289
C	-1.02308	0.94700	0.86589
H	-1.91846	1.45312	1.29768
H	-0.50115	0.48442	1.73010
C	-0.11593	2.00162	0.21497
H	-0.67715	2.57978	-0.54995
H	0.26090	2.72094	0.97881
O	0.97297	1.40302	-0.50413
C	2.00324	0.80531	0.29477
H	2.92943	1.42380	0.24810
H	1.69001	0.76313	1.36102
C	2.29381	-0.59874	-0.25164
H	2.81869	-0.52952	-1.22816
H	2.95015	-1.16182	0.45166
O	1.08623	-1.31446	-0.53420
C	0.32687	-1.75886	0.60114
H	0.47703	-2.85324	0.74679
H	0.68851	-1.25652	1.52527
C	-1.15965	-1.47897	0.36195
H	-1.72456	-1.73238	1.29682

H	-1.53180	-2.16210	-0.43333
C	-2.77848	0.07118	-0.59817
H	-2.90196	1.09630	-1.00808
H	-3.54759	-0.08188	0.20210
H	-2.97798	-0.65164	-1.41858
Li	0.02582	-0.00325	-1.70227

ACE1_Li_upbe_def2svp

26

N	-1.40889	-0.10279	-0.10408
C	-1.01864	0.94532	0.86497
H	-1.90857	1.44827	1.30561
H	-0.49140	0.48262	1.72351
C	-0.11644	2.00146	0.21408
H	-0.67878	2.57957	-0.54713
H	0.25616	2.72125	0.97617
O	0.97399	1.40410	-0.50585
C	2.00012	0.80198	0.29661
H	2.92598	1.41778	0.26224
H	1.68208	0.75450	1.35926
C	2.29330	-0.59797	-0.25233
H	2.81948	-0.52596	-1.22569
H	2.95222	-1.15761	0.44771
O	1.08631	-1.31585	-0.53564
C	0.32553	-1.75510	0.60198
H	0.47809	-2.84529	0.75925
H	0.68245	-1.24601	1.52204
C	-1.15890	-1.47901	0.36013
H	-1.72339	-1.73474	1.29161
H	-1.53062	-2.16175	-0.43260
C	-2.77949	0.07154	-0.59534

H	-2.90610	1.09182	-1.00774
H	-3.54510	-0.07799	0.20420
H	-2.98351	-0.65177	-1.40994
Li	0.02599	-0.00348	-1.70288

ACE1_Li_upbe_def2sv

26

N	-1.40941	-0.10319	-0.10289
C	-1.02308	0.94700	0.86589
H	-1.91846	1.45312	1.29768
H	-0.50115	0.48442	1.73010
C	-0.11593	2.00162	0.21497
H	-0.67715	2.57978	-0.54995
H	0.26090	2.72094	0.97881
O	0.97297	1.40302	-0.50413
C	2.00324	0.80531	0.29477
H	2.92943	1.42380	0.24810
H	1.69001	0.76313	1.36102
C	2.29381	-0.59874	-0.25164
H	2.81869	-0.52952	-1.22816
H	2.95015	-1.16182	0.45166
O	1.08623	-1.31446	-0.53420
C	0.32687	-1.75886	0.60114
H	0.47703	-2.85324	0.74679
H	0.68851	-1.25652	1.52527
C	-1.15965	-1.47897	0.36195
H	-1.72456	-1.73238	1.29682
H	-1.53180	-2.16210	-0.43333
C	-2.77848	0.07118	-0.59817
H	-2.90196	1.09630	-1.00808
H	-3.54759	-0.08188	0.20210

H -2.97798 -0.65164 -1.41858

Li 0.02582 -0.00325 -1.70227

ACE1_Li_upbe_def2tzvpp

26

N -1.40206 -0.10143 -0.11956

C -1.01520 0.94479 0.85381

H -1.90306 1.44205 1.28013

H -0.49574 0.48401 1.70521

C -0.12055 2.00469 0.20796

H -0.67755 2.57243 -0.55023

H 0.24390 2.71466 0.96840

O 0.98504 1.42238 -0.51235

C 1.98458 0.79532 0.31432

H 2.90390 1.40303 0.30771

H 1.63517 0.73902 1.35498

C 2.29211 -0.59140 -0.24475

H 2.81391 -0.50561 -1.20775

H 2.94469 -1.14279 0.45188

O 1.09723 -1.33463 -0.54213

C 0.32655 -1.74974 0.60586

H 0.47402 -2.82888 0.76881

H 0.68615 -1.23266 1.50703

C -1.15330 -1.47370 0.35783

H -1.71186 -1.71150 1.28586

H -1.52193 -2.15601 -0.42290

C -2.78800 0.06078 -0.57573

H -2.92780 1.07156 -0.98012

H -3.51978 -0.09278 0.24047

H -3.00113 -0.66096 -1.37543

Li 0.01872 -0.01068 -1.72572

ACE1_Li_upbe_def2tzvp

26

N 1.40201 -0.10156 0.11885

C 1.01562 0.94513 -0.85444

H 1.90417 1.44293 -1.28058

H 0.49605 0.48428 -1.70652

C 0.12071 2.00489 -0.20860

H 0.67816 2.57332 0.54982

H -0.24430 2.71500 -0.96976

O -0.98453 1.42233 0.51243

C -1.98503 0.79559 -0.31360

H -2.90499 1.40376 -0.30562

H -1.63659 0.73949 -1.35530

C -2.29216 -0.59130 0.24536

H -2.81389 -0.50559 1.20929

H -2.94551 -1.14274 -0.45176

O -1.09696 -1.33460 0.54225

C -0.32674 -1.75023 -0.60608

H -0.47416 -2.83026 -0.76836

H -0.68683 -1.23332 -1.50794

C 1.15320 -1.47395 -0.35869

H 1.71166 -1.71195 -1.28764

H 1.52228 -2.15680 0.42235

C 2.78749 0.06065 0.57694

H 2.92663 1.07204 0.98191

H 3.52104 -0.09292 -0.23864

H 2.99958 -0.66144 1.37753

Li -0.01798 -0.01049 1.72554

ACE1_Li_upbe_def2tzv

26

N	-1.41280	-0.10256	-0.11715
C	-1.02402	0.95213	0.87135
H	-1.91538	1.44032	1.30411
H	-0.49392	0.49137	1.71685
C	-0.14221	2.02492	0.22047
H	-0.69788	2.58421	-0.54340
H	0.23240	2.73443	0.97481
O	0.98922	1.42656	-0.52629
C	2.02760	0.79804	0.32017
H	2.93493	1.42000	0.29841
H	1.68020	0.74112	1.36139
C	2.33558	-0.58701	-0.24989
H	2.84594	-0.50541	-1.21781
H	2.97827	-1.15299	0.44235
O	1.10024	-1.33575	-0.55660
C	0.31470	-1.77239	0.62188
H	0.47129	-2.85069	0.77044
H	0.67396	-1.25384	1.52230
C	-1.16689	-1.49504	0.36083
H	-1.73481	-1.73114	1.28459
H	-1.53105	-2.17126	-0.42700
C	-2.80987	0.06891	-0.59278
H	-2.94056	1.08249	-0.99372
H	-3.54912	-0.08645	0.21723
H	-3.01570	-0.64868	-1.39829
Li	0.00872	-0.01315	-1.71376

ACE1_Li_uscs-mp2_def2svpp

26

N	-1.39103	-0.10434	-0.11642
C	-1.02049	0.93731	0.86129

H	-1.91822	1.43335	1.28124
H	-0.50364	0.48203	1.72208
C	-0.12371	1.99155	0.21011
H	-0.68912	2.55787	-0.54891
H	0.25789	2.70797	0.96275
O	0.95850	1.38681	-0.50391
C	1.98427	0.80636	0.30366
H	2.89605	1.43182	0.26249
H	1.66265	0.75951	1.35782
C	2.28657	-0.58807	-0.24472
H	2.81593	-0.50857	-1.20877
H	2.92685	-1.15547	0.45712
O	1.08164	-1.29614	-0.53603
C	0.33545	-1.74823	0.59735
H	0.49295	-2.83475	0.73356
H	0.69532	-1.25071	1.51379
C	-1.14805	-1.47292	0.36042
H	-1.71005	-1.70445	1.29280
H	-1.51406	-2.16088	-0.42287
C	-2.77056	0.06042	-0.58529
H	-2.90743	1.08482	-0.97188
H	-3.51175	-0.11365	0.22400
H	-2.97115	-0.64765	-1.40774
Li	0.04433	0.00175	-1.70997

ACE1_Li_uscs-mp2_def2svp

26

N	-1.39045	-0.10457	-0.11798
C	-1.01686	0.93524	0.85822
H	-1.90781	1.42924	1.28203
H	-0.49621	0.48002	1.71120

C	-0.12312	1.99027	0.20885
H	-0.68776	2.55592	-0.54488
H	0.25355	2.70125	0.96212
O	0.95909	1.38790	-0.50411
C	1.97868	0.80394	0.30586
H	2.88924	1.42326	0.27678
H	1.65063	0.75034	1.35330
C	2.28399	-0.58709	-0.24296
H	2.81472	-0.50487	-1.20100
H	2.92219	-1.14758	0.45873
O	1.08268	-1.29740	-0.53645
C	0.33563	-1.74383	0.59648
H	0.49152	-2.82442	0.74275
H	0.69015	-1.24052	1.50690
C	-1.14609	-1.47127	0.35954
H	-1.70301	-1.70038	1.28965
H	-1.51247	-2.15742	-0.41881
C	-2.77024	0.05960	-0.57982
H	-2.90928	1.07750	-0.96731
H	-3.50348	-0.10983	0.23016
H	-2.97579	-0.64858	-1.39358
Li	0.04362	0.00099	-1.72490

ACE1_Li_uscs-mp2_def2sv

26

N	-1.39103	-0.10434	-0.11642
C	-1.02049	0.93731	0.86129
H	-1.91822	1.43335	1.28124
H	-0.50364	0.48203	1.72208
C	-0.12371	1.99155	0.21011
H	-0.68912	2.55787	-0.54891

H	0.25789	2.70797	0.96275
O	0.95850	1.38681	-0.50391
C	1.98427	0.80636	0.30366
H	2.89605	1.43182	0.26249
H	1.66265	0.75951	1.35782
C	2.28657	-0.58807	-0.24472
H	2.81593	-0.50857	-1.20877
H	2.92685	-1.15547	0.45712
O	1.08164	-1.29614	-0.53603
C	0.33545	-1.74823	0.59735
H	0.49295	-2.83475	0.73356
H	0.69532	-1.25071	1.51379
C	-1.14805	-1.47292	0.36042
H	-1.71005	-1.70445	1.29280
H	-1.51406	-2.16088	-0.42287
C	-2.77056	0.06042	-0.58529
H	-2.90743	1.08482	-0.97188
H	-3.51175	-0.11365	0.22400
H	-2.97115	-0.64765	-1.40774
Li	0.04433	0.00175	-1.70997

ACE1_Li_uscs-mp2_def2tzvpp

26

N	-1.37976	-0.10139	-0.13838
C	-1.00887	0.93236	0.84710
H	-1.89511	1.41716	1.25948
H	-0.49499	0.47559	1.68803
C	-0.12363	1.99180	0.20272
H	-0.68314	2.54619	-0.54559
H	0.24604	2.69186	0.95168
O	0.96985	1.40192	-0.51207

C	1.96125	0.79548	0.32330
H	2.86411	1.40395	0.31994
H	1.60380	0.73616	1.34760
C	2.28017	-0.58426	-0.23371
H	2.80685	-0.48979	-1.17875
H	2.90877	-1.13666	0.46397
O	1.08916	-1.31313	-0.54244
C	0.33439	-1.73380	0.60152
H	0.48721	-2.79997	0.75802
H	0.68780	-1.21796	1.49031
C	-1.14260	-1.46494	0.35428
H	-1.69601	-1.67481	1.27663
H	-1.50370	-2.14893	-0.41178
C	-2.77749	0.05297	-0.55585
H	-2.92923	1.05871	-0.93809
H	-3.46932	-0.11483	0.27488
H	-2.99960	-0.65628	-1.34845
Li	0.03778	-0.00953	-1.74643

ACE1_Li_uscs-mp2_def2tzvp

26

N	-1.38008	-0.10180	-0.13617
C	-1.01097	0.93346	0.84851
H	-1.90166	1.42108	1.26024
H	-0.49914	0.47670	1.69596
C	-0.12443	1.99205	0.20397
H	-0.68505	2.55025	-0.54729
H	0.24771	2.69600	0.95479
O	0.96822	1.40097	-0.51197
C	1.96364	0.79691	0.32111
H	2.86935	1.40975	0.31362

H	1.60988	0.74072	1.35124
C	2.28135	-0.58361	-0.23514
H	2.80947	-0.49138	-1.18488
H	2.91404	-1.13851	0.46408
O	1.08914	-1.31211	-0.54202
C	0.33507	-1.73545	0.60206
H	0.48937	-2.80661	0.75639
H	0.69185	-1.22181	1.49572
C	-1.14213	-1.46576	0.35567
H	-1.69852	-1.67840	1.28110
H	-1.50561	-2.15236	-0.41316
C	-2.77639	0.05221	-0.55909
H	-2.92974	1.06242	-0.94042
H	-3.47581	-0.11937	0.27007
H	-2.99705	-0.65630	-1.35827
Li	0.03521	-0.00979	-1.74555

ACE1_Li_uscs-mp2_def2tzv

26

N	-1.40270	-0.10077	-0.12873
C	-1.02888	0.95078	0.87534
H	-1.92462	1.43231	1.29482
H	-0.50028	0.49012	1.71539
C	-0.14177	2.02944	0.22631
H	-0.69092	2.59105	-0.53184
H	0.25177	2.71964	0.98092
O	0.98174	1.41619	-0.52400
C	2.03281	0.80517	0.32015
H	2.92964	1.43036	0.28247
H	1.68924	0.75091	1.35715
C	2.33993	-0.59194	-0.24522

H	2.85900	-0.52188	-1.20223
H	2.94885	-1.16582	0.46164
O	1.09218	-1.32165	-0.55941
C	0.31873	-1.78245	0.61735
H	0.47467	-2.85847	0.73839
H	0.68769	-1.28015	1.51660
C	-1.17314	-1.49182	0.37591
H	-1.72053	-1.68003	1.31604
H	-1.56008	-2.17442	-0.38759
C	-2.81353	0.06750	-0.59652
H	-2.94702	1.08865	-0.96309
H	-3.53558	-0.11972	0.21378
H	-3.00543	-0.62805	-1.41747
Li	0.02210	-0.01520	-1.74883

ACE1_Na_ub2plyp_def2svpp

26

N	-1.23594	1.28126	0.26520
C	-0.37154	1.36628	-0.89130
H	-0.52390	2.32377	-1.43899
H	0.67434	1.39089	-0.54404
C	-0.54385	0.26894	-1.96075
H	-1.50300	0.41494	-2.48028
H	0.25853	0.39353	-2.71730
O	-0.59355	-1.05766	-1.48136
C	0.58369	-1.56776	-0.89710
H	1.03080	-2.34398	-1.54922
H	1.34214	-0.77509	-0.78193
C	0.24902	-2.20133	0.44969
H	-0.41443	-3.06475	0.28389
H	1.18378	-2.57613	0.91549

O	-0.45913	-1.34442	1.31819
C	0.22725	-0.18607	1.74578
H	0.50015	-0.28081	2.81617
H	1.17375	-0.06181	1.19239
C	-0.64948	1.05820	1.56888
H	-0.03015	1.92868	1.87163
H	-1.47851	1.00416	2.29322
C	-2.59707	0.85504	0.03737
H	-2.69855	-0.21506	-0.23150
H	-3.05003	1.45345	-0.77570
H	-3.19699	1.03863	0.94425
Na	3.85609	1.09614	-0.06041

ACE1_Na_ub2plyp_def2svp

26

N	-1.23145	1.29044	0.27461
C	-0.36253	1.37546	-0.87943
H	-0.49825	2.33717	-1.41810
H	0.68080	1.38282	-0.53180
C	-0.54447	0.29328	-1.95928
H	-1.49997	0.45294	-2.47683
H	0.25539	0.42357	-2.71361
O	-0.60140	-1.03863	-1.49443
C	0.57202	-1.55351	-0.90572
H	1.02947	-2.31843	-1.55949
H	1.32510	-0.76186	-0.77372
C	0.23213	-2.20140	0.43021
H	-0.43019	-3.06060	0.25369
H	1.16394	-2.58497	0.88852
O	-0.47237	-1.35131	1.30884
C	0.22076	-0.19894	1.74345

H	0.49674	-0.29978	2.80978
H	1.16573	-0.07653	1.19136
C	-0.64633	1.05125	1.57626
H	-0.02193	1.91166	1.88651
H	-1.47372	0.99881	2.29903
C	-2.58722	0.84785	0.04416
H	-2.67421	-0.22006	-0.22465
H	-3.04718	1.43806	-0.76599
H	-3.18896	1.02204	0.94737
Na	3.87467	1.06888	-0.04978

ACE1_Na_ub2plyp_def2sv

26

N	-1.23594	1.28126	0.26520
C	-0.37154	1.36628	-0.89130
H	-0.52390	2.32377	-1.43899
H	0.67434	1.39089	-0.54404
C	-0.54385	0.26894	-1.96075
H	-1.50300	0.41494	-2.48028
H	0.25853	0.39353	-2.71730
O	-0.59355	-1.05766	-1.48136
C	0.58369	-1.56776	-0.89710
H	1.03080	-2.34398	-1.54922
H	1.34214	-0.77509	-0.78193
C	0.24902	-2.20133	0.44969
H	-0.41443	-3.06475	0.28389
H	1.18378	-2.57613	0.91549
O	-0.45913	-1.34442	1.31819
C	0.22725	-0.18607	1.74578
H	0.50015	-0.28081	2.81617
H	1.17375	-0.06181	1.19239

C	-0.64948	1.05820	1.56888
H	-0.03015	1.92868	1.87163
H	-1.47851	1.00416	2.29322
C	-2.59707	0.85504	0.03737
H	-2.69855	-0.21506	-0.23150
H	-3.05003	1.45345	-0.77570
H	-3.19699	1.03863	0.94425
Na	3.85609	1.09614	-0.06041

ACE1_Na_ub2plyp_def2tzvpp

26

N	1.03911	-1.27513	0.15003
C	0.18919	-1.01847	-1.00678
H	0.01720	-1.93404	-1.58349
H	-0.79777	-0.69297	-0.68347
C	0.78973	0.01262	-1.95978
H	1.72008	-0.37138	-2.37356
H	0.09345	0.17856	-2.78912
O	1.14405	1.24857	-1.35155
C	0.07638	1.97614	-0.76500
H	-0.07872	2.90678	-1.31788
H	-0.85516	1.41627	-0.82666
C	0.39880	2.34873	0.67517
H	1.25796	3.01611	0.68764
H	-0.45838	2.89352	1.08650
O	0.76640	1.26512	1.51430
C	-0.21085	0.24907	1.68929
H	-0.58078	0.27817	2.71703
H	-1.06938	0.42458	1.04334
C	0.38557	-1.12920	1.44033
H	-0.42160	-1.86636	1.59303

H	1.13708	-1.32345	2.20607
C	1.75858	-2.53158	0.04721
H	2.30882	-2.56718	-0.89101
H	1.09461	-3.40876	0.08501
H	2.47620	-2.61456	0.86157
Na	-4.42957	-0.63297	-0.26271

ACE1_Na_ub2plyp_def2tzvp

26

N	1.04258	-1.27274	0.14993
C	0.19235	-1.01984	-1.00749
H	0.02546	-1.93761	-1.58578
H	-0.79842	-0.69991	-0.68462
C	0.78864	0.01445	-1.95996
H	1.72135	-0.36671	-2.37590
H	0.08980	0.17943	-2.78985
O	1.14024	1.25079	-1.35074
C	0.07076	1.97708	-0.76538
H	-0.08474	2.90919	-1.31960
H	-0.86158	1.41542	-0.82859
C	0.39155	2.34997	0.67520
H	1.25022	3.02098	0.68868
H	-0.46899	2.89257	1.08743
O	0.76272	1.26682	1.51365
C	-0.21172	0.24799	1.68995
H	-0.58022	0.27606	2.72032
H	-1.07360	0.42172	1.04516
C	0.38850	-1.12865	1.44017
H	-0.41801	-1.86932	1.59317
H	1.14154	-1.32126	2.20729
C	1.76886	-2.52537	0.04752

H	2.31878	-2.55912	-0.89287
H	1.10919	-3.40785	0.08731
H	2.48957	-2.60392	0.86179
Na	-4.42871	-0.64319	-0.26243

ACE1_Na_ub2plyp_def2tzv

26

N	1.04860	-1.28593	0.15835
C	0.20469	-1.03081	-1.02255
H	0.04052	-1.95349	-1.59699
H	-0.78662	-0.70052	-0.71070
C	0.81404	-0.00381	-1.98665
H	1.76664	-0.36498	-2.36846
H	0.12940	0.15002	-2.82796
O	1.14634	1.28975	-1.37778
C	0.03712	2.01731	-0.76433
H	-0.12641	2.94067	-1.32587
H	-0.88205	1.43460	-0.82037
C	0.36772	2.40203	0.67627
H	1.24149	3.04946	0.69326
H	-0.48661	2.95078	1.08852
O	0.73067	1.29296	1.55872
C	-0.25580	0.22291	1.71266
H	-0.63068	0.24630	2.73824
H	-1.10685	0.39084	1.05255
C	0.38186	-1.14579	1.46355
H	-0.41214	-1.90231	1.61082
H	1.13815	-1.32076	2.23061
C	1.83389	-2.52660	0.05677
H	2.39351	-2.53236	-0.87815
H	1.20427	-3.43163	0.08680

H	2.54775	-2.57720	0.87876
Na	-4.42617	-0.69555	-0.29286

ACE1_Na_ub3lyp_def2svpp

26

N	1.53624	-0.73706	0.10062
C	0.74001	-0.77634	-1.11768
H	1.09136	-1.57126	-1.80976
H	-0.30152	-1.04999	-0.87829
C	0.75911	0.55168	-1.89411
H	1.78413	0.75083	-2.25152
H	0.10830	0.45185	-2.79027
O	0.40919	1.69361	-1.13709
C	-0.89890	1.74575	-0.60813
H	-1.46560	2.57315	-1.08331
H	-1.45939	0.81986	-0.83040
C	-0.86235	2.01628	0.89955
H	-0.43884	3.02112	1.06894
H	-1.90787	2.02692	1.27873
O	-0.04352	1.14569	1.64855
C	-0.37082	-0.22882	1.65532
H	-0.73847	-0.52069	2.66014
H	-1.19263	-0.44701	0.95032
C	0.86021	-1.08400	1.33794
H	0.53933	-2.15605	1.37967
H	1.58804	-0.94744	2.15820
C	2.85229	-1.32457	-0.03215
H	3.36878	-0.90944	-0.91589
H	2.83549	-2.43708	-0.14427
H	3.46855	-1.08700	0.85379
Na	-3.58509	-1.95206	-0.53514

ACE1_Na_ub3lyp_def2svp

26

N	1.48397	-0.79596	0.12198
C	0.68951	-0.81649	-1.09801
H	0.99208	-1.64465	-1.77076
H	-0.36542	-1.02253	-0.85730
C	0.78578	0.48885	-1.90269
H	1.81886	0.62154	-2.26252
H	0.13580	0.40457	-2.79870
O	0.49683	1.66450	-1.17056
C	-0.80797	1.79529	-0.64494
H	-1.33571	2.63416	-1.14028
H	-1.41335	0.89453	-0.84280
C	-0.76014	2.10066	0.85352
H	-0.28563	3.08420	1.00035
H	-1.80352	2.18061	1.22471
O	0.00551	1.20261	1.62764
C	-0.39715	-0.15290	1.65281
H	-0.79493	-0.40923	2.65383
H	-1.21862	-0.33813	0.94068
C	0.78502	-1.07990	1.36255
H	0.40519	-2.12928	1.42477
H	1.51438	-0.97097	2.18319
C	2.76420	-1.46209	0.00768
H	3.31100	-1.09577	-0.87591
H	2.68385	-2.57062	-0.08638
H	3.38702	-1.24916	0.89185
Na	-3.61756	-1.90864	-0.50735

ACE1_Na_ub3lyp_def2sv

26

N	1.53624	-0.73706	0.10062
C	0.74001	-0.77634	-1.11768
H	1.09136	-1.57126	-1.80976
H	-0.30152	-1.04999	-0.87829
C	0.75911	0.55168	-1.89411
H	1.78413	0.75083	-2.25152
H	0.10830	0.45185	-2.79027
O	0.40919	1.69361	-1.13709
C	-0.89890	1.74575	-0.60813
H	-1.46560	2.57315	-1.08331
H	-1.45939	0.81986	-0.83040
C	-0.86235	2.01628	0.89955
H	-0.43884	3.02112	1.06894
H	-1.90787	2.02692	1.27873
O	-0.04352	1.14569	1.64855
C	-0.37082	-0.22882	1.65532
H	-0.73847	-0.52069	2.66014
H	-1.19263	-0.44701	0.95032
C	0.86021	-1.08400	1.33794
H	0.53933	-2.15605	1.37967
H	1.58804	-0.94744	2.15820
C	2.85229	-1.32457	-0.03215
H	3.36878	-0.90944	-0.91589
H	2.83549	-2.43708	-0.14427
H	3.46855	-1.08700	0.85379
Na	-3.58509	-1.95206	-0.53514

ACE1_Na_ub3lyp_def2tzvpp

26

N	-0.76558	-1.41247	-0.15172
C	0.05536	-1.02967	0.99236

H	0.38088	-1.91067	1.56042
H	0.97675	-0.55592	0.65187
C	-0.67041	-0.10511	1.97547
H	-1.51642	-0.63178	2.41827
H	0.02061	0.15936	2.78662
O	-1.23551	1.06723	1.39938
C	-0.33080	1.97363	0.78557
H	-0.32138	2.91501	1.34683
H	0.68825	1.58542	0.81147
C	-0.75953	2.29775	-0.64251
H	-1.72355	2.80693	-0.62103
H	-0.02359	2.99197	-1.06982
O	-0.95905	1.17666	-1.49095
C	0.16072	0.32844	-1.71080
H	0.48370	0.42301	-2.75299
H	1.00637	0.63763	-1.09504
C	-0.19511	-1.13490	-1.46144
H	0.71652	-1.72794	-1.66428
H	-0.93400	-1.43948	-2.20614
C	-1.29738	-2.76006	-0.05269
H	-1.81367	-2.88991	0.89888
H	-0.51866	-3.53887	-0.12304
H	-2.02024	-2.93539	-0.85064
Na	4.15788	-0.12489	0.21704

ACE1_Na_ub3lyp_def2tzvp

26

N	-0.76209	-1.41447	-0.15149
C	0.05839	-1.02987	0.99230
H	0.38566	-1.91078	1.56118
H	0.97947	-0.55402	0.65147

C	-0.66908	-0.10646	1.97508
H	-1.51431	-0.63570	2.41844
H	0.02231	0.16034	2.78632
O	-1.23753	1.06385	1.39828
C	-0.33568	1.97413	0.78599
H	-0.33099	2.91607	1.34815
H	0.68598	1.59045	0.81216
C	-0.76487	2.29690	-0.64234
H	-1.73068	2.80451	-0.62120
H	-0.02924	2.99235	-1.07041
O	-0.96283	1.17426	-1.48924
C	0.15913	0.32908	-1.71025
H	0.48168	0.42496	-2.75339
H	1.00475	0.63986	-1.09384
C	-0.19336	-1.13510	-1.46159
H	0.72028	-1.72631	-1.66565
H	-0.93292	-1.44080	-2.20634
C	-1.29210	-2.76269	-0.05230
H	-1.80729	-2.89339	0.90066
H	-0.51213	-3.54133	-0.12419
H	-2.01654	-2.93836	-0.84983
Na	4.15971	-0.11703	0.21575

ACE1_Na_ub3lyp_def2tzv

26

N	-0.74020	-1.41844	-0.22039
C	-0.09648	-1.02591	1.04262
H	0.15473	-1.90686	1.65005
H	0.85544	-0.53521	0.83940
C	-0.96900	-0.11427	1.91602
H	-1.88771	-0.62775	2.19403

H	-0.42034	0.13664	2.83126
O	-1.43107	1.11074	1.26182
C	-0.40182	2.01552	0.76215
H	-0.46480	2.95436	1.31947
H	0.59273	1.60498	0.93655
C	-0.62151	2.34173	-0.71355
H	-1.58232	2.83727	-0.83944
H	0.16839	3.03279	-1.03100
O	-0.69048	1.19813	-1.61652
C	0.45758	0.29558	-1.64678
H	0.93412	0.37936	-2.62648
H	1.19862	0.59091	-0.90321
C	0.02013	-1.15772	-1.45104
H	0.93342	-1.77958	-1.52133
H	-0.61724	-1.44085	-2.29100
C	-1.34345	-2.75620	-0.18821
H	-2.00139	-2.84865	0.67598
H	-0.59291	-3.56381	-0.13212
H	-1.94486	-2.91439	-1.08399
Na	4.05048	-0.18667	0.54863

ACE1_Na_ub971_def2svpp

26

N	1.43786	0.84395	-0.06825
C	0.88646	1.45361	1.12606
H	1.66670	1.38992	1.91042
H	0.65978	2.54646	1.00663
C	-0.39881	0.80379	1.67078
H	-0.57133	1.22222	2.68235
H	-1.27862	1.09753	1.06313
O	-0.32820	-0.60226	1.77683

C	-1.09667	-1.37314	0.87869
H	-1.85374	-1.95840	1.44416
H	-1.66073	-0.72168	0.18400
C	-0.24745	-2.38296	0.09379
H	0.14813	-3.13734	0.79834
H	-0.92978	-2.90974	-0.61282
O	0.88467	-1.88179	-0.57052
C	0.71060	-0.96030	-1.62175
H	1.63108	-1.04361	-2.22929
H	-0.14349	-1.24595	-2.27544
C	0.54397	0.51222	-1.16334
H	-0.49946	0.69858	-0.86518
H	0.71072	1.16393	-2.05157
C	2.71245	1.39389	-0.47139
H	3.41694	1.39187	0.38265
H	2.64506	2.44538	-0.85106
H	3.16040	0.77716	-1.27307
Na	-3.66197	1.41507	-1.05119

ACE1_Na_ub971_def2svp

26

N	1.43862	0.84268	-0.07049
C	0.89206	1.45139	1.12677
H	1.67371	1.39051	1.90713
H	0.66489	2.54221	1.01059
C	-0.39054	0.80214	1.67220
H	-0.56811	1.22673	2.67822
H	-1.26690	1.09143	1.06119
O	-0.31935	-0.60466	1.78239
C	-1.09123	-1.37113	0.88187
H	-1.85199	-1.95355	1.44138

H	-1.65149	-0.71584	0.19075
C	-0.25023	-2.38177	0.09303
H	0.14223	-3.13980	0.79242
H	-0.93934	-2.90420	-0.60696
O	0.88057	-1.88396	-0.57795
C	0.70192	-0.95707	-1.62484
H	1.61584	-1.03768	-2.23929
H	-0.15319	-1.23706	-2.27601
C	0.53806	0.51229	-1.16035
H	-0.50195	0.69580	-0.85488
H	0.69405	1.16503	-2.04708
C	2.71214	1.39189	-0.47853
H	3.41826	1.39313	0.37031
H	2.64541	2.43977	-0.85925
H	3.15901	0.77689	-1.27803
Na	-3.66487	1.41730	-1.04485

ACE1_Na_ub971_def2sv

26

N	1.43786	0.84395	-0.06825
C	0.88646	1.45361	1.12606
H	1.66670	1.38992	1.91042
H	0.65978	2.54646	1.00663
C	-0.39881	0.80379	1.67078
H	-0.57133	1.22222	2.68235
H	-1.27862	1.09753	1.06313
O	-0.32820	-0.60226	1.77683
C	-1.09667	-1.37314	0.87869
H	-1.85374	-1.95840	1.44416
H	-1.66073	-0.72168	0.18400
C	-0.24745	-2.38296	0.09379

H 0.14813 -3.13734 0.79834

H -0.92978 -2.90974 -0.61282

O 0.88467 -1.88179 -0.57052

C 0.71060 -0.96030 -1.62175

H 1.63108 -1.04361 -2.22929

H -0.14349 -1.24595 -2.27544

C 0.54397 0.51222 -1.16334

H -0.49946 0.69858 -0.86518

H 0.71072 1.16393 -2.05157

C 2.71245 1.39389 -0.47139

H 3.41694 1.39187 0.38265

H 2.64506 2.44538 -0.85106

H 3.16040 0.77716 -1.27307

Na -3.66197 1.41507 -1.05119

ACE1_Na_ub971_def2tzvpp

26

N 0.69945 -1.58211 0.22773

C 0.16923 -1.23223 -1.07734

H 0.15689 -2.11586 -1.73802

H -0.87852 -0.94163 -0.95904

C 0.93545 -0.16298 -1.89191

H 1.89368 -0.57705 -2.21403

H 0.35136 0.04734 -2.80051

O 1.27100 1.03605 -1.21508

C 0.18955 1.85449 -0.80821

H 0.17456 2.77913 -1.40121

H -0.76684 1.35230 -0.98226

C 0.34871 2.23592 0.66413

H 1.24711 2.84596 0.78507

H -0.51676 2.84589 0.96375

O	0.52662	1.12926	1.53101
C	-0.55196	0.20913	1.58192
H	-1.05658	0.28829	2.55533
H	-1.30086	0.44838	0.82098
C	-0.07085	-1.23934	1.41068
H	-0.97723	-1.86276	1.46288
H	0.53675	-1.50871	2.27886
C	2.14269	-1.63265	0.35214
H	2.63458	-0.64996	0.30647
H	2.56199	-2.25527	-0.44638
H	2.40929	-2.10213	1.30205
Na	-4.06576	-0.45780	-0.49491

ACE1_Na_ub971_def2tzvp

26

N	0.69952	-1.58122	0.22909
C	0.17013	-1.23359	-1.07683
H	0.15960	-2.11863	-1.73710
H	-0.87838	-0.94382	-0.95977
C	0.93553	-0.16431	-1.89190
H	1.89457	-0.57837	-2.21436
H	0.35038	0.04585	-2.80099
O	1.27127	1.03486	-1.21514
C	0.18982	1.85443	-0.80963
H	0.17622	2.77949	-1.40375
H	-0.76774	1.35254	-0.98378
C	0.34886	2.23685	0.66258
H	1.24843	2.84664	0.78345
H	-0.51716	2.84787	0.96166
O	0.52653	1.13054	1.53041
C	-0.55238	0.21060	1.58194

H	-1.05678	0.29026	2.55640
H	-1.30202	0.44952	0.82053
C	-0.07121	-1.23800	1.41158
H	-0.97832	-1.86175	1.46394
H	0.53674	-1.50688	2.28062
C	2.14255	-1.63332	0.35332
H	2.63582	-0.65060	0.30424
H	2.56094	-2.25941	-0.44404
H	2.40901	-2.10051	1.30524
Na	-4.06637	-0.45844	-0.49495

ACE1_Na_ub971_def2tzv

26

N	0.69582	-1.56162	0.26093
C	0.17118	-1.25755	-1.06801
H	0.15943	-2.16395	-1.69872
H	-0.87578	-0.95465	-0.96815
C	0.95697	-0.22024	-1.91117
H	1.92328	-0.63635	-2.19966
H	0.38580	-0.02355	-2.82912
O	1.30160	1.03143	-1.24979
C	0.19178	1.87465	-0.83479
H	0.18217	2.78359	-1.44758
H	-0.76148	1.36344	-0.99455
C	0.36454	2.28983	0.62729
H	1.28293	2.86702	0.74110
H	-0.48517	2.92591	0.91158
O	0.50943	1.17949	1.55906
C	-0.59607	0.22925	1.59217
H	-1.11110	0.32196	2.55592
H	-1.32295	0.46294	0.80932

C	-0.09371	-1.21940	1.44450
H	-0.99399	-1.85331	1.49846
H	0.51231	-1.46738	2.32016
C	2.14642	-1.68606	0.39235
H	2.68509	-0.73032	0.31376
H	2.53472	-2.35679	-0.38368
H	2.38801	-2.13521	1.35903
Na	-4.06445	-0.47476	-0.52206

ACE1_Na_ucam-b3lyp_def2svpp

26

N	0.82450	1.50101	-0.18082
C	0.22072	1.21765	1.09859
H	0.25025	2.10738	1.76656
H	-0.85016	1.00913	0.93728
C	0.86235	0.08357	1.91859
H	1.84062	0.41862	2.29653
H	0.22300	-0.11097	2.80416
O	1.13484	-1.10268	1.21611
C	0.04111	-1.85049	0.75355
H	-0.04215	-2.79948	1.31908
H	-0.90597	-1.30651	0.91278
C	0.23658	-2.18743	-0.71996
H	1.11480	-2.84447	-0.82276
H	-0.64894	-2.74983	-1.08136
O	0.50570	-1.06894	-1.52561
C	-0.51068	-0.10203	-1.61881
H	-0.98025	-0.13704	-2.62225
H	-1.31241	-0.30884	-0.89008
C	0.05430	1.29927	-1.38458
H	-0.80384	2.00232	-1.42474

H	0.70325	1.56028	-2.23609
C	2.26174	1.44678	-0.26071
H	2.67855	0.42080	-0.21529
H	2.71205	2.03149	0.56373
H	2.59932	1.91039	-1.20288
Na	-4.04278	0.56533	0.44700

ACE1_Na_ucam-b3lyp_def2svp

26

N	0.81095	1.50829	-0.19134
C	0.20765	1.22698	1.08916
H	0.22333	2.11946	1.75102
H	-0.85919	1.00544	0.93082
C	0.85944	0.10898	1.91932
H	1.83113	0.45823	2.29747
H	0.22254	-0.08134	2.80555
O	1.14479	-1.08165	1.22776
C	0.05679	-1.83935	0.76514
H	-0.03022	-2.78150	1.33842
H	-0.89266	-1.29885	0.91102
C	0.25999	-2.19061	-0.70147
H	1.14239	-2.84120	-0.79477
H	-0.61708	-2.76803	-1.05513
O	0.51713	-1.07797	-1.52028
C	-0.50805	-0.11924	-1.61590
H	-0.98423	-0.16475	-2.61403
H	-1.30415	-0.32662	-0.88330
C	0.04446	1.28690	-1.39447
H	-0.81805	1.98102	-1.44515
H	0.69090	1.54591	-2.24671
C	2.24855	1.44829	-0.27205

H	2.66005	0.42302	-0.22002
H	2.70309	2.03506	0.54504
H	2.58709	1.90248	-1.21549
Na	-4.04908	0.54354	0.43967

ACE1_Na_ucam-b3lyp_def2sv

26

N	0.82450	1.50101	-0.18082
C	0.22072	1.21765	1.09859
H	0.25025	2.10738	1.76656
H	-0.85016	1.00913	0.93728
C	0.86235	0.08357	1.91859
H	1.84062	0.41862	2.29653
H	0.22300	-0.11097	2.80416
O	1.13484	-1.10268	1.21611
C	0.04111	-1.85049	0.75355
H	-0.04215	-2.79948	1.31908
H	-0.90597	-1.30651	0.91278
C	0.23658	-2.18743	-0.71996
H	1.11480	-2.84447	-0.82276
H	-0.64894	-2.74983	-1.08136
O	0.50570	-1.06894	-1.52561
C	-0.51068	-0.10203	-1.61881
H	-0.98025	-0.13704	-2.62225
H	-1.31241	-0.30884	-0.89008
C	0.05430	1.29927	-1.38458
H	-0.80384	2.00232	-1.42474
H	0.70325	1.56028	-2.23609
C	2.26174	1.44678	-0.26071
H	2.67855	0.42080	-0.21529
H	2.71205	2.03149	0.56373

H	2.59932	1.91039	-1.20288
Na	-4.04278	0.56533	0.44700

ACE1_Na_ucam-b3lyp_def2tzvpp

26

N	1.67994	0.57276	-0.06524
C	0.80175	0.82052	1.06409
H	1.19227	1.62272	1.70127
H	-0.16504	1.18023	0.71481
C	0.60786	-0.40549	1.94913
H	1.56115	-0.68798	2.39470
H	-0.08011	-0.15076	2.76428
O	0.16359	-1.56498	1.26942
C	-1.09368	-1.48264	0.63344
H	-1.79913	-2.15975	1.12564
H	-1.51262	-0.48002	0.72267
C	-0.99297	-1.90641	-0.82202
H	-0.70820	-2.95688	-0.86850
H	-1.98554	-1.80826	-1.27829
O	-0.01452	-1.22629	-1.57919
C	-0.16173	0.17398	-1.70266
H	-0.43147	0.42034	-2.73378
H	-0.97630	0.53344	-1.07417
C	1.13459	0.89154	-1.36904
H	0.95196	1.97281	-1.49912
H	1.88350	0.61089	-2.11174
C	3.01327	1.09881	0.11976
H	3.42658	0.74742	1.06448
H	3.04269	2.20024	0.12687
H	3.66672	0.75022	-0.67975
Na	-3.71573	1.94404	0.30266

ACE1_Na_ucam-b3lyp_def2tzvp

26

N	1.67996	0.57347	-0.06518
C	0.80221	0.82116	1.06447
H	1.19393	1.62323	1.70257
H	-0.16510	1.18218	0.71549
C	0.60772	-0.40503	1.94912
H	1.56148	-0.68779	2.39556
H	-0.08172	-0.15010	2.76415
O	0.16449	-1.56459	1.26876
C	-1.09347	-1.48357	0.63366
H	-1.79784	-2.16297	1.12627
H	-1.51425	-0.48091	0.72385
C	-0.99282	-1.90654	-0.82201
H	-0.70750	-2.95771	-0.86896
H	-1.98615	-1.80770	-1.27861
O	-0.01391	-1.22624	-1.57860
C	-0.16155	0.17403	-1.70283
H	-0.43045	0.41979	-2.73526
H	-0.97729	0.53400	-1.07474
C	1.13448	0.89187	-1.36895
H	0.95136	1.97393	-1.49948
H	1.88392	0.61076	-2.11214
C	3.01364	1.09864	0.11959
H	3.42664	0.74735	1.06541
H	3.04401	2.20092	0.12575
H	3.66746	0.74840	-0.67999
Na	-3.71766	1.94323	0.30244

ACE1_Na_ucam-b3lyp_def2tzv

26

N	1.70480	0.55935	-0.06757
C	0.81880	0.83867	1.06171
H	1.21811	1.65088	1.68233
H	-0.14351	1.19952	0.70179
C	0.60747	-0.36199	1.98178
H	1.55632	-0.67369	2.41132
H	-0.06425	-0.07510	2.79692
O	0.10251	-1.55498	1.32419
C	-1.16635	-1.44159	0.63836
H	-1.89397	-2.08249	1.14011
H	-1.54712	-0.42252	0.69251
C	-1.04941	-1.91643	-0.80068
H	-0.75721	-2.96319	-0.81469
H	-2.03218	-1.82596	-1.27420
O	-0.04028	-1.24905	-1.59647
C	-0.14139	0.19003	-1.73354
H	-0.39344	0.42282	-2.76924
H	-0.94762	0.57804	-1.11277
C	1.18253	0.86259	-1.39888
H	1.04361	1.94707	-1.55548
H	1.92772	0.53317	-2.12354
C	3.07760	1.01517	0.12897
H	3.46240	0.64737	1.07873
H	3.16390	2.11350	0.12927
H	3.71639	0.62518	-0.66219
Na	-3.70146	1.97485	0.27855

ACE1_Na_udsdpbepb86_def2svpp

26

N	1.58735	0.53642	-0.11772
C	0.64148	1.02164	0.87301

H 0.98489 1.97138 1.33794

H -0.31704 1.27132 0.38865

C 0.40632 0.00561 1.99239

H 1.34361 -0.13587 2.55801

H -0.35518 0.40602 2.69720

O 0.05026 -1.28259 1.53974

C -1.17249 -1.38223 0.84684

H -1.90246 -1.96727 1.44400

H -1.62497 -0.38732 0.68828

C -0.96475 -2.10301 -0.48152

H -0.65602 -3.14226 -0.27634

H -1.93535 -2.13921 -1.02294

O 0.05961 -1.56676 -1.28487

C -0.12500 -0.24779 -1.74788

H -0.32059 -0.25548 -2.83979

H -1.00939 0.22023 -1.27848

C 1.13161 0.57908 -1.49197

H 0.93966 1.61881 -1.85964

H 1.94237 0.16590 -2.11985

C 2.91648 1.07970 0.05408

H 3.25113 0.93094 1.09632

H 2.97925 2.17206 -0.17151

H 3.62949 0.55348 -0.60595

Na -3.26743 2.18538 -0.13823

ACE1_Na_udsdpbepb86_def2svp

26

N 1.58311 0.54485 -0.11814

C 0.63720 1.01210 0.87998

H 0.96860 1.95974 1.34996

H -0.32379 1.25314 0.40273

C	0.41519	-0.01191	1.99233
H	1.35248	-0.14916	2.55335
H	-0.34198	0.38146	2.70073
O	0.06345	-1.29869	1.53300
C	-1.15821	-1.39605	0.83804
H	-1.88943	-1.98200	1.42672
H	-1.61069	-0.40347	0.68433
C	-0.95257	-2.10691	-0.49369
H	-0.64121	-3.14399	-0.29710
H	-1.92524	-2.14485	-1.02480
O	0.06277	-1.55960	-1.30081
C	-0.13156	-0.23528	-1.74396
H	-0.34103	-0.22497	-2.83004
H	-1.00950	0.22165	-1.25867
C	1.12018	0.59550	-1.48929
H	0.91844	1.63340	-1.84667
H	1.92932	0.19761	-2.12392
C	2.90743	1.09770	0.05234
H	3.25078	0.94171	1.08646
H	2.95938	2.18931	-0.15948
H	3.61966	0.58971	-0.61608
Na	-3.27575	2.18202	-0.11751

ACE1_Na_udsdpbepb86_def2sv

26

N	1.58735	0.53642	-0.11772
C	0.64148	1.02164	0.87301
H	0.98489	1.97138	1.33794
H	-0.31704	1.27132	0.38865
C	0.40632	0.00561	1.99239
H	1.34361	-0.13587	2.55801

H	-0.35518	0.40602	2.69720
O	0.05026	-1.28259	1.53974
C	-1.17249	-1.38223	0.84684
H	-1.90246	-1.96727	1.44400
H	-1.62497	-0.38732	0.68828
C	-0.96475	-2.10301	-0.48152
H	-0.65602	-3.14226	-0.27634
H	-1.93535	-2.13921	-1.02294
O	0.05961	-1.56676	-1.28487
C	-0.12500	-0.24779	-1.74788
H	-0.32059	-0.25548	-2.83979
H	-1.00939	0.22023	-1.27848
C	1.13161	0.57908	-1.49197
H	0.93966	1.61881	-1.85964
H	1.94237	0.16590	-2.11985
C	2.91648	1.07970	0.05408
H	3.25113	0.93094	1.09632
H	2.97925	2.17206	-0.17151
H	3.62949	0.55348	-0.60595
Na	-3.26743	2.18538	-0.13823

ACE1_Na_udsdpbepb86_def2tzvpp

26

N	1.62007	0.48248	-0.10345
C	0.67044	0.96852	0.88776
H	1.02426	1.89374	1.36029
H	-0.27015	1.23152	0.40541
C	0.42153	-0.04820	1.99596
H	1.34923	-0.22417	2.54093
H	-0.31492	0.35921	2.70019
O	0.01720	-1.32866	1.53616

C	-1.20301	-1.35346	0.81942
H	-1.95836	-1.90016	1.39394
H	-1.58859	-0.34447	0.67356
C	-1.02740	-2.06642	-0.51190
H	-0.76717	-3.10853	-0.32809
H	-1.98688	-2.04323	-1.04436
O	0.01929	-1.56254	-1.32078
C	-0.12548	-0.21542	-1.73721
H	-0.33117	-0.18641	-2.81182
H	-0.97847	0.25276	-1.24334
C	1.15289	0.56528	-1.47539
H	0.97936	1.60632	-1.80337
H	1.94348	0.15918	-2.11050
C	2.92920	1.08643	0.05099
H	3.28295	0.94238	1.07217
H	2.92636	2.16826	-0.16051
H	3.63910	0.60803	-0.62450
Na	-3.22642	2.24704	-0.10879

ACE1_Na_udsdpbepb86_def2tzvp

26

N	1.61685	0.48658	-0.10509
C	0.66858	0.97384	0.88685
H	1.02459	1.90131	1.35950
H	-0.27396	1.23937	0.40439
C	0.41954	-0.04176	1.99650
H	1.34747	-0.21448	2.54714
H	-0.32193	0.36608	2.69943
O	0.02093	-1.32475	1.53851
C	-1.19902	-1.35827	0.82184
H	-1.95259	-1.91095	1.39841

H	-1.59338	-0.35006	0.67547
C	-1.01960	-2.07245	-0.50885
H	-0.75236	-3.11516	-0.32318
H	-1.98236	-2.05631	-1.04124
O	0.02327	-1.56421	-1.31989
C	-0.12689	-0.21811	-1.73811
H	-0.33280	-0.19133	-2.81551
H	-0.98443	0.24841	-1.24535
C	1.14871	0.56815	-1.47660
H	0.97028	1.61097	-1.80519
H	1.94198	0.16536	-2.11464
C	2.92797	1.08637	0.04966
H	3.28023	0.94445	1.07406
H	2.93030	2.16988	-0.16565
H	3.63977	0.60257	-0.62363
Na	-3.22983	2.24263	-0.11137

ACE1_Na_udsdpbepb86_def2tzv

26

N	1.66095	0.46279	-0.10764
C	0.76128	0.87643	0.98399
H	1.16958	1.74486	1.52681
H	-0.19751	1.20939	0.57614
C	0.53240	-0.23673	2.01408
H	1.47762	-0.51770	2.48153
H	-0.15815	0.12324	2.78953
O	0.02825	-1.49112	1.44856
C	-1.25090	-1.39468	0.75294
H	-1.99927	-1.97049	1.31002
H	-1.59315	-0.35695	0.72374
C	-1.15349	-1.99351	-0.64980

H	-0.89823	-3.05189	-0.58191
H	-2.13522	-1.89436	-1.13421
O	-0.11007	-1.42933	-1.50187
C	-0.19451	0.00605	-1.76051
H	-0.43226	0.15443	-2.81963
H	-1.00800	0.44926	-1.18060
C	1.14101	0.69271	-1.46566
H	1.00547	1.77259	-1.68205
H	1.89153	0.31248	-2.16611
C	3.02927	0.98127	0.05445
H	3.40837	0.71226	1.04359
H	3.07973	2.08112	-0.04967
H	3.68370	0.53095	-0.69621
Na	-3.22333	2.29415	0.13291

ACE1_Na_uhse06_def2svpp

26

N	0.90702	1.46421	-0.19161
C	0.27138	1.23375	1.07863
H	0.35029	2.12885	1.73887
H	-0.81166	1.09664	0.91036
C	0.82946	0.07445	1.92242
H	1.81819	0.36011	2.31774
H	0.16265	-0.07141	2.79918
O	1.05150	-1.13036	1.24034
C	-0.07203	-1.82138	0.76928
H	-0.22483	-2.75373	1.35065
H	-0.99294	-1.22326	0.89872
C	0.13158	-2.19672	-0.69234
H	0.97512	-2.90317	-0.76702
H	-0.77714	-2.72015	-1.05897

O	0.47655	-1.11395	-1.51347
C	-0.48021	-0.09340	-1.63154
H	-0.93937	-0.11345	-2.64205
H	-1.30588	-0.24605	-0.91273
C	0.15374	1.27858	-1.40454
H	-0.66817	2.02439	-1.47641
H	0.83294	1.49412	-2.24715
C	2.33851	1.35665	-0.23543
H	2.72336	0.31863	-0.14894
H	2.79081	1.95170	0.58252
H	2.71482	1.77974	-1.18322
Na	-4.02330	0.69004	0.41144

ACE1_Na_uhse06_def2svp

26

N	0.88261	1.47872	-0.18904
C	0.23994	1.23479	1.07573
H	0.28385	2.12983	1.73556
H	-0.83511	1.06989	0.89333
C	0.81694	0.09274	1.92654
H	1.79460	0.40120	2.32784
H	0.15097	-0.05970	2.80019
O	1.06732	-1.11192	1.25136
C	-0.04291	-1.81798	0.76679
H	-0.19294	-2.75096	1.34413
H	-0.97020	-1.23092	0.88643
C	0.17589	-2.19077	-0.69086
H	1.02898	-2.88374	-0.75972
H	-0.71939	-2.73379	-1.05694
O	0.50243	-1.10262	-1.51411
C	-0.47368	-0.09913	-1.62929

H	-0.93960	-0.12721	-2.63422
H	-1.29137	-0.26547	-0.90746
C	0.13532	1.28157	-1.40392
H	-0.69690	2.01176	-1.47875
H	0.81026	1.50903	-2.24450
C	2.31437	1.36360	-0.23556
H	2.69040	0.32455	-0.15971
H	2.77364	1.94700	0.58264
H	2.69082	1.79042	-1.17827
Na	-4.02813	0.64041	0.40165

ACE1_Na_uhse06_def2sv

26

N	0.90744	1.46381	-0.19192
C	0.27134	1.23417	1.07820
H	0.35024	2.12978	1.73785
H	-0.81168	1.09714	0.90987
C	0.82889	0.07513	1.92274
H	1.81759	0.36075	2.31829
H	0.16172	-0.07027	2.79936
O	1.05088	-1.13004	1.24115
C	-0.07285	-1.82108	0.76980
H	-0.22615	-2.75327	1.35145
H	-0.99369	-1.22272	0.89871
C	0.13114	-2.19705	-0.69174
H	0.97452	-2.90380	-0.76594
H	-0.77773	-2.72047	-1.05838
O	0.47664	-1.11424	-1.51340
C	-0.47970	-0.09361	-1.63199
H	-0.93851	-0.11388	-2.64261
H	-1.30552	-0.24579	-0.91343

C 0.15454 1.27805 -1.40507

H -0.66698 2.02406 -1.47745

H 0.83406 1.49309 -2.24746

C 2.33902 1.35575 -0.23522

H 2.72353 0.31766 -0.14816

H 2.79127 1.95096 0.58263

H 2.71583 1.77833 -1.18304

Na -4.02319 0.69071 0.41086

ACE1_Na_uhse06_def2tzvpp

26

N -0.70876 -1.55821 -0.22829

C -0.15161 -1.21925 1.05622

H -0.10815 -2.10593 1.71019

H 0.88816 -0.91475 0.91402

C -0.90334 -0.17352 1.88640

H -1.85776 -0.59050 2.21265

H -0.31262 0.02587 2.79168

O -1.24181 1.02669 1.22885

C -0.16471 1.82969 0.80729

H -0.11982 2.74689 1.40798

H 0.78666 1.31154 0.95508

C -0.35082 2.22204 -0.64562

H -1.24594 2.83949 -0.74172

H 0.50994 2.82875 -0.96032

O -0.55341 1.12749 -1.51004

C 0.51567 0.21043 -1.58701

H 1.00830 0.29696 -2.56449

H 1.27558 0.44107 -0.83536

C 0.03353 -1.22403 -1.41857

H 0.93123 -1.85548 -1.49758

H	-0.59491	-1.48299	-2.27373
C	-2.14334	-1.61275	-0.31765
H	-2.63747	-0.63350	-0.25230
H	-2.54348	-2.24254	0.48364
H	-2.43273	-2.07535	-1.26290
Na	4.06943	-0.46466	0.46130

ACE1_Na_uhse06_def2tzvp

26

N	-0.70852	-1.55777	-0.22860
C	-0.15423	-1.21995	1.05747
H	-0.11442	-2.10735	1.71206
H	0.88725	-0.91759	0.91782
C	-0.90673	-0.17290	1.88544
H	-1.86235	-0.58962	2.21096
H	-0.31674	0.02769	2.79191
O	-1.24451	1.02639	1.22571
C	-0.16708	1.83079	0.80724
H	-0.12568	2.74911	1.40801
H	0.78528	1.31387	0.95832
C	-0.34959	2.22211	-0.64647
H	-1.24526	2.83968	-0.74518
H	0.51271	2.82893	-0.95958
O	-0.55050	1.12674	-1.51045
C	0.51914	0.20998	-1.58541
H	1.01334	0.29613	-2.56305
H	1.27821	0.44110	-0.83209
C	0.03675	-1.22441	-1.41721
H	0.93528	-1.85634	-1.49393
H	-0.59011	-1.48365	-2.27432
C	-2.14290	-1.61285	-0.32075

H	-2.63766	-0.63327	-0.25395
H	-2.54446	-2.24507	0.47894
H	-2.43054	-2.07374	-1.26812
Na	4.06933	-0.46428	0.46441

ACE1_Na_uhse06_def2tzv

26

N	-0.70483	-1.52175	-0.28300
C	-0.12155	-1.25609	1.01517
H	-0.05520	-2.17880	1.61687
H	0.91050	-0.92626	0.87321
C	-0.87851	-0.26662	1.91310
H	-1.82962	-0.69990	2.22167
H	-0.27974	-0.09487	2.81681
O	-1.24682	0.99217	1.30182
C	-0.15795	1.83544	0.86666
H	-0.10822	2.72320	1.50577
H	0.79638	1.31289	0.96645
C	-0.38899	2.29302	-0.56097
H	-1.30833	2.87500	-0.61727
H	0.44701	2.93843	-0.85948
O	-0.57252	1.21853	-1.51189
C	0.51901	0.27331	-1.62036
H	0.99172	0.38932	-2.60164
H	1.28435	0.48220	-0.86821
C	0.01899	-1.16389	-1.48904
H	0.90728	-1.80326	-1.61500
H	-0.63519	-1.38037	-2.33646
C	-2.14177	-1.70182	-0.33844
H	-2.71470	-0.77573	-0.18945
H	-2.46135	-2.41800	0.42713

H -2.42322 -2.12059 -1.30631

Na 4.07922 -0.49425 0.44643

ACE1_Na_um062x_def2svpp

26

N 1.20118 1.30437 -0.27582

C 0.30611 1.38447 0.85641

H 0.40074 2.36241 1.37753

H -0.73269 1.34661 0.48579

C 0.51056 0.32467 1.95142

H 1.46266 0.51640 2.47030

H -0.29931 0.43707 2.70092

O 0.60163 -1.00162 1.49740

C -0.54886 -1.54495 0.90514

H -0.99731 -2.31211 1.56516

H -1.31611 -0.76570 0.75260

C -0.16728 -2.19589 -0.41796

H 0.51281 -3.03896 -0.21778

H -1.07742 -2.59911 -0.90617

O 0.53649 -1.32883 -1.26927

C -0.18379 -0.21977 -1.74825

H -0.42903 -0.35825 -2.81962

H -1.14788 -0.11669 -1.21719

C 0.64238 1.05676 -1.58439

H 0.00156 1.90530 -1.89774

H 1.48372 1.02369 -2.29638

C 2.54542 0.85079 -0.00996

H 2.60776 -0.21428 0.28681

H 2.99703 1.45961 0.79590

H 3.16388 0.99869 -0.91053

Na -3.88827 0.99382 0.02016

ACE1_Na_um062x_def2svp

26

N	1.19975	1.31477	-0.27047
C	0.30023	1.38240	0.86035
H	0.38366	2.35660	1.38538
H	-0.73561	1.33932	0.48846
C	0.50790	0.32283	1.95226
H	1.45649	0.51773	2.47243
H	-0.29998	0.43424	2.70085
O	0.60247	-1.00383	1.49761
C	-0.54499	-1.54677	0.89751
H	-0.99849	-2.31360	1.55077
H	-1.31097	-0.76948	0.74290
C	-0.15859	-2.19367	-0.42406
H	0.52008	-3.03546	-0.22443
H	-1.06547	-2.59916	-0.91173
O	0.54383	-1.32258	-1.27354
C	-0.17947	-0.21193	-1.74646
H	-0.42961	-0.34457	-2.81526
H	-1.14075	-0.11274	-1.21377
C	0.64232	1.06531	-1.58007
H	-0.00025	1.90947	-1.89366
H	1.48205	1.03726	-2.29104
C	2.53983	0.84503	-0.00688
H	2.59009	-0.22307	0.27038
H	2.99345	1.43300	0.80896
H	3.16230	1.00249	-0.89936
Na	-3.89264	0.98151	0.01944

ACE1_Na_um062x_def2sv

26

N	1.20118	1.30437	-0.27582
C	0.30611	1.38447	0.85641
H	0.40074	2.36241	1.37753
H	-0.73269	1.34661	0.48579
C	0.51056	0.32467	1.95142
H	1.46266	0.51640	2.47030
H	-0.29931	0.43707	2.70092
O	0.60163	-1.00162	1.49740
C	-0.54886	-1.54495	0.90514
H	-0.99731	-2.31211	1.56516
H	-1.31611	-0.76570	0.75260
C	-0.16728	-2.19589	-0.41796
H	0.51281	-3.03896	-0.21778
H	-1.07742	-2.59911	-0.90617
O	0.53649	-1.32883	-1.26927
C	-0.18379	-0.21977	-1.74825
H	-0.42903	-0.35825	-2.81962
H	-1.14788	-0.11669	-1.21719
C	0.64238	1.05676	-1.58439
H	0.00156	1.90530	-1.89774
H	1.48372	1.02369	-2.29638
C	2.54542	0.85079	-0.00996
H	2.60776	-0.21428	0.28681
H	2.99703	1.45961	0.79590
H	3.16388	0.99869	-0.91053
Na	-3.88827	0.99382	0.02016

ACE1_Na_um062x_def2tzvpp

26

N	0.72505	1.59078	-0.15055
C	0.22757	1.16456	1.14284

H	0.21857	2.00780	1.84796
H	-0.81472	0.86124	1.03154
C	1.02439	0.06923	1.86296
H	1.99522	0.46509	2.16108
H	0.48197	-0.19392	2.77927
O	1.31726	-1.08541	1.10548
C	0.21299	-1.86847	0.71182
H	0.21270	-2.81820	1.25634
H	-0.72463	-1.36256	0.95056
C	0.31834	-2.15799	-0.77607
H	1.19930	-2.77263	-0.96203
H	-0.56595	-2.71998	-1.09945
O	0.49626	-0.99115	-1.54871
C	-0.59078	-0.08901	-1.53577
H	-1.12113	-0.13495	-2.49349
H	-1.31269	-0.36529	-0.76341
C	-0.10079	1.33726	-1.31342
H	-0.99534	1.97138	-1.28089
H	0.47097	1.64893	-2.18898
C	2.15959	1.53466	-0.33181
H	2.56046	0.51552	-0.36822
H	2.65272	2.06742	0.48516
H	2.42409	2.04501	-1.25782
Na	-4.16117	0.39280	0.53989

ACE1_Na_um062x_def2tzvp

26

N	-0.76850	1.58200	0.13095
C	-0.24309	1.15255	-1.15070
H	-0.24424	1.98834	-1.86580
H	0.80505	0.87546	-1.02192

C	-1.00357	0.03088	-1.86938
H	-1.98148	0.39890	-2.18182
H	-0.44385	-0.22583	-2.77784
O	-1.27509	-1.12538	-1.10603
C	-0.15500	-1.87481	-0.69182
H	-0.12496	-2.83109	-1.22547
H	0.77216	-1.34751	-0.92790
C	-0.26909	-2.15094	0.79811
H	-1.13528	-2.78836	0.98074
H	0.62738	-2.68445	1.13823
O	-0.48907	-0.98134	1.55578
C	0.57299	-0.04986	1.54883
H	1.09242	-0.07314	2.51421
H	1.31319	-0.31181	0.78809
C	0.04614	1.36012	1.30866
H	0.92237	2.02072	1.28655
H	-0.54857	1.66036	2.17352
C	-2.20406	1.49277	0.29206
H	-2.58135	0.46394	0.32626
H	-2.69808	2.01167	-0.53411
H	-2.49365	2.00022	1.21280
Na	4.15871	0.44164	-0.52861

ACE1_Na_um062x_def2tzv

26

N	0.79471	1.55523	-0.14156
C	0.25503	1.17122	1.15868
H	0.25958	2.02530	1.84962
H	-0.79325	0.89388	1.03393
C	1.01296	0.06024	1.90294
H	1.99930	0.41505	2.19278

H	0.45824	-0.18306	2.81445
O	1.27368	-1.14181	1.14030
C	0.11967	-1.88300	0.68948
H	0.06334	-2.82920	1.23094
H	-0.79635	-1.32927	0.89853
C	0.26601	-2.17580	-0.79471
H	1.14950	-2.78762	-0.96111
H	-0.61401	-2.72558	-1.14204
O	0.47331	-0.98697	-1.59067
C	-0.60420	-0.02037	-1.57134
H	-1.11749	-0.03833	-2.53546
H	-1.33775	-0.28007	-0.80586
C	-0.04219	1.37942	-1.32274
H	-0.89915	2.06176	-1.28165
H	0.55265	1.67345	-2.18812
C	2.24170	1.45493	-0.30345
H	2.60311	0.42212	-0.29140
H	2.74311	2.00793	0.49471
H	2.52881	1.91548	-1.24791
Na	-4.16659	0.45290	0.54357

ACE1_Na_um06_def2svpp

26

N	-1.15270	-1.34345	-0.06975
C	-0.42817	-1.13732	1.15654
H	-0.59853	-1.97245	1.87955
H	0.65680	-1.16841	0.93383
C	-0.77223	0.13631	1.93455
H	-1.78158	0.03519	2.37470
H	-0.06111	0.22831	2.78706
O	-0.81944	1.31736	1.18138

C	0.38271	1.76733	0.62381
H	0.72726	2.69353	1.13294
H	1.19073	1.02163	0.76624
C	0.18162	2.07457	-0.84859
H	-0.53342	2.91232	-0.94596
H	1.14981	2.41213	-1.28373
O	-0.37499	1.01454	-1.57563
C	0.39512	-0.15390	-1.66734
H	0.80034	-0.27081	-2.69667
H	1.27875	-0.10250	-0.99846
C	-0.44071	-1.37774	-1.32130
H	0.23907	-2.25955	-1.37021
H	-1.19073	-1.52948	-2.12073
C	-2.54945	-1.01258	-0.06793
H	-2.76195	0.08029	-0.05594
H	-3.04953	-1.46106	0.81621
H	-3.04078	-1.44179	-0.96117
Na	3.99871	-0.92804	0.41147

ACE1_Na_um06_def2svp

26

N	-1.13038	-1.35672	-0.10419
C	-0.40511	-1.17117	1.12529
H	-0.55305	-2.02575	1.82518
H	0.67698	-1.17572	0.90022
C	-0.76926	0.07268	1.93767
H	-1.77111	-0.06057	2.38033
H	-0.06201	0.15010	2.79092
O	-0.83951	1.27373	1.21646
C	0.35540	1.75610	0.66716
H	0.69381	2.66772	1.19971

H	1.17269	1.01931	0.78207
C	0.14696	2.10416	-0.79261
H	-0.57573	2.93444	-0.86461
H	1.10700	2.47288	-1.21280
O	-0.39136	1.05680	-1.55293
C	0.39754	-0.09836	-1.66729
H	0.81396	-0.18585	-2.69190
H	1.27380	-0.04966	-0.99244
C	-0.42030	-1.34179	-1.35748
H	0.27015	-2.20908	-1.43729
H	-1.16680	-1.48085	-2.15862
C	-2.53005	-1.03641	-0.09262
H	-2.75087	0.05029	-0.04386
H	-3.02936	-1.51433	0.77156
H	-3.01727	-1.43666	-0.99666
Na	4.00241	-0.91042	0.38623

ACE1_Na_um06_def2sv

26

N	-1.13002	-1.35506	-0.09166
C	-0.40906	-1.15825	1.13787
H	-0.56632	-2.00757	1.84720
H	0.67600	-1.17036	0.91462
C	-0.77240	0.09766	1.93599
H	-1.77998	-0.02695	2.37411
H	-0.06337	0.18716	2.79035
O	-0.83875	1.29014	1.20179
C	0.35573	1.76904	0.65178
H	0.68533	2.69224	1.17622
H	1.17697	1.03536	0.78144
C	0.14878	2.09708	-0.81526

H	-0.58042	2.92399	-0.89924
H	1.11088	2.45819	-1.24506
O	-0.38950	1.03968	-1.55942
C	0.39985	-0.11409	-1.66818
H	0.80787	-0.20818	-2.69877
H	1.28137	-0.05770	-0.99692
C	-0.41731	-1.35665	-1.34323
H	0.27639	-2.22667	-1.40720
H	-1.16482	-1.50539	-2.14549
C	-2.53187	-1.04670	-0.08484
H	-2.76231	0.04236	-0.05254
H	-3.02561	-1.51913	0.79040
H	-3.01550	-1.46703	-0.98645
Na	4.00338	-0.91200	0.39776

ACE1_Na_um06_def2tzvpp

26

N	0.68937	1.52777	-0.23926
C	0.14537	1.22894	1.05738
H	0.11127	2.13028	1.69205
H	-0.89736	0.92471	0.93271
C	0.90722	0.19869	1.88999
H	1.85301	0.63275	2.22196
H	0.31956	-0.01183	2.79504
O	1.26290	-0.98922	1.22845
C	0.20476	-1.81368	0.81462
H	0.17506	-2.72903	1.41934
H	-0.75971	-1.31732	0.96094
C	0.40036	-2.20578	-0.63290
H	1.31170	-2.80051	-0.72371
H	-0.44170	-2.83931	-0.94583

O	0.57095	-1.11117	-1.49811
C	-0.52307	-0.23153	-1.58606
H	-1.01254	-0.33733	-2.56319
H	-1.28094	-0.47816	-0.83568
C	-0.07279	1.20846	-1.41800
H	-0.98080	1.82767	-1.47160
H	0.53665	1.48641	-2.28149
C	2.11838	1.62104	-0.34579
H	2.63612	0.65307	-0.29232
H	2.51434	2.25494	0.45440
H	2.38769	2.09422	-1.29152
Na	-4.09547	0.41648	0.46233

ACE1_Na_um06_def2tzvp

26

N	0.74733	1.50790	-0.22185
C	0.18986	1.21873	1.07062
H	0.19247	2.11478	1.71610
H	-0.86598	0.95782	0.94352
C	0.90995	0.14920	1.89064
H	1.87299	0.54226	2.22835
H	0.31386	-0.04821	2.79479
O	1.21801	-1.04331	1.21477
C	0.12834	-1.82074	0.79345
H	0.06353	-2.74310	1.38718
H	-0.81666	-1.28850	0.94969
C	0.30554	-2.20267	-0.65931
H	1.18931	-2.83853	-0.75810
H	-0.56575	-2.79491	-0.97840
O	0.52503	-1.10661	-1.51052
C	-0.53167	-0.18229	-1.58987

H	-1.02715	-0.25781	-2.56830
H	-1.29849	-0.40547	-0.83991
C	-0.02275	1.23503	-1.40633
H	-0.90390	1.89417	-1.45899
H	0.60236	1.49557	-2.26550
C	2.17810	1.55656	-0.32269
H	2.66615	0.57163	-0.27070
H	2.59135	2.17509	0.48251
H	2.46645	2.02474	-1.26646
Na	-4.05458	0.50196	0.46947

ACE1_Na_um06_def2tzv

26

N	0.70638	1.48935	-0.26505
C	0.18360	1.24023	1.05783
H	0.17755	2.15947	1.66844
H	-0.86461	0.93781	0.96991
C	0.96896	0.22175	1.89687
H	1.93522	0.64425	2.17434
H	0.41041	0.02890	2.82188
O	1.29679	-1.01401	1.23372
C	0.19167	-1.83919	0.82858
H	0.16302	-2.74473	1.44349
H	-0.75759	-1.31825	0.98215
C	0.36287	-2.25513	-0.61950
H	1.28179	-2.83086	-0.72703
H	-0.48201	-2.89692	-0.90070
O	0.49957	-1.15540	-1.53918
C	-0.60205	-0.22781	-1.58537
H	-1.11865	-0.32594	-2.54594
H	-1.32979	-0.45882	-0.80260

C	-0.09841	1.20424	-1.43349
H	-0.98809	1.85233	-1.46946
H	0.50219	1.45386	-2.31149
C	2.13615	1.66235	-0.39532
H	2.70710	0.72921	-0.28770
H	2.50267	2.36531	0.36245
H	2.36855	2.08995	-1.37228
Na	-4.06182	0.47323	0.52691

ACE1_Na_ump2_def2svpp

26

N	-0.89705	-1.50576	-0.10642
C	-0.31239	-1.15476	1.16743
H	-0.38801	-1.99917	1.89166
H	0.76941	-0.98703	1.02530
C	-0.93924	0.04545	1.89584
H	-1.94305	-0.23373	2.25435
H	-0.32242	0.27000	2.79261
O	-1.14468	1.20056	1.11254
C	0.01690	1.85914	0.66178
H	0.14048	2.82837	1.18767
H	0.92140	1.26403	0.87906
C	-0.12074	2.12534	-0.83063
H	-0.95647	2.82641	-0.99179
H	0.80724	2.60596	-1.20765
O	-0.44866	0.96953	-1.56991
C	0.54422	-0.03362	-1.58229
H	1.04552	-0.06538	-2.57291
H	1.33178	0.17998	-0.83882
C	-0.08238	-1.39565	-1.29388
H	0.74780	-2.13473	-1.26412

H	-0.71573	-1.67619	-2.15365
C	-2.32401	-1.32916	-0.23698
H	-2.65166	-0.27131	-0.22605
H	-2.84705	-1.86155	0.58132
H	-2.65974	-1.78600	-1.18403
Na	4.09567	-0.58914	0.50393

ACE1_Na_ump2_def2svp

26

N	-0.83854	-1.53043	-0.11965
C	-0.28109	-1.15864	1.15999
H	-0.31755	-2.00323	1.87893
H	0.78722	-0.93659	1.02692
C	-0.96665	0.00482	1.88938
H	-1.95748	-0.31924	2.23329
H	-0.37192	0.24186	2.79173
O	-1.20821	1.15691	1.11375
C	-0.06570	1.85439	0.67622
H	0.03056	2.81817	1.20728
H	0.85123	1.28594	0.89053
C	-0.19581	2.12887	-0.81271
H	-1.04926	2.80025	-0.97735
H	0.71581	2.64515	-1.16725
O	-0.46933	0.96924	-1.56596
C	0.56067	0.00559	-1.56713
H	1.08066	-0.00375	-2.54290
H	1.32421	0.24398	-0.81185
C	-0.01482	-1.37992	-1.29570
H	0.84021	-2.08155	-1.26218
H	-0.62464	-1.67744	-2.16102
C	-2.26562	-1.38722	-0.27284

H	-2.61603	-0.34208	-0.27182
H	-2.78847	-1.92388	0.53485
H	-2.57570	-1.85256	-1.21792
Na	4.12140	-0.50896	0.51279

ACE1_Na_ump2_def2sv

26

N	-0.89705	-1.50576	-0.10642
C	-0.31239	-1.15476	1.16743
H	-0.38801	-1.99917	1.89166
H	0.76941	-0.98703	1.02530
C	-0.93924	0.04545	1.89584
H	-1.94305	-0.23373	2.25435
H	-0.32242	0.27000	2.79261
O	-1.14468	1.20056	1.11254
C	0.01690	1.85914	0.66178
H	0.14048	2.82837	1.18767
H	0.92140	1.26403	0.87906
C	-0.12074	2.12534	-0.83063
H	-0.95647	2.82641	-0.99179
H	0.80724	2.60596	-1.20765
O	-0.44866	0.96953	-1.56991
C	0.54422	-0.03362	-1.58229
H	1.04552	-0.06538	-2.57291
H	1.33178	0.17998	-0.83882
C	-0.08238	-1.39565	-1.29388
H	0.74780	-2.13473	-1.26412
H	-0.71573	-1.67619	-2.15365
C	-2.32401	-1.32916	-0.23698
H	-2.65166	-0.27131	-0.22605
H	-2.84705	-1.86155	0.58132

H	-2.65974	-1.78600	-1.18403
Na	4.09567	-0.58914	0.50393

ACE1_Na_ump2_def2tzvpp

26

N	0.83785	1.56754	-0.16350
C	0.15452	1.21080	1.06514
H	0.10915	2.07282	1.74058
H	-0.87960	0.96796	0.82948
C	0.77862	0.10287	1.91587
H	1.72785	0.44774	2.31474
H	0.11155	-0.07940	2.76347
O	1.08568	-1.10648	1.24599
C	-0.03511	-1.81079	0.74818
H	-0.18930	-2.72600	1.32242
H	-0.93950	-1.21444	0.84643
C	0.22485	-2.18361	-0.69679
H	1.06543	-2.86978	-0.74357
H	-0.65588	-2.68882	-1.10257
O	0.59700	-1.07353	-1.49317
C	-0.42014	-0.09599	-1.62438
H	-0.85449	-0.14465	-2.62566
H	-1.22760	-0.28671	-0.92071
C	0.14474	1.29790	-1.40714
H	-0.69635	1.98861	-1.51630
H	0.83940	1.52178	-2.21441
C	2.27110	1.36069	-0.16962
H	2.56596	0.31115	-0.12071
H	2.71695	1.88403	0.67502
H	2.68463	1.79647	-1.07523
Na	-4.03782	0.56322	0.36013

ACE1_Na_ump2_def2tzvp

26

N	0.83059	1.56425	-0.16723
C	0.16572	1.21000	1.07125
H	0.13506	2.07573	1.75058
H	-0.87734	0.97289	0.85133
C	0.79998	0.09868	1.91174
H	1.75626	0.44412	2.30491
H	0.13999	-0.08937	2.76940
O	1.10369	-1.10739	1.23407
C	-0.01944	-1.81805	0.74984
H	-0.16003	-2.73919	1.32703
H	-0.92989	-1.22655	0.86450
C	0.22414	-2.18654	-0.69970
H	1.06973	-2.87247	-0.75972
H	-0.66268	-2.69641	-1.09823
O	0.58338	-1.07334	-1.49844
C	-0.43861	-0.09900	-1.61827
H	-0.88345	-0.14814	-2.62006
H	-1.24266	-0.29546	-0.90641
C	0.12311	1.29702	-1.40267
H	-0.72583	1.98737	-1.49852
H	0.80734	1.52930	-2.22175
C	2.26625	1.38148	-0.18833
H	2.58311	0.33445	-0.13326
H	2.71615	1.92135	0.64914
H	2.66530	1.81673	-1.10521
Na	-4.03897	0.56184	0.37898

ACE1_Na_ump2_def2tzv

26

N	1.16630	1.43141	-0.02564
C	0.42601	1.11669	1.20738
H	0.54465	1.92498	1.95021
H	-0.64189	1.07871	0.96512
C	0.84351	-0.17125	1.95806
H	1.86104	-0.07963	2.34023
H	0.15632	-0.31178	2.80490
O	0.89488	-1.40083	1.14849
C	-0.38310	-1.83318	0.57146
H	-0.70493	-2.75866	1.06311
H	-1.15297	-1.07522	0.74343
C	-0.19006	-2.10847	-0.92067
H	0.53635	-2.90877	-1.06650
H	-1.15400	-2.40412	-1.35753
O	0.37450	-0.96685	-1.65790
C	-0.45418	0.24899	-1.64853
H	-0.88570	0.39195	-2.64725
H	-1.28052	0.13946	-0.93838
C	0.41147	1.47047	-1.29041
H	-0.26449	2.34071	-1.28610
H	1.14217	1.62969	-2.09045
C	2.57876	1.01006	-0.07501
H	2.71298	-0.07941	-0.05873
H	3.12221	1.44592	0.77241
H	3.02473	1.40656	-0.99161
Na	-4.06632	0.88922	0.47620

ACE1_Na_upbe0_def2svpp

26

N	1.43299	-0.81541	0.13757
C	0.64081	-0.85017	-1.07234

H	0.92263	-1.70572	-1.72255
H	-0.42060	-1.02381	-0.82340
C	0.77307	0.42903	-1.90103
H	1.81014	0.52064	-2.26678
H	0.11491	0.35342	-2.79372
O	0.52987	1.61391	-1.18903
C	-0.76345	1.79335	-0.67827
H	-1.26573	2.63582	-1.19654
H	-1.39363	0.90328	-0.85897
C	-0.70836	2.12892	0.80771
H	-0.21424	3.10724	0.93195
H	-1.74904	2.23362	1.18412
O	0.04114	1.23911	1.58781
C	-0.40363	-0.08974	1.65485
H	-0.80055	-0.30507	2.66747
H	-1.23913	-0.26417	0.95376
C	0.73894	-1.05916	1.37807
H	0.32603	-2.09596	1.45978
H	1.47830	-0.95629	2.19265
C	2.69790	-1.49180	0.03046
H	3.24357	-1.14328	-0.86408
H	2.60625	-2.60286	-0.04392
H	3.32736	-1.26557	0.90946
Na	-3.56335	-1.87910	-0.47265

ACE1_Na_upbe0_def2svp

26

N	1.42106	-0.83138	0.14007
C	0.62374	-0.85676	-1.06655
H	0.87922	-1.72142	-1.71197
H	-0.43845	-1.00375	-0.81243

C	0.77712	0.41183	-1.90434
H	1.81262	0.48291	-2.27372
H	0.11979	0.33736	-2.79522
O	0.55145	1.60641	-1.20086
C	-0.73704	1.80106	-0.68149
H	-1.23916	2.64017	-1.20115
H	-1.37562	0.91651	-0.84939
C	-0.67232	2.14735	0.79929
H	-0.16416	3.11762	0.91455
H	-1.70938	2.27640	1.17246
O	0.06052	1.24932	1.58729
C	-0.40302	-0.07419	1.65351
H	-0.81053	-0.28522	2.66066
H	-1.23575	-0.23870	0.94898
C	0.72541	-1.05882	1.38282
H	0.29996	-2.08757	1.47240
H	1.46479	-0.96412	2.19591
C	2.67291	-1.53199	0.03324
H	3.22455	-1.19969	-0.86026
H	2.56178	-2.63858	-0.03725
H	3.30740	-1.31738	0.90797
Na	-3.58736	-1.85196	-0.46385

ACE1_Na_upbe0_def2sv

26

N	1.43299	-0.81541	0.13757
C	0.64081	-0.85017	-1.07234
H	0.92263	-1.70572	-1.72255
H	-0.42060	-1.02381	-0.82340
C	0.77307	0.42903	-1.90103
H	1.81014	0.52064	-2.26678

H 0.11491 0.35342 -2.79372

O 0.52987 1.61391 -1.18903

C -0.76345 1.79335 -0.67827

H -1.26573 2.63582 -1.19654

H -1.39363 0.90328 -0.85897

C -0.70836 2.12892 0.80771

H -0.21424 3.10724 0.93195

H -1.74904 2.23362 1.18412

O 0.04114 1.23911 1.58781

C -0.40363 -0.08974 1.65485

H -0.80055 -0.30507 2.66747

H -1.23913 -0.26417 0.95376

C 0.73894 -1.05916 1.37807

H 0.32603 -2.09596 1.45978

H 1.47830 -0.95629 2.19265

C 2.69790 -1.49180 0.03046

H 3.24357 -1.14328 -0.86408

H 2.60625 -2.60286 -0.04392

H 3.32736 -1.26557 0.90946

Na -3.56335 -1.87910 -0.47265

ACE1_Na_upbe0_def2tzvpp

26

N -1.62686 0.58478 0.12425

C -0.64371 1.07697 -0.82090

H -0.94600 2.04515 -1.24445

H 0.30247 1.27587 -0.31178

C -0.41445 0.12407 -1.98826

H -1.33540 0.02959 -2.56790

H 0.35275 0.55134 -2.65003

O -0.07703 -1.19630 -1.61933

C	1.11510	-1.34419	-0.88432
H	1.85583	-1.89063	-1.48234
H	1.55821	-0.37103	-0.65877
C	0.87691	-2.14083	0.38635
H	0.56347	-3.15250	0.12019
H	1.83266	-2.21973	0.92476
O	-0.14383	-1.64770	1.22224
C	0.03983	-0.34444	1.72976
H	0.22010	-0.39508	2.81047
H	0.92664	0.12173	1.29209
C	-1.19136	0.51519	1.50089
H	-0.97860	1.51110	1.93544
H	-2.01806	0.09465	2.08139
C	-2.91436	1.21961	-0.01508
H	-3.25630	1.15054	-1.04967
H	-2.90381	2.28765	0.26621
H	-3.65073	0.71383	0.61333
Na	3.58399	2.02434	0.25250

ACE1_Na_upbe0_def2tzvp

26

N	1.58033	0.63599	-0.11442
C	0.71404	0.88167	1.02032
H	1.05782	1.74217	1.61267
H	-0.29036	1.14802	0.67971
C	0.63524	-0.32077	1.95681
H	1.61530	-0.52646	2.39438
H	-0.06723	-0.09353	2.77278
O	0.27572	-1.53461	1.32122
C	-0.99956	-1.55113	0.71894
H	-1.66903	-2.23526	1.25977

H	-1.46818	-0.55952	0.76487
C	-0.91858	-2.03079	-0.71720
H	-0.56822	-3.06605	-0.72359
H	-1.93077	-2.01262	-1.14757
O	-0.00831	-1.33717	-1.53374
C	-0.24777	0.04594	-1.71526
H	-0.52996	0.22490	-2.76091
H	-1.10366	0.37934	-1.12463
C	0.99738	0.86716	-1.41619
H	0.74346	1.93088	-1.59807
H	1.76728	0.61596	-2.15325
C	2.89120	1.21666	0.03230
H	3.32987	0.92393	0.98846
H	2.88026	2.32209	-0.00808
H	3.55299	0.86034	-0.76140
Na	-3.54117	2.01961	0.27524

ACE1_Na_upbe0_def2tzv

26

N	1.44026	0.80623	-0.14487
C	0.55678	0.90326	1.01468
H	0.76442	1.81338	1.59769
H	-0.48003	1.00520	0.68657
C	0.67733	-0.27795	1.97380
H	1.69016	-0.33235	2.37404
H	-0.01670	-0.12777	2.81128
O	0.45786	-1.57882	1.37279
C	-0.82560	-1.78328	0.74140
H	-1.37025	-2.55950	1.28964
H	-1.43232	-0.87606	0.78999
C	-0.66484	-2.26605	-0.69044

H	-0.13786	-3.22026	-0.69513
H	-1.66751	-2.42579	-1.10913
O	0.12062	-1.41683	-1.55710
C	-0.32746	-0.05018	-1.71550
H	-0.66433	0.08951	-2.74751
H	-1.18799	0.15211	-1.07256
C	0.80335	0.93456	-1.45182
H	0.39154	1.94899	-1.62452
H	1.57825	0.77927	-2.20760
C	2.65503	1.60216	-0.02328
H	3.16216	1.37366	0.91584
H	2.45972	2.68950	-0.04751
H	3.34115	1.36353	-0.83834
Na	-3.48979	2.02481	0.29753

ACE1_Na_upbe_def2svpp

26

N	0.72378	1.54407	-0.20907
C	0.21732	1.22691	1.10998
H	0.24520	2.12445	1.78835
H	-0.86055	0.96499	1.01642
C	0.97057	0.12243	1.89826
H	1.95927	0.51657	2.21849
H	0.38948	-0.09561	2.83208
O	1.28065	-1.06312	1.18722
C	0.19249	-1.86936	0.77441
H	0.18141	-2.83140	1.34680
H	-0.78159	-1.36993	0.98347
C	0.32709	-2.20465	-0.71646
H	1.24516	-2.81334	-0.86888
H	-0.54959	-2.83394	-1.02134

O	0.48769	-1.07574	-1.55680
C	-0.59652	-0.16199	-1.57933
H	-1.13228	-0.22027	-2.56141
H	-1.34645	-0.41981	-0.79679
C	-0.10378	1.28177	-1.36894
H	-1.02009	1.92971	-1.36018
H	0.48045	1.58920	-2.26408
C	2.15979	1.59030	-0.37147
H	2.66231	0.58954	-0.33547
H	2.61755	2.22547	0.42539
H	2.41210	2.06318	-1.34661
Na	-4.06522	0.45193	0.53502

ACE1_Na_upbe_def2svp

26

N	0.75521	1.52721	-0.22228
C	0.19911	1.24255	1.08417
H	0.21184	2.14967	1.74629
H	-0.87703	0.99332	0.96416
C	0.90903	0.14602	1.91746
H	1.89077	0.53069	2.26254
H	0.29881	-0.04171	2.83636
O	1.22102	-1.05906	1.23828
C	0.12827	-1.84427	0.79457
H	0.05876	-2.78981	1.38544
H	-0.83734	-1.31335	0.94738
C	0.31187	-2.21609	-0.67933
H	1.22328	-2.84064	-0.78568
H	-0.55898	-2.84349	-0.99639
O	0.51335	-1.10890	-1.54192
C	-0.55229	-0.17322	-1.60520

H	-1.06881	-0.23822	-2.59430
H	-1.32369	-0.40021	-0.83653
C	-0.03479	1.26098	-1.40703
H	-0.93132	1.93044	-1.45135
H	0.58890	1.53537	-2.28350
C	2.19625	1.54558	-0.33740
H	2.67820	0.53922	-0.27522
H	2.64118	2.17928	0.46320
H	2.48940	2.00096	-1.30582
Na	-4.05364	0.49931	0.48229

ACE1_Na_upbe_def2sv

26

N	0.78343	1.51345	-0.21518
C	0.20704	1.24294	1.08458
H	0.24271	2.15167	1.74778
H	-0.87648	1.02510	0.95093
C	0.88137	0.12506	1.92353
H	1.86744	0.48925	2.28490
H	0.25021	-0.05773	2.83207
O	1.18231	-1.08153	1.24474
C	0.08602	-1.85427	0.79101
H	0.01145	-2.80537	1.37674
H	-0.87734	-1.31498	0.94493
C	0.28008	-2.21981	-0.68591
H	1.18505	-2.85868	-0.78322
H	-0.60002	-2.82839	-1.02155
O	0.51497	-1.11256	-1.53783
C	-0.53372	-0.16156	-1.62003
H	-1.02907	-0.21686	-2.62308
H	-1.32482	-0.37899	-0.86625

C 0.00265 1.26612 -1.40931

H -0.88728 1.94860 -1.45544

H 0.64051 1.53546 -2.27956

C 2.22524 1.53404 -0.30920

H 2.70990 0.52579 -0.24372

H 2.65501 2.16665 0.50471

H 2.53286 1.99604 -1.27344

Na -4.04132 0.54417 0.46430

ACE1_Na_upbe_def2tzvpp

26

N -0.48020 1.71024 0.26258

C -0.70360 1.58846 -1.16481

H -1.64084 2.12217 -1.38571

H 0.09760 2.12245 -1.71297

C -0.77165 0.18582 -1.80233

H -1.13431 0.30249 -2.84110

H 0.23838 -0.24822 -1.86742

O -1.64131 -0.70774 -1.09964

C -1.06071 -1.94503 -0.69320

H -1.72213 -2.77101 -1.00880

H -0.10079 -2.09992 -1.21227

C -0.89629 -2.07002 0.82660

H -1.88533 -2.17866 1.29497

H -0.33213 -3.00588 1.01706

O -0.30764 -0.95882 1.49693

C 0.86659 -0.38732 0.92188

H 1.74647 -0.62671 1.55064

H 1.06774 -0.80939 -0.07198

C 0.72252 1.14838 0.84480

H 1.60293 1.52493 0.29010

H	0.80039	1.54824	1.86934
C	-1.67315	1.72232	1.09627
H	-2.19768	0.75435	1.16949
H	-2.38095	2.46814	0.70041
H	-1.39582	2.03234	2.11513
Na	4.29884	-0.11184	-0.46350

ACE1_Na_upbe_def2tzvp

26

N	-0.48068	1.70993	0.26354
C	-0.70409	1.58926	-1.16408
H	-1.64220	2.12305	-1.38459
H	0.09758	2.12406	-1.71224
C	-0.77206	0.18698	-1.80236
H	-1.13583	0.30434	-2.84156
H	0.23876	-0.24682	-1.86861
O	-1.64135	-0.70679	-1.09927
C	-1.06116	-1.94487	-0.69393
H	-1.72464	-2.77052	-1.00893
H	-0.10149	-2.10118	-1.21457
C	-0.89583	-2.07016	0.82582
H	-1.88554	-2.17855	1.29460
H	-0.33137	-3.00699	1.01564
O	-0.30696	-0.95910	1.49641
C	0.86718	-0.38724	0.92150
H	1.74786	-0.62657	1.55071
H	1.06881	-0.80887	-0.07322
C	0.72268	1.14845	0.84490
H	1.60314	1.52533	0.28896
H	0.80153	1.54846	1.87009
C	-1.67393	1.71995	1.09709

H	-2.19947	0.75157	1.16647
H	-2.38142	2.46851	0.70383
H	-1.39635	2.02589	2.11783
Na	4.29956	-0.11166	-0.46364

ACE1_Na_upbe_def2tzv

26

N	-0.69891	1.66849	0.23866
C	-0.64306	1.56736	-1.21787
H	-1.56914	2.02054	-1.60524
H	0.19567	2.18479	-1.59813
C	-0.46220	0.17618	-1.87112
H	-0.67997	0.26606	-2.94995
H	0.58212	-0.16159	-1.77463
O	-1.36466	-0.84128	-1.30481
C	-0.70874	-2.02812	-0.74543
H	-1.20423	-2.91907	-1.16638
H	0.34309	-2.06128	-1.07252
C	-0.82736	-2.13576	0.77770
H	-1.87771	-2.28791	1.06070
H	-0.25403	-3.03280	1.08361
O	-0.41864	-0.96833	1.56561
C	0.78510	-0.24113	1.14547
H	1.57373	-0.38550	1.90612
H	1.17062	-0.63101	0.19424
C	0.45942	1.26713	1.03385
H	1.36402	1.76231	0.63050
H	0.30829	1.65916	2.05351
C	-2.02637	1.53930	0.85645
H	-2.43866	0.51628	0.84110
H	-2.73228	2.20754	0.33606

H	-1.96607	1.86657	1.90595
Na	4.26205	0.08397	-0.31603

ACE1_Na_uscs-mp2_def2svpp

26

N	1.44763	0.75918	-0.14086
C	0.58754	0.88872	1.02938
H	0.85039	1.78496	1.63310
H	-0.45929	1.04927	0.71993
C	0.67733	-0.33815	1.94234
H	1.69939	-0.40916	2.35423
H	-0.02427	-0.21543	2.79685
O	0.46093	-1.57063	1.28011
C	-0.82913	-1.76032	0.73279
H	-1.36113	-2.56313	1.28543
H	-1.44709	-0.84993	0.83263
C	-0.71264	-2.18956	-0.72916
H	-0.22767	-3.18030	-0.76863
H	-1.73419	-2.29390	-1.15631
O	0.10078	-1.35051	-1.52374
C	-0.34929	-0.01996	-1.69322
H	-0.69468	0.12831	-2.73764
H	-1.21839	0.19351	-1.04614
C	0.79049	0.96068	-1.42142
H	0.38866	1.99797	-1.54920
H	1.55415	0.81654	-2.20839
C	2.67534	1.52531	-0.02263
H	3.18079	1.27670	0.92790
H	2.50943	2.62936	-0.04487
H	3.36318	1.26504	-0.84767
Na	-3.45857	2.00226	0.33776

ACE1_Na_uscs-mp2_def2svp

26

N	1.44302	0.76706	-0.14138
C	0.58000	0.88954	1.02596
H	0.82832	1.78555	1.62710
H	-0.46343	1.03484	0.71370
C	0.67750	-0.33190	1.94250
H	1.69398	-0.39173	2.35844
H	-0.02438	-0.20666	2.79037
O	0.46988	-1.56740	1.28614
C	-0.81543	-1.76077	0.73256
H	-1.35204	-2.55730	1.28020
H	-1.43152	-0.85347	0.82319
C	-0.69674	-2.19307	-0.72630
H	-0.21468	-3.18057	-0.76312
H	-1.71628	-2.30012	-1.14548
O	0.11101	-1.35422	-1.52425
C	-0.34508	-0.02687	-1.69194
H	-0.69532	0.12381	-2.72935
H	-1.20849	0.18179	-1.04263
C	0.78619	0.96089	-1.42199
H	0.37641	1.98951	-1.55103
H	1.54924	0.82456	-2.20492
C	2.66210	1.54271	-0.02201
H	3.16808	1.30173	0.92395
H	2.48713	2.63981	-0.04345
H	3.34979	1.29262	-0.84306
Na	-3.47148	1.98508	0.33345

ACE1_Na_uscs-mp2_def2sv

26

N 1.44763 0.75918 -0.14086

C 0.58754 0.88872 1.02938

H 0.85039 1.78496 1.63310

H -0.45929 1.04927 0.71993

C 0.67733 -0.33815 1.94234

H 1.69939 -0.40916 2.35423

H -0.02427 -0.21543 2.79685

O 0.46093 -1.57063 1.28011

C -0.82913 -1.76032 0.73279

H -1.36113 -2.56313 1.28543

H -1.44709 -0.84993 0.83263

C -0.71264 -2.18956 -0.72916

H -0.22767 -3.18030 -0.76863

H -1.73419 -2.29390 -1.15631

O 0.10078 -1.35051 -1.52374

C -0.34929 -0.01996 -1.69322

H -0.69468 0.12831 -2.73764

H -1.21839 0.19351 -1.04614

C 0.79049 0.96068 -1.42142

H 0.38866 1.99797 -1.54920

H 1.55415 0.81654 -2.20839

C 2.67534 1.52531 -0.02263

H 3.18079 1.27670 0.92790

H 2.50943 2.62936 -0.04487

H 3.36318 1.26504 -0.84767

Na -3.45857 2.00226 0.33776

ACE1_Na_uscs-mp2_def2tzvpp

26

N -1.63070 0.48451 0.09415

C -0.66680 0.97008 -0.89071

H	-1.01707	1.89148	-1.36616
H	0.26882	1.23193	-0.40307
C	-0.41220	-0.04763	-1.99897
H	-1.33126	-0.21425	-2.55551
H	0.33710	0.35450	-2.68795
O	-0.02426	-1.33564	-1.53729
C	1.19693	-1.36838	-0.81599
H	1.94446	-1.92431	-1.38612
H	1.59071	-0.36506	-0.67239
C	1.00882	-2.08091	0.51590
H	0.74601	-3.11883	0.32894
H	1.96079	-2.06072	1.05565
O	-0.04557	-1.57304	1.31762
C	0.11048	-0.22459	1.73628
H	0.30928	-0.19973	2.80929
H	0.96695	0.23582	1.24761
C	-1.16197	0.56760	1.47025
H	-0.97844	1.60586	1.79046
H	-1.95411	0.17119	2.10484
C	-2.92342	1.13717	-0.05240
H	-3.28111	1.01314	-1.07194
H	-2.88031	2.21272	0.16840
H	-3.64410	0.67700	0.61995
Na	3.27497	2.24065	0.12092

ACE1_Na_uscs-mp2_def2tzvp

26

N	-1.62877	0.48780	0.09547
C	-0.66681	0.97418	-0.89088
H	-1.02088	1.89849	-1.36869
H	0.27202	1.24184	-0.40310

C -0.41209 -0.04407 -1.99924

H -1.33395 -0.21010 -2.56022

H 0.34151 0.35805 -2.69096

O -0.02743 -1.33364 -1.53783

C 1.19548 -1.37200 -0.81867

H 1.94192 -1.93421 -1.39340

H 1.59712 -0.36684 -0.67758

C 1.00654 -2.08358 0.51411

H 0.74027 -3.12562 0.32832

H 1.96271 -2.06567 1.05592

O -0.04685 -1.57352 1.31656

C 0.11123 -0.22576 1.73865

H 0.30811 -0.20464 2.81701

H 0.97477 0.23510 1.25361

C -1.15980 0.56926 1.47165

H -0.97489 1.61131 1.79474

H -1.95589 0.17289 2.10882

C -2.92447 1.13463 -0.05159

H -3.28198 1.01158 -1.07567

H -2.88837 2.21400 0.17250

H -3.64832 0.66848 0.61979

Na 3.27828 2.23836 0.12151

ACE1_Na_uscs-mp2_def2tzv

26

N -1.67762 0.45510 0.10293

C -0.77065 0.87626 -0.99420

H -1.18537 1.74103 -1.54095

H 0.18739 1.21371 -0.58415

C -0.53380 -0.24456 -2.02584

H -1.47587 -0.53269 -2.49738

H	0.16669	0.11184	-2.79539
O	-0.02960	-1.50760	-1.45033
C	1.26016	-1.40526	-0.75194
H	2.00624	-1.98520	-1.30988
H	1.59984	-0.36544	-0.72673
C	1.16135	-2.00217	0.66031
H	0.90646	-3.06197	0.59969
H	2.13955	-1.89148	1.15115
O	0.10367	-1.43503	1.51252
C	0.19081	0.01126	1.77060
H	0.42114	0.15735	2.83298
H	1.01029	0.45095	1.19432
C	-1.14972	0.70269	1.46652
H	-1.00733	1.78640	1.66364
H	-1.90313	0.33285	2.17112
C	-3.04833	1.00264	-0.05510
H	-3.42804	0.74329	-1.04769
H	-3.07821	2.10274	0.05692
H	-3.70631	0.55386	0.69467
Na	3.25805	2.30481	-0.13659

ACE1_nm_ub2plyp_def2svpp

25

N	-1.42047	-0.11217	-0.13694
C	-1.01963	0.93707	0.78925
H	-1.89883	1.45974	1.21960
H	-0.49824	0.49845	1.65454
C	-0.12636	1.99426	0.12897
H	-0.70209	2.52342	-0.64754
H	0.17305	2.74280	0.89222
O	1.00379	1.47605	-0.54422

C	1.95934	0.80031	0.24540
H	2.90893	1.37103	0.26074
H	1.62739	0.72074	1.29397
C	2.25366	-0.57968	-0.34163
H	2.72461	-0.45033	-1.32917
H	2.98625	-1.09320	0.31573
O	1.11713	-1.37997	-0.58132
C	0.34174	-1.75223	0.53956
H	0.46020	-2.83601	0.73395
H	0.68534	-1.23561	1.45117
C	-1.13786	-1.47356	0.27893
H	-1.70593	-1.78181	1.19064
H	-1.46918	-2.13949	-0.53641
C	-2.76038	0.05872	-0.65735
H	-2.88042	1.07635	-1.06525
H	-3.55642	-0.09466	0.10949
H	-2.94178	-0.65418	-1.47961

ACE1_nm_ub2plyp_def2svp

25

N	-1.42048	-0.11234	-0.13852
C	-1.01614	0.93595	0.78661
H	-1.89015	1.45683	1.22351
H	-0.49019	0.49763	1.64678
C	-0.12680	1.99429	0.12699
H	-0.70299	2.52441	-0.64559
H	0.16751	2.74137	0.89035
O	1.00529	1.47856	-0.54518
C	1.95516	0.79751	0.24706
H	2.90424	1.36407	0.27584
H	1.61669	0.71128	1.29097

C	2.25312	-0.57884	-0.34138
H	2.72813	-0.44787	-1.32449
H	2.98553	-1.08720	0.31635
O	1.11885	-1.38214	-0.58263
C	0.34149	-1.74853	0.53908
H	0.46052	-2.82785	0.74484
H	0.68060	-1.22531	1.44635
C	-1.13643	-1.47318	0.27711
H	-1.70225	-1.78209	1.18703
H	-1.46856	-2.13931	-0.53490
C	-2.76214	0.05740	-0.65330
H	-2.88678	1.07080	-1.06172
H	-3.55317	-0.09438	0.11437
H	-2.94847	-0.65503	-1.47062

ACE1_nm_ub2plyp_def2sv

25

N	-1.42060	-0.11213	-0.13682
C	-1.01967	0.93710	0.78929
H	-1.89879	1.45998	1.21951
H	-0.49852	0.49840	1.65468
C	-0.12616	1.99412	0.12882
H	-0.70204	2.52325	-0.64762
H	0.17344	2.74274	0.89187
O	1.00375	1.47564	-0.54438
C	1.95936	0.80027	0.24554
H	2.90880	1.37120	0.26091
H	1.62722	0.72081	1.29404
C	2.25378	-0.57979	-0.34150
H	2.72503	-0.45040	-1.32889
H	2.98601	-1.09362	0.31595

O	1.11699	-1.37956	-0.58147
C	0.34185	-1.75202	0.53952
H	0.46047	-2.83579	0.73385
H	0.68547	-1.23541	1.45111
C	-1.13786	-1.47348	0.27894
H	-1.70593	-1.78191	1.19054
H	-1.46889	-2.13940	-0.53651
C	-2.76050	0.05865	-0.65724
H	-2.88082	1.07651	-1.06441
H	-3.55653	-0.09560	0.10937
H	-2.94153	-0.65369	-1.48006

ACE1_nm_ub2plyp_def2tzvpp

25

N	-1.42183	-0.10263	-0.17243
C	-1.00944	0.93921	0.76279
H	-1.87715	1.45765	1.18706
H	-0.49438	0.49707	1.61422
C	-0.12147	1.99897	0.11258
H	-0.68140	2.52104	-0.66177
H	0.16960	2.73276	0.87304
O	1.02855	1.48782	-0.55208
C	1.95391	0.78700	0.26498
H	2.89398	1.34482	0.31338
H	1.58536	0.70420	1.28654
C	2.26507	-0.58389	-0.32057
H	2.75251	-0.45796	-1.28584
H	2.96900	-1.08997	0.35062
O	1.13426	-1.39964	-0.58625
C	0.33193	-1.74193	0.53608
H	0.44139	-2.80947	0.74536

H	0.66924	-1.21321	1.42668
C	-1.13951	-1.46316	0.25876
H	-1.70173	-1.74656	1.16586
H	-1.47261	-2.12935	-0.53852
C	-2.79865	0.05053	-0.60811
H	-2.95154	1.05176	-1.00785
H	-3.52478	-0.10514	0.20528
H	-3.01819	-0.66510	-1.39944

ACE1_nm_ub2plyp_def2tzvp

25

N	-1.42209	-0.10259	-0.17172
C	-1.01055	0.93990	0.76313
H	-1.87992	1.45992	1.18591
H	-0.49738	0.49775	1.61734
C	-0.12108	1.99893	0.11353
H	-0.68102	2.52320	-0.66135
H	0.17155	2.73275	0.87553
O	1.02803	1.48696	-0.55227
C	1.95526	0.78732	0.26405
H	2.89645	1.34658	0.30962
H	1.58857	0.70598	1.28782
C	2.26541	-0.58416	-0.32082
H	2.75319	-0.45927	-1.28769
H	2.96990	-1.09128	0.35143
O	1.13369	-1.39899	-0.58623
C	0.33188	-1.74251	0.53637
H	0.44135	-2.81198	0.74388
H	0.67062	-1.21527	1.42889
C	-1.13961	-1.46303	0.25976
H	-1.70244	-1.74662	1.16823

H -1.47359 -2.13024 -0.53814

C -2.79828 0.05041 -0.60969

H -2.95124 1.05347 -1.00827

H -3.52694 -0.10743 0.20281

H -3.01642 -0.66435 -1.40392

ACE1_nm_ub2plyp_def2tzv

25

N -1.44206 -0.11032 -0.16234

C -1.02702 0.94627 0.77935

H -1.90035 1.45116 1.21582

H -0.49206 0.50760 1.62215

C -0.16132 2.03026 0.12360

H -0.71773 2.53080 -0.66617

H 0.12363 2.76954 0.88011

O 1.04050 1.52980 -0.55459

C 1.99104 0.79323 0.27648

H 2.92209 1.36351 0.32889

H 1.61351 0.69187 1.29371

C 2.31322 -0.56697 -0.33920

H 2.76362 -0.43013 -1.31976

H 3.03275 -1.07577 0.31214

O 1.16158 -1.43272 -0.59501

C 0.32545 -1.77236 0.55798

H 0.43758 -2.83975 0.76082

H 0.66449 -1.23729 1.44512

C -1.14973 -1.49062 0.26332

H -1.71922 -1.77725 1.16746

H -1.47428 -2.15031 -0.54328

C -2.82728 0.04691 -0.63696

H -2.96744 1.05032 -1.03793

H	-3.57027	-0.11145	0.16259
H	-3.02468	-0.66763	-1.43590

ACE1_nm_ub3lyp_def2svpp

25

N	-1.43431	-0.11385	-0.12314
C	-1.02652	0.94255	0.79318
H	-1.90357	1.47285	1.22210
H	-0.50743	0.50645	1.66289
C	-0.12630	1.99984	0.13150
H	-0.69912	2.53188	-0.64767
H	0.16743	2.75021	0.89775
O	1.00854	1.48701	-0.53868
C	1.96889	0.80334	0.23914
H	2.92288	1.37028	0.24402
H	1.64981	0.72601	1.29373
C	2.25729	-0.58186	-0.34900
H	2.71913	-0.45510	-1.34330
H	3.00233	-1.08911	0.30247
O	1.12405	-1.39101	-0.57516
C	0.34105	-1.75981	0.54236
H	0.45395	-2.84600	0.73589
H	0.68441	-1.24712	1.45834
C	-1.14185	-1.47815	0.28048
H	-1.70914	-1.80093	1.18998
H	-1.47195	-2.13978	-0.54081
C	-2.76288	0.06003	-0.67099
H	-2.87445	1.07648	-1.08886
H	-3.57858	-0.08555	0.08020
H	-2.93439	-0.65731	-1.49396

ACE1_nm_ub3lyp_def2svp

25

N	-1.43426	-0.11374	-0.12441
C	-1.02307	0.94185	0.79113
H	-1.89529	1.47041	1.22694
H	-0.49931	0.50655	1.65637
C	-0.12698	2.00016	0.12946
H	-0.70054	2.53258	-0.64646
H	0.16161	2.75080	0.89466
O	1.01023	1.48916	-0.53964
C	1.96559	0.80029	0.24107
H	2.91925	1.36389	0.25889
H	1.64072	0.71645	1.29167
C	2.25728	-0.58105	-0.34931
H	2.72246	-0.45213	-1.33990
H	3.00340	-1.08414	0.30096
O	1.12542	-1.39305	-0.57648
C	0.34046	-1.75638	0.54245
H	0.45448	-2.83859	0.74699
H	0.67938	-1.23758	1.45474
C	-1.14066	-1.47798	0.27872
H	-1.70680	-1.80243	1.18583
H	-1.47108	-2.14002	-0.53976
C	-2.76505	0.05880	-0.66739
H	-2.88138	1.07112	-1.08649
H	-3.57650	-0.08531	0.08436
H	-2.94116	-0.65846	-1.48569

ACE1_nm_ub3lyp_def2sv

25

N	-1.43437	-0.11386	-0.12280
C	-1.02666	0.94261	0.79333

H	-1.90368	1.47315	1.22200
H	-0.50789	0.50655	1.66323
C	-0.12615	1.99975	0.13147
H	-0.69915	2.53183	-0.64756
H	0.16779	2.75016	0.89756
O	1.00838	1.48667	-0.53879
C	1.96887	0.80336	0.23918
H	2.92275	1.37045	0.24398
H	1.64977	0.72616	1.29375
C	2.25730	-0.58196	-0.34897
H	2.71937	-0.45513	-1.34314
H	3.00208	-1.08947	0.30253
O	1.12382	-1.39059	-0.57534
C	0.34120	-1.75975	0.54232
H	0.45424	-2.84595	0.73567
H	0.68471	-1.24720	1.45830
C	-1.14185	-1.47808	0.28072
H	-1.70904	-1.80105	1.19017
H	-1.47180	-2.13971	-0.54064
C	-2.76265	0.05994	-0.67125
H	-2.87436	1.07666	-1.08835
H	-3.57874	-0.08661	0.07928
H	-2.93339	-0.65673	-1.49494

ACE1_nm_ub3lyp_def2tzvpp

25

N	-1.43447	-0.10544	-0.15565
C	-1.01791	0.94461	0.76887
H	-1.88440	1.46924	1.19177
H	-0.50468	0.50720	1.62548
C	-0.12401	2.00393	0.11669

H	-0.68151	2.52928	-0.65952
H	0.16362	2.74014	0.87862
O	1.02927	1.49664	-0.54670
C	1.96445	0.79180	0.25710
H	2.90730	1.34959	0.29232
H	1.61162	0.71120	1.28597
C	2.26974	-0.58363	-0.32895
H	2.74760	-0.46102	-1.30149
H	2.98630	-1.08497	0.33541
O	1.14076	-1.40739	-0.58022
C	0.33242	-1.75082	0.53861
H	0.43822	-2.82157	0.74275
H	0.67025	-1.22972	1.43525
C	-1.14200	-1.46854	0.26295
H	-1.70284	-1.76730	1.16824
H	-1.47421	-2.13052	-0.54007
C	-2.79927	0.05062	-0.62564
H	-2.94219	1.05152	-1.03395
H	-3.55083	-0.10033	0.16827
H	-3.00376	-0.66648	-1.42193

ACE1_nm_ub3lyp_def2tzvp

25

N	-1.43473	-0.10540	-0.15526
C	-1.01828	0.94496	0.76900
H	-1.88551	1.47059	1.19139
H	-0.50524	0.50742	1.62665
C	-0.12375	2.00384	0.11692
H	-0.68149	2.52984	-0.65985
H	0.16454	2.74004	0.87989
O	1.02900	1.49586	-0.54693

C	1.96501	0.79183	0.25684
H	2.90853	1.35034	0.29078
H	1.61259	0.71135	1.28672
C	2.27003	-0.58375	-0.32898
H	2.74785	-0.46113	-1.30247
H	2.98689	-1.08547	0.33623
O	1.14046	-1.40686	-0.58025
C	0.33240	-1.75076	0.53878
H	0.43811	-2.82254	0.74237
H	0.67041	-1.22964	1.43633
C	-1.14198	-1.46851	0.26317
H	-1.70311	-1.76792	1.16914
H	-1.47397	-2.13081	-0.54076
C	-2.79927	0.05058	-0.62608
H	-2.94216	1.05250	-1.03396
H	-3.55201	-0.10147	0.16770
H	-3.00290	-0.66643	-1.42375

ACE1_nm_ub3lyp_def2tzv

25

N	-1.45284	-0.11377	-0.14442
C	-1.03156	0.94866	0.78287
H	-1.90109	1.46051	1.21864
H	-0.49917	0.51582	1.63029
C	-0.16256	2.02890	0.12389
H	-0.71991	2.53002	-0.66565
H	0.11557	2.77332	0.87870
O	1.03853	1.53325	-0.55034
C	1.99375	0.79674	0.26979
H	2.92675	1.36575	0.31216
H	1.63038	0.69934	1.29276

C	2.31216	-0.56498	-0.34315
H	2.75822	-0.42859	-1.32657
H	3.04317	-1.06695	0.30175
O	1.16721	-1.43564	-0.58874
C	0.32692	-1.77401	0.55780
H	0.43682	-2.84220	0.76006
H	0.66278	-1.24362	1.44906
C	-1.14818	-1.49372	0.26261
H	-1.71787	-1.80312	1.15979
H	-1.46557	-2.14804	-0.55164
C	-2.82262	0.04672	-0.64770
H	-2.95482	1.04690	-1.06048
H	-3.58734	-0.10218	0.13440
H	-3.01130	-0.67133	-1.44642

ACE1_nm_ub971_def2svpp

25

N	-1.43068	-0.11429	-0.12579
C	-1.02514	0.94084	0.79500
H	-1.90432	1.46992	1.22523
H	-0.50398	0.50243	1.66482
C	-0.12446	2.00097	0.13137
H	-0.70072	2.53359	-0.64724
H	0.17101	2.75177	0.89892
O	1.00697	1.48802	-0.54028
C	1.96462	0.80611	0.23947
H	2.92038	1.37303	0.24460
H	1.64364	0.73013	1.29569
C	2.25450	-0.58346	-0.34725
H	2.72150	-0.45720	-1.34089
H	2.99729	-1.09105	0.30916

O	1.12273	-1.39029	-0.57737
C	0.34407	-1.76177	0.54000
H	0.45760	-2.84994	0.73112
H	0.68894	-1.24992	1.45841
C	-1.14265	-1.47936	0.28196
H	-1.70870	-1.79818	1.19526
H	-1.47643	-2.14263	-0.53882
C	-2.76197	0.06122	-0.67007
H	-2.87141	1.07956	-1.08813
H	-3.57629	-0.08345	0.08465
H	-2.93501	-0.65714	-1.49380

ACE1_nm_ub971_def2svp

25

N	-1.43097	-0.11419	-0.12669
C	-1.02189	0.94032	0.79309
H	-1.89600	1.46772	1.22997
H	-0.49663	0.50285	1.65859
C	-0.12505	2.00141	0.12944
H	-0.70183	2.53434	-0.64580
H	0.16508	2.75235	0.89581
O	1.00877	1.49022	-0.54111
C	1.96171	0.80316	0.24125
H	2.91695	1.36656	0.25913
H	1.63528	0.72091	1.29343
C	2.25464	-0.58272	-0.34774
H	2.72465	-0.45443	-1.33756
H	2.99857	-1.08601	0.30713
O	1.12411	-1.39238	-0.57857
C	0.34345	-1.75868	0.54011
H	0.45791	-2.84272	0.74194

H	0.68401	-1.24132	1.45494
C	-1.14161	-1.47934	0.28031
H	-1.70634	-1.80001	1.19101
H	-1.47580	-2.14265	-0.53757
C	-2.76423	0.06013	-0.66686
H	-2.87811	1.07416	-1.08612
H	-3.57469	-0.08296	0.08771
H	-2.94146	-0.65789	-1.48594

ACE1_nm_ub971_def2sv

25

N	-1.43078	-0.11435	-0.12551
C	-1.02536	0.94079	0.79515
H	-1.90450	1.47005	1.22522
H	-0.50447	0.50231	1.66509
C	-0.12442	2.00078	0.13133
H	-0.70092	2.53337	-0.64713
H	0.17122	2.75168	0.89868
O	1.00671	1.48761	-0.54042
C	1.96456	0.80617	0.23950
H	2.92015	1.37333	0.24460
H	1.64354	0.73024	1.29570
C	2.25460	-0.58345	-0.34728
H	2.72176	-0.45706	-1.34082
H	2.99720	-1.09126	0.30910
O	1.12263	-1.38985	-0.57748
C	0.34433	-1.76141	0.54010
H	0.45815	-2.84951	0.73140
H	0.68923	-1.24931	1.45836
C	-1.14255	-1.47932	0.28208
H	-1.70860	-1.79845	1.19522

H	-1.47588	-2.14259	-0.53886
C	-2.76184	0.06099	-0.67028
H	-2.87151	1.07961	-1.08751
H	-3.57647	-0.08477	0.08382
H	-2.93408	-0.65667	-1.49476

ACE1_nm_ub971_def2tzvpp

25

N	-1.43102	-0.10410	-0.16364
C	-1.01710	0.94409	0.76715
H	-1.88635	1.47015	1.18805
H	-0.50557	0.50378	1.62660
C	-0.11847	2.00511	0.11471
H	-0.67908	2.53310	-0.66096
H	0.17040	2.74096	0.87999
O	1.03039	1.49662	-0.54940
C	1.95991	0.79377	0.25865
H	2.90637	1.34986	0.29643
H	1.60324	0.71502	1.28918
C	2.26703	-0.58771	-0.32496
H	2.75319	-0.46506	-1.29599
H	2.98052	-1.08875	0.34676
O	1.14020	-1.40810	-0.58238
C	0.33582	-1.75056	0.53641
H	0.44027	-2.82354	0.74227
H	0.67467	-1.22773	1.43501
C	-1.14307	-1.46743	0.26252
H	-1.70364	-1.75996	1.17242
H	-1.47861	-2.13345	-0.53903
C	-2.80292	0.05010	-0.61880
H	-2.95031	1.05356	-1.02515

H	-3.54606	-0.10320	0.18516
H	-3.01383	-0.66841	-1.41508

ACE1_nm_ub971_def2tzvp

25

N	-1.43110	-0.10420	-0.16326
C	-1.01733	0.94418	0.76749
H	-1.88729	1.47089	1.18829
H	-0.50552	0.50355	1.62756
C	-0.11853	2.00515	0.11511
H	-0.67947	2.53373	-0.66105
H	0.17086	2.74098	0.88148
O	1.03004	1.49638	-0.54950
C	1.96025	0.79390	0.25837
H	2.90751	1.35050	0.29503
H	1.60403	0.71505	1.28992
C	2.26714	-0.58764	-0.32528
H	2.75296	-0.46485	-1.29738
H	2.98132	-1.08876	0.34711
O	1.14001	-1.40793	-0.58246
C	0.33586	-1.75069	0.53661
H	0.44019	-2.82467	0.74200
H	0.67499	-1.22780	1.43605
C	-1.14308	-1.46754	0.26291
H	-1.70373	-1.76054	1.17366
H	-1.47871	-2.13389	-0.53936
C	-2.80267	0.05017	-0.61956
H	-2.94967	1.05455	-1.02582
H	-3.54728	-0.10372	0.18408
H	-3.01280	-0.66838	-1.41705

ACE1_nm_ub971_def2tzv

25

N	-1.44885	-0.11193	-0.15343
C	-1.03017	0.94814	0.78068
H	-1.90263	1.46175	1.21453
H	-0.49946	0.51176	1.63103
C	-0.15621	2.03031	0.12267
H	-0.71668	2.53508	-0.66607
H	0.12305	2.77362	0.88207
O	1.04060	1.53428	-0.55268
C	1.98965	0.79812	0.27090
H	2.92746	1.36409	0.31488
H	1.62326	0.70280	1.29612
C	2.30873	-0.56990	-0.33942
H	2.76285	-0.43369	-1.32201
H	3.03729	-1.07121	0.31323
O	1.16601	-1.43743	-0.59081
C	0.32933	-1.77448	0.55552
H	0.43664	-2.84542	0.75898
H	0.66668	-1.24321	1.44937
C	-1.14999	-1.49201	0.26265
H	-1.71904	-1.79347	1.16577
H	-1.47215	-2.15124	-0.54925
C	-2.82633	0.04712	-0.64087
H	-2.96305	1.04994	-1.05213
H	-3.58263	-0.10347	0.15209
H	-3.02252	-0.67255	-1.43951

ACE1_nm_ucam-b3lyp_def2svpp

25

N	-1.42369	-0.11570	-0.12251
C	-1.01866	0.93468	0.79181

H	-1.89571	1.45809	1.22421
H	-0.49483	0.49849	1.65662
C	-0.12893	1.98962	0.12936
H	-0.70657	2.51761	-0.64678
H	0.16804	2.74096	0.89048
O	0.99769	1.47431	-0.53778
C	1.95470	0.80130	0.23986
H	2.90413	1.37193	0.25094
H	1.62972	0.72064	1.29088
C	2.24749	-0.57590	-0.34706
H	2.71378	-0.44570	-1.33691
H	2.98617	-1.08665	0.30531
O	1.11638	-1.37513	-0.57564
C	0.34375	-1.75013	0.53749
H	0.46357	-2.83380	0.73052
H	0.68617	-1.23534	1.45097
C	-1.13404	-1.47466	0.27952
H	-1.70073	-1.79334	1.18756
H	-1.46292	-2.13601	-0.54029
C	-2.74762	0.05943	-0.66661
H	-2.85902	1.07573	-1.08082
H	-3.55823	-0.08761	0.08603
H	-2.92053	-0.65454	-1.49000

ACE1_nm_ucam-b3lyp_def2svp

25

N	-1.42385	-0.11546	-0.12376
C	-1.01554	0.93422	0.78958
H	-1.88810	1.45649	1.22813
H	-0.48795	0.49875	1.65067
C	-0.12920	1.98982	0.12766

H	-0.70733	2.51899	-0.64524
H	0.16305	2.74108	0.88838
O	0.99956	1.47647	-0.53864
C	1.95189	0.79829	0.24132
H	2.90133	1.36550	0.26417
H	1.62196	0.71200	1.28891
C	2.24734	-0.57540	-0.34723
H	2.71728	-0.44372	-1.33360
H	2.98701	-1.08218	0.30448
O	1.11764	-1.37731	-0.57681
C	0.34295	-1.74697	0.53749
H	0.46353	-2.82710	0.74088
H	0.68152	-1.22673	1.44770
C	-1.13301	-1.47423	0.27802
H	-1.69852	-1.79424	1.18426
H	-1.46277	-2.13626	-0.53888
C	-2.74978	0.05832	-0.66330
H	-2.86583	1.07088	-1.07862
H	-3.55659	-0.08742	0.08991
H	-2.92718	-0.65550	-1.48248

ACE1_nm_ucam-b3lyp_def2sv

25

N	-1.42375	-0.11576	-0.12217
C	-1.01868	0.93465	0.79197
H	-1.89567	1.45819	1.22432
H	-0.49496	0.49844	1.65682
C	-0.12885	1.98954	0.12926
H	-0.70669	2.51755	-0.64673
H	0.16842	2.74088	0.89021
O	0.99747	1.47393	-0.53799

C 1.95453 0.80134 0.24000

H 2.90376 1.37229 0.25126

H 1.62924 0.72072 1.29092

C 2.24757 -0.57591 -0.34694

H 2.71426 -0.44572 -1.33660

H 2.98579 -1.08699 0.30561

O 1.11619 -1.37460 -0.57592

C 0.34397 -1.75002 0.53738

H 0.46408 -2.83368 0.73025

H 0.68646 -1.23527 1.45082

C -1.13398 -1.47469 0.27968

H -1.70051 -1.79359 1.18769

H -1.46271 -2.13597 -0.54022

C -2.74747 0.05934 -0.66673

H -2.85895 1.07592 -1.08016

H -3.55839 -0.08859 0.08536

H -2.91972 -0.65398 -1.49079

ACE1_nm_ucam-b3lyp_def2tzvpp

25

N -1.42463 -0.10600 -0.15569

C -1.00945 0.93725 0.76575

H -1.87566 1.45761 1.19106

H -0.49383 0.49946 1.61919

C -0.12398 1.99294 0.11375

H -0.68559 2.51485 -0.66046

H 0.16554 2.73050 0.87180

O 1.02121 1.48395 -0.54491

C 1.95125 0.78819 0.25767

H 2.89146 1.34740 0.29810

H 1.59394 0.70590 1.28387

C	2.25852	-0.58000	-0.32665
H	2.74116	-0.45445	-1.29523
H	2.96933	-1.08481	0.33897
O	1.13205	-1.39270	-0.57986
C	0.33338	-1.74042	0.53363
H	0.44415	-2.80925	0.73857
H	0.67048	-1.21756	1.42840
C	-1.13532	-1.46305	0.26082
H	-1.69659	-1.75787	1.16488
H	-1.46638	-2.12539	-0.54095
C	-2.78446	0.05130	-0.61959
H	-2.92905	1.05286	-1.02306
H	-3.52980	-0.10237	0.17729
H	-2.99250	-0.66218	-1.41671

ACE1_nm_ucam-b3lyp_def2tzvp

25

N	-1.42488	-0.10599	-0.15529
C	-1.00995	0.93759	0.76590
H	-1.87699	1.45897	1.19047
H	-0.49487	0.49979	1.62067
C	-0.12376	1.99283	0.11405
H	-0.68559	2.51549	-0.66067
H	0.16653	2.73036	0.87312
O	1.02084	1.48317	-0.54516
C	1.95189	0.78841	0.25728
H	2.89277	1.34840	0.29601
H	1.59540	0.70665	1.28471
C	2.25884	-0.58008	-0.32655
H	2.74161	-0.45479	-1.29604
H	2.96982	-1.08528	0.34005

O	1.13183	-1.39213	-0.57985
C	0.33338	-1.74054	0.53371
H	0.44395	-2.81050	0.73771
H	0.67085	-1.21816	1.42964
C	-1.13529	-1.46302	0.26104
H	-1.69688	-1.75845	1.16578
H	-1.46620	-2.12574	-0.54157
C	-2.78443	0.05124	-0.62006
H	-2.92897	1.05390	-1.02296
H	-3.53094	-0.10360	0.17669
H	-2.99168	-0.66196	-1.41872

ACE1_nm_ucam-b3lyp_def2tzv

25

N	-1.44319	-0.11499	-0.14028
C	-1.02223	0.94071	0.78074
H	-1.89068	1.44859	1.21889
H	-0.48703	0.50876	1.62495
C	-0.16236	2.01602	0.12014
H	-0.72398	2.51081	-0.66845
H	0.11550	2.76405	0.86897
O	1.02957	1.51889	-0.54533
C	1.97942	0.79235	0.26947
H	2.90946	1.36228	0.31635
H	1.61379	0.69152	1.29004
C	2.29818	-0.56113	-0.34363
H	2.74538	-0.42027	-1.32423
H	3.02612	-1.06675	0.29875
O	1.15629	-1.41823	-0.58663
C	0.32826	-1.76244	0.55229
H	0.44405	-2.82828	0.75489

H	0.66238	-1.23085	1.44204
C	-1.14083	-1.48818	0.26105
H	-1.71090	-1.79600	1.15547
H	-1.45613	-2.14116	-0.55322
C	-2.80366	0.04854	-0.64431
H	-2.93446	1.04926	-1.05269
H	-3.56616	-0.10258	0.13642
H	-2.99262	-0.66501	-1.44504

ACE1_nm_udsdpbepb86_def2svpp

25

N	-1.40818	-0.11441	-0.14408
C	-1.01468	0.92888	0.78953
H	-1.89864	1.44330	1.22559
H	-0.48728	0.48757	1.65240
C	-0.13087	1.98941	0.12809
H	-0.71430	2.51659	-0.64687
H	0.17325	2.74048	0.88983
O	0.99383	1.46932	-0.54861
C	1.94629	0.80239	0.24893
H	2.89397	1.37968	0.27267
H	1.60491	0.72048	1.29640
C	2.24903	-0.57427	-0.33713
H	2.72956	-0.44166	-1.32170
H	2.97558	-1.09200	0.32670
O	1.11347	-1.36964	-0.58698
C	0.34727	-1.74629	0.53640
H	0.47337	-2.83096	0.73232
H	0.69226	-1.22490	1.44731
C	-1.13199	-1.47281	0.27933
H	-1.70109	-1.77270	1.19529

H	-1.46523	-2.14398	-0.53380
C	-2.75221	0.05612	-0.65130
H	-2.87536	1.07773	-1.05370
H	-3.54057	-0.10219	0.12453
H	-2.93863	-0.65462	-1.47683

ACE1_nm_udsdpbepb86_def2svp

25

N	-1.40824	-0.11485	-0.14539
C	-1.01134	0.92750	0.78695
H	-1.88981	1.44002	1.22916
H	-0.47939	0.48626	1.64411
C	-0.13143	1.98927	0.12642
H	-0.71518	2.51771	-0.64399
H	0.16768	2.73765	0.88894
O	0.99496	1.47208	-0.54933
C	1.94181	0.79988	0.25010
H	2.88877	1.37244	0.28707
H	1.59405	0.71118	1.29258
C	2.24813	-0.57327	-0.33700
H	2.73268	-0.43939	-1.31675
H	2.97394	-1.08510	0.32782
O	1.11529	-1.37183	-0.58804
C	0.34722	-1.74261	0.53579
H	0.47361	-2.82239	0.74321
H	0.68753	-1.21431	1.44187
C	-1.13043	-1.47255	0.27765
H	-1.69661	-1.77262	1.19195
H	-1.46469	-2.14346	-0.53196
C	-2.75325	0.05487	-0.64776
H	-2.88019	1.07201	-1.05070

H -3.53673 -0.10115 0.12846

H -2.94430 -0.65546 -1.46788

ACE1_nm_udsdpbepb86_def2sv

25

N -1.40858 -0.11415 -0.14412

C -1.01468 0.92915 0.78933

H -1.89847 1.44421 1.22492

H -0.48798 0.48776 1.65259

C -0.13029 1.98919 0.12768

H -0.71374 2.51626 -0.64735

H 0.17406 2.74044 0.88912

O 0.99416 1.46861 -0.54889

C 1.94663 0.80217 0.24915

H 2.89413 1.37969 0.27294

H 1.60492 0.72055 1.29652

C 2.24937 -0.57464 -0.33672

H 2.73041 -0.44218 -1.32106

H 2.97528 -1.09288 0.32734

O 1.11336 -1.36919 -0.58703

C 0.34717 -1.74594 0.53634

H 0.47332 -2.83060 0.73226

H 0.69207 -1.22456 1.44726

C -1.13209 -1.47254 0.27899

H -1.70145 -1.77284 1.19463

H -1.46481 -2.14364 -0.53443

C -2.75291 0.05608 -0.65062

H -2.87677 1.07791 -1.05215

H -3.54084 -0.10333 0.12540

H -2.93932 -0.65406 -1.47666

ACE1_nm_udsdpbepb86_def2tzvpp

25

N	-1.41037	-0.10286	-0.18277
C	-1.00286	0.93163	0.76048
H	-1.87434	1.44446	1.18940
H	-0.48361	0.48459	1.60960
C	-0.12136	1.99387	0.11040
H	-0.68851	2.51469	-0.66279
H	0.17283	2.72918	0.87140
O	1.02306	1.48152	-0.55618
C	1.94085	0.78666	0.26927
H	2.88133	1.34696	0.32793
H	1.56130	0.70203	1.28918
C	2.25845	-0.58225	-0.31511
H	2.75637	-0.45267	-1.27696
H	2.95596	-1.09183	0.36361
O	1.12945	-1.39171	-0.59138
C	0.33553	-1.73440	0.53295
H	0.44999	-2.80257	0.74759
H	0.67344	-1.19855	1.42203
C	-1.13566	-1.46011	0.25722
H	-1.69945	-1.73332	1.16875
H	-1.47097	-2.13212	-0.53742
C	-2.79227	0.04978	-0.59822
H	-2.95045	1.05475	-0.99288
H	-3.50567	-0.10928	0.22825
H	-3.02178	-0.66584	-1.38975

ACE1_nm_udsdpbepb86_def2tzvp

25

N	-1.41063	-0.10291	-0.18176
C	-1.00442	0.93249	0.76101

H	-1.87819	1.44717	1.18807
H	-0.48791	0.48550	1.61399
C	-0.12111	1.99387	0.11173
H	-0.68822	2.51767	-0.66214
H	0.17503	2.72957	0.87445
O	1.02227	1.48072	-0.55633
C	1.94259	0.78723	0.26797
H	2.88452	1.34949	0.32298
H	1.56573	0.70479	1.29089
C	2.25885	-0.58243	-0.31555
H	2.75728	-0.45445	-1.27953
H	2.95742	-1.09336	0.36423
O	1.12888	-1.39107	-0.59131
C	0.33555	-1.73536	0.53333
H	0.45015	-2.80606	0.74565
H	0.67564	-1.20174	1.42497
C	-1.13571	-1.46001	0.25864
H	-1.70044	-1.73346	1.17190
H	-1.47256	-2.13340	-0.53664
C	-2.79163	0.04951	-0.60052
H	-2.94992	1.05673	-0.99395
H	-3.50862	-0.11208	0.22462
H	-3.01945	-0.66504	-1.39573

ACE1_nm_udsdpbepb86_def2tzv

25

N	-1.43152	-0.11022	-0.17292
C	-1.02130	0.93989	0.77751
H	-1.89924	1.43984	1.21893
H	-0.48154	0.49611	1.61882
C	-0.16077	2.02719	0.12222

H	-0.72343	2.52839	-0.66732
H	0.12823	2.76715	0.88152
O	1.03606	1.52533	-0.55866
C	1.98004	0.79350	0.28023
H	2.91270	1.36644	0.34220
H	1.59188	0.68957	1.29686
C	2.30809	-0.56636	-0.33470
H	2.76861	-0.42745	-1.31385
H	3.02188	-1.07880	0.32567
O	1.15717	-1.42645	-0.60014
C	0.32896	-1.76737	0.55516
H	0.44487	-2.83705	0.76191
H	0.66975	-1.22667	1.44265
C	-1.14704	-1.48811	0.26286
H	-1.71765	-1.76283	1.17386
H	-1.47668	-2.15425	-0.54098
C	-2.82325	0.04627	-0.62840
H	-2.96928	1.05573	-1.02106
H	-3.55513	-0.11972	0.18395
H	-3.02859	-0.66605	-1.43159

ACE1_nm_uhse06_def2svpp

25

N	-1.41870	-0.11842	-0.12079
C	-1.01551	0.92856	0.79342
H	-1.89217	1.44777	1.23651
H	-0.48492	0.48939	1.65524
C	-0.13174	1.98699	0.13183
H	-0.71318	2.51625	-0.64250
H	0.16204	2.73975	0.89512
O	0.99304	1.47860	-0.53871

C	1.94476	0.80219	0.23916
H	2.89607	1.37229	0.25796
H	1.61628	0.71886	1.29095
C	2.24018	-0.57298	-0.34764
H	2.70757	-0.44170	-1.33827
H	2.98388	-1.07994	0.30467
O	1.11503	-1.37615	-0.57784
C	0.34576	-1.74745	0.53619
H	0.46699	-2.83131	0.73515
H	0.68939	-1.22912	1.44979
C	-1.13138	-1.47406	0.28165
H	-1.69663	-1.79674	1.19159
H	-1.46241	-2.13658	-0.53848
C	-2.73600	0.06017	-0.67027
H	-2.84067	1.07629	-1.08981
H	-3.55473	-0.08015	0.07730
H	-2.90760	-0.65625	-1.49332

ACE1_nm_uhse06_def2svp

25

N	-1.41914	-0.11846	-0.12162
C	-1.01206	0.92791	0.79144
H	-1.88319	1.44514	1.24236
H	-0.47619	0.48919	1.64816
C	-0.13257	1.98743	0.12972
H	-0.71466	2.51697	-0.64141
H	0.15564	2.74053	0.89196
O	0.99478	1.48093	-0.53956
C	1.94141	0.79904	0.24105
H	2.89209	1.36572	0.27405
H	1.60636	0.70839	1.28852

C	2.24041	-0.57207	-0.34835
H	2.71096	-0.43860	-1.33526
H	2.98547	-1.07461	0.30254
O	1.11661	-1.37825	-0.57927
C	0.34544	-1.74372	0.53635
H	0.46826	-2.82312	0.74761
H	0.68448	-1.21806	1.44563
C	-1.13009	-1.47410	0.28017
H	-1.69377	-1.79885	1.18775
H	-1.46162	-2.13692	-0.53705
C	-2.73844	0.05869	-0.66699
H	-2.84775	1.07049	-1.08821
H	-3.55360	-0.07988	0.08041
H	-2.91424	-0.65770	-1.48538

ACE1_nm_uhse06_def2sv

25

N	-1.41887	-0.11834	-0.12060
C	-1.01544	0.92862	0.79347
H	-1.89192	1.44816	1.23645
H	-0.48502	0.48937	1.65536
C	-0.13145	1.98688	0.13154
H	-0.71309	2.51613	-0.64265
H	0.16271	2.73967	0.89460
O	0.99294	1.47803	-0.53907
C	1.94465	0.80220	0.23937
H	2.89573	1.37263	0.25839
H	1.61569	0.71899	1.29101
C	2.24037	-0.57310	-0.34730
H	2.70835	-0.44189	-1.33766
H	2.98345	-1.08041	0.30535

O	1.11491	-1.37562	-0.57809
C	0.34591	-1.74728	0.53602
H	0.46730	-2.83115	0.73480
H	0.68956	-1.22903	1.44963
C	-1.13134	-1.47396	0.28159
H	-1.69651	-1.79693	1.19142
H	-1.46209	-2.13643	-0.53870
C	-2.73623	0.06007	-0.66997
H	-2.84145	1.07660	-1.08828
H	-3.55492	-0.08172	0.07732
H	-2.90729	-0.65543	-1.49393

ACE1_nm_uhse06_def2tzvpp

25

N	-1.41919	-0.10823	-0.15720
C	-1.00612	0.93159	0.76530
H	-1.87296	1.44871	1.20009
H	-0.48436	0.49035	1.61723
C	-0.12535	1.99076	0.11447
H	-0.69164	2.51466	-0.65847
H	0.16026	2.73012	0.87606
O	1.01761	1.48819	-0.54687
C	1.93946	0.78877	0.25817
H	2.88292	1.34669	0.31049
H	1.57544	0.70218	1.28490
C	2.25143	-0.57773	-0.32615
H	2.73788	-0.44970	-1.29520
H	2.96745	-1.07784	0.34183
O	1.13210	-1.39474	-0.58257
C	0.33680	-1.73568	0.53233
H	0.44842	-2.80500	0.74781

H	0.67402	-1.20574	1.42676
C	-1.13207	-1.46179	0.26187
H	-1.69203	-1.75911	1.16932
H	-1.46570	-2.12728	-0.53968
C	-2.77613	0.05003	-0.61932
H	-2.91891	1.05202	-1.02805
H	-3.52556	-0.09846	0.17821
H	-2.98660	-0.66733	-1.41528

ACE1_nm_uhse06_def2tzvp

25

N	-1.41959	-0.10804	-0.15680
C	-1.00668	0.93222	0.76541
H	-1.87420	1.45062	1.19920
H	-0.48582	0.49098	1.61875
C	-0.12478	1.99074	0.11483
H	-0.69110	2.51559	-0.65850
H	0.16164	2.72992	0.87746
O	1.01752	1.48734	-0.54713
C	1.94045	0.78880	0.25772
H	2.88462	1.34732	0.30818
H	1.57741	0.70291	1.28562
C	2.25178	-0.57810	-0.32606
H	2.73830	-0.45041	-1.29597
H	2.96794	-1.07862	0.34277
O	1.13171	-1.39427	-0.58254
C	0.33659	-1.73593	0.53246
H	0.44787	-2.80632	0.74691
H	0.67425	-1.20670	1.42800
C	-1.13224	-1.46164	0.26220
H	-1.69246	-1.75957	1.17026

H	-1.46582	-2.12742	-0.54009
C	-2.77633	0.05008	-0.61984
H	-2.91921	1.05314	-1.02774
H	-3.52701	-0.09994	0.17727
H	-2.98580	-0.66675	-1.41749

ACE1_nm_uhse06_def2tzv

25

N	-1.43770	-0.11529	-0.14591
C	-1.01834	0.93643	0.77797
H	-1.88714	1.44327	1.22437
H	-0.47922	0.50004	1.62174
C	-0.16048	2.01447	0.12178
H	-0.72586	2.51558	-0.66409
H	0.11462	2.76089	0.87778
O	1.02909	1.52403	-0.54893
C	1.96962	0.79162	0.26975
H	2.90500	1.35791	0.32716
H	1.59753	0.68893	1.29127
C	2.29117	-0.56162	-0.34050
H	2.74621	-0.42065	-1.32061
H	3.02166	-1.06221	0.30837
O	1.15577	-1.42260	-0.59028
C	0.32923	-1.75896	0.55040
H	0.44241	-2.82638	0.76234
H	0.66476	-1.22251	1.44087
C	-1.13945	-1.48481	0.26203
H	-1.70722	-1.79151	1.16258
H	-1.46013	-2.14328	-0.54949
C	-2.79862	0.04838	-0.64106
H	-2.93053	1.04974	-1.05393

H	-3.56177	-0.09798	0.14426
H	-2.99412	-0.66923	-1.43993

ACE1_nm_um062x_def2svpp

25

N	-1.40805	-0.11397	-0.13784
C	-1.00717	0.92735	0.79177
H	-1.88652	1.43989	1.23279
H	-0.47571	0.48190	1.64854
C	-0.12543	1.98697	0.12797
H	-0.70998	2.51353	-0.64415
H	0.18147	2.73587	0.88670
O	0.99434	1.46417	-0.54413
C	1.94459	0.79699	0.24741
H	2.88999	1.37230	0.27326
H	1.60153	0.71114	1.29329
C	2.24306	-0.57794	-0.34233
H	2.71912	-0.44324	-1.32663
H	2.96766	-1.09983	0.31565
O	1.10444	-1.36233	-0.58349
C	0.34378	-1.74489	0.53476
H	0.47277	-2.82690	0.72751
H	0.68719	-1.22349	1.44553
C	-1.13516	-1.47320	0.27866
H	-1.70535	-1.77436	1.18953
H	-1.46608	-2.13884	-0.53743
C	-2.74458	0.06510	-0.65465
H	-2.85800	1.08363	-1.06229
H	-3.53524	-0.08303	0.11710
H	-2.93132	-0.64796	-1.47510

ACE1_nm_um062x_def2svp

25

N	-1.40838	-0.11342	-0.13919
C	-1.00521	0.92767	0.78951
H	-1.88049	1.43958	1.23368
H	-0.47313	0.48326	1.64388
C	-0.12506	1.98747	0.12695
H	-0.70896	2.51586	-0.64154
H	0.17811	2.73490	0.88555
O	0.99631	1.46615	-0.54482
C	1.94301	0.79458	0.24822
H	2.88788	1.36618	0.28257
H	1.59702	0.70566	1.29085
C	2.24305	-0.57802	-0.34211
H	2.72231	-0.44313	-1.32260
H	2.96755	-1.09609	0.31548
O	1.10551	-1.36469	-0.58430
C	0.34285	-1.74302	0.53476
H	0.47131	-2.82160	0.73496
H	0.68398	-1.21898	1.44256
C	-1.13459	-1.47241	0.27781
H	-1.70329	-1.77350	1.18702
H	-1.46710	-2.13840	-0.53454
C	-2.74675	0.06400	-0.65210
H	-2.86441	1.07862	-1.06019
H	-3.53344	-0.08317	0.11974
H	-2.93703	-0.64849	-1.46824

ACE1_nm_um062x_def2sv

25

N	-1.40827	-0.11418	-0.13736
C	-1.00733	0.92721	0.79199

H	-1.88660	1.43979	1.23310
H	-0.47582	0.48180	1.64874
C	-0.12554	1.98680	0.12792
H	-0.71026	2.51342	-0.64404
H	0.18169	2.73572	0.88645
O	0.99383	1.46362	-0.54442
C	1.94441	0.79731	0.24743
H	2.88943	1.37321	0.27334
H	1.60120	0.71139	1.29324
C	2.24333	-0.57770	-0.34215
H	2.71973	-0.44313	-1.32629
H	2.96756	-1.09975	0.31604
O	1.10451	-1.36164	-0.58364
C	0.34417	-1.74470	0.53462
H	0.47354	-2.82670	0.72721
H	0.68744	-1.22338	1.44548
C	-1.13494	-1.47339	0.27865
H	-1.70496	-1.77522	1.18936
H	-1.46544	-2.13889	-0.53771
C	-2.74447	0.06495	-0.65475
H	-2.85771	1.08346	-1.06244
H	-3.53556	-0.08316	0.11655
H	-2.93090	-0.64804	-1.47532

ACE1_nm_um062x_def2tzvpp

25

N	-1.40891	-0.10629	-0.16739
C	-1.00319	0.93090	0.77034
H	-1.87492	1.44190	1.19764
H	-0.48433	0.48706	1.62034
C	-0.12317	1.99172	0.11591

H -0.69312 2.51151 -0.65522

H 0.17482 2.72814 0.87247

O 1.01495 1.47676 -0.54838

C 1.94343 0.78800 0.26161

H 2.88165 1.35063 0.30632

H 1.57932 0.70329 1.28686

C 2.25278 -0.58116 -0.32619

H 2.74045 -0.45042 -1.29246

H 2.95480 -1.09465 0.34263

O 1.11999 -1.38135 -0.58688

C 0.33632 -1.73908 0.53322

H 0.45593 -2.80803 0.73478

H 0.67587 -1.21361 1.42756

C -1.13616 -1.46452 0.26495

H -1.69944 -1.74030 1.17337

H -1.47126 -2.13203 -0.53217

C -2.77637 0.05594 -0.61777

H -2.91809 1.06186 -1.01359

H -3.51006 -0.10043 0.18894

H -2.99057 -0.65503 -1.41617

ACE1_nm_um062x_def2tzvp

25

N -1.40940 -0.10594 -0.16770

C -1.00380 0.93159 0.76996

H -1.87626 1.44395 1.19579

H -0.48676 0.48767 1.62181

C -0.12239 1.99177 0.11607

H -0.69203 2.51269 -0.65543

H 0.17656 2.72777 0.87360

O 1.01525 1.47612 -0.54873

C	1.94449	0.78807	0.26129
H	2.88338	1.35115	0.30413
H	1.58132	0.70493	1.28766
C	2.25332	-0.58168	-0.32559
H	2.74169	-0.45174	-1.29234
H	2.95483	-1.09570	0.34444
O	1.12001	-1.38122	-0.58685
C	0.33605	-1.73936	0.53316
H	0.45495	-2.80935	0.73351
H	0.67652	-1.21511	1.42860
C	-1.13638	-1.46419	0.26500
H	-1.69990	-1.73990	1.17404
H	-1.47157	-2.13228	-0.53246
C	-2.77742	0.05582	-0.61743
H	-2.91984	1.06293	-1.01166
H	-3.51104	-0.10262	0.18973
H	-2.99139	-0.65409	-1.41770

ACE1_nm_um062x_def2tzv

25

N	-1.42900	-0.11483	-0.15173
C	-1.01318	0.93367	0.78390
H	-1.88461	1.43281	1.22708
H	-0.47193	0.49526	1.62236
C	-0.16007	2.01338	0.11942
H	-0.73085	2.50392	-0.66662
H	0.12415	2.76199	0.86561
O	1.02536	1.51077	-0.54909
C	1.97034	0.79097	0.27485
H	2.89834	1.36357	0.33012
H	1.59417	0.68580	1.29239

C	2.29239	-0.56290	-0.34127
H	2.74807	-0.41662	-1.31783
H	3.00958	-1.07735	0.30622
O	1.14440	-1.40589	-0.59413
C	0.33086	-1.75907	0.55003
H	0.45582	-2.82417	0.75229
H	0.66495	-1.22242	1.43836
C	-1.14150	-1.48965	0.26219
H	-1.71348	-1.78148	1.15980
H	-1.45856	-2.14639	-0.54932
C	-2.79876	0.05450	-0.63768
H	-2.92788	1.05977	-1.03671
H	-3.54661	-0.10008	0.15545
H	-2.99667	-0.65528	-1.43995

ACE1_nm_um06_def2svpp

25

N	-1.41555	-0.12139	-0.12708
C	-1.01622	0.92766	0.78851
H	-1.89921	1.44090	1.23240
H	-0.48488	0.49243	1.65607
C	-0.14367	1.98335	0.12144
H	-0.73240	2.50240	-0.65907
H	0.14745	2.74930	0.87638
O	0.98312	1.47241	-0.54164
C	1.93606	0.80945	0.24297
H	2.88358	1.38995	0.27205
H	1.60166	0.72615	1.29669
C	2.24629	-0.55932	-0.33840
H	2.72545	-0.42549	-1.32640
H	2.98919	-1.06307	0.32153

O	1.12810	-1.36806	-0.57618
C	0.35411	-1.74463	0.53137
H	0.47902	-2.83019	0.73223
H	0.69527	-1.22898	1.45160
C	-1.11780	-1.47748	0.26938
H	-1.69303	-1.80687	1.17404
H	-1.44099	-2.14066	-0.55822
C	-2.74375	0.04689	-0.65099
H	-2.86897	1.06692	-1.06212
H	-3.54536	-0.10660	0.11560
H	-2.92788	-0.66692	-1.47633

ACE1_nm_um06_def2svp

25

N	-1.41521	-0.12214	-0.12801
C	-1.01306	0.92651	0.78664
H	-1.89047	1.43702	1.23725
H	-0.47710	0.49368	1.64939
C	-0.14557	1.98362	0.11929
H	-0.73589	2.50192	-0.65671
H	0.13816	2.75004	0.87224
O	0.98410	1.47529	-0.54266
C	1.93201	0.80721	0.24522
H	2.87805	1.38363	0.28952
H	1.59178	0.71599	1.29389
C	2.24648	-0.55735	-0.33838
H	2.73046	-0.42109	-1.32065
H	2.98991	-1.05559	0.32027
O	1.13006	-1.36933	-0.57832
C	0.35450	-1.74199	0.53058
H	0.48042	-2.82250	0.74235

H	0.69087	-1.22184	1.44718
C	-1.11581	-1.47817	0.26798
H	-1.68850	-1.80929	1.16987
H	-1.44012	-2.14173	-0.55519
C	-2.74554	0.04517	-0.64748
H	-2.87525	1.06087	-1.05784
H	-3.54283	-0.10751	0.11813
H	-2.93434	-0.66626	-1.46881

ACE1_nm_um06_def2sv

25

N	-1.41589	-0.12141	-0.12690
C	-1.01633	0.92763	0.78850
H	-1.89921	1.44125	1.23208
H	-0.48524	0.49237	1.65619
C	-0.14343	1.98311	0.12120
H	-0.73225	2.50224	-0.65917
H	0.14802	2.74900	0.87604
O	0.98298	1.47175	-0.54201
C	1.93613	0.80958	0.24303
H	2.88332	1.39053	0.27214
H	1.60143	0.72636	1.29663
C	2.24667	-0.55945	-0.33800
H	2.72650	-0.42594	-1.32570
H	2.98880	-1.06347	0.32247
O	1.12811	-1.36745	-0.57631
C	0.35434	-1.74416	0.53130
H	0.47958	-2.82963	0.73236
H	0.69523	-1.22820	1.45141
C	-1.11772	-1.47750	0.26911
H	-1.69288	-1.80746	1.17352

H	-1.44028	-2.14055	-0.55881
C	-2.74416	0.04677	-0.65058
H	-2.86949	1.06676	-1.06173
H	-3.54567	-0.10675	0.11605
H	-2.92836	-0.66703	-1.47587

ACE1_nm_um06_def2tzvpp

25

N	-1.40861	-0.11493	-0.15092
C	-1.00649	0.92626	0.77513
H	-1.87927	1.43203	1.21097
H	-0.48005	0.49002	1.62712
C	-0.14107	1.98455	0.11389
H	-0.72184	2.49548	-0.65856
H	0.14404	2.73660	0.86340
O	0.99563	1.47979	-0.54766
C	1.92806	0.80072	0.25502
H	2.86601	1.36837	0.30398
H	1.57283	0.71495	1.28597
C	2.24807	-0.56194	-0.32470
H	2.73909	-0.43059	-1.29158
H	2.96629	-1.05844	0.34388
O	1.13408	-1.37855	-0.58204
C	0.34765	-1.73849	0.52800
H	0.46713	-2.80886	0.73502
H	0.68303	-1.21892	1.43052
C	-1.11899	-1.46981	0.26099
H	-1.68402	-1.77119	1.16363
H	-1.44994	-2.13132	-0.54574
C	-2.76035	0.04336	-0.62359
H	-2.90374	1.04994	-1.02151

H	-3.51391	-0.11636	0.16735
H	-2.96429	-0.66510	-1.42872

ACE1_nm_um06_def2tzvp

25

N	-1.40885	-0.11508	-0.15088
C	-1.00669	0.92612	0.77470
H	-1.88075	1.43272	1.21030
H	-0.48066	0.48934	1.62811
C	-0.14114	1.98374	0.11381
H	-0.72286	2.49577	-0.65891
H	0.14417	2.73615	0.86487
O	0.99489	1.47914	-0.54814
C	1.92805	0.80142	0.25442
H	2.86724	1.36976	0.30208
H	1.57350	0.71656	1.28698
C	2.24812	-0.56154	-0.32354
H	2.74150	-0.43131	-1.29073
H	2.96602	-1.05774	0.34787
O	1.13455	-1.37788	-0.58194
C	0.34775	-1.73856	0.52726
H	0.46644	-2.81048	0.73381
H	0.68304	-1.21968	1.43165
C	-1.11829	-1.46950	0.26019
H	-1.68420	-1.77185	1.16372
H	-1.44977	-2.13153	-0.54751
C	-2.76022	0.04318	-0.62284
H	-2.90407	1.05140	-1.01930
H	-3.51399	-0.11806	0.16945
H	-2.96471	-0.66482	-1.42968

ACE1_nm_um06_def2tzv

25

N	-1.42994	-0.12665	-0.12720
C	-1.02012	0.92805	0.79352
H	-1.89334	1.42635	1.24069
H	-0.47110	0.49973	1.63533
C	-0.17865	2.00613	0.11979
H	-0.76014	2.48818	-0.66735
H	0.09492	2.76703	0.86183
O	1.00293	1.51358	-0.54903
C	1.95425	0.80688	0.26739
H	2.88293	1.38411	0.32215
H	1.58807	0.70006	1.29152
C	2.28599	-0.54380	-0.34152
H	2.74406	-0.39700	-1.31965
H	3.01687	-1.04203	0.30869
O	1.15567	-1.39947	-0.59205
C	0.34594	-1.76028	0.54472
H	0.47125	-2.82833	0.74676
H	0.67830	-1.23023	1.44134
C	-1.12468	-1.49692	0.26347
H	-1.69255	-1.81814	1.15797
H	-1.43805	-2.14662	-0.55883
C	-2.77553	0.04285	-0.65307
H	-2.89396	1.04827	-1.06209
H	-3.55519	-0.10542	0.11512
H	-2.95450	-0.66976	-1.46026

ACE1_nm_ump2_def2svpp

25

N	-1.40420	-0.11075	-0.15452
C	-1.01324	0.92911	0.78531

H	-1.89809	1.44400	1.21890
H	-0.48970	0.48710	1.64970
C	-0.12703	1.98772	0.12635
H	-0.70978	2.51514	-0.64838
H	0.18013	2.73752	0.88779
O	0.99661	1.46258	-0.55299
C	1.94753	0.79920	0.25317
H	2.89436	1.37753	0.28054
H	1.60128	0.71823	1.29836
C	2.25077	-0.57671	-0.33204
H	2.73522	-0.44311	-1.31404
H	2.97135	-1.09912	0.33408
O	1.11016	-1.36673	-0.59004
C	0.34510	-1.74233	0.53695
H	0.47132	-2.82665	0.73483
H	0.68947	-1.21975	1.44652
C	-1.13258	-1.46834	0.27713
H	-1.70442	-1.76101	1.19354
H	-1.46398	-2.14290	-0.53352
C	-2.75845	0.05561	-0.64109
H	-2.89043	1.07849	-1.03499
H	-3.53268	-0.11152	0.14551
H	-2.95146	-0.65105	-1.46757

ACE1_nm_ump2_def2svp

25

N	-1.40309	-0.11208	-0.15628
C	-1.00887	0.92598	0.78214
H	-1.88701	1.43710	1.22383
H	-0.47857	0.48334	1.63792
C	-0.12820	1.98713	0.12485

H	-0.71166	2.51591	-0.64358
H	0.17291	2.73193	0.88816
O	0.99660	1.46679	-0.55368
C	1.93970	0.79712	0.25418
H	2.88538	1.36890	0.29831
H	1.58484	0.70724	1.29245
C	2.24802	-0.57485	-0.33144
H	2.73747	-0.43982	-1.30696
H	2.96658	-1.08873	0.33729
O	1.11240	-1.36926	-0.59147
C	0.34600	-1.73798	0.53524
H	0.47236	-2.81575	0.74700
H	0.68461	-1.20652	1.43819
C	-1.13006	-1.46811	0.27551
H	-1.69702	-1.75916	1.19062
H	-1.46346	-2.14187	-0.53033
C	-2.75712	0.05423	-0.63706
H	-2.89223	1.07139	-1.03232
H	-3.52522	-0.10830	0.15016
H	-2.95620	-0.65242	-1.45608

ACE1_nm_ump2_def2sv

25

N	-1.40428	-0.11071	-0.15449
C	-1.01333	0.92917	0.78526
H	-1.89816	1.44440	1.21853
H	-0.49021	0.48718	1.64995
C	-0.12690	1.98763	0.12626
H	-0.70980	2.51506	-0.64837
H	0.18035	2.73749	0.88762
O	0.99663	1.46242	-0.55310

C	1.94749	0.79915	0.25324
H	2.89430	1.37753	0.28073
H	1.60108	0.71821	1.29839
C	2.25085	-0.57672	-0.33196
H	2.73548	-0.44310	-1.31388
H	2.97130	-1.09924	0.33421
O	1.11016	-1.36658	-0.59009
C	0.34517	-1.74217	0.53696
H	0.47146	-2.82647	0.73493
H	0.68954	-1.21952	1.44650
C	-1.13251	-1.46829	0.27711
H	-1.70440	-1.76108	1.19345
H	-1.46369	-2.14288	-0.53360
C	-2.75856	0.05547	-0.64100
H	-2.89069	1.07829	-1.03500
H	-3.53273	-0.11174	0.14565
H	-2.95153	-0.65125	-1.46746

ACE1_nm_ump2_def2tzvpp

25

N	-1.40360	-0.09981	-0.19523
C	-0.99780	0.92918	0.75510
H	-1.86855	1.43878	1.18434
H	-0.47919	0.47886	1.60050
C	-0.11814	1.99178	0.10827
H	-0.68493	2.51138	-0.66267
H	0.17758	2.72321	0.86901
O	1.02564	1.47684	-0.56100
C	1.93575	0.78190	0.27529
H	2.87409	1.33989	0.34409
H	1.54494	0.69630	1.28857

C	2.25681	-0.58392	-0.30862
H	2.75999	-0.45201	-1.26460
H	2.94561	-1.09567	0.37343
O	1.12553	-1.38953	-0.59597
C	0.33339	-1.72825	0.53239
H	0.44858	-2.79268	0.75295
H	0.67018	-1.18641	1.41570
C	-1.13551	-1.45567	0.25411
H	-1.69996	-1.71835	1.16560
H	-1.47029	-2.13004	-0.53582
C	-2.79446	0.05000	-0.58577
H	-2.95993	1.05305	-0.97570
H	-3.48804	-0.11094	0.25347
H	-3.03450	-0.66537	-1.37107

ACE1_nm_ump2_def2tzvp

25

N	-1.40396	-0.10000	-0.19305
C	-1.00042	0.93057	0.75641
H	-1.87488	1.44294	1.18270
H	-0.48668	0.48059	1.60831
C	-0.11798	1.99193	0.11063
H	-0.68452	2.51647	-0.66137
H	0.18092	2.72440	0.87358
O	1.02436	1.47603	-0.56098
C	1.93878	0.78301	0.27300
H	2.87937	1.34430	0.33577
H	1.55284	0.70133	1.29129
C	2.25738	-0.58415	-0.30979
H	2.76112	-0.45523	-1.26933
H	2.94822	-1.09834	0.37329

O	1.12453	-1.38868	-0.59576
C	0.33341	-1.73037	0.53307
H	0.44908	-2.79894	0.74932
H	0.67427	-1.19300	1.42069
C	-1.13574	-1.45575	0.25671
H	-1.70184	-1.71882	1.17078
H	-1.47342	-2.13216	-0.53412
C	-2.79307	0.04976	-0.59028
H	-2.95829	1.05613	-0.97888
H	-3.49337	-0.11448	0.24617
H	-3.03040	-0.66397	-1.38137

ACE1_nm_ump2_def2tzv

25

N	-1.43053	-0.10712	-0.18453
C	-1.02448	0.94370	0.77879
H	-1.90629	1.44046	1.21981
H	-0.48305	0.49522	1.61779
C	-0.16174	2.03734	0.12713
H	-0.71935	2.54196	-0.66397
H	0.13602	2.76917	0.89125
O	1.04234	1.53131	-0.56438
C	1.98961	0.79294	0.28427
H	2.92155	1.36748	0.35026
H	1.59240	0.68645	1.29770
C	2.31931	-0.56929	-0.33466
H	2.77758	-0.43382	-1.31540
H	3.02553	-1.08708	0.33006
O	1.15759	-1.43387	-0.60651
C	0.32628	-1.77541	0.55991
H	0.44079	-2.84627	0.76409

H	0.67251	-1.23365	1.44549
C	-1.15327	-1.49009	0.26787
H	-1.72174	-1.74617	1.18662
H	-1.49106	-2.15926	-0.53067
C	-2.83689	0.04585	-0.62928
H	-2.98513	1.05847	-1.01333
H	-3.55824	-0.12908	0.19042
H	-3.04016	-0.66368	-1.43548

ACE1_nm_upbe0_def2svpp

25

N	-1.41881	-0.11840	-0.12017
C	-1.01626	0.92919	0.79344
H	-1.89331	1.44947	1.23433
H	-0.48763	0.49077	1.65670
C	-0.13120	1.98678	0.13209
H	-0.71230	2.51698	-0.64189
H	0.16320	2.73904	0.89568
O	0.99252	1.47816	-0.53870
C	1.94465	0.80271	0.23847
H	2.89615	1.37268	0.25492
H	1.61806	0.72096	1.29088
C	2.23964	-0.57312	-0.34721
H	2.70762	-0.44251	-1.33769
H	2.98302	-1.07981	0.30570
O	1.11505	-1.37592	-0.57766
C	0.34619	-1.74781	0.53577
H	0.46664	-2.83218	0.73284
H	0.69064	-1.23152	1.45008
C	-1.13112	-1.47375	0.28251
H	-1.69555	-1.79627	1.19288

H	-1.46279	-2.13629	-0.53731
C	-2.73554	0.05962	-0.67097
H	-2.83979	1.07556	-1.09109
H	-3.55511	-0.08068	0.07559
H	-2.90582	-0.65704	-1.49414

ACE1_nm_upbe0_def2svp

25

N	-1.41936	-0.11838	-0.12089
C	-1.01292	0.92867	0.79149
H	-1.88446	1.44718	1.23988
H	-0.47943	0.49081	1.64990
C	-0.13191	1.98724	0.13007
H	-0.71350	2.51790	-0.64067
H	0.15699	2.73975	0.89263
O	0.99435	1.48049	-0.53951
C	1.94155	0.79958	0.24025
H	2.89248	1.36604	0.27046
H	1.60881	0.71084	1.28847
C	2.23987	-0.57231	-0.34792
H	2.71099	-0.43971	-1.33468
H	2.98459	-1.07460	0.30354
O	1.11657	-1.37807	-0.57903
C	0.34576	-1.74429	0.53593
H	0.46761	-2.82428	0.74490
H	0.68581	-1.22119	1.44611
C	-1.12993	-1.47375	0.28110
H	-1.69275	-1.79835	1.18909
H	-1.46221	-2.13654	-0.53576
C	-2.73800	0.05822	-0.66784
H	-2.84681	1.06982	-1.08962

H	-3.55415	-0.08034	0.07833
H	-2.91236	-0.65830	-1.48642

ACE1_nm_upbe0_def2sv

25

N	-1.41898	-0.11830	-0.11998
C	-1.01618	0.92925	0.79348
H	-1.89304	1.44987	1.23426
H	-0.48773	0.49076	1.65682
C	-0.13090	1.98667	0.13179
H	-0.71218	2.51687	-0.64208
H	0.16389	2.73897	0.89513
O	0.99245	1.47758	-0.53906
C	1.94456	0.80271	0.23870
H	2.89583	1.37301	0.25540
H	1.61745	0.72106	1.29095
C	2.23982	-0.57325	-0.34686
H	2.70841	-0.44273	-1.33706
H	2.98258	-1.08033	0.30640
O	1.11491	-1.37539	-0.57792
C	0.34632	-1.74766	0.53558
H	0.46694	-2.83203	0.73246
H	0.69079	-1.23144	1.44991
C	-1.13110	-1.47365	0.28243
H	-1.69546	-1.79645	1.19270
H	-1.46250	-2.13612	-0.53755
C	-2.73579	0.05953	-0.67064
H	-2.84057	1.07587	-1.08957
H	-3.55529	-0.08218	0.07567
H	-2.90556	-0.65622	-1.49469

ACE1_nm_upbe0_def2tzvpp

25

N	-1.41922	-0.10719	-0.15872
C	-1.00610	0.93256	0.76395
H	-1.87314	1.45154	1.19646
H	-0.48683	0.49101	1.61745
C	-0.12266	1.99046	0.11400
H	-0.68835	2.51631	-0.65847
H	0.16385	2.72881	0.87669
O	1.01876	1.48706	-0.54749
C	1.93939	0.78826	0.25816
H	2.88380	1.34528	0.30972
H	1.57547	0.70332	1.28526
C	2.25064	-0.57943	-0.32465
H	2.73937	-0.45226	-1.29302
H	2.96522	-1.07980	0.34515
O	1.13152	-1.39480	-0.58276
C	0.33659	-1.73519	0.53160
H	0.44694	-2.80508	0.74664
H	0.67445	-1.20617	1.42653
C	-1.13251	-1.46036	0.26197
H	-1.69188	-1.75631	1.17040
H	-1.46725	-2.12663	-0.53880
C	-2.77755	0.04992	-0.61737
H	-2.92173	1.05163	-1.02698
H	-3.52501	-0.09821	0.18229
H	-2.98942	-0.66861	-1.41227

ACE1_nm_upbe0_def2tzvp

25

N	-1.41957	-0.10703	-0.15826
C	-1.00667	0.93317	0.76411

H	-1.87443	1.45338	1.19565
H	-0.48830	0.49168	1.61903
C	-0.12219	1.99044	0.11438
H	-0.68793	2.51717	-0.65851
H	0.16509	2.72868	0.87805
O	1.01860	1.48623	-0.54771
C	1.94035	0.78835	0.25770
H	2.88548	1.34598	0.30737
H	1.57752	0.70410	1.28599
C	2.25095	-0.57973	-0.32460
H	2.73971	-0.45291	-1.29387
H	2.96578	-1.08049	0.34598
O	1.13114	-1.39432	-0.58271
C	0.33641	-1.73549	0.53173
H	0.44644	-2.80647	0.74569
H	0.67473	-1.20727	1.42782
C	-1.13264	-1.46024	0.26232
H	-1.69232	-1.75685	1.17133
H	-1.46737	-2.12678	-0.53921
C	-2.77762	0.04995	-0.61797
H	-2.92188	1.05271	-1.02682
H	-3.52644	-0.09964	0.18119
H	-2.98848	-0.66807	-1.41455

ACE1_nm_upbe0_def2tzv

25

N	-1.43787	-0.11427	-0.14701
C	-1.01841	0.93750	0.77672
H	-1.88742	1.44643	1.22080
H	-0.48175	0.50092	1.62225
C	-0.15774	2.01418	0.12132

H	-0.72263	2.51712	-0.66423
H	0.11783	2.75984	0.87849
O	1.03025	1.52320	-0.54909
C	1.96965	0.79128	0.26935
H	2.90631	1.35632	0.32534
H	1.59848	0.69031	1.29165
C	2.29009	-0.56339	-0.33934
H	2.74702	-0.42326	-1.31911
H	3.01982	-1.06381	0.31116
O	1.15527	-1.42288	-0.59001
C	0.32905	-1.75876	0.54961
H	0.44060	-2.82689	0.76086
H	0.66511	-1.22365	1.44097
C	-1.13989	-1.48344	0.26216
H	-1.70709	-1.78904	1.16369
H	-1.46171	-2.14260	-0.54878
C	-2.79985	0.04835	-0.63947
H	-2.93301	1.04949	-1.05328
H	-3.56160	-0.09770	0.14757
H	-2.99648	-0.67033	-1.43755

ACE1_nm_upbe_def2svpp

25

N	-1.43013	-0.11745	-0.12248
C	-1.02537	0.93756	0.79837
H	-1.90854	1.46438	1.24397
H	-0.49338	0.49439	1.66848
C	-0.13132	2.00338	0.13531
H	-0.71353	2.53737	-0.64823
H	0.15869	2.76176	0.90926
O	1.00637	1.49721	-0.54467

C	1.96212	0.80648	0.24229
H	2.92612	1.37503	0.26128
H	1.63175	0.72335	1.30296
C	2.25515	-0.57921	-0.35022
H	2.71937	-0.44719	-1.35247
H	3.01291	-1.08505	0.30465
O	1.12373	-1.39681	-0.58372
C	0.34450	-1.76279	0.54371
H	0.45941	-2.85650	0.74773
H	0.69222	-1.23996	1.46412
C	-1.14165	-1.48267	0.28420
H	-1.71227	-1.81210	1.20172
H	-1.47635	-2.14873	-0.54355
C	-2.75561	0.06178	-0.67727
H	-2.85887	1.08372	-1.10594
H	-3.58557	-0.07361	0.07488
H	-2.92887	-0.66507	-1.50269

ACE1_nm_upbe_def2svp

25

N	-1.43035	-0.11765	-0.12345
C	-1.02139	0.93649	0.79643
H	-1.89830	1.46034	1.25154
H	-0.48284	0.49397	1.66036
C	-0.13274	2.00388	0.13266
H	-0.71589	2.53711	-0.64762
H	0.15085	2.76342	0.90438
O	1.00795	1.49979	-0.54558
C	1.95792	0.80321	0.24471
H	2.92062	1.36848	0.28017
H	1.61984	0.71149	1.30020

C	2.25547	-0.57782	-0.35114
H	2.72243	-0.44277	-1.34933
H	3.01511	-1.07880	0.30121
O	1.12574	-1.39907	-0.58516
C	0.34444	-1.75842	0.54406
H	0.46175	-2.84678	0.76216
H	0.68659	-1.22704	1.45935
C	-1.14001	-1.48293	0.28215
H	-1.70948	-1.81520	1.19616
H	-1.47433	-2.14903	-0.54276
C	-2.75815	0.05996	-0.67338
H	-2.86682	1.07695	-1.10394
H	-3.58372	-0.07365	0.07866
H	-2.93612	-0.66687	-1.49343

ACE1_nm_upbe_def2sv

25

N	-1.43030	-0.11740	-0.12210
C	-1.02543	0.93759	0.79855
H	-1.90845	1.46489	1.24382
H	-0.49388	0.49435	1.66887
C	-0.13107	2.00319	0.13514
H	-0.71369	2.53702	-0.64822
H	0.15909	2.76175	0.90880
O	1.00629	1.49682	-0.54484
C	1.96207	0.80649	0.24241
H	2.92597	1.37512	0.26136
H	1.63158	0.72349	1.30303
C	2.25517	-0.57934	-0.35015
H	2.71966	-0.44703	-1.35224
H	3.01271	-1.08546	0.30466

O	1.12357	-1.39643	-0.58385
C	0.34466	-1.76257	0.54371
H	0.45960	-2.85626	0.74766
H	0.69246	-1.23978	1.46408
C	-1.14163	-1.48254	0.28425
H	-1.71229	-1.81236	1.20154
H	-1.47578	-2.14857	-0.54374
C	-2.75558	0.06168	-0.67736
H	-2.85920	1.08402	-1.10486
H	-3.58581	-0.07521	0.07413
H	-2.92793	-0.66421	-1.50379

ACE1_nm_upbe_def2tzvpp

25

N	-1.43018	-0.10721	-0.16225
C	-1.01524	0.94013	0.76952
H	-1.88850	1.46095	1.20864
H	-0.49027	0.49352	1.62702
C	-0.12761	2.00851	0.11799
H	-0.69353	2.53694	-0.66317
H	0.15725	2.75149	0.88859
O	1.03044	1.50776	-0.55491
C	1.95613	0.79323	0.26270
H	2.90898	1.35206	0.31810
H	1.58530	0.70711	1.29530
C	2.26969	-0.58175	-0.32671
H	2.75429	-0.45370	-1.30561
H	2.99604	-1.08143	0.34493
O	1.14394	-1.41698	-0.58941
C	0.33535	-1.75070	0.54020
H	0.44234	-2.82805	0.76055

H 0.67732 -1.21436 1.43857
C -1.14167 -1.47113 0.26344
H -1.70695 -1.77187 1.17783
H -1.47875 -2.14040 -0.54424
C -2.79960 0.04978 -0.62346
H -2.94341 1.05664 -1.03983
H -3.55415 -0.09582 0.18213
H -3.01202 -0.67729 -1.42057

ACE1_nm_upbe_def2tzvp

25

N -1.43062 -0.10724 -0.16167
C -1.01636 0.94107 0.76951
H -1.89049 1.46360 1.20672
H -0.49266 0.49506 1.62890
C -0.12716 2.00852 0.11810
H -0.69310 2.53791 -0.66348
H 0.15854 2.75123 0.88985
O 1.03005 1.50655 -0.55530
C 1.95688 0.79329 0.26246
H 2.91010 1.35319 0.31647
H 1.58653 0.70717 1.29598
C 2.27040 -0.58207 -0.32659
H 2.75554 -0.45445 -1.30612
H 2.99627 -1.08237 0.34644
O 1.14358 -1.41574 -0.58967
C 0.33574 -1.75053 0.54033
H 0.44307 -2.82884 0.76000
H 0.67795 -1.21436 1.43956
C -1.14141 -1.47111 0.26411
H -1.70654 -1.77258 1.17935

H	-1.47844	-2.14077	-0.54421
C	-2.79985	0.04896	-0.62401
H	-2.94413	1.05668	-1.03994
H	-3.55583	-0.09818	0.18102
H	-3.01094	-0.67795	-1.42258

ACE1_nm_upbe_def2tzv

25

N	-1.44881	-0.11455	-0.15381
C	-1.02901	0.94548	0.78137
H	-1.90546	1.45563	1.23171
H	-0.48673	0.50339	1.63095
C	-0.16456	2.03393	0.12625
H	-0.72961	2.54199	-0.66699
H	0.11200	2.78178	0.89343
O	1.04149	1.54562	-0.56166
C	1.98640	0.79782	0.27562
H	2.93059	1.36699	0.33690
H	1.60434	0.69716	1.30227
C	2.31301	-0.56421	-0.33671
H	2.77135	-0.42554	-1.32503
H	3.04989	-1.06416	0.32129
O	1.17098	-1.44681	-0.60032
C	0.32861	-1.77540	0.55826
H	0.43644	-2.85169	0.77380
H	0.67028	-1.23299	1.45276
C	-1.14830	-1.49485	0.26338
H	-1.72076	-1.80355	1.17291
H	-1.47377	-2.15865	-0.55312
C	-2.82584	0.04557	-0.64274
H	-2.96178	1.05200	-1.06283

H	-3.59135	-0.09874	0.15414
H	-3.02548	-0.68228	-1.44229

ACE1_nm_uscs-mp2_def2svpp

25

N	-1.41009	-0.10850	-0.15586
C	-1.01581	0.93490	0.78424
H	-1.90064	1.45521	1.21199
H	-0.49795	0.49350	1.65293
C	-0.12292	1.99144	0.12505
H	-0.70242	2.52115	-0.65148
H	0.18573	2.74080	0.88699
O	1.00294	1.46433	-0.55310
C	1.95542	0.79877	0.25312
H	2.90336	1.37650	0.27454
H	1.61246	0.72210	1.30007
C	2.25585	-0.58119	-0.33069
H	2.74243	-0.45072	-1.31287
H	2.97385	-1.10567	0.33731
O	1.11276	-1.37066	-0.58944
C	0.34317	-1.74619	0.53727
H	0.46686	-2.83212	0.73089
H	0.68892	-1.22765	1.44896
C	-1.13654	-1.46901	0.27677
H	-1.70838	-1.76062	1.19372
H	-1.46883	-2.14427	-0.53378
C	-2.76930	0.05582	-0.63896
H	-2.90425	1.07877	-1.03427
H	-3.54116	-0.11167	0.15045
H	-2.96419	-0.65236	-1.46488

ACE1_nm_uscs-mp2_def2svp

25

N	-1.40915	-0.10976	-0.15658
C	-1.01244	0.93232	0.78185
H	-1.89044	1.44972	1.21461
H	-0.48972	0.49131	1.64244
C	-0.12391	1.99056	0.12418
H	-0.70319	2.52174	-0.64531
H	0.17963	2.73363	0.88753
O	1.00237	1.46743	-0.55343
C	1.94881	0.79709	0.25343
H	2.89485	1.36813	0.28764
H	1.59993	0.71311	1.29351
C	2.25307	-0.57952	-0.33042
H	2.74360	-0.44827	-1.30566
H	2.96804	-1.09560	0.33963
O	1.11430	-1.37240	-0.59037
C	0.34393	-1.74238	0.53585
H	0.46705	-2.82151	0.74048
H	0.68441	-1.21724	1.44089
C	-1.13425	-1.46872	0.27590
H	-1.70022	-1.75844	1.19112
H	-1.46816	-2.14245	-0.52930
C	-2.76719	0.05466	-0.63662
H	-2.90401	1.07143	-1.03206
H	-3.53365	-0.10897	0.15112
H	-2.96566	-0.65244	-1.45525

ACE1_nm_uscs-mp2_def2sv

25

N	-1.41017	-0.10845	-0.15589
C	-1.01590	0.93496	0.78418

H	-1.90070	1.45557	1.21162
H	-0.49840	0.49355	1.65312
C	-0.12278	1.99134	0.12497
H	-0.70241	2.52105	-0.65149
H	0.18595	2.74075	0.88682
O	1.00297	1.46414	-0.55320
C	1.95542	0.79871	0.25318
H	2.90335	1.37649	0.27469
H	1.61232	0.72207	1.30010
C	2.25593	-0.58122	-0.33061
H	2.74268	-0.45073	-1.31271
H	2.97380	-1.10581	0.33744
O	1.11277	-1.37052	-0.58947
C	0.34322	-1.74601	0.53730
H	0.46697	-2.83192	0.73104
H	0.68894	-1.22735	1.44894
C	-1.13648	-1.46895	0.27673
H	-1.70838	-1.76064	1.19360
H	-1.46854	-2.14423	-0.53390
C	-2.76945	0.05569	-0.63883
H	-2.90457	1.07859	-1.03423
H	-3.54118	-0.11186	0.15070
H	-2.96435	-0.65257	-1.46469

ACE1_nm_uscs-mp2_def2tzvpp

25

N	-1.40957	-0.09710	-0.19640
C	-1.00190	0.93586	0.75395
H	-1.87127	1.45132	1.17364
H	-0.49219	0.48747	1.60345
C	-0.11245	1.99469	0.10820

H -0.67380 2.51811 -0.66203

H 0.18581 2.72242 0.86924

O 1.03119 1.47653 -0.56046

C 1.94530 0.78199 0.27374

H 2.88318 1.33876 0.33204

H 1.56202 0.70198 1.28835

C 2.26114 -0.58930 -0.30723

H 2.76496 -0.46224 -1.26192

H 2.94569 -1.10242 0.37548

O 1.12691 -1.39224 -0.59413

C 0.33104 -1.73273 0.53261

H 0.44226 -2.79760 0.74522

H 0.66879 -1.19892 1.41807

C -1.13969 -1.45519 0.25482

H -1.70229 -1.71557 1.16572

H -1.47515 -2.12878 -0.53333

C -2.80446 0.05043 -0.58487

H -2.97089 1.05223 -0.97418

H -3.49554 -0.11170 0.25394

H -3.04316 -0.66418 -1.36953

ACE1_nm_uscs-mp2_def2tzvp

25

N -1.40982 -0.09783 -0.19379

C -1.00440 0.93663 0.75587

H -1.87860 1.45512 1.17405

H -0.49745 0.48829 1.61227

C -0.11325 1.99493 0.11049

H -0.67605 2.52288 -0.66199

H 0.18804 2.72540 0.87438

O 1.02920 1.47610 -0.56052

C	1.94785	0.78369	0.27135
H	2.88934	1.34446	0.32399
H	1.56890	0.70713	1.29228
C	2.26192	-0.58889	-0.30869
H	2.76684	-0.46400	-1.26828
H	2.94995	-1.10460	0.37594
O	1.12636	-1.39123	-0.59407
C	0.33166	-1.73489	0.53325
H	0.44368	-2.80528	0.74239
H	0.67344	-1.20413	1.42397
C	-1.13960	-1.45600	0.25719
H	-1.70452	-1.71822	1.17172
H	-1.47779	-2.13252	-0.53330
C	-2.80266	0.05016	-0.58969
H	-2.96907	1.05656	-0.97850
H	-3.50231	-0.11515	0.24684
H	-3.03923	-0.66381	-1.38115

ACE1_nm_uscs-mp2_def2tzv

25

N	-1.43571	-0.10526	-0.18610
C	-1.02773	0.94824	0.77761
H	-1.90947	1.45125	1.21156
H	-0.49351	0.50053	1.62206
C	-0.15568	2.03884	0.12503
H	-0.71180	2.54727	-0.66572
H	0.14407	2.77047	0.88959
O	1.04593	1.52929	-0.56542
C	1.99542	0.79476	0.28339
H	2.92872	1.36918	0.34062
H	1.60397	0.69550	1.30010

C	2.32242	-0.57455	-0.33100
H	2.78690	-0.44247	-1.31015
H	3.02485	-1.09368	0.33766
O	1.15955	-1.43460	-0.60573
C	0.32644	-1.77745	0.55872
H	0.43912	-2.84998	0.75879
H	0.67314	-1.24012	1.44711
C	-1.15634	-1.48999	0.26675
H	-1.72511	-1.74710	1.18517
H	-1.49349	-2.16018	-0.53205
C	-2.84576	0.04584	-0.62528
H	-2.99751	1.05888	-1.00918
H	-3.56464	-0.13041	0.19684
H	-3.05173	-0.66389	-1.43175

ACE2_Li_B2PLYP_SV(P)

33

O	-0.32173	-1.69179	0.06627
O	-0.24039	1.38925	-0.39283
O	-2.17498	0.14096	0.86884
N	1.97280	-0.07758	0.30416
C	2.03944	-1.46283	-0.17926
C	2.02317	0.93201	-0.76818
C	0.74689	-1.90848	-0.84804
C	1.01243	2.04411	-0.52225
C	-1.62904	-1.80469	-0.47913
C	-1.38771	2.16843	-0.10216
C	-2.58948	-1.17856	0.52019
C	-2.53615	1.17822	-0.04388
H	2.21545	-2.10942	0.69483
H	2.88276	-1.62558	-0.88199

H	1.77265	0.45758	-1.72862
H	3.03864	1.35846	-0.87523
H	0.55347	-1.34221	-1.77717
H	0.81677	-2.97860	-1.11447
H	1.24316	2.61044	0.40016
H	0.99848	2.75275	-1.37005
H	-1.89674	-2.86333	-0.65136
H	-1.67020	-1.27771	-1.44977
H	-1.57257	2.92306	-0.88766
H	-1.26200	2.69134	0.86506
H	-3.61732	-1.18076	0.11915
H	-2.58215	-1.75377	1.45808
H	-3.46296	1.67518	0.29149
H	-2.70689	0.76139	-1.05013
C	2.96933	0.18788	1.34170
H	2.81092	-0.48923	2.19558
H	2.86286	1.22245	1.70332
H	4.00707	0.05449	0.97125
Li	-0.07914	0.00419	1.03543

ACE2_Li_B2PLYP_SVP

33

O	-0.32016	-1.69219	0.06881
O	-0.24215	1.38655	-0.39031
O	-2.17864	0.13935	0.87124
N	1.97398	-0.07588	0.30456
C	2.04000	-1.46079	-0.17858
C	2.01992	0.93197	-0.76880
C	0.74879	-1.90460	-0.84654
C	1.01006	2.04231	-0.52250
C	-1.62647	-1.80089	-0.48026

C	-1.39047	2.16513	-0.10126
C	-2.58902	-1.18001	0.51726
C	-2.53697	1.17565	-0.04378
H	2.21687	-2.10597	0.69334
H	2.88074	-1.62478	-0.87982
H	1.76673	0.45698	-1.72557
H	3.03171	1.35867	-0.88124
H	0.55562	-1.33585	-1.77155
H	0.81955	-2.97031	-1.11969
H	1.24236	2.60908	0.39641
H	0.99603	2.74992	-1.36780
H	-1.89619	-2.85505	-0.66002
H	-1.66472	-1.27112	-1.44689
H	-1.57638	2.91840	-0.88415
H	-1.26732	2.68863	0.86320
H	-3.61301	-1.18592	0.11355
H	-2.58431	-1.75882	1.45026
H	-3.46324	1.67280	0.28420
H	-2.70385	0.75872	-1.04817
C	2.97451	0.19002	1.33726
H	2.82429	-0.48752	2.18792
H	2.86885	1.22000	1.70192
H	4.00779	0.06132	0.96334
Li	-0.07796	0.00321	1.04140

ACE2_Li_B2PLYP_SV

33

O	-0.32173	-1.69179	0.06627
O	-0.24039	1.38925	-0.39283
O	-2.17498	0.14096	0.86884
N	1.97280	-0.07758	0.30416

C	2.03944	-1.46283	-0.17926
C	2.02317	0.93201	-0.76818
C	0.74689	-1.90848	-0.84804
C	1.01243	2.04411	-0.52225
C	-1.62904	-1.80469	-0.47913
C	-1.38771	2.16843	-0.10216
C	-2.58948	-1.17856	0.52019
C	-2.53615	1.17822	-0.04388
H	2.21545	-2.10942	0.69483
H	2.88276	-1.62558	-0.88199
H	1.77265	0.45758	-1.72862
H	3.03864	1.35846	-0.87523
H	0.55347	-1.34221	-1.77717
H	0.81677	-2.97860	-1.11447
H	1.24316	2.61044	0.40016
H	0.99848	2.75275	-1.37005
H	-1.89674	-2.86333	-0.65136
H	-1.67020	-1.27771	-1.44977
H	-1.57257	2.92306	-0.88766
H	-1.26200	2.69134	0.86506
H	-3.61732	-1.18076	0.11915
H	-2.58215	-1.75377	1.45808
H	-3.46296	1.67518	0.29149
H	-2.70689	0.76139	-1.05013
C	2.96933	0.18788	1.34170
H	2.81092	-0.48923	2.19558
H	2.86286	1.22245	1.70332
H	4.00707	0.05449	0.97125
Li	-0.07914	0.00419	1.03543

ACE2_Li_B2PLYP_TZVP

33

O	-0.30981	-1.70730	0.07995
O	-0.25844	1.36306	-0.42972
O	-2.19743	0.13190	0.89472
N	1.97031	-0.06555	0.31388
C	2.04683	-1.44624	-0.18303
C	2.01912	0.94633	-0.75845
C	0.75918	-1.88377	-0.85382
C	0.99475	2.03827	-0.51475
C	-1.61429	-1.77918	-0.49513
C	-1.39959	2.14683	-0.10057
C	-2.58488	-1.19115	0.50802
C	-2.54888	1.16771	-0.03355
H	2.22858	-2.09290	0.67584
H	2.87937	-1.59101	-0.88424
H	1.78892	0.47480	-1.71275
H	3.01899	1.38273	-0.84638
H	0.55102	-1.29223	-1.74915
H	0.83279	-2.93328	-1.14953
H	1.19172	2.57667	0.41802
H	0.98892	2.75734	-1.33787
H	-1.88464	-2.81632	-0.71206
H	-1.62697	-1.21622	-1.43212
H	-1.59222	2.90565	-0.86321
H	-1.24805	2.64493	0.86242
H	-3.59720	-1.19818	0.09919
H	-2.57694	-1.77909	1.42441
H	-3.46113	1.66633	0.29892
H	-2.72229	0.74353	-1.02326
C	2.99267	0.19190	1.33014

H	2.85655	-0.48816	2.16938
H	2.89353	1.21172	1.69852
H	4.00823	0.06471	0.93429
Li	-0.06177	-0.00256	1.05985

ACE2_Li_B2PLYP_TZV

33

O	-0.32587	-1.72940	0.07584
O	-0.23902	1.39379	-0.40198
O	-2.19461	0.14857	0.89550
N	1.98089	-0.07898	0.31497
C	2.06040	-1.47795	-0.19328
C	2.05755	0.94736	-0.76868
C	0.77175	-1.91412	-0.88259
C	1.05337	2.07114	-0.52752
C	-1.67302	-1.82000	-0.49499
C	-1.41761	2.20448	-0.10214
C	-2.62393	-1.21094	0.52593
C	-2.56750	1.21331	-0.05590
H	2.23857	-2.12703	0.66380
H	2.89697	-1.61225	-0.89258
H	1.82303	0.48137	-1.72482
H	3.06843	1.36307	-0.84474
H	0.56290	-1.31557	-1.77202
H	0.83761	-2.96402	-1.17225
H	1.26702	2.62225	0.39200
H	1.03472	2.76637	-1.36854
H	-1.93681	-2.86243	-0.68188
H	-1.69521	-1.27164	-1.43926
H	-1.58622	2.94773	-0.88315
H	-1.28373	2.70899	0.85763

H	-3.64226	-1.19675	0.13576
H	-2.60473	-1.77556	1.45415
H	-3.48418	1.69411	0.28375
H	-2.72890	0.78598	-1.04571
C	2.99135	0.17231	1.37478
H	2.82120	-0.50647	2.20865
H	2.89097	1.19403	1.73714
H	4.01510	0.03159	1.00435
Li	-0.06628	-0.00936	1.02476

ACE2_Li_B3LYP_SV(P)

33

O	-0.32212	-1.69886	0.06297
O	-0.23922	1.40661	-0.38278
O	-2.18604	0.14101	0.86280
N	1.98616	-0.07844	0.29919
C	2.04750	-1.46815	-0.18080
C	2.02862	0.93771	-0.77128
C	0.75154	-1.92410	-0.84558
C	1.01659	2.05496	-0.52416
C	-1.63357	-1.82257	-0.47333
C	-1.39400	2.17968	-0.10230
C	-2.59731	-1.18233	0.52065
C	-2.54392	1.18398	-0.04705
H	2.22988	-2.11108	0.69627
H	2.88835	-1.63702	-0.88724
H	1.77758	0.46333	-1.73316
H	3.04334	1.36813	-0.88210
H	0.55850	-1.37116	-1.78496
H	0.82550	-2.99868	-1.09913
H	1.25417	2.63032	0.39294

H	1.00134	2.75863	-1.37811
H	-1.90092	-2.88593	-0.62686
H	-1.68074	-1.31366	-1.45529
H	-1.57941	2.93147	-0.89270
H	-1.27670	2.71012	0.86398
H	-3.62522	-1.19067	0.11588
H	-2.59438	-1.75019	1.46517
H	-3.47115	1.68269	0.29007
H	-2.71737	0.77087	-1.05619
C	2.97277	0.18956	1.34785
H	2.81249	-0.49214	2.19997
H	2.85809	1.22303	1.71515
H	4.01749	0.06485	0.98805
Li	-0.07810	0.00458	1.02866

ACE2_Li_B3LYP_SVP

33

O	-0.31966	-1.69999	0.06562
O	-0.24199	1.40423	-0.38193
O	-2.18791	0.13856	0.86503
N	1.98640	-0.07595	0.29966
C	2.04888	-1.46602	-0.17906
C	2.02481	0.93790	-0.77282
C	0.75519	-1.92116	-0.84407
C	1.01397	2.05364	-0.52523
C	-1.63060	-1.81971	-0.47502
C	-1.39773	2.17697	-0.10072
C	-2.59581	-1.18516	0.51797
C	-2.54537	1.18171	-0.04605
H	2.23169	-2.10767	0.69673
H	2.88910	-1.63667	-0.88274

H	1.77091	0.46286	-1.73175
H	3.03676	1.36823	-0.89007
H	0.56244	-1.36660	-1.78075
H	0.83047	-2.99231	-1.10383
H	1.25307	2.62970	0.38902
H	0.99894	2.75750	-1.37668
H	-1.89986	-2.87949	-0.63670
H	-1.67519	-1.30777	-1.45372
H	-1.58504	2.92923	-0.88766
H	-1.28190	2.70747	0.86376
H	-3.62134	-1.19749	0.11214
H	-2.59482	-1.75706	1.45826
H	-3.47261	1.67975	0.28616
H	-2.71713	0.76954	-1.05413
C	2.97619	0.19370	1.34495
H	2.82465	-0.48983	2.19377
H	2.86038	1.22260	1.71666
H	4.01776	0.07566	0.98258
Li	-0.07788	0.00348	1.03060

ACE2_Li_B3LYP_SV

33

O	-0.32212	-1.69886	0.06297
O	-0.23922	1.40661	-0.38278
O	-2.18604	0.14101	0.86280
N	1.98616	-0.07844	0.29919
C	2.04750	-1.46815	-0.18080
C	2.02862	0.93771	-0.77128
C	0.75154	-1.92410	-0.84558
C	1.01659	2.05496	-0.52416
C	-1.63357	-1.82257	-0.47333

C	-1.39400	2.17968	-0.10230
C	-2.59731	-1.18233	0.52065
C	-2.54392	1.18398	-0.04705
H	2.22988	-2.11108	0.69627
H	2.88835	-1.63702	-0.88724
H	1.77758	0.46333	-1.73316
H	3.04334	1.36813	-0.88210
H	0.55850	-1.37116	-1.78496
H	0.82550	-2.99868	-1.09913
H	1.25417	2.63032	0.39294
H	1.00134	2.75863	-1.37811
H	-1.90092	-2.88593	-0.62686
H	-1.68074	-1.31366	-1.45529
H	-1.57941	2.93147	-0.89270
H	-1.27670	2.71012	0.86398
H	-3.62522	-1.19067	0.11588
H	-2.59438	-1.75019	1.46517
H	-3.47115	1.68269	0.29007
H	-2.71737	0.77087	-1.05619
C	2.97277	0.18956	1.34785
H	2.81249	-0.49214	2.19997
H	2.85809	1.22303	1.71515
H	4.01749	0.06485	0.98805
Li	-0.07810	0.00458	1.02866

ACE2_Li_B3LYP_TZVPP

33

O	-0.30874	-1.71937	0.07594
O	-0.25880	1.38524	-0.42505
O	-2.20520	0.13060	0.88745
N	1.98267	-0.06472	0.30808

C	2.05667	-1.45111	-0.18138
C	2.02323	0.95222	-0.76319
C	0.76856	-1.90595	-0.84875
C	0.99957	2.05087	-0.51971
C	-1.61820	-1.80046	-0.49018
C	-1.40728	2.16042	-0.09857
C	-2.59031	-1.19661	0.50806
C	-2.55678	1.17520	-0.03357
H	2.24396	-2.09058	0.68168
H	2.88774	-1.60330	-0.88302
H	1.79089	0.48074	-1.71714
H	3.02197	1.39030	-0.85786
H	0.56212	-1.33404	-1.75772
H	0.85047	-2.96028	-1.12577
H	1.20357	2.59707	0.40734
H	0.99643	2.76485	-1.34779
H	-1.89019	-2.84125	-0.68884
H	-1.63687	-1.25503	-1.43779
H	-1.60434	2.91867	-0.86132
H	-1.26165	2.66226	0.86391
H	-3.60220	-1.20913	0.09760
H	-2.58709	-1.77642	1.43030
H	-3.46813	1.67290	0.30443
H	-2.73592	0.75831	-1.02573
C	2.98832	0.19962	1.34130
H	2.84816	-0.48516	2.17665
H	2.87409	1.21617	1.71531
H	4.01285	0.08599	0.96309
Li	-0.06176	-0.00533	1.03840

ACE2_Li_B3LYP_TZVP

33

O	-0.30893	-1.71933	0.07635
O	-0.25843	1.38517	-0.42407
O	-2.20536	0.13086	0.88692
N	1.98294	-0.06495	0.30773
C	2.05638	-1.45120	-0.18223
C	2.02354	0.95247	-0.76313
C	0.76798	-1.90569	-0.84901
C	0.99983	2.05102	-0.51965
C	-1.61848	-1.80070	-0.48986
C	-1.40708	2.16064	-0.09830
C	-2.59072	-1.19648	0.50789
C	-2.55660	1.17551	-0.03425
H	2.24406	-2.09141	0.68122
H	2.88760	-1.60341	-0.88506
H	1.79114	0.48099	-1.71796
H	3.02295	1.39125	-0.85742
H	0.56110	-1.33325	-1.75854
H	0.84997	-2.96089	-1.12627
H	1.20447	2.59849	0.40753
H	0.99643	2.76479	-1.34911
H	-1.89068	-2.84253	-0.68786
H	-1.63730	-1.25536	-1.43847
H	-1.60400	2.91938	-0.86190
H	-1.26217	2.66290	0.86510
H	-3.60317	-1.20904	0.09649
H	-2.58778	-1.77636	1.43110
H	-3.46872	1.67377	0.30382
H	-2.73522	0.75873	-1.02747
C	2.98854	0.19859	1.34128

H	2.84645	-0.48547	2.17792
H	2.87587	1.21651	1.71428
H	4.01386	0.08240	0.96349
Li	-0.06133	-0.00514	1.04169

ACE2_Li_B3LYP_TZV

33

O	-0.32194	-1.73039	0.07211
O	-0.24333	1.40972	-0.40324
O	-2.19779	0.14360	0.88865
N	1.98891	-0.07598	0.30905
C	2.06661	-1.47942	-0.18414
C	2.05108	0.94931	-0.77508
C	0.78209	-1.93332	-0.87082
C	1.05068	2.07680	-0.53196
C	-1.66808	-1.83369	-0.49162
C	-1.42423	2.20888	-0.09755
C	-2.62056	-1.21559	0.52257
C	-2.57073	1.21345	-0.05334
H	2.24345	-2.11781	0.68119
H	2.90583	-1.62759	-0.87744
H	1.80699	0.48017	-1.72705
H	3.06034	1.36538	-0.86789
H	0.57957	-1.36053	-1.77897
H	0.85514	-2.98968	-1.13567
H	1.27050	2.63256	0.38383
H	1.03707	2.77108	-1.37450
H	-1.92997	-2.88000	-0.66213
H	-1.69803	-1.30342	-1.44631
H	-1.60135	2.95718	-0.87263
H	-1.29265	2.71232	0.86390

H	-3.63929	-1.21170	0.13264
H	-2.60195	-1.77647	1.45381
H	-3.48762	1.69261	0.28992
H	-2.73626	0.79384	-1.04585
C	2.98414	0.18862	1.37633
H	2.82115	-0.49477	2.20842
H	2.86648	1.20648	1.74516
H	4.01528	0.06769	1.01790
Li	-0.06623	-0.00639	1.00727

ACE2_Li_B97-1_SV(P)

33

O	-0.31949	-1.70101	0.06591
O	-0.24259	1.39955	-0.38288
O	-2.19351	0.13991	0.86638
N	1.98795	-0.07583	0.29893
C	2.05065	-1.46614	-0.18023
C	2.02663	0.93884	-0.77389
C	0.75049	-1.92111	-0.84411
C	1.00781	2.05295	-0.52387
C	-1.62632	-1.81586	-0.47706
C	-1.39448	2.17353	-0.10467
C	-2.59644	-1.18174	0.51952
C	-2.54772	1.17707	-0.04721
H	2.23348	-2.10939	0.69886
H	2.89167	-1.63539	-0.88891
H	1.77506	0.46214	-1.73661
H	3.04104	1.37383	-0.88579
H	0.55612	-1.36298	-1.78260
H	0.82571	-2.99613	-1.10322
H	1.24576	2.62829	0.39563

H	0.98962	2.75904	-1.37804
H	-1.89674	-2.87836	-0.64297
H	-1.66856	-1.29520	-1.45541
H	-1.58008	2.92474	-0.89797
H	-1.27769	2.70672	0.86225
H	-3.62497	-1.19504	0.11152
H	-2.59225	-1.75467	1.46290
H	-3.47645	1.67924	0.28586
H	-2.71895	0.76070	-1.05753
C	2.97985	0.19574	1.34264
H	2.82395	-0.48662	2.19708
H	2.86266	1.23104	1.70909
H	4.02441	0.07308	0.97717
Li	-0.06919	0.00616	1.05803

ACE2_Li_B97-1_SVP

33

O	-0.31742	-1.70287	0.06848
O	-0.24465	1.39777	-0.38229
O	-2.19517	0.13797	0.86847
N	1.98799	-0.07378	0.29918
C	2.05169	-1.46442	-0.17899
C	2.02344	0.93910	-0.77520
C	0.75366	-1.91884	-0.84289
C	1.00610	2.05211	-0.52467
C	-1.62397	-1.81322	-0.47849
C	-1.39762	2.17139	-0.10332
C	-2.59519	-1.18413	0.51715
C	-2.54875	1.17540	-0.04643
H	2.23528	-2.10633	0.69847
H	2.89175	-1.63540	-0.88499

H	1.76938	0.46233	-1.73510
H	3.03512	1.37345	-0.89298
H	0.55914	-1.35927	-1.77843
H	0.83037	-2.99013	-1.10828
H	1.24536	2.62767	0.39210
H	0.98860	2.75867	-1.37581
H	-1.89666	-2.87171	-0.65241
H	-1.66343	-1.28981	-1.45346
H	-1.58515	2.92290	-0.89300
H	-1.28229	2.70477	0.86148
H	-3.62119	-1.20106	0.10842
H	-2.59302	-1.76071	1.45622
H	-3.47720	1.67700	0.28153
H	-2.71837	0.75997	-1.05547
C	2.98215	0.19901	1.34060
H	2.83393	-0.48498	2.19136
H	2.86381	1.22954	1.71137
H	4.02374	0.08279	0.97381
Li	-0.06877	0.00511	1.05900

ACE2_Li_B97-1_SV

33

O	-0.31949	-1.70101	0.06591
O	-0.24259	1.39955	-0.38288
O	-2.19351	0.13991	0.86638
N	1.98795	-0.07583	0.29893
C	2.05065	-1.46614	-0.18023
C	2.02663	0.93884	-0.77389
C	0.75049	-1.92111	-0.84411
C	1.00781	2.05295	-0.52387
C	-1.62632	-1.81586	-0.47706

C	-1.39448	2.17353	-0.10467
C	-2.59644	-1.18174	0.51952
C	-2.54772	1.17707	-0.04721
H	2.23348	-2.10939	0.69886
H	2.89167	-1.63539	-0.88891
H	1.77506	0.46214	-1.73661
H	3.04104	1.37383	-0.88579
H	0.55612	-1.36298	-1.78260
H	0.82571	-2.99613	-1.10322
H	1.24576	2.62829	0.39563
H	0.98962	2.75904	-1.37804
H	-1.89674	-2.87836	-0.64297
H	-1.66856	-1.29520	-1.45541
H	-1.58008	2.92474	-0.89797
H	-1.27769	2.70672	0.86225
H	-3.62497	-1.19504	0.11152
H	-2.59225	-1.75467	1.46290
H	-3.47645	1.67924	0.28586
H	-2.71895	0.76070	-1.05753
C	2.97985	0.19574	1.34264
H	2.82395	-0.48662	2.19708
H	2.86266	1.23104	1.70909
H	4.02441	0.07308	0.97717
Li	-0.06919	0.00616	1.05803

ACE2_Li_B97-1_TZVPP

33

O	-0.30828	-1.72239	0.07879
O	-0.26122	1.37701	-0.43079
O	-2.21135	0.13045	0.89276
N	1.98240	-0.06356	0.31032

C	2.05752	-1.44934	-0.18269
C	2.02379	0.95288	-0.76277
C	0.76322	-1.89942	-0.85006
C	0.99161	2.04852	-0.51814
C	-1.61246	-1.79218	-0.49412
C	-1.40458	2.15414	-0.10116
C	-2.59102	-1.19346	0.50745
C	-2.55950	1.16897	-0.03179
H	2.24541	-2.09260	0.68056
H	2.88843	-1.59958	-0.88827
H	1.79389	0.48004	-1.71949
H	3.02303	1.39573	-0.85397
H	0.55467	-1.31619	-1.75473
H	0.84572	-2.95294	-1.13944
H	1.19254	2.59199	0.41449
H	0.98909	2.76798	-1.34465
H	-1.88935	-2.83152	-0.70645
H	-1.62581	-1.23413	-1.43776
H	-1.60390	2.91453	-0.86461
H	-1.25572	2.65652	0.86351
H	-3.60366	-1.20832	0.09254
H	-2.58776	-1.78038	1.42799
H	-3.47154	1.67240	0.30319
H	-2.73820	0.74884	-1.02557
C	2.99835	0.19944	1.33527
H	2.86383	-0.48762	2.17255
H	2.88623	1.21822	1.71068
H	4.02112	0.08529	0.94663
Li	-0.05318	-0.00038	1.07086

ACE2_Li_B97-1_TZVP

33

O	-0.30842	-1.72213	0.07908
O	-0.26093	1.37668	-0.43017
O	-2.21158	0.13067	0.89232
N	1.98267	-0.06378	0.31006
C	2.05724	-1.44938	-0.18359
C	2.02419	0.95311	-0.76263
C	0.76267	-1.89889	-0.85047
C	0.99180	2.04852	-0.51820
C	-1.61271	-1.79227	-0.49383
C	-1.40434	2.15414	-0.10087
C	-2.59134	-1.19343	0.50738
C	-2.55938	1.16924	-0.03241
H	2.24544	-2.09349	0.67993
H	2.88835	-1.59965	-0.89024
H	1.79438	0.48025	-1.72021
H	3.02408	1.39666	-0.85343
H	0.55379	-1.31502	-1.75559
H	0.84517	-2.95320	-1.14036
H	1.19314	2.59327	0.41458
H	0.98912	2.76785	-1.34599
H	-1.88973	-2.83262	-0.70571
H	-1.62641	-1.23425	-1.43839
H	-1.60359	2.91526	-0.86488
H	-1.25598	2.65677	0.86475
H	-3.60463	-1.20854	0.09181
H	-2.58813	-1.78046	1.42884
H	-3.47218	1.67325	0.30255
H	-2.73755	0.74927	-1.02722
C	2.99850	0.19837	1.33545

H	2.86188	-0.48779	2.17409
H	2.88810	1.21851	1.70975
H	4.02208	0.08144	0.94744
Li	-0.05282	0.00022	1.07441

ACE2_Li_B97-1_TZV

33

O	-0.31931	-1.73501	0.07488
O	-0.24776	1.40221	-0.41296
O	-2.20231	0.14169	0.89593
N	1.98725	-0.07303	0.31202
C	2.06947	-1.47602	-0.18351
C	2.05064	0.95096	-0.77427
C	0.78051	-1.92917	-0.87098
C	1.04148	2.07558	-0.53142
C	-1.66036	-1.82553	-0.49713
C	-1.42339	2.20247	-0.09981
C	-2.61906	-1.21425	0.52160
C	-2.57428	1.20617	-0.04841
H	2.24754	-2.11723	0.68291
H	2.90950	-1.62137	-0.88021
H	1.80949	0.47990	-1.72923
H	3.06057	1.37243	-0.86349
H	0.57603	-1.34822	-1.77713
H	0.85653	-2.98581	-1.14462
H	1.25648	2.62835	0.39086
H	1.03041	2.77562	-1.37257
H	-1.92787	-2.87049	-0.68286
H	-1.68412	-1.28053	-1.44713
H	-1.60610	2.95293	-0.87511
H	-1.28609	2.70628	0.86377

H	-3.63892	-1.21283	0.12725
H	-2.59995	-1.78376	1.45058
H	-3.49136	1.69085	0.29451
H	-2.74203	0.78352	-1.04243
C	2.99030	0.19255	1.37287
H	2.83107	-0.49121	2.20854
H	2.87403	1.21365	1.74068
H	4.02101	0.07025	1.00678
Li	-0.06126	-0.00622	1.02275

ACE2_Li_CAM-B3LYP_SV(P)

33

O	-0.32232	-1.68308	0.05958
O	-0.23733	1.39439	-0.38579
O	-2.16351	0.13970	0.85924
N	1.97057	-0.07943	0.30039
C	2.03470	-1.46246	-0.17702
C	2.01922	0.92810	-0.76747
C	0.74451	-1.91082	-0.84376
C	1.01167	2.04081	-0.52382
C	-1.62652	-1.80776	-0.47336
C	-1.38304	2.16723	-0.10334
C	-2.58274	-1.17420	0.52239
C	-2.52894	1.17694	-0.04113
H	2.21116	-2.10642	0.69871
H	2.87813	-1.62802	-0.87808
H	1.76844	0.45323	-1.72755
H	3.03461	1.35331	-0.87572
H	0.55535	-1.35360	-1.77972
H	0.81286	-2.98336	-1.10023
H	1.24687	2.61297	0.39412

H	0.99667	2.74506	-1.37512
H	-1.89119	-2.86916	-0.63206
H	-1.67542	-1.29287	-1.45063
H	-1.56864	2.91730	-0.89295
H	-1.26006	2.69682	0.86080
H	-3.61130	-1.17548	0.12347
H	-2.57703	-1.74590	1.46275
H	-3.45368	1.67318	0.30087
H	-2.70646	0.76380	-1.04815
C	2.95266	0.18853	1.34447
H	2.79178	-0.48923	2.19764
H	2.84098	1.22223	1.70780
H	3.99430	0.05999	0.98311
Li	-0.08174	0.00529	1.01408

ACE2_Li_CAM-B3LYP_SVP

33

O	-0.32027	-1.68453	0.06203
O	-0.23952	1.39240	-0.38503
O	-2.16547	0.13776	0.86136
N	1.97074	-0.07734	0.30078
C	2.03561	-1.46049	-0.17576
C	2.01594	0.92834	-0.76856
C	0.74753	-1.90814	-0.84240
C	1.00957	2.03959	-0.52470
C	-1.62400	-1.80512	-0.47464
C	-1.38607	2.16472	-0.10198
C	-2.58148	-1.17643	0.51983
C	-2.52992	1.17510	-0.04029
H	2.21292	-2.10356	0.69860
H	2.87829	-1.62765	-0.87472

H	1.76288	0.45304	-1.72621
H	3.02890	1.35356	-0.88241
H	0.55838	-1.34920	-1.77579
H	0.81733	-2.97751	-1.10509
H	1.24606	2.61245	0.39079
H	0.99507	2.74421	-1.37371
H	-1.89097	-2.86319	-0.64079
H	-1.67037	-1.28766	-1.44915
H	-1.57360	2.91535	-0.88844
H	-1.26444	2.69461	0.86055
H	-3.60785	-1.18146	0.11955
H	-2.57806	-1.75185	1.45640
H	-3.45473	1.67119	0.29679
H	-2.70590	0.76262	-1.04641
C	2.95557	0.19165	1.34189
H	2.80204	-0.48728	2.19238
H	2.84373	1.22144	1.70888
H	3.99444	0.06825	0.97856
Li	-0.08124	0.00458	1.01588

ACE2_Li_CAM-B3LYP_SV

33

O	-0.32232	-1.68308	0.05958
O	-0.23733	1.39439	-0.38579
O	-2.16351	0.13970	0.85924
N	1.97057	-0.07943	0.30039
C	2.03470	-1.46246	-0.17702
C	2.01922	0.92810	-0.76747
C	0.74451	-1.91082	-0.84376
C	1.01167	2.04081	-0.52382
C	-1.62652	-1.80776	-0.47336

C	-1.38304	2.16723	-0.10334
C	-2.58274	-1.17420	0.52239
C	-2.52894	1.17694	-0.04113
H	2.21116	-2.10642	0.69871
H	2.87813	-1.62802	-0.87808
H	1.76844	0.45323	-1.72755
H	3.03461	1.35331	-0.87572
H	0.55535	-1.35360	-1.77972
H	0.81286	-2.98336	-1.10023
H	1.24687	2.61297	0.39412
H	0.99667	2.74506	-1.37512
H	-1.89119	-2.86916	-0.63206
H	-1.67542	-1.29287	-1.45063
H	-1.56864	2.91730	-0.89295
H	-1.26006	2.69682	0.86080
H	-3.61130	-1.17548	0.12347
H	-2.57703	-1.74590	1.46275
H	-3.45368	1.67318	0.30087
H	-2.70646	0.76380	-1.04815
C	2.95266	0.18853	1.34447
H	2.79178	-0.48923	2.19764
H	2.84098	1.22223	1.70780
H	3.99430	0.05999	0.98311
Li	-0.08174	0.00529	1.01408

ACE2_Li_CAM-B3LYP_TZVPP

33

O	-0.31070	-1.70240	0.07203
O	-0.25472	1.37356	-0.42719
O	-2.18185	0.13119	0.88343
N	1.96692	-0.06770	0.30873

C	2.04129	-1.44656	-0.17808
C	2.01501	0.94097	-0.75837
C	0.75842	-1.89123	-0.84745
C	0.99671	2.03556	-0.51761
C	-1.61257	-1.78479	-0.48880
C	-1.39345	2.14794	-0.09976
C	-2.57589	-1.18620	0.51120
C	-2.53901	1.16849	-0.02932
H	2.22154	-2.08828	0.68368
H	2.87519	-1.59693	-0.87461
H	1.78315	0.47032	-1.71176
H	3.01510	1.37381	-0.84977
H	0.55616	-1.31301	-1.75235
H	0.83379	-2.94352	-1.12984
H	1.19854	2.57758	0.41138
H	0.99468	2.75174	-1.34232
H	-1.88306	-2.82395	-0.69134
H	-1.63409	-1.23472	-1.43286
H	-1.59011	2.90574	-0.86143
H	-1.24305	2.64844	0.86160
H	-3.58939	-1.19221	0.10738
H	-2.56892	-1.76932	1.43013
H	-3.44899	1.66480	0.31071
H	-2.71992	0.75098	-1.02014
C	2.96657	0.19496	1.33787
H	2.82294	-0.48395	2.17610
H	2.85868	1.21338	1.70605
H	3.98877	0.07336	0.95957
Li	-0.06790	-0.00137	1.01816

ACE2_Li_CAM-B3LYP_TZVP

33

O	-0.31108	-1.70218	0.07233
O	-0.25420	1.37334	-0.42641
O	-2.18193	0.13156	0.88301
N	1.96730	-0.06810	0.30837
C	2.04082	-1.44674	-0.17921
C	2.01569	0.94128	-0.75804
C	0.75753	-1.89071	-0.84796
C	0.99712	2.03560	-0.51739
C	-1.61308	-1.78492	-0.48843
C	-1.39301	2.14813	-0.09972
C	-2.57644	-1.18590	0.51115
C	-2.53874	1.16894	-0.02990
H	2.22146	-2.08950	0.68273
H	2.87483	-1.59709	-0.87700
H	1.78436	0.47088	-1.71259
H	3.01643	1.37497	-0.84857
H	0.55473	-1.31180	-1.75329
H	0.83274	-2.94382	-1.13087
H	1.19933	2.57881	0.41187
H	0.99489	2.75176	-1.34332
H	-1.88373	-2.82516	-0.69031
H	-1.63485	-1.23505	-1.43356
H	-1.58947	2.90632	-0.86237
H	-1.24311	2.64934	0.86238
H	-3.59055	-1.19176	0.10650
H	-2.56986	-1.76904	1.43109
H	-3.44935	1.66594	0.31037
H	-2.71950	0.75149	-1.02169
C	2.96679	0.19336	1.33804

H	2.82104	-0.48488	2.17748
H	2.86062	1.21311	1.70538
H	3.98982	0.06912	0.96031
Li	-0.06779	-0.00102	1.02140

ACE2_Li_CAM-B3LYP_TZV

33

O	-0.32383	-1.71086	0.06554
O	-0.23773	1.39969	-0.39954
O	-2.17247	0.14464	0.88024
N	1.97243	-0.07967	0.30776
C	2.04965	-1.47446	-0.18106
C	2.04055	0.93626	-0.77068
C	0.77085	-1.91813	-0.86887
C	1.04733	2.06000	-0.52925
C	-1.66136	-1.81778	-0.48800
C	-1.40927	2.19548	-0.09995
C	-2.60301	-1.20324	0.52795
C	-2.55036	1.20469	-0.05137
H	2.21994	-2.11446	0.68286
H	2.89129	-1.62128	-0.86886
H	1.79524	0.46812	-1.72142
H	3.05103	1.34575	-0.86194
H	0.57387	-1.34090	-1.77401
H	0.83707	-2.97248	-1.13770
H	1.26894	2.61347	0.38608
H	1.03361	2.75477	-1.36949
H	-1.92155	-2.86264	-0.65960
H	-1.69695	-1.28543	-1.44017
H	-1.58454	2.94058	-0.87641
H	-1.27763	2.70081	0.85894

H	-3.62380	-1.19550	0.14731
H	-2.57834	-1.76516	1.45682
H	-3.46665	1.68137	0.29219
H	-2.71596	0.78465	-1.04264
C	2.95695	0.18483	1.37275
H	2.78900	-0.49186	2.20755
H	2.84363	1.20426	1.73493
H	3.98660	0.05692	1.01769
Li	-0.06921	-0.00565	0.99152

ACE2_Li_DSDPBEP86_SV(P)

33

O	-0.32129	-1.68447	0.06554
O	-0.24062	1.37034	-0.41305
O	-2.16576	0.14164	0.87818
N	1.96162	-0.07885	0.30817
C	2.03161	-1.45897	-0.17957
C	2.02351	0.92669	-0.76294
C	0.73997	-1.88884	-0.85525
C	1.00834	2.03168	-0.52065
C	-1.62391	-1.78392	-0.48573
C	-1.37726	2.15471	-0.10508
C	-2.58069	-1.17311	0.52329
C	-2.52813	1.17158	-0.03779
H	2.19813	-2.11317	0.69361
H	2.88117	-1.61860	-0.87864
H	1.78015	0.45209	-1.72752
H	3.04198	1.35363	-0.86061
H	0.54676	-1.30120	-1.77348
H	0.80403	-2.95590	-1.14295
H	1.22667	2.58774	0.41358

H	1.00061	2.75169	-1.36152
H	-1.89426	-2.84021	-0.67948
H	-1.66138	-1.23607	-1.44736
H	-1.56591	2.91685	-0.88516
H	-1.23657	2.67041	0.86631
H	-3.61334	-1.17298	0.12880
H	-2.56469	-1.75873	1.45685
H	-3.45495	1.67270	0.29759
H	-2.70138	0.74911	-1.04350
C	2.95535	0.18360	1.34578
H	2.79076	-0.49304	2.20138
H	2.85024	1.22117	1.70541
H	3.99461	0.04523	0.97623
Li	-0.07509	0.00685	1.04188

ACE2_Li_DSDPBEP86_SVP

33

O	-0.32010	-1.68411	0.06735
O	-0.24194	1.36725	-0.41020
O	-2.16950	0.14052	0.88048
N	1.96321	-0.07779	0.30847
C	2.03162	-1.45696	-0.18012
C	2.02080	0.92677	-0.76263
C	0.74073	-1.88372	-0.85472
C	1.00601	2.02963	-0.52004
C	-1.62171	-1.77986	-0.48632
C	-1.37949	2.15103	-0.10519
C	-2.58022	-1.17390	0.52112
C	-2.52854	1.16870	-0.03816
H	2.19895	-2.10997	0.68999
H	2.87732	-1.61721	-0.87845

H	1.77548	0.45207	-1.72337
H	3.03492	1.35442	-0.86429
H	0.54773	-1.29231	-1.76726
H	0.80516	-2.94538	-1.15016
H	1.22536	2.58540	0.41070
H	0.99832	2.74837	-1.35783
H	-1.89388	-2.83107	-0.68674
H	-1.65684	-1.22962	-1.44345
H	-1.56875	2.91051	-0.88316
H	-1.24177	2.66779	0.86269
H	-3.60839	-1.17740	0.12393
H	-2.56625	-1.76254	1.44943
H	-3.45424	1.67032	0.28895
H	-2.69722	0.74540	-1.04127
C	2.96072	0.18413	1.34122
H	2.80400	-0.49304	2.19284
H	2.85657	1.21661	1.70382
H	3.99497	0.05036	0.96811
Li	-0.07371	0.00589	1.05143

ACE2_Li_DSDPBEP86_SV

33

O	-0.32129	-1.68447	0.06554
O	-0.24062	1.37034	-0.41305
O	-2.16576	0.14164	0.87818
N	1.96162	-0.07885	0.30817
C	2.03161	-1.45897	-0.17957
C	2.02351	0.92669	-0.76294
C	0.73997	-1.88884	-0.85525
C	1.00834	2.03168	-0.52065
C	-1.62391	-1.78392	-0.48573

C	-1.37726	2.15471	-0.10508
C	-2.58069	-1.17311	0.52329
C	-2.52813	1.17158	-0.03779
H	2.19813	-2.11317	0.69361
H	2.88117	-1.61860	-0.87864
H	1.78015	0.45209	-1.72752
H	3.04198	1.35363	-0.86061
H	0.54676	-1.30120	-1.77348
H	0.80403	-2.95590	-1.14295
H	1.22667	2.58774	0.41358
H	1.00061	2.75169	-1.36152
H	-1.89426	-2.84021	-0.67948
H	-1.66138	-1.23607	-1.44736
H	-1.56591	2.91685	-0.88516
H	-1.23657	2.67041	0.86631
H	-3.61334	-1.17298	0.12880
H	-2.56469	-1.75873	1.45685
H	-3.45495	1.67270	0.29759
H	-2.70138	0.74911	-1.04350
C	2.95535	0.18360	1.34578
H	2.79076	-0.49304	2.20138
H	2.85024	1.22117	1.70541
H	3.99461	0.04523	0.97623
Li	-0.07509	0.00685	1.04188

ACE2_Li_DSDPBEP86_TZVPP

33

O	-0.30833	-1.70106	0.07941
O	-0.25979	1.34148	-0.45178
O	-2.18804	0.13065	0.90733
N	1.95718	-0.06582	0.32009

C	2.03950	-1.44076	-0.18138
C	2.01829	0.94032	-0.75220
C	0.75249	-1.86184	-0.85996
C	0.98755	2.02413	-0.51326
C	-1.60558	-1.75400	-0.50392
C	-1.38907	2.12972	-0.10550
C	-2.57461	-1.18566	0.50979
C	-2.54148	1.15781	-0.02389
H	2.21174	-2.09378	0.67512
H	2.87646	-1.57999	-0.87817
H	1.79679	0.46772	-1.70839
H	3.01841	1.37796	-0.82890
H	0.54482	-1.24619	-1.73961
H	0.82067	-2.90438	-1.18007
H	1.17003	2.54915	0.43049
H	0.98705	2.75372	-1.32685
H	-1.87908	-2.78375	-0.74903
H	-1.61082	-1.16458	-1.42526
H	-1.58685	2.89227	-0.86283
H	-1.22083	2.62167	0.85815
H	-3.58886	-1.18913	0.10565
H	-2.55819	-1.78685	1.41739
H	-3.44981	1.66233	0.30953
H	-2.72037	0.72716	-1.01012
C	2.98027	0.18967	1.33201
H	2.84449	-0.49213	2.17028
H	2.88047	1.21011	1.69990
H	3.99376	0.06212	0.93097
Li	-0.06168	0.00412	1.06041

ACE2_Li_DSDPBEP86_TZVP

33

O	-0.31037	-1.69844	0.07949
O	-0.25795	1.34114	-0.44819
O	-2.18890	0.13229	0.90554
N	1.95865	-0.06727	0.31929
C	2.03781	-1.44195	-0.18349
C	2.01982	0.93996	-0.75207
C	0.74950	-1.86075	-0.86087
C	0.98929	2.02391	-0.51270
C	-1.60823	-1.75472	-0.50241
C	-1.38756	2.13065	-0.10504
C	-2.57700	-1.18434	0.51031
C	-2.54068	1.15944	-0.02639
H	2.21092	-2.09766	0.67384
H	2.87590	-1.58245	-0.88272
H	1.79872	0.46747	-1.71103
H	3.02235	1.37845	-0.82860
H	0.54113	-1.24473	-1.74291
H	0.81570	-2.90605	-1.18159
H	1.17367	2.55248	0.43156
H	0.98724	2.75433	-1.32909
H	-1.88112	-2.78815	-0.74486
H	-1.61529	-1.16784	-1.42807
H	-1.58287	2.89539	-0.86457
H	-1.22116	2.62443	0.86088
H	-3.59374	-1.18792	0.10557
H	-2.56153	-1.78495	1.42134
H	-3.45203	1.66488	0.30607
H	-2.71849	0.72842	-1.01530
C	2.98327	0.18523	1.33063

H	2.84388	-0.49414	2.17318
H	2.88982	1.20934	1.69651
H	3.99857	0.05041	0.93003
Li	-0.06057	0.00487	1.07073

ACE2_Li_DSDPBEP86_TZV

33

O	-0.32761	-1.72610	0.07517
O	-0.23755	1.37352	-0.42076
O	-2.18721	0.15116	0.90602
N	1.97111	-0.08174	0.32028
C	2.05183	-1.47415	-0.19831
C	2.06245	0.94341	-0.76006
C	0.76022	-1.88959	-0.89359
C	1.05004	2.05943	-0.52353
C	-1.67043	-1.79819	-0.50114
C	-1.40515	2.19167	-0.10697
C	-2.61868	-1.20310	0.53048
C	-2.56039	1.20756	-0.04967
H	2.22446	-2.13537	0.65516
H	2.89228	-1.60188	-0.89936
H	1.84082	0.47864	-1.72392
H	3.07647	1.36281	-0.81998
H	0.54848	-1.26117	-1.76593
H	0.81986	-2.93423	-1.21444
H	1.24978	2.60103	0.40907
H	1.03705	2.76657	-1.35892
H	-1.94023	-2.83777	-0.71244
H	-1.68659	-1.22571	-1.43538
H	-1.57639	2.94029	-0.88665
H	-1.25652	2.69254	0.85650

H	-3.64186	-1.18470	0.14371
H	-2.59339	-1.77891	1.45548
H	-3.47690	1.69583	0.28996
H	-2.72573	0.77328	-1.03980
C	2.98275	0.16047	1.37875
H	2.80395	-0.51732	2.21584
H	2.89051	1.18752	1.73846
H	4.00743	0.00810	1.00602
Li	-0.06275	-0.01036	1.03514

ACE2_Li_HSE06_SV(P)

33

O	-0.31629	-1.68633	0.06656
O	-0.24238	1.38469	-0.37946
O	-2.17851	0.13745	0.86364
N	1.97593	-0.07461	0.29691
C	2.03820	-1.45553	-0.17575
C	2.01111	0.92930	-0.77056
C	0.74819	-1.90468	-0.83773
C	1.00029	2.03569	-0.52258
C	-1.61446	-1.79767	-0.47703
C	-1.38731	2.15625	-0.10447
C	-2.57892	-1.17612	0.51550
C	-2.53206	1.16697	-0.04595
H	2.21680	-2.09751	0.70311
H	2.88113	-1.62834	-0.87845
H	1.75576	0.45053	-1.72954
H	3.02428	1.36078	-0.89016
H	0.55574	-1.34405	-1.77326
H	0.82190	-2.97648	-1.10196
H	1.23914	2.61160	0.39415

H	0.98086	2.74099	-1.37475
H	-1.88378	-2.85638	-0.65487
H	-1.65465	-1.26883	-1.44918
H	-1.57180	2.90630	-0.89629
H	-1.27075	2.69022	0.85990
H	-3.60678	-1.18978	0.11086
H	-2.57387	-1.75330	1.45413
H	-3.46130	1.66722	0.28187
H	-2.70067	0.74884	-1.05420
C	2.95981	0.19988	1.33246
H	2.80746	-0.47835	2.18833
H	2.84248	1.23380	1.69686
H	4.00294	0.07888	0.96834
Li	-0.06935	0.00502	1.05919

ACE2_Li_HSE06_SVP

33

O	-0.31517	-1.68844	0.06905
O	-0.24347	1.38347	-0.37858
O	-2.18004	0.13598	0.86580
N	1.97585	-0.07320	0.29720
C	2.03844	-1.45456	-0.17409
C	2.00819	0.92841	-0.77223
C	0.75043	-1.90226	-0.83646
C	0.99978	2.03458	-0.52388
C	-1.61298	-1.79442	-0.47863
C	-1.38940	2.15461	-0.10270
C	-2.57829	-1.17770	0.51301
C	-2.53224	1.16595	-0.04504
H	2.21717	-2.09515	0.70350
H	2.88059	-1.62990	-0.87386

H	1.74915	0.44881	-1.72786
H	3.01900	1.35828	-0.89865
H	0.55819	-1.33905	-1.76870
H	0.82537	-2.97019	-1.10808
H	1.24078	2.61101	0.38993
H	0.98131	2.74009	-1.37346
H	-1.88530	-2.84889	-0.66509
H	-1.65000	-1.26222	-1.44727
H	-1.57595	2.90520	-0.89090
H	-1.27432	2.68873	0.85976
H	-3.60373	-1.19448	0.10745
H	-2.57564	-1.75870	1.44739
H	-3.46140	1.66591	0.27722
H	-2.69889	0.74863	-1.05222
C	2.96172	0.20246	1.33064
H	2.81651	-0.47720	2.18315
H	2.84355	1.23188	1.69923
H	4.00216	0.08746	0.96569
Li	-0.06797	0.00387	1.05950

ACE2_Li_HSE06_SV

33

O	-0.31647	-1.68684	0.06668
O	-0.24218	1.38498	-0.37909
O	-2.17843	0.13754	0.86352
N	1.97586	-0.07464	0.29685
C	2.03813	-1.45561	-0.17547
C	2.01095	0.92903	-0.77078
C	0.74817	-1.90509	-0.83742
C	1.00054	2.03584	-0.52272
C	-1.61458	-1.79771	-0.47713

C	-1.38721	2.15652	-0.10443
C	-2.57899	-1.17595	0.51532
C	-2.53189	1.16715	-0.04595
H	2.21685	-2.09740	0.70351
H	2.88103	-1.62864	-0.87818
H	1.75484	0.45015	-1.72950
H	3.02428	1.35998	-0.89107
H	0.55570	-1.34468	-1.77309
H	0.82210	-2.97691	-1.10146
H	1.23968	2.61203	0.39376
H	0.98093	2.74087	-1.37509
H	-1.88421	-2.85632	-0.65507
H	-1.65444	-1.26883	-1.44928
H	-1.57175	2.90635	-0.89645
H	-1.27071	2.69075	0.85979
H	-3.60680	-1.18937	0.11057
H	-2.57415	-1.75307	1.45399
H	-3.46111	1.66744	0.28192
H	-2.70060	0.74915	-1.05424
C	2.95984	0.20012	1.33224
H	2.80736	-0.47767	2.18845
H	2.84275	1.23425	1.69610
H	4.00292	0.07871	0.96814
Li	-0.06960	0.00482	1.05945

ACE2_Li_HSE06_TZVPP

33

O	-0.30563	-1.70595	0.07941
O	-0.26043	1.36125	-0.42626
O	-2.19748	0.12850	0.89022
N	1.97195	-0.06325	0.30773

C	2.04403	-1.43899	-0.17926
C	2.00847	0.94340	-0.75834
C	0.75920	-1.88069	-0.84394
C	0.98418	2.03023	-0.51381
C	-1.60075	-1.77243	-0.49282
C	-1.39667	2.13570	-0.10198
C	-2.57372	-1.18701	0.50417
C	-2.54304	1.15815	-0.03135
H	2.22818	-2.08242	0.68363
H	2.87651	-1.59358	-0.88056
H	1.77476	0.46958	-1.71285
H	3.00698	1.38413	-0.85700
H	0.55228	-1.29270	-1.74511
H	0.83882	-2.93111	-1.14124
H	1.18564	2.57247	0.41842
H	0.98066	2.75192	-1.33692
H	-1.87651	-2.80824	-0.71776
H	-1.61281	-1.20602	-1.43067
H	-1.59447	2.89504	-0.86531
H	-1.24861	2.64058	0.86048
H	-3.58618	-1.20296	0.09147
H	-2.56979	-1.77876	1.42047
H	-3.45666	1.66123	0.29644
H	-2.71840	0.73513	-1.02369
C	2.98009	0.20130	1.32365
H	2.84947	-0.48220	2.16308
H	2.86982	1.21964	1.69763
H	4.00185	0.08750	0.93512
Li	-0.05585	-0.00159	1.06849

ACE2_Li_HSE06_TZVP

33

O	-0.30599	-1.70556	0.07964
O	-0.25996	1.36108	-0.42536
O	-2.19789	0.12892	0.88968
N	1.97238	-0.06359	0.30743
C	2.04363	-1.43915	-0.18041
C	2.00906	0.94375	-0.75809
C	0.75836	-1.88031	-0.84444
C	0.98456	2.03039	-0.51363
C	-1.60132	-1.77271	-0.49236
C	-1.39636	2.13589	-0.10171
C	-2.57430	-1.18683	0.50426
C	-2.54292	1.15856	-0.03222
H	2.22820	-2.08350	0.68260
H	2.87612	-1.59376	-0.88293
H	1.77553	0.47017	-1.71354
H	3.00812	1.38523	-0.85624
H	0.55098	-1.29200	-1.74616
H	0.83788	-2.93154	-1.14194
H	1.18649	2.57391	0.41868
H	0.98084	2.75186	-1.33803
H	-1.87716	-2.80960	-0.71638
H	-1.61387	-1.20674	-1.43132
H	-1.59390	2.89581	-0.86572
H	-1.24903	2.64107	0.86164
H	-3.58737	-1.20310	0.09097
H	-2.57038	-1.77841	1.42159
H	-3.45723	1.66224	0.29545
H	-2.71764	0.73570	-1.02555
C	2.98073	0.19971	1.32365

H	2.84769	-0.48252	2.16462
H	2.87279	1.21952	1.69625
H	4.00314	0.08244	0.93573
Li	-0.05530	-0.00100	1.07212

ACE2_Li_HSE06_TZV

33

O	-0.31676	-1.72049	0.07497
O	-0.24600	1.38806	-0.40513
O	-2.18687	0.14007	0.89121
N	1.97558	-0.07277	0.30861
C	2.05470	-1.46539	-0.17913
C	2.03375	0.93998	-0.77095
C	0.77614	-1.91119	-0.86354
C	1.03404	2.05649	-0.52870
C	-1.64781	-1.80587	-0.49530
C	-1.41449	2.18415	-0.10023
C	-2.59986	-1.20643	0.51833
C	-2.55588	1.19482	-0.04860
H	2.22940	-2.10586	0.68716
H	2.89621	-1.61550	-0.87114
H	1.78801	0.46747	-1.72288
H	3.04293	1.35838	-0.86879
H	0.57392	-1.32638	-1.76655
H	0.85117	-2.96458	-1.14485
H	1.25293	2.61066	0.39052
H	1.02140	2.75614	-1.36849
H	-1.91641	-2.84697	-0.69312
H	-1.67034	-1.25303	-1.43963
H	-1.59563	2.93240	-0.87622
H	-1.28056	2.69170	0.86052

H	-3.61952	-1.20713	0.12677
H	-2.58006	-1.77991	1.44348
H	-3.47483	1.67854	0.28656
H	-2.71943	0.77044	-1.04152
C	2.96821	0.19584	1.36143
H	2.81085	-0.48211	2.20047
H	2.85370	1.21707	1.72543
H	3.99853	0.07264	0.99837
Li	-0.06236	-0.00674	1.02182

ACE2_Li_M062x_SV(P)

33

O	-0.31214	-1.66622	0.06240
O	-0.25229	1.35003	-0.39974
O	-2.17457	0.12862	0.88260
N	1.97046	-0.07014	0.30791
C	2.04147	-1.45006	-0.17576
C	2.01644	0.93306	-0.76465
C	0.74904	-1.87815	-0.84970
C	0.98460	2.02267	-0.52458
C	-1.60763	-1.76716	-0.49316
C	-1.38998	2.13439	-0.11329
C	-2.57434	-1.18372	0.52204
C	-2.54194	1.15341	-0.02904
H	2.20916	-2.10101	0.69754
H	2.88810	-1.60766	-0.87478
H	1.78218	0.45271	-1.72737
H	3.02585	1.37477	-0.86007
H	0.55866	-1.29270	-1.76915
H	0.80712	-2.94428	-1.13302
H	1.20170	2.58881	0.40223

H	0.95828	2.73316	-1.36981
H	-1.86408	-2.81942	-0.71157
H	-1.64362	-1.19636	-1.44054
H	-1.57290	2.87973	-0.90728
H	-1.25143	2.66697	0.84718
H	-3.60451	-1.19061	0.12721
H	-2.55089	-1.77967	1.44680
H	-3.46211	1.65712	0.31205
H	-2.72502	0.72647	-1.03005
C	2.96914	0.19967	1.33686
H	2.81860	-0.47881	2.19094
H	2.85985	1.23470	1.69729
H	4.00266	0.06909	0.95509
Li	-0.06652	0.01124	1.05922

ACE2_Li_M062x_SVP

33

O	-0.31092	-1.66883	0.06464
O	-0.25394	1.34864	-0.40083
O	-2.17548	0.12718	0.88477
N	1.97004	-0.06867	0.30880
C	2.04168	-1.44836	-0.17579
C	2.01454	0.93390	-0.76434
C	0.75069	-1.87543	-0.84948
C	0.98328	2.02210	-0.52444
C	-1.60619	-1.76453	-0.49415
C	-1.39208	2.13260	-0.11221
C	-2.57351	-1.18519	0.52026
C	-2.54254	1.15235	-0.02756
H	2.21084	-2.09897	0.69482
H	2.88664	-1.60572	-0.87333

H	1.77998	0.45402	-1.72491
H	3.02137	1.37532	-0.86238
H	0.55907	-1.28741	-1.76476
H	0.80997	-2.93748	-1.13969
H	1.19980	2.58709	0.40090
H	0.95851	2.73367	-1.36604
H	-1.86498	-2.81243	-0.71993
H	-1.63911	-1.19065	-1.43756
H	-1.57688	2.87847	-0.90232
H	-1.25341	2.66448	0.84636
H	-3.60098	-1.19416	0.12441
H	-2.55228	-1.78406	1.44090
H	-3.46175	1.65572	0.30981
H	-2.72487	0.72597	-1.02689
C	2.97177	0.20025	1.33519
H	2.82688	-0.47881	2.18607
H	2.86390	1.23130	1.69860
H	4.00175	0.07267	0.95141
Li	-0.06594	0.00989	1.05647

ACE2_Li_M062x_SV

33

O	-0.31214	-1.66622	0.06240
O	-0.25229	1.35003	-0.39974
O	-2.17457	0.12862	0.88260
N	1.97046	-0.07014	0.30791
C	2.04147	-1.45006	-0.17576
C	2.01644	0.93306	-0.76465
C	0.74904	-1.87815	-0.84970
C	0.98460	2.02267	-0.52458
C	-1.60763	-1.76716	-0.49316

C	-1.38998	2.13439	-0.11329
C	-2.57434	-1.18372	0.52204
C	-2.54194	1.15341	-0.02904
H	2.20916	-2.10101	0.69754
H	2.88810	-1.60766	-0.87478
H	1.78218	0.45271	-1.72737
H	3.02585	1.37477	-0.86007
H	0.55866	-1.29270	-1.76915
H	0.80712	-2.94428	-1.13302
H	1.20170	2.58881	0.40223
H	0.95828	2.73316	-1.36981
H	-1.86408	-2.81942	-0.71157
H	-1.64362	-1.19636	-1.44054
H	-1.57290	2.87973	-0.90728
H	-1.25143	2.66697	0.84718
H	-3.60451	-1.19061	0.12721
H	-2.55089	-1.77967	1.44680
H	-3.46211	1.65712	0.31205
H	-2.72502	0.72647	-1.03005
C	2.96914	0.19967	1.33686
H	2.81860	-0.47881	2.19094
H	2.85985	1.23470	1.69729
H	4.00266	0.06909	0.95509
Li	-0.06652	0.01124	1.05922

ACE2_Li_M062x_TZVPP

33

O	-0.30513	-1.68332	0.07445
O	-0.26419	1.33695	-0.44248
O	-2.18773	0.12449	0.89959
N	1.96440	-0.06382	0.31587

C	2.04470	-1.44087	-0.17927
C	2.01579	0.94293	-0.75592
C	0.75583	-1.86356	-0.85436
C	0.97817	2.01991	-0.51360
C	-1.60008	-1.75231	-0.50119
C	-1.39419	2.12274	-0.10762
C	-2.57195	-1.19058	0.51411
C	-2.54756	1.15096	-0.02216
H	2.21766	-2.09120	0.67914
H	2.88078	-1.58144	-0.87597
H	1.79614	0.47076	-1.71299
H	3.01254	1.38698	-0.83422
H	0.55409	-1.25953	-1.74413
H	0.81580	-2.91078	-1.16064
H	1.16383	2.54663	0.42917
H	0.96801	2.74976	-1.32673
H	-1.86019	-2.78506	-0.74738
H	-1.61523	-1.16102	-1.42213
H	-1.58537	2.88182	-0.86973
H	-1.22948	2.61962	0.85439
H	-3.58727	-1.19823	0.11264
H	-2.55119	-1.79098	1.42241
H	-3.45503	1.65319	0.31683
H	-2.73089	0.72353	-1.00979
C	2.98164	0.19811	1.33160
H	2.84574	-0.48133	2.17146
H	2.87946	1.21936	1.69636
H	3.99559	0.07074	0.93295
Li	-0.06117	0.01165	1.05172

ACE2_Li_M062x_TZVP

33

O	-0.30535	-1.68281	0.07450
O	-0.26396	1.33627	-0.44162
O	-2.18831	0.12449	0.89948
N	1.96497	-0.06392	0.31568
C	2.04446	-1.44079	-0.18031
C	2.01664	0.94354	-0.75553
C	0.75525	-1.86290	-0.85494
C	0.97812	2.01987	-0.51403
C	-1.60037	-1.75221	-0.50123
C	-1.39410	2.12254	-0.10770
C	-2.57243	-1.19067	0.51387
C	-2.54782	1.15125	-0.02220
H	2.21764	-2.09212	0.67814
H	2.88073	-1.58126	-0.87791
H	1.79865	0.47154	-1.71377
H	3.01369	1.38883	-0.83230
H	0.55287	-1.25865	-1.74516
H	0.81503	-2.91090	-1.16133
H	1.16395	2.54868	0.42830
H	0.96727	2.74885	-1.32895
H	-1.86034	-2.78587	-0.74717
H	-1.61557	-1.16097	-1.42294
H	-1.58513	2.88149	-0.87105
H	-1.22965	2.62061	0.85452
H	-3.58829	-1.19828	0.11184
H	-2.55186	-1.79126	1.42291
H	-3.45542	1.65436	0.31759
H	-2.73191	0.72399	-1.01046
C	2.98240	0.19661	1.33188

H	2.84377	-0.48129	2.17338
H	2.88274	1.21935	1.69504
H	3.99689	0.06559	0.93399
Li	-0.06058	0.01256	1.05593

ACE2_Li_M062x_TZV

33

O	-0.31740	-1.70001	0.06768
O	-0.24768	1.36464	-0.41948
O	-2.17159	0.13713	0.89732
N	1.96586	-0.07502	0.31512
C	2.05190	-1.46835	-0.17860
C	2.04068	0.93614	-0.76894
C	0.77005	-1.89058	-0.87625
C	1.02947	2.04486	-0.53007
C	-1.64769	-1.78303	-0.50392
C	-1.40883	2.17159	-0.10534
C	-2.59477	-1.20707	0.53100
C	-2.55638	1.18706	-0.03942
H	2.21032	-2.11536	0.68382
H	2.89856	-1.60732	-0.86198
H	1.80729	0.46461	-1.72241
H	3.04878	1.35439	-0.84710
H	0.57279	-1.28240	-1.76227
H	0.82496	-2.93791	-1.17459
H	1.23397	2.58658	0.39729
H	1.01096	2.74978	-1.36177
H	-1.90386	-2.81902	-0.72750
H	-1.67165	-1.20059	-1.42827
H	-1.58367	2.91854	-0.87999
H	-1.25853	2.67121	0.85435

H	-3.61824	-1.19972	0.15629
H	-2.55443	-1.78873	1.44784
H	-3.46990	1.66735	0.30643
H	-2.72555	0.75831	-1.02762
C	2.96064	0.19240	1.37278
H	2.79148	-0.47785	2.21310
H	2.85348	1.21720	1.72369
H	3.98488	0.05344	1.00675
Li	-0.06323	0.00013	1.00886

ACE2_Li_M06_SV(P)

33

O	-0.32866	-1.70612	0.07598
O	-0.24271	1.35766	-0.39908
O	-2.20495	0.14630	0.87337
N	1.99622	-0.08109	0.30339
C	2.01979	-1.45168	-0.20256
C	2.02868	0.94266	-0.74767
C	0.71871	-1.85415	-0.85946
C	0.99814	2.02295	-0.49786
C	-1.62592	-1.77916	-0.47114
C	-1.38042	2.13720	-0.11013
C	-2.59005	-1.16965	0.52097
C	-2.53746	1.17000	-0.04737
H	2.19915	-2.12390	0.65753
H	2.85520	-1.61857	-0.92090
H	1.80455	0.47709	-1.72500
H	3.03881	1.39352	-0.84114
H	0.50642	-1.22081	-1.74843
H	0.78343	-2.90398	-1.21242
H	1.20814	2.57686	0.44446

H	0.98402	2.75965	-1.32658
H	-1.91128	-2.82956	-0.68784
H	-1.65406	-1.22514	-1.43432
H	-1.55294	2.90498	-0.89109
H	-1.24626	2.66140	0.86186
H	-3.62070	-1.18958	0.11586
H	-2.58431	-1.75301	1.45887
H	-3.46368	1.68962	0.26660
H	-2.71117	0.74482	-1.05578
C	3.01968	0.14599	1.30956
H	2.88347	-0.55093	2.15561
H	2.94345	1.17517	1.70457
H	4.04788	0.00762	0.90275
Li	-0.06000	0.07486	1.12779

ACE2_Li_M06_SVP

33

O	-0.32777	-1.70724	0.07805
O	-0.24385	1.35581	-0.39675
O	-2.20740	0.14525	0.87600
N	1.99644	-0.07979	0.30378
C	2.01966	-1.45064	-0.20176
C	2.02575	0.94243	-0.74869
C	0.72043	-1.85140	-0.85847
C	0.99752	2.02194	-0.49727
C	-1.62483	-1.77530	-0.47230
C	-1.38318	2.13494	-0.10963
C	-2.58940	-1.17112	0.51928
C	-2.53776	1.16803	-0.04784
H	2.19957	-2.12174	0.65562
H	2.85356	-1.62006	-0.91669

H	1.79786	0.47798	-1.72283
H	3.03275	1.39148	-0.84935
H	0.50935	-1.21645	-1.74363
H	0.78591	-2.89554	-1.21931
H	1.21006	2.57518	0.44187
H	0.98426	2.75978	-1.32118
H	-1.91324	-2.81990	-0.69831
H	-1.65129	-1.21839	-1.43114
H	-1.55741	2.90188	-0.88707
H	-1.25286	2.66097	0.85887
H	-3.61715	-1.19612	0.11474
H	-2.58537	-1.75866	1.45157
H	-3.46400	1.68709	0.25709
H	-2.70765	0.74297	-1.05429
C	3.02452	0.14724	1.30562
H	2.89514	-0.54758	2.14980
H	2.95224	1.17236	1.70181
H	4.04809	0.01040	0.89702
Li	-0.05966	0.07523	1.13229

ACE2_Li_M06_SV

33

O	-0.32866	-1.70612	0.07598
O	-0.24271	1.35766	-0.39908
O	-2.20495	0.14630	0.87337
N	1.99622	-0.08109	0.30339
C	2.01979	-1.45168	-0.20256
C	2.02868	0.94266	-0.74767
C	0.71871	-1.85415	-0.85946
C	0.99814	2.02295	-0.49786
C	-1.62592	-1.77916	-0.47114

C	-1.38042	2.13720	-0.11013
C	-2.59005	-1.16965	0.52097
C	-2.53746	1.17000	-0.04737
H	2.19915	-2.12390	0.65753
H	2.85520	-1.61857	-0.92090
H	1.80455	0.47709	-1.72500
H	3.03881	1.39352	-0.84114
H	0.50642	-1.22081	-1.74843
H	0.78343	-2.90398	-1.21242
H	1.20814	2.57686	0.44446
H	0.98402	2.75965	-1.32658
H	-1.91128	-2.82956	-0.68784
H	-1.65406	-1.22514	-1.43432
H	-1.55294	2.90498	-0.89109
H	-1.24626	2.66140	0.86186
H	-3.62070	-1.18958	0.11586
H	-2.58431	-1.75301	1.45887
H	-3.46368	1.68962	0.26660
H	-2.71117	0.74482	-1.05578
C	3.01968	0.14599	1.30956
H	2.88347	-0.55093	2.15561
H	2.94345	1.17517	1.70457
H	4.04788	0.00762	0.90275
Li	-0.06000	0.07486	1.12779

ACE2_Li_M06_TZVPP

33

O	-0.32776	-1.71405	0.08430
O	-0.24753	1.34559	-0.42208
O	-2.21508	0.14708	0.88659
N	1.98767	-0.07673	0.30973

C	2.01529	-1.44569	-0.20175
C	2.02220	0.94526	-0.74449
C	0.71832	-1.84336	-0.85934
C	0.99422	2.01861	-0.48518
C	-1.62182	-1.76155	-0.47870
C	-1.38017	2.12581	-0.10792
C	-2.58716	-1.16901	0.51538
C	-2.53530	1.16415	-0.04952
H	2.19496	-2.11024	0.64655
H	2.84000	-1.60215	-0.91263
H	1.79596	0.48606	-1.70868
H	3.01986	1.38907	-0.83297
H	0.50228	-1.20449	-1.72565
H	0.78490	-2.87477	-1.22241
H	1.18807	2.53740	0.46423
H	0.99048	2.76679	-1.28448
H	-1.91210	-2.79188	-0.71400
H	-1.63515	-1.19470	-1.41839
H	-1.55744	2.89656	-0.86502
H	-1.23673	2.62442	0.86030
H	-3.60284	-1.19260	0.10947
H	-2.57934	-1.75559	1.43552
H	-3.45162	1.68005	0.25059
H	-2.69836	0.73475	-1.04253
C	3.03883	0.14224	1.29055
H	2.92149	-0.55022	2.12408
H	2.97703	1.15746	1.68385
H	4.04026	0.00181	0.85927
Li	-0.05295	0.08385	1.15340

ACE2_Li_M06_TZVP

33

O	-0.32863	-1.71283	0.08475
O	-0.24684	1.34357	-0.42028
O	-2.21847	0.14759	0.88663
N	1.98908	-0.07686	0.30975
C	2.01421	-1.44571	-0.20117
C	2.02229	0.94444	-0.74488
C	0.71737	-1.84249	-0.85817
C	0.99410	2.01694	-0.48631
C	-1.62205	-1.75991	-0.47886
C	-1.37909	2.12435	-0.10771
C	-2.58872	-1.16826	0.51371
C	-2.53529	1.16469	-0.05018
H	2.19431	-2.11093	0.64812
H	2.83968	-1.60338	-0.91311
H	1.79623	0.48381	-1.70973
H	3.02095	1.38908	-0.83413
H	0.50180	-1.20320	-1.72583
H	0.78380	-2.87502	-1.22237
H	1.18933	2.53852	0.46287
H	0.98911	2.76518	-1.28742
H	-1.91263	-2.79131	-0.71578
H	-1.63502	-1.19259	-1.41977
H	-1.55510	2.89651	-0.86569
H	-1.23602	2.62435	0.86145
H	-3.60465	-1.19228	0.10487
H	-2.58311	-1.75619	1.43447
H	-3.45221	1.68339	0.24823
H	-2.69825	0.73485	-1.04441
C	3.04229	0.14167	1.28775

H	2.92559	-0.54948	2.12385
H	2.98354	1.15895	1.67911
H	4.04361	-0.00187	0.85382
Li	-0.05124	0.08627	1.16642

ACE2_Li_M06_TZV

33

O	-0.30753	-1.73453	0.08888
O	-0.26204	1.36674	-0.40371
O	-2.20024	0.13444	0.88578
N	1.98876	-0.06292	0.30490
C	2.05759	-1.45476	-0.18173
C	2.02015	0.95035	-0.77477
C	0.77595	-1.89821	-0.85599
C	1.00676	2.04798	-0.51754
C	-1.62903	-1.80053	-0.49114
C	-1.42608	2.16404	-0.10345
C	-2.59095	-1.21212	0.51709
C	-2.56875	1.17876	-0.05388
H	2.23676	-2.09551	0.68436
H	2.89584	-1.60255	-0.87884
H	1.77387	0.47566	-1.72645
H	3.02239	1.38382	-0.87852
H	0.56563	-1.30208	-1.75178
H	0.85604	-2.94536	-1.16104
H	1.22113	2.58682	0.41397
H	0.98361	2.76654	-1.34123
H	-1.90277	-2.83548	-0.71535
H	-1.64274	-1.23068	-1.42766
H	-1.59735	2.91823	-0.87583
H	-1.29184	2.66699	0.86126

H	-3.60954	-1.22738	0.12171
H	-2.56675	-1.78771	1.44149
H	-3.49041	1.66366	0.27249
H	-2.72808	0.75360	-1.04841
C	3.00588	0.20505	1.33180
H	2.87345	-0.47917	2.17017
H	2.89830	1.22456	1.70392
H	4.02577	0.08585	0.93772
Li	-0.06510	0.06802	1.09125

ACE2_Li_MP2_SV(P)

33

O	-0.32370	-1.68474	0.06996
O	-0.24120	1.36217	-0.41562
O	-2.16808	0.14408	0.88156
N	1.96083	-0.07887	0.31132
C	2.02716	-1.45561	-0.18436
C	2.02372	0.92689	-0.75856
C	0.73454	-1.87785	-0.85902
C	1.00755	2.02847	-0.51539
C	-1.62627	-1.77777	-0.48693
C	-1.37583	2.15133	-0.10409
C	-2.58315	-1.17131	0.52201
C	-2.52754	1.17165	-0.04004
H	2.19308	-2.11651	0.68356
H	2.87525	-1.61219	-0.88613
H	1.78245	0.45610	-1.72515
H	3.04216	1.35470	-0.85421
H	0.53774	-1.28025	-1.76924
H	0.79845	-2.94149	-1.15926
H	1.22303	2.57917	0.42219

H	1.00164	2.75273	-1.35263
H	-1.89910	-2.83190	-0.68846
H	-1.66032	-1.22367	-1.44428
H	-1.56238	2.91698	-0.88109
H	-1.23475	2.66224	0.86932
H	-3.61541	-1.16999	0.12682
H	-2.56764	-1.75974	1.45329
H	-3.45570	1.67392	0.28979
H	-2.69589	0.74832	-1.04551
C	2.97150	0.17431	1.33566
H	2.81236	-0.50123	2.19211
H	2.87979	1.21188	1.69623
H	4.00321	0.02678	0.95124
Li	-0.07667	0.00450	1.06643

ACE2_Li_MP2_SVP

33

O	-0.32228	-1.68122	0.07132
O	-0.24274	1.35605	-0.40840
O	-2.17582	0.14274	0.88511
N	1.96396	-0.07735	0.31221
C	2.02711	-1.45231	-0.18485
C	2.01968	0.92659	-0.75782
C	0.73472	-1.86986	-0.85775
C	1.00262	2.02446	-0.51489
C	-1.62246	-1.77066	-0.48805
C	-1.37851	2.14450	-0.10552
C	-2.58289	-1.17149	0.51868
C	-2.52841	1.16614	-0.04119
H	2.19356	-2.11090	0.67890
H	2.86907	-1.60882	-0.88596

H	1.77596	0.45456	-1.71810
H	3.03126	1.35620	-0.85823
H	0.53948	-1.26797	-1.76020
H	0.79811	-2.92608	-1.16626
H	1.22041	2.57611	0.41629
H	0.99420	2.74415	-1.34958
H	-1.89620	-2.81776	-0.69779
H	-1.65387	-1.21307	-1.43836
H	-1.56409	2.90340	-0.88235
H	-1.24312	2.65861	0.86174
H	-3.60810	-1.17544	0.11881
H	-2.56935	-1.76370	1.44227
H	-3.45442	1.66990	0.27558
H	-2.68873	0.74038	-1.04217
C	2.98162	0.17533	1.32654
H	2.83438	-0.49950	2.17861
H	2.89239	1.20660	1.68924
H	4.00499	0.03272	0.93445
Li	-0.07262	0.00176	1.08750

ACE2_Li_MP2_SV

33

O	-0.32370	-1.68474	0.06996
O	-0.24120	1.36217	-0.41562
O	-2.16808	0.14408	0.88156
N	1.96083	-0.07887	0.31132
C	2.02716	-1.45561	-0.18436
C	2.02372	0.92689	-0.75856
C	0.73454	-1.87785	-0.85902
C	1.00755	2.02847	-0.51539
C	-1.62627	-1.77777	-0.48693

C	-1.37583	2.15133	-0.10409
C	-2.58315	-1.17131	0.52201
C	-2.52754	1.17165	-0.04004
H	2.19308	-2.11651	0.68356
H	2.87525	-1.61219	-0.88613
H	1.78245	0.45610	-1.72515
H	3.04216	1.35470	-0.85421
H	0.53774	-1.28025	-1.76924
H	0.79845	-2.94149	-1.15926
H	1.22303	2.57917	0.42219
H	1.00164	2.75273	-1.35263
H	-1.89910	-2.83190	-0.68846
H	-1.66032	-1.22367	-1.44428
H	-1.56238	2.91698	-0.88109
H	-1.23475	2.66224	0.86932
H	-3.61541	-1.16999	0.12682
H	-2.56764	-1.75974	1.45329
H	-3.45570	1.67392	0.28979
H	-2.69589	0.74832	-1.04551
C	2.97150	0.17431	1.33566
H	2.81236	-0.50123	2.19211
H	2.87979	1.21188	1.69623
H	4.00321	0.02678	0.95124
Li	-0.07667	0.00450	1.06643

ACE2_Li_MP2_TZVP

33

O	-0.30959	-1.68531	0.08454
O	-0.26243	1.31932	-0.43814
O	-2.20061	0.13076	0.91096
N	1.95931	-0.06316	0.32527

C	2.03489	-1.43432	-0.18318
C	2.01211	0.93829	-0.75047
C	0.74684	-1.84181	-0.86053
C	0.97829	2.01430	-0.51074
C	-1.60312	-1.73836	-0.50783
C	-1.39015	2.11694	-0.10452
C	-2.57817	-1.18546	0.50359
C	-2.54433	1.15173	-0.03008
H	2.20642	-2.09436	0.66949
H	2.87143	-1.57145	-0.88333
H	1.79041	0.46293	-1.70633
H	3.01148	1.37994	-0.83118
H	0.53773	-1.21706	-1.73413
H	0.80866	-2.88286	-1.19121
H	1.16305	2.54585	0.42995
H	0.96575	2.74044	-1.32905
H	-1.87048	-2.76824	-0.76486
H	-1.60469	-1.14035	-1.42431
H	-1.57691	2.87831	-0.86754
H	-1.22792	2.61214	0.85949
H	-3.59205	-1.19247	0.09536
H	-2.56148	-1.79263	1.40829
H	-3.45639	1.65951	0.29151
H	-2.71381	0.71602	-1.01653
C	3.01530	0.18423	1.30476
H	2.89731	-0.49148	2.15109
H	2.94004	1.20857	1.66881
H	4.01436	0.04128	0.87239
Li	-0.05559	-0.00115	1.13343

ACE2_Li_MP2_TZV

33

O	-0.34425	-1.74294	0.07887
O	-0.22470	1.36286	-0.41289
O	-2.20384	0.16759	0.91476
N	1.98413	-0.09295	0.32578
C	2.04214	-1.47924	-0.23088
C	2.09210	0.95245	-0.74356
C	0.73469	-1.85658	-0.92404
C	1.06800	2.06314	-0.50994
C	-1.69979	-1.79566	-0.49888
C	-1.39664	2.20009	-0.11315
C	-2.64515	-1.19493	0.53691
C	-2.56386	1.22506	-0.05979
H	2.21867	-2.16561	0.60270
H	2.87158	-1.59301	-0.94942
H	1.89616	0.50122	-1.72074
H	3.10586	1.37899	-0.77082
H	0.50691	-1.17890	-1.75468
H	0.78357	-2.88408	-1.29960
H	1.25782	2.60130	0.42696
H	1.05088	2.77107	-1.34521
H	-1.97969	-2.83168	-0.71677
H	-1.70761	-1.21404	-1.42771
H	-1.55190	2.94210	-0.90297
H	-1.25049	2.70501	0.84861
H	-3.66740	-1.16393	0.14641
H	-2.62578	-1.76651	1.46485
H	-3.47826	1.72355	0.27196
H	-2.72548	0.78358	-1.04794
C	3.02350	0.10587	1.37915

H	2.83417	-0.58084	2.20677
H	2.96091	1.13073	1.75191
H	4.03728	-0.06977	0.98654
Li	-0.05790	-0.01989	1.09595

ACE2_Li_PBE0_SV(P)

33

O	-0.31695	-1.68612	0.06632
O	-0.24140	1.38514	-0.37956
O	-2.17747	0.13820	0.86290
N	1.97502	-0.07516	0.29658
C	2.03702	-1.45578	-0.17631
C	2.01131	0.92903	-0.77022
C	0.74665	-1.90512	-0.83762
C	1.00078	2.03568	-0.52223
C	-1.61467	-1.79807	-0.47630
C	-1.38569	2.15642	-0.10466
C	-2.57866	-1.17448	0.51557
C	-2.53064	1.16736	-0.04593
H	2.21599	-2.09787	0.70240
H	2.87957	-1.62870	-0.87941
H	1.75612	0.45094	-1.72952
H	3.02477	1.36001	-0.88920
H	0.55428	-1.34547	-1.77372
H	0.82058	-2.97725	-1.10084
H	1.23972	2.61164	0.39449
H	0.98194	2.74096	-1.37454
H	-1.88466	-2.85713	-0.65160
H	-1.65543	-1.27152	-1.44968
H	-1.57068	2.90630	-0.89664
H	-1.26913	2.69070	0.85960

H	-3.60653	-1.18786	0.11071
H	-2.57439	-1.75109	1.45466
H	-3.45953	1.66824	0.28236
H	-2.70001	0.74996	-1.05436
C	2.95825	0.19858	1.33274
H	2.80484	-0.47982	2.18837
H	2.84091	1.23247	1.69735
H	4.00161	0.07725	0.96942
Li	-0.06823	0.00497	1.05884

ACE2_Li_PBE0_SVP

33

O	-0.31530	-1.68878	0.06851
O	-0.24299	1.38435	-0.37906
O	-2.17878	0.13666	0.86487
N	1.97504	-0.07354	0.29691
C	2.03760	-1.45441	-0.17555
C	2.00858	0.92926	-0.77112
C	0.74912	-1.90292	-0.83691
C	0.99972	2.03514	-0.52290
C	-1.61291	-1.79579	-0.47730
C	-1.38846	2.15486	-0.10335
C	-2.57765	-1.17640	0.51376
C	-2.53123	1.16588	-0.04530
H	2.21724	-2.09544	0.70136
H	2.87905	-1.62913	-0.87611
H	1.75124	0.45100	-1.72764
H	3.01931	1.35956	-0.89551
H	0.55659	-1.34066	-1.76945
H	0.82472	-2.97101	-1.10775
H	1.24023	2.61132	0.39109

H	0.98246	2.74077	-1.37239
H	-1.88586	-2.85065	-0.66052
H	-1.65117	-1.26646	-1.44733
H	-1.57579	2.90508	-0.89166
H	-1.27370	2.68927	0.85893
H	-3.60301	-1.19329	0.10810
H	-2.57563	-1.75631	1.44878
H	-3.46006	1.66618	0.27744
H	-2.69860	0.74913	-1.05248
C	2.96030	0.20062	1.33115
H	2.81348	-0.47900	2.18329
H	2.84286	1.22992	1.70003
H	4.00082	0.08460	0.96730
Li	-0.06779	0.00483	1.05889

ACE2_Li_PBE0_SV

33

O	-0.31695	-1.68612	0.06632
O	-0.24140	1.38514	-0.37956
O	-2.17747	0.13820	0.86290
N	1.97502	-0.07516	0.29658
C	2.03702	-1.45578	-0.17631
C	2.01131	0.92903	-0.77022
C	0.74665	-1.90512	-0.83762
C	1.00078	2.03568	-0.52223
C	-1.61467	-1.79807	-0.47630
C	-1.38569	2.15642	-0.10466
C	-2.57866	-1.17448	0.51557
C	-2.53064	1.16736	-0.04593
H	2.21599	-2.09787	0.70240
H	2.87957	-1.62870	-0.87941

H	1.75612	0.45094	-1.72952
H	3.02477	1.36001	-0.88920
H	0.55428	-1.34547	-1.77372
H	0.82058	-2.97725	-1.10084
H	1.23972	2.61164	0.39449
H	0.98194	2.74096	-1.37454
H	-1.88466	-2.85713	-0.65160
H	-1.65543	-1.27152	-1.44968
H	-1.57068	2.90630	-0.89664
H	-1.26913	2.69070	0.85960
H	-3.60653	-1.18786	0.11071
H	-2.57439	-1.75109	1.45466
H	-3.45953	1.66824	0.28236
H	-2.70001	0.74996	-1.05436
C	2.95825	0.19858	1.33274
H	2.80484	-0.47982	2.18837
H	2.84091	1.23247	1.69735
H	4.00161	0.07725	0.96942
Li	-0.06823	0.00497	1.05884

ACE2_Li_PBE0_TZVPP

33

O	-0.30583	-1.70653	0.07935
O	-0.26006	1.36131	-0.42893
O	-2.19559	0.12890	0.89059
N	1.97005	-0.06354	0.30820
C	2.04289	-1.43858	-0.18048
C	2.00903	0.94381	-0.75694
C	0.75748	-1.88013	-0.84465
C	0.98387	2.03028	-0.51336
C	-1.60030	-1.77240	-0.49247

C	-1.39498	2.13536	-0.10226
C	-2.57296	-1.18536	0.50440
C	-2.54170	1.15790	-0.03015
H	2.22786	-2.08299	0.68180
H	2.87508	-1.59186	-0.88268
H	1.77747	0.47061	-1.71249
H	3.00785	1.38489	-0.85278
H	0.54994	-1.29139	-1.74544
H	0.83770	-2.93057	-1.14291
H	1.18389	2.57176	0.41994
H	0.98224	2.75294	-1.33605
H	-1.87740	-2.80841	-0.71633
H	-1.61234	-1.20681	-1.43109
H	-1.59451	2.89541	-0.86486
H	-1.24548	2.63972	0.86059
H	-3.58553	-1.20053	0.09117
H	-2.56997	-1.77764	1.42075
H	-3.45484	1.66185	0.29873
H	-2.71850	0.73522	-1.02263
C	2.97800	0.19926	1.32474
H	2.84665	-0.48561	2.16329
H	2.86765	1.21734	1.70011
H	3.99999	0.08566	0.93627
Li	-0.05476	-0.00170	1.06542

ACE2_Li_PBE0_TZVP

33

O	-0.30615	-1.70615	0.07959
O	-0.25962	1.36108	-0.42796
O	-2.19609	0.12931	0.89004
N	1.97052	-0.06385	0.30788

C	2.04251	-1.43871	-0.18160
C	2.00955	0.94414	-0.75674
C	0.75667	-1.87968	-0.84512
C	0.98418	2.03040	-0.51315
C	-1.60081	-1.77261	-0.49202
C	-1.39471	2.13549	-0.10201
C	-2.57351	-1.18520	0.50449
C	-2.54160	1.15826	-0.03108
H	2.22788	-2.08403	0.68084
H	2.87475	-1.59207	-0.88497
H	1.77810	0.47117	-1.71321
H	3.00892	1.38598	-0.85218
H	0.54867	-1.29055	-1.74643
H	0.83676	-2.93091	-1.14372
H	1.18470	2.57314	0.42025
H	0.98229	2.75291	-1.33707
H	-1.87800	-2.80967	-0.71512
H	-1.61331	-1.20738	-1.43172
H	-1.59391	2.89614	-0.86530
H	-1.24598	2.64018	0.86172
H	-3.58671	-1.20083	0.09070
H	-2.57044	-1.77732	1.42187
H	-3.45550	1.66281	0.29758
H	-2.71773	0.73572	-1.02455
C	2.97872	0.19776	1.32462
H	2.84511	-0.48584	2.16474
H	2.87068	1.21733	1.69863
H	4.00135	0.08080	0.93668
Li	-0.05414	-0.00121	1.06947

ACE2_Li_PBE0_TZV

33

O	-0.31704	-1.72000	0.07461
O	-0.24538	1.38834	-0.40582
O	-2.18583	0.14066	0.89080
N	1.97422	-0.07314	0.30878
C	2.05357	-1.46510	-0.18029
C	2.03391	0.94014	-0.76988
C	0.77421	-1.91086	-0.86384
C	1.03358	2.05647	-0.52796
C	-1.64729	-1.80612	-0.49439
C	-1.41285	2.18373	-0.10057
C	-2.59915	-1.20464	0.51884
C	-2.55455	1.19428	-0.04835
H	2.22923	-2.10643	0.68557
H	2.89461	-1.61440	-0.87342
H	1.78931	0.46821	-1.72266
H	3.04337	1.35892	-0.86603
H	0.57191	-1.32623	-1.76732
H	0.84967	-2.96463	-1.14516
H	1.25228	2.61058	0.39176
H	1.02214	2.75662	-1.36786
H	-1.91690	-2.84779	-0.68996
H	-1.67074	-1.25519	-1.44021
H	-1.59509	2.93220	-0.87660
H	-1.27902	2.69154	0.86047
H	-3.61903	-1.20572	0.12685
H	-2.57980	-1.77815	1.44445
H	-3.47344	1.67901	0.28683
H	-2.71862	0.77027	-1.04169
C	2.96701	0.19393	1.36160

H	2.80950	-0.48522	2.20007
H	2.85268	1.21503	1.72696
H	3.99750	0.07090	0.99818
Li	-0.06125	-0.00683	1.02019

ACE2_Li_PBE_SV(P)

33

O	-0.31323	-1.70975	0.07353
O	-0.24953	1.39197	-0.39493
O	-2.20265	0.13653	0.87915
N	1.99050	-0.07093	0.30111
C	2.05813	-1.46287	-0.17744
C	2.02565	0.94283	-0.77478
C	0.76230	-1.92119	-0.84373
C	1.00540	2.05454	-0.52755
C	-1.62270	-1.81155	-0.48485
C	-1.40357	2.17128	-0.10297
C	-2.59690	-1.19144	0.51402
C	-2.55588	1.17625	-0.04494
H	2.24240	-2.10731	0.70928
H	2.90758	-1.63613	-0.88722
H	1.77210	0.45881	-1.74152
H	3.04556	1.38214	-0.89323
H	0.56254	-1.35527	-1.78587
H	0.84378	-3.00088	-1.11337
H	1.23825	2.63237	0.40101
H	0.99095	2.76912	-1.38444
H	-1.89871	-2.87667	-0.67468
H	-1.65497	-1.27078	-1.46149
H	-1.59558	2.93407	-0.89429
H	-1.27868	2.70298	0.87275

H	-3.63177	-1.21039	0.10239
H	-2.59059	-1.77417	1.45983
H	-3.49160	1.68442	0.28482
H	-2.72675	0.75346	-1.06065
C	2.98211	0.20694	1.34444
H	2.83163	-0.48109	2.20393
H	2.85710	1.24684	1.71509
H	4.03522	0.09283	0.97774
Li	-0.06528	0.00448	1.06235

ACE2_Li_PBE_SVP

33

O	-0.31093	-1.71116	0.07613
O	-0.25186	1.38993	-0.39374
O	-2.20470	0.13427	0.88159
N	1.99077	-0.06875	0.30146
C	2.05932	-1.46121	-0.17566
C	2.02171	0.94250	-0.77670
C	0.76575	-1.91797	-0.84256
C	1.00343	2.05329	-0.52852
C	-1.61999	-1.80818	-0.48654
C	-1.40718	2.16879	-0.10136
C	-2.59550	-1.19418	0.51167
C	-2.55700	1.17389	-0.04444
H	2.24361	-2.10402	0.70956
H	2.90824	-1.63706	-0.88156
H	1.76416	0.45764	-1.73965
H	3.03857	1.38052	-0.90325
H	0.56624	-1.34948	-1.78093
H	0.84790	-2.99323	-1.12018
H	1.23828	2.63150	0.39677

H	0.98925	2.76843	-1.38199
H	-1.89767	-2.86868	-0.68598
H	-1.64937	-1.26399	-1.45911
H	-1.60069	2.93203	-0.88864
H	-1.28429	2.70071	0.87202
H	-3.62777	-1.21748	0.09981
H	-2.59104	-1.78088	1.45272
H	-3.49313	1.68066	0.27866
H	-2.72496	0.75158	-1.05867
C	2.98498	0.21093	1.34187
H	2.84427	-0.47976	2.19686
H	2.85785	1.24512	1.71844
H	4.03478	0.10545	0.97333
Li	-0.06431	0.00358	1.06440

ACE2_Li_PBE_SV

33

O	-0.31323	-1.70975	0.07353
O	-0.24953	1.39197	-0.39493
O	-2.20265	0.13653	0.87915
N	1.99050	-0.07093	0.30111
C	2.05813	-1.46287	-0.17744
C	2.02565	0.94283	-0.77478
C	0.76230	-1.92119	-0.84373
C	1.00540	2.05454	-0.52755
C	-1.62270	-1.81155	-0.48485
C	-1.40357	2.17128	-0.10297
C	-2.59690	-1.19144	0.51402
C	-2.55588	1.17625	-0.04494
H	2.24240	-2.10731	0.70928
H	2.90758	-1.63613	-0.88722

H	1.77210	0.45881	-1.74152
H	3.04556	1.38214	-0.89323
H	0.56254	-1.35527	-1.78587
H	0.84378	-3.00088	-1.11337
H	1.23825	2.63237	0.40101
H	0.99095	2.76912	-1.38444
H	-1.89871	-2.87667	-0.67468
H	-1.65497	-1.27078	-1.46149
H	-1.59558	2.93407	-0.89429
H	-1.27868	2.70298	0.87275
H	-3.63177	-1.21039	0.10239
H	-2.59059	-1.77417	1.45983
H	-3.49160	1.68442	0.28482
H	-2.72675	0.75346	-1.06065
C	2.98211	0.20694	1.34444
H	2.83163	-0.48109	2.20393
H	2.85710	1.24684	1.71509
H	4.03522	0.09283	0.97774
Li	-0.06528	0.00448	1.06235

ACE2_Li_PBE_TZVPP

33

O	-0.30297	-1.73050	0.08693
O	-0.26783	1.36852	-0.44433
O	-2.22309	0.12812	0.90659
N	1.98674	-0.05971	0.31251
C	2.06420	-1.44655	-0.18261
C	2.02352	0.95770	-0.76222
C	0.77371	-1.89564	-0.85207
C	0.99085	2.04969	-0.51798
C	-1.61090	-1.78635	-0.50085

C	-1.41373	2.15153	-0.09920
C	-2.59313	-1.20292	0.50211
C	-2.56726	1.16895	-0.03183
H	2.25406	-2.09275	0.68667
H	2.90247	-1.59977	-0.89054
H	1.79139	0.47881	-1.72347
H	3.02837	1.40456	-0.85922
H	0.55753	-1.29996	-1.75590
H	0.85948	-2.95215	-1.15603
H	1.18361	2.59079	0.42622
H	0.99243	2.78067	-1.34387
H	-1.89159	-2.82743	-0.73636
H	-1.61341	-1.20915	-1.44165
H	-1.61713	2.92453	-0.85933
H	-1.25554	2.64980	0.87441
H	-3.61164	-1.22220	0.08304
H	-2.58885	-1.79889	1.42506
H	-3.48744	1.67726	0.29713
H	-2.74229	0.73959	-1.02996
C	3.00495	0.20652	1.33572
H	2.87817	-0.48975	2.17499
H	2.88533	1.22812	1.71952
H	4.03465	0.10276	0.94220
Li	-0.04962	-0.00451	1.07989

ACE2_Li_PBE_TZVP

33

O	-0.30323	-1.73057	0.08731
O	-0.26754	1.36847	-0.44343
O	-2.22327	0.12838	0.90597
N	1.98712	-0.05988	0.31221

C	2.06388	-1.44671	-0.18328
C	2.02375	0.95790	-0.76233
C	0.77312	-1.89556	-0.85221
C	0.99105	2.04988	-0.51823
C	-1.61123	-1.78681	-0.50052
C	-1.41351	2.15184	-0.09870
C	-2.59358	-1.20279	0.50191
C	-2.56716	1.16944	-0.03238
H	2.25399	-2.09358	0.68639
H	2.90237	-1.60024	-0.89216
H	1.79149	0.47896	-1.72432
H	3.02916	1.40544	-0.85931
H	0.55657	-1.29967	-1.75673
H	0.85904	-2.95290	-1.15640
H	1.18459	2.59254	0.42587
H	0.99233	2.78049	-1.34559
H	-1.89218	-2.82892	-0.73536
H	-1.61399	-1.20997	-1.44242
H	-1.61682	2.92553	-0.85939
H	-1.25596	2.65038	0.87584
H	-3.61267	-1.22234	0.08211
H	-2.58950	-1.79877	1.42580
H	-3.48800	1.67831	0.29676
H	-2.74187	0.74055	-1.03160
C	3.00549	0.20563	1.33562
H	2.87651	-0.48917	2.17668
H	2.88800	1.22879	1.71784
H	4.03595	0.09856	0.94281
Li	-0.04912	-0.00396	1.08320

ACE2_Li_PBE_TZV

33

O	-0.31261	-1.74787	0.08666
O	-0.25757	1.38891	-0.43435
O	-2.21574	0.13809	0.91484
N	1.99210	-0.06708	0.31512
C	2.07721	-1.47127	-0.18413
C	2.05055	0.95910	-0.77317
C	0.79270	-1.92421	-0.87333
C	1.03811	2.07719	-0.53182
C	-1.65719	-1.81704	-0.50737
C	-1.43444	2.19783	-0.09725
C	-2.62145	-1.22579	0.51270
C	-2.58482	1.20635	-0.04558
H	2.25837	-2.11623	0.68777
H	2.92463	-1.61835	-0.88400
H	1.81064	0.48199	-1.73367
H	3.06544	1.38738	-0.86572
H	0.57863	-1.32866	-1.77694
H	0.87499	-2.98337	-1.16431
H	1.24056	2.62557	0.40478
H	1.03327	2.79077	-1.37124
H	-1.93094	-2.86255	-0.72385
H	-1.66559	-1.24627	-1.45110
H	-1.62382	2.96357	-0.86657
H	-1.28277	2.69460	0.87649
H	-3.64661	-1.22792	0.11199
H	-2.60320	-1.80775	1.44249
H	-3.50864	1.69758	0.29374
H	-2.75236	0.77430	-1.04340
C	3.00085	0.20079	1.37223

H	2.85007	-0.49125	2.21125
H	2.87923	1.22535	1.74789
H	4.03814	0.08715	0.99948
Li	-0.05552	-0.00901	1.04275

ACE2_Li_SCS-MP2_SV(P)

33

O	-0.32572	-1.68990	0.06852
O	-0.24063	1.37074	-0.41745
O	-2.17068	0.14492	0.88143
N	1.96506	-0.08009	0.31194
C	2.03150	-1.46044	-0.18803
C	2.03120	0.93224	-0.75865
C	0.73467	-1.88575	-0.86285
C	1.01209	2.03707	-0.51543
C	-1.63288	-1.78775	-0.48554
C	-1.37869	2.16020	-0.10406
C	-2.59018	-1.17315	0.52466
C	-2.53292	1.17695	-0.03959
H	2.19985	-2.12270	0.67940
H	2.87869	-1.61461	-0.89218
H	1.79356	0.46312	-1.72782
H	3.05025	1.36110	-0.84912
H	0.53694	-1.29130	-1.77540
H	0.79829	-2.95158	-1.15798
H	1.22600	2.58787	0.42306
H	1.00722	2.76109	-1.35383
H	-1.90452	-2.84489	-0.67666
H	-1.67002	-1.24131	-1.44764
H	-1.56581	2.92568	-0.88211
H	-1.23699	2.67096	0.87001

H	-3.62330	-1.16930	0.12973
H	-2.57569	-1.75861	1.45889
H	-3.46060	1.67823	0.29567
H	-2.70463	0.75509	-1.04584
C	2.97724	0.16915	1.34238
H	2.81329	-0.50829	2.19791
H	2.88680	1.20737	1.70499
H	4.01033	0.01936	0.96032
Li	-0.07701	0.00540	1.05587

ACE2_Li_SCS-MP2_SVP

33

O	-0.32489	-1.68560	0.06927
O	-0.24113	1.36604	-0.40943
O	-2.17685	0.14446	0.88314
N	1.96785	-0.07942	0.31206
C	2.03074	-1.45787	-0.18948
C	2.02812	0.93207	-0.75765
C	0.73385	-1.87875	-0.86215
C	1.00859	2.03375	-0.51407
C	-1.63025	-1.78271	-0.48526
C	-1.38062	2.15468	-0.10563
C	-2.59003	-1.17269	0.52248
C	-2.53297	1.17234	-0.04211
H	2.19972	-2.11802	0.67373
H	2.87140	-1.61201	-0.89368
H	1.78869	0.46293	-1.72139
H	3.04065	1.36280	-0.85125
H	0.53764	-1.28164	-1.76814
H	0.79679	-2.93801	-1.16328
H	1.22487	2.58542	0.41817

H	1.00134	2.75313	-1.35011
H	-1.90226	-2.83401	-0.68004
H	-1.66660	-1.23622	-1.44240
H	-1.56607	2.91336	-0.88383
H	-1.24543	2.66843	0.86263
H	-3.61642	-1.17354	0.12337
H	-2.57731	-1.76004	1.45036
H	-3.45859	1.67474	0.28100
H	-2.69673	0.74847	-1.04417
C	2.98562	0.16829	1.33428
H	2.83133	-0.50892	2.18480
H	2.89769	1.20030	1.69892
H	4.01097	0.02256	0.94599
Li	-0.07403	0.00411	1.07622

ACE2_Li_SCS-MP2_SV

33

O	-0.32572	-1.68990	0.06852
O	-0.24063	1.37074	-0.41745
O	-2.17068	0.14492	0.88143
N	1.96506	-0.08009	0.31194
C	2.03150	-1.46044	-0.18803
C	2.03120	0.93224	-0.75865
C	0.73467	-1.88575	-0.86285
C	1.01209	2.03707	-0.51543
C	-1.63288	-1.78775	-0.48554
C	-1.37869	2.16020	-0.10406
C	-2.59018	-1.17315	0.52466
C	-2.53292	1.17695	-0.03959
H	2.19985	-2.12270	0.67940
H	2.87869	-1.61461	-0.89218

H	1.79356	0.46312	-1.72782
H	3.05025	1.36110	-0.84912
H	0.53694	-1.29130	-1.77540
H	0.79829	-2.95158	-1.15798
H	1.22600	2.58787	0.42306
H	1.00722	2.76109	-1.35383
H	-1.90452	-2.84489	-0.67666
H	-1.67002	-1.24131	-1.44764
H	-1.56581	2.92568	-0.88211
H	-1.23699	2.67096	0.87001
H	-3.62330	-1.16930	0.12973
H	-2.57569	-1.75861	1.45889
H	-3.46060	1.67823	0.29567
H	-2.70463	0.75509	-1.04584
C	2.97724	0.16915	1.34238
H	2.81329	-0.50829	2.19791
H	2.88680	1.20737	1.70499
H	4.01033	0.01936	0.96032
Li	-0.07701	0.00540	1.05587

ACE2_Li_SCS-MP2_TZVP

33

O	-0.31250	-1.69535	0.08367
O	-0.26018	1.33432	-0.43610
O	-2.20202	0.13343	0.90743
N	1.96419	-0.06524	0.32419
C	2.03955	-1.44033	-0.18863
C	2.02107	0.94463	-0.75094
C	0.74617	-1.85480	-0.86386
C	0.98548	2.02637	-0.50989
C	-1.61195	-1.75538	-0.50348

C	-1.39310	2.13075	-0.10459
C	-2.58673	-1.18622	0.50747
C	-2.54934	1.15945	-0.03211
H	2.21611	-2.10103	0.66338
H	2.87332	-1.57517	-0.89313
H	1.80231	0.47286	-1.70995
H	3.02122	1.38681	-0.82616
H	0.53517	-1.23659	-1.74196
H	0.81018	-2.89908	-1.18626
H	1.17180	2.55925	0.43027
H	0.97447	2.75107	-1.33043
H	-1.88035	-2.78994	-0.74290
H	-1.61808	-1.17105	-1.42904
H	-1.57987	2.89069	-0.86995
H	-1.23420	2.62721	0.85992
H	-3.60109	-1.19013	0.09857
H	-2.57359	-1.78666	1.41770
H	-3.46167	1.66501	0.29491
H	-2.72098	0.72684	-1.02017
C	3.01944	0.17740	1.31309
H	2.89312	-0.50043	2.15790
H	2.94453	1.20213	1.67918
H	4.02141	0.03178	0.88672
Li	-0.05632	0.00093	1.11658

ACE2_Li_SCS-MP2_TZV

33

O	-0.34183	-1.74193	0.07955
O	-0.22758	1.37430	-0.41017
O	-2.20325	0.16457	0.91048
N	1.98445	-0.09055	0.32518

C	2.04976	-1.48194	-0.22581
C	2.08952	0.95444	-0.74935
C	0.74042	-1.87536	-0.91702
C	1.06597	2.07116	-0.51257
C	-1.69726	-1.80815	-0.49692
C	-1.40209	2.20735	-0.11151
C	-2.64700	-1.19773	0.53631
C	-2.56880	1.22440	-0.06008
H	2.23076	-2.16245	0.61241
H	2.87950	-1.59597	-0.94437
H	1.88607	0.50091	-1.72445
H	3.10415	1.37927	-0.78413
H	0.51545	-1.21729	-1.76456
H	0.79251	-2.91165	-1.26954
H	1.26109	2.61208	0.42235
H	1.04835	2.77742	-1.35028
H	-1.97257	-2.84915	-0.70016
H	-1.71016	-1.23922	-1.43399
H	-1.55947	2.94973	-0.90156
H	-1.25996	2.71282	0.85137
H	-3.66899	-1.16732	0.14337
H	-2.63071	-1.76700	1.46674
H	-3.48531	1.71828	0.27534
H	-2.73007	0.78609	-1.05029
C	3.02530	0.11628	1.37886
H	2.83684	-0.56651	2.21110
H	2.96099	1.14371	1.74681
H	4.04027	-0.05988	0.98799
Li	-0.06116	-0.01610	1.07980

ACE2_Na_B2PLYP_SV(P)

33

O	-0.32402	-1.71116	-0.03483
O	-0.27322	1.34693	-0.52558
O	-2.31935	0.13316	0.76589
N	2.07726	-0.05902	0.18968
C	2.05542	-1.43777	-0.30663
C	2.02706	0.96295	-0.86816
C	0.74201	-1.84188	-0.96091
C	0.96807	2.02225	-0.59609
C	-1.61413	-1.76584	-0.61704
C	-1.41518	2.12138	-0.22479
C	-2.62792	-1.19663	0.36374
C	-2.59017	1.16152	-0.18260
H	2.23238	-2.10342	0.55404
H	2.87208	-1.63151	-1.03629
H	1.78870	0.49146	-1.83362
H	3.01070	1.45371	-0.99393
H	0.53319	-1.21777	-1.84810
H	0.82604	-2.89017	-1.30606
H	1.17187	2.55423	0.35477
H	0.96184	2.77204	-1.41076
H	-1.89410	-2.80866	-0.86318
H	-1.61992	-1.18616	-1.55779
H	-1.59442	2.89688	-0.99408
H	-1.29272	2.63146	0.75151
H	-3.63882	-1.25166	-0.07875
H	-2.62795	-1.79375	1.28860
H	-3.50999	1.70232	0.10317
H	-2.74166	0.73181	-1.18611
C	3.19134	0.16639	1.10621

H	3.12455	-0.52850	1.95874
H	3.14572	1.19349	1.50180
H	4.17990	0.02745	0.61714
Na	-0.05041	0.00583	1.52044

ACE2_Na_B2PLYP_SVP

33

O	-0.32283	-1.71270	-0.03387
O	-0.27470	1.34393	-0.52451
O	-2.32245	0.13163	0.76804
N	2.07834	-0.05765	0.19059
C	2.05502	-1.43514	-0.30818
C	2.02495	0.96393	-0.86669
C	0.74266	-1.83489	-0.96227
C	0.96601	2.02085	-0.59517
C	-1.61219	-1.76101	-0.61891
C	-1.41747	2.11794	-0.22480
C	-2.62688	-1.19772	0.36107
C	-2.59076	1.15918	-0.18221
H	2.23268	-2.10154	0.54871
H	2.86899	-1.62888	-1.03666
H	1.78569	0.49308	-1.82966
H	3.00485	1.45527	-0.99569
H	0.53280	-1.20427	-1.84188
H	0.82716	-2.87663	-1.31879
H	1.16922	2.55284	0.35318
H	0.96076	2.77049	-1.40637
H	-1.89472	-2.79812	-0.87440
H	-1.61486	-1.17688	-1.55433
H	-1.59819	2.89222	-0.99112
H	-1.29662	2.62906	0.74854

H	-3.63415	-1.25627	-0.08275
H	-2.62809	-1.79859	1.28085
H	-3.50918	1.70053	0.09707
H	-2.73980	0.72953	-1.18368
C	3.19651	0.16528	1.10181
H	3.13719	-0.53194	1.94861
H	3.15352	1.18681	1.50271
H	4.17985	0.03088	0.60911
Na	-0.05027	0.00388	1.52611

ACE2_Na_B2PLYP_SV

33

O	-0.32402	-1.71116	-0.03483
O	-0.27322	1.34693	-0.52558
O	-2.31935	0.13316	0.76589
N	2.07726	-0.05902	0.18968
C	2.05542	-1.43777	-0.30663
C	2.02706	0.96295	-0.86816
C	0.74201	-1.84188	-0.96091
C	0.96807	2.02225	-0.59609
C	-1.61413	-1.76584	-0.61704
C	-1.41518	2.12138	-0.22479
C	-2.62792	-1.19663	0.36374
C	-2.59017	1.16152	-0.18260
H	2.23238	-2.10342	0.55404
H	2.87208	-1.63151	-1.03629
H	1.78870	0.49146	-1.83362
H	3.01070	1.45371	-0.99393
H	0.53319	-1.21777	-1.84810
H	0.82604	-2.89017	-1.30606
H	1.17187	2.55423	0.35477

H	0.96184	2.77204	-1.41076
H	-1.89410	-2.80866	-0.86318
H	-1.61992	-1.18616	-1.55779
H	-1.59442	2.89688	-0.99408
H	-1.29272	2.63146	0.75151
H	-3.63882	-1.25166	-0.07875
H	-2.62795	-1.79375	1.28860
H	-3.50999	1.70232	0.10317
H	-2.74166	0.73181	-1.18611
C	3.19134	0.16639	1.10621
H	3.12455	-0.52850	1.95874
H	3.14572	1.19349	1.50180
H	4.17990	0.02745	0.61714
Na	-0.05041	0.00583	1.52044

ACE2_Na_B2PLYP_TZVP

33

O	-0.31876	1.72616	0.02924
O	-0.28540	-1.32472	0.55635
O	-2.34149	-0.12616	-0.78319
N	2.07442	0.04976	-0.19989
C	2.05584	1.42426	0.31208
C	2.02530	-0.97491	0.85802
C	0.74760	1.81384	0.97150
C	0.95624	-2.01664	0.58916
C	-1.60651	1.73879	0.63524
C	-1.42269	-2.10381	0.22281
C	-2.62619	1.20545	-0.34954
C	-2.59964	-1.15587	0.17722
H	2.23437	2.09121	-0.53304
H	2.86355	1.60167	1.03826

H	1.80829	-0.50806	1.81847
H	2.99432	-1.47277	0.96264
H	0.52646	1.16188	1.82011
H	0.83161	2.83768	1.34958
H	1.13027	-2.52480	-0.36699
H	0.95500	-2.77274	1.38128
H	-1.88755	2.75744	0.92390
H	-1.58671	1.12554	1.54003
H	-1.60544	-2.88251	0.97061
H	-1.27831	-2.59151	-0.74840
H	-3.62198	1.26202	0.09799
H	-2.62416	1.81230	-1.25452
H	-3.50394	-1.69818	-0.10778
H	-2.75559	-0.72305	1.16593
C	3.21931	-0.15943	-1.08451
H	3.17539	0.53988	-1.91861
H	3.19176	-1.17109	-1.48720
H	4.17791	-0.02195	-0.56483
Na	-0.04332	-0.00032	-1.55448

ACE2_Na_B2PLYP_TZV

33

O	-0.34323	-1.77743	-0.02841
O	-0.26908	1.35328	-0.55508
O	-2.34467	0.14845	0.80950
N	2.09645	-0.06567	0.21030
C	2.06099	-1.45162	-0.33030
C	2.07472	0.98620	-0.84842
C	0.74685	-1.81425	-1.01081
C	1.01816	2.05158	-0.57788
C	-1.67604	-1.76477	-0.63609

C	-1.43735	2.16072	-0.21086
C	-2.66973	-1.21996	0.37816
C	-2.61808	1.20772	-0.17753
H	2.22747	-2.13403	0.50399
H	2.87047	-1.61816	-1.05788
H	1.86185	0.53451	-1.81734
H	3.05451	1.46978	-0.92965
H	0.51170	-1.11689	-1.81662
H	0.82401	-2.81958	-1.43166
H	1.18553	2.54315	0.38564
H	1.02236	2.80677	-1.36787
H	-1.96796	-2.77823	-0.92268
H	-1.65094	-1.14171	-1.53233
H	-1.60705	2.93735	-0.96059
H	-1.28773	2.63545	0.76314
H	-3.67602	-1.25171	-0.04427
H	-2.65076	-1.81140	1.29043
H	-3.52363	1.73790	0.11797
H	-2.76910	0.77067	-1.16456
C	3.23983	0.11738	1.13865
H	3.15970	-0.59109	1.96174
H	3.21579	1.12553	1.54947
H	4.20637	-0.03180	0.63696
Na	-0.04199	-0.02175	1.47331

ACE2_Na_B3LYP_SV(P)

33

O	-0.32243	-1.72070	-0.03625
O	-0.27598	1.36495	-0.51652
O	-2.33092	0.13143	0.76151
N	2.09063	-0.05711	0.18408

C	2.06563	-1.44190	-0.30312
C	2.02762	0.96784	-0.87367
C	0.75055	-1.86159	-0.95451
C	0.96885	2.03327	-0.59781
C	-1.61605	-1.78292	-0.61230
C	-1.42491	2.13242	-0.22302
C	-2.63363	-1.20227	0.36471
C	-2.60016	1.16551	-0.18332
H	2.24829	-2.10045	0.56353
H	2.88136	-1.64375	-1.03389
H	1.78268	0.49424	-1.83794
H	3.00994	1.46134	-1.00952
H	0.54263	-1.25509	-1.85637
H	0.84195	-2.91652	-1.28224
H	1.18024	2.57175	0.34973
H	0.96252	2.78007	-1.41735
H	-1.89735	-2.83048	-0.84417
H	-1.62600	-1.21690	-1.56335
H	-1.60702	2.90610	-0.99589
H	-1.30979	2.64874	0.75310
H	-3.64444	-1.26474	-0.08084
H	-2.63681	-1.79361	1.29546
H	-3.52090	1.70677	0.10515
H	-2.75429	0.73968	-1.18998
C	3.19709	0.17716	1.10973
H	3.13177	-0.51905	1.96354
H	3.14229	1.20452	1.50819
H	4.19331	0.04709	0.62902
Na	-0.04819	0.00451	1.51138

ACE2_Na_B3LYP_SVP

33

O	-0.32166	-1.72397	-0.03439
O	-0.27703	1.36316	-0.51617
O	-2.33255	0.13049	0.76395
N	2.09076	-0.05609	0.18482
C	2.06486	-1.44037	-0.30402
C	2.02578	0.96837	-0.87297
C	0.75117	-1.85609	-0.95577
C	0.96830	2.03256	-0.59693
C	-1.61520	-1.77915	-0.61353
C	-1.42685	2.13046	-0.22199
C	-2.63268	-1.20339	0.36316
C	-2.60024	1.16442	-0.18274
H	2.24779	-2.09990	0.55965
H	2.87936	-1.64336	-1.03277
H	1.77894	0.49576	-1.83542
H	3.00572	1.46113	-1.01316
H	0.54183	-1.24298	-1.85095
H	0.84314	-2.90528	-1.29537
H	1.17977	2.57097	0.34874
H	0.96349	2.78058	-1.41278
H	-1.89977	-2.82176	-0.85466
H	-1.62202	-1.20917	-1.56044
H	-1.61070	2.90471	-0.99114
H	-1.31304	2.64726	0.75215
H	-3.64132	-1.26912	-0.08228
H	-2.63709	-1.79842	1.28976
H	-3.52070	1.70555	0.10002
H	-2.75242	0.73915	-1.18821
C	3.20015	0.17617	1.10756

H	3.14134	-0.52314	1.95583
H	3.14679	1.19830	1.51217
H	4.19291	0.05134	0.62503
Na	-0.04738	0.00324	1.51240

ACE2_Na_B3LYP_SV

33

O	-0.32243	-1.72070	-0.03625
O	-0.27598	1.36495	-0.51652
O	-2.33092	0.13143	0.76151
N	2.09063	-0.05711	0.18408
C	2.06563	-1.44190	-0.30312
C	2.02762	0.96784	-0.87367
C	0.75055	-1.86159	-0.95451
C	0.96885	2.03327	-0.59781
C	-1.61605	-1.78292	-0.61230
C	-1.42491	2.13242	-0.22302
C	-2.63363	-1.20227	0.36471
C	-2.60016	1.16551	-0.18332
H	2.24829	-2.10045	0.56353
H	2.88136	-1.64375	-1.03389
H	1.78268	0.49424	-1.83794
H	3.00994	1.46134	-1.00952
H	0.54263	-1.25509	-1.85637
H	0.84195	-2.91652	-1.28224
H	1.18024	2.57175	0.34973
H	0.96252	2.78007	-1.41735
H	-1.89735	-2.83048	-0.84417
H	-1.62600	-1.21690	-1.56335
H	-1.60702	2.90610	-0.99589
H	-1.30979	2.64874	0.75310

H	-3.64444	-1.26474	-0.08084
H	-2.63681	-1.79361	1.29546
H	-3.52090	1.70677	0.10515
H	-2.75429	0.73968	-1.18998
C	3.19709	0.17716	1.10973
H	3.13177	-0.51905	1.96354
H	3.14229	1.20452	1.50819
H	4.19331	0.04709	0.62902
Na	-0.04819	0.00451	1.51138

ACE2_Na_B3LYP_TZVPP

33

O	-0.32292	1.73885	0.03564
O	-0.28467	-1.35342	0.55980
O	-2.34843	-0.12843	-0.77548
N	2.08918	0.05264	-0.19227
C	2.06050	1.43007	0.31907
C	2.03059	-0.98189	0.85878
C	0.74818	1.82781	0.97447
C	0.96486	-2.03149	0.58310
C	-1.61691	1.75915	0.63077
C	-1.42768	-2.12105	0.21943
C	-2.63541	1.20666	-0.35073
C	-2.60399	-1.16613	0.17935
H	2.24432	2.09578	-0.52591
H	2.86384	1.61383	1.04887
H	1.80691	-0.52070	1.82053
H	2.99953	-1.47883	0.97013
H	0.52625	1.18444	1.82992
H	0.83805	2.85401	1.34589
H	1.14058	-2.53275	-0.37681

H	0.97608	-2.79400	1.36965
H	-1.90275	2.78245	0.89924
H	-1.60416	1.16473	1.54859
H	-1.61791	-2.90474	0.96102
H	-1.28670	-2.60521	-0.75464
H	-3.63159	1.26592	0.09642
H	-2.63859	1.80514	-1.26190
H	-3.50960	-1.70646	-0.10756
H	-2.76045	-0.74009	1.17132
C	3.22689	-0.15518	-1.08849
H	3.18138	0.55115	-1.91717
H	3.19234	-1.16271	-1.50183
H	4.19263	-0.02792	-0.57793
Na	-0.04259	0.01127	-1.56420

ACE2_Na_B3LYP_TZVP

33

O	-0.32329	-1.73853	-0.03529
O	-0.28411	1.35327	-0.55848
O	-2.34815	0.12895	0.77530
N	2.08931	-0.05315	0.19190
C	2.06007	-1.43046	-0.31971
C	2.03103	0.98179	-0.85879
C	0.74746	-1.82794	-0.97460
C	0.96545	2.03154	-0.58319
C	-1.61766	-1.75992	-0.62983
C	-1.42747	2.12141	-0.21968
C	-2.63582	-1.20636	0.35143
C	-2.60373	1.16644	-0.17988
H	2.24417	-2.09686	0.52570
H	2.86353	-1.61453	-1.05063

H	1.80729	0.52063	-1.82144
H	3.00070	1.47928	-0.96974
H	0.52544	-1.18436	-1.83089
H	0.83727	-2.85516	-1.34590
H	1.14206	2.53439	0.37670
H	0.97640	2.79364	-1.37139
H	-1.90376	-2.78458	-0.89666
H	-1.60576	-1.16637	-1.54919
H	-1.61755	2.90487	-0.96286
H	-1.28741	2.60688	0.75486
H	-3.63272	-1.26571	-0.09627
H	-2.63898	-1.80462	1.26376
H	-3.50998	1.70748	0.10718
H	-2.76000	0.74020	-1.17270
C	3.22634	0.15422	1.08913
H	3.17945	-0.55225	1.91865
H	3.19197	1.16264	1.50237
H	4.19326	0.02594	0.57926
Na	-0.04182	-0.01030	1.56312

ACE2_Na_B3LYP_TZV

33

O	-0.34004	-1.77951	-0.02509
O	-0.27295	1.37456	-0.55296
O	-2.34722	0.14453	0.80167
N	2.10337	-0.06407	0.20255
C	2.06783	-1.45627	-0.31880
C	2.06484	0.98490	-0.85677
C	0.75668	-1.84170	-0.99430
C	1.01745	2.05895	-0.57981
C	-1.67136	-1.78147	-0.62849

C	-1.44409	2.16870	-0.20458
C	-2.66805	-1.22310	0.37576
C	-2.61944	1.20836	-0.17734
H	2.23795	-2.12587	0.52517
H	2.87805	-1.63725	-1.04218
H	1.83385	0.52912	-1.81923
H	3.04440	1.46424	-0.96147
H	0.52710	-1.17230	-1.82556
H	0.84200	-2.85861	-1.38557
H	1.19445	2.55375	0.38076
H	1.02882	2.81442	-1.37014
H	-1.96244	-2.80104	-0.89583
H	-1.65312	-1.17878	-1.53915
H	-1.62239	2.95048	-0.94784
H	-1.30014	2.64205	0.77171
H	-3.67315	-1.26307	-0.04954
H	-2.65488	-1.80987	1.29191
H	-3.52800	1.73467	0.11802
H	-2.76869	0.77824	-1.16785
C	3.23309	0.13739	1.13903
H	3.16150	-0.57262	1.96217
H	3.19140	1.14347	1.55445
H	4.20895	0.00677	0.64896
Na	-0.04083	-0.01824	1.45602

ACE2_Na_B97-1_SV(P)

33

O	-0.31985	-1.71950	-0.03782
O	-0.28008	1.35822	-0.52729
O	-2.33525	0.12946	0.76330
N	2.09000	-0.05516	0.18325

C	2.06797	-1.43997	-0.30563
C	2.02735	0.96969	-0.87549
C	0.74915	-1.85793	-0.95728
C	0.96059	2.03088	-0.59944
C	-1.60995	-1.77773	-0.61656
C	-1.42447	2.12606	-0.22680
C	-2.63192	-1.20197	0.36389
C	-2.60400	1.15974	-0.18194
H	2.25090	-2.10020	0.56200
H	2.88429	-1.64049	-1.03856
H	1.78669	0.49517	-1.84259
H	3.00936	1.46845	-1.00732
H	0.54124	-1.24637	-1.85837
H	0.84042	-2.91328	-1.28967
H	1.16743	2.56632	0.35324
H	0.95594	2.78298	-1.41658
H	-1.89210	-2.82500	-0.85655
H	-1.61910	-1.20335	-1.56511
H	-1.61038	2.90263	-0.99838
H	-1.30412	2.64140	0.75133
H	-3.64373	-1.26846	-0.08333
H	-2.63264	-1.79737	1.29401
H	-3.52375	1.70512	0.10788
H	-2.76144	0.73272	-1.18972
C	3.19902	0.18027	1.10669
H	3.13559	-0.51811	1.96093
H	3.14183	1.20889	1.50619
H	4.19564	0.05213	0.62286
Na	-0.04300	0.00575	1.53407

ACE2_Na_B97-1_SVP

33

O	-0.31869	-1.72302	-0.03581
O	-0.28167	1.35665	-0.52659
O	-2.33736	0.12790	0.76557
N	2.09063	-0.05364	0.18391
C	2.06804	-1.43850	-0.30517
C	2.02477	0.96972	-0.87580
C	0.75083	-1.85338	-0.95741
C	0.95955	2.03011	-0.59952
C	-1.60845	-1.77398	-0.61820
C	-1.42712	2.12412	-0.22538
C	-2.63107	-1.20361	0.36162
C	-2.60472	1.15834	-0.18107
H	2.25121	-2.09856	0.56004
H	2.88324	-1.64085	-1.03539
H	1.78119	0.49504	-1.84002
H	3.00407	1.46738	-1.01367
H	0.54201	-1.23639	-1.85248
H	0.84302	-2.90296	-1.30068
H	1.16705	2.56621	0.35049
H	0.95600	2.78266	-1.41329
H	-1.89365	-2.81582	-0.86827
H	-1.61394	-1.19527	-1.56212
H	-1.61490	2.90106	-0.99301
H	-1.30802	2.64001	0.75047
H	-3.64017	-1.27343	-0.08598
H	-2.63351	-1.80283	1.28719
H	-3.52411	1.70323	0.10301
H	-2.76027	0.73217	-1.18757
C	3.20268	0.18133	1.10401

H	3.14607	-0.51878	1.95351
H	3.14685	1.20501	1.50819
H	4.19561	0.05802	0.61863
Na	-0.04250	0.00477	1.53543

ACE2_Na_B97-1_SV

33

O	-0.31985	-1.71950	-0.03782
O	-0.28008	1.35822	-0.52729
O	-2.33525	0.12946	0.76330
N	2.09000	-0.05516	0.18325
C	2.06797	-1.43997	-0.30563
C	2.02735	0.96969	-0.87549
C	0.74915	-1.85793	-0.95728
C	0.96059	2.03088	-0.59944
C	-1.60995	-1.77773	-0.61656
C	-1.42447	2.12606	-0.22680
C	-2.63192	-1.20197	0.36389
C	-2.60400	1.15974	-0.18194
H	2.25090	-2.10020	0.56200
H	2.88429	-1.64049	-1.03856
H	1.78669	0.49517	-1.84259
H	3.00936	1.46845	-1.00732
H	0.54124	-1.24637	-1.85837
H	0.84042	-2.91328	-1.28967
H	1.16743	2.56632	0.35324
H	0.95594	2.78298	-1.41658
H	-1.89210	-2.82500	-0.85655
H	-1.61910	-1.20335	-1.56511
H	-1.61038	2.90263	-0.99838
H	-1.30412	2.64140	0.75133

H	-3.64373	-1.26846	-0.08333
H	-2.63264	-1.79737	1.29401
H	-3.52375	1.70512	0.10788
H	-2.76144	0.73272	-1.18972
C	3.19902	0.18027	1.10669
H	3.13559	-0.51811	1.96093
H	3.14183	1.20889	1.50619
H	4.19564	0.05213	0.62286
Na	-0.04300	0.00575	1.53407

ACE2_Na_B97-1_TZVPP

33

O	-0.31793	-1.73764	-0.03688
O	-0.29243	1.34368	-0.57065
O	-2.35666	0.12206	0.77775
N	2.08818	-0.04580	0.19381
C	2.06701	-1.42547	-0.31414
C	2.02653	0.98210	-0.86481
C	0.75247	-1.82700	-0.97214
C	0.95108	2.02767	-0.59137
C	-1.60434	-1.74896	-0.64243
C	-1.42881	2.11226	-0.22041
C	-2.63413	-1.20794	0.34065
C	-2.61164	1.15872	-0.17405
H	2.25071	-2.08954	0.53538
H	2.87321	-1.60891	-1.04423
H	1.80501	0.51370	-1.82649
H	2.99440	1.48565	-0.97725
H	0.53314	-1.18292	-1.83144
H	0.84560	-2.85556	-1.34355
H	1.12488	2.53001	0.37149

H	0.96081	2.79251	-1.37914
H	-1.89056	-2.76990	-0.92876
H	-1.58498	-1.13845	-1.55308
H	-1.62379	2.90072	-0.95935
H	-1.28042	2.59400	0.75671
H	-3.62878	-1.26938	-0.11550
H	-2.63948	-1.81740	1.24739
H	-3.51484	1.70532	0.11734
H	-2.77465	0.73433	-1.16859
C	3.23505	0.16917	1.07842
H	3.19816	-0.53514	1.91220
H	3.20049	1.18096	1.48736
H	4.19765	0.04147	0.55764
Na	-0.04063	-0.01445	1.59865

ACE2_Na_B97-1_TZVP

33

O	-0.31856	-1.73739	-0.03659
O	-0.29154	1.34374	-0.56953
O	-2.35638	0.12283	0.77747
N	2.08825	-0.04648	0.19345
C	2.06636	-1.42603	-0.31482
C	2.02721	0.98190	-0.86475
C	0.75156	-1.82726	-0.97233
C	0.95209	2.02785	-0.59149
C	-1.60540	-1.74964	-0.64149
C	-1.42819	2.11277	-0.22039
C	-2.63472	-1.20744	0.34136
C	-2.61102	1.15926	-0.17484
H	2.25030	-2.09081	0.53511
H	2.87274	-1.60996	-1.04585

H	1.80557	0.51372	-1.82736
H	2.99593	1.48569	-0.97686
H	0.53211	-1.18313	-1.83252
H	0.84459	-2.85682	-1.34365
H	1.12671	2.53175	0.37135
H	0.96172	2.79236	-1.38081
H	-1.89211	-2.77191	-0.92619
H	-1.58685	-1.14022	-1.55383
H	-1.62297	2.90153	-0.96039
H	-1.28064	2.59538	0.75739
H	-3.63015	-1.26893	-0.11513
H	-2.64009	-1.81661	1.24929
H	-3.51507	1.70644	0.11613
H	-2.77326	0.73469	-1.17031
C	3.23439	0.16774	1.07919
H	3.19585	-0.53649	1.91395
H	3.20038	1.18044	1.48792
H	4.19824	0.03863	0.55937
Na	-0.04023	-0.01351	1.59771

ACE2_Na_B97-1_TZV

33

O	-0.33494	-1.77659	-0.02648
O	-0.27957	1.36514	-0.56365
O	-2.35582	0.14046	0.80592
N	2.10298	-0.05982	0.20464
C	2.07297	-1.45145	-0.32023
C	2.06341	0.98786	-0.85710
C	0.75758	-1.83703	-0.99613
C	1.00480	2.05640	-0.58149
C	-1.66164	-1.77569	-0.63369

C	-1.44632	2.15955	-0.20990
C	-2.66575	-1.22473	0.37393
C	-2.62664	1.19878	-0.17524
H	2.24524	-2.12410	0.52436
H	2.88392	-1.62859	-1.04755
H	1.83772	0.52958	-1.82283
H	3.04205	1.47552	-0.95753
H	0.52845	-1.16364	-1.82837
H	0.84463	-2.85529	-1.39116
H	1.17675	2.54891	0.38463
H	1.01700	2.81717	-1.37042
H	-1.95336	-2.79555	-0.90997
H	-1.64289	-1.16406	-1.54204
H	-1.62957	2.94308	-0.95396
H	-1.29796	2.63432	0.76819
H	-3.67152	-1.27108	-0.05603
H	-2.64998	-1.81839	1.28870
H	-3.53443	1.73153	0.12030
H	-2.77946	0.76597	-1.16716
C	3.24086	0.14259	1.13229
H	3.17566	-0.56954	1.95736
H	3.20022	1.15112	1.54846
H	4.21477	0.01279	0.63291
Na	-0.03646	-0.01552	1.47898

ACE2_Na_CAM-B3LYP_SV(P)

33

O	-0.32241	-1.70640	-0.03373
O	-0.27297	1.34983	-0.51435
O	-2.30748	0.12916	0.76128
N	2.07425	-0.05769	0.18789

C	2.05401	-1.43776	-0.29216
C	2.01890	0.95530	-0.87154
C	0.74569	-1.85221	-0.94538
C	0.96421	2.01713	-0.60395
C	-1.60716	-1.76671	-0.61174
C	-1.41234	2.11885	-0.21988
C	-2.62031	-1.19453	0.36449
C	-2.58483	1.15915	-0.17367
H	2.23004	-2.09406	0.57568
H	2.87333	-1.63836	-1.01596
H	1.77550	0.47716	-1.83222
H	3.00215	1.44354	-1.00609
H	0.54213	-1.24375	-1.84508
H	0.83192	-2.90568	-1.27310
H	1.17634	2.56113	0.33841
H	0.95273	2.75719	-1.42704
H	-1.88538	-2.81089	-0.85309
H	-1.61386	-1.19172	-1.55585
H	-1.59238	2.89120	-0.99183
H	-1.29073	2.63316	0.75454
H	-3.63061	-1.24699	-0.07917
H	-2.62490	-1.79219	1.28927
H	-3.50268	1.69864	0.12049
H	-2.74379	0.73440	-1.17854
C	3.17312	0.18046	1.11211
H	3.10450	-0.50850	1.96958
H	3.11949	1.20953	1.50217
H	4.16732	0.04478	0.63367
Na	-0.05233	0.01150	1.48215

ACE2_Na_CAM-B3LYP_SVP

33

O	-0.32151	-1.70994	-0.03167
O	-0.27430	1.34792	-0.51431
O	-2.30961	0.12804	0.76372
N	2.07477	-0.05645	0.18861
C	2.05356	-1.43604	-0.29274
C	2.01703	0.95587	-0.87088
C	0.74664	-1.84710	-0.94595
C	0.96325	2.01613	-0.60342
C	-1.60600	-1.76300	-0.61277
C	-1.41440	2.11655	-0.21875
C	-2.61941	-1.19566	0.36263
C	-2.58524	1.15803	-0.17269
H	2.23050	-2.09325	0.57237
H	2.87164	-1.63771	-1.01498
H	1.77234	0.47829	-1.82997
H	2.99792	1.44398	-1.00954
H	0.54148	-1.23260	-1.83961
H	0.83374	-2.89527	-1.28501
H	1.17517	2.56043	0.33727
H	0.95321	2.75749	-1.42315
H	-1.88754	-2.80239	-0.86352
H	-1.60920	-1.18393	-1.55290
H	-1.59638	2.88982	-0.98697
H	-1.29344	2.63136	0.75396
H	-3.62757	-1.25141	-0.08154
H	-2.62569	-1.79699	1.28352
H	-3.50275	1.69780	0.11629
H	-2.74299	0.73377	-1.17652
C	3.17631	0.18008	1.10997

H	3.11386	-0.51137	1.96276
H	3.12416	1.20455	1.50544
H	4.16740	0.04906	0.62989
Na	-0.05148	0.01057	1.48251

ACE2_Na_CAM-B3LYP_SV

33

O	-0.32208	-1.70689	-0.03380
O	-0.27330	1.35030	-0.51413
O	-2.30748	0.12897	0.76123
N	2.07397	-0.05725	0.18778
C	2.05430	-1.43755	-0.29169
C	2.01828	0.95536	-0.87214
C	0.74631	-1.85282	-0.94510
C	0.96397	2.01743	-0.60403
C	-1.60672	-1.76664	-0.61214
C	-1.41273	2.11908	-0.21956
C	-2.61998	-1.19473	0.36409
C	-2.58506	1.15909	-0.17354
H	2.23023	-2.09341	0.57650
H	2.87394	-1.63820	-1.01510
H	1.77427	0.47678	-1.83244
H	3.00155	1.44335	-1.00735
H	0.54279	-1.24499	-1.84524
H	0.83308	-2.90642	-1.27223
H	1.17672	2.56099	0.33845
H	0.95223	2.75790	-1.42672
H	-1.88506	-2.81065	-0.85405
H	-1.61320	-1.19114	-1.55596
H	-1.59295	2.89151	-0.99136
H	-1.29126	2.63321	0.75499

H	-3.63022	-1.24724	-0.07970
H	-2.62461	-1.79256	1.28876
H	-3.50296	1.69834	0.12086
H	-2.74414	0.73442	-1.17843
C	3.17281	0.18163	1.11183
H	3.10443	-0.50688	1.96968
H	3.11895	1.21089	1.50138
H	4.16702	0.04598	0.63342
Na	-0.05208	0.01042	1.48249

ACE2_Na_CAM-B3LYP_TZVPP

33

O	-0.32029	-1.72592	-0.03225
O	-0.28286	1.33633	-0.55568
O	-2.32558	0.12535	0.77660
N	2.07142	-0.05224	0.19575
C	2.05028	-1.42537	-0.30485
C	2.02009	0.96829	-0.85774
C	0.74617	-1.82133	-0.96250
C	0.95787	2.01395	-0.59181
C	-1.60465	-1.74267	-0.62808
C	-1.41639	2.10504	-0.21772
C	-2.61845	-1.20029	0.35350
C	-2.58940	1.15672	-0.16926
H	2.22760	-2.08725	0.54330
H	2.85880	-1.60877	-1.02668
H	1.79870	0.50118	-1.81590
H	2.98977	1.46147	-0.96853
H	0.52893	-1.17882	-1.81867
H	0.83295	-2.84738	-1.33156
H	1.13614	2.52473	0.36152

H	0.96223	2.76801	-1.38476
H	-1.88836	-2.76208	-0.90796
H	-1.58914	-1.13765	-1.53796
H	-1.60492	2.88586	-0.96075
H	-1.27023	2.59041	0.75387
H	-3.61501	-1.25374	-0.09066
H	-2.61964	-1.80484	1.25940
H	-3.49250	1.69626	0.12237
H	-2.75101	0.73045	-1.15942
C	3.19640	0.16386	1.09469
H	3.14557	-0.53264	1.93003
H	3.16039	1.17511	1.49628
H	4.16319	0.03046	0.59090
Na	-0.04501	0.00178	1.52438

ACE2_Na_CAM-B3LYP_TZVP

33

O	-0.32057	-1.72542	-0.03209
O	-0.28236	1.33622	-0.55443
O	-2.32549	0.12577	0.77633
N	2.07165	-0.05261	0.19535
C	2.05002	-1.42560	-0.30562
C	2.02052	0.96842	-0.85768
C	0.74563	-1.82147	-0.96270
C	0.95833	2.01406	-0.59170
C	-1.60524	-1.74326	-0.62743
C	-1.41622	2.10537	-0.21806
C	-2.61886	-1.20006	0.35386
C	-2.58923	1.15708	-0.16978
H	2.22775	-2.08828	0.54286
H	2.85869	-1.60913	-1.02858

H	1.79926	0.50148	-1.81685
H	2.99089	1.46225	-0.96800
H	0.52820	-1.17914	-1.82000
H	0.83237	-2.84867	-1.33135
H	1.13732	2.52622	0.36176
H	0.96239	2.76795	-1.38605
H	-1.88909	-2.76403	-0.90592
H	-1.59050	-1.13904	-1.53885
H	-1.60462	2.88591	-0.96276
H	-1.27090	2.59230	0.75387
H	-3.61610	-1.25352	-0.09095
H	-2.62021	-1.80452	1.26084
H	-3.49290	1.69734	0.12217
H	-2.75093	0.73072	-1.16080
C	3.19612	0.16303	1.09506
H	3.14422	-0.53357	1.93129
H	3.16038	1.17518	1.49656
H	4.16397	0.02868	0.59169
Na	-0.04461	0.00202	1.52432

ACE2_Na_CAM-B3LYP_TZV

33

O	-0.33778	-1.76513	-0.02595
O	-0.27140	1.36143	-0.54869
O	-2.32081	0.14110	0.79705
N	2.08581	-0.06333	0.20324
C	2.05564	-1.44974	-0.30675
C	2.05216	0.97195	-0.85537
C	0.75318	-1.83237	-0.98392
C	1.00910	2.04118	-0.58600
C	-1.65792	-1.76395	-0.62714

C	-1.43155	2.15297	-0.20091
C	-2.64871	-1.21455	0.37692
C	-2.60232	1.19866	-0.16774
H	2.21888	-2.11569	0.53986
H	2.87063	-1.63106	-1.02166
H	1.82292	0.51204	-1.81475
H	3.03195	1.44754	-0.95990
H	0.52852	-1.16290	-1.81499
H	0.83596	-2.84802	-1.37472
H	1.18918	2.54185	0.36954
H	1.01588	2.79089	-1.37965
H	-1.94754	-2.77915	-0.90526
H	-1.63751	-1.15167	-1.53008
H	-1.60875	2.93413	-0.94257
H	-1.28306	2.62452	0.77400
H	-3.65426	-1.24928	-0.04393
H	-2.63332	-1.80667	1.28792
H	-3.50879	1.72236	0.13274
H	-2.75573	0.77124	-1.15763
C	3.20094	0.14555	1.14183
H	3.12406	-0.55490	1.97093
H	3.15855	1.15463	1.54647
H	4.17736	0.00866	0.65880
Na	-0.04444	-0.01478	1.42887

ACE2_Na_DSDPBEP86_SV(P)

33

O	-0.32050	-1.70521	-0.03298
O	-0.27509	1.32700	-0.54486
O	-2.30812	0.12995	0.77565
N	2.06160	-0.05787	0.19391

C	2.05102	-1.43454	-0.29875
C	2.02403	0.95417	-0.86953
C	0.74164	-1.83197	-0.95986
C	0.96143	2.00831	-0.60654
C	-1.60418	-1.74593	-0.62401
C	-1.40594	2.10652	-0.22423
C	-2.61722	-1.19412	0.36395
C	-2.58406	1.15469	-0.17127
H	2.21915	-2.10108	0.56588
H	2.87649	-1.62664	-1.02186
H	1.79267	0.47674	-1.83614
H	3.01108	1.44450	-0.98937
H	0.53492	-1.19666	-1.84204
H	0.82202	-2.87930	-1.31499
H	1.15753	2.53974	0.34874
H	0.95590	2.76127	-1.42094
H	-1.88459	-2.78457	-0.89457
H	-1.60334	-1.14339	-1.55275
H	-1.58949	2.89017	-0.98694
H	-1.26639	2.60864	0.75615
H	-3.63178	-1.24551	-0.07571
H	-2.61143	-1.80293	1.28339
H	-3.50174	1.69937	0.12092
H	-2.74405	0.72302	-1.17485
C	3.16650	0.17479	1.11622
H	3.09068	-0.51405	1.97544
H	3.11565	1.20739	1.50240
H	4.16018	0.03123	0.63519
Na	-0.04585	0.01379	1.51148

ACE2_Na_DSDPBEP86_SVP

33

O	-0.31854	-1.70584	-0.03296
O	-0.27742	1.32317	-0.54535
O	-2.31104	0.12759	0.77806
N	2.06286	-0.05626	0.19480
C	2.05122	-1.43138	-0.30050
C	2.02165	0.95548	-0.86777
C	0.74283	-1.82447	-0.96152
C	0.95798	2.00635	-0.60612
C	-1.60113	-1.74057	-0.62621
C	-1.40874	2.10192	-0.22528
C	-2.61527	-1.19572	0.36142
C	-2.58529	1.15126	-0.16993
H	2.22000	-2.09825	0.55995
H	2.87328	-1.62254	-1.02257
H	1.79064	0.47850	-1.83148
H	3.00391	1.44746	-0.98955
H	0.53534	-1.18266	-1.83547
H	0.82364	-2.86463	-1.32770
H	1.15210	2.53711	0.34674
H	0.95365	2.75890	-1.41651
H	-1.88345	-2.77272	-0.90644
H	-1.59744	-1.13283	-1.54833
H	-1.59451	2.88329	-0.98504
H	-1.26958	2.60527	0.75133
H	-3.62546	-1.25073	-0.07973
H	-2.60973	-1.80833	1.27499
H	-3.50038	1.69680	0.11676
H	-2.74402	0.71954	-1.17069
C	3.17110	0.17459	1.11213

H	3.10294	-0.51652	1.96513
H	3.12175	1.20120	1.50335
H	4.15927	0.03644	0.62766
Na	-0.04565	0.01222	1.51898

ACE2_Na_DSDPBEP86_SV

33

O	-0.32050	-1.70521	-0.03298
O	-0.27509	1.32700	-0.54486
O	-2.30812	0.12995	0.77565
N	2.06160	-0.05787	0.19391
C	2.05102	-1.43454	-0.29875
C	2.02403	0.95417	-0.86953
C	0.74164	-1.83197	-0.95986
C	0.96143	2.00831	-0.60654
C	-1.60418	-1.74593	-0.62401
C	-1.40594	2.10652	-0.22423
C	-2.61722	-1.19412	0.36395
C	-2.58406	1.15469	-0.17127
H	2.21915	-2.10108	0.56588
H	2.87649	-1.62664	-1.02186
H	1.79267	0.47674	-1.83614
H	3.01108	1.44450	-0.98937
H	0.53492	-1.19666	-1.84204
H	0.82202	-2.87930	-1.31499
H	1.15753	2.53974	0.34874
H	0.95590	2.76127	-1.42094
H	-1.88459	-2.78457	-0.89457
H	-1.60334	-1.14339	-1.55275
H	-1.58949	2.89017	-0.98694
H	-1.26639	2.60864	0.75615

H	-3.63178	-1.24551	-0.07571
H	-2.61143	-1.80293	1.28339
H	-3.50174	1.69937	0.12092
H	-2.74405	0.72302	-1.17485
C	3.16650	0.17479	1.11622
H	3.09068	-0.51405	1.97544
H	3.11565	1.20739	1.50240
H	4.16018	0.03123	0.63519
Na	-0.04585	0.01379	1.51148

ACE2_Na_DSDPBEP86_TZVPP

33

O	-0.31204	-1.72274	-0.02640
O	-0.28921	1.30031	-0.57151
O	-2.33202	0.12090	0.79640
N	2.05668	-0.04602	0.20678
C	2.05369	-1.41823	-0.30111
C	2.01972	0.96613	-0.85883
C	0.74979	-1.80289	-0.96812
C	0.94436	2.00096	-0.60050
C	-1.59078	-1.71477	-0.64224
C	-1.41537	2.08448	-0.22446
C	-2.61213	-1.20373	0.35021
C	-2.59563	1.14391	-0.16377
H	2.22397	-2.08412	0.54703
H	2.86871	-1.59096	-1.01984
H	1.81082	0.49118	-1.81734
H	2.98894	1.46509	-0.95674
H	0.53044	-1.13862	-1.80830
H	0.83254	-2.82229	-1.35781
H	1.11109	2.51004	0.35687

H	0.93978	2.75603	-1.39327
H	-1.87310	-2.72345	-0.96189
H	-1.56119	-1.07292	-1.52726
H	-1.60197	2.86480	-0.96926
H	-1.25574	2.56860	0.74640
H	-3.60862	-1.25861	-0.09598
H	-2.60227	-1.82395	1.24601
H	-3.49493	1.69137	0.12619
H	-2.76056	0.70701	-1.14949
C	3.19436	0.17300	1.09402
H	3.14649	-0.52126	1.93255
H	3.15930	1.18847	1.48769
H	4.15477	0.03457	0.57813
Na	-0.03882	0.01093	1.53543

ACE2_Na_DSDPBEP86_TZVP

33

O	-0.31325	-1.72083	-0.02696
O	-0.28785	1.30090	-0.56919
O	-2.33086	0.12206	0.79591
N	2.05665	-0.04714	0.20606
C	2.05254	-1.41930	-0.30212
C	2.02110	0.96594	-0.85898
C	0.74840	-1.80344	-0.96910
C	0.94609	2.00140	-0.60115
C	-1.59314	-1.71667	-0.64100
C	-1.41443	2.08608	-0.22443
C	-2.61320	-1.20300	0.35157
C	-2.59490	1.14570	-0.16403
H	2.22296	-2.08714	0.54748
H	2.86956	-1.59314	-1.02217

H 1.81352 0.49094 -1.82033

H 2.99292 1.46554 -0.95577

H 0.52875 -1.14016 -1.81283

H 0.83007 -2.82616 -1.35774

H 1.11454 2.51427 0.35669

H 0.94043 2.75693 -1.39700

H -1.87509 -2.72950 -0.95696

H -1.56594 -1.07805 -1.53116

H -1.60020 2.86737 -0.97218

H -1.25553 2.57331 0.74775

H -3.61253 -1.25749 -0.09444

H -2.60371 -1.82302 1.25052

H -3.49625 1.69420 0.12743

H -2.76145 0.70874 -1.15201

C 3.19267 0.17082 1.09588

H 3.14104 -0.52204 1.93823

H 3.15974 1.18933 1.48799

H 4.15659 0.02821 0.58278

Na -0.03737 0.01052 1.53394

ACE2_Na_DSDPBEP86_TZV

33

O -0.33920 -1.77359 -0.03105

O -0.27246 1.32765 -0.57413

O -2.33838 0.14441 0.82161

N 2.08418 -0.06260 0.21709

C 2.05807 -1.44486 -0.32582

C 2.07601 0.98152 -0.84597

C 0.74585 -1.79542 -1.01483

C 1.00727 2.03646 -0.58859

C -1.66498 -1.74021 -0.64658

C	-1.42843	2.14242	-0.21219
C	-2.66013	-1.21802	0.37786
C	-2.61659	1.19961	-0.16373
H	2.21679	-2.13297	0.50993
H	2.87567	-1.60649	-1.05041
H	1.88146	0.52473	-1.82040
H	3.05682	1.47307	-0.91161
H	0.51068	-1.07832	-1.80803
H	0.82047	-2.79557	-1.45662
H	1.16367	2.52924	0.38039
H	1.00932	2.79467	-1.38015
H	-1.95947	-2.74670	-0.96551
H	-1.63094	-1.08735	-1.52542
H	-1.60109	2.92575	-0.95880
H	-1.25993	2.61192	0.76513
H	-3.67046	-1.24770	-0.04372
H	-2.63406	-1.82395	1.28428
H	-3.51775	1.73866	0.14053
H	-2.78127	0.76023	-1.15148
C	3.22326	0.12147	1.14806
H	3.13406	-0.58229	1.97860
H	3.20084	1.13662	1.55065
H	4.19323	-0.03753	0.64913
Na	-0.03990	-0.01849	1.47210

ACE2_Na_HSE06_SV(P)

33

O	-0.31732	-1.70381	-0.03530
O	-0.27759	1.34163	-0.52287
O	-2.31763	0.12716	0.76016
N	2.07574	-0.05512	0.18175

C	2.05515	-1.43173	-0.29728
C	2.01299	0.95666	-0.87399
C	0.74705	-1.84560	-0.94697
C	0.95524	2.01207	-0.60347
C	-1.59782	-1.75957	-0.61587
C	-1.41398	2.10890	-0.22566
C	-2.61467	-1.19526	0.35870
C	-2.58632	1.15141	-0.17935
H	2.23374	-2.08875	0.57159
H	2.87386	-1.63713	-1.02341
H	1.76884	0.47783	-1.83632
H	2.99459	1.45020	-1.01400
H	0.54251	-1.23481	-1.84753
H	0.83599	-2.89864	-1.28051
H	1.16483	2.55352	0.34305
H	0.94696	2.75897	-1.42251
H	-1.87793	-2.80252	-0.86723
H	-1.60383	-1.17807	-1.55834
H	-1.59744	2.88475	-0.99553
H	-1.29231	2.62453	0.75003
H	-3.62519	-1.25995	-0.08689
H	-2.61685	-1.79530	1.28361
H	-3.50534	1.69573	0.10700
H	-2.74325	0.72386	-1.18522
C	3.17249	0.18627	1.10135
H	3.11051	-0.50635	1.95791
H	3.11284	1.21438	1.49640
H	4.16956	0.05944	0.62268
Na	-0.04570	0.01139	1.52657

ACE2_Na_HSE06_SVP

33

O	-0.31727	-1.70731	-0.03372
O	-0.27816	1.33993	-0.52237
O	-2.31943	0.12667	0.76300
N	2.07665	-0.05475	0.18241
C	2.05392	-1.43062	-0.29900
C	2.01186	0.95694	-0.87293
C	0.74663	-1.83878	-0.94912
C	0.95533	2.01118	-0.60225
C	-1.59799	-1.75543	-0.61670
C	-1.41565	2.10699	-0.22488
C	-2.61432	-1.19593	0.35795
C	-2.58621	1.15040	-0.17878
H	2.23256	-2.08920	0.56642
H	2.87093	-1.63755	-1.02338
H	1.76568	0.47892	-1.83329
H	2.99102	1.44953	-1.01746
H	0.54025	-1.21903	-1.84129
H	0.83572	-2.88510	-1.29716
H	1.16483	2.55262	0.34240
H	0.94899	2.75931	-1.41752
H	-1.88190	-2.79289	-0.87789
H	-1.60075	-1.16951	-1.55469
H	-1.60089	2.88306	-0.99128
H	-1.29540	2.62370	0.74851
H	-3.62277	-1.26392	-0.08711
H	-2.61746	-1.79953	1.27868
H	-3.50515	1.69469	0.10078
H	-2.74059	0.72266	-1.18325
C	3.17603	0.18379	1.09976

H	3.12017	-0.51253	1.95022
H	3.11784	1.20627	1.50199
H	4.16993	0.06261	0.61986
Na	-0.04440	0.01031	1.52718

ACE2_Na_HSE06_SV

33

O	-0.31731	-1.70249	-0.03538
O	-0.27771	1.34087	-0.52289
O	-2.31840	0.12698	0.76035
N	2.07607	-0.05502	0.18178
C	2.05532	-1.43166	-0.29702
C	2.01304	0.95642	-0.87417
C	0.74715	-1.84527	-0.94674
C	0.95499	2.01152	-0.60384
C	-1.59769	-1.75906	-0.61610
C	-1.41398	2.10833	-0.22558
C	-2.61497	-1.19537	0.35834
C	-2.58667	1.15133	-0.17914
H	2.23373	-2.08858	0.57196
H	2.87404	-1.63733	-1.02307
H	1.76889	0.47724	-1.83632
H	2.99453	1.45015	-1.01443
H	0.54298	-1.23502	-1.84774
H	0.83571	-2.89857	-1.27956
H	1.16444	2.55341	0.34246
H	0.94650	2.75813	-1.42315
H	-1.87718	-2.80220	-0.86743
H	-1.60387	-1.17766	-1.55862
H	-1.59737	2.88415	-0.99550
H	-1.29209	2.62410	0.75002

H	-3.62536	-1.26028	-0.08753
H	-2.61719	-1.79569	1.28307
H	-3.50549	1.69601	0.10720
H	-2.74380	0.72384	-1.18500
C	3.17305	0.18660	1.10106
H	3.11124	-0.50582	1.95779
H	3.11350	1.21480	1.49588
H	4.17002	0.05964	0.62221
Na	-0.04544	0.01135	1.52707

ACE2_Na_HSE06_TZVPP

33

O	-0.31679	-1.72259	-0.03586
O	-0.28809	1.32743	-0.56649
O	-2.33828	0.12248	0.77680
N	2.07482	-0.04891	0.19171
C	2.05103	-1.41710	-0.31387
C	2.01542	0.97247	-0.85695
C	0.74486	-1.80750	-0.96816
C	0.94710	2.00954	-0.58761
C	-1.59574	-1.73106	-0.63684
C	-1.41765	2.09442	-0.22314
C	-2.61620	-1.19955	0.34277
C	-2.59206	1.14887	-0.17311
H	2.23226	-2.08412	0.53272
H	2.85764	-1.60231	-1.04151
H	1.79562	0.50585	-1.81907
H	2.98336	1.47373	-0.96997
H	0.52568	-1.15488	-1.82027
H	0.83340	-2.83080	-1.35178
H	1.11967	2.51407	0.37333

H	0.95612	2.77434	-1.37385
H	-1.88206	-2.74805	-0.93269
H	-1.57689	-1.11486	-1.54293
H	-1.61072	2.88034	-0.96354
H	-1.26946	2.58077	0.75067
H	-3.61195	-1.26170	-0.10838
H	-2.61899	-1.81174	1.24648
H	-3.49628	1.69563	0.11068
H	-2.75263	0.71977	-1.16520
C	3.20740	0.16398	1.07640
H	3.16914	-0.54011	1.90891
H	3.17251	1.17381	1.48727
H	4.17217	0.03801	0.56154
Na	-0.04017	-0.00803	1.58391

ACE2_Na_HSE06_TZVP

33

O	-0.31714	-1.72232	-0.03543
O	-0.28754	1.32746	-0.56526
O	-2.33815	0.12301	0.77653
N	2.07501	-0.04937	0.19134
C	2.05072	-1.41751	-0.31454
C	2.01591	0.97251	-0.85699
C	0.74424	-1.80801	-0.96817
C	0.94773	2.00975	-0.58766
C	-1.59650	-1.73207	-0.63584
C	-1.41746	2.09492	-0.22331
C	-2.61668	-1.19935	0.34345
C	-2.59186	1.14931	-0.17373
H	2.23238	-2.08516	0.53238
H	2.85738	-1.60302	-1.04325

H	1.79603	0.50608	-1.81993
H	2.98449	1.47425	-0.96970
H	0.52482	-1.15582	-1.82144
H	0.83280	-2.83243	-1.35118
H	1.12114	2.51583	0.37319
H	0.95656	2.77415	-1.37543
H	-1.88305	-2.75046	-0.92984
H	-1.57850	-1.11711	-1.54363
H	-1.61044	2.88066	-0.96513
H	-1.27019	2.58252	0.75090
H	-3.61308	-1.26173	-0.10814
H	-2.61944	-1.81118	1.24833
H	-3.49666	1.69669	0.11021
H	-2.75221	0.72024	-1.16665
C	3.20698	0.16306	1.07709
H	3.16704	-0.54064	1.91076
H	3.17257	1.17386	1.48742
H	4.17290	0.03551	0.56320
Na	-0.03960	-0.00705	1.58274

ACE2_Na_HSE06_TZV

33

O	-0.33055	-1.76364	-0.02484
O	-0.27978	1.34802	-0.55882
O	-2.33862	0.13567	0.80254
N	2.08880	-0.05736	0.20186
C	2.06070	-1.44069	-0.30946
C	2.04573	0.97553	-0.85635
C	0.75799	-1.82468	-0.98318
C	0.99491	2.03592	-0.58751
C	-1.64491	-1.75380	-0.63435

C	-1.43676	2.13958	-0.20657
C	-2.64517	-1.21815	0.36653
C	-2.60917	1.18815	-0.16912
H	2.22987	-2.10912	0.53768
H	2.87459	-1.62356	-1.03021
H	1.81874	0.51237	-1.81827
H	3.02312	1.46140	-0.96501
H	0.53233	-1.15178	-1.81560
H	0.84612	-2.84046	-1.38077
H	1.17069	2.53659	0.37245
H	1.00354	2.79135	-1.37977
H	-1.93693	-2.76688	-0.92939
H	-1.62040	-1.12931	-1.53273
H	-1.61994	2.92361	-0.94812
H	-1.28603	2.61470	0.76967
H	-3.64912	-1.26445	-0.06455
H	-2.63212	-1.81938	1.27496
H	-3.51619	1.72089	0.12401
H	-2.76321	0.75699	-1.16050
C	3.21118	0.15414	1.12672
H	3.14715	-0.55031	1.95676
H	3.16790	1.16384	1.53622
H	4.18724	0.02437	0.63445
Na	-0.03767	-0.01325	1.47038

ACE2_Na_M062x_SV(P)

33

O	-0.32587	-1.68734	-0.03381
O	-0.27284	1.30686	-0.53526
O	-2.30170	0.12832	0.77826
N	2.06714	-0.06250	0.19768

C	2.04452	-1.43616	-0.29983
C	2.03122	0.95182	-0.86234
C	0.73014	-1.81174	-0.96139
C	0.95611	1.99409	-0.60757
C	-1.60676	-1.73446	-0.61958
C	-1.40079	2.09228	-0.23241
C	-2.61779	-1.19124	0.37418
C	-2.58475	1.14858	-0.16392
H	2.20631	-2.10808	0.55960
H	2.86580	-1.62796	-1.02361
H	1.81834	0.47525	-1.83231
H	3.01208	1.45190	-0.96575
H	0.52639	-1.15844	-1.83046
H	0.79582	-2.85195	-1.33232
H	1.14834	2.53326	0.34263
H	0.93900	2.73899	-1.42538
H	-1.87802	-2.77119	-0.89590
H	-1.61124	-1.12477	-1.54303
H	-1.57454	2.86356	-1.00583
H	-1.25922	2.60578	0.74009
H	-3.63278	-1.23934	-0.05885
H	-2.60561	-1.80248	1.28990
H	-3.49304	1.69890	0.13662
H	-2.75784	0.71350	-1.16280
C	3.17325	0.15993	1.11876
H	3.09780	-0.53367	1.97148
H	3.12883	1.18877	1.51066
H	4.16120	0.01459	0.63183
Na	-0.04592	0.03177	1.48735

ACE2_Na_M062x_SVP

33

O	-0.32480	-1.69071	-0.03234
O	-0.27469	1.30370	-0.53739
O	-2.30403	0.12676	0.78178
N	2.06819	-0.06128	0.19938
C	2.04426	-1.43372	-0.30196
C	2.03120	0.95431	-0.85924
C	0.73056	-1.80502	-0.96337
C	0.95425	1.99307	-0.60619
C	-1.60568	-1.73040	-0.62060
C	-1.40293	2.08908	-0.23204
C	-2.61694	-1.19290	0.37321
C	-2.58610	1.14725	-0.16144
H	2.20724	-2.10757	0.55307
H	2.86349	-1.62453	-1.02491
H	1.82192	0.47984	-1.82881
H	3.00869	1.45628	-0.96178
H	0.52437	-1.14440	-1.82386
H	0.79654	-2.83851	-1.34652
H	1.14298	2.53153	0.34300
H	0.93888	2.73943	-1.41978
H	-1.87948	-2.76146	-0.90694
H	-1.60656	-1.11522	-1.53822
H	-1.57867	2.86076	-1.00151
H	-1.25994	2.60276	0.73803
H	-3.62937	-1.24361	-0.06003
H	-2.60576	-1.80756	1.28441
H	-3.49243	1.69838	0.13583
H	-2.76011	0.71257	-1.15828
C	3.17753	0.15705	1.11784

H	3.10668	-0.53913	1.96515
H	3.13603	1.18088	1.51505
H	4.16154	0.01469	0.62885
Na	-0.04565	0.03122	1.48526

ACE2_Na_M062x_SV

33

O	-0.32566	-1.68631	-0.03398
O	-0.27294	1.30654	-0.53522
O	-2.30213	0.12812	0.77825
N	2.06736	-0.06236	0.19776
C	2.04476	-1.43597	-0.29984
C	2.03122	0.95195	-0.86221
C	0.73039	-1.81153	-0.96139
C	0.95578	1.99398	-0.60763
C	-1.60650	-1.73421	-0.61972
C	-1.40093	2.09191	-0.23230
C	-2.61779	-1.19143	0.37400
C	-2.58498	1.14833	-0.16398
H	2.20648	-2.10795	0.55956
H	2.86611	-1.62775	-1.02357
H	1.81863	0.47528	-1.83219
H	3.01197	1.45230	-0.96551
H	0.52679	-1.15879	-1.83091
H	0.79588	-2.85202	-1.33164
H	1.14780	2.53357	0.34237
H	0.93864	2.73855	-1.42576
H	-1.87723	-2.77114	-0.89584
H	-1.61127	-1.12473	-1.54331
H	-1.57462	2.86335	-1.00558
H	-1.25936	2.60525	0.74028

H	-3.63271	-1.23982	-0.05922
H	-2.60557	-1.80277	1.28965
H	-3.49334	1.69867	0.13630
H	-2.75782	0.71320	-1.16290
C	3.17364	0.16006	1.11863
H	3.09831	-0.53350	1.97140
H	3.12934	1.18892	1.51050
H	4.16151	0.01465	0.63155
Na	-0.04626	0.03145	1.48771

ACE2_Na_M062x_TZVPP

33

O	-0.31957	-1.70094	-0.03258
O	-0.28430	1.29218	-0.56789
O	-2.32728	0.12414	0.79071
N	2.06522	-0.05436	0.20309
C	2.04710	-1.42455	-0.30961
C	2.02976	0.96544	-0.85471
C	0.73606	-1.79004	-0.97389
C	0.94612	1.99164	-0.59651
C	-1.60035	-1.71360	-0.63335
C	-1.40878	2.07795	-0.23137
C	-2.61583	-1.19990	0.36407
C	-2.59423	1.14365	-0.16487
H	2.21315	-2.09767	0.53350
H	2.85830	-1.59864	-1.03120
H	1.83397	0.49869	-1.82011
H	2.99608	1.47124	-0.93982
H	0.52276	-1.11701	-1.80950
H	0.80181	-2.80792	-1.37046
H	1.11053	2.49864	0.36294

H	0.93678	2.75040	-1.38551
H	-1.87251	-2.72812	-0.94226
H	-1.58683	-1.07913	-1.52510
H	-1.58578	2.85552	-0.98074
H	-1.25006	2.56657	0.73785
H	-3.61665	-1.25626	-0.07199
H	-2.59814	-1.81499	1.26355
H	-3.49002	1.69462	0.12859
H	-2.76569	0.70724	-1.15034
C	3.19663	0.15464	1.09984
H	3.13789	-0.53957	1.93736
H	3.16756	1.17001	1.49419
H	4.15878	0.00855	0.59051
Na	-0.03620	0.02218	1.53068

ACE2_Na_M062x_TZVP

33

O	-0.31959	-1.70110	-0.03230
O	-0.28441	1.29201	-0.56765
O	-2.32728	0.12390	0.79099
N	2.06534	-0.05425	0.20329
C	2.04719	-1.42450	-0.30962
C	2.03014	0.96576	-0.85450
C	0.73621	-1.79057	-0.97368
C	0.94605	1.99176	-0.59722
C	-1.60049	-1.71398	-0.63324
C	-1.40903	2.07806	-0.23135
C	-2.61606	-1.20015	0.36401
C	-2.59462	1.14396	-0.16415
H	2.21359	-2.09815	0.53384
H	2.85881	-1.59846	-1.03179

H	1.83569	0.49889	-1.82079
H	2.99679	1.47252	-0.93852
H	0.52233	-1.11822	-1.81045
H	0.80231	-2.80959	-1.36935
H	1.11058	2.50051	0.36199
H	0.93659	2.74982	-1.38789
H	-1.87273	-2.72942	-0.94184
H	-1.58685	-1.07957	-1.52576
H	-1.58659	2.85546	-0.98184
H	-1.25005	2.56786	0.73796
H	-3.61738	-1.25607	-0.07267
H	-2.59874	-1.81558	1.26409
H	-3.49037	1.69565	0.13084
H	-2.76753	0.70791	-1.15020
C	3.19677	0.15450	1.10043
H	3.13697	-0.53902	1.93928
H	3.16843	1.17090	1.49388
H	4.15947	0.00678	0.59125
Na	-0.03595	0.02273	1.52933

ACE2_Na_M062x_TZV

33

O	-0.33847	-1.73507	-0.02698
O	-0.27130	1.31597	-0.56074
O	-2.31935	0.14057	0.80980
N	2.08024	-0.06725	0.20954
C	2.04994	-1.44969	-0.31209
C	2.06222	0.96697	-0.85201
C	0.74030	-1.79745	-0.99557
C	0.99892	2.01733	-0.58808
C	-1.65465	-1.73348	-0.63201

C	-1.42150	2.12499	-0.21366
C	-2.64565	-1.21241	0.38839
C	-2.60425	1.18452	-0.16476
H	2.19838	-2.12696	0.52954
H	2.86737	-1.62521	-1.02564
H	1.85533	0.50731	-1.81792
H	3.03910	1.45351	-0.93293
H	0.52115	-1.09712	-1.80421
H	0.80053	-2.80396	-1.41410
H	1.16519	2.51042	0.37518
H	0.99320	2.77393	-1.37534
H	-1.93223	-2.74428	-0.93731
H	-1.63726	-1.09312	-1.51707
H	-1.58666	2.90429	-0.95967
H	-1.26070	2.59570	0.76036
H	-3.65584	-1.24921	-0.02213
H	-2.61068	-1.81549	1.29245
H	-3.50494	1.71826	0.13543
H	-2.76367	0.74648	-1.15013
C	3.20032	0.13350	1.14710
H	3.11459	-0.56538	1.97721
H	3.16453	1.14593	1.54593
H	4.17239	-0.01521	0.65936
Na	-0.03839	0.00668	1.43397

ACE2_Na_M06_SV(P)

33

O	-0.30475	-1.69164	-0.04602
O	-0.29366	1.33355	-0.59630
O	-2.32716	0.11211	0.75728
N	2.07787	-0.04602	0.18347

C	2.06241	-1.41929	-0.30284
C	2.01106	0.97108	-0.86711
C	0.76036	-1.82547	-0.95610
C	0.94039	2.00314	-0.59287
C	-1.57689	-1.73306	-0.64296
C	-1.41259	2.08747	-0.21989
C	-2.60444	-1.20609	0.33239
C	-2.59241	1.14828	-0.16780
H	2.24212	-2.08460	0.56405
H	2.88905	-1.61515	-1.02784
H	1.78674	0.49851	-1.84156
H	2.99120	1.47740	-0.99408
H	0.55544	-1.20194	-1.85248
H	0.84266	-2.87611	-1.30822
H	1.11092	2.49236	0.39539
H	0.95980	2.80273	-1.36503
H	-1.84707	-2.76943	-0.94019
H	-1.57123	-1.11661	-1.56813
H	-1.61293	2.91008	-0.94004
H	-1.24717	2.55613	0.77800
H	-3.61514	-1.27617	-0.11861
H	-2.60430	-1.82627	1.24756
H	-3.50340	1.70315	0.13253
H	-2.77212	0.73267	-1.17898
C	3.18450	0.19330	1.08873
H	3.13420	-0.49648	1.95106
H	3.13580	1.22577	1.48103
H	4.17666	0.06003	0.59489
Na	-0.07511	-0.02021	1.61431

ACE2_Na_M06_SVP

33

O	-0.30416	-1.69442	-0.04387
O	-0.29494	1.33295	-0.59727
O	-2.32852	0.11076	0.75950
N	2.07769	-0.04486	0.18394
C	2.06204	-1.41834	-0.30237
C	2.00850	0.97094	-0.86754
C	0.76186	-1.82315	-0.95522
C	0.94008	2.00261	-0.59295
C	-1.57587	-1.72933	-0.64436
C	-1.41454	2.08603	-0.21814
C	-2.60393	-1.20699	0.32949
C	-2.59207	1.14728	-0.16705
H	2.24293	-2.08280	0.56146
H	2.88705	-1.61631	-1.02396
H	1.78129	0.49939	-1.83897
H	2.98546	1.47530	-1.00109
H	0.55705	-1.19796	-1.84764
H	0.84548	-2.86782	-1.31584
H	1.11156	2.49082	0.39282
H	0.96209	2.80377	-1.35918
H	-1.84922	-2.75896	-0.95125
H	-1.56755	-1.11045	-1.56513
H	-1.61746	2.91018	-0.93126
H	-1.25001	2.55379	0.77746
H	-3.61099	-1.28008	-0.12204
H	-2.60727	-1.83178	1.23843
H	-3.50278	1.70126	0.12545
H	-2.76875	0.73309	-1.17672
C	3.18761	0.19412	1.08581

H	3.14377	-0.49496	1.94440
H	3.14177	1.22186	1.48062
H	4.17532	0.06380	0.59112
Na	-0.07497	-0.02105	1.61644

ACE2_Na_M06_SV

33

O	-0.30478	-1.69134	-0.04614
O	-0.29356	1.33349	-0.59642
O	-2.32741	0.11219	0.75749
N	2.07787	-0.04619	0.18334
C	2.06247	-1.41937	-0.30309
C	2.01118	0.97108	-0.86707
C	0.76026	-1.82537	-0.95624
C	0.94056	2.00315	-0.59264
C	-1.57702	-1.73283	-0.64285
C	-1.41259	2.08733	-0.22006
C	-2.60451	-1.20609	0.33270
C	-2.59243	1.14816	-0.16786
H	2.24238	-2.08469	0.56374
H	2.88894	-1.61522	-1.02828
H	1.78660	0.49865	-1.84151
H	2.99143	1.47717	-0.99408
H	0.55552	-1.20165	-1.85253
H	0.84227	-2.87599	-1.30845
H	1.11095	2.49203	0.39580
H	0.95993	2.80306	-1.36443
H	-1.84713	-2.76920	-0.94018
H	-1.57156	-1.11630	-1.56797
H	-1.61296	2.90988	-0.94027
H	-1.24728	2.55612	0.77780

H	-3.61525	-1.27628	-0.11817
H	-2.60416	-1.82627	1.24787
H	-3.50333	1.70328	0.13230
H	-2.77217	0.73235	-1.17896
C	3.18411	0.19320	1.08899
H	3.13370	-0.49675	1.95118
H	3.13512	1.22558	1.48151
H	4.17650	0.06028	0.59551
Na	-0.07462	-0.02027	1.61409

ACE2_Na_M06_TZVPP

33

O	-0.31289	-1.70856	-0.05397
O	-0.30003	1.34608	-0.62917
O	-2.32606	0.11048	0.76288
N	2.05968	-0.04183	0.18157
C	2.04850	-1.41338	-0.31337
C	1.99969	0.97518	-0.87136
C	0.75249	-1.81136	-0.97270
C	0.93936	2.00916	-0.58895
C	-1.58055	-1.71102	-0.66779
C	-1.40845	2.08935	-0.19940
C	-2.60593	-1.19877	0.30956
C	-2.58440	1.15351	-0.16001
H	2.22492	-2.07274	0.54118
H	2.86716	-1.59735	-1.02829
H	1.77077	0.50943	-1.83237
H	2.97151	1.46730	-0.99334
H	0.54335	-1.17190	-1.83936
H	0.83861	-2.83934	-1.34600
H	1.09602	2.46298	0.40257

H	0.97533	2.81723	-1.33081
H	-1.86020	-2.72197	-0.99095
H	-1.55533	-1.07695	-1.56383
H	-1.62020	2.92804	-0.87516
H	-1.22374	2.51210	0.79977
H	-3.60044	-1.25371	-0.14605
H	-2.61585	-1.83020	1.20085
H	-3.48517	1.69854	0.13879
H	-2.75725	0.74836	-1.16160
C	3.19281	0.18470	1.06081
H	3.15963	-0.50576	1.90465
H	3.16009	1.20128	1.45535
H	4.15522	0.05015	0.54342
Na	-0.04285	-0.06189	1.69378

ACE2_Na_M06_TZVP

33

O	-0.31320	-1.70674	-0.05506
O	-0.30022	1.34564	-0.63037
O	-2.32640	0.11017	0.76287
N	2.06002	-0.04186	0.18107
C	2.04766	-1.41277	-0.31455
C	1.99962	0.97572	-0.87099
C	0.75192	-1.81013	-0.97339
C	0.93884	2.00844	-0.58889
C	-1.58055	-1.70975	-0.66867
C	-1.40803	2.08849	-0.19934
C	-2.60620	-1.19831	0.30799
C	-2.58428	1.15383	-0.15910
H	2.22495	-2.07336	0.54041
H	2.86720	-1.59679	-1.03061

H	1.77188	0.50981	-1.83352
H	2.97234	1.46906	-0.99225
H	0.54299	-1.17035	-1.84135
H	0.83785	-2.83925	-1.34773
H	1.09501	2.46220	0.40415
H	0.97557	2.81878	-1.33034
H	-1.86019	-2.72189	-0.99301
H	-1.55580	-1.07517	-1.56583
H	-1.62044	2.92923	-0.87467
H	-1.22185	2.51141	0.80093
H	-3.60122	-1.25263	-0.14972
H	-2.61788	-1.83155	1.19946
H	-3.48525	1.70076	0.14058
H	-2.75895	0.74912	-1.16192
C	3.19331	0.18397	1.05947
H	3.16060	-0.50692	1.90438
H	3.16151	1.20175	1.45384
H	4.15630	0.04861	0.54063
Na	-0.04181	-0.06448	1.69949

ACE2_Na_M06_TZV

33

O	-0.31106	-1.75100	-0.04357
O	-0.30099	1.36287	-0.64591
O	-2.31994	0.11628	0.78799
N	2.07355	-0.04568	0.19115
C	2.06995	-1.43200	-0.30459
C	2.02469	0.98072	-0.86975
C	0.77907	-1.82836	-0.98709
C	0.97498	2.03247	-0.58564
C	-1.61194	-1.73471	-0.66598

C	-1.43304	2.12553	-0.19261
C	-2.61859	-1.22679	0.33935
C	-2.60603	1.17968	-0.15508
H	2.23065	-2.08939	0.55381
H	2.89803	-1.61229	-1.00972
H	1.78838	0.51711	-1.82981
H	3.00289	1.46383	-0.98654
H	0.56357	-1.16710	-1.83364
H	0.86895	-2.84895	-1.37326
H	1.12221	2.46777	0.41302
H	1.01914	2.84103	-1.32319
H	-1.89576	-2.73990	-0.99489
H	-1.58222	-1.08246	-1.54667
H	-1.64474	2.96196	-0.86711
H	-1.23649	2.53408	0.80733
H	-3.62294	-1.27560	-0.09083
H	-2.59790	-1.84398	1.23765
H	-3.50807	1.70900	0.15894
H	-2.77644	0.76882	-1.15378
C	3.18176	0.19372	1.12265
H	3.12289	-0.50339	1.95937
H	3.11837	1.20811	1.51925
H	4.16298	0.07414	0.63709
Na	-0.05977	-0.05725	1.58085

ACE2_Na_MP2_SV(P)

33

O	-0.32604	-1.70233	-0.03578
O	-0.27115	1.32140	-0.54559
O	-2.31464	0.13600	0.77182
N	2.06526	-0.06097	0.19331

C	2.04353	-1.42982	-0.31910
C	2.03011	0.96037	-0.86064
C	0.72897	-1.80971	-0.97589
C	0.96481	2.00808	-0.59224
C	-1.61158	-1.74025	-0.62766
C	-1.40251	2.10527	-0.22905
C	-2.62363	-1.18945	0.35930
C	-2.58148	1.15660	-0.18372
H	2.21335	-2.11121	0.53312
H	2.86275	-1.61461	-1.05132
H	1.80541	0.49461	-1.83431
H	3.01665	1.45414	-0.97079
H	0.51616	-1.15480	-1.84121
H	0.80456	-2.84904	-1.35494
H	1.15585	2.52823	0.36973
H	0.96243	2.77013	-1.39828
H	-1.89358	-2.77764	-0.90116
H	-1.61076	-1.13587	-1.55424
H	-1.58032	2.88988	-0.99211
H	-1.26852	2.60591	0.75222
H	-3.63788	-1.24093	-0.08097
H	-2.61826	-1.79814	1.27826
H	-3.50164	1.70330	0.09674
H	-2.73147	0.72253	-1.18709
C	3.18986	0.14868	1.09817
H	3.12084	-0.54824	1.95034
H	3.15769	1.17702	1.49501
H	4.17183	-0.00173	0.59747
Na	-0.04870	0.00128	1.56379

ACE2_Na_MP2_SVP

33

O	-0.32703	-1.69844	-0.03920
O	-0.27154	1.31614	-0.54629
O	-2.31957	0.13503	0.77389
N	2.06863	-0.06109	0.19479
C	2.04014	-1.42510	-0.32682
C	2.03047	0.96335	-0.85426
C	0.72456	-1.79299	-0.98258
C	0.96193	2.00487	-0.58753
C	-1.61104	-1.73113	-0.63158
C	-1.40242	2.09886	-0.23097
C	-2.62417	-1.18778	0.35492
C	-2.58087	1.15335	-0.18324
H	2.20984	-2.11016	0.51661
H	2.85240	-1.60666	-1.05988
H	1.81078	0.50119	-1.82596
H	3.00965	1.46101	-0.96052
H	0.51156	-1.12508	-1.83248
H	0.79751	-2.82048	-1.37991
H	1.14834	2.52052	0.37285
H	0.96250	2.76757	-1.38618
H	-1.89458	-2.76025	-0.91463
H	-1.60836	-1.12179	-1.54984
H	-1.58180	2.88027	-0.98975
H	-1.26893	2.59922	0.74548
H	-3.63184	-1.24175	-0.08781
H	-2.61960	-1.80082	1.26575
H	-3.49626	1.70269	0.08821
H	-2.72754	0.71888	-1.18223
C	3.20131	0.13847	1.08862

H	3.14217	-0.56337	1.93034
H	3.17694	1.15769	1.49439
H	4.17258	-0.00784	0.57862
Na	-0.04910	-0.00695	1.58426

ACE2_Na_MP2_SV

33

O	-0.32604	-1.70233	-0.03578
O	-0.27115	1.32140	-0.54559
O	-2.31464	0.13600	0.77182
N	2.06526	-0.06097	0.19331
C	2.04353	-1.42982	-0.31910
C	2.03011	0.96037	-0.86064
C	0.72897	-1.80971	-0.97589
C	0.96481	2.00808	-0.59224
C	-1.61158	-1.74025	-0.62766
C	-1.40251	2.10527	-0.22905
C	-2.62363	-1.18945	0.35930
C	-2.58148	1.15660	-0.18372
H	2.21335	-2.11121	0.53312
H	2.86275	-1.61461	-1.05132
H	1.80541	0.49461	-1.83431
H	3.01665	1.45414	-0.97079
H	0.51616	-1.15480	-1.84121
H	0.80456	-2.84904	-1.35494
H	1.15585	2.52823	0.36973
H	0.96243	2.77013	-1.39828
H	-1.89358	-2.77764	-0.90116
H	-1.61076	-1.13587	-1.55424
H	-1.58032	2.88988	-0.99211
H	-1.26852	2.60591	0.75222

H	-3.63788	-1.24093	-0.08097
H	-2.61826	-1.79814	1.27826
H	-3.50164	1.70330	0.09674
H	-2.73147	0.72253	-1.18709
C	3.18986	0.14868	1.09817
H	3.12084	-0.54824	1.95034
H	3.15769	1.17702	1.49501
H	4.17183	-0.00173	0.59747
Na	-0.04870	0.00128	1.56379

ACE2_Na_MP2_TZVP

33

O	-0.31331	1.70805	0.02524
O	-0.28721	-1.28276	0.56192
O	-2.33580	-0.12315	-0.79419
N	2.05538	0.04580	-0.20799
C	2.04808	1.41214	0.31139
C	2.01953	-0.96825	0.85402
C	0.74249	1.78332	0.97529
C	0.94142	-1.99469	0.59280
C	-1.59066	1.70363	0.64632
C	-1.41325	-2.07629	0.22917
C	-2.61380	1.20105	-0.34358
C	-2.59401	-1.14097	0.17346
H	2.21935	2.08742	-0.53035
H	2.86159	1.57906	1.03528
H	1.81613	-0.49719	1.81682
H	2.98879	-1.47081	0.94576
H	0.52019	1.10924	1.80767
H	0.81750	2.80017	1.37640
H	1.10719	-2.50421	-0.36538

H	0.92841	-2.75116	1.38569
H	-1.86740	2.71349	0.97106
H	-1.56013	1.05863	1.52973
H	-1.58931	-2.85287	0.98189
H	-1.25999	-2.56665	-0.74030
H	-3.61039	1.25625	0.10499
H	-2.60432	1.82424	-1.23830
H	-3.49562	-1.69070	-0.10855
H	-2.75324	-0.69962	1.15890
C	3.21120	-0.15987	-1.07449
H	3.17262	0.53601	-1.91261
H	3.19376	-1.17531	-1.47037
H	4.16012	-0.01260	-0.53921
Na	-0.04076	-0.00377	-1.57120

ACE2_Na_MP2_TZV

33

O	-0.36323	-1.77860	-0.04569
O	-0.25109	1.31832	-0.56121
O	-2.35494	0.16719	0.82539
N	2.10302	-0.08027	0.22042
C	2.03728	-1.44561	-0.37937
C	2.11561	0.99766	-0.81894
C	0.70501	-1.73407	-1.06400
C	1.03302	2.04236	-0.55705
C	-1.70807	-1.74002	-0.64677
C	-1.41635	2.15244	-0.22656
C	-2.69042	-1.20609	0.38917
C	-2.61562	1.21799	-0.18387
H	2.19102	-2.17134	0.42576
H	2.84095	-1.58997	-1.12482

H	1.95362	0.56802	-1.81338
H	3.09525	1.49779	-0.83835
H	0.45482	-0.95661	-1.79302
H	0.75771	-2.70177	-1.57717
H	1.17555	2.51970	0.42194
H	1.03272	2.80989	-1.34036
H	-2.01077	-2.74737	-0.95661
H	-1.67930	-1.08997	-1.52801
H	-1.56878	2.92387	-0.99032
H	-1.25760	2.63236	0.74730
H	-3.70481	-1.22627	-0.02496
H	-2.65934	-1.80224	1.30203
H	-3.51551	1.76787	0.10622
H	-2.77285	0.76746	-1.16837
C	3.27593	0.03418	1.13527
H	3.17463	-0.69133	1.94550
H	3.29714	1.03849	1.56445
H	4.22620	-0.14687	0.60628
Na	-0.03782	-0.03711	1.53253

ACE2_Na_PBE0_SV(P)

33

O	-0.31853	-1.70454	-0.03464
O	-0.27683	1.34233	-0.52307
O	-2.31618	0.12775	0.75975
N	2.07438	-0.05577	0.18135
C	2.05358	-1.43234	-0.29738
C	2.01293	0.95592	-0.87427
C	0.74513	-1.84705	-0.94602
C	0.95562	2.01197	-0.60433
C	-1.59849	-1.76041	-0.61483

C	-1.41246	2.10938	-0.22538
C	-2.61499	-1.19364	0.35880
C	-2.58504	1.15209	-0.17852
H	2.23284	-2.08900	0.57162
H	2.87177	-1.63796	-1.02395
H	1.76906	0.47734	-1.83672
H	2.99491	1.44888	-1.01369
H	0.54042	-1.23783	-1.84753
H	0.83462	-2.90062	-1.27799
H	1.16575	2.55445	0.34150
H	0.94793	2.75799	-1.42429
H	-1.87972	-2.80367	-0.86399
H	-1.60440	-1.18088	-1.55847
H	-1.59668	2.88499	-0.99540
H	-1.29045	2.62543	0.75009
H	-3.62541	-1.25727	-0.08736
H	-2.61876	-1.79339	1.28398
H	-3.50365	1.69674	0.10895
H	-2.74305	0.72557	-1.18463
C	3.17084	0.18519	1.10128
H	3.10798	-0.50730	1.95794
H	3.11130	1.21337	1.49626
H	4.16810	0.05791	0.62322
Na	-0.04306	0.01315	1.52533

ACE2_Na_PBE0_SVP

33

O	-0.31780	-1.70851	-0.03291
O	-0.27787	1.34081	-0.52275
O	-2.31813	0.12688	0.76262
N	2.07543	-0.05490	0.18202

C	2.05308	-1.43092	-0.29874
C	2.01152	0.95660	-0.87326
C	0.74571	-1.84084	-0.94772
C	0.95524	2.01132	-0.60319
C	-1.59797	-1.75649	-0.61573
C	-1.41477	2.10736	-0.22456
C	-2.61427	-1.19474	0.35776
C	-2.58534	1.15071	-0.17785
H	2.23279	-2.08869	0.56700
H	2.86972	-1.63804	-1.02329
H	1.76601	0.47874	-1.83372
H	2.99082	1.44890	-1.01722
H	0.53899	-1.22360	-1.84140
H	0.83571	-2.88784	-1.29351
H	1.16485	2.55389	0.34072
H	0.94940	2.75867	-1.41919
H	-1.88288	-2.79416	-0.87503
H	-1.60035	-1.17259	-1.55486
H	-1.60104	2.88337	-0.99076
H	-1.29389	2.62433	0.74859
H	-3.62248	-1.26197	-0.08796
H	-2.61921	-1.79790	1.27881
H	-3.50389	1.69510	0.10303
H	-2.74119	0.72399	-1.18245
C	3.17419	0.18380	1.09993
H	3.11761	-0.51224	1.95056
H	3.11561	1.20633	1.50193
H	4.16837	0.06254	0.62097
Na	-0.04244	0.01193	1.52529

ACE2_Na_PBE0_SV

33

O	-0.31733	-1.70336	-0.03496
O	-0.27777	1.34149	-0.52350
O	-2.31665	0.12689	0.76014
N	2.07454	-0.05498	0.18157
C	2.05466	-1.43163	-0.29686
C	2.01239	0.95632	-0.87445
C	0.74663	-1.84697	-0.94581
C	0.95440	2.01167	-0.60478
C	-1.59721	-1.76008	-0.61528
C	-1.41347	2.10841	-0.22565
C	-2.61422	-1.19460	0.35856
C	-2.58595	1.15105	-0.17819
H	2.23373	-2.08799	0.57241
H	2.87326	-1.63712	-1.02299
H	1.76907	0.47709	-1.83670
H	2.99397	1.45002	-1.01390
H	0.54229	-1.23874	-1.84808
H	0.83629	-2.90090	-1.27659
H	1.16412	2.55420	0.34113
H	0.94632	2.75787	-1.42455
H	-1.87749	-2.80351	-0.86478
H	-1.60358	-1.18021	-1.55871
H	-1.59811	2.88399	-0.99558
H	-1.29122	2.62440	0.74983
H	-3.62453	-1.25897	-0.08772
H	-2.61755	-1.79468	1.28352
H	-3.50442	1.69574	0.10960
H	-2.74445	0.72431	-1.18411
C	3.17079	0.18684	1.10151

H	3.10832	-0.50545	1.95836
H	3.11066	1.21508	1.49626
H	4.16815	0.05999	0.62353
Na	-0.04370	0.01305	1.52548

ACE2_Na_PBE0_TZVPP

33

O	-0.31677	-1.72359	-0.03534
O	-0.28851	1.32710	-0.56726
O	-2.33732	0.12232	0.77730
N	2.07327	-0.04862	0.19209
C	2.05032	-1.41633	-0.31470
C	2.01502	0.97296	-0.85637
C	0.74361	-1.80781	-0.96791
C	0.94557	2.00960	-0.58828
C	-1.59491	-1.73137	-0.63628
C	-1.41730	2.09376	-0.22367
C	-2.61563	-1.19863	0.34297
C	-2.59184	1.14787	-0.17204
H	2.23302	-2.08388	0.53148
H	2.85636	-1.60033	-1.04352
H	1.79718	0.50633	-1.81914
H	2.98298	1.47500	-0.96744
H	0.52358	-1.15570	-1.82045
H	0.83337	-2.83141	-1.35135
H	1.11775	2.51531	0.37241
H	0.95507	2.77387	-1.37545
H	-1.88247	-2.74850	-0.93156
H	-1.57575	-1.11555	-1.54284
H	-1.61173	2.87925	-0.96460
H	-1.26875	2.58104	0.74993

H	-3.61130	-1.26045	-0.10913
H	-2.61946	-1.81143	1.24664
H	-3.49577	1.69531	0.11266
H	-2.75367	0.71889	-1.16420
C	3.20687	0.16313	1.07580
H	3.16890	-0.54190	1.90789
H	3.17251	1.17285	1.48766
H	4.17129	0.03724	0.55988
Na	-0.03778	-0.00649	1.58380

ACE2_Na_PBE0_TZVP

33

O	-0.31710	-1.72328	-0.03492
O	-0.28794	1.32718	-0.56602
O	-2.33720	0.12285	0.77696
N	2.07345	-0.04907	0.19168
C	2.05003	-1.41675	-0.31531
C	2.01546	0.97296	-0.85647
C	0.74304	-1.80838	-0.96786
C	0.94621	2.00981	-0.58832
C	-1.59563	-1.73237	-0.63530
C	-1.41709	2.09427	-0.22381
C	-2.61608	-1.19843	0.34359
C	-2.59160	1.14831	-0.17271
H	2.23316	-2.08486	0.53126
H	2.85616	-1.60110	-1.04514
H	1.79741	0.50649	-1.82003
H	2.98409	1.47542	-0.96737
H	0.52281	-1.15681	-1.82167
H	0.83279	-2.83315	-1.35062
H	1.11928	2.51704	0.37229

H	0.95551	2.77375	-1.37698
H	-1.88340	-2.75090	-0.92880
H	-1.57733	-1.11779	-1.54359
H	-1.61141	2.87966	-0.96611
H	-1.26949	2.58276	0.75022
H	-3.61242	-1.26053	-0.10893
H	-2.61988	-1.81084	1.24847
H	-3.49618	1.69633	0.11203
H	-2.75314	0.71938	-1.16574
C	3.20641	0.16229	1.07641
H	3.16686	-0.54230	1.90970
H	3.17252	1.17301	1.48771
H	4.17199	0.03485	0.56146
Na	-0.03727	-0.00556	1.58278

ACE2_Na_PBE0_TZV

33

O	-0.33047	-1.76349	-0.02502
O	-0.28004	1.34786	-0.55993
O	-2.33788	0.13544	0.80257
N	2.08759	-0.05721	0.20212
C	2.05996	-1.43990	-0.31049
C	2.04554	0.97610	-0.85546
C	0.75659	-1.82450	-0.98318
C	0.99329	2.03578	-0.58782
C	-1.64386	-1.75402	-0.63386
C	-1.43599	2.13863	-0.20714
C	-2.64438	-1.21711	0.36669
C	-2.60873	1.18706	-0.16807
H	2.23045	-2.10909	0.53617
H	2.87328	-1.62171	-1.03250

H	1.82077	0.51324	-1.81835
H	3.02284	1.46319	-0.96193
H	0.53063	-1.15205	-1.81630
H	0.84562	-2.84069	-1.38061
H	1.16857	2.53725	0.37222
H	1.00285	2.79117	-1.38064
H	-1.93689	-2.76756	-0.92774
H	-1.61983	-1.13044	-1.53326
H	-1.62081	2.92257	-0.94892
H	-1.28488	2.61452	0.76907
H	-3.64835	-1.26373	-0.06526
H	-2.63203	-1.81890	1.27520
H	-3.51530	1.72090	0.12592
H	-2.76423	0.75639	-1.15976
C	3.21065	0.15291	1.12628
H	3.14690	-0.55273	1.95576
H	3.16784	1.16239	1.53719
H	4.18659	0.02343	0.63317
Na	-0.03623	-0.01235	1.47119

ACE2_Na_PBE_SV(P)

33

O	-0.31846	-1.72361	-0.03344
O	-0.28325	1.35031	-0.54032
O	-2.34597	0.12957	0.77469
N	2.09436	-0.05451	0.18475
C	2.07074	-1.43921	-0.30837
C	2.03030	0.97240	-0.87374
C	0.75328	-1.85403	-0.96122
C	0.96192	2.03085	-0.60018
C	-1.61235	-1.77459	-0.62043

C	-1.42925	2.12420	-0.22859
C	-2.63532	-1.20757	0.36204
C	-2.60971	1.16131	-0.18240
H	2.25502	-2.10498	0.56371
H	2.89295	-1.64435	-1.04552
H	1.78982	0.49391	-1.84722
H	3.01769	1.47593	-1.00871
H	0.54024	-1.23060	-1.86297
H	0.84688	-2.91248	-1.30845
H	1.16246	2.56617	0.36312
H	0.96295	2.79378	-1.41774
H	-1.89948	-2.82514	-0.87651
H	-1.61698	-1.18733	-1.57051
H	-1.61999	2.91200	-0.99885
H	-1.30184	2.63957	0.75768
H	-3.65389	-1.28052	-0.08746
H	-2.63286	-1.80950	1.29649
H	-3.53513	1.71542	0.10204
H	-2.76690	0.72632	-1.19481
C	3.20500	0.17997	1.10723
H	3.14624	-0.52724	1.96321
H	3.14493	1.21184	1.51600
H	4.20933	0.05826	0.61974
Na	-0.03893	0.00913	1.54274

ACE2_Na_PBE_SVP

33

O	-0.31790	-1.72730	-0.03179
O	-0.28414	1.34857	-0.53959
O	-2.34806	0.12862	0.77759
N	2.09512	-0.05375	0.18544

C	2.06982	-1.43797	-0.30952
C	2.02845	0.97265	-0.87305
C	0.75354	-1.84708	-0.96320
C	0.96169	2.03017	-0.59894
C	-1.61182	-1.76987	-0.62185
C	-1.43144	2.12213	-0.22745
C	-2.63458	-1.20880	0.36092
C	-2.60974	1.15976	-0.18208
H	2.25384	-2.10479	0.55910
H	2.89072	-1.64511	-1.04373
H	1.78485	0.49498	-1.84395
H	3.01320	1.47443	-1.01425
H	0.53890	-1.21475	-1.85603
H	0.84683	-2.89802	-1.32551
H	1.16221	2.56549	0.36196
H	0.96410	2.79440	-1.41202
H	-1.90221	-2.81412	-0.88950
H	-1.61275	-1.17749	-1.56655
H	-1.62358	2.91043	-0.99339
H	-1.30569	2.63830	0.75622
H	-3.65084	-1.28555	-0.08747
H	-2.63299	-1.81453	1.29063
H	-3.53546	1.71287	0.09456
H	-2.76353	0.72470	-1.19285
C	3.20868	0.17838	1.10505
H	3.15731	-0.53274	1.95421
H	3.14991	1.20389	1.52152
H	4.20928	0.06332	0.61615
Na	-0.03789	0.00775	1.54398

ACE2_Na_PBE_SV

33

O	-0.31849	-1.72309	-0.03370
O	-0.28319	1.35025	-0.54022
O	-2.34615	0.12967	0.77477
N	2.09453	-0.05470	0.18463
C	2.07062	-1.43917	-0.30917
C	2.03058	0.97281	-0.87323
C	0.75287	-1.85321	-0.96187
C	0.96193	2.03098	-0.59940
C	-1.61252	-1.77446	-0.62022
C	-1.42937	2.12405	-0.22878
C	-2.63533	-1.20761	0.36254
C	-2.60978	1.16110	-0.18266
H	2.25503	-2.10543	0.56250
H	2.89259	-1.64400	-1.04667
H	1.79033	0.49494	-1.84708
H	3.01797	1.47650	-1.00776
H	0.53980	-1.22911	-1.86315
H	0.84608	-2.91145	-1.30988
H	1.16213	2.56595	0.36416
H	0.96303	2.79426	-1.41664
H	-1.89950	-2.82510	-0.87612
H	-1.61759	-1.18737	-1.57041
H	-1.61995	2.91178	-0.99916
H	-1.30218	2.63959	0.75744
H	-3.65405	-1.28085	-0.08657
H	-2.63237	-1.80942	1.29707
H	-3.53543	1.71511	0.10130
H	-2.76651	0.72591	-1.19505
C	3.20516	0.17907	1.10731

H	3.14629	-0.52864	1.96286
H	3.14518	1.21071	1.51669
H	4.20949	0.05758	0.61979
Na	-0.03859	0.00872	1.54276

ACE2_Na_PBE_TZVPP

33

O	-0.31706	-1.74463	-0.03249
O	-0.29615	1.33534	-0.58541
O	-2.37005	0.12091	0.79161
N	2.09315	-0.04452	0.19550
C	2.06965	-1.42384	-0.31867
C	2.02938	0.98686	-0.86192
C	0.75706	-1.81999	-0.97784
C	0.95257	2.02841	-0.59071
C	-1.60720	-1.74063	-0.64955
C	-1.43471	2.10982	-0.22062
C	-2.63877	-1.21452	0.33550
C	-2.61768	1.16065	-0.17318
H	2.25606	-2.09447	0.53393
H	2.88132	-1.60962	-1.05368
H	1.80927	0.51542	-1.83062
H	3.00275	1.49492	-0.97595
H	0.53007	-1.15982	-1.83265
H	0.85127	-2.84899	-1.36845
H	1.11641	2.52833	0.38343
H	0.96706	2.80474	-1.37731
H	-1.89768	-2.76128	-0.96029
H	-1.57813	-1.11030	-1.55556
H	-1.63404	2.90959	-0.95733
H	-1.27621	2.59024	0.76381

H	-3.63932	-1.27897	-0.12468
H	-2.64330	-1.83333	1.24422
H	-3.52671	1.71484	0.11284
H	-2.78001	0.72683	-1.17139
C	3.24411	0.16711	1.07706
H	3.21328	-0.54816	1.91020
H	3.20839	1.18103	1.49728
H	4.21210	0.04622	0.54918
Na	-0.03501	-0.01561	1.61221

ACE2_Na_PBE_TZVP

33

O	-0.31728	-1.74439	-0.03163
O	-0.29568	1.33508	-0.58366
O	-2.37008	0.12142	0.79135
N	2.09340	-0.04494	0.19505
C	2.06942	-1.42442	-0.31884
C	2.02961	0.98659	-0.86236
C	0.75659	-1.82086	-0.97732
C	0.95306	2.02844	-0.59119
C	-1.60779	-1.74195	-0.64819
C	-1.43471	2.11022	-0.22087
C	-2.63920	-1.21446	0.33635
C	-2.61760	1.16093	-0.17396
H	2.25606	-2.09535	0.53443
H	2.88124	-1.61097	-1.05470
H	1.80902	0.51505	-1.83167
H	3.00361	1.49508	-0.97667
H	0.52949	-1.16143	-1.83359
H	0.85088	-2.85113	-1.36705
H	1.11823	2.53057	0.38249

H	0.96700	2.80394	-1.37980
H	-1.89847	-2.76414	-0.95687
H	-1.57962	-1.11318	-1.55619
H	-1.63380	2.90961	-0.95931
H	-1.27752	2.59222	0.76392
H	-3.64037	-1.27944	-0.12435
H	-2.64368	-1.83283	1.24633
H	-3.52739	1.71572	0.11183
H	-2.77938	0.72703	-1.17305
C	3.24365	0.16670	1.07766
H	3.21136	-0.54800	1.91216
H	3.20816	1.18166	1.49724
H	4.21295	0.04443	0.55093
Na	-0.03414	-0.01347	1.61022

ACE2_Na_PBE_TZV

33

O	-0.32672	-1.78819	-0.01875
O	-0.29102	1.35082	-0.58397
O	-2.37107	0.13358	0.82516
N	2.10626	-0.05235	0.20823
C	2.08214	-1.44542	-0.32034
C	2.06104	0.99614	-0.85642
C	0.77213	-1.83401	-0.99820
C	0.99780	2.05740	-0.58575
C	-1.65591	-1.76543	-0.64483
C	-1.45982	2.15311	-0.21001
C	-2.66558	-1.23805	0.36455
C	-2.63954	1.19701	-0.16941
H	2.25967	-2.12212	0.52904
H	2.90065	-1.62220	-1.05150

H	1.83879	0.53111	-1.82800
H	3.04361	1.49186	-0.95965
H	0.53470	-1.14969	-1.82931
H	0.86560	-2.85426	-1.40701
H	1.15773	2.54950	0.39109
H	1.01313	2.82801	-1.37547
H	-1.95153	-2.78265	-0.95559
H	-1.62271	-1.12517	-1.54220
H	-1.65076	2.94778	-0.95163
H	-1.29730	2.62673	0.77489
H	-3.67621	-1.28941	-0.07232
H	-2.64921	-1.84516	1.27908
H	-3.55177	1.73733	0.12646
H	-2.79770	0.75558	-1.16448
C	3.24895	0.15465	1.13136
H	3.19364	-0.56556	1.95881
H	3.20305	1.16670	1.55529
H	4.22899	0.03435	0.62480
Na	-0.02935	-0.01532	1.49045

ACE2_Na_SCS-MP2_SV(P)

33

O	-0.32921	-1.70854	-0.03535
O	-0.26967	1.33135	-0.54294
O	-2.31708	0.13845	0.77208
N	2.07003	-0.06301	0.19428
C	2.04669	-1.43549	-0.32217
C	2.03679	0.96484	-0.86066
C	0.72750	-1.81794	-0.97871
C	0.97048	2.01722	-0.58983
C	-1.61991	-1.75176	-0.62403

C	-1.40559	2.11549	-0.22901
C	-2.63156	-1.19046	0.36426
C	-2.58583	1.16154	-0.18553
H	2.21853	-2.11807	0.52974
H	2.86479	-1.61869	-1.05671
H	1.81263	0.50116	-1.83625
H	3.02455	1.45816	-0.96776
H	0.51330	-1.16602	-1.84637
H	0.80289	-2.85979	-1.35294
H	1.16213	2.53690	0.37285
H	0.96850	2.77908	-1.39697
H	-1.90188	-2.79325	-0.88446
H	-1.62246	-1.15718	-1.55736
H	-1.58261	2.89827	-0.99510
H	-1.27437	2.61762	0.75249
H	-3.64728	-1.23938	-0.07473
H	-2.62723	-1.79490	1.28712
H	-3.50778	1.70594	0.09674
H	-2.73498	0.72726	-1.18965
C	3.19875	0.14237	1.10257
H	3.12613	-0.55590	1.95482
H	3.16926	1.17147	1.50101
H	4.18120	-0.01094	0.60209
Na	-0.04835	0.00313	1.55363

ACE2_Na_SCS-MP2_SVP

33

O	-0.33144	-1.70384	-0.03861
O	-0.26815	1.32815	-0.54083
O	-2.32042	0.13941	0.77185
N	2.07300	-0.06451	0.19458

C	2.04219	-1.43240	-0.33021
C	2.03773	0.96683	-0.85508
C	0.72153	-1.80356	-0.98512
C	0.96997	2.01480	-0.58425
C	-1.62139	-1.74588	-0.62566
C	-1.40419	2.11131	-0.23137
C	-2.63284	-1.18738	0.36141
C	-2.58360	1.15965	-0.18776
H	2.21382	-2.11815	0.51345
H	2.85290	-1.61316	-1.06608
H	1.81698	0.50762	-1.82878
H	3.01898	1.46273	-0.95841
H	0.50730	-1.14137	-1.83967
H	0.79390	-2.83520	-1.37407
H	1.15884	2.53030	0.37642
H	0.97047	2.77671	-1.38472
H	-1.90472	-2.78117	-0.88887
H	-1.62413	-1.15175	-1.55432
H	-1.58111	2.88999	-0.99461
H	-1.27583	2.61388	0.74537
H	-3.64242	-1.23839	-0.07939
H	-2.62947	-1.79345	1.27810
H	-3.50157	1.70598	0.08488
H	-2.72780	0.72488	-1.18777
C	3.20862	0.13074	1.09332
H	3.14403	-0.57207	1.93542
H	3.18663	1.15118	1.49963
H	4.18110	-0.01938	0.58488
Na	-0.04881	-0.00382	1.57322

ACE2_Na_SCS-MP2_SV

33

O	-0.32921	-1.70854	-0.03535
O	-0.26967	1.33135	-0.54294
O	-2.31708	0.13845	0.77208
N	2.07003	-0.06301	0.19428
C	2.04669	-1.43549	-0.32217
C	2.03679	0.96484	-0.86066
C	0.72750	-1.81794	-0.97871
C	0.97048	2.01722	-0.58983
C	-1.61991	-1.75176	-0.62403
C	-1.40559	2.11549	-0.22901
C	-2.63156	-1.19046	0.36426
C	-2.58583	1.16154	-0.18553
H	2.21853	-2.11807	0.52974
H	2.86479	-1.61869	-1.05671
H	1.81263	0.50116	-1.83625
H	3.02455	1.45816	-0.96776
H	0.51330	-1.16602	-1.84637
H	0.80289	-2.85979	-1.35294
H	1.16213	2.53690	0.37285
H	0.96850	2.77908	-1.39697
H	-1.90188	-2.79325	-0.88446
H	-1.62246	-1.15718	-1.55736
H	-1.58261	2.89827	-0.99510
H	-1.27437	2.61762	0.75249
H	-3.64728	-1.23938	-0.07473
H	-2.62723	-1.79490	1.28712
H	-3.50778	1.70594	0.09674
H	-2.73498	0.72726	-1.18965
C	3.19875	0.14237	1.10257

H	3.12613	-0.55590	1.95482
H	3.16926	1.17147	1.50101
H	4.18120	-0.01094	0.60209
Na	-0.04835	0.00313	1.55363

ACE2_Na_SCS-MP2_TZVP

33

O	-0.31784	-1.71606	-0.02570
O	-0.28526	1.29656	-0.55895
O	-2.34052	0.12665	0.79142
N	2.06293	-0.04811	0.20787
C	2.05167	-1.41860	-0.31501
C	2.02780	0.97314	-0.85506
C	0.74050	-1.79444	-0.97778
C	0.94835	2.00537	-0.59117
C	-1.60097	-1.71985	-0.64248
C	-1.41613	2.08933	-0.22901
C	-2.62441	-1.20163	0.34759
C	-2.59946	1.14853	-0.17680
H	2.22561	-2.09444	0.52666
H	2.86242	-1.58564	-1.04256
H	1.82519	0.50495	-1.82005
H	2.99768	1.47623	-0.94375
H	0.51751	-1.12644	-1.81500
H	0.81715	-2.81513	-1.37093
H	1.11648	2.51507	0.36708
H	0.93688	2.76112	-1.38573
H	-1.87874	-2.73594	-0.94875
H	-1.57534	-1.08913	-1.53644
H	-1.59144	2.86483	-0.98396
H	-1.26693	2.58013	0.74144

H	-3.62189	-1.25472	-0.10098
H	-2.61781	-1.81839	1.24781
H	-3.50220	1.69667	0.10795
H	-2.75836	0.70937	-1.16380
C	3.22396	0.15266	1.07698
H	3.18134	-0.54434	1.91536
H	3.20962	1.16885	1.47402
H	4.17331	0.00188	0.54212
Na	-0.04090	0.00524	1.56879

ACE2_Na_SCS-MP2_TZV

33

O	-0.36246	1.78042	0.04142
O	-0.25281	-1.32980	0.55931
O	-2.35402	-0.16684	-0.82154
N	2.10428	0.07986	-0.22006
C	2.04355	1.45033	0.37575
C	2.11438	-0.99875	0.82351
C	0.70778	1.75201	1.05838
C	1.03320	-2.04984	0.55638
C	-1.70794	1.75300	0.64217
C	-1.42039	-2.16068	0.22676
C	-2.69376	1.20677	-0.39108
C	-2.61907	-1.21798	0.18676
H	2.20276	2.17186	-0.43295
H	2.84641	1.59503	1.12236
H	1.94394	-0.56771	1.81631
H	3.09554	-1.49636	0.85050
H	0.45887	0.98837	1.80276
H	0.76296	2.72947	1.55413
H	1.18007	-2.52676	-0.42282

H	1.03435	-2.81844	1.33970
H	-2.00832	2.76647	0.93655
H	-1.68352	1.11583	1.53325
H	-1.57452	-2.93171	0.99162
H	-1.26631	-2.64209	-0.74786
H	-3.70801	1.22647	0.02510
H	-2.66686	1.79950	-1.30734
H	-3.52244	-1.76342	-0.10397
H	-2.77308	-0.76839	1.17276
C	3.27959	-0.04128	-1.13476
H	3.17993	0.68085	-1.94946
H	3.29904	-1.04835	-1.55997
H	4.23104	0.14036	-0.60695
Na	-0.03923	0.03258	-1.52454

ACE2_nm_B2PLYP_SV(P)

32

O	-0.30905	-1.72344	0.12224
O	-0.31631	1.35429	-0.58176
O	-2.39264	0.10304	0.96961
N	2.09070	-0.04909	0.34166
C	2.06362	-1.42832	-0.11412
C	2.03217	0.96190	-0.70913
C	0.76004	-1.82919	-0.78665
C	0.93684	1.98693	-0.45639
C	-1.57219	-1.69625	-0.49286
C	-1.38948	2.05534	-0.00506
C	-2.61512	-1.20867	0.49765
C	-2.60740	1.15568	0.04878
H	2.20394	-2.07609	0.76765
H	2.89631	-1.66559	-0.81896

H	1.84032	0.49110	-1.68677
H	3.00102	1.49271	-0.80985
H	0.56809	-1.18983	-1.66806
H	0.86001	-2.87043	-1.16003
H	1.05404	2.40708	0.56253
H	1.02549	2.82873	-1.17561
H	-1.86985	-2.70555	-0.85071
H	-1.54888	-1.03010	-1.37563
H	-1.63258	2.97108	-0.58465
H	-1.13470	2.37165	1.02645
H	-3.61726	-1.30383	0.03338
H	-2.59509	-1.85489	1.38994
H	-3.47888	1.75577	0.37401
H	-2.82712	0.77266	-0.96309
C	3.14086	0.20766	1.30579
H	3.04627	-0.47995	2.16288
H	3.05476	1.23685	1.69251
H	4.16717	0.09078	0.88367

ACE2_nm_B2PLYP_SVP

32

O	-0.30528	-1.72879	0.12574
O	-0.32220	1.35004	-0.58695
O	-2.40163	0.09622	0.97375
N	2.09097	-0.04408	0.34387
C	2.06716	-1.42522	-0.10535
C	2.02654	0.96011	-0.71235
C	0.76798	-1.82946	-0.77947
C	0.93131	1.98363	-0.46432
C	-1.56379	-1.68582	-0.49880
C	-1.39149	2.04820	0.00109

C	-2.61369	-1.21233	0.48763
C	-2.61045	1.15342	0.05647
H	2.20796	-2.06768	0.77724
H	2.89979	-1.66509	-0.80514
H	1.83195	0.48428	-1.68440
H	2.99102	1.49149	-0.82206
H	0.57723	-1.19049	-1.65866
H	0.87267	-2.86587	-1.15716
H	1.04788	2.40871	0.54998
H	1.02086	2.82197	-1.18339
H	-1.86318	-2.68390	-0.87784
H	-1.53007	-1.00616	-1.36804
H	-1.63732	2.96701	-0.56744
H	-1.13054	2.35869	1.03030
H	-3.60870	-1.30933	0.01454
H	-2.59993	-1.86823	1.37006
H	-3.47668	1.75681	0.38074
H	-2.83340	0.77611	-0.95439
C	3.14382	0.21954	1.30240
H	3.05814	-0.46405	2.15922
H	3.05818	1.24587	1.68706
H	4.16583	0.10620	0.87816

ACE2_nm_B2PLYP_SV

32

O	-0.30905	-1.72344	0.12224
O	-0.31631	1.35429	-0.58176
O	-2.39264	0.10304	0.96961
N	2.09070	-0.04909	0.34166
C	2.06362	-1.42832	-0.11412
C	2.03217	0.96190	-0.70913

C	0.76004	-1.82919	-0.78665
C	0.93684	1.98693	-0.45639
C	-1.57219	-1.69625	-0.49286
C	-1.38948	2.05534	-0.00506
C	-2.61512	-1.20867	0.49765
C	-2.60740	1.15568	0.04878
H	2.20394	-2.07609	0.76765
H	2.89631	-1.66559	-0.81896
H	1.84032	0.49110	-1.68677
H	3.00102	1.49271	-0.80985
H	0.56809	-1.18983	-1.66806
H	0.86001	-2.87043	-1.16003
H	1.05404	2.40708	0.56253
H	1.02549	2.82873	-1.17561
H	-1.86985	-2.70555	-0.85071
H	-1.54888	-1.03010	-1.37563
H	-1.63258	2.97108	-0.58465
H	-1.13470	2.37165	1.02645
H	-3.61726	-1.30383	0.03338
H	-2.59509	-1.85489	1.38994
H	-3.47888	1.75577	0.37401
H	-2.82712	0.77266	-0.96309
C	3.14086	0.20766	1.30579
H	3.04627	-0.47995	2.16288
H	3.05476	1.23685	1.69251
H	4.16717	0.09078	0.88367

ACE2_nm_B2PLYP_TZVPP

32

O	-0.30606	1.81849	-0.11807
O	-0.32638	-1.42124	0.64089

O	-2.33581	-0.09851	-0.98429
N	2.04492	0.04942	-0.35966
C	2.05525	1.42161	0.12706
C	2.01017	-0.96832	0.69113
C	0.76611	1.83333	0.80931
C	0.94444	-2.01663	0.43489
C	-1.57549	1.71087	0.49774
C	-1.39991	-2.08322	0.00152
C	-2.59156	1.21500	-0.50792
C	-2.58541	-1.14992	-0.05759
H	2.21768	2.07582	-0.72950
H	2.88205	1.60886	0.83162
H	1.80590	-0.50780	1.65649
H	2.98190	-1.46623	0.78536
H	0.53966	1.15690	1.63575
H	0.89317	2.83620	1.23126
H	1.02361	-2.38207	-0.59300
H	1.07942	-2.86989	1.10868
H	-1.90759	2.68225	0.88282
H	-1.51637	1.02069	1.34224
H	-1.67942	-2.99394	0.54312
H	-1.11519	-2.36900	-1.01545
H	-3.58766	1.27775	-0.05959
H	-2.57721	1.85572	-1.38755
H	-3.46493	-1.71098	-0.38252
H	-2.78888	-0.75516	0.93810
C	3.11782	-0.19592	-1.30823
H	3.04352	0.50052	-2.14116
H	3.03612	-1.20579	-1.70596
H	4.11720	-0.09040	-0.85884

ACE2_nm_B2PLYP_TZVP

32

O	-0.30780	-1.81461	0.11857
O	-0.32476	1.41883	-0.63706
O	-2.33726	0.10019	0.98329
N	2.04746	-0.05046	0.35940
C	2.05407	-1.42210	-0.12932
C	2.01263	0.96911	-0.68988
C	0.76337	-1.83143	-0.81018
C	0.94563	2.01620	-0.43362
C	-1.57836	-1.71209	-0.49614
C	-1.39937	2.08363	-0.00214
C	-2.59422	-1.21393	0.50880
C	-2.58594	1.15150	0.05592
H	2.21714	-2.07908	0.72721
H	2.88086	-1.61013	-0.83662
H	1.81099	0.50966	-1.65823
H	2.98545	1.46934	-0.78158
H	0.53664	-1.15469	-1.63845
H	0.88851	-2.83670	-1.23224
H	1.02631	2.38448	0.59503
H	1.07869	2.87008	-1.11023
H	-1.90948	-2.68772	-0.87705
H	-1.52198	-1.02509	-1.34559
H	-1.67669	2.99499	-0.54770
H	-1.11707	2.37227	1.01666
H	-3.59196	-1.27684	0.05962
H	-2.58044	-1.85391	1.39128
H	-3.46655	1.71432	0.38138
H	-2.79018	0.75663	-0.94149

C	3.12248	0.19107	1.30677
H	3.04608	-0.50501	2.14199
H	3.04553	1.20316	1.70431
H	4.12270	0.08059	0.85622

ACE2_nm_B2PLYP_TZV

32

O	-0.32273	-1.90540	0.10748
O	-0.32596	1.46274	-0.68879
O	-2.37255	0.11096	1.01381
N	2.07660	-0.05626	0.36654
C	2.07107	-1.44666	-0.12728
C	2.04801	0.97586	-0.69496
C	0.78734	-1.84497	-0.84270
C	0.99399	2.04650	-0.44031
C	-1.62904	-1.72337	-0.51658
C	-1.42010	2.12548	0.01462
C	-2.62757	-1.24413	0.52171
C	-2.61437	1.19453	0.05394
H	2.21164	-2.10521	0.73133
H	2.90891	-1.63508	-0.82300
H	1.83807	0.51786	-1.66245
H	3.02831	1.46506	-0.78397
H	0.54417	-1.12609	-1.62709
H	0.92592	-2.82642	-1.30757
H	1.05315	2.38828	0.59694
H	1.14581	2.90348	-1.10435
H	-1.98124	-2.67218	-0.93598
H	-1.54274	-0.99730	-1.32769
H	-1.69275	3.05328	-0.49891
H	-1.11175	2.36625	1.03594

H	-3.63431	-1.30262	0.09593
H	-2.58299	-1.87646	1.40575
H	-3.49581	1.74963	0.38189
H	-2.80742	0.79480	-0.94302
C	3.14961	0.18356	1.34891
H	3.05262	-0.51615	2.17830
H	3.06469	1.19466	1.74635
H	4.15589	0.07073	0.91170

ACE2_nm_B3LYP_SV(P)

32

O	-0.30984	-1.73503	0.11856
O	-0.31611	1.37962	-0.58579
O	-2.40767	0.10492	0.96344
N	2.10924	-0.05169	0.33235
C	2.07132	-1.43532	-0.11510
C	2.03749	0.96672	-0.71236
C	0.76409	-1.84621	-0.78506
C	0.94311	1.99881	-0.45223
C	-1.57821	-1.71549	-0.48702
C	-1.39468	2.06761	-0.00296
C	-2.62236	-1.21236	0.50176
C	-2.61392	1.16147	0.04390
H	2.21457	-2.07789	0.77178
H	2.90177	-1.68478	-0.82136
H	1.83852	0.49878	-1.69159
H	3.00581	1.49995	-0.82121
H	0.57246	-1.21830	-1.67731
H	0.87013	-2.89245	-1.14804
H	1.06091	2.41443	0.57051
H	1.04263	2.84634	-1.16617

H	-1.88070	-2.73128	-0.82817
H	-1.56130	-1.06466	-1.38389
H	-1.64489	2.98882	-0.57451
H	-1.14169	2.37927	1.03263
H	-3.62541	-1.31876	0.03740
H	-2.60258	-1.84973	1.40285
H	-3.48743	1.76326	0.36671
H	-2.83104	0.78229	-0.97198
C	3.14342	0.20995	1.31317
H	3.04515	-0.48274	2.16811
H	3.04568	1.23755	1.70645
H	4.18020	0.10456	0.90712

ACE2_nm_B3LYP_SVP

32

O	-0.30757	-1.74388	0.12104
O	-0.32003	1.38089	-0.59304
O	-2.41085	0.10058	0.96720
N	2.10799	-0.04915	0.33422
C	2.07273	-1.43353	-0.11174
C	2.03343	0.96627	-0.71298
C	0.76869	-1.84463	-0.78287
C	0.94093	1.99830	-0.45536
C	-1.57392	-1.70932	-0.49064
C	-1.39635	2.06442	0.00140
C	-2.62068	-1.21523	0.49648
C	-2.61436	1.16007	0.04913
H	2.21752	-2.07480	0.77365
H	2.90301	-1.68362	-0.81483
H	1.83145	0.49702	-1.68910
H	2.99944	1.49835	-0.82891

H	0.57596	-1.21200	-1.66954
H	0.87859	-2.88466	-1.15647
H	1.05625	2.41451	0.56544
H	1.04491	2.84616	-1.16523
H	-1.88067	-2.71572	-0.84901
H	-1.54899	-1.04817	-1.37774
H	-1.65085	2.98895	-0.55914
H	-1.13807	2.37108	1.03532
H	-3.61964	-1.32301	0.02797
H	-2.60539	-1.85995	1.39039
H	-3.48591	1.76277	0.36938
H	-2.83249	0.78470	-0.96618
C	3.14426	0.21482	1.31238
H	3.05375	-0.47835	2.16420
H	3.04652	1.23822	1.70891
H	4.17790	0.11485	0.90487

ACE2_nm_B3LYP_SV

32

O	-0.30984	-1.73503	0.11856
O	-0.31611	1.37962	-0.58579
O	-2.40767	0.10492	0.96344
N	2.10924	-0.05169	0.33235
C	2.07132	-1.43532	-0.11510
C	2.03749	0.96672	-0.71236
C	0.76409	-1.84621	-0.78506
C	0.94311	1.99881	-0.45223
C	-1.57821	-1.71549	-0.48702
C	-1.39468	2.06761	-0.00296
C	-2.62236	-1.21236	0.50176
C	-2.61392	1.16147	0.04390

H	2.21457	-2.07789	0.77178
H	2.90177	-1.68478	-0.82136
H	1.83852	0.49878	-1.69159
H	3.00581	1.49995	-0.82121
H	0.57246	-1.21830	-1.67731
H	0.87013	-2.89245	-1.14804
H	1.06091	2.41443	0.57051
H	1.04263	2.84634	-1.16617
H	-1.88070	-2.73128	-0.82817
H	-1.56130	-1.06466	-1.38389
H	-1.64489	2.98882	-0.57451
H	-1.14169	2.37927	1.03263
H	-3.62541	-1.31876	0.03740
H	-2.60258	-1.84973	1.40285
H	-3.48743	1.76326	0.36671
H	-2.83104	0.78229	-0.97198
C	3.14342	0.20995	1.31317
H	3.04515	-0.48274	2.16811
H	3.04568	1.23755	1.70645
H	4.18020	0.10456	0.90712

ACE2_nm_B3LYP_TZVPP

32

O	-0.30584	-1.83691	0.11207
O	-0.32650	1.45523	-0.64810
O	-2.33615	0.10053	0.97717
N	2.05967	-0.05312	0.34886
C	2.06223	-1.42832	-0.13448
C	2.01523	0.97561	-0.69232
C	0.76996	-1.85137	-0.81313
C	0.95189	2.03179	-0.42950

C	-1.58374	-1.73724	-0.48982
C	-1.40744	2.10078	-0.00257
C	-2.59373	-1.21969	0.51724
C	-2.58865	1.15561	0.05331
H	2.22960	-2.08078	0.72481
H	2.88750	-1.62387	-0.84227
H	1.80652	0.52157	-1.66183
H	2.98826	1.47518	-0.79176
H	0.54079	-1.18425	-1.64916
H	0.90486	-2.85885	-1.22805
H	1.02985	2.39183	0.60257
H	1.10173	2.89139	-1.09613
H	-1.92374	-2.71711	-0.85314
H	-1.53472	-1.06540	-1.35232
H	-1.69864	3.01407	-0.53880
H	-1.12462	2.38729	1.01721
H	-3.59480	-1.29012	0.07544
H	-2.57737	-1.84905	1.40805
H	-3.47388	1.71214	0.37943
H	-2.79132	0.76418	-0.94620
C	3.10831	0.19536	1.32408
H	3.02410	-0.50948	2.15185
H	3.00854	1.20172	1.73234
H	4.12321	0.10540	0.89995

ACE2_nm_B3LYP_TZVP

32

O	-0.30616	-1.83498	0.11264
O	-0.32638	1.45355	-0.64665
O	-2.33723	0.10062	0.97693
N	2.06079	-0.05306	0.34885

C	2.06209	-1.42817	-0.13492
C	2.01586	0.97607	-0.69203
C	0.76942	-1.85078	-0.81289
C	0.95174	2.03146	-0.42956
C	-1.58414	-1.73724	-0.48958
C	-1.40748	2.10032	-0.00242
C	-2.59475	-1.21964	0.51677
C	-2.58935	1.15605	0.05319
H	2.22956	-2.08137	0.72481
H	2.88738	-1.62417	-0.84394
H	1.80786	0.52202	-1.66259
H	2.98916	1.47711	-0.79063
H	0.54047	-1.18390	-1.65024
H	0.90401	-2.85963	-1.22707
H	1.03053	2.39324	0.60273
H	1.10070	2.89081	-1.09825
H	-1.92355	-2.71879	-0.85182
H	-1.53591	-1.06586	-1.35353
H	-1.69809	3.01404	-0.54013
H	-1.12548	2.38749	1.01834
H	-3.59616	-1.29017	0.07361
H	-2.57856	-1.84931	1.40843
H	-3.47446	1.71392	0.38039
H	-2.79286	0.76511	-0.94726
C	3.11062	0.19426	1.32319
H	3.02538	-0.51028	2.15218
H	3.01274	1.20185	1.73095
H	4.12585	0.10187	0.89818

ACE2_nm_B3LYP_TZV

32

O	-0.29295	-1.98783	0.09480
O	-0.34269	1.54676	-0.73124
O	-2.29341	0.09320	0.99935
N	2.02392	-0.04264	0.35298
C	2.08562	-1.44166	-0.10476
C	2.01165	0.97454	-0.71760
C	0.83459	-1.92306	-0.82997
C	0.99125	2.08181	-0.47256
C	-1.59501	-1.76784	-0.52083
C	-1.42992	2.15624	0.01950
C	-2.57615	-1.25733	0.52036
C	-2.59218	1.18570	0.07037
H	2.23681	-2.07174	0.77323
H	2.94559	-1.61971	-0.77586
H	1.77338	0.50671	-1.67298
H	3.00531	1.43368	-0.83291
H	0.58588	-1.25532	-1.65748
H	1.02526	-2.91776	-1.24593
H	1.05571	2.43320	0.56110
H	1.18187	2.92933	-1.13910
H	-1.97827	-2.70641	-0.93700
H	-1.49615	-1.04700	-1.33582
H	-1.75082	3.08668	-0.46158
H	-1.10142	2.38865	1.03686
H	-3.58839	-1.30617	0.10572
H	-2.53700	-1.88690	1.40759
H	-3.48397	1.70718	0.42705
H	-2.79584	0.80103	-0.93077
C	3.00508	0.25655	1.40661
H	2.88240	-0.43844	2.23714

H	2.84732	1.26501	1.78936
H	4.04779	0.18975	1.05049

ACE2_nm_B97-1_SV(P)

32

O	-0.30546	-1.72927	0.12201
O	-0.32054	1.36942	-0.58392
O	-2.41590	0.09946	0.96656
N	2.10846	-0.04680	0.33490
C	2.07785	-1.43352	-0.10721
C	2.03444	0.96539	-0.71743
C	0.76883	-1.84976	-0.77726
C	0.93417	1.99578	-0.45910
C	-1.56793	-1.70780	-0.49232
C	-1.39547	2.06094	-0.00268
C	-2.62279	-1.21364	0.49475
C	-2.61976	1.15633	0.04935
H	2.22243	-2.07197	0.78462
H	2.91024	-1.68312	-0.81335
H	1.83486	0.49033	-1.69516
H	3.00238	1.50180	-0.82979
H	0.58113	-1.22769	-1.67712
H	0.87501	-2.90063	-1.13170
H	1.05528	2.41772	0.56287
H	1.02717	2.84085	-1.17929
H	-1.86658	-2.72291	-0.84374
H	-1.54641	-1.04770	-1.38496
H	-1.64597	2.98174	-0.57793
H	-1.14039	2.37631	1.03326
H	-3.62315	-1.32001	0.02072
H	-2.60771	-1.85865	1.39232

H	-3.49019	1.76274	0.37666
H	-2.84295	0.77831	-0.96770
C	3.14816	0.22378	1.30937
H	3.05514	-0.46620	2.16903
H	3.04648	1.25469	1.69756
H	4.18412	0.12004	0.89741

ACE2_nm_B97-1_SVP

32

O	-0.30558	-1.73862	0.12341
O	-0.32194	1.37275	-0.59038
O	-2.41682	0.09808	0.96955
N	2.10780	-0.04657	0.33632
C	2.07656	-1.43299	-0.10728
C	2.03244	0.96516	-0.71623
C	0.76949	-1.84557	-0.77853
C	0.93483	1.99608	-0.45875
C	-1.56770	-1.70426	-0.49355
C	-1.39560	2.06012	0.00056
C	-2.62220	-1.21444	0.49274
C	-2.61808	1.15635	0.05200
H	2.22193	-2.07240	0.78129
H	2.90780	-1.68339	-0.81102
H	1.83019	0.49115	-1.69190
H	2.99845	1.49944	-0.83394
H	0.57942	-1.21455	-1.66943
H	0.87827	-2.88852	-1.14844
H	1.05291	2.41568	0.56238
H	1.03365	2.84333	-1.17223
H	-1.87210	-2.71101	-0.85703
H	-1.54029	-1.03829	-1.37938

H	-1.64956	2.98384	-0.56428
H	-1.13675	2.37093	1.03485
H	-3.61958	-1.32197	0.01750
H	-2.61061	-1.86408	1.38474
H	-3.48807	1.76270	0.37371
H	-2.83942	0.78050	-0.96428
C	3.14992	0.22199	1.30905
H	3.06252	-0.46944	2.16431
H	3.05083	1.24812	1.70214
H	4.18252	0.12158	0.89613

ACE2_nm_B97-1_SV

32

O	-0.30546	-1.72927	0.12201
O	-0.32054	1.36942	-0.58392
O	-2.41590	0.09946	0.96656
N	2.10846	-0.04680	0.33490
C	2.07785	-1.43352	-0.10721
C	2.03444	0.96539	-0.71743
C	0.76883	-1.84976	-0.77726
C	0.93417	1.99578	-0.45910
C	-1.56793	-1.70780	-0.49232
C	-1.39547	2.06094	-0.00268
C	-2.62279	-1.21364	0.49475
C	-2.61976	1.15633	0.04935
H	2.22243	-2.07197	0.78462
H	2.91024	-1.68312	-0.81335
H	1.83486	0.49033	-1.69516
H	3.00238	1.50180	-0.82979
H	0.58113	-1.22769	-1.67712
H	0.87501	-2.90063	-1.13170

H	1.05528	2.41772	0.56287
H	1.02717	2.84085	-1.17929
H	-1.86658	-2.72291	-0.84374
H	-1.54641	-1.04770	-1.38496
H	-1.64597	2.98174	-0.57793
H	-1.14039	2.37631	1.03326
H	-3.62315	-1.32001	0.02072
H	-2.60771	-1.85865	1.39232
H	-3.49019	1.76274	0.37666
H	-2.84295	0.77831	-0.96770
C	3.14816	0.22378	1.30937
H	3.05514	-0.46620	2.16903
H	3.04648	1.25469	1.69756
H	4.18412	0.12004	0.89741

ACE2_nm_B97-1_TZVPP

32

O	-0.30304	-1.83172	0.11538
O	-0.33060	1.44640	-0.64825
O	-2.34233	0.09624	0.98111
N	2.05603	-0.04887	0.35425
C	2.06673	-1.42640	-0.12683
C	2.01241	0.97350	-0.69575
C	0.77175	-1.85323	-0.80712
C	0.94307	2.02966	-0.43490
C	-1.57437	-1.73035	-0.49497
C	-1.40646	2.09483	-0.00194
C	-2.59500	-1.21900	0.51155
C	-2.59348	1.14977	0.05868
H	2.23475	-2.07740	0.73652
H	2.89449	-1.62040	-0.83527

H	1.80287	0.51219	-1.66446
H	2.98627	1.47552	-0.79747
H	0.54606	-1.18795	-1.64938
H	0.90777	-2.86482	-1.21796
H	1.02387	2.39315	0.59871
H	1.08932	2.88952	-1.10581
H	-1.91270	-2.70978	-0.86799
H	-1.52107	-1.04982	-1.35414
H	-1.69892	3.01012	-0.53885
H	-1.12095	2.38182	1.01977
H	-3.59439	-1.28906	0.06014
H	-2.58332	-1.85697	1.39913
H	-3.47707	1.71189	0.38715
H	-2.80035	0.75879	-0.94301
C	3.11700	0.20539	1.31713
H	3.04093	-0.49673	2.15086
H	3.01947	1.21636	1.72064
H	4.12853	0.11318	0.88063

ACE2_nm_B97-1_TZVP

32

O	-0.30305	-1.83064	0.11584
O	-0.33067	1.44537	-0.64726
O	-2.34278	0.09612	0.98089
N	2.05657	-0.04864	0.35425
C	2.06686	-1.42614	-0.12715
C	2.01279	0.97402	-0.69561
C	0.77174	-1.85349	-0.80677
C	0.94286	2.02969	-0.43541
C	-1.57445	-1.73050	-0.49486
C	-1.40668	2.09460	-0.00175

C	-2.59557	-1.21912	0.51109
C	-2.59407	1.15004	0.05875
H	2.23538	-2.07764	0.73670
H	2.89481	-1.62030	-0.83665
H	1.80390	0.51260	-1.66525
H	2.98703	1.47722	-0.79661
H	0.54617	-1.18919	-1.65084
H	0.90801	-2.86657	-1.21635
H	1.02449	2.39529	0.59825
H	1.08854	2.88914	-1.10847
H	-1.91256	-2.71130	-0.86726
H	-1.52175	-1.05014	-1.35522
H	-1.69901	3.01035	-0.53981
H	-1.12180	2.38225	1.02085
H	-3.59534	-1.28922	0.05846
H	-2.58419	-1.85747	1.39944
H	-3.47770	1.71315	0.38843
H	-2.80183	0.75961	-0.94387
C	3.11803	0.20500	1.31687
H	3.04068	-0.49654	2.15197
H	3.02192	1.21722	1.71957
H	4.13021	0.11055	0.88029

ACE2_nm_B97-1_TZV

32

O	-0.29802	-1.98121	0.09682
O	-0.34030	1.54036	-0.73138
O	-2.29958	0.09475	1.00369
N	2.02126	-0.04439	0.35847
C	2.08325	-1.44425	-0.10138
C	2.01409	0.96934	-0.71790

C	0.82639	-1.92044	-0.82801
C	0.99013	2.07864	-0.47427
C	-1.59477	-1.75972	-0.52400
C	-1.42182	2.15333	0.02053
C	-2.58289	-1.25038	0.51761
C	-2.59172	1.18497	0.07368
H	2.23319	-2.07693	0.77826
H	2.94384	-1.62243	-0.77546
H	1.77540	0.49785	-1.67453
H	3.01021	1.42883	-0.83218
H	0.58143	-1.24645	-1.65569
H	1.01442	-2.91674	-1.24826
H	1.05688	2.43053	0.56226
H	1.18099	2.92777	-1.14291
H	-1.97938	-2.69822	-0.94585
H	-1.49330	-1.03311	-1.33776
H	-1.74218	3.08749	-0.45949
H	-1.09123	2.38328	1.04079
H	-3.59481	-1.29654	0.09495
H	-2.54899	-1.88716	1.40303
H	-3.48241	1.71450	0.42893
H	-2.79665	0.79975	-0.93010
C	3.01966	0.25251	1.39873
H	2.90281	-0.44061	2.23478
H	2.87137	1.26582	1.77954
H	4.05885	0.17787	1.02745

ACE2_nm_CAM-B3LYP_SV(P)

32

O	-0.30556	-1.71819	0.11819
O	-0.31748	1.35329	-0.57707

O	-2.39556	0.10098	0.96302
N	2.09933	-0.04845	0.33247
C	2.06409	-1.42646	-0.11229
C	2.02830	0.96182	-0.71079
C	0.76188	-1.82827	-0.78202
C	0.93099	1.98142	-0.45604
C	-1.56521	-1.69377	-0.48929
C	-1.38949	2.04847	-0.00692
C	-2.60796	-1.20844	0.49909
C	-2.60635	1.15093	0.04871
H	2.20240	-2.06925	0.77327
H	2.89570	-1.67234	-0.81447
H	1.83629	0.49114	-1.68833
H	2.99346	1.49772	-0.81472
H	0.57236	-1.19271	-1.66723
H	0.86133	-2.87073	-1.15162
H	1.04956	2.40283	0.56255
H	1.01771	2.82397	-1.17436
H	-1.86151	-2.70349	-0.84652
H	-1.54492	-1.02838	-1.37327
H	-1.63312	2.96307	-0.58785
H	-1.13650	2.36809	1.02431
H	-3.60985	-1.31250	0.03617
H	-2.58398	-1.85310	1.39273
H	-3.47577	1.75381	0.37403
H	-2.82880	0.76934	-0.96348
C	3.12933	0.21526	1.30871
H	3.03184	-0.47417	2.16431
H	3.03131	1.24259	1.69839
H	4.16244	0.10883	0.90058

ACE2_nm_CAM-B3LYP_SVP

32

O	-0.30281	-1.72525	0.12125
O	-0.32244	1.35130	-0.58238
O	-2.40198	0.09552	0.96660
N	2.09910	-0.04432	0.33437
C	2.06648	-1.42408	-0.10521
C	2.02346	0.96052	-0.71347
C	0.76848	-1.82866	-0.77660
C	0.92715	1.97938	-0.46243
C	-1.55912	-1.68556	-0.49423
C	-1.39135	2.04354	-0.00114
C	-2.60675	-1.21169	0.49103
C	-2.60843	1.14975	0.05524
H	2.20515	-2.06339	0.78097
H	2.89906	-1.67275	-0.80267
H	1.82866	0.48612	-1.68706
H	2.98581	1.49635	-0.82610
H	0.57908	-1.19226	-1.65955
H	0.87268	-2.86679	-1.15223
H	1.04523	2.40490	0.55286
H	1.01568	2.82034	-1.17989
H	-1.85848	-2.68553	-0.87077
H	-1.52936	-1.00798	-1.36695
H	-1.63784	2.96216	-0.57147
H	-1.13271	2.35793	1.02878
H	-3.60374	-1.31745	0.02163
H	-2.58830	-1.86515	1.37668
H	-3.47469	1.75486	0.37952
H	-2.83364	0.77334	-0.95683

C	3.13206	0.22435	1.30611
H	3.04277	-0.46269	2.16133
H	3.03499	1.24913	1.69573
H	4.16194	0.12121	0.89581

ACE2_nm_CAM-B3LYP_SV

32

O	-0.30556	-1.71819	0.11819
O	-0.31748	1.35329	-0.57707
O	-2.39556	0.10098	0.96302
N	2.09933	-0.04845	0.33247
C	2.06409	-1.42646	-0.11229
C	2.02830	0.96182	-0.71079
C	0.76188	-1.82827	-0.78202
C	0.93099	1.98142	-0.45604
C	-1.56521	-1.69377	-0.48929
C	-1.38949	2.04847	-0.00692
C	-2.60796	-1.20844	0.49909
C	-2.60635	1.15093	0.04871
H	2.20240	-2.06925	0.77327
H	2.89570	-1.67234	-0.81447
H	1.83629	0.49114	-1.68833
H	2.99346	1.49772	-0.81472
H	0.57236	-1.19271	-1.66723
H	0.86133	-2.87073	-1.15162
H	1.04956	2.40283	0.56255
H	1.01771	2.82397	-1.17436
H	-1.86151	-2.70349	-0.84652
H	-1.54492	-1.02838	-1.37327
H	-1.63312	2.96307	-0.58785
H	-1.13650	2.36809	1.02431

H	-3.60985	-1.31250	0.03617
H	-2.58398	-1.85310	1.39273
H	-3.47577	1.75381	0.37403
H	-2.82880	0.76934	-0.96348
C	3.12933	0.21526	1.30871
H	3.03184	-0.47417	2.16431
H	3.03131	1.24259	1.69839
H	4.16244	0.10883	0.90058

ACE2_nm_CAM-B3LYP_TZVPP

32

O	-0.30658	-1.82106	0.11039
O	-0.32247	1.43372	-0.63980
O	-2.32293	0.10126	0.97425
N	2.04814	-0.05367	0.34851
C	2.04983	-1.42217	-0.13224
C	2.00848	0.96754	-0.68965
C	0.76202	-1.83467	-0.81043
C	0.94624	2.01534	-0.43129
C	-1.57598	-1.71759	-0.48974
C	-1.39599	2.08515	-0.00522
C	-2.58224	-1.21054	0.51602
C	-2.57564	1.14898	0.05546
H	2.21168	-2.07639	0.72551
H	2.87620	-1.61573	-0.83660
H	1.80445	0.51280	-1.65864
H	2.98088	1.46625	-0.78387
H	0.53525	-1.15941	-1.63922
H	0.89044	-2.83837	-1.23316
H	1.02656	2.37827	0.59874
H	1.08778	2.87260	-1.10057

H	-1.91366	-2.69267	-0.86364
H	-1.52476	-1.03624	-1.34344
H	-1.68045	2.99567	-0.54693
H	-1.11406	2.37479	1.01282
H	-3.58296	-1.27743	0.07585
H	-2.56390	-1.84508	1.40176
H	-3.45818	1.70764	0.38078
H	-2.77979	0.75577	-0.94209
C	3.09247	0.19316	1.31885
H	3.00568	-0.50622	2.14960
H	2.99844	1.20135	1.72128
H	4.10407	0.09547	0.89300

ACE2_nm_CAM-B3LYP_TZVP

32

O	-0.30686	-1.81905	0.11104
O	-0.32234	1.43190	-0.63812
O	-2.32414	0.10140	0.97390
N	2.04935	-0.05355	0.34843
C	2.04975	-1.42201	-0.13258
C	2.00911	0.96800	-0.68947
C	0.76154	-1.83422	-0.81005
C	0.94604	2.01493	-0.43131
C	-1.57633	-1.71765	-0.48944
C	-1.39610	2.08468	-0.00517
C	-2.58328	-1.21049	0.51555
C	-2.57644	1.14944	0.05521
H	2.21168	-2.07692	0.72566
H	2.87621	-1.61612	-0.83807
H	1.80593	0.51327	-1.65954
H	2.98175	1.46823	-0.78289

H	0.53497	-1.15950	-1.64040
H	0.88962	-2.83941	-1.23183
H	1.02735	2.37959	0.59892
H	1.08647	2.87203	-1.10258
H	-1.91331	-2.69449	-0.86224
H	-1.52592	-1.03692	-1.34474
H	-1.67975	2.99558	-0.54854
H	-1.11516	2.37517	1.01384
H	-3.58435	-1.27752	0.07405
H	-2.56520	-1.84519	1.40224
H	-3.45888	1.70944	0.38161
H	-2.78155	0.75668	-0.94325
C	3.09485	0.19227	1.31783
H	3.00712	-0.50658	2.14998
H	3.00269	1.20177	1.71958
H	4.10677	0.09215	0.89107

ACE2_nm_CAM-B3LYP_TZV

32

O	-0.30981	-1.89070	0.10677
O	-0.33160	1.46332	-0.67839
O	-2.36057	0.10367	0.99719
N	2.07956	-0.05298	0.34850
C	2.07139	-1.43816	-0.12566
C	2.02974	0.97574	-0.69828
C	0.79304	-1.84638	-0.83030
C	0.97737	2.03659	-0.43468
C	-1.60689	-1.72346	-0.50695
C	-1.42370	2.11225	0.01002
C	-2.60356	-1.24412	0.52249
C	-2.60624	1.17763	0.05015

H	2.21265	-2.08823	0.73776
H	2.90849	-1.63799	-0.81613
H	1.81435	0.52073	-1.66423
H	3.00462	1.47017	-0.79888
H	0.55402	-1.14173	-1.62771
H	0.93295	-2.83277	-1.28136
H	1.04297	2.37520	0.60236
H	1.12916	2.89814	-1.09107
H	-1.95501	-2.67518	-0.91993
H	-1.52956	-1.00440	-1.32421
H	-1.70292	3.03635	-0.50440
H	-1.12465	2.36155	1.03123
H	-3.60993	-1.31822	0.10115
H	-2.55370	-1.87323	1.40763
H	-3.49043	1.72787	0.37542
H	-2.79764	0.78119	-0.94735
C	3.11402	0.20504	1.34867
H	3.01945	-0.49850	2.17395
H	3.00104	1.20986	1.75279
H	4.13204	0.11890	0.93624

ACE2_nm_DSDPBEP86_SV(P)

32

O	-0.30468	-1.71202	0.12652
O	-0.31878	1.32835	-0.58026
O	-2.38734	0.09751	0.97628
N	2.07739	-0.04449	0.34795
C	2.06216	-1.42197	-0.10673
C	2.02679	0.95536	-0.71152
C	0.76168	-1.81811	-0.78252
C	0.92731	1.97364	-0.46699

C	-1.55937	-1.67385	-0.50133
C	-1.38411	2.03995	-0.00590
C	-2.60848	-1.20691	0.48903
C	-2.60525	1.14866	0.05720
H	2.19899	-2.07141	0.77712
H	2.90122	-1.65383	-0.80866
H	1.84133	0.47595	-1.68857
H	2.99743	1.48755	-0.80958
H	0.57156	-1.17132	-1.66134
H	0.85709	-2.85975	-1.16166
H	1.04647	2.40270	0.55023
H	1.00432	2.81160	-1.19487
H	-1.85143	-2.67759	-0.88423
H	-1.52690	-0.98591	-1.36919
H	-1.62204	2.95547	-0.59148
H	-1.12393	2.36004	1.02516
H	-3.61062	-1.29543	0.01891
H	-2.59059	-1.86707	1.37339
H	-3.47359	1.75305	0.38874
H	-2.83436	0.76427	-0.95427
C	3.13211	0.21796	1.30365
H	3.03857	-0.46357	2.16815
H	3.04757	1.25269	1.68146
H	4.15667	0.09490	0.87530

ACE2_nm_DSDPBEP86_SVP

32

O	-0.30109	-1.71580	0.12969
O	-0.32411	1.32376	-0.58557
O	-2.39608	0.09055	0.98008
N	2.07705	-0.03979	0.34980

C	2.06575	-1.41943	-0.09656
C	2.02123	0.95196	-0.71579
C	0.76982	-1.81984	-0.77417
C	0.92229	1.96960	-0.47709
C	-1.55039	-1.66241	-0.50765
C	-1.38473	2.03245	0.00085
C	-2.60695	-1.20977	0.47809
C	-2.60744	1.14660	0.06549
H	2.20255	-2.06195	0.78865
H	2.90439	-1.65414	-0.79284
H	1.83226	0.46638	-1.68583
H	2.98733	1.48385	-0.82334
H	0.58205	-1.17540	-1.65187
H	0.86971	-2.85707	-1.15484
H	1.04112	2.40509	0.53420
H	1.00024	2.80204	-1.20626
H	-1.84343	-2.65421	-0.91101
H	-1.50738	-0.96143	-1.36113
H	-1.62523	2.95084	-0.57308
H	-1.11764	2.34570	1.02914
H	-3.60091	-1.29900	-0.00180
H	-2.59574	-1.87987	1.35140
H	-3.46950	1.75460	0.39615
H	-2.83956	0.76853	-0.94467
C	3.13159	0.23165	1.30173
H	3.04545	-0.44430	2.16629
H	3.04583	1.26363	1.67523
H	4.15244	0.11235	0.87387

ACE2_nm_DSDPBEP86_SV

32

O	-0.30468	-1.71202	0.12652
O	-0.31878	1.32835	-0.58026
O	-2.38734	0.09751	0.97628
N	2.07739	-0.04449	0.34795
C	2.06216	-1.42197	-0.10673
C	2.02679	0.95536	-0.71152
C	0.76168	-1.81811	-0.78252
C	0.92731	1.97364	-0.46699
C	-1.55937	-1.67385	-0.50133
C	-1.38411	2.03995	-0.00590
C	-2.60848	-1.20691	0.48903
C	-2.60525	1.14866	0.05720
H	2.19899	-2.07141	0.77712
H	2.90122	-1.65383	-0.80866
H	1.84133	0.47595	-1.68857
H	2.99743	1.48755	-0.80958
H	0.57156	-1.17132	-1.66134
H	0.85709	-2.85975	-1.16166
H	1.04647	2.40270	0.55023
H	1.00432	2.81160	-1.19487
H	-1.85143	-2.67759	-0.88423
H	-1.52690	-0.98591	-1.36919
H	-1.62204	2.95547	-0.59148
H	-1.12393	2.36004	1.02516
H	-3.61062	-1.29543	0.01891
H	-2.59059	-1.86707	1.37339
H	-3.47359	1.75305	0.38874
H	-2.83436	0.76427	-0.95427
C	3.13211	0.21796	1.30365
H	3.03857	-0.46357	2.16815

H	3.04757	1.25269	1.68146
H	4.15667	0.09490	0.87530

ACE2_nm_DSDPBEP86_TZVPP

32

O	-0.30894	-1.78617	0.12479
O	-0.32264	1.37337	-0.61872
O	-2.35444	0.09868	0.99007
N	2.04409	-0.04589	0.36792
C	2.05008	-1.41629	-0.11731
C	2.01200	0.95971	-0.69213
C	0.75904	-1.81186	-0.80166
C	0.93595	1.99552	-0.44286
C	-1.56805	-1.67994	-0.50458
C	-1.38931	2.06067	-0.00242
C	-2.59781	-1.20727	0.49641
C	-2.58767	1.14575	0.05913
H	2.20456	-2.07330	0.74133
H	2.87925	-1.60612	-0.82107
H	1.81663	0.48934	-1.65695
H	2.98197	1.46480	-0.78257
H	0.54001	-1.12705	-1.62609
H	0.87374	-2.81622	-1.22849
H	1.02448	2.37912	0.58022
H	1.04627	2.84063	-1.13430
H	-1.88831	-2.64919	-0.90977
H	-1.50357	-0.97315	-1.33761
H	-1.64987	2.96883	-0.56135
H	-1.10964	2.35682	1.01538
H	-3.59034	-1.26938	0.03549
H	-2.58729	-1.86200	1.36798

H	-3.46086	1.72225	0.37949
H	-2.79497	0.74766	-0.93670
C	3.13912	0.19535	1.28949
H	3.07344	-0.49347	2.13197
H	3.07642	1.21256	1.67731
H	4.12742	0.07218	0.81671

ACE2_nm_DSDPBEP86_TZVP

32

O	-0.31149	-1.77475	0.12676
O	-0.32055	1.36422	-0.60799
O	-2.36389	0.10092	0.98856
N	2.05227	-0.04655	0.36765
C	2.04937	-1.41635	-0.11987
C	2.01630	0.96148	-0.69035
C	0.75505	-1.80631	-0.80141
C	0.93605	1.99265	-0.43984
C	-1.57192	-1.67978	-0.50209
C	-1.38972	2.05854	-0.00360
C	-2.60409	-1.20641	0.49635
C	-2.59133	1.14756	0.05524
H	2.20428	-2.07717	0.73881
H	2.87760	-1.60950	-0.82790
H	1.82560	0.49219	-1.65930
H	2.98618	1.47239	-0.77805
H	0.53669	-1.12176	-1.62917
H	0.86468	-2.81461	-1.22741
H	1.02792	2.38194	0.58344
H	1.03936	2.83806	-1.13632
H	-1.88757	-2.65619	-0.90116
H	-1.51217	-0.97827	-1.34289

H	-1.64378	2.96623	-0.57146
H	-1.11595	2.36177	1.01642
H	-3.59799	-1.27142	0.03272
H	-2.59454	-1.86018	1.37181
H	-3.46544	1.72852	0.37400
H	-2.79900	0.74864	-0.94278
C	3.15433	0.18812	1.28280
H	3.08947	-0.50075	2.12824
H	3.10061	1.20792	1.67141
H	4.14147	0.05799	0.80411

ACE2_nm_DSDPBEP86_TZV

32

O	-0.32201	-1.88668	0.11205
O	-0.32516	1.43125	-0.68972
O	-2.38443	0.10662	1.02192
N	2.06675	-0.05340	0.37603
C	2.06950	-1.44210	-0.11921
C	2.04979	0.96793	-0.69545
C	0.78572	-1.83142	-0.83783
C	0.98770	2.03197	-0.45393
C	-1.61740	-1.69333	-0.52696
C	-1.40794	2.10521	0.01823
C	-2.62999	-1.24057	0.50951
C	-2.61484	1.19010	0.06247
H	2.20832	-2.10474	0.74092
H	2.91202	-1.62414	-0.81614
H	1.85147	0.50068	-1.66497
H	3.03235	1.46140	-0.77553
H	0.54688	-1.10326	-1.61996
H	0.91736	-2.81413	-1.30991

H	1.04782	2.38453	0.58350
H	1.12818	2.88611	-1.12941
H	-1.96167	-2.63445	-0.97770
H	-1.51956	-0.94199	-1.31800
H	-1.67020	3.04109	-0.49290
H	-1.09143	2.33722	1.04276
H	-3.63502	-1.29535	0.07080
H	-2.58995	-1.88951	1.38564
H	-3.48997	1.75904	0.39398
H	-2.81771	0.79071	-0.93663
C	3.15006	0.18716	1.34648
H	3.05332	-0.50624	2.18536
H	3.07460	1.20638	1.73402
H	4.15316	0.06119	0.89804

ACE2_nm_HSE06_SV(P)

32

O	-0.30237	-1.71317	0.12331
O	-0.31807	1.34866	-0.57600
O	-2.40298	0.09858	0.96416
N	2.09973	-0.04727	0.33130
C	2.06590	-1.42308	-0.11020
C	2.02372	0.95962	-0.71104
C	0.76357	-1.82834	-0.77462
C	0.92705	1.97781	-0.45505
C	-1.55735	-1.68895	-0.48898
C	-1.38820	2.04271	-0.00716
C	-2.60691	-1.20697	0.49219
C	-2.60495	1.14669	0.04851
H	2.20910	-2.06413	0.77784
H	2.89627	-1.67415	-0.81508

H	1.82739	0.48612	-1.68852
H	2.98909	1.49675	-0.82372
H	0.57478	-1.19726	-1.66606
H	0.86487	-2.87323	-1.14126
H	1.04723	2.40044	0.56488
H	1.01496	2.82260	-1.17291
H	-1.85321	-2.69851	-0.85171
H	-1.53464	-1.02133	-1.37403
H	-1.63443	2.95887	-0.58743
H	-1.13604	2.36514	1.02534
H	-3.60566	-1.31438	0.01969
H	-2.59140	-1.85541	1.38490
H	-3.47491	1.75370	0.36885
H	-2.82689	0.76443	-0.96545
C	3.12741	0.22157	1.30343
H	3.03482	-0.46909	2.16005
H	3.02297	1.24887	1.69491
H	4.16350	0.12259	0.89642

ACE2_nm_HSE06_SVP

32

O	-0.30058	-1.72375	0.12523
O	-0.32167	1.34932	-0.58277
O	-2.40571	0.09468	0.96810
N	2.09872	-0.04472	0.33288
C	2.06701	-1.42165	-0.10568
C	2.01984	0.95826	-0.71267
C	0.76829	-1.82720	-0.77242
C	0.92528	1.97693	-0.45976
C	-1.55337	-1.68196	-0.49298
C	-1.38930	2.03981	-0.00260

C	-2.60507	-1.20921	0.48703
C	-2.60501	1.14574	0.05407
H	2.21017	-2.06057	0.78161
H	2.89785	-1.67501	-0.80592
H	1.81980	0.48216	-1.68620
H	2.98290	1.49358	-0.83384
H	0.57806	-1.19022	-1.65739
H	0.87435	-2.86516	-1.15104
H	1.04361	2.40148	0.55760
H	1.01732	2.82120	-1.17458
H	-1.85480	-2.68047	-0.87492
H	-1.52119	-1.00208	-1.36638
H	-1.63958	2.95926	-0.57207
H	-1.13140	2.35751	1.02803
H	-3.59991	-1.31790	0.01104
H	-2.59349	-1.86518	1.37216
H	-3.47327	1.75348	0.37110
H	-2.82740	0.76699	-0.95934
C	3.12678	0.22757	1.30377
H	3.04088	-0.46274	2.15775
H	3.02148	1.25083	1.69772
H	4.16055	0.13415	0.89747

ACE2_nm_HSE06_SV

32

O	-0.30237	-1.71317	0.12331
O	-0.31807	1.34866	-0.57600
O	-2.40298	0.09858	0.96416
N	2.09973	-0.04727	0.33130
C	2.06590	-1.42308	-0.11020
C	2.02372	0.95962	-0.71104

C	0.76357	-1.82834	-0.77462
C	0.92705	1.97781	-0.45505
C	-1.55735	-1.68895	-0.48898
C	-1.38820	2.04271	-0.00716
C	-2.60691	-1.20697	0.49219
C	-2.60495	1.14669	0.04851
H	2.20910	-2.06413	0.77784
H	2.89627	-1.67415	-0.81508
H	1.82739	0.48612	-1.68852
H	2.98909	1.49675	-0.82372
H	0.57478	-1.19726	-1.66606
H	0.86487	-2.87323	-1.14126
H	1.04723	2.40044	0.56488
H	1.01496	2.82260	-1.17291
H	-1.85321	-2.69851	-0.85171
H	-1.53464	-1.02133	-1.37403
H	-1.63443	2.95887	-0.58743
H	-1.13604	2.36514	1.02534
H	-3.60566	-1.31438	0.01969
H	-2.59140	-1.85541	1.38490
H	-3.47491	1.75370	0.36885
H	-2.82689	0.76443	-0.96545
C	3.12741	0.22157	1.30343
H	3.03482	-0.46909	2.16005
H	3.02297	1.24887	1.69491
H	4.16350	0.12259	0.89642

ACE2_nm_HSE06_TZVPP

32

O	-0.29701	-1.81807	0.11684
O	-0.33022	1.42276	-0.63893

O	-2.33369	0.09321	0.97877
N	2.04706	-0.04637	0.34972
C	2.05753	-1.41457	-0.12378
C	1.99872	0.96683	-0.69261
C	0.77199	-1.83637	-0.79946
C	0.93319	2.01081	-0.43492
C	-1.55852	-1.70862	-0.49338
C	-1.40075	2.07435	-0.00565
C	-2.57708	-1.21385	0.50587
C	-2.58044	1.13842	0.05899
H	2.22427	-2.06431	0.73963
H	2.88590	-1.61209	-0.82893
H	1.79030	0.50426	-1.66008
H	2.97037	1.47027	-0.79844
H	0.54649	-1.16681	-1.63761
H	0.90673	-2.84369	-1.21813
H	1.01597	2.37846	0.59631
H	1.07265	2.86974	-1.10669
H	-1.89533	-2.68003	-0.88489
H	-1.50046	-1.01517	-1.34112
H	-1.68978	2.98617	-0.54802
H	-1.11808	2.36923	1.01370
H	-3.57414	-1.28722	0.05231
H	-2.56618	-1.85777	1.38790
H	-3.46380	1.70267	0.38068
H	-2.78647	0.74400	-0.94076
C	3.09278	0.21315	1.31062
H	3.01817	-0.48600	2.14558
H	2.99037	1.22319	1.71232
H	4.10634	0.12567	0.88065

ACE2_nm_HSE06_TZVP

32

O	-0.29741	-1.81609	0.11762
O	-0.33006	1.42103	-0.63713
O	-2.33512	0.09333	0.97834
N	2.04834	-0.04623	0.34967
C	2.05748	-1.41448	-0.12405
C	1.99940	0.96725	-0.69255
C	0.77150	-1.83625	-0.79887
C	0.93312	2.01055	-0.43525
C	-1.55895	-1.70873	-0.49317
C	-1.40076	2.07400	-0.00527
C	-2.57836	-1.21379	0.50515
C	-2.58128	1.13911	0.05881
H	2.22453	-2.06477	0.73978
H	2.88575	-1.61261	-0.83040
H	1.79161	0.50464	-1.66091
H	2.97122	1.47209	-0.79782
H	0.54623	-1.16768	-1.63878
H	0.90596	-2.84513	-1.21614
H	1.01704	2.38043	0.59586
H	1.07141	2.86890	-1.10939
H	-1.89501	-2.68185	-0.88357
H	-1.50159	-1.01597	-1.34243
H	-1.68891	2.98627	-0.54907
H	-1.11909	2.36951	1.01501
H	-3.57560	-1.28730	0.05013
H	-2.56793	-1.85793	1.38798
H	-3.46441	1.70470	0.38160
H	-2.78816	0.74553	-0.94189

C	3.09541	0.21222	1.30959
H	3.01948	-0.48587	2.14622
H	2.99540	1.22369	1.71010
H	4.10923	0.12153	0.87898

ACE2_nm_HSE06_TZV

32

O	-0.30100	-1.87572	0.11872
O	-0.33977	1.44497	-0.67384
O	-2.38313	0.09483	1.00245
N	2.08580	-0.04597	0.35116
C	2.08046	-1.42873	-0.12321
C	2.02326	0.97918	-0.69640
C	0.79956	-1.84106	-0.81908
C	0.96228	2.03041	-0.43184
C	-1.59099	-1.71459	-0.50819
C	-1.43074	2.09794	0.00888
C	-2.60560	-1.24815	0.50888
C	-2.61626	1.16720	0.05225
H	2.23269	-2.07986	0.74108
H	2.91396	-1.62995	-0.82302
H	1.80892	0.51989	-1.66414
H	2.99474	1.48500	-0.80380
H	0.56237	-1.14055	-1.62551
H	0.94083	-2.83222	-1.26679
H	1.02900	2.37101	0.60782
H	1.10927	2.89626	-1.08882
H	-1.93209	-2.66735	-0.93311
H	-1.51130	-0.98859	-1.32378
H	-1.71197	3.02303	-0.50933
H	-1.13440	2.35473	1.03237

H	-3.60557	-1.32937	0.06624
H	-2.56930	-1.88767	1.39069
H	-3.49924	1.72765	0.37318
H	-2.81101	0.77001	-0.94763
C	3.13489	0.21595	1.33123
H	3.05923	-0.49075	2.15901
H	3.02292	1.22145	1.74060
H	4.15032	0.13752	0.90296

ACE2_nm_M062x_SV(P)

32

O	-0.32059	-1.67053	0.11843
O	-0.30720	1.29333	-0.54732
O	-2.41622	0.11011	0.97558
N	2.11808	-0.05863	0.34755
C	2.04698	-1.41977	-0.14536
C	2.05284	0.97187	-0.67993
C	0.72391	-1.74569	-0.81213
C	0.92480	1.95532	-0.42562
C	-1.58286	-1.66112	-0.48474
C	-1.38323	2.02109	-0.02310
C	-2.62654	-1.20015	0.51339
C	-2.61321	1.14245	0.03836
H	2.17458	-2.10080	0.71359
H	2.86426	-1.65347	-0.86762
H	1.90681	0.52010	-1.67600
H	3.00534	1.53445	-0.73618
H	0.53087	-1.03913	-1.64226
H	0.78446	-2.76241	-1.25358
H	1.03295	2.36943	0.59786
H	0.98352	2.80432	-1.13816

H	-1.85977	-2.67113	-0.85436
H	-1.57495	-0.98173	-1.35976
H	-1.59751	2.91998	-0.63753
H	-1.14585	2.36554	1.00389
H	-3.63074	-1.30689	0.05707
H	-2.58839	-1.84854	1.40350
H	-3.47757	1.75998	0.34564
H	-2.83180	0.74036	-0.96734
C	3.20248	0.14807	1.28073
H	3.12147	-0.56489	2.11800
H	3.14840	1.16667	1.70018
H	4.20845	0.02281	0.81709

ACE2_nm_M062x_SVP

32

O	-0.32186	-1.67510	0.11955
O	-0.30804	1.29011	-0.55074
O	-2.42329	0.10866	0.97907
N	2.12146	-0.05856	0.35023
C	2.04524	-1.41770	-0.14822
C	2.05466	0.97424	-0.67494
C	0.72193	-1.73517	-0.81441
C	0.92406	1.95322	-0.42343
C	-1.58383	-1.65452	-0.48616
C	-1.38340	2.01670	-0.02079
C	-2.62883	-1.20111	0.51101
C	-2.61427	1.14231	0.04106
H	2.17435	-2.10262	0.70470
H	2.85915	-1.65043	-0.87139
H	1.91448	0.52612	-1.67150
H	3.00285	1.53983	-0.72850

H	0.52634	-1.01606	-1.63044
H	0.78163	-2.74153	-1.27396
H	1.02704	2.36633	0.59880
H	0.98470	2.80362	-1.13082
H	-1.86370	-2.65607	-0.86973
H	-1.57025	-0.96637	-1.35187
H	-1.59805	2.91761	-0.62834
H	-1.14299	2.35850	1.00425
H	-3.62952	-1.30997	0.05240
H	-2.59332	-1.85400	1.39550
H	-3.47532	1.76281	0.34406
H	-2.83370	0.74219	-0.96316
C	3.21320	0.14006	1.27701
H	3.13782	-0.57504	2.10915
H	3.16795	1.15362	1.70193
H	4.21317	0.01388	0.80746

ACE2_nm_M062x_SV

32

O	-0.32059	-1.67053	0.11843
O	-0.30720	1.29333	-0.54732
O	-2.41622	0.11011	0.97558
N	2.11808	-0.05863	0.34755
C	2.04698	-1.41977	-0.14536
C	2.05284	0.97187	-0.67993
C	0.72391	-1.74569	-0.81213
C	0.92480	1.95532	-0.42562
C	-1.58286	-1.66112	-0.48474
C	-1.38323	2.02109	-0.02310
C	-2.62654	-1.20015	0.51339
C	-2.61321	1.14245	0.03836

H	2.17458	-2.10080	0.71359
H	2.86426	-1.65347	-0.86762
H	1.90681	0.52010	-1.67600
H	3.00534	1.53445	-0.73618
H	0.53087	-1.03913	-1.64226
H	0.78446	-2.76241	-1.25358
H	1.03295	2.36943	0.59786
H	0.98352	2.80432	-1.13816
H	-1.85977	-2.67113	-0.85436
H	-1.57495	-0.98173	-1.35976
H	-1.59751	2.91998	-0.63753
H	-1.14585	2.36554	1.00389
H	-3.63074	-1.30689	0.05707
H	-2.58839	-1.84854	1.40350
H	-3.47757	1.75998	0.34564
H	-2.83180	0.74036	-0.96734
C	3.20248	0.14807	1.28073
H	3.12147	-0.56489	2.11800
H	3.14840	1.16667	1.70018
H	4.20845	0.02281	0.81709

ACE2_nm_M062x_TZVPP

32

O	-0.31455	-1.72077	0.12819
O	-0.31968	1.31933	-0.56632
O	-2.41412	0.10240	0.98093
N	2.09420	-0.04616	0.36155
C	2.05371	-1.41509	-0.12541
C	2.02933	0.96991	-0.68615
C	0.74589	-1.77428	-0.79835
C	0.92364	1.97145	-0.42543

C	-1.57436	-1.66176	-0.49504
C	-1.39586	2.03396	-0.00853
C	-2.62038	-1.20965	0.49886
C	-2.61213	1.14167	0.04017
H	2.20013	-2.07938	0.72905
H	2.86906	-1.62323	-0.83888
H	1.85692	0.50874	-1.66012
H	2.98122	1.50767	-0.76450
H	0.54032	-1.08341	-1.62202
H	0.82856	-2.78115	-1.22644
H	1.02400	2.36660	0.59291
H	0.99304	2.81430	-1.12462
H	-1.86220	-2.64525	-0.88959
H	-1.53237	-0.96357	-1.33799
H	-1.62001	2.93126	-0.59918
H	-1.14578	2.35271	1.01061
H	-3.60867	-1.29970	0.03372
H	-2.59796	-1.85829	1.37499
H	-3.47915	1.73661	0.34108
H	-2.81195	0.73869	-0.95574
C	3.21254	0.17421	1.25825
H	3.15919	-0.51985	2.09701
H	3.17222	1.18894	1.65528
H	4.18657	0.04137	0.76096

ACE2_nm_M062x_TZVP

32

O	-0.31483	-1.71981	0.12859
O	-0.31986	1.31869	-0.56556
O	-2.41498	0.10230	0.98081
N	2.09524	-0.04599	0.36190

C	2.05361	-1.41479	-0.12592
C	2.02985	0.97065	-0.68550
C	0.74547	-1.77362	-0.79839
C	0.92339	1.97142	-0.42500
C	-1.57482	-1.66229	-0.49484
C	-1.39640	2.03382	-0.00857
C	-2.62122	-1.20986	0.49854
C	-2.61302	1.14201	0.04026
H	2.20009	-2.08013	0.72852
H	2.86899	-1.62288	-0.84032
H	1.85886	0.50976	-1.66054
H	2.98175	1.50981	-0.76267
H	0.53934	-1.08293	-1.62281
H	0.82792	-2.78147	-1.22610
H	1.02405	2.36726	0.59367
H	0.99238	2.81441	-1.12521
H	-1.86211	-2.64724	-0.88826
H	-1.53312	-0.96493	-1.33924
H	-1.62026	2.93130	-0.60034
H	-1.14687	2.35319	1.01118
H	-3.60988	-1.29963	0.03264
H	-2.59905	-1.85854	1.37549
H	-3.47980	1.73790	0.34268
H	-2.81434	0.73946	-0.95618
C	3.21555	0.17290	1.25694
H	3.16188	-0.52058	2.09701
H	3.17748	1.18862	1.65336
H	4.18906	0.03767	0.75800

ACE2_nm_M062x_TZV

32

O	-0.30241	-1.85744	0.11429
O	-0.33960	1.40065	-0.66008
O	-2.38803	0.09600	1.00734
N	2.08424	-0.04408	0.35881
C	2.07921	-1.42923	-0.12144
C	2.03286	0.97836	-0.69815
C	0.79387	-1.81251	-0.82876
C	0.95493	2.01408	-0.43734
C	-1.59020	-1.68323	-0.51608
C	-1.42822	2.07974	0.00537
C	-2.60633	-1.24859	0.51591
C	-2.62572	1.16208	0.05196
H	2.21253	-2.08555	0.73970
H	2.91690	-1.62233	-0.81239
H	1.83702	0.51712	-1.66656
H	2.99931	1.49087	-0.78480
H	0.55976	-1.08398	-1.60764
H	0.91470	-2.79176	-1.30033
H	1.02375	2.35649	0.59939
H	1.07152	2.87575	-1.10067
H	-1.91746	-2.62278	-0.97165
H	-1.50342	-0.92709	-1.30024
H	-1.68312	3.00029	-0.52712
H	-1.13483	2.33507	1.02736
H	-3.60870	-1.33168	0.08658
H	-2.54681	-1.89322	1.39004
H	-3.50316	1.72468	0.37364
H	-2.82048	0.75814	-0.94302
C	3.14963	0.21315	1.33123
H	3.06716	-0.48361	2.16395

H	3.05340	1.22425	1.72528
H	4.15245	0.11118	0.88820

ACE2_nm_M06_SV(P)

32

O	-0.29806	-1.71811	0.12747
O	-0.32650	1.34871	-0.58471
O	-2.38682	0.08880	0.96293
N	2.08113	-0.04024	0.33616
C	2.06235	-1.41990	-0.09692
C	2.00830	0.95825	-0.71664
C	0.77148	-1.83711	-0.76405
C	0.91815	1.97595	-0.46361
C	-1.54528	-1.67485	-0.49695
C	-1.38933	2.03524	0.00409
C	-2.59552	-1.21195	0.48315
C	-2.60177	1.14178	0.05816
H	2.20596	-2.05651	0.79858
H	2.90620	-1.66447	-0.79244
H	1.81059	0.47727	-1.69397
H	2.98014	1.48842	-0.83581
H	0.58142	-1.21438	-1.66615
H	0.87493	-2.88600	-1.12701
H	1.03801	2.39829	0.56075
H	1.00409	2.82765	-1.17757
H	-1.83736	-2.67463	-0.89588
H	-1.50956	-0.97882	-1.36428
H	-1.63349	2.96742	-0.55622
H	-1.12687	2.33777	1.04377
H	-3.59737	-1.31319	0.00972
H	-2.58202	-1.87320	1.36997

H	-3.47641	1.74368	0.38394
H	-2.83007	0.76519	-0.96052
C	3.12060	0.23695	1.29279
H	3.04014	-0.44289	2.16180
H	3.02981	1.27189	1.67418
H	4.15109	0.12675	0.86848

ACE2_nm_M06_SVP

32

O	-0.29710	-1.72439	0.12915
O	-0.32818	1.35153	-0.59070
O	-2.38690	0.08684	0.96590
N	2.07925	-0.03969	0.33686
C	2.06235	-1.41923	-0.09716
C	2.00551	0.95807	-0.71632
C	0.77355	-1.83630	-0.76358
C	0.91828	1.97629	-0.46398
C	-1.54374	-1.67122	-0.49782
C	-1.39005	2.03423	0.00645
C	-2.59406	-1.21323	0.48098
C	-2.59984	1.14076	0.06031
H	2.20965	-2.05576	0.79444
H	2.90416	-1.66358	-0.79053
H	1.80492	0.47862	-1.69094
H	2.97488	1.48540	-0.84158
H	0.58266	-1.21139	-1.66117
H	0.87969	-2.87851	-1.13622
H	1.03619	2.39631	0.55876
H	1.00992	2.83009	-1.17018
H	-1.83994	-2.66177	-0.90850
H	-1.50436	-0.97048	-1.35829

H	-1.63807	2.96817	-0.54354
H	-1.12504	2.33416	1.04350
H	-3.59202	-1.31661	0.00639
H	-2.58456	-1.87957	1.36074
H	-3.47449	1.74178	0.37822
H	-2.82473	0.76629	-0.95724
C	3.11882	0.23797	1.29366
H	3.04311	-0.44077	2.15934
H	3.02939	1.26826	1.67746
H	4.14643	0.13143	0.87136

ACE2_nm_M06_SV

32

O	-0.29806	-1.71811	0.12747
O	-0.32650	1.34871	-0.58471
O	-2.38682	0.08880	0.96293
N	2.08113	-0.04024	0.33616
C	2.06235	-1.41990	-0.09692
C	2.00830	0.95825	-0.71664
C	0.77148	-1.83711	-0.76405
C	0.91815	1.97595	-0.46361
C	-1.54528	-1.67485	-0.49695
C	-1.38933	2.03524	0.00409
C	-2.59552	-1.21195	0.48315
C	-2.60177	1.14178	0.05816
H	2.20596	-2.05651	0.79858
H	2.90620	-1.66447	-0.79244
H	1.81059	0.47727	-1.69397
H	2.98014	1.48842	-0.83581
H	0.58142	-1.21438	-1.66615
H	0.87493	-2.88600	-1.12701

H	1.03801	2.39829	0.56075
H	1.00409	2.82765	-1.17757
H	-1.83736	-2.67463	-0.89588
H	-1.50956	-0.97882	-1.36428
H	-1.63349	2.96742	-0.55622
H	-1.12687	2.33777	1.04377
H	-3.59737	-1.31319	0.00972
H	-2.58202	-1.87320	1.36997
H	-3.47641	1.74368	0.38394
H	-2.83007	0.76519	-0.96052
C	3.12060	0.23695	1.29279
H	3.04014	-0.44289	2.16180
H	3.02981	1.27189	1.67418
H	4.15109	0.12675	0.86848

ACE2_nm_M06_TZVPP

32

O	-0.29885	-1.76388	0.12577
O	-0.33070	1.37462	-0.61179
O	-2.34723	0.08872	0.97409
N	2.04898	-0.04001	0.34999
C	2.05342	-1.41462	-0.10573
C	1.99385	0.96048	-0.70572
C	0.77097	-1.83201	-0.77942
C	0.92129	1.98866	-0.44856
C	-1.54928	-1.68006	-0.49926
C	-1.39270	2.04939	0.00165
C	-2.58175	-1.21114	0.49021
C	-2.58439	1.13690	0.06166
H	2.21152	-2.05364	0.76798
H	2.88814	-1.62194	-0.80027

H	1.78844	0.48718	-1.66921
H	2.96249	1.46722	-0.81791
H	0.56377	-1.18501	-1.64242
H	0.88611	-2.85282	-1.17174
H	1.02032	2.37586	0.57627
H	1.02801	2.84270	-1.13298
H	-1.85876	-2.65403	-0.90730
H	-1.49587	-0.97692	-1.34204
H	-1.65418	2.96715	-0.54550
H	-1.11512	2.34472	1.02393
H	-3.57483	-1.28729	0.02776
H	-2.57519	-1.86758	1.36353
H	-3.46162	1.71136	0.38181
H	-2.79774	0.75021	-0.94072
C	3.11232	0.22499	1.28892
H	3.04364	-0.45406	2.14044
H	3.03460	1.24521	1.66987
H	4.11526	0.11057	0.84152

ACE2_nm_M06_TZVP

32

O	-0.29862	-1.76160	0.12726
O	-0.33087	1.37115	-0.60939
O	-2.35118	0.08822	0.97383
N	2.05093	-0.03923	0.34986
C	2.05364	-1.41375	-0.10480
C	1.99384	0.96081	-0.70583
C	0.77130	-1.83183	-0.77706
C	0.92007	1.98692	-0.44896
C	-1.54806	-1.67826	-0.49910
C	-1.39287	2.04715	0.00186

C	-2.58289	-1.21113	0.48797
C	-2.58583	1.13702	0.06185
H	2.21288	-2.05278	0.77018
H	2.88884	-1.62212	-0.80071
H	1.78960	0.48643	-1.67032
H	2.96274	1.46979	-0.81805
H	0.56445	-1.18624	-1.64270
H	0.88658	-2.85455	-1.16833
H	1.02067	2.37633	0.57623
H	1.02501	2.84162	-1.13514
H	-1.85666	-2.65315	-0.90949
H	-1.49407	-0.97375	-1.34229
H	-1.65305	2.96611	-0.54677
H	-1.11604	2.34382	1.02536
H	-3.57546	-1.28797	0.02140
H	-2.57859	-1.86921	1.36162
H	-3.46250	1.71461	0.38251
H	-2.80106	0.75115	-0.94179
C	3.11567	0.22593	1.28631
H	3.04804	-0.45237	2.13991
H	3.03953	1.24786	1.66597
H	4.11870	0.10971	0.83631

ACE2_nm_M06_TZV

32

O	-0.29426	-1.83996	0.12311
O	-0.34466	1.40277	-0.65884
O	-2.38208	0.08865	0.99888
N	2.07934	-0.03694	0.34655
C	2.08083	-1.42642	-0.10310
C	2.01334	0.97352	-0.71324

C	0.80703	-1.84377	-0.80328
C	0.94520	2.01199	-0.44871
C	-1.57136	-1.68637	-0.51587
C	-1.42451	2.07361	0.01141
C	-2.59455	-1.24731	0.50077
C	-2.61762	1.15775	0.05540
H	2.21839	-2.06181	0.77601
H	2.92484	-1.63737	-0.78630
H	1.79900	0.50272	-1.67575
H	2.98243	1.48217	-0.82623
H	0.58505	-1.16234	-1.63226
H	0.94026	-2.84662	-1.22760
H	1.02390	2.36298	0.58838
H	1.06579	2.87633	-1.11329
H	-1.89563	-2.63315	-0.96663
H	-1.49085	-0.93966	-1.31483
H	-1.68643	3.00417	-0.50737
H	-1.12911	2.32541	1.03770
H	-3.59350	-1.33484	0.05684
H	-2.54992	-1.89697	1.37505
H	-3.49696	1.72312	0.37676
H	-2.81709	0.76305	-0.94542
C	3.12007	0.24517	1.32772
H	3.03978	-0.44616	2.16795
H	3.00592	1.25914	1.71593
H	4.13612	0.15771	0.90341

ACE2_nm_MP2_SV(P)

32

O	-0.31012	-1.71129	0.12702
O	-0.31412	1.32476	-0.58047

O	-2.37177	0.10305	0.97828
N	2.06721	-0.04819	0.35354
C	2.05236	-1.42047	-0.11624
C	2.02940	0.95498	-0.70324
C	0.75100	-1.80599	-0.79201
C	0.93197	1.97293	-0.46030
C	-1.56762	-1.67382	-0.49941
C	-1.37961	2.04237	-0.00845
C	-2.60841	-1.20173	0.49441
C	-2.59782	1.15030	0.05450
H	2.18833	-2.08043	0.75930
H	2.89048	-1.64317	-0.82173
H	1.84914	0.48239	-1.68413
H	3.00257	1.48392	-0.79179
H	0.55712	-1.14664	-1.65944
H	0.84337	-2.84224	-1.18576
H	1.04875	2.39628	0.55879
H	1.01099	2.81338	-1.18473
H	-1.86246	-2.67777	-0.87861
H	-1.53752	-0.98811	-1.36789
H	-1.61510	2.95642	-0.59659
H	-1.12177	2.36273	1.02229
H	-3.61389	-1.28320	0.03133
H	-2.58833	-1.86060	1.37893
H	-3.46972	1.75100	0.38287
H	-2.82241	0.76289	-0.95598
C	3.13533	0.19887	1.30008
H	3.04088	-0.48384	2.16236
H	3.06510	1.23263	1.68049
H	4.15253	0.06485	0.86067

ACE2_nm_MP2_SVP

32

O	-0.30594	-1.71003	0.13076
O	-0.31990	1.31526	-0.58380
O	-2.38606	0.09505	0.98296
N	2.06897	-0.04282	0.35626
C	2.05629	-1.41645	-0.10562
C	2.02359	0.95174	-0.70635
C	0.75881	-1.80418	-0.78222
C	0.92436	1.96605	-0.46986
C	-1.55640	-1.65822	-0.50601
C	-1.38020	2.03045	-0.00187
C	-2.60753	-1.20441	0.48160
C	-2.60134	1.14612	0.06295
H	2.19229	-2.06868	0.77007
H	2.89150	-1.64155	-0.80590
H	1.84127	0.47259	-1.67873
H	2.98915	1.48250	-0.80365
H	0.56871	-1.14687	-1.64683
H	0.85412	-2.83416	-1.17788
H	1.04039	2.39535	0.54197
H	1.00245	2.79968	-1.19468
H	-1.84988	-2.64817	-0.90791
H	-1.51583	-0.95834	-1.35737
H	-1.61674	2.94579	-0.57827
H	-1.11654	2.34375	1.02481
H	-3.60143	-1.28803	0.00558
H	-2.59476	-1.87418	1.35240
H	-3.46428	1.75261	0.38757
H	-2.82784	0.76469	-0.94471

C	3.13968	0.21235	1.29442
H	3.05559	-0.46364	2.15597
H	3.07095	1.24216	1.66991
H	4.14983	0.08124	0.85221

ACE2_nm_MP2_SV

32

O	-0.31012	-1.71129	0.12702
O	-0.31412	1.32476	-0.58047
O	-2.37177	0.10305	0.97828
N	2.06721	-0.04819	0.35354
C	2.05236	-1.42047	-0.11624
C	2.02940	0.95498	-0.70324
C	0.75100	-1.80599	-0.79201
C	0.93197	1.97293	-0.46030
C	-1.56762	-1.67382	-0.49941
C	-1.37961	2.04237	-0.00845
C	-2.60841	-1.20173	0.49441
C	-2.59782	1.15030	0.05450
H	2.18833	-2.08043	0.75930
H	2.89048	-1.64317	-0.82173
H	1.84914	0.48239	-1.68413
H	3.00257	1.48392	-0.79179
H	0.55712	-1.14664	-1.65944
H	0.84337	-2.84224	-1.18576
H	1.04875	2.39628	0.55879
H	1.01099	2.81338	-1.18473
H	-1.86246	-2.67777	-0.87861
H	-1.53752	-0.98811	-1.36789
H	-1.61510	2.95642	-0.59659
H	-1.12177	2.36273	1.02229

H	-3.61389	-1.28320	0.03133
H	-2.58833	-1.86060	1.37893
H	-3.46972	1.75100	0.38287
H	-2.82241	0.76289	-0.95598
C	3.13533	0.19887	1.30008
H	3.04088	-0.48384	2.16236
H	3.06510	1.23263	1.68049
H	4.15253	0.06485	0.86067

ACE2_nm_MP2_TZVPP

32

O	-0.31059	1.75470	-0.13404
O	-0.32329	-1.33150	0.59626
O	-2.37640	-0.09705	-0.99474
N	2.05163	0.04136	-0.37709
C	2.04612	1.40714	0.11793
C	2.01299	-0.96168	0.68405
C	0.75190	1.78307	0.79875
C	0.92507	-1.97955	0.43624
C	-1.56502	1.65811	0.50703
C	-1.39026	-2.04057	0.00344
C	-2.60539	1.20645	-0.48681
C	-2.59531	-1.14024	-0.05578
H	2.19784	2.06991	-0.73265
H	2.86840	1.59404	0.82534
H	1.82885	-0.49114	1.64780
H	2.97358	-1.47760	0.76724
H	0.53590	1.08838	1.61145
H	0.85367	2.78108	1.23443
H	1.01479	-2.36879	-0.58105
H	1.01387	-2.81771	1.13357

H	-1.86971	2.62415	0.92185
H	-1.49950	0.94417	1.32947
H	-1.63292	-2.94001	0.57721
H	-1.12025	-2.34512	-1.01076
H	-3.58989	1.27115	-0.01742
H	-2.59547	1.86718	-1.34919
H	-3.46108	-1.72538	-0.36739
H	-2.79978	-0.73891	0.93557
C	3.18229	-0.18243	-1.25954
H	3.13617	0.50691	-2.09826
H	3.14620	-1.19627	-1.64931
H	4.14597	-0.04656	-0.74997

ACE2_nm_MP2_TZVP

32

O	-0.31310	-1.74903	0.13467
O	-0.31995	1.33081	-0.58930
O	-2.37605	0.10087	0.99166
N	2.05488	-0.04368	0.37514
C	2.04469	-1.40896	-0.12180
C	2.01658	0.96244	-0.68343
C	0.74812	-1.78306	-0.80015
C	0.92858	1.98023	-0.43361
C	-1.57054	-1.66393	-0.50305
C	-1.38973	2.04381	-0.00543
C	-2.60912	-1.20480	0.49000
C	-2.59526	1.14344	0.05128
H	2.19838	-2.07631	0.73007
H	2.86877	-1.59789	-0.83340
H	1.83459	0.49374	-1.65293
H	2.98135	1.48004	-0.76490

H	0.53078	-1.09087	-1.61958
H	0.84655	-2.78753	-1.23346
H	1.02172	2.37406	0.58603
H	1.01427	2.82140	-1.13486
H	-1.87383	-2.64007	-0.90743
H	-1.51075	-0.95906	-1.33874
H	-1.62856	2.94481	-0.58686
H	-1.12437	2.35534	1.01240
H	-3.59857	-1.27022	0.02111
H	-2.60054	-1.86271	1.36001
H	-3.46591	1.72969	0.36311
H	-2.80017	0.74082	-0.94388
C	3.18526	0.17630	1.25917
H	3.13562	-0.51259	2.10308
H	3.15423	1.19445	1.64891
H	4.15346	0.03462	0.75115

ACE2_nm_MP2_TZV

32

O	-0.34512	-1.91378	0.09838
O	-0.30662	1.44061	-0.70570
O	-2.36570	0.12573	1.03489
N	2.05452	-0.06649	0.38853
C	2.05111	-1.45501	-0.13012
C	2.07398	0.96181	-0.68643
C	0.76491	-1.82139	-0.86318
C	1.01809	2.03945	-0.45956
C	-1.65343	-1.69443	-0.53183
C	-1.38835	2.12541	0.01887
C	-2.64545	-1.22773	0.52325
C	-2.60147	1.21403	0.06581

H	2.17963	-2.13062	0.72218
H	2.89575	-1.62635	-0.82840
H	1.88696	0.49866	-1.66141
H	3.06551	1.44299	-0.74281
H	0.51925	-1.06422	-1.61518
H	0.89441	-2.78812	-1.36841
H	1.06829	2.39116	0.57888
H	1.16773	2.88895	-1.13896
H	-2.01644	-2.62914	-0.98143
H	-1.54868	-0.94024	-1.31932
H	-1.64438	3.06413	-0.49006
H	-1.05999	2.34602	1.04215
H	-3.65772	-1.25928	0.09848
H	-2.60128	-1.87258	1.40214
H	-3.47567	1.78364	0.39937
H	-2.80409	0.80978	-0.93199
C	3.14981	0.14466	1.36704
H	3.02704	-0.54648	2.20429
H	3.09853	1.16712	1.75008
H	4.14785	-0.01119	0.91706

ACE2_nm_PBE0_SV(P)

32

O	-0.30246	-1.71341	0.12339
O	-0.31757	1.34891	-0.57583
O	-2.40188	0.09895	0.96360
N	2.09925	-0.04747	0.33090
C	2.06543	-1.42298	-0.11115
C	2.02400	0.96009	-0.71066
C	0.76273	-1.82883	-0.77461
C	0.92699	1.97805	-0.45512

C	-1.55717	-1.69009	-0.48839
C	-1.38736	2.04305	-0.00773
C	-2.60659	-1.20599	0.49205
C	-2.60414	1.14699	0.04880
H	2.20935	-2.06416	0.77664
H	2.89525	-1.67369	-0.81666
H	1.82904	0.48733	-1.68868
H	2.98931	1.49755	-0.82184
H	0.57349	-1.19869	-1.66648
H	0.86440	-2.87408	-1.14024
H	1.04733	2.40130	0.56449
H	1.01503	2.82235	-1.17358
H	-1.85363	-2.70041	-0.84857
H	-1.53470	-1.02463	-1.37494
H	-1.63412	2.95853	-0.58891
H	-1.13544	2.36662	1.02444
H	-3.60523	-1.31288	0.01918
H	-2.59220	-1.85404	1.38512
H	-3.47355	1.75424	0.37036
H	-2.82741	0.76547	-0.96510
C	3.12586	0.22076	1.30418
H	3.03192	-0.47025	2.16042
H	3.02069	1.24786	1.69607
H	4.16242	0.12193	0.89852

ACE2_nm_PBE0_SVP

32

O	-0.30069	-1.72383	0.12532
O	-0.32118	1.34960	-0.58255
O	-2.40488	0.09505	0.96743
N	2.09842	-0.04487	0.33241

C	2.06661	-1.42156	-0.10655
C	2.02023	0.95880	-0.71237
C	0.76754	-1.82795	-0.77225
C	0.92523	1.97718	-0.45998
C	-1.55323	-1.68326	-0.49239
C	-1.38847	2.04016	-0.00308
C	-2.60486	-1.20826	0.48678
C	-2.60433	1.14619	0.05438
H	2.21053	-2.06049	0.78054
H	2.89684	-1.67467	-0.80738
H	1.82192	0.48355	-1.68652
H	2.98311	1.49464	-0.83183
H	0.57690	-1.19253	-1.65804
H	0.87405	-2.86646	-1.14926
H	1.04383	2.40255	0.55690
H	1.01733	2.82083	-1.17553
H	-1.85520	-2.68272	-0.87139
H	-1.52139	-1.00595	-1.36759
H	-1.63921	2.95899	-0.57335
H	-1.13087	2.35889	1.02723
H	-3.59950	-1.31651	0.01034
H	-2.59451	-1.86373	1.37230
H	-3.47183	1.75430	0.37286
H	-2.82838	0.76844	-0.95894
C	3.12536	0.22682	1.30453
H	3.03791	-0.46354	2.15830
H	3.01949	1.24996	1.69863
H	4.15961	0.13327	0.89979

ACE2_nm_PBE0_SV

32

O	-0.30246	-1.71341	0.12339
O	-0.31757	1.34891	-0.57583
O	-2.40188	0.09895	0.96360
N	2.09925	-0.04747	0.33090
C	2.06543	-1.42298	-0.11115
C	2.02400	0.96009	-0.71066
C	0.76273	-1.82883	-0.77461
C	0.92699	1.97805	-0.45512
C	-1.55717	-1.69009	-0.48839
C	-1.38736	2.04305	-0.00773
C	-2.60659	-1.20599	0.49205
C	-2.60414	1.14699	0.04880
H	2.20935	-2.06416	0.77664
H	2.89525	-1.67369	-0.81666
H	1.82904	0.48733	-1.68868
H	2.98931	1.49755	-0.82184
H	0.57349	-1.19869	-1.66648
H	0.86440	-2.87408	-1.14024
H	1.04733	2.40130	0.56449
H	1.01503	2.82235	-1.17358
H	-1.85363	-2.70041	-0.84857
H	-1.53470	-1.02463	-1.37494
H	-1.63412	2.95853	-0.58891
H	-1.13544	2.36662	1.02444
H	-3.60523	-1.31288	0.01918
H	-2.59220	-1.85404	1.38512
H	-3.47355	1.75424	0.37036
H	-2.82741	0.76547	-0.96510
C	3.12586	0.22076	1.30418
H	3.03192	-0.47025	2.16042

H	3.02069	1.24786	1.69607
H	4.16242	0.12193	0.89852

ACE2_nm_PBE0_TZVPP

32

O	-0.29790	-1.81811	0.11704
O	-0.32942	1.42381	-0.63952
O	-2.33195	0.09389	0.97862
N	2.04572	-0.04692	0.35028
C	2.05613	-1.41444	-0.12526
C	1.99909	0.96715	-0.69134
C	0.76967	-1.83622	-0.79982
C	0.93319	2.01126	-0.43411
C	-1.55891	-1.70960	-0.49277
C	-1.39922	2.07481	-0.00596
C	-2.57723	-1.21205	0.50593
C	-2.57904	1.13847	0.05937
H	2.22417	-2.06528	0.73738
H	2.88367	-1.61085	-0.83193
H	1.79219	0.50534	-1.65972
H	2.97106	1.47075	-0.79503
H	0.54361	-1.16672	-1.63809
H	0.90480	-2.84377	-1.21864
H	1.01551	2.37845	0.59756
H	1.07391	2.87064	-1.10555
H	-1.89691	-2.68196	-0.88170
H	-1.50123	-1.01827	-1.34246
H	-1.68933	2.98674	-0.54818
H	-1.11640	2.36982	1.01359
H	-3.57433	-1.28426	0.05159
H	-2.56791	-1.85613	1.38824

H	-3.46223	1.70329	0.38162
H	-2.78580	0.74434	-0.94057
C	3.09329	0.21022	1.30986
H	3.01902	-0.49028	2.14406
H	2.99206	1.21999	1.71320
H	4.10631	0.12245	0.87824

ACE2_nm_PBE0_TZVP

32

O	-0.29826	-1.81623	0.11776
O	-0.32925	1.42223	-0.63783
O	-2.33314	0.09401	0.97815
N	2.04682	-0.04679	0.35019
C	2.05609	-1.41437	-0.12549
C	1.99971	0.96752	-0.69134
C	0.76927	-1.83626	-0.79923
C	0.93316	2.01104	-0.43451
C	-1.55930	-1.70979	-0.49256
C	-1.39919	2.07453	-0.00560
C	-2.57837	-1.21199	0.50526
C	-2.57978	1.13915	0.05921
H	2.22443	-2.06568	0.73765
H	2.88364	-1.61141	-0.83322
H	1.79337	0.50567	-1.66059
H	2.97193	1.47240	-0.79452
H	0.54346	-1.16787	-1.63939
H	0.90415	-2.84540	-1.21657
H	1.01662	2.38049	0.59704
H	1.07279	2.86988	-1.10829
H	-1.89660	-2.68386	-0.88035
H	-1.50237	-1.01921	-1.34383

H	-1.68849	2.98695	-0.54917
H	-1.11732	2.37019	1.01485
H	-3.57572	-1.28436	0.04960
H	-2.56946	-1.85626	1.38841
H	-3.46279	1.70523	0.38259
H	-2.78743	0.74588	-0.94170
C	3.09545	0.20944	1.30901
H	3.01982	-0.49002	2.14488
H	2.99641	1.22063	1.71118
H	4.10886	0.11864	0.87700

ACE2_nm_PBE0_TZV

32

O	-0.30210	-1.87274	0.11969
O	-0.33870	1.44523	-0.67332
O	-2.38457	0.09545	1.00170
N	2.08651	-0.04690	0.35137
C	2.07926	-1.42879	-0.12545
C	2.02409	0.97980	-0.69466
C	0.79634	-1.83968	-0.81900
C	0.96202	2.03020	-0.42965
C	-1.59141	-1.71531	-0.50707
C	-1.42917	2.09744	0.00873
C	-2.60693	-1.24648	0.50864
C	-2.61558	1.16712	0.05196
H	2.23331	-2.08151	0.73769
H	2.91091	-1.62936	-0.82795
H	1.81138	0.52196	-1.66377
H	2.99534	1.48692	-0.79988
H	0.55907	-1.13898	-1.62562
H	0.93641	-2.83134	-1.26692

H	1.02831	2.36975	0.61070
H	1.10990	2.89711	-1.08570
H	-1.93229	-2.67004	-0.92869
H	-1.51302	-0.99206	-1.32556
H	-1.71093	3.02289	-0.50936
H	-1.13348	2.35419	1.03278
H	-3.60657	-1.32803	0.06445
H	-2.57206	-1.88601	1.39094
H	-3.49793	1.72945	0.37262
H	-2.81090	0.77067	-0.94842
C	3.13867	0.21173	1.32890
H	3.06445	-0.49695	2.15555
H	3.02860	1.21668	1.74096
H	4.15308	0.13326	0.89765

ACE2_nm_PBE_SV(P)

32

O	-0.30149	-1.71899	0.13169
O	-0.32377	1.35586	-0.58642
O	-2.44001	0.09666	0.97471
N	2.11973	-0.04441	0.33402
C	2.08534	-1.43264	-0.10355
C	2.03636	0.96629	-0.71915
C	0.77625	-1.84828	-0.77161
C	0.93285	1.99206	-0.45973
C	-1.56418	-1.70065	-0.49562
C	-1.40024	2.05357	-0.00080
C	-2.62955	-1.21997	0.48560
C	-2.62969	1.15704	0.04757
H	2.23041	-2.07115	0.79695
H	2.92246	-1.69520	-0.81320

H	1.83295	0.48493	-1.70129
H	3.00759	1.51039	-0.84201
H	0.58835	-1.22504	-1.68100
H	0.88169	-2.90687	-1.12884
H	1.05254	2.41594	0.57037
H	1.02476	2.84703	-1.18105
H	-1.85949	-2.72033	-0.86302
H	-1.53859	-1.02926	-1.39023
H	-1.64884	2.98701	-0.57266
H	-1.14259	2.36638	1.04412
H	-3.63148	-1.33782	-0.00114
H	-2.61629	-1.87362	1.38593
H	-3.50257	1.77707	0.36912
H	-2.85337	0.77562	-0.97631
C	3.16007	0.23204	1.30516
H	3.07695	-0.46547	2.16847
H	3.05122	1.26662	1.70082
H	4.20500	0.13962	0.88985

ACE2_nm_PBE_SVP

32

O	-0.29994	-1.73107	0.13360
O	-0.32712	1.35859	-0.59433
O	-2.44091	0.09319	0.97869
N	2.11812	-0.04245	0.33563
C	2.08588	-1.43135	-0.10056
C	2.03229	0.96519	-0.71992
C	0.78018	-1.84609	-0.77094
C	0.93182	1.99205	-0.46239
C	-1.56106	-1.69469	-0.49894
C	-1.40147	2.05168	0.00341

C	-2.62737	-1.22204	0.48168
C	-2.62884	1.15582	0.05236
H	2.23124	-2.06861	0.79808
H	2.92319	-1.69552	-0.80534
H	1.82401	0.48246	-1.69806
H	3.00136	1.50617	-0.85214
H	0.59013	-1.21503	-1.67199
H	0.88997	-2.89640	-1.14319
H	1.04862	2.41536	0.56577
H	1.02916	2.84808	-1.17785
H	-1.86241	-2.70289	-0.88502
H	-1.52636	-1.01224	-1.38232
H	-1.65444	2.98830	-0.55642
H	-1.13815	2.35936	1.04603
H	-3.62568	-1.34098	-0.00686
H	-2.61771	-1.88245	1.37453
H	-3.50117	1.77510	0.36894
H	-2.85148	0.77738	-0.97062
C	3.15904	0.23609	1.30564
H	3.08294	-0.46200	2.16498
H	3.04986	1.26566	1.70537
H	4.20104	0.14971	0.89101

ACE2_nm_PBE_SV

32

O	-0.30149	-1.71899	0.13169
O	-0.32377	1.35586	-0.58642
O	-2.44001	0.09666	0.97471
N	2.11973	-0.04441	0.33402
C	2.08534	-1.43264	-0.10355
C	2.03636	0.96629	-0.71915

C	0.77625	-1.84828	-0.77161
C	0.93285	1.99206	-0.45973
C	-1.56418	-1.70065	-0.49562
C	-1.40024	2.05357	-0.00080
C	-2.62955	-1.21997	0.48560
C	-2.62969	1.15704	0.04757
H	2.23041	-2.07115	0.79695
H	2.92246	-1.69520	-0.81320
H	1.83295	0.48493	-1.70129
H	3.00759	1.51039	-0.84201
H	0.58835	-1.22504	-1.68100
H	0.88169	-2.90687	-1.12884
H	1.05254	2.41594	0.57037
H	1.02476	2.84703	-1.18105
H	-1.85949	-2.72033	-0.86302
H	-1.53859	-1.02926	-1.39023
H	-1.64884	2.98701	-0.57266
H	-1.14259	2.36638	1.04412
H	-3.63148	-1.33782	-0.00114
H	-2.61629	-1.87362	1.38593
H	-3.50257	1.77707	0.36912
H	-2.85337	0.77562	-0.97631
C	3.16007	0.23204	1.30516
H	3.07695	-0.46547	2.16847
H	3.05122	1.26662	1.70082
H	4.20500	0.13962	0.88985

ACE2_nm_PBE_TZVPP

32

O	-0.29544	-1.83389	0.12615
O	-0.33848	1.43397	-0.65233

O	-2.36620	0.09047	0.99284
N	2.06648	-0.04304	0.35498
C	2.07766	-1.42236	-0.12384
C	2.01078	0.97846	-0.69627
C	0.78502	-1.85116	-0.80274
C	0.93835	2.02852	-0.43356
C	-1.56742	-1.72179	-0.49993
C	-1.41832	2.08796	-0.00063
C	-2.59923	-1.23020	0.50177
C	-2.60626	1.14715	0.05710
H	2.24956	-2.07396	0.74702
H	2.91105	-1.62332	-0.83640
H	1.79707	0.51052	-1.66899
H	2.98804	1.48793	-0.80893
H	0.55319	-1.17923	-1.64814
H	0.92392	-2.86669	-1.22248
H	1.01394	2.39074	0.60869
H	1.08231	2.89871	-1.10393
H	-1.90421	-2.69967	-0.90012
H	-1.50207	-1.02046	-1.35142
H	-1.71080	3.01298	-0.53658
H	-1.12825	2.37434	1.02800
H	-3.60061	-1.30921	0.03841
H	-2.58950	-1.87809	1.39071
H	-3.49658	1.71755	0.37745
H	-2.80958	0.74768	-0.94967
C	3.13167	0.21818	1.31152
H	3.06913	-0.49253	2.14760
H	3.02645	1.23194	1.72342
H	4.14919	0.13905	0.86671

ACE2_nm_PBE_TZVP

32

O	-0.29585	-1.83123	0.12721
O	-0.33841	1.43162	-0.65027
O	-2.36867	0.09046	0.99242
N	2.06821	-0.04278	0.35492
C	2.07771	-1.42229	-0.12377
C	2.01148	0.97887	-0.69637
C	0.78460	-1.85093	-0.80178
C	0.93810	2.02800	-0.43399
C	-1.56767	-1.72148	-0.49984
C	-1.41830	2.08720	-0.00000
C	-2.60085	-1.23021	0.50060
C	-2.60744	1.14788	0.05695
H	2.24958	-2.07423	0.74777
H	2.91109	-1.62445	-0.83729
H	1.79843	0.51081	-1.66995
H	2.98878	1.48998	-0.80869
H	0.55331	-1.18023	-1.64925
H	0.92303	-2.86819	-1.21985
H	1.01505	2.39266	0.60812
H	1.08056	2.89762	-1.10688
H	-1.90333	-2.70110	-0.89928
H	-1.50296	-1.02066	-1.35276
H	-1.70953	3.01297	-0.53715
H	-1.12935	2.37390	1.02973
H	-3.60221	-1.30970	0.03536
H	-2.59172	-1.87860	1.39019
H	-3.49734	1.72003	0.37829
H	-2.81171	0.74948	-0.95089

C	3.13519	0.21743	1.30994
H	3.07182	-0.49224	2.14777
H	3.03258	1.23263	1.72079
H	4.15288	0.13516	0.86416

ACE2_nm_PBE_TZV

32

O	-0.30569	-1.87996	0.13573
O	-0.34471	1.44428	-0.68116
O	-2.42924	0.09414	1.01976
N	2.11351	-0.04416	0.35792
C	2.09911	-1.43905	-0.12276
C	2.04123	0.99031	-0.69954
C	0.80763	-1.85283	-0.81929
C	0.97128	2.04541	-0.42888
C	-1.60615	-1.72327	-0.51643
C	-1.44593	2.11023	0.01541
C	-2.64035	-1.26203	0.49694
C	-2.64566	1.18226	0.04994
H	2.25609	-2.09352	0.74865
H	2.93505	-1.64810	-0.83320
H	1.82282	0.52582	-1.67346
H	3.01757	1.50454	-0.81334
H	0.56649	-1.15095	-1.63500
H	0.94718	-2.85525	-1.26462
H	1.03291	2.38349	0.62083
H	1.11645	2.92105	-1.08859
H	-1.94117	-2.68599	-0.94841
H	-1.51834	-0.99150	-1.33768
H	-1.72315	3.04827	-0.50166
H	-1.14458	2.36085	1.04838

H	-3.64162	-1.34368	0.03556
H	-2.61476	-1.90905	1.38417
H	-3.53190	1.75531	0.37005
H	-2.84106	0.78056	-0.95692
C	3.19619	0.21300	1.32187
H	3.13684	-0.50390	2.15268
H	3.09304	1.22381	1.74142
H	4.21075	0.13548	0.86743

ACE2_nm_SCS-MP2_SV(P)

32

O	-0.31612	-1.72336	0.12395
O	-0.31130	1.34082	-0.58164
O	-2.36948	0.10867	0.97742
N	2.07186	-0.05217	0.35443
C	2.05199	-1.42631	-0.12510
C	2.03707	0.96120	-0.70001
C	0.74519	-1.81018	-0.80110
C	0.93987	1.98450	-0.45223
C	-1.58106	-1.68887	-0.49543
C	-1.38240	2.05654	-0.00966
C	-2.61610	-1.20121	0.50352
C	-2.59965	1.15603	0.04959
H	2.19028	-2.09163	0.74713
H	2.88805	-1.64543	-0.83475
H	1.85760	0.49441	-1.68469
H	3.01213	1.48863	-0.78305
H	0.54708	-1.14679	-1.66480
H	0.83816	-2.84561	-1.19882
H	1.05446	2.40203	0.56999
H	1.02411	2.82838	-1.17315

H	-1.88070	-2.69785	-0.85907
H	-1.55580	-1.01436	-1.37321
H	-1.61990	2.96933	-0.60016
H	-1.12760	2.37808	1.02199
H	-3.62577	-1.28012	0.04749
H	-2.59416	-1.85293	1.39450
H	-3.47673	1.75170	0.37634
H	-2.81880	0.76603	-0.96173
C	3.14831	0.18383	1.30186
H	3.05117	-0.50304	2.16205
H	3.08562	1.21728	1.68789
H	4.16378	0.04483	0.85850

ACE2_nm_SCS-MP2_SVP

32

O	-0.31327	-1.72302	0.12555
O	-0.31452	1.33532	-0.58393
O	-2.37685	0.10391	0.98047
N	2.07222	-0.04929	0.35590
C	2.05371	-1.42355	-0.11920
C	2.03301	0.95892	-0.70119
C	0.74972	-1.80776	-0.79565
C	0.93523	1.97993	-0.45824
C	-1.57379	-1.67903	-0.49908
C	-1.38226	2.04876	-0.00589
C	-2.61431	-1.20226	0.49615
C	-2.60028	1.15271	0.05495
H	2.19253	-2.08356	0.75141
H	2.88600	-1.64342	-0.82548
H	1.85228	0.48899	-1.67931
H	3.00157	1.48735	-0.78919

H	0.55408	-1.14509	-1.65532
H	0.84494	-2.83673	-1.19637
H	1.04867	2.40192	0.55755
H	1.02025	2.81783	-1.17870
H	-1.87414	-2.67748	-0.87634
H	-1.54222	-0.99699	-1.36568
H	-1.62163	2.96055	-0.58821
H	-1.12397	2.36659	1.02138
H	-3.61485	-1.28280	0.03165
H	-2.59767	-1.86078	1.37678
H	-3.47086	1.75172	0.37674
H	-2.81887	0.76650	-0.95335
C	3.14799	0.19157	1.29954
H	3.05734	-0.49108	2.15678
H	3.08595	1.22034	1.68273
H	4.15805	0.05584	0.85655

ACE2_nm_SCS-MP2_SV

32

O	-0.31612	-1.72336	0.12395
O	-0.31130	1.34082	-0.58164
O	-2.36948	0.10867	0.97742
N	2.07186	-0.05217	0.35443
C	2.05199	-1.42631	-0.12510
C	2.03707	0.96120	-0.70001
C	0.74519	-1.81018	-0.80110
C	0.93987	1.98450	-0.45223
C	-1.58106	-1.68887	-0.49543
C	-1.38240	2.05654	-0.00966
C	-2.61610	-1.20121	0.50352
C	-2.59965	1.15603	0.04959

H	2.19028	-2.09163	0.74713
H	2.88805	-1.64543	-0.83475
H	1.85760	0.49441	-1.68469
H	3.01213	1.48863	-0.78305
H	0.54708	-1.14679	-1.66480
H	0.83816	-2.84561	-1.19882
H	1.05446	2.40203	0.56999
H	1.02411	2.82838	-1.17315
H	-1.88070	-2.69785	-0.85907
H	-1.55580	-1.01436	-1.37321
H	-1.61990	2.96933	-0.60016
H	-1.12760	2.37808	1.02199
H	-3.62577	-1.28012	0.04749
H	-2.59416	-1.85293	1.39450
H	-3.47673	1.75170	0.37634
H	-2.81880	0.76603	-0.96173
C	3.14831	0.18383	1.30186
H	3.05117	-0.50304	2.16205
H	3.08562	1.21728	1.68789
H	4.16378	0.04483	0.85850

ACE2_nm_SCS-MP2_TZVPP

32

O	-0.31438	-1.79159	0.12540
O	-0.32077	1.37513	-0.61895
O	-2.34660	0.10303	0.99277
N	2.03954	-0.04771	0.37569
C	2.04543	-1.41629	-0.12649
C	2.01672	0.96470	-0.68446
C	0.75032	-1.80532	-0.81079
C	0.93910	2.00130	-0.43536

C	-1.57808	-1.68584	-0.50367
C	-1.39000	2.06751	-0.00343
C	-2.60277	-1.20487	0.50128
C	-2.58737	1.14847	0.05674
H	2.20242	-2.08085	0.72239
H	2.86935	-1.59374	-0.83454
H	1.82759	0.50044	-1.65049
H	2.98498	1.46823	-0.76323
H	0.52706	-1.11291	-1.62314
H	0.86354	-2.80303	-1.24568
H	1.02311	2.37951	0.58652
H	1.05011	2.84431	-1.12432
H	-1.90017	-2.65495	-0.89887
H	-1.51507	-0.98564	-1.33779
H	-1.64871	2.97045	-0.56530
H	-1.11307	2.36267	1.01172
H	-3.59479	-1.25916	0.04551
H	-2.59160	-1.85589	1.37190
H	-3.46055	1.71954	0.37647
H	-2.79037	0.74837	-0.93573
C	3.15742	0.17630	1.28227
H	3.09474	-0.51399	2.12027
H	3.11246	1.19066	1.67241
H	4.13152	0.04165	0.79170

ACE2_nm_SCS-MP2_TZVP

32

O	-0.31756	-1.77568	0.12821
O	-0.31780	1.36286	-0.60359
O	-2.36049	0.10628	0.99034
N	2.05139	-0.04864	0.37485

C	2.04511	-1.41663	-0.12985
C	2.02281	0.96722	-0.68240
C	0.74551	-1.79824	-0.81039
C	0.93961	1.99757	-0.43066
C	-1.58351	-1.68627	-0.49981
C	-1.39114	2.06491	-0.00552
C	-2.61162	-1.20415	0.50164
C	-2.59288	1.15091	0.05039
H	2.20270	-2.08677	0.72008
H	2.86909	-1.59940	-0.84366
H	1.83968	0.50428	-1.65501
H	2.99216	1.47861	-0.75830
H	0.52279	-1.10654	-1.62880
H	0.85136	-2.80268	-1.24394
H	1.02847	2.38344	0.59268
H	1.04048	2.84268	-1.12626
H	-1.89889	-2.66649	-0.88648
H	-1.52737	-0.99374	-1.34607
H	-1.64037	2.96805	-0.58045
H	-1.12271	2.37088	1.01354
H	-3.60687	-1.26336	0.04260
H	-2.60154	-1.85366	1.37906
H	-3.46902	1.72804	0.36722
H	-2.79604	0.74873	-0.94585
C	3.17780	0.16716	1.27323
H	3.11712	-0.52416	2.11582
H	3.14415	1.18548	1.66545
H	4.15162	0.02458	0.77507

ACE2_nm_SCS-MP2_TZV

32

O	-0.36684	-1.98998	0.07588
O	-0.27757	1.53686	-0.73891
O	-2.26937	0.15461	1.02130
N	2.00440	-0.09265	0.38157
C	2.02108	-1.47997	-0.14962
C	2.07573	0.94768	-0.68317
C	0.73678	-1.85735	-0.89055
C	1.06419	2.07122	-0.44501
C	-1.68966	-1.74144	-0.51632
C	-1.36008	2.18564	0.01542
C	-2.62848	-1.19749	0.55881
C	-2.54955	1.23620	0.05576
H	2.15105	-2.16119	0.69882
H	2.87218	-1.63741	-0.84295
H	1.86357	0.50196	-1.66125
H	3.08992	1.38190	-0.73627
H	0.47592	-1.08997	-1.62731
H	0.88102	-2.81312	-1.41322
H	1.10745	2.39536	0.60279
H	1.26736	2.93004	-1.09913
H	-2.10214	-2.67828	-0.91666
H	-1.58987	-1.02133	-1.33627
H	-1.64503	3.12753	-0.47264
H	-1.02473	2.39452	1.03913
H	-3.65564	-1.19548	0.16823
H	-2.58354	-1.82092	1.45399
H	-3.44565	1.77424	0.38718
H	-2.72969	0.82876	-0.94550
C	3.05955	0.10724	1.40925
H	2.89052	-0.58014	2.24273

H	3.00401	1.13194	1.78832
H	4.07540	-0.06219	1.00543
